# Comparison of Intentional and Unintentional Injuries Among Chinese Children and Adolescents

**DOI:** 10.2188/jea.JE20190152

**Published:** 2020-12-05

**Authors:** Xiling Yin, Deyun Li, Kejing Zhu, Xiaodong Liang, Songxu Peng, Aijun Tan, Yukai Du

**Affiliations:** 1 Department of Maternal and Child Health, School of Public Health, Tongji Medical College, Huazhong University of Science and Technology, Wuhan, Hubei, China; 2 Zhuhai Center of Disease Control and Prevention, Zhuhai, Guangdong, China

**Keywords:** intentional injury, unintentional injury, children, adolescent, violent attacks, self-mutilation, suicide, China

## Abstract

**Background:**

The patterns and risk factors of intentional injuries compared to unintentional injuries among Chinese children and adolescents have not been examined in depth. This work comprehensively describes patterns of intentional injuries in China, for which little information has been previously published.

**Methods:**

All cases involving individuals 0–17 years old registered at emergency rooms and outpatient clinics were examined using data submitted to the National Injury Surveillance System from 2006 through 2017. A logistic regression model was performed to explore the risk factors related to intentional injuries compared to unintentional injuries.

**Results:**

A total of 81,459 (95.1%) unintentional injuries, 4,218 (4.9%) intentional injuries (4,013 violent attacks and 205 self-mutilation/suicide) cases were identified. Blunt injuries accounted for 59.4% of violent attacks, while cuts and poisoning accounted for 37.1% and 23.4% of injuries involving self-mutilation/suicide, respectively. For unintentional injuries, falls (50.4%) ranked first. Additional risk factors for intentional injuries included being male (odds ratio [OR] 1.6), coming from rural areas (OR 1.9), being staff or workers (OR 2.2), and being a student (OR 1.8). As the age of the patients increased, so did the risk of intentional injuries (OR 5.0 in the 15–17 age group). Intentional injuries were more likely to occur at 00:00–03:00 am (OR 2.0).

**Conclusions:**

Intentional injuries affected more males, rural and older children, school students, and staff or workers. The mechanisms and occurrence times differed according to age group. Preventive measures should be taken to reduce the dropout of rural students, strengthen the school’s violence prevention plan, and reduce self-harm.

## INTRODUCTION

The patterns of injuries in children and adolescents reflect the underlying risk profiles of specific characteristics.^1^ According to the intent of the injury, injuries can be divided into intentional and unintentional injuries. Intentional injuries include violent attacks and self-mutilation or suicide. Currently, unintentional and intentional injuries among children and adolescents have specific focuses or directions in the field of intervention. Intentional injury prevention initiatives tend to focus on personal factors and behavioral choices. Unintentional injury prevention initiatives tend to focus on the relationship between people, things, and the environment.^2^

Characterizing injuries by intentions is necessary for appropriate injury management.^3^ Intentional injuries often have the same mechanisms as unintentional injuries. For example, road traffic injuries may be intentional injuries caused by suicide or unintentional injuries caused by accidental traffic collisions. However, these mechanisms have different frequencies within intentional injuries and between the two types of injuries. Studies have shown that falls are the most common cause of unintentional injuries in children, followed by burns in preschool children, whereas road traffic injuries are the most likely cause of injury during adolescence.^4^ Among adolescents, 61% of firearm injuries and 98% of suffocation deaths are intentional injuries.^5^ Studies of intentional injuries in children under the age of 16 in Australia indicate that poisoning is the most common form of self-harm/suicide.^6^ Common self-harm/suicide methods include cuts and poisoning, whereas blunt injuries mainly involve violent attacks. With regard to the nature of the injury, some studies have found that soft tissue damage is common in aggressive injuries, and cuts and fractures are more common in self-injury.^7^ Gallaher^8^ showed that with regard to intentional injuries of children (<18 years old), the average age of patients is higher, more men than women are injured, more injuries occur at night, soft tissue damage is more common, and head injury is the most common cause of death. Studies have also shown that deliberate harm is higher among rural students than urban students.^9^

Currently, research on the influencing factors of injury mostly focuses on the relationship between unintentional injury and non-injury or between intentional injury and non-injury.^10^^,^^11^ However, a study of adults showed that victims of intentional injuries were more likely to be unemployed and to have lower average intelligence scores than victims of unintentional harm.^12^ Compared to unintentional falls, the intentional falls could be caused by an increased risk of higher height of falls, more serious injury, and victims of higher education.^13^ Lama BB^14^ contrasted the characteristics of patients with intentional and unintentional burns indicating that most intentional burns patients were women, almost all intentional burns occurred in the house, and intentional burns could lead to more serious adverse consequences. This comparison between different injury intentions studies provides opportunities for prevention programs for injuries at further details.

In China, injuries are the leading cause of death among people aged 1–14 years old.^15^ The ratio of the occurrence of injury to death in China is approximately 100:1.^16^ Injury has brought a huge disease burden to Chinese children and adolescents. There is little research on the trends and patterns of intentional injuries in Chinese children and adolescents, especially the additional risk factors compared with unintentional injuries. This work is novel because it comprehensively describes patterns of intentional injuries in China, where little information has previously been published.

## METHOD

### Setting

Data were used from the National Injury Surveillance System (NISS) in Zhuhai City, China, from January 1, 2006, through December 31, 2017. NISS, which collects injury case data on the initial visits for all injuries in emergency rooms and outpatient clinics in sentinel hospitals, is a hospital-based injury monitoring system that was launched in 2006 with the support of the Chinese Ministry of Health.

There are three sentinel monitoring hospitals in Zhuhai City that are included in the NISS. These three hospitals are government-sponsored comprehensive hospitals with a strong ability to treat patients with emergency injuries. Since 2006, they have been used as fixed sentinel surveillance hospitals for injuries.

### Data collection

The NISS captures nonfatal and fatal injuries in emergency rooms and outpatient clinics, but it cannot capture cases in which the patient does not see a doctor. Doctors or nurses of emergency rooms and outpatient clinics who have received standardized training complete a standardized national injury monitoring report card; then, the public health doctors review the report card and input the information into NISS. NISS has a standardized program that defines all aspects of quality control.^17^

The contents of the injury report card include the general personal information of the patient (eg, gender, age, family registration, and occupation), the situation of the injury event (eg, time of injury, mechanism of injury, and intent of injury) and the clinical information.

### Measures

The intent of the injuries was determined by a combination of inquiry and clinical diagnosis. There are three types of intent resulting in injuries. Unintentional injury indicates an injury due to accidental events. Intentional injury indicates either self-mutilation/suicide or violent attacks. Self-mutilation/suicide refers to injuries inflicted by the patient who was known to be injured, either directly or indirectly, by some positive or negative action that could have resulted in injury or death. Violent attacks indicate that the patient was deliberately attacked or violently injured by another person. If the doctors could not determine the intent behind the injury or the patient did not answer the question, the intent behind the injuries was judged as unclear.

This article defines children and adolescents as individuals under 18 years of age. According to school enrollment, children aged 0–17 years are classified into five age groups as follows: 0–2 years old (preschool), 3–5 years old (kindergarten), 6–11 years old (elementary school), 12–14 years old (junior high school) and 15–17 years old (high school).

### Data analysis

The proportions were calculated to analyze the intent resulting in injury cases involving children of different sexes and ages, as well as the mechanisms. The time-trend analysis used the Cochran-Armitage trend test. The distribution of the time of day injuries occurred and the mechanisms and results of the injuries were described. An unconditional multivariate logistic regression was used to calculate the odds ratio (OR) and 95% confidence interval (CI) to explore the factors associated with intentional compared to unintentional injuries. Ten variables, such as gender, region, age group, the time that injuries occurred, and occupation, were entered to study the injury patterns. Data analysis was performed using Statistical Package for the Social Sciences (19.0; IBM Corp, Armonk, NY, USA) and R (3.4.0; R Foundation for Statistical Computing, Vienna, Austria) software with a test level of α = 0.05.

## RESULTS

### General description

**Figure 1.**
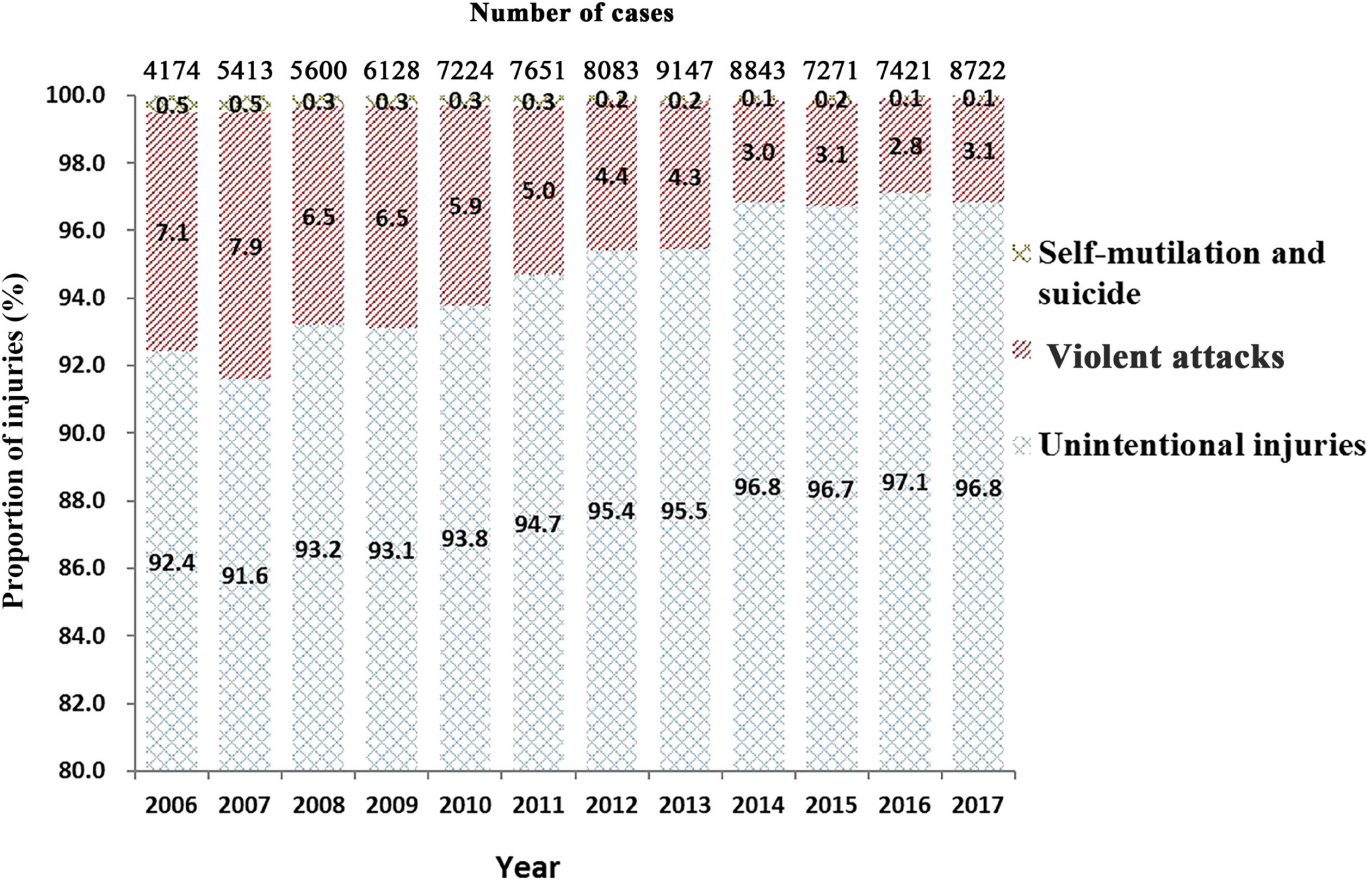
Number of cases and proportion of unintentional and intentional injuries (self-mutilation/suicide and violent attacks) cases among 0- to 17-year-olds from 2006 to 2017 in Zhuhai City, China

A total of 85,677 persons aged ≤17 years old were identified; of these, 81,459 were unintentional injuries (95.1%) and 4,218 were intentional injuries (4.9%). Of the intentional injuries, violent attacks accounted for 95.1% (4,013 cases), and self-mutilation or suicide accounted for 4.9% (205 cases). From 2006 through 2017, the proportion of cases of intentional injury showed a decreasing trend (z = 22.7, *P* < 0.001) (Figure 1).

Approximately 85.6% of the injured patients went home after treatment, and 12.6% needed hospitalization; 1.8% of the injury cases were unclear about the outcome. Of the 29 cases resulting in death, 25 were unintentional accidents, 2 were suicide, and 2 were violent attacks.

### Risk factors of intentional injuries compared to unintentional injuries

Our study identified six factors related to intentional injuries compared to unintentional injuries. In general, boys had a greater chance of suffering intentional injuries than girls (OR 1.6; 95% CI, 1.4–1.7). Children and adolescents who came from rural areas were more susceptible to intentional injuries (OR 1.9; 95% CI, 1.7–2.0). Compared with children aged <3 years old, as the age of the children increased, so did the risk of intentional injury (the ORs of the age groups 3–5, 6–11, 12–14, and 15–17 were 1.3, 2.0, 4.6, and 5.0, respectively). Interestingly, 00:00–03:00 am was a likely time to suffer an intentional injury (OR 2.0; 95% CI, 1.7–2.3). Children who were staff or workers (OR 2.2; 95% CI, 1.7–2.9) or school students (OR 1.8; 95% CI, 1.4–2.2) were prone to intentional injuries. Most importantly, public places (OR 2.4; 95% CI, 2.2–2.6) or the workplace (OR 1.7; 95% CI, 1.4–2.0) were the most common locations for intentional injuries (Table 1).

**Table 1.** Number and proportion of unintentional and intentional injuries cases and logistic regression results among 0- to 17-year-olds from 2006 to 2017 in Zhuhai City, China

		Total *n* (%)	Unintentional injuries *n* (%)	Intentional injuries			Multivariable logistic regression	
	
				Violent attacks *n* (%)	Self-mutilation and suicide *n* (%)	Subtotal *n* (%)	OR (95% CI)	*P*-value
Total		85,677 (100)	81,459 (100)	4,013 (100)	205 (100)	4,218 (100)	/	/

Gender	Female	27,642 (32.3)	26,822 (32.9)	735 (18.3)	85 (41.5)	820 (19.4)	Reference	—
	Male	58,035 (67.7)	54,637 (67.1)	3,278 (81.7)	120 (58.5)	3,398 (80.6)	1.6 (1.4–1.7)	<0.001^*^

Region	Urban	60,528 (70.6)	58,092 (71.3)	2,307 (57.5)	129 (62.9)	2,436 (57.8)	Reference	—
	Rural	25,149 (29.4)	23,367 (28.7)	1,706 (42.5)	76 (37.1)	1,782 (42.2)	1.9 (1.7–2.0)	<0.001^*^

Age group, years old	0∼	17,957 (21.0)	17,788 (21.8)	164 (4.1)	5 (2.4)	169 (4.0)	Reference	—
	3∼	18,983 (22.2)	18,702 (23.1)	271 (6.8)	10 (4.9)	281 (6.7)	1.3 (1.1–1.6)	0.005^*^
	6∼	25,298 (29.4)	24,302 (29.8)	973 (24.2)	23 (11.2)	996 (23.6)	2.0 (1.6–2.6)	<0.001^*^
	12∼	9,513 (11.1)	8,581 (10.5)	900 (22.4)	32 (15.6)	932 (22.1)	4.6 (3.5–6.0)	<0.001^*^
	15∼17	13,926 (16.3)	12,086 (14.8)	1,705 (42.5)	135 (65.9)	1,840 (43.6)	5.0 (3.8–6.6)	<0.001^*^

Time of injury occurrence (o’clock)	0–3	2,915 (3.4)	2,482 (3.0)	401 (10.0)	32 (15.6)	433 (10.3)	2.0 (1.7–2.3)	<0.001^*^
	4–7	2,241 (2.6)	2,092 (2.6)	138 (3.4)	11 (5.4)	149 (3.5)	1.0 (0.8–1.2)	0.998
	8–11	15,987 (18.7)	15,320 (18.7)	645 (16.1)	22 (10.7)	667 (15.8)	0.8 (0.7–0.9)	<0.001^*^
	12–15	16,498 (19.3)	15,769 (19.4)	697 (17.4)	32 (15.6)	729 (17.3)	0.8 (0.7–0.9)	<0.001^*^
	16–19	29,023 (33.8)	27,667 (34.0)	1,319 (32.9)	37 (18.0)	1,356 (32.1)	0.8 (0.7–0.9)	<0.001^*^
	20–23	19,013 (22.2)	18,129 (22.3)	813 (20.2)	71 (34.6)	884 (21.0)	Reference	—

Occupation	Preschool children	37,624 (43.9)	37,158 (45.6)	452 (11.3)	14 (6.8)	466 (11.0)	Reference	—
	School students	43,498 (50.8)	40,499 (49.7)	2,873 (71.6)	126 (61.5)	2,999 (71.1)	1.8 (1.4–2.2)	<0.001^*^
	Staff or workers	3,135 (3.7)	2,694 (3.3)	414 (10.3)	27 (13.2)	441 (10.5)	2.2 (1.7–2.9)	<0.001^*^
	Other	1,420 (1.6)	1,108 (1.3)	274 (6.8)	38 (18.6)	312 (7.4)	/	/

Injury place	At home	35,277 (41.2)	34,556 (42.4)	621 (15.5)	100 (48.8)	721 (17.1)	Reference	—
	Public place	32,537 (38.0)	29,840 (36.6)	2,624 (65.4)	73 (35.6)	2,697 (63.9)	2.4 (2.2–2.6)	<0.001^*^
	On the road	14,261 (16.6)	13,945 (17.1)	308 (7.7)	8 (3.9)	316 (7.5)	0.5 (0.4–0.5)	<0.001^*^
	Workplace	3,062 (3.6)	2,706 (3.3)	342 (8.5)	14 (6.8)	356 (8.4)	1.7 (1.4–2.0)	<0.001^*^
	Other	540 (0.6)	412 (0.5)	118 (2.9)	10 (4.9)	128 (3.0)	/	/

^*^P < 0.01.

### Distribution of the time of day injuries occurred

**Figure 2.**
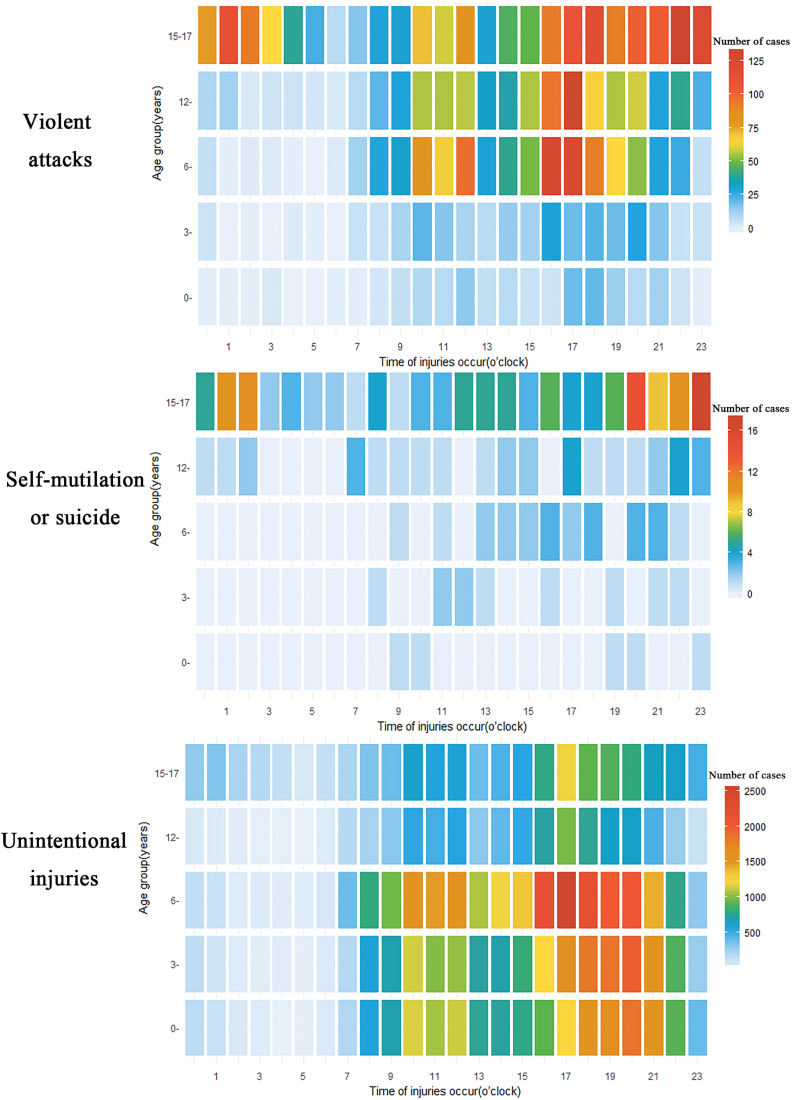
Heat chart of occurring time (o’clock) distribution of the number of intentional and unintentional injury cases by different age groups among 0- to 17-year-olds from 2006 to 2017 in Zhuhai City, China

Children in different age groups had different common times of injury. For violent attacks, 40.4% of children aged 6–14 years old were injured at 16:00–19:00 and 21.9% of children were injured at 10:00–12:00; 71.3% of teenagers aged 15–17 years old experienced violent attacks from 16:00 to 03:00 the next morning. For self-mutilation or suicide, 56.3% of adolescents aged 15–17 years old were injured from 20:00 to 02:00 the next morning. For unintentional injuries, 45.6% of children aged 0–5 years old were injured at 17:00–21:00; 84.2% of children 6 to 11 years old were at greater risk of injury from 10:00 to 21:00, especially between 16:00 and 18:00; and children aged 12–17 years old were hurt more often around 17:00 (Figure 2).

### Mechanisms of intentional injuries and unintentional injuries

Blunt injuries (59.4%) accounted for a large proportion of violent attacks, especially for males (62.4% of blunt injuries). Cuts (37.1%) and poisoning (23.4%) accounted for a large proportion of self-mutilation injuries and suicide. In contrast, for unintentional injuries, falls (50.4%) ranked first (Table 2).

**Table 2.** Number and proportion of unintentional and intentional injury cases by different genders and mechanisms of injury among 0- to 17-year-olds from 2006 to 2017 in Zhuhai City, China

	Total *n* = 85,677	Unintentional injuries				Intentional injuries								

						Violent attacks				Self-mutilation and suicide				ALL *n* = 4,218
		
		Male *n* = 54,637	Female *n* = 26,822	ALL *n* = 81,459	Rank	Male *n* = 3,278	Female *n* = 735	Subtotal *n* = 4,013	Rank	Male *n* = 120	Female *n* = 85	Subtotal *n* = 205	Rank	
Falls (%)	41,229 (48.1)	28,213 (51.6)	12,817 (47.8)	41,030 (50.4)	1	137 (4.2)	33 (4.5)	170 (4.2)	4	14 (11.7)	15 (17.6)	29 (14.1)	3	199 (4.7)
Animal bites (%)	11,936 (13.9)	6,776 (12.4)	4,471 (16.7)	11,247 (13.8)	2	446 (13.6)	237 (32.2)	683 (17.0)	2	3 (2.5)	3 (3.5)	6 (2.9)	6	689 (16.3)
Road traffic injuries (%)	9,812 (11.5)	6,477 (11.9)	3,313 (12.4)	9,790 (12.0)	3	11 (0.3)	2 (0.3)	13 (0.3)	7	6 (5.0)	3 (3.5)	9 (4.4)	5	22 (0.5)
Blunt injuries (%)	9,624 (11.2)	5,144 (9.4)	2,070 (7.7)	7,214 (8.9)	4	2,045 (62.4)	340 (46.3)	2,385 (59.4)	1	22 (18.3)	3 (3.5)	25 (12.2)	4	2,410 (57.1)
Cuts (%)	5,836 (6.8)	3,849 (7.0)	1,443 (5.4)	5,292 (6.5)	5	403 (12.3)	65 (8.8)	468 (11.7)	3	42 (35.0)	34 (40.0)	76 (37.1)	1	544 (12.9)
Burns (%)	4,568 (5.3)	2,745 (5.0)	1,806 (6.7)	4,551 (5.6)	6	8 (0.2)	7 (1.0)	15 (0.4)	6	2 (1.7)	0	2 (1.0)		17 (0.4)
Poisoning (%)	402 (0.5)	169 (0.3)	163 (0.6)	332 (0.4)	7	13 (0.4)	9 (1.2)	22 (0.5)	5	22 (18.3)	26 (30.6)	48 (23.4)	2	70 (1.7)
Firearm injuries (%)	48 (0.1)	37 (0.1)	8 (0.03)	45 (0.1)	8	3 (0.1)	0	3 (0.1)	9	0	0	0	/	3 (0.1)
Hanging or suffocation (%)	23 (0.03)	16 (0.03)	5 (0.02)	21 (0.03)	9	2 (0.06)	0	2 (0.05)	10	0	0	0	/	2 (0.05)
Drowning (%)	16 (0.02)	6 (0.01)	7 (0.03)	13 (0.02)	10	2 (0.06)	0	2 (0.05)	10	0	1 (1.18)	1 (0.49)	7	3 (0.1)
Sexual assault (%)	9 (0.01)	0	0	0	/	6 (0.18)	3 (0.41)	9 (0.2)	8	0	0	0	/	9 (0.2)
Other injuries (%)	2,174 (2.5)	1,205 (2.2)	719 (2.7)	1,924 (2.4)	/	202 (6.2)	39 (5.3)	241 (6.0)	/	9 (7.5)	0	9 (4.4)	/	250 (5.9)

For violent attacks, animal bites accounted for the largest proportion in the 0–2 and 3–5 age groups, accounting for 61.6% and 50.2% of violent attacks, respectively. Blunt injuries accounted for the largest proportion in the 6–11, 12–14, and 15–17 age groups (51.2%, 73.1%, and 66.3%, respectively). Among self-mutilation and suicide, 81.5% of injured children were aged 12–17. The proportions of cuts (37.5–44.4%) and poisoning (18.8–29.6%) were the largest in the 12–17 age group. For unintentional injuries, the largest proportion for all age groups was falls (36.1–55.6%) (Table 3).

**Table 3.** Number and proportion of unintentional and intentional injury cases by different age groups (years) and mechanisms of injury among 0- to 17-year-olds from 2006 to 2017 in Zhuhai City, China

	Unintentional injuries					Intentional injuries									

						Violent attacks					Self-mutilation and suicide				
		
	0–	3–	6–	12–	15–17	0–	3–	6–	12–	15–17	0–^a^	3–^a^	6–^a^	12–	15–17
	*n* = 17,788	*n* = 18,702	*n* = 24,302	*n* = 8,581	*n* = 12,086	*n* = 164	*n* = 271	*n* = 973	*n* = 900	*n* = 1,705	*n* = 5	*n* = 10	*n* = 23	*n* = 32	*n* = 135
Falls (%)	9,615 (54.1)	10,403 (55.6)	12,438 (51.2)	4,212 (49.1)	4,362 (36.1)	8 (4.9)	8 (3.0)	76 (7.8)	36 (4.0)	42 (2.5)	2 (40.0)	4 (40.0)	7 (30.4)	5 (15.6)	11 (8.1)
Animal bites (%)	1,611 (9.1)	2,443 (13.1)	4,590 (18.9)	1,374 (16.0)	1,229 (10.2)	101 (61.6)	136 (50.2)	283 (29.1)	87 (9.7)	76 (4.5)	0	2 (20.0)	3 (13.0)	1 (3.1)	0
Road traffic injuries (%)	1,229 (6.9)	2,196 (11.7)	2,660 (10.9)	1,161 (13.5)	2,544 (21.0)	1 (0.6)	0	2 (0.2)	2 (0.2)	8 (0.5)	1 (20.0)	2 (20.0)	3 (13.0)	1 (3.1)	2 (1.5)
Blunt injuries (%)	1,304 (7.3)	1,550 (8.3)	2,075 (8.5)	782 (9.1)	1,503 (12.4)	19 (11.6)	80 (29.5)	498 (51.2)	658 (73.1)	1,130 (66.3)	1 (20.0)	1 (10.0)	3 (13.0)	5 (15.6)	15 (11.1)
Cuts (%)	692 (3.9)	797 (4.3)	1,508 (6.2)	689 (8.0)	1,606 (13.3)	6 (3.7)	15 (5.5)	50 (5.1)	57 (6.3)	340 (19.9)	0	0	4 (17.4)	12 (37.5)	60 (44.4)
Burns (%)	2,611 (14.7)	847 (4.5)	654 (2.7)	140 (1.6)	299 (2.5)	7 (4.3)	3 (1.1)	4 (0.4)	1 (0.1)	0	0	0	0	0	2 (1.5)
Poisoning (%)	10 (0.1)	17 (0.1)	24 (0.1)	60 (0.7)	221 (1.8)	0	2 (0.7)	1 (0.1)	1 (0.1)	18 (1.1)	0	1 (10.0)	1 (4.3)	6 (18.8)	40 (29.6)
Firearm injuries (%)	9 (0.05)	7 (0.04)	20 (0.08)	3 (0.03)	6 (0.05)	0	2 (0.7)	1 (0.1)	0	0	0	0	0	0	0
Hanging or suffocation (%)	7 (0.04)	3 (0.02)	6 (0.02)	2 (0.02)	3 (0.02)	0	1 (0.4)	0	1 (0.1)	0	0	0	0	0	0
Drowning (%)	2 (0.01)	4 (0.02)	6 (0.02)	1 (0.01)	0	0	0	0	1 (0.1)	1 (0.06)	0	0	0	0	1 (0.7)
Sexual assault (%)	0	0	0	0	0	2 (1.2)	2 (0.7)	3 (0.3)	0	2 (0.12)	0	0	0	0	0
Other injuries (%)	698 (3.9)	435 (2.3)	321 (1.3)	157 (1.8)	313 (2.6)	20 (12.2)	22 (8.1)	55 (5.7)	56 (6.2)	88 (5.2)	1 (20.0)	0	2 (8.7)	2 (6.3)	4 (3.0)

^a^Due to the small number of cases, the ratio may be unstable.

## DISCUSSION

Our study demonstrated that an intentional injury in children and adolescents was common, comprising 4.9% of pediatric injury cases over the 12 years of the study period, which was basically the same as the national study from 2006 to 2014 (4.8%) in China.^18^ Studies in Pakistan have shown that intentional injuries account for 8.2% of emergency rooms and outpatient clinics injuries in children ≤18 years old.^19^ Studies in Korea have shown that 10.5% of intentional injuries among adolescents were reported in emergency rooms visits.^20^ Gallaher^8^ analyzed all injuries cases <18 years old in a traumatic center in Malawi, showing that intentional injuries accounted for 8.1%. Surveys in China have shown that the combined incidence rate of child sexual abuse was 18.2%,^21^ 13.2% of students self-reported to be threatened or injured by violence in schools.^9^ Compared with other countries and survey studies in China, the severity of the intentional injury problem may be underestimated in NISS, which is a passive reporting system, with the possibility of underreporting or misreporting. Whether NISS captured a lower level of intentional injury requires further research.

The proportion of cases of intentional injury increased with increasing age. The increase in risky behavior during adolescence and the resulting propensity for harm may be due to the developmental changes in the brain’s social emotional system.^22^ The transition to adulthood is a period of increased risk of injury.^23^ Boys reported more physical activity injuries than girls,^24^ as well as more intentional injury.^8^ Unlike unintentional injuries, girls with self-injury/suicide report rates and frequency of occurrence higher than boys,^25^ but a meta-analysis in China found no gender difference in the prevalence of non-suicidal self-injury in urban areas among secondary school students.^26^ Boys are more likely to be victims and perpetrators of homicides, and their violence/aggression usually involves weapons, such as firearms and knives.^27^ The proportions of cases of self-mutilation and suicide were similar among different genders in this study, but the proportion of boys injured by violent attacks was >80%.

Children and adolescents who came from rural areas and those who were staff or workers were more susceptible to intentional injuries. For intentional injuries, they were more likely to occur in public places and workplaces; in contrast, the most common location for unintentional injuries to occur was at home.^28^^,^^29^ In China, some rural children may drop out of school or go to work after primary or junior high school education. There is a lack of a safety net supported by institutions, especially for young people who are not registered with educational institutions or employment agencies.^30^^,^^31^ Reducing school dropouts among rural students was one of the key factors in preventing injuries.

Bullying often occurs in schools and other gathering places for children.^32^ Campus violence is one of the most concerning issues in the area of intentional injury research.^33^^,^^34^ Children between the ages of 6 and 16 are often physically assaulted.^35^ Our study found that children in junior high school and high school experienced more blunt injuries and cuts during violent attacks. Young people may experience school-based violence or bullying,^32^ suggesting that strict management systems are needed in schools. Violence prevention plans should be incorporated into health education and schools’ embedded policies.^34^

Unintentional injuries usually occur at higher frequencies in the afternoon.^36^ Children in primary school experienced the largest number of injuries from 16:00 to 20:00 (violent attacks and unintentional injuries showed the same characteristics). This finding was related to children’s daily activities. From 16:00 to 20:00, pupils are after school and going home. With school safety education received more and more attention, students’ time of off campus should be the key monitoring and protection period. Interestingly, 00:00 to 03:00 was a more common time for intentional injuries. Teenagers who were in high school frequently experienced self-mutilation or suicide from late at night to the early morning. This pattern differs from the unique time characteristics of children of other ages. High school students are in a period of preparation for an independent social life, and they expect to have an ideal and perfect self. Because of their high expectations, the ideal self and the real self are distant from each other, which may result in psychological setbacks or pessimistic and negative psychological states.^37^ In addition, depressive symptoms could come from students’ academic stress.^38^ Efforts should be made to cultivate students’ psychological endurance as a goal.

Investigations conducted in some areas have shown that falls, animal injuries, and traffic injuries were the most common causes of injuries.^4^^,^^39^ Our analysis showed that falls were the most common cause of unintentional injuries in children, while the most common means of self-harm and suicide were cuts and poisoning.^40^ Blunt injuries accounted for a large proportion of violent attacks. This feature was also related to the age distribution. For self-inflicted injuries and suicide, older children tended to choose sharp objects and drugs or pesticides, resulting in more cuts and poisoning. This finding suggests that injury prevention and control should be specific to children of different ages. In some countries, firearms were most often used in both assaults and self-inflicted injuries.^41^^,^^42^ Chinese families are not allowed to possess firearms legally, and firearms are rarely involved in injuries of children and adolescents in China.

Child victims of intentional injury have different demographic and injury patterns compared to pediatric patients who have suffered unintentional injuries. Notably, increasing age, male gender, being staff/workers or students, and coming from rural areas or late midnight injury setting were associated with intentionality. Considerable effort should be devoted to preventing that type of injury in high-risk groups. Preventative efforts should pay attention to reduce school dropouts among rural students, strengthen violence prevention plans in school, and cultivate students’ psychological endurance to reduce self-harm.

### Limitations

In our study, only nine cases of sexual assault were identified. We do not rule out the existence of concealed crimes, such as undiscovered or untreated injuries, and case identification is important.^43^ We identified a low level of self-harm/suicide injuries. It was difficult to collect cases of minor injuries if the patients did not seek medical treatment, especially for self-harm. Adolescents who were forced to seek medical help for self-injury or suicide may be self-respecting, may intentionally conceal the true intention of harm, and may self-report the injury as an unintentional injury, resulting in a misclassification of the injury. Some suicidal teenagers may choose more extreme methods, such as drowning or hanging. If the consequences are serious, they die immediately and are not sent to the hospital for rescue or treatment.

There are some other limitations of this study. NISS is a hospital-based passive injury monitoring system with certain limitations.^44^ We could not obtain the exact incidence of injuries, but up to 12 years of continuous monitoring can still provide useful information. Moreover, the household income, living arrangement, and psychological factors of children and adolescents were not collected sufficiently.

### Conclusions

It appears that intentional injuries to children and adolescents occur more often among males, rural and older children, school students, and staff or workers. The most common means of violent attacks was blunt injuries, while the most common methods of self-harm and suicide were cuts and poisoning, respectively. The distribution of the time of day of injuries differed between unintentional and intentional injuries and in different age groups. Intentional injury prevention and control should receive focused attention in high-risk groups. Preventive measures should be taken to reduce the dropout of rural students, strengthen the school’s violence prevention plan, and reduce self-harm.
