# Supplementary material for: Comparison of Intentional and Unintentional Injuries Among Chinese Children and Adolescents
Source: J Epidemiol. 2020 Dec 5;30(12):529–36. doi: 10.2188/jea.JE20190152 (PMC7661334; doi:10.2188/jea.JE20190152)
Supplement: Supplementary file 1 [file je-30-529-s001.pdf]

NO	Hospital code	Region 1=urban;0=rural	Date of Injury occur	Time of injuries occur(o’clock)	Gender 1=male;2=female	Age	Occupation 1=Preschool children;2=School students;3=Staff or workers;4=Others	Household registration 1=The city;2=The Province;3=Other provinces;4=Other countries	Intent of of injuries 1=Unintentional injuries;2=Self-mutilation/suicide;3=Violence attacks	Whether intentional injury 1=intentional injuries;0=Unintentional injuries	Mechanisms of injuries 1=Road traffic injuries;3=Falls;4=Blunt injuries;5=Firearm injuries;6=Cuts;7=Burns;8=Hanging or suffocation;9=Drowning;10=Poisoning;11=Animal bites;12=Sexual assault;13=Other injuries	Injury place 1=At home;2=Public place;3=On the road;4=Workplace;5=Others	Injured body sites 1=head;2=upper limbs;3=lower limbs;4=trunk;5=multiple parts;6=body wide;7=others	Injury ending 1=went home after treatment;2=hospitalization;3=died;4=others
1	101	1	1-Mar-07	13	1	13	4	3	2	1	6	3	1	1
2	202	0	1-Nov-10	17	1	17	4	1	1	0	1	3	2	1
3	101	1	20-Aug-09	20	1	17	4	1	1	0	4	2	2	1
4	101	1	24-Mar-15	16	1	17	4	1	1	0	11	2	2	1
5	201	1	18-Oct-13	22	2	17	4	3	1	0	13	1	2	2
6	101	1	5-Nov-07	11	1	9	4	3	2	1	6	3	1	1
7	101	1	8-Dec-14	2	2	15	4	3	2	1	3	3	3	2
8	101	1	20-Apr-13	16	1	9	4	1	1	0	3	3	4	1
9	101	1	27-Jan-06	17	1	10	4	2	1	0	3	2	1	1
10	201	1	25-Nov-09	9	1	11	4	2	1	0	11	2	3	1
11	101	1	25-Nov-12	22	1	10	4	1	1	0	6	1	3	1
12	202	0	30-Mar-12	16	1	11	4	4	1	0	7	1	2	2
13	202	0	12-Dec-10	17	1	12	4	1	1	0	6	1	2	1
14	202	0	10-Feb-10	17	1	15	4	3	1	0	13	1	2	1
15	202	0	8-Nov-10	23	1	17	4	1	1	0	6	1	2	1
16	101	1	11-Apr-17	18	1	17	4	1	1	0	6	1	2	1
17	202	0	22-Jul-11	9	1	16	4	1	1	0	1	3	4	1
18	202	0	12-Oct-13	4	1	17	3	3	3	1	6	4	5	1
19	202	0	16-Oct-14	8	1	15	3	1	1	0	3	4	1	1
20	202	0	16-Feb-16	14	1	17	3	1	1	0	6	4	3	1
21	202	0	4-Aug-16	20	1	17	3	2	1	0	6	4	1	1
22	202	0	7-Apr-08	23	1	17	3	1	1	0	10	4	7	1
23	202	0	8-May-06	17	1	17	3	2	1	0	6	4	2	1
24	202	0	14-Jul-08	23	2	17	3	1	1	0	3	4	1	1
25	201	1	21-Jun-11	2	1	17	3	1	1	0	4	4	2	2
26	201	1	8-Nov-17	12	2	17	3	1	1	0	6	4	2	1
27	202	0	6-Sep-16	21	1	15	3	3	1	0	1	3	2	1
28	202	0	13-Aug-17	0	1	16	3	1	1	0	1	3	5	1
29	202	0	21-Jul-14	15	1	16	3	3	1	0	6	3	5	1
30	202	0	4-Dec-11	22	1	16	3	1	1	0	4	3	3	2
31	201	1	6-Feb-14	14	1	16	3	2	1	0	1	3	5	1
32	101	1	7-Aug-17	8	2	16	3	2	1	0	1	3	4	1
33	202	0	8-Mar-06	12	1	17	3	1	1	0	1	3	1	1
34	202	0	2-Feb-11	11	2	17	3	3	1	0	3	3	3	1
35	201	1	17-Jan-10	12	1	17	3	1	1	0	1	3	6	1
36	101	1	26-Dec-14	17	2	17	3	1	1	0	1	3	3	1
37	201	1	25-Jan-17	21	2	17	3	2	1	0	3	3	3	1
38	101	1	6-Mar-11	19	2	15	3	1	1	0	3	2	3	1
39	202	0	15-Jul-16	1	1	17	3	1	1	0	3	2	1	1
40	202	0	1-Nov-16	17	2	17	3	2	1	0	11	2	3	1
41	101	1	8-Aug-13	16	1	17	3	1	1	0	3	2	3	1
42	201	1	1-Sep-11	7	1	17	3	3	1	0	3	2	3	1
43	101	1	19-Oct-07	20	1	17	3	3	1	0	3	2	1	1
44	101	1	10-Sep-13	12	1	17	3	1	1	0	6	2	3	1
45	101	1	26-Mar-08	12	1	17	3	3	1	0	6	2	2	1
46	101	1	31-Jan-07	14	1	17	3	3	1	0	6	2	2	1
47	101	1	6-Jul-14	9	2	17	3	1	1	0	11	2	2	1
48	201	1	24-Nov-06	14	2	17	3	1	1	0	3	2	3	1
49	101	1	7-May-11	17	2	17	3	1	1	0	13	2	3	1
50	101	1	12-Mar-12	11	1	16	3	1	1	0	6	1	2	1
51	202	0	10-Jan-17	10	1	17	3	1	1	0	3	1	2	1
52	101	1	30-Jul-14	18	1	17	3	3	1	0	11	1	2	1
53	101	1	15-Oct-13	16	1	17	3	1	1	0	11	1	2	1
54	101	1	16-Jul-14	15	1	17	3	3	1	0	6	1	2	1
55	101	1	20-Jan-12	10	2	17	3	2	1	0	11	1	2	1
56	201	1	23-Jul-17	20	2	17	3	2	1	0	11	1	2	1
57	201	1	3-Jul-17	20	2	17	3	3	1	0	11	1	2	1
58	101	1	31-Oct-10	9	2	17	3	1	1	0	6	1	2	1
59	202	0	27-Nov-12	2	2	17	2	1	1	0	4	4	1	1
60	101	1	12-Aug-14	19	1	17	2	1	1	0	3	4	1	1
61	201	1	2-Feb-09	2	1	17	2	2	1	0	6	4	1	1
62	201	1	26-Jul-15	13	2	17	2	2	1	0	6	4	4	2
63	101	1	10-Feb-14	19	2	17	2	2	1	0	7	4	3	1
64	101	1	30-May-07	7	2	17	2	1	1	0	3	4	4	1
65	201	1	27-Apr-10	8	1	15	2	2	1	0	1	3	1	1
66	201	1	19-May-17	11	1	16	2	3	1	0	1	3	5	1
67	201	1	13-Dec-07	13	1	16	2	1	1	0	1	3	1	1
68	101	1	25-Nov-13	19	1	17	2	1	1	0	1	3	1	2
69	101	1	4-Sep-15	22	1	17	2	2	1	0	1	3	5	1
70	201	1	14-Sep-11	14	1	17	2	1	1	0	1	3	1	1
71	201	1	18-Aug-09	2	1	17	2	2	1	0	1	3	2	1
72	201	1	12-Nov-09	18	1	17	2	1	1	0	1	3	4	1
73	101	1	13-May-12	14	2	17	2	2	1	0	1	3	3	1
74	101	1	13-Nov-12	2	2	17	2	1	1	0	7	3	3	1
75	201	1	18-Mar-11	10	2	17	2	1	1	0	1	3	6	1
76	201	1	2-Oct-10	12	2	17	2	1	1	0	1	3	3	1
77	101	1	26-Nov-17	1	2	17	2	3	1	0	1	3	2	1
78	101	1	14-Sep-14	8	1	15	2	1	3	1	4	2	1	2
79	202	0	17-Sep-17	20	1	16	2	2	3	1	4	2	6	1
80	101	1	2-Dec-16	23	1	16	2	2	3	1	4	2	1	4
81	202	0	29-Jun-16	14	1	17	2	1	3	1	4	2	1	2
82	101	1	25-Sep-06	9	1	14	2	3	1	0	3	2	3	1
83	101	1	18-Jun-11	17	1	15	2	1	1	0	3	2	2	1
84	101	1	27-Jan-10	17	1	15	2	1	1	0	3	2	2	1
85	202	0	13-Feb-17	21	2	16	2	2	1	0	3	2	3	1
86	101	1	3-Nov-14	14	2	16	2	1	1	0	4	2	3	1
87	202	0	17-Jun-17	3	1	17	2	2	1	0	10	2	7	1
88	202	0	1-Jun-15	15	1	17	2	3	1	0	3	2	1	1
89	202	0	27-Apr-13	17	1	17	2	1	1	0	3	2	2	1
90	202	0	26-Dec-16	22	2	17	2	3	1	0	3	2	1	1
91	202	0	17-Oct-14	10	2	17	2	3	1	0	3	2	5	1
92	201	1	26-Sep-17	20	1	17	2	2	1	0	4	2	2	1
93	101	1	18-Mar-14	21	1	17	2	3	1	0	7	2	3	1
94	201	1	30-Dec-17	16	1	17	2	2	1	0	13	2	3	1
95	101	1	15-May-14	10	1	17	2	1	1	0	4	2	5	1
96	101	1	23-Apr-14	13	1	17	2	1	1	0	4	2	2	1
97	101	1	13-Jul-10	12	1	17	2	1	1	0	4	2	2	1
98	101	1	5-Jan-15	7	1	17	2	1	1	0	3	2	4	4
99	201	1	10-Jan-15	21	1	17	2	2	1	0	3	2	3	1
100	101	1	15-Sep-13	22	1	17	2	1	1	0	3	2	3	1
101	101	1	23-Aug-13	18	1	17	2	1	1	0	3	2	2	2
102	201	1	11-Oct-11	14	1	17	2	2	1	0	3	2	1	2
103	201	1	9-Jan-11	23	1	17	2	1	1	0	3	2	2	1
104	201	1	2-Jan-07	17	1	17	2	2	1	0	3	2	3	1
105	201	1	15-Nov-17	17	1	17	2	2	1	0	3	2	3	2
106	101	1	26-Jul-13	19	1	17	2	1	1	0	6	2	2	4
107	101	1	15-Jan-08	15	1	17	2	3	1	0	6	2	2	1
108	201	1	17-Jun-13	16	2	17	2	3	1	0	13	2	3	1
109	201	1	9-Jul-17	14	2	17	2	3	1	0	4	2	4	1
110	201	1	15-Jun-10	9	2	17	2	1	1	0	11	2	2	1
111	201	1	8-Aug-13	15	2	17	2	2	1	0	3	2	1	1
112	101	1	31-May-09	16	2	17	2	1	1	0	3	2	3	1
113	201	1	19-Oct-07	19	2	17	2	2	1	0	3	2	4	1
114	101	1	9-Mar-13	22	1	13	2	1	1	0	6	1	1	1
115	202	0	7-May-16	16	1	16	2	2	1	0	11	1	2	1
116	101	1	7-Dec-14	7	1	16	2	1	1	0	3	1	5	1
117	101	1	9-Aug-12	10	1	16	2	1	1	0	6	1	2	1

118	101	1	14-May-07	19	2	16	2	3	1	0	13	1	7	1
119	201	1	30-Dec-16	12	2	16	2	1	1	0	11	1	2	1
120	101	1	15-Jun-16	12	2	16	2	1	1	0	6	1	2	1
121	202	0	22-Oct-13	18	1	17	2	1	1	0	3	1	1	1
122	202	0	30-Sep-11	19	2	17	2	1	1	0	11	1	2	1
123	202	0	5-Dec-13	13	2	17	2	1	1	0	3	1	1	1
124	202	0	22-Oct-13	12	2	17	2	1	1	0	3	1	2	2
125	201	1	17-Sep-17	8	1	17	2	1	1	0	6	1	2	1
126	101	1	5-Jul-07	20	1	17	2	1	1	0	7	1	1	1
127	101	1	11-Dec-15	23	1	17	2	1	1	0	4	1	3	1
128	201	1	28-Jun-15	8	1	17	2	2	1	0	11	1	3	1
129	101	1	26-Mar-13	15	1	17	2	2	1	0	11	1	2	1
130	101	1	18-Oct-12	19	1	17	2	1	1	0	3	1	1	2
131	101	1	22-Feb-12	21	1	17	2	2	1	0	6	1	2	2
132	201	1	26-Aug-09	20	1	17	2	1	1	0	6	1	2	1
133	201	1	24-Jul-07	2	1	17	2	2	1	0	6	1	2	1
134	101	1	13-Feb-13	16	2	17	2	1	1	0	11	1	2	1
135	101	1	22-Jul-07	15	2	17	2	1	1	0	11	1	2	1
136	101	1	22-Apr-12	23	2	17	2	1	1	0	6	1	2	1
137	202	0	31-Jan-06	15	1	14	4	1	2	1	13	5	1	1
138	101	1	12-Nov-17	12	1	17	4	1	1	0	4	5	7	1
139	101	1	13-Apr-15	19	1	16	4	1	1	0	1	3	5	1
140	202	0	28-Oct-12	17	1	17	4	1	1	0	1	3	1	2
141	202	0	5-Feb-17	18	1	17	4	1	1	0	1	3	7	1
142	202	0	1-Oct-12	0	2	17	4	1	1	0	1	3	5	2
143	202	0	26-Oct-09	15	2	17	4	1	1	0	1	3	5	1
144	201	1	24-Aug-11	11	1	17	4	1	1	0	1	3	1	1
145	202	0	20-Jun-17	3	2	16	4	2	3	1	4	2	1	1
146	201	1	18-Aug-11	19	1	15	4	2	1	0	4	2	3	1
147	202	0	8-Aug-17	10	1	17	4	2	1	0	3	2	3	1
148	202	0	17-Jul-08	19	2	17	4	4	2	1	10	1	7	2
149	101	1	27-Feb-10	20	1	14	4	2	3	1	4	5	5	1
150	101	1	15-Jun-14	14	1	15	4	1	3	1	4	5	6	2
151	101	1	19-Jan-14	9	1	15	4	2	3	1	4	5	1	1
152	202	0	4-Jun-06	4	1	16	4	1	3	1	6	5	4	1
153	101	1	5-Aug-12	22	1	16	4	2	3	1	4	5	5	2
154	101	1	4-Aug-11	4	1	16	4	2	3	1	4	5	5	1
155	101	1	4-Aug-11	0	1	16	4	2	3	1	6	5	2	1
156	202	0	17-Jan-06	1	1	17	4	1	3	1	6	5	7	2
157	101	1	13-Dec-13	20	1	17	4	2	3	1	4	5	5	1
158	101	1	15-Aug-11	23	1	17	4	1	3	1	4	5	1	1
159	101	1	6-Dec-11	3	1	17	4	3	3	1	4	5	1	1
160	101	1	17-Aug-14	22	1	17	4	2	3	1	6	5	1	1
161	101	1	7-Aug-09	1	1	17	4	1	3	1	6	5	4	2
162	101	1	2-Oct-15	22	2	17	4	1	2	1	6	5	2	1
163	202	0	25-Apr-09	1	1	16	4	1	1	0	10	5	7	1
164	101	1	26-Sep-09	17	1	16	4	2	1	0	4	5	4	1
165	101	1	16-Jan-07	8	2	16	4	3	1	0	6	5	2	1
166	202	0	19-Nov-10	23	1	17	4	1	1	0	1	5	1	1
167	202	0	1-Oct-09	0	1	17	4	1	1	0	3	5	2	2
168	202	0	8-Nov-06	1	1	17	4	2	1	0	6	5	2	2
169	101	1	7-Apr-08	15	1	17	4	1	1	0	3	5	2	1
170	202	0	13-Jun-06	4	1	15	4	1	3	1	4	4	1	1
171	202	0	23-Nov-12	0	1	15	4	3	3	1	6	4	2	1
172	101	1	24-Sep-12	2	1	15	4	2	3	1	4	4	1	1
173	201	1	11-Feb-09	5	1	15	4	3	3	1	4	4	4	2
174	101	1	8-Apr-07	2	1	15	4	3	3	1	4	4	1	1
175	202	0	4-Feb-17	4	1	16	4	1	3	1	4	4	2	1
176	202	0	21-Oct-17	23	1	16	4	3	3	1	3	4	5	1
177	202	0	23-Nov-12	0	1	16	4	3	3	1	6	4	4	1
178	202	0	18-Jul-15	5	2	16	4	3	3	1	6	4	5	2
179	202	0	24-Oct-07	21	2	16	4	3	3	1	4	4	2	2
180	202	0	3-Dec-10	16	2	16	4	3	3	1	6	4	2	2
181	101	1	2-Oct-17	2	1	16	4	1	3	1	6	4	7	1
182	101	1	2-Oct-17	2	1	16	4	1	3	1	6	4	4	1
183	101	1	12-Sep-11	15	1	16	4	3	3	1	4	4	5	1
184	101	1	12-Jul-11	1	1	16	4	2	3	1	4	4	3	1
185	101	1	16-Oct-09	0	1	16	4	2	3	1	4	4	1	2
186	101	1	18-Aug-08	18	1	16	4	1	3	1	4	4	1	1
187	101	1	7-Oct-07	3	1	16	4	1	3	1	4	4	6	1
188	101	1	22-Jan-06	23	1	16	4	2	3	1	4	4	1	1
189	101	1	2-Aug-17	22	1	16	4	1	3	1	4	4	1	1
190	101	1	2-Aug-17	22	1	16	4	2	3	1	4	4	1	1
191	202	0	10-Apr-12	18	1	17	4	1	3	1	6	4	4	1
192	202	0	19-Feb-13	23	1	17	4	1	3	1	4	4	3	1
193	202	0	20-Oct-12	20	1	17	4	3	3	1	4	4	1	1
194	202	0	13-Apr-12	2	1	17	4	1	3	1	4	4	2	2
195	202	0	5-Dec-09	21	1	17	4	1	3	1	4	4	1	1
196	202	0	19-Mar-08	20	1	17	4	1	3	1	4	4	1	1
197	202	0	7-Jan-08	17	1	17	4	1	3	1	4	4	7	1
198	202	0	25-Jul-08	0	1	17	4	1	3	1	6	4	2	2
199	202	0	25-Jul-08	0	1	17	4	1	3	1	6	4	6	1
200	202	0	13-Dec-07	4	1	17	4	1	3	1	6	4	2	2
201	202	0	30-Jun-07	21	1	17	4	1	3	1	6	4	6	1
202	202	0	23-Sep-07	2	1	17	4	1	3	1	6	4	5	2
203	202	0	28-Aug-07	3	1	17	4	1	3	1	6	4	2	2
204	202	0	25-Mar-06	4	1	17	4	1	3	1	6	4	4	2
205	202	0	4-Feb-17	4	1	17	4	1	3	1	6	4	1	1
206	202	0	4-Oct-07	2	2	17	4	1	3	1	6	4	2	1
207	201	1	25-Apr-15	14	1	17	4	2	3	1	6	4	2	1
208	201	1	20-May-13	18	1	17	4	3	3	1	13	4	1	1
209	101	1	22-Jul-15	19	1	17	4	3	3	1	4	4	1	1
210	101	1	11-May-12	1	1	17	4	2	3	1	4	4	1	1
211	101	1	1-Mar-08	20	1	17	4	1	3	1	4	4	6	1
212	101	1	22-Oct-06	4	1	17	4	1	3	1	4	4	1	1
213	101	1	6-Dec-11	3	1	17	4	2	3	1	6	4	3	1
214	101	1	8-Apr-08	23	1	17	4	2	3	1	6	4	4	2
215	101	1	24-Aug-06	5	1	17	4	3	3	1	6	4	4	2
216	101	1	24-Jul-06	0	1	17	4	1	3	1	6	4	1	2
217	202	0	8-Dec-07	1	2	16	4	1	2	1	10	4	7	2
218	101	1	3-Apr-11	2	1	17	4	2	2	1	6	4	2	1
219	202	0	25-Aug-07	9	1	14	4	3	1	0	3	4	1	2
220	201	1	4-Jan-10	0	1	14	4	2	1	0	4	4	1	1
221	202	0	9-Jun-15	19	1	15	4	2	1	0	6	4	2	1
222	202	0	11-Oct-08	2	1	15	4	3	1	0	1	4	1	1
223	202	0	20-May-10	22	2	15	4	1	1	0	3	4	1	2
224	101	1	5-Oct-17	19	1	15	4	2	1	0	6	4	3	1
225	201	1	21-Dec-10	17	1	15	4	3	1	0	4	4	2	2
226	101	1	5-Jul-11	23	1	15	4	2	1	0	11	4	3	1
227	201	1	5-Sep-16	22	1	15	4	2	1	0	3	4	1	1
228	202	0	16-Sep-14	1	1	16	4	1	1	0	10	4	6	2
229	202	0	7-Mar-10	22	1	16	4	1	1	0	10	4	7	1
230	202	0	9-Aug-07	1	1	16	4	3	1	0	7	4	2	2
231	202	0	12-Aug-06	21	1	16	4	1	1	0	11	4	7	1
232	202	0	22-Aug-16	13	1	16	4	1	1	0	6	4	2	1
233	202	0	23-Aug-10	1	1	16	4	1	1	0	6	4	2	2
234	202	0	12-Apr-08	19	1	16	4	3	1	0	6	4	2	1
235	202	0	30-Jun-07	21	1	16	4	1	1	0	6	4	6	1
236	202	0	3-Jul-14	1	2	16	4	1	1	0	6	4	2	1
237	202	0	30-Nov-06	3	2	16	4	1	1	0	10	4	7	2
238	202	0	8-Aug-07	23	2	16	4	3	1	0	7	4	2	2
239	202	0	15-Sep-15	22	2	16	4	2	1	0	11	4	1	1
240	201	1	26-Oct-14	20	1	16	4	2	1	0	10	4	7	1
241	101	1	21-Jun-16	2	1	16	4	1	1	0	4	4	1	1

242	201	1	22-Dec-10	2	1	16	4	2	1	0	4	4	2	1
243	101	1	22-Dec-13	1	1	16	4	1	1	0	6	4	2	1
244	101	1	9-Aug-07	13	1	16	4	3	1	0	6	4	2	1
245	201	1	6-Sep-06	4	1	16	4	2	1	0	6	4	1	1
246	101	1	15-Mar-17	14	1	16	4	2	1	0	6	4	2	1
247	202	0	18-Feb-14	22	1	17	4	1	1	0	10	4	7	1
248	202	0	28-Dec-16	15	1	17	4	1	1	0	6	4	1	2
249	202	0	7-May-16	19	1	17	4	1	1	0	6	4	2	2
250	202	0	19-Nov-14	0	1	17	4	3	1	0	10	4	7	1
251	202	0	12-Jan-13	12	1	17	4	1	1	0	10	4	4	2
252	202	0	8-Oct-07	4	1	17	4	1	1	0	10	4	7	1
253	202	0	7-Jan-06	0	1	17	4	1	1	0	10	4	7	2
254	202	0	19-Aug-14	1	1	17	4	1	1	0	4	4	1	1
255	202	0	30-Apr-13	10	1	17	4	1	1	0	4	4	3	1
256	202	0	14-Jul-12	22	1	17	4	3	1	0	4	4	1	1
257	202	0	19-Feb-11	1	1	17	4	1	1	0	4	4	6	1
258	202	0	29-Mar-10	9	1	17	4	4	1	0	4	4	2	2
259	202	0	10-Oct-09	21	1	17	4	2	1	0	4	4	2	1
260	202	0	7-Jul-07	21	1	17	4	1	1	0	11	4	3	1
261	202	0	30-Jul-13	17	1	17	4	1	1	0	3	4	1	2
262	202	0	9-Jul-09	12	1	17	4	1	1	0	3	4	6	1
263	202	0	15-Jul-09	20	1	17	4	1	1	0	3	4	1	2
264	202	0	24-Oct-09	23	1	17	4	1	1	0	6	4	1	1
265	202	0	22-Apr-08	21	1	17	4	2	1	0	6	4	2	2
266	202	0	23-Nov-07	22	1	17	4	1	1	0	6	4	6	2
267	202	0	31-Jan-14	5	2	17	4	1	1	0	6	4	2	1
268	202	0	13-Dec-13	2	2	17	4	1	1	0	7	4	1	1
269	202	0	28-Aug-07	23	2	17	4	3	1	0	13	4	4	1
270	202	0	23-Dec-14	7	2	17	4	1	1	0	4	4	5	1
271	202	0	16-Jul-13	10	2	17	4	3	1	0	4	4	2	2
272	202	0	14-May-13	19	2	17	4	3	1	0	4	4	2	1
273	202	0	16-Jan-08	20	2	17	4	1	1	0	4	4	4	1
274	201	1	6-Jul-17	2	1	17	4	2	1	0	10	4	6	1
275	201	1	12-Jun-17	2	1	17	4	3	1	0	3	4	1	1
276	101	1	18-Sep-13	22	1	17	4	2	1	0	10	4	7	2
277	101	1	12-Nov-15	12	1	17	4	2	1	0	4	4	1	1
278	101	1	4-Dec-11	14	1	17	4	1	1	0	4	4	2	1
279	201	1	10-Sep-10	23	1	17	4	3	1	0	4	4	1	1
280	101	1	15-Nov-06	0	1	17	4	3	1	0	4	4	1	1
281	101	1	18-Mar-15	15	1	17	4	2	1	0	3	4	1	1
282	101	1	17-May-13	21	1	17	4	2	1	0	3	4	1	1
283	101	1	19-Jan-06	9	1	17	4	3	1	0	3	4	2	2
284	101	1	22-Aug-14	20	1	17	4	2	1	0	6	4	2	1
285	201	1	5-Apr-11	23	1	17	4	3	1	0	6	4	1	2
286	201	1	21-Dec-10	16	1	17	4	2	1	0	6	4	2	1
287	101	1	28-May-08	17	1	17	4	1	1	0	6	4	2	2
288	201	1	8-Sep-06	4	1	17	4	2	1	0	6	4	1	1
289	202	0	31-Jan-13	12	1	15	4	1	3	1	4	3	1	1
290	202	0	3-Sep-15	3	1	15	4	3	3	1	4	3	5	1
291	202	0	26-Jan-11	18	1	15	4	2	3	1	6	3	6	2
292	101	1	1-Mar-06	4	1	15	4	3	3	1	6	3	5	2
293	202	0	4-Jun-16	23	1	16	4	3	3	1	6	3	2	2
294	202	0	4-Mar-12	0	1	16	4	1	3	1	6	3	4	2
295	202	0	5-Oct-06	1	1	16	4	1	3	1	6	3	1	2
296	202	0	5-Oct-06	1	1	16	4	1	3	1	6	3	1	2
297	101	1	1-Sep-10	1	1	16	4	1	3	1	4	3	5	2
298	201	1	29-Oct-17	23	1	16	4	3	3	1	3	3	5	1
299	101	1	14-Oct-11	18	1	16	4	2	3	1	6	3	2	2
300	202	0	6-Jan-14	3	1	17	4	3	3	1	4	3	1	1
301	202	0	18-Jun-11	18	1	17	4	1	3	1	13	3	1	1
302	202	0	25-Jul-07	1	1	17	4	1	3	1	1	3	6	2
303	202	0	11-Apr-13	2	1	17	4	1	3	1	4	3	4	1
304	202	0	1-Feb-11	3	1	17	4	1	3	1	6	3	4	2
305	202	0	19-Sep-07	0	1	17	4	1	3	1	6	3	5	2
306	202	0	31-May-06	3	1	17	4	1	3	1	6	3	1	1
307	202	0	30-May-13	19	2	17	4	3	3	1	4	3	1	1
308	201	1	4-Jun-12	19	1	17	4	3	3	1	13	3	5	2
309	101	1	19-May-15	4	1	17	4	3	3	1	4	3	4	1
310	201	1	19-May-07	4	1	17	4	1	3	1	4	3	6	2
311	101	1	14-Oct-07	2	1	17	4	3	3	1	4	3	6	1
312	101	1	14-Apr-07	20	1	17	4	3	3	1	4	3	1	2
313	201	1	6-Jan-06	22	1	17	4	3	3	1	4	3	1	4
314	101	1	10-Mar-09	3	1	17	4	3	3	1	6	3	2	1
315	101	1	24-Dec-08	2	1	17	4	1	3	1	6	3	4	2
316	101	1	29-Dec-07	17	1	17	4	1	3	1	6	3	7	2
317	101	1	29-Dec-07	17	1	17	4	1	3	1	6	3	7	2
318	201	1	31-May-06	22	1	17	4	1	3	1	6	3	3	2
319	202	0	6-Nov-11	15	1	14	4	1	1	0	1	3	1	2
320	202	0	3-Jan-13	23	1	14	4	1	1	0	3	3	1	1
321	202	0	9-Jan-17	21	2	14	4	2	1	0	1	3	3	1
322	201	1	18-Oct-12	13	2	14	4	2	1	0	6	3	5	1
323	202	0	1-Dec-16	0	1	15	4	2	1	0	1	3	2	2
324	202	0	28-Mar-13	21	1	15	4	2	1	0	1	3	3	1
325	202	0	1-Jan-12	1	1	15	4	2	1	0	1	3	1	2
326	202	0	10-Nov-10	14	1	15	4	3	1	0	1	3	1	2
327	202	0	21-Nov-09	21	1	15	4	1	1	0	1	3	6	1
328	202	0	18-Jul-08	22	1	15	4	1	1	0	1	3	6	2
329	202	0	8-Feb-11	17	1	15	4	1	1	0	3	3	1	1
330	202	0	16-Aug-17	18	2	15	4	1	1	0	1	3	5	2
331	202	0	28-Aug-11	20	2	15	4	1	1	0	1	3	6	2
332	202	0	28-Mar-11	1	2	15	4	1	1	0	1	3	3	1
333	201	1	15-Aug-12	21	1	15	4	3	1	0	1	3	5	1
334	101	1	8-Mar-12	19	1	15	4	2	1	0	1	3	3	1
335	201	1	19-Sep-07	4	1	15	4	2	1	0	1	3	1	1
336	201	1	22-Mar-11	20	1	15	4	3	1	0	1	3	4	1
337	101	1	23-Jul-06	7	1	15	4	3	1	0	3	3	4	1
338	101	1	3-Jul-11	1	1	15	4	3	1	0	6	3	2	1
339	101	1	10-Mar-11	18	2	15	4	2	1	0	1	3	3	1
340	202	0	31-Jan-14	23	1	16	4	3	1	0	1	3	3	2
341	202	0	24-Nov-13	21	1	16	4	3	1	0	1	3	1	2
342	202	0	5-Feb-16	20	1	16	4	3	1	0	1	3	3	2
343	202	0	18-Jun-13	16	1	16	4	2	1	0	1	3	4	2
344	202	0	12-Jan-12	16	1	16	4	1	1	0	1	3	3	2
345	202	0	12-Feb-12	19	1	16	4	2	1	0	1	3	1	2
346	202	0	9-Sep-16	10	1	16	4	1	1	0	1	3	2	1
347	202	0	13-Dec-15	23	1	16	4	1	1	0	1	3	5	2
348	202	0	30-May-15	22	1	16	4	2	1	0	1	3	5	1
349	202	0	2-Feb-14	19	1	16	4	1	1	0	1	3	1	2
350	202	0	25-Sep-13	12	1	16	4	2	1	0	1	3	5	1
351	202	0	5-Jun-13	23	1	16	4	3	1	0	1	3	1	2
352	202	0	9-Aug-13	12	1	16	4	1	1	0	1	3	2	1
353	202	0	24-Jun-13	3	1	16	4	1	1	0	1	3	2	2
354	202	0	8-Aug-12	23	1	16	4	1	1	0	1	3	6	2
355	202	0	3-Jan-12	0	1	16	4	2	1	0	1	3	5	2
356	202	0	12-Aug-14	14	1	16	4	2	1	0	13	3	1	1
357	202	0	7-Aug-08	19	1	16	4	1	1	0	13	3	4	2
358	202	0	20-Nov-11	16	1	16	4	3	1	0	1	3	4	2
359	202	0	11-Jul-11	21	1	16	4	1	1	0	1	3	6	1
360	202	0	7-Oct-10	21	1	16	4	1	1	0	1	3	6	2
361	202	0	17-May-10	8	1	16	4	3	1	0	1	3	3	1
362	202	0	21-Aug-10	3	1	16	4	1	1	0	1	3	6	1
363	202	0	4-Dec-09	22	1	16	4	1	1	0	1	3	2	2
364	202	0	20-Feb-07	20	1	16	4	3	1	0	1	3	1	2
365	202	0	6-Feb-06	23	1	16	4	3	1	0	1	3	1	1

366	202	0	24-Sep-06	10	1	16	4	3	1	0	1	3	1	1
367	202	0	19-Jun-11	9	1	16	4	1	1	0	4	3	1	1
368	202	0	30-Jul-16	4	1	16	4	3	1	0	3	3	1	1
369	202	0	12-Mar-16	2	1	16	4	1	1	0	3	3	3	1
370	202	0	5-Sep-16	10	1	16	4	3	1	0	3	3	3	1
371	202	0	8-Nov-15	18	1	16	4	1	1	0	3	3	5	1
372	202	0	3-Mar-14	12	1	16	4	1	1	0	3	3	2	1
373	202	0	29-May-11	4	1	16	4	1	1	0	3	3	1	2
374	202	0	12-May-13	16	1	16	4	1	1	0	6	3	3	1
375	202	0	9-Nov-16	8	2	16	4	3	1	0	1	3	3	1
376	202	0	13-Oct-15	3	2	16	4	1	1	0	1	3	1	2
377	202	0	18-Oct-14	5	2	16	4	3	1	0	1	3	5	1
378	202	0	11-Jul-14	0	2	16	4	1	1	0	1	3	1	2
379	202	0	20-Aug-12	0	2	16	4	2	1	0	1	3	2	2
380	202	0	15-Jan-12	19	2	16	4	1	1	0	1	3	4	1
381	202	0	11-Jan-12	3	2	16	4	2	1	0	1	3	5	2
382	202	0	28-Aug-11	20	2	16	4	1	1	0	1	3	6	2
383	202	0	1-Oct-10	3	2	16	4	1	1	0	1	3	6	2
384	202	0	2-Jul-09	2	2	16	4	1	1	0	1	3	1	1
385	202	0	11-Nov-06	3	2	16	4	1	1	0	1	3	1	1
386	202	0	20-Jan-06	14	2	16	4	3	1	0	1	3	1	2
387	202	0	3-Oct-06	4	2	16	4	1	1	0	1	3	3	1
388	202	0	24-Jul-06	11	2	16	4	3	1	0	1	3	3	1
389	202	0	29-Oct-06	21	2	16	4	2	1	0	1	3	1	1
390	202	0	30-Jul-16	4	2	16	4	3	1	0	3	3	3	1
391	201	1	13-Sep-14	20	1	16	4	2	1	0	1	3	1	1
392	201	1	10-Apr-12	11	1	16	4	2	1	0	1	3	3	1
393	101	1	30-Jun-15	1	1	16	4	2	1	0	1	3	3	1
394	201	1	20-Oct-15	22	1	16	4	2	1	0	3	3	5	1
395	101	1	4-May-11	3	1	16	4	1	1	0	1	3	1	2
396	101	1	27-Mar-11	1	1	16	4	3	1	0	1	3	5	1
397	201	1	21-May-11	1	1	16	4	2	1	0	1	3	3	2
398	101	1	3-Nov-09	8	1	16	4	2	1	0	1	3	5	2
399	201	1	16-Jan-08	19	1	16	4	3	1	0	1	3	1	2
400	201	1	18-Jul-11	5	1	16	4	2	1	0	1	3	3	1
401	101	1	14-Jul-08	23	1	16	4	3	1	0	4	3	5	1
402	101	1	22-Sep-08	20	1	16	4	2	1	0	11	3	3	1
403	201	1	10-Mar-08	21	1	16	4	3	1	0	6	3	1	1
404	201	1	30-Sep-15	0	2	16	4	3	1	0	1	3	3	1
405	201	1	30-Sep-15	0	2	16	4	3	1	0	1	3	2	1
406	201	1	29-Aug-09	21	2	16	4	1	1	0	1	3	2	1
407	101	1	18-Aug-08	20	2	16	4	3	1	0	1	3	1	2
408	101	1	17-Oct-06	0	2	16	4	3	1	0	1	3	2	1
409	202	0	19-Oct-13	9	1	17	4	1	1	0	1	3	5	2
410	202	0	19-Oct-13	9	1	17	4	2	1	0	1	3	5	1
411	202	0	15-Nov-15	21	1	17	4	2	1	0	1	3	2	1
412	202	0	1-Sep-14	16	1	17	4	3	1	0	1	3	1	2
413	202	0	19-Aug-13	2	1	17	4	1	1	0	1	3	3	2
414	202	0	18-Aug-13	22	1	17	4	3	1	0	1	3	1	1
415	202	0	29-Aug-15	22	1	17	4	3	1	0	1	3	1	1
416	202	0	30-Jul-13	17	1	17	4	3	1	0	1	3	5	2
417	202	0	12-Oct-15	4	1	17	4	2	1	0	1	3	6	1
418	202	0	4-Aug-15	23	1	17	4	1	1	0	1	3	5	1
419	202	0	16-Dec-12	12	1	17	4	3	1	0	1	3	4	2
420	202	0	11-Sep-15	13	1	17	4	3	1	0	1	3	5	1
421	202	0	29-May-16	17	1	17	4	1	1	0	1	3	1	2
422	202	0	7-Jun-16	1	1	17	4	1	1	0	1	3	2	1
423	202	0	29-Aug-15	17	1	17	4	1	1	0	1	3	1	1
424	202	0	20-Jul-14	13	1	17	4	3	1	0	1	3	1	2
425	202	0	13-Mar-14	14	1	17	4	3	1	0	1	3	3	1
426	202	0	15-Sep-14	0	1	17	4	2	1	0	1	3	3	1
427	202	0	21-Aug-14	1	1	17	4	1	1	0	1	3	5	2
428	202	0	6-Jul-14	1	1	17	4	1	1	0	1	3	3	1
429	202	0	29-Dec-13	0	1	17	4	1	1	0	1	3	3	1
430	202	0	26-Nov-13	1	1	17	4	2	1	0	1	3	3	1
431	202	0	9-Oct-13	4	1	17	4	1	1	0	1	3	1	2
432	202	0	26-Jun-13	23	1	17	4	3	1	0	1	3	2	1
433	202	0	19-Mar-13	2	1	17	4	3	1	0	1	3	3	1
434	202	0	16-Feb-13	20	1	17	4	1	1	0	1	3	3	1
435	202	0	22-Aug-12	0	1	17	4	1	1	0	1	3	5	2
436	202	0	22-Apr-12	15	1	17	4	1	1	0	1	3	1	1
437	202	0	9-Mar-12	3	1	17	4	1	1	0	1	3	3	1
438	202	0	12-Jan-12	16	1	17	4	1	1	0	1	3	5	1
439	202	0	30-Jan-17	3	1	17	4	1	1	0	1	3	1	1
440	202	0	25-May-17	12	1	17	4	1	1	0	1	3	1	2
441	202	0	5-Jul-17	14	1	17	4	1	1	0	1	3	5	1
442	202	0	5-Jul-17	13	1	17	4	1	1	0	1	3	5	1
443	202	0	17-Sep-17	21	1	17	4	1	1	0	1	3	3	2
444	202	0	11-Apr-14	22	1	17	4	2	1	0	6	3	3	1
445	202	0	16-Jul-12	17	1	17	4	1	1	0	1	3	6	1
446	202	0	11-Jan-12	3	1	17	4	2	1	0	1	3	3	2
447	202	0	19-Nov-11	16	1	17	4	3	1	0	1	3	5	2
448	202	0	17-May-11	15	1	17	4	1	1	0	1	3	5	2
449	202	0	11-Sep-11	23	1	17	4	1	1	0	1	3	6	1
450	202	0	7-Jun-11	13	1	17	4	1	1	0	1	3	3	2
451	202	0	25-Jun-11	17	1	17	4	1	1	0	1	3	5	1
452	202	0	22-Oct-11	0	1	17	4	1	1	0	1	3	1	2
453	202	0	29-Jul-10	20	1	17	4	1	1	0	1	3	6	2
454	202	0	9-Jul-10	0	1	17	4	2	1	0	1	3	6	2
455	202	0	18-Mar-10	22	1	17	4	1	1	0	1	3	6	1
456	202	0	29-Sep-10	17	1	17	4	1	1	0	1	3	5	1
457	202	0	13-Apr-10	0	1	17	4	1	1	0	1	3	1	2
458	202	0	15-Aug-10	18	1	17	4	1	1	0	1	3	1	2
459	202	0	11-May-10	2	1	17	4	1	1	0	1	3	1	2
460	202	0	7-Jun-10	9	1	17	4	1	1	0	1	3	1	1
461	202	0	14-Feb-10	11	1	17	4	1	1	0	1	3	3	1
462	202	0	21-Aug-10	3	1	17	4	1	1	0	1	3	6	1
463	202	0	21-Jun-09	23	1	17	4	1	1	0	1	3	6	2
464	202	0	30-Mar-09	14	1	17	4	3	1	0	1	3	2	2
465	202	0	22-Nov-09	5	1	17	4	1	1	0	1	3	1	2
466	202	0	1-Dec-09	23	1	17	4	1	1	0	1	3	1	2
467	202	0	22-Nov-09	5	1	17	4	1	1	0	1	3	4	2
468	202	0	3-Oct-09	0	1	17	4	1	1	0	1	3	7	2
469	202	0	2-Jan-08	19	1	17	4	1	1	0	1	3	1	2
470	202	0	28-Dec-08	5	1	17	4	1	1	0	1	3	1	1
471	202	0	13-Jan-08	1	1	17	4	1	1	0	1	3	3	2
472	202	0	4-Jan-08	1	1	17	4	1	1	0	1	3	1	1
473	202	0	20-Dec-07	19	1	17	4	1	1	0	1	3	1	2
474	202	0	14-Aug-07	21	1	17	4	2	1	0	1	3	2	1
475	202	0	25-Feb-07	1	1	17	4	1	1	0	1	3	5	1
476	202	0	22-Oct-07	7	1	17	4	2	1	0	1	3	6	1
477	202	0	15-Jul-07	22	1	17	4	3	1	0	1	3	6	1
478	202	0	27-Feb-07	13	1	17	4	1	1	0	1	3	1	2
479	202	0	27-Sep-07	20	1	17	4	3	1	0	1	3	1	2
480	202	0	13-Dec-07	5	1	17	4	3	1	0	1	3	1	1
481	202	0	29-Dec-07	21	1	17	4	1	1	0	1	3	1	1
482	202	0	11-Feb-07	0	1	17	4	1	1	0	1	3	1	1
483	202	0	4-Feb-07	20	1	17	4	1	1	0	1	3	3	2
484	202	0	18-Nov-07	5	1	17	4	2	1	0	1	3	3	1
485	202	0	18-Dec-07	3	1	17	4	1	1	0	1	3	6	1
486	202	0	20-Dec-07	20	1	17	4	1	1	0	1	3	3	1
487	202	0	3-Aug-07	10	1	17	4	2	1	0	1	3	5	2
488	202	0	25-May-07	23	1	17	4	2	1	0	1	3	6	2
489	202	0	12-Sep-07	7	1	17	4	1	1	0	1	3	1	2

490	202	0	5-Mar-06	6	1	17	4	2	1	0	1	3	6	1
491	202	0	8-Dec-06	11	1	17	4	3	1	0	1	3	1	2
492	202	0	13-Jan-06	5	1	17	4	1	1	0	1	3	1	2
493	202	0	16-Dec-06	2	1	17	4	1	1	0	1	3	3	1
494	202	0	13-Oct-06	8	1	17	4	1	1	0	1	3	3	1
495	202	0	15-Jun-13	16	1	17	4	1	1	0	11	3	3	1
496	202	0	25-Oct-16	1	1	17	4	3	1	0	3	3	1	1
497	202	0	21-Jul-12	0	1	17	4	1	1	0	3	3	2	2
498	202	0	23-Aug-09	0	1	17	4	1	1	0	3	3	6	1
499	202	0	10-Dec-08	21	1	17	4	1	1	0	3	3	2	2
500	202	0	17-Mar-07	13	1	17	4	2	1	0	3	3	1	1
501	202	0	11-Sep-07	10	1	17	4	1	1	0	3	3	2	1
502	202	0	25-Feb-17	19	1	17	4	3	1	0	6	3	3	1
503	202	0	18-Sep-10	0	1	17	4	1	1	0	13	3	2	1
504	202	0	25-May-13	23	2	17	4	1	1	0	1	3	1	1
505	202	0	13-Nov-16	0	2	17	4	1	1	0	1	3	5	2
506	202	0	17-Apr-16	5	2	17	4	1	1	0	1	3	5	1
507	202	0	17-Oct-15	0	2	17	4	3	1	0	1	3	2	1
508	202	0	10-Oct-14	3	2	17	4	1	1	0	1	3	5	1
509	202	0	4-Jun-13	17	2	17	4	3	1	0	1	3	6	1
510	202	0	13-Apr-13	20	2	17	4	1	1	0	1	3	1	1
511	202	0	14-May-13	23	2	17	4	2	1	0	1	3	4	2
512	202	0	22-Jun-12	22	2	17	4	1	1	0	1	3	6	2
513	202	0	11-Mar-12	3	2	17	4	1	1	0	1	3	1	1
514	202	0	7-Dec-12	15	2	17	4	1	1	0	1	3	5	1
515	202	0	5-Jun-11	21	2	17	4	1	1	0	1	3	5	2
516	202	0	7-Sep-10	4	2	17	4	1	1	0	1	3	5	1
517	202	0	25-Feb-10	23	2	17	4	1	1	0	1	3	1	1
518	202	0	23-Jan-10	19	2	17	4	1	1	0	1	3	2	2
519	202	0	30-Mar-09	14	2	17	4	1	1	0	1	3	2	2
520	202	0	13-Sep-09	4	2	17	4	1	1	0	1	3	1	2
521	202	0	24-Jul-09	0	2	17	4	1	1	0	1	3	3	1
522	202	0	30-Oct-08	18	2	17	4	1	1	0	1	3	2	1
523	202	0	20-Jun-08	13	2	17	4	1	1	0	1	3	2	1
524	202	0	12-Sep-07	3	2	17	4	1	1	0	1	3	5	1
525	202	0	9-Oct-07	2	2	17	4	2	1	0	1	3	6	1
526	202	0	19-Aug-07	0	2	17	4	2	1	0	1	3	3	2
527	202	0	10-Nov-07	18	2	17	4	3	1	0	1	3	3	1
528	202	0	29-Dec-06	22	2	17	4	2	1	0	1	3	1	1
529	202	0	30-Jan-06	14	2	17	4	1	1	0	1	3	3	1
530	202	0	27-Dec-06	22	2	17	4	1	1	0	1	3	3	2
531	202	0	1-Jan-06	15	2	17	4	3	1	0	1	3	1	2
532	202	0	28-Jan-07	21	2	17	4	3	1	0	1	3	3	2
533	202	0	9-Oct-15	4	2	17	4	3	1	0	3	3	5	1
534	202	0	15-Apr-14	8	2	17	4	2	1	0	3	3	3	1
535	202	0	16-Oct-07	0	2	17	4	1	1	0	6	3	2	1
536	101	1	12-Apr-12	12	1	17	4	1	1	0	1	3	5	1
537	101	1	22-Apr-15	9	1	17	4	3	1	0	1	3	4	2
538	201	1	21-Aug-16	18	1	17	4	2	1	0	1	3	5	2
539	101	1	8-Jan-12	11	1	17	4	2	1	0	1	3	4	2
540	101	1	14-Aug-16	2	1	17	4	1	1	0	1	3	4	2
541	201	1	30-Sep-15	0	1	17	4	3	1	0	1	3	5	1
542	201	1	10-Aug-12	15	1	17	4	1	1	0	1	3	3	1
543	101	1	9-Oct-15	1	1	17	4	1	1	0	3	3	3	1
544	101	1	19-Sep-12	23	1	17	4	1	1	0	1	3	1	2
545	101	1	22-Oct-11	23	1	17	4	3	1	0	1	3	5	2
546	201	1	23-Jan-11	18	1	17	4	2	1	0	1	3	6	1
547	101	1	19-Sep-11	0	1	17	4	2	1	0	1	3	1	1
548	201	1	21-May-11	1	1	17	4	2	1	0	1	3	3	2
549	101	1	28-Apr-10	17	1	17	4	2	1	0	1	3	5	1
550	101	1	12-Jul-09	18	1	17	4	1	1	0	1	3	5	1
551	101	1	14-Feb-09	6	1	17	4	3	1	0	1	3	1	1
552	201	1	14-May-09	22	1	17	4	3	1	0	1	3	4	2
553	101	1	28-May-07	0	1	17	4	1	1	0	1	3	1	1
554	101	1	28-May-07	0	1	17	4	1	1	0	1	3	1	1
555	101	1	21-Nov-06	18	1	17	4	3	1	0	1	3	5	1
556	101	1	19-Aug-06	21	1	17	4	1	1	0	1	3	1	2
557	101	1	21-Sep-06	15	1	17	4	2	1	0	1	3	5	1
558	101	1	2-Oct-06	21	1	17	4	3	1	0	1	3	1	2
559	101	1	6-Aug-09	18	1	17	4	2	1	0	11	3	3	2
560	101	1	2-Jul-13	22	1	17	4	1	1	0	3	3	4	1
561	101	1	5-Sep-08	22	1	17	4	2	1	0	3	3	2	1
562	101	1	6-Apr-17	22	1	17	4	3	1	0	3	3	3	1
563	101	1	10-Aug-14	4	1	17	4	2	1	0	6	3	3	1
564	201	1	8-Jun-12	8	2	17	4	3	1	0	1	3	3	2
565	201	1	23-Aug-12	20	2	17	4	3	1	0	1	3	1	1
566	201	1	23-Aug-12	20	2	17	4	3	1	0	1	3	2	1
567	201	1	11-Feb-13	1	2	17	4	1	1	0	1	3	3	1
568	201	1	8-Feb-11	23	2	17	4	2	1	0	1	3	4	2
569	101	1	20-Nov-11	19	2	17	4	3	1	0	1	3	1	1
570	101	1	10-Nov-11	6	2	17	4	3	1	0	1	3	3	1
571	201	1	6-Jun-09	10	2	17	4	3	1	0	1	3	1	2
572	201	1	12-Jan-06	21	2	17	4	3	1	0	1	3	3	1
573	101	1	8-Mar-17	19	2	17	4	2	1	0	1	3	6	1
574	101	1	3-Jul-09	20	2	17	4	1	1	0	1	3	5	1
575	201	1	30-Jan-07	16	2	17	4	3	1	0	1	3	6	1
576	101	1	14-Jan-13	20	2	17	4	1	1	0	11	3	2	1
577	101	1	12-Aug-10	22	2	17	4	1	1	0	3	3	3	1
578	202	0	29-Sep-13	15	1	14	4	1	3	1	4	2	1	1
579	202	0	28-Jun-10	19	1	14	4	1	3	1	4	2	6	1
580	202	0	29-Sep-07	22	1	14	4	3	3	1	6	2	4	1
581	202	0	30-Aug-09	17	1	15	4	1	3	1	4	2	1	2
582	202	0	29-Jun-17	5	1	15	4	4	3	1	4	2	1	1
583	101	1	17-May-15	20	1	15	4	2	3	1	4	2	1	1
584	101	1	2-Mar-13	22	1	15	4	3	3	1	4	2	1	2
585	101	1	11-Nov-06	20	1	15	4	2	3	1	4	2	5	1
586	101	1	9-Jan-11	21	1	15	4	2	3	1	6	2	3	2
587	201	1	23-Aug-12	23	2	15	4	2	3	1	6	2	6	2
588	202	0	9-Dec-14	23	1	16	4	3	3	1	6	2	3	1
589	202	0	21-Jul-17	21	1	16	4	2	3	1	6	2	2	2
590	202	0	2-Nov-13	14	1	16	4	3	3	1	4	2	1	1
591	202	0	23-Dec-10	16	1	16	4	3	3	1	4	2	6	2
592	202	0	21-Dec-09	11	1	16	4	1	3	1	4	2	6	1
593	202	0	11-Sep-09	23	1	16	4	1	3	1	4	2	5	1
594	202	0	6-Jul-08	1	1	16	4	3	3	1	4	2	5	1
595	202	0	19-Oct-13	20	1	16	4	1	3	1	6	2	5	2
596	202	0	28-Nov-10	18	1	16	4	1	3	1	6	2	4	2
597	202	0	25-Jun-09	23	1	16	4	3	3	1	6	2	2	2
598	202	0	18-Sep-07	18	1	16	4	1	3	1	6	2	3	2
599	202	0	9-Sep-06	0	1	16	4	1	3	1	6	2	1	1
600	202	0	25-Dec-06	0	1	16	4	1	3	1	6	2	6	2
601	202	0	7-Feb-07	5	2	16	4	3	3	1	6	2	1	1
602	101	1	21-Dec-14	11	1	16	4	2	3	1	4	2	1	1
603	101	1	21-Dec-14	11	1	16	4	2	3	1	4	2	1	1
604	201	1	24-Feb-13	1	1	16	4	2	3	1	4	2	1	1
605	101	1	26-Jul-12	1	1	16	4	1	3	1	4	2	4	1
606	101	1	27-Apr-10	1	1	16	4	3	3	1	4	2	1	1
607	101	1	28-Oct-13	19	1	16	4	1	3	1	6	2	5	2
608	101	1	16-Mar-16	16	2	16	4	2	3	1	4	2	1	1
609	202	0	22-Sep-13	10	1	17	4	1	3	1	6	2	4	2
610	202	0	23-Mar-07	22	1	17	4	1	3	1	10	2	7	2
611	202	0	7-Sep-14	23	1	17	4	3	3	1	4	2	1	1
612	202	0	21-Jul-13	20	1	17	4	1	3	1	4	2	1	1
613	202	0	17-Feb-13	2	1	17	4	1	3	1	4	2	1	1

614	202	0	27-Mar-13	10	1	17	4	1	3	1	4	2	4	2
615	202	0	6-Jun-13	21	1	17	4	1	3	1	4	2	4	1
616	202	0	13-Mar-08	21	1	17	4	1	3	1	4	2	1	2
617	202	0	13-Mar-08	21	1	17	4	1	3	1	4	2	1	2
618	202	0	11-Jan-07	19	1	17	4	3	3	1	4	2	1	2
619	202	0	20-Jul-07	1	1	17	4	2	3	1	4	2	2	2
620	202	0	29-Oct-13	1	1	17	4	2	3	1	6	2	2	2
621	202	0	4-Jul-10	13	1	17	4	3	3	1	6	2	1	1
622	202	0	16-May-07	15	1	17	4	1	3	1	6	2	6	2
623	202	0	16-Jan-07	2	1	17	4	1	3	1	6	2	1	1
624	202	0	12-Mar-07	0	1	17	4	3	3	1	6	2	3	2
625	202	0	6-Feb-06	19	1	17	4	1	3	1	6	2	1	1
626	202	0	27-Jun-06	23	1	17	4	3	3	1	6	2	4	2
627	201	1	29-Jul-13	1	1	17	4	3	3	1	6	2	2	1
628	101	1	18-Jul-06	22	1	17	4	2	3	1	13	2	4	1
629	101	1	18-Jul-06	22	1	17	4	3	3	1	13	2	4	1
630	101	1	7-Jun-13	1	1	17	4	3	3	1	4	2	1	1
631	101	1	26-Jan-12	10	1	17	4	1	3	1	4	2	5	1
632	201	1	9-Jun-10	0	1	17	4	2	3	1	4	2	1	1
633	201	1	7-May-09	17	1	17	4	3	3	1	4	2	1	1
634	201	1	26-Mar-08	10	1	17	4	3	3	1	4	2	1	1
635	201	1	7-Apr-08	1	1	17	4	2	3	1	4	2	1	1
636	101	1	15-May-07	19	1	17	4	2	3	1	4	2	1	1
637	101	1	16-Jan-15	21	1	17	4	2	3	1	6	2	5	1
638	201	1	16-May-11	4	1	17	4	1	3	1	6	2	6	1
639	101	1	15-Dec-10	1	1	17	4	2	3	1	6	2	1	1
640	101	1	20-Jul-09	5	1	17	4	2	3	1	6	2	4	3
641	101	1	8-Jun-08	19	1	17	4	3	3	1	6	2	2	1
642	201	1	10-Mar-06	23	1	17	4	3	3	1	6	2	1	1
643	201	1	1-Dec-12	3	2	17	4	3	3	1	4	2	1	1
644	201	1	21-Nov-16	7	1	16	4	3	2	1	3	2	4	2
645	202	0	30-Mar-13	23	1	17	4	3	2	1	4	2	2	1
646	202	0	17-Mar-07	6	2	17	4	1	2	1	10	2	7	2
647	201	1	5-Dec-17	8	1	17	4	3	2	1	4	2	1	1
648	202	0	12-Nov-13	14	1	15	4	3	1	0	1	2	1	1
649	202	0	31-Jan-13	13	1	15	4	1	1	0	4	2	1	1
650	202	0	8-Mar-10	21	1	15	4	1	1	0	1	2	2	2
651	202	0	11-Apr-08	21	1	15	4	1	1	0	4	2	1	1
652	202	0	6-Nov-08	13	1	15	4	3	1	0	3	2	2	1
653	202	0	8-Jul-17	1	1	15	4	1	1	0	3	2	3	2
654	201	1	21-Aug-14	3	1	15	4	3	1	0	10	2	7	1
655	201	1	23-Sep-12	19	1	15	4	2	1	0	6	2	1	1
656	202	0	1-Jan-16	1	1	16	4	3	1	0	4	2	1	2
657	202	0	27-Jul-13	2	1	16	4	3	1	0	10	2	7	2
658	202	0	2-Feb-13	19	1	16	4	3	1	0	3	2	1	1
659	202	0	15-Jun-17	15	1	16	4	2	1	0	3	2	2	1
660	202	0	18-Aug-11	4	2	16	4	1	1	0	5	2	1	2
661	101	1	15-Jul-09	1	1	16	4	2	1	0	4	2	3	1
662	101	1	8-Aug-13	1	1	16	4	3	1	0	11	2	3	1
663	101	1	6-Nov-12	7	1	16	4	1	1	0	11	2	2	1
664	101	1	5-Nov-16	10	1	16	4	2	1	0	3	2	3	1
665	201	1	29-Mar-14	17	1	16	4	2	1	0	3	2	2	1
666	101	1	12-Mar-12	22	1	16	4	2	1	0	3	2	3	1
667	201	1	4-Sep-12	22	1	16	4	3	1	0	3	2	1	1
668	101	1	8-Jan-12	22	1	16	4	1	1	0	3	2	3	4
669	101	1	2-Jul-12	10	1	16	4	3	1	0	3	2	2	1
670	201	1	20-Apr-12	17	1	16	4	2	1	0	3	2	2	1
671	201	1	29-Nov-07	12	1	16	4	3	1	0	3	2	1	2
672	101	1	19-Dec-10	23	1	16	4	2	1	0	6	2	3	1
673	101	1	24-Mar-13	12	2	16	4	2	1	0	3	2	1	1
674	202	0	23-Sep-13	22	1	17	4	1	1	0	10	2	7	2
675	202	0	25-Jun-16	0	1	17	4	3	1	0	10	2	7	2
676	202	0	15-Dec-12	13	1	17	4	1	1	0	1	2	1	2
677	202	0	24-Feb-14	0	1	17	4	1	1	0	4	2	1	1
678	202	0	22-Feb-11	17	1	17	4	1	1	0	4	2	1	1
679	202	0	5-Oct-16	6	1	17	4	1	1	0	3	2	5	1
680	202	0	17-Feb-16	0	1	17	4	1	1	0	3	2	1	2
681	202	0	23-Sep-16	16	1	17	4	3	1	0	3	2	4	1
682	202	0	11-Oct-15	3	1	17	4	3	1	0	3	2	5	1
683	202	0	17-Jun-13	11	1	17	4	1	1	0	3	2	1	1
684	202	0	27-Aug-13	20	1	17	4	1	1	0	3	2	3	1
685	202	0	18-Jun-11	17	1	17	4	1	1	0	3	2	2	1
686	202	0	13-Jan-08	0	1	17	4	1	1	0	3	2	3	2
687	202	0	18-Feb-17	15	1	17	4	3	1	0	3	2	2	1
688	202	0	4-Jul-09	0	1	17	4	1	1	0	6	2	2	2
689	202	0	24-Oct-07	22	1	17	4	1	1	0	6	2	3	1
690	202	0	12-Jul-08	21	1	17	4	1	1	0	13	2	2	1
691	202	0	23-May-15	2	2	17	4	2	1	0	10	2	6	1
692	202	0	9-Jan-17	0	2	17	4	2	1	0	6	2	4	2
693	202	0	15-Dec-13	1	2	17	4	1	1	0	3	2	2	2
694	202	0	13-Dec-12	11	2	17	4	3	1	0	3	2	2	2
695	202	0	14-Apr-10	22	2	17	4	1	1	0	3	2	3	2
696	202	0	7-Jul-11	0	2	17	4	1	1	0	6	2	2	2
697	201	1	22-Jul-14	22	1	17	4	2	1	0	10	2	7	1
698	201	1	7-Jul-13	16	1	17	4	3	1	0	4	2	3	1
699	201	1	26-Oct-12	20	1	17	4	2	1	0	4	2	1	1
700	201	1	26-Oct-12	20	1	17	4	2	1	0	4	2	1	1
701	201	1	26-Aug-10	1	1	17	4	3	1	0	4	2	1	1
702	101	1	24-Apr-15	14	1	17	4	2	1	0	11	2	3	1
703	201	1	30-Jul-07	17	1	17	4	2	1	0	11	2	2	1
704	101	1	13-Jun-14	8	1	17	4	3	1	0	3	2	3	1
705	101	1	26-Nov-13	2	1	17	4	2	1	0	3	2	1	1
706	101	1	29-May-13	16	1	17	4	3	1	0	3	2	3	1
707	101	1	1-Sep-12	23	1	17	4	3	1	0	3	2	2	4
708	101	1	6-Oct-07	19	1	17	4	3	1	0	3	2	4	1
709	201	1	25-Sep-06	21	1	17	4	2	1	0	3	2	2	1
710	101	1	22-Feb-17	20	1	17	4	2	1	0	3	2	1	1
711	101	1	11-Apr-15	20	1	17	4	1	1	0	6	2	2	2
712	201	1	27-Nov-13	21	1	17	4	3	1	0	6	2	2	1
713	201	1	4-Dec-10	1	1	17	4	2	1	0	6	2	1	1
714	201	1	15-Sep-08	4	1	17	4	3	1	0	6	2	4	1
715	201	1	7-Apr-16	19	2	17	4	3	1	0	10	2	6	1
716	201	1	14-Sep-06	17	2	17	4	3	1	0	3	2	6	1
717	101	1	3-Oct-06	23	2	17	4	1	1	0	3	2	3	1
718	101	1	23-Nov-17	18	2	17	4	3	1	0	3	2	1	1
719	202	0	28-May-10	3	1	17	4	1	3	1	4	1	1	1
720	202	0	5-Nov-09	10	1	17	4	1	3	1	6	1	4	2
721	201	1	21-Feb-11	22	2	17	4	3	3	1	6	1	2	1
722	202	0	18-Oct-10	9	2	14	4	2	2	1	6	1	7	2
723	202	0	30-Dec-09	0	2	16	4	2	2	1	10	1	7	2
724	101	1	3-Feb-08	14	1	16	4	3	2	1	6	1	2	1
725	201	1	15-Mar-15	16	2	16	4	3	2	1	10	1	6	2
726	202	0	18-Jun-14	21	1	17	4	2	2	1	6	1	2	2
727	202	0	4-May-07	1	1	17	4	3	2	1	10	1	7	2
728	202	0	14-Aug-07	18	2	17	4	3	2	1	10	1	7	2
729	202	0	14-Sep-07	2	2	17	4	1	2	1	10	1	7	1
730	101	1	22-Nov-15	19	1	17	4	2	2	1	6	1	4	2
731	201	1	5-Oct-12	14	1	17	4	3	2	1	6	1	2	1
732	101	1	26-Oct-06	23	2	17	4	3	2	1	6	1	2	1
733	202	0	11-Apr-07	19	1	14	4	3	1	0	3	1	2	2
734	202	0	17-Jul-07	17	2	14	4	3	1	0	6	1	2	2
735	101	1	23-Sep-10	22	2	14	4	2	1	0	6	1	2	1
736	202	0	31-May-09	21	1	15	4	1	1	0	3	1	1	2
737	101	1	22-Mar-12	4	1	15	4	3	1	0	6	1	2	1

738	101	1	1-Jun-10	7	1	15	4	2	1	0	13	1	2	1
739	201	1	20-Nov-13	8	1	15	4	3	1	0	11	1	3	1
740	101	1	25-Apr-10	1	1	15	4	3	1	0	11	1	2	1
741	101	1	19-Jul-13	2	1	15	4	2	1	0	6	1	2	1
742	101	1	11-Mar-11	21	2	15	4	3	1	0	6	1	2	1
743	101	1	27-Mar-07	23	2	15	4	2	1	0	6	1	2	1
744	202	0	13-Nov-10	20	1	16	4	1	1	0	11	1	3	1
745	202	0	16-Jul-16	19	1	16	4	1	1	0	3	1	1	1
746	202	0	20-Nov-15	21	2	16	4	3	1	0	11	1	2	1
747	202	0	9-Sep-14	6	2	16	4	3	1	0	11	1	2	1
748	202	0	9-Mar-11	18	2	16	4	1	1	0	11	1	2	2
749	202	0	16-Oct-06	21	2	16	4	1	1	0	6	1	2	1
750	201	1	5-Sep-13	10	1	16	4	2	1	0	6	1	2	1
751	101	1	10-Jul-14	22	1	16	4	2	1	0	7	1	2	1
752	101	1	24-Nov-11	20	1	16	4	3	1	0	11	1	2	1
753	101	1	18-Jul-08	0	1	16	4	1	1	0	11	1	3	1
754	101	1	11-Aug-11	3	1	16	4	3	1	0	3	1	1	2
755	101	1	21-Nov-06	2	1	16	4	2	1	0	3	1	1	2
756	101	1	1-Jul-06	23	1	16	4	3	1	0	6	1	3	1
757	201	1	14-Mar-13	14	2	16	4	3	1	0	7	1	3	1
758	201	1	10-May-13	18	2	16	4	3	1	0	7	1	2	1
759	101	1	2-Sep-09	6	2	16	4	1	1	0	11	1	2	1
760	202	0	20-May-13	19	1	17	4	1	1	0	1	1	4	1
761	202	0	4-Apr-16	17	1	17	4	1	1	0	7	1	2	1
762	202	0	5-Dec-10	20	1	17	4	2	1	0	7	1	5	2
763	202	0	6-Oct-08	18	1	17	4	2	1	0	7	1	1	1
764	202	0	28-May-11	0	1	17	4	1	1	0	11	1	3	1
765	202	0	5-Feb-14	2	1	17	4	2	1	0	3	1	4	1
766	202	0	13-Jul-13	23	1	17	4	1	1	0	3	1	5	1
767	202	0	1-Apr-13	15	1	17	4	2	1	0	3	1	3	1
768	202	0	3-Aug-10	23	1	17	4	1	1	0	3	1	1	2
769	202	0	15-Sep-09	0	1	17	4	1	1	0	3	1	7	2
770	202	0	11-Aug-12	19	1	17	4	3	1	0	6	1	2	1
771	202	0	31-Jul-07	19	1	17	4	1	1	0	6	1	2	1
772	202	0	23-Jul-07	19	1	17	4	2	1	0	6	1	2	2
773	202	0	23-Feb-06	16	1	17	4	3	1	0	6	1	2	1
774	202	0	21-Jan-13	13	2	17	4	1	1	0	6	1	2	2
775	202	0	30-Jan-10	15	2	17	4	3	1	0	10	1	7	2
776	202	0	7-Feb-08	19	2	17	4	1	1	0	10	1	4	2
777	202	0	2-Feb-08	21	2	17	4	1	1	0	10	1	7	1
778	202	0	2-Mar-14	3	2	17	4	1	1	0	11	1	1	1
779	202	0	2-Mar-13	19	2	17	4	1	1	0	11	1	2	1
780	202	0	13-Apr-13	4	2	17	4	1	1	0	3	1	1	2
781	202	0	17-Jun-10	23	2	17	4	2	1	0	6	1	2	2
782	201	1	28-Mar-16	22	1	17	4	2	1	0	6	1	2	2
783	201	1	19-May-07	16	1	17	4	1	1	0	4	1	2	1
784	201	1	8-Sep-06	7	1	17	4	1	1	0	4	1	2	1
785	201	1	20-Nov-15	20	1	17	4	3	1	0	11	1	2	1
786	101	1	24-Feb-15	22	1	17	4	2	1	0	11	1	3	1
787	201	1	7-Mar-12	14	1	17	4	3	1	0	11	1	3	1
788	101	1	4-Aug-10	13	1	17	4	1	1	0	11	1	2	1
789	201	1	28-Jun-17	10	1	17	4	2	1	0	11	1	2	1
790	201	1	22-Sep-16	21	1	17	4	1	1	0	3	1	1	1
791	201	1	21-Dec-07	19	1	17	4	3	1	0	3	1	1	1
792	201	1	16-Dec-17	18	1	17	4	3	1	0	3	1	2	1
793	101	1	9-Jul-12	8	1	17	4	1	1	0	6	1	2	1
794	201	1	9-Jan-08	6	1	17	4	3	1	0	6	1	1	1
795	101	1	2-May-06	9	2	17	4	2	1	0	13	1	2	1
796	101	1	26-Jan-12	1	2	17	4	2	1	0	4	1	3	4
797	201	1	14-Jul-13	5	2	17	4	3	1	0	3	1	3	1
798	101	1	25-Oct-09	18	2	17	4	1	1	0	6	1	1	1
799	201	1	26-Apr-07	17	2	17	4	3	1	0	6	1	2	1
800	202	0	20-Jun-11	1	1	15	3	3	3	1	6	5	4	2
801	202	0	18-Jun-07	17	1	17	3	3	3	1	4	5	5	1
802	202	0	17-Oct-07	10	1	17	3	3	3	1	4	5	4	1
803	202	0	1-Jun-06	18	1	17	3	3	3	1	13	5	4	1
804	101	1	23-Apr-11	17	1	17	3	2	3	1	4	5	1	2
805	101	1	5-Jan-11	19	1	17	3	2	3	1	4	5	5	2
806	101	1	13-Jun-10	1	1	17	3	2	3	1	4	5	5	1
807	101	1	4-Nov-11	2	1	17	3	1	3	1	6	5	5	2
808	101	1	24-Jun-11	21	2	17	3	3	3	1	4	5	4	1
809	202	0	7-Sep-09	19	2	15	3	1	1	0	7	5	3	2
810	101	1	4-Aug-13	8	1	15	3	2	1	0	13	5	3	2
811	202	0	14-Oct-07	0	1	16	3	1	1	0	13	5	2	1
812	202	0	16-Aug-16	6	1	17	3	1	1	0	6	5	2	2
813	202	0	10-Mar-06	18	1	17	3	3	1	0	4	5	2	1
814	202	0	2-Mar-08	9	2	17	3	3	1	0	13	5	2	1
815	201	1	10-Jul-12	5	1	17	3	3	1	0	13	5	2	1
816	101	1	18-Sep-14	17	1	17	3	1	1	0	4	5	2	1
817	101	1	29-Apr-15	18	1	17	3	3	1	0	3	5	3	4
818	201	1	6-Aug-14	9	1	17	3	3	1	0	3	5	1	1
819	101	1	7-Jun-12	18	1	17	3	1	1	0	3	5	2	1
820	101	1	21-Jan-07	14	1	17	3	3	1	0	3	5	2	2
821	101	1	24-May-15	1	1	17	3	2	1	0	6	5	2	2
822	101	1	28-May-15	23	2	14	3	3	3	1	4	4	5	2
823	202	0	13-May-09	9	2	15	3	1	3	1	6	4	2	2
824	101	1	15-Apr-14	18	1	15	3	3	3	1	4	4	1	1
825	101	1	4-Jun-12	10	1	15	3	2	3	1	4	4	1	1
826	101	1	25-Sep-09	11	1	15	3	2	3	1	11	4	2	1
827	101	1	16-Apr-12	2	1	15	3	3	3	1	6	4	5	2
828	202	0	1-Jul-15	23	1	16	3	3	3	1	6	4	3	2
829	202	0	15-Jul-15	4	1	16	3	3	3	1	4	4	3	1
830	202	0	15-Jul-15	2	1	16	3	3	3	1	4	4	3	1
831	202	0	18-Mar-08	1	1	16	3	3	3	1	4	4	1	1
832	202	0	22-Dec-07	12	1	16	3	3	3	1	4	4	1	1
833	202	0	16-Apr-11	18	1	16	3	3	3	1	6	4	2	1
834	202	0	13-Feb-10	2	2	16	3	3	3	1	4	4	1	2
835	202	0	1-Jul-09	23	2	16	3	1	3	1	4	4	1	2
836	202	0	10-Nov-17	3	2	16	3	3	3	1	4	4	1	1
837	202	0	17-Mar-17	1	2	16	3	3	3	1	3	4	4	1
838	202	0	30-Aug-13	2	2	16	3	1	3	1	6	4	5	1
839	202	0	10-Jun-09	23	2	16	3	3	3	1	6	4	1	2
840	201	1	31-May-08	0	1	16	3	3	3	1	1	4	1	2
841	101	1	6-Jun-15	4	1	16	3	2	3	1	4	4	1	1
842	101	1	15-Apr-14	18	1	16	3	3	3	1	4	4	2	1
843	101	1	2-Oct-12	17	1	16	3	3	3	1	4	4	1	1
844	101	1	23-May-10	10	1	16	3	3	3	1	4	4	1	1
845	101	1	6-Aug-10	18	1	16	3	3	3	1	4	4	5	1
846	101	1	23-Apr-09	23	1	16	3	2	3	1	4	4	4	1
847	101	1	22-Oct-09	2	1	16	3	2	3	1	4	4	1	1
848	101	1	12-May-08	20	1	16	3	3	3	1	4	4	6	1
849	101	1	21-Oct-07	13	1	16	3	2	3	1	4	4	1	1
850	101	1	9-Sep-11	14	1	16	3	2	3	1	11	4	3	1
851	101	1	21-Sep-09	14	1	16	3	2	3	1	11	4	2	1
852	101	1	10-May-13	21	1	16	3	3	3	1	6	4	5	2
853	201	1	7-Feb-12	15	1	16	3	3	3	1	6	4	3	1
854	201	1	9-Feb-08	19	2	16	3	3	3	1	6	4	1	1
855	202	0	1-Nov-15	0	1	17	3	2	3	1	10	4	6	1
856	202	0	27-Feb-16	1	1	17	3	3	3	1	6	4	5	2
857	202	0	30-Jun-08	21	1	17	3	1	3	1	10	4	7	1
858	202	0	30-Dec-06	1	1	17	3	2	3	1	10	4	7	2
859	202	0	19-Mar-10	13	1	17	3	1	3	1	13	4	1	1
860	202	0	22-Oct-06	9	1	17	3	3	3	1	13	4	7	1
861	202	0	5-Oct-16	19	1	17	3	3	3	1	4	4	2	1

862	202	0	29-May-16	21	1	17	3	3	3	1	4	4	1	1
863	202	0	5-Jul-15	18	1	17	3	1	3	1	4	4	1	1
864	202	0	12-May-15	23	1	17	3	3	3	1	4	4	1	1
865	202	0	14-Jun-14	0	1	17	3	1	3	1	4	4	1	2
866	202	0	5-May-12	14	1	17	3	3	3	1	4	4	1	2
867	202	0	16-Nov-11	2	1	17	3	3	3	1	4	4	1	1
868	202	0	25-Jan-10	17	1	17	3	2	3	1	4	4	2	2
869	202	0	30-Jul-09	11	1	17	3	1	3	1	4	4	1	1
870	202	0	2-Aug-08	3	1	17	3	3	3	1	4	4	4	1
871	202	0	2-Aug-08	3	1	17	3	3	3	1	4	4	5	1
872	202	0	4-Mar-08	3	1	17	3	3	3	1	4	4	1	2
873	202	0	1-May-08	23	1	17	3	1	3	1	4	4	4	1
874	202	0	30-Nov-08	3	1	17	3	1	3	1	4	4	2	1
875	202	0	9-Oct-07	23	1	17	3	3	3	1	4	4	4	1
876	202	0	9-Sep-07	22	1	17	3	3	3	1	4	4	6	2
877	202	0	1-Dec-07	22	1	17	3	3	3	1	4	4	1	2
878	202	0	15-Oct-17	23	1	17	3	1	3	1	4	4	1	1
879	202	0	25-Jun-17	15	1	17	3	3	3	1	4	4	1	1
880	202	0	23-Dec-13	3	1	17	3	3	3	1	3	4	4	1
881	202	0	22-Mar-17	18	1	17	3	3	3	1	3	4	1	1
882	202	0	4-Aug-17	3	1	17	3	2	3	1	3	4	2	1
883	202	0	21-Aug-15	2	1	17	3	1	3	1	6	4	5	2
884	202	0	19-Mar-13	3	1	17	3	2	3	1	6	4	1	2
885	202	0	23-Mar-13	23	1	17	3	3	3	1	6	4	2	2
886	202	0	7-Aug-12	12	1	17	3	2	3	1	6	4	1	1
887	202	0	24-Oct-10	0	1	17	3	2	3	1	6	4	5	2
888	202	0	12-Aug-09	16	1	17	3	3	3	1	6	4	2	2
889	202	0	2-Nov-08	14	1	17	3	1	3	1	6	4	2	2
890	202	0	18-Sep-06	11	1	17	3	3	3	1	6	4	2	2
891	202	0	8-Mar-07	10	1	17	3	1	3	1	13	4	7	2
892	202	0	18-Jun-16	23	2	17	3	1	3	1	4	4	5	1
893	202	0	2-Oct-14	1	2	17	3	3	3	1	4	4	4	1
894	202	0	30-Aug-14	23	2	17	3	1	3	1	4	4	4	1
895	202	0	21-Sep-08	17	2	17	3	1	3	1	4	4	1	1
896	202	0	16-Jul-08	11	2	17	3	3	3	1	4	4	1	1
897	202	0	5-Jan-08	14	2	17	3	1	3	1	4	4	4	1
898	202	0	24-Jun-08	1	2	17	3	3	3	1	4	4	1	1
899	202	0	20-Aug-06	2	2	17	3	3	3	1	4	4	1	2
900	202	0	11-Jun-13	2	2	17	3	3	3	1	6	4	1	2
901	202	0	21-Jun-11	1	2	17	3	1	3	1	6	4	1	2
902	202	0	31-Dec-10	1	2	17	3	3	3	1	6	4	1	2
903	202	0	15-Feb-10	22	2	17	3	3	3	1	6	4	2	1
904	201	1	8-Aug-14	10	1	17	3	2	3	1	4	4	5	2
905	201	1	14-Apr-08	16	1	17	3	3	3	1	10	4	2	2
906	201	1	29-Dec-12	1	1	17	3	3	3	1	13	4	6	1
907	201	1	11-Oct-08	22	1	17	3	3	3	1	13	4	1	1
908	201	1	27-Nov-08	1	1	17	3	3	3	1	13	4	1	1
909	201	1	10-Jun-07	7	1	17	3	3	3	1	13	4	1	1
910	201	1	23-Jul-08	20	1	17	3	3	3	1	1	4	2	1
911	101	1	11-Jun-15	10	1	17	3	3	3	1	4	4	5	1
912	101	1	26-Mar-15	23	1	17	3	2	3	1	4	4	5	1
913	101	1	14-Jan-14	2	1	17	3	3	3	1	4	4	5	1
914	101	1	16-Jan-13	16	1	17	3	3	3	1	4	4	1	1
915	101	1	15-May-11	8	1	17	3	2	3	1	4	4	1	1
916	101	1	8-Mar-11	23	1	17	3	2	3	1	4	4	1	2
917	101	1	1-May-11	21	1	17	3	3	3	1	4	4	5	1
918	101	1	26-Jul-11	21	1	17	3	2	3	1	4	4	5	1
919	101	1	29-Oct-11	13	1	17	3	3	3	1	4	4	5	1
920	101	1	1-May-11	21	1	17	3	3	3	1	4	4	5	1
921	101	1	21-Oct-11	20	1	17	3	2	3	1	4	4	1	1
922	101	1	10-Feb-10	12	1	17	3	2	3	1	4	4	5	1
923	101	1	6-May-10	20	1	17	3	3	3	1	4	4	2	1
924	201	1	14-Apr-09	5	1	17	3	3	3	1	4	4	2	1
925	101	1	5-Aug-08	3	1	17	3	3	3	1	4	4	6	1
926	101	1	7-Jan-08	10	1	17	3	2	3	1	4	4	6	1
927	201	1	24-Nov-08	14	1	17	3	1	3	1	4	4	1	1
928	201	1	28-May-08	22	1	17	3	3	3	1	4	4	5	1
929	101	1	27-Apr-08	20	1	17	3	3	3	1	4	4	1	1
930	101	1	31-May-08	19	1	17	3	2	3	1	4	4	4	1
931	101	1	30-Jan-07	19	1	17	3	1	3	1	4	4	6	1
932	201	1	19-Aug-07	18	1	17	3	3	3	1	4	4	3	2
933	101	1	13-Apr-07	0	1	17	3	1	3	1	4	4	1	2
934	101	1	19-Mar-07	1	1	17	3	2	3	1	4	4	5	1
935	201	1	25-Oct-07	21	1	17	3	2	3	1	4	4	1	1
936	101	1	14-Mar-07	3	1	17	3	3	3	1	4	4	1	1
937	101	1	2-Jun-07	15	1	17	3	1	3	1	4	4	2	2
938	101	1	19-Apr-06	23	1	17	3	1	3	1	4	4	2	2
939	101	1	26-Apr-06	4	1	17	3	2	3	1	4	4	1	1
940	101	1	11-Oct-13	2	1	17	3	1	3	1	6	4	2	1
941	201	1	9-Nov-12	3	1	17	3	3	3	1	6	4	1	1
942	101	1	20-May-12	23	1	17	3	2	3	1	6	4	2	1
943	101	1	1-Oct-11	5	1	17	3	3	3	1	6	4	2	1
944	201	1	6-Feb-09	2	1	17	3	2	3	1	6	4	5	1
945	101	1	6-Apr-08	4	1	17	3	1	3	1	6	4	2	1
946	201	1	23-Feb-08	23	1	17	3	3	3	1	6	4	1	2
947	201	1	8-Jun-08	1	1	17	3	3	3	1	6	4	1	1
948	101	1	1-Jul-07	23	1	17	3	3	3	1	6	4	2	1
949	101	1	11-Nov-07	22	1	17	3	3	3	1	6	4	2	2
950	201	1	5-May-17	21	2	17	3	3	3	1	4	4	1	1
951	101	1	29-Mar-14	23	2	17	3	3	3	1	4	4	1	1
952	101	1	23-Apr-12	20	2	17	3	2	3	1	4	4	4	1
953	101	1	19-Feb-10	23	2	17	3	2	3	1	4	4	1	1
954	101	1	18-Dec-09	22	2	17	3	3	3	1	4	4	1	1
955	201	1	20-Apr-11	10	2	17	3	2	3	1	6	4	1	2
956	101	1	24-Nov-17	17	2	17	3	2	3	1	13	4	1	1
957	201	1	26-May-08	3	2	16	3	3	2	1	6	4	2	1
958	201	1	28-Nov-08	22	1	17	3	3	2	1	10	4	7	1
959	101	1	28-Mar-07	0	1	17	3	3	2	1	6	4	2	1
960	101	1	7-Jan-10	22	2	17	3	2	2	1	6	4	2	1
961	101	1	3-Jul-11	20	1	12	3	3	1	0	4	4	4	1
962	202	0	14-Jun-11	16	1	13	3	3	1	0	4	4	2	1
963	202	0	20-Jun-17	11	2	14	3	3	1	0	4	4	1	1
964	101	1	9-May-12	16	1	14	3	1	1	0	6	4	2	1
965	101	1	8-Oct-11	5	1	14	3	3	1	0	4	4	3	1
966	201	1	16-Apr-12	22	1	14	3	3	1	0	3	4	1	1
967	101	1	26-Jan-13	9	1	14	3	2	1	0	6	4	2	1
968	202	0	18-Apr-13	16	1	15	3	1	1	0	6	4	4	2
969	202	0	12-Sep-15	0	1	15	3	1	1	0	4	4	3	1
970	202	0	16-Oct-14	16	1	15	3	1	1	0	4	4	1	1
971	202	0	31-Jul-14	14	1	15	3	3	1	0	4	4	1	1
972	202	0	30-Jul-14	17	1	15	3	3	1	0	4	4	1	1
973	202	0	26-Apr-14	15	1	15	3	1	1	0	4	4	2	2
974	202	0	17-Jul-13	11	1	15	3	1	1	0	4	4	2	1
975	202	0	18-Jan-11	9	1	15	3	1	1	0	4	4	2	1
976	202	0	24-Mar-10	14	1	15	3	1	1	0	4	4	2	2
977	202	0	2-Sep-17	17	1	15	3	2	1	0	4	4	3	1
978	202	0	11-Sep-16	18	1	15	3	2	1	0	6	4	2	2
979	202	0	14-Aug-14	9	1	15	3	3	1	0	6	4	2	1
980	202	0	5-Sep-10	2	1	15	3	1	1	0	6	4	2	2
981	202	0	9-Aug-10	11	1	15	3	3	1	0	6	4	2	1
982	202	0	21-Nov-10	11	1	15	3	2	1	0	6	4	2	2
983	202	0	27-May-14	17	2	15	3	2	1	0	4	4	2	1
984	101	1	11-Jan-12	18	1	15	3	3	1	0	4	4	2	1
985	101	1	27-May-12	15	1	15	3	3	1	0	4	4	2	1

986	201	1	22-Apr-12	11	1	15	3	2	1	0	6	4	2	1
987	201	1	8-Aug-16	14	1	15	3	3	1	0	6	4	2	1
988	101	1	26-Feb-14	21	1	15	3	2	1	0	7	4	3	1
989	101	1	15-Oct-12	17	1	15	3	3	1	0	7	4	3	2
990	101	1	22-May-10	16	1	15	3	2	1	0	7	4	5	2
991	101	1	11-Jul-09	20	1	15	3	3	1	0	7	4	2	1
992	201	1	19-Aug-09	12	1	15	3	3	1	0	7	4	3	1
993	101	1	15-Apr-15	10	1	15	3	3	1	0	4	4	2	2
994	201	1	3-Jan-13	16	1	15	3	3	1	0	4	4	2	2
995	201	1	17-Aug-13	11	1	15	3	3	1	0	4	4	2	2
996	101	1	31-Jan-13	19	1	15	3	2	1	0	4	4	2	4
997	201	1	10-Aug-11	16	1	15	3	2	1	0	4	4	2	2
998	101	1	5-Apr-11	16	1	15	3	3	1	0	4	4	2	4
999	101	1	28-Feb-11	18	1	15	3	3	1	0	4	4	2	1
1000	101	1	15-Aug-11	10	1	15	3	2	1	0	4	4	2	1
1001	201	1	27-Feb-10	14	1	15	3	3	1	0	4	4	2	2
1002	101	1	15-Aug-10	9	1	15	3	1	1	0	4	4	2	2
1003	101	1	4-Sep-12	21	1	15	3	3	1	0	3	4	1	1
1004	201	1	5-Aug-12	19	1	15	3	3	1	0	3	4	1	1
1005	101	1	4-Apr-11	21	1	15	3	3	1	0	3	4	2	1
1006	101	1	1-Jun-14	18	1	15	3	2	1	0	6	4	2	1
1007	101	1	3-Nov-12	21	1	15	3	2	1	0	6	4	2	4
1008	101	1	7-Jul-11	20	1	15	3	3	1	0	6	4	2	1
1009	101	1	12-Sep-11	15	1	15	3	3	1	0	6	4	2	1
1010	101	1	19-Dec-10	10	1	15	3	2	1	0	6	4	2	1
1011	101	1	3-Dec-10	12	1	15	3	3	1	0	6	4	2	1
1012	101	1	21-Aug-10	19	1	15	3	2	1	0	6	4	2	1
1013	101	1	6-May-10	20	1	15	3	2	1	0	6	4	4	1
1014	101	1	15-Jun-12	10	2	15	3	1	1	0	7	4	2	1
1015	101	1	6-Jun-11	8	2	15	3	2	1	0	7	4	2	1
1016	201	1	1-Sep-12	17	2	15	3	3	1	0	4	4	2	1
1017	101	1	6-Sep-09	9	2	15	3	2	1	0	4	4	2	1
1018	101	1	31-May-12	21	2	15	3	3	1	0	3	4	1	1
1019	202	0	23-Nov-12	17	1	16	3	1	1	0	4	4	2	2
1020	202	0	9-Aug-12	20	1	16	3	1	1	0	6	4	2	2
1021	202	0	16-Aug-16	9	1	16	3	1	1	0	4	4	2	1
1022	202	0	27-May-16	19	1	16	3	3	1	0	4	4	2	1
1023	202	0	15-Feb-17	4	1	16	3	3	1	0	6	4	2	1
1024	202	0	2-May-14	17	1	16	3	3	1	0	4	4	2	2
1025	202	0	22-Sep-13	14	1	16	3	2	1	0	4	4	2	2
1026	202	0	4-Nov-17	16	1	16	3	3	1	0	6	4	2	2
1027	202	0	18-Mar-14	1	1	16	3	2	1	0	6	4	2	1
1028	202	0	8-Jun-13	12	1	16	3	3	1	0	6	4	2	1
1029	202	0	12-Jan-17	14	1	16	3	1	1	0	6	4	2	2
1030	202	0	21-Oct-14	21	1	16	3	3	1	0	6	4	2	1
1031	202	0	1-Jun-14	21	1	16	3	3	1	0	6	4	2	1
1032	202	0	20-May-08	1	1	16	3	1	1	0	10	4	7	2
1033	202	0	12-May-15	20	1	16	3	3	1	0	7	4	2	1
1034	202	0	8-Oct-13	10	1	16	3	1	1	0	7	4	1	1
1035	202	0	19-Nov-13	11	1	16	3	3	1	0	7	4	1	2
1036	202	0	18-Nov-07	13	1	16	3	1	1	0	7	4	2	2
1037	202	0	11-Feb-17	11	1	16	3	3	1	0	7	4	1	1
1038	202	0	4-Aug-14	8	1	16	3	2	1	0	13	4	1	1
1039	202	0	6-May-13	15	1	16	3	2	1	0	13	4	1	1
1040	202	0	17-Jan-10	20	1	16	3	3	1	0	13	4	1	1
1041	202	0	16-Jan-10	20	1	16	3	3	1	0	13	4	1	1
1042	202	0	10-Jun-08	14	1	16	3	1	1	0	13	4	1	1
1043	202	0	13-Aug-07	22	1	16	3	3	1	0	13	4	1	2
1044	202	0	22-Nov-07	22	1	16	3	3	1	0	13	4	1	1
1045	202	0	8-Nov-06	7	1	16	3	3	1	0	13	4	2	1
1046	202	0	27-Feb-08	7	1	16	3	3	1	0	1	4	3	1
1047	202	0	2-Oct-16	9	1	16	3	3	1	0	4	4	2	2
1048	202	0	31-May-15	9	1	16	3	3	1	0	4	4	2	2
1049	202	0	8-Aug-14	11	1	16	3	2	1	0	4	4	1	1
1050	202	0	3-Jul-14	18	1	16	3	3	1	0	4	4	4	1
1051	202	0	22-Feb-14	22	1	16	3	3	1	0	4	4	2	1
1052	202	0	5-Dec-14	1	1	16	3	3	1	0	4	4	3	1
1053	202	0	27-Aug-14	8	1	16	3	1	1	0	4	4	1	1
1054	202	0	12-Apr-14	18	1	16	3	3	1	0	4	4	2	2
1055	202	0	28-Jan-13	15	1	16	3	3	1	0	4	4	1	1
1056	202	0	23-Oct-13	17	1	16	3	1	1	0	4	4	3	2
1057	202	0	8-Jul-13	3	1	16	3	3	1	0	4	4	3	1
1058	202	0	17-Nov-12	14	1	16	3	3	1	0	4	4	2	2
1059	202	0	10-Dec-10	12	1	16	3	1	1	0	4	4	2	2
1060	202	0	9-Aug-10	19	1	16	3	1	1	0	4	4	2	1
1061	202	0	10-Dec-09	16	1	16	3	3	1	0	4	4	1	1
1062	202	0	20-Jul-08	10	1	16	3	2	1	0	4	4	2	2
1063	202	0	16-Dec-07	22	1	16	3	1	1	0	4	4	2	2
1064	202	0	28-May-07	6	1	16	3	3	1	0	4	4	2	2
1065	202	0	1-Jul-07	19	1	16	3	2	1	0	4	4	3	2
1066	202	0	8-Apr-06	13	1	16	3	3	1	0	4	4	3	2
1067	202	0	5-Jan-06	10	1	16	3	3	1	0	4	4	6	1
1068	202	0	10-Apr-06	14	1	16	3	3	1	0	4	4	2	1
1069	202	0	28-Jul-06	11	1	16	3	1	1	0	4	4	3	2
1070	202	0	22-May-16	9	1	16	3	3	1	0	11	4	3	1
1071	202	0	2-Oct-14	19	1	16	3	3	1	0	11	4	3	2
1072	202	0	22-Mar-13	12	1	16	3	1	1	0	11	4	2	1
1073	202	0	13-Jun-14	17	1	16	3	1	1	0	3	4	3	2
1074	202	0	26-Jun-14	11	1	16	3	3	1	0	3	4	1	1
1075	202	0	12-Oct-14	17	1	16	3	3	1	0	3	4	2	1
1076	202	0	2-Jan-13	15	1	16	3	3	1	0	3	4	1	1
1077	202	0	22-Dec-09	10	1	16	3	3	1	0	3	4	1	1
1078	202	0	7-Aug-08	7	1	16	3	3	1	0	3	4	2	2
1079	202	0	5-Apr-08	13	1	16	3	3	1	0	3	4	1	2
1080	202	0	31-Aug-08	6	1	16	3	2	1	0	3	4	1	2
1081	202	0	23-May-08	9	1	16	3	3	1	0	3	4	2	2
1082	202	0	16-Oct-08	8	1	16	3	2	1	0	3	4	2	2
1083	202	0	14-Feb-08	23	1	16	3	1	1	0	3	4	1	1
1084	202	0	15-Nov-07	8	1	16	3	3	1	0	3	4	6	1
1085	202	0	13-Dec-16	2	1	16	3	3	1	0	6	4	3	1
1086	202	0	25-Aug-15	17	1	16	3	2	1	0	6	4	3	1
1087	202	0	22-Nov-13	13	1	16	3	1	1	0	6	4	2	1
1088	202	0	8-Mar-13	19	1	16	3	2	1	0	6	4	2	1
1089	202	0	17-Jul-12	0	1	16	3	2	1	0	6	4	4	2
1090	202	0	25-Jul-11	17	1	16	3	3	1	0	6	4	2	1
1091	202	0	22-Apr-11	9	1	16	3	1	1	0	6	4	2	1
1092	202	0	7-Sep-11	16	1	16	3	3	1	0	6	4	2	1
1093	202	0	9-Dec-11	18	1	16	3	3	1	0	6	4	2	2
1094	202	0	18-Jul-10	3	1	16	3	1	1	0	6	4	2	2
1095	202	0	28-Nov-10	23	1	16	3	1	1	0	6	4	1	2
1096	202	0	17-Nov-09	15	1	16	3	2	1	0	6	4	1	1
1097	202	0	3-Nov-09	21	1	16	3	3	1	0	6	4	2	2
1098	202	0	5-Oct-09	20	1	16	3	3	1	0	6	4	2	1
1099	202	0	23-Nov-08	2	1	16	3	2	1	0	6	4	2	2
1100	202	0	13-Jun-07	16	1	16	3	2	1	0	6	4	2	2
1101	202	0	5-May-07	22	1	16	3	3	1	0	6	4	2	1
1102	202	0	13-Jun-07	16	1	16	3	3	1	0	6	4	2	1
1103	202	0	28-May-06	10	1	16	3	3	1	0	6	4	2	1
1104	202	0	3-Sep-06	13	1	16	3	3	1	0	6	4	2	1
1105	202	0	10-Jun-17	10	1	16	3	2	1	0	6	4	2	2
1106	202	0	28-May-07	20	1	16	3	3	1	0	13	4	1	1
1107	202	0	6-Jul-06	13	1	16	3	3	1	0	13	4	2	2
1108	202	0	12-Nov-16	2	2	16	3	2	1	0	10	4	7	2
1109	202	0	10-Mar-17	1	2	16	3	1	1	0	10	4	2	1

1110	202	0	5-Aug-14	20	2	16	3	3	1	0	6	4	2	1
1111	202	0	12-Aug-16	4	2	16	3	1	1	0	7	4	2	1
1112	202	0	31-Jul-08	20	2	16	3	3	1	0	7	4	2	2
1113	202	0	29-Aug-06	16	2	16	3	3	1	0	7	4	3	1
1114	202	0	5-Jul-15	8	2	16	3	2	1	0	13	4	1	1
1115	202	0	2-Dec-14	14	2	16	3	2	1	0	4	4	2	1
1116	202	0	10-Dec-14	4	2	16	3	3	1	0	4	4	2	1
1117	202	0	14-Jul-11	9	2	16	3	3	1	0	4	4	2	2
1118	202	0	11-Sep-10	2	2	16	3	3	1	0	4	4	3	1
1119	202	0	28-Mar-09	19	2	16	3	3	1	0	4	4	2	2
1120	202	0	21-Sep-09	18	2	16	3	1	1	0	4	4	2	2
1121	202	0	29-Jun-07	14	2	16	3	1	1	0	4	4	4	1
1122	202	0	13-Jul-14	10	2	16	3	3	1	0	3	4	1	1
1123	202	0	4-Oct-14	8	2	16	3	1	1	0	3	4	1	1
1124	202	0	11-Feb-11	21	2	16	3	1	1	0	3	4	1	2
1125	202	0	31-Oct-08	20	2	16	3	3	1	0	3	4	3	1
1126	202	0	15-Aug-07	16	2	16	3	3	1	0	3	4	2	1
1127	202	0	26-Dec-13	21	2	16	3	1	1	0	6	4	2	1
1128	202	0	12-Dec-12	14	2	16	3	3	1	0	6	4	2	1
1129	202	0	21-Aug-10	18	2	16	3	1	1	0	6	4	2	1
1130	202	0	24-Mar-08	9	2	16	3	1	1	0	6	4	2	1
1131	202	0	10-Dec-06	11	2	16	3	3	1	0	6	4	2	1
1132	202	0	14-Jan-06	0	2	16	3	3	1	0	6	4	2	1
1133	202	0	12-Jan-06	9	2	16	3	3	1	0	6	4	2	1
1134	202	0	31-Jul-06	12	2	16	3	1	1	0	6	4	1	1
1135	201	1	21-Apr-17	0	1	16	3	3	1	0	10	4	6	1
1136	101	1	22-Apr-12	10	1	16	3	1	1	0	4	4	2	1
1137	101	1	25-May-12	22	1	16	3	3	1	0	6	4	2	1
1138	201	1	13-May-12	18	1	16	3	3	1	0	3	4	7	1
1139	201	1	9-May-12	9	1	16	3	3	1	0	6	4	2	1
1140	201	1	4-May-13	19	1	16	3	2	1	0	4	4	2	1
1141	201	1	5-May-14	16	1	16	3	2	1	0	6	4	3	1
1142	201	1	27-Apr-17	10	1	16	3	3	1	0	6	4	2	2
1143	101	1	22-Feb-12	8	1	16	3	2	1	0	4	4	2	1
1144	201	1	26-Aug-12	10	1	16	3	3	1	0	4	4	2	1
1145	201	1	10-Sep-12	18	1	16	3	3	1	0	13	4	2	1
1146	201	1	14-Dec-12	9	1	16	3	2	1	0	4	4	2	2
1147	201	1	22-Jun-12	9	1	16	3	3	1	0	4	4	2	1
1148	201	1	19-Jun-12	10	1	16	3	2	1	0	4	4	2	1
1149	101	1	22-May-13	20	1	16	3	1	1	0	4	4	2	2
1150	201	1	2-Aug-13	10	1	16	3	3	1	0	6	4	2	1
1151	101	1	9-May-12	10	1	16	3	2	1	0	6	4	2	1
1152	201	1	3-Apr-17	15	1	16	3	1	1	0	6	4	2	1
1153	101	1	6-Feb-15	12	1	16	3	2	1	0	7	4	3	1
1154	101	1	6-Dec-14	13	1	16	3	3	1	0	7	4	5	1
1155	101	1	20-Aug-13	14	1	16	3	3	1	0	7	4	5	2
1156	101	1	15-Jun-13	8	1	16	3	3	1	0	7	4	2	1
1157	101	1	29-Nov-11	10	1	16	3	2	1	0	7	4	2	1
1158	101	1	4-Mar-10	10	1	16	3	1	1	0	7	4	5	1
1159	101	1	28-Oct-10	2	1	16	3	3	1	0	7	4	1	1
1160	201	1	19-Oct-07	4	1	16	3	3	1	0	7	4	2	1
1161	201	1	28-Sep-07	16	1	16	3	3	1	0	7	4	1	1
1162	101	1	27-Jul-07	20	1	16	3	1	1	0	7	4	1	1
1163	101	1	9-Jan-11	16	1	16	3	3	1	0	5	4	1	1
1164	101	1	22-Apr-14	18	1	16	3	2	1	0	4	4	2	1
1165	101	1	18-Jun-14	21	1	16	3	1	1	0	4	4	3	1
1166	101	1	6-Dec-14	12	1	16	3	3	1	0	4	4	2	4
1167	101	1	30-Jul-13	1	1	16	3	3	1	0	4	4	3	1
1168	201	1	29-Aug-13	19	1	16	3	3	1	0	4	4	2	1
1169	101	1	23-May-12	12	1	16	3	1	1	0	4	4	1	1
1170	101	1	3-Apr-12	11	1	16	3	1	1	0	4	4	1	1
1171	101	1	29-Nov-12	16	1	16	3	3	1	0	4	4	1	1
1172	201	1	7-Dec-11	6	1	16	3	2	1	0	4	4	2	1
1173	101	1	6-May-11	10	1	16	3	3	1	0	4	4	2	1
1174	101	1	29-May-11	10	1	16	3	3	1	0	4	4	2	1
1175	101	1	16-Mar-11	9	1	16	3	2	1	0	4	4	2	1
1176	201	1	10-Jan-11	10	1	16	3	3	1	0	4	4	2	1
1177	201	1	20-Sep-11	22	1	16	3	3	1	0	4	4	2	1
1178	201	1	10-Nov-10	11	1	16	3	3	1	0	4	4	2	1
1179	101	1	12-Jul-10	10	1	16	3	3	1	0	4	4	2	1
1180	101	1	22-Apr-10	11	1	16	3	1	1	0	4	4	2	1
1181	101	1	2-Nov-10	17	1	16	3	3	1	0	4	4	2	1
1182	201	1	20-Nov-10	12	1	16	3	2	1	0	4	4	2	1
1183	201	1	10-Oct-10	10	1	16	3	3	1	0	4	4	2	2
1184	201	1	26-Nov-10	15	1	16	3	3	1	0	4	4	2	1
1185	101	1	19-Jul-09	20	1	16	3	3	1	0	4	4	2	2
1186	201	1	31-Oct-09	9	1	16	3	3	1	0	4	4	2	1
1187	201	1	20-Apr-09	8	1	16	3	3	1	0	4	4	2	1
1188	201	1	24-Oct-09	20	1	16	3	3	1	0	4	4	2	1
1189	201	1	14-Nov-08	21	1	16	3	3	1	0	4	4	1	1
1190	201	1	11-Jun-08	11	1	16	3	3	1	0	4	4	3	2
1191	201	1	15-Oct-08	20	1	16	3	3	1	0	4	4	2	1
1192	201	1	4-Jun-08	22	1	16	3	3	1	0	4	4	2	1
1193	201	1	16-Apr-08	15	1	16	3	3	1	0	4	4	3	2
1194	101	1	5-Dec-08	17	1	16	3	2	1	0	4	4	1	1
1195	101	1	18-Nov-07	15	1	16	3	3	1	0	4	4	2	2
1196	201	1	30-Jul-07	7	1	16	3	3	1	0	4	4	2	1
1197	201	1	4-Oct-07	16	1	16	3	3	1	0	4	4	2	1
1198	201	1	31-Jul-07	22	1	16	3	3	1	0	4	4	6	1
1199	101	1	21-Sep-06	20	1	16	3	3	1	0	4	4	2	1
1200	201	1	20-Apr-06	22	1	16	3	3	1	0	4	4	2	1
1201	101	1	11-Aug-06	11	1	16	3	3	1	0	4	4	2	1
1202	101	1	15-Jul-06	15	1	16	3	2	1	0	4	4	2	1
1203	201	1	6-Mar-06	15	1	16	3	3	1	0	4	4	2	1
1204	201	1	11-Apr-17	14	1	16	3	3	1	0	4	4	2	1
1205	101	1	23-Sep-17	2	1	16	3	2	1	0	4	4	2	1
1206	201	1	25-Nov-12	11	1	16	3	2	1	0	11	4	3	1
1207	201	1	12-Dec-12	11	1	16	3	3	1	0	11	4	2	1
1208	201	1	21-Oct-08	21	1	16	3	2	1	0	11	4	3	1
1209	101	1	7-Jan-15	6	1	16	3	1	1	0	3	4	3	2
1210	201	1	10-Sep-13	18	1	16	3	2	1	0	3	4	1	1
1211	201	1	2-Apr-13	10	1	16	3	3	1	0	3	4	2	1
1212	201	1	26-Apr-12	10	1	16	3	3	1	0	3	4	6	2
1213	201	1	8-Nov-12	16	1	16	3	2	1	0	3	4	2	1
1214	201	1	14-Dec-12	14	1	16	3	3	1	0	3	4	4	1
1215	101	1	12-Jun-12	10	1	16	3	3	1	0	3	4	1	1
1216	101	1	16-Dec-11	11	1	16	3	2	1	0	3	4	1	2
1217	101	1	15-Feb-11	17	1	16	3	2	1	0	3	4	4	1
1218	201	1	16-Nov-10	11	1	16	3	3	1	0	3	4	1	1
1219	201	1	10-Dec-10	17	1	16	3	3	1	0	3	4	3	1
1220	201	1	3-May-09	9	1	16	3	3	1	0	3	4	3	1
1221	201	1	7-Dec-08	8	1	16	3	3	1	0	3	4	4	2
1222	201	1	22-Apr-08	16	1	16	3	3	1	0	3	4	2	1
1223	201	1	23-Jan-07	19	1	16	3	3	1	0	3	4	2	1
1224	201	1	23-Apr-07	18	1	16	3	3	1	0	3	4	1	2
1225	101	1	25-Jun-15	13	1	16	3	1	1	0	6	4	3	4
1226	101	1	12-Sep-14	4	1	16	3	1	1	0	6	4	2	1
1227	101	1	24-Aug-14	20	1	16	3	3	1	0	6	4	2	1
1228	101	1	12-Sep-14	15	1	16	3	2	1	0	6	4	3	1
1229	101	1	28-Mar-13	12	1	16	3	2	1	0	6	4	2	1
1230	101	1	2-Aug-13	18	1	16	3	2	1	0	6	4	2	1
1231	101	1	20-Oct-12	17	1	16	3	3	1	0	6	4	2	1
1232	101	1	20-Jan-12	8	1	16	3	1	1	0	6	4	2	1
1233	101	1	19-Dec-12	18	1	16	3	1	1	0	6	4	2	1

1234	101	1	28-Mar-12	6	1	16	3	2	1	0	6	4	2	4
1235	101	1	22-Jun-12	19	1	16	3	3	1	0	6	4	2	1
1236	101	1	3-Jul-11	15	1	16	3	3	1	0	6	4	2	2
1237	201	1	1-Apr-11	8	1	16	3	3	1	0	6	4	2	1
1238	101	1	19-Nov-11	12	1	16	3	2	1	0	6	4	2	1
1239	101	1	27-Dec-11	17	1	16	3	2	1	0	6	4	2	1
1240	101	1	11-Dec-10	8	1	16	3	2	1	0	6	4	2	1
1241	101	1	9-Sep-10	10	1	16	3	2	1	0	6	4	2	1
1242	101	1	23-May-10	17	1	16	3	2	1	0	6	4	2	1
1243	101	1	24-Oct-09	12	1	16	3	3	1	0	6	4	2	1
1244	201	1	17-Jun-09	9	1	16	3	3	1	0	6	4	2	2
1245	201	1	3-Sep-09	9	1	16	3	1	1	0	6	4	2	1
1246	201	1	17-Feb-09	11	1	16	3	3	1	0	6	4	2	1
1247	101	1	12-Jun-08	22	1	16	3	3	1	0	6	4	2	1
1248	201	1	6-Dec-08	9	1	16	3	3	1	0	6	4	2	2
1249	101	1	13-May-08	13	1	16	3	2	1	0	6	4	3	1
1250	201	1	29-Feb-08	8	1	16	3	3	1	0	6	4	2	1
1251	101	1	8-Dec-08	20	1	16	3	1	1	0	6	4	2	1
1252	101	1	2-Jan-07	11	1	16	3	3	1	0	6	4	3	2
1253	201	1	10-Apr-07	22	1	16	3	3	1	0	6	4	2	1
1254	101	1	11-Jul-07	12	1	16	3	3	1	0	6	4	2	1
1255	201	1	15-Oct-07	21	1	16	3	3	1	0	6	4	3	2
1256	201	1	13-Oct-06	10	1	16	3	3	1	0	6	4	2	1
1257	201	1	27-Dec-06	23	1	16	3	3	1	0	6	4	2	1
1258	101	1	19-Sep-06	11	1	16	3	3	1	0	6	4	2	1
1259	101	1	23-Jul-06	9	1	16	3	3	1	0	6	4	2	1
1260	201	1	15-Feb-12	15	2	16	3	3	1	0	4	4	2	1
1261	201	1	28-Nov-13	9	2	16	3	3	1	0	6	4	2	1
1262	201	1	26-May-12	21	2	16	3	3	1	0	6	4	3	1
1263	201	1	4-Mar-09	19	2	16	3	3	1	0	10	4	7	1
1264	101	1	2-Aug-15	22	2	16	3	3	1	0	7	4	2	2
1265	101	1	21-Jun-11	17	2	16	3	2	1	0	7	4	2	1
1266	201	1	29-Apr-11	18	2	16	3	3	1	0	7	4	3	1
1267	201	1	1-Nov-07	9	2	16	3	3	1	0	13	4	2	1
1268	201	1	10-Feb-07	18	2	16	3	2	1	0	13	4	2	1
1269	101	1	4-Apr-06	19	2	16	3	3	1	0	13	4	1	1
1270	101	1	29-Jul-13	17	2	16	3	3	1	0	4	4	3	1
1271	201	1	12-Jul-11	8	2	16	3	3	1	0	4	4	2	1
1272	101	1	2-Jul-09	21	2	16	3	3	1	0	4	4	3	1
1273	101	1	23-Feb-08	14	2	16	3	3	1	0	4	4	1	1
1274	101	1	13-Aug-08	9	2	16	3	3	1	0	4	4	2	1
1275	201	1	8-Jul-08	13	2	16	3	3	1	0	4	4	2	2
1276	201	1	5-Nov-08	15	2	16	3	3	1	0	4	4	7	1
1277	201	1	4-Jan-07	13	2	16	3	3	1	0	4	4	2	1
1278	201	1	26-Nov-07	16	2	16	3	2	1	0	4	4	2	1
1279	101	1	9-Apr-06	23	2	16	3	3	1	0	4	4	2	1
1280	101	1	18-Aug-06	11	2	16	3	3	1	0	4	4	2	1
1281	101	1	10-Oct-06	17	2	16	3	3	1	0	4	4	2	1
1282	101	1	15-Oct-07	16	2	16	3	3	1	0	11	4	3	1
1283	201	1	11-Sep-10	22	2	16	3	3	1	0	3	4	3	1
1284	201	1	25-Nov-07	22	2	16	3	3	1	0	3	4	2	1
1285	101	1	27-Sep-06	19	2	16	3	3	1	0	3	4	2	1
1286	101	1	7-Feb-17	10	2	16	3	3	1	0	3	4	3	2
1287	101	1	26-Apr-14	14	2	16	3	2	1	0	6	4	2	1
1288	201	1	21-Apr-11	16	2	16	3	3	1	0	6	4	2	2
1289	101	1	30-Jul-11	1	2	16	3	1	1	0	6	4	4	1
1290	101	1	23-Oct-10	9	2	16	3	3	1	0	6	4	2	1
1291	101	1	26-Sep-09	16	2	16	3	2	1	0	6	4	2	1
1292	201	1	13-Nov-09	19	2	16	3	2	1	0	6	4	2	1
1293	201	1	18-Feb-09	10	2	16	3	3	1	0	6	4	2	1
1294	201	1	21-Jan-07	2	2	16	3	2	1	0	6	4	2	1
1295	101	1	24-May-07	11	2	16	3	1	1	0	13	4	2	1
1296	202	0	19-Jun-14	11	1	17	3	2	1	0	7	4	2	2
1297	202	0	14-Mar-17	18	1	17	3	1	1	0	4	4	3	1
1298	202	0	15-Jul-14	16	1	17	3	2	1	0	6	4	2	1
1299	202	0	1-Dec-16	11	1	17	3	3	1	0	4	4	2	2
1300	202	0	25-May-14	18	1	17	3	1	1	0	6	4	2	2
1301	202	0	13-Oct-16	21	1	17	3	3	1	0	6	4	2	1
1302	202	0	4-Dec-13	2	1	17	3	1	1	0	7	4	2	2
1303	202	0	17-Sep-13	9	1	17	3	3	1	0	4	4	2	2
1304	202	0	27-Dec-16	20	1	17	3	2	1	0	4	4	2	2
1305	202	0	20-Mar-12	0	1	17	3	3	1	0	6	4	2	1
1306	202	0	18-Feb-14	13	1	17	3	2	1	0	4	4	2	2
1307	202	0	26-Apr-14	21	1	17	3	2	1	0	4	4	2	1
1308	202	0	15-Jan-13	2	1	17	3	1	1	0	4	4	2	1
1309	202	0	23-Jan-16	20	1	17	3	3	1	0	4	4	2	2
1310	202	0	23-Mar-16	14	1	17	3	1	1	0	6	4	2	2
1311	202	0	16-Oct-17	0	1	17	3	1	1	0	4	4	2	1
1312	202	0	2-Dec-16	16	1	17	3	3	1	0	4	4	2	1
1313	202	0	12-Jun-17	10	1	17	3	3	1	0	4	4	1	1
1314	202	0	21-Jul-15	14	1	17	3	3	1	0	4	4	5	1
1315	202	0	6-Sep-16	10	1	17	3	1	1	0	4	4	1	2
1316	202	0	16-Dec-14	20	1	17	3	1	1	0	8	4	4	2
1317	202	0	12-Jun-13	12	1	17	3	1	1	0	6	4	2	1
1318	202	0	21-Sep-16	20	1	17	3	2	1	0	6	4	3	1
1319	202	0	19-Nov-15	12	1	17	3	1	1	0	6	4	2	2
1320	202	0	18-Mar-17	14	1	17	3	3	1	0	6	4	1	1
1321	202	0	9-Oct-15	20	1	17	3	3	1	0	4	4	2	1
1322	202	0	22-May-12	14	1	17	3	1	1	0	4	4	2	2
1323	202	0	15-Jan-13	4	1	17	3	1	1	0	6	4	3	2
1324	202	0	7-Aug-08	20	1	17	3	2	1	0	10	4	7	1
1325	202	0	30-Jul-16	12	1	17	3	3	1	0	7	4	2	1
1326	202	0	3-Nov-15	19	1	17	3	3	1	0	7	4	1	1
1327	202	0	19-Apr-11	9	1	17	3	3	1	0	7	4	1	2
1328	202	0	20-May-11	10	1	17	3	3	1	0	7	4	2	2
1329	202	0	15-Jul-10	8	1	17	3	1	1	0	7	4	5	2
1330	202	0	16-May-08	16	1	17	3	1	1	0	7	4	2	1
1331	202	0	21-Apr-07	8	1	17	3	3	1	0	7	4	1	1
1332	202	0	28-Dec-06	2	1	17	3	3	1	0	7	4	4	2
1333	202	0	26-Oct-06	17	1	17	3	3	1	0	7	4	5	2
1334	202	0	29-Aug-06	5	1	17	3	3	1	0	7	4	6	2
1335	202	0	12-Mar-17	21	1	17	3	3	1	0	7	4	2	1
1336	202	0	29-Apr-17	9	1	17	3	2	1	0	7	4	1	1
1337	202	0	4-May-16	17	1	17	3	3	1	0	13	4	1	1
1338	202	0	6-Sep-15	8	1	17	3	2	1	0	13	4	1	1
1339	202	0	19-Sep-13	9	1	17	3	2	1	0	13	4	1	1
1340	202	0	6-Oct-10	18	1	17	3	3	1	0	13	4	2	2
1341	202	0	6-Apr-10	8	1	17	3	3	1	0	13	4	1	1
1342	202	0	28-Apr-10	4	1	17	3	1	1	0	13	4	2	1
1343	202	0	12-Apr-09	18	1	17	3	3	1	0	13	4	1	1
1344	202	0	24-Aug-09	17	1	17	3	3	1	0	13	4	1	1
1345	202	0	15-Apr-09	11	1	17	3	3	1	0	13	4	1	1
1346	202	0	8-Jun-09	17	1	17	3	1	1	0	13	4	1	1
1347	202	0	28-Oct-08	17	1	17	3	3	1	0	13	4	1	1
1348	202	0	20-Aug-08	17	1	17	3	3	1	0	13	4	1	1
1349	202	0	6-Sep-08	17	1	17	3	3	1	0	13	4	1	1
1350	202	0	6-Sep-08	17	1	17	3	3	1	0	13	4	1	1
1351	202	0	25-May-08	17	1	17	3	3	1	0	13	4	1	1
1352	202	0	1-Sep-08	18	1	17	3	3	1	0	13	4	7	1
1353	202	0	25-Oct-07	3	1	17	3	3	1	0	13	4	2	2
1354	202	0	21-Dec-07	22	1	17	3	3	1	0	13	4	2	2
1355	202	0	15-Dec-07	23	1	17	3	1	1	0	13	4	7	1
1356	202	0	28-May-07	16	1	17	3	1	1	0	13	4	1	1
1357	202	0	3-Dec-06	9	1	17	3	3	1	0	13	4	1	1

1358	202	0	26-Dec-06	19	1	17	3	3	1	0	13	4	1	1
1359	202	0	10-Feb-06	15	1	17	3	3	1	0	13	4	2	2
1360	202	0	23-Oct-06	9	1	17	3	2	1	0	13	4	1	1
1361	202	0	14-Nov-16	15	1	17	3	2	1	0	4	4	1	1
1362	202	0	22-Jul-16	0	1	17	3	3	1	0	4	4	1	1
1363	202	0	15-Nov-16	22	1	17	3	3	1	0	4	4	2	1
1364	202	0	30-Mar-16	16	1	17	3	3	1	0	4	4	2	2
1365	202	0	5-May-16	16	1	17	3	3	1	0	4	4	2	1
1366	202	0	5-Dec-16	2	1	17	3	3	1	0	4	4	2	1
1367	202	0	27-Oct-16	15	1	17	3	1	1	0	4	4	2	1
1368	202	0	2-Jul-15	12	1	17	3	3	1	0	4	4	3	1
1369	202	0	12-Jun-15	21	1	17	3	1	1	0	4	4	2	1
1370	202	0	13-Oct-15	16	1	17	3	1	1	0	4	4	3	1
1371	202	0	15-Apr-14	10	1	17	3	3	1	0	4	4	2	1
1372	202	0	29-Nov-14	23	1	17	3	3	1	0	4	4	1	1
1373	202	0	8-Oct-14	16	1	17	3	3	1	0	4	4	3	1
1374	202	0	28-Oct-14	12	1	17	3	3	1	0	4	4	2	1
1375	202	0	29-Oct-14	11	1	17	3	3	1	0	4	4	2	1
1376	202	0	9-Dec-14	9	1	17	3	3	1	0	4	4	4	1
1377	202	0	9-Apr-14	10	1	17	3	3	1	0	4	4	3	1
1378	202	0	6-Jul-14	22	1	17	3	3	1	0	4	4	2	2
1379	202	0	1-Sep-14	8	1	17	3	2	1	0	4	4	1	1
1380	202	0	24-Sep-14	8	1	17	3	1	1	0	4	4	1	1
1381	202	0	25-Sep-14	10	1	17	3	1	1	0	4	4	2	1
1382	202	0	10-Dec-13	20	1	17	3	3	1	0	4	4	2	1
1383	202	0	26-Feb-13	21	1	17	3	2	1	0	4	4	2	2
1384	202	0	5-Jul-13	15	1	17	3	3	1	0	4	4	2	2
1385	202	0	23-Feb-13	14	1	17	3	3	1	0	4	4	2	2
1386	202	0	27-Nov-13	16	1	17	3	2	1	0	4	4	2	1
1387	202	0	15-Sep-13	10	1	17	3	2	1	0	4	4	2	1
1388	202	0	28-Mar-13	7	1	17	3	3	1	0	4	4	2	1
1389	202	0	27-Dec-12	10	1	17	3	3	1	0	4	4	2	1
1390	202	0	11-Oct-12	22	1	17	3	3	1	0	4	4	2	2
1391	202	0	28-Aug-12	22	1	17	3	4	1	0	4	4	2	2
1392	202	0	23-Jul-12	19	1	17	3	3	1	0	4	4	3	2
1393	202	0	17-Apr-12	19	1	17	3	3	1	0	4	4	1	2
1394	202	0	21-Oct-12	2	1	17	3	1	1	0	4	4	6	1
1395	202	0	10-Oct-10	13	1	17	3	3	1	0	4	4	2	1
1396	202	0	22-Mar-10	6	1	17	3	2	1	0	4	4	2	2
1397	202	0	16-Dec-10	3	1	17	3	3	1	0	4	4	3	2
1398	202	0	3-Oct-10	22	1	17	3	3	1	0	4	4	2	1
1399	202	0	30-May-10	7	1	17	3	1	1	0	4	4	2	1
1400	202	0	14-Aug-10	4	1	17	3	3	1	0	4	4	2	2
1401	202	0	6-Jun-10	10	1	17	3	3	1	0	4	4	2	2
1402	202	0	22-Jun-10	0	1	17	3	3	1	0	4	4	1	1
1403	202	0	28-Nov-09	20	1	17	3	2	1	0	4	4	3	2
1404	202	0	15-Nov-09	2	1	17	3	3	1	0	4	4	3	2
1405	202	0	22-Jul-09	14	1	17	3	3	1	0	4	4	2	2
1406	202	0	15-Aug-09	20	1	17	3	3	1	0	4	4	2	1
1407	202	0	15-Apr-09	12	1	17	3	2	1	0	4	4	2	1
1408	202	0	23-Dec-08	10	1	17	3	2	1	0	4	4	2	2
1409	202	0	17-Mar-08	21	1	17	3	2	1	0	4	4	2	2
1410	202	0	16-Sep-08	16	1	17	3	3	1	0	4	4	2	2
1411	202	0	3-Aug-08	12	1	17	3	1	1	0	4	4	1	1
1412	202	0	15-Apr-08	20	1	17	3	3	1	0	4	4	2	1
1413	202	0	11-Apr-08	6	1	17	3	2	1	0	4	4	2	1
1414	202	0	3-Jun-07	17	1	17	3	1	1	0	4	4	2	2
1415	202	0	1-Jan-07	16	1	17	3	2	1	0	4	4	2	1
1416	202	0	16-Mar-07	10	1	17	3	2	1	0	4	4	2	1
1417	202	0	26-Jul-07	0	1	17	3	3	1	0	4	4	2	1
1418	202	0	16-Jul-07	7	1	17	3	3	1	0	4	4	2	2
1419	202	0	10-Jul-07	18	1	17	3	3	1	0	4	4	2	2
1420	202	0	27-Oct-07	17	1	17	3	3	1	0	4	4	2	2
1421	202	0	22-Apr-07	16	1	17	3	3	1	0	4	4	2	2
1422	202	0	15-Feb-07	5	1	17	3	3	1	0	4	4	2	1
1423	202	0	30-Apr-07	10	1	17	3	3	1	0	4	4	2	2
1424	202	0	18-Jul-07	8	1	17	3	1	1	0	4	4	2	2
1425	202	0	23-Sep-07	12	1	17	3	3	1	0	4	4	3	1
1426	202	0	16-Mar-07	11	1	17	3	3	1	0	4	4	2	1
1427	202	0	9-Jun-07	11	1	17	3	2	1	0	4	4	2	2
1428	202	0	22-Aug-07	3	1	17	3	3	1	0	4	4	7	2
1429	202	0	8-Mar-06	22	1	17	3	3	1	0	4	4	4	1
1430	202	0	12-Nov-06	18	1	17	3	2	1	0	4	4	1	1
1431	202	0	24-Jul-06	14	1	17	3	1	1	0	4	4	2	1
1432	202	0	1-Dec-06	3	1	17	3	3	1	0	4	4	2	1
1433	202	0	9-Nov-06	9	1	17	3	3	1	0	4	4	2	2
1434	202	0	21-Nov-06	16	1	17	3	2	1	0	4	4	2	2
1435	202	0	26-Apr-06	14	1	17	3	3	1	0	4	4	2	1
1436	202	0	23-May-06	2	1	17	3	2	1	0	4	4	2	1
1437	202	0	3-Jun-06	11	1	17	3	3	1	0	4	4	6	1
1438	202	0	1-Sep-06	15	1	17	3	1	1	0	4	4	3	1
1439	202	0	10-Nov-06	7	1	17	3	3	1	0	4	4	2	2
1440	202	0	10-Nov-06	4	1	17	3	2	1	0	4	4	2	2
1441	202	0	14-Apr-06	0	1	17	3	3	1	0	4	4	2	2
1442	202	0	8-Mar-06	12	1	17	3	3	1	0	4	4	1	1
1443	202	0	1-Mar-17	19	1	17	3	3	1	0	4	4	2	2
1444	202	0	1-Aug-17	19	1	17	3	3	1	0	4	4	2	1
1445	202	0	9-Jul-17	0	1	17	3	3	1	0	4	4	2	2
1446	202	0	30-May-12	8	1	17	3	2	1	0	11	4	3	2
1447	202	0	22-Aug-11	8	1	17	3	3	1	0	11	4	1	2
1448	202	0	18-Jul-10	19	1	17	3	1	1	0	11	4	3	1
1449	202	0	12-Aug-09	22	1	17	3	3	1	0	11	4	6	2
1450	202	0	23-Jun-16	14	1	17	3	3	1	0	3	4	2	1
1451	202	0	26-May-16	19	1	17	3	1	1	0	3	4	3	1
1452	202	0	3-Sep-15	16	1	17	3	3	1	0	3	4	4	1
1453	202	0	1-Nov-15	0	1	17	3	1	1	0	3	4	2	2
1454	202	0	30-Sep-14	13	1	17	3	3	1	0	3	4	2	1
1455	202	0	5-Jan-14	18	1	17	3	3	1	0	3	4	3	1
1456	202	0	26-Oct-14	16	1	17	3	3	1	0	3	4	5	1
1457	202	0	7-Apr-13	20	1	17	3	3	1	0	3	4	2	2
1458	202	0	23-Jul-13	20	1	17	3	1	1	0	3	4	5	2
1459	202	0	27-Nov-13	23	1	17	3	2	1	0	3	4	3	2
1460	202	0	16-Apr-12	12	1	17	3	1	1	0	3	4	1	1
1461	202	0	3-Feb-12	8	1	17	3	3	1	0	3	4	3	2
1462	202	0	14-Jul-12	23	1	17	3	1	1	0	3	4	1	1
1463	202	0	3-May-11	10	1	17	3	1	1	0	3	4	4	2
1464	202	0	7-Mar-10	11	1	17	3	1	1	0	3	4	3	1
1465	202	0	16-Jul-08	21	1	17	3	3	1	0	3	4	2	1
1466	202	0	20-Dec-06	8	1	17	3	3	1	0	3	4	1	2
1467	202	0	29-Jul-06	3	1	17	3	3	1	0	3	4	5	2
1468	202	0	14-Dec-06	4	1	17	3	2	1	0	3	4	1	2
1469	202	0	9-Nov-06	22	1	17	3	3	1	0	3	4	1	1
1470	202	0	6-Nov-06	14	1	17	3	3	1	0	3	4	3	1
1471	202	0	25-Aug-06	15	1	17	3	3	1	0	3	4	1	1
1472	202	0	15-Jun-17	8	1	17	3	3	1	0	3	4	1	1
1473	202	0	4-Dec-17	20	1	17	3	3	1	0	3	4	2	1
1474	202	0	25-Apr-17	14	1	17	3	3	1	0	3	4	1	1
1475	202	0	19-Dec-16	16	1	17	3	3	1	0	6	4	2	1
1476	202	0	5-Jul-16	9	1	17	3	1	1	0	6	4	3	1
1477	202	0	24-May-13	8	1	17	3	1	1	0	6	4	1	1
1478	202	0	1-Sep-13	5	1	17	3	3	1	0	6	4	2	1
1479	202	0	1-Jan-13	10	1	17	3	3	1	0	6	4	1	1
1480	202	0	19-Oct-13	2	1	17	3	2	1	0	6	4	2	2
1481	202	0	9-Sep-13	11	1	17	3	3	1	0	6	4	1	2

1482	202	0	13-Sep-12	21	1	17	3	3	1	0	6	4	2	1
1483	202	0	28-Oct-12	16	1	17	3	1	1	0	6	4	2	2
1484	202	0	27-Nov-11	11	1	17	3	1	1	0	6	4	2	2
1485	202	0	11-Apr-11	8	1	17	3	2	1	0	6	4	2	2
1486	202	0	22-Dec-10	11	1	17	3	1	1	0	6	4	2	2
1487	202	0	4-Dec-10	18	1	17	3	2	1	0	6	4	2	1
1488	202	0	25-Sep-10	12	1	17	3	1	1	0	6	4	2	1
1489	202	0	20-Apr-10	12	1	17	3	3	1	0	6	4	2	1
1490	202	0	5-Oct-10	19	1	17	3	3	1	0	6	4	2	2
1491	202	0	29-Dec-10	18	1	17	3	1	1	0	6	4	2	2
1492	202	0	6-Nov-10	4	1	17	3	1	1	0	6	4	6	2
1493	202	0	31-Oct-10	1	1	17	3	1	1	0	6	4	1	2
1494	202	0	29-Jul-10	19	1	17	3	3	1	0	6	4	2	1
1495	202	0	10-Nov-09	18	1	17	3	2	1	0	6	4	2	2
1496	202	0	25-Jul-09	12	1	17	3	3	1	0	6	4	2	2
1497	202	0	14-Dec-09	21	1	17	3	3	1	0	6	4	2	1
1498	202	0	18-Jun-09	20	1	17	3	3	1	0	6	4	2	2
1499	202	0	20-Jul-09	6	1	17	3	3	1	0	6	4	2	2
1500	202	0	14-Oct-08	11	1	17	3	3	1	0	6	4	2	1
1501	202	0	14-Aug-08	19	1	17	3	1	1	0	6	4	2	1
1502	202	0	23-Jul-08	2	1	17	3	3	1	0	6	4	2	2
1503	202	0	16-Mar-08	17	1	17	3	1	1	0	6	4	2	2
1504	202	0	25-May-08	14	1	17	3	3	1	0	6	4	2	1
1505	202	0	2-Mar-08	19	1	17	3	3	1	0	6	4	2	2
1506	202	0	19-Apr-07	21	1	17	3	3	1	0	6	4	2	2
1507	202	0	1-Jul-07	15	1	17	3	3	1	0	6	4	2	2
1508	202	0	18-Jul-07	8	1	17	3	1	1	0	6	4	2	1
1509	202	0	10-Aug-07	9	1	17	3	3	1	0	6	4	2	1
1510	202	0	11-Mar-07	20	1	17	3	1	1	0	6	4	2	1
1511	202	0	8-Dec-07	18	1	17	3	1	1	0	6	4	2	2
1512	202	0	31-Aug-07	9	1	17	3	1	1	0	6	4	2	1
1513	202	0	21-Mar-07	0	1	17	3	3	1	0	6	4	2	1
1514	202	0	26-Jun-07	19	1	17	3	2	1	0	6	4	3	1
1515	202	0	28-Jan-07	11	1	17	3	2	1	0	6	4	1	1
1516	202	0	8-Jul-07	20	1	17	3	1	1	0	6	4	2	1
1517	202	0	8-Mar-07	1	1	17	3	1	1	0	6	4	7	1
1518	202	0	10-Dec-06	12	1	17	3	2	1	0	6	4	1	2
1519	202	0	5-Mar-06	12	1	17	3	3	1	0	6	4	2	1
1520	202	0	10-Sep-06	2	1	17	3	3	1	0	6	4	2	1
1521	202	0	31-Oct-06	21	1	17	3	1	1	0	6	4	2	1
1522	202	0	24-Jun-06	17	1	17	3	3	1	0	6	4	2	2
1523	202	0	5-Mar-06	14	1	17	3	3	1	0	6	4	2	1
1524	202	0	5-Aug-17	22	1	17	3	3	1	0	6	4	2	1
1525	202	0	21-Sep-17	0	1	17	3	2	1	0	6	4	1	1
1526	202	0	5-Oct-09	2	1	17	3	3	1	0	13	4	2	2
1527	202	0	28-May-08	21	1	17	3	2	1	0	13	4	7	1
1528	202	0	12-Jun-07	15	1	17	3	1	1	0	13	4	3	1
1529	202	0	12-Aug-06	13	1	17	3	1	1	0	13	4	2	1
1530	202	0	1-Feb-14	17	2	17	3	3	1	0	1	4	1	1
1531	202	0	10-Jul-14	9	2	17	3	3	1	0	4	4	1	2
1532	202	0	16-Sep-15	21	2	17	3	1	1	0	6	4	2	1
1533	202	0	20-Feb-17	10	2	17	3	3	1	0	6	4	2	2
1534	202	0	5-Apr-12	6	2	17	3	1	1	0	6	4	2	1
1535	202	0	19-Jun-14	9	2	17	3	1	1	0	10	4	7	1
1536	202	0	25-May-09	23	2	17	3	1	1	0	10	4	7	1
1537	202	0	22-Dec-07	1	2	17	3	3	1	0	10	4	7	1
1538	202	0	22-Dec-07	1	2	17	3	3	1	0	10	4	7	2
1539	202	0	22-Dec-07	0	2	17	3	3	1	0	10	4	7	1
1540	202	0	22-Dec-07	0	2	17	3	3	1	0	10	4	7	1
1541	202	0	4-Mar-07	14	2	17	3	3	1	0	10	4	7	2
1542	202	0	11-Apr-13	1	2	17	3	3	1	0	7	4	2	2
1543	202	0	16-Dec-12	12	2	17	3	2	1	0	7	4	2	1
1544	202	0	12-Nov-07	21	2	17	3	1	1	0	13	4	2	2
1545	202	0	30-Aug-07	11	2	17	3	3	1	0	13	4	7	1
1546	202	0	20-Jul-06	17	2	17	3	3	1	0	13	4	2	2
1547	202	0	31-Jul-14	16	2	17	3	2	1	0	4	4	2	1
1548	202	0	17-Apr-13	7	2	17	3	2	1	0	4	4	2	2
1549	202	0	29-Nov-12	18	2	17	3	3	1	0	4	4	2	1
1550	202	0	21-Apr-10	17	2	17	3	1	1	0	4	4	2	1
1551	202	0	23-Jun-08	23	2	17	3	3	1	0	4	4	2	1
1552	202	0	20-Jul-08	14	2	17	3	2	1	0	4	4	2	2
1553	202	0	21-Nov-08	10	2	17	3	2	1	0	4	4	1	1
1554	202	0	13-Apr-07	17	2	17	3	3	1	0	4	4	2	2
1555	202	0	10-Apr-07	9	2	17	3	3	1	0	4	4	2	1
1556	202	0	8-Jul-07	0	2	17	3	3	1	0	4	4	1	1
1557	202	0	19-Jul-07	15	2	17	3	1	1	0	4	4	3	1
1558	202	0	23-Oct-06	6	2	17	3	3	1	0	4	4	2	1
1559	202	0	29-May-06	15	2	17	3	2	1	0	4	4	2	2
1560	202	0	6-Sep-06	19	2	17	3	3	1	0	4	4	3	2
1561	202	0	27-Sep-06	18	2	17	3	2	1	0	4	4	2	1
1562	202	0	24-Jun-06	18	2	17	3	3	1	0	4	4	2	2
1563	202	0	24-May-16	23	2	17	3	3	1	0	11	4	3	1
1564	202	0	8-Jul-14	21	2	17	3	1	1	0	11	4	7	2
1565	202	0	14-Aug-07	13	2	17	3	1	1	0	11	4	2	1
1566	202	0	24-Jul-12	6	2	17	3	3	1	0	3	4	6	1
1567	202	0	17-Nov-11	17	2	17	3	3	1	0	3	4	1	1
1568	202	0	1-Jul-11	11	2	17	3	1	1	0	3	4	1	1
1569	202	0	10-Nov-10	0	2	17	3	1	1	0	3	4	1	2
1570	202	0	25-Oct-07	7	2	17	3	3	1	0	3	4	1	1
1571	202	0	30-Aug-07	3	2	17	3	3	1	0	3	4	2	2
1572	202	0	9-Jan-12	18	2	17	3	1	1	0	6	4	2	2
1573	202	0	16-Sep-08	12	2	17	3	2	1	0	6	4	2	2
1574	202	0	6-Oct-08	10	2	17	3	3	1	0	6	4	7	1
1575	202	0	13-Apr-07	17	2	17	3	3	1	0	6	4	7	2
1576	202	0	19-Jan-06	13	2	17	3	4	1	0	6	4	2	1
1577	202	0	2-Dec-06	9	2	17	3	3	1	0	6	4	2	1
1578	201	1	11-Dec-12	2	1	17	3	3	1	0	4	4	1	1
1579	201	1	16-Aug-16	22	1	17	3	3	1	0	4	4	1	1
1580	101	1	7-Nov-14	13	1	17	3	2	1	0	6	4	2	1
1581	101	1	23-Jul-14	8	1	17	3	1	1	0	4	4	2	1
1582	201	1	28-May-15	3	1	17	3	3	1	0	6	4	2	1
1583	201	1	17-May-15	10	1	17	3	3	1	0	6	4	2	1
1584	201	1	19-Jun-13	10	1	17	3	2	1	0	4	4	3	2
1585	201	1	24-Oct-15	16	1	17	3	3	1	0	4	4	2	1
1586	201	1	5-Aug-12	4	1	17	3	2	1	0	4	4	4	1
1587	201	1	30-Sep-13	16	1	17	3	3	1	0	6	4	3	1
1588	201	1	7-Jul-12	16	1	17	3	3	1	0	6	4	3	1
1589	101	1	5-Feb-12	15	1	17	3	3	1	0	4	4	2	1
1590	101	1	23-Mar-12	9	1	17	3	1	1	0	4	4	2	2
1591	201	1	5-Nov-16	20	1	17	3	3	1	0	10	4	6	1
1592	201	1	15-Apr-17	17	1	17	3	3	1	0	6	4	4	1
1593	201	1	7-Nov-14	6	1	17	3	2	1	0	4	4	2	1
1594	201	1	16-Feb-14	13	1	17	3	3	1	0	4	4	2	2
1595	201	1	18-Jun-12	6	1	17	3	3	1	0	4	4	2	1
1596	201	1	24-Mar-12	19	1	17	3	3	1	0	4	4	2	2
1597	201	1	29-Aug-15	0	1	17	3	3	1	0	4	4	2	1
1598	201	1	8-Nov-15	18	1	17	3	3	1	0	6	4	2	1
1599	101	1	6-Aug-17	19	1	17	3	1	1	0	7	4	6	2
1600	201	1	3-Sep-13	1	1	17	3	3	1	0	4	4	3	1
1601	201	1	26-Jan-16	11	1	17	3	3	1	0	6	4	2	1
1602	101	1	10-Apr-12	18	1	17	3	1	1	0	6	4	2	1
1603	101	1	13-Jan-15	18	1	17	3	2	1	0	13	4	5	2
1604	101	1	2-Sep-12	18	1	17	3	3	1	0	6	4	2	2
1605	201	1	7-Jan-16	11	1	17	3	3	1	0	6	4	2	2

1606	201	1	13-May-15	15	1	17	3	3	1	0	6	4	2	1
1607	201	1	25-Mar-15	16	1	17	3	3	1	0	6	4	2	1
1608	201	1	29-Jun-12	22	1	17	3	3	1	0	6	4	2	2
1609	101	1	2-Apr-15	11	1	17	3	3	1	0	4	4	2	2
1610	201	1	21-Jun-16	21	1	17	3	3	1	0	4	4	3	1
1611	201	1	3-Jan-13	10	1	17	3	2	1	0	6	4	2	1
1612	201	1	6-Jul-12	18	1	17	3	3	1	0	6	4	2	1
1613	101	1	30-Jul-12	12	1	17	3	2	1	0	6	4	2	1
1614	101	1	30-Jul-12	12	1	17	3	2	1	0	6	4	2	1
1615	201	1	22-Dec-15	19	1	17	3	3	1	0	6	4	2	1
1616	101	1	16-Jan-12	19	1	17	3	1	1	0	6	4	2	1
1617	101	1	11-Feb-17	12	1	17	3	1	1	0	6	4	2	1
1618	201	1	27-Sep-17	22	1	17	3	2	1	0	6	4	2	1
1619	201	1	13-Jan-13	10	1	17	3	3	1	0	6	4	3	1
1620	201	1	2-Apr-12	9	1	17	3	3	1	0	6	4	2	1
1621	201	1	1-Dec-17	4	1	17	3	2	1	0	6	4	2	1
1622	201	1	22-May-16	10	1	17	3	3	1	0	6	4	2	1
1623	201	1	11-Jun-13	12	1	17	3	2	1	0	6	4	3	1
1624	201	1	26-Nov-13	8	1	17	3	3	1	0	6	4	2	1
1625	201	1	11-Sep-09	22	1	17	3	3	1	0	10	4	7	2
1626	101	1	6-Feb-15	14	1	17	3	2	1	0	7	4	3	1
1627	201	1	9-Jun-13	19	1	17	3	3	1	0	7	4	2	1
1628	101	1	21-Jul-12	11	1	17	3	2	1	0	7	4	3	2
1629	201	1	10-Jun-12	18	1	17	3	2	1	0	7	4	3	1
1630	101	1	25-Aug-12	11	1	17	3	2	1	0	7	4	2	1
1631	101	1	2-Feb-12	20	1	17	3	1	1	0	7	4	5	2
1632	101	1	18-Jul-11	13	1	17	3	3	1	0	7	4	3	2
1633	201	1	7-Mar-09	22	1	17	3	3	1	0	7	4	2	1
1634	101	1	27-Feb-09	6	1	17	3	3	1	0	7	4	3	1
1635	101	1	23-Oct-08	12	1	17	3	3	1	0	7	4	5	2
1636	101	1	23-Oct-08	12	1	17	3	3	1	0	7	4	5	1
1637	101	1	11-May-08	1	1	17	3	3	1	0	7	4	2	2
1638	101	1	14-Jul-08	8	1	17	3	3	1	0	7	4	2	1
1639	201	1	12-May-08	13	1	17	3	2	1	0	7	4	3	1
1640	101	1	5-Mar-06	8	1	17	3	3	1	0	7	4	2	1
1641	101	1	11-Mar-11	15	1	17	3	3	1	0	13	4	2	2
1642	101	1	25-Sep-09	0	1	17	3	3	1	0	13	4	2	4
1643	101	1	3-May-09	20	1	17	3	3	1	0	13	4	2	4
1644	201	1	7-Nov-07	14	1	17	3	3	1	0	13	4	2	1
1645	101	1	3-Jun-08	17	1	17	3	3	1	0	1	4	3	1
1646	201	1	4-Oct-10	15	1	17	3	3	1	0	1	4	2	1
1647	101	1	28-Apr-16	19	1	17	3	3	1	0	4	4	2	1
1648	201	1	30-Jun-15	16	1	17	3	2	1	0	4	4	2	1
1649	101	1	24-Dec-15	15	1	17	3	1	1	0	4	4	2	1
1650	101	1	7-Jul-15	17	1	17	3	3	1	0	4	4	2	4
1651	101	1	1-Jan-15	14	1	17	3	2	1	0	4	4	2	1
1652	101	1	11-Jun-15	10	1	17	3	3	1	0	4	4	5	1
1653	101	1	20-May-14	17	1	17	3	3	1	0	4	4	2	1
1654	101	1	8-Dec-14	12	1	17	3	2	1	0	4	4	2	1
1655	101	1	17-Sep-14	20	1	17	3	3	1	0	4	4	2	1
1656	101	1	21-Mar-14	10	1	17	3	3	1	0	4	4	2	1
1657	201	1	19-Jun-14	22	1	17	3	3	1	0	4	4	2	1
1658	201	1	27-Sep-14	20	1	17	3	2	1	0	4	4	2	1
1659	101	1	4-Jul-14	18	1	17	3	3	1	0	4	4	1	1
1660	101	1	12-Apr-14	10	1	17	3	3	1	0	4	4	2	4
1661	201	1	13-Jul-14	7	1	17	3	3	1	0	4	4	3	1
1662	101	1	16-Jan-13	16	1	17	3	3	1	0	4	4	1	1
1663	101	1	25-Apr-13	4	1	17	3	1	1	0	4	4	2	2
1664	101	1	15-Jun-13	14	1	17	3	3	1	0	4	4	2	1
1665	101	1	2-Jun-13	16	1	17	3	3	1	0	4	4	2	1
1666	101	1	21-Oct-13	10	1	17	3	3	1	0	4	4	2	1
1667	101	1	12-Aug-13	22	1	17	3	3	1	0	4	4	2	1
1668	101	1	31-Jul-13	20	1	17	3	2	1	0	4	4	2	2
1669	101	1	9-May-13	17	1	17	3	2	1	0	4	4	2	1
1670	201	1	14-Jun-12	4	1	17	3	3	1	0	4	4	1	1
1671	101	1	30-Jul-12	18	1	17	3	3	1	0	4	4	3	1
1672	101	1	28-Feb-12	17	1	17	3	3	1	0	4	4	2	1
1673	101	1	8-Feb-12	16	1	17	3	2	1	0	4	4	2	2
1674	201	1	18-Jun-12	17	1	17	3	3	1	0	4	4	2	1
1675	101	1	30-Dec-12	9	1	17	3	3	1	0	4	4	2	4
1676	101	1	15-Oct-12	23	1	17	3	3	1	0	4	4	2	1
1677	101	1	14-Aug-12	9	1	17	3	3	1	0	4	4	2	1
1678	201	1	7-Apr-11	15	1	17	3	1	1	0	4	4	2	1
1679	101	1	16-Nov-11	15	1	17	3	3	1	0	4	4	3	2
1680	101	1	9-Aug-11	9	1	17	3	3	1	0	4	4	2	1
1681	101	1	3-Jul-11	15	1	17	3	3	1	0	4	4	2	2
1682	101	1	24-Mar-11	18	1	17	3	3	1	0	4	4	2	1
1683	101	1	16-Oct-11	16	1	17	3	3	1	0	4	4	2	1
1684	201	1	9-Apr-11	16	1	17	3	1	1	0	4	4	3	4
1685	201	1	22-Oct-11	15	1	17	3	2	1	0	4	4	2	2
1686	201	1	2-Jun-11	8	1	17	3	3	1	0	4	4	2	1
1687	201	1	4-Sep-11	20	1	17	3	3	1	0	4	4	2	2
1688	201	1	23-Aug-11	7	1	17	3	3	1	0	4	4	2	1
1689	201	1	28-Feb-11	15	1	17	3	3	1	0	4	4	2	1
1690	201	1	6-Jun-11	2	1	17	3	3	1	0	4	4	2	1
1691	101	1	20-Nov-11	23	1	17	3	2	1	0	4	4	2	2
1692	101	1	30-May-11	4	1	17	3	2	1	0	4	4	1	1
1693	101	1	22-Dec-11	22	1	17	3	3	1	0	4	4	1	1
1694	201	1	19-Jan-10	17	1	17	3	2	1	0	4	4	2	1
1695	101	1	3-Feb-10	18	1	17	3	3	1	0	4	4	2	1
1696	101	1	17-Oct-10	15	1	17	3	1	1	0	4	4	2	1
1697	101	1	7-Dec-10	18	1	17	3	2	1	0	4	4	3	1
1698	101	1	8-May-10	9	1	17	3	3	1	0	4	4	2	1
1699	101	1	23-Mar-10	7	1	17	3	3	1	0	4	4	3	1
1700	201	1	28-Jul-10	14	1	17	3	3	1	0	4	4	2	1
1701	101	1	18-Nov-10	11	1	17	3	3	1	0	4	4	2	1
1702	201	1	3-Jun-10	16	1	17	3	3	1	0	4	4	2	1
1703	201	1	26-Nov-10	17	1	17	3	3	1	0	4	4	2	1
1704	201	1	29-Apr-10	10	1	17	3	3	1	0	4	4	2	2
1705	201	1	17-Sep-10	19	1	17	3	3	1	0	4	4	2	1
1706	201	1	27-Jul-10	1	1	17	3	3	1	0	4	4	2	1
1707	201	1	18-Aug-10	19	1	17	3	3	1	0	4	4	2	1
1708	201	1	15-Dec-10	13	1	17	3	2	1	0	4	4	3	1
1709	101	1	28-Aug-09	1	1	17	3	3	1	0	4	4	2	1
1710	101	1	8-Nov-09	12	1	17	3	3	1	0	4	4	4	1
1711	101	1	21-May-09	19	1	17	3	3	1	0	4	4	2	1
1712	101	1	21-Jul-09	9	1	17	3	3	1	0	4	4	2	4
1713	201	1	30-Mar-09	18	1	17	3	3	1	0	4	4	2	2
1714	201	1	26-Sep-09	21	1	17	3	3	1	0	4	4	2	1
1715	201	1	9-Dec-09	23	1	17	3	2	1	0	4	4	2	2
1716	201	1	13-Sep-09	16	1	17	3	2	1	0	4	4	2	1
1717	201	1	25-Aug-09	6	1	17	3	3	1	0	4	4	2	1
1718	101	1	16-Sep-09	2	1	17	3	2	1	0	4	4	3	2
1719	101	1	25-Dec-09	9	1	17	3	3	1	0	4	4	2	4
1720	101	1	15-Feb-08	17	1	17	3	3	1	0	4	4	3	1
1721	101	1	4-Mar-08	19	1	17	3	2	1	0	4	4	2	2
1722	101	1	4-Nov-08	17	1	17	3	3	1	0	4	4	2	1
1723	201	1	3-Sep-08	12	1	17	3	3	1	0	4	4	4	2
1724	201	1	28-Mar-08	18	1	17	3	3	1	0	4	4	2	1
1725	201	1	21-Dec-08	13	1	17	3	3	1	0	4	4	2	1
1726	201	1	23-Jul-08	19	1	17	3	3	1	0	4	4	2	2
1727	201	1	7-Aug-08	9	1	17	3	3	1	0	4	4	4	1
1728	201	1	25-Jul-08	14	1	17	3	3	1	0	4	4	7	1
1729	201	1	29-Sep-08	20	1	17	3	3	1	0	4	4	2	2

1730	201	1	25-Dec-08	21	1	17	3	3	1	0	4	4	2	1
1731	201	1	28-Aug-08	12	1	17	3	3	1	0	4	4	2	1
1732	201	1	15-May-08	18	1	17	3	3	1	0	4	4	2	1
1733	201	1	26-Jul-08	16	1	17	3	3	1	0	4	4	4	1
1734	201	1	7-Dec-08	15	1	17	3	3	1	0	4	4	2	1
1735	101	1	3-Sep-08	10	1	17	3	3	1	0	4	4	2	1
1736	201	1	7-May-08	10	1	17	3	3	1	0	4	4	3	1
1737	101	1	4-Jul-08	17	1	17	3	1	1	0	4	4	2	1
1738	201	1	12-Sep-08	21	1	17	3	2	1	0	4	4	2	1
1739	101	1	12-Jul-07	10	1	17	3	3	1	0	4	4	2	2
1740	101	1	28-Mar-07	10	1	17	3	3	1	0	4	4	1	1
1741	201	1	13-Dec-07	23	1	17	3	3	1	0	4	4	2	2
1742	201	1	19-Jul-07	19	1	17	3	3	1	0	4	4	2	1
1743	201	1	16-Feb-07	6	1	17	3	2	1	0	4	4	2	1
1744	101	1	30-Jul-07	14	1	17	3	2	1	0	4	4	3	1
1745	101	1	6-Dec-07	19	1	17	3	3	1	0	4	4	2	2
1746	101	1	14-Jul-07	2	1	17	3	2	1	0	4	4	2	2
1747	201	1	7-Jan-07	18	1	17	3	3	1	0	4	4	1	1
1748	201	1	6-Aug-07	17	1	17	3	3	1	0	4	4	2	1
1749	201	1	26-Jun-07	15	1	17	3	3	1	0	4	4	2	1
1750	201	1	24-Dec-07	16	1	17	3	3	1	0	4	4	2	2
1751	101	1	9-Oct-07	21	1	17	3	1	1	0	4	4	1	1
1752	201	1	18-Sep-07	16	1	17	3	2	1	0	4	4	2	1
1753	101	1	14-Nov-07	20	1	17	3	3	1	0	4	4	2	1
1754	101	1	5-Sep-07	10	1	17	3	2	1	0	4	4	3	1
1755	201	1	18-Sep-07	1	1	17	3	3	1	0	4	4	1	1
1756	101	1	8-Jun-07	14	1	17	3	3	1	0	4	4	3	1
1757	101	1	8-Dec-06	13	1	17	3	3	1	0	4	4	2	1
1758	101	1	28-Mar-06	7	1	17	3	3	1	0	4	4	2	2
1759	101	1	5-Dec-06	21	1	17	3	3	1	0	4	4	2	1
1760	101	1	6-Nov-06	19	1	17	3	3	1	0	4	4	2	1
1761	101	1	11-Dec-06	16	1	17	3	3	1	0	4	4	2	1
1762	101	1	8-Apr-06	14	1	17	3	3	1	0	4	4	2	2
1763	201	1	15-May-06	3	1	17	3	1	1	0	4	4	2	2
1764	101	1	6-Oct-06	12	1	17	3	1	1	0	4	4	2	1
1765	201	1	14-Apr-06	17	1	17	3	3	1	0	4	4	2	2
1766	201	1	10-Nov-06	13	1	17	3	1	1	0	4	4	1	1
1767	201	1	6-Mar-06	14	1	17	3	3	1	0	4	4	2	1
1768	201	1	17-Jun-06	8	1	17	3	3	1	0	4	4	2	1
1769	101	1	7-Oct-06	20	1	17	3	3	1	0	4	4	3	1
1770	101	1	17-Jan-17	14	1	17	3	1	1	0	4	4	3	1
1771	201	1	14-Nov-17	22	1	17	3	2	1	0	4	4	1	1
1772	101	1	21-May-15	12	1	17	3	3	1	0	11	4	3	1
1773	101	1	26-Dec-11	10	1	17	3	1	1	0	11	4	2	1
1774	101	1	27-Mar-16	17	1	17	3	3	1	0	3	4	2	2
1775	201	1	6-Nov-15	19	1	17	3	2	1	0	3	4	4	1
1776	101	1	24-Apr-14	12	1	17	3	3	1	0	3	4	1	2
1777	201	1	10-Apr-14	13	1	17	3	3	1	0	3	4	2	1
1778	101	1	23-Jul-14	20	1	17	3	3	1	0	3	4	1	2
1779	101	1	15-Sep-13	18	1	17	3	3	1	0	3	4	1	2
1780	101	1	29-May-13	11	1	17	3	3	1	0	3	4	3	2
1781	101	1	25-Feb-13	13	1	17	3	3	1	0	3	4	1	1
1782	201	1	14-Nov-12	15	1	17	3	3	1	0	3	4	3	2
1783	101	1	10-Oct-12	10	1	17	3	3	1	0	3	4	3	1
1784	201	1	13-Dec-11	6	1	17	3	2	1	0	3	4	3	1
1785	201	1	13-Oct-11	10	1	17	3	3	1	0	3	4	3	1
1786	101	1	2-Jun-11	15	1	17	3	2	1	0	3	4	2	4
1787	101	1	12-Dec-10	18	1	17	3	3	1	0	3	4	1	1
1788	201	1	21-Aug-10	10	1	17	3	2	1	0	3	4	2	2
1789	201	1	1-Nov-10	22	1	17	3	2	1	0	3	4	1	1
1790	201	1	19-Aug-10	20	1	17	3	3	1	0	3	4	3	1
1791	201	1	17-Oct-10	22	1	17	3	3	1	0	3	4	2	1
1792	101	1	21-May-10	19	1	17	3	2	1	0	3	4	1	2
1793	201	1	9-Oct-09	13	1	17	3	3	1	0	3	4	1	1
1794	201	1	9-Jul-09	8	1	17	3	3	1	0	3	4	1	1
1795	201	1	19-Jun-08	6	1	17	3	3	1	0	3	4	2	1
1796	201	1	23-Jul-08	20	1	17	3	3	1	0	3	4	1	2
1797	201	1	11-Apr-08	18	1	17	3	3	1	0	3	4	4	2
1798	101	1	6-Jan-08	8	1	17	3	3	1	0	3	4	5	2
1799	201	1	23-Mar-08	14	1	17	3	2	1	0	3	4	2	1
1800	201	1	1-Feb-08	7	1	17	3	3	1	0	3	4	5	1
1801	201	1	25-Nov-07	10	1	17	3	3	1	0	3	4	3	2
1802	201	1	17-Jul-07	18	1	17	3	3	1	0	3	4	3	1
1803	201	1	24-Jun-07	9	1	17	3	1	1	0	3	4	2	1
1804	201	1	14-Jul-07	19	1	17	3	3	1	0	3	4	4	2
1805	201	1	31-Oct-07	22	1	17	3	3	1	0	3	4	1	4
1806	101	1	16-Aug-07	8	1	17	3	3	1	0	3	4	3	1
1807	101	1	29-Apr-06	11	1	17	3	2	1	0	3	4	5	1
1808	101	1	1-Sep-06	15	1	17	3	1	1	0	3	4	1	1
1809	101	1	25-Jan-15	12	1	17	3	3	1	0	6	4	2	1
1810	101	1	30-Jun-15	10	1	17	3	2	1	0	6	4	2	1
1811	101	1	6-Apr-14	18	1	17	3	2	1	0	6	4	3	1
1812	101	1	12-Mar-14	19	1	17	3	3	1	0	6	4	2	1
1813	101	1	31-Jan-13	11	1	17	3	2	1	0	6	4	2	1
1814	101	1	11-Dec-13	8	1	17	3	2	1	0	6	4	2	1
1815	101	1	28-Sep-13	1	1	17	3	3	1	0	6	4	3	1
1816	101	1	3-Oct-13	12	1	17	3	3	1	0	6	4	2	1
1817	101	1	9-Feb-13	8	1	17	3	2	1	0	6	4	2	1
1818	101	1	25-Sep-12	11	1	17	3	2	1	0	6	4	2	1
1819	101	1	18-Jun-12	10	1	17	3	2	1	0	6	4	2	1
1820	101	1	19-Sep-12	14	1	17	3	2	1	0	6	4	2	1
1821	101	1	2-May-12	11	1	17	3	3	1	0	6	4	2	1
1822	201	1	15-Aug-12	0	1	17	3	3	1	0	6	4	1	1
1823	101	1	18-Oct-11	11	1	17	3	2	1	0	6	4	2	1
1824	201	1	27-Apr-11	16	1	17	3	2	1	0	6	4	2	1
1825	201	1	28-Mar-11	15	1	17	3	2	1	0	6	4	2	2
1826	201	1	15-Mar-11	10	1	17	3	1	1	0	6	4	2	1
1827	101	1	17-Mar-11	2	1	17	3	3	1	0	6	4	2	2
1828	201	1	9-Mar-11	1	1	17	3	2	1	0	6	4	1	1
1829	101	1	26-Mar-10	1	1	17	3	1	1	0	6	4	2	1
1830	101	1	25-Aug-10	20	1	17	3	3	1	0	6	4	3	1
1831	101	1	3-Dec-10	14	1	17	3	3	1	0	6	4	2	1
1832	201	1	31-Mar-10	5	1	17	3	3	1	0	6	4	2	1
1833	101	1	14-Apr-10	19	1	17	3	2	1	0	6	4	2	1
1834	201	1	27-Apr-10	13	1	17	3	3	1	0	6	4	3	1
1835	101	1	4-Feb-10	18	1	17	3	2	1	0	6	4	2	1
1836	201	1	11-Jan-10	16	1	17	3	1	1	0	6	4	2	1
1837	101	1	28-Aug-10	13	1	17	3	2	1	0	6	4	2	1
1838	101	1	4-Oct-10	17	1	17	3	2	1	0	6	4	2	2
1839	201	1	13-Aug-10	12	1	17	3	3	1	0	6	4	3	1
1840	101	1	18-Jan-10	12	1	17	3	2	1	0	6	4	2	1
1841	101	1	29-Apr-10	17	1	17	3	2	1	0	6	4	2	1
1842	201	1	30-Aug-10	21	1	17	3	3	1	0	6	4	2	2
1843	201	1	3-Mar-10	23	1	17	3	1	1	0	6	4	2	1
1844	201	1	24-Oct-09	14	1	17	3	2	1	0	6	4	2	1
1845	101	1	27-Aug-09	12	1	17	3	2	1	0	6	4	2	1
1846	201	1	17-Feb-09	11	1	17	3	2	1	0	6	4	2	2
1847	201	1	1-Oct-09	15	1	17	3	2	1	0	6	4	2	1
1848	101	1	26-Sep-09	16	1	17	3	3	1	0	6	4	3	1
1849	201	1	17-Oct-09	15	1	17	3	2	1	0	6	4	2	1
1850	101	1	14-Sep-09	12	1	17	3	3	1	0	6	4	2	1
1851	101	1	1-Sep-09	1	1	17	3	2	1	0	6	4	2	4
1852	101	1	26-Jun-09	13	1	17	3	2	1	0	6	4	2	1
1853	101	1	21-Dec-09	2	1	17	3	2	1	0	6	4	2	1

1854	101	1	22-Feb-09	3	1	17	3	1	1	0	6	4	2	1
1855	101	1	16-Jul-09	11	1	17	3	2	1	0	6	4	2	4
1856	101	1	30-Aug-08	11	1	17	3	3	1	0	6	4	2	1
1857	101	1	17-May-08	16	1	17	3	2	1	0	6	4	2	1
1858	201	1	8-Nov-08	11	1	17	3	3	1	0	6	4	2	2
1859	201	1	5-Mar-08	17	1	17	3	3	1	0	6	4	2	1
1860	201	1	24-Nov-08	16	1	17	3	2	1	0	6	4	2	1
1861	201	1	3-Apr-08	22	1	17	3	3	1	0	6	4	2	2
1862	201	1	25-May-08	9	1	17	3	3	1	0	6	4	2	1
1863	201	1	2-Aug-08	21	1	17	3	3	1	0	6	4	2	1
1864	101	1	22-Mar-08	7	1	17	3	2	1	0	6	4	2	1
1865	201	1	12-Dec-08	18	1	17	3	3	1	0	6	4	2	1
1866	101	1	11-Oct-08	15	1	17	3	1	1	0	6	4	2	1
1867	101	1	8-Nov-08	10	1	17	3	3	1	0	6	4	2	2
1868	101	1	10-Jan-08	18	1	17	3	2	1	0	6	4	2	1
1869	201	1	30-Sep-08	1	1	17	3	3	1	0	6	4	2	1
1870	201	1	14-May-07	0	1	17	3	1	1	0	6	4	1	1
1871	201	1	13-Aug-07	11	1	17	3	3	1	0	6	4	3	1
1872	201	1	22-Apr-07	22	1	17	3	3	1	0	6	4	4	1
1873	201	1	8-Oct-07	8	1	17	3	3	1	0	6	4	2	1
1874	201	1	16-Dec-07	10	1	17	3	3	1	0	6	4	2	1
1875	201	1	24-Apr-07	5	1	17	3	3	1	0	6	4	2	1
1876	201	1	17-Apr-07	18	1	17	3	2	1	0	6	4	3	1
1877	201	1	22-Dec-07	14	1	17	3	3	1	0	6	4	2	1
1878	201	1	21-Jul-07	19	1	17	3	1	1	0	6	4	2	1
1879	201	1	8-Oct-07	9	1	17	3	3	1	0	6	4	4	1
1880	201	1	9-Jun-07	21	1	17	3	3	1	0	6	4	2	1
1881	201	1	29-Jul-07	21	1	17	3	3	1	0	6	4	2	1
1882	101	1	11-Nov-07	11	1	17	3	1	1	0	6	4	2	1
1883	201	1	25-Aug-07	9	1	17	3	3	1	0	6	4	2	1
1884	101	1	7-Jul-07	23	1	17	3	2	1	0	6	4	2	1
1885	201	1	2-Oct-07	22	1	17	3	3	1	0	6	4	2	1
1886	101	1	2-Jul-07	18	1	17	3	1	1	0	6	4	5	1
1887	201	1	15-Mar-07	6	1	17	3	3	1	0	6	4	2	1
1888	201	1	15-Mar-07	6	1	17	3	3	1	0	6	4	2	1
1889	101	1	7-Sep-07	18	1	17	3	3	1	0	6	4	1	1
1890	101	1	3-Apr-07	15	1	17	3	2	1	0	6	4	2	1
1891	201	1	31-May-07	22	1	17	3	3	1	0	6	4	1	1
1892	101	1	2-Jan-06	20	1	17	3	3	1	0	6	4	2	1
1893	101	1	6-Oct-06	11	1	17	3	3	1	0	6	4	2	2
1894	101	1	23-Apr-06	22	1	17	3	3	1	0	6	4	2	2
1895	201	1	29-Jul-06	19	1	17	3	3	1	0	6	4	2	1
1896	101	1	30-Jul-06	11	1	17	3	3	1	0	6	4	3	1
1897	101	1	8-Sep-06	11	1	17	3	3	1	0	6	4	2	1
1898	201	1	19-Aug-06	11	1	17	3	2	1	0	6	4	2	1
1899	201	1	6-Sep-06	17	1	17	3	3	1	0	6	4	2	1
1900	201	1	10-Jan-06	2	1	17	3	3	1	0	6	4	2	1
1901	101	1	6-Aug-06	14	1	17	3	3	1	0	6	4	3	1
1902	101	1	30-Mar-06	8	1	17	3	3	1	0	6	4	2	1
1903	201	1	23-Aug-17	12	1	17	3	3	1	0	6	4	3	1
1904	201	1	11-Sep-11	6	1	17	3	3	1	0	13	4	2	1
1905	201	1	5-Jul-12	11	2	17	3	3	1	0	6	4	2	1
1906	101	1	27-Jul-12	14	2	17	3	1	1	0	6	4	2	1
1907	201	1	10-Apr-09	19	2	17	3	3	1	0	10	4	7	1
1908	101	1	4-May-16	13	2	17	3	1	1	0	7	4	4	1
1909	101	1	4-May-16	13	2	17	3	3	1	0	7	4	2	1
1910	101	1	14-Apr-12	20	2	17	3	3	1	0	7	4	3	1
1911	201	1	16-Sep-09	14	2	17	3	3	1	0	7	4	2	1
1912	101	1	19-Oct-08	20	2	17	3	3	1	0	7	4	2	1
1913	101	1	23-Oct-07	4	2	17	3	3	1	0	7	4	2	2
1914	201	1	18-Nov-06	23	2	17	3	3	1	0	13	4	2	1
1915	201	1	9-Jun-06	23	2	17	3	2	1	0	13	4	2	1
1916	101	1	20-Jan-14	7	2	17	3	3	1	0	4	4	2	1
1917	101	1	25-May-13	8	2	17	3	3	1	0	4	4	1	1
1918	101	1	4-Dec-11	10	2	17	3	3	1	0	4	4	1	1
1919	101	1	23-Apr-11	13	2	17	3	3	1	0	4	4	2	1
1920	101	1	17-May-10	21	2	17	3	3	1	0	4	4	2	4
1921	101	1	25-Oct-10	9	2	17	3	3	1	0	4	4	2	1
1922	201	1	22-Sep-10	11	2	17	3	2	1	0	4	4	2	1
1923	101	1	24-Feb-09	15	2	17	3	1	1	0	4	4	2	1
1924	201	1	10-Jul-09	17	2	17	3	3	1	0	4	4	2	1
1925	201	1	10-Sep-08	23	2	17	3	3	1	0	4	4	2	1
1926	201	1	14-Sep-08	9	2	17	3	2	1	0	4	4	2	1
1927	201	1	12-May-08	11	2	17	3	2	1	0	4	4	2	2
1928	201	1	31-Oct-08	1	2	17	3	3	1	0	4	4	2	1
1929	101	1	7-Apr-08	10	2	17	3	2	1	0	4	4	1	1
1930	101	1	2-Jul-07	3	2	17	3	3	1	0	4	4	2	1
1931	101	1	23-May-07	19	2	17	3	3	1	0	4	4	2	1
1932	101	1	16-Apr-07	8	2	17	3	3	1	0	4	4	2	2
1933	101	1	27-Aug-07	21	2	17	3	3	1	0	4	4	2	1
1934	101	1	1-Sep-07	22	2	17	3	3	1	0	4	4	2	1
1935	201	1	6-Feb-07	17	2	17	3	2	1	0	4	4	3	1
1936	101	1	5-Jul-07	16	2	17	3	3	1	0	4	4	2	1
1937	201	1	10-Oct-07	20	2	17	3	2	1	0	4	4	2	1
1938	201	1	23-Apr-07	10	2	17	3	3	1	0	4	4	2	1
1939	101	1	8-Jun-07	15	2	17	3	3	1	0	4	4	2	1
1940	101	1	8-Jun-07	6	2	17	3	3	1	0	4	4	2	2
1941	101	1	11-Apr-06	16	2	17	3	2	1	0	4	4	2	1
1942	101	1	7-Jun-06	22	2	17	3	3	1	0	4	4	2	1
1943	201	1	4-Mar-06	18	2	17	3	3	1	0	4	4	2	1
1944	201	1	22-Jan-06	16	2	17	3	3	1	0	4	4	2	2
1945	201	1	24-Oct-06	23	2	17	3	3	1	0	4	4	2	1
1946	201	1	7-Jan-06	9	2	17	3	2	1	0	4	4	2	1
1947	201	1	14-Aug-06	12	2	17	3	3	1	0	4	4	2	1
1948	101	1	10-Sep-06	11	2	17	3	2	1	0	4	4	2	1
1949	201	1	20-Oct-16	19	2	17	3	2	1	0	3	4	1	1
1950	101	1	4-Mar-14	10	2	17	3	1	1	0	3	4	4	1
1951	201	1	11-Apr-14	4	2	17	3	1	1	0	3	4	2	1
1952	101	1	2-Mar-12	18	2	17	3	3	1	0	3	4	4	1
1953	101	1	7-Jan-11	9	2	17	3	3	1	0	3	4	2	1
1954	201	1	8-Feb-11	2	2	17	3	1	1	0	3	4	4	2
1955	101	1	4-Feb-10	0	2	17	3	3	1	0	3	4	1	1
1956	101	1	20-Apr-10	20	2	17	3	1	1	0	3	4	2	1
1957	201	1	14-May-09	6	2	17	3	1	1	0	3	4	3	1
1958	201	1	7-Jun-09	22	2	17	3	3	1	0	3	4	1	1
1959	201	1	17-Oct-09	21	2	17	3	2	1	0	3	4	5	1
1960	201	1	15-Nov-08	20	2	17	3	2	1	0	3	4	1	1
1961	201	1	4-May-08	20	2	17	3	3	1	0	3	4	2	1
1962	201	1	30-Dec-08	20	2	17	3	2	1	0	3	4	3	1
1963	201	1	22-Jul-08	20	2	17	3	3	1	0	3	4	3	1
1964	201	1	13-May-07	8	2	17	3	3	1	0	3	4	2	1
1965	101	1	25-Dec-06	21	2	17	3	3	1	0	3	4	4	2
1966	201	1	19-Jun-06	20	2	17	3	3	1	0	3	4	3	1
1967	101	1	31-Jul-13	23	2	17	3	2	1	0	6	4	3	1
1968	101	1	12-Sep-12	22	2	17	3	2	1	0	6	4	2	1
1969	201	1	21-Sep-12	15	2	17	3	3	1	0	6	4	2	1
1970	101	1	7-Jun-11	22	2	17	3	3	1	0	6	4	1	1
1971	101	1	2-Oct-10	21	2	17	3	3	1	0	6	4	1	1
1972	101	1	10-Dec-09	9	2	17	3	3	1	0	6	4	2	1
1973	201	1	11-Jun-09	9	2	17	3	3	1	0	6	4	2	1
1974	101	1	24-Dec-09	18	2	17	3	3	1	0	6	4	3	1
1975	201	1	11-Nov-09	10	2	17	3	3	1	0	6	4	2	1
1976	101	1	13-Jul-08	2	2	17	3	3	1	0	6	4	2	1
1977	101	1	23-Apr-08	19	2	17	3	2	1	0	6	4	2	1

1978	201	1	9-Feb-08	18	2	17	3	3	1	0	6	4	1	1
1979	201	1	13-Mar-07	18	2	17	3	3	1	0	6	4	2	1
1980	201	1	8-Oct-07	9	2	17	3	3	1	0	6	4	2	1
1981	101	1	9-Jul-07	19	2	17	3	3	1	0	6	4	2	1
1982	201	1	7-Jul-07	6	2	17	3	3	1	0	6	4	2	1
1983	201	1	1-Dec-07	22	2	17	3	3	1	0	6	4	2	1
1984	201	1	30-Jul-07	0	2	17	3	3	1	0	6	4	2	1
1985	101	1	3-Nov-07	19	2	17	3	3	1	0	6	4	2	1
1986	201	1	12-May-06	18	2	17	3	3	1	0	6	4	2	1
1987	201	1	28-Apr-06	14	2	17	3	3	1	0	6	4	2	1
1988	201	1	19-Sep-06	11	2	17	3	1	1	0	6	4	2	1
1989	201	1	26-Aug-06	8	2	17	3	3	1	0	6	4	2	1
1990	101	1	14-Apr-06	17	2	17	3	3	1	0	6	4	2	1
1991	201	1	17-Mar-06	19	2	17	3	2	1	0	6	4	2	1
1992	201	1	19-Sep-06	10	2	17	3	2	1	0	6	4	2	1
1993	201	1	10-Dec-12	8	2	17	3	3	1	0	13	4	4	1
1994	202	0	4-Jan-12	21	1	15	3	1	3	1	6	3	1	1
1995	101	1	7-Jul-13	4	1	15	3	2	3	1	4	3	1	1
1996	202	0	19-Nov-11	5	1	16	3	3	3	1	4	3	1	2
1997	202	0	22-Mar-11	17	1	16	3	3	3	1	6	3	6	2
1998	202	0	28-Jul-10	5	1	16	3	3	3	1	6	3	4	2
1999	202	0	15-May-14	19	2	16	3	3	3	1	4	3	1	1
2000	202	0	24-Aug-14	23	2	16	3	3	3	1	11	3	3	1
2001	202	0	20-May-11	1	2	16	3	3	3	1	6	3	7	2
2002	202	0	11-Mar-07	15	2	16	3	2	3	1	6	3	2	1
2003	101	1	2-Jul-15	2	1	16	3	3	3	1	4	3	1	1
2004	101	1	4-Jul-08	1	1	16	3	1	3	1	4	3	1	1
2005	101	1	5-Apr-07	18	1	16	3	3	3	1	4	3	4	1
2006	101	1	5-Apr-07	18	1	16	3	3	3	1	4	3	2	1
2007	101	1	29-Sep-07	3	1	16	3	1	3	1	4	3	6	1
2008	202	0	16-Oct-13	3	1	17	3	3	3	1	1	3	3	1
2009	202	0	5-Mar-12	0	1	17	3	3	3	1	6	3	5	2
2010	202	0	23-Jul-15	0	1	17	3	3	3	1	6	3	3	1
2011	202	0	12-Feb-08	21	1	17	3	3	3	1	13	3	1	2
2012	202	0	22-Mar-16	17	1	17	3	3	3	1	4	3	1	1
2013	202	0	9-Oct-15	13	1	17	3	3	3	1	4	3	1	1
2014	202	0	16-Jun-13	23	1	17	3	3	3	1	4	3	1	1
2015	202	0	16-Jun-13	23	1	17	3	3	3	1	4	3	1	1
2016	202	0	30-Oct-17	3	1	17	3	3	3	1	3	3	5	1
2017	202	0	18-Oct-16	5	1	17	3	3	3	1	6	3	3	2
2018	202	0	18-Sep-10	23	1	17	3	1	3	1	6	3	6	2
2019	202	0	28-Jul-10	5	1	17	3	3	3	1	6	3	4	2
2020	202	0	4-Sep-08	3	1	17	3	3	3	1	6	3	1	2
2021	202	0	20-Aug-08	18	1	17	3	3	3	1	6	3	1	1
2022	202	0	26-Oct-08	22	1	17	3	3	3	1	6	3	3	2
2023	202	0	1-Nov-07	21	1	17	3	3	3	1	6	3	6	2
2024	202	0	16-Jun-07	2	2	17	3	3	3	1	6	3	3	1
2025	101	1	6-Feb-13	17	1	17	3	2	3	1	4	3	1	2
2026	101	1	24-Aug-13	17	1	17	3	2	3	1	4	3	5	1
2027	101	1	5-Apr-07	18	1	17	3	3	3	1	4	3	2	1
2028	201	1	18-Mar-07	1	1	17	3	3	3	1	4	3	1	1
2029	201	1	25-Feb-07	14	1	17	3	3	3	1	11	3	3	1
2030	101	1	23-May-07	4	1	17	3	3	3	1	6	3	1	2
2031	101	1	19-Mar-06	2	1	17	3	2	3	1	6	3	1	2
2032	201	1	3-Feb-06	23	2	17	3	3	3	1	6	3	2	1
2033	202	0	23-Apr-11	22	1	12	3	1	1	0	1	3	6	1
2034	202	0	26-Jan-14	16	1	13	3	1	1	0	1	3	3	1
2035	202	0	22-Jul-17	21	1	13	3	2	1	0	3	3	5	2
2036	202	0	19-Oct-16	12	2	13	3	3	1	0	1	3	1	1
2037	202	0	26-Jun-13	19	2	13	3	1	1	0	1	3	3	1
2038	201	1	6-Mar-11	21	1	13	3	2	1	0	1	3	6	1
2039	202	0	4-May-14	20	1	14	3	1	1	0	1	3	2	2
2040	202	0	18-Apr-16	22	1	14	3	3	1	0	1	3	2	1
2041	202	0	24-May-13	11	1	14	3	2	1	0	1	3	2	1
2042	202	0	11-Mar-10	1	1	14	3	3	1	0	1	3	1	1
2043	202	0	30-Apr-09	19	1	14	3	2	1	0	1	3	1	2
2044	201	1	28-Oct-11	3	1	14	3	3	1	0	1	3	6	1
2045	202	0	18-Aug-16	5	1	15	3	3	1	0	1	3	1	2
2046	202	0	18-Aug-16	5	1	15	3	3	1	0	1	3	1	2
2047	202	0	7-Oct-13	12	1	15	3	1	1	0	1	3	5	2
2048	202	0	23-Aug-12	18	1	15	3	1	1	0	1	3	1	2
2049	202	0	7-Jun-17	18	1	15	3	1	1	0	1	3	1	1
2050	202	0	29-Mar-17	6	1	15	3	3	1	0	1	3	3	1
2051	202	0	9-Nov-17	19	1	15	3	3	1	0	1	3	3	2
2052	202	0	6-Jan-17	20	1	15	3	1	1	0	1	3	2	1
2053	202	0	11-Sep-17	0	1	15	3	1	1	0	1	3	5	1
2054	202	0	2-Jul-17	14	1	15	3	1	1	0	1	3	3	1
2055	202	0	22-Oct-11	1	1	15	3	3	1	0	1	3	5	2
2056	202	0	17-Dec-11	20	1	15	3	3	1	0	1	3	5	2
2057	202	0	27-Feb-11	1	1	15	3	1	1	0	1	3	4	2
2058	202	0	24-Oct-11	19	1	15	3	1	1	0	1	3	1	1
2059	202	0	28-Feb-11	8	1	15	3	1	1	0	1	3	4	1
2060	202	0	10-Dec-10	21	1	15	3	1	1	0	1	3	2	2
2061	202	0	11-Jan-10	3	1	15	3	1	1	0	1	3	1	2
2062	202	0	18-Aug-10	21	1	15	3	3	1	0	1	3	6	1
2063	202	0	12-Feb-10	20	1	15	3	3	1	0	1	3	3	2
2064	202	0	13-Dec-09	23	1	15	3	3	1	0	1	3	1	1
2065	202	0	19-Jun-15	11	1	15	3	1	1	0	3	3	2	1
2066	202	0	24-Oct-15	2	1	15	3	2	1	0	3	3	2	1
2067	202	0	29-Oct-14	23	1	15	3	3	1	0	3	3	5	1
2068	202	0	29-Sep-11	12	1	15	3	3	1	0	3	3	1	1
2069	202	0	12-May-17	12	1	15	3	2	1	0	3	3	2	1
2070	202	0	17-May-15	0	1	15	3	1	1	0	6	3	2	1
2071	202	0	8-Oct-12	17	1	15	3	1	1	0	6	3	2	2
2072	202	0	12-Oct-10	17	1	15	3	1	1	0	6	3	3	2
2073	202	0	4-Oct-13	22	2	15	3	1	1	0	4	3	4	1
2074	202	0	1-Jan-12	2	2	15	3	1	1	0	1	3	5	1
2075	202	0	19-Oct-11	15	2	15	3	3	1	0	1	3	5	1
2076	202	0	27-May-11	1	2	15	3	1	1	0	1	3	5	2
2077	202	0	28-Oct-11	23	2	15	3	3	1	0	1	3	5	1
2078	202	0	20-Aug-10	16	2	15	3	1	1	0	1	3	6	2
2079	202	0	29-Dec-10	20	2	15	3	1	1	0	1	3	1	2
2080	202	0	21-Jan-10	23	2	15	3	3	1	0	1	3	3	2
2081	202	0	29-Dec-10	20	2	15	3	3	1	0	1	3	1	1
2082	202	0	30-Oct-09	14	2	15	3	3	1	0	3	3	2	1
2083	101	1	11-Sep-15	5	1	15	3	1	1	0	1	3	4	4
2084	101	1	15-Apr-12	2	1	15	3	2	1	0	1	3	1	3
2085	201	1	22-Nov-14	8	1	15	3	3	1	0	1	3	5	1
2086	201	1	29-Dec-12	18	1	15	3	3	1	0	1	3	1	1
2087	201	1	1-Nov-14	2	1	15	3	3	1	0	1	3	1	1
2088	101	1	3-Jun-11	17	1	15	3	3	1	0	1	3	1	2
2089	101	1	15-Nov-11	14	1	15	3	1	1	0	1	3	2	1
2090	101	1	12-Nov-09	23	1	15	3	3	1	0	1	3	1	1
2091	201	1	25-Aug-11	17	1	15	3	2	1	0	1	3	1	1
2092	201	1	28-Jul-10	10	1	15	3	3	1	0	11	3	3	1
2093	201	1	24-Aug-12	11	1	15	3	2	1	0	3	3	3	2
2094	101	1	12-Nov-10	13	1	15	3	3	1	0	6	3	5	1
2095	101	1	1-Jul-11	18	2	15	3	1	1	0	3	3	2	1
2096	202	0	26-Nov-16	19	1	16	3	2	1	0	1	3	1	2
2097	202	0	3-Jul-17	2	1	16	3	2	1	0	1	3	6	1
2098	202	0	28-Mar-16	23	1	16	3	3	1	0	1	3	3	1
2099	202	0	2-Jul-14	21	1	16	3	3	1	0	1	3	1	2
2100	202	0	31-May-13	17	1	16	3	2	1	0	1	3	4	1
2101	202	0	26-Nov-13	23	1	16	3	1	1	0	1	3	3	1

2102	202	0	9-Apr-16	1	1	16	3	1	1	0	1	3	1	1
2103	202	0	11-Nov-12	21	1	16	3	3	1	0	1	3	1	2
2104	202	0	18-Aug-14	23	1	16	3	3	1	0	1	3	1	1
2105	202	0	8-Sep-15	21	1	16	3	3	1	0	1	3	4	2
2106	202	0	12-Apr-14	2	1	16	3	2	1	0	1	3	2	2
2107	202	0	14-Sep-14	19	1	16	3	3	1	0	1	3	2	2
2108	202	0	7-Sep-16	4	1	16	3	3	1	0	1	3	1	2
2109	202	0	26-Jan-16	0	1	16	3	2	1	0	1	3	3	2
2110	202	0	11-Oct-15	12	1	16	3	3	1	0	1	3	3	1
2111	202	0	11-Jul-15	1	1	16	3	1	1	0	1	3	5	1
2112	202	0	11-Oct-15	16	1	16	3	3	1	0	1	3	1	1
2113	202	0	18-Sep-14	19	1	16	3	3	1	0	1	3	5	1
2114	202	0	13-Mar-14	14	1	16	3	3	1	0	1	3	5	1
2115	202	0	16-Dec-14	10	1	16	3	2	1	0	1	3	5	1
2116	202	0	28-Sep-13	2	1	16	3	2	1	0	1	3	1	1
2117	202	0	23-Sep-13	11	1	16	3	1	1	0	1	3	4	1
2118	202	0	9-Dec-13	21	1	16	3	3	1	0	1	3	3	2
2119	202	0	19-Feb-13	4	1	16	3	2	1	0	1	3	1	1
2120	202	0	7-May-12	11	1	16	3	2	1	0	1	3	6	2
2121	202	0	15-Aug-12	0	1	16	3	1	1	0	1	3	1	1
2122	202	0	25-Feb-12	18	1	16	3	3	1	0	1	3	2	2
2123	202	0	17-Feb-12	21	1	16	3	2	1	0	1	3	3	2
2124	202	0	8-Nov-12	21	1	16	3	3	1	0	3	3	1	2
2125	202	0	10-Jun-14	12	1	16	3	3	1	0	6	3	2	1
2126	202	0	3-Mar-12	18	1	16	3	1	1	0	6	3	2	2
2127	202	0	25-May-08	23	1	16	3	2	1	0	10	3	3	2
2128	202	0	15-Jun-10	5	1	16	3	1	1	0	13	3	2	2
2129	202	0	17-Nov-11	23	1	16	3	1	1	0	1	3	5	2
2130	202	0	23-Mar-11	19	1	16	3	1	1	0	1	3	6	2
2131	202	0	11-Dec-11	8	1	16	3	3	1	0	1	3	3	2
2132	202	0	10-May-11	0	1	16	3	1	1	0	1	3	5	2
2133	202	0	23-Jan-11	17	1	16	3	1	1	0	1	3	6	2
2134	202	0	17-Dec-11	20	1	16	3	3	1	0	1	3	5	2
2135	202	0	26-Jul-11	15	1	16	3	3	1	0	1	3	6	1
2136	202	0	14-Apr-11	22	1	16	3	1	1	0	1	3	1	1
2137	202	0	25-Sep-11	0	1	16	3	1	1	0	1	3	6	2
2138	202	0	4-Jan-11	18	1	16	3	1	1	0	1	3	1	1
2139	202	0	8-Dec-11	12	1	16	3	1	1	0	1	3	2	1
2140	202	0	27-Oct-10	23	1	16	3	1	1	0	1	3	6	2
2141	202	0	19-Dec-10	1	1	16	3	1	1	0	1	3	6	2
2142	202	0	24-Jul-10	22	1	16	3	1	1	0	1	3	6	1
2143	202	0	19-Jun-09	13	1	16	3	3	1	0	1	3	5	1
2144	202	0	29-Oct-09	13	1	16	3	1	1	0	1	3	6	1
2145	202	0	22-Sep-09	1	1	16	3	3	1	0	1	3	1	2
2146	202	0	6-Jun-09	23	1	16	3	3	1	0	1	3	3	2
2147	202	0	26-Feb-08	1	1	16	3	1	1	0	1	3	1	1
2148	202	0	31-Aug-08	13	1	16	3	3	1	0	1	3	2	1
2149	202	0	28-Jun-08	9	1	16	3	3	1	0	1	3	2	1
2150	202	0	23-Jun-08	7	1	16	3	1	1	0	1	3	2	1
2151	202	0	23-Jul-08	17	1	16	3	1	1	0	1	3	2	1
2152	202	0	8-Dec-06	18	1	16	3	1	1	0	1	3	3	1
2153	202	0	10-Jan-06	10	1	16	3	3	1	0	1	3	3	2
2154	202	0	27-Aug-06	19	1	16	3	3	1	0	1	3	1	1
2155	202	0	27-Jul-08	7	1	16	3	3	1	0	1	3	2	2
2156	202	0	22-Jul-15	20	1	16	3	3	1	0	4	3	3	1
2157	202	0	12-Jun-15	12	1	16	3	1	1	0	4	3	4	1
2158	202	0	24-Oct-15	3	1	16	3	3	1	0	4	3	1	1
2159	202	0	24-Oct-15	0	1	16	3	3	1	0	4	3	1	1
2160	202	0	22-Aug-16	18	1	16	3	3	1	0	3	3	5	1
2161	202	0	24-Dec-13	20	1	16	3	2	1	0	3	3	1	2
2162	202	0	20-Sep-11	9	1	16	3	1	1	0	3	3	1	1
2163	202	0	12-May-10	11	1	16	3	1	1	0	3	3	1	1
2164	202	0	19-Mar-09	9	1	16	3	2	1	0	3	3	2	2
2165	202	0	20-Aug-08	10	1	16	3	1	1	0	3	3	2	1
2166	202	0	29-Mar-17	10	1	16	3	3	1	0	3	3	2	2
2167	202	0	6-Oct-16	15	1	16	3	3	1	0	6	3	3	1
2168	202	0	25-Jun-09	0	1	16	3	1	1	0	6	3	3	2
2169	202	0	7-Jan-16	3	2	16	3	3	1	0	1	3	3	2
2170	202	0	7-Jan-16	3	2	16	3	1	1	0	1	3	5	2
2171	202	0	17-Jul-15	8	2	16	3	3	1	0	1	3	1	1
2172	202	0	1-Nov-14	23	2	16	3	3	1	0	1	3	1	2
2173	202	0	28-May-13	4	2	16	3	3	1	0	1	3	5	2
2174	202	0	23-Sep-12	17	2	16	3	3	1	0	1	3	6	1
2175	202	0	16-Jun-12	20	2	16	3	3	1	0	1	3	6	2
2176	202	0	2-Jul-12	1	2	16	3	1	1	0	1	3	1	1
2177	202	0	30-Jul-12	16	2	16	3	3	1	0	1	3	3	1
2178	202	0	15-Mar-12	19	2	16	3	3	1	0	1	3	1	2
2179	202	0	21-Nov-12	3	2	16	3	3	1	0	1	3	6	1
2180	202	0	28-May-11	22	2	16	3	3	1	0	1	3	5	2
2181	202	0	19-Oct-11	15	2	16	3	1	1	0	1	3	1	2
2182	202	0	27-Mar-10	18	2	16	3	3	1	0	1	3	6	2
2183	202	0	13-Nov-10	9	2	16	3	1	1	0	1	3	1	2
2184	202	0	19-Oct-10	13	2	16	3	3	1	0	1	3	1	1
2185	202	0	4-Nov-09	8	2	16	3	3	1	0	1	3	1	2
2186	202	0	18-May-07	19	2	16	3	3	1	0	1	3	6	1
2187	202	0	2-May-07	9	2	16	3	1	1	0	1	3	3	1
2188	202	0	4-Dec-06	1	2	16	3	3	1	0	1	3	2	1
2189	202	0	28-Aug-06	1	2	16	3	3	1	0	1	3	2	1
2190	202	0	20-Apr-06	12	2	16	3	2	1	0	1	3	1	1
2191	202	0	20-Jan-06	13	2	16	3	3	1	0	1	3	3	2
2192	202	0	4-Aug-10	23	2	16	3	1	1	0	1	3	3	2
2193	202	0	7-Nov-08	20	2	16	3	2	1	0	1	3	1	2
2194	202	0	26-Jun-07	8	2	16	3	3	1	0	4	3	3	1
2195	202	0	6-Jan-17	22	2	16	3	3	1	0	4	3	3	1
2196	202	0	17-Aug-16	4	2	16	3	3	1	0	3	3	3	2
2197	202	0	10-Dec-08	1	2	16	3	2	1	0	3	3	6	2
2198	202	0	14-Dec-08	22	2	16	3	3	1	0	3	3	2	1
2199	202	0	30-Nov-17	0	2	16	3	2	1	0	3	3	1	1
2200	201	1	10-Jun-14	16	1	16	3	3	1	0	1	3	5	1
2201	201	1	18-Jun-16	4	1	16	3	3	1	0	1	3	1	2
2202	201	1	3-Mar-12	9	1	16	3	2	1	0	1	3	5	1
2203	201	1	22-Apr-12	13	1	16	3	2	1	0	1	3	5	1
2204	201	1	15-Apr-14	13	1	16	3	1	1	0	1	3	3	1
2205	201	1	3-Jun-13	10	1	16	3	3	1	0	1	3	2	1
2206	201	1	9-Apr-17	4	1	16	3	3	1	0	1	3	5	2
2207	101	1	1-Jan-13	17	1	16	3	2	1	0	1	3	5	1
2208	201	1	19-Sep-12	22	1	16	3	2	1	0	1	3	5	1
2209	101	1	14-Aug-12	12	1	16	3	2	1	0	1	3	2	4
2210	101	1	29-Nov-12	18	1	16	3	3	1	0	1	3	3	1
2211	101	1	14-Sep-13	0	1	16	3	2	1	0	1	3	1	2
2212	101	1	14-Jan-12	11	1	16	3	3	1	0	1	3	4	1
2213	101	1	23-Apr-17	21	1	16	3	3	1	0	1	3	1	1
2214	201	1	22-Apr-13	12	1	16	3	3	1	0	1	3	6	2
2215	201	1	29-Jun-13	21	1	16	3	3	1	0	1	3	5	1
2216	201	1	20-Apr-13	19	1	16	3	3	1	0	1	3	5	1
2217	201	1	23-Jun-12	13	1	16	3	3	1	0	1	3	4	2
2218	101	1	30-Aug-11	12	1	16	3	3	1	0	1	3	1	1
2219	201	1	29-Mar-11	18	1	16	3	3	1	0	1	3	1	2
2220	201	1	14-May-11	2	1	16	3	4	1	0	1	3	2	1
2221	201	1	14-Mar-10	9	1	16	3	3	1	0	1	3	3	2
2222	101	1	11-Jun-10	18	1	16	3	3	1	0	1	3	2	2
2223	101	1	14-May-10	22	1	16	3	3	1	0	1	3	5	2
2224	101	1	3-Aug-09	10	1	16	3	3	1	0	1	3	1	2
2225	201	1	1-Mar-09	11	1	16	3	3	1	0	1	3	5	1

2226	101	1	29-Jan-09	14	1	16	3	3	1	0	1	3	1	2
2227	201	1	23-Jun-09	13	1	16	3	3	1	0	1	3	3	1
2228	101	1	9-Nov-08	9	1	16	3	3	1	0	1	3	5	2
2229	201	1	7-Aug-08	13	1	16	3	3	1	0	1	3	4	1
2230	201	1	16-Jun-07	18	1	16	3	3	1	0	1	3	6	1
2231	201	1	15-Jun-07	21	1	16	3	3	1	0	1	3	6	1
2232	101	1	5-Aug-13	19	1	16	3	1	1	0	1	3	3	1
2233	101	1	10-Apr-12	2	1	16	3	1	1	0	1	3	5	1
2234	201	1	23-Dec-11	14	1	16	3	2	1	0	1	3	3	1
2235	101	1	5-May-11	2	1	16	3	3	1	0	1	3	1	2
2236	201	1	22-Aug-10	0	1	16	3	1	1	0	1	3	2	1
2237	101	1	22-Jun-10	20	1	16	3	2	1	0	1	3	2	2
2238	201	1	16-Nov-09	22	1	16	3	1	1	0	1	3	1	1
2239	201	1	14-Aug-08	12	1	16	3	3	1	0	1	3	5	1
2240	201	1	9-Aug-06	16	1	16	3	2	1	0	1	3	2	1
2241	101	1	11-Sep-12	10	1	16	3	3	1	0	4	3	4	1
2242	101	1	4-Oct-13	2	1	16	3	2	1	0	3	3	1	1
2243	201	1	18-Dec-10	19	1	16	3	3	1	0	3	3	2	1
2244	201	1	22-Mar-08	21	1	16	3	3	1	0	3	3	3	1
2245	201	1	7-Mar-12	4	2	16	3	2	1	0	1	3	2	1
2246	201	1	28-Feb-16	20	2	16	3	3	1	0	1	3	3	1
2247	201	1	10-Oct-15	22	2	16	3	3	1	0	1	3	1	1
2248	201	1	19-Mar-13	14	2	16	3	3	1	0	1	3	3	1
2249	201	1	6-Jul-14	4	2	16	3	3	1	0	1	3	5	1
2250	101	1	26-Sep-14	22	2	16	3	2	1	0	1	3	3	1
2251	101	1	12-Mar-12	19	2	16	3	2	1	0	1	3	1	2
2252	101	1	15-Jul-13	19	2	16	3	2	1	0	1	3	5	1
2253	101	1	26-Dec-13	9	2	16	3	3	1	0	1	3	3	2
2254	101	1	12-Sep-12	23	2	16	3	2	1	0	1	3	1	2
2255	101	1	22-Jun-12	20	2	16	3	3	1	0	1	3	1	2
2256	101	1	21-Mar-11	1	2	16	3	3	1	0	1	3	4	1
2257	101	1	3-Aug-09	23	2	16	3	1	1	0	1	3	2	1
2258	201	1	10-Nov-08	19	2	16	3	3	1	0	1	3	5	1
2259	201	1	27-Nov-07	2	2	16	3	3	1	0	1	3	7	1
2260	101	1	1-Jan-07	15	2	16	3	3	1	0	1	3	3	1
2261	201	1	13-Aug-07	12	2	16	3	3	1	0	1	3	2	1
2262	201	1	6-Sep-06	15	2	16	3	3	1	0	1	3	1	1
2263	201	1	14-Jan-08	7	2	16	3	3	1	0	1	3	6	2
2264	201	1	23-Aug-07	8	2	16	3	2	1	0	1	3	6	1
2265	201	1	21-Oct-07	19	2	16	3	3	1	0	1	3	2	1
2266	101	1	3-May-12	22	2	16	3	3	1	0	3	3	2	1
2267	201	1	15-Feb-09	2	2	16	3	3	1	0	3	3	3	4
2268	201	1	1-May-09	3	2	16	3	3	1	0	3	3	1	1
2269	101	1	16-Nov-07	13	2	16	3	3	1	0	3	3	1	1
2270	202	0	18-Jul-15	7	1	17	3	3	1	0	1	3	5	1
2271	202	0	7-Jan-16	4	1	17	3	3	1	0	1	3	4	2
2272	202	0	10-Oct-14	8	1	17	3	3	1	0	1	3	5	1
2273	202	0	4-Feb-13	19	1	17	3	2	1	0	1	3	2	2
2274	202	0	23-Sep-12	9	1	17	3	2	1	0	1	3	2	2
2275	202	0	30-Jan-16	2	1	17	3	3	1	0	1	3	3	1
2276	202	0	17-Aug-15	0	1	17	3	1	1	0	1	3	4	1
2277	202	0	12-Mar-14	13	1	17	3	2	1	0	1	3	3	2
2278	202	0	13-May-15	16	1	17	3	1	1	0	1	3	3	1
2279	202	0	9-Oct-14	18	1	17	3	2	1	0	1	3	4	2
2280	202	0	17-Jul-14	20	1	17	3	1	1	0	1	3	4	1
2281	202	0	1-Aug-17	23	1	17	3	2	1	0	1	3	5	1
2282	202	0	24-Sep-16	2	1	17	3	2	1	0	1	3	4	2
2283	202	0	29-Jun-16	23	1	17	3	3	1	0	1	3	5	1
2284	202	0	10-May-16	9	1	17	3	2	1	0	1	3	3	1
2285	202	0	14-Aug-13	21	1	17	3	2	1	0	1	3	5	1
2286	202	0	18-Sep-14	15	1	17	3	1	1	0	1	3	1	2
2287	202	0	4-Mar-14	13	1	17	3	2	1	0	1	3	1	2
2288	202	0	19-Aug-13	2	1	17	3	1	1	0	1	3	1	1
2289	202	0	17-Aug-12	20	1	17	3	3	1	0	1	3	3	1
2290	202	0	26-Aug-15	20	1	17	3	1	1	0	1	3	1	1
2291	202	0	8-Jun-12	15	1	17	3	1	1	0	1	3	3	2
2292	202	0	8-Jun-12	15	1	17	3	1	1	0	1	3	2	2
2293	202	0	14-May-16	9	1	17	3	3	1	0	1	3	1	2
2294	202	0	15-May-16	22	1	17	3	3	1	0	1	3	1	2
2295	202	0	17-Jan-16	21	1	17	3	2	1	0	1	3	3	1
2296	202	0	6-Sep-15	21	1	17	3	1	1	0	1	3	1	2
2297	202	0	17-Jul-15	22	1	17	3	1	1	0	1	3	1	1
2298	202	0	11-Sep-15	19	1	17	3	3	1	0	1	3	1	2
2299	202	0	24-Sep-15	3	1	17	3	3	1	0	1	3	5	1
2300	202	0	6-Nov-15	18	1	17	3	2	1	0	1	3	2	2
2301	202	0	20-Sep-15	9	1	17	3	1	1	0	1	3	5	1
2302	202	0	4-Nov-15	10	1	17	3	3	1	0	1	3	2	1
2303	202	0	11-Aug-15	1	1	17	3	3	1	0	1	3	1	2
2304	202	0	31-May-14	1	1	17	3	3	1	0	1	3	1	1
2305	202	0	26-May-14	3	1	17	3	3	1	0	1	3	2	1
2306	202	0	15-May-14	13	1	17	3	3	1	0	1	3	3	1
2307	202	0	10-Aug-14	21	1	17	3	2	1	0	1	3	5	2
2308	202	0	9-Jul-14	3	1	17	3	3	1	0	1	3	3	1
2309	202	0	21-May-14	23	1	17	3	2	1	0	1	3	5	1
2310	202	0	10-Aug-14	19	1	17	3	1	1	0	1	3	5	1
2311	202	0	7-Jun-14	4	1	17	3	1	1	0	1	3	3	2
2312	202	0	16-Apr-14	23	1	17	3	1	1	0	1	3	4	1
2313	202	0	16-Apr-14	16	1	17	3	3	1	0	1	3	3	1
2314	202	0	4-Jul-14	23	1	17	3	1	1	0	1	3	3	1
2315	202	0	30-Jun-14	13	1	17	3	2	1	0	1	3	1	2
2316	202	0	4-Jul-14	23	1	17	3	1	1	0	1	3	4	1
2317	202	0	27-Jan-14	20	1	17	3	1	1	0	1	3	1	2
2318	202	0	3-Jun-14	22	1	17	3	3	1	0	1	3	4	1
2319	202	0	8-Jun-14	3	1	17	3	3	1	0	1	3	1	2
2320	202	0	20-Mar-14	20	1	17	3	3	1	0	1	3	5	2
2321	202	0	19-Jul-13	13	1	17	3	3	1	0	1	3	5	2
2322	202	0	4-Aug-13	3	1	17	3	3	1	0	1	3	4	1
2323	202	0	16-Nov-13	2	1	17	3	2	1	0	1	3	5	1
2324	202	0	10-Nov-13	22	1	17	3	1	1	0	1	3	5	1
2325	202	0	24-Apr-13	16	1	17	3	2	1	0	1	3	3	1
2326	202	0	1-Oct-13	19	1	17	3	1	1	0	1	3	5	1
2327	202	0	18-Sep-13	12	1	17	3	2	1	0	1	3	5	2
2328	202	0	7-Apr-13	14	1	17	3	2	1	0	1	3	3	1
2329	202	0	24-Nov-13	21	1	17	3	1	1	0	1	3	3	1
2330	202	0	17-Oct-13	21	1	17	3	2	1	0	1	3	5	1
2331	202	0	25-Sep-13	5	1	17	3	3	1	0	1	3	5	1
2332	202	0	1-Aug-13	1	1	17	3	3	1	0	1	3	5	1
2333	202	0	17-May-13	2	1	17	3	3	1	0	1	3	3	1
2334	202	0	21-Oct-12	3	1	17	3	1	1	0	1	3	6	2
2335	202	0	20-Jul-12	14	1	17	3	1	1	0	1	3	1	2
2336	202	0	5-Jul-12	0	1	17	3	3	1	0	1	3	6	2
2337	202	0	22-Jul-12	23	1	17	3	1	1	0	1	3	6	2
2338	202	0	26-Dec-12	13	1	17	3	3	1	0	1	3	1	2
2339	202	0	17-Dec-12	4	1	17	3	3	1	0	1	3	1	1
2340	202	0	31-Jul-12	1	1	17	3	1	1	0	1	3	2	2
2341	202	0	4-Jul-12	18	1	17	3	1	1	0	1	3	2	2
2342	202	0	29-Apr-12	22	1	17	3	1	1	0	1	3	3	2
2343	202	0	14-Jul-12	12	1	17	3	1	1	0	1	3	5	1
2344	202	0	31-Mar-12	21	1	17	3	1	1	0	1	3	5	2
2345	202	0	14-Aug-12	1	1	17	3	1	1	0	1	3	1	2
2346	202	0	23-Mar-12	12	1	17	3	3	1	0	1	3	4	1
2347	202	0	20-Sep-12	23	1	17	3	1	1	0	1	3	3	1
2348	202	0	3-Jan-12	0	1	17	3	1	1	0	1	3	5	2
2349	202	0	12-Oct-12	22	1	17	3	1	1	0	1	3	5	1

2350	202	0	8-Jun-12	0	1	17	3	3	1	0	1	3	2	1
2351	202	0	19-Jun-12	20	1	17	3	1	1	0	1	3	5	1
2352	202	0	31-Dec-11	23	1	17	3	3	1	0	1	3	1	2
2353	202	0	17-Sep-17	4	1	17	3	3	1	0	1	3	1	2
2354	202	0	14-Jul-17	23	1	17	3	1	1	0	1	3	4	2
2355	202	0	3-Oct-17	22	1	17	3	2	1	0	1	3	1	1
2356	202	0	3-Oct-17	22	1	17	3	2	1	0	1	3	6	2
2357	202	0	29-Jun-17	21	1	17	3	3	1	0	1	3	3	1
2358	202	0	27-Jan-17	18	1	17	3	3	1	0	1	3	4	2
2359	202	0	9-Sep-17	1	1	17	3	2	1	0	1	3	1	1
2360	202	0	3-Oct-17	23	1	17	3	3	1	0	1	3	1	2
2361	202	0	15-Mar-17	7	1	17	3	2	1	0	1	3	5	1
2362	202	0	29-Dec-17	14	1	17	3	1	1	0	1	3	1	1
2363	202	0	17-May-17	20	1	17	3	1	1	0	1	3	1	2
2364	202	0	20-Aug-17	3	1	17	3	3	1	0	1	3	3	1
2365	202	0	7-Jan-17	23	1	17	3	3	1	0	1	3	1	2
2366	202	0	7-Jan-17	16	1	17	3	3	1	0	1	3	5	1
2367	202	0	12-Mar-16	22	1	17	3	2	1	0	3	3	5	2
2368	202	0	12-Mar-16	22	1	17	3	2	1	0	3	3	1	1
2369	202	0	27-Jul-15	1	1	17	3	3	1	0	3	3	5	1
2370	202	0	25-Dec-13	20	1	17	3	3	1	0	3	3	3	1
2371	202	0	9-Jul-15	14	1	17	3	3	1	0	4	3	3	2
2372	202	0	6-Jun-12	19	1	17	3	1	1	0	6	3	3	1
2373	202	0	1-Sep-16	16	1	17	3	2	1	0	6	3	3	1
2374	202	0	9-Jan-07	21	1	17	3	3	1	0	7	3	2	1
2375	202	0	26-Mar-13	8	1	17	3	3	1	0	1	3	3	1
2376	202	0	24-Jun-11	14	1	17	3	3	1	0	1	3	5	2
2377	202	0	19-Nov-11	21	1	17	3	3	1	0	1	3	5	2
2378	202	0	26-Nov-11	20	1	17	3	3	1	0	1	3	4	2
2379	202	0	10-May-11	0	1	17	3	1	1	0	1	3	5	1
2380	202	0	25-May-11	16	1	17	3	1	1	0	1	3	5	2
2381	202	0	11-Dec-11	18	1	17	3	1	1	0	1	3	5	1
2382	202	0	11-Jun-11	0	1	17	3	1	1	0	1	3	1	1
2383	202	0	16-Aug-11	13	1	17	3	1	1	0	1	3	1	1
2384	202	0	5-Feb-11	21	1	17	3	1	1	0	1	3	1	1
2385	202	0	5-Jun-11	21	1	17	3	1	1	0	1	3	5	2
2386	202	0	6-Nov-11	0	1	17	3	3	1	0	1	3	3	1
2387	202	0	28-Oct-11	22	1	17	3	1	1	0	1	3	5	1
2388	202	0	11-Sep-11	23	1	17	3	3	1	0	1	3	1	1
2389	202	0	21-Jan-11	19	1	17	3	1	1	0	1	3	1	1
2390	202	0	10-Dec-11	18	1	17	3	1	1	0	1	3	3	1
2391	202	0	22-Oct-11	1	1	17	3	1	1	0	1	3	1	1
2392	202	0	11-Oct-10	23	1	17	3	1	1	0	1	3	6	2
2393	202	0	5-Jan-10	17	1	17	3	1	1	0	1	3	3	2
2394	202	0	3-Oct-10	18	1	17	3	1	1	0	1	3	6	2
2395	202	0	25-Oct-10	2	1	17	3	1	1	0	1	3	6	1
2396	202	0	17-Nov-10	10	1	17	3	3	1	0	1	3	1	1
2397	202	0	19-Jun-10	1	1	17	3	1	1	0	1	3	3	1
2398	202	0	14-Aug-10	13	1	17	3	1	1	0	1	3	3	1
2399	202	0	2-May-10	19	1	17	3	1	1	0	1	3	1	1
2400	202	0	10-Jan-10	8	1	17	3	1	1	0	1	3	3	2
2401	202	0	9-Jan-10	15	1	17	3	1	1	0	1	3	1	1
2402	202	0	28-Jul-10	20	1	17	3	3	1	0	1	3	6	2
2403	202	0	21-Jun-10	16	1	17	3	1	1	0	1	3	1	1
2404	202	0	1-Aug-10	20	1	17	3	3	1	0	1	3	6	1
2405	202	0	9-Oct-10	8	1	17	3	1	1	0	1	3	3	1
2406	202	0	19-Jan-10	2	1	17	3	3	1	0	1	3	2	2
2407	202	0	14-Jul-10	21	1	17	3	1	1	0	1	3	2	1
2408	202	0	27-Oct-10	20	1	17	3	3	1	0	1	3	6	2
2409	202	0	16-Apr-10	11	1	17	3	2	1	0	1	3	1	2
2410	202	0	2-Jul-09	1	1	17	3	3	1	0	1	3	4	2
2411	202	0	29-Mar-09	6	1	17	3	1	1	0	1	3	1	2
2412	202	0	23-Oct-09	23	1	17	3	3	1	0	1	3	6	1
2413	202	0	8-Jul-09	20	1	17	3	1	1	0	1	3	1	2
2414	202	0	21-Jun-09	23	1	17	3	1	1	0	1	3	6	1
2415	202	0	23-Apr-09	18	1	17	3	3	1	0	1	3	2	1
2416	202	0	23-Apr-09	20	1	17	3	1	1	0	1	3	3	1
2417	202	0	21-Dec-08	3	1	17	3	1	1	0	1	3	3	2
2418	202	0	6-Jun-08	22	1	17	3	2	1	0	1	3	1	1
2419	202	0	1-Aug-08	19	1	17	3	1	1	0	1	3	6	1
2420	202	0	25-Mar-08	19	1	17	3	3	1	0	1	3	3	2
2421	202	0	18-Jun-07	11	1	17	3	2	1	0	1	3	6	2
2422	202	0	10-Oct-07	21	1	17	3	2	1	0	1	3	6	1
2423	202	0	3-Jul-07	12	1	17	3	1	1	0	1	3	1	1
2424	202	0	29-Sep-07	17	1	17	3	3	1	0	1	3	3	1
2425	202	0	23-Feb-07	0	1	17	3	1	1	0	1	3	1	2
2426	202	0	24-Jan-07	22	1	17	3	3	1	0	1	3	6	2
2427	202	0	30-Aug-07	15	1	17	3	3	1	0	1	3	2	2
2428	202	0	13-Dec-07	4	1	17	3	3	1	0	1	3	1	1
2429	202	0	19-Mar-07	13	1	17	3	1	1	0	1	3	6	1
2430	202	0	20-Dec-07	17	1	17	3	1	1	0	1	3	1	1
2431	202	0	21-Nov-07	19	1	17	3	1	1	0	1	3	6	2
2432	202	0	24-Apr-06	7	1	17	3	2	1	0	1	3	1	1
2433	202	0	12-May-06	21	1	17	3	1	1	0	1	3	3	1
2434	202	0	10-Mar-06	0	1	17	3	1	1	0	1	3	6	1
2435	202	0	18-May-06	9	1	17	3	3	1	0	1	3	1	1
2436	202	0	25-Jun-06	9	1	17	3	1	1	0	1	3	1	2
2437	202	0	16-Dec-06	2	1	17	3	1	1	0	1	3	2	1
2438	202	0	15-Dec-06	22	1	17	3	2	1	0	1	3	1	2
2439	202	0	31-Mar-06	10	1	17	3	3	1	0	1	3	2	1
2440	202	0	21-Aug-06	12	1	17	3	1	1	0	1	3	2	1
2441	202	0	30-Jul-10	13	1	17	3	1	1	0	1	3	6	2
2442	202	0	13-Nov-10	12	1	17	3	1	1	0	1	3	6	2
2443	202	0	12-Nov-10	18	1	17	3	1	1	0	1	3	6	2
2444	202	0	16-Aug-09	1	1	17	3	1	1	0	1	3	3	2
2445	202	0	8-Sep-07	11	1	17	3	1	1	0	1	3	2	1
2446	202	0	28-Oct-16	19	1	17	3	3	1	0	4	3	1	1
2447	202	0	16-Jul-14	15	1	17	3	2	1	0	4	3	1	1
2448	202	0	31-Jul-10	8	1	17	3	1	1	0	4	3	3	2
2449	202	0	4-Apr-17	1	1	17	3	3	1	0	4	3	2	1
2450	202	0	25-Sep-07	22	1	17	3	1	1	0	11	3	3	2
2451	202	0	2-Aug-16	17	1	17	3	3	1	0	3	3	3	2
2452	202	0	17-Jul-16	16	1	17	3	1	1	0	3	3	2	1
2453	202	0	24-Nov-16	17	1	17	3	1	1	0	3	3	3	1
2454	202	0	18-Jul-15	10	1	17	3	1	1	0	3	3	1	1
2455	202	0	29-Oct-15	2	1	17	3	3	1	0	3	3	1	1
2456	202	0	7-Jul-14	11	1	17	3	3	1	0	3	3	2	1
2457	202	0	10-Dec-14	8	1	17	3	1	1	0	3	3	5	2
2458	202	0	15-Mar-14	3	1	17	3	2	1	0	3	3	5	1
2459	202	0	23-Jul-13	15	1	17	3	3	1	0	3	3	2	1
2460	202	0	11-Oct-13	13	1	17	3	3	1	0	3	3	5	2
2461	202	0	17-Nov-13	22	1	17	3	3	1	0	3	3	3	1
2462	202	0	3-Nov-12	18	1	17	3	3	1	0	3	3	2	2
2463	202	0	31-Oct-11	7	1	17	3	1	1	0	3	3	1	2
2464	202	0	8-May-11	22	1	17	3	3	1	0	3	3	2	2
2465	202	0	18-Jun-11	19	1	17	3	1	1	0	3	3	1	1
2466	202	0	25-Jun-10	17	1	17	3	3	1	0	3	3	6	1
2467	202	0	11-Jun-10	21	1	17	3	1	1	0	3	3	6	1
2468	202	0	6-Jan-10	18	1	17	3	1	1	0	3	3	1	1
2469	202	0	16-Feb-10	10	1	17	3	1	1	0	3	3	1	2
2470	202	0	9-Dec-08	15	1	17	3	1	1	0	3	3	1	2
2471	202	0	26-Jan-07	1	1	17	3	3	1	0	3	3	1	1
2472	202	0	11-Sep-07	13	1	17	3	1	1	0	3	3	1	1
2473	202	0	4-Jun-17	20	1	17	3	3	1	0	3	3	6	1

2474	202	0	31-Jul-17	16	1	17	3	1	1	0	3	3	3	1
2475	202	0	13-Aug-17	20	1	17	3	3	1	0	3	3	2	1
2476	202	0	6-Aug-11	13	1	17	3	1	1	0	6	3	6	2
2477	202	0	18-Jul-11	8	1	17	3	2	1	0	6	3	2	1
2478	202	0	11-Mar-08	1	1	17	3	3	1	0	6	3	7	2
2479	202	0	20-Jun-07	20	1	17	3	3	1	0	6	3	3	2
2480	202	0	16-Feb-07	0	1	17	3	3	1	0	13	3	1	1
2481	202	0	4-Jun-17	18	2	17	3	3	1	0	1	3	3	1
2482	202	0	26-Oct-15	13	2	17	3	3	1	0	1	3	2	2
2483	202	0	19-Sep-14	22	2	17	3	3	1	0	1	3	5	1
2484	202	0	23-Jun-14	8	2	17	3	3	1	0	1	3	5	1
2485	202	0	21-Nov-15	14	2	17	3	2	1	0	1	3	1	1
2486	202	0	18-Jul-14	20	2	17	3	3	1	0	1	3	4	2
2487	202	0	18-Nov-14	14	2	17	3	3	1	0	1	3	4	1
2488	202	0	1-Dec-13	2	2	17	3	1	1	0	1	3	1	1
2489	202	0	26-Nov-13	21	2	17	3	1	1	0	1	3	3	2
2490	202	0	25-Aug-16	16	2	17	3	1	1	0	1	3	4	1
2491	202	0	24-Jul-16	15	2	17	3	1	1	0	1	3	3	1
2492	202	0	29-May-15	20	2	17	3	3	1	0	1	3	1	2
2493	202	0	3-Oct-15	0	2	17	3	2	1	0	1	3	3	2
2494	202	0	8-Apr-14	22	2	17	3	3	1	0	1	3	1	2
2495	202	0	7-Sep-14	5	2	17	3	3	1	0	1	3	5	1
2496	202	0	5-Jul-14	13	2	17	3	3	1	0	1	3	3	1
2497	202	0	21-Oct-14	23	2	17	3	3	1	0	1	3	3	1
2498	202	0	26-Mar-14	16	2	17	3	3	1	0	1	3	5	1
2499	202	0	11-Oct-14	20	2	17	3	3	1	0	1	3	3	1
2500	202	0	22-Jan-13	1	2	17	3	2	1	0	1	3	5	2
2501	202	0	15-Dec-13	8	2	17	3	2	1	0	1	3	1	2
2502	202	0	31-Oct-13	17	2	17	3	3	1	0	1	3	1	1
2503	202	0	18-Oct-13	19	2	17	3	1	1	0	1	3	1	1
2504	202	0	27-Jun-13	13	2	17	3	3	1	0	1	3	1	2
2505	202	0	18-Feb-13	18	2	17	3	1	1	0	1	3	3	2
2506	202	0	13-Jun-13	16	2	17	3	1	1	0	1	3	2	1
2507	202	0	28-Nov-13	18	2	17	3	3	1	0	1	3	3	1
2508	202	0	25-Apr-13	15	2	17	3	1	1	0	1	3	4	2
2509	202	0	6-Jul-13	0	2	17	3	3	1	0	1	3	1	2
2510	202	0	18-May-13	2	2	17	3	1	1	0	1	3	1	2
2511	202	0	4-Jun-12	16	2	17	3	3	1	0	1	3	1	2
2512	202	0	8-Dec-12	19	2	17	3	3	1	0	1	3	5	1
2513	202	0	4-May-12	8	2	17	3	3	1	0	1	3	1	1
2514	202	0	31-May-12	4	2	17	3	3	1	0	1	3	5	1
2515	202	0	31-May-12	4	2	17	3	3	1	0	1	3	5	1
2516	202	0	7-Jul-12	19	2	17	3	1	1	0	1	3	5	1
2517	202	0	12-Dec-12	22	2	17	3	3	1	0	1	3	5	1
2518	202	0	23-Apr-17	13	2	17	3	2	1	0	1	3	1	1
2519	202	0	18-Dec-17	0	2	17	3	2	1	0	1	3	1	1
2520	202	0	24-Jun-17	0	2	17	3	2	1	0	1	3	5	1
2521	202	0	20-Aug-17	16	2	17	3	3	1	0	1	3	3	1
2522	202	0	31-Dec-16	12	2	17	3	1	1	0	1	3	3	1
2523	202	0	15-Nov-17	0	2	17	3	3	1	0	1	3	1	2
2524	202	0	2-Sep-13	0	2	17	3	3	1	0	3	3	1	2
2525	202	0	1-May-12	14	2	17	3	3	1	0	1	3	6	2
2526	202	0	7-Jan-12	22	2	17	3	1	1	0	1	3	5	1
2527	202	0	2-Sep-12	19	2	17	3	3	1	0	1	3	1	2
2528	202	0	19-Oct-11	17	2	17	3	1	1	0	1	3	2	1
2529	202	0	19-Mar-11	4	2	17	3	1	1	0	1	3	6	2
2530	202	0	15-Nov-11	11	2	17	3	3	1	0	1	3	5	2
2531	202	0	14-Mar-11	4	2	17	3	1	1	0	1	3	1	1
2532	202	0	4-Dec-11	1	2	17	3	1	1	0	1	3	1	1
2533	202	0	4-Apr-11	17	2	17	3	3	1	0	1	3	6	1
2534	202	0	28-Oct-11	23	2	17	3	3	1	0	1	3	1	1
2535	202	0	21-Sep-11	0	2	17	3	1	1	0	1	3	3	2
2536	202	0	26-Jan-11	20	2	17	3	3	1	0	1	3	6	2
2537	202	0	3-Oct-11	23	2	17	3	3	1	0	1	3	5	2
2538	202	0	22-Dec-10	2	2	17	3	1	1	0	1	3	6	2
2539	202	0	3-Oct-10	19	2	17	3	1	1	0	1	3	6	2
2540	202	0	8-Jul-10	4	2	17	3	1	1	0	1	3	1	2
2541	202	0	3-May-10	1	2	17	3	1	1	0	1	3	6	1
2542	202	0	25-Sep-10	23	2	17	3	1	1	0	1	3	6	1
2543	202	0	3-May-10	1	2	17	3	1	1	0	1	3	6	2
2544	202	0	9-Feb-10	1	2	17	3	3	1	0	1	3	6	2
2545	202	0	7-May-10	23	2	17	3	3	1	0	1	3	3	2
2546	202	0	7-May-10	23	2	17	3	1	1	0	1	3	3	2
2547	202	0	27-Jul-10	19	2	17	3	2	1	0	1	3	6	1
2548	202	0	19-Apr-10	21	2	17	3	3	1	0	1	3	6	2
2549	202	0	17-Apr-10	12	2	17	3	3	1	0	1	3	3	1
2550	202	0	16-Apr-10	21	2	17	3	3	1	0	1	3	5	1
2551	202	0	14-Jun-09	20	2	17	3	1	1	0	1	3	2	1
2552	202	0	5-Apr-09	23	2	17	3	1	1	0	1	3	7	2
2553	202	0	5-Apr-09	23	2	17	3	1	1	0	1	3	6	2
2554	202	0	29-Dec-09	1	2	17	3	3	1	0	1	3	5	1
2555	202	0	16-Nov-08	23	2	17	3	1	1	0	1	3	1	1
2556	202	0	19-Jun-08	17	2	17	3	3	1	0	1	3	4	1
2557	202	0	10-Feb-08	17	2	17	3	2	1	0	1	3	6	1
2558	202	0	24-Jun-08	17	2	17	3	1	1	0	1	3	6	1
2559	202	0	28-Nov-08	22	2	17	3	3	1	0	1	3	3	2
2560	202	0	24-Mar-08	13	2	17	3	3	1	0	1	3	3	2
2561	202	0	18-Apr-08	19	2	17	3	3	1	0	1	3	6	1
2562	202	0	10-Aug-08	4	2	17	3	3	1	0	1	3	1	1
2563	202	0	7-May-07	15	2	17	3	3	1	0	1	3	1	1
2564	202	0	19-Feb-07	21	2	17	3	3	1	0	1	3	1	2
2565	202	0	28-Apr-07	8	2	17	3	1	1	0	1	3	4	1
2566	202	0	9-Dec-07	18	2	17	3	3	1	0	1	3	6	1
2567	202	0	25-Oct-07	15	2	17	3	3	1	0	1	3	6	1
2568	202	0	11-Mar-07	19	2	17	3	3	1	0	1	3	1	1
2569	202	0	10-Dec-07	14	2	17	3	3	1	0	1	3	3	2
2570	202	0	8-Aug-07	7	2	17	3	3	1	0	1	3	1	1
2571	202	0	5-Jul-07	21	2	17	3	3	1	0	1	3	1	1
2572	202	0	12-Sep-07	3	2	17	3	1	1	0	1	3	5	1
2573	202	0	20-Nov-06	8	2	17	3	3	1	0	1	3	3	1
2574	202	0	18-Nov-06	15	2	17	3	2	1	0	1	3	1	1
2575	202	0	5-Aug-06	12	2	17	3	3	1	0	1	3	1	1
2576	202	0	11-Jun-06	3	2	17	3	3	1	0	1	3	6	2
2577	202	0	27-Dec-06	8	2	17	3	3	1	0	1	3	3	1
2578	202	0	27-Dec-06	8	2	17	3	3	1	0	1	3	3	1
2579	202	0	18-May-06	8	2	17	3	3	1	0	1	3	1	1
2580	202	0	18-Nov-06	20	2	17	3	3	1	0	1	3	1	1
2581	202	0	31-Oct-09	1	2	17	3	1	1	0	1	3	6	2
2582	202	0	6-Nov-16	16	2	17	3	1	1	0	4	3	5	1
2583	202	0	17-Feb-17	2	2	17	3	3	1	0	4	3	1	2
2584	202	0	27-Mar-14	19	2	17	3	3	1	0	11	3	3	1
2585	202	0	18-Oct-08	21	2	17	3	1	1	0	11	3	3	1
2586	202	0	27-Oct-16	14	2	17	3	3	1	0	3	3	2	1
2587	202	0	12-May-15	8	2	17	3	2	1	0	3	3	5	1
2588	202	0	14-May-15	23	2	17	3	2	1	0	3	3	1	1
2589	202	0	2-Apr-14	23	2	17	3	3	1	0	3	3	1	1
2590	202	0	26-Aug-13	23	2	17	3	3	1	0	3	3	1	1
2591	202	0	7-Aug-11	17	2	17	3	1	1	0	3	3	1	1
2592	202	0	2-Mar-17	11	2	17	3	1	1	0	3	3	5	1
2593	202	0	18-Oct-17	5	2	17	3	1	1	0	3	3	5	1
2594	202	0	31-Mar-10	17	2	17	3	1	1	0	13	3	6	2
2595	101	1	10-Dec-15	5	1	17	3	2	1	0	1	3	2	2
2596	101	1	15-Apr-16	19	1	17	3	1	1	0	1	3	4	1
2597	201	1	17-Jan-14	2	1	17	3	1	1	0	1	3	5	1

2598	201	1	22-Apr-13	16	1	17	3	2	1	0	1	3	6	1
2599	201	1	18-Nov-17	6	1	17	3	3	1	0	1	3	2	1
2600	201	1	31-Jan-16	21	1	17	3	2	1	0	1	3	3	1
2601	201	1	18-Sep-13	8	1	17	3	3	1	0	1	3	5	1
2602	101	1	21-Apr-16	6	1	17	3	1	1	0	1	3	4	4
2603	101	1	9-Apr-12	17	1	17	3	3	1	0	1	3	4	1
2604	201	1	16-May-14	7	1	17	3	3	1	0	1	3	5	2
2605	201	1	27-Apr-13	14	1	17	3	2	1	0	1	3	3	1
2606	101	1	3-Jul-12	20	1	17	3	3	1	0	1	3	2	2
2607	201	1	16-Dec-12	12	1	17	3	2	1	0	1	3	6	1
2608	201	1	24-Apr-12	14	1	17	3	2	1	0	1	3	6	1
2609	101	1	28-Oct-14	19	1	17	3	2	1	0	1	3	5	2
2610	101	1	25-Dec-13	23	1	17	3	3	1	0	1	3	3	1
2611	101	1	21-Nov-12	8	1	17	3	2	1	0	1	3	3	1
2612	201	1	28-Oct-12	11	1	17	3	3	1	0	1	3	5	1
2613	201	1	14-Mar-12	18	1	17	3	3	1	0	1	3	6	1
2614	201	1	18-Oct-14	19	1	17	3	1	1	0	1	3	5	1
2615	201	1	4-May-14	20	1	17	3	2	1	0	1	3	4	1
2616	201	1	16-Nov-13	19	1	17	3	3	1	0	1	3	1	2
2617	101	1	24-Nov-12	23	1	17	3	3	1	0	1	3	1	2
2618	101	1	17-Jan-12	4	1	17	3	2	1	0	1	3	1	2
2619	101	1	20-Jul-12	20	1	17	3	3	1	0	1	3	4	2
2620	101	1	4-Feb-17	0	1	17	3	2	1	0	1	3	3	1
2621	201	1	18-Feb-13	12	1	17	3	3	1	0	6	3	2	1
2622	201	1	25-Aug-16	0	1	17	3	3	1	0	3	3	2	1
2623	201	1	3-Dec-11	10	1	17	3	3	1	0	1	3	4	1
2624	201	1	8-Jan-11	17	1	17	3	3	1	0	1	3	1	1
2625	201	1	7-Jun-11	2	1	17	3	3	1	0	1	3	4	2
2626	101	1	19-Jul-11	15	1	17	3	3	1	0	1	3	3	2
2627	201	1	2-Jun-11	21	1	17	3	1	1	0	1	3	3	2
2628	101	1	14-Sep-10	11	1	17	3	1	1	0	1	3	4	1
2629	101	1	28-Jul-10	15	1	17	3	3	1	0	1	3	5	1
2630	101	1	16-Feb-10	2	1	17	3	3	1	0	1	3	1	2
2631	201	1	13-Apr-10	23	1	17	3	3	1	0	1	3	1	2
2632	101	1	3-Mar-10	17	1	17	3	2	1	0	1	3	1	1
2633	101	1	4-Oct-10	8	1	17	3	3	1	0	1	3	2	1
2634	101	1	10-Oct-10	9	1	17	3	3	1	0	1	3	5	1
2635	201	1	19-Aug-10	14	1	17	3	3	1	0	1	3	6	1
2636	201	1	7-Dec-10	5	1	17	3	2	1	0	1	3	1	1
2637	201	1	25-Oct-10	1	1	17	3	2	1	0	1	3	1	1
2638	101	1	23-Nov-10	8	1	17	3	2	1	0	1	3	1	2
2639	201	1	16-Jan-10	19	1	17	3	3	1	0	1	3	5	1
2640	101	1	1-Apr-10	12	1	17	3	3	1	0	1	3	3	1
2641	201	1	26-Feb-10	20	1	17	3	3	1	0	1	3	4	4
2642	201	1	29-Nov-10	21	1	17	3	3	1	0	1	3	1	2
2643	201	1	31-Jul-10	21	1	17	3	3	1	0	1	3	2	1
2644	201	1	15-Mar-09	16	1	17	3	2	1	0	1	3	1	2
2645	101	1	31-May-09	15	1	17	3	2	1	0	1	3	3	2
2646	201	1	21-Aug-09	12	1	17	3	3	1	0	1	3	1	1
2647	201	1	18-Dec-09	19	1	17	3	2	1	0	1	3	1	2
2648	101	1	17-Aug-09	2	1	17	3	2	1	0	1	3	3	1
2649	101	1	14-Aug-09	21	1	17	3	3	1	0	1	3	3	1
2650	201	1	6-Aug-09	15	1	17	3	3	1	0	1	3	1	2
2651	201	1	24-Oct-09	22	1	17	3	2	1	0	1	3	2	2
2652	201	1	27-Nov-09	1	1	17	3	3	1	0	1	3	1	2
2653	201	1	14-Jul-08	2	1	17	3	3	1	0	1	3	5	1
2654	201	1	24-Nov-08	1	1	17	3	3	1	0	1	3	1	2
2655	201	1	10-Nov-08	12	1	17	3	2	1	0	1	3	3	2
2656	101	1	5-May-08	6	1	17	3	3	1	0	1	3	4	1
2657	101	1	5-Jun-08	20	1	17	3	3	1	0	1	3	5	2
2658	101	1	12-Oct-08	12	1	17	3	3	1	0	1	3	1	4
2659	201	1	28-Jan-08	14	1	17	3	3	1	0	1	3	2	2
2660	201	1	14-Oct-08	12	1	17	3	3	1	0	1	3	3	1
2661	101	1	17-Oct-07	0	1	17	3	3	1	0	1	3	1	2
2662	101	1	7-Mar-07	1	1	17	3	3	1	0	1	3	1	1
2663	201	1	12-Nov-07	8	1	17	3	3	1	0	1	3	5	1
2664	101	1	8-Jan-07	9	1	17	3	2	1	0	1	3	1	2
2665	101	1	5-Feb-07	16	1	17	3	2	1	0	1	3	4	2
2666	201	1	2-Sep-07	2	1	17	3	3	1	0	1	3	1	1
2667	201	1	11-May-06	12	1	17	3	1	1	0	1	3	1	2
2668	101	1	22-Feb-06	14	1	17	3	2	1	0	1	3	2	1
2669	201	1	27-Sep-06	9	1	17	3	2	1	0	1	3	6	1
2670	201	1	17-Mar-06	20	1	17	3	3	1	0	1	3	4	2
2671	201	1	13-Dec-06	7	1	17	3	3	1	0	1	3	1	1
2672	201	1	16-Sep-06	20	1	17	3	3	1	0	1	3	4	1
2673	201	1	3-May-06	9	1	17	3	3	1	0	1	3	5	1
2674	201	1	30-Apr-06	20	1	17	3	3	1	0	1	3	1	1
2675	201	1	30-Oct-06	15	1	17	3	2	1	0	1	3	2	1
2676	101	1	29-Jan-17	17	1	17	3	3	1	0	1	3	2	1
2677	201	1	8-May-11	11	1	17	3	3	1	0	1	3	6	1
2678	201	1	25-Sep-10	20	1	17	3	2	1	0	1	3	3	2
2679	201	1	23-Feb-08	15	1	17	3	3	1	0	1	3	1	1
2680	101	1	3-Aug-08	20	1	17	3	3	1	0	1	3	2	1
2681	101	1	21-Aug-07	20	1	17	3	3	1	0	1	3	3	1
2682	201	1	17-Sep-07	21	1	17	3	3	1	0	1	3	6	1
2683	201	1	2-Jun-07	19	1	17	3	3	1	0	1	3	6	1
2684	201	1	11-Sep-07	20	1	17	3	3	1	0	1	3	5	1
2685	201	1	2-Jul-07	12	1	17	3	1	1	0	1	3	2	1
2686	201	1	13-Nov-07	18	1	17	3	3	1	0	1	3	1	1
2687	201	1	8-Sep-07	17	1	17	3	3	1	0	1	3	1	1
2688	101	1	1-Jul-12	2	1	17	3	1	1	0	4	3	5	1
2689	101	1	9-Oct-07	17	1	17	3	3	1	0	4	3	3	1
2690	101	1	6-Aug-17	13	1	17	3	3	1	0	4	3	3	4
2691	101	1	2-Aug-11	18	1	17	3	2	1	0	11	3	3	1
2692	201	1	25-Feb-14	19	1	17	3	3	1	0	3	3	3	1
2693	201	1	13-Dec-14	5	1	17	3	2	1	0	3	3	4	1
2694	101	1	15-Aug-12	3	1	17	3	2	1	0	3	3	3	2
2695	201	1	20-Sep-08	1	1	17	3	2	1	0	3	3	5	1
2696	101	1	16-Jun-06	15	1	17	3	2	1	0	3	3	2	1
2697	201	1	15-Jun-13	0	1	17	3	2	1	0	6	3	2	1
2698	101	1	9-Dec-11	19	1	17	3	3	1	0	6	3	1	2
2699	201	1	20-Apr-06	17	1	17	3	3	1	0	6	3	3	1
2700	201	1	29-Mar-07	19	1	17	3	3	1	0	13	3	1	1
2701	201	1	12-Jan-13	11	2	17	3	3	1	0	1	3	1	1
2702	201	1	9-Sep-14	10	2	17	3	3	1	0	1	3	4	1
2703	101	1	12-Jul-13	2	2	17	3	3	1	0	1	3	1	2
2704	201	1	27-Jun-13	1	2	17	3	3	1	0	1	3	5	1
2705	201	1	7-Aug-13	22	2	17	3	3	1	0	1	3	3	2
2706	201	1	2-Sep-12	13	2	17	3	1	1	0	1	3	6	1
2707	201	1	19-Dec-15	23	2	17	3	3	1	0	1	3	4	2
2708	101	1	29-Jul-12	14	2	17	3	1	1	0	1	3	1	2
2709	201	1	7-Nov-13	13	2	17	3	3	1	0	1	3	5	1
2710	201	1	31-Aug-12	23	2	17	3	3	1	0	6	3	2	1
2711	101	1	17-Jan-15	5	2	17	3	2	1	0	1	3	1	2
2712	201	1	10-Sep-11	22	2	17	3	3	1	0	1	3	2	1
2713	201	1	14-Aug-11	14	2	17	3	1	1	0	1	3	5	1
2714	201	1	26-Mar-11	2	2	17	3	1	1	0	1	3	6	1
2715	201	1	7-Mar-10	18	2	17	3	3	1	0	1	3	1	1
2716	101	1	29-Dec-10	21	2	17	3	2	1	0	1	3	3	2
2717	101	1	13-Apr-10	22	2	17	3	3	1	0	1	3	3	1
2718	201	1	24-Jun-08	22	2	17	3	3	1	0	1	3	5	1
2719	101	1	28-Mar-08	8	2	17	3	3	1	0	1	3	1	1
2720	201	1	9-Dec-08	12	2	17	3	3	1	0	1	3	1	2
2721	201	1	5-Dec-08	1	2	17	3	2	1	0	1	3	5	1

2722	101	1	20-Jul-08	18	2	17	3	3	1	0	1	3	5	1
2723	101	1	21-Jan-08	10	2	17	3	3	1	0	1	3	1	1
2724	201	1	14-Oct-08	12	2	17	3	3	1	0	1	3	3	1
2725	201	1	9-Mar-08	23	2	17	3	3	1	0	1	3	2	2
2726	201	1	19-Mar-07	17	2	17	3	3	1	0	1	3	3	1
2727	201	1	1-Apr-07	10	2	17	3	3	1	0	1	3	1	1
2728	201	1	1-May-07	16	2	17	3	1	1	0	1	3	1	2
2729	201	1	19-Jun-07	17	2	17	3	3	1	0	1	3	3	2
2730	201	1	12-Feb-07	22	2	17	3	3	1	0	1	3	1	2
2731	201	1	6-Mar-07	21	2	17	3	3	1	0	1	3	1	2
2732	201	1	31-Dec-07	17	2	17	3	2	1	0	1	3	1	1
2733	201	1	25-Nov-07	12	2	17	3	3	1	0	1	3	3	1
2734	101	1	13-Dec-07	21	2	17	3	3	1	0	1	3	3	1
2735	201	1	15-Apr-07	21	2	17	3	3	1	0	1	3	1	1
2736	201	1	3-Oct-06	9	2	17	3	3	1	0	1	3	1	2
2737	101	1	11-Feb-06	7	2	17	3	3	1	0	1	3	5	2
2738	201	1	21-Feb-06	23	2	17	3	3	1	0	1	3	1	1
2739	201	1	29-Mar-06	0	2	17	3	3	1	0	1	3	1	1
2740	201	1	2-May-06	20	2	17	3	3	1	0	1	3	2	1
2741	201	1	14-Mar-11	17	2	17	3	3	1	0	1	3	1	2
2742	201	1	25-Mar-11	22	2	17	3	2	1	0	1	3	1	2
2743	101	1	26-Nov-10	17	2	17	3	1	1	0	1	3	5	1
2744	201	1	21-Oct-09	20	2	17	3	3	1	0	1	3	2	2
2745	201	1	29-Nov-07	12	2	17	3	3	1	0	1	3	4	1
2746	201	1	17-Sep-06	19	2	17	3	3	1	0	1	3	2	1
2747	201	1	3-May-06	20	2	17	3	3	1	0	1	3	3	1
2748	101	1	6-Feb-07	4	2	17	3	3	1	0	4	3	1	1
2749	201	1	9-Jul-08	17	2	17	3	3	1	0	11	3	5	1
2750	201	1	4-Nov-16	20	2	17	3	2	1	0	3	3	3	1
2751	101	1	21-Apr-15	2	2	17	3	1	1	0	3	3	2	4
2752	201	1	10-Mar-13	22	2	17	3	2	1	0	3	3	2	1
2753	201	1	6-Jul-12	21	2	17	3	3	1	0	3	3	1	1
2754	201	1	22-Nov-11	11	2	17	3	3	1	0	3	3	3	1
2755	101	1	15-May-11	23	2	17	3	2	1	0	3	3	3	1
2756	201	1	4-May-10	22	2	17	3	3	1	0	3	3	3	1
2757	201	1	24-Jan-10	19	2	17	3	3	1	0	3	3	4	1
2758	201	1	9-Jul-09	12	2	17	3	3	1	0	3	3	3	1
2759	201	1	16-Oct-09	18	2	17	3	3	1	0	3	3	2	1
2760	201	1	22-May-07	18	2	17	3	3	1	0	3	3	3	1
2761	201	1	2-Oct-07	22	2	17	3	3	1	0	3	3	3	1
2762	201	1	31-Aug-17	4	2	17	3	3	1	0	3	3	3	1
2763	101	1	24-Oct-17	19	2	17	3	3	1	0	3	3	1	1
2764	101	1	22-Mar-15	3	2	17	3	2	1	0	6	3	2	1
2765	202	0	6-Jun-13	21	2	14	3	3	3	1	4	2	4	1
2766	202	0	4-May-14	7	1	15	3	2	3	1	4	2	1	2
2767	202	0	20-Dec-09	22	1	15	3	3	3	1	6	2	2	2
2768	202	0	10-Jun-16	15	2	15	3	3	3	1	4	2	1	1
2769	202	0	4-Jun-17	20	2	15	3	2	3	1	4	2	2	1
2770	101	1	20-Apr-13	3	1	15	3	2	3	1	4	2	1	1
2771	101	1	6-Jul-13	10	1	15	3	2	3	1	4	2	1	1
2772	101	1	27-Jan-13	23	1	15	3	1	3	1	4	2	1	2
2773	101	1	9-Feb-12	14	1	15	3	2	3	1	4	2	4	1
2774	101	1	11-May-11	23	1	15	3	3	3	1	4	2	1	1
2775	201	1	27-Jul-10	4	1	15	3	3	3	1	4	2	1	1
2776	201	1	23-Dec-10	23	1	15	3	3	3	1	4	2	4	1
2777	101	1	15-Jun-09	11	1	15	3	3	3	1	4	2	5	1
2778	101	1	21-Jul-09	20	1	15	3	3	3	1	4	2	5	1
2779	101	1	9-Jul-11	20	1	15	3	1	3	1	11	2	3	1
2780	101	1	18-Aug-12	1	2	15	3	2	3	1	4	2	4	1
2781	202	0	21-Aug-13	7	1	16	3	3	3	1	4	2	5	1
2782	202	0	5-Jan-16	22	1	16	3	2	3	1	4	2	1	1
2783	202	0	24-Nov-15	13	1	16	3	3	3	1	4	2	1	1
2784	202	0	25-Sep-14	21	1	16	3	3	3	1	4	2	1	1
2785	202	0	29-Jul-14	3	1	16	3	1	3	1	4	2	2	1
2786	202	0	26-May-10	2	1	16	3	3	3	1	4	2	1	1
2787	202	0	29-Sep-07	11	1	16	3	3	3	1	4	2	1	2
2788	202	0	24-May-17	16	1	16	3	3	3	1	4	2	5	1
2789	202	0	19-Apr-11	14	1	16	3	1	3	1	6	2	1	1
2790	202	0	6-Nov-08	1	1	16	3	1	3	1	6	2	1	2
2791	202	0	1-Jan-07	0	1	16	3	3	3	1	6	2	2	1
2792	202	0	17-Dec-07	2	2	16	3	2	3	1	10	2	2	1
2793	202	0	17-Oct-16	21	2	16	3	3	3	1	4	2	1	1
2794	202	0	21-Mar-13	22	2	16	3	1	3	1	4	2	4	1
2795	202	0	24-Oct-09	12	2	16	3	3	3	1	4	2	1	2
2796	202	0	4-Oct-12	19	2	16	3	3	3	1	11	2	4	2
2797	201	1	19-Dec-17	5	1	16	3	2	3	1	6	2	5	1
2798	201	1	13-Dec-12	8	1	16	3	3	3	1	13	2	6	1
2799	101	1	10-Apr-15	21	1	16	3	2	3	1	4	2	2	1
2800	101	1	13-Jun-13	20	1	16	3	3	3	1	4	2	1	1
2801	101	1	2-Nov-13	1	1	16	3	2	3	1	4	2	1	1
2802	101	1	8-Oct-13	16	1	16	3	2	3	1	4	2	1	1
2803	101	1	27-Jan-13	23	1	16	3	1	3	1	4	2	5	1
2804	101	1	28-Jun-12	23	1	16	3	2	3	1	4	2	1	1
2805	101	1	21-Mar-12	1	1	16	3	3	3	1	4	2	1	1
2806	101	1	15-Jan-12	17	1	16	3	1	3	1	4	2	1	2
2807	101	1	2-Mar-11	3	1	16	3	2	3	1	4	2	2	1
2808	101	1	7-Jan-11	2	1	16	3	2	3	1	4	2	1	1
2809	101	1	14-Jul-11	8	1	16	3	3	3	1	4	2	2	4
2810	201	1	23-Sep-11	10	1	16	3	3	3	1	4	2	1	1
2811	201	1	1-Aug-10	23	1	16	3	3	3	1	4	2	6	1
2812	101	1	7-Feb-10	1	1	16	3	3	3	1	4	2	1	4
2813	201	1	27-Jul-10	4	1	16	3	3	3	1	4	2	1	1
2814	201	1	17-Apr-10	19	1	16	3	3	3	1	4	2	1	1
2815	101	1	10-Nov-09	19	1	16	3	3	3	1	4	2	1	1
2816	101	1	24-Aug-09	19	1	16	3	3	3	1	4	2	5	2
2817	101	1	15-Nov-09	22	1	16	3	3	3	1	4	2	1	1
2818	101	1	27-Sep-09	8	1	16	3	3	3	1	4	2	2	4
2819	101	1	22-Nov-07	15	1	16	3	3	3	1	4	2	5	1
2820	101	1	30-Jul-07	6	1	16	3	3	3	1	4	2	2	4
2821	101	1	28-Sep-07	10	1	16	3	3	3	1	4	2	1	1
2822	101	1	31-Aug-12	21	1	16	3	2	3	1	6	2	2	1
2823	101	1	6-Nov-11	2	1	16	3	3	3	1	6	2	2	1
2824	101	1	29-Nov-09	0	1	16	3	2	3	1	6	2	1	4
2825	101	1	10-Jul-07	1	1	16	3	3	3	1	6	2	1	2
2826	101	1	18-Apr-07	20	1	16	3	3	3	1	6	2	2	2
2827	201	1	9-Jan-13	15	2	16	3	3	3	1	13	2	1	1
2828	101	1	23-Oct-13	23	2	16	3	3	3	1	4	2	1	1
2829	101	1	18-Aug-12	0	2	16	3	3	3	1	4	2	5	1
2830	201	1	25-Oct-12	19	2	16	3	3	3	1	4	2	1	1
2831	101	1	26-Sep-12	16	2	16	3	3	3	1	4	2	5	1
2832	101	1	1-May-12	23	2	16	3	2	3	1	4	2	1	1
2833	101	1	29-Jul-08	3	2	16	3	3	3	1	4	2	1	1
2834	101	1	27-Aug-07	9	2	16	3	3	3	1	4	2	1	2
2835	202	0	31-Oct-15	20	1	17	3	3	3	1	4	2	1	1
2836	202	0	12-Mar-14	23	1	17	3	3	3	1	4	2	1	1
2837	202	0	11-Sep-15	17	1	17	3	1	3	1	4	2	4	1
2838	202	0	31-Jan-14	0	1	17	3	1	3	1	6	2	5	2
2839	202	0	27-Apr-14	5	1	17	3	1	3	1	6	2	1	1
2840	202	0	31-Jul-14	3	1	17	3	3	3	1	6	2	4	1
2841	202	0	11-Jun-14	21	1	17	3	1	3	1	6	2	4	2
2842	202	0	22-May-13	21	1	17	3	3	3	1	6	2	4	2
2843	202	0	26-May-15	0	1	17	3	2	3	1	6	2	2	1
2844	202	0	27-Apr-14	1	1	17	3	1	3	1	6	2	1	1
2845	202	0	27-Dec-13	0	1	17	3	2	3	1	6	2	5	2

2846	202	0	2-May-14	3	1	17	3	2	3	1	4	2	1	1
2847	202	0	16-Mar-08	10	1	17	3	1	3	1	13	2	4	2
2848	202	0	6-Dec-07	1	1	17	3	2	3	1	13	2	6	2
2849	202	0	18-Apr-14	18	1	17	3	2	3	1	4	2	4	1
2850	202	0	27-Jun-13	21	1	17	3	1	3	1	4	2	2	1
2851	202	0	19-Jun-13	12	1	17	3	2	3	1	4	2	5	1
2852	202	0	13-Jun-13	3	1	17	3	1	3	1	4	2	1	1
2853	202	0	15-Nov-13	2	1	17	3	2	3	1	4	2	1	2
2854	202	0	11-Jul-13	12	1	17	3	2	3	1	4	2	1	1
2855	202	0	4-Sep-12	18	1	17	3	1	3	1	4	2	1	1
2856	202	0	23-Feb-12	17	1	17	3	3	3	1	4	2	5	1
2857	202	0	28-Apr-09	1	1	17	3	2	3	1	4	2	1	1
2858	202	0	26-Dec-09	22	1	17	3	1	3	1	4	2	1	1
2859	202	0	27-Jul-09	23	1	17	3	3	3	1	4	2	1	2
2860	202	0	2-Apr-08	1	1	17	3	1	3	1	4	2	1	1
2861	202	0	7-Nov-08	19	1	17	3	1	3	1	4	2	4	2
2862	202	0	13-Oct-07	18	1	17	3	3	3	1	4	2	4	1
2863	202	0	24-Oct-07	22	1	17	3	3	3	1	4	2	1	1
2864	202	0	22-Oct-06	2	1	17	3	2	3	1	4	2	1	1
2865	202	0	14-Jul-06	1	1	17	3	1	3	1	4	2	1	1
2866	202	0	5-Jan-06	22	1	17	3	3	3	1	4	2	1	1
2867	202	0	27-Aug-17	3	1	17	3	2	3	1	4	2	1	1
2868	202	0	30-Oct-17	2	1	17	3	3	3	1	3	2	5	1
2869	202	0	19-Mar-13	4	1	17	3	2	3	1	6	2	2	2
2870	202	0	1-Jan-12	1	1	17	3	2	3	1	6	2	1	2
2871	202	0	16-Dec-11	3	1	17	3	1	3	1	6	2	2	2
2872	202	0	1-Sep-09	0	1	17	3	1	3	1	6	2	1	2
2873	202	0	16-Feb-07	2	1	17	3	1	3	1	6	2	4	2
2874	202	0	12-Mar-07	0	1	17	3	3	3	1	6	2	3	1
2875	202	0	17-Nov-07	9	1	17	3	3	3	1	6	2	2	2
2876	202	0	4-Jun-16	12	2	17	3	2	3	1	4	2	4	1
2877	202	0	27-May-15	21	2	17	3	1	3	1	4	2	4	1
2878	202	0	5-Dec-15	21	2	17	3	3	3	1	4	2	4	1
2879	202	0	18-May-10	10	2	17	3	1	3	1	11	2	3	1
2880	202	0	19-Jul-10	1	2	17	3	1	3	1	6	2	1	1
2881	201	1	23-Jan-12	16	1	17	3	3	3	1	13	2	1	1
2882	101	1	14-Apr-14	21	1	17	3	3	3	1	4	2	1	1
2883	101	1	7-Mar-14	0	1	17	3	3	3	1	4	2	1	1
2884	201	1	9-Jul-12	5	1	17	3	3	3	1	4	2	5	1
2885	101	1	28-Jun-12	22	1	17	3	2	3	1	4	2	1	1
2886	101	1	15-Sep-12	21	1	17	3	2	3	1	4	2	1	1
2887	101	1	8-Nov-12	22	1	17	3	2	3	1	4	2	5	1
2888	101	1	1-Feb-10	16	1	17	3	2	3	1	4	2	1	1
2889	101	1	23-Aug-10	20	1	17	3	2	3	1	4	2	1	1
2890	201	1	13-Jul-10	11	1	17	3	2	3	1	4	2	5	1
2891	201	1	31-May-10	10	1	17	3	3	3	1	4	2	6	1
2892	201	1	1-Aug-10	23	1	17	3	3	3	1	4	2	1	1
2893	201	1	28-Jul-10	9	1	17	3	3	3	1	4	2	1	1
2894	101	1	12-May-10	2	1	17	3	3	3	1	4	2	1	1
2895	101	1	27-Jun-09	16	1	17	3	1	3	1	4	2	1	1
2896	101	1	28-Dec-08	3	1	17	3	2	3	1	4	2	1	1
2897	201	1	25-Jun-08	10	1	17	3	3	3	1	4	2	6	1
2898	201	1	28-Mar-08	2	1	17	3	2	3	1	4	2	1	1
2899	201	1	28-Jun-08	17	1	17	3	3	3	1	4	2	1	1
2900	101	1	9-Aug-08	20	1	17	3	1	3	1	4	2	1	1
2901	201	1	31-Aug-08	22	1	17	3	3	3	1	4	2	2	1
2902	101	1	26-Feb-08	22	1	17	3	3	3	1	4	2	1	1
2903	201	1	19-Jun-08	11	1	17	3	3	3	1	4	2	1	1
2904	101	1	24-Dec-07	19	1	17	3	3	3	1	4	2	1	1
2905	101	1	18-Jul-07	1	1	17	3	3	3	1	4	2	1	2
2906	201	1	16-Dec-07	3	1	17	3	3	3	1	4	2	1	1
2907	201	1	28-Oct-07	2	1	17	3	2	3	1	4	2	1	2
2908	101	1	9-Apr-07	22	1	17	3	2	3	1	4	2	1	1
2909	101	1	27-Aug-17	11	1	17	3	2	3	1	4	2	1	1
2910	201	1	8-May-13	1	1	17	3	3	3	1	6	2	1	1
2911	101	1	21-Apr-12	1	1	17	3	3	3	1	6	2	2	2
2912	101	1	29-Oct-12	21	1	17	3	4	3	1	6	2	2	4
2913	101	1	21-May-12	20	1	17	3	2	3	1	6	2	3	1
2914	101	1	24-Nov-10	1	1	17	3	3	3	1	6	2	1	2
2915	101	1	11-Aug-10	22	1	17	3	1	3	1	6	2	2	1
2916	201	1	28-Aug-09	21	1	17	3	2	3	1	6	2	3	2
2917	101	1	14-May-08	23	1	17	3	3	3	1	6	2	4	2
2918	201	1	29-Sep-08	19	1	17	3	3	3	1	6	2	4	2
2919	201	1	13-May-07	1	1	17	3	3	3	1	6	2	2	1
2920	101	1	23-Oct-07	16	1	17	3	3	3	1	6	2	5	2
2921	101	1	3-Jul-06	22	1	17	3	3	3	1	6	2	5	2
2922	201	1	21-Nov-13	5	2	17	3	3	3	1	6	2	1	2
2923	201	1	22-Nov-08	21	2	17	3	4	3	1	1	2	1	2
2924	101	1	27-Mar-13	21	2	17	3	2	3	1	4	2	5	1
2925	101	1	8-Jun-11	10	2	17	3	3	3	1	4	2	1	1
2926	201	1	2-Aug-10	0	2	17	3	3	3	1	4	2	1	1
2927	101	1	21-Dec-09	21	2	17	3	3	3	1	4	2	1	1
2928	101	1	9-Jul-08	22	2	17	3	2	3	1	4	2	1	1
2929	201	1	15-Jun-08	1	2	17	3	3	3	1	4	2	1	1
2930	201	1	17-Sep-07	19	2	17	3	3	3	1	4	2	2	1
2931	101	1	24-Nov-13	22	2	17	3	3	3	1	6	2	3	1
2932	101	1	27-Apr-14	12	2	15	3	3	2	1	6	2	2	1
2933	201	1	29-Jul-09	22	1	16	3	2	2	1	6	2	2	2
2934	202	0	18-Feb-17	23	1	17	3	3	2	1	4	2	1	1
2935	101	1	15-Nov-11	13	1	17	3	3	2	1	7	2	2	1
2936	101	1	25-Nov-11	23	1	17	3	3	2	1	4	2	2	1
2937	101	1	28-Mar-13	21	2	17	3	2	2	1	6	2	2	1
2938	202	0	2-Aug-14	15	2	10	3	2	1	0	3	2	1	1
2939	201	1	1-Jun-11	4	1	12	3	3	1	0	6	2	2	1
2940	202	0	29-Sep-13	18	1	13	3	1	1	0	11	2	3	1
2941	202	0	22-Aug-17	21	2	13	3	3	1	0	11	2	2	1
2942	101	1	24-Dec-11	22	1	13	3	3	1	0	6	2	2	4
2943	202	0	18-Aug-16	21	1	14	3	3	1	0	11	2	2	1
2944	202	0	12-Feb-16	12	1	14	3	1	1	0	3	2	4	1
2945	202	0	11-Aug-17	22	1	14	3	3	1	0	3	2	3	1
2946	201	1	21-Jan-16	9	1	14	3	3	1	0	3	2	3	1
2947	101	1	23-Mar-12	4	1	14	3	2	1	0	3	2	1	1
2948	101	1	8-Dec-10	10	1	14	3	3	1	0	3	2	3	1
2949	101	1	28-May-15	16	1	14	3	1	1	0	6	2	2	1
2950	101	1	29-Apr-14	10	2	14	3	3	1	0	3	2	3	1
2951	201	1	25-Feb-11	19	2	14	3	3	1	0	3	2	6	1
2952	101	1	24-Jul-10	19	2	14	3	3	1	0	3	2	3	1
2953	202	0	29-Jan-17	21	1	15	3	3	1	0	10	2	6	2
2954	202	0	8-Jul-13	22	1	15	3	1	1	0	6	2	2	1
2955	202	0	3-Dec-16	7	1	15	3	2	1	0	4	2	2	1
2956	202	0	17-Jun-15	0	1	15	3	1	1	0	3	2	5	1
2957	202	0	23-Jun-10	12	1	15	3	2	1	0	6	2	2	1
2958	202	0	28-Aug-15	21	2	15	3	3	1	0	3	2	1	1
2959	201	1	29-Mar-13	5	1	15	3	3	1	0	6	2	1	1
2960	201	1	5-Apr-15	17	1	15	3	3	1	0	4	2	1	1
2961	201	1	21-Aug-13	21	1	15	3	3	1	0	4	2	5	1
2962	201	1	11-Nov-10	16	1	15	3	3	1	0	4	2	1	1
2963	101	1	5-Oct-09	10	1	15	3	2	1	0	4	2	1	1
2964	201	1	14-Dec-12	18	1	15	3	2	1	0	11	2	2	1
2965	201	1	14-Jan-12	20	1	15	3	3	1	0	11	2	3	1
2966	101	1	8-Aug-12	22	1	15	3	3	1	0	3	2	4	1
2967	201	1	1-Dec-10	3	1	15	3	3	1	0	3	2	2	2
2968	101	1	6-Oct-09	11	1	15	3	3	1	0	3	2	1	2
2969	101	1	2-Nov-09	19	1	15	3	3	1	0	3	2	1	2

2970	101	1	17-May-10	23	1	15	3	3	1	0	6	2	2	1
2971	101	1	5-Nov-09	18	1	15	3	2	1	0	6	2	2	1
2972	201	1	17-Dec-12	11	2	15	3	3	1	0	3	2	1	1
2973	201	1	18-Nov-11	10	2	15	3	3	1	0	3	2	3	1
2974	101	1	9-Jul-10	22	2	15	3	2	1	0	3	2	1	1
2975	101	1	17-Dec-10	14	2	15	3	3	1	0	6	2	2	1
2976	202	0	15-Jun-15	6	1	16	3	2	1	0	10	2	6	1
2977	202	0	11-Jun-15	3	1	16	3	1	1	0	10	2	6	1
2978	202	0	22-Sep-12	1	1	16	3	1	1	0	10	2	7	1
2979	202	0	19-Aug-14	22	1	16	3	2	1	0	4	2	1	1
2980	202	0	16-Oct-14	19	1	16	3	1	1	0	6	2	2	1
2981	202	0	29-Jun-13	22	1	16	3	3	1	0	6	2	5	2
2982	202	0	5-Apr-17	3	1	16	3	3	1	0	6	2	5	1
2983	202	0	24-Jun-16	1	1	16	3	3	1	0	10	2	6	2
2984	202	0	1-Dec-16	1	1	16	3	3	1	0	4	2	3	2
2985	202	0	7-Dec-14	21	1	16	3	3	1	0	4	2	5	1
2986	202	0	1-Jan-12	2	1	16	3	2	1	0	4	2	2	2
2987	202	0	6-Jun-10	18	1	16	3	1	1	0	4	2	1	1
2988	202	0	16-Jul-17	22	1	16	3	3	1	0	4	2	2	1
2989	202	0	6-Sep-16	23	1	16	3	1	1	0	3	2	1	1
2990	202	0	25-Sep-16	0	1	16	3	3	1	0	3	2	1	2
2991	202	0	22-Dec-16	17	1	16	3	1	1	0	3	2	2	1
2992	202	0	27-Nov-15	15	1	16	3	1	1	0	3	2	3	1
2993	202	0	27-Sep-15	23	1	16	3	2	1	0	3	2	3	1
2994	202	0	24-Nov-14	9	1	16	3	3	1	0	3	2	4	2
2995	202	0	16-Jun-11	3	1	16	3	1	1	0	3	2	1	1
2996	202	0	12-Nov-17	20	1	16	3	3	1	0	3	2	3	1
2997	202	0	10-Aug-17	3	1	16	3	3	1	0	3	2	1	2
2998	202	0	20-Mar-17	15	1	16	3	2	1	0	3	2	3	1
2999	202	0	11-Jun-12	9	1	16	3	1	1	0	6	2	2	2
3000	202	0	4-Oct-10	12	1	16	3	1	1	0	6	2	2	2
3001	202	0	25-Apr-09	2	1	16	3	1	1	0	6	2	2	1
3002	202	0	16-Sep-17	19	1	16	3	2	1	0	6	2	3	1
3003	202	0	1-Dec-17	1	1	16	3	1	1	0	6	2	2	1
3004	202	0	28-Jul-17	16	2	16	3	3	1	0	7	2	2	1
3005	202	0	20-Sep-16	23	2	16	3	2	1	0	6	2	2	1
3006	202	0	3-Feb-10	4	2	16	3	1	1	0	10	2	7	2
3007	202	0	23-Mar-14	1	2	16	3	1	1	0	4	2	1	1
3008	202	0	14-Nov-16	20	2	16	3	1	1	0	11	2	2	1
3009	202	0	12-Nov-14	21	2	16	3	1	1	0	11	2	3	1
3010	202	0	22-May-17	10	2	16	3	2	1	0	11	2	3	1
3011	202	0	9-Jan-16	22	2	16	3	3	1	0	3	2	1	2
3012	202	0	23-Jan-16	11	2	16	3	3	1	0	3	2	3	2
3013	202	0	14-Dec-13	1	2	16	3	1	1	0	3	2	2	2
3014	202	0	12-May-12	19	2	16	3	2	1	0	3	2	3	2
3015	202	0	31-May-11	22	2	16	3	3	1	0	3	2	2	2
3016	202	0	31-Oct-06	21	2	16	3	3	1	0	3	2	2	2
3017	202	0	19-Oct-06	22	2	16	3	2	1	0	3	2	1	2
3018	202	0	5-Feb-17	12	2	16	3	3	1	0	3	2	3	1
3019	201	1	20-Oct-15	19	1	16	3	3	1	0	10	2	6	1
3020	101	1	11-Mar-12	22	1	16	3	3	1	0	4	2	2	1
3021	201	1	30-Sep-14	13	1	16	3	3	1	0	6	2	3	1
3022	201	1	17-Jul-13	8	1	16	3	2	1	0	6	2	5	1
3023	101	1	25-Jul-08	15	1	16	3	2	1	0	7	2	2	1
3024	201	1	28-Aug-10	5	1	16	3	3	1	0	1	2	3	1
3025	101	1	17-Mar-14	10	1	16	3	2	1	0	4	2	1	1
3026	201	1	29-Jun-13	17	1	16	3	3	1	0	4	2	5	1
3027	201	1	24-Jan-13	13	1	16	3	3	1	0	4	2	1	1
3028	201	1	2-Aug-13	22	1	16	3	3	1	0	4	2	1	1
3029	101	1	20-Apr-12	17	1	16	3	1	1	0	4	2	1	1
3030	101	1	10-Oct-12	10	1	16	3	3	1	0	4	2	3	1
3031	101	1	15-Feb-12	18	1	16	3	3	1	0	4	2	2	4
3032	101	1	17-Jul-11	15	1	16	3	3	1	0	4	2	2	1
3033	201	1	26-Sep-11	8	1	16	3	3	1	0	4	2	1	1
3034	201	1	8-Feb-11	14	1	16	3	3	1	0	4	2	1	1
3035	101	1	16-Aug-09	1	1	16	3	3	1	0	4	2	2	1
3036	101	1	11-Sep-09	11	1	16	3	3	1	0	4	2	2	1
3037	101	1	15-Nov-14	22	1	16	3	2	1	0	11	2	3	1
3038	201	1	16-Oct-12	18	1	16	3	3	1	0	11	2	3	1
3039	201	1	1-Mar-09	17	1	16	3	2	1	0	11	2	3	1
3040	101	1	6-Oct-09	15	1	16	3	3	1	0	11	2	2	1
3041	201	1	15-Oct-08	12	1	16	3	3	1	0	11	2	3	1
3042	201	1	6-Nov-17	11	1	16	3	3	1	0	11	2	2	1
3043	101	1	15-Mar-16	16	1	16	3	3	1	0	3	2	1	1
3044	201	1	21-Jan-16	10	1	16	3	3	1	0	3	2	1	1
3045	101	1	5-May-15	3	1	16	3	2	1	0	3	2	1	2
3046	201	1	24-Feb-15	14	1	16	3	2	1	0	3	2	4	1
3047	201	1	20-Apr-15	18	1	16	3	3	1	0	3	2	3	1
3048	201	1	14-Jun-15	3	1	16	3	3	1	0	3	2	1	1
3049	101	1	21-Dec-13	15	1	16	3	3	1	0	3	2	1	1
3050	101	1	1-Apr-13	23	1	16	3	3	1	0	3	2	1	1
3051	201	1	3-Sep-13	15	1	16	3	3	1	0	3	2	3	1
3052	201	1	12-Mar-13	10	1	16	3	3	1	0	3	2	3	1
3053	201	1	24-Aug-13	20	1	16	3	3	1	0	3	2	3	1
3054	101	1	26-Mar-13	14	1	16	3	2	1	0	3	2	7	1
3055	101	1	26-Oct-12	14	1	16	3	2	1	0	3	2	4	1
3056	101	1	12-Jun-12	23	1	16	3	2	1	0	3	2	3	1
3057	101	1	23-Jul-12	23	1	16	3	1	1	0	3	2	1	1
3058	101	1	26-May-12	23	1	16	3	2	1	0	3	2	1	4
3059	101	1	3-Nov-12	19	1	16	3	2	1	0	3	2	2	4
3060	201	1	16-Oct-12	15	1	16	3	2	1	0	3	2	2	1
3061	101	1	8-Feb-11	21	1	16	3	3	1	0	3	2	3	1
3062	201	1	12-May-11	10	1	16	3	3	1	0	3	2	6	1
3063	201	1	12-May-11	9	1	16	3	3	1	0	3	2	2	2
3064	101	1	29-Oct-10	20	1	16	3	2	1	0	3	2	1	1
3065	101	1	24-May-10	17	1	16	3	3	1	0	3	2	3	2
3066	101	1	28-Dec-09	13	1	16	3	3	1	0	3	2	3	1
3067	201	1	22-Mar-09	0	1	16	3	2	1	0	3	2	2	2
3068	201	1	6-May-09	21	1	16	3	2	1	0	3	2	3	2
3069	101	1	15-Oct-08	22	1	16	3	3	1	0	3	2	3	4
3070	201	1	28-Oct-08	3	1	16	3	3	1	0	3	2	1	1
3071	101	1	15-Apr-08	16	1	16	3	3	1	0	3	2	2	4
3072	201	1	14-Apr-08	18	1	16	3	3	1	0	3	2	2	1
3073	201	1	29-Mar-08	14	1	16	3	3	1	0	3	2	2	1
3074	101	1	27-Aug-08	23	1	16	3	3	1	0	3	2	3	2
3075	201	1	5-Jun-07	15	1	16	3	2	1	0	3	2	6	1
3076	101	1	21-Oct-06	20	1	16	3	3	1	0	3	2	2	1
3077	201	1	10-Nov-06	2	1	16	3	3	1	0	3	2	1	1
3078	201	1	30-Oct-17	12	1	16	3	2	1	0	3	2	1	1
3079	101	1	24-Sep-15	3	1	16	3	1	1	0	6	2	2	2
3080	201	1	9-Aug-13	11	1	16	3	3	1	0	6	2	3	1
3081	101	1	23-Apr-12	15	1	16	3	2	1	0	6	2	3	1
3082	101	1	8-Jul-12	23	1	16	3	2	1	0	6	2	2	2
3083	201	1	20-Jul-11	17	1	16	3	3	1	0	6	2	7	2
3084	201	1	14-Jun-11	19	1	16	3	3	1	0	6	2	2	1
3085	201	1	14-Mar-11	20	1	16	3	3	1	0	6	2	2	2
3086	201	1	27-May-10	18	1	16	3	2	1	0	6	2	2	1
3087	201	1	25-Jun-09	13	1	16	3	3	1	0	6	2	3	1
3088	101	1	17-Sep-09	1	1	16	3	3	1	0	6	2	2	1
3089	101	1	4-Nov-07	3	1	16	3	1	1	0	6	2	3	4
3090	101	1	12-Dec-06	19	1	16	3	1	1	0	6	2	3	2
3091	101	1	26-May-11	12	2	16	3	2	1	0	7	2	3	1
3092	201	1	17-Dec-12	11	2	16	3	3	1	0	4	2	1	1
3093	201	1	26-Nov-10	15	2	16	3	3	1	0	4	2	3	1

3094	101	1	17-May-08	22	2	16	3	3	1	0	4	2	1	2
3095	101	1	1-Sep-14	23	2	16	3	3	1	0	11	2	2	1
3096	201	1	20-Jul-12	23	2	16	3	3	1	0	11	2	2	1
3097	201	1	11-Jul-10	18	2	16	3	2	1	0	11	2	3	1
3098	201	1	21-Mar-16	11	2	16	3	3	1	0	3	2	3	1
3099	101	1	17-Oct-15	15	2	16	3	3	1	0	3	2	3	1
3100	201	1	18-Jul-13	20	2	16	3	3	1	0	3	2	3	1
3101	201	1	12-Feb-13	8	2	16	3	2	1	0	3	2	1	1
3102	201	1	23-Jul-13	15	2	16	3	3	1	0	3	2	1	1
3103	201	1	24-Nov-13	11	2	16	3	3	1	0	3	2	3	1
3104	101	1	8-Mar-11	23	2	16	3	2	1	0	3	2	2	1
3105	101	1	26-Jun-11	8	2	16	3	3	1	0	3	2	2	4
3106	201	1	18-Feb-11	20	2	16	3	1	1	0	3	2	4	1
3107	101	1	19-Jul-11	2	2	16	3	3	1	0	3	2	1	1
3108	101	1	28-Apr-06	22	2	16	3	3	1	0	3	2	1	1
3109	201	1	17-May-17	18	2	16	3	3	1	0	3	2	2	1
3110	101	1	29-May-11	15	2	16	3	3	1	0	6	2	3	1
3111	202	0	28-Jul-15	19	1	17	3	3	1	0	6	2	2	1
3112	202	0	7-Sep-17	20	1	17	3	3	1	0	6	2	2	1
3113	202	0	27-Feb-14	4	1	17	3	1	1	0	6	2	2	1
3114	202	0	19-Jan-17	0	1	17	3	3	1	0	10	2	7	2
3115	202	0	2-Feb-16	14	1	17	3	3	1	0	6	2	2	1
3116	202	0	2-Feb-12	2	1	17	3	1	1	0	10	2	7	1
3117	202	0	25-Dec-15	8	1	17	3	3	1	0	6	2	2	1
3118	202	0	3-Feb-16	19	1	17	3	3	1	0	6	2	3	1
3119	202	0	6-Jul-13	7	1	17	3	1	1	0	3	2	2	2
3120	202	0	2-Nov-14	0	1	17	3	3	1	0	4	2	1	1
3121	202	0	24-Apr-16	1	1	17	3	3	1	0	6	2	2	2
3122	202	0	3-Jul-16	22	1	17	3	3	1	0	4	2	2	1
3123	202	0	8-Jul-16	19	1	17	3	3	1	0	10	2	7	2
3124	202	0	25-Sep-15	14	1	17	3	1	1	0	10	2	6	1
3125	202	0	25-May-14	23	1	17	3	1	1	0	10	2	7	1
3126	202	0	1-May-10	22	1	17	3	3	1	0	10	2	7	2
3127	202	0	2-Apr-16	10	1	17	3	3	1	0	7	2	3	1
3128	202	0	30-Jun-10	11	1	17	3	2	1	0	7	2	5	2
3129	202	0	7-Jan-17	22	1	17	3	3	1	0	7	2	1	1
3130	202	0	27-Nov-08	1	1	17	3	1	1	0	1	2	3	2
3131	202	0	9-Nov-15	10	1	17	3	3	1	0	4	2	3	1
3132	202	0	26-Jan-14	11	1	17	3	1	1	0	4	2	1	1
3133	202	0	26-Dec-14	14	1	17	3	3	1	0	4	2	1	2
3134	202	0	15-Jul-14	19	1	17	3	3	1	0	4	2	1	1
3135	202	0	18-Nov-14	1	1	17	3	2	1	0	4	2	1	1
3136	202	0	1-Aug-14	22	1	17	3	1	1	0	4	2	3	1
3137	202	0	21-Nov-12	12	1	17	3	1	1	0	4	2	1	1
3138	202	0	18-Dec-11	2	1	17	3	1	1	0	4	2	2	2
3139	202	0	28-Jul-11	0	1	17	3	1	1	0	4	2	2	1
3140	202	0	22-Nov-07	11	1	17	3	2	1	0	4	2	3	2
3141	202	0	1-Oct-06	14	1	17	3	3	1	0	4	2	3	1
3142	202	0	11-Apr-06	18	1	17	3	2	1	0	4	2	1	1
3143	202	0	25-Aug-17	17	1	17	3	2	1	0	4	2	5	1
3144	202	0	19-Jul-17	3	1	17	3	3	1	0	4	2	1	1
3145	202	0	9-Jun-17	2	1	17	3	3	1	0	4	2	1	1
3146	202	0	26-Dec-17	6	1	17	3	2	1	0	4	2	3	1
3147	202	0	17-Mar-17	9	1	17	3	2	1	0	4	2	1	1
3148	202	0	22-Jul-16	0	1	17	3	3	1	0	11	2	3	1
3149	202	0	13-Jun-12	0	1	17	3	3	1	0	11	2	6	1
3150	202	0	16-Aug-11	7	1	17	3	1	1	0	11	2	6	2
3151	202	0	6-Dec-16	20	1	17	3	3	1	0	3	2	1	1
3152	202	0	5-Nov-16	11	1	17	3	2	1	0	3	2	2	1
3153	202	0	17-Jun-16	19	1	17	3	3	1	0	3	2	3	1
3154	202	0	10-Sep-15	21	1	17	3	3	1	0	3	2	1	1
3155	202	0	13-Nov-15	16	1	17	3	3	1	0	3	2	2	1
3156	202	0	25-Nov-15	9	1	17	3	1	1	0	3	2	1	1
3157	202	0	31-Jul-14	3	1	17	3	2	1	0	3	2	2	1
3158	202	0	1-Sep-14	2	1	17	3	1	1	0	3	2	3	1
3159	202	0	5-Oct-14	4	1	17	3	3	1	0	3	2	1	1
3160	202	0	5-Dec-14	17	1	17	3	3	1	0	3	2	3	1
3161	202	0	4-May-14	0	1	17	3	1	1	0	3	2	4	1
3162	202	0	30-Sep-14	23	1	17	3	2	1	0	3	2	4	1
3163	202	0	30-Dec-13	4	1	17	3	1	1	0	3	2	2	1
3164	202	0	21-Sep-13	16	1	17	3	1	1	0	3	2	2	2
3165	202	0	12-Jul-12	16	1	17	3	3	1	0	3	2	1	1
3166	202	0	23-Apr-12	18	1	17	3	1	1	0	3	2	1	2
3167	202	0	9-Aug-12	3	1	17	3	1	1	0	3	2	1	1
3168	202	0	26-May-12	18	1	17	3	1	1	0	3	2	1	2
3169	202	0	1-Feb-10	18	1	17	3	1	1	0	3	2	2	2
3170	202	0	11-Nov-08	17	1	17	3	3	1	0	3	2	1	2
3171	202	0	14-Jun-08	7	1	17	3	3	1	0	3	2	2	1
3172	202	0	11-Jul-08	5	1	17	3	3	1	0	3	2	2	2
3173	202	0	15-Jan-07	20	1	17	3	3	1	0	3	2	2	1
3174	202	0	24-Aug-07	23	1	17	3	3	1	0	3	2	4	1
3175	202	0	20-Feb-07	22	1	17	3	3	1	0	3	2	7	1
3176	202	0	27-Aug-07	9	1	17	3	1	1	0	3	2	1	1
3177	202	0	7-Dec-06	23	1	17	3	3	1	0	3	2	1	2
3178	202	0	24-Dec-06	21	1	17	3	3	1	0	3	2	1	1
3179	202	0	18-Aug-06	17	1	17	3	2	1	0	3	2	1	1
3180	202	0	20-Aug-06	19	1	17	3	3	1	0	3	2	7	2
3181	202	0	8-Mar-17	12	1	17	3	2	1	0	3	2	1	1
3182	202	0	28-Nov-17	17	1	17	3	1	1	0	3	2	7	1
3183	202	0	9-Oct-15	4	1	17	3	2	1	0	6	2	2	1
3184	202	0	19-Mar-12	23	1	17	3	3	1	0	6	2	5	2
3185	202	0	28-Jan-10	8	1	17	3	3	1	0	6	2	2	1
3186	202	0	9-Jan-10	1	1	17	3	3	1	0	6	2	1	2
3187	202	0	25-Apr-10	4	1	17	3	3	1	0	6	2	1	2
3188	202	0	4-May-09	22	1	17	3	1	1	0	6	2	2	2
3189	202	0	4-Jan-08	18	1	17	3	1	1	0	6	2	2	1
3190	202	0	5-Nov-08	0	1	17	3	1	1	0	6	2	2	1
3191	202	0	25-Jul-07	19	1	17	3	3	1	0	6	2	2	1
3192	202	0	29-Aug-07	11	1	17	3	3	1	0	6	2	2	2
3193	202	0	9-Apr-07	14	1	17	3	3	1	0	6	2	2	1
3194	202	0	19-Apr-06	19	1	17	3	1	1	0	6	2	2	1
3195	202	0	31-Mar-06	6	1	17	3	3	1	0	6	2	2	1
3196	202	0	23-May-17	7	1	17	3	3	1	0	6	2	2	2
3197	202	0	22-Jun-14	18	2	17	3	3	1	0	1	2	1	1
3198	202	0	2-Aug-15	2	2	17	3	2	1	0	6	2	2	1
3199	202	0	2-Oct-15	5	2	17	3	3	1	0	6	2	3	1
3200	202	0	22-Aug-14	0	2	17	3	1	1	0	6	2	3	1
3201	202	0	17-Sep-07	4	2	17	3	1	1	0	13	2	2	1
3202	202	0	24-Dec-07	17	2	17	3	3	1	0	1	2	3	1
3203	202	0	1-Jan-12	2	2	17	3	2	1	0	4	2	1	2
3204	202	0	24-Jan-06	17	2	17	3	3	1	0	4	2	2	1
3205	202	0	9-Aug-17	21	2	17	3	3	1	0	4	2	1	1
3206	202	0	27-Sep-15	11	2	17	3	3	1	0	11	2	3	1
3207	202	0	7-Oct-14	21	2	17	3	3	1	0	11	2	2	1
3208	202	0	22-Sep-12	22	2	17	3	1	1	0	11	2	3	1
3209	202	0	26-Aug-10	20	2	17	3	3	1	0	11	2	3	1
3210	202	0	15-Jun-06	13	2	17	3	3	1	0	11	2	3	1
3211	202	0	8-May-14	17	2	17	3	1	1	0	3	2	4	1
3212	202	0	13-Jul-14	20	2	17	3	3	1	0	3	2	2	2
3213	202	0	25-Sep-13	17	2	17	3	1	1	0	3	2	2	1
3214	202	0	15-Jan-12	4	2	17	3	1	1	0	3	2	1	1
3215	202	0	20-Dec-12	23	2	17	3	3	1	0	3	2	2	2
3216	202	0	9-Oct-11	21	2	17	3	3	1	0	3	2	4	1
3217	202	0	9-Apr-10	21	2	17	3	1	1	0	3	2	1	1

3218	202	0	3-Oct-09	1	2	17	3	1	1	0	3	2	1	1
3219	202	0	10-Jul-08	0	2	17	3	2	1	0	3	2	3	2
3220	202	0	23-Nov-17	0	2	17	3	1	1	0	3	2	1	1
3221	202	0	23-Aug-07	1	2	17	3	3	1	0	6	2	1	1
3222	202	0	18-Aug-06	11	2	17	3	3	1	0	6	2	2	1
3223	202	0	6-Dec-06	10	2	17	3	2	1	0	6	2	2	1
3224	201	1	28-May-13	12	1	17	3	3	1	0	1	2	3	1
3225	201	1	24-Sep-12	15	1	17	3	3	1	0	6	2	3	1
3226	201	1	9-May-15	20	1	17	3	2	1	0	10	2	4	1
3227	201	1	11-Oct-13	19	1	17	3	3	1	0	6	2	2	1
3228	201	1	23-May-15	22	1	17	3	3	1	0	10	2	1	2
3229	101	1	4-Jan-15	18	1	17	3	3	1	0	7	2	1	2
3230	101	1	31-Jan-14	0	1	17	3	2	1	0	7	2	3	4
3231	101	1	21-Jan-13	11	1	17	3	3	1	0	7	2	4	1
3232	101	1	27-Jun-09	16	1	17	3	2	1	0	7	2	1	1
3233	101	1	18-Aug-09	21	1	17	3	2	1	0	7	2	2	1
3234	101	1	17-Sep-08	14	1	17	3	3	1	0	7	2	3	1
3235	101	1	18-Jul-08	10	1	17	3	3	1	0	7	2	2	1
3236	101	1	18-Aug-07	21	1	17	3	3	1	0	7	2	2	1
3237	101	1	2-Aug-10	17	1	17	3	3	1	0	13	2	3	1
3238	101	1	7-Dec-12	9	1	17	3	3	1	0	5	2	2	1
3239	201	1	18-Jul-10	12	1	17	3	2	1	0	1	2	5	1
3240	201	1	1-Oct-10	12	1	17	3	3	1	0	1	2	2	1
3241	201	1	6-Feb-09	23	1	17	3	3	1	0	1	2	1	1
3242	201	1	15-May-15	21	1	17	3	2	1	0	4	2	3	1
3243	101	1	29-Jan-15	13	1	17	3	1	1	0	4	2	2	1
3244	201	1	26-Sep-15	4	1	17	3	2	1	0	4	2	1	2
3245	201	1	8-Aug-15	19	1	17	3	3	1	0	4	2	4	1
3246	201	1	14-Dec-15	21	1	17	3	3	1	0	4	2	3	1
3247	201	1	1-Jun-15	21	1	17	3	3	1	0	4	2	1	1
3248	101	1	17-Apr-14	15	1	17	3	3	1	0	4	2	3	1
3249	201	1	29-Nov-14	19	1	17	3	3	1	0	4	2	4	1
3250	201	1	3-Jun-13	10	1	17	3	3	1	0	4	2	4	1
3251	201	1	8-Sep-13	19	1	17	3	3	1	0	4	2	3	1
3252	201	1	24-Nov-13	21	1	17	3	3	1	0	4	2	2	1
3253	101	1	1-Jun-13	3	1	17	3	3	1	0	4	2	4	1
3254	201	1	10-Aug-12	0	1	17	3	3	1	0	4	2	1	1
3255	101	1	14-Dec-12	21	1	17	3	2	1	0	4	2	3	1
3256	101	1	12-Sep-12	17	1	17	3	1	1	0	4	2	2	1
3257	101	1	6-Oct-12	17	1	17	3	3	1	0	4	2	2	4
3258	101	1	16-Dec-11	15	1	17	3	2	1	0	4	2	2	1
3259	201	1	7-Mar-11	18	1	17	3	3	1	0	4	2	1	1
3260	201	1	5-Jul-11	21	1	17	3	3	1	0	4	2	1	1
3261	101	1	26-Jun-11	12	1	17	3	1	1	0	4	2	3	2
3262	201	1	7-Mar-11	11	1	17	3	3	1	0	4	2	3	1
3263	101	1	30-Sep-10	9	1	17	3	3	1	0	4	2	2	4
3264	101	1	13-Jul-10	14	1	17	3	3	1	0	4	2	2	4
3265	201	1	12-Nov-10	20	1	17	3	2	1	0	4	2	3	1
3266	201	1	1-Jan-10	9	1	17	3	1	1	0	4	2	2	1
3267	201	1	8-Dec-10	19	1	17	3	3	1	0	4	2	2	1
3268	201	1	18-Aug-10	21	1	17	3	3	1	0	4	2	3	1
3269	201	1	9-Dec-10	15	1	17	3	3	1	0	4	2	4	2
3270	201	1	8-Sep-10	20	1	17	3	1	1	0	4	2	3	1
3271	101	1	20-Aug-09	23	1	17	3	3	1	0	4	2	2	2
3272	201	1	29-Sep-08	18	1	17	3	3	1	0	4	2	3	1
3273	201	1	10-Sep-08	12	1	17	3	3	1	0	4	2	1	1
3274	201	1	18-Jul-08	20	1	17	3	3	1	0	4	2	2	1
3275	201	1	13-Oct-07	11	1	17	3	3	1	0	4	2	3	1
3276	101	1	28-Mar-17	0	1	17	3	2	1	0	4	2	2	1
3277	201	1	3-Jul-17	17	1	17	3	3	1	0	4	2	1	1
3278	201	1	29-Aug-17	11	1	17	3	3	1	0	4	2	2	1
3279	201	1	30-Dec-17	18	1	17	3	2	1	0	4	2	2	1
3280	101	1	15-Jul-16	18	1	17	3	1	1	0	11	2	2	1
3281	201	1	12-Jul-13	10	1	17	3	3	1	0	11	2	3	1
3282	201	1	26-Sep-13	21	1	17	3	3	1	0	11	2	5	1
3283	101	1	12-Apr-13	18	1	17	3	3	1	0	11	2	4	1
3284	201	1	20-Jul-13	18	1	17	3	1	1	0	11	2	3	1
3285	101	1	25-Oct-12	20	1	17	3	3	1	0	11	2	2	1
3286	101	1	16-Feb-10	9	1	17	3	1	1	0	11	2	1	1
3287	201	1	11-Dec-09	9	1	17	3	2	1	0	11	2	3	1
3288	101	1	13-Jul-07	22	1	17	3	2	1	0	11	2	3	2
3289	201	1	18-Aug-06	19	1	17	3	1	1	0	11	2	3	1
3290	101	1	10-Dec-16	2	1	17	3	2	1	0	3	2	1	2
3291	201	1	9-Jul-16	13	1	17	3	3	1	0	3	2	1	1
3292	101	1	22-Aug-16	19	1	17	3	2	1	0	3	2	2	2
3293	101	1	29-Nov-15	22	1	17	3	1	1	0	3	2	2	1
3294	101	1	14-Jul-15	5	1	17	3	2	1	0	3	2	2	1
3295	201	1	17-Apr-15	20	1	17	3	2	1	0	3	2	3	1
3296	101	1	5-Apr-14	0	1	17	3	3	1	0	3	2	1	1
3297	201	1	19-Feb-14	16	1	17	3	2	1	0	3	2	2	1
3298	201	1	23-May-14	1	1	17	3	1	1	0	3	2	1	1
3299	201	1	15-Nov-14	0	1	17	3	3	1	0	3	2	1	1
3300	101	1	24-Nov-14	17	1	17	3	1	1	0	3	2	3	1
3301	201	1	30-May-14	18	1	17	3	3	1	0	3	2	3	1
3302	201	1	28-Aug-14	11	1	17	3	3	1	0	3	2	2	1
3303	201	1	12-Feb-14	17	1	17	3	3	1	0	3	2	1	1
3304	201	1	28-Jul-13	16	1	17	3	3	1	0	3	2	3	1
3305	101	1	21-May-13	16	1	17	3	2	1	0	3	2	2	1
3306	201	1	23-Oct-12	10	1	17	3	3	1	0	3	2	2	1
3307	101	1	14-Jan-12	15	1	17	3	3	1	0	3	2	3	1
3308	101	1	28-Sep-12	16	1	17	3	2	1	0	3	2	1	2
3309	101	1	27-Aug-12	17	1	17	3	2	1	0	3	2	2	1
3310	101	1	31-Aug-12	20	1	17	3	2	1	0	3	2	2	1
3311	101	1	25-Feb-12	18	1	17	3	3	1	0	3	2	3	1
3312	101	1	7-Aug-12	17	1	17	3	2	1	0	3	2	3	2
3313	101	1	26-May-12	17	1	17	3	2	1	0	3	2	3	1
3314	101	1	18-Jun-12	10	1	17	3	2	1	0	3	2	3	1
3315	201	1	11-May-12	8	1	17	3	2	1	0	3	2	3	1
3316	201	1	16-Oct-11	22	1	17	3	3	1	0	3	2	1	1
3317	101	1	19-Sep-11	15	1	17	3	2	1	0	3	2	3	1
3318	101	1	9-Jun-11	20	1	17	3	3	1	0	3	2	1	1
3319	201	1	11-Jul-11	13	1	17	3	3	1	0	3	2	3	1
3320	201	1	6-Jun-11	19	1	17	3	3	1	0	3	2	2	1
3321	101	1	15-Apr-10	10	1	17	3	3	1	0	3	2	3	1
3322	201	1	19-Aug-10	13	1	17	3	3	1	0	3	2	3	1
3323	101	1	9-Sep-10	1	1	17	3	2	1	0	3	2	1	1
3324	101	1	1-Sep-10	7	1	17	3	3	1	0	3	2	3	1
3325	101	1	22-Jul-10	20	1	17	3	1	1	0	3	2	1	1
3326	101	1	20-Aug-10	20	1	17	3	3	1	0	3	2	2	4
3327	101	1	15-Jun-10	18	1	17	3	3	1	0	3	2	3	1
3328	101	1	19-Mar-10	8	1	17	3	3	1	0	3	2	3	1
3329	201	1	29-Jan-09	18	1	17	3	3	1	0	3	2	3	1
3330	101	1	10-Mar-09	18	1	17	3	3	1	0	3	2	2	1
3331	101	1	31-Dec-09	10	1	17	3	2	1	0	3	2	2	2
3332	101	1	27-Jan-09	20	1	17	3	3	1	0	3	2	3	4
3333	201	1	17-Feb-09	20	1	17	3	3	1	0	3	2	3	1
3334	201	1	2-Aug-09	20	1	17	3	3	1	0	3	2	2	1
3335	201	1	3-Oct-09	22	1	17	3	1	1	0	3	2	2	1
3336	201	1	22-Mar-09	9	1	17	3	2	1	0	3	2	2	2
3337	101	1	16-Sep-09	23	1	17	3	2	1	0	3	2	3	1
3338	101	1	18-Oct-08	17	1	17	3	3	1	0	3	2	2	4
3339	201	1	4-Feb-08	19	1	17	3	3	1	0	3	2	4	1
3340	101	1	20-Dec-08	23	1	17	3	1	1	0	3	2	3	2
3341	101	1	28-Oct-08	20	1	17	3	3	1	0	3	2	2	4

3342	101	1	30-Sep-08	10	1	17	3	3	1	0	3	2	4	1
3343	101	1	4-Jul-08	15	1	17	3	3	1	0	3	2	2	1
3344	101	1	27-Dec-08	18	1	17	3	1	1	0	3	2	2	1
3345	201	1	8-Apr-08	14	1	17	3	3	1	0	3	2	3	1
3346	201	1	5-Apr-08	23	1	17	3	3	1	0	3	2	2	1
3347	201	1	15-May-07	9	1	17	3	3	1	0	3	2	1	1
3348	201	1	22-Sep-07	22	1	17	3	3	1	0	3	2	4	2
3349	101	1	22-Jul-07	22	1	17	3	3	1	0	3	2	3	2
3350	101	1	7-Oct-07	22	1	17	3	1	1	0	3	2	2	1
3351	201	1	27-Apr-07	1	1	17	3	3	1	0	3	2	1	1
3352	101	1	8-Jun-06	16	1	17	3	3	1	0	3	2	3	1
3353	201	1	6-May-06	23	1	17	3	3	1	0	3	2	2	1
3354	101	1	8-Jan-06	21	1	17	3	2	1	0	3	2	2	1
3355	101	1	22-Oct-17	1	1	17	3	3	1	0	3	2	1	1
3356	201	1	4-Oct-17	23	1	17	3	2	1	0	3	2	1	1
3357	201	1	4-Oct-17	20	1	17	3	3	1	0	3	2	4	1
3358	101	1	1-May-17	17	1	17	3	1	1	0	3	2	6	1
3359	101	1	6-Nov-16	21	1	17	3	1	1	0	6	2	2	1
3360	101	1	18-Aug-15	19	1	17	3	1	1	0	6	2	5	1
3361	101	1	15-Jun-14	20	1	17	3	2	1	0	6	2	2	4
3362	201	1	28-Aug-13	12	1	17	3	3	1	0	6	2	2	1
3363	101	1	3-Nov-12	12	1	17	3	3	1	0	6	2	2	1
3364	101	1	6-Sep-12	4	1	17	3	2	1	0	6	2	2	1
3365	101	1	17-Dec-12	17	1	17	3	2	1	0	6	2	2	1
3366	101	1	10-Oct-12	17	1	17	3	3	1	0	6	2	2	1
3367	101	1	16-Dec-11	3	1	17	3	3	1	0	6	2	2	4
3368	101	1	26-May-10	16	1	17	3	3	1	0	6	2	2	1
3369	101	1	23-Jan-10	20	1	17	3	3	1	0	6	2	2	1
3370	101	1	25-Nov-10	1	1	17	3	2	1	0	6	2	2	1
3371	201	1	17-Dec-09	7	1	17	3	3	1	0	6	2	2	1
3372	101	1	2-Nov-09	13	1	17	3	1	1	0	6	2	2	1
3373	201	1	31-Oct-09	14	1	17	3	1	1	0	6	2	2	1
3374	201	1	22-Apr-09	14	1	17	3	3	1	0	6	2	2	1
3375	101	1	5-Oct-08	2	1	17	3	1	1	0	6	2	2	1
3376	101	1	9-Nov-08	21	1	17	3	3	1	0	6	2	2	1
3377	201	1	17-Sep-08	14	1	17	3	1	1	0	6	2	2	1
3378	201	1	5-Jun-07	16	1	17	3	2	1	0	6	2	2	1
3379	101	1	13-Oct-07	1	1	17	3	3	1	0	6	2	2	1
3380	201	1	29-Aug-06	21	1	17	3	2	1	0	6	2	2	1
3381	101	1	23-Aug-17	15	1	17	3	2	1	0	6	2	3	1
3382	201	1	20-Jan-06	10	2	17	3	3	1	0	10	2	7	2
3383	201	1	24-Dec-10	5	2	17	3	3	1	0	1	2	3	1
3384	201	1	6-Sep-06	16	2	17	3	2	1	0	1	2	2	1
3385	201	1	18-Jan-14	21	2	17	3	3	1	0	4	2	5	1
3386	201	1	6-Jan-13	11	2	17	3	3	1	0	4	2	2	1
3387	201	1	11-Apr-13	9	2	17	3	3	1	0	4	2	3	1
3388	201	1	6-Oct-13	23	2	17	3	3	1	0	4	2	2	2
3389	201	1	7-Jun-11	3	2	17	3	3	1	0	4	2	1	1
3390	101	1	24-Dec-09	15	2	17	3	3	1	0	4	2	2	1
3391	201	1	30-Sep-09	14	2	17	3	3	1	0	4	2	2	1
3392	101	1	26-Jan-17	14	2	17	3	1	1	0	4	2	2	1
3393	201	1	24-Nov-13	9	2	17	3	3	1	0	11	2	2	1
3394	101	1	26-Jul-12	23	2	17	3	3	1	0	11	2	3	2
3395	101	1	24-Jul-11	18	2	17	3	3	1	0	11	2	2	1
3396	101	1	28-Nov-11	19	2	17	3	1	1	0	11	2	3	1
3397	201	1	27-Mar-09	10	2	17	3	3	1	0	11	2	3	1
3398	201	1	10-Jun-08	10	2	17	3	1	1	0	11	2	3	1
3399	201	1	15-Aug-17	20	2	17	3	2	1	0	11	2	3	1
3400	101	1	16-Aug-15	21	2	17	3	2	1	0	3	2	3	1
3401	101	1	25-Mar-15	17	2	17	3	2	1	0	3	2	2	1
3402	101	1	8-Dec-15	20	2	17	3	3	1	0	3	2	2	2
3403	101	1	12-Dec-15	0	2	17	3	2	1	0	3	2	1	1
3404	201	1	17-May-15	13	2	17	3	3	1	0	3	2	3	1
3405	201	1	7-Aug-15	16	2	17	3	3	1	0	3	2	2	1
3406	201	1	2-Jan-14	22	2	17	3	2	1	0	3	2	3	1
3407	201	1	8-Nov-14	21	2	17	3	3	1	0	3	2	3	1
3408	101	1	11-Jun-13	11	2	17	3	2	1	0	3	2	3	1
3409	101	1	29-Aug-13	11	2	17	3	3	1	0	3	2	2	1
3410	101	1	14-Mar-13	21	2	17	3	3	1	0	3	2	2	1
3411	101	1	18-Jun-12	17	2	17	3	2	1	0	3	2	3	1
3412	101	1	26-Aug-12	15	2	17	3	3	1	0	3	2	3	1
3413	101	1	9-Apr-12	23	2	17	3	1	1	0	3	2	1	1
3414	101	1	7-Oct-12	0	2	17	3	3	1	0	3	2	1	1
3415	101	1	25-Dec-11	1	2	17	3	3	1	0	3	2	1	2
3416	201	1	31-Jan-11	21	2	17	3	2	1	0	3	2	1	1
3417	101	1	6-Aug-11	21	2	17	3	1	1	0	3	2	1	2
3418	101	1	5-Jul-10	0	2	17	3	3	1	0	3	2	3	2
3419	101	1	23-Mar-09	10	2	17	3	3	1	0	3	2	2	4
3420	201	1	12-Apr-09	18	2	17	3	3	1	0	3	2	3	1
3421	201	1	8-May-09	21	2	17	3	3	1	0	3	2	4	1
3422	201	1	3-Jul-09	17	2	17	3	2	1	0	3	2	1	1
3423	101	1	16-Jan-09	20	2	17	3	3	1	0	3	2	2	1
3424	201	1	27-Mar-07	15	2	17	3	3	1	0	3	2	4	1
3425	101	1	1-Jun-07	22	2	17	3	3	1	0	3	2	3	1
3426	201	1	9-Jul-07	23	2	17	3	3	1	0	3	2	2	2
3427	201	1	10-Mar-06	20	2	17	3	3	1	0	3	2	2	1
3428	101	1	19-Feb-06	21	2	17	3	3	1	0	3	2	3	1
3429	101	1	18-Jul-11	2	2	17	3	2	1	0	6	2	2	1
3430	101	1	4-Mar-11	16	2	17	3	3	1	0	6	2	3	1
3431	101	1	14-May-10	2	2	17	3	3	1	0	6	2	2	1
3432	101	1	24-Feb-09	21	2	17	3	2	1	0	6	2	2	1
3433	201	1	30-Jan-06	1	2	17	3	3	1	0	6	2	3	1
3434	202	0	16-Jul-17	19	1	15	3	2	3	1	6	1	3	1
3435	202	0	7-Nov-06	8	1	16	3	3	3	1	4	1	1	2
3436	202	0	17-Feb-16	16	2	16	3	1	3	1	11	1	2	1
3437	202	0	29-Apr-09	22	2	16	3	3	3	1	3	1	3	1
3438	101	1	27-Mar-10	23	1	16	3	3	3	1	6	1	5	1
3439	101	1	8-Sep-10	2	2	16	3	3	3	1	4	1	5	2
3440	202	0	24-Oct-10	17	1	17	3	1	3	1	4	1	1	1
3441	202	0	11-May-08	20	1	17	3	3	3	1	11	1	3	1
3442	202	0	16-Feb-16	6	2	17	3	3	3	1	4	1	1	1
3443	202	0	14-May-14	1	2	17	3	3	3	1	4	1	1	2
3444	202	0	22-Sep-12	23	2	17	3	3	3	1	11	1	4	1
3445	202	0	27-Sep-15	2	2	17	3	3	3	1	6	1	2	2
3446	201	1	19-Sep-17	20	1	17	3	3	3	1	6	1	2	2
3447	101	1	31-Jul-12	1	1	17	3	3	3	1	4	1	1	1
3448	101	1	21-Apr-12	23	1	17	3	3	3	1	4	1	1	2
3449	101	1	3-Aug-10	23	1	17	3	2	3	1	4	1	5	1
3450	101	1	7-Jan-10	21	1	17	3	3	3	1	11	1	2	1
3451	101	1	8-Jul-10	23	1	17	3	3	3	1	11	1	2	1
3452	101	1	15-Dec-09	18	1	17	3	2	3	1	11	1	2	1
3453	101	1	4-Sep-10	23	1	17	3	3	3	1	6	1	2	4
3454	101	1	23-Sep-07	0	1	17	3	1	3	1	6	1	2	2
3455	201	1	28-Jun-08	2	2	17	3	3	3	1	10	1	7	2
3456	101	1	1-Apr-08	0	2	17	3	3	3	1	4	1	1	1
3457	101	1	20-Jun-09	19	2	17	3	1	3	1	11	1	2	1
3458	101	1	21-Feb-08	23	2	17	3	3	3	1	3	1	1	1
3459	201	1	2-Mar-07	8	2	17	3	3	3	1	6	1	2	1
3460	101	1	20-Sep-11	8	1	14	3	3	2	1	11	1	2	1
3461	202	0	6-May-06	1	2	16	3	3	2	1	3	1	6	2
3462	202	0	2-Sep-10	1	2	16	3	3	2	1	6	1	2	1
3463	202	0	13-Oct-06	4	2	16	3	3	2	1	6	1	2	1
3464	201	1	20-May-11	20	1	16	3	2	2	1	6	1	2	2
3465	202	0	24-Sep-14	2	2	17	3	2	2	1	6	1	2	1

3466	202	0	3-Sep-12	22	2	17	3	3	2	1	6	1	2	2
3467	202	0	15-May-10	16	2	17	3	3	2	1	6	1	2	1
3468	202	0	2-Mar-08	1	2	17	3	1	2	1	6	1	2	1
3469	202	0	7-Dec-06	0	2	17	3	3	2	1	6	1	2	1
3470	202	0	4-Nov-06	1	2	17	3	2	2	1	6	1	2	1
3471	101	1	13-Feb-09	23	1	17	3	1	2	1	6	1	2	2
3472	101	1	2-Apr-06	0	1	17	3	3	2	1	6	1	2	2
3473	201	1	23-May-07	23	2	17	3	3	2	1	6	1	2	1
3474	101	1	16-Jan-07	23	2	17	3	1	2	1	6	1	2	1
3475	202	0	30-Jul-13	22	1	14	3	3	1	0	4	1	3	1
3476	202	0	26-Feb-17	10	2	14	3	3	1	0	10	1	1	2
3477	202	0	4-Apr-17	21	2	14	3	3	1	0	11	1	2	1
3478	201	1	31-Aug-10	8	1	14	3	3	1	0	11	1	3	1
3479	201	1	13-Jul-10	9	2	14	3	3	1	0	7	1	3	1
3480	202	0	11-Aug-16	15	1	15	3	1	1	0	6	1	2	1
3481	202	0	9-Sep-16	22	1	15	3	1	1	0	3	1	4	1
3482	202	0	9-Aug-11	18	1	15	3	1	1	0	6	1	2	2
3483	202	0	26-Jan-10	19	2	15	3	1	1	0	10	1	7	2
3484	201	1	21-Sep-12	20	1	15	3	3	1	0	4	1	2	1
3485	101	1	14-Mar-10	14	1	15	3	3	1	0	7	1	3	1
3486	101	1	24-Oct-17	16	1	15	3	1	1	0	7	1	2	1
3487	101	1	15-Jun-12	11	1	15	3	2	1	0	4	1	1	1
3488	201	1	18-Aug-10	20	1	15	3	3	1	0	4	1	2	1
3489	101	1	3-Jul-14	15	1	15	3	1	1	0	11	1	3	1
3490	101	1	26-Jul-14	23	1	15	3	1	1	0	6	1	2	1
3491	201	1	11-Jan-15	21	2	15	3	3	1	0	10	1	5	2
3492	101	1	4-Sep-14	23	2	15	3	3	1	0	6	1	2	1
3493	202	0	21-Oct-11	19	1	16	3	1	1	0	4	1	3	1
3494	202	0	22-Sep-08	19	1	16	3	1	1	0	4	1	1	1
3495	202	0	19-May-13	22	1	16	3	3	1	0	11	1	2	1
3496	202	0	1-Jan-12	17	1	16	3	1	1	0	11	1	3	1
3497	202	0	10-Jul-14	11	1	16	3	3	1	0	3	1	1	1
3498	202	0	1-Dec-08	20	1	16	3	3	1	0	3	1	2	2
3499	202	0	5-Oct-06	18	1	16	3	1	1	0	3	1	4	1
3500	202	0	31-Jan-16	17	1	16	3	2	1	0	6	1	2	1
3501	202	0	6-Sep-08	5	1	16	3	3	1	0	6	1	2	1
3502	202	0	12-Jul-17	9	1	16	3	1	1	0	6	1	3	1
3503	202	0	19-Jan-12	12	2	16	3	1	1	0	10	1	7	1
3504	202	0	14-Jan-11	23	2	16	3	1	1	0	10	1	7	1
3505	202	0	16-Dec-08	0	2	16	3	3	1	0	10	1	7	1
3506	202	0	3-Mar-06	0	2	16	3	3	1	0	10	1	7	1
3507	202	0	15-Sep-14	18	2	16	3	3	1	0	7	1	2	1
3508	202	0	5-Nov-10	18	2	16	3	1	1	0	3	1	4	2
3509	202	0	5-May-11	4	2	16	3	1	1	0	6	1	2	2
3510	202	0	23-Feb-17	14	2	16	3	1	1	0	13	1	1	1
3511	201	1	10-May-15	15	1	16	3	1	1	0	6	1	2	1
3512	101	1	5-Apr-13	4	1	16	3	2	1	0	7	1	3	1
3513	101	1	30-Apr-13	22	1	16	3	3	1	0	7	1	3	2
3514	101	1	1-Jan-10	8	1	16	3	1	1	0	7	1	3	1
3515	101	1	7-Sep-12	23	1	16	3	2	1	0	4	1	2	1
3516	101	1	13-May-09	23	1	16	3	1	1	0	4	1	1	1
3517	201	1	30-Jul-08	19	1	16	3	3	1	0	4	1	3	1
3518	201	1	17-Oct-14	12	1	16	3	3	1	0	11	1	2	1
3519	101	1	8-Jan-13	20	1	16	3	2	1	0	11	1	2	1
3520	101	1	31-Oct-12	15	1	16	3	2	1	0	11	1	2	1
3521	201	1	25-Jul-11	11	1	16	3	2	1	0	11	1	2	1
3522	101	1	31-Jan-11	20	1	16	3	3	1	0	11	1	2	1
3523	201	1	5-Nov-11	9	1	16	3	3	1	0	11	1	3	1
3524	201	1	28-Oct-10	14	1	16	3	3	1	0	11	1	2	1
3525	101	1	21-Jan-10	13	1	16	3	3	1	0	11	1	3	1
3526	101	1	10-Sep-09	6	1	16	3	3	1	0	11	1	2	1
3527	201	1	4-Apr-08	9	1	16	3	2	1	0	11	1	1	1
3528	201	1	4-Oct-06	20	1	16	3	3	1	0	11	1	2	1
3529	101	1	14-Mar-17	18	1	16	3	1	1	0	11	1	2	1
3530	101	1	3-Nov-12	20	1	16	3	2	1	0	3	1	1	1
3531	201	1	26-Nov-10	0	1	16	3	3	1	0	3	1	3	2
3532	101	1	21-Sep-12	1	1	16	3	2	1	0	6	1	3	1
3533	101	1	1-Aug-11	9	1	16	3	1	1	0	6	1	2	1
3534	101	1	28-Jul-09	17	1	16	3	1	1	0	6	1	2	1
3535	101	1	25-Mar-17	2	1	16	3	2	1	0	6	1	2	1
3536	101	1	14-Apr-17	1	1	16	3	2	1	0	6	1	2	2
3537	201	1	2-Mar-12	15	2	16	3	3	1	0	6	1	7	1
3538	201	1	28-Jul-11	9	2	16	3	3	1	0	7	1	2	1
3539	201	1	22-Aug-08	10	2	16	3	3	1	0	13	1	2	1
3540	101	1	2-Nov-16	11	2	16	3	1	1	0	11	1	2	1
3541	201	1	7-Mar-15	17	2	16	3	3	1	0	11	1	2	1
3542	201	1	29-Mar-14	12	2	16	3	3	1	0	11	1	3	1
3543	101	1	31-Dec-14	21	2	16	3	1	1	0	11	1	2	1
3544	201	1	8-May-11	13	2	16	3	3	1	0	11	1	3	1
3545	101	1	20-Jan-13	23	2	16	3	2	1	0	3	1	5	1
3546	101	1	18-Aug-13	8	2	16	3	2	1	0	3	1	3	1
3547	101	1	3-Mar-12	22	2	16	3	1	1	0	3	1	3	1
3548	101	1	24-Jan-10	17	2	16	3	3	1	0	3	1	1	1
3549	201	1	4-Mar-09	19	2	16	3	3	1	0	3	1	1	1
3550	201	1	20-Oct-06	18	2	16	3	3	1	0	3	1	1	2
3551	201	1	10-Nov-06	20	2	16	3	2	1	0	3	1	1	1
3552	201	1	30-Aug-08	23	2	16	3	3	1	0	6	1	3	1
3553	202	0	27-Feb-16	10	1	17	3	3	1	0	10	1	1	2
3554	202	0	20-Apr-13	3	1	17	3	2	1	0	10	1	1	1
3555	202	0	17-May-14	7	1	17	3	3	1	0	4	1	2	1
3556	202	0	16-Jan-12	0	1	17	3	3	1	0	6	1	2	1
3557	202	0	8-Sep-14	12	1	17	3	1	1	0	6	1	2	1
3558	202	0	25-Feb-12	22	1	17	3	1	1	0	10	1	7	1
3559	202	0	21-Dec-09	20	1	17	3	1	1	0	10	1	7	2
3560	202	0	24-May-07	11	1	17	3	1	1	0	10	1	7	2
3561	202	0	16-Feb-07	1	1	17	3	1	1	0	10	1	7	2
3562	202	0	20-Oct-14	20	1	17	3	1	1	0	7	1	2	1
3563	202	0	14-Oct-10	22	1	17	3	1	1	0	7	1	3	1
3564	202	0	10-Sep-10	12	1	17	3	1	1	0	7	1	2	2
3565	202	0	19-Nov-08	1	1	17	3	1	1	0	7	1	2	2
3566	202	0	17-Nov-17	0	1	17	3	2	1	0	7	1	4	1
3567	202	0	21-Oct-07	18	1	17	3	3	1	0	13	1	1	1
3568	202	0	24-Sep-07	18	1	17	3	1	1	0	13	1	1	1
3569	202	0	23-Sep-11	19	1	17	3	1	1	0	1	1	7	2
3570	202	0	11-Aug-09	5	1	17	3	1	1	0	1	1	5	2
3571	202	0	18-Jun-14	5	1	17	3	2	1	0	4	1	1	1
3572	202	0	26-Sep-13	20	1	17	3	2	1	0	4	1	1	1
3573	202	0	2-Aug-12	2	1	17	3	1	1	0	4	1	2	1
3574	202	0	28-May-17	0	1	17	3	3	1	0	4	1	4	1
3575	202	0	22-Nov-14	17	1	17	3	3	1	0	11	1	3	1
3576	202	0	24-Mar-14	18	1	17	3	2	1	0	11	1	3	2
3577	202	0	26-Jan-12	13	1	17	3	3	1	0	11	1	2	1
3578	202	0	28-Aug-16	13	1	17	3	2	1	0	3	1	1	1
3579	202	0	17-May-14	13	1	17	3	1	1	0	3	1	2	1
3580	202	0	25-May-14	12	1	17	3	1	1	0	3	1	3	1
3581	202	0	28-Nov-13	16	1	17	3	2	1	0	3	1	2	1
3582	202	0	28-May-13	3	1	17	3	2	1	0	3	1	1	1
3583	202	0	2-Oct-10	21	1	17	3	1	1	0	3	1	6	2
3584	202	0	29-Jul-10	20	1	17	3	3	1	0	3	1	4	1
3585	202	0	17-Jan-10	9	1	17	3	1	1	0	3	1	1	1
3586	202	0	11-Dec-06	1	1	17	3	3	1	0	3	1	2	1
3587	202	0	25-Mar-17	12	1	17	3	1	1	0	3	1	3	1
3588	202	0	18-Feb-17	10	1	17	3	1	1	0	3	1	1	1
3589	202	0	18-Mar-13	12	1	17	3	3	1	0	6	1	3	1

3590	202	0	24-Nov-12	16	1	17	3	1	1	0	6	1	2	1
3591	202	0	29-Jul-12	2	1	17	3	3	1	0	6	1	2	2
3592	202	0	17-Dec-11	9	1	17	3	3	1	0	6	1	3	1
3593	202	0	5-Jun-10	13	1	17	3	1	1	0	6	1	2	2
3594	202	0	5-Jun-10	13	1	17	3	3	1	0	6	1	2	1
3595	202	0	19-Jul-08	0	1	17	3	3	1	0	6	1	7	2
3596	202	0	15-Aug-07	2	1	17	3	1	1	0	6	1	3	1
3597	202	0	18-Aug-06	12	1	17	3	1	1	0	6	1	2	1
3598	202	0	19-Nov-14	1	2	17	3	3	1	0	6	1	2	1
3599	202	0	12-Jul-15	23	2	17	3	2	1	0	6	1	4	2
3600	202	0	20-Aug-15	23	2	17	3	3	1	0	6	1	2	1
3601	202	0	7-Jan-12	19	2	17	3	3	1	0	10	1	7	2
3602	202	0	11-Feb-16	20	2	17	3	3	1	0	10	1	6	2
3603	202	0	10-Jun-13	13	2	17	3	3	1	0	6	1	2	1
3604	202	0	24-Apr-14	19	2	17	3	3	1	0	6	1	2	1
3605	202	0	19-Oct-07	8	2	17	3	3	1	0	10	1	7	1
3606	202	0	7-Nov-15	22	2	17	3	3	1	0	7	1	2	1
3607	202	0	12-Jun-14	17	2	17	3	1	1	0	7	1	3	1
3608	202	0	18-Jun-14	2	2	17	3	1	1	0	7	1	2	1
3609	202	0	11-May-14	15	2	17	3	1	1	0	4	1	2	1
3610	202	0	5-Jun-12	1	2	17	3	3	1	0	4	1	2	1
3611	202	0	27-Sep-15	17	2	17	3	3	1	0	11	1	3	1
3612	202	0	1-Jan-16	20	2	17	3	1	1	0	3	1	1	1
3613	202	0	16-Oct-13	17	2	17	3	1	1	0	3	1	4	1
3614	202	0	11-Feb-10	18	2	17	3	3	1	0	3	1	6	2
3615	202	0	20-Sep-06	8	2	17	3	3	1	0	3	1	2	2
3616	202	0	13-Nov-17	21	2	17	3	3	1	0	3	1	1	1
3617	202	0	31-Jul-11	22	2	17	3	3	1	0	6	1	2	2
3618	202	0	14-Jun-06	4	2	17	3	1	1	0	6	1	2	1
3619	201	1	8-Nov-13	0	1	17	3	3	1	0	3	1	3	1
3620	201	1	7-Jul-13	17	1	17	3	2	1	0	6	1	2	1
3621	201	1	20-Jun-13	0	1	17	3	2	1	0	6	1	2	1
3622	201	1	8-Oct-12	19	1	17	3	2	1	0	6	1	2	1
3623	101	1	12-Aug-15	23	1	17	3	1	1	0	7	1	1	1
3624	101	1	26-Aug-13	23	1	17	3	3	1	0	6	1	2	1
3625	201	1	17-Nov-13	9	1	17	3	3	1	0	3	1	1	1
3626	201	1	12-Jan-15	10	1	17	3	3	1	0	6	1	2	1
3627	201	1	7-Nov-12	15	1	17	3	2	1	0	6	1	3	1
3628	201	1	7-Jan-09	23	1	17	3	3	1	0	10	1	7	2
3629	101	1	9-Feb-15	19	1	17	3	2	1	0	7	1	5	2
3630	201	1	21-Apr-14	14	1	17	3	3	1	0	7	1	4	1
3631	101	1	10-Aug-13	10	1	17	3	3	1	0	7	1	2	1
3632	101	1	20-Jan-11	3	1	17	3	3	1	0	7	1	5	2
3633	201	1	13-Jul-10	10	1	17	3	3	1	0	7	1	3	1
3634	101	1	21-Aug-17	12	1	17	3	3	1	0	7	1	3	1
3635	101	1	28-Sep-17	20	1	17	3	1	1	0	13	1	3	1
3636	101	1	23-Jun-08	22	1	17	3	3	1	0	1	1	2	1
3637	101	1	9-Feb-12	20	1	17	3	2	1	0	4	1	2	1
3638	101	1	6-Feb-10	1	1	17	3	3	1	0	4	1	1	1
3639	201	1	5-Jul-15	22	1	17	3	3	1	0	11	1	1	1
3640	101	1	29-Sep-14	19	1	17	3	2	1	0	11	1	3	1
3641	101	1	3-Apr-14	6	1	17	3	2	1	0	11	1	3	1
3642	101	1	22-Oct-13	16	1	17	3	3	1	0	11	1	2	1
3643	101	1	19-May-12	10	1	17	3	3	1	0	11	1	3	1
3644	101	1	22-Oct-12	11	1	17	3	3	1	0	11	1	2	1
3645	101	1	10-Nov-12	12	1	17	3	2	1	0	11	1	2	1
3646	201	1	12-Jun-12	22	1	17	3	3	1	0	11	1	3	1
3647	101	1	18-Oct-12	11	1	17	3	2	1	0	11	1	5	1
3648	101	1	22-Nov-12	1	1	17	3	2	1	0	11	1	2	1
3649	101	1	10-Sep-12	20	1	17	3	1	1	0	11	1	3	1
3650	201	1	12-May-11	14	1	17	3	3	1	0	11	1	2	1
3651	101	1	5-Sep-10	10	1	17	3	3	1	0	11	1	3	1
3652	201	1	9-Oct-09	23	1	17	3	2	1	0	11	1	2	1
3653	201	1	25-Jul-07	20	1	17	3	1	1	0	11	1	3	1
3654	101	1	13-Sep-17	12	1	17	3	2	1	0	11	1	2	1
3655	101	1	22-Jun-17	20	1	17	3	2	1	0	11	1	2	1
3656	201	1	23-Sep-17	19	1	17	3	2	1	0	11	1	3	1
3657	101	1	22-Jan-17	10	1	17	3	1	1	0	11	1	2	1
3658	201	1	23-Mar-14	20	1	17	3	2	1	0	3	1	5	1
3659	101	1	5-Mar-14	23	1	17	3	3	1	0	3	1	3	1
3660	201	1	19-Dec-13	20	1	17	3	2	1	0	3	1	1	1
3661	101	1	4-May-13	10	1	17	3	3	1	0	3	1	3	1
3662	201	1	22-Nov-12	13	1	17	3	3	1	0	3	1	2	1
3663	201	1	23-Mar-12	17	1	17	3	3	1	0	3	1	3	1
3664	101	1	7-Aug-12	20	1	17	3	3	1	0	3	1	1	4
3665	101	1	23-Jul-12	10	1	17	3	3	1	0	3	1	1	2
3666	101	1	20-Aug-10	19	1	17	3	3	1	0	3	1	4	1
3667	101	1	7-Aug-10	12	1	17	3	2	1	0	3	1	3	4
3668	201	1	26-Sep-10	22	1	17	3	3	1	0	3	1	4	1
3669	201	1	12-Aug-09	2	1	17	3	2	1	0	3	1	3	2
3670	201	1	11-Oct-08	17	1	17	3	3	1	0	3	1	2	1
3671	201	1	31-Jul-08	21	1	17	3	2	1	0	3	1	1	2
3672	101	1	26-Mar-07	22	1	17	3	3	1	0	3	1	1	2
3673	201	1	29-Oct-17	1	1	17	3	2	1	0	3	1	4	1
3674	101	1	16-Jul-16	23	1	17	3	2	1	0	6	1	1	1
3675	101	1	14-Jul-15	12	1	17	3	3	1	0	6	1	2	1
3676	101	1	11-May-14	13	1	17	3	3	1	0	6	1	2	4
3677	201	1	17-Apr-13	15	1	17	3	2	1	0	6	1	1	1
3678	101	1	17-Jun-13	23	1	17	3	2	1	0	6	1	3	1
3679	101	1	12-Dec-12	19	1	17	3	3	1	0	6	1	2	1
3680	101	1	18-Aug-12	16	1	17	3	2	1	0	6	1	2	1
3681	101	1	17-Nov-11	19	1	17	3	3	1	0	6	1	2	1
3682	101	1	8-Dec-11	7	1	17	3	2	1	0	6	1	2	1
3683	101	1	3-Apr-11	20	1	17	3	3	1	0	6	1	2	4
3684	101	1	28-Jun-11	1	1	17	3	1	1	0	6	1	3	1
3685	101	1	29-Aug-10	4	1	17	3	1	1	0	6	1	2	1
3686	201	1	27-May-10	21	1	17	3	2	1	0	6	1	2	1
3687	101	1	2-Dec-09	20	1	17	3	2	1	0	6	1	2	4
3688	101	1	21-Jun-08	2	1	17	3	2	1	0	6	1	3	4
3689	201	1	23-Aug-08	12	1	17	3	3	1	0	6	1	2	1
3690	201	1	6-Sep-07	11	1	17	3	1	1	0	6	1	2	1
3691	101	1	22-Oct-07	20	1	17	3	2	1	0	6	1	3	1
3692	201	1	17-Nov-17	19	1	17	3	2	1	0	6	1	2	1
3693	201	1	23-Jan-12	11	2	17	3	2	1	0	13	1	3	2
3694	201	1	12-Jan-13	12	2	17	3	2	1	0	6	1	2	1
3695	201	1	18-Jun-15	21	2	17	3	2	1	0	7	1	3	1
3696	101	1	30-Jan-13	21	2	17	3	3	1	0	7	1	2	1
3697	101	1	14-Aug-07	7	2	17	3	1	1	0	7	1	5	2
3698	101	1	21-Aug-06	19	2	17	3	3	1	0	7	1	2	1
3699	101	1	19-Aug-12	1	2	17	3	2	1	0	4	1	1	1
3700	101	1	5-Jun-11	19	2	17	3	2	1	0	4	1	1	1
3701	101	1	12-Nov-10	23	2	17	3	2	1	0	4	1	2	1
3702	201	1	8-Oct-07	6	2	17	3	3	1	0	4	1	1	1
3703	201	1	17-Jul-15	11	2	17	3	2	1	0	11	1	2	1
3704	201	1	26-Sep-15	14	2	17	3	3	1	0	11	1	2	1
3705	101	1	13-Nov-14	4	2	17	3	2	1	0	11	1	2	1
3706	101	1	17-Apr-14	3	2	17	3	3	1	0	11	1	2	1
3707	201	1	16-Feb-13	4	2	17	3	3	1	0	11	1	1	1
3708	101	1	27-Aug-13	9	2	17	3	3	1	0	11	1	3	1
3709	101	1	22-Oct-12	17	2	17	3	1	1	0	11	1	2	1
3710	201	1	5-Dec-12	7	2	17	3	3	1	0	11	1	2	1
3711	101	1	10-Jul-11	17	2	17	3	3	1	0	11	1	2	1
3712	201	1	27-Jun-11	1	2	17	3	3	1	0	11	1	3	1
3713	201	1	16-Nov-11	20	2	17	3	3	1	0	11	1	3	1

3714	101	1	22-Jul-10	10	2	17	3	3	1	0	11	1	1	1
3715	201	1	22-Apr-10	13	2	17	3	3	1	0	11	1	3	1
3716	101	1	17-Feb-10	6	2	17	3	1	1	0	11	1	2	1
3717	201	1	14-Mar-09	23	2	17	3	3	1	0	11	1	1	1
3718	201	1	13-Apr-09	14	2	17	3	3	1	0	11	1	3	1
3719	201	1	6-Sep-07	5	2	17	3	2	1	0	11	1	3	1
3720	201	1	21-Jul-07	20	2	17	3	3	1	0	11	1	3	1
3721	201	1	1-Apr-07	8	2	17	3	3	1	0	11	1	2	1
3722	101	1	8-Mar-06	10	2	17	3	3	1	0	11	1	3	1
3723	201	1	30-Jun-17	16	2	17	3	3	1	0	11	1	3	1
3724	201	1	2-May-17	11	2	17	3	3	1	0	11	1	3	1
3725	101	1	7-Jun-11	23	2	17	3	2	1	0	3	1	3	2
3726	201	1	1-Mar-09	6	2	17	3	2	1	0	3	1	1	1
3727	201	1	10-Jun-09	19	2	17	3	3	1	0	3	1	3	2
3728	101	1	8-Dec-09	0	2	17	3	2	1	0	3	1	4	2
3729	201	1	31-Jul-08	19	2	17	3	3	1	0	3	1	3	1
3730	101	1	7-Feb-12	15	2	17	3	2	1	0	6	1	2	1
3731	201	1	18-Jul-11	5	2	17	3	1	1	0	6	1	2	1
3732	101	1	30-Dec-10	19	2	17	3	2	1	0	6	1	2	1
3733	101	1	10-Nov-07	23	2	17	3	3	1	0	6	1	4	1
3734	201	1	26-Aug-06	20	2	17	3	3	1	0	6	1	2	1
3735	101	1	16-Jun-17	20	2	17	3	1	1	0	6	1	2	1
3736	202	0	26-Apr-17	9	2	12	2	2	3	1	4	5	5	2
3737	202	0	4-Jul-06	21	1	13	2	1	3	1	4	5	1	1
3738	202	0	2-Jan-07	10	2	13	2	1	3	1	4	5	3	1
3739	202	0	8-Dec-07	1	2	13	2	1	3	1	13	5	7	1
3740	101	1	3-Apr-15	19	1	13	2	1	3	1	4	5	5	1
3741	101	1	10-Nov-12	19	1	13	2	1	3	1	4	5	2	1
3742	101	1	2-Nov-10	19	1	13	2	1	3	1	4	5	5	1
3743	202	0	31-Aug-06	0	1	14	2	1	3	1	10	5	2	1
3744	202	0	30-Jul-11	14	1	14	2	1	3	1	4	5	1	1
3745	202	0	13-Jun-08	17	1	14	2	1	3	1	4	5	6	1
3746	202	0	29-Jul-06	12	1	14	2	1	3	1	4	5	1	1
3747	202	0	16-Sep-06	5	2	14	2	1	3	1	4	5	1	1
3748	101	1	11-Jun-14	19	1	14	2	1	3	1	4	5	5	1
3749	101	1	9-Feb-14	14	1	14	2	1	3	1	4	5	1	1
3750	101	1	14-Mar-11	17	1	14	2	1	3	1	4	5	1	1
3751	101	1	5-Nov-10	17	1	14	2	3	3	1	4	5	1	1
3752	101	1	14-Jul-09	18	2	14	2	3	3	1	4	5	4	1
3753	202	0	21-Jan-06	17	1	15	2	1	3	1	4	5	1	1
3754	202	0	30-Jan-06	19	1	15	2	1	3	1	4	5	1	1
3755	101	1	12-Sep-15	20	1	15	2	1	3	1	4	5	5	1
3756	101	1	25-Nov-14	14	1	15	2	3	3	1	4	5	5	1
3757	101	1	31-Aug-13	23	1	15	2	1	3	1	4	5	5	1
3758	101	1	2-Nov-10	21	1	15	2	1	3	1	4	5	5	1
3759	201	1	21-Jun-10	21	1	15	2	2	3	1	4	5	1	2
3760	101	1	26-Oct-07	20	1	15	2	1	3	1	4	5	1	1
3761	201	1	29-Jul-06	0	1	15	2	1	3	1	6	5	4	2
3762	101	1	9-Nov-15	1	1	15	2	1	3	1	13	5	5	1
3763	101	1	25-Aug-13	16	2	15	2	1	3	1	3	5	1	1
3764	202	0	16-Sep-06	20	1	16	2	1	3	1	4	5	5	1
3765	202	0	9-Feb-13	2	2	16	2	1	3	1	10	5	1	2
3766	202	0	18-Apr-08	17	2	16	2	1	3	1	4	5	6	2
3767	101	1	21-May-15	1	1	16	2	2	3	1	4	5	5	1
3768	101	1	23-Sep-15	19	1	16	2	1	3	1	4	5	3	1
3769	101	1	27-Dec-09	20	1	16	2	1	3	1	4	5	5	1
3770	101	1	25-Aug-10	22	2	16	2	1	3	1	4	5	1	2
3771	101	1	2-Sep-08	17	2	16	2	1	3	1	4	5	5	1
3772	202	0	12-May-11	12	1	17	2	1	3	1	4	5	1	1
3773	202	0	12-Jul-11	22	1	17	2	1	3	1	4	5	1	2
3774	202	0	30-Jan-06	19	1	17	2	1	3	1	4	5	1	1
3775	202	0	23-Jun-06	16	1	17	2	1	3	1	4	5	6	1
3776	202	0	15-Jan-08	2	1	17	2	1	3	1	6	5	1	2
3777	202	0	15-Jul-07	22	2	17	2	2	3	1	6	5	4	2
3778	101	1	3-Feb-12	22	1	17	2	1	3	1	4	5	5	2
3779	101	1	2-Feb-12	23	1	17	2	1	3	1	4	5	1	1
3780	101	1	10-Sep-08	18	1	17	2	1	3	1	4	5	5	1
3781	101	1	20-Oct-10	17	1	14	2	2	2	1	4	5	2	1
3782	101	1	12-Sep-15	12	1	16	2	1	2	1	4	5	2	1
3783	101	1	17-Jan-07	22	1	17	2	1	2	1	6	5	2	1
3784	202	0	7-Jul-06	15	1	10	2	1	1	0	3	5	1	1
3785	101	1	17-Nov-17	19	2	10	2	1	1	0	11	5	2	1
3786	202	0	12-Aug-10	11	1	11	2	1	1	0	11	5	2	2
3787	202	0	27-Oct-16	17	2	11	2	2	1	0	4	5	4	1
3788	101	1	24-Mar-11	8	1	11	2	1	1	0	1	5	1	1
3789	201	1	7-Sep-13	17	1	12	2	2	1	0	4	5	2	1
3790	101	1	23-Nov-15	23	1	12	2	1	1	0	3	5	2	2
3791	101	1	31-Mar-17	10	1	12	2	1	1	0	3	5	3	2
3792	101	1	30-Sep-07	17	1	12	2	1	1	0	6	5	3	1
3793	101	1	1-Nov-17	1	1	12	2	1	1	0	13	5	2	1
3794	201	1	19-Nov-08	14	2	12	2	2	1	0	13	5	3	1
3795	202	0	3-Oct-17	10	1	13	2	1	1	0	6	5	2	1
3796	201	1	9-Feb-12	17	1	13	2	1	1	0	4	5	7	1
3797	101	1	24-Oct-06	19	1	13	2	1	1	0	6	5	1	1
3798	201	1	19-Sep-09	10	1	13	2	3	1	0	13	5	1	1
3799	101	1	11-Oct-17	12	1	13	2	1	1	0	13	5	1	1
3800	101	1	28-Oct-12	12	2	13	2	1	1	0	13	5	3	1
3801	201	1	4-Oct-09	13	2	13	2	2	1	0	13	5	3	1
3802	202	0	22-May-09	18	1	14	2	1	1	0	3	5	1	1
3803	202	0	17-Mar-17	15	1	14	2	1	1	0	3	5	3	1
3804	202	0	8-Jun-07	13	2	14	2	1	1	0	13	5	7	1
3805	202	0	11-Jun-07	9	2	14	2	1	1	0	13	5	7	1
3806	101	1	11-Dec-10	21	1	14	2	1	1	0	4	5	2	1
3807	101	1	29-Jan-16	14	1	14	2	2	1	0	3	5	3	1
3808	201	1	21-May-10	16	2	14	2	3	1	0	3	5	3	1
3809	101	1	24-Dec-14	16	2	14	2	1	1	0	13	5	3	1
3810	202	0	6-May-06	13	1	15	2	1	1	0	10	5	2	1
3811	202	0	7-Aug-07	21	1	15	2	3	1	0	13	5	7	2
3812	202	0	30-Aug-06	1	1	15	2	1	1	0	4	5	1	1
3813	202	0	3-Nov-17	21	1	15	2	1	1	0	4	5	1	1
3814	202	0	21-Jan-12	11	1	15	2	1	1	0	11	5	3	1
3815	202	0	28-Jun-17	15	1	15	2	2	1	0	11	5	5	1
3816	202	0	10-Jul-17	17	1	15	2	2	1	0	3	5	1	1
3817	202	0	1-Oct-09	8	1	15	2	1	1	0	6	5	3	1
3818	101	1	7-Jan-15	19	1	15	2	1	1	0	4	5	1	1
3819	101	1	7-May-06	1	1	15	2	1	1	0	4	5	2	1
3820	101	1	1-Apr-17	20	1	15	2	1	1	0	4	5	2	1
3821	101	1	25-Dec-14	19	1	15	2	1	1	0	3	5	1	1
3822	101	1	19-Sep-12	17	1	15	2	1	1	0	3	5	2	1
3823	101	1	3-Aug-11	21	1	15	2	1	1	0	3	5	3	1
3824	101	1	22-Mar-06	17	1	15	2	1	1	0	3	5	2	1
3825	201	1	7-Aug-17	16	1	15	2	2	1	0	3	5	1	1
3826	101	1	17-Nov-17	19	1	15	2	1	1	0	13	5	5	2
3827	101	1	20-Jun-14	10	2	15	2	3	1	0	3	5	3	1
3828	201	1	5-Aug-08	21	2	15	2	3	1	0	13	5	3	1
3829	202	0	3-Oct-09	8	1	16	2	3	1	0	7	5	2	1
3830	202	0	24-Aug-10	21	1	16	2	1	1	0	11	5	3	2
3831	202	0	3-Sep-08	16	1	16	2	1	1	0	3	5	2	2
3832	202	0	8-Jan-17	18	1	16	2	1	1	0	3	5	3	1
3833	202	0	19-Jul-09	11	1	16	2	1	1	0	6	5	2	2
3834	101	1	28-Jan-06	17	1	16	2	3	1	0	13	5	7	1
3835	101	1	14-Jan-06	18	1	16	2	1	1	0	13	5	7	1
3836	101	1	3-Nov-17	13	1	16	2	1	1	0	4	5	6	1
3837	201	1	9-Aug-12	10	1	16	2	3	1	0	11	5	3	1

3838	101	1	2-May-09	16	1	16	2	2	1	0	11	5	2	1
3839	101	1	1-Dec-17	19	1	16	2	1	1	0	11	5	2	1
3840	101	1	26-Jul-13	2	1	16	2	1	1	0	6	5	1	2
3841	201	1	9-Feb-12	19	1	16	2	2	1	0	13	5	1	1
3842	101	1	12-Dec-07	12	2	16	2	1	1	0	11	5	2	1
3843	202	0	24-Jul-06	18	1	17	2	1	1	0	4	5	1	1
3844	202	0	27-May-17	14	1	17	2	1	1	0	3	5	2	1
3845	202	0	1-Oct-10	0	1	17	2	1	1	0	6	5	1	1
3846	202	0	2-Oct-10	3	1	17	2	1	1	0	13	5	5	2
3847	202	0	24-Dec-09	17	1	17	2	2	1	0	13	5	7	1
3848	202	0	26-Feb-08	10	2	17	2	1	1	0	13	5	7	1
3849	201	1	19-Mar-06	18	1	17	2	1	1	0	13	5	1	1
3850	101	1	7-Jul-15	21	1	17	2	1	1	0	11	5	5	1
3851	101	1	14-Mar-11	16	1	17	2	1	1	0	3	5	2	1
3852	201	1	18-Jun-11	18	1	17	2	1	1	0	3	5	2	1
3853	201	1	25-Apr-06	23	1	17	2	1	1	0	6	5	3	1
3854	201	1	17-Feb-12	9	1	17	2	2	1	0	13	5	2	1
3855	201	1	17-Feb-12	19	1	17	2	2	1	0	13	5	2	1
3856	101	1	14-Oct-17	16	1	17	2	1	1	0	13	5	2	1
3857	101	1	2-Oct-17	19	1	17	2	1	1	0	13	5	1	2
3858	201	1	31-May-11	9	2	17	2	1	1	0	7	5	3	1
3859	101	1	20-Aug-16	18	2	17	2	1	1	0	13	5	2	1
3860	201	1	9-Feb-12	9	2	17	2	1	1	0	13	5	7	1
3861	101	1	8-Apr-15	22	1	12	2	1	3	1	4	4	1	1
3862	101	1	30-Sep-07	20	1	12	2	1	3	1	3	4	5	1
3863	201	1	8-May-08	15	1	13	2	1	3	1	13	4	5	1
3864	201	1	4-May-07	15	1	13	2	2	3	1	4	4	1	1
3865	101	1	18-Oct-10	2	1	13	2	1	3	1	6	4	2	1
3866	101	1	4-Dec-08	1	1	13	2	1	3	1	6	4	2	1
3867	101	1	18-Sep-11	12	2	13	2	3	3	1	7	4	5	1
3868	101	1	22-Jan-15	20	2	13	2	1	3	1	4	4	1	1
3869	101	1	24-May-14	21	2	13	2	1	3	1	4	4	4	1
3870	202	0	11-May-13	0	1	14	2	1	3	1	4	4	4	1
3871	202	0	4-Oct-10	0	1	14	2	1	3	1	4	4	1	1
3872	202	0	28-May-17	3	1	14	2	3	3	1	4	4	1	1
3873	202	0	7-Aug-14	18	1	14	2	1	3	1	11	4	3	2
3874	101	1	28-Mar-14	23	1	14	2	1	3	1	4	4	1	1
3875	101	1	4-Oct-13	17	1	14	2	1	3	1	4	4	1	1
3876	101	1	17-Oct-12	14	1	14	2	1	3	1	4	4	5	4
3877	101	1	17-Sep-10	22	1	14	2	1	3	1	4	4	1	2
3878	101	1	28-Jan-08	14	1	14	2	1	3	1	4	4	4	1
3879	101	1	16-Jun-07	11	1	14	2	1	3	1	4	4	1	1
3880	101	1	16-Mar-13	23	1	14	2	1	3	1	3	4	1	1
3881	101	1	2-Feb-08	23	1	14	2	2	3	1	6	4	2	4
3882	101	1	13-May-07	4	2	14	2	3	3	1	4	4	1	1
3883	202	0	13-Oct-15	22	1	15	2	1	3	1	4	4	5	1
3884	202	0	1-Oct-15	0	1	15	2	1	3	1	4	4	6	1
3885	202	0	30-Jun-08	19	1	15	2	2	3	1	4	4	1	2
3886	202	0	11-Aug-07	23	1	15	2	1	3	1	4	4	1	1
3887	202	0	1-Jun-08	1	2	15	2	1	3	1	4	4	1	1
3888	201	1	14-Jun-16	23	1	15	2	3	3	1	6	4	2	1
3889	101	1	4-Jan-15	2	1	15	2	3	3	1	4	4	2	1
3890	101	1	16-Feb-14	20	1	15	2	1	3	1	4	4	5	1
3891	101	1	3-Jan-14	11	1	15	2	2	3	1	4	4	5	2
3892	101	1	8-Mar-14	23	1	15	2	1	3	1	4	4	5	1
3893	101	1	3-Jun-14	21	1	15	2	1	3	1	4	4	1	1
3894	101	1	4-Dec-08	1	1	15	2	1	3	1	4	4	4	1
3895	101	1	20-Aug-07	2	1	15	2	1	3	1	4	4	4	1
3896	101	1	10-Nov-06	21	1	15	2	1	3	1	6	4	6	2
3897	101	1	31-Mar-14	16	2	15	2	1	3	1	4	4	1	1
3898	201	1	15-Oct-08	22	2	15	2	1	3	1	6	4	5	2
3899	202	0	31-May-09	22	1	16	2	3	3	1	13	4	1	2
3900	202	0	26-Nov-14	0	1	16	2	2	3	1	4	4	5	1
3901	202	0	29-Jun-13	0	1	16	2	1	3	1	4	4	5	2
3902	202	0	8-Jun-08	2	1	16	2	1	3	1	4	4	5	1
3903	202	0	23-Jun-06	0	1	16	2	1	3	1	4	4	1	1
3904	202	0	5-Nov-17	22	1	16	2	2	3	1	4	4	5	1
3905	202	0	5-Nov-17	1	1	16	2	2	3	1	4	4	1	2
3906	202	0	12-Sep-17	21	1	16	2	2	3	1	4	4	1	1
3907	202	0	14-Nov-15	2	1	16	2	1	3	1	3	4	5	1
3908	202	0	4-Feb-17	21	1	16	2	1	3	1	3	4	1	1
3909	202	0	5-May-07	22	1	16	2	1	3	1	6	4	1	2
3910	202	0	5-Jul-06	3	1	16	2	1	3	1	6	4	4	2
3911	202	0	4-May-08	3	2	16	2	1	3	1	10	4	7	1
3912	202	0	17-Oct-06	20	2	16	2	3	3	1	4	4	1	1
3913	101	1	17-May-15	22	1	16	2	2	3	1	4	4	1	2
3914	101	1	27-Jan-15	3	1	16	2	3	3	1	4	4	1	1
3915	101	1	13-Jan-14	22	1	16	2	1	3	1	4	4	1	1
3916	101	1	29-Nov-14	16	1	16	2	1	3	1	4	4	4	1
3917	101	1	27-Sep-13	22	1	16	2	1	3	1	4	4	5	1
3918	101	1	28-Aug-12	2	1	16	2	1	3	1	4	4	2	2
3919	201	1	29-Aug-08	21	1	16	2	2	3	1	4	4	4	1
3920	101	1	5-May-08	21	1	16	2	2	3	1	6	4	4	1
3921	201	1	10-May-08	22	1	16	2	1	3	1	6	4	2	1
3922	201	1	29-Oct-07	13	1	16	2	1	3	1	6	4	1	1
3923	201	1	1-Oct-07	0	1	16	2	3	3	1	6	4	1	1
3924	101	1	8-Sep-14	1	2	16	2	2	3	1	4	4	5	1
3925	101	1	9-Oct-08	22	2	16	2	3	3	1	4	4	5	1
3926	202	0	6-Nov-16	19	1	17	2	2	3	1	4	4	5	1
3927	202	0	7-Nov-11	14	1	17	2	1	3	1	4	4	5	2
3928	202	0	7-Nov-11	14	1	17	2	1	3	1	4	4	1	2
3929	202	0	7-Nov-11	14	1	17	2	1	3	1	4	4	1	2
3930	202	0	30-Jun-07	15	1	17	2	1	3	1	4	4	6	1
3931	202	0	1-Jan-11	16	1	17	2	1	3	1	11	4	3	1
3932	202	0	26-Jul-16	22	1	17	2	2	3	1	6	4	2	2
3933	202	0	30-Dec-10	2	1	17	2	1	3	1	6	4	1	2
3934	202	0	1-Sep-09	0	1	17	2	1	3	1	6	4	2	1
3935	202	0	16-Jun-07	20	1	17	2	1	3	1	6	4	2	1
3936	202	0	30-Aug-14	1	2	17	2	1	3	1	4	4	1	1
3937	202	0	23-Nov-13	10	2	17	2	2	3	1	4	4	1	1
3938	101	1	15-Feb-17	2	1	17	2	1	3	1	6	4	4	2
3939	101	1	15-Feb-17	2	1	17	2	1	3	1	6	4	4	2
3940	201	1	3-Nov-16	1	1	17	2	2	3	1	4	4	1	1
3941	101	1	7-Nov-14	17	1	17	2	1	3	1	4	4	1	1
3942	101	1	19-May-14	21	1	17	2	1	3	1	4	4	4	1
3943	101	1	15-Aug-11	23	1	17	2	2	3	1	4	4	5	1
3944	101	1	30-May-11	12	1	17	2	1	3	1	4	4	1	1
3945	101	1	28-Jan-08	14	1	17	2	1	3	1	4	4	4	1
3946	201	1	17-Oct-07	0	1	17	2	2	3	1	4	4	4	1
3947	101	1	7-Nov-06	23	1	17	2	1	3	1	4	4	5	1
3948	101	1	22-Sep-06	20	1	17	2	1	3	1	4	4	1	1
3949	201	1	30-Aug-07	18	1	17	2	1	3	1	6	4	1	1
3950	101	1	28-Oct-07	21	2	17	2	1	3	1	4	4	4	1
3951	202	0	2-May-13	21	1	15	2	1	2	1	4	4	1	2
3952	101	1	2-Jun-15	23	1	15	2	1	2	1	6	4	2	1
3953	101	1	17-Jun-14	21	1	16	2	1	2	1	6	4	2	1
3954	101	1	23-Jun-07	5	1	16	2	1	2	1	6	4	2	1
3955	201	1	2-Feb-09	21	1	17	2	1	2	1	10	4	7	2
3956	201	1	8-Dec-06	23	1	10	2	3	1	0	13	4	2	1
3957	101	1	26-Sep-06	15	1	10	2	3	1	0	4	4	3	1
3958	202	0	5-Dec-13	9	1	11	2	2	1	0	13	4	1	1
3959	202	0	1-Oct-07	11	1	11	2	1	1	0	4	4	1	2
3960	202	0	7-Jun-09	11	1	11	2	2	1	0	11	4	3	2
3961	101	1	15-Jul-14	22	1	11	2	1	1	0	3	4	2	1

3962	101	1	1-May-12	17	2	11	2	1	1	0	4	4	1	1
3963	202	0	9-Dec-14	8	1	12	2	1	1	0	7	4	1	1
3964	202	0	12-Oct-07	8	1	12	2	3	1	0	4	4	1	1
3965	202	0	5-Sep-06	15	2	12	2	2	1	0	3	4	1	1
3966	101	1	15-Mar-17	16	1	12	2	1	1	0	4	4	3	1
3967	101	1	3-Feb-15	13	1	12	2	1	1	0	4	4	3	1
3968	101	1	19-Sep-13	15	1	12	2	1	1	0	4	4	5	1
3969	201	1	5-Jul-11	19	1	12	2	3	1	0	4	4	2	1
3970	201	1	22-May-09	9	1	12	2	1	1	0	4	4	2	1
3971	201	1	17-Oct-08	19	1	12	2	3	1	0	4	4	2	2
3972	101	1	12-Aug-06	15	1	12	2	3	1	0	4	4	3	1
3973	101	1	23-May-11	12	1	12	2	1	1	0	11	4	2	1
3974	101	1	18-Mar-07	11	1	12	2	1	1	0	11	4	5	1
3975	101	1	9-Jan-15	10	1	12	2	1	1	0	3	4	2	4
3976	201	1	28-Nov-14	20	1	12	2	2	1	0	3	4	5	1
3977	101	1	24-Nov-14	20	1	12	2	1	1	0	3	4	3	1
3978	101	1	24-Feb-13	16	1	12	2	1	1	0	3	4	4	1
3979	201	1	1-Jun-08	17	1	12	2	3	1	0	3	4	2	1
3980	101	1	3-May-13	18	1	12	2	1	1	0	6	4	2	1
3981	101	1	18-Jul-07	16	1	12	2	3	1	0	6	4	2	1
3982	101	1	14-May-17	17	2	12	2	1	1	0	11	4	3	1
3983	101	1	1-Aug-16	7	2	12	2	1	1	0	3	4	3	1
3984	101	1	12-Jan-15	18	2	12	2	3	1	0	3	4	2	2
3985	101	1	21-Nov-14	11	2	12	2	1	1	0	3	4	4	1
3986	101	1	1-May-11	22	2	12	2	1	1	0	3	4	3	1
3987	101	1	25-Jul-06	17	2	12	2	1	1	0	3	4	2	1
3988	202	0	15-Dec-11	21	1	13	2	1	1	0	10	4	7	2
3989	202	0	16-Oct-12	2	1	13	2	1	1	0	4	4	2	2
3990	202	0	14-Jun-11	17	1	13	2	3	1	0	4	4	2	1
3991	202	0	3-May-07	18	1	13	2	1	1	0	11	4	2	1
3992	202	0	25-Oct-08	16	1	13	2	1	1	0	3	4	2	1
3993	202	0	20-Feb-11	17	1	13	2	2	1	0	6	4	7	1
3994	202	0	16-Jan-12	1	2	13	2	1	1	0	10	4	7	2
3995	202	0	10-Jun-11	18	2	13	2	1	1	0	11	4	3	2
3996	202	0	9-Jun-08	21	2	13	2	3	1	0	3	4	3	1
3997	201	1	14-Aug-12	17	1	13	2	3	1	0	6	4	3	1
3998	101	1	19-Oct-14	11	1	13	2	1	1	0	4	4	3	1
3999	201	1	25-Jun-12	17	1	13	2	2	1	0	4	4	3	1
4000	101	1	23-Jul-12	15	1	13	2	3	1	0	4	4	3	4
4001	101	1	3-Dec-10	21	1	13	2	1	1	0	4	4	3	2
4002	201	1	6-Mar-09	11	1	13	2	1	1	0	4	4	2	1
4003	101	1	21-Jul-06	16	1	13	2	1	1	0	4	4	2	2
4004	101	1	27-Apr-17	13	1	13	2	1	1	0	4	4	2	1
4005	201	1	7-Aug-15	20	1	13	2	3	1	0	11	4	2	1
4006	101	1	3-Aug-13	22	1	13	2	1	1	0	11	4	2	2
4007	101	1	6-Apr-13	17	1	13	2	1	1	0	11	4	3	1
4008	201	1	28-Apr-09	16	1	13	2	3	1	0	11	4	2	1
4009	201	1	26-Mar-15	10	1	13	2	1	1	0	3	4	1	1
4010	101	1	25-Oct-14	18	1	13	2	1	1	0	3	4	2	1
4011	101	1	15-Oct-14	17	1	13	2	1	1	0	3	4	3	1
4012	101	1	24-Nov-14	21	1	13	2	1	1	0	3	4	3	1
4013	101	1	25-Feb-14	18	1	13	2	1	1	0	3	4	3	1
4014	201	1	28-Jul-13	17	1	13	2	1	1	0	3	4	3	1
4015	101	1	17-Oct-13	17	1	13	2	1	1	0	3	4	2	4
4016	101	1	23-Dec-13	12	1	13	2	1	1	0	3	4	2	1
4017	201	1	6-Aug-13	13	1	13	2	2	1	0	3	4	3	1
4018	201	1	28-Oct-17	22	1	13	2	1	1	0	3	4	4	1
4019	101	1	27-Nov-12	15	1	13	2	2	1	0	6	4	2	1
4020	101	1	28-Apr-06	19	1	13	2	2	1	0	6	4	2	1
4021	201	1	4-Sep-10	6	1	13	2	2	1	0	13	4	3	1
4022	201	1	8-Sep-16	19	2	13	2	2	1	0	6	4	3	1
4023	101	1	21-Dec-08	16	2	13	2	1	1	0	4	4	1	1
4024	101	1	13-Jan-08	15	2	13	2	1	1	0	4	4	1	1
4025	201	1	25-Jun-16	21	2	13	2	1	1	0	11	4	2	1
4026	101	1	15-Feb-16	22	2	13	2	1	1	0	3	4	3	1
4027	201	1	25-Jun-16	20	2	13	2	1	1	0	3	4	2	1
4028	101	1	20-Mar-12	15	2	13	2	1	1	0	3	4	4	1
4029	201	1	10-Sep-11	17	2	13	2	1	1	0	3	4	3	1
4030	101	1	29-Dec-09	20	2	13	2	3	1	0	3	4	4	1
4031	201	1	29-Jun-07	20	2	13	2	3	1	0	3	4	3	1
4032	101	1	30-Aug-14	10	2	13	2	1	1	0	6	4	2	1
4033	202	0	18-Aug-14	10	1	14	2	2	1	0	4	4	2	2
4034	202	0	29-Oct-16	5	1	14	2	3	1	0	10	4	7	1
4035	202	0	10-Nov-13	19	1	14	2	1	1	0	10	4	5	1
4036	202	0	26-May-06	12	1	14	2	1	1	0	10	4	1	1
4037	202	0	30-Jul-09	10	1	14	2	3	1	0	4	4	4	2
4038	202	0	18-Jan-06	11	1	14	2	4	1	0	4	4	3	1
4039	202	0	30-Sep-06	17	1	14	2	3	1	0	4	4	3	1
4040	202	0	6-Nov-17	11	1	14	2	1	1	0	4	4	1	1
4041	202	0	27-Oct-15	16	1	14	2	2	1	0	11	4	5	1
4042	202	0	3-Nov-12	0	1	14	2	1	1	0	3	4	5	2
4043	202	0	15-Apr-11	17	1	14	2	1	1	0	3	4	2	2
4044	202	0	5-Mar-11	16	1	14	2	1	1	0	3	4	1	2
4045	202	0	26-Mar-09	6	1	14	2	1	1	0	3	4	2	1
4046	202	0	25-Jul-17	22	1	14	2	3	1	0	3	4	2	1
4047	202	0	19-Aug-14	17	2	14	2	3	1	0	6	4	1	1
4048	202	0	11-Dec-11	9	2	14	2	3	1	0	13	4	1	1
4049	202	0	21-Aug-13	22	2	14	2	3	1	0	4	4	3	1
4050	202	0	14-Aug-13	16	2	14	2	1	1	0	11	4	5	1
4051	202	0	25-May-08	20	2	14	2	2	1	0	3	4	2	1
4052	201	1	1-Oct-15	18	1	14	2	2	1	0	10	4	6	1
4053	201	1	8-Jul-14	13	1	14	2	3	1	0	4	4	2	1
4054	201	1	18-Jul-12	17	1	14	2	2	1	0	4	4	2	2
4055	201	1	3-Jul-15	9	1	14	2	1	1	0	10	4	4	2
4056	201	1	11-Jun-11	1	1	14	2	1	1	0	13	4	1	2
4057	101	1	8-Aug-14	19	1	14	2	1	1	0	1	4	1	1
4058	201	1	13-Dec-16	1	1	14	2	1	1	0	4	4	4	1
4059	101	1	3-Nov-14	18	1	14	2	1	1	0	4	4	4	1
4060	201	1	12-Mar-13	8	1	14	2	3	1	0	4	4	2	1
4061	201	1	30-Aug-11	13	1	14	2	1	1	0	4	4	3	1
4062	201	1	1-Aug-08	21	1	14	2	3	1	0	4	4	2	1
4063	201	1	6-Apr-08	17	1	14	2	3	1	0	4	4	2	1
4064	101	1	3-Mar-07	8	1	14	2	1	1	0	4	4	3	1
4065	101	1	7-Nov-14	8	1	14	2	1	1	0	11	4	2	1
4066	101	1	28-Jan-13	21	1	14	2	1	1	0	11	4	2	1
4067	201	1	1-May-16	15	1	14	2	1	1	0	3	4	1	1
4068	201	1	17-Aug-16	17	1	14	2	2	1	0	3	4	1	1
4069	201	1	3-May-16	21	1	14	2	3	1	0	3	4	3	1
4070	101	1	1-Jan-15	9	1	14	2	1	1	0	3	4	3	1
4071	201	1	6-May-15	11	1	14	2	1	1	0	3	4	2	1
4072	201	1	15-Aug-14	15	1	14	2	2	1	0	3	4	3	1
4073	101	1	28-Nov-14	7	1	14	2	1	1	0	3	4	5	1
4074	101	1	19-Oct-14	11	1	14	2	1	1	0	3	4	1	1
4075	101	1	31-Oct-14	23	1	14	2	1	1	0	3	4	3	1
4076	201	1	16-Aug-14	23	1	14	2	1	1	0	3	4	3	2
4077	101	1	2-Nov-14	1	1	14	2	1	1	0	3	4	3	1
4078	201	1	23-Oct-13	15	1	14	2	1	1	0	3	4	3	1
4079	201	1	15-Oct-13	18	1	14	2	2	1	0	3	4	4	1
4080	201	1	25-Oct-11	15	1	14	2	2	1	0	3	4	5	1
4081	201	1	29-Jul-10	22	1	14	2	1	1	0	3	4	6	2
4082	201	1	14-Sep-10	23	1	14	2	1	1	0	3	4	5	1
4083	201	1	11-Apr-10	20	1	14	2	3	1	0	3	4	3	1
4084	201	1	27-Sep-11	19	1	14	2	3	1	0	6	4	2	2
4085	201	1	27-Sep-11	19	1	14	2	3	1	0	6	4	2	2

4086	101	1	16-Jun-11	1	1	14	2	3	1	0	6	4	2	1
4087	201	1	21-May-17	17	2	14	2	3	1	0	4	4	2	1
4088	201	1	21-Jul-06	20	2	14	2	3	1	0	3	4	1	1
4089	202	0	16-Oct-14	15	1	15	2	1	1	0	4	4	5	1
4090	202	0	1-Sep-13	16	1	15	2	1	1	0	4	4	2	1
4091	202	0	13-Aug-13	0	1	15	2	1	1	0	10	4	7	1
4092	202	0	20-Aug-09	19	1	15	2	3	1	0	7	4	3	2
4093	202	0	8-Jan-08	17	1	15	2	1	1	0	13	4	1	1
4094	202	0	18-Jul-08	8	1	15	2	1	1	0	1	4	6	1
4095	202	0	19-Jul-14	11	1	15	2	3	1	0	4	4	1	1
4096	202	0	25-Jun-07	17	1	15	2	2	1	0	4	4	2	2
4097	202	0	1-Nov-07	16	1	15	2	1	1	0	4	4	1	1
4098	202	0	8-Dec-16	19	1	15	2	1	1	0	11	4	3	1
4099	202	0	2-Dec-14	4	1	15	2	3	1	0	3	4	4	2
4100	202	0	3-Jun-07	18	1	15	2	1	1	0	3	4	2	2
4101	202	0	13-Apr-07	12	1	15	2	1	1	0	3	4	2	1
4102	202	0	20-Oct-06	17	1	15	2	3	1	0	3	4	4	2
4103	202	0	16-Apr-17	2	1	15	2	1	1	0	3	4	1	1
4104	202	0	18-Sep-07	12	1	15	2	1	1	0	6	4	2	1
4105	202	0	18-Jan-06	21	1	15	2	1	1	0	6	4	2	1
4106	202	0	25-Aug-13	21	2	15	2	3	1	0	10	4	6	2
4107	202	0	17-Jul-15	18	2	15	2	1	1	0	10	4	6	2
4108	202	0	18-Aug-10	2	2	15	2	2	1	0	10	4	7	4
4109	202	0	10-Aug-09	1	2	15	2	1	1	0	10	4	7	2
4110	202	0	21-Jun-12	1	2	15	2	3	1	0	4	4	5	1
4111	202	0	10-Sep-12	2	2	15	2	1	1	0	3	4	1	1
4112	202	0	15-Feb-08	8	2	15	2	1	1	0	6	4	2	1
4113	202	0	25-Feb-08	16	2	15	2	2	1	0	6	4	2	1
4114	202	0	9-Mar-06	10	2	15	2	3	1	0	6	4	2	1
4115	201	1	15-Jul-12	20	1	15	2	3	1	0	6	4	2	1
4116	201	1	1-Jul-16	13	1	15	2	1	1	0	6	4	3	1
4117	201	1	23-Jun-16	1	1	15	2	3	1	0	10	4	6	1
4118	201	1	24-Dec-17	22	1	15	2	1	1	0	10	4	6	2
4119	201	1	15-Aug-17	14	1	15	2	3	1	0	6	4	2	1
4120	201	1	27-Dec-17	13	1	15	2	2	1	0	6	4	2	1
4121	101	1	6-Jul-07	6	1	15	2	3	1	0	7	4	5	2
4122	201	1	29-Jun-16	8	1	15	2	1	1	0	4	4	5	1
4123	201	1	6-Apr-15	19	1	15	2	2	1	0	4	4	3	1
4124	101	1	9-Feb-15	11	1	15	2	1	1	0	4	4	3	1
4125	201	1	9-Oct-10	12	1	15	2	2	1	0	4	4	1	1
4126	201	1	27-May-10	19	1	15	2	3	1	0	4	4	2	1
4127	201	1	29-Jul-09	18	1	15	2	2	1	0	4	4	2	2
4128	101	1	23-May-08	18	1	15	2	3	1	0	4	4	2	1
4129	201	1	11-Oct-16	18	1	15	2	2	1	0	11	4	2	1
4130	201	1	14-Sep-13	17	1	15	2	1	1	0	11	4	3	1
4131	101	1	22-Aug-12	17	1	15	2	3	1	0	11	4	3	2
4132	101	1	25-Jan-11	17	1	15	2	2	1	0	11	4	2	1
4133	101	1	29-Nov-10	17	1	15	2	1	1	0	11	4	3	1
4134	201	1	25-Aug-17	11	1	15	2	2	1	0	11	4	3	1
4135	201	1	4-Feb-15	0	1	15	2	2	1	0	3	4	1	1
4136	201	1	12-Dec-14	17	1	15	2	3	1	0	3	4	3	1
4137	101	1	24-Oct-14	7	1	15	2	1	1	0	3	4	1	1
4138	201	1	20-Jun-14	17	1	15	2	1	1	0	3	4	2	1
4139	201	1	11-Oct-14	20	1	15	2	2	1	0	3	4	3	1
4140	201	1	18-Oct-13	22	1	15	2	2	1	0	3	4	3	1
4141	201	1	4-Oct-13	20	1	15	2	3	1	0	3	4	2	1
4142	101	1	6-Sep-12	8	1	15	2	1	1	0	3	4	3	1
4143	201	1	10-Mar-11	22	1	15	2	3	1	0	3	4	1	1
4144	101	1	31-May-11	10	1	15	2	1	1	0	3	4	3	1
4145	201	1	29-Jul-10	19	1	15	2	1	1	0	3	4	2	2
4146	101	1	20-Dec-07	20	1	15	2	3	1	0	3	4	3	1
4147	201	1	10-Jun-06	23	1	15	2	1	1	0	3	4	6	2
4148	101	1	3-Oct-13	10	1	15	2	1	1	0	6	4	2	1
4149	101	1	31-Aug-13	12	2	15	2	1	1	0	7	4	4	1
4150	101	1	23-Dec-12	17	2	15	2	3	1	0	1	4	3	1
4151	201	1	20-Aug-17	4	2	15	2	3	1	0	6	4	2	1
4152	201	1	8-Feb-10	18	2	15	2	3	1	0	11	4	2	1
4153	101	1	24-Aug-08	14	2	15	2	1	1	0	11	4	3	1
4154	101	1	16-Mar-14	10	2	15	2	1	1	0	3	4	1	1
4155	101	1	2-Oct-14	16	2	15	2	2	1	0	3	4	1	1
4156	101	1	15-Apr-14	20	2	15	2	1	1	0	3	4	3	1
4157	201	1	9-Nov-06	21	2	15	2	3	1	0	6	4	2	1
4158	202	0	4-Aug-13	21	1	16	2	1	1	0	1	4	2	1
4159	202	0	4-Aug-15	1	1	16	2	3	1	0	10	4	7	1
4160	202	0	14-Sep-14	0	1	16	2	1	1	0	10	4	7	1
4161	202	0	20-Nov-14	22	1	16	2	1	1	0	10	4	1	1
4162	202	0	20-Sep-16	16	1	16	2	3	1	0	6	4	2	2
4163	202	0	17-Aug-14	1	1	16	2	1	1	0	10	4	7	1
4164	202	0	18-Dec-10	21	1	16	2	1	1	0	1	4	6	2
4165	202	0	31-Jul-16	10	1	16	2	1	1	0	4	4	1	1
4166	202	0	2-Jul-15	11	1	16	2	1	1	0	4	4	2	2
4167	202	0	28-Sep-14	9	1	16	2	2	1	0	4	4	1	1
4168	202	0	18-Aug-14	19	1	16	2	1	1	0	4	4	2	2
4169	202	0	13-Sep-12	0	1	16	2	1	1	0	4	4	4	2
4170	202	0	10-Jul-10	8	1	16	2	1	1	0	4	4	1	1
4171	202	0	31-Dec-10	10	1	16	2	1	1	0	4	4	2	1
4172	202	0	29-Oct-08	8	1	16	2	1	1	0	4	4	6	1
4173	202	0	16-Dec-17	9	1	16	2	1	1	0	4	4	1	1
4174	202	0	10-Jul-16	18	1	16	2	1	1	0	11	4	3	1
4175	202	0	25-Aug-15	11	1	16	2	1	1	0	11	4	1	1
4176	202	0	16-Mar-09	12	1	16	2	1	1	0	11	4	1	1
4177	202	0	26-Aug-15	6	1	16	2	1	1	0	6	4	2	1
4178	202	0	10-Nov-14	8	1	16	2	3	1	0	6	4	2	2
4179	202	0	26-Aug-06	2	1	16	2	1	1	0	6	4	4	1
4180	202	0	28-Oct-13	1	2	16	2	1	1	0	10	4	7	2
4181	202	0	6-Dec-17	20	2	16	2	2	1	0	10	4	6	1
4182	202	0	4-Aug-11	23	2	16	2	1	1	0	10	4	5	2
4183	202	0	16-Jul-11	10	2	16	2	1	1	0	10	4	7	2
4184	202	0	16-Feb-10	13	2	16	2	1	1	0	10	4	7	1
4185	202	0	8-Aug-09	15	2	16	2	1	1	0	4	4	6	1
4186	202	0	6-Aug-16	0	2	16	2	2	1	0	3	4	1	2
4187	202	0	1-Sep-08	12	2	16	2	3	1	0	3	4	4	1
4188	202	0	19-Jul-09	3	2	16	2	1	1	0	6	4	2	1
4189	202	0	13-Dec-08	22	2	16	2	3	1	0	6	4	2	2
4190	201	1	8-Aug-12	10	1	16	2	2	1	0	4	4	2	1
4191	201	1	27-Sep-17	16	1	16	2	1	1	0	3	4	2	1
4192	201	1	9-Jun-16	11	1	16	2	1	1	0	6	4	2	1
4193	201	1	2-Aug-12	10	1	16	2	2	1	0	6	4	2	2
4194	201	1	23-Jul-14	21	1	16	2	1	1	0	6	4	3	2
4195	201	1	4-Apr-10	1	1	16	2	3	1	0	10	4	7	2
4196	101	1	17-Aug-11	23	1	16	2	1	1	0	7	4	4	2
4197	201	1	3-Dec-15	19	1	16	2	1	1	0	4	4	1	1
4198	101	1	28-Jan-15	20	1	16	2	1	1	0	4	4	1	1
4199	101	1	3-Mar-14	10	1	16	2	2	1	0	4	4	2	1
4200	101	1	29-Mar-14	12	1	16	2	1	1	0	4	4	1	1
4201	101	1	3-Jul-14	22	1	16	2	1	1	0	4	4	1	1
4202	101	1	19-Oct-14	10	1	16	2	1	1	0	4	4	3	1
4203	201	1	9-May-10	23	1	16	2	1	1	0	4	4	2	1
4204	201	1	19-Jul-06	13	1	16	2	1	1	0	4	4	1	1
4205	101	1	24-Jun-16	7	1	16	2	1	1	0	11	4	3	2
4206	201	1	14-Nov-15	15	1	16	2	3	1	0	11	4	2	1
4207	101	1	4-Oct-17	13	1	16	2	1	1	0	11	4	3	1
4208	201	1	10-Sep-16	21	1	16	2	3	1	0	3	4	1	1
4209	201	1	24-Feb-15	16	1	16	2	1	1	0	3	4	4	1

4210	101	1	1-Jan-15	10	1	16	2	1	1	0	3	4	3	2
4211	101	1	10-Apr-14	22	1	16	2	1	1	0	3	4	5	1
4212	101	1	19-Apr-14	19	1	16	2	1	1	0	3	4	5	1
4213	201	1	18-Jun-14	12	1	16	2	2	1	0	3	4	1	1
4214	101	1	10-Jan-14	11	1	16	2	2	1	0	3	4	1	1
4215	101	1	23-Dec-14	18	1	16	2	1	1	0	3	4	2	2
4216	101	1	13-Dec-14	15	1	16	2	1	1	0	3	4	2	1
4217	101	1	24-Mar-14	0	1	16	2	1	1	0	3	4	3	1
4218	201	1	31-Dec-07	5	1	16	2	2	1	0	3	4	3	1
4219	101	1	12-Feb-07	19	1	16	2	1	1	0	3	4	2	1
4220	201	1	18-Apr-15	13	1	16	2	2	1	0	6	4	3	1
4221	101	1	9-Nov-13	12	1	16	2	3	1	0	6	4	2	4
4222	201	1	28-Sep-10	19	1	16	2	1	1	0	6	4	2	1
4223	201	1	6-Sep-09	20	1	16	2	3	1	0	6	4	3	1
4224	201	1	31-Jul-08	12	1	16	2	1	1	0	6	4	2	1
4225	201	1	23-Nov-08	23	1	16	2	2	1	0	6	4	2	1
4226	201	1	1-Jul-07	21	1	16	2	1	1	0	6	4	2	1
4227	201	1	28-Sep-17	19	2	16	2	3	1	0	6	4	2	1
4228	201	1	23-Nov-11	4	2	16	2	2	1	0	1	4	1	1
4229	101	1	28-Nov-14	10	2	16	2	1	1	0	4	4	1	1
4230	201	1	29-Oct-11	1	2	16	2	3	1	0	4	4	1	1
4231	201	1	9-Jan-09	14	2	16	2	1	1	0	4	4	3	1
4232	101	1	30-Aug-12	12	2	16	2	3	1	0	11	4	2	1
4233	201	1	10-Jul-17	12	2	16	2	1	1	0	11	4	2	1
4234	201	1	23-Aug-13	1	2	16	2	2	1	0	3	4	1	1
4235	101	1	5-Sep-08	19	2	16	2	1	1	0	3	4	3	1
4236	201	1	19-Mar-12	11	2	16	2	2	1	0	6	4	3	1
4237	101	1	21-Aug-11	14	2	16	2	3	1	0	6	4	3	1
4238	201	1	1-Oct-08	21	2	16	2	2	1	0	6	4	3	1
4239	202	0	23-Sep-12	21	1	17	2	1	1	0	1	4	6	2
4240	202	0	18-Sep-13	0	1	17	2	1	1	0	10	4	6	1
4241	202	0	31-Jul-16	1	1	17	2	1	1	0	10	4	6	2
4242	202	0	29-Dec-14	12	1	17	2	1	1	0	7	4	5	2
4243	202	0	6-May-16	23	1	17	2	3	1	0	4	4	2	1
4244	202	0	13-Aug-14	4	1	17	2	2	1	0	4	4	2	2
4245	202	0	10-Nov-14	15	1	17	2	3	1	0	4	4	3	1
4246	202	0	9-Jun-13	23	1	17	2	1	1	0	4	4	3	1
4247	202	0	20-Oct-12	11	1	17	2	1	1	0	4	4	2	2
4248	202	0	13-Jun-11	18	1	17	2	3	1	0	4	4	3	1
4249	202	0	1-Nov-10	14	1	17	2	1	1	0	4	4	2	2
4250	202	0	13-Aug-10	17	1	17	2	1	1	0	4	4	1	1
4251	202	0	3-Jan-07	15	1	17	2	3	1	0	4	4	2	1
4252	202	0	28-May-12	22	1	17	2	3	1	0	11	4	3	1
4253	202	0	15-Aug-14	22	1	17	2	1	1	0	3	4	1	1
4254	202	0	25-Jan-16	18	1	17	2	2	1	0	6	4	2	2
4255	202	0	19-Dec-15	2	1	17	2	3	1	0	6	4	5	2
4256	202	0	16-Jul-10	16	1	17	2	1	1	0	6	4	2	2
4257	202	0	31-Jul-07	18	1	17	2	1	1	0	6	4	2	1
4258	202	0	13-May-17	0	1	17	2	1	1	0	6	4	1	1
4259	202	0	24-Jun-06	0	1	17	2	1	1	0	13	4	1	2
4260	202	0	14-Oct-15	19	2	17	2	1	1	0	10	4	6	1
4261	202	0	30-Oct-14	3	2	17	2	2	1	0	3	4	1	1
4262	202	0	14-Jun-07	11	2	17	2	1	1	0	13	4	7	2
4263	202	0	3-Apr-10	2	2	17	2	1	1	0	4	4	2	1
4264	202	0	8-Aug-12	23	2	17	2	1	1	0	11	4	2	2
4265	202	0	27-Jul-10	22	2	17	2	1	1	0	11	4	3	1
4266	202	0	23-Jul-16	19	2	17	2	1	1	0	3	4	4	1
4267	202	0	21-Jun-11	3	2	17	2	1	1	0	3	4	3	2
4268	202	0	28-Sep-07	19	2	17	2	1	1	0	3	4	7	1
4269	202	0	2-May-07	12	2	17	2	1	1	0	3	4	2	1
4270	202	0	9-Jul-07	21	2	17	2	1	1	0	6	4	2	1
4271	201	1	8-Aug-12	14	1	17	2	2	1	0	4	4	3	1
4272	201	1	1-May-14	10	1	17	2	1	1	0	6	4	3	1
4273	201	1	17-Aug-16	14	1	17	2	2	1	0	4	4	2	1
4274	101	1	10-Dec-17	14	1	17	2	1	1	0	6	4	1	1
4275	201	1	31-Dec-15	15	1	17	2	2	1	0	6	4	2	2
4276	201	1	9-May-14	10	1	17	2	1	1	0	6	4	2	2
4277	101	1	12-Apr-17	2	1	17	2	1	1	0	6	4	2	1
4278	101	1	15-Mar-14	19	1	17	2	2	1	0	7	4	5	2
4279	101	1	15-Mar-14	19	1	17	2	2	1	0	7	4	5	1
4280	101	1	29-Jul-12	12	1	17	2	2	1	0	13	4	3	1
4281	201	1	15-Sep-10	21	1	17	2	3	1	0	1	4	2	1
4282	101	1	19-Aug-14	22	1	17	2	1	1	0	4	4	3	1
4283	101	1	27-Mar-14	17	1	17	2	1	1	0	11	4	3	1
4284	101	1	20-May-17	20	1	17	2	1	1	0	11	4	3	1
4285	201	1	8-Nov-15	0	1	17	2	2	1	0	3	4	2	1
4286	201	1	22-Aug-15	11	1	17	2	2	1	0	3	4	3	2
4287	201	1	27-Jul-15	22	1	17	2	2	1	0	3	4	1	2
4288	101	1	15-May-14	19	1	17	2	1	1	0	3	4	5	1
4289	101	1	30-Nov-14	9	1	17	2	1	1	0	3	4	5	1
4290	101	1	19-May-14	20	1	17	2	1	1	0	3	4	3	1
4291	101	1	23-Nov-14	11	1	17	2	1	1	0	3	4	3	1
4292	101	1	3-Nov-13	20	1	17	2	1	1	0	3	4	4	1
4293	201	1	12-Sep-09	20	1	17	2	2	1	0	3	4	3	1
4294	101	1	15-Oct-12	14	1	17	2	1	1	0	6	4	2	1
4295	101	1	18-Sep-10	11	1	17	2	1	1	0	6	4	3	2
4296	201	1	8-Sep-10	15	1	17	2	2	1	0	6	4	2	1
4297	101	1	12-Jun-10	22	1	17	2	1	1	0	6	4	3	1
4298	201	1	3-Jun-07	12	1	17	2	1	1	0	6	4	2	1
4299	101	1	2-May-07	16	1	17	2	1	1	0	6	4	1	1
4300	101	1	17-Mar-11	9	2	17	2	1	1	0	4	4	1	1
4301	101	1	26-Sep-15	15	2	17	2	1	1	0	11	4	3	1
4302	101	1	27-Apr-13	19	2	17	2	1	1	0	11	4	2	1
4303	101	1	29-May-14	0	2	17	2	1	1	0	3	4	1	1
4304	101	1	17-Nov-11	1	2	17	2	1	1	0	3	4	1	1
4305	201	1	26-Jul-17	11	2	17	2	1	1	0	3	4	2	1
4306	101	1	15-Feb-14	17	2	17	2	1	1	0	6	4	4	1
4307	101	1	6-Dec-12	13	2	17	2	1	1	0	6	4	2	1
4308	101	1	20-Feb-13	20	1	12	2	1	3	1	4	3	4	1
4309	201	1	3-Nov-17	16	1	12	2	3	3	1	4	3	1	1
4310	202	0	11-Oct-13	11	1	13	2	1	3	1	4	3	1	1
4311	202	0	27-May-11	19	1	13	2	1	3	1	13	3	1	1
4312	202	0	29-Jun-16	17	1	13	2	1	3	1	4	3	5	1
4313	202	0	24-Oct-14	17	1	13	2	1	3	1	4	3	5	1
4314	202	0	24-Oct-14	18	1	13	2	2	3	1	4	3	1	1
4315	202	0	30-Nov-11	19	1	13	2	1	3	1	4	3	2	2
4316	202	0	26-Jan-10	22	1	13	2	1	3	1	4	3	1	2
4317	202	0	7-Jul-15	18	1	13	2	3	3	1	3	3	5	1
4318	202	0	5-Jun-07	20	1	13	2	1	3	1	3	3	1	1
4319	202	0	4-Dec-07	11	2	13	2	1	3	1	4	3	1	2
4320	101	1	13-Jan-13	18	1	13	2	1	3	1	4	3	2	2
4321	101	1	28-Apr-13	20	1	13	2	3	3	1	4	3	1	1
4322	101	1	25-May-13	22	1	13	2	2	3	1	4	3	4	1
4323	101	1	11-May-12	19	1	13	2	1	3	1	4	3	1	1
4324	101	1	21-Nov-11	18	1	13	2	1	3	1	4	3	1	1
4325	101	1	8-Aug-10	11	1	13	2	1	3	1	4	3	1	1
4326	101	1	5-Sep-09	22	1	13	2	1	3	1	4	3	1	1
4327	101	1	3-Jul-09	16	1	13	2	1	3	1	4	3	5	1
4328	201	1	21-May-08	13	1	13	2	2	3	1	4	3	4	1
4329	101	1	28-Apr-17	17	1	13	2	1	3	1	4	3	5	1
4330	101	1	27-May-10	20	1	13	2	1	3	1	11	3	3	1
4331	201	1	26-Oct-07	22	1	13	2	1	3	1	6	3	1	1
4332	202	0	10-Jul-15	16	1	14	2	1	3	1	4	3	1	1
4333	202	0	24-Oct-14	17	1	14	2	1	3	1	4	3	5	1

4334	202	0	24-Oct-14	18	1	14	2	1	3	1	4	3	1	1
4335	202	0	7-Oct-14	14	1	14	2	1	3	1	4	3	5	1
4336	202	0	12-Apr-08	12	1	14	2	2	3	1	4	3	1	2
4337	202	0	9-Jun-06	9	1	14	2	1	3	1	4	3	1	1
4338	202	0	24-Sep-17	18	1	14	2	2	3	1	4	3	1	1
4339	202	0	4-Sep-17	4	1	14	2	2	3	1	4	3	1	1
4340	202	0	30-Nov-16	16	1	14	2	3	3	1	3	3	1	1
4341	202	0	27-Apr-11	10	1	14	2	1	3	1	6	3	1	1
4342	202	0	29-Sep-07	22	1	14	2	3	3	1	6	3	5	2
4343	202	0	29-Sep-07	21	1	14	2	3	3	1	6	3	2	1
4344	202	0	24-Apr-06	23	1	14	2	1	3	1	6	3	2	1
4345	202	0	12-Apr-16	18	2	14	2	1	3	1	4	3	1	1
4346	101	1	24-Jun-06	10	1	14	2	1	3	1	13	3	6	1
4347	101	1	31-Oct-14	13	1	14	2	2	3	1	4	3	2	1
4348	101	1	24-Oct-14	20	1	14	2	1	3	1	4	3	1	2
4349	101	1	13-Jan-13	18	1	14	2	1	3	1	4	3	1	1
4350	101	1	22-Jun-13	21	1	14	2	1	3	1	4	3	5	1
4351	101	1	1-Apr-13	19	1	14	2	1	3	1	4	3	5	1
4352	101	1	7-Jun-12	21	1	14	2	1	3	1	4	3	1	1
4353	101	1	8-Sep-12	19	1	14	2	3	3	1	4	3	1	4
4354	101	1	24-Jun-09	12	1	14	2	1	3	1	4	3	5	1
4355	101	1	29-Jul-09	12	1	14	2	1	3	1	4	3	4	1
4356	101	1	3-Oct-06	11	1	14	2	1	3	1	4	3	1	1
4357	201	1	2-Dec-06	18	1	14	2	2	3	1	4	3	4	1
4358	101	1	15-Nov-17	11	1	14	2	1	3	1	4	3	4	1
4359	101	1	30-Oct-09	20	1	14	2	1	3	1	11	3	2	1
4360	201	1	10-Aug-06	18	1	14	2	2	3	1	11	3	3	1
4361	201	1	27-Jul-07	13	1	14	2	1	3	1	6	3	2	1
4362	101	1	13-Oct-06	22	1	14	2	1	3	1	6	3	1	2
4363	101	1	2-Jul-12	18	2	14	2	1	3	1	4	3	5	4
4364	101	1	10-Sep-08	6	2	14	2	1	3	1	11	3	2	1
4365	202	0	15-Oct-14	20	1	15	2	1	3	1	4	3	5	1
4366	202	0	10-Jul-15	21	1	15	2	2	3	1	4	3	1	1
4367	202	0	8-Jul-14	16	1	15	2	3	3	1	4	3	4	1
4368	202	0	20-Feb-12	14	1	15	2	1	3	1	4	3	5	1
4369	202	0	9-Jun-07	14	1	15	2	1	3	1	4	3	5	1
4370	202	0	25-Jun-07	1	1	15	2	1	3	1	4	3	1	1
4371	202	0	4-Apr-10	9	1	15	2	1	3	1	6	3	1	2
4372	202	0	31-Jul-08	4	1	15	2	2	3	1	6	3	2	2
4373	101	1	22-Jul-09	19	1	15	2	1	3	1	13	3	6	2
4374	201	1	20-Dec-08	14	1	15	2	1	3	1	13	3	4	1
4375	101	1	23-Sep-15	18	1	15	2	1	3	1	4	3	5	1
4376	101	1	17-Sep-15	10	1	15	2	1	3	1	4	3	5	1
4377	101	1	10-Feb-13	22	1	15	2	1	3	1	4	3	5	1
4378	101	1	25-Feb-13	18	1	15	2	1	3	1	4	3	1	1
4379	201	1	22-Sep-13	17	1	15	2	1	3	1	4	3	1	1
4380	101	1	3-Sep-12	18	1	15	2	3	3	1	4	3	1	1
4381	101	1	1-Nov-11	22	1	15	2	1	3	1	4	3	1	1
4382	101	1	27-Dec-09	17	1	15	2	1	3	1	4	3	5	1
4383	101	1	27-Jul-09	22	1	15	2	2	3	1	4	3	5	1
4384	101	1	25-Sep-09	20	1	15	2	1	3	1	4	3	1	1
4385	101	1	21-Jul-06	16	1	15	2	1	3	1	4	3	6	1
4386	101	1	15-Sep-06	2	1	15	2	1	3	1	4	3	1	1
4387	101	1	16-Jul-09	22	1	15	2	1	3	1	11	3	2	1
4388	201	1	10-Apr-07	21	1	15	2	1	3	1	11	3	3	1
4389	201	1	18-Aug-07	10	1	15	2	1	3	1	11	3	2	1
4390	101	1	24-May-07	20	2	15	2	1	3	1	4	3	1	1
4391	202	0	11-Apr-10	18	1	16	2	1	3	1	1	3	6	2
4392	202	0	8-Jan-14	23	1	16	2	3	3	1	4	3	1	1
4393	202	0	30-Jul-12	2	1	16	2	1	3	1	4	3	6	2
4394	202	0	30-Jul-12	2	1	16	2	1	3	1	4	3	6	1
4395	202	0	30-Jul-12	2	1	16	2	1	3	1	4	3	1	2
4396	202	0	29-Sep-12	18	1	16	2	1	3	1	4	3	1	1
4397	202	0	20-Feb-12	17	1	16	2	2	3	1	4	3	5	1
4398	202	0	9-Sep-11	22	1	16	2	1	3	1	4	3	1	1
4399	202	0	21-Dec-09	12	1	16	2	1	3	1	4	3	6	1
4400	202	0	1-Feb-17	4	1	16	2	2	3	1	4	3	5	1
4401	202	0	30-Sep-11	22	1	16	2	1	3	1	6	3	2	2
4402	202	0	24-Mar-10	3	1	16	2	1	3	1	6	3	2	1
4403	202	0	5-Jul-06	1	1	16	2	3	3	1	6	3	3	1
4404	202	0	23-Jun-10	7	1	16	2	1	3	1	13	3	5	2
4405	202	0	18-Oct-16	23	2	16	2	3	3	1	4	3	4	1
4406	202	0	13-Oct-13	23	2	16	2	3	3	1	4	3	5	1
4407	202	0	17-Jul-06	5	2	16	2	1	3	1	6	3	2	1
4408	201	1	11-Jan-12	22	1	16	2	1	3	1	13	3	1	1
4409	201	1	18-Dec-08	10	1	16	2	1	3	1	13	3	1	1
4410	201	1	22-Dec-16	6	1	16	2	2	3	1	4	3	3	1
4411	101	1	26-Jun-15	14	1	16	2	1	3	1	4	3	5	1
4412	101	1	15-Jan-14	20	1	16	2	1	3	1	4	3	1	1
4413	201	1	22-Sep-13	17	1	16	2	1	3	1	4	3	1	1
4414	101	1	21-Nov-11	19	1	16	2	1	3	1	4	3	1	1
4415	101	1	21-Nov-11	19	1	16	2	1	3	1	4	3	1	1
4416	101	1	21-Nov-11	19	1	16	2	1	3	1	4	3	2	1
4417	101	1	3-Jun-09	17	1	16	2	1	3	1	4	3	5	1
4418	101	1	13-Mar-09	17	1	16	2	1	3	1	4	3	1	2
4419	101	1	1-Aug-08	16	1	16	2	1	3	1	4	3	5	1
4420	101	1	15-Jan-08	17	1	16	2	1	3	1	4	3	1	1
4421	101	1	5-May-07	11	1	16	2	1	3	1	4	3	6	1
4422	101	1	18-Apr-11	20	1	16	2	1	3	1	11	3	3	1
4423	101	1	8-Aug-09	18	1	16	2	1	3	1	6	3	2	1
4424	202	0	5-Jul-16	21	1	17	2	3	3	1	4	3	1	1
4425	202	0	18-Apr-14	15	1	17	2	1	3	1	4	3	1	1
4426	202	0	15-Feb-14	3	1	17	2	1	3	1	4	3	3	1
4427	202	0	4-Apr-10	15	1	17	2	1	3	1	4	3	6	1
4428	202	0	11-Jul-16	2	1	17	2	3	3	1	6	3	2	2
4429	202	0	11-Apr-10	18	1	17	2	1	3	1	6	3	1	1
4430	202	0	8-Oct-10	0	1	17	2	1	3	1	6	3	1	1
4431	202	0	8-Oct-10	0	1	17	2	1	3	1	6	3	1	1
4432	202	0	10-May-10	4	1	17	2	1	3	1	6	3	1	1
4433	202	0	23-Apr-10	1	1	17	2	1	3	1	6	3	1	1
4434	202	0	11-Dec-09	2	1	17	2	1	3	1	6	3	4	2
4435	202	0	22-Nov-08	2	1	17	2	1	3	1	6	3	1	1
4436	202	0	7-Dec-08	1	1	17	2	1	3	1	6	3	1	1
4437	202	0	2-May-07	4	1	17	2	1	3	1	6	3	6	2
4438	202	0	5-Jan-10	22	2	17	2	1	3	1	4	3	1	1
4439	202	0	9-Sep-17	10	2	17	2	2	3	1	11	3	3	1
4440	201	1	2-Dec-17	1	1	17	2	3	3	1	4	3	1	1
4441	101	1	17-Apr-14	17	1	17	2	1	3	1	4	3	5	1
4442	101	1	14-Sep-13	18	1	17	2	1	3	1	4	3	5	1
4443	101	1	28-Feb-13	1	1	17	2	3	3	1	4	3	5	1
4444	101	1	23-May-13	12	1	17	2	1	3	1	4	3	1	4
4445	101	1	18-Oct-08	11	1	17	2	3	3	1	4	3	4	1
4446	101	1	6-Mar-08	21	1	17	2	1	3	1	4	3	1	2
4447	201	1	21-Oct-08	14	1	17	2	1	3	1	4	3	1	1
4448	101	1	16-Jun-07	23	1	17	2	1	3	1	4	3	1	1
4449	101	1	4-Oct-06	22	1	17	2	1	3	1	4	3	7	2
4450	101	1	4-Jul-17	10	1	17	2	1	3	1	4	3	1	1
4451	201	1	13-Feb-07	14	1	17	2	1	3	1	11	3	3	1
4452	201	1	9-Jul-07	0	1	17	2	3	3	1	3	3	2	1
4453	101	1	19-Nov-08	17	1	17	2	1	3	1	6	3	5	2
4454	101	1	19-Nov-08	17	1	17	2	1	3	1	6	3	5	2
4455	101	1	28-Mar-08	19	1	17	2	1	3	1	6	3	1	1
4456	201	1	3-Jan-07	21	1	17	2	1	3	1	6	3	1	2
4457	201	1	17-Aug-07	9	2	17	2	1	3	1	6	3	2	1

4458	202	0	15-Aug-17	17	1	10	2	1	1	0	1	3	5	1
4459	202	0	7-May-16	14	1	10	2	1	1	0	11	3	4	1
4460	202	0	14-Apr-16	22	1	10	2	1	1	0	3	3	1	1
4461	202	0	21-Dec-15	15	2	10	2	2	1	0	1	3	4	2
4462	202	0	3-Jun-17	0	2	10	2	3	1	0	1	3	3	2
4463	201	1	25-May-10	14	1	10	2	1	1	0	1	3	4	1
4464	201	1	14-Dec-09	12	1	10	2	2	1	0	1	3	3	2
4465	202	0	30-Jun-14	19	1	11	2	1	1	0	1	3	3	1
4466	202	0	24-Aug-14	15	1	11	2	3	1	0	1	3	3	2
4467	202	0	13-Nov-16	16	1	11	2	1	1	0	1	3	1	1
4468	202	0	7-May-16	16	1	11	2	2	1	0	1	3	3	2
4469	202	0	21-Feb-10	1	1	11	2	1	1	0	1	3	1	2
4470	202	0	29-Aug-15	19	1	11	2	1	1	0	3	3	3	1
4471	202	0	12-Nov-15	16	1	11	2	1	1	0	3	3	1	1
4472	202	0	28-Feb-16	17	2	11	2	2	1	0	1	3	3	1
4473	202	0	13-Nov-10	15	2	11	2	1	1	0	1	3	3	1
4474	101	1	19-Nov-12	13	1	11	2	1	1	0	1	3	4	1
4475	201	1	9-Nov-15	15	1	11	2	3	1	0	1	3	3	1
4476	101	1	1-Feb-13	22	1	11	2	1	1	0	1	3	3	1
4477	201	1	19-Oct-10	20	1	11	2	2	1	0	1	3	1	1
4478	101	1	22-Jul-10	21	1	11	2	1	1	0	1	3	3	1
4479	101	1	11-Mar-17	17	1	11	2	1	1	0	1	3	2	4
4480	201	1	24-Aug-10	17	1	11	2	3	1	0	11	3	3	1
4481	201	1	17-Apr-14	16	1	11	2	3	1	0	3	3	2	1
4482	201	1	26-Dec-13	18	1	11	2	1	1	0	3	3	2	2
4483	101	1	9-Feb-06	13	1	11	2	1	1	0	3	3	1	1
4484	201	1	11-Jul-06	19	1	11	2	1	1	0	6	3	3	1
4485	101	1	28-Aug-17	11	1	11	2	1	1	0	6	3	2	1
4486	101	1	12-Feb-12	10	2	11	2	1	1	0	1	3	3	2
4487	201	1	1-Jul-11	22	2	11	2	1	1	0	1	3	3	2
4488	201	1	4-Jun-11	23	2	11	2	1	1	0	1	3	1	2
4489	201	1	18-Sep-11	0	2	11	2	1	1	0	1	3	5	2
4490	101	1	28-May-11	19	2	11	2	1	1	0	1	3	1	2
4491	201	1	5-Nov-16	17	2	11	2	1	1	0	11	3	3	1
4492	202	0	20-Apr-14	14	1	12	2	1	1	0	1	3	1	2
4493	202	0	16-Mar-14	17	1	12	2	2	1	0	1	3	1	1
4494	202	0	10-Sep-13	17	1	12	2	1	1	0	1	3	2	1
4495	202	0	22-Sep-12	12	1	12	2	1	1	0	1	3	5	1
4496	202	0	2-Oct-15	18	1	12	2	3	1	0	1	3	1	2
4497	202	0	23-Oct-16	15	1	12	2	2	1	0	1	3	5	2
4498	202	0	24-Jan-12	17	1	12	2	1	1	0	1	3	3	1
4499	202	0	8-Nov-13	18	1	12	2	1	1	0	7	3	3	1
4500	202	0	20-Nov-14	21	1	12	2	3	1	0	1	3	4	2
4501	202	0	24-Oct-14	6	1	12	2	3	1	0	1	3	3	1
4502	202	0	9-Dec-16	17	1	12	2	2	1	0	1	3	3	2
4503	202	0	13-Oct-16	12	1	12	2	2	1	0	1	3	3	1
4504	202	0	15-Jun-12	19	1	12	2	1	1	0	1	3	6	1
4505	202	0	21-Mar-16	19	1	12	2	1	1	0	4	3	3	1
4506	202	0	10-Jun-16	19	1	12	2	3	1	0	6	3	2	1
4507	202	0	24-Oct-12	7	1	12	2	2	1	0	1	3	1	2
4508	202	0	17-Nov-11	12	1	12	2	1	1	0	1	3	1	1
4509	202	0	20-Sep-10	13	1	12	2	2	1	0	1	3	6	2
4510	202	0	2-Nov-10	9	1	12	2	3	1	0	1	3	2	2
4511	202	0	29-Oct-07	12	1	12	2	3	1	0	1	3	7	2
4512	202	0	12-Nov-07	21	1	12	2	3	1	0	1	3	2	2
4513	202	0	22-Dec-06	21	1	12	2	1	1	0	4	3	1	1
4514	202	0	5-Sep-17	12	1	12	2	3	1	0	4	3	3	1
4515	202	0	25-Sep-16	15	1	12	2	1	1	0	3	3	3	1
4516	202	0	26-Jun-15	20	1	12	2	2	1	0	3	3	2	1
4517	202	0	25-Nov-14	19	1	12	2	2	1	0	3	3	5	1
4518	202	0	11-Sep-17	22	1	12	2	2	1	0	3	3	4	2
4519	202	0	26-Oct-17	16	1	12	2	3	1	0	3	3	2	1
4520	202	0	23-Mar-17	17	1	12	2	3	1	0	3	3	2	1
4521	202	0	13-Nov-16	16	2	12	2	1	1	0	1	3	1	1
4522	202	0	5-Jun-16	12	2	12	2	1	1	0	1	3	1	2
4523	202	0	22-Oct-13	19	2	12	2	1	1	0	1	3	1	1
4524	202	0	20-Jul-12	21	2	12	2	3	1	0	1	3	6	2
4525	202	0	22-Oct-14	7	2	12	2	2	1	0	1	3	3	1
4526	202	0	11-Jul-15	0	2	12	2	3	1	0	1	3	3	1
4527	202	0	12-Oct-13	12	2	12	2	1	1	0	1	3	5	2
4528	202	0	20-Sep-13	11	2	12	2	1	1	0	1	3	2	1
4529	202	0	18-Jun-09	18	2	12	2	1	1	0	7	3	3	1
4530	202	0	5-Jan-10	5	2	12	2	1	1	0	1	3	1	2
4531	202	0	13-Nov-09	6	2	12	2	1	1	0	1	3	5	2
4532	202	0	25-Jun-09	14	2	12	2	1	1	0	1	3	2	1
4533	202	0	11-Nov-09	19	2	12	2	1	1	0	1	3	1	1
4534	202	0	9-Mar-09	14	2	12	2	1	1	0	4	3	7	2
4535	202	0	1-Oct-09	16	2	12	2	1	1	0	11	3	6	1
4536	202	0	19-Mar-16	21	2	12	2	1	1	0	3	3	4	2
4537	202	0	18-Apr-14	17	2	12	2	1	1	0	3	3	2	1
4538	202	0	25-Apr-10	17	2	12	2	1	1	0	3	3	1	2
4539	202	0	19-May-17	0	2	12	2	1	1	0	3	3	3	1
4540	101	1	2-May-15	12	1	12	2	1	1	0	1	3	2	2
4541	101	1	27-Dec-15	18	1	12	2	1	1	0	1	3	3	1
4542	101	1	1-Aug-15	21	1	12	2	1	1	0	1	3	4	1
4543	201	1	26-Oct-14	16	1	12	2	2	1	0	1	3	5	1
4544	201	1	13-Nov-14	19	1	12	2	1	1	0	1	3	1	1
4545	201	1	11-Oct-14	14	1	12	2	1	1	0	1	3	1	1
4546	101	1	26-May-14	17	1	12	2	2	1	0	1	3	1	4
4547	201	1	5-Jan-14	7	1	12	2	2	1	0	1	3	2	1
4548	201	1	19-Jun-14	17	1	12	2	3	1	0	1	3	5	1
4549	101	1	21-Nov-14	21	1	12	2	1	1	0	1	3	5	1
4550	101	1	28-Sep-13	12	1	12	2	1	1	0	1	3	2	1
4551	201	1	23-Dec-13	16	1	12	2	1	1	0	1	3	3	1
4552	201	1	18-Nov-13	14	1	12	2	1	1	0	1	3	2	1
4553	101	1	10-Mar-13	15	1	12	2	1	1	0	1	3	2	1
4554	101	1	2-Dec-13	12	1	12	2	1	1	0	1	3	2	1
4555	101	1	27-Oct-13	20	1	12	2	1	1	0	1	3	3	1
4556	101	1	27-Jan-13	15	1	12	2	1	1	0	1	3	2	1
4557	101	1	3-Aug-12	13	1	12	2	1	1	0	1	3	1	1
4558	101	1	5-Jan-12	7	1	12	2	1	1	0	1	3	1	1
4559	101	1	30-Mar-17	16	1	12	2	1	1	0	1	3	5	1
4560	101	1	19-May-17	14	1	12	2	1	1	0	1	3	4	1
4561	201	1	12-Nov-17	9	1	12	2	1	1	0	1	3	3	1
4562	201	1	25-Jun-14	13	1	12	2	2	1	0	1	3	4	1
4563	201	1	27-Sep-12	12	1	12	2	1	1	0	1	3	6	1
4564	201	1	11-Dec-14	17	1	12	2	2	1	0	1	3	5	1
4565	201	1	4-Nov-13	22	1	12	2	2	1	0	1	3	5	1
4566	101	1	31-Mar-12	19	1	12	2	1	1	0	1	3	1	1
4567	101	1	5-Mar-12	13	1	12	2	1	1	0	1	3	3	1
4568	101	1	2-Feb-12	14	1	12	2	1	1	0	1	3	3	1
4569	101	1	19-Apr-13	7	1	12	2	1	1	0	1	3	5	1
4570	101	1	15-Nov-17	17	1	12	2	1	1	0	1	3	2	2
4571	101	1	14-May-12	18	1	12	2	1	1	0	1	3	2	1
4572	101	1	8-Aug-14	22	1	12	2	1	1	0	1	3	3	2
4573	201	1	5-Oct-12	22	1	12	2	1	1	0	4	3	2	1
4574	201	1	23-Oct-13	12	1	12	2	2	1	0	6	3	3	1
4575	101	1	13-May-17	19	1	12	2	1	1	0	3	3	2	1
4576	101	1	13-May-10	10	1	12	2	1	1	0	7	3	3	1
4577	101	1	16-Oct-14	17	1	12	2	1	1	0	1	3	1	2
4578	201	1	22-Mar-11	19	1	12	2	1	1	0	1	3	1	1
4579	201	1	3-Dec-11	15	1	12	2	3	1	0	1	3	1	1
4580	201	1	10-Sep-11	21	1	12	2	1	1	0	1	3	2	1
4581	101	1	7-Oct-11	19	1	12	2	1	1	0	1	3	4	2

4582	101	1	24-Jul-11	19	1	12	2	2	1	0	1	3	3	1
4583	101	1	30-Aug-11	15	1	12	2	1	1	0	1	3	1	2
4584	101	1	31-Dec-10	6	1	12	2	1	1	0	1	3	4	1
4585	101	1	14-Sep-10	12	1	12	2	1	1	0	1	3	1	2
4586	101	1	12-Apr-10	15	1	12	2	1	1	0	1	3	5	2
4587	201	1	17-Aug-09	12	1	12	2	3	1	0	1	3	1	1
4588	201	1	17-Sep-09	21	1	12	2	3	1	0	1	3	3	1
4589	101	1	11-Nov-09	15	1	12	2	1	1	0	1	3	3	4
4590	201	1	16-Sep-09	16	1	12	2	3	1	0	1	3	3	1
4591	101	1	31-Aug-08	10	1	12	2	1	1	0	1	3	3	2
4592	201	1	15-Jul-08	9	1	12	2	3	1	0	1	3	2	1
4593	201	1	13-Dec-07	11	1	12	2	1	1	0	1	3	2	2
4594	101	1	14-Jul-07	16	1	12	2	1	1	0	1	3	3	1
4595	201	1	8-Dec-06	17	1	12	2	2	1	0	1	3	2	2
4596	101	1	14-Dec-06	12	1	12	2	1	1	0	1	3	1	2
4597	101	1	17-Mar-13	13	1	12	2	1	1	0	1	3	1	1
4598	101	1	29-Oct-12	12	1	12	2	1	1	0	1	3	5	1
4599	101	1	13-Mar-11	11	1	12	2	1	1	0	1	3	1	1
4600	201	1	9-Feb-10	23	1	12	2	2	1	0	1	3	2	1
4601	101	1	9-May-09	19	1	12	2	1	1	0	1	3	2	2
4602	101	1	21-Feb-09	17	1	12	2	1	1	0	1	3	2	1
4603	201	1	12-Feb-08	14	1	12	2	3	1	0	1	3	3	1
4604	101	1	8-Sep-06	12	1	12	2	1	1	0	1	3	2	1
4605	101	1	30-Aug-11	15	1	12	2	1	1	0	4	3	1	1
4606	101	1	9-Oct-16	17	1	12	2	1	1	0	11	3	2	1
4607	201	1	29-Dec-16	16	1	12	2	2	1	0	11	3	2	1
4608	101	1	19-Apr-11	18	1	12	2	1	1	0	11	3	2	1
4609	201	1	20-Jul-06	19	1	12	2	1	1	0	11	3	3	1
4610	201	1	29-Mar-15	19	1	12	2	1	1	0	3	3	1	1
4611	101	1	8-May-15	20	1	12	2	1	1	0	3	3	2	2
4612	101	1	8-Sep-13	16	1	12	2	1	1	0	3	3	3	1
4613	101	1	6-Mar-12	12	1	12	2	1	1	0	3	3	2	4
4614	101	1	1-Oct-12	17	1	12	2	1	1	0	3	3	4	1
4615	101	1	24-Mar-12	17	1	12	2	1	1	0	3	3	2	1
4616	101	1	1-Jul-11	19	1	12	2	1	1	0	3	3	4	1
4617	101	1	3-Jun-11	11	1	12	2	1	1	0	3	3	2	1
4618	101	1	1-Apr-10	18	1	12	2	1	1	0	3	3	3	1
4619	101	1	3-Nov-09	18	1	12	2	1	1	0	3	3	1	2
4620	201	1	3-Dec-09	20	1	12	2	1	1	0	3	3	3	2
4621	201	1	9-Aug-08	19	1	12	2	1	1	0	3	3	5	1
4622	101	1	23-Dec-08	17	1	12	2	1	1	0	3	3	2	1
4623	101	1	31-Jul-07	19	1	12	2	1	1	0	3	3	3	2
4624	101	1	19-Aug-06	19	1	12	2	1	1	0	3	3	2	1
4625	201	1	17-May-17	18	1	12	2	1	1	0	3	3	3	1
4626	101	1	22-Jun-17	15	1	12	2	1	1	0	3	3	4	2
4627	101	1	14-Aug-13	15	1	12	2	1	1	0	6	3	3	1
4628	101	1	3-Dec-10	17	1	12	2	1	1	0	6	3	3	1
4629	201	1	31-Mar-08	9	1	12	2	1	1	0	6	3	3	1
4630	201	1	13-Mar-13	14	2	12	2	1	1	0	1	3	5	1
4631	201	1	12-May-17	18	2	12	2	2	1	0	3	3	3	1
4632	201	1	18-Dec-13	7	2	12	2	3	1	0	1	3	4	2
4633	201	1	26-Apr-14	11	2	12	2	1	1	0	1	3	2	1
4634	201	1	30-May-17	10	2	12	2	3	1	0	1	3	2	1
4635	101	1	13-Dec-15	19	2	12	2	1	1	0	1	3	3	1
4636	101	1	26-Aug-12	2	2	12	2	1	1	0	1	3	3	2
4637	101	1	17-Jun-17	17	2	12	2	1	1	0	4	3	5	1
4638	101	1	3-Sep-13	7	2	12	2	1	1	0	1	3	1	1
4639	101	1	19-Jan-11	14	2	12	2	1	1	0	1	3	1	2
4640	201	1	12-Jun-11	12	2	12	2	1	1	0	1	3	1	1
4641	101	1	22-Dec-11	14	2	12	2	1	1	0	1	3	3	2
4642	201	1	7-Aug-11	10	2	12	2	3	1	0	1	3	5	1
4643	101	1	13-Feb-10	18	2	12	2	3	1	0	1	3	1	1
4644	201	1	7-Oct-10	20	2	12	2	1	1	0	1	3	3	1
4645	201	1	16-Apr-10	15	2	12	2	1	1	0	1	3	1	2
4646	201	1	12-Jan-10	19	2	12	2	1	1	0	1	3	1	1
4647	101	1	13-Nov-10	17	2	12	2	1	1	0	1	3	3	1
4648	201	1	18-Nov-09	21	2	12	2	1	1	0	1	3	1	1
4649	101	1	12-Jul-08	15	2	12	2	2	1	0	1	3	3	1
4650	201	1	21-Jul-07	8	2	12	2	1	1	0	1	3	5	1
4651	201	1	20-Aug-11	9	2	12	2	2	1	0	1	3	3	1
4652	101	1	14-Jul-09	21	2	12	2	1	1	0	1	3	3	2
4653	101	1	23-Mar-09	14	2	12	2	2	1	0	1	3	1	1
4654	101	1	15-Sep-07	15	2	12	2	1	1	0	1	3	2	1
4655	201	1	19-Dec-06	9	2	12	2	3	1	0	1	3	1	1
4656	201	1	22-Sep-06	6	2	12	2	1	1	0	1	3	2	1
4657	101	1	16-Apr-06	9	2	12	2	3	1	0	1	3	2	1
4658	101	1	3-Oct-16	18	2	12	2	1	1	0	11	3	3	1
4659	101	1	18-Apr-13	17	2	12	2	1	1	0	11	3	3	1
4660	201	1	1-Aug-16	20	2	12	2	1	1	0	3	3	1	1
4661	201	1	29-Apr-14	17	2	12	2	1	1	0	3	3	2	2
4662	101	1	28-Oct-13	17	2	12	2	1	1	0	3	3	2	1
4663	101	1	22-Mar-12	19	2	12	2	1	1	0	3	3	2	4
4664	101	1	10-Jul-12	17	2	12	2	3	1	0	3	3	5	1
4665	101	1	9-Oct-12	18	2	12	2	1	1	0	3	3	5	1
4666	101	1	20-Dec-12	14	2	12	2	1	1	0	3	3	1	1
4667	101	1	30-Jan-10	10	2	12	2	1	1	0	3	3	4	2
4668	101	1	30-Jan-10	10	2	12	2	1	1	0	3	3	4	2
4669	101	1	15-Jul-09	15	2	12	2	1	1	0	3	3	3	1
4670	201	1	25-Oct-06	7	2	12	2	2	1	0	3	3	3	1
4671	101	1	9-Feb-06	15	2	12	2	1	1	0	3	3	1	1
4672	201	1	30-Apr-17	14	2	12	2	1	1	0	3	3	1	1
4673	201	1	6-Dec-17	16	2	12	2	2	1	0	3	3	3	1
4674	202	0	13-May-17	9	1	13	2	2	1	0	1	3	5	1
4675	202	0	12-Oct-17	20	1	13	2	2	1	0	1	3	3	1
4676	202	0	7-Nov-14	12	1	13	2	3	1	0	1	3	5	2
4677	202	0	26-Sep-15	13	1	13	2	1	1	0	1	3	1	1
4678	202	0	9-Oct-17	19	1	13	2	2	1	0	1	3	1	1
4679	202	0	16-Feb-16	14	1	13	2	1	1	0	1	3	1	2
4680	202	0	7-Oct-16	11	1	13	2	3	1	0	1	3	3	1
4681	202	0	26-Sep-15	11	1	13	2	2	1	0	1	3	1	1
4682	202	0	15-Dec-15	12	1	13	2	1	1	0	1	3	1	1
4683	202	0	7-Jul-14	15	1	13	2	1	1	0	1	3	3	1
4684	202	0	3-Oct-14	17	1	13	2	3	1	0	1	3	1	1
4685	202	0	8-Jun-13	17	1	13	2	1	1	0	1	3	2	1
4686	202	0	16-Oct-13	12	1	13	2	1	1	0	1	3	4	2
4687	202	0	10-Sep-13	9	1	13	2	1	1	0	1	3	5	1
4688	202	0	28-Jun-13	14	1	13	2	2	1	0	1	3	2	1
4689	202	0	12-May-17	0	1	13	2	1	1	0	1	3	1	2
4690	202	0	8-Oct-16	17	1	13	2	1	1	0	1	3	4	1
4691	202	0	23-Jan-12	17	1	13	2	3	1	0	1	3	3	2
4692	202	0	26-Oct-17	9	1	13	2	1	1	0	1	3	1	1
4693	202	0	2-Jun-15	20	1	13	2	3	1	0	1	3	5	2
4694	202	0	13-May-16	12	1	13	2	1	1	0	1	3	5	1
4695	202	0	30-Aug-14	14	1	13	2	1	1	0	1	3	5	2
4696	202	0	8-May-17	0	1	13	2	1	1	0	1	3	3	2
4697	202	0	8-Jun-17	13	1	13	2	1	1	0	1	3	5	1
4698	202	0	11-Mar-17	16	1	13	2	1	1	0	1	3	2	1
4699	202	0	29-Aug-15	22	1	13	2	3	1	0	1	3	3	1
4700	202	0	27-Dec-16	17	1	13	2	1	1	0	1	3	2	1
4701	202	0	12-Jan-14	16	1	13	2	1	1	0	1	3	1	2
4702	202	0	26-Apr-14	15	1	13	2	2	1	0	1	3	3	2
4703	202	0	29-May-14	11	1	13	2	1	1	0	1	3	4	1
4704	202	0	12-Apr-14	7	1	13	2	2	1	0	1	3	3	1
4705	202	0	28-Nov-14	23	1	13	2	1	1	0	1	3	5	1

4706	202	0	5-Oct-12	17	1	13	2	3	1	0	1	3	1	2
4707	202	0	22-Sep-12	19	1	13	2	1	1	0	1	3	3	1
4708	202	0	24-Jan-17	13	1	13	2	3	1	0	1	3	5	1
4709	202	0	15-Jul-17	15	1	13	2	3	1	0	1	3	1	2
4710	202	0	23-Sep-08	17	1	13	2	1	1	0	13	3	1	1
4711	202	0	15-Jun-12	21	1	13	2	1	1	0	1	3	7	2
4712	202	0	24-Apr-11	0	1	13	2	1	1	0	1	3	5	1
4713	202	0	31-Mar-11	12	1	13	2	1	1	0	1	3	6	1
4714	202	0	23-Apr-11	21	1	13	2	1	1	0	1	3	5	1
4715	202	0	17-Jul-11	12	1	13	2	1	1	0	1	3	1	2
4716	202	0	19-Jun-11	11	1	13	2	2	1	0	1	3	3	2
4717	202	0	2-Aug-11	16	1	13	2	3	1	0	1	3	3	1
4718	202	0	9-Feb-11	14	1	13	2	1	1	0	1	3	3	1
4719	202	0	18-Jul-11	12	1	13	2	1	1	0	1	3	3	1
4720	202	0	20-Feb-11	10	1	13	2	1	1	0	1	3	3	1
4721	202	0	8-Dec-10	14	1	13	2	1	1	0	1	3	1	2
4722	202	0	10-Feb-10	15	1	13	2	2	1	0	1	3	2	1
4723	202	0	30-Sep-09	6	1	13	2	1	1	0	1	3	6	2
4724	202	0	14-Sep-09	22	1	13	2	1	1	0	1	3	1	2
4725	202	0	20-Nov-09	17	1	13	2	2	1	0	1	3	2	2
4726	202	0	11-Nov-09	19	1	13	2	1	1	0	1	3	4	1
4727	202	0	7-Aug-08	17	1	13	2	1	1	0	1	3	6	2
4728	202	0	10-Sep-08	14	1	13	2	1	1	0	1	3	1	2
4729	202	0	20-Apr-08	20	1	13	2	2	1	0	1	3	2	1
4730	202	0	18-Aug-08	11	1	13	2	2	1	0	1	3	2	1
4731	202	0	22-Jul-08	18	1	13	2	1	1	0	1	3	3	1
4732	202	0	18-Oct-07	7	1	13	2	3	1	0	1	3	5	1
4733	202	0	30-Sep-07	16	1	13	2	1	1	0	1	3	1	1
4734	202	0	20-Mar-07	22	1	13	2	1	1	0	1	3	3	1
4735	202	0	5-Dec-07	18	1	13	2	1	1	0	1	3	2	1
4736	202	0	27-Nov-07	10	1	13	2	1	1	0	1	3	3	1
4737	202	0	4-Oct-07	21	1	13	2	1	1	0	1	3	2	1
4738	202	0	29-Dec-06	6	1	13	2	1	1	0	1	3	3	1
4739	202	0	6-Aug-11	20	1	13	2	3	1	0	1	3	1	2
4740	202	0	12-Sep-07	12	1	13	2	1	1	0	1	3	6	1
4741	202	0	12-Oct-07	17	1	13	2	1	1	0	1	3	1	1
4742	202	0	21-Dec-07	13	1	13	2	1	1	0	1	3	1	1
4743	202	0	21-Jul-07	7	1	13	2	1	1	0	1	3	1	1
4744	202	0	8-Jun-06	12	1	13	2	1	1	0	1	3	2	1
4745	202	0	7-Oct-13	20	1	13	2	1	1	0	4	3	2	1
4746	202	0	30-May-06	14	1	13	2	1	1	0	4	3	1	1
4747	202	0	21-Sep-10	9	1	13	2	1	1	0	11	3	4	2
4748	202	0	6-Apr-10	21	1	13	2	1	1	0	11	3	7	2
4749	202	0	27-Aug-16	13	1	13	2	1	1	0	3	3	2	1
4750	202	0	17-Jun-16	21	1	13	2	3	1	0	3	3	2	1
4751	202	0	1-Dec-15	14	1	13	2	1	1	0	3	3	4	1
4752	202	0	22-May-14	16	1	13	2	1	1	0	3	3	2	1
4753	202	0	26-Sep-14	15	1	13	2	1	1	0	3	3	3	1
4754	202	0	14-Jul-14	12	1	13	2	1	1	0	3	3	2	1
4755	202	0	12-Dec-14	23	1	13	2	2	1	0	3	3	2	2
4756	202	0	13-Feb-13	10	1	13	2	3	1	0	3	3	2	2
4757	202	0	20-Apr-13	20	1	13	2	1	1	0	3	3	3	2
4758	202	0	7-Dec-12	20	1	13	2	1	1	0	3	3	5	1
4759	202	0	11-Aug-10	9	1	13	2	3	1	0	3	3	3	1
4760	202	0	5-Aug-10	15	1	13	2	3	1	0	3	3	3	1
4761	202	0	22-Oct-10	23	1	13	2	2	1	0	3	3	1	2
4762	202	0	12-Apr-10	9	1	13	2	1	1	0	3	3	2	1
4763	202	0	18-Oct-09	12	1	13	2	1	1	0	3	3	2	1
4764	202	0	11-Mar-08	6	1	13	2	1	1	0	3	3	1	2
4765	202	0	10-Sep-08	9	1	13	2	2	1	0	3	3	2	1
4766	202	0	14-Mar-08	18	1	13	2	1	1	0	3	3	1	1
4767	202	0	21-Dec-06	21	1	13	2	1	1	0	3	3	1	1
4768	202	0	11-Nov-11	23	1	13	2	1	1	0	6	3	2	2
4769	202	0	27-Nov-10	12	1	13	2	1	1	0	6	3	3	1
4770	202	0	18-Oct-08	18	1	13	2	1	1	0	6	3	2	1
4771	202	0	25-Nov-07	10	1	13	2	1	1	0	6	3	1	2
4772	202	0	1-Apr-07	19	1	13	2	1	1	0	6	3	3	1
4773	202	0	16-Feb-14	15	2	13	2	1	1	0	1	3	2	2
4774	202	0	29-Dec-17	18	2	13	2	1	1	0	1	3	1	1
4775	202	0	12-Jul-12	19	2	13	2	1	1	0	3	3	5	1
4776	202	0	6-Feb-13	9	2	13	2	1	1	0	1	3	1	2
4777	202	0	31-Jan-14	11	2	13	2	1	1	0	1	3	1	1
4778	202	0	21-Dec-16	18	2	13	2	1	1	0	1	3	5	1
4779	202	0	18-May-16	18	2	13	2	2	1	0	1	3	5	1
4780	202	0	28-Sep-15	14	2	13	2	1	1	0	1	3	3	1
4781	202	0	14-Jun-14	4	2	13	2	3	1	0	1	3	1	2
4782	202	0	30-Jul-14	14	2	13	2	1	1	0	1	3	1	1
4783	202	0	15-May-13	18	2	13	2	1	1	0	1	3	1	1
4784	202	0	15-Sep-12	21	2	13	2	1	1	0	1	3	6	2
4785	202	0	26-Sep-17	19	2	13	2	1	1	0	1	3	5	1
4786	202	0	17-Feb-17	17	2	13	2	1	1	0	1	3	1	1
4787	202	0	12-Dec-17	9	2	13	2	1	1	0	1	3	1	1
4788	202	0	11-Dec-17	13	2	13	2	3	1	0	1	3	5	1
4789	202	0	16-Nov-11	17	2	13	2	1	1	0	1	3	1	2
4790	202	0	15-Nov-11	20	2	13	2	1	1	0	1	3	1	2
4791	202	0	23-Jan-10	11	2	13	2	1	1	0	1	3	4	1
4792	202	0	2-Apr-10	17	2	13	2	3	1	0	1	3	5	1
4793	202	0	20-Nov-09	19	2	13	2	1	1	0	1	3	6	1
4794	202	0	20-Nov-09	19	2	13	2	1	1	0	1	3	2	2
4795	202	0	14-May-09	12	2	13	2	1	1	0	1	3	1	2
4796	202	0	6-Dec-09	16	2	13	2	1	1	0	1	3	3	2
4797	202	0	12-Apr-08	7	2	13	2	1	1	0	1	3	1	1
4798	202	0	30-Jul-08	19	2	13	2	1	1	0	1	3	4	1
4799	202	0	12-Jan-08	14	2	13	2	1	1	0	1	3	6	1
4800	202	0	24-Dec-06	19	2	13	2	1	1	0	1	3	3	1
4801	202	0	21-Dec-06	7	2	13	2	1	1	0	1	3	5	1
4802	202	0	2-Sep-06	13	2	13	2	1	1	0	1	3	6	1
4803	202	0	13-Jan-06	20	2	13	2	3	1	0	1	3	1	1
4804	202	0	23-Dec-06	11	2	13	2	1	1	0	1	3	2	2
4805	202	0	4-Jan-08	17	2	13	2	1	1	0	1	3	1	1
4806	202	0	9-May-08	16	2	13	2	1	1	0	1	3	3	1
4807	202	0	25-Apr-16	9	2	13	2	2	1	0	4	3	3	1
4808	202	0	12-Apr-16	18	2	13	2	1	1	0	4	3	2	1
4809	202	0	24-Jan-14	15	2	13	2	1	1	0	4	3	1	1
4810	202	0	29-Mar-17	11	2	13	2	1	1	0	4	3	1	1
4811	202	0	9-Nov-09	8	2	13	2	1	1	0	11	3	3	1
4812	202	0	30-Oct-16	16	2	13	2	1	1	0	3	3	1	1
4813	202	0	14-May-15	15	2	13	2	3	1	0	3	3	1	1
4814	202	0	1-Aug-15	1	2	13	2	3	1	0	3	3	5	1
4815	202	0	15-Aug-14	22	2	13	2	1	1	0	3	3	4	2
4816	202	0	22-Oct-13	4	2	13	2	2	1	0	3	3	5	1
4817	202	0	20-Feb-10	10	2	13	2	1	1	0	3	3	1	2
4818	202	0	23-Oct-08	21	2	13	2	2	1	0	3	3	7	2
4819	202	0	8-Jul-07	14	2	13	2	1	1	0	3	3	2	1
4820	101	1	2-Oct-13	13	1	13	2	1	1	0	1	3	3	1
4821	101	1	18-Jun-13	14	1	13	2	1	1	0	1	3	5	2
4822	101	1	27-Dec-13	13	1	13	2	1	1	0	1	3	1	1
4823	201	1	23-Sep-16	21	1	13	2	2	1	0	1	3	3	1
4824	201	1	4-Sep-16	19	1	13	2	2	1	0	1	3	4	1
4825	201	1	24-May-16	6	1	13	2	1	1	0	1	3	5	1
4826	101	1	5-May-16	18	1	13	2	1	1	0	1	3	2	1
4827	201	1	9-Sep-15	18	1	13	2	2	1	0	1	3	5	1
4828	201	1	27-Nov-15	15	1	13	2	3	1	0	1	3	5	1
4829	101	1	31-Dec-15	20	1	13	2	1	1	0	1	3	5	1

4830	201	1	22-Dec-15	19	1	13	2	2	1	0	1	3	4	1
4831	101	1	12-Nov-15	14	1	13	2	1	1	0	1	3	2	1
4832	201	1	23-Jan-15	11	1	13	2	2	1	0	1	3	2	1
4833	201	1	5-Jan-14	16	1	13	2	3	1	0	1	3	2	1
4834	201	1	2-Jan-14	18	1	13	2	1	1	0	1	3	2	1
4835	201	1	23-May-14	7	1	13	2	1	1	0	1	3	5	1
4836	201	1	15-Apr-14	14	1	13	2	2	1	0	1	3	5	1
4837	101	1	31-Dec-14	18	1	13	2	1	1	0	1	3	1	1
4838	101	1	19-Mar-14	12	1	13	2	1	1	0	1	3	1	2
4839	201	1	5-Sep-14	17	1	13	2	2	1	0	1	3	3	1
4840	101	1	8-Aug-14	11	1	13	2	1	1	0	1	3	2	4
4841	101	1	24-Mar-14	17	1	13	2	1	1	0	1	3	2	2
4842	101	1	18-Nov-13	7	1	13	2	1	1	0	1	3	5	1
4843	201	1	19-May-13	9	1	13	2	1	1	0	1	3	2	1
4844	101	1	4-Jul-13	7	1	13	2	1	1	0	1	3	4	1
4845	101	1	4-Mar-13	17	1	13	2	1	1	0	1	3	4	1
4846	101	1	5-Nov-13	15	1	13	2	1	1	0	1	3	2	1
4847	101	1	27-Mar-13	7	1	13	2	1	1	0	1	3	4	1
4848	101	1	3-Jun-13	12	1	13	2	1	1	0	1	3	2	1
4849	201	1	22-Sep-12	20	1	13	2	2	1	0	1	3	5	1
4850	201	1	22-Apr-12	20	1	13	2	2	1	0	1	3	5	1
4851	201	1	25-Aug-17	7	1	13	2	1	1	0	1	3	1	1
4852	201	1	16-Jun-17	18	1	13	2	2	1	0	1	3	2	1
4853	101	1	11-Sep-17	17	1	13	2	1	1	0	1	3	5	1
4854	201	1	4-Nov-16	18	1	13	2	2	1	0	4	3	1	1
4855	201	1	28-Dec-13	20	1	13	2	1	1	0	1	3	4	1
4856	101	1	20-Nov-14	20	1	13	2	1	1	0	1	3	1	1
4857	101	1	3-Jul-12	16	1	13	2	1	1	0	1	3	3	1
4858	201	1	8-Jan-16	21	1	13	2	1	1	0	1	3	5	1
4859	201	1	7-Jan-14	8	1	13	2	2	1	0	1	3	2	1
4860	101	1	24-May-15	16	1	13	2	1	1	0	1	3	2	2
4861	201	1	11-May-15	12	1	13	2	1	1	0	1	3	1	2
4862	201	1	21-Mar-15	15	1	13	2	3	1	0	1	3	1	2
4863	201	1	15-Jul-14	10	1	13	2	1	1	0	1	3	1	1
4864	101	1	28-Feb-14	17	1	13	2	2	1	0	1	3	3	2
4865	101	1	11-Jul-14	10	1	13	2	3	1	0	1	3	1	1
4866	101	1	8-Nov-13	6	1	13	2	1	1	0	1	3	3	1
4867	101	1	1-Oct-13	10	1	13	2	2	1	0	1	3	3	2
4868	101	1	15-May-12	14	1	13	2	2	1	0	1	3	5	4
4869	101	1	14-May-12	13	1	13	2	1	1	0	1	3	5	1
4870	101	1	14-May-12	13	1	13	2	1	1	0	1	3	1	1
4871	101	1	23-May-12	16	1	13	2	1	1	0	1	3	5	1
4872	201	1	14-Oct-15	7	1	13	2	1	1	0	1	3	2	1
4873	101	1	19-Mar-15	7	1	13	2	1	1	0	1	3	5	1
4874	101	1	16-Apr-17	14	1	13	2	1	1	0	1	3	3	1
4875	201	1	11-Jan-14	11	1	13	2	2	1	0	1	3	5	2
4876	101	1	2-Sep-12	21	1	13	2	1	1	0	1	3	5	1
4877	101	1	28-Dec-12	21	1	13	2	1	1	0	1	3	3	2
4878	101	1	12-May-12	17	1	13	2	1	1	0	1	3	2	1
4879	101	1	5-Feb-12	13	1	13	2	1	1	0	1	3	1	2
4880	201	1	16-Jun-12	13	1	13	2	3	1	0	6	3	2	1
4881	201	1	14-Aug-16	19	1	13	2	1	1	0	6	3	3	1
4882	201	1	3-Jul-06	18	1	13	2	2	1	0	13	3	3	1
4883	101	1	22-Dec-13	9	1	13	2	3	1	0	1	3	5	1
4884	101	1	5-Jun-12	20	1	13	2	1	1	0	1	3	1	1
4885	101	1	17-Sep-12	10	1	13	2	1	1	0	1	3	2	1
4886	101	1	10-Apr-12	22	1	13	2	1	1	0	1	3	5	1
4887	201	1	2-Oct-11	11	1	13	2	2	1	0	1	3	4	1
4888	101	1	2-Oct-11	20	1	13	2	2	1	0	1	3	5	1
4889	201	1	3-Dec-11	14	1	13	2	2	1	0	1	3	1	1
4890	101	1	9-Nov-11	6	1	13	2	1	1	0	1	3	5	1
4891	101	1	30-May-11	13	1	13	2	1	1	0	1	3	5	1
4892	101	1	22-Jul-11	17	1	13	2	1	1	0	1	3	5	1
4893	201	1	24-Mar-11	13	1	13	2	2	1	0	1	3	6	1
4894	101	1	4-Nov-11	18	1	13	2	1	1	0	1	3	3	2
4895	201	1	1-Mar-11	9	1	13	2	1	1	0	1	3	2	2
4896	201	1	15-Oct-11	1	1	13	2	2	1	0	1	3	1	1
4897	201	1	14-Mar-10	9	1	13	2	3	1	0	1	3	1	2
4898	201	1	24-Jan-10	23	1	13	2	1	1	0	1	3	5	1
4899	101	1	2-Feb-10	22	1	13	2	2	1	0	1	3	3	4
4900	101	1	8-Nov-10	12	1	13	2	1	1	0	1	3	4	1
4901	201	1	29-Dec-10	17	1	13	2	1	1	0	1	3	1	2
4902	101	1	28-Dec-10	19	1	13	2	1	1	0	1	3	1	1
4903	101	1	20-Sep-10	17	1	13	2	1	1	0	1	3	1	1
4904	201	1	25-Mar-10	20	1	13	2	2	1	0	1	3	3	1
4905	201	1	7-May-10	8	1	13	2	1	1	0	1	3	4	1
4906	201	1	13-Oct-10	13	1	13	2	2	1	0	1	3	4	1
4907	101	1	6-Nov-09	22	1	13	2	1	1	0	1	3	1	1
4908	101	1	14-Jun-09	9	1	13	2	1	1	0	1	3	1	1
4909	201	1	16-Nov-09	19	1	13	2	3	1	0	1	3	2	2
4910	101	1	19-Sep-09	20	1	13	2	2	1	0	1	3	2	1
4911	101	1	20-Oct-08	11	1	13	2	1	1	0	1	3	4	1
4912	101	1	12-Jul-08	17	1	13	2	1	1	0	1	3	3	1
4913	201	1	4-Apr-08	10	1	13	2	2	1	0	1	3	5	1
4914	201	1	1-Oct-08	22	1	13	2	2	1	0	1	3	2	1
4915	101	1	20-Aug-08	12	1	13	2	1	1	0	1	3	3	1
4916	101	1	23-Apr-08	11	1	13	2	1	1	0	1	3	3	2
4917	201	1	12-Dec-08	21	1	13	2	3	1	0	1	3	3	1
4918	101	1	10-Jun-08	13	1	13	2	1	1	0	1	3	3	2
4919	101	1	9-Mar-07	18	1	13	2	1	1	0	1	3	5	1
4920	201	1	13-Mar-07	13	1	13	2	1	1	0	1	3	1	2
4921	201	1	16-Apr-07	12	1	13	2	1	1	0	1	3	5	1
4922	201	1	13-Nov-07	12	1	13	2	1	1	0	1	3	3	1
4923	201	1	6-Nov-07	19	1	13	2	3	1	0	1	3	6	1
4924	201	1	7-Jan-07	22	1	13	2	1	1	0	1	3	1	1
4925	201	1	15-May-07	16	1	13	2	1	1	0	1	3	4	1
4926	101	1	15-Apr-07	19	1	13	2	1	1	0	1	3	3	1
4927	101	1	17-Nov-07	17	1	13	2	1	1	0	1	3	2	2
4928	101	1	21-Feb-06	19	1	13	2	3	1	0	1	3	6	1
4929	201	1	20-Jul-06	2	1	13	2	1	1	0	1	3	2	1
4930	101	1	6-Jun-06	7	1	13	2	1	1	0	1	3	4	1
4931	201	1	16-Sep-06	9	1	13	2	1	1	0	1	3	3	1
4932	101	1	3-Mar-06	17	1	13	2	1	1	0	1	3	3	1
4933	201	1	9-Sep-10	21	1	13	2	1	1	0	5	3	3	1
4934	101	1	13-Oct-13	12	1	13	2	1	1	0	1	3	5	1
4935	101	1	25-May-13	20	1	13	2	1	1	0	1	3	3	1
4936	101	1	21-Mar-12	20	1	13	2	1	1	0	1	3	5	1
4937	101	1	25-Feb-11	14	1	13	2	1	1	0	1	3	5	1
4938	201	1	22-Dec-11	7	1	13	2	1	1	0	1	3	2	1
4939	101	1	10-May-11	13	1	13	2	1	1	0	1	3	2	1
4940	201	1	19-May-11	7	1	13	2	2	1	0	1	3	2	1
4941	101	1	15-Jan-11	21	1	13	2	1	1	0	1	3	2	1
4942	201	1	23-Nov-11	4	1	13	2	2	1	0	1	3	1	2
4943	101	1	16-Oct-11	17	1	13	2	1	1	0	1	3	5	1
4944	101	1	19-Jan-10	7	1	13	2	1	1	0	1	3	2	1
4945	101	1	27-Jul-10	14	1	13	2	1	1	0	1	3	6	2
4946	101	1	28-Oct-10	21	1	13	2	1	1	0	1	3	2	2
4947	101	1	16-May-10	18	1	13	2	1	1	0	1	3	2	1
4948	201	1	6-Jul-10	20	1	13	2	3	1	0	1	3	3	1
4949	101	1	23-Jul-10	11	1	13	2	1	1	0	1	3	3	1
4950	101	1	8-Nov-09	15	1	13	2	1	1	0	1	3	5	2
4951	101	1	13-Apr-09	12	1	13	2	2	1	0	1	3	1	2
4952	201	1	4-Apr-09	20	1	13	2	1	1	0	1	3	5	1
4953	201	1	6-Sep-09	22	1	13	2	3	1	0	1	3	4	2

4954	201	1	17-Nov-09	20	1	13	2	1	1	0	1	3	1	1
4955	201	1	25-Nov-09	20	1	13	2	1	1	0	1	3	2	1
4956	101	1	1-Aug-08	21	1	13	2	3	1	0	1	3	2	2
4957	101	1	31-Aug-08	17	1	13	2	1	1	0	1	3	2	1
4958	101	1	31-Mar-08	16	1	13	2	3	1	0	1	3	4	2
4959	101	1	10-Jan-08	14	1	13	2	1	1	0	1	3	1	1
4960	201	1	17-Dec-07	17	1	13	2	1	1	0	1	3	5	1
4961	201	1	2-Jul-07	9	1	13	2	1	1	0	1	3	1	1
4962	101	1	25-Apr-07	21	1	13	2	1	1	0	1	3	4	1
4963	101	1	24-Nov-07	20	1	13	2	1	1	0	1	3	2	1
4964	101	1	30-Mar-06	8	1	13	2	1	1	0	1	3	3	1
4965	101	1	2-Oct-06	17	1	13	2	1	1	0	1	3	2	1
4966	101	1	10-Aug-06	19	1	13	2	1	1	0	1	3	1	1
4967	101	1	2-Nov-06	7	1	13	2	1	1	0	1	3	2	1
4968	101	1	24-Sep-17	13	1	13	2	1	1	0	1	3	2	1
4969	101	1	8-May-15	18	1	13	2	1	1	0	4	3	2	1
4970	101	1	29-Nov-10	18	1	13	2	1	1	0	4	3	3	1
4971	101	1	17-Jul-10	19	1	13	2	2	1	0	4	3	2	1
4972	201	1	5-Jun-07	19	1	13	2	1	1	0	4	3	4	1
4973	201	1	1-Sep-16	20	1	13	2	2	1	0	11	3	3	1
4974	101	1	24-Apr-14	12	1	13	2	1	1	0	11	3	2	1
4975	101	1	23-Dec-13	17	1	13	2	1	1	0	11	3	2	1
4976	201	1	2-Aug-12	12	1	13	2	2	1	0	11	3	3	1
4977	101	1	17-Jun-12	19	1	13	2	1	1	0	11	3	3	1
4978	101	1	13-May-09	14	1	13	2	1	1	0	11	3	2	1
4979	201	1	17-Nov-08	14	1	13	2	3	1	0	11	3	3	1
4980	101	1	1-May-07	16	1	13	2	1	1	0	11	3	2	1
4981	101	1	11-Dec-06	19	1	13	2	1	1	0	11	3	3	1
4982	101	1	8-Mar-17	14	1	13	2	1	1	0	11	3	2	1
4983	101	1	1-Aug-16	10	1	13	2	1	1	0	3	3	5	1
4984	201	1	19-Jun-16	21	1	13	2	2	1	0	3	3	3	1
4985	201	1	20-Jan-16	20	1	13	2	2	1	0	3	3	2	1
4986	201	1	10-Apr-16	13	1	13	2	1	1	0	3	3	3	1
4987	201	1	7-Mar-16	8	1	13	2	1	1	0	3	3	3	1
4988	201	1	22-Sep-16	19	1	13	2	1	1	0	3	3	3	1
4989	101	1	27-Apr-16	17	1	13	2	1	1	0	3	3	3	4
4990	201	1	21-Mar-16	14	1	13	2	2	1	0	3	3	2	1
4991	101	1	18-Nov-15	17	1	13	2	1	1	0	3	3	2	2
4992	201	1	24-Apr-15	17	1	13	2	1	1	0	3	3	2	1
4993	201	1	25-Jun-15	19	1	13	2	1	1	0	3	3	2	2
4994	101	1	2-Aug-15	14	1	13	2	1	1	0	3	3	3	1
4995	101	1	16-Jan-15	12	1	13	2	1	1	0	3	3	3	1
4996	201	1	15-May-15	15	1	13	2	2	1	0	3	3	2	1
4997	101	1	4-Oct-14	16	1	13	2	1	1	0	3	3	2	1
4998	101	1	4-Nov-14	15	1	13	2	1	1	0	3	3	3	1
4999	101	1	20-Nov-14	9	1	13	2	3	1	0	3	3	2	2
5000	101	1	9-Nov-14	20	1	13	2	2	1	0	3	3	1	4
5001	101	1	25-Feb-14	14	1	13	2	1	1	0	3	3	2	1
5002	101	1	21-Apr-14	21	1	13	2	1	1	0	3	3	2	1
5003	101	1	15-Aug-14	17	1	13	2	1	1	0	3	3	3	1
5004	101	1	25-Dec-13	16	1	13	2	1	1	0	3	3	1	1
5005	101	1	31-Jul-13	17	1	13	2	1	1	0	3	3	5	1
5006	101	1	12-Oct-13	21	1	13	2	1	1	0	3	3	2	1
5007	101	1	16-Apr-13	17	1	13	2	1	1	0	3	3	3	1
5008	201	1	21-Dec-12	18	1	13	2	1	1	0	3	3	5	1
5009	101	1	28-Jul-12	12	1	13	2	1	1	0	3	3	5	1
5010	201	1	12-Oct-12	12	1	13	2	2	1	0	3	3	2	1
5011	201	1	3-Nov-12	11	1	13	2	2	1	0	3	3	3	1
5012	101	1	29-Aug-12	19	1	13	2	3	1	0	3	3	3	1
5013	201	1	12-Nov-12	18	1	13	2	1	1	0	3	3	3	1
5014	101	1	26-Nov-12	13	1	13	2	1	1	0	3	3	3	1
5015	101	1	11-Aug-11	20	1	13	2	1	1	0	3	3	3	1
5016	201	1	13-Oct-11	4	1	13	2	2	1	0	3	3	4	1
5017	101	1	21-Jul-11	19	1	13	2	1	1	0	3	3	3	1
5018	201	1	2-Mar-11	18	1	13	2	1	1	0	3	3	3	1
5019	101	1	15-Nov-11	21	1	13	2	2	1	0	3	3	4	2
5020	201	1	20-Jan-11	19	1	13	2	1	1	0	3	3	3	1
5021	101	1	28-May-11	21	1	13	2	1	1	0	3	3	2	1
5022	101	1	21-Sep-10	17	1	13	2	1	1	0	3	3	4	1
5023	101	1	14-Oct-10	10	1	13	2	1	1	0	3	3	2	1
5024	201	1	20-Jul-10	6	1	13	2	2	1	0	3	3	2	1
5025	201	1	17-Mar-09	11	1	13	2	1	1	0	3	3	2	1
5026	201	1	17-Jul-09	7	1	13	2	2	1	0	3	3	1	1
5027	201	1	3-Feb-09	20	1	13	2	1	1	0	3	3	4	1
5028	101	1	14-Oct-09	18	1	13	2	1	1	0	3	3	2	4
5029	101	1	28-May-09	16	1	13	2	1	1	0	3	3	3	1
5030	101	1	28-May-09	16	1	13	2	1	1	0	3	3	3	1
5031	101	1	28-Sep-08	18	1	13	2	1	1	0	3	3	3	1
5032	201	1	14-Nov-08	11	1	13	2	1	1	0	3	3	1	1
5033	101	1	16-Dec-08	17	1	13	2	1	1	0	3	3	2	1
5034	101	1	4-Dec-08	13	1	13	2	1	1	0	3	3	2	1
5035	101	1	17-Sep-08	17	1	13	2	1	1	0	3	3	2	1
5036	101	1	24-Jan-07	15	1	13	2	1	1	0	3	3	7	1
5037	201	1	2-Sep-07	13	1	13	2	3	1	0	3	3	3	2
5038	201	1	28-Jul-07	19	1	13	2	3	1	0	3	3	2	1
5039	101	1	25-Jul-07	20	1	13	2	1	1	0	3	3	3	1
5040	101	1	21-Mar-07	17	1	13	2	1	1	0	3	3	2	2
5041	101	1	14-Aug-06	17	1	13	2	1	1	0	3	3	3	1
5042	101	1	1-Dec-06	17	1	13	2	1	1	0	3	3	2	1
5043	101	1	18-Aug-06	18	1	13	2	1	1	0	3	3	3	1
5044	101	1	1-Feb-06	17	1	13	2	1	1	0	3	3	2	2
5045	201	1	19-Oct-06	17	1	13	2	1	1	0	3	3	2	1
5046	101	1	29-Jan-06	18	1	13	2	1	1	0	3	3	2	2
5047	101	1	25-Apr-06	20	1	13	2	1	1	0	3	3	2	1
5048	101	1	13-Jan-17	10	1	13	2	1	1	0	3	3	5	1
5049	101	1	6-Aug-17	19	1	13	2	1	1	0	3	3	3	1
5050	201	1	19-Mar-17	10	1	13	2	1	1	0	3	3	3	1
5051	201	1	23-May-17	19	1	13	2	1	1	0	3	3	3	1
5052	101	1	5-Jan-17	11	1	13	2	1	1	0	3	3	2	1
5053	201	1	19-Oct-17	20	1	13	2	2	1	0	3	3	2	1
5054	101	1	16-Sep-17	12	1	13	2	1	1	0	3	3	2	1
5055	201	1	30-Dec-06	17	1	13	2	1	1	0	6	3	3	1
5056	101	1	12-Nov-17	19	1	13	2	1	1	0	13	3	3	1
5057	101	1	20-Nov-17	18	1	13	2	1	1	0	13	3	7	1
5058	101	1	16-Nov-17	8	1	13	2	1	1	0	13	3	2	1
5059	101	1	23-Jan-13	17	2	13	2	1	1	0	1	3	7	1
5060	101	1	2-Dec-16	10	2	13	2	1	1	0	1	3	3	1
5061	201	1	16-Sep-15	18	2	13	2	1	1	0	1	3	4	1
5062	101	1	6-Oct-14	17	2	13	2	1	1	0	1	3	2	2
5063	101	1	14-Sep-12	19	2	13	2	1	1	0	1	3	2	1
5064	201	1	25-Aug-16	20	2	13	2	1	1	0	3	3	3	1
5065	101	1	29-Nov-13	20	2	13	2	1	1	0	1	3	5	2
5066	101	1	7-May-15	12	2	13	2	1	1	0	1	3	4	2
5067	101	1	19-Apr-16	7	2	13	2	1	1	0	1	3	1	1
5068	201	1	26-Aug-15	22	2	13	2	3	1	0	1	3	3	1
5069	101	1	19-Oct-12	22	2	13	2	1	1	0	1	3	5	1
5070	101	1	26-Aug-12	1	2	13	2	1	1	0	1	3	5	2
5071	101	1	17-Oct-16	7	2	13	2	1	1	0	1	3	5	1
5072	101	1	16-Jun-15	18	2	13	2	1	1	0	1	3	5	2
5073	101	1	3-Jul-12	11	2	13	2	1	1	0	1	3	5	1
5074	101	1	14-Sep-11	13	2	13	2	1	1	0	1	3	3	1
5075	101	1	6-Apr-11	14	2	13	2	1	1	0	1	3	3	2
5076	101	1	21-Oct-11	12	2	13	2	1	1	0	1	3	3	1
5077	101	1	10-Oct-11	20	2	13	2	1	1	0	1	3	2	4

5078	101	1	16-Apr-11	16	2	13	2	1	1	0	1	3	3	1
5079	101	1	6-Aug-10	20	2	13	2	1	1	0	1	3	5	1
5080	201	1	20-Jan-09	9	2	13	2	2	1	0	1	3	4	2
5081	101	1	23-Apr-09	19	2	13	2	3	1	0	1	3	1	2
5082	101	1	28-Jul-09	8	2	13	2	1	1	0	1	3	3	2
5083	101	1	12-Sep-09	18	2	13	2	1	1	0	1	3	3	1
5084	201	1	24-Jul-08	18	2	13	2	1	1	0	1	3	5	1
5085	101	1	4-Dec-08	18	2	13	2	1	1	0	1	3	3	1
5086	101	1	23-Jun-08	18	2	13	2	1	1	0	1	3	3	2
5087	201	1	24-Mar-08	8	2	13	2	3	1	0	1	3	3	2
5088	101	1	20-Apr-07	7	2	13	2	1	1	0	1	3	1	1
5089	101	1	17-Sep-07	18	2	13	2	1	1	0	1	3	3	1
5090	101	1	29-Sep-07	12	2	13	2	1	1	0	1	3	5	1
5091	201	1	12-Jul-07	6	2	13	2	3	1	0	1	3	1	2
5092	201	1	30-Apr-07	20	2	13	2	1	1	0	1	3	4	1
5093	201	1	18-Dec-06	8	2	13	2	2	1	0	1	3	5	1
5094	101	1	1-Dec-06	22	2	13	2	1	1	0	1	3	5	1
5095	101	1	13-Dec-11	21	2	13	2	1	1	0	1	3	1	1
5096	201	1	1-Apr-10	18	2	13	2	2	1	0	1	3	1	2
5097	201	1	31-Jul-09	21	2	13	2	1	1	0	1	3	5	1
5098	201	1	14-Nov-08	12	2	13	2	1	1	0	1	3	1	2
5099	101	1	10-Apr-08	17	2	13	2	1	1	0	1	3	2	1
5100	101	1	7-Jun-07	18	2	13	2	1	1	0	1	3	2	2
5101	101	1	16-Mar-06	8	2	13	2	1	1	0	1	3	1	1
5102	101	1	18-Mar-11	18	2	13	2	1	1	0	4	3	3	1
5103	201	1	20-Jul-10	21	2	13	2	2	1	0	11	3	3	1
5104	101	1	16-Feb-10	2	2	13	2	3	1	0	11	3	2	1
5105	101	1	14-Dec-09	17	2	13	2	1	1	0	11	3	4	1
5106	201	1	2-Apr-08	13	2	13	2	1	1	0	11	3	3	1
5107	101	1	17-Mar-07	12	2	13	2	1	1	0	11	3	3	1
5108	201	1	15-Feb-17	17	2	13	2	1	1	0	11	3	3	1
5109	101	1	12-May-15	11	2	13	2	1	1	0	3	3	1	1
5110	101	1	4-Mar-15	14	2	13	2	1	1	0	3	3	5	1
5111	101	1	28-Jan-14	10	2	13	2	2	1	0	3	3	2	1
5112	101	1	14-Jun-13	17	2	13	2	1	1	0	3	3	5	1
5113	201	1	6-Apr-10	17	2	13	2	1	1	0	3	3	2	1
5114	101	1	25-Nov-09	17	2	13	2	1	1	0	3	3	2	1
5115	101	1	19-Nov-09	10	2	13	2	1	1	0	3	3	2	1
5116	201	1	12-May-08	19	2	13	2	3	1	0	3	3	1	1
5117	101	1	21-Aug-08	12	2	13	2	3	1	0	3	3	1	1
5118	201	1	22-Jul-08	21	2	13	2	1	1	0	3	3	1	1
5119	201	1	6-Mar-17	9	2	13	2	1	1	0	3	3	2	1
5120	101	1	18-Nov-17	15	2	13	2	1	1	0	3	3	3	1
5121	201	1	25-May-10	8	2	13	2	2	1	0	6	3	4	1
5122	202	0	19-Dec-14	7	1	14	2	1	1	0	1	3	1	1
5123	202	0	4-Oct-16	13	1	14	2	3	1	0	1	3	2	2
5124	202	0	26-Sep-15	8	1	14	2	1	1	0	1	3	5	1
5125	202	0	17-May-14	17	1	14	2	1	1	0	1	3	2	1
5126	202	0	15-Dec-14	20	1	14	2	1	1	0	1	3	2	2
5127	202	0	30-Sep-17	21	1	14	2	1	1	0	1	3	6	1
5128	202	0	17-Nov-15	11	1	14	2	2	1	0	1	3	1	1
5129	202	0	18-Oct-17	21	1	14	2	1	1	0	1	3	1	1
5130	202	0	24-Mar-17	20	1	14	2	3	1	0	1	3	1	2
5131	202	0	11-Mar-17	17	1	14	2	1	1	0	1	3	1	1
5132	202	0	4-Dec-15	20	1	14	2	1	1	0	1	3	1	1
5133	202	0	18-Oct-14	14	1	14	2	1	1	0	1	3	3	1
5134	202	0	7-Nov-14	18	1	14	2	1	1	0	1	3	1	2
5135	202	0	25-Aug-16	13	1	14	2	3	1	0	1	3	4	1
5136	202	0	30-Dec-16	14	1	14	2	1	1	0	1	3	1	1
5137	202	0	3-Sep-15	3	1	14	2	2	1	0	1	3	4	2
5138	202	0	1-Oct-15	16	1	14	2	1	1	0	1	3	5	1
5139	202	0	13-May-14	16	1	14	2	3	1	0	1	3	1	1
5140	202	0	14-Dec-14	14	1	14	2	1	1	0	1	3	3	2
5141	202	0	13-Apr-14	16	1	14	2	1	1	0	1	3	3	2
5142	202	0	3-Feb-14	22	1	14	2	1	1	0	1	3	5	1
5143	202	0	7-Jul-14	12	1	14	2	3	1	0	1	3	1	1
5144	202	0	29-Nov-13	18	1	14	2	1	1	0	1	3	1	2
5145	202	0	22-Jul-13	15	1	14	2	1	1	0	1	3	1	1
5146	202	0	14-Feb-13	15	1	14	2	1	1	0	1	3	1	2
5147	202	0	27-Apr-13	9	1	14	2	2	1	0	1	3	3	1
5148	202	0	15-Feb-13	21	1	14	2	1	1	0	1	3	1	1
5149	202	0	21-Jul-13	23	1	14	2	1	1	0	1	3	1	2
5150	202	0	14-Feb-13	15	1	14	2	1	1	0	1	3	4	2
5151	202	0	31-Jan-13	5	1	14	2	3	1	0	1	3	1	2
5152	202	0	11-Apr-13	20	1	14	2	1	1	0	1	3	2	2
5153	202	0	23-Sep-12	2	1	14	2	2	1	0	1	3	6	2
5154	202	0	2-Oct-12	11	1	14	2	1	1	0	1	3	2	1
5155	202	0	22-Sep-12	12	1	14	2	1	1	0	1	3	2	2
5156	202	0	16-Jul-12	21	1	14	2	1	1	0	1	3	3	2
5157	202	0	21-Feb-12	7	1	14	2	1	1	0	1	3	3	2
5158	202	0	16-Jul-12	22	1	14	2	1	1	0	1	3	3	2
5159	202	0	27-Jul-17	16	1	14	2	3	1	0	1	3	1	1
5160	202	0	11-Mar-17	15	1	14	2	1	1	0	1	3	2	2
5161	202	0	6-Mar-17	22	1	14	2	3	1	0	1	3	5	1
5162	202	0	1-May-17	13	1	14	2	3	1	0	1	3	3	1
5163	202	0	26-Nov-16	1	1	14	2	1	1	0	4	3	5	1
5164	202	0	29-Jun-13	0	1	14	2	1	1	0	4	3	1	1
5165	202	0	17-Dec-17	0	1	14	2	1	1	0	10	3	6	2
5166	202	0	26-Sep-06	14	1	14	2	1	1	0	7	3	3	2
5167	202	0	29-Apr-11	16	1	14	2	1	1	0	1	3	5	2
5168	202	0	15-Sep-11	19	1	14	2	1	1	0	1	3	4	1
5169	202	0	14-Jun-11	5	1	14	2	3	1	0	1	3	1	2
5170	202	0	17-Sep-11	9	1	14	2	1	1	0	1	3	1	1
5171	202	0	6-Nov-11	15	1	14	2	1	1	0	1	3	1	2
5172	202	0	25-Sep-11	18	1	14	2	1	1	0	1	3	1	2
5173	202	0	14-Nov-11	23	1	14	2	1	1	0	1	3	3	2
5174	202	0	6-Jul-11	15	1	14	2	1	1	0	1	3	3	1
5175	202	0	5-Sep-10	3	1	14	2	1	1	0	1	3	5	2
5176	202	0	10-Oct-10	14	1	14	2	1	1	0	1	3	4	1
5177	202	0	3-Aug-10	17	1	14	2	1	1	0	1	3	6	1
5178	202	0	5-Sep-10	3	1	14	2	1	1	0	1	3	6	1
5179	202	0	22-Jan-10	11	1	14	2	1	1	0	1	3	3	1
5180	202	0	10-Oct-10	14	1	14	2	1	1	0	1	3	4	1
5181	202	0	14-Feb-10	11	1	14	2	2	1	0	1	3	3	1
5182	202	0	1-Apr-10	22	1	14	2	1	1	0	1	3	3	2
5183	202	0	16-Jun-10	19	1	14	2	1	1	0	1	3	6	1
5184	202	0	3-May-10	0	1	14	2	1	1	0	1	3	6	2
5185	202	0	7-Apr-10	17	1	14	2	1	1	0	1	3	3	2
5186	202	0	15-Nov-10	18	1	14	2	2	1	0	1	3	2	2
5187	202	0	26-May-10	20	1	14	2	2	1	0	1	3	1	2
5188	202	0	9-Dec-09	23	1	14	2	1	1	0	1	3	7	2
5189	202	0	29-Jul-09	23	1	14	2	1	1	0	1	3	6	1
5190	202	0	3-Jun-09	12	1	14	2	3	1	0	1	3	6	2
5191	202	0	15-Aug-09	17	1	14	2	3	1	0	1	3	1	2
5192	202	0	11-Sep-09	7	1	14	2	1	1	0	1	3	2	2
5193	202	0	15-Dec-09	19	1	14	2	1	1	0	1	3	3	2
5194	202	0	25-Mar-09	14	1	14	2	1	1	0	1	3	3	1
5195	202	0	17-Nov-09	22	1	14	2	2	1	0	1	3	1	2
5196	202	0	22-Mar-09	15	1	14	2	1	1	0	1	3	1	2
5197	202	0	10-Dec-08	12	1	14	2	1	1	0	1	3	3	1
5198	202	0	20-Nov-08	18	1	14	2	1	1	0	1	3	4	2
5199	202	0	14-Aug-08	20	1	14	2	1	1	0	1	3	3	1
5200	202	0	17-May-07	7	1	14	2	1	1	0	1	3	2	2
5201	202	0	27-Sep-07	11	1	14	2	1	1	0	1	3	6	1

5202	202	0	2-Sep-07	19	1	14	2	1	1	0	1	3	1	2
5203	202	0	29-Sep-07	11	1	14	2	1	1	0	1	3	1	1
5204	202	0	12-Sep-07	19	1	14	2	1	1	0	1	3	3	1
5205	202	0	27-Jan-07	22	1	14	2	1	1	0	1	3	3	2
5206	202	0	13-Aug-07	0	1	14	2	1	1	0	1	3	3	1
5207	202	0	24-Aug-07	14	1	14	2	1	1	0	1	3	3	2
5208	202	0	22-Sep-07	13	1	14	2	1	1	0	1	3	1	2
5209	202	0	29-Oct-07	14	1	14	2	1	1	0	1	3	3	1
5210	202	0	14-Dec-06	17	1	14	2	1	1	0	1	3	4	1
5211	202	0	1-Mar-06	7	1	14	2	2	1	0	1	3	3	1
5212	202	0	9-Nov-06	18	1	14	2	1	1	0	1	3	4	1
5213	202	0	30-Dec-06	18	1	14	2	1	1	0	1	3	1	2
5214	202	0	5-Nov-08	17	1	14	2	1	1	0	1	3	6	2
5215	202	0	15-Aug-08	22	1	14	2	1	1	0	1	3	1	1
5216	202	0	28-Dec-07	6	1	14	2	1	1	0	1	3	5	1
5217	202	0	17-Sep-07	11	1	14	2	3	1	0	1	3	3	1
5218	202	0	28-Feb-12	15	1	14	2	1	1	0	4	3	7	1
5219	202	0	3-May-12	4	1	14	2	3	1	0	4	3	1	1
5220	202	0	20-Oct-10	19	1	14	2	1	1	0	4	3	1	1
5221	202	0	18-Apr-17	17	1	14	2	1	1	0	4	3	2	1
5222	202	0	21-Aug-16	21	1	14	2	1	1	0	11	3	5	2
5223	202	0	31-Oct-15	15	1	14	2	1	1	0	11	3	2	2
5224	202	0	19-Jul-14	19	1	14	2	3	1	0	11	3	3	1
5225	202	0	5-Apr-13	17	1	14	2	1	1	0	11	3	2	1
5226	202	0	20-Oct-06	19	1	14	2	1	1	0	11	3	2	1
5227	202	0	29-Dec-16	14	1	14	2	1	1	0	3	3	1	1
5228	202	0	28-Jul-16	3	1	14	2	2	1	0	3	3	1	2
5229	202	0	9-Jan-16	19	1	14	2	1	1	0	3	3	2	1
5230	202	0	14-Feb-16	15	1	14	2	3	1	0	3	3	3	1
5231	202	0	16-May-15	9	1	14	2	1	1	0	3	3	1	1
5232	202	0	13-Mar-14	23	1	14	2	2	1	0	3	3	1	1
5233	202	0	27-Sep-14	1	1	14	2	1	1	0	3	3	1	1
5234	202	0	10-Sep-14	15	1	14	2	1	1	0	3	3	3	1
5235	202	0	26-Dec-13	17	1	14	2	1	1	0	3	3	3	1
5236	202	0	11-Dec-11	4	1	14	2	1	1	0	3	3	3	2
5237	202	0	3-Jul-11	22	1	14	2	1	1	0	3	3	1	1
5238	202	0	28-Sep-10	22	1	14	2	1	1	0	3	3	5	2
5239	202	0	24-Apr-09	17	1	14	2	1	1	0	3	3	6	1
5240	202	0	11-Nov-07	17	1	14	2	1	1	0	3	3	2	2
5241	202	0	26-Jun-07	16	1	14	2	1	1	0	3	3	2	1
5242	202	0	14-Aug-17	22	1	14	2	2	1	0	3	3	2	2
5243	202	0	12-Mar-17	14	1	14	2	2	1	0	3	3	1	1
5244	202	0	9-Jan-17	17	1	14	2	3	1	0	3	3	3	1
5245	202	0	10-Mar-17	19	1	14	2	3	1	0	3	3	2	1
5246	202	0	18-Feb-17	13	1	14	2	3	1	0	3	3	1	3
5247	202	0	30-Nov-14	20	1	14	2	2	1	0	6	3	2	1
5248	202	0	12-Oct-09	12	1	14	2	1	1	0	6	3	3	1
5249	202	0	18-Feb-13	12	2	14	2	3	1	0	1	3	3	1
5250	202	0	18-Sep-12	13	2	14	2	1	1	0	1	3	2	2
5251	202	0	22-Sep-17	17	2	14	2	2	1	0	1	3	2	2
5252	202	0	6-Aug-16	0	2	14	2	1	1	0	1	3	5	1
5253	202	0	22-Aug-15	15	2	14	2	1	1	0	1	3	1	1
5254	202	0	2-Apr-14	6	2	14	2	1	1	0	1	3	2	1
5255	202	0	25-Apr-14	11	2	14	2	1	1	0	1	3	6	1
5256	202	0	13-Sep-14	15	2	14	2	1	1	0	1	3	2	1
5257	202	0	3-Feb-14	22	2	14	2	1	1	0	1	3	5	1
5258	202	0	5-Feb-14	18	2	14	2	1	1	0	1	3	1	3
5259	202	0	16-Jan-14	15	2	14	2	1	1	0	1	3	3	2
5260	202	0	11-Apr-14	2	2	14	2	1	1	0	1	3	1	1
5261	202	0	26-Mar-14	6	2	14	2	1	1	0	1	3	1	1
5262	202	0	17-Nov-13	3	2	14	2	1	1	0	1	3	1	1
5263	202	0	17-Nov-13	3	2	14	2	1	1	0	1	3	4	1
5264	202	0	24-Aug-13	0	2	14	2	1	1	0	1	3	4	2
5265	202	0	17-Apr-13	17	2	14	2	1	1	0	1	3	1	1
5266	202	0	27-Jan-13	21	2	14	2	1	1	0	1	3	3	1
5267	202	0	24-Apr-13	17	2	14	2	1	1	0	1	3	3	2
5268	202	0	8-Dec-12	13	2	14	2	1	1	0	1	3	1	1
5269	202	0	24-Oct-12	22	2	14	2	1	1	0	1	3	5	1
5270	202	0	24-Oct-12	22	2	14	2	1	1	0	1	3	5	1
5271	202	0	17-Nov-17	21	2	14	2	1	1	0	1	3	3	1
5272	202	0	29-May-17	16	2	14	2	3	1	0	1	3	3	1
5273	202	0	22-Apr-17	1	2	14	2	1	1	0	1	3	5	2
5274	202	0	10-Apr-11	15	2	14	2	1	1	0	1	3	1	1
5275	202	0	27-May-11	22	2	14	2	2	1	0	1	3	5	2
5276	202	0	3-Jun-11	22	2	14	2	1	1	0	1	3	3	2
5277	202	0	23-Jul-11	15	2	14	2	1	1	0	1	3	1	1
5278	202	0	31-Aug-11	11	2	14	2	1	1	0	1	3	5	1
5279	202	0	21-Mar-11	13	2	14	2	2	1	0	1	3	2	2
5280	202	0	14-Jun-11	18	2	14	2	1	1	0	1	3	1	2
5281	202	0	11-Nov-11	14	2	14	2	1	1	0	1	3	1	2
5282	202	0	14-Jun-11	17	2	14	2	1	1	0	1	3	1	2
5283	202	0	4-Jun-11	19	2	14	2	2	1	0	1	3	3	2
5284	202	0	11-Nov-11	14	2	14	2	1	1	0	1	3	3	2
5285	202	0	23-Jan-10	11	2	14	2	1	1	0	1	3	1	2
5286	202	0	15-May-10	3	2	14	2	1	1	0	1	3	2	1
5287	202	0	31-Jan-10	18	2	14	2	2	1	0	1	3	6	2
5288	202	0	31-Jan-10	18	2	14	2	2	1	0	1	3	6	2
5289	202	0	4-Sep-10	5	2	14	2	1	1	0	1	3	3	1
5290	202	0	14-Sep-09	6	2	14	2	1	1	0	1	3	3	2
5291	202	0	24-Jul-09	22	2	14	2	1	1	0	1	3	1	2
5292	202	0	18-Jun-09	7	2	14	2	1	1	0	1	3	1	2
5293	202	0	17-Nov-09	22	2	14	2	3	1	0	1	3	1	2
5294	202	0	8-Aug-08	18	2	14	2	1	1	0	1	3	1	2
5295	202	0	12-Nov-08	12	2	14	2	1	1	0	1	3	1	1
5296	202	0	27-Jul-07	19	2	14	2	3	1	0	1	3	6	2
5297	202	0	31-Oct-07	18	2	14	2	1	1	0	1	3	6	2
5298	202	0	14-Feb-07	17	2	14	2	1	1	0	1	3	1	2
5299	202	0	6-Jan-07	21	2	14	2	1	1	0	1	3	3	1
5300	202	0	15-Jan-07	18	2	14	2	1	1	0	1	3	3	2
5301	202	0	13-Jul-07	20	2	14	2	1	1	0	1	3	1	1
5302	202	0	27-Dec-06	13	2	14	2	1	1	0	1	3	1	2
5303	202	0	2-Sep-06	13	2	14	2	1	1	0	1	3	6	1
5304	202	0	25-Jan-06	23	2	14	2	1	1	0	1	3	1	1
5305	202	0	3-Apr-06	18	2	14	2	1	1	0	1	3	3	2
5306	202	0	16-Dec-06	2	2	14	2	1	1	0	1	3	3	1
5307	202	0	17-Jun-06	11	2	14	2	1	1	0	1	3	3	1
5308	202	0	12-Jun-06	14	2	14	2	1	1	0	1	3	1	2
5309	202	0	28-Jul-06	23	2	14	2	3	1	0	1	3	3	1
5310	202	0	16-Dec-06	13	2	14	2	1	1	0	1	3	3	1
5311	202	0	27-Oct-11	18	2	14	2	1	1	0	1	3	3	1
5312	202	0	5-Nov-08	17	2	14	2	1	1	0	1	3	6	2
5313	202	0	25-Oct-15	5	2	14	2	2	1	0	4	3	1	1
5314	202	0	27-Jan-16	16	2	14	2	3	1	0	11	3	3	1
5315	202	0	3-Sep-15	16	2	14	2	2	1	0	11	3	3	1
5316	202	0	26-Aug-09	13	2	14	2	1	1	0	11	3	3	1
5317	202	0	31-Aug-15	0	2	14	2	1	1	0	3	3	1	1
5318	202	0	1-Jan-14	0	2	14	2	1	1	0	3	3	1	1
5319	202	0	13-Dec-13	12	2	14	2	1	1	0	3	3	5	1
5320	202	0	30-Jun-11	17	2	14	2	1	1	0	3	3	2	2
5321	202	0	30-Jun-11	17	2	14	2	1	1	0	3	3	4	1
5322	202	0	27-Jul-07	12	2	14	2	1	1	0	3	3	1	1
5323	202	0	26-Jul-17	12	2	14	2	1	1	0	3	3	1	2
5324	202	0	26-Feb-17	16	2	14	2	3	1	0	3	3	3	1
5325	201	1	19-Aug-16	10	1	14	2	2	1	0	1	3	1	1

5326	201	1	23-May-16	22	1	14	2	1	1	0	1	3	5	1
5327	201	1	3-May-15	12	1	14	2	2	1	0	1	3	2	2
5328	201	1	24-Jan-14	15	1	14	2	3	1	0	1	3	3	2
5329	201	1	20-May-13	2	1	14	2	3	1	0	1	3	5	1
5330	101	1	9-May-13	17	1	14	2	1	1	0	1	3	3	1
5331	201	1	8-May-12	14	1	14	2	2	1	0	1	3	2	2
5332	101	1	17-Feb-17	21	1	14	2	1	1	0	1	3	3	1
5333	101	1	21-Aug-12	17	1	14	2	1	1	0	1	3	3	1
5334	201	1	3-Feb-15	16	1	14	2	1	1	0	1	3	4	2
5335	201	1	13-Jun-12	18	1	14	2	1	1	0	1	3	5	1
5336	201	1	4-Feb-15	20	1	14	2	1	1	0	1	3	2	1
5337	201	1	12-Oct-14	15	1	14	2	1	1	0	1	3	3	2
5338	201	1	27-Apr-12	0	1	14	2	2	1	0	1	3	1	1
5339	201	1	1-Jul-16	17	1	14	2	1	1	0	1	3	1	1
5340	201	1	31-Oct-16	18	1	14	2	1	1	0	1	3	1	1
5341	201	1	17-Nov-16	14	1	14	2	2	1	0	1	3	3	2
5342	201	1	27-Dec-16	6	1	14	2	3	1	0	1	3	2	1
5343	101	1	9-Dec-16	20	1	14	2	1	1	0	1	3	2	1
5344	201	1	3-Jun-16	6	1	14	2	1	1	0	1	3	2	1
5345	101	1	9-Sep-15	20	1	14	2	1	1	0	1	3	5	1
5346	201	1	7-Jul-15	11	1	14	2	1	1	0	1	3	5	1
5347	201	1	9-Jun-15	22	1	14	2	1	1	0	1	3	1	1
5348	201	1	28-Nov-15	17	1	14	2	2	1	0	1	3	3	1
5349	201	1	4-Dec-15	18	1	14	2	2	1	0	1	3	5	1
5350	201	1	10-Sep-14	19	1	14	2	2	1	0	1	3	5	1
5351	201	1	6-Jan-14	8	1	14	2	1	1	0	1	3	2	1
5352	201	1	29-Dec-14	19	1	14	2	1	1	0	1	3	4	1
5353	201	1	21-Nov-14	19	1	14	2	3	1	0	1	3	5	1
5354	201	1	20-Nov-14	18	1	14	2	2	1	0	1	3	5	1
5355	201	1	27-Nov-14	8	1	14	2	3	1	0	1	3	3	1
5356	201	1	3-Jan-14	13	1	14	2	1	1	0	1	3	2	1
5357	201	1	4-Oct-14	6	1	14	2	2	1	0	1	3	2	1
5358	101	1	25-Apr-14	21	1	14	2	1	1	0	1	3	4	2
5359	201	1	16-May-14	19	1	14	2	2	1	0	1	3	2	2
5360	201	1	25-Oct-13	20	1	14	2	3	1	0	1	3	5	1
5361	201	1	3-Dec-13	19	1	14	2	1	1	0	1	3	5	1
5362	101	1	13-Oct-13	18	1	14	2	1	1	0	1	3	1	2
5363	101	1	3-Apr-13	12	1	14	2	1	1	0	1	3	2	1
5364	101	1	13-Nov-13	12	1	14	2	1	1	0	1	3	2	4
5365	201	1	14-Nov-13	17	1	14	2	3	1	0	1	3	2	1
5366	201	1	12-Jun-13	17	1	14	2	1	1	0	1	3	3	1
5367	101	1	13-May-13	21	1	14	2	1	1	0	1	3	5	1
5368	201	1	30-Nov-12	20	1	14	2	3	1	0	1	3	6	1
5369	201	1	8-Jun-12	11	1	14	2	2	1	0	1	3	1	2
5370	201	1	6-Jan-12	21	1	14	2	1	1	0	1	3	1	2
5371	101	1	7-Oct-12	11	1	14	2	1	1	0	1	3	1	1
5372	201	1	26-May-12	21	1	14	2	1	1	0	1	3	3	1
5373	201	1	5-Jun-12	21	1	14	2	2	1	0	1	3	2	1
5374	201	1	5-Jul-17	20	1	14	2	1	1	0	1	3	5	1
5375	101	1	28-Apr-17	18	1	14	2	1	1	0	1	3	2	1
5376	101	1	29-Apr-17	1	1	14	2	1	1	0	1	3	1	1
5377	201	1	15-Nov-17	12	1	14	2	1	1	0	1	3	1	1
5378	201	1	16-Sep-17	18	1	14	2	2	1	0	1	3	3	1
5379	201	1	9-Mar-17	20	1	14	2	2	1	0	1	3	1	1
5380	201	1	24-Sep-16	8	1	14	2	2	1	0	1	3	1	1
5381	201	1	8-Aug-14	10	1	14	2	3	1	0	1	3	5	1
5382	201	1	5-Apr-15	9	1	14	2	3	1	0	1	3	5	1
5383	101	1	22-Nov-14	3	1	14	2	1	1	0	1	3	5	2
5384	101	1	26-Nov-12	14	1	14	2	1	1	0	1	3	3	1
5385	201	1	15-Jul-13	3	1	14	2	3	1	0	1	3	6	1
5386	201	1	19-Jun-16	11	1	14	2	3	1	0	1	3	3	1
5387	201	1	19-Jul-12	21	1	14	2	2	1	0	1	3	5	1
5388	201	1	28-Jun-16	13	1	14	2	3	1	0	1	3	5	1
5389	201	1	26-Apr-14	17	1	14	2	3	1	0	1	3	5	1
5390	201	1	30-Apr-14	22	1	14	2	3	1	0	1	3	1	1
5391	201	1	14-Jan-14	22	1	14	2	1	1	0	1	3	1	1
5392	201	1	14-Mar-12	12	1	14	2	1	1	0	1	3	5	1
5393	201	1	10-Jun-12	18	1	14	2	2	1	0	1	3	4	1
5394	101	1	8-Feb-12	21	1	14	2	2	1	0	1	3	5	2
5395	201	1	3-Mar-17	17	1	14	2	1	1	0	1	3	5	1
5396	201	1	20-Dec-17	14	1	14	2	1	1	0	1	3	5	1
5397	101	1	18-Apr-15	18	1	14	2	1	1	0	1	3	3	1
5398	101	1	16-Apr-16	7	1	14	2	1	1	0	1	3	1	2
5399	201	1	11-Jan-14	16	1	14	2	2	1	0	1	3	5	2
5400	201	1	21-Mar-16	2	1	14	2	1	1	0	1	3	3	2
5401	101	1	26-Feb-16	0	1	14	2	1	1	0	1	3	3	2
5402	101	1	5-Jun-16	22	1	14	2	1	1	0	1	3	3	2
5403	101	1	4-Jan-16	15	1	14	2	1	1	0	1	3	5	1
5404	101	1	3-Mar-14	7	1	14	2	1	1	0	1	3	2	1
5405	101	1	21-Apr-12	15	1	14	2	1	1	0	1	3	5	2
5406	101	1	18-Jun-11	16	1	14	2	1	1	0	1	3	3	1
5407	101	1	18-Jun-11	16	1	14	2	1	1	0	1	3	1	1
5408	101	1	23-Jul-11	14	1	14	2	1	1	0	1	3	1	1
5409	201	1	20-Jan-11	19	1	14	2	2	1	0	1	3	4	1
5410	201	1	14-Jun-11	22	1	14	2	1	1	0	1	3	2	1
5411	101	1	21-Sep-11	0	1	14	2	1	1	0	1	3	3	1
5412	201	1	9-Mar-11	21	1	14	2	2	1	0	1	3	4	1
5413	101	1	30-Nov-11	13	1	14	2	1	1	0	1	3	3	2
5414	201	1	6-Jan-11	21	1	14	2	2	1	0	1	3	5	1
5415	101	1	20-Jul-11	15	1	14	2	1	1	0	1	3	1	2
5416	201	1	19-Oct-11	8	1	14	2	2	1	0	1	3	1	2
5417	201	1	8-Dec-11	14	1	14	2	1	1	0	1	3	3	2
5418	201	1	4-Feb-10	16	1	14	2	1	1	0	1	3	5	1
5419	101	1	26-Jan-10	16	1	14	2	1	1	0	1	3	1	1
5420	101	1	26-Mar-10	15	1	14	2	1	1	0	1	3	1	2
5421	201	1	16-Apr-10	20	1	14	2	3	1	0	1	3	1	1
5422	101	1	2-Mar-10	12	1	14	2	1	1	0	1	3	5	1
5423	201	1	19-Sep-10	18	1	14	2	1	1	0	1	3	1	1
5424	201	1	12-May-10	14	1	14	2	3	1	0	1	3	1	1
5425	101	1	18-Mar-10	7	1	14	2	2	1	0	1	3	1	1
5426	201	1	24-Feb-09	7	1	14	2	1	1	0	1	3	1	2
5427	201	1	14-Jan-09	11	1	14	2	1	1	0	1	3	1	1
5428	101	1	28-Jul-09	20	1	14	2	1	1	0	1	3	1	1
5429	101	1	17-Jul-09	11	1	14	2	1	1	0	1	3	5	1
5430	201	1	10-Sep-08	22	1	14	2	1	1	0	1	3	1	1
5431	201	1	16-Sep-08	22	1	14	2	1	1	0	1	3	5	1
5432	101	1	16-Mar-08	16	1	14	2	1	1	0	1	3	5	1
5433	201	1	27-Sep-08	22	1	14	2	1	1	0	1	3	1	2
5434	201	1	13-Jun-08	14	1	14	2	2	1	0	1	3	5	1
5435	101	1	6-Jun-08	13	1	14	2	1	1	0	1	3	3	1
5436	201	1	20-Dec-08	19	1	14	2	1	1	0	1	3	5	1
5437	101	1	10-Jan-08	18	1	14	2	1	1	0	1	3	2	1
5438	101	1	6-Jul-07	12	1	14	2	1	1	0	1	3	5	1
5439	101	1	5-Oct-07	13	1	14	2	2	1	0	1	3	1	2
5440	101	1	25-Feb-07	23	1	14	2	1	1	0	1	3	1	1
5441	101	1	26-Dec-07	7	1	14	2	1	1	0	1	3	1	2
5442	101	1	2-Mar-07	13	1	14	2	1	1	0	1	3	1	1
5443	201	1	19-May-07	21	1	14	2	1	1	0	1	3	1	1
5444	101	1	17-Feb-06	18	1	14	2	2	1	0	1	3	3	1
5445	101	1	9-Feb-06	16	1	14	2	1	1	0	1	3	1	1
5446	101	1	13-Feb-06	19	1	14	2	1	1	0	1	3	3	1
5447	201	1	15-Dec-06	16	1	14	2	2	1	0	1	3	5	2
5448	101	1	15-Feb-06	18	1	14	2	1	1	0	1	3	1	1
5449	101	1	3-Mar-06	20	1	14	2	1	1	0	1	3	3	1

5450	101	1	4-Oct-06	18	1	14	2	3	1	0	1	3	3	1
5451	101	1	22-Mar-06	20	1	14	2	1	1	0	1	3	3	1
5452	201	1	30-Nov-06	13	1	14	2	2	1	0	1	3	3	1
5453	101	1	21-Dec-06	16	1	14	2	1	1	0	1	3	3	1
5454	101	1	24-Mar-15	14	1	14	2	1	1	0	1	3	2	1
5455	101	1	23-Jan-13	21	1	14	2	1	1	0	1	3	3	1
5456	201	1	27-Sep-11	11	1	14	2	2	1	0	1	3	6	1
5457	101	1	13-May-11	22	1	14	2	1	1	0	1	3	2	2
5458	101	1	18-May-11	19	1	14	2	1	1	0	1	3	2	1
5459	101	1	30-Oct-11	15	1	14	2	1	1	0	1	3	3	1
5460	101	1	21-Apr-10	7	1	14	2	1	1	0	1	3	5	1
5461	101	1	5-Mar-10	13	1	14	2	1	1	0	1	3	5	1
5462	201	1	20-Dec-10	22	1	14	2	3	1	0	1	3	6	1
5463	201	1	1-May-10	17	1	14	2	1	1	0	1	3	3	1
5464	101	1	30-Jan-10	2	1	14	2	1	1	0	1	3	2	1
5465	201	1	10-Mar-10	17	1	14	2	1	1	0	1	3	5	1
5466	201	1	12-Jun-09	22	1	14	2	1	1	0	1	3	1	1
5467	201	1	23-Feb-09	8	1	14	2	1	1	0	1	3	1	1
5468	201	1	23-Jul-09	10	1	14	2	2	1	0	1	3	1	1
5469	101	1	26-Jul-09	18	1	14	2	1	1	0	1	3	2	2
5470	101	1	18-Nov-08	20	1	14	2	1	1	0	1	3	2	1
5471	101	1	18-Jan-08	7	1	14	2	1	1	0	1	3	2	1
5472	101	1	6-Dec-07	12	1	14	2	1	1	0	1	3	4	1
5473	101	1	23-Jun-06	16	1	14	2	2	1	0	1	3	4	1
5474	101	1	29-May-06	17	1	14	2	1	1	0	1	3	1	1
5475	201	1	21-Jul-06	10	1	14	2	1	1	0	1	3	4	1
5476	201	1	25-Dec-06	13	1	14	2	1	1	0	1	3	3	1
5477	101	1	28-Jun-06	10	1	14	2	1	1	0	1	3	1	1
5478	101	1	7-Mar-13	20	1	14	2	1	1	0	4	3	3	1
5479	201	1	25-Nov-12	18	1	14	2	1	1	0	4	3	3	1
5480	101	1	23-Dec-09	17	1	14	2	1	1	0	4	3	3	1
5481	101	1	7-Dec-07	21	1	14	2	1	1	0	4	3	1	1
5482	201	1	21-Dec-16	19	1	14	2	2	1	0	11	3	3	1
5483	201	1	26-Nov-16	18	1	14	2	2	1	0	11	3	2	1
5484	201	1	22-Aug-15	14	1	14	2	2	1	0	11	3	3	1
5485	101	1	19-Mar-15	20	1	14	2	1	1	0	11	3	2	1
5486	201	1	24-Aug-14	13	1	14	2	2	1	0	11	3	3	1
5487	101	1	27-Jan-14	16	1	14	2	1	1	0	11	3	2	1
5488	101	1	20-Nov-14	13	1	14	2	1	1	0	11	3	3	1
5489	101	1	19-Jul-13	18	1	14	2	1	1	0	11	3	5	1
5490	101	1	14-Apr-13	19	1	14	2	1	1	0	11	3	2	1
5491	101	1	12-Oct-12	16	1	14	2	1	1	0	11	3	3	1
5492	101	1	3-Jan-12	12	1	14	2	1	1	0	11	3	4	1
5493	101	1	10-Aug-12	19	1	14	2	1	1	0	11	3	3	1
5494	201	1	9-Aug-12	17	1	14	2	3	1	0	11	3	3	1
5495	201	1	7-Jul-11	22	1	14	2	2	1	0	11	3	7	1
5496	201	1	16-Nov-11	7	1	14	2	2	1	0	11	3	2	1
5497	201	1	17-Feb-11	16	1	14	2	1	1	0	11	3	3	1
5498	101	1	20-Jul-10	9	1	14	2	2	1	0	11	3	5	1
5499	201	1	17-Jul-10	0	1	14	2	3	1	0	11	3	2	1
5500	201	1	17-Jul-09	22	1	14	2	2	1	0	11	3	3	1
5501	101	1	8-Nov-09	9	1	14	2	1	1	0	11	3	3	1
5502	101	1	12-Jul-08	17	1	14	2	1	1	0	11	3	5	2
5503	201	1	8-Nov-08	19	1	14	2	2	1	0	11	3	7	1
5504	201	1	15-Apr-07	8	1	14	2	1	1	0	11	3	2	1
5505	101	1	22-Sep-06	21	1	14	2	1	1	0	11	3	2	1
5506	201	1	20-Mar-17	14	1	14	2	1	1	0	11	3	3	1
5507	201	1	9-Dec-17	16	1	14	2	2	1	0	11	3	3	1
5508	201	1	12-May-16	17	1	14	2	1	1	0	3	3	5	1
5509	201	1	29-Jun-16	20	1	14	2	1	1	0	3	3	2	1
5510	201	1	21-Nov-16	18	1	14	2	2	1	0	3	3	2	1
5511	201	1	18-Oct-16	19	1	14	2	1	1	0	3	3	2	1
5512	101	1	18-Nov-16	2	1	14	2	1	1	0	3	3	3	1
5513	201	1	2-Mar-16	13	1	14	2	2	1	0	3	3	3	1
5514	201	1	10-Jun-16	21	1	14	2	1	1	0	3	3	3	1
5515	101	1	2-Nov-15	20	1	14	2	1	1	0	3	3	1	1
5516	101	1	18-Apr-15	18	1	14	2	1	1	0	3	3	2	1
5517	101	1	18-Jul-15	20	1	14	2	1	1	0	3	3	3	1
5518	101	1	24-May-15	15	1	14	2	1	1	0	3	3	1	1
5519	201	1	3-Nov-15	14	1	14	2	2	1	0	3	3	2	1
5520	201	1	21-Aug-15	15	1	14	2	2	1	0	3	3	2	1
5521	201	1	7-Nov-15	15	1	14	2	2	1	0	3	3	2	1
5522	101	1	14-Jun-15	21	1	14	2	1	1	0	3	3	2	1
5523	201	1	3-Nov-15	14	1	14	2	2	1	0	3	3	3	1
5524	101	1	6-Apr-14	20	1	14	2	1	1	0	3	3	2	1
5525	201	1	22-Jul-14	19	1	14	2	1	1	0	3	3	2	1
5526	101	1	25-Dec-14	15	1	14	2	1	1	0	3	3	3	1
5527	201	1	25-Feb-14	17	1	14	2	2	1	0	3	3	3	1
5528	201	1	18-Jan-13	21	1	14	2	1	1	0	3	3	6	1
5529	101	1	1-Apr-13	17	1	14	2	1	1	0	3	3	3	4
5530	101	1	25-Nov-13	20	1	14	2	1	1	0	3	3	2	1
5531	101	1	31-Oct-13	14	1	14	2	1	1	0	3	3	2	2
5532	201	1	15-Aug-13	17	1	14	2	3	1	0	3	3	2	2
5533	201	1	3-Oct-12	13	1	14	2	1	1	0	3	3	2	1
5534	201	1	16-Oct-12	12	1	14	2	2	1	0	3	3	2	1
5535	201	1	11-Nov-12	15	1	14	2	3	1	0	3	3	2	1
5536	101	1	8-Feb-12	14	1	14	2	3	1	0	3	3	2	1
5537	101	1	24-Jul-12	15	1	14	2	3	1	0	3	3	2	1
5538	101	1	20-Jul-11	11	1	14	2	1	1	0	3	3	5	1
5539	101	1	27-Apr-11	14	1	14	2	1	1	0	3	3	1	1
5540	101	1	26-Apr-11	11	1	14	2	1	1	0	3	3	2	2
5541	201	1	15-Jun-11	12	1	14	2	1	1	0	3	3	3	1
5542	101	1	22-Mar-10	12	1	14	2	1	1	0	3	3	2	1
5543	101	1	29-Nov-10	10	1	14	2	1	1	0	3	3	4	1
5544	101	1	14-Jul-10	20	1	14	2	1	1	0	3	3	2	1
5545	101	1	14-Oct-09	19	1	14	2	1	1	0	3	3	5	1
5546	101	1	22-Jul-09	9	1	14	2	1	1	0	3	3	2	1
5547	101	1	28-Sep-08	12	1	14	2	2	1	0	3	3	1	1
5548	101	1	24-Sep-08	18	1	14	2	2	1	0	3	3	2	1
5549	201	1	4-Jun-08	12	1	14	2	1	1	0	3	3	3	1
5550	101	1	10-Apr-08	20	1	14	2	1	1	0	3	3	2	1
5551	101	1	25-Jul-07	13	1	14	2	1	1	0	3	3	3	1
5552	201	1	13-Feb-07	15	1	14	2	1	1	0	3	3	1	1
5553	101	1	11-Nov-07	16	1	14	2	1	1	0	3	3	1	2
5554	201	1	3-Sep-07	7	1	14	2	1	1	0	3	3	1	1
5555	201	1	30-Oct-07	18	1	14	2	2	1	0	3	3	2	1
5556	101	1	27-Nov-07	18	1	14	2	1	1	0	3	3	1	1
5557	201	1	29-Mar-07	18	1	14	2	1	1	0	3	3	4	1
5558	201	1	5-Jan-07	18	1	14	2	1	1	0	3	3	2	1
5559	201	1	29-Oct-07	17	1	14	2	1	1	0	3	3	2	1
5560	101	1	31-Mar-06	19	1	14	2	1	1	0	3	3	1	1
5561	101	1	28-Sep-06	17	1	14	2	1	1	0	3	3	3	1
5562	101	1	11-Sep-06	19	1	14	2	1	1	0	3	3	3	1
5563	201	1	6-Apr-17	12	1	14	2	1	1	0	3	3	4	1
5564	101	1	24-Mar-17	19	1	14	2	1	1	0	3	3	3	1
5565	101	1	18-Mar-17	13	1	14	2	1	1	0	3	3	2	1
5566	101	1	20-Apr-17	9	1	14	2	1	1	0	3	3	5	2
5567	201	1	18-Oct-17	21	1	14	2	2	1	0	3	3	5	1
5568	201	1	21-Apr-17	13	1	14	2	2	1	0	3	3	4	1
5569	201	1	29-Aug-17	6	1	14	2	3	1	0	3	3	1	1
5570	101	1	12-Nov-17	19	1	14	2	1	1	0	3	3	1	1
5571	101	1	15-Jul-14	20	1	14	2	1	1	0	6	3	2	2
5572	101	1	10-Nov-11	21	1	14	2	1	1	0	6	3	3	1
5573	101	1	17-Aug-11	22	1	14	2	1	1	0	6	3	3	1

5574	101	1	19-Jul-06	20	1	14	2	3	1	0	6	3	2	2
5575	101	1	9-Jul-17	15	1	14	2	1	1	0	6	3	3	1
5576	101	1	28-Mar-17	18	1	14	2	1	1	0	6	3	2	1
5577	101	1	9-Jun-13	7	2	14	2	2	1	0	1	3	5	1
5578	201	1	1-Jun-16	18	2	14	2	2	1	0	1	3	1	1
5579	101	1	2-Dec-16	17	2	14	2	1	1	0	1	3	1	1
5580	201	1	5-Feb-15	16	2	14	2	1	1	0	1	3	2	1
5581	201	1	24-Nov-13	7	2	14	2	1	1	0	1	3	3	1
5582	101	1	24-Apr-12	18	2	14	2	1	1	0	1	3	2	1
5583	201	1	13-Nov-17	20	2	14	2	1	1	0	1	3	3	1
5584	201	1	16-Apr-17	16	2	14	2	3	1	0	1	3	3	1
5585	101	1	16-Oct-17	12	2	14	2	1	1	0	1	3	4	1
5586	101	1	8-Nov-17	7	2	14	2	1	1	0	1	3	3	1
5587	101	1	7-Jun-14	20	2	14	2	1	1	0	1	3	2	1
5588	201	1	4-Oct-12	20	2	14	2	2	1	0	1	3	1	2
5589	201	1	24-Jan-12	22	2	14	2	3	1	0	1	3	3	2
5590	201	1	15-Jul-14	10	2	14	2	1	1	0	1	3	1	1
5591	201	1	7-Jul-12	9	2	14	2	2	1	0	1	3	3	2
5592	101	1	2-Jan-14	18	2	14	2	1	1	0	1	3	3	1
5593	101	1	19-Dec-14	12	2	14	2	1	1	0	1	3	5	1
5594	101	1	30-Oct-13	8	2	14	2	2	1	0	1	3	1	1
5595	101	1	24-Nov-13	19	2	14	2	1	1	0	1	3	1	2
5596	101	1	5-Jul-13	8	2	14	2	3	1	0	1	3	3	1
5597	101	1	22-Jun-13	1	2	14	2	1	1	0	1	3	2	1
5598	101	1	17-Mar-14	7	2	14	2	1	1	0	1	3	5	1
5599	101	1	7-Jun-11	14	2	14	2	1	1	0	1	3	1	2
5600	201	1	3-Mar-11	22	2	14	2	1	1	0	1	3	4	1
5601	101	1	2-May-10	19	2	14	2	3	1	0	1	3	3	1
5602	201	1	2-Mar-10	6	2	14	2	2	1	0	1	3	2	1
5603	201	1	26-Feb-10	11	2	14	2	1	1	0	1	3	1	2
5604	201	1	16-Nov-09	18	2	14	2	1	1	0	1	3	3	1
5605	101	1	28-Aug-09	17	2	14	2	1	1	0	1	3	3	1
5606	201	1	12-Mar-09	23	2	14	2	2	1	0	1	3	6	1
5607	101	1	5-Nov-09	7	2	14	2	1	1	0	1	3	3	1
5608	101	1	2-May-09	15	2	14	2	3	1	0	1	3	3	1
5609	201	1	2-Jun-08	1	2	14	2	1	1	0	1	3	4	1
5610	101	1	19-Dec-07	6	2	14	2	1	1	0	1	3	5	1
5611	201	1	21-Sep-07	19	2	14	2	1	1	0	1	3	1	2
5612	101	1	19-Dec-07	7	2	14	2	1	1	0	1	3	5	1
5613	101	1	26-Oct-07	20	2	14	2	1	1	0	1	3	1	1
5614	201	1	27-Dec-06	19	2	14	2	1	1	0	1	3	1	1
5615	101	1	29-Jun-06	17	2	14	2	1	1	0	1	3	4	2
5616	101	1	28-Mar-15	15	2	14	2	2	1	0	1	3	1	1
5617	201	1	10-Jun-11	22	2	14	2	1	1	0	1	3	6	1
5618	201	1	31-Jul-10	19	2	14	2	3	1	0	1	3	3	1
5619	101	1	22-Oct-07	17	2	14	2	1	1	0	1	3	1	1
5620	201	1	26-Feb-07	11	2	14	2	1	1	0	1	3	3	1
5621	201	1	11-Oct-06	13	2	14	2	3	1	0	1	3	2	1
5622	101	1	1-Aug-06	22	2	14	2	1	1	0	4	3	3	1
5623	101	1	11-Dec-06	19	2	14	2	1	1	0	4	3	2	1
5624	201	1	6-Jul-16	18	2	14	2	1	1	0	11	3	3	1
5625	101	1	2-Jan-15	15	2	14	2	1	1	0	11	3	3	1
5626	101	1	19-Jan-15	13	2	14	2	1	1	0	11	3	3	1
5627	101	1	25-Dec-14	21	2	14	2	1	1	0	11	3	5	1
5628	101	1	16-Oct-14	14	2	14	2	1	1	0	11	3	3	1
5629	101	1	4-Jun-13	17	2	14	2	1	1	0	11	3	2	2
5630	201	1	8-Nov-13	12	2	14	2	3	1	0	11	3	1	1
5631	101	1	24-Aug-13	21	2	14	2	1	1	0	11	3	3	1
5632	201	1	19-Oct-11	12	2	14	2	1	1	0	11	3	3	1
5633	101	1	3-Feb-10	16	2	14	2	1	1	0	11	3	2	1
5634	101	1	9-May-07	12	2	14	2	1	1	0	11	3	2	1
5635	201	1	4-Jan-17	17	2	14	2	1	1	0	11	3	2	1
5636	101	1	12-Dec-16	11	2	14	2	1	1	0	3	3	3	1
5637	201	1	3-Aug-15	5	2	14	2	1	1	0	3	3	4	1
5638	101	1	3-Jan-15	21	2	14	2	1	1	0	3	3	1	1
5639	201	1	16-Oct-15	22	2	14	2	2	1	0	3	3	3	1
5640	201	1	6-Jan-15	18	2	14	2	2	1	0	3	3	2	1
5641	101	1	16-Apr-15	10	2	14	2	1	1	0	3	3	3	1
5642	101	1	12-Aug-14	9	2	14	2	1	1	0	3	3	2	1
5643	101	1	31-Dec-14	16	2	14	2	1	1	0	3	3	2	1
5644	101	1	22-Mar-13	19	2	14	2	1	1	0	3	3	1	4
5645	101	1	1-Jun-13	10	2	14	2	2	1	0	3	3	3	1
5646	101	1	14-Nov-13	16	2	14	2	1	1	0	3	3	2	1
5647	101	1	29-Jul-11	18	2	14	2	3	1	0	3	3	5	1
5648	201	1	23-Mar-10	11	2	14	2	1	1	0	3	3	1	1
5649	201	1	20-Feb-09	17	2	14	2	1	1	0	3	3	5	1
5650	201	1	14-May-08	12	2	14	2	1	1	0	3	3	5	1
5651	201	1	3-Jun-08	18	2	14	2	2	1	0	3	3	3	1
5652	201	1	11-Oct-07	20	2	14	2	3	1	0	3	3	2	2
5653	101	1	13-Apr-07	19	2	14	2	1	1	0	3	3	3	1
5654	101	1	19-Nov-07	17	2	14	2	1	1	0	3	3	2	1
5655	101	1	8-Jan-17	10	2	14	2	1	1	0	3	3	3	2
5656	201	1	17-Jul-17	16	2	14	2	2	1	0	3	3	2	1
5657	201	1	10-May-15	16	2	14	2	1	1	0	6	3	3	1
5658	202	0	19-Jul-13	18	1	15	2	3	1	0	1	3	1	1
5659	202	0	15-Nov-13	20	1	15	2	2	1	0	1	3	1	1
5660	202	0	30-Sep-17	21	1	15	2	2	1	0	1	3	5	1
5661	202	0	28-Mar-17	21	1	15	2	2	1	0	1	3	4	1
5662	202	0	1-Mar-14	20	1	15	2	1	1	0	1	3	5	2
5663	202	0	10-Jul-12	15	1	15	2	2	1	0	1	3	6	2
5664	202	0	28-Jun-13	22	1	15	2	1	1	0	1	3	5	1
5665	202	0	23-Jul-16	22	1	15	2	1	1	0	1	3	3	1
5666	202	0	17-Aug-14	22	1	15	2	1	1	0	1	3	2	1
5667	202	0	8-Jan-14	21	1	15	2	1	1	0	1	3	2	2
5668	202	0	14-Dec-13	0	1	15	2	1	1	0	1	3	2	1
5669	202	0	20-Sep-13	19	1	15	2	3	1	0	1	3	2	1
5670	202	0	18-Jul-15	21	1	15	2	3	1	0	1	3	1	1
5671	202	0	25-May-12	6	1	15	2	1	1	0	1	3	3	1
5672	202	0	18-Jul-15	19	1	15	2	1	1	0	1	3	1	1
5673	202	0	23-Mar-13	23	1	15	2	1	1	0	1	3	1	2
5674	202	0	2-Dec-15	14	1	15	2	3	1	0	1	3	3	1
5675	202	0	15-Oct-14	7	1	15	2	1	1	0	1	3	3	1
5676	202	0	3-Mar-12	19	1	15	2	1	1	0	1	3	2	1
5677	202	0	16-Apr-14	19	1	15	2	3	1	0	1	3	1	1
5678	202	0	15-Mar-13	19	1	15	2	2	1	0	1	3	4	2
5679	202	0	20-Jul-13	12	1	15	2	1	1	0	1	3	3	1
5680	202	0	15-Sep-12	16	1	15	2	1	1	0	1	3	6	1
5681	202	0	29-Aug-15	22	1	15	2	3	1	0	1	3	3	1
5682	202	0	29-Sep-15	11	1	15	2	1	1	0	1	3	6	1
5683	202	0	15-Mar-13	20	1	15	2	3	1	0	1	3	3	1
5684	202	0	21-Sep-13	2	1	15	2	1	1	0	1	3	5	1
5685	202	0	21-Sep-13	1	1	15	2	1	1	0	1	3	5	2
5686	202	0	2-Feb-12	15	1	15	2	1	1	0	1	3	2	2
5687	202	0	7-Nov-14	18	1	15	2	1	1	0	1	3	1	2
5688	202	0	23-Dec-13	7	1	15	2	2	1	0	1	3	5	1
5689	202	0	23-Dec-13	7	1	15	2	2	1	0	1	3	5	1
5690	202	0	24-Aug-16	15	1	15	2	1	1	0	1	3	3	2
5691	202	0	4-May-16	20	1	15	2	1	1	0	1	3	5	1
5692	202	0	2-Jun-16	0	1	15	2	1	1	0	1	3	1	1
5693	202	0	27-Aug-16	23	1	15	2	3	1	0	1	3	1	1
5694	202	0	2-Jun-16	0	1	15	2	3	1	0	1	3	1	1
5695	202	0	20-Jun-16	23	1	15	2	2	1	0	1	3	3	1
5696	202	0	10-Apr-16	0	1	15	2	1	1	0	1	3	2	1
5697	202	0	7-Apr-16	7	1	15	2	3	1	0	1	3	3	1

5698	202	0	3-Jul-15	1	1	15	2	1	1	0	1	3	3	2
5699	202	0	13-Jun-15	1	1	15	2	1	1	0	1	3	4	1
5700	202	0	11-Oct-15	16	1	15	2	1	1	0	1	3	3	1
5701	202	0	22-Feb-14	19	1	15	2	1	1	0	1	3	2	1
5702	202	0	31-Jan-14	11	1	15	2	1	1	0	1	3	4	1
5703	202	0	14-Dec-14	14	1	15	2	1	1	0	1	3	4	1
5704	202	0	14-Jun-14	4	1	15	2	1	1	0	1	3	1	1
5705	202	0	24-Sep-14	19	1	15	2	3	1	0	1	3	5	1
5706	202	0	3-Feb-14	22	1	15	2	1	1	0	1	3	5	2
5707	202	0	10-Aug-14	15	1	15	2	1	1	0	1	3	1	2
5708	202	0	2-Nov-13	0	1	15	2	1	1	0	1	3	1	2
5709	202	0	27-Apr-13	22	1	15	2	1	1	0	1	3	1	2
5710	202	0	24-Jan-13	18	1	15	2	2	1	0	1	3	3	2
5711	202	0	3-Mar-13	17	1	15	2	1	1	0	1	3	1	1
5712	202	0	1-Sep-12	19	1	15	2	1	1	0	1	3	6	1
5713	202	0	23-Sep-12	2	1	15	2	2	1	0	1	3	6	2
5714	202	0	24-Jun-12	2	1	15	2	1	1	0	1	3	5	2
5715	202	0	24-Jun-12	2	1	15	2	1	1	0	1	3	5	2
5716	202	0	6-Jun-12	20	1	15	2	1	1	0	1	3	5	2
5717	202	0	10-Jul-12	15	1	15	2	2	1	0	1	3	1	2
5718	202	0	25-May-12	23	1	15	2	1	1	0	1	3	6	1
5719	202	0	16-Sep-12	21	1	15	2	3	1	0	1	3	3	1
5720	202	0	21-Sep-12	18	1	15	2	3	1	0	1	3	1	1
5721	202	0	15-Sep-12	16	1	15	2	1	1	0	1	3	3	1
5722	202	0	15-Sep-12	15	1	15	2	1	1	0	1	3	3	2
5723	202	0	1-Aug-12	19	1	15	2	1	1	0	1	3	3	1
5724	202	0	27-Feb-12	18	1	15	2	2	1	0	1	3	5	2
5725	202	0	3-Jan-12	19	1	15	2	1	1	0	1	3	6	2
5726	202	0	17-Jan-12	23	1	15	2	1	1	0	1	3	3	1
5727	202	0	8-Jan-12	21	1	15	2	1	1	0	1	3	3	1
5728	202	0	16-Jul-17	3	1	15	2	1	1	0	1	3	1	2
5729	202	0	11-Jan-17	18	1	15	2	1	1	0	1	3	3	1
5730	202	0	5-Aug-17	20	1	15	2	3	1	0	1	3	1	1
5731	202	0	13-Jun-13	3	1	15	2	1	1	0	3	3	3	1
5732	202	0	23-Sep-12	12	1	15	2	1	1	0	1	3	5	2
5733	202	0	18-May-16	23	1	15	2	1	1	0	1	3	3	2
5734	202	0	17-Nov-10	0	1	15	2	3	1	0	10	3	7	4
5735	202	0	1-Oct-10	3	1	15	2	1	1	0	13	3	1	1
5736	202	0	13-Feb-13	14	1	15	2	3	1	0	1	3	3	1
5737	202	0	27-Feb-13	23	1	15	2	1	1	0	1	3	4	1
5738	202	0	20-Mar-12	20	1	15	2	1	1	0	1	3	6	2
5739	202	0	23-Sep-12	2	1	15	2	1	1	0	1	3	6	2
5740	202	0	20-May-12	16	1	15	2	1	1	0	1	3	1	2
5741	202	0	27-Aug-11	3	1	15	2	1	1	0	1	3	3	2
5742	202	0	15-May-11	16	1	15	2	1	1	0	1	3	5	1
5743	202	0	10-Sep-11	1	1	15	2	1	1	0	1	3	5	1
5744	202	0	24-Oct-11	20	1	15	2	1	1	0	1	3	1	1
5745	202	0	25-Sep-11	18	1	15	2	1	1	0	1	3	1	2
5746	202	0	9-Feb-11	1	1	15	2	1	1	0	1	3	1	2
5747	202	0	19-Jan-11	22	1	15	2	1	1	0	1	3	1	2
5748	202	0	2-Aug-11	16	1	15	2	3	1	0	1	3	1	2
5749	202	0	16-Apr-11	20	1	15	2	1	1	0	1	3	1	1
5750	202	0	12-Mar-11	6	1	15	2	1	1	0	1	3	1	1
5751	202	0	2-Jul-11	20	1	15	2	3	1	0	1	3	3	2
5752	202	0	11-Jun-10	13	1	15	2	1	1	0	1	3	6	1
5753	202	0	11-Jun-10	13	1	15	2	1	1	0	1	3	6	1
5754	202	0	12-Apr-10	1	1	15	2	1	1	0	1	3	6	1
5755	202	0	25-Jun-10	13	1	15	2	1	1	0	1	3	6	1
5756	202	0	23-Oct-10	22	1	15	2	1	1	0	1	3	1	2
5757	202	0	2-Dec-10	20	1	15	2	1	1	0	1	3	1	2
5758	202	0	15-Mar-10	20	1	15	2	1	1	0	1	3	1	1
5759	202	0	4-Apr-10	17	1	15	2	1	1	0	1	3	1	1
5760	202	0	18-Nov-10	14	1	15	2	1	1	0	1	3	1	1
5761	202	0	19-Feb-10	19	1	15	2	1	1	0	1	3	3	1
5762	202	0	15-Feb-10	18	1	15	2	1	1	0	1	3	6	2
5763	202	0	5-Sep-10	3	1	15	2	1	1	0	1	3	2	1
5764	202	0	6-Nov-10	20	1	15	2	1	1	0	1	3	1	1
5765	202	0	22-Nov-10	6	1	15	2	2	1	0	1	3	6	2
5766	202	0	30-Sep-10	18	1	15	2	1	1	0	1	3	1	1
5767	202	0	30-May-09	1	1	15	2	1	1	0	1	3	3	1
5768	202	0	1-Oct-09	2	1	15	2	1	1	0	1	3	5	1
5769	202	0	1-Oct-09	1	1	15	2	1	1	0	1	3	6	2
5770	202	0	11-Nov-09	19	1	15	2	1	1	0	1	3	6	2
5771	202	0	17-May-09	1	1	15	2	1	1	0	1	3	6	2
5772	202	0	24-Jul-09	22	1	15	2	1	1	0	1	3	4	2
5773	202	0	2-May-09	21	1	15	2	1	1	0	1	3	2	1
5774	202	0	4-Dec-09	18	1	15	2	1	1	0	1	3	1	2
5775	202	0	1-Oct-09	1	1	15	2	1	1	0	1	3	1	2
5776	202	0	10-Jun-09	10	1	15	2	1	1	0	1	3	3	2
5777	202	0	25-Dec-09	23	1	15	2	1	1	0	1	3	3	1
5778	202	0	28-Nov-09	21	1	15	2	2	1	0	1	3	3	2
5779	202	0	5-Jul-08	12	1	15	2	2	1	0	1	3	3	2
5780	202	0	23-Jul-08	22	1	15	2	1	1	0	1	3	6	1
5781	202	0	10-Dec-08	12	1	15	2	1	1	0	1	3	3	1
5782	202	0	30-Oct-08	17	1	15	2	1	1	0	1	3	2	2
5783	202	0	6-Mar-07	7	1	15	2	1	1	0	1	3	5	1
5784	202	0	18-Jun-07	13	1	15	2	1	1	0	1	3	4	1
5785	202	0	18-Jun-07	14	1	15	2	1	1	0	1	3	7	1
5786	202	0	25-Mar-07	10	1	15	2	1	1	0	1	3	6	1
5787	202	0	7-Nov-07	18	1	15	2	1	1	0	1	3	6	1
5788	202	0	9-Apr-07	14	1	15	2	1	1	0	1	3	1	1
5789	202	0	21-Jul-07	0	1	15	2	1	1	0	1	3	3	1
5790	202	0	25-Mar-07	10	1	15	2	1	1	0	1	3	6	1
5791	202	0	16-Nov-07	17	1	15	2	1	1	0	1	3	2	2
5792	202	0	29-Oct-07	14	1	15	2	1	1	0	1	3	2	1
5793	202	0	1-Dec-07	11	1	15	2	1	1	0	1	3	6	2
5794	202	0	13-Apr-07	10	1	15	2	2	1	0	1	3	2	1
5795	202	0	29-Jan-06	16	1	15	2	1	1	0	1	3	6	2
5796	202	0	21-Jan-06	17	1	15	2	1	1	0	1	3	1	2
5797	202	0	19-Mar-06	3	1	15	2	3	1	0	1	3	1	1
5798	202	0	31-Jan-06	0	1	15	2	1	1	0	1	3	1	2
5799	202	0	27-Jul-06	20	1	15	2	1	1	0	1	3	4	1
5800	202	0	30-Jan-06	14	1	15	2	1	1	0	1	3	7	1
5801	202	0	30-Dec-06	18	1	15	2	1	1	0	1	3	1	2
5802	202	0	31-Oct-06	23	1	15	2	1	1	0	1	3	1	2
5803	202	0	25-Nov-06	15	1	15	2	1	1	0	1	3	3	2
5804	202	0	3-Jun-09	12	1	15	2	1	1	0	1	3	6	1
5805	202	0	4-Apr-09	2	1	15	2	1	1	0	1	3	1	2
5806	202	0	5-Nov-08	17	1	15	2	1	1	0	1	3	6	2
5807	202	0	3-Jul-07	19	1	15	2	1	1	0	1	3	5	1
5808	202	0	6-Mar-07	21	1	15	2	1	1	0	1	3	3	1
5809	202	0	12-Jun-15	15	1	15	2	3	1	0	4	3	1	1
5810	202	0	2-Mar-11	17	1	15	2	1	1	0	4	3	6	1
5811	202	0	4-Jul-08	0	1	15	2	1	1	0	4	3	6	1
5812	202	0	10-Oct-08	18	1	15	2	1	1	0	4	3	1	1
5813	202	0	8-Nov-06	14	1	15	2	3	1	0	4	3	1	1
5814	202	0	7-Dec-17	10	1	15	2	1	1	0	4	3	1	1
5815	202	0	22-Sep-17	20	1	15	2	2	1	0	4	3	5	1
5816	202	0	13-Sep-17	15	1	15	2	2	1	0	4	3	3	1
5817	202	0	14-Aug-17	20	1	15	2	2	1	0	4	3	2	2
5818	202	0	23-Mar-17	17	1	15	2	3	1	0	4	3	1	1
5819	202	0	28-May-14	22	1	15	2	1	1	0	11	3	3	1
5820	202	0	23-May-14	23	1	15	2	1	1	0	11	3	2	2
5821	202	0	2-May-13	14	1	15	2	1	1	0	11	3	3	1

5822	202	0	12-Mar-13	12	1	15	2	1	1	0	11	3	3	1
5823	202	0	15-Apr-12	2	1	15	2	1	1	0	11	3	3	1
5824	202	0	9-Jan-11	17	1	15	2	1	1	0	11	3	1	1
5825	202	0	24-Jun-11	18	1	15	2	1	1	0	11	3	3	1
5826	202	0	24-Jun-06	12	1	15	2	1	1	0	11	3	7	1
5827	202	0	2-Aug-17	16	1	15	2	2	1	0	11	3	7	1
5828	202	0	31-Dec-17	20	1	15	2	1	1	0	11	3	3	1
5829	202	0	30-May-17	0	1	15	2	1	1	0	11	3	2	1
5830	202	0	16-Feb-16	18	1	15	2	3	1	0	3	3	5	1
5831	202	0	13-Jul-16	21	1	15	2	1	1	0	3	3	3	1
5832	202	0	13-May-16	0	1	15	2	1	1	0	3	3	5	1
5833	202	0	24-May-15	18	1	15	2	1	1	0	3	3	1	1
5834	202	0	14-Aug-15	22	1	15	2	1	1	0	3	3	2	1
5835	202	0	12-Oct-14	2	1	15	2	1	1	0	3	3	2	2
5836	202	0	24-Oct-14	2	1	15	2	2	1	0	3	3	5	1
5837	202	0	29-Jan-14	2	1	15	2	1	1	0	3	3	5	2
5838	202	0	26-May-14	17	1	15	2	1	1	0	3	3	1	1
5839	202	0	5-Oct-14	1	1	15	2	1	1	0	3	3	2	1
5840	202	0	19-Feb-14	15	1	15	2	1	1	0	3	3	2	1
5841	202	0	3-Mar-14	17	1	15	2	3	1	0	3	3	2	1
5842	202	0	16-Feb-13	11	1	15	2	3	1	0	3	3	1	1
5843	202	0	26-Dec-13	23	1	15	2	2	1	0	3	3	1	2
5844	202	0	13-Apr-10	12	1	15	2	1	1	0	3	3	1	2
5845	202	0	3-Apr-10	7	1	15	2	1	1	0	3	3	1	1
5846	202	0	7-Jun-10	22	1	15	2	1	1	0	3	3	2	1
5847	202	0	18-Nov-10	21	1	15	2	1	1	0	3	3	3	1
5848	202	0	1-Apr-09	17	1	15	2	1	1	0	3	3	7	1
5849	202	0	17-Apr-09	23	1	15	2	2	1	0	3	3	2	2
5850	202	0	22-Oct-08	19	1	15	2	1	1	0	3	3	2	2
5851	202	0	15-Aug-08	18	1	15	2	2	1	0	3	3	1	2
5852	202	0	23-Aug-07	17	1	15	2	2	1	0	3	3	3	2
5853	202	0	6-Nov-07	12	1	15	2	1	1	0	3	3	1	1
5854	202	0	7-Sep-07	21	1	15	2	1	1	0	3	3	1	1
5855	202	0	3-Oct-17	16	1	15	2	3	1	0	3	3	5	1
5856	202	0	3-Aug-17	3	1	15	2	1	1	0	3	3	6	1
5857	202	0	10-Jul-15	20	1	15	2	1	1	0	6	3	1	1
5858	202	0	10-Feb-12	10	1	15	2	1	1	0	6	3	3	1
5859	202	0	19-Jun-10	12	1	15	2	1	1	0	6	3	1	1
5860	202	0	15-Dec-08	12	1	15	2	1	1	0	6	3	3	1
5861	202	0	2-Dec-17	22	1	15	2	3	1	0	6	3	3	1
5862	202	0	27-Oct-15	9	2	15	2	1	1	0	1	3	1	1
5863	202	0	23-Apr-17	18	2	15	2	1	1	0	1	3	1	1
5864	202	0	25-Nov-16	19	2	15	2	1	1	0	1	3	1	2
5865	202	0	25-Dec-14	18	2	15	2	1	1	0	1	3	1	2
5866	202	0	25-Mar-17	8	2	15	2	1	1	0	1	3	3	1
5867	202	0	14-Oct-14	12	2	15	2	1	1	0	1	3	4	1
5868	202	0	14-Feb-12	17	2	15	2	1	1	0	1	3	2	2
5869	202	0	8-May-13	10	2	15	2	1	1	0	1	3	3	1
5870	202	0	25-May-12	6	2	15	2	1	1	0	1	3	5	2
5871	202	0	25-May-12	6	2	15	2	1	1	0	1	3	5	1
5872	202	0	25-May-12	6	2	15	2	1	1	0	1	3	5	1
5873	202	0	25-May-12	6	2	15	2	1	1	0	1	3	2	1
5874	202	0	25-May-12	6	2	15	2	1	1	0	1	3	1	1
5875	202	0	25-May-12	6	2	15	2	1	1	0	1	3	1	1
5876	202	0	25-Jun-14	15	2	15	2	1	1	0	1	3	3	1
5877	202	0	3-Jan-16	13	2	15	2	1	1	0	1	3	1	1
5878	202	0	3-Mar-12	19	2	15	2	1	1	0	1	3	1	1
5879	202	0	3-Mar-12	19	2	15	2	1	1	0	1	3	2	1
5880	202	0	19-Jun-14	8	2	15	2	2	1	0	1	3	4	1
5881	202	0	12-Jul-16	7	2	15	2	1	1	0	1	3	3	1
5882	202	0	21-Jul-13	0	2	15	2	1	1	0	1	3	3	1
5883	202	0	18-Jan-14	1	2	15	2	2	1	0	1	3	1	2
5884	202	0	23-Mar-13	9	2	15	2	1	1	0	1	3	3	1
5885	202	0	30-Jul-16	0	2	15	2	2	1	0	1	3	5	2
5886	202	0	10-Jun-16	4	2	15	2	1	1	0	1	3	1	2
5887	202	0	17-Apr-16	5	2	15	2	3	1	0	1	3	1	1
5888	202	0	28-Feb-16	17	2	15	2	2	1	0	1	3	3	1
5889	202	0	4-Oct-16	1	2	15	2	3	1	0	1	3	1	1
5890	202	0	13-Jun-14	7	2	15	2	1	1	0	1	3	4	1
5891	202	0	13-Sep-14	18	2	15	2	3	1	0	1	3	4	1
5892	202	0	21-Jul-14	20	2	15	2	1	1	0	1	3	2	1
5893	202	0	5-Jan-14	21	2	15	2	2	1	0	1	3	1	2
5894	202	0	4-Jun-13	6	2	15	2	2	1	0	1	3	5	2
5895	202	0	28-Jan-13	13	2	15	2	2	1	0	1	3	1	2
5896	202	0	30-Apr-13	22	2	15	2	1	1	0	1	3	1	1
5897	202	0	18-Sep-13	21	2	15	2	3	1	0	1	3	5	1
5898	202	0	26-Jun-12	11	2	15	2	1	1	0	1	3	5	1
5899	202	0	10-Jul-12	16	2	15	2	2	1	0	1	3	1	2
5900	202	0	24-Oct-12	22	2	15	2	1	1	0	1	3	5	1
5901	202	0	26-Jan-12	15	2	15	2	1	1	0	1	3	5	2
5902	202	0	24-Jan-12	1	2	15	2	1	1	0	1	3	2	2
5903	202	0	3-Feb-12	0	2	15	2	1	1	0	1	3	3	2
5904	202	0	15-Aug-12	8	2	15	2	1	1	0	1	3	5	1
5905	202	0	15-May-17	13	2	15	2	1	1	0	1	3	2	1
5906	202	0	22-Oct-07	18	2	15	2	1	1	0	13	3	1	1
5907	202	0	11-Oct-11	12	2	15	2	1	1	0	1	3	6	2
5908	202	0	9-Mar-11	11	2	15	2	1	1	0	1	3	5	1
5909	202	0	26-Jul-11	0	2	15	2	1	1	0	1	3	5	2
5910	202	0	5-Feb-11	16	2	15	2	3	1	0	1	3	2	1
5911	202	0	7-Feb-11	11	2	15	2	1	1	0	1	3	3	1
5912	202	0	20-Sep-10	18	2	15	2	1	1	0	1	3	1	2
5913	202	0	9-Dec-10	13	2	15	2	1	1	0	1	3	5	1
5914	202	0	7-Mar-10	17	2	15	2	1	1	0	1	3	1	2
5915	202	0	10-Dec-10	20	2	15	2	1	1	0	1	3	1	1
5916	202	0	25-Jun-10	17	2	15	2	2	1	0	1	3	1	2
5917	202	0	10-Feb-10	15	2	15	2	2	1	0	1	3	1	1
5918	202	0	2-Aug-10	4	2	15	2	1	1	0	1	3	3	1
5919	202	0	10-Sep-10	15	2	15	2	1	1	0	1	3	3	1
5920	202	0	1-Jan-10	21	2	15	2	1	1	0	1	3	3	2
5921	202	0	12-Jul-10	1	2	15	2	1	1	0	1	3	6	1
5922	202	0	14-Apr-10	12	2	15	2	1	1	0	1	3	1	2
5923	202	0	14-Feb-10	11	2	15	2	3	1	0	1	3	1	2
5924	202	0	2-May-09	21	2	15	2	1	1	0	1	3	5	1
5925	202	0	16-Apr-09	4	2	15	2	1	1	0	1	3	6	2
5926	202	0	2-May-09	21	2	15	2	1	1	0	1	3	3	1
5927	202	0	22-Mar-09	11	2	15	2	1	1	0	1	3	3	1
5928	202	0	13-Sep-09	15	2	15	2	1	1	0	1	3	3	1
5929	202	0	6-Mar-08	7	2	15	2	1	1	0	1	3	4	2
5930	202	0	8-Jan-08	7	2	15	2	1	1	0	1	3	3	1
5931	202	0	13-Jun-08	18	2	15	2	1	1	0	1	3	6	1
5932	202	0	19-Jul-08	22	2	15	2	2	1	0	1	3	1	1
5933	202	0	5-Nov-08	17	2	15	2	1	1	0	1	3	6	2
5934	202	0	14-Apr-08	18	2	15	2	3	1	0	1	3	1	1
5935	202	0	15-Oct-08	22	2	15	2	1	1	0	1	3	2	1
5936	202	0	15-Oct-08	22	2	15	2	1	1	0	1	3	3	2
5937	202	0	10-Oct-08	15	2	15	2	1	1	0	1	3	3	1
5938	202	0	17-Jul-08	0	2	15	2	1	1	0	1	3	3	1
5939	202	0	7-Sep-08	20	2	15	2	1	1	0	1	3	3	2
5940	202	0	26-Oct-08	16	2	15	2	1	1	0	1	3	3	2
5941	202	0	29-Jun-07	0	2	15	2	1	1	0	1	3	3	1
5942	202	0	14-Feb-07	17	2	15	2	1	1	0	1	3	2	1
5943	202	0	12-May-07	14	2	15	2	2	1	0	1	3	4	1
5944	202	0	24-Dec-07	7	2	15	2	1	1	0	1	3	3	2
5945	202	0	11-Jun-07	19	2	15	2	2	1	0	1	3	1	1

5946	202	0	21-Dec-07	13	2	15	2	1	1	0	1	3	4	1
5947	202	0	15-Jul-06	12	2	15	2	1	1	0	1	3	1	1
5948	202	0	18-Jul-06	14	2	15	2	1	1	0	1	3	1	2
5949	202	0	10-Mar-06	17	2	15	2	1	1	0	1	3	6	1
5950	202	0	21-Aug-10	18	2	15	2	1	1	0	1	3	1	1
5951	202	0	22-Apr-08	19	2	15	2	1	1	0	1	3	3	2
5952	202	0	22-Sep-06	5	2	15	2	1	1	0	4	3	1	1
5953	202	0	15-Jul-16	19	2	15	2	1	1	0	3	3	3	1
5954	202	0	13-Oct-13	11	2	15	2	1	1	0	3	3	1	1
5955	202	0	1-Apr-11	17	2	15	2	1	1	0	3	3	2	1
5956	202	0	27-Sep-10	13	2	15	2	1	1	0	3	3	3	1
5957	202	0	7-Jul-09	0	2	15	2	1	1	0	3	3	3	2
5958	202	0	30-Oct-08	21	2	15	2	1	1	0	3	3	6	2
5959	202	0	17-Jul-07	10	2	15	2	1	1	0	3	3	7	1
5960	101	1	25-Nov-12	10	1	15	2	1	1	0	1	3	3	2
5961	101	1	18-Oct-16	12	1	15	2	2	1	0	1	3	3	1
5962	201	1	24-Aug-16	19	1	15	2	1	1	0	1	3	1	1
5963	201	1	28-Aug-14	20	1	15	2	1	1	0	1	3	5	1
5964	201	1	20-Mar-13	17	1	15	2	2	1	0	1	3	2	1
5965	101	1	24-Oct-12	20	1	15	2	1	1	0	1	3	5	2
5966	101	1	9-May-12	22	1	15	2	1	1	0	1	3	2	2
5967	201	1	5-Sep-12	12	1	15	2	1	1	0	1	3	4	1
5968	101	1	16-Oct-15	7	1	15	2	1	1	0	1	3	2	1
5969	201	1	2-Jul-15	19	1	15	2	3	1	0	1	3	6	1
5970	201	1	25-Oct-13	21	1	15	2	1	1	0	1	3	5	2
5971	201	1	14-May-12	13	1	15	2	2	1	0	1	3	3	1
5972	201	1	14-Oct-16	20	1	15	2	1	1	0	1	3	5	1
5973	201	1	13-Jul-16	10	1	15	2	2	1	0	1	3	2	1
5974	201	1	19-Dec-16	12	1	15	2	1	1	0	1	3	3	1
5975	201	1	16-Feb-16	22	1	15	2	3	1	0	1	3	3	1
5976	201	1	5-Dec-16	18	1	15	2	1	1	0	1	3	2	1
5977	101	1	23-Jan-15	19	1	15	2	1	1	0	1	3	1	1
5978	201	1	23-Jun-15	18	1	15	2	1	1	0	1	3	5	1
5979	201	1	14-Jan-15	20	1	15	2	2	1	0	1	3	3	1
5980	201	1	5-Oct-15	16	1	15	2	1	1	0	1	3	5	1
5981	201	1	1-Feb-15	18	1	15	2	1	1	0	1	3	1	1
5982	201	1	4-Oct-15	21	1	15	2	1	1	0	1	3	1	1
5983	201	1	7-May-15	17	1	15	2	2	1	0	1	3	2	1
5984	201	1	14-Nov-15	11	1	15	2	3	1	0	1	3	2	2
5985	201	1	23-Jan-15	15	1	15	2	1	1	0	1	3	2	1
5986	101	1	12-May-14	20	1	15	2	1	1	0	1	3	5	1
5987	101	1	9-Aug-14	21	1	15	2	1	1	0	1	3	5	1
5988	101	1	16-Apr-14	12	1	15	2	1	1	0	1	3	4	2
5989	201	1	3-Jun-14	0	1	15	2	3	1	0	1	3	5	1
5990	201	1	4-Oct-14	9	1	15	2	1	1	0	1	3	1	1
5991	101	1	22-Feb-14	11	1	15	2	1	1	0	1	3	5	1
5992	201	1	8-Jan-14	15	1	15	2	2	1	0	1	3	1	1
5993	101	1	3-Oct-14	9	1	15	2	2	1	0	1	3	1	1
5994	201	1	12-Apr-14	11	1	15	2	2	1	0	1	3	3	1
5995	101	1	26-Feb-14	18	1	15	2	1	1	0	1	3	4	1
5996	201	1	1-Nov-14	19	1	15	2	2	1	0	1	3	3	2
5997	201	1	19-Sep-14	18	1	15	2	2	1	0	1	3	3	1
5998	201	1	20-Apr-13	12	1	15	2	2	1	0	1	3	6	1
5999	201	1	2-Nov-13	0	1	15	2	1	1	0	1	3	1	1
6000	201	1	16-Nov-13	11	1	15	2	3	1	0	1	3	3	1
6001	101	1	8-Jan-13	17	1	15	2	1	1	0	1	3	2	1
6002	201	1	3-Oct-13	10	1	15	2	1	1	0	1	3	3	1
6003	101	1	23-Jul-13	22	1	15	2	1	1	0	1	3	2	1
6004	201	1	2-May-13	19	1	15	2	3	1	0	1	3	3	1
6005	201	1	13-Aug-12	18	1	15	2	2	1	0	1	3	3	4
6006	101	1	17-Mar-12	18	1	15	2	1	1	0	1	3	2	1
6007	201	1	4-Jan-17	12	1	15	2	1	1	0	1	3	5	1
6008	201	1	5-May-17	12	1	15	2	1	1	0	1	3	2	1
6009	201	1	17-May-17	18	1	15	2	1	1	0	1	3	2	1
6010	201	1	3-Jul-17	7	1	15	2	1	1	0	1	3	1	1
6011	201	1	26-Jan-17	15	1	15	2	2	1	0	1	3	5	1
6012	201	1	25-Jul-17	17	1	15	2	2	1	0	1	3	3	1
6013	201	1	19-Jul-17	19	1	15	2	2	1	0	1	3	3	1
6014	201	1	23-Feb-17	22	1	15	2	2	1	0	1	3	1	1
6015	201	1	1-Jan-16	19	1	15	2	2	1	0	3	3	3	1
6016	101	1	29-Nov-15	21	1	15	2	1	1	0	3	3	1	1
6017	101	1	14-Jul-12	18	1	15	2	1	1	0	1	3	1	2
6018	101	1	20-Nov-14	19	1	15	2	2	1	0	1	3	2	1
6019	101	1	14-Aug-14	13	1	15	2	1	1	0	1	3	3	1
6020	201	1	7-Jan-15	10	1	15	2	1	1	0	1	3	1	1
6021	201	1	20-Dec-13	20	1	15	2	2	1	0	1	3	5	2
6022	201	1	14-Jan-14	1	1	15	2	2	1	0	1	3	5	1
6023	201	1	25-Jun-14	13	1	15	2	2	1	0	1	3	1	1
6024	201	1	8-Dec-16	19	1	15	2	1	1	0	1	3	2	1
6025	101	1	2-Oct-14	2	1	15	2	1	1	0	1	3	5	1
6026	201	1	26-Dec-13	7	1	15	2	1	1	0	1	3	5	1
6027	201	1	27-Jun-13	8	1	15	2	1	1	0	1	3	5	1
6028	201	1	21-Apr-12	14	1	15	2	2	1	0	1	3	6	2
6029	201	1	2-Jan-12	12	1	15	2	2	1	0	1	3	6	2
6030	101	1	7-Sep-12	19	1	15	2	1	1	0	1	3	3	2
6031	201	1	2-Jan-17	14	1	15	2	2	1	0	1	3	5	1
6032	101	1	3-Oct-14	16	1	15	2	1	1	0	1	3	1	2
6033	101	1	7-Feb-12	17	1	15	2	1	1	0	1	3	3	1
6034	101	1	20-Dec-16	8	1	15	2	1	1	0	1	3	3	4
6035	101	1	3-Aug-15	20	1	15	2	2	1	0	1	3	5	2
6036	201	1	11-Sep-15	18	1	15	2	1	1	0	1	3	1	2
6037	201	1	17-Sep-14	20	1	15	2	2	1	0	1	3	1	2
6038	101	1	10-Nov-13	17	1	15	2	1	1	0	1	3	1	1
6039	201	1	20-Mar-16	19	1	15	2	3	1	0	1	3	1	2
6040	101	1	4-Jul-16	10	1	15	2	2	1	0	1	3	1	1
6041	101	1	16-Nov-15	23	1	15	2	1	1	0	1	3	1	2
6042	101	1	9-Jul-13	13	1	15	2	1	1	0	1	3	6	2
6043	201	1	29-Oct-13	21	1	15	2	1	1	0	1	3	5	2
6044	201	1	19-May-13	16	1	15	2	1	1	0	1	3	6	2
6045	201	1	31-May-13	6	1	15	2	2	1	0	1	3	5	2
6046	101	1	19-Sep-13	22	1	15	2	1	1	0	1	3	1	2
6047	201	1	18-May-13	16	1	15	2	1	1	0	1	3	4	2
6048	201	1	17-Mar-13	16	1	15	2	2	1	0	1	3	5	2
6049	101	1	10-Nov-12	19	1	15	2	1	1	0	1	3	5	1
6050	101	1	3-Nov-12	1	1	15	2	1	1	0	1	3	5	1
6051	201	1	26-Nov-16	19	1	15	2	2	1	0	4	3	3	1
6052	101	1	17-Jul-14	0	1	15	2	1	1	0	6	3	2	1
6053	201	1	30-Aug-15	9	1	15	2	2	1	0	6	3	2	1
6054	201	1	23-Oct-16	9	1	15	2	1	1	0	6	3	2	1
6055	201	1	23-Jan-12	21	1	15	2	1	1	0	7	3	2	1
6056	101	1	11-Feb-14	11	1	15	2	3	1	0	1	3	5	1
6057	101	1	5-Jun-12	13	1	15	2	1	1	0	1	3	5	2
6058	101	1	1-Jan-11	18	1	15	2	1	1	0	1	3	5	1
6059	101	1	1-Jan-11	20	1	15	2	1	1	0	1	3	5	1
6060	101	1	14-Aug-11	18	1	15	2	1	1	0	1	3	5	1
6061	201	1	17-May-11	13	1	15	2	2	1	0	1	3	6	1
6062	201	1	3-Mar-11	19	1	15	2	1	1	0	1	3	5	2
6063	101	1	1-Nov-11	22	1	15	2	1	1	0	1	3	1	2
6064	201	1	8-Jul-11	14	1	15	2	1	1	0	1	3	7	2
6065	201	1	6-Jun-11	3	1	15	2	1	1	0	1	3	6	1
6066	201	1	22-Feb-11	12	1	15	2	1	1	0	1	3	1	1
6067	101	1	19-Jul-11	23	1	15	2	2	1	0	1	3	3	1
6068	101	1	30-May-11	11	1	15	2	1	1	0	1	3	3	1
6069	201	1	4-May-11	22	1	15	2	1	1	0	1	3	3	1

6070	101	1	19-Apr-11	8	1	15	2	4	1	0	1	3	2	1
6071	201	1	11-Oct-11	10	1	15	2	3	1	0	1	3	6	2
6072	201	1	23-Jul-11	22	1	15	2	3	1	0	1	3	1	2
6073	201	1	15-Jan-11	10	1	15	2	2	1	0	1	3	3	2
6074	201	1	6-Aug-11	9	1	15	2	3	1	0	1	3	3	2
6075	101	1	16-Feb-11	11	1	15	2	2	1	0	1	3	3	4
6076	101	1	11-Apr-10	21	1	15	2	1	1	0	1	3	5	1
6077	101	1	24-Jul-10	20	1	15	2	2	1	0	1	3	1	2
6078	201	1	19-Nov-10	7	1	15	2	1	1	0	1	3	3	1
6079	201	1	8-Sep-10	21	1	15	2	2	1	0	1	3	5	1
6080	201	1	17-May-10	17	1	15	2	2	1	0	1	3	5	1
6081	201	1	25-Jun-10	13	1	15	2	2	1	0	1	3	1	1
6082	201	1	9-Oct-10	22	1	15	2	1	1	0	1	3	1	1
6083	201	1	8-Jun-10	21	1	15	2	1	1	0	1	3	1	1
6084	201	1	18-Nov-10	13	1	15	2	1	1	0	1	3	3	1
6085	201	1	21-Feb-10	16	1	15	2	1	1	0	1	3	2	1
6086	201	1	24-Apr-10	23	1	15	2	2	1	0	1	3	3	1
6087	201	1	22-Sep-09	16	1	15	2	3	1	0	1	3	5	1
6088	101	1	24-Nov-09	19	1	15	2	1	1	0	1	3	1	1
6089	201	1	1-Dec-09	8	1	15	2	1	1	0	1	3	1	1
6090	201	1	24-Feb-09	7	1	15	2	1	1	0	1	3	4	2
6091	201	1	10-Aug-09	23	1	15	2	2	1	0	1	3	1	1
6092	201	1	17-Feb-09	17	1	15	2	3	1	0	1	3	1	2
6093	101	1	17-Sep-09	10	1	15	2	1	1	0	1	3	5	1
6094	201	1	4-Jun-09	22	1	15	2	3	1	0	1	3	1	2
6095	201	1	14-Jan-08	16	1	15	2	2	1	0	1	3	1	1
6096	201	1	31-Mar-08	17	1	15	2	1	1	0	1	3	1	2
6097	201	1	28-Jun-08	10	1	15	2	1	1	0	1	3	2	1
6098	201	1	2-Jun-08	1	1	15	2	1	1	0	1	3	2	1
6099	101	1	21-Aug-08	23	1	15	2	3	1	0	1	3	3	2
6100	201	1	10-Feb-08	18	1	15	2	3	1	0	1	3	2	1
6101	201	1	10-Sep-08	22	1	15	2	1	1	0	1	3	2	1
6102	101	1	3-Dec-08	13	1	15	2	1	1	0	1	3	3	1
6103	101	1	3-Dec-08	13	1	15	2	1	1	0	1	3	4	1
6104	201	1	5-Dec-08	20	1	15	2	3	1	0	1	3	7	2
6105	201	1	28-Mar-07	19	1	15	2	1	1	0	1	3	6	1
6106	101	1	18-May-07	17	1	15	2	1	1	0	1	3	5	1
6107	201	1	3-Jan-07	20	1	15	2	1	1	0	1	3	1	1
6108	101	1	2-Aug-07	15	1	15	2	1	1	0	1	3	2	2
6109	201	1	28-Apr-07	9	1	15	2	1	1	0	1	3	6	1
6110	201	1	30-Jan-07	18	1	15	2	2	1	0	1	3	2	2
6111	101	1	23-May-07	19	1	15	2	3	1	0	1	3	4	1
6112	101	1	1-Apr-06	10	1	15	2	1	1	0	1	3	1	1
6113	201	1	1-Jan-06	10	1	15	2	3	1	0	1	3	4	1
6114	201	1	19-Dec-06	9	1	15	2	3	1	0	1	3	5	1
6115	201	1	29-Jun-06	18	1	15	2	2	1	0	1	3	6	1
6116	201	1	22-Apr-06	9	1	15	2	1	1	0	1	3	6	1
6117	101	1	8-Sep-06	14	1	15	2	1	1	0	1	3	4	2
6118	101	1	24-Nov-06	8	1	15	2	3	1	0	1	3	1	1
6119	201	1	4-Dec-06	8	1	15	2	3	1	0	1	3	4	1
6120	201	1	3-Jul-06	0	1	15	2	2	1	0	1	3	4	1
6121	101	1	24-Feb-14	7	1	15	2	1	1	0	1	3	5	1
6122	101	1	27-Dec-13	21	1	15	2	1	1	0	1	3	5	1
6123	201	1	15-Aug-11	14	1	15	2	2	1	0	1	3	5	1
6124	101	1	24-Dec-11	11	1	15	2	1	1	0	1	3	2	1
6125	201	1	13-Apr-11	18	1	15	2	1	1	0	1	3	2	1
6126	101	1	5-Oct-11	11	1	15	2	1	1	0	1	3	2	2
6127	201	1	30-Apr-10	8	1	15	2	2	1	0	1	3	2	1
6128	201	1	5-Jul-10	1	1	15	2	2	1	0	1	3	1	1
6129	101	1	20-Oct-10	17	1	15	2	1	1	0	1	3	2	1
6130	201	1	6-Jan-10	16	1	15	2	1	1	0	1	3	4	1
6131	101	1	26-Aug-09	9	1	15	2	1	1	0	1	3	5	1
6132	201	1	7-Feb-09	20	1	15	2	1	1	0	1	3	1	1
6133	201	1	1-Jun-09	17	1	15	2	1	1	0	1	3	2	1
6134	101	1	26-Aug-08	21	1	15	2	1	1	0	1	3	1	2
6135	101	1	4-Jul-08	16	1	15	2	1	1	0	1	3	5	1
6136	101	1	1-Mar-08	11	1	15	2	1	1	0	1	3	6	1
6137	101	1	4-Jan-08	22	1	15	2	1	1	0	1	3	3	1
6138	201	1	24-Jul-08	7	1	15	2	2	1	0	1	3	2	1
6139	201	1	1-Nov-07	16	1	15	2	2	1	0	1	3	4	1
6140	201	1	13-Sep-07	19	1	15	2	3	1	0	1	3	5	1
6141	101	1	22-Apr-07	11	1	15	2	1	1	0	1	3	1	2
6142	201	1	19-Aug-07	20	1	15	2	2	1	0	1	3	2	1
6143	201	1	26-Jun-06	17	1	15	2	1	1	0	1	3	3	1
6144	101	1	6-Oct-06	14	1	15	2	1	1	0	1	3	3	1
6145	201	1	24-Dec-06	17	1	15	2	1	1	0	1	3	3	1
6146	201	1	11-Oct-07	22	1	15	2	2	1	0	4	3	1	1
6147	101	1	4-Nov-16	1	1	15	2	1	1	0	11	3	4	1
6148	101	1	4-Jul-15	12	1	15	2	1	1	0	11	3	3	1
6149	101	1	5-Aug-15	17	1	15	2	1	1	0	11	3	3	1
6150	101	1	13-Dec-15	18	1	15	2	1	1	0	11	3	3	1
6151	201	1	26-Apr-15	8	1	15	2	2	1	0	11	3	3	1
6152	201	1	23-Dec-14	12	1	15	2	2	1	0	11	3	2	1
6153	101	1	17-May-14	18	1	15	2	1	1	0	11	3	1	1
6154	101	1	23-Aug-14	23	1	15	2	1	1	0	11	3	3	1
6155	101	1	28-Jan-13	20	1	15	2	3	1	0	11	3	4	1
6156	201	1	27-Dec-13	7	1	15	2	3	1	0	11	3	2	1
6157	201	1	3-Dec-12	18	1	15	2	3	1	0	11	3	2	1
6158	101	1	28-Apr-12	17	1	15	2	1	1	0	11	3	2	1
6159	101	1	6-Sep-12	19	1	15	2	1	1	0	11	3	2	1
6160	201	1	18-Aug-12	10	1	15	2	2	1	0	11	3	3	1
6161	101	1	14-Mar-10	20	1	15	2	1	1	0	11	3	2	1
6162	201	1	25-Jun-09	22	1	15	2	2	1	0	11	3	3	1
6163	201	1	18-Mar-06	19	1	15	2	1	1	0	11	3	1	1
6164	101	1	14-Apr-17	17	1	15	2	1	1	0	11	3	3	1
6165	101	1	8-Aug-16	22	1	15	2	1	1	0	3	3	4	1
6166	101	1	7-Dec-16	17	1	15	2	1	1	0	3	3	2	1
6167	201	1	5-Dec-16	6	1	15	2	1	1	0	3	3	3	1
6168	101	1	5-Apr-16	17	1	15	2	1	1	0	3	3	3	1
6169	101	1	18-Nov-16	3	1	15	2	1	1	0	3	3	1	1
6170	101	1	9-Dec-16	1	1	15	2	1	1	0	3	3	2	1
6171	101	1	26-Nov-16	2	1	15	2	1	1	0	3	3	2	1
6172	201	1	30-Mar-16	19	1	15	2	1	1	0	3	3	2	1
6173	101	1	25-Nov-16	1	1	15	2	1	1	0	3	3	4	2
6174	201	1	22-Jul-16	15	1	15	2	1	1	0	3	3	3	1
6175	101	1	18-Apr-15	17	1	15	2	1	1	0	3	3	3	1
6176	101	1	7-Apr-15	15	1	15	2	1	1	0	3	3	3	1
6177	201	1	16-Sep-15	17	1	15	2	1	1	0	3	3	5	1
6178	101	1	25-May-15	19	1	15	2	1	1	0	3	3	3	1
6179	101	1	30-May-15	16	1	15	2	1	1	0	3	3	3	1
6180	201	1	28-Sep-15	18	1	15	2	1	1	0	3	3	3	1
6181	201	1	23-Jun-15	17	1	15	2	1	1	0	3	3	3	1
6182	201	1	14-Apr-14	11	1	15	2	2	1	0	3	3	2	1
6183	201	1	27-Nov-14	16	1	15	2	2	1	0	3	3	4	2
6184	101	1	8-Mar-14	18	1	15	2	1	1	0	3	3	2	1
6185	201	1	23-Oct-14	19	1	15	2	3	1	0	3	3	4	1
6186	201	1	20-Sep-13	21	1	15	2	1	1	0	3	3	5	2
6187	201	1	22-Jun-13	11	1	15	2	1	1	0	3	3	6	1
6188	201	1	6-Dec-13	2	1	15	2	3	1	0	3	3	1	1
6189	101	1	17-Dec-13	7	1	15	2	1	1	0	3	3	2	1
6190	201	1	1-Sep-13	21	1	15	2	1	1	0	3	3	3	1
6191	101	1	13-May-12	8	1	15	2	1	1	0	3	3	2	1
6192	201	1	26-May-11	13	1	15	2	1	1	0	3	3	4	1
6193	201	1	18-Oct-11	6	1	15	2	1	1	0	3	3	2	1

6194	101	1	25-Oct-11	20	1	15	2	1	1	0	3	3	1	1
6195	101	1	22-Sep-11	20	1	15	2	1	1	0	3	3	5	1
6196	101	1	26-Jul-11	19	1	15	2	1	1	0	3	3	3	1
6197	101	1	2-Oct-11	22	1	15	2	1	1	0	3	3	3	1
6198	101	1	3-Jan-10	14	1	15	2	1	1	0	3	3	1	2
6199	201	1	12-Oct-10	13	1	15	2	2	1	0	3	3	5	1
6200	101	1	13-Feb-10	20	1	15	2	1	1	0	3	3	2	1
6201	201	1	24-Feb-09	9	1	15	2	1	1	0	3	3	4	1
6202	101	1	12-Aug-09	16	1	15	2	1	1	0	3	3	2	1
6203	201	1	29-Jan-09	8	1	15	2	1	1	0	3	3	5	1
6204	101	1	17-Nov-09	18	1	15	2	1	1	0	3	3	2	1
6205	101	1	1-Apr-09	22	1	15	2	1	1	0	3	3	5	1
6206	101	1	19-Sep-08	21	1	15	2	1	1	0	3	3	2	4
6207	101	1	22-Jun-08	20	1	15	2	1	1	0	3	3	4	1
6208	101	1	13-Jun-08	12	1	15	2	1	1	0	3	3	1	1
6209	201	1	6-Jun-08	18	1	15	2	3	1	0	3	3	2	1
6210	101	1	9-Sep-08	18	1	15	2	1	1	0	3	3	2	1
6211	101	1	25-Oct-08	7	1	15	2	1	1	0	3	3	2	1
6212	101	1	19-Mar-08	18	1	15	2	1	1	0	3	3	2	1
6213	101	1	15-Aug-08	12	1	15	2	1	1	0	3	3	3	1
6214	101	1	25-Apr-07	14	1	15	2	1	1	0	3	3	5	2
6215	101	1	22-Sep-07	19	1	15	2	1	1	0	3	3	1	4
6216	101	1	2-Feb-07	21	1	15	2	1	1	0	3	3	1	2
6217	101	1	15-Jan-07	17	1	15	2	1	1	0	3	3	1	1
6218	101	1	12-Jun-07	21	1	15	2	1	1	0	3	3	1	1
6219	201	1	7-Feb-07	18	1	15	2	1	1	0	3	3	3	1
6220	101	1	28-Sep-07	18	1	15	2	1	1	0	3	3	2	1
6221	101	1	14-Oct-06	19	1	15	2	1	1	0	3	3	1	1
6222	101	1	11-Jan-17	10	1	15	2	1	1	0	3	3	3	1
6223	101	1	18-Mar-17	12	1	15	2	1	1	0	3	3	2	1
6224	201	1	8-May-17	16	1	15	2	2	1	0	3	3	1	1
6225	101	1	23-May-17	12	1	15	2	1	1	0	3	3	3	1
6226	201	1	2-Jan-17	10	1	15	2	1	1	0	3	3	3	1
6227	101	1	7-Jan-17	11	1	15	2	1	1	0	3	3	2	2
6228	101	1	11-Nov-17	9	1	15	2	1	1	0	3	3	3	1
6229	201	1	31-Dec-14	18	1	15	2	1	1	0	6	3	3	2
6230	101	1	11-Jul-12	22	1	15	2	2	1	0	6	3	2	1
6231	201	1	2-Aug-11	11	1	15	2	1	1	0	6	3	2	1
6232	101	1	18-Nov-11	8	1	15	2	1	1	0	6	3	2	1
6233	101	1	14-Dec-10	22	1	15	2	1	1	0	6	3	2	1
6234	101	1	23-Jun-17	10	1	15	2	1	1	0	6	3	3	1
6235	101	1	19-Nov-17	10	1	15	2	1	1	0	13	3	1	1
6236	101	1	18-Nov-17	23	1	15	2	1	1	0	13	3	5	1
6237	101	1	8-Mar-12	14	2	15	2	1	1	0	1	3	1	1
6238	101	1	14-Jun-13	18	2	15	2	1	1	0	1	3	3	1
6239	201	1	4-Jan-13	11	2	15	2	2	1	0	1	3	1	1
6240	101	1	18-Jan-13	12	2	15	2	1	1	0	1	3	3	1
6241	201	1	9-Jun-16	17	2	15	2	1	1	0	1	3	1	1
6242	201	1	20-Mar-15	17	2	15	2	1	1	0	1	3	5	2
6243	201	1	13-May-15	13	2	15	2	1	1	0	1	3	2	1
6244	101	1	27-Feb-15	12	2	15	2	3	1	0	1	3	2	1
6245	201	1	7-Dec-14	20	2	15	2	1	1	0	1	3	5	1
6246	201	1	2-Aug-14	21	2	15	2	2	1	0	1	3	5	1
6247	201	1	30-Jun-14	9	2	15	2	3	1	0	1	3	2	1
6248	201	1	28-May-13	17	2	15	2	2	1	0	1	3	1	1
6249	101	1	27-Apr-17	12	2	15	2	1	1	0	1	3	5	1
6250	101	1	2-Mar-14	8	2	15	2	1	1	0	1	3	1	2
6251	101	1	14-Jun-16	8	2	15	2	1	1	0	1	3	3	1
6252	101	1	12-Dec-14	21	2	15	2	1	1	0	1	3	4	1
6253	101	1	16-Jul-14	19	2	15	2	2	1	0	1	3	5	1
6254	201	1	3-May-12	17	2	15	2	2	1	0	1	3	6	1
6255	201	1	20-Apr-17	16	2	15	2	3	1	0	1	3	5	1
6256	101	1	13-Apr-15	19	2	15	2	3	1	0	1	3	3	1
6257	101	1	27-Jun-13	22	2	15	2	1	1	0	1	3	1	1
6258	201	1	9-Sep-12	21	2	15	2	2	1	0	1	3	6	1
6259	101	1	30-Aug-11	13	2	15	2	1	1	0	7	3	3	1
6260	201	1	28-Jul-11	12	2	15	2	1	1	0	1	3	6	1
6261	201	1	3-Apr-11	6	2	15	2	2	1	0	1	3	2	1
6262	201	1	3-Sep-11	19	2	15	2	1	1	0	1	3	1	2
6263	101	1	7-Sep-11	20	2	15	2	1	1	0	1	3	2	4
6264	201	1	8-Apr-11	10	2	15	2	1	1	0	1	3	1	1
6265	101	1	8-Apr-11	12	2	15	2	1	1	0	1	3	5	1
6266	101	1	17-Sep-10	14	2	15	2	1	1	0	1	3	5	2
6267	101	1	1-Jan-10	12	2	15	2	1	1	0	1	3	4	1
6268	101	1	1-Jan-10	17	2	15	2	1	1	0	1	3	3	1
6269	201	1	27-Aug-10	18	2	15	2	2	1	0	1	3	1	1
6270	201	1	19-May-10	22	2	15	2	1	1	0	1	3	1	1
6271	101	1	3-Jun-10	13	2	15	2	3	1	0	1	3	5	2
6272	201	1	27-Apr-10	8	2	15	2	3	1	0	1	3	4	1
6273	201	1	6-Dec-10	23	2	15	2	2	1	0	1	3	3	1
6274	101	1	27-Nov-09	11	2	15	2	1	1	0	1	3	5	1
6275	201	1	15-Dec-09	16	2	15	2	2	1	0	1	3	6	1
6276	101	1	13-Jun-09	22	2	15	2	1	1	0	1	3	1	1
6277	101	1	30-Oct-09	22	2	15	2	2	1	0	1	3	1	2
6278	201	1	12-Mar-09	23	2	15	2	2	1	0	1	3	5	1
6279	201	1	17-Nov-09	13	2	15	2	2	1	0	1	3	3	1
6280	201	1	31-Jan-09	22	2	15	2	3	1	0	1	3	3	1
6281	101	1	23-Dec-09	18	2	15	2	1	1	0	1	3	3	2
6282	101	1	12-Apr-08	20	2	15	2	1	1	0	1	3	6	1
6283	201	1	20-Jul-07	11	2	15	2	1	1	0	1	3	6	1
6284	101	1	13-Mar-07	7	2	15	2	1	1	0	1	3	5	1
6285	101	1	2-Oct-07	18	2	15	2	1	1	0	1	3	1	1
6286	201	1	20-Apr-07	8	2	15	2	1	1	0	1	3	2	1
6287	101	1	23-Nov-07	21	2	15	2	1	1	0	1	3	5	1
6288	201	1	13-Apr-07	17	2	15	2	2	1	0	1	3	1	1
6289	201	1	7-Jan-07	15	2	15	2	3	1	0	1	3	3	1
6290	201	1	3-Feb-06	17	2	15	2	2	1	0	1	3	3	1
6291	101	1	5-Oct-06	18	2	15	2	1	1	0	1	3	3	1
6292	101	1	4-Jan-06	8	2	15	2	1	1	0	1	3	2	1
6293	201	1	17-Nov-11	23	2	15	2	1	1	0	1	3	3	1
6294	201	1	8-Sep-11	17	2	15	2	3	1	0	1	3	4	1
6295	101	1	23-Sep-11	18	2	15	2	1	1	0	1	3	1	1
6296	201	1	16-Dec-09	16	2	15	2	1	1	0	1	3	2	2
6297	201	1	22-Jun-07	20	2	15	2	1	1	0	1	3	6	1
6298	201	1	25-Apr-07	15	2	15	2	1	1	0	1	3	1	1
6299	201	1	6-Sep-07	7	2	15	2	1	1	0	1	3	1	1
6300	201	1	6-Jan-07	20	2	15	2	1	1	0	1	3	3	2
6301	101	1	3-Nov-06	14	2	15	2	2	1	0	1	3	1	1
6302	101	1	3-Nov-17	19	2	15	2	1	1	0	1	3	6	1
6303	201	1	18-Oct-08	12	2	15	2	1	1	0	4	3	3	1
6304	201	1	3-Jan-17	11	2	15	2	2	1	0	4	3	3	1
6305	101	1	11-Feb-15	11	2	15	2	1	1	0	11	3	3	1
6306	201	1	30-Nov-14	11	2	15	2	1	1	0	11	3	1	1
6307	201	1	24-Nov-10	13	2	15	2	1	1	0	11	3	2	1
6308	201	1	2-Aug-08	16	2	15	2	1	1	0	11	3	3	1
6309	101	1	12-Nov-16	1	2	15	2	1	1	0	3	3	3	1
6310	101	1	17-Sep-15	21	2	15	2	1	1	0	3	3	1	1
6311	201	1	4-Jun-15	11	2	15	2	1	1	0	3	3	3	1
6312	201	1	9-Dec-15	18	2	15	2	1	1	0	3	3	3	1
6313	201	1	4-Jan-15	19	2	15	2	3	1	0	3	3	2	1
6314	101	1	5-Apr-14	16	2	15	2	1	1	0	3	3	3	1
6315	201	1	23-Apr-14	21	2	15	2	1	1	0	3	3	4	1
6316	101	1	14-Jun-13	17	2	15	2	1	1	0	3	3	4	1
6317	201	1	24-Aug-13	20	2	15	2	1	1	0	3	3	2	1

6318	101	1	21-Aug-10	22	2	15	2	1	1	0	3	3	1	1
6319	201	1	23-Feb-09	11	2	15	2	1	1	0	3	3	5	1
6320	201	1	27-Dec-09	13	2	15	2	1	1	0	3	3	4	1
6321	201	1	18-Jun-09	19	2	15	2	1	1	0	3	3	2	1
6322	201	1	1-Dec-08	17	2	15	2	1	1	0	3	3	3	1
6323	201	1	16-Mar-06	21	2	15	2	1	1	0	3	3	3	1
6324	201	1	5-Jul-17	19	2	15	2	1	1	0	3	3	2	1
6325	101	1	26-Aug-13	21	2	15	2	1	1	0	6	3	2	1
6326	202	0	28-Apr-12	17	1	16	2	1	1	0	1	3	4	1
6327	202	0	5-Aug-14	12	1	16	2	2	1	0	1	3	1	2
6328	202	0	17-May-12	6	1	16	2	1	1	0	1	3	1	1
6329	202	0	8-Jun-16	22	1	16	2	1	1	0	1	3	3	1
6330	202	0	12-Jan-16	14	1	16	2	1	1	0	1	3	3	1
6331	202	0	28-Jul-15	20	1	16	2	2	1	0	1	3	4	1
6332	202	0	14-Nov-15	3	1	16	2	1	1	0	1	3	1	2
6333	202	0	2-Aug-14	20	1	16	2	3	1	0	1	3	2	2
6334	202	0	9-Nov-13	0	1	16	2	1	1	0	1	3	2	1
6335	202	0	31-Jul-13	23	1	16	2	1	1	0	1	3	5	1
6336	202	0	5-Nov-17	8	1	16	2	3	1	0	1	3	3	1
6337	202	0	3-Jun-17	0	1	16	2	1	1	0	1	3	3	2
6338	202	0	17-Jun-16	16	1	16	2	1	1	0	1	3	5	1
6339	202	0	22-Mar-13	18	1	16	2	2	1	0	1	3	3	2
6340	202	0	13-Nov-15	13	1	16	2	1	1	0	1	3	5	1
6341	202	0	22-Aug-13	19	1	16	2	1	1	0	1	3	5	1
6342	202	0	15-Mar-17	13	1	16	2	1	1	0	1	3	3	1
6343	202	0	14-Nov-12	4	1	16	2	1	1	0	1	3	3	2
6344	202	0	6-Sep-14	1	1	16	2	2	1	0	1	3	1	2
6345	202	0	6-Sep-14	1	1	16	2	1	1	0	1	3	2	1
6346	202	0	7-Mar-14	23	1	16	2	1	1	0	1	3	1	2
6347	202	0	23-Jan-14	23	1	16	2	3	1	0	1	3	3	2
6348	202	0	30-Nov-13	22	1	16	2	1	1	0	1	3	3	2
6349	202	0	19-Jul-12	14	1	16	2	3	1	0	1	3	6	1
6350	202	0	20-Nov-12	13	1	16	2	1	1	0	1	3	4	1
6351	202	0	28-May-16	14	1	16	2	3	1	0	1	3	1	1
6352	202	0	15-Mar-13	20	1	16	2	3	1	0	1	3	3	1
6353	202	0	2-Jun-13	2	1	16	2	1	1	0	1	3	2	1
6354	202	0	19-Sep-13	16	1	16	2	1	1	0	1	3	5	1
6355	202	0	12-May-16	23	1	16	2	1	1	0	1	3	4	2
6356	202	0	22-Jan-12	12	1	16	2	3	1	0	1	3	2	2
6357	202	0	20-Feb-12	18	1	16	2	1	1	0	1	3	6	1
6358	202	0	27-Oct-15	9	1	16	2	1	1	0	1	3	5	2
6359	202	0	29-May-15	16	1	16	2	1	1	0	1	3	5	2
6360	202	0	3-Feb-16	12	1	16	2	2	1	0	1	3	4	1
6361	202	0	29-Nov-16	4	1	16	2	1	1	0	1	3	5	1
6362	202	0	7-Sep-16	4	1	16	2	3	1	0	1	3	2	1
6363	202	0	8-Apr-16	22	1	16	2	1	1	0	1	3	2	1
6364	202	0	5-Apr-16	20	1	16	2	1	1	0	1	3	1	1
6365	202	0	15-Apr-16	6	1	16	2	1	1	0	1	3	1	1
6366	202	0	7-Jan-16	6	1	16	2	2	1	0	1	3	4	2
6367	202	0	28-Feb-16	22	1	16	2	1	1	0	1	3	3	1
6368	202	0	15-Sep-15	13	1	16	2	3	1	0	1	3	5	1
6369	202	0	6-Nov-15	15	1	16	2	1	1	0	1	3	3	1
6370	202	0	10-Aug-14	16	1	16	2	1	1	0	1	3	5	1
6371	202	0	30-Dec-14	16	1	16	2	3	1	0	1	3	4	1
6372	202	0	29-Jan-14	2	1	16	2	1	1	0	1	3	5	1
6373	202	0	19-Mar-14	22	1	16	2	2	1	0	1	3	1	1
6374	202	0	1-Jan-14	0	1	16	2	2	1	0	1	3	5	1
6375	202	0	14-May-14	22	1	16	2	3	1	0	1	3	2	2
6376	202	0	14-Dec-14	13	1	16	2	1	1	0	1	3	1	1
6377	202	0	18-Apr-14	22	1	16	2	1	1	0	1	3	4	2
6378	202	0	3-Jan-14	7	1	16	2	2	1	0	1	3	4	2
6379	202	0	2-Jan-14	12	1	16	2	1	1	0	1	3	5	1
6380	202	0	4-Feb-14	0	1	16	2	1	1	0	1	3	5	1
6381	202	0	12-Mar-14	11	1	16	2	2	1	0	1	3	5	2
6382	202	0	23-Feb-14	20	1	16	2	1	1	0	1	3	5	1
6383	202	0	6-Sep-14	0	1	16	2	1	1	0	1	3	5	2
6384	202	0	31-Jan-14	3	1	16	2	1	1	0	1	3	3	2
6385	202	0	19-Apr-14	0	1	16	2	1	1	0	1	3	3	2
6386	202	0	11-May-13	21	1	16	2	2	1	0	1	3	3	1
6387	202	0	4-Feb-13	14	1	16	2	1	1	0	1	3	3	1
6388	202	0	9-Jul-13	17	1	16	2	1	1	0	1	3	5	2
6389	202	0	1-May-13	1	1	16	2	1	1	0	1	3	2	1
6390	202	0	9-Jun-13	22	1	16	2	3	1	0	1	3	6	1
6391	202	0	9-Jul-13	3	1	16	2	1	1	0	1	3	1	1
6392	202	0	1-May-13	1	1	16	2	1	1	0	1	3	1	1
6393	202	0	24-Jan-13	19	1	16	2	1	1	0	1	3	3	2
6394	202	0	6-Oct-13	16	1	16	2	1	1	0	1	3	5	2
6395	202	0	10-Apr-13	22	1	16	2	1	1	0	1	3	3	1
6396	202	0	5-Mar-13	18	1	16	2	1	1	0	1	3	3	1
6397	202	0	4-May-13	13	1	16	2	1	1	0	1	3	3	1
6398	202	0	26-Nov-13	15	1	16	2	1	1	0	1	3	3	1
6399	202	0	4-Feb-13	14	1	16	2	1	1	0	1	3	3	1
6400	202	0	27-Jul-13	20	1	16	2	1	1	0	1	3	5	1
6401	202	0	18-Aug-12	2	1	16	2	1	1	0	1	3	6	2
6402	202	0	19-Dec-12	14	1	16	2	1	1	0	1	3	1	2
6403	202	0	13-Jul-12	20	1	16	2	1	1	0	1	3	2	1
6404	202	0	2-Feb-12	1	1	16	2	1	1	0	1	3	5	1
6405	202	0	20-Jan-12	21	1	16	2	1	1	0	1	3	3	2
6406	202	0	21-Oct-12	16	1	16	2	1	1	0	1	3	1	2
6407	202	0	19-Aug-17	15	1	16	2	1	1	0	1	3	3	1
6408	202	0	30-Jan-17	22	1	16	2	1	1	0	1	3	1	1
6409	202	0	23-Sep-17	18	1	16	2	1	1	0	1	3	5	3
6410	202	0	30-Sep-17	20	1	16	2	2	1	0	1	3	6	1
6411	202	0	21-Jan-13	23	1	16	2	2	1	0	4	3	1	2
6412	202	0	23-Dec-15	1	1	16	2	1	1	0	3	3	5	1
6413	202	0	20-May-16	17	1	16	2	1	1	0	6	3	2	1
6414	202	0	7-Dec-17	0	1	16	2	2	1	0	4	3	1	1
6415	202	0	21-Feb-17	0	1	16	2	2	1	0	6	3	3	2
6416	202	0	12-Apr-12	20	1	16	2	1	1	0	1	3	6	1
6417	202	0	1-Jan-12	1	1	16	2	2	1	0	1	3	1	2
6418	202	0	2-May-11	16	1	16	2	1	1	0	1	3	5	2
6419	202	0	6-Apr-11	18	1	16	2	1	1	0	1	3	6	1
6420	202	0	21-Jul-11	15	1	16	2	2	1	0	1	3	5	2
6421	202	0	24-Aug-11	22	1	16	2	1	1	0	1	3	6	2
6422	202	0	20-Aug-11	21	1	16	2	1	1	0	1	3	6	1
6423	202	0	22-Jun-11	1	1	16	2	1	1	0	1	3	2	2
6424	202	0	30-Jun-11	20	1	16	2	1	1	0	1	3	4	2
6425	202	0	30-Oct-11	8	1	16	2	1	1	0	1	3	1	2
6426	202	0	8-Apr-11	22	1	16	2	1	1	0	1	3	1	2
6427	202	0	18-Jul-11	11	1	16	2	1	1	0	1	3	1	1
6428	202	0	12-May-11	17	1	16	2	1	1	0	1	3	1	1
6429	202	0	28-Feb-11	8	1	16	2	1	1	0	1	3	2	1
6430	202	0	30-Mar-11	18	1	16	2	1	1	0	1	3	3	2
6431	202	0	6-Feb-11	17	1	16	2	1	1	0	1	3	2	1
6432	202	0	10-Sep-11	19	1	16	2	3	1	0	1	3	3	1
6433	202	0	12-Jul-10	22	1	16	2	1	1	0	1	3	5	1
6434	202	0	18-Nov-10	22	1	16	2	1	1	0	1	3	6	1
6435	202	0	30-Aug-10	16	1	16	2	2	1	0	1	3	1	1
6436	202	0	11-Jul-10	10	1	16	2	1	1	0	1	3	3	2
6437	202	0	14-Nov-10	3	1	16	2	1	1	0	1	3	1	1
6438	202	0	21-Oct-10	1	1	16	2	1	1	0	1	3	5	1
6439	202	0	18-Dec-10	21	1	16	2	1	1	0	1	3	3	1
6440	202	0	10-Oct-09	0	1	16	2	1	1	0	1	3	5	1
6441	202	0	17-May-09	1	1	16	2	1	1	0	1	3	6	1

6442	202	0	8-Nov-09	21	1	16	2	1	1	0	1	3	6	1
6443	202	0	4-Oct-09	8	1	16	2	3	1	0	1	3	3	1
6444	202	0	28-Dec-09	12	1	16	2	1	1	0	1	3	1	2
6445	202	0	30-Mar-09	16	1	16	2	2	1	0	1	3	3	1
6446	202	0	22-Apr-09	1	1	16	2	1	1	0	1	3	3	1
6447	202	0	21-Apr-09	19	1	16	2	1	1	0	1	3	3	1
6448	202	0	22-Mar-09	19	1	16	2	1	1	0	1	3	3	1
6449	202	0	11-Nov-09	18	1	16	2	1	1	0	1	3	2	1
6450	202	0	1-Oct-09	21	1	16	2	1	1	0	1	3	3	1
6451	202	0	6-Jul-08	17	1	16	2	1	1	0	1	3	1	1
6452	202	0	29-Nov-08	16	1	16	2	3	1	0	1	3	1	2
6453	202	0	22-Sep-08	14	1	16	2	1	1	0	1	3	6	2
6454	202	0	22-Sep-08	14	1	16	2	1	1	0	1	3	6	2
6455	202	0	30-Jun-08	19	1	16	2	1	1	0	1	3	3	1
6456	202	0	15-Jul-08	14	1	16	2	1	1	0	1	3	3	2
6457	202	0	19-Sep-08	0	1	16	2	2	1	0	1	3	6	2
6458	202	0	3-Sep-07	15	1	16	2	1	1	0	1	3	5	1
6459	202	0	26-Mar-07	18	1	16	2	1	1	0	1	3	2	1
6460	202	0	26-Mar-07	18	1	16	2	1	1	0	1	3	2	1
6461	202	0	15-May-07	18	1	16	2	1	1	0	1	3	1	2
6462	202	0	16-Nov-07	17	1	16	2	1	1	0	1	3	3	1
6463	202	0	26-Nov-07	0	1	16	2	2	1	0	1	3	6	2
6464	202	0	12-Mar-07	13	1	16	2	1	1	0	1	3	1	2
6465	202	0	25-Nov-07	18	1	16	2	1	1	0	1	3	3	1
6466	202	0	4-Jun-07	7	1	16	2	1	1	0	1	3	2	1
6467	202	0	1-Jan-07	20	1	16	2	1	1	0	1	3	4	2
6468	202	0	20-Sep-07	7	1	16	2	1	1	0	1	3	1	1
6469	202	0	12-May-07	17	1	16	2	1	1	0	1	3	3	1
6470	202	0	15-Feb-07	2	1	16	2	1	1	0	1	3	3	2
6471	202	0	1-Nov-06	0	1	16	2	1	1	0	1	3	6	1
6472	202	0	2-Jan-06	11	1	16	2	1	1	0	1	3	6	1
6473	202	0	20-Feb-06	2	1	16	2	2	1	0	1	3	3	2
6474	202	0	10-Jan-06	11	1	16	2	1	1	0	1	3	3	2
6475	202	0	10-Mar-06	16	1	16	2	1	1	0	1	3	6	1
6476	202	0	21-Sep-11	19	1	16	2	1	1	0	1	3	4	1
6477	202	0	4-Jan-09	12	1	16	2	3	1	0	1	3	2	2
6478	202	0	15-Nov-08	11	1	16	2	1	1	0	1	3	2	1
6479	202	0	21-Aug-08	12	1	16	2	3	1	0	1	3	2	2
6480	202	0	20-Nov-08	19	1	16	2	1	1	0	1	3	2	1
6481	202	0	12-Oct-06	13	1	16	2	1	1	0	1	3	1	2
6482	202	0	27-May-06	7	1	16	2	1	1	0	1	3	1	2
6483	202	0	23-Jun-16	18	1	16	2	1	1	0	4	3	2	2
6484	202	0	23-Jun-16	17	1	16	2	1	1	0	4	3	5	1
6485	202	0	23-Aug-16	1	1	16	2	1	1	0	4	3	3	1
6486	202	0	12-Jun-15	15	1	16	2	3	1	0	4	3	5	1
6487	202	0	14-Sep-10	20	1	16	2	1	1	0	4	3	1	2
6488	202	0	18-Nov-07	17	1	16	2	3	1	0	4	3	2	1
6489	202	0	10-Apr-17	1	1	16	2	1	1	0	4	3	1	1
6490	202	0	24-Aug-11	23	1	16	2	3	1	0	11	3	2	2
6491	202	0	22-Sep-11	19	1	16	2	1	1	0	11	3	3	2
6492	202	0	12-Jul-10	13	1	16	2	1	1	0	11	3	3	1
6493	202	0	9-Jun-16	9	1	16	2	2	1	0	3	3	3	1
6494	202	0	14-Dec-16	8	1	16	2	1	1	0	3	3	5	1
6495	202	0	31-May-16	2	1	16	2	3	1	0	3	3	1	1
6496	202	0	17-Oct-15	23	1	16	2	1	1	0	3	3	5	1
6497	202	0	12-May-15	23	1	16	2	3	1	0	3	3	1	1
6498	202	0	19-Apr-14	1	1	16	2	1	1	0	3	3	5	1
6499	202	0	30-Nov-14	19	1	16	2	1	1	0	3	3	5	1
6500	202	0	20-Feb-14	23	1	16	2	1	1	0	3	3	3	1
6501	202	0	26-Feb-14	18	1	16	2	1	1	0	3	3	2	1
6502	202	0	19-Oct-14	18	1	16	2	2	1	0	3	3	2	2
6503	202	0	15-Nov-13	21	1	16	2	1	1	0	3	3	1	1
6504	202	0	12-May-13	16	1	16	2	1	1	0	3	3	3	2
6505	202	0	2-Oct-13	20	1	16	2	1	1	0	3	3	2	1
6506	202	0	14-Dec-12	15	1	16	2	1	1	0	3	3	3	1
6507	202	0	18-Nov-10	22	1	16	2	1	1	0	3	3	5	1
6508	202	0	29-Jul-10	11	1	16	2	1	1	0	3	3	2	1
6509	202	0	19-Mar-10	23	1	16	2	1	1	0	3	3	7	2
6510	202	0	14-Mar-10	14	1	16	2	1	1	0	3	3	1	2
6511	202	0	22-Sep-10	20	1	16	2	1	1	0	3	3	3	1
6512	202	0	25-Jul-10	16	1	16	2	1	1	0	3	3	2	1
6513	202	0	29-May-10	16	1	16	2	3	1	0	3	3	2	2
6514	202	0	6-Apr-10	20	1	16	2	1	1	0	3	3	3	2
6515	202	0	17-Jan-10	13	1	16	2	1	1	0	3	3	2	2
6516	202	0	10-Dec-09	13	1	16	2	1	1	0	3	3	3	1
6517	202	0	11-Aug-08	12	1	16	2	3	1	0	3	3	2	1
6518	202	0	3-Sep-08	7	1	16	2	2	1	0	3	3	2	2
6519	202	0	8-Aug-08	19	1	16	2	1	1	0	3	3	2	2
6520	202	0	6-Nov-08	18	1	16	2	1	1	0	3	3	2	2
6521	202	0	1-May-06	10	1	16	2	3	1	0	3	3	3	1
6522	202	0	22-Nov-17	15	1	16	2	2	1	0	3	3	2	2
6523	202	0	2-Feb-17	16	1	16	2	1	1	0	3	3	2	1
6524	202	0	11-Dec-17	21	1	16	2	2	1	0	3	3	5	1
6525	202	0	30-Jan-11	17	1	16	2	1	1	0	6	3	6	1
6526	202	0	27-Oct-09	18	1	16	2	1	1	0	6	3	1	1
6527	202	0	21-Nov-09	2	1	16	2	1	1	0	6	3	2	1
6528	202	0	13-Sep-08	20	1	16	2	1	1	0	6	3	1	1
6529	202	0	3-Sep-08	4	1	16	2	1	1	0	6	3	1	1
6530	202	0	19-Jul-17	2	1	16	2	1	1	0	6	3	2	2
6531	202	0	21-Apr-17	19	1	16	2	1	1	0	6	3	2	1
6532	202	0	30-Jun-08	22	1	16	2	3	1	0	13	3	3	1
6533	202	0	15-Jan-07	21	1	16	2	1	1	0	13	3	1	1
6534	202	0	5-Apr-12	18	2	16	2	1	1	0	1	3	5	1
6535	202	0	17-Dec-16	12	2	16	2	1	1	0	1	3	4	1
6536	202	0	28-Sep-14	16	2	16	2	1	1	0	1	3	3	1
6537	202	0	30-Jan-13	23	2	16	2	1	1	0	1	3	1	1
6538	202	0	7-Jan-16	17	2	16	2	3	1	0	1	3	5	1
6539	202	0	25-May-12	6	2	16	2	1	1	0	1	3	5	2
6540	202	0	4-Jul-16	20	2	16	2	3	1	0	1	3	3	1
6541	202	0	19-Jul-16	21	2	16	2	1	1	0	1	3	5	1
6542	202	0	13-Oct-16	20	2	16	2	3	1	0	1	3	3	1
6543	202	0	4-Feb-14	13	2	16	2	3	1	0	1	3	5	1
6544	202	0	2-Feb-12	15	2	16	2	3	1	0	1	3	2	1
6545	202	0	31-Dec-14	19	2	16	2	3	1	0	1	3	5	2
6546	202	0	15-Aug-14	2	2	16	2	1	1	0	1	3	3	2
6547	202	0	13-Mar-13	19	2	16	2	1	1	0	1	3	3	2
6548	202	0	30-Jul-16	0	2	16	2	3	1	0	1	3	1	2
6549	202	0	5-Oct-16	19	2	16	2	2	1	0	1	3	2	1
6550	202	0	18-Aug-15	23	2	16	2	1	1	0	1	3	2	1
6551	202	0	9-Aug-15	3	2	16	2	3	1	0	1	3	2	1
6552	202	0	8-Jul-15	19	2	16	2	1	1	0	1	3	5	1
6553	202	0	20-Jun-15	9	2	16	2	1	1	0	1	3	1	2
6554	202	0	26-Apr-14	18	2	16	2	2	1	0	1	3	1	1
6555	202	0	19-Oct-14	1	2	16	2	1	1	0	1	3	1	2
6556	202	0	14-Jun-14	11	2	16	2	1	1	0	1	3	1	1
6557	202	0	13-Jun-14	15	2	16	2	1	1	0	1	3	3	1
6558	202	0	25-Feb-14	12	2	16	2	1	1	0	1	3	1	1
6559	202	0	23-Jul-13	22	2	16	2	1	1	0	1	3	1	1
6560	202	0	4-Jun-13	6	2	16	2	2	1	0	1	3	2	1
6561	202	0	25-Jul-13	23	2	16	2	3	1	0	1	3	3	1
6562	202	0	3-Jun-13	7	2	16	2	1	1	0	1	3	3	1
6563	202	0	28-Jul-13	18	2	16	2	1	1	0	1	3	5	1
6564	202	0	19-Oct-13	23	2	16	2	1	1	0	1	3	2	2
6565	202	0	14-Apr-13	4	2	16	2	1	1	0	1	3	3	1

6566	202	0	4-Jun-13	22	2	16	2	3	1	0	1	3	3	1
6567	202	0	16-Dec-12	22	2	16	2	1	1	0	1	3	1	2
6568	202	0	2-Jul-12	18	2	16	2	1	1	0	1	3	5	1
6569	202	0	12-Apr-12	22	2	16	2	1	1	0	1	3	3	1
6570	202	0	23-Jan-17	5	2	16	2	1	1	0	1	3	1	1
6571	202	0	12-Apr-17	7	2	16	2	1	1	0	1	3	1	1
6572	202	0	27-May-17	6	2	16	2	3	1	0	1	3	1	1
6573	202	0	14-Jul-14	9	2	16	2	1	1	0	6	3	3	1
6574	202	0	20-Dec-13	16	2	16	2	1	1	0	6	3	1	1
6575	202	0	28-Sep-09	12	2	16	2	2	1	0	7	3	3	1
6576	202	0	11-Sep-12	12	2	16	2	1	1	0	1	3	5	2
6577	202	0	16-Dec-12	22	2	16	2	1	1	0	1	3	5	2
6578	202	0	22-Jan-11	0	2	16	2	1	1	0	1	3	5	1
6579	202	0	9-Dec-11	11	2	16	2	1	1	0	1	3	1	2
6580	202	0	11-Jun-11	4	2	16	2	1	1	0	1	3	2	1
6581	202	0	24-May-11	21	2	16	2	1	1	0	1	3	5	2
6582	202	0	21-Dec-10	8	2	16	2	1	1	0	1	3	6	1
6583	202	0	18-Jun-10	20	2	16	2	1	1	0	1	3	1	1
6584	202	0	25-Jul-10	0	2	16	2	1	1	0	1	3	2	1
6585	202	0	28-May-10	17	2	16	2	1	1	0	1	3	3	1
6586	202	0	21-Nov-10	20	2	16	2	3	1	0	1	3	1	2
6587	202	0	12-Jul-10	1	2	16	2	1	1	0	1	3	6	1
6588	202	0	2-Jan-09	23	2	16	2	1	1	0	1	3	6	1
6589	202	0	9-Apr-09	23	2	16	2	1	1	0	1	3	2	1
6590	202	0	26-Jul-09	23	2	16	2	1	1	0	1	3	5	2
6591	202	0	6-Nov-09	1	2	16	2	1	1	0	1	3	3	1
6592	202	0	8-Jan-08	7	2	16	2	1	1	0	1	3	3	1
6593	202	0	5-Feb-08	14	2	16	2	1	1	0	1	3	3	1
6594	202	0	3-Oct-08	18	2	16	2	1	1	0	1	3	2	1
6595	202	0	20-Jun-07	14	2	16	2	3	1	0	1	3	2	2
6596	202	0	23-Jan-07	7	2	16	2	1	1	0	1	3	1	1
6597	202	0	2-Dec-07	19	2	16	2	1	1	0	1	3	3	1
6598	202	0	29-Jan-06	17	2	16	2	1	1	0	1	3	6	1
6599	202	0	19-Mar-06	1	2	16	2	1	1	0	1	3	1	1
6600	202	0	28-Apr-06	16	2	16	2	1	1	0	1	3	1	2
6601	202	0	28-Aug-06	10	2	16	2	3	1	0	1	3	2	1
6602	202	0	18-Mar-06	19	2	16	2	2	1	0	1	3	3	1
6603	202	0	6-May-10	7	2	16	2	1	1	0	1	3	6	1
6604	202	0	17-Jan-07	7	2	16	2	1	1	0	1	3	4	2
6605	202	0	27-Aug-07	16	2	16	2	1	1	0	1	3	3	2
6606	202	0	25-Dec-16	1	2	16	2	3	1	0	4	3	2	1
6607	202	0	11-Feb-10	13	2	16	2	1	1	0	4	3	3	1
6608	202	0	12-Sep-09	12	2	16	2	1	1	0	4	3	3	1
6609	202	0	24-Jul-14	4	2	16	2	1	1	0	3	3	5	1
6610	202	0	7-Jun-13	21	2	16	2	1	1	0	3	3	2	2
6611	202	0	4-May-10	9	2	16	2	1	1	0	3	3	3	1
6612	202	0	22-Jun-08	16	2	16	2	1	1	0	3	3	1	1
6613	202	0	29-May-08	7	2	16	2	1	1	0	3	3	1	1
6614	202	0	27-Mar-08	12	2	16	2	1	1	0	3	3	4	1
6615	202	0	31-Jul-17	10	2	16	2	1	1	0	3	3	2	1
6616	202	0	18-May-08	21	2	16	2	1	1	0	6	3	1	1
6617	101	1	25-Sep-13	17	1	16	2	1	1	0	1	3	2	2
6618	101	1	31-Mar-16	1	1	16	2	1	1	0	1	3	5	2
6619	201	1	14-Nov-13	18	1	16	2	1	1	0	1	3	4	1
6620	201	1	17-Mar-13	16	1	16	2	2	1	0	1	3	3	2
6621	201	1	13-Jul-12	18	1	16	2	3	1	0	1	3	6	1
6622	201	1	1-Aug-12	8	1	16	2	3	1	0	1	3	5	1
6623	101	1	4-Nov-14	19	1	16	2	1	1	0	1	3	2	2
6624	201	1	13-Jan-14	21	1	16	2	2	1	0	1	3	1	2
6625	201	1	25-Mar-12	11	1	16	2	2	1	0	1	3	4	2
6626	101	1	11-Aug-14	17	1	16	2	1	1	0	1	3	3	1
6627	101	1	14-Mar-16	21	1	16	2	1	1	0	1	3	2	1
6628	201	1	23-Jul-16	13	1	16	2	1	1	0	1	3	1	1
6629	201	1	6-Oct-16	18	1	16	2	3	1	0	1	3	5	1
6630	201	1	27-May-16	6	1	16	2	1	1	0	1	3	1	1
6631	201	1	3-Sep-16	16	1	16	2	1	1	0	1	3	3	1
6632	201	1	8-Dec-16	10	1	16	2	1	1	0	1	3	2	1
6633	201	1	24-Nov-16	21	1	16	2	2	1	0	1	3	2	1
6634	201	1	13-Oct-16	11	1	16	2	1	1	0	1	3	4	1
6635	201	1	14-Nov-16	18	1	16	2	2	1	0	1	3	2	1
6636	101	1	26-Jun-15	20	1	16	2	1	1	0	1	3	4	1
6637	101	1	14-Apr-15	6	1	16	2	1	1	0	1	3	2	1
6638	201	1	26-Jun-15	12	1	16	2	2	1	0	1	3	1	1
6639	201	1	3-Feb-15	6	1	16	2	2	1	0	1	3	5	1
6640	201	1	6-Feb-15	9	1	16	2	2	1	0	1	3	1	1
6641	201	1	13-Jul-15	18	1	16	2	2	1	0	1	3	3	1
6642	201	1	20-May-15	18	1	16	2	1	1	0	1	3	3	1
6643	201	1	1-Oct-15	17	1	16	2	2	1	0	1	3	2	1
6644	201	1	20-Feb-14	16	1	16	2	1	1	0	1	3	5	1
6645	201	1	13-Nov-14	15	1	16	2	1	1	0	1	3	5	1
6646	201	1	8-Mar-14	10	1	16	2	3	1	0	1	3	1	1
6647	201	1	3-Jun-14	17	1	16	2	2	1	0	1	3	3	1
6648	101	1	27-Oct-14	17	1	16	2	1	1	0	1	3	2	1
6649	101	1	11-Jun-14	20	1	16	2	1	1	0	1	3	2	2
6650	201	1	29-May-13	12	1	16	2	2	1	0	1	3	6	1
6651	201	1	6-Nov-13	8	1	16	2	1	1	0	1	3	5	1
6652	101	1	25-Apr-13	21	1	16	2	1	1	0	1	3	2	1
6653	201	1	20-Nov-13	6	1	16	2	1	1	0	1	3	1	1
6654	201	1	20-Jul-13	18	1	16	2	2	1	0	1	3	3	1
6655	101	1	20-Feb-13	20	1	16	2	1	1	0	1	3	3	1
6656	201	1	14-Jun-13	17	1	16	2	2	1	0	1	3	3	1
6657	201	1	12-Apr-12	19	1	16	2	2	1	0	1	3	4	1
6658	101	1	5-May-12	18	1	16	2	1	1	0	1	3	2	1
6659	201	1	11-Aug-17	15	1	16	2	1	1	0	1	3	5	1
6660	101	1	24-Nov-17	23	1	16	2	1	1	0	1	3	3	1
6661	201	1	1-Jun-17	20	1	16	2	1	1	0	1	3	3	1
6662	201	1	6-Mar-17	12	1	16	2	1	1	0	1	3	2	1
6663	101	1	3-Nov-17	22	1	16	2	1	1	0	1	3	6	1
6664	201	1	3-Nov-17	16	1	16	2	2	1	0	1	3	1	1
6665	201	1	8-Dec-17	21	1	16	2	3	1	0	1	3	3	1
6666	201	1	19-Mar-16	20	1	16	2	2	1	0	3	3	5	1
6667	201	1	7-Aug-16	10	1	16	2	1	1	0	3	3	5	1
6668	201	1	7-Aug-16	19	1	16	2	2	1	0	3	3	5	1
6669	101	1	12-Apr-15	11	1	16	2	1	1	0	3	3	1	1
6670	201	1	30-Oct-13	13	1	16	2	3	1	0	1	3	3	1
6671	201	1	18-Jun-13	8	1	16	2	2	1	0	1	3	3	1
6672	201	1	1-Mar-16	13	1	16	2	1	1	0	1	3	2	1
6673	201	1	18-May-17	23	1	16	2	2	1	0	1	3	5	1
6674	201	1	16-Aug-15	1	1	16	2	3	1	0	1	3	2	2
6675	101	1	22-Nov-14	1	1	16	2	1	1	0	1	3	5	2
6676	201	1	24-Sep-16	10	1	16	2	1	1	0	1	3	1	1
6677	201	1	3-Apr-15	20	1	16	2	1	1	0	1	3	1	1
6678	201	1	16-May-14	17	1	16	2	1	1	0	1	3	4	1
6679	101	1	14-Oct-13	22	1	16	2	1	1	0	1	3	5	2
6680	201	1	27-Oct-13	16	1	16	2	1	1	0	1	3	5	2
6681	201	1	5-Jun-15	9	1	16	2	1	1	0	1	3	3	2
6682	101	1	6-Sep-14	20	1	16	2	1	1	0	1	3	5	2
6683	201	1	6-Feb-14	15	1	16	2	2	1	0	1	3	5	1
6684	101	1	10-Jun-14	20	1	16	2	2	1	0	1	3	2	2
6685	101	1	6-Aug-12	8	1	16	2	2	1	0	1	3	3	1
6686	201	1	14-Sep-12	14	1	16	2	2	1	0	1	3	4	1
6687	201	1	1-Aug-16	0	1	16	2	1	1	0	1	3	5	2
6688	101	1	7-Jun-16	14	1	16	2	1	1	0	1	3	1	2
6689	101	1	6-Apr-16	23	1	16	2	1	1	0	1	3	5	1

6690	101	1	7-Apr-16	17	1	16	2	1	1	0	1	3	4	2
6691	201	1	23-Dec-12	23	1	16	2	1	1	0	1	3	1	2
6692	201	1	17-Jul-14	1	1	16	2	1	1	0	1	3	1	2
6693	101	1	10-Apr-16	1	1	16	2	1	1	0	1	3	2	4
6694	101	1	10-Jun-16	21	1	16	2	1	1	0	1	3	1	1
6695	101	1	5-Nov-15	21	1	16	2	1	1	0	1	3	1	2
6696	201	1	18-May-13	16	1	16	2	2	1	0	1	3	1	1
6697	201	1	3-Nov-13	23	1	16	2	1	1	0	1	3	5	2
6698	101	1	1-Mar-13	22	1	16	2	2	1	0	1	3	1	2
6699	201	1	18-May-13	16	1	16	2	1	1	0	1	3	1	1
6700	101	1	28-Feb-13	15	1	16	2	1	1	0	1	3	3	2
6701	201	1	23-Apr-12	20	1	16	2	1	1	0	1	3	6	1
6702	101	1	1-Dec-12	23	1	16	2	1	1	0	1	3	4	2
6703	101	1	1-Aug-17	20	1	16	2	2	1	0	1	3	1	2
6704	101	1	8-Feb-16	22	1	16	2	1	1	0	7	3	2	1
6705	201	1	20-Sep-16	20	1	16	2	1	1	0	6	3	3	1
6706	201	1	23-Sep-17	11	1	16	2	2	1	0	4	3	1	1
6707	201	1	5-Mar-15	1	1	16	2	2	1	0	3	3	5	1
6708	101	1	28-Oct-14	14	1	16	2	2	1	0	1	3	3	1
6709	101	1	14-Oct-13	22	1	16	2	1	1	0	1	3	3	1
6710	101	1	13-Jul-11	17	1	16	2	1	1	0	1	3	5	1
6711	101	1	11-Sep-11	2	1	16	2	1	1	0	1	3	5	1
6712	101	1	10-Oct-11	1	1	16	2	1	1	0	1	3	1	1
6713	101	1	9-Jun-11	19	1	16	2	1	1	0	1	3	3	1
6714	201	1	4-Sep-11	19	1	16	2	2	1	0	1	3	6	1
6715	201	1	30-Apr-11	18	1	16	2	1	1	0	1	3	6	1
6716	201	1	20-Jan-11	17	1	16	2	1	1	0	1	3	6	2
6717	201	1	1-Oct-11	13	1	16	2	1	1	0	1	3	1	1
6718	201	1	12-Aug-11	9	1	16	2	2	1	0	1	3	4	2
6719	201	1	9-Nov-11	9	1	16	2	1	1	0	1	3	3	1
6720	201	1	17-Mar-11	10	1	16	2	2	1	0	1	3	4	2
6721	201	1	3-Mar-11	16	1	16	2	2	1	0	1	3	3	2
6722	201	1	27-Aug-11	14	1	16	2	1	1	0	1	3	2	1
6723	201	1	19-Jun-11	12	1	16	2	1	1	0	1	3	1	1
6724	201	1	20-May-11	23	1	16	2	2	1	0	1	3	6	1
6725	101	1	6-May-11	21	1	16	2	1	1	0	1	3	5	1
6726	201	1	23-Apr-11	9	1	16	2	1	1	0	1	3	1	1
6727	201	1	10-Aug-11	11	1	16	2	1	1	0	1	3	1	1
6728	101	1	24-Apr-11	19	1	16	2	1	1	0	1	3	3	1
6729	201	1	25-Jul-11	7	1	16	2	3	1	0	1	3	3	1
6730	101	1	22-Aug-10	14	1	16	2	1	1	0	1	3	1	1
6731	101	1	27-Apr-10	7	1	16	2	1	1	0	1	3	5	1
6732	201	1	7-Oct-10	19	1	16	2	1	1	0	1	3	6	1
6733	101	1	24-Apr-10	23	1	16	2	2	1	0	1	3	1	1
6734	101	1	23-Jun-10	13	1	16	2	2	1	0	1	3	1	1
6735	101	1	30-Jun-10	10	1	16	2	1	1	0	1	3	5	1
6736	101	1	3-May-10	19	1	16	2	1	1	0	1	3	5	2
6737	201	1	20-Jan-10	16	1	16	2	2	1	0	1	3	1	4
6738	201	1	12-Aug-10	22	1	16	2	1	1	0	1	3	3	2
6739	101	1	23-Aug-09	23	1	16	2	1	1	0	1	3	4	1
6740	201	1	27-Jun-09	23	1	16	2	2	1	0	1	3	5	1
6741	101	1	21-Feb-09	15	1	16	2	1	1	0	1	3	1	1
6742	201	1	30-Aug-08	13	1	16	2	2	1	0	1	3	5	2
6743	101	1	13-Dec-08	17	1	16	2	1	1	0	1	3	2	1
6744	101	1	12-Jul-08	8	1	16	2	1	1	0	1	3	1	2
6745	201	1	2-Oct-08	15	1	16	2	3	1	0	1	3	5	1
6746	201	1	21-Apr-08	17	1	16	2	1	1	0	1	3	5	1
6747	201	1	14-Apr-07	19	1	16	2	2	1	0	1	3	1	2
6748	201	1	29-May-07	20	1	16	2	1	1	0	1	3	3	1
6749	201	1	15-Jun-07	21	1	16	2	1	1	0	1	3	1	1
6750	201	1	19-Jan-07	19	1	16	2	4	1	0	1	3	6	1
6751	201	1	23-Dec-07	19	1	16	2	2	1	0	1	3	5	2
6752	201	1	6-Jul-07	15	1	16	2	1	1	0	1	3	1	2
6753	101	1	12-Jul-07	3	1	16	2	1	1	0	1	3	3	2
6754	201	1	4-Apr-07	10	1	16	2	1	1	0	1	3	5	1
6755	101	1	22-Jun-07	23	1	16	2	1	1	0	1	3	2	1
6756	101	1	19-Dec-07	18	1	16	2	1	1	0	1	3	3	1
6757	101	1	21-Jul-07	23	1	16	2	1	1	0	1	3	2	1
6758	201	1	7-Jan-07	22	1	16	2	3	1	0	1	3	1	1
6759	201	1	8-Dec-07	16	1	16	2	3	1	0	1	3	4	1
6760	101	1	12-Jun-07	7	1	16	2	1	1	0	1	3	3	2
6761	201	1	21-Jan-07	4	1	16	2	1	1	0	1	3	2	1
6762	201	1	3-Nov-07	12	1	16	2	1	1	0	1	3	3	1
6763	201	1	22-Sep-06	11	1	16	2	1	1	0	1	3	1	2
6764	201	1	8-Oct-06	19	1	16	2	1	1	0	1	3	4	1
6765	101	1	20-Jul-06	1	1	16	2	1	1	0	1	3	1	1
6766	101	1	15-Apr-06	17	1	16	2	1	1	0	1	3	4	2
6767	201	1	1-May-06	13	1	16	2	1	1	0	1	3	3	1
6768	201	1	17-Aug-06	22	1	16	2	1	1	0	1	3	4	1
6769	201	1	1-Aug-06	22	1	16	2	1	1	0	1	3	3	2
6770	201	1	27-Sep-06	15	1	16	2	2	1	0	1	3	4	1
6771	201	1	12-Jun-06	17	1	16	2	2	1	0	1	3	3	2
6772	101	1	21-Aug-06	14	1	16	2	1	1	0	1	3	3	1
6773	101	1	13-Nov-06	19	1	16	2	1	1	0	1	3	3	1
6774	101	1	4-Nov-06	19	1	16	2	1	1	0	1	3	3	1
6775	201	1	7-Apr-06	13	1	16	2	1	1	0	5	3	3	1
6776	101	1	17-Oct-13	18	1	16	2	1	1	0	1	3	5	1
6777	201	1	23-Jul-11	17	1	16	2	2	1	0	1	3	1	1
6778	101	1	25-Sep-11	18	1	16	2	1	1	0	1	3	4	1
6779	201	1	24-Feb-11	20	1	16	2	2	1	0	1	3	3	1
6780	201	1	22-Apr-11	14	1	16	2	2	1	0	1	3	6	1
6781	201	1	17-Aug-11	20	1	16	2	2	1	0	1	3	1	1
6782	201	1	21-Apr-11	20	1	16	2	1	1	0	1	3	3	1
6783	101	1	19-Oct-10	12	1	16	2	1	1	0	1	3	2	1
6784	201	1	12-Nov-10	11	1	16	2	1	1	0	1	3	1	1
6785	201	1	18-Jun-10	14	1	16	2	3	1	0	1	3	3	1
6786	201	1	16-Oct-09	23	1	16	2	1	1	0	1	3	1	2
6787	101	1	14-Feb-09	23	1	16	2	1	1	0	1	3	3	1
6788	201	1	23-Mar-09	7	1	16	2	1	1	0	1	3	3	1
6789	101	1	10-Apr-09	22	1	16	2	1	1	0	1	3	1	2
6790	201	1	15-Oct-09	19	1	16	2	1	1	0	1	3	2	1
6791	201	1	10-Jan-09	18	1	16	2	3	1	0	1	3	2	2
6792	101	1	11-Feb-09	16	1	16	2	1	1	0	1	3	3	4
6793	101	1	1-Jun-08	9	1	16	2	1	1	0	1	3	5	1
6794	201	1	8-May-08	16	1	16	2	3	1	0	1	3	5	1
6795	101	1	5-May-08	6	1	16	2	3	1	0	1	3	1	1
6796	101	1	14-Feb-08	19	1	16	2	2	1	0	1	3	2	1
6797	201	1	22-Jul-08	7	1	16	2	1	1	0	1	3	3	1
6798	201	1	30-Aug-08	16	1	16	2	2	1	0	1	3	4	1
6799	201	1	29-Dec-07	18	1	16	2	1	1	0	1	3	7	1
6800	101	1	30-Mar-07	7	1	16	2	1	1	0	1	3	1	1
6801	201	1	9-Sep-07	10	1	16	2	1	1	0	1	3	1	1
6802	201	1	1-Jan-07	21	1	16	2	1	1	0	1	3	1	1
6803	201	1	28-May-07	14	1	16	2	1	1	0	1	3	4	1
6804	201	1	10-Feb-07	21	1	16	2	1	1	0	1	3	1	1
6805	201	1	5-Aug-06	20	1	16	2	1	1	0	1	3	1	2
6806	201	1	2-Jun-06	10	1	16	2	1	1	0	1	3	1	1
6807	201	1	15-Jun-11	17	1	16	2	1	1	0	4	3	2	1
6808	101	1	10-Mar-11	16	1	16	2	2	1	0	4	3	3	1
6809	101	1	14-Sep-10	18	1	16	2	1	1	0	4	3	3	1
6810	201	1	6-Dec-09	10	1	16	2	1	1	0	4	3	3	1
6811	101	1	3-Feb-06	20	1	16	2	1	1	0	4	3	3	1
6812	201	1	13-Nov-16	15	1	16	2	1	1	0	11	3	1	1
6813	101	1	11-Jul-13	23	1	16	2	1	1	0	11	3	3	1

6814	201	1	9-Jul-11	14	1	16	2	2	1	0	11	3	3	1
6815	101	1	2-Oct-11	20	1	16	2	1	1	0	11	3	2	1
6816	201	1	5-Sep-10	17	1	16	2	1	1	0	11	3	3	1
6817	201	1	24-Nov-10	18	1	16	2	1	1	0	11	3	2	1
6818	201	1	4-Jan-08	13	1	16	2	1	1	0	11	3	2	1
6819	101	1	5-Jul-06	18	1	16	2	2	1	0	11	3	3	1
6820	201	1	3-Aug-17	22	1	16	2	1	1	0	11	3	3	1
6821	101	1	3-Dec-16	16	1	16	2	1	1	0	3	3	1	1
6822	201	1	5-Mar-16	21	1	16	2	1	1	0	3	3	1	1
6823	101	1	22-Jul-16	19	1	16	2	1	1	0	3	3	5	1
6824	201	1	22-Mar-16	19	1	16	2	1	1	0	3	3	1	1
6825	201	1	17-Jan-16	5	1	16	2	1	1	0	3	3	5	1
6826	101	1	15-Jan-16	9	1	16	2	1	1	0	3	3	1	1
6827	101	1	2-Apr-15	21	1	16	2	1	1	0	3	3	3	1
6828	101	1	22-Oct-15	12	1	16	2	1	1	0	3	3	1	1
6829	201	1	22-May-15	20	1	16	2	2	1	0	3	3	2	2
6830	201	1	12-Aug-15	17	1	16	2	1	1	0	3	3	3	1
6831	101	1	5-Feb-15	10	1	16	2	1	1	0	3	3	3	1
6832	201	1	6-Jun-15	22	1	16	2	2	1	0	3	3	3	1
6833	201	1	15-Apr-14	19	1	16	2	3	1	0	3	3	5	2
6834	201	1	29-Dec-14	21	1	16	2	1	1	0	3	3	1	1
6835	101	1	5-Apr-14	14	1	16	2	1	1	0	3	3	2	2
6836	201	1	16-Dec-14	18	1	16	2	1	1	0	3	3	3	1
6837	201	1	30-Mar-14	15	1	16	2	3	1	0	3	3	2	1
6838	101	1	26-May-14	19	1	16	2	1	1	0	3	3	1	1
6839	101	1	19-Jul-14	11	1	16	2	1	1	0	3	3	3	1
6840	101	1	18-Nov-14	23	1	16	2	2	1	0	3	3	2	4
6841	201	1	21-Dec-13	18	1	16	2	2	1	0	3	3	3	1
6842	101	1	11-Jul-13	18	1	16	2	1	1	0	3	3	2	1
6843	201	1	2-Feb-11	14	1	16	2	2	1	0	3	3	1	1
6844	201	1	17-Jan-11	15	1	16	2	1	1	0	3	3	2	1
6845	101	1	18-May-11	22	1	16	2	1	1	0	3	3	1	1
6846	201	1	2-Mar-11	7	1	16	2	1	1	0	3	3	1	1
6847	101	1	23-Jul-11	6	1	16	2	1	1	0	3	3	1	1
6848	201	1	10-Aug-11	17	1	16	2	1	1	0	3	3	3	1
6849	201	1	7-Oct-11	8	1	16	2	2	1	0	3	3	2	2
6850	101	1	19-Oct-10	12	1	16	2	1	1	0	3	3	1	1
6851	201	1	20-Mar-10	22	1	16	2	2	1	0	3	3	2	1
6852	201	1	20-Sep-09	13	1	16	2	1	1	0	3	3	5	1
6853	101	1	18-Jul-09	22	1	16	2	3	1	0	3	3	3	1
6854	101	1	1-Apr-09	22	1	16	2	1	1	0	3	3	5	1
6855	101	1	23-Jun-08	8	1	16	2	1	1	0	3	3	6	1
6856	201	1	9-Dec-08	21	1	16	2	3	1	0	3	3	1	1
6857	201	1	6-Nov-08	17	1	16	2	1	1	0	3	3	2	1
6858	201	1	13-Aug-08	10	1	16	2	2	1	0	3	3	2	2
6859	201	1	21-Apr-08	19	1	16	2	1	1	0	3	3	3	1
6860	201	1	31-Oct-08	22	1	16	2	2	1	0	3	3	3	1
6861	101	1	26-Mar-07	18	1	16	2	1	1	0	3	3	2	1
6862	101	1	23-Jul-07	11	1	16	2	1	1	0	3	3	5	1
6863	101	1	26-Mar-07	19	1	16	2	2	1	0	3	3	2	1
6864	101	1	12-Jun-07	21	1	16	2	1	1	0	3	3	3	1
6865	201	1	26-Jun-06	20	1	16	2	1	1	0	3	3	3	1
6866	101	1	24-Nov-06	11	1	16	2	1	1	0	3	3	3	1
6867	201	1	3-Sep-06	19	1	16	2	1	1	0	3	3	2	2
6868	101	1	8-Jul-06	21	1	16	2	1	1	0	3	3	2	1
6869	101	1	14-May-17	17	1	16	2	1	1	0	3	3	2	2
6870	201	1	8-Aug-17	2	1	16	2	2	1	0	3	3	1	1
6871	101	1	28-Nov-17	21	1	16	2	1	1	0	3	3	3	1
6872	201	1	13-Nov-17	21	1	16	2	1	1	0	3	3	3	1
6873	201	1	11-May-17	17	1	16	2	1	1	0	3	3	3	1
6874	101	1	20-Mar-14	19	1	16	2	1	1	0	6	3	3	1
6875	201	1	16-Jun-11	15	1	16	2	1	1	0	6	3	2	1
6876	201	1	10-Jul-11	9	1	16	2	2	1	0	6	3	3	1
6877	101	1	1-Aug-10	12	1	16	2	1	1	0	6	3	2	1
6878	101	1	23-Nov-07	13	1	16	2	1	1	0	6	3	3	1
6879	101	1	15-Aug-06	17	1	16	2	1	1	0	6	3	3	1
6880	101	1	17-Nov-17	19	1	16	2	1	1	0	13	3	3	1
6881	101	1	16-Nov-17	9	1	16	2	1	1	0	13	3	1	1
6882	201	1	22-Oct-13	21	2	16	2	2	1	0	1	3	5	1
6883	201	1	5-Apr-12	14	2	16	2	2	1	0	1	3	2	1
6884	101	1	18-Nov-14	19	2	16	2	1	1	0	1	3	1	1
6885	101	1	18-Mar-16	12	2	16	2	1	1	0	1	3	5	1
6886	201	1	17-Mar-15	17	2	16	2	1	1	0	1	3	3	1
6887	101	1	20-Jan-14	8	2	16	2	1	1	0	1	3	5	1
6888	101	1	1-Jan-14	15	2	16	2	1	1	0	1	3	2	1
6889	201	1	19-Mar-13	19	2	16	2	3	1	0	1	3	6	1
6890	101	1	20-Feb-13	10	2	16	2	1	1	0	1	3	4	1
6891	101	1	3-Oct-17	16	2	16	2	1	1	0	1	3	5	1
6892	101	1	28-Nov-17	19	2	16	2	1	1	0	1	3	3	1
6893	201	1	20-Aug-12	12	2	16	2	2	1	0	1	3	3	2
6894	101	1	22-Aug-14	20	2	16	2	1	1	0	1	3	1	1
6895	201	1	8-Apr-14	17	2	16	2	2	1	0	1	3	5	1
6896	201	1	9-Sep-13	11	2	16	2	2	1	0	1	3	3	1
6897	101	1	12-Feb-13	15	2	16	2	1	1	0	1	3	5	1
6898	201	1	28-Jan-14	0	2	16	2	2	1	0	1	3	5	1
6899	101	1	29-Mar-14	8	2	16	2	1	1	0	1	3	5	1
6900	101	1	18-Mar-13	7	2	16	2	1	1	0	1	3	3	1
6901	101	1	30-Oct-12	19	2	16	2	2	1	0	1	3	4	1
6902	201	1	7-May-17	7	2	16	2	3	1	0	1	3	5	1
6903	201	1	18-Apr-17	11	2	16	2	1	1	0	1	3	3	1
6904	101	1	28-Feb-16	17	2	16	2	1	1	0	1	3	3	2
6905	101	1	2-Nov-13	9	2	16	2	1	1	0	1	3	3	2
6906	201	1	21-Feb-12	20	2	16	2	3	1	0	1	3	1	1
6907	101	1	1-Aug-13	20	2	16	2	2	1	0	1	3	1	2
6908	201	1	22-Oct-13	18	2	16	2	1	1	0	1	3	3	2
6909	101	1	18-Apr-12	20	2	16	2	1	1	0	1	3	3	1
6910	201	1	21-May-13	13	2	16	2	1	1	0	6	3	3	1
6911	101	1	27-Nov-11	19	2	16	2	1	1	0	1	3	1	1
6912	101	1	31-May-11	8	2	16	2	1	1	0	1	3	5	2
6913	201	1	31-Mar-11	7	2	16	2	2	1	0	1	3	1	1
6914	201	1	11-Dec-11	7	2	16	2	2	1	0	1	3	1	1
6915	101	1	11-Jun-11	2	2	16	2	1	1	0	1	3	1	4
6916	201	1	5-Dec-11	14	2	16	2	2	1	0	1	3	1	2
6917	101	1	18-Jul-10	17	2	16	2	1	1	0	1	3	1	1
6918	201	1	23-Oct-10	23	2	16	2	3	1	0	1	3	5	1
6919	201	1	12-Mar-10	20	2	16	2	2	1	0	1	3	2	1
6920	101	1	9-May-09	17	2	16	2	1	1	0	1	3	5	1
6921	201	1	15-Dec-09	16	2	16	2	2	1	0	1	3	3	1
6922	201	1	30-Nov-09	16	2	16	2	1	1	0	1	3	3	1
6923	201	1	7-Oct-09	19	2	16	2	1	1	0	1	3	3	1
6924	201	1	6-Aug-09	0	2	16	2	2	1	0	1	3	1	1
6925	201	1	7-Oct-09	19	2	16	2	1	1	0	1	3	1	1
6926	201	1	18-Nov-09	8	2	16	2	3	1	0	1	3	6	1
6927	201	1	24-Oct-08	0	2	16	2	1	1	0	1	3	7	2
6928	101	1	28-May-08	9	2	16	2	3	1	0	1	3	4	2
6929	201	1	1-Apr-07	16	2	16	2	2	1	0	1	3	6	1
6930	201	1	1-May-07	18	2	16	2	3	1	0	1	3	1	1
6931	101	1	3-Dec-07	8	2	16	2	1	1	0	1	3	1	2
6932	101	1	4-May-07	15	2	16	2	1	1	0	1	3	3	1
6933	201	1	22-Jun-06	19	2	16	2	3	1	0	1	3	1	2
6934	201	1	16-Dec-06	18	2	16	2	2	1	0	1	3	5	1
6935	101	1	5-Jul-06	21	2	16	2	3	1	0	1	3	3	2
6936	101	1	23-Feb-06	18	2	16	2	1	1	0	1	3	3	1
6937	101	1	23-Nov-11	17	2	16	2	1	1	0	1	3	1	1

6938	101	1	25-Aug-11	5	2	16	2	1	1	0	1	3	3	1
6939	201	1	4-Sep-09	23	2	16	2	1	1	0	1	3	4	1
6940	201	1	26-Oct-07	11	2	16	2	3	1	0	1	3	3	1
6941	201	1	1-Dec-07	17	2	16	2	1	1	0	1	3	3	2
6942	201	1	30-Dec-16	16	2	16	2	1	1	0	11	3	2	1
6943	201	1	26-Jul-16	19	2	16	2	1	1	0	11	3	2	1
6944	201	1	19-Apr-16	20	2	16	2	3	1	0	11	3	2	1
6945	201	1	20-Oct-13	16	2	16	2	1	1	0	11	3	3	1
6946	201	1	21-Sep-10	13	2	16	2	1	1	0	11	3	3	1
6947	201	1	2-Feb-10	22	2	16	2	1	1	0	11	3	3	1
6948	101	1	9-Aug-06	2	2	16	2	1	1	0	11	3	2	1
6949	201	1	28-Jul-16	21	2	16	2	2	1	0	3	3	3	1
6950	101	1	30-May-15	11	2	16	2	1	1	0	3	3	3	1
6951	201	1	3-Mar-14	18	2	16	2	3	1	0	3	3	5	1
6952	101	1	24-Dec-14	14	2	16	2	1	1	0	3	3	2	1
6953	101	1	8-Nov-14	15	2	16	2	1	1	0	3	3	2	1
6954	201	1	16-Sep-14	15	2	16	2	1	1	0	3	3	3	1
6955	101	1	12-Mar-13	17	2	16	2	1	1	0	3	3	5	1
6956	101	1	13-Jul-13	23	2	16	2	1	1	0	3	3	3	1
6957	201	1	5-Oct-12	18	2	16	2	2	1	0	3	3	1	2
6958	201	1	21-Aug-12	21	2	16	2	3	1	0	3	3	2	1
6959	101	1	29-Jun-12	7	2	16	2	1	1	0	3	3	2	1
6960	101	1	15-Jun-11	17	2	16	2	1	1	0	3	3	1	1
6961	101	1	27-Oct-11	22	2	16	2	1	1	0	3	3	2	1
6962	201	1	29-Aug-09	20	2	16	2	1	1	0	3	3	3	1
6963	101	1	10-Jan-08	7	2	16	2	1	1	0	3	3	2	1
6964	101	1	24-Nov-06	8	2	16	2	1	1	0	3	3	3	1
6965	101	1	14-Oct-11	8	2	16	2	1	1	0	6	3	2	2
6966	202	0	23-Feb-12	17	1	17	2	1	1	0	1	3	6	2
6967	202	0	3-Nov-13	15	1	17	2	3	1	0	1	3	1	1
6968	202	0	7-May-16	0	1	17	2	1	1	0	1	3	1	1
6969	202	0	13-Dec-15	5	1	17	2	1	1	0	1	3	5	1
6970	202	0	11-Jul-17	23	1	17	2	1	1	0	1	3	5	1
6971	202	0	25-Apr-16	19	1	17	2	2	1	0	1	3	5	1
6972	202	0	12-Jan-12	12	1	17	2	1	1	0	1	3	2	1
6973	202	0	19-Jul-16	10	1	17	2	1	1	0	1	3	3	2
6974	202	0	13-Jul-13	17	1	17	2	1	1	0	1	3	5	1
6975	202	0	1-Oct-13	2	1	17	2	1	1	0	1	3	1	2
6976	202	0	31-Mar-12	17	1	17	2	1	1	0	1	3	3	1
6977	202	0	24-May-15	14	1	17	2	3	1	0	1	3	5	1
6978	202	0	7-Feb-12	16	1	17	2	1	1	0	1	3	3	1
6979	202	0	21-Nov-15	4	1	17	2	1	1	0	1	3	5	1
6980	202	0	31-Oct-15	6	1	17	2	1	1	0	1	3	1	1
6981	202	0	2-Jun-13	1	1	17	2	1	1	0	1	3	3	2
6982	202	0	11-Sep-12	12	1	17	2	1	1	0	1	3	1	2
6983	202	0	16-Aug-12	14	1	17	2	1	1	0	1	3	3	1
6984	202	0	19-Dec-15	1	1	17	2	1	1	0	1	3	1	1
6985	202	0	19-Dec-15	1	1	17	2	1	1	0	1	3	5	1
6986	202	0	19-Dec-15	1	1	17	2	1	1	0	1	3	1	1
6987	202	0	24-Jun-16	21	1	17	2	1	1	0	1	3	1	2
6988	202	0	29-May-16	22	1	17	2	1	1	0	1	3	5	1
6989	202	0	19-Mar-16	0	1	17	2	3	1	0	1	3	6	1
6990	202	0	19-Mar-16	0	1	17	2	1	1	0	1	3	6	1
6991	202	0	20-Feb-16	21	1	17	2	1	1	0	1	3	1	1
6992	202	0	18-Nov-16	23	1	17	2	1	1	0	1	3	1	1
6993	202	0	28-Aug-16	2	1	17	2	1	1	0	1	3	4	1
6994	202	0	25-Nov-16	13	1	17	2	2	1	0	1	3	2	4
6995	202	0	4-Oct-16	1	1	17	2	3	1	0	1	3	5	1
6996	202	0	3-Sep-15	23	1	17	2	3	1	0	1	3	1	2
6997	202	0	5-Oct-15	12	1	17	2	1	1	0	1	3	4	2
6998	202	0	24-Oct-15	3	1	17	2	1	1	0	1	3	1	1
6999	202	0	25-Sep-15	17	1	17	2	1	1	0	1	3	5	2
7000	202	0	23-Sep-15	22	1	17	2	3	1	0	1	3	5	1
7001	202	0	7-Nov-15	20	1	17	2	1	1	0	1	3	3	1
7002	202	0	13-Jan-14	17	1	17	2	1	1	0	1	3	1	1
7003	202	0	21-Nov-14	1	1	17	2	1	1	0	1	3	5	1
7004	202	0	25-Jul-14	21	1	17	2	3	1	0	1	3	5	1
7005	202	0	18-Apr-14	22	1	17	2	1	1	0	1	3	1	2
7006	202	0	11-Jul-14	16	1	17	2	1	1	0	1	3	1	2
7007	202	0	1-Dec-14	19	1	17	2	1	1	0	1	3	1	2
7008	202	0	10-Mar-14	2	1	17	2	1	1	0	1	3	1	1
7009	202	0	13-Nov-13	18	1	17	2	1	1	0	1	3	2	1
7010	202	0	10-Nov-13	3	1	17	2	1	1	0	1	3	1	1
7011	202	0	27-Jul-13	5	1	17	2	1	1	0	1	3	5	1
7012	202	0	19-Sep-13	5	1	17	2	1	1	0	1	3	6	1
7013	202	0	1-Aug-13	3	1	17	2	3	1	0	1	3	5	2
7014	202	0	4-May-13	13	1	17	2	1	1	0	1	3	3	1
7015	202	0	12-Aug-13	2	1	17	2	1	1	0	1	3	1	2
7016	202	0	21-Sep-13	1	1	17	2	1	1	0	1	3	3	1
7017	202	0	4-Jul-13	3	1	17	2	1	1	0	1	3	1	2
7018	202	0	21-Nov-13	21	1	17	2	1	1	0	1	3	5	1
7019	202	0	20-Sep-13	0	1	17	2	1	1	0	1	3	5	1
7020	202	0	19-Sep-13	5	1	17	2	1	1	0	1	3	5	1
7021	202	0	7-Sep-13	19	1	17	2	1	1	0	1	3	5	2
7022	202	0	10-Aug-12	13	1	17	2	3	1	0	1	3	5	2
7023	202	0	3-Oct-12	1	1	17	2	1	1	0	1	3	5	1
7024	202	0	8-Nov-12	21	1	17	2	1	1	0	1	3	5	2
7025	202	0	18-Aug-12	1	1	17	2	1	1	0	1	3	6	2
7026	202	0	18-Jul-12	2	1	17	2	1	1	0	1	3	6	1
7027	202	0	21-Dec-12	14	1	17	2	1	1	0	1	3	1	1
7028	202	0	13-Apr-12	7	1	17	2	1	1	0	1	3	1	2
7029	202	0	12-Apr-12	22	1	17	2	1	1	0	1	3	3	1
7030	202	0	30-Mar-12	21	1	17	2	1	1	0	1	3	3	1
7031	202	0	2-Nov-12	20	1	17	2	1	1	0	1	3	3	2
7032	202	0	3-Oct-17	23	1	17	2	1	1	0	1	3	6	1
7033	202	0	19-Aug-17	15	1	17	2	1	1	0	1	3	5	1
7034	202	0	25-Jan-17	22	1	17	2	1	1	0	1	3	1	2
7035	202	0	14-Jan-17	0	1	17	2	2	1	0	1	3	5	1
7036	202	0	17-Apr-17	19	1	17	2	3	1	0	1	3	3	1
7037	202	0	22-Apr-17	1	1	17	2	1	1	0	1	3	4	2
7038	202	0	26-Mar-13	17	1	17	2	1	1	0	1	3	1	1
7039	202	0	28-Feb-13	10	1	17	2	1	1	0	4	3	2	1
7040	202	0	18-Apr-13	19	1	17	2	1	1	0	10	3	3	1
7041	202	0	23-Mar-11	13	1	17	2	1	1	0	13	3	6	2
7042	202	0	28-Aug-12	17	1	17	2	1	1	0	1	3	3	1
7043	202	0	16-Apr-11	3	1	17	2	1	1	0	1	3	6	1
7044	202	0	26-Aug-11	23	1	17	2	1	1	0	1	3	6	2
7045	202	0	24-Aug-11	3	1	17	2	1	1	0	1	3	6	1
7046	202	0	16-Oct-11	3	1	17	2	1	1	0	1	3	6	1
7047	202	0	14-Oct-11	19	1	17	2	1	1	0	1	3	2	1
7048	202	0	21-Oct-11	23	1	17	2	1	1	0	1	3	1	2
7049	202	0	5-Feb-11	18	1	17	2	1	1	0	1	3	1	2
7050	202	0	8-May-11	0	1	17	2	1	1	0	1	3	1	1
7051	202	0	21-Oct-11	22	1	17	2	1	1	0	1	3	1	1
7052	202	0	7-Apr-11	1	1	17	2	1	1	0	1	3	3	2
7053	202	0	27-Jan-11	21	1	17	2	1	1	0	1	3	3	2
7054	202	0	7-Mar-11	12	1	17	2	3	1	0	1	3	2	2
7055	202	0	23-Jan-11	11	1	17	2	3	1	0	1	3	2	1
7056	202	0	16-Apr-11	3	1	17	2	1	1	0	1	3	3	2
7057	202	0	12-Nov-10	18	1	17	2	3	1	0	1	3	5	1
7058	202	0	12-Nov-10	17	1	17	2	1	1	0	1	3	5	1
7059	202	0	12-Nov-10	18	1	17	2	3	1	0	1	3	5	1
7060	202	0	19-Jul-10	22	1	17	2	1	1	0	1	3	1	1
7061	202	0	3-Jul-10	17	1	17	2	1	1	0	1	3	1	1

7062	202	0	21-Jul-10	2	1	17	2	1	1	0	1	3	1	2
7063	202	0	6-Jul-10	15	1	17	2	1	1	0	1	3	1	1
7064	202	0	28-Dec-10	18	1	17	2	1	1	0	1	3	1	1
7065	202	0	20-Dec-10	22	1	17	2	1	1	0	1	3	2	1
7066	202	0	12-Nov-10	17	1	17	2	1	1	0	1	3	3	2
7067	202	0	5-Jul-10	19	1	17	2	1	1	0	1	3	3	2
7068	202	0	21-Nov-10	19	1	17	2	3	1	0	1	3	3	2
7069	202	0	19-Sep-09	19	1	17	2	1	1	0	1	3	1	2
7070	202	0	30-Nov-09	6	1	17	2	1	1	0	1	3	6	2
7071	202	0	12-Sep-09	18	1	17	2	1	1	0	1	3	6	1
7072	202	0	9-Dec-09	0	1	17	2	1	1	0	1	3	6	1
7073	202	0	23-Aug-09	0	1	17	2	1	1	0	1	3	6	2
7074	202	0	15-Nov-09	3	1	17	2	1	1	0	1	3	1	2
7075	202	0	30-Apr-09	16	1	17	2	1	1	0	1	3	2	2
7076	202	0	21-Apr-09	19	1	17	2	1	1	0	1	3	3	1
7077	202	0	26-May-09	20	1	17	2	1	1	0	1	3	2	1
7078	202	0	15-Oct-09	3	1	17	2	1	1	0	1	3	6	2
7079	202	0	30-Nov-09	5	1	17	2	1	1	0	1	3	1	2
7080	202	0	10-Feb-08	3	1	17	2	1	1	0	1	3	3	2
7081	202	0	7-Feb-08	9	1	17	2	1	1	0	1	3	1	1
7082	202	0	26-Mar-08	15	1	17	2	1	1	0	1	3	6	1
7083	202	0	22-Jan-08	19	1	17	2	1	1	0	1	3	1	1
7084	202	0	1-Oct-08	23	1	17	2	1	1	0	1	3	5	2
7085	202	0	5-Dec-08	10	1	17	2	1	1	0	1	3	3	2
7086	202	0	21-Nov-07	21	1	17	2	3	1	0	1	3	3	1
7087	202	0	25-Nov-07	13	1	17	2	1	1	0	1	3	3	1
7088	202	0	25-Feb-07	2	1	17	2	1	1	0	1	3	3	2
7089	202	0	26-May-07	19	1	17	2	1	1	0	1	3	6	2
7090	202	0	6-Oct-07	22	1	17	2	1	1	0	1	3	6	2
7091	202	0	12-Mar-07	13	1	17	2	1	1	0	1	3	1	2
7092	202	0	24-May-07	0	1	17	2	1	1	0	1	3	6	2
7093	202	0	3-May-07	19	1	17	2	1	1	0	1	3	3	2
7094	202	0	7-Nov-07	10	1	17	2	3	1	0	1	3	2	2
7095	202	0	29-Jan-06	17	1	17	2	1	1	0	1	3	6	1
7096	202	0	8-Mar-06	17	1	17	2	1	1	0	1	3	2	2
7097	202	0	4-Mar-06	0	1	17	2	1	1	0	1	3	1	2
7098	202	0	8-Mar-06	12	1	17	2	2	1	0	1	3	1	1
7099	202	0	22-Feb-06	17	1	17	2	1	1	0	1	3	1	2
7100	202	0	27-Feb-06	13	1	17	2	1	1	0	1	3	2	2
7101	202	0	29-Apr-10	18	1	17	2	1	1	0	1	3	1	2
7102	202	0	23-Feb-10	22	1	17	2	2	1	0	1	3	6	1
7103	202	0	6-Jan-10	19	1	17	2	1	1	0	1	3	2	2
7104	202	0	21-Mar-09	22	1	17	2	1	1	0	1	3	3	2
7105	202	0	8-Jul-08	10	1	17	2	3	1	0	1	3	2	2
7106	202	0	22-Mar-13	16	1	17	2	1	1	0	4	3	1	1
7107	202	0	5-Nov-11	2	1	17	2	1	1	0	4	3	2	2
7108	202	0	1-Oct-10	2	1	17	2	1	1	0	4	3	1	2
7109	202	0	13-Feb-08	1	1	17	2	2	1	0	4	3	1	2
7110	202	0	17-Nov-08	18	1	17	2	1	1	0	4	3	1	2
7111	202	0	9-Jun-07	22	1	17	2	1	1	0	4	3	1	1
7112	202	0	11-Dec-07	18	1	17	2	1	1	0	4	3	2	1
7113	202	0	13-Dec-06	14	1	17	2	1	1	0	4	3	1	1
7114	202	0	21-May-14	12	1	17	2	3	1	0	11	3	3	1
7115	202	0	27-Apr-16	15	1	17	2	2	1	0	3	3	5	1
7116	202	0	2-Apr-14	22	1	17	2	1	1	0	3	3	2	1
7117	202	0	30-Nov-14	4	1	17	2	1	1	0	3	3	5	2
7118	202	0	9-Dec-13	20	1	17	2	3	1	0	3	3	5	1
7119	202	0	22-Mar-13	20	1	17	2	1	1	0	3	3	2	2
7120	202	0	9-Jun-11	16	1	17	2	1	1	0	3	3	2	2
7121	202	0	14-Jun-11	14	1	17	2	2	1	0	3	3	5	1
7122	202	0	18-Sep-11	3	1	17	2	1	1	0	3	3	2	1
7123	202	0	22-Jul-10	8	1	17	2	1	1	0	3	3	6	2
7124	202	0	29-Jul-10	19	1	17	2	1	1	0	3	3	5	1
7125	202	0	4-Jun-09	14	1	17	2	1	1	0	3	3	3	1
7126	202	0	2-Aug-09	15	1	17	2	1	1	0	3	3	3	2
7127	202	0	24-Dec-09	18	1	17	2	2	1	0	3	3	2	1
7128	202	0	16-Mar-08	7	1	17	2	1	1	0	3	3	1	1
7129	202	0	16-Nov-08	1	1	17	2	1	1	0	3	3	2	2
7130	202	0	26-Nov-06	11	1	17	2	1	1	0	3	3	2	1
7131	202	0	29-Jan-17	10	1	17	2	1	1	0	3	3	7	1
7132	202	0	1-Oct-17	3	1	17	2	1	1	0	3	3	2	1
7133	202	0	16-Aug-17	2	1	17	2	1	1	0	3	3	1	1
7134	202	0	24-Aug-17	1	1	17	2	1	1	0	3	3	2	1
7135	202	0	12-Aug-17	18	1	17	2	1	1	0	3	3	3	2
7136	202	0	7-Sep-17	0	1	17	2	3	1	0	3	3	5	1
7137	202	0	20-Feb-16	12	1	17	2	1	1	0	6	3	2	1
7138	202	0	8-Jun-16	14	1	17	2	1	1	0	6	3	3	1
7139	202	0	17-Aug-07	22	1	17	2	1	1	0	13	3	1	1
7140	202	0	22-Dec-06	18	1	17	2	2	1	0	13	3	1	1
7141	202	0	10-Feb-17	14	2	17	2	1	1	0	1	3	4	2
7142	202	0	27-Mar-16	11	2	17	2	1	1	0	1	3	1	2
7143	202	0	25-Jul-14	12	2	17	2	1	1	0	1	3	3	2
7144	202	0	8-Jun-12	22	2	17	2	1	1	0	1	3	2	2
7145	202	0	19-Jul-17	22	2	17	2	1	1	0	1	3	3	1
7146	202	0	6-Jan-17	22	2	17	2	1	1	0	1	3	5	1
7147	202	0	18-Feb-16	20	2	17	2	1	1	0	1	3	3	1
7148	202	0	9-Jun-16	23	2	17	2	1	1	0	1	3	1	1
7149	202	0	5-Feb-14	3	2	17	2	1	1	0	1	3	3	2
7150	202	0	14-Jun-14	17	2	17	2	1	1	0	1	3	3	1
7151	202	0	13-Oct-13	17	2	17	2	1	1	0	1	3	4	1
7152	202	0	21-Nov-15	19	2	17	2	2	1	0	1	3	5	1
7153	202	0	2-Mar-12	21	2	17	2	1	1	0	1	3	5	1
7154	202	0	15-Jul-15	23	2	17	2	1	1	0	1	3	3	1
7155	202	0	20-Jun-16	2	2	17	2	2	1	0	1	3	3	1
7156	202	0	26-Aug-16	4	2	17	2	1	1	0	1	3	3	1
7157	202	0	26-Jun-16	2	2	17	2	1	1	0	1	3	6	2
7158	202	0	18-Aug-15	23	2	17	2	2	1	0	1	3	2	1
7159	202	0	14-Nov-15	3	2	17	2	1	1	0	1	3	5	1
7160	202	0	5-Dec-15	13	2	17	2	1	1	0	1	3	5	1
7161	202	0	13-Nov-15	20	2	17	2	1	1	0	1	3	3	1
7162	202	0	17-Jun-14	18	2	17	2	1	1	0	1	3	2	1
7163	202	0	9-May-14	20	2	17	2	1	1	0	1	3	1	1
7164	202	0	27-Dec-14	17	2	17	2	1	1	0	1	3	3	1
7165	202	0	1-Aug-14	0	2	17	2	1	1	0	1	3	5	2
7166	202	0	23-May-13	21	2	17	2	1	1	0	1	3	1	1
7167	202	0	12-Jun-13	1	2	17	2	1	1	0	1	3	4	1
7168	202	0	9-Sep-12	15	2	17	2	1	1	0	1	3	5	1
7169	202	0	25-Aug-12	23	2	17	2	1	1	0	1	3	6	1
7170	202	0	11-Aug-12	22	2	17	2	2	1	0	1	3	1	2
7171	202	0	2-Jul-12	0	2	17	2	1	1	0	1	3	1	1
7172	202	0	3-Aug-12	2	2	17	2	1	1	0	1	3	2	2
7173	202	0	2-Jul-12	17	2	17	2	1	1	0	1	3	3	2
7174	202	0	12-Jan-12	12	2	17	2	1	1	0	1	3	4	1
7175	202	0	12-Jan-12	12	2	17	2	1	1	0	1	3	4	1
7176	202	0	2-Mar-12	23	2	17	2	1	1	0	1	3	3	1
7177	202	0	11-Sep-17	0	2	17	2	2	1	0	1	3	1	1
7178	202	0	24-Mar-13	20	2	17	2	1	1	0	1	3	3	1
7179	202	0	5-Apr-11	3	2	17	2	1	1	0	1	3	6	1
7180	202	0	27-Dec-11	10	2	17	2	1	1	0	1	3	1	1
7181	202	0	16-Aug-11	3	2	17	2	1	1	0	1	3	1	2
7182	202	0	12-Nov-10	17	2	17	2	1	1	0	1	3	6	2
7183	202	0	12-Nov-10	17	2	17	2	1	1	0	1	3	5	1
7184	202	0	23-May-10	21	2	17	2	1	1	0	1	3	6	1
7185	202	0	22-Jul-10	12	2	17	2	1	1	0	1	3	6	2

7186	202	0	29-Sep-10	13	2	17	2	3	1	0	1	3	3	1
7187	202	0	12-Nov-10	17	2	17	2	1	1	0	1	3	3	2
7188	202	0	26-Jul-10	14	2	17	2	3	1	0	1	3	6	3
7189	202	0	22-Nov-09	22	2	17	2	1	1	0	1	3	1	1
7190	202	0	11-Oct-09	19	2	17	2	1	1	0	1	3	1	2
7191	202	0	13-Dec-09	3	2	17	2	1	1	0	1	3	3	1
7192	202	0	15-Dec-09	6	2	17	2	1	1	0	1	3	1	1
7193	202	0	3-Mar-08	20	2	17	2	1	1	0	1	3	5	2
7194	202	0	4-Jan-08	12	2	17	2	1	1	0	1	3	3	2
7195	202	0	5-Feb-08	20	2	17	2	1	1	0	1	3	1	1
7196	202	0	2-Jun-08	23	2	17	2	1	1	0	1	3	3	1
7197	202	0	14-Nov-08	22	2	17	2	1	1	0	1	3	2	1
7198	202	0	31-Jan-07	17	2	17	2	3	1	0	1	3	3	1
7199	202	0	13-May-07	10	2	17	2	1	1	0	1	3	1	2
7200	202	0	2-May-07	7	2	17	2	2	1	0	1	3	1	1
7201	202	0	29-Aug-07	4	2	17	2	1	1	0	1	3	2	2
7202	202	0	25-Nov-07	16	2	17	2	1	1	0	1	3	4	1
7203	202	0	24-Apr-06	20	2	17	2	1	1	0	1	3	3	1
7204	202	0	29-Mar-07	13	2	17	2	1	1	0	1	3	5	1
7205	202	0	14-Mar-07	7	2	17	2	1	1	0	1	3	3	1
7206	202	0	5-Jul-06	16	2	17	2	1	1	0	1	3	6	1
7207	202	0	15-Aug-09	22	2	17	2	1	1	0	11	3	3	2
7208	202	0	5-Aug-17	12	2	17	2	2	1	0	11	3	3	2
7209	202	0	24-Nov-16	17	2	17	2	2	1	0	3	3	1	1
7210	202	0	12-Nov-16	23	2	17	2	3	1	0	3	3	5	1
7211	202	0	8-May-16	4	2	17	2	2	1	0	3	3	3	1
7212	202	0	2-Jul-15	16	2	17	2	1	1	0	3	3	1	1
7213	202	0	29-Oct-15	2	2	17	2	3	1	0	3	3	5	1
7214	202	0	15-Sep-14	20	2	17	2	1	1	0	3	3	1	1
7215	202	0	10-Mar-11	13	2	17	2	1	1	0	3	3	1	1
7216	202	0	24-Jan-11	22	2	17	2	3	1	0	3	3	1	1
7217	202	0	12-Nov-07	18	2	17	2	1	1	0	3	3	1	1
7218	101	1	6-May-12	11	1	17	2	1	1	0	1	3	3	1
7219	201	1	3-Sep-15	5	1	17	2	2	1	0	1	3	5	1
7220	201	1	5-Oct-14	0	1	17	2	2	1	0	1	3	3	1
7221	201	1	27-Oct-16	19	1	17	2	2	1	0	1	3	5	1
7222	201	1	22-Sep-16	13	1	17	2	2	1	0	1	3	5	1
7223	201	1	25-Sep-16	6	1	17	2	2	1	0	1	3	1	1
7224	201	1	25-Mar-16	12	1	17	2	2	1	0	1	3	3	2
7225	101	1	9-Dec-16	19	1	17	2	1	1	0	1	3	3	1
7226	201	1	24-Jul-16	19	1	17	2	2	1	0	1	3	3	2
7227	201	1	23-Sep-15	4	1	17	2	2	1	0	1	3	1	1
7228	201	1	4-Dec-15	13	1	17	2	1	1	0	1	3	5	1
7229	101	1	26-Dec-15	22	1	17	2	1	1	0	1	3	2	1
7230	201	1	24-Jul-15	19	1	17	2	3	1	0	1	3	5	1
7231	201	1	28-Nov-15	20	1	17	2	3	1	0	1	3	4	1
7232	201	1	6-Aug-15	23	1	17	2	3	1	0	1	3	3	2
7233	201	1	6-Feb-15	19	1	17	2	2	1	0	1	3	2	1
7234	201	1	2-May-15	11	1	17	2	2	1	0	1	3	4	2
7235	201	1	28-Apr-15	21	1	17	2	3	1	0	1	3	3	1
7236	101	1	8-Oct-14	20	1	17	2	1	1	0	1	3	5	1
7237	201	1	24-Feb-14	19	1	17	2	1	1	0	1	3	2	1
7238	201	1	26-Sep-14	16	1	17	2	1	1	0	1	3	5	1
7239	201	1	25-May-14	11	1	17	2	3	1	0	1	3	5	1
7240	101	1	28-Mar-14	15	1	17	2	1	1	0	1	3	1	1
7241	101	1	10-Nov-14	21	1	17	2	1	1	0	1	3	1	1
7242	201	1	14-Jul-14	19	1	17	2	3	1	0	1	3	4	1
7243	201	1	19-Feb-14	14	1	17	2	2	1	0	1	3	3	1
7244	201	1	8-Jan-14	20	1	17	2	1	1	0	1	3	2	1
7245	201	1	6-Jan-14	9	1	17	2	2	1	0	1	3	2	1
7246	201	1	20-Jul-14	0	1	17	2	2	1	0	1	3	3	1
7247	201	1	18-Jul-13	20	1	17	2	1	1	0	1	3	5	1
7248	201	1	16-Nov-13	11	1	17	2	3	1	0	1	3	4	1
7249	201	1	7-May-13	13	1	17	2	3	1	0	1	3	2	1
7250	101	1	7-Sep-13	17	1	17	2	2	1	0	1	3	2	4
7251	201	1	17-Jul-13	10	1	17	2	3	1	0	1	3	3	1
7252	101	1	23-Sep-13	15	1	17	2	1	1	0	1	3	2	1
7253	101	1	8-Jul-12	17	1	17	2	1	1	0	1	3	1	2
7254	201	1	13-Nov-12	14	1	17	2	2	1	0	1	3	6	1
7255	201	1	1-May-17	7	1	17	2	1	1	0	1	3	1	1
7256	201	1	17-Jun-17	9	1	17	2	2	1	0	1	3	5	1
7257	201	1	18-May-17	12	1	17	2	2	1	0	1	3	5	1
7258	201	1	1-Jan-16	16	1	17	2	1	1	0	3	3	5	1
7259	201	1	4-Feb-12	7	1	17	2	1	1	0	1	3	2	2
7260	201	1	24-Feb-14	0	1	17	2	3	1	0	1	3	3	2
7261	201	1	8-Dec-12	23	1	17	2	3	1	0	1	3	1	1
7262	101	1	13-Feb-12	22	1	17	2	2	1	0	1	3	3	1
7263	201	1	26-Jun-16	7	1	17	2	2	1	0	1	3	5	1
7264	201	1	31-Dec-16	6	1	17	2	1	1	0	1	3	2	1
7265	201	1	20-Jun-15	16	1	17	2	3	1	0	1	3	1	2
7266	201	1	28-Aug-16	23	1	17	2	2	1	0	1	3	4	2
7267	201	1	22-Apr-16	9	1	17	2	1	1	0	1	3	1	1
7268	201	1	12-Jul-14	1	1	17	2	2	1	0	1	3	5	1
7269	201	1	27-Aug-13	14	1	17	2	3	1	0	1	3	5	1
7270	201	1	29-Aug-13	15	1	17	2	3	1	0	1	3	1	1
7271	201	1	10-Sep-13	1	1	17	2	3	1	0	1	3	1	2
7272	101	1	16-Aug-12	2	1	17	2	3	1	0	1	3	2	2
7273	101	1	22-Oct-12	19	1	17	2	3	1	0	1	3	3	1
7274	201	1	26-Jan-12	19	1	17	2	1	1	0	1	3	1	2
7275	201	1	14-Oct-14	20	1	17	2	2	1	0	1	3	5	2
7276	101	1	30-Oct-17	13	1	17	2	1	1	0	1	3	3	2
7277	101	1	7-Apr-16	17	1	17	2	1	1	0	1	3	2	1
7278	101	1	22-Jan-12	15	1	17	2	1	1	0	1	3	5	4
7279	101	1	11-Aug-13	19	1	17	2	3	1	0	7	3	3	1
7280	201	1	22-Feb-13	21	1	17	2	2	1	0	1	3	6	2
7281	101	1	28-Feb-13	15	1	17	2	1	1	0	1	3	1	2
7282	201	1	13-Nov-13	7	1	17	2	2	1	0	1	3	5	1
7283	101	1	19-Dec-13	19	1	17	2	1	1	0	1	3	2	2
7284	201	1	16-Jan-12	23	1	17	2	2	1	0	1	3	3	2
7285	101	1	31-May-12	3	1	17	2	1	1	0	1	3	5	1
7286	201	1	13-Jan-12	20	1	17	2	2	1	0	1	3	1	1
7287	101	1	31-May-12	3	1	17	2	1	1	0	1	3	1	2
7288	201	1	8-Nov-12	4	1	17	2	3	1	0	1	3	6	1
7289	201	1	6-Apr-14	23	1	17	2	2	1	0	6	3	2	1
7290	101	1	21-Dec-12	12	1	17	2	3	1	0	10	3	2	2
7291	101	1	1-Nov-16	1	1	17	2	1	1	0	1	3	2	2
7292	101	1	13-Jul-16	7	1	17	2	1	1	0	1	3	3	1
7293	101	1	29-Mar-13	21	1	17	2	1	1	0	1	3	3	2
7294	101	1	25-Oct-13	16	1	17	2	1	1	0	1	3	3	1
7295	201	1	10-Mar-12	3	1	17	2	1	1	0	1	3	1	1
7296	101	1	26-Jul-11	22	1	17	2	1	1	0	1	3	5	2
7297	101	1	19-Oct-11	9	1	17	2	1	1	0	1	3	5	1
7298	101	1	2-Dec-11	17	1	17	2	2	1	0	1	3	4	2
7299	101	1	13-Nov-11	19	1	17	2	1	1	0	1	3	1	4
7300	101	1	26-Nov-11	2	1	17	2	1	1	0	1	3	1	2
7301	101	1	11-Sep-11	2	1	17	2	1	1	0	1	3	5	1
7302	201	1	24-Mar-11	17	1	17	2	1	1	0	1	3	4	2
7303	201	1	11-Dec-11	8	1	17	2	2	1	0	1	3	6	2
7304	201	1	17-Mar-11	16	1	17	2	2	1	0	1	3	1	1
7305	101	1	18-Sep-11	15	1	17	2	1	1	0	1	3	1	1
7306	201	1	14-Nov-10	13	1	17	2	3	1	0	1	3	4	2
7307	101	1	9-Mar-10	17	1	17	2	1	1	0	1	3	5	1
7308	101	1	15-May-10	15	1	17	2	2	1	0	1	3	1	2
7309	101	1	23-Mar-10	18	1	17	2	1	1	0	1	3	4	1

7310	201	1	27-Dec-10	12	1	17	2	3	1	0	1	3	5	1
7311	201	1	29-May-10	20	1	17	2	2	1	0	1	3	4	1
7312	201	1	29-Aug-10	23	1	17	2	2	1	0	1	3	1	2
7313	201	1	5-Dec-10	20	1	17	2	1	1	0	1	3	3	1
7314	101	1	23-Aug-09	23	1	17	2	2	1	0	1	3	1	1
7315	201	1	21-Aug-09	7	1	17	2	2	1	0	1	3	4	1
7316	201	1	7-Dec-09	22	1	17	2	2	1	0	1	3	3	1
7317	201	1	27-Apr-09	20	1	17	2	1	1	0	1	3	5	1
7318	101	1	15-Nov-08	3	1	17	2	1	1	0	1	3	1	2
7319	101	1	2-Nov-08	14	1	17	2	1	1	0	1	3	3	1
7320	101	1	5-Sep-08	10	1	17	2	2	1	0	1	3	4	1
7321	201	1	15-Sep-08	3	1	17	2	3	1	0	1	3	1	2
7322	201	1	16-Jan-08	17	1	17	2	2	1	0	1	3	2	1
7323	101	1	20-Jul-07	11	1	17	2	1	1	0	1	3	3	1
7324	201	1	17-Jan-07	7	1	17	2	1	1	0	1	3	5	1
7325	201	1	2-Jul-07	12	1	17	2	1	1	0	1	3	1	1
7326	201	1	17-Feb-07	9	1	17	2	1	1	0	1	3	1	2
7327	201	1	5-Jan-07	3	1	17	2	1	1	0	1	3	6	1
7328	101	1	29-Jun-07	22	1	17	2	1	1	0	1	3	5	1
7329	201	1	10-Mar-07	23	1	17	2	1	1	0	1	3	1	1
7330	201	1	8-Sep-07	1	1	17	2	1	1	0	1	3	1	1
7331	201	1	11-Dec-07	21	1	17	2	1	1	0	1	3	6	1
7332	201	1	22-Jun-07	22	1	17	2	2	1	0	1	3	1	1
7333	201	1	16-Aug-07	9	1	17	2	1	1	0	1	3	1	2
7334	201	1	25-Jul-07	19	1	17	2	4	1	0	1	3	3	1
7335	201	1	15-May-07	20	1	17	2	1	1	0	1	3	2	1
7336	201	1	8-Jan-07	0	1	17	2	2	1	0	1	3	1	1
7337	201	1	12-Jun-07	11	1	17	2	2	1	0	1	3	3	4
7338	201	1	12-Jun-07	11	1	17	2	2	1	0	1	3	3	4
7339	101	1	22-Jun-07	22	1	17	2	1	1	0	1	3	3	1
7340	101	1	6-Feb-06	12	1	17	2	3	1	0	1	3	1	1
7341	201	1	4-Mar-06	15	1	17	2	1	1	0	1	3	5	2
7342	101	1	28-Mar-06	14	1	17	2	1	1	0	1	3	3	1
7343	101	1	28-Sep-06	7	1	17	2	1	1	0	1	3	1	4
7344	201	1	26-Dec-06	22	1	17	2	2	1	0	1	3	3	1
7345	201	1	2-Jul-06	20	1	17	2	2	1	0	1	3	2	2
7346	201	1	19-Sep-06	2	1	17	2	1	1	0	1	3	6	1
7347	101	1	3-Dec-06	18	1	17	2	1	1	0	1	3	1	1
7348	201	1	28-Oct-06	21	1	17	2	1	1	0	1	3	1	2
7349	101	1	27-Oct-06	11	1	17	2	1	1	0	1	3	3	1
7350	201	1	9-Jun-06	22	1	17	2	1	1	0	1	3	4	2
7351	101	1	31-Dec-12	0	1	17	2	1	1	0	1	3	5	1
7352	101	1	31-Aug-11	7	1	17	2	2	1	0	1	3	5	1
7353	201	1	11-Jun-11	17	1	17	2	2	1	0	1	3	6	1
7354	201	1	25-Jan-11	0	1	17	2	1	1	0	1	3	2	2
7355	201	1	30-Jun-11	22	1	17	2	1	1	0	1	3	3	1
7356	101	1	15-Dec-10	13	1	17	2	1	1	0	1	3	5	1
7357	201	1	12-Jan-10	19	1	17	2	2	1	0	1	3	2	1
7358	201	1	24-Oct-10	18	1	17	2	2	1	0	1	3	2	1
7359	101	1	25-Apr-10	8	1	17	2	1	1	0	1	3	5	1
7360	201	1	21-Aug-10	12	1	17	2	3	1	0	1	3	3	1
7361	101	1	31-May-09	20	1	17	2	1	1	0	1	3	5	1
7362	201	1	11-Apr-09	18	1	17	2	1	1	0	1	3	5	1
7363	101	1	21-Dec-09	22	1	17	2	1	1	0	1	3	1	1
7364	201	1	9-Jul-09	13	1	17	2	2	1	0	1	3	5	1
7365	101	1	26-Aug-09	17	1	17	2	1	1	0	1	3	3	1
7366	201	1	31-Jul-09	14	1	17	2	1	1	0	1	3	1	1
7367	201	1	16-Mar-08	20	1	17	2	2	1	0	1	3	5	1
7368	201	1	26-May-08	10	1	17	2	1	1	0	1	3	5	1
7369	201	1	14-Feb-08	1	1	17	2	1	1	0	1	3	7	1
7370	101	1	12-Nov-08	12	1	17	2	1	1	0	1	3	2	1
7371	101	1	6-May-07	20	1	17	2	1	1	0	1	3	1	1
7372	101	1	8-Dec-06	18	1	17	2	1	1	0	1	3	1	1
7373	201	1	10-Sep-06	22	1	17	2	1	1	0	1	3	1	2
7374	201	1	27-Apr-06	17	1	17	2	1	1	0	1	3	6	1
7375	201	1	7-Jun-06	15	1	17	2	1	1	0	1	3	5	1
7376	201	1	25-Dec-06	12	1	17	2	1	1	0	1	3	4	1
7377	101	1	17-Sep-15	10	1	17	2	1	1	0	4	3	4	1
7378	101	1	7-Sep-15	12	1	17	2	1	1	0	4	3	3	1
7379	101	1	30-Apr-11	17	1	17	2	1	1	0	4	3	3	1
7380	101	1	6-Apr-11	22	1	17	2	1	1	0	4	3	2	1
7381	101	1	24-Mar-17	20	1	17	2	1	1	0	4	3	4	1
7382	101	1	2-Oct-17	18	1	17	2	1	1	0	4	3	3	1
7383	101	1	21-Jun-16	12	1	17	2	1	1	0	11	3	2	1
7384	201	1	13-Dec-14	17	1	17	2	2	1	0	11	3	2	1
7385	101	1	18-Apr-12	16	1	17	2	1	1	0	11	3	3	1
7386	101	1	14-Sep-12	17	1	17	2	1	1	0	11	3	2	1
7387	201	1	22-Nov-11	10	1	17	2	3	1	0	11	3	2	1
7388	201	1	5-May-11	12	1	17	2	1	1	0	11	3	3	1
7389	201	1	9-Jul-11	13	1	17	2	2	1	0	11	3	3	1
7390	201	1	2-Mar-10	12	1	17	2	2	1	0	11	3	2	1
7391	201	1	26-Dec-10	15	1	17	2	1	1	0	11	3	3	1
7392	201	1	26-Jul-17	9	1	17	2	1	1	0	11	3	3	1
7393	101	1	10-Jul-17	12	1	17	2	1	1	0	11	3	2	1
7394	201	1	19-Mar-17	6	1	17	2	1	1	0	11	3	2	1
7395	101	1	9-Jul-17	10	1	17	2	3	1	0	11	3	3	1
7396	101	1	2-Mar-16	2	1	17	2	1	1	0	3	3	1	1
7397	101	1	24-Sep-16	1	1	17	2	1	1	0	3	3	2	1
7398	101	1	23-Nov-16	2	1	17	2	1	1	0	3	3	1	1
7399	201	1	22-Jul-16	15	1	17	2	2	1	0	3	3	3	1
7400	201	1	2-Feb-16	15	1	17	2	1	1	0	3	3	2	1
7401	201	1	3-Apr-15	10	1	17	2	1	1	0	3	3	3	1
7402	101	1	13-Jan-15	17	1	17	2	1	1	0	3	3	3	1
7403	101	1	5-Apr-15	17	1	17	2	1	1	0	3	3	3	1
7404	101	1	2-Apr-14	8	1	17	2	1	1	0	3	3	3	1
7405	201	1	23-Jan-14	21	1	17	2	3	1	0	3	3	3	1
7406	101	1	11-Aug-13	16	1	17	2	1	1	0	3	3	3	1
7407	201	1	5-Jul-13	11	1	17	2	2	1	0	3	3	5	1
7408	201	1	22-Oct-13	23	1	17	2	2	1	0	3	3	1	1
7409	101	1	11-Jun-13	17	1	17	2	1	1	0	3	3	2	2
7410	201	1	27-Aug-12	10	1	17	2	3	1	0	3	3	3	1
7411	201	1	2-Mar-11	2	1	17	2	2	1	0	3	3	3	1
7412	201	1	19-Mar-11	9	1	17	2	1	1	0	3	3	1	1
7413	101	1	2-Jul-11	22	1	17	2	3	1	0	3	3	3	1
7414	101	1	4-Dec-11	16	1	17	2	1	1	0	3	3	5	1
7415	101	1	4-Dec-11	17	1	17	2	1	1	0	3	3	5	1
7416	201	1	1-Nov-10	19	1	17	2	1	1	0	3	3	3	1
7417	201	1	1-Nov-10	18	1	17	2	1	1	0	3	3	3	1
7418	101	1	5-Jul-10	19	1	17	2	1	1	0	3	3	1	2
7419	201	1	6-Aug-09	13	1	17	2	3	1	0	3	3	1	2
7420	201	1	18-Oct-09	9	1	17	2	1	1	0	3	3	5	1
7421	101	1	1-Apr-09	22	1	17	2	1	1	0	3	3	5	1
7422	101	1	1-Apr-09	22	1	17	2	1	1	0	3	3	5	1
7423	101	1	26-Oct-08	9	1	17	2	1	1	0	3	3	5	1
7424	201	1	23-May-08	11	1	17	2	1	1	0	3	3	1	1
7425	101	1	28-Mar-08	12	1	17	2	1	1	0	3	3	4	2
7426	201	1	23-Sep-08	20	1	17	2	3	1	0	3	3	1	1
7427	101	1	12-Aug-08	1	1	17	2	1	1	0	3	3	1	1
7428	101	1	8-Jul-08	16	1	17	2	1	1	0	3	3	2	1
7429	201	1	21-Feb-08	8	1	17	2	2	1	0	3	3	1	1
7430	201	1	3-Sep-08	11	1	17	2	3	1	0	3	3	3	1
7431	201	1	22-Nov-08	12	1	17	2	1	1	0	3	3	3	1
7432	101	1	26-Apr-07	18	1	17	2	1	1	0	3	3	5	1
7433	201	1	5-Feb-07	15	1	17	2	1	1	0	3	3	1	1

7434	101	1	20-Jan-07	10	1	17	2	1	1	0	3	3	3	1
7435	101	1	27-Jun-07	18	1	17	2	1	1	0	3	3	3	1
7436	101	1	25-Apr-06	19	1	17	2	1	1	0	3	3	2	1
7437	201	1	6-Dec-06	0	1	17	2	3	1	0	3	3	1	1
7438	201	1	10-Mar-06	23	1	17	2	1	1	0	3	3	3	1
7439	101	1	7-Dec-06	23	1	17	2	1	1	0	3	3	3	1
7440	201	1	21-Feb-17	19	1	17	2	1	1	0	3	3	3	1
7441	201	1	22-Aug-17	19	1	17	2	1	1	0	3	3	3	1
7442	101	1	13-Jan-17	10	1	17	2	1	1	0	3	3	2	1
7443	201	1	30-Jun-17	3	1	17	2	2	1	0	3	3	2	1
7444	201	1	31-Jul-17	10	1	17	2	2	1	0	3	3	2	1
7445	101	1	18-Oct-17	13	1	17	2	1	1	0	3	3	2	1
7446	101	1	8-Oct-17	9	1	17	2	2	1	0	3	3	2	1
7447	201	1	22-May-15	16	1	17	2	2	1	0	6	3	2	2
7448	101	1	18-Jul-14	12	1	17	2	1	1	0	6	3	2	1
7449	201	1	24-Jul-11	11	1	17	2	3	1	0	6	3	2	1
7450	201	1	8-Oct-06	23	1	17	2	2	1	0	6	3	3	1
7451	101	1	16-Nov-17	9	1	17	2	1	1	0	13	3	7	1
7452	101	1	13-Sep-12	8	2	17	2	1	1	0	1	3	3	1
7453	101	1	12-Dec-13	11	2	17	2	1	1	0	1	3	4	1
7454	201	1	1-Oct-16	20	2	17	2	2	1	0	1	3	5	1
7455	201	1	14-Jun-16	1	2	17	2	1	1	0	1	3	1	2
7456	101	1	14-Mar-15	18	2	17	2	1	1	0	1	3	2	4
7457	201	1	27-Jul-15	15	2	17	2	2	1	0	1	3	1	1
7458	201	1	7-Sep-14	0	2	17	2	3	1	0	1	3	1	1
7459	201	1	31-Oct-14	11	2	17	2	3	1	0	1	3	3	1
7460	201	1	13-Feb-14	15	2	17	2	3	1	0	1	3	3	1
7461	201	1	6-Jun-17	19	2	17	2	3	1	0	1	3	2	1
7462	201	1	27-May-13	18	2	17	2	2	1	0	1	3	6	1
7463	201	1	18-Aug-16	1	2	17	2	3	1	0	1	3	1	2
7464	201	1	19-Jan-14	19	2	17	2	2	1	0	1	3	1	2
7465	101	1	7-Jun-14	20	2	17	2	1	1	0	1	3	1	2
7466	201	1	2-Aug-13	11	2	17	2	3	1	0	1	3	3	2
7467	101	1	14-Oct-13	22	2	17	2	1	1	0	1	3	5	2
7468	201	1	7-Jan-15	20	2	17	2	3	1	0	1	3	1	1
7469	201	1	22-Jul-14	22	2	17	2	3	1	0	1	3	5	1
7470	201	1	25-Jul-14	20	2	17	2	2	1	0	1	3	2	1
7471	201	1	10-May-14	23	2	17	2	1	1	0	1	3	3	1
7472	101	1	24-Oct-13	13	2	17	2	1	1	0	1	3	3	1
7473	101	1	7-Mar-12	13	2	17	2	1	1	0	1	3	5	2
7474	101	1	16-Dec-13	7	2	17	2	1	1	0	1	3	3	1
7475	201	1	19-Jan-12	7	2	17	2	3	1	0	1	3	5	1
7476	101	1	27-May-12	18	2	17	2	1	1	0	1	3	5	1
7477	101	1	10-Feb-12	6	2	17	2	1	1	0	1	3	3	1
7478	201	1	3-Aug-16	21	2	17	2	2	1	0	6	3	5	1
7479	101	1	21-Nov-14	7	2	17	2	1	1	0	1	3	5	2
7480	101	1	14-Oct-13	22	2	17	2	1	1	0	1	3	4	1
7481	201	1	26-Mar-11	1	2	17	2	3	1	0	1	3	1	2
7482	201	1	12-Oct-11	10	2	17	2	2	1	0	1	3	1	2
7483	101	1	14-Oct-11	3	2	17	2	1	1	0	1	3	1	1
7484	101	1	11-Jun-10	22	2	17	2	1	1	0	1	3	5	2
7485	201	1	20-Jul-10	22	2	17	2	2	1	0	1	3	3	1
7486	201	1	20-Jul-10	22	2	17	2	2	1	0	1	3	3	1
7487	101	1	18-Mar-09	15	2	17	2	2	1	0	1	3	1	1
7488	101	1	12-Jul-09	19	2	17	2	1	1	0	1	3	5	2
7489	101	1	8-May-08	17	2	17	2	1	1	0	1	3	5	1
7490	101	1	16-May-08	12	2	17	2	1	1	0	1	3	5	1
7491	101	1	9-Sep-07	0	2	17	2	1	1	0	1	3	3	1
7492	101	1	18-Feb-07	9	2	17	2	1	1	0	1	3	5	1
7493	201	1	21-Jan-07	19	2	17	2	3	1	0	1	3	6	1
7494	101	1	28-Sep-07	11	2	17	2	1	1	0	1	3	5	1
7495	101	1	19-Feb-07	10	2	17	2	3	1	0	1	3	1	1
7496	101	1	19-Feb-07	10	2	17	2	3	1	0	1	3	3	1
7497	101	1	29-Dec-07	11	2	17	2	1	1	0	1	3	3	1
7498	201	1	25-Mar-06	18	2	17	2	3	1	0	1	3	2	1
7499	201	1	22-Aug-06	22	2	17	2	2	1	0	1	3	1	2
7500	201	1	26-Jun-06	0	2	17	2	2	1	0	1	3	6	2
7501	201	1	22-Apr-06	6	2	17	2	1	1	0	1	3	2	2
7502	201	1	31-Jan-06	20	2	17	2	1	1	0	1	3	2	2
7503	101	1	29-Jan-06	14	2	17	2	3	1	0	1	3	4	2
7504	201	1	7-Aug-06	7	2	17	2	1	1	0	1	3	5	1
7505	101	1	27-Sep-06	16	2	17	2	1	1	0	1	3	2	1
7506	101	1	4-Jun-13	21	2	17	2	1	1	0	1	3	3	1
7507	201	1	8-Sep-11	22	2	17	2	2	1	0	1	3	3	1
7508	201	1	22-Sep-07	21	2	17	2	3	1	0	1	3	1	1
7509	201	1	17-Sep-06	18	2	17	2	1	1	0	1	3	2	1
7510	201	1	10-May-11	9	2	17	2	3	1	0	11	3	3	1
7511	101	1	27-Mar-11	20	2	17	2	1	1	0	11	3	3	1
7512	101	1	9-Jul-17	14	2	17	2	1	1	0	11	3	5	1
7513	201	1	4-Jun-17	10	2	17	2	2	1	0	11	3	4	1
7514	101	1	20-May-15	19	2	17	2	1	1	0	3	3	2	1
7515	101	1	13-Aug-15	18	2	17	2	1	1	0	3	3	2	1
7516	101	1	1-Oct-14	19	2	17	2	2	1	0	3	3	3	1
7517	101	1	27-Mar-13	17	2	17	2	1	1	0	3	3	5	4
7518	101	1	25-Jul-12	11	2	17	2	1	1	0	3	3	4	1
7519	201	1	8-Dec-12	15	2	17	2	2	1	0	3	3	1	1
7520	101	1	29-Jun-11	20	2	17	2	1	1	0	3	3	1	1
7521	101	1	19-Jul-10	16	2	17	2	1	1	0	3	3	1	1
7522	201	1	3-Feb-10	21	2	17	2	1	1	0	3	3	2	2
7523	201	1	30-Dec-08	22	2	17	2	3	1	0	3	3	3	1
7524	101	1	23-Oct-07	23	2	17	2	1	1	0	3	3	3	1
7525	201	1	19-Aug-06	23	2	17	2	3	1	0	3	3	1	1
7526	101	1	20-Jun-06	20	2	17	2	1	1	0	3	3	1	1
7527	201	1	24-Jul-17	20	2	17	2	1	1	0	3	3	3	1
7528	101	1	8-Nov-17	19	2	17	2	1	1	0	3	3	2	1
7529	201	1	27-Aug-17	22	2	17	2	2	1	0	3	3	1	1
7530	101	1	2-Dec-11	23	2	17	2	1	1	0	6	3	2	1
7531	101	1	4-Jul-10	19	2	17	2	1	1	0	6	3	3	1
7532	201	1	19-Jun-10	18	2	17	2	1	1	0	6	3	3	1
7533	202	0	5-Dec-07	2	1	9	2	1	3	1	13	2	1	1
7534	202	0	12-Jul-08	23	1	11	2	1	3	1	4	2	5	1
7535	101	1	11-Apr-08	13	1	11	2	1	3	1	4	2	1	1
7536	202	0	12-Dec-16	10	1	12	2	3	3	1	4	2	5	1
7537	202	0	18-Nov-15	22	1	12	2	1	3	1	4	2	5	1
7538	202	0	2-Sep-13	16	1	12	2	1	3	1	4	2	5	2
7539	202	0	8-Sep-11	14	1	12	2	1	3	1	4	2	1	1
7540	202	0	6-Jan-10	15	1	12	2	1	3	1	4	2	6	1
7541	202	0	4-Dec-09	11	1	12	2	3	3	1	4	2	1	1
7542	202	0	5-Jan-09	9	1	12	2	1	3	1	4	2	1	1
7543	202	0	22-Sep-06	18	1	12	2	1	3	1	4	2	1	1
7544	202	0	18-Sep-06	2	1	12	2	3	3	1	4	2	1	1
7545	202	0	7-Apr-17	16	1	12	2	1	3	1	4	2	1	1
7546	202	0	28-Dec-17	17	1	12	2	3	3	1	4	2	1	1
7547	202	0	6-Jul-10	11	1	12	2	1	3	1	11	2	3	1
7548	202	0	2-Mar-17	13	1	12	2	1	3	1	11	2	3	1
7549	202	0	31-Oct-17	11	1	12	2	1	3	1	3	2	2	1
7550	202	0	21-Mar-17	13	1	12	2	2	3	1	3	2	4	1
7551	202	0	10-Oct-14	16	2	12	2	1	3	1	4	2	4	1
7552	202	0	5-Dec-13	20	2	12	2	1	3	1	4	2	4	1
7553	202	0	5-Dec-12	20	2	12	2	1	3	1	4	2	4	1
7554	202	0	15-Mar-08	20	2	12	2	2	3	1	4	2	1	1
7555	202	0	9-Apr-07	20	2	12	2	1	3	1	4	2	1	1
7556	202	0	2-Jun-13	20	2	12	2	1	3	1	11	2	3	1
7557	101	1	31-Mar-11	11	1	12	2	1	3	1	13	2	4	1

7558	201	1	5-Nov-07	15	1	12	2	1	3	1	13	2	1	1
7559	101	1	30-Sep-07	11	1	12	2	1	3	1	13	2	1	1
7560	101	1	27-Sep-15	10	1	12	2	1	3	1	4	2	4	1
7561	101	1	21-Sep-15	11	1	12	2	1	3	1	4	2	1	2
7562	101	1	23-Sep-15	17	1	12	2	1	3	1	4	2	1	1
7563	101	1	16-Apr-14	17	1	12	2	1	3	1	4	2	1	1
7564	101	1	19-Sep-14	19	1	12	2	1	3	1	4	2	4	1
7565	101	1	15-Oct-14	13	1	12	2	1	3	1	4	2	4	1
7566	101	1	16-Mar-14	16	1	12	2	1	3	1	4	2	5	1
7567	101	1	16-May-14	15	1	12	2	1	3	1	4	2	4	1
7568	101	1	2-Dec-14	11	1	12	2	1	3	1	4	2	4	1
7569	101	1	22-Apr-14	19	1	12	2	1	3	1	4	2	2	1
7570	101	1	7-Jun-13	12	1	12	2	1	3	1	4	2	1	1
7571	101	1	9-Jan-13	11	1	12	2	1	3	1	4	2	7	4
7572	101	1	7-Jun-13	12	1	12	2	1	3	1	4	2	4	1
7573	101	1	8-Jan-13	14	1	12	2	1	3	1	4	2	1	1
7574	101	1	6-Dec-13	16	1	12	2	1	3	1	4	2	4	1
7575	101	1	13-Sep-13	16	1	12	2	1	3	1	4	2	1	1
7576	101	1	26-Mar-13	12	1	12	2	1	3	1	4	2	4	1
7577	101	1	10-Jan-13	7	1	12	2	1	3	1	4	2	4	4
7578	101	1	6-Jun-12	16	1	12	2	1	3	1	4	2	2	2
7579	201	1	4-May-12	15	1	12	2	2	3	1	4	2	7	1
7580	101	1	7-Dec-12	22	1	12	2	1	3	1	4	2	5	1
7581	101	1	31-May-12	12	1	12	2	1	3	1	4	2	1	1
7582	101	1	1-Jan-12	20	1	12	2	1	3	1	4	2	1	1
7583	101	1	29-Oct-12	19	1	12	2	1	3	1	4	2	1	1
7584	101	1	24-Sep-12	12	1	12	2	1	3	1	4	2	4	1
7585	101	1	31-Oct-11	22	1	12	2	1	3	1	4	2	2	1
7586	101	1	22-Apr-11	17	1	12	2	1	3	1	4	2	1	1
7587	101	1	30-Nov-11	11	1	12	2	1	3	1	4	2	1	1
7588	101	1	17-Jun-10	17	1	12	2	1	3	1	4	2	1	1
7589	101	1	24-Dec-10	17	1	12	2	1	3	1	4	2	1	1
7590	101	1	5-May-10	14	1	12	2	1	3	1	4	2	5	1
7591	101	1	30-Apr-10	9	1	12	2	1	3	1	4	2	7	1
7592	101	1	13-Jun-10	10	1	12	2	1	3	1	4	2	4	1
7593	101	1	11-Jun-10	11	1	12	2	1	3	1	4	2	4	1
7594	101	1	23-Nov-09	17	1	12	2	1	3	1	4	2	1	1
7595	101	1	20-Oct-09	11	1	12	2	1	3	1	4	2	1	1
7596	101	1	14-Apr-09	17	1	12	2	1	3	1	4	2	1	1
7597	101	1	19-Jul-07	22	1	12	2	1	3	1	4	2	5	1
7598	101	1	4-Sep-06	16	1	12	2	1	3	1	4	2	1	1
7599	101	1	3-Feb-06	20	1	12	2	1	3	1	4	2	1	1
7600	101	1	13-May-15	16	1	12	2	1	3	1	3	2	1	1
7601	101	1	4-Dec-12	17	1	12	2	1	3	1	3	2	1	1
7602	101	1	25-Aug-13	17	1	12	2	1	3	1	6	2	1	1
7603	201	1	19-Jun-09	16	1	12	2	3	3	1	13	2	1	1
7604	101	1	12-Jan-14	13	2	12	2	1	3	1	4	2	1	1
7605	101	1	28-Feb-14	8	2	12	2	1	3	1	4	2	1	1
7606	101	1	11-Sep-13	8	2	12	2	1	3	1	4	2	3	1
7607	101	1	20-Dec-12	18	2	12	2	1	3	1	4	2	1	1
7608	101	1	11-Jan-10	16	2	12	2	1	3	1	4	2	1	1
7609	101	1	25-Sep-08	16	2	12	2	1	3	1	4	2	1	1
7610	101	1	12-Jan-07	9	2	12	2	1	3	1	4	2	4	1
7611	202	0	11-Oct-13	9	1	13	2	1	3	1	6	2	1	2
7612	202	0	20-Jan-08	18	1	13	2	1	3	1	13	2	1	1
7613	202	0	5-Jul-16	11	1	13	2	1	3	1	4	2	5	1
7614	202	0	6-Jun-16	9	1	13	2	1	3	1	4	2	1	1
7615	202	0	7-Mar-16	15	1	13	2	1	3	1	4	2	1	1
7616	202	0	14-Mar-16	17	1	13	2	2	3	1	4	2	1	1
7617	202	0	3-Sep-15	18	1	13	2	1	3	1	4	2	2	2
7618	202	0	30-Sep-15	11	1	13	2	1	3	1	4	2	5	1
7619	202	0	26-May-15	22	1	13	2	1	3	1	4	2	2	1
7620	202	0	27-Jul-14	21	1	13	2	1	3	1	4	2	5	1
7621	202	0	28-Jun-14	22	1	13	2	2	3	1	4	2	1	1
7622	202	0	21-Oct-14	1	1	13	2	4	3	1	4	2	1	1
7623	202	0	4-Jan-13	16	1	13	2	1	3	1	4	2	5	1
7624	202	0	7-Dec-13	14	1	13	2	1	3	1	4	2	1	1
7625	202	0	8-Mar-13	8	1	13	2	1	3	1	4	2	4	1
7626	202	0	11-Dec-13	22	1	13	2	1	3	1	4	2	1	1
7627	202	0	25-Oct-13	0	1	13	2	1	3	1	4	2	4	1
7628	202	0	20-Nov-12	15	1	13	2	1	3	1	4	2	4	1
7629	202	0	17-Apr-10	14	1	13	2	1	3	1	4	2	6	2
7630	202	0	20-Sep-10	17	1	13	2	1	3	1	4	2	4	1
7631	202	0	22-Dec-10	17	1	13	2	2	3	1	4	2	4	1
7632	202	0	11-Dec-09	18	1	13	2	1	3	1	4	2	6	1
7633	202	0	7-Sep-09	9	1	13	2	1	3	1	4	2	1	1
7634	202	0	6-Nov-09	18	1	13	2	1	3	1	4	2	6	2
7635	202	0	22-Jul-09	18	1	13	2	1	3	1	4	2	5	1
7636	202	0	7-Nov-08	10	1	13	2	1	3	1	4	2	6	2
7637	202	0	4-Dec-08	18	1	13	2	1	3	1	4	2	4	1
7638	202	0	28-Nov-07	16	1	13	2	1	3	1	4	2	1	1
7639	202	0	18-Dec-07	10	1	13	2	3	3	1	4	2	1	1
7640	202	0	25-Apr-07	12	1	13	2	1	3	1	4	2	1	1
7641	202	0	27-Apr-07	18	1	13	2	1	3	1	4	2	4	1
7642	202	0	16-Oct-07	12	1	13	2	1	3	1	4	2	1	1
7643	202	0	9-Oct-07	18	1	13	2	1	3	1	4	2	1	1
7644	202	0	2-Nov-06	19	1	13	2	1	3	1	4	2	1	1
7645	202	0	19-Dec-06	8	1	13	2	1	3	1	4	2	1	1
7646	202	0	27-Oct-06	17	1	13	2	1	3	1	4	2	1	1
7647	202	0	18-Apr-06	16	1	13	2	1	3	1	4	2	1	1
7648	202	0	11-Apr-17	13	1	13	2	1	3	1	4	2	1	1
7649	202	0	20-Feb-17	20	1	13	2	3	3	1	4	2	1	1
7650	202	0	25-Dec-17	18	1	13	2	3	3	1	4	2	4	1
7651	202	0	12-Oct-12	17	1	13	2	1	3	1	11	2	2	1
7652	202	0	17-Apr-11	13	1	13	2	2	3	1	11	2	3	1
7653	202	0	24-Jun-17	23	1	13	2	1	3	1	11	2	3	1
7654	202	0	28-Dec-12	13	1	13	2	1	3	1	3	2	1	1
7655	202	0	25-Apr-08	18	1	13	2	1	3	1	3	2	1	1
7656	202	0	13-Dec-07	19	1	13	2	1	3	1	3	2	4	1
7657	202	0	20-Nov-06	5	2	13	2	1	3	1	13	2	4	1
7658	202	0	29-Sep-15	12	2	13	2	1	3	1	4	2	1	1
7659	202	0	23-Mar-17	17	2	13	2	2	3	1	4	2	1	1
7660	202	0	2-Nov-17	0	2	13	2	2	3	1	4	2	1	1
7661	202	0	6-Jul-10	12	2	13	2	3	3	1	11	2	1	1
7662	101	1	18-Sep-12	12	1	13	2	1	3	1	13	2	2	1
7663	201	1	18-Dec-09	17	1	13	2	2	3	1	13	2	1	1
7664	201	1	14-Nov-08	15	1	13	2	3	3	1	13	2	1	1
7665	201	1	13-Apr-07	13	1	13	2	2	3	1	13	2	6	1
7666	201	1	11-May-07	16	1	13	2	1	3	1	13	2	5	1
7667	201	1	21-Feb-07	16	1	13	2	1	3	1	13	2	1	1
7668	101	1	28-Apr-06	10	1	13	2	1	3	1	13	2	1	1
7669	101	1	23-Feb-16	1	1	13	2	1	3	1	4	2	1	1
7670	101	1	21-Sep-15	18	1	13	2	1	3	1	4	2	5	1
7671	101	1	1-Apr-15	20	1	13	2	3	3	1	4	2	1	1
7672	201	1	4-Dec-15	17	1	13	2	2	3	1	4	2	4	1
7673	101	1	11-Dec-15	22	1	13	2	1	3	1	4	2	5	1
7674	101	1	14-Jan-15	15	1	13	2	1	3	1	4	2	4	1
7675	101	1	20-Jan-15	20	1	13	2	1	3	1	4	2	1	2
7676	101	1	13-Oct-14	16	1	13	2	1	3	1	4	2	5	1
7677	101	1	14-Oct-14	15	1	13	2	1	3	1	4	2	1	1
7678	101	1	27-Apr-14	17	1	13	2	1	3	1	4	2	1	1
7679	101	1	21-Apr-14	19	1	13	2	1	3	1	4	2	1	1
7680	101	1	19-Mar-14	20	1	13	2	1	3	1	4	2	1	1
7681	101	1	13-Feb-14	20	1	13	2	1	3	1	4	2	1	1

7682	101	1	3-Dec-14	12	1	13	2	1	3	1	4	2	1	1
7683	101	1	25-Aug-14	23	1	13	2	1	3	1	4	2	1	1
7684	101	1	5-Jun-13	12	1	13	2	1	3	1	4	2	1	1
7685	101	1	3-Jun-13	15	1	13	2	1	3	1	4	2	4	1
7686	101	1	19-Dec-13	17	1	13	2	1	3	1	4	2	1	1
7687	101	1	22-Oct-13	17	1	13	2	1	3	1	4	2	2	1
7688	201	1	10-Jan-13	12	1	13	2	3	3	1	4	2	5	1
7689	101	1	9-Jan-13	11	1	13	2	1	3	1	4	2	1	2
7690	101	1	14-Mar-13	21	1	13	2	1	3	1	4	2	1	1
7691	101	1	12-Sep-13	13	1	13	2	1	3	1	4	2	4	1
7692	101	1	13-Nov-12	11	1	13	2	1	3	1	4	2	1	1
7693	101	1	20-Mar-12	16	1	13	2	1	3	1	4	2	1	1
7694	201	1	9-Jul-12	4	1	13	2	2	3	1	4	2	5	1
7695	201	1	21-Nov-12	14	1	13	2	2	3	1	4	2	1	1
7696	101	1	16-May-12	17	1	13	2	1	3	1	4	2	5	1
7697	101	1	6-Jun-12	12	1	13	2	1	3	1	4	2	1	1
7698	101	1	16-Mar-12	13	1	13	2	1	3	1	4	2	1	1
7699	101	1	6-Sep-12	19	1	13	2	1	3	1	4	2	5	2
7700	101	1	14-Jun-12	8	1	13	2	1	3	1	4	2	4	1
7701	101	1	16-Apr-12	13	1	13	2	1	3	1	4	2	4	1
7702	201	1	6-Sep-12	12	1	13	2	2	3	1	4	2	4	1
7703	101	1	4-Nov-11	17	1	13	2	1	3	1	4	2	4	1
7704	101	1	10-Sep-11	16	1	13	2	1	3	1	4	2	5	1
7705	101	1	20-Oct-11	8	1	13	2	1	3	1	4	2	7	1
7706	101	1	22-Nov-11	10	1	13	2	1	3	1	4	2	5	1
7707	101	1	30-Jun-11	17	1	13	2	1	3	1	4	2	1	1
7708	101	1	13-Oct-11	10	1	13	2	1	3	1	4	2	1	2
7709	101	1	11-Sep-11	20	1	13	2	1	3	1	4	2	1	1
7710	101	1	5-Aug-11	23	1	13	2	1	3	1	4	2	7	2
7711	101	1	15-Jan-10	17	1	13	2	1	3	1	4	2	1	1
7712	101	1	19-Apr-10	18	1	13	2	1	3	1	4	2	5	1
7713	101	1	17-Nov-10	8	1	13	2	1	3	1	4	2	5	1
7714	101	1	1-Jun-10	18	1	13	2	1	3	1	4	2	5	4
7715	101	1	9-Jun-09	10	1	13	2	1	3	1	4	2	5	1
7716	101	1	9-Sep-09	11	1	13	2	1	3	1	4	2	1	1
7717	101	1	1-Sep-09	17	1	13	2	1	3	1	4	2	4	1
7718	101	1	18-Sep-09	17	1	13	2	1	3	1	4	2	4	1
7719	101	1	25-Dec-09	10	1	13	2	1	3	1	4	2	4	1
7720	101	1	26-Nov-09	18	1	13	2	1	3	1	4	2	1	1
7721	101	1	29-Oct-09	17	1	13	2	1	3	1	4	2	1	1
7722	101	1	10-Oct-08	17	1	13	2	1	3	1	4	2	4	1
7723	101	1	10-Oct-08	17	1	13	2	1	3	1	4	2	1	1
7724	101	1	26-Jun-08	10	1	13	2	1	3	1	4	2	1	1
7725	101	1	4-Dec-08	10	1	13	2	1	3	1	4	2	5	1
7726	101	1	16-Oct-08	10	1	13	2	1	3	1	4	2	1	1
7727	101	1	15-Feb-08	16	1	13	2	1	3	1	4	2	1	1
7728	201	1	4-Dec-08	1	1	13	2	2	3	1	4	2	2	2
7729	101	1	30-Sep-08	15	1	13	2	1	3	1	4	2	1	1
7730	101	1	13-Nov-07	18	1	13	2	1	3	1	4	2	5	1
7731	101	1	24-Apr-07	11	1	13	2	1	3	1	4	2	1	1
7732	201	1	2-Apr-07	19	1	13	2	1	3	1	4	2	4	1
7733	101	1	18-Jun-07	22	1	13	2	1	3	1	4	2	6	1
7734	101	1	18-Jun-07	22	1	13	2	1	3	1	4	2	6	1
7735	101	1	8-Aug-07	14	1	13	2	3	3	1	4	2	5	1
7736	101	1	16-Oct-07	17	1	13	2	1	3	1	4	2	3	1
7737	101	1	17-May-06	11	1	13	2	1	3	1	4	2	1	1
7738	101	1	27-Dec-06	15	1	13	2	1	3	1	4	2	4	1
7739	101	1	21-Sep-06	15	1	13	2	1	3	1	4	2	2	1
7740	201	1	17-Dec-17	17	1	13	2	3	3	1	4	2	5	1
7741	101	1	26-May-17	16	1	13	2	1	3	1	4	2	4	1
7742	201	1	24-Nov-17	14	1	13	2	1	3	1	4	2	4	1
7743	101	1	6-Jan-17	17	1	13	2	1	3	1	4	2	1	1
7744	101	1	20-Apr-17	19	1	13	2	1	3	1	4	2	1	1
7745	101	1	5-May-17	17	1	13	2	1	3	1	4	2	1	2
7746	101	1	25-May-14	17	1	13	2	1	3	1	11	2	2	1
7747	101	1	25-May-14	18	1	13	2	1	3	1	11	2	2	1
7748	101	1	16-Mar-17	16	1	13	2	1	3	1	11	2	2	1
7749	101	1	11-Oct-10	17	1	13	2	1	3	1	3	2	1	1
7750	101	1	28-Apr-10	17	1	13	2	1	3	1	3	2	1	1
7751	201	1	30-Dec-15	16	1	13	2	2	3	1	6	2	1	1
7752	101	1	16-Oct-12	8	1	13	2	1	3	1	6	2	2	1
7753	101	1	12-Mar-08	19	1	13	2	1	3	1	6	2	1	1
7754	101	1	12-Mar-08	19	1	13	2	1	3	1	6	2	2	1
7755	101	1	1-Apr-08	8	1	13	2	1	3	1	6	2	4	1
7756	101	1	26-Jun-07	10	1	13	2	1	3	1	6	2	4	1
7757	101	1	30-Mar-17	22	1	13	2	1	3	1	13	2	6	1
7758	101	1	15-Jun-15	11	2	13	2	1	3	1	4	2	1	2
7759	101	1	21-Mar-13	17	2	13	2	1	3	1	4	2	5	1
7760	101	1	13-Apr-12	16	2	13	2	1	3	1	4	2	4	1
7761	101	1	5-Dec-11	18	2	13	2	1	3	1	4	2	1	1
7762	101	1	16-Dec-10	12	2	13	2	1	3	1	4	2	1	1
7763	101	1	20-Jan-10	16	2	13	2	1	3	1	4	2	1	1
7764	101	1	18-May-10	11	2	13	2	1	3	1	4	2	1	1
7765	101	1	15-Sep-10	16	2	13	2	1	3	1	4	2	3	1
7766	101	1	15-Sep-10	16	2	13	2	1	3	1	4	2	4	1
7767	101	1	15-Sep-10	16	2	13	2	1	3	1	4	2	1	1
7768	101	1	15-Sep-10	16	2	13	2	1	3	1	4	2	4	1
7769	101	1	9-Dec-10	8	2	13	2	1	3	1	4	2	1	1
7770	101	1	22-May-07	17	2	13	2	1	3	1	4	2	1	1
7771	101	1	30-Aug-07	22	2	13	2	1	3	1	4	2	5	1
7772	101	1	6-Apr-06	15	2	13	2	1	3	1	4	2	1	1
7773	101	1	28-Nov-11	8	2	13	2	1	3	1	3	2	4	4
7774	202	0	31-Jan-14	3	1	14	2	1	3	1	6	2	1	1
7775	202	0	13-Apr-10	10	1	14	2	1	3	1	13	2	5	1
7776	202	0	8-Jan-10	18	1	14	2	1	3	1	13	2	1	2
7777	202	0	5-May-10	9	1	14	2	1	3	1	13	2	1	1
7778	202	0	3-Jul-09	16	1	14	2	2	3	1	13	2	6	1
7779	202	0	14-Dec-09	18	1	14	2	1	3	1	13	2	2	2
7780	202	0	20-May-09	0	1	14	2	1	3	1	13	2	1	1
7781	202	0	5-Mar-08	20	1	14	2	1	3	1	13	2	1	1
7782	202	0	8-Jul-08	17	1	14	2	1	3	1	13	2	6	2
7783	202	0	12-Nov-07	18	1	14	2	1	3	1	13	2	1	1
7784	202	0	7-May-13	14	1	14	2	1	3	1	9	2	4	1
7785	202	0	7-Mar-16	12	1	14	2	2	3	1	4	2	5	1
7786	202	0	5-Jul-16	10	1	14	2	1	3	1	4	2	5	1
7787	202	0	2-Jul-15	13	1	14	2	1	3	1	4	2	1	1
7788	202	0	28-May-15	12	1	14	2	1	3	1	4	2	1	1
7789	202	0	13-May-15	19	1	14	2	1	3	1	4	2	3	1
7790	202	0	11-Dec-15	12	1	14	2	3	3	1	4	2	1	1
7791	202	0	22-Oct-15	9	1	14	2	1	3	1	4	2	4	1
7792	202	0	25-Dec-14	17	1	14	2	1	3	1	4	2	1	1
7793	202	0	19-May-14	15	1	14	2	1	3	1	4	2	1	1
7794	202	0	23-Dec-14	19	1	14	2	1	3	1	4	2	1	1
7795	202	0	26-Dec-14	15	1	14	2	1	3	1	4	2	4	1
7796	202	0	29-May-14	16	1	14	2	3	3	1	4	2	4	1
7797	202	0	30-Oct-14	20	1	14	2	1	3	1	4	2	5	2
7798	202	0	13-Jun-14	15	1	14	2	1	3	1	4	2	1	1
7799	202	0	21-Nov-13	17	1	14	2	1	3	1	4	2	5	1
7800	202	0	3-Dec-13	9	1	14	2	1	3	1	4	2	5	1
7801	202	0	29-Sep-13	21	1	14	2	1	3	1	4	2	4	1
7802	202	0	16-May-13	16	1	14	2	2	3	1	4	2	1	1
7803	202	0	17-Apr-13	17	1	14	2	1	3	1	4	2	1	2
7804	202	0	10-Jan-13	9	1	14	2	1	3	1	4	2	1	1
7805	202	0	29-Oct-13	0	1	14	2	1	3	1	4	2	1	2

7806	202	0	18-Mar-13	17	1	14	2	1	3	1	4	2	2	1
7807	202	0	9-Oct-12	8	1	14	2	1	3	1	4	2	5	2
7808	202	0	22-Nov-12	22	1	14	2	1	3	1	4	2	1	1
7809	202	0	22-Nov-12	12	1	14	2	1	3	1	4	2	4	1
7810	202	0	29-Mar-12	10	1	14	2	1	3	1	4	2	1	1
7811	202	0	31-Mar-12	18	1	14	2	1	3	1	4	2	4	2
7812	202	0	7-Dec-12	13	1	14	2	3	3	1	4	2	4	1
7813	202	0	18-Dec-12	16	1	14	2	1	3	1	4	2	4	1
7814	202	0	23-Dec-11	9	1	14	2	1	3	1	4	2	1	2
7815	202	0	23-Dec-11	10	1	14	2	1	3	1	4	2	1	2
7816	202	0	25-Jun-11	16	1	14	2	1	3	1	4	2	1	1
7817	202	0	12-Apr-10	14	1	14	2	1	3	1	4	2	6	1
7818	202	0	30-Nov-10	17	1	14	2	1	3	1	4	2	6	1
7819	202	0	6-Jul-10	16	1	14	2	1	3	1	4	2	5	1
7820	202	0	5-Nov-10	17	1	14	2	1	3	1	4	2	1	1
7821	202	0	28-Jun-10	18	1	14	2	1	3	1	4	2	6	1
7822	202	0	7-May-10	17	1	14	2	1	3	1	4	2	6	2
7823	202	0	28-Jun-10	19	1	14	2	1	3	1	4	2	6	2
7824	202	0	22-Apr-09	18	1	14	2	1	3	1	4	2	6	1
7825	202	0	11-Dec-09	18	1	14	2	1	3	1	4	2	6	1
7826	202	0	24-Jun-09	20	1	14	2	3	3	1	4	2	5	1
7827	202	0	27-May-09	10	1	14	2	1	3	1	4	2	6	1
7828	202	0	27-Mar-09	17	1	14	2	1	3	1	4	2	2	2
7829	202	0	15-Oct-09	11	1	14	2	1	3	1	4	2	6	1
7830	202	0	17-Jun-09	12	1	14	2	1	3	1	4	2	1	1
7831	202	0	9-May-09	21	1	14	2	1	3	1	4	2	1	2
7832	202	0	4-Mar-08	21	1	14	2	1	3	1	4	2	4	2
7833	202	0	12-Nov-08	15	1	14	2	1	3	1	4	2	6	2
7834	202	0	11-Sep-08	13	1	14	2	1	3	1	4	2	1	1
7835	202	0	11-Apr-08	15	1	14	2	1	3	1	4	2	4	1
7836	202	0	5-Mar-08	16	1	14	2	1	3	1	4	2	1	1
7837	202	0	12-Jan-08	12	1	14	2	1	3	1	4	2	3	1
7838	202	0	30-Jun-08	17	1	14	2	1	3	1	4	2	1	2
7839	202	0	15-Jan-08	11	1	14	2	2	3	1	4	2	1	1
7840	202	0	2-Jun-08	12	1	14	2	1	3	1	4	2	4	1
7841	202	0	7-Aug-08	20	1	14	2	1	3	1	4	2	6	1
7842	202	0	22-Nov-08	22	1	14	2	2	3	1	4	2	1	2
7843	202	0	21-Jan-08	13	1	14	2	2	3	1	4	2	4	1
7844	202	0	7-Aug-08	20	1	14	2	1	3	1	4	2	4	1
7845	202	0	8-Mar-07	12	1	14	2	1	3	1	4	2	6	1
7846	202	0	27-Nov-07	16	1	14	2	1	3	1	4	2	4	1
7847	202	0	18-Apr-07	14	1	14	2	1	3	1	4	2	4	1
7848	202	0	7-Jul-07	12	1	14	2	1	3	1	4	2	4	1
7849	202	0	12-Nov-07	21	1	14	2	1	3	1	4	2	6	1
7850	202	0	7-Nov-07	17	1	14	2	1	3	1	4	2	4	1
7851	202	0	8-Oct-07	15	1	14	2	1	3	1	4	2	1	2
7852	202	0	27-Sep-07	16	1	14	2	1	3	1	4	2	1	1
7853	202	0	19-Apr-07	20	1	14	2	1	3	1	4	2	1	1
7854	202	0	23-Jun-07	16	1	14	2	1	3	1	4	2	2	1
7855	202	0	8-Oct-07	11	1	14	2	1	3	1	4	2	1	1
7856	202	0	29-Jun-07	10	1	14	2	1	3	1	4	2	2	1
7857	202	0	24-Oct-07	16	1	14	2	1	3	1	4	2	1	2
7858	202	0	29-Apr-07	17	1	14	2	1	3	1	4	2	4	1
7859	202	0	26-Apr-06	15	1	14	2	1	3	1	4	2	1	1
7860	202	0	12-Jan-06	19	1	14	2	1	3	1	4	2	6	1
7861	202	0	17-Dec-06	20	1	14	2	1	3	1	4	2	1	1
7862	202	0	27-Dec-06	10	1	14	2	1	3	1	4	2	1	1
7863	202	0	30-Sep-06	7	1	14	2	1	3	1	4	2	2	1
7864	202	0	11-May-06	17	1	14	2	1	3	1	4	2	1	1
7865	202	0	7-Apr-17	16	1	14	2	1	3	1	4	2	5	1
7866	202	0	15-Jun-17	23	1	14	2	1	3	1	4	2	2	1
7867	202	0	2-May-17	0	1	14	2	1	3	1	4	2	1	1
7868	202	0	16-Apr-17	16	1	14	2	1	3	1	4	2	1	1
7869	202	0	27-Apr-17	16	1	14	2	1	3	1	4	2	1	1
7870	202	0	17-Mar-17	15	1	14	2	2	3	1	4	2	1	1
7871	202	0	24-May-17	12	1	14	2	2	3	1	4	2	1	1
7872	202	0	28-Mar-17	13	1	14	2	2	3	1	4	2	1	2
7873	202	0	17-Aug-17	4	1	14	2	2	3	1	4	2	1	1
7874	202	0	28-Aug-17	0	1	14	2	2	3	1	11	2	3	1
7875	202	0	17-Aug-12	20	1	14	2	1	3	1	3	2	4	1
7876	202	0	17-Sep-12	18	1	14	2	3	3	1	3	2	1	1
7877	202	0	8-Nov-11	13	1	14	2	1	3	1	3	2	1	1
7878	202	0	26-May-10	17	1	14	2	1	3	1	3	2	6	1
7879	202	0	19-Mar-09	20	1	14	2	1	3	1	3	2	6	2
7880	202	0	13-Sep-06	15	1	14	2	1	3	1	3	2	1	1
7881	202	0	27-Nov-06	20	1	14	2	1	3	1	3	2	1	1
7882	202	0	20-Dec-06	21	1	14	2	1	3	1	3	2	1	1
7883	202	0	9-Feb-16	15	1	14	2	1	3	1	6	2	1	1
7884	202	0	31-Aug-15	19	1	14	2	2	3	1	6	2	4	2
7885	202	0	31-Aug-15	19	1	14	2	2	3	1	6	2	2	1
7886	202	0	31-Aug-15	19	1	14	2	2	3	1	6	2	2	1
7887	202	0	3-Jul-13	9	1	14	2	2	3	1	6	2	1	1
7888	202	0	25-Sep-09	19	1	14	2	1	3	1	6	2	1	1
7889	202	0	11-Apr-12	0	1	14	2	1	3	1	13	2	1	2
7890	202	0	20-Apr-07	10	1	14	2	1	3	1	13	2	1	2
7891	202	0	26-Nov-11	10	2	14	2	1	3	1	13	2	5	1
7892	202	0	19-Oct-07	18	2	14	2	1	3	1	13	2	2	1
7893	202	0	5-Nov-08	18	2	14	2	1	3	1	1	2	4	1
7894	202	0	9-Sep-16	14	2	14	2	1	3	1	4	2	1	1
7895	202	0	1-Dec-16	20	2	14	2	1	3	1	4	2	1	1
7896	202	0	5-Nov-15	17	2	14	2	1	3	1	4	2	2	1
7897	202	0	30-Nov-13	17	2	14	2	1	3	1	4	2	5	2
7898	202	0	23-Dec-13	17	2	14	2	2	3	1	4	2	5	1
7899	202	0	31-Jan-13	22	2	14	2	3	3	1	4	2	1	2
7900	202	0	9-Jul-13	18	2	14	2	1	3	1	4	2	4	1
7901	202	0	11-Mar-11	17	2	14	2	1	3	1	4	2	4	1
7902	202	0	2-Apr-08	13	2	14	2	1	3	1	4	2	1	1
7903	202	0	25-Dec-08	17	2	14	2	1	3	1	4	2	1	1
7904	202	0	23-Oct-08	18	2	14	2	1	3	1	4	2	4	1
7905	202	0	22-May-07	15	2	14	2	1	3	1	4	2	1	1
7906	202	0	21-Dec-07	10	2	14	2	1	3	1	4	2	6	1
7907	202	0	7-Jun-10	20	2	14	2	1	3	1	11	2	2	1
7908	202	0	11-Dec-07	17	2	14	2	1	3	1	11	2	3	1
7909	101	1	24-May-13	19	1	14	2	2	3	1	1	2	5	1
7910	201	1	18-Apr-14	8	1	14	2	2	3	1	6	2	4	1
7911	201	1	13-Oct-12	14	1	14	2	3	3	1	13	2	5	1
7912	201	1	18-Dec-09	18	1	14	2	2	3	1	13	2	6	1
7913	201	1	13-Apr-07	13	1	14	2	2	3	1	13	2	6	1
7914	101	1	15-Sep-07	23	1	14	2	1	3	1	13	2	5	1
7915	101	1	3-Oct-16	17	1	14	2	1	3	1	4	2	1	1
7916	101	1	27-Mar-15	14	1	14	2	1	3	1	4	2	2	1
7917	101	1	8-May-15	18	1	14	2	1	3	1	4	2	4	1
7918	101	1	7-May-15	11	1	14	2	1	3	1	4	2	2	1
7919	101	1	17-Apr-15	18	1	14	2	1	3	1	4	2	1	1
7920	101	1	7-Jul-15	16	1	14	2	1	3	1	4	2	5	4
7921	101	1	22-Jul-15	20	1	14	2	1	3	1	4	2	7	1
7922	101	1	28-Jan-15	23	1	14	2	1	3	1	4	2	5	1
7923	101	1	9-Feb-15	16	1	14	2	1	3	1	4	2	5	1
7924	101	1	7-May-15	17	1	14	2	1	3	1	4	2	5	1
7925	101	1	8-Oct-15	20	1	14	2	1	3	1	4	2	1	1
7926	101	1	1-Apr-14	10	1	14	2	1	3	1	4	2	5	1
7927	101	1	18-Jun-14	9	1	14	2	1	3	1	4	2	4	4
7928	101	1	27-Mar-14	18	1	14	2	1	3	1	4	2	5	1
7929	101	1	30-Sep-14	15	1	14	2	1	3	1	4	2	4	1

7930	101	1	23-Jul-14	22	1	14	2	1	3	1	4	2	4	1
7931	101	1	4-Jun-14	17	1	14	2	1	3	1	4	2	2	1
7932	101	1	9-Dec-14	17	1	14	2	1	3	1	4	2	4	1
7933	101	1	3-Jul-14	20	1	14	2	1	3	1	4	2	1	1
7934	101	1	29-Jan-14	21	1	14	2	1	3	1	4	2	1	1
7935	101	1	14-Aug-14	15	1	14	2	1	3	1	4	2	5	1
7936	101	1	18-Dec-14	19	1	14	2	1	3	1	4	2	5	1
7937	101	1	13-Jun-14	16	1	14	2	1	3	1	4	2	4	1
7938	101	1	28-Oct-14	10	1	14	2	1	3	1	4	2	2	1
7939	101	1	31-Oct-13	15	1	14	2	1	3	1	4	2	5	1
7940	101	1	21-Nov-13	16	1	14	2	1	3	1	4	2	5	1
7941	101	1	25-Oct-13	16	1	14	2	1	3	1	4	2	4	1
7942	201	1	7-Jan-13	19	1	14	2	3	3	1	4	2	1	1
7943	101	1	24-May-13	14	1	14	2	1	3	1	4	2	1	1
7944	101	1	26-Jun-13	16	1	14	2	1	3	1	4	2	5	4
7945	101	1	19-Mar-13	8	1	14	2	1	3	1	4	2	5	1
7946	101	1	20-May-13	10	1	14	2	1	3	1	4	2	5	1
7947	101	1	17-May-13	14	1	14	2	1	3	1	4	2	1	1
7948	101	1	14-Nov-13	12	1	14	2	1	3	1	4	2	1	1
7949	101	1	14-Mar-13	19	1	14	2	1	3	1	4	2	4	1
7950	101	1	19-Jan-13	12	1	14	2	1	3	1	4	2	7	1
7951	101	1	19-Nov-13	16	1	14	2	1	3	1	4	2	1	1
7952	101	1	1-Mar-12	17	1	14	2	1	3	1	4	2	5	1
7953	101	1	13-Jun-12	17	1	14	2	1	3	1	4	2	5	1
7954	101	1	12-Nov-12	16	1	14	2	1	3	1	4	2	5	1
7955	101	1	12-Oct-12	15	1	14	2	2	3	1	4	2	1	1
7956	101	1	20-Nov-12	9	1	14	2	1	3	1	4	2	4	1
7957	101	1	3-Nov-12	16	1	14	2	2	3	1	4	2	5	2
7958	101	1	3-Nov-12	16	1	14	2	1	3	1	4	2	5	4
7959	101	1	6-Jan-12	17	1	14	2	1	3	1	4	2	1	1
7960	101	1	20-Feb-12	17	1	14	2	1	3	1	4	2	5	1
7961	101	1	21-Mar-12	14	1	14	2	1	3	1	4	2	5	1
7962	101	1	30-Mar-12	17	1	14	2	1	3	1	4	2	1	1
7963	101	1	11-Oct-12	16	1	14	2	1	3	1	4	2	3	1
7964	101	1	12-Nov-12	16	1	14	2	1	3	1	4	2	1	4
7965	101	1	19-Jun-12	19	1	14	2	1	3	1	4	2	5	1
7966	101	1	17-Mar-12	22	1	14	2	1	3	1	4	2	1	1
7967	101	1	28-Mar-12	17	1	14	2	1	3	1	4	2	1	1
7968	101	1	5-Nov-11	9	1	14	2	1	3	1	4	2	5	1
7969	101	1	26-Sep-11	17	1	14	2	1	3	1	4	2	5	1
7970	101	1	26-Sep-11	17	1	14	2	1	3	1	4	2	5	1
7971	101	1	2-Mar-11	22	1	14	2	1	3	1	4	2	1	1
7972	101	1	19-Oct-11	16	1	14	2	1	3	1	4	2	1	1
7973	101	1	29-Jun-11	17	1	14	2	1	3	1	4	2	1	2
7974	101	1	8-Apr-11	11	1	14	2	1	3	1	4	2	1	1
7975	101	1	11-Jan-11	17	1	14	2	1	3	1	4	2	1	1
7976	101	1	5-Oct-11	22	1	14	2	1	3	1	4	2	1	1
7977	101	1	8-Nov-11	16	1	14	2	1	3	1	4	2	4	1
7978	101	1	4-Nov-10	17	1	14	2	1	3	1	4	2	1	1
7979	101	1	1-Dec-10	17	1	14	2	1	3	1	4	2	2	1
7980	101	1	8-Jan-10	15	1	14	2	1	3	1	4	2	2	1
7981	101	1	2-Nov-10	19	1	14	2	1	3	1	4	2	5	1
7982	101	1	19-Oct-10	17	1	14	2	1	3	1	4	2	1	1
7983	101	1	30-Dec-10	8	1	14	2	1	3	1	4	2	5	1
7984	101	1	1-Jun-10	17	1	14	2	1	3	1	4	2	5	2
7985	101	1	1-Oct-10	10	1	14	2	1	3	1	4	2	5	1
7986	101	1	4-May-10	17	1	14	2	1	3	1	4	2	1	1
7987	101	1	4-May-10	22	1	14	2	1	3	1	4	2	1	1
7988	101	1	18-Dec-10	23	1	14	2	1	3	1	4	2	7	1
7989	101	1	22-Aug-10	17	1	14	2	3	3	1	4	2	4	1
7990	101	1	13-May-10	17	1	14	2	1	3	1	4	2	1	1
7991	101	1	30-Dec-10	16	1	14	2	1	3	1	4	2	1	1
7992	101	1	30-Aug-09	12	1	14	2	1	3	1	4	2	6	1
7993	101	1	14-Apr-09	17	1	14	2	1	3	1	4	2	1	1
7994	101	1	11-Dec-09	18	1	14	2	1	3	1	4	2	1	1
7995	201	1	27-Apr-09	15	1	14	2	2	3	1	4	2	1	1
7996	101	1	1-Dec-09	12	1	14	2	1	3	1	4	2	1	1
7997	101	1	12-Dec-09	17	1	14	2	3	3	1	4	2	1	4
7998	101	1	10-Jul-09	1	1	14	2	1	3	1	4	2	4	1
7999	101	1	4-Nov-09	17	1	14	2	1	3	1	4	2	4	1
8000	101	1	26-Nov-09	18	1	14	2	1	3	1	4	2	1	1
8001	101	1	25-Dec-09	9	1	14	2	1	3	1	4	2	1	1
8002	101	1	11-Nov-09	8	1	14	2	1	3	1	4	2	4	1
8003	101	1	28-Jun-09	11	1	14	2	1	3	1	4	2	1	1
8004	101	1	27-Jun-08	17	1	14	2	1	3	1	4	2	5	1
8005	101	1	19-Dec-08	18	1	14	2	1	3	1	4	2	5	1
8006	101	1	24-Oct-08	22	1	14	2	1	3	1	4	2	1	1
8007	201	1	29-Apr-08	16	1	14	2	2	3	1	4	2	1	1
8008	101	1	24-Oct-08	19	1	14	2	1	3	1	4	2	3	1
8009	201	1	21-Nov-08	12	1	14	2	1	3	1	4	2	3	2
8010	101	1	29-Jun-07	20	1	14	2	1	3	1	4	2	4	1
8011	101	1	2-Nov-07	17	1	14	2	1	3	1	4	2	6	1
8012	101	1	15-Mar-07	17	1	14	2	1	3	1	4	2	6	1
8013	201	1	25-Oct-07	11	1	14	2	2	3	1	4	2	5	1
8014	201	1	5-Nov-07	18	1	14	2	2	3	1	4	2	1	1
8015	101	1	5-Jul-07	17	1	14	2	1	3	1	4	2	1	4
8016	101	1	9-Jan-07	10	1	14	2	1	3	1	4	2	2	1
8017	101	1	21-Feb-07	14	1	14	2	1	3	1	4	2	1	1
8018	201	1	24-Aug-07	23	1	14	2	1	3	1	4	2	4	1
8019	101	1	22-Jan-06	10	1	14	2	1	3	1	4	2	1	1
8020	101	1	16-Jun-06	10	1	14	2	1	3	1	4	2	1	1
8021	101	1	3-Mar-06	16	1	14	2	1	3	1	4	2	1	1
8022	101	1	5-Nov-06	13	1	14	2	1	3	1	4	2	1	1
8023	101	1	28-Sep-06	15	1	14	2	1	3	1	4	2	1	1
8024	101	1	29-May-06	11	1	14	2	1	3	1	4	2	1	1
8025	101	1	21-Sep-06	18	1	14	2	1	3	1	4	2	5	2
8026	201	1	22-May-06	17	1	14	2	1	3	1	4	2	1	1
8027	101	1	16-Dec-06	15	1	14	2	1	3	1	4	2	1	1
8028	101	1	2-May-06	8	1	14	2	1	3	1	4	2	1	1
8029	101	1	11-Oct-06	9	1	14	2	1	3	1	4	2	1	1
8030	101	1	10-Nov-06	17	1	14	2	1	3	1	4	2	2	1
8031	101	1	21-Apr-06	18	1	14	2	1	3	1	4	2	1	1
8032	101	1	16-May-06	21	1	14	2	1	3	1	4	2	1	1
8033	101	1	16-Mar-17	16	1	14	2	1	3	1	4	2	5	1
8034	101	1	19-May-17	19	1	14	2	1	3	1	4	2	1	1
8035	101	1	21-Dec-12	16	1	14	2	1	3	1	11	2	2	1
8036	101	1	14-Aug-11	19	1	14	2	1	3	1	11	2	3	1
8037	101	1	16-Mar-11	10	1	14	2	1	3	1	3	2	3	4
8038	101	1	23-Apr-10	16	1	14	2	1	3	1	3	2	1	2
8039	101	1	26-Sep-14	8	1	14	2	1	3	1	6	2	2	1
8040	101	1	6-Jun-13	7	1	14	2	3	3	1	6	2	4	2
8041	101	1	25-Apr-13	18	1	14	2	1	3	1	6	2	4	2
8042	201	1	24-May-13	23	1	14	2	3	3	1	6	2	2	2
8043	101	1	18-Nov-12	3	1	14	2	2	3	1	6	2	2	2
8044	101	1	22-Nov-11	20	1	14	2	1	3	1	6	2	4	2
8045	101	1	28-Jun-10	16	1	14	2	1	3	1	6	2	1	1
8046	101	1	20-Oct-09	10	1	14	2	1	3	1	6	2	1	2
8047	101	1	6-Apr-06	7	1	14	2	1	3	1	6	2	7	1
8048	201	1	30-Sep-06	23	1	14	2	1	3	1	6	2	2	1
8049	101	1	8-Apr-11	12	1	14	2	1	3	1	13	2	1	1
8050	201	1	28-Dec-06	9	2	14	2	1	3	1	13	2	1	1
8051	101	1	8-Apr-15	17	2	14	2	1	3	1	4	2	4	1
8052	101	1	2-Sep-14	18	2	14	2	1	3	1	4	2	5	1
8053	101	1	15-Jun-14	15	2	14	2	1	3	1	4	2	4	1

8054	101	1	8-Jun-12	12	2	14	2	1	3	1	4	2	1	1
8055	101	1	6-May-12	2	2	14	2	1	3	1	4	2	5	1
8056	101	1	1-Mar-10	14	2	14	2	1	3	1	4	2	5	1
8057	101	1	15-Sep-10	16	2	14	2	1	3	1	4	2	4	1
8058	101	1	13-Nov-08	16	2	14	2	1	3	1	4	2	1	1
8059	201	1	26-Oct-07	18	2	14	2	1	3	1	4	2	4	1
8060	101	1	24-Oct-07	11	2	14	2	1	3	1	4	2	6	1
8061	101	1	19-Sep-06	16	2	14	2	1	3	1	4	2	1	1
8062	101	1	18-Dec-06	18	2	14	2	1	3	1	4	2	4	1
8063	202	0	30-Sep-15	18	1	15	2	1	3	1	4	2	1	1
8064	202	0	29-Nov-13	22	1	15	2	1	3	1	4	2	1	1
8065	202	0	12-Oct-13	22	1	15	2	1	3	1	4	2	5	2
8066	202	0	13-Sep-13	10	1	15	2	1	3	1	4	2	4	1
8067	202	0	15-Dec-14	20	1	15	2	1	3	1	4	2	4	2
8068	202	0	25-Sep-12	13	1	15	2	1	3	1	6	2	4	1
8069	202	0	27-May-12	0	1	15	2	1	3	1	6	2	1	2
8070	202	0	18-Sep-13	0	1	15	2	1	3	1	6	2	1	2
8071	202	0	19-Jun-15	9	1	15	2	1	3	1	10	2	1	1
8072	202	0	20-Jul-14	11	1	15	2	1	3	1	10	2	5	1
8073	202	0	10-Jul-10	11	1	15	2	1	3	1	13	2	1	1
8074	202	0	1-Dec-09	20	1	15	2	1	3	1	13	2	2	1
8075	202	0	16-Mar-09	18	1	15	2	1	3	1	13	2	1	1
8076	202	0	26-Oct-09	18	1	15	2	1	3	1	13	2	5	1
8077	202	0	6-May-09	11	1	15	2	1	3	1	13	2	1	1
8078	202	0	12-Nov-07	20	1	15	2	1	3	1	13	2	6	1
8079	202	0	28-Jul-07	15	1	15	2	1	3	1	13	2	1	1
8080	202	0	2-Jun-07	22	1	15	2	1	3	1	13	2	6	2
8081	202	0	3-Jun-16	8	1	15	2	3	3	1	4	2	1	1
8082	202	0	16-May-16	17	1	15	2	1	3	1	4	2	1	1
8083	202	0	22-Apr-16	15	1	15	2	1	3	1	4	2	1	1
8084	202	0	6-Nov-16	21	1	15	2	1	3	1	4	2	1	1
8085	202	0	1-Sep-16	17	1	15	2	2	3	1	4	2	1	1
8086	202	0	5-Jul-16	19	1	15	2	1	3	1	4	2	1	2
8087	202	0	8-Jun-16	23	1	15	2	3	3	1	4	2	1	1
8088	202	0	1-Oct-16	18	1	15	2	1	3	1	4	2	1	1
8089	202	0	23-Nov-15	18	1	15	2	1	3	1	4	2	5	1
8090	202	0	10-Sep-15	17	1	15	2	3	3	1	4	2	4	1
8091	202	0	14-Sep-15	0	1	15	2	2	3	1	4	2	1	1
8092	202	0	13-May-15	2	1	15	2	1	3	1	4	2	1	1
8093	202	0	15-Oct-14	21	1	15	2	3	3	1	4	2	5	1
8094	202	0	6-Jul-14	10	1	15	2	1	3	1	4	2	1	1
8095	202	0	29-May-14	18	1	15	2	1	3	1	4	2	1	1
8096	202	0	24-Nov-14	10	1	15	2	1	3	1	4	2	4	1
8097	202	0	13-Nov-14	16	1	15	2	1	3	1	4	2	2	1
8098	202	0	8-Jan-13	11	1	15	2	1	3	1	4	2	6	1
8099	202	0	28-Apr-13	16	1	15	2	1	3	1	4	2	1	1
8100	202	0	24-Apr-13	6	1	15	2	1	3	1	4	2	1	1
8101	202	0	28-Jun-13	10	1	15	2	1	3	1	4	2	1	1
8102	202	0	14-Jun-13	12	1	15	2	1	3	1	4	2	1	1
8103	202	0	25-Jun-13	19	1	15	2	1	3	1	4	2	1	1
8104	202	0	13-Jun-13	19	1	15	2	1	3	1	4	2	1	1
8105	202	0	4-Dec-13	17	1	15	2	1	3	1	4	2	4	2
8106	202	0	27-Jun-13	16	1	15	2	1	3	1	4	2	4	1
8107	202	0	7-Jul-13	19	1	15	2	1	3	1	4	2	1	1
8108	202	0	30-May-13	20	1	15	2	1	3	1	4	2	1	1
8109	202	0	30-Dec-13	8	1	15	2	1	3	1	4	2	5	2
8110	202	0	6-Mar-13	16	1	15	2	1	3	1	4	2	1	1
8111	202	0	26-Sep-12	18	1	15	2	1	3	1	4	2	4	1
8112	202	0	5-Jun-12	18	1	15	2	1	3	1	4	2	2	2
8113	202	0	31-Dec-12	22	1	15	2	2	3	1	4	2	1	1
8114	202	0	10-Jun-11	17	1	15	2	1	3	1	4	2	5	2
8115	202	0	2-Apr-11	16	1	15	2	1	3	1	4	2	1	1
8116	202	0	3-Jun-11	18	1	15	2	1	3	1	4	2	1	1
8117	202	0	12-May-11	20	1	15	2	2	3	1	4	2	4	1
8118	202	0	20-Jan-10	17	1	15	2	3	3	1	4	2	1	1
8119	202	0	15-Sep-10	14	1	15	2	1	3	1	4	2	1	1
8120	202	0	27-Sep-10	15	1	15	2	1	3	1	4	2	1	2
8121	202	0	17-Dec-10	16	1	15	2	1	3	1	4	2	1	1
8122	202	0	4-Jul-10	22	1	15	2	1	3	1	4	2	1	1
8123	202	0	20-Mar-09	16	1	15	2	1	3	1	4	2	6	1
8124	202	0	2-Apr-09	18	1	15	2	1	3	1	4	2	6	1
8125	202	0	19-Jun-09	10	1	15	2	1	3	1	4	2	1	1
8126	202	0	15-Dec-09	17	1	15	2	1	3	1	4	2	1	2
8127	202	0	10-Jul-09	21	1	15	2	3	3	1	4	2	3	1
8128	202	0	21-Dec-08	16	1	15	2	2	3	1	4	2	1	1
8129	202	0	4-Dec-08	18	1	15	2	1	3	1	4	2	1	2
8130	202	0	28-Sep-08	16	1	15	2	1	3	1	4	2	1	1
8131	202	0	23-May-08	9	1	15	2	1	3	1	4	2	1	1
8132	202	0	4-Jul-08	10	1	15	2	1	3	1	4	2	1	1
8133	202	0	9-Apr-08	16	1	15	2	1	3	1	4	2	1	1
8134	202	0	12-Nov-08	15	1	15	2	1	3	1	4	2	1	1
8135	202	0	18-Dec-08	17	1	15	2	1	3	1	4	2	1	1
8136	202	0	26-Nov-08	12	1	15	2	1	3	1	4	2	4	1
8137	202	0	23-May-08	10	1	15	2	1	3	1	4	2	7	1
8138	202	0	15-Jan-08	11	1	15	2	1	3	1	4	2	1	2
8139	202	0	17-Oct-08	17	1	15	2	1	3	1	4	2	6	1
8140	202	0	10-Jan-08	19	1	15	2	1	3	1	4	2	1	1
8141	202	0	7-Mar-08	9	1	15	2	1	3	1	4	2	1	1
8142	202	0	25-Apr-08	10	1	15	2	1	3	1	4	2	1	1
8143	202	0	23-May-08	14	1	15	2	1	3	1	4	2	1	1
8144	202	0	13-Jun-08	12	1	15	2	1	3	1	4	2	2	2
8145	202	0	2-Jun-08	23	1	15	2	1	3	1	4	2	2	1
8146	202	0	22-Jul-08	23	1	15	2	3	3	1	4	2	7	1
8147	202	0	26-Apr-08	20	1	15	2	1	3	1	4	2	1	1
8148	202	0	26-Jul-08	20	1	15	2	1	3	1	4	2	1	1
8149	202	0	13-Jun-08	12	1	15	2	1	3	1	4	2	2	1
8150	202	0	29-Apr-07	16	1	15	2	1	3	1	4	2	1	2
8151	202	0	25-Oct-07	8	1	15	2	1	3	1	4	2	1	1
8152	202	0	22-Oct-07	17	1	15	2	1	3	1	4	2	5	1
8153	202	0	10-Sep-07	18	1	15	2	1	3	1	4	2	6	2
8154	202	0	30-Apr-07	16	1	15	2	1	3	1	4	2	6	1
8155	202	0	7-Jun-07	17	1	15	2	1	3	1	4	2	1	1
8156	202	0	18-Sep-07	17	1	15	2	1	3	1	4	2	1	1
8157	202	0	21-Nov-07	22	1	15	2	1	3	1	4	2	4	1
8158	202	0	18-Dec-07	22	1	15	2	2	3	1	4	2	6	1
8159	202	0	18-Dec-07	22	1	15	2	1	3	1	4	2	6	1
8160	202	0	17-Jun-07	16	1	15	2	1	3	1	4	2	6	1
8161	202	0	10-May-07	7	1	15	2	1	3	1	4	2	1	1
8162	202	0	20-Jul-07	17	1	15	2	1	3	1	4	2	1	2
8163	202	0	3-Jul-07	20	1	15	2	1	3	1	4	2	1	2
8164	202	0	6-Mar-07	11	1	15	2	1	3	1	4	2	1	1
8165	202	0	4-Jul-07	9	1	15	2	1	3	1	4	2	1	1
8166	202	0	13-May-07	20	1	15	2	3	3	1	4	2	7	1
8167	202	0	7-Nov-07	12	1	15	2	1	3	1	4	2	1	1
8168	202	0	11-Sep-06	10	1	15	2	1	3	1	4	2	1	1
8169	202	0	4-Apr-06	18	1	15	2	1	3	1	4	2	1	1
8170	202	0	30-Sep-06	7	1	15	2	1	3	1	4	2	1	2
8171	202	0	11-Sep-06	10	1	15	2	1	3	1	4	2	1	1
8172	202	0	13-Sep-06	15	1	15	2	1	3	1	4	2	1	1
8173	202	0	9-Mar-06	22	1	15	2	1	3	1	4	2	1	1
8174	202	0	14-Nov-06	20	1	15	2	1	3	1	4	2	1	1
8175	202	0	4-May-06	2	1	15	2	1	3	1	4	2	1	1
8176	202	0	24-Sep-06	19	1	15	2	1	3	1	4	2	4	1
8177	202	0	23-Nov-06	18	1	15	2	1	3	1	4	2	1	1

8178	202	0	7-Dec-06	7	1	15	2	1	3	1	4	2	4	1
8179	202	0	31-Dec-06	13	1	15	2	1	3	1	4	2	5	1
8180	202	0	9-Jun-17	11	1	15	2	1	3	1	4	2	4	1
8181	202	0	18-May-17	17	1	15	2	1	3	1	4	2	5	1
8182	202	0	23-May-17	21	1	15	2	1	3	1	4	2	1	1
8183	202	0	2-Sep-17	19	1	15	2	3	3	1	4	2	1	1
8184	202	0	1-May-17	20	1	15	2	3	3	1	4	2	1	1
8185	202	0	12-Dec-17	11	1	15	2	3	3	1	4	2	5	1
8186	202	0	25-Sep-12	10	1	15	2	3	3	1	11	2	3	2
8187	202	0	14-Sep-11	18	1	15	2	1	3	1	11	2	2	1
8188	202	0	11-Sep-17	0	1	15	2	2	3	1	11	2	2	1
8189	202	0	8-Jul-13	22	1	15	2	1	3	1	3	2	1	1
8190	202	0	20-May-10	16	1	15	2	1	3	1	3	2	6	1
8191	202	0	12-Nov-09	14	1	15	2	1	3	1	3	2	6	1
8192	202	0	18-Sep-09	22	1	15	2	3	3	1	3	2	2	1
8193	202	0	25-Apr-07	17	1	15	2	1	3	1	3	2	1	1
8194	202	0	2-Aug-07	3	1	15	2	1	3	1	3	2	2	2
8195	202	0	30-Nov-17	15	1	15	2	2	3	1	3	2	5	1
8196	202	0	14-Feb-14	19	1	15	2	3	3	1	6	2	1	1
8197	202	0	22-Feb-13	9	1	15	2	1	3	1	6	2	1	2
8198	202	0	4-Dec-13	12	1	15	2	1	3	1	6	2	2	2
8199	202	0	11-Mar-11	1	1	15	2	2	3	1	6	2	7	2
8200	202	0	1-Oct-09	8	1	15	2	3	3	1	6	2	3	2
8201	202	0	19-Aug-09	22	1	15	2	3	3	1	6	2	1	1
8202	202	0	16-Sep-08	14	1	15	2	1	3	1	6	2	4	2
8203	202	0	9-Sep-08	7	1	15	2	2	3	1	6	2	2	2
8204	202	0	9-Sep-08	7	1	15	2	1	3	1	6	2	2	2
8205	202	0	24-Dec-07	16	1	15	2	1	3	1	6	2	4	2
8206	202	0	23-Nov-06	18	1	15	2	1	3	1	6	2	5	1
8207	202	0	25-May-09	12	1	15	2	2	3	1	13	2	1	1
8208	202	0	4-Dec-08	18	1	15	2	1	3	1	13	2	1	1
8209	202	0	4-Jan-07	17	1	15	2	1	3	1	13	2	4	2
8210	202	0	21-Sep-13	16	2	15	2	1	3	1	4	2	1	1
8211	202	0	13-Sep-08	18	2	15	2	3	3	1	10	2	7	2
8212	202	0	13-Dec-07	10	2	15	2	1	3	1	13	2	6	1
8213	202	0	27-Dec-16	19	2	15	2	1	3	1	4	2	4	1
8214	202	0	10-Jun-16	15	2	15	2	3	3	1	4	2	1	1
8215	202	0	28-Oct-15	14	2	15	2	1	3	1	4	2	4	1
8216	202	0	20-May-13	10	2	15	2	1	3	1	4	2	1	1
8217	202	0	12-Mar-10	9	2	15	2	1	3	1	4	2	1	1
8218	202	0	25-Nov-08	18	2	15	2	3	3	1	4	2	1	1
8219	202	0	10-Jan-08	17	2	15	2	1	3	1	4	2	1	1
8220	202	0	5-Sep-07	20	2	15	2	1	3	1	4	2	5	2
8221	202	0	16-Oct-07	21	2	15	2	1	3	1	4	2	5	1
8222	202	0	28-Apr-06	21	2	15	2	1	3	1	4	2	1	1
8223	202	0	9-Aug-12	11	2	15	2	1	3	1	11	2	1	1
8224	202	0	17-Sep-12	21	2	15	2	1	3	1	11	2	7	2
8225	202	0	14-Jul-17	22	2	15	2	1	3	1	11	2	2	1
8226	202	0	22-Nov-10	23	2	15	2	1	3	1	6	2	2	1
8227	201	1	10-Jul-12	19	1	15	2	3	3	1	13	2	6	1
8228	201	1	2-Jun-09	21	1	15	2	2	3	1	13	2	4	1
8229	201	1	14-Nov-08	12	1	15	2	1	3	1	13	2	1	1
8230	101	1	18-Sep-06	12	1	15	2	1	3	1	13	2	2	1
8231	201	1	23-Sep-16	19	1	15	2	3	3	1	4	2	4	1
8232	101	1	8-Oct-15	20	1	15	2	1	3	1	4	2	1	1
8233	101	1	17-Dec-15	14	1	15	2	1	3	1	4	2	4	1
8234	101	1	24-Oct-15	22	1	15	2	1	3	1	4	2	1	1
8235	101	1	27-Nov-14	16	1	15	2	1	3	1	4	2	1	1
8236	101	1	29-Apr-14	21	1	15	2	1	3	1	4	2	4	1
8237	101	1	5-May-14	15	1	15	2	1	3	1	4	2	1	1
8238	101	1	18-Jun-14	16	1	15	2	1	3	1	4	2	1	1
8239	101	1	28-Oct-14	19	1	15	2	1	3	1	4	2	5	1
8240	101	1	11-Mar-14	18	1	15	2	1	3	1	4	2	5	1
8241	101	1	30-Mar-14	20	1	15	2	1	3	1	4	2	1	1
8242	101	1	13-Jan-14	20	1	15	2	1	3	1	4	2	1	1
8243	101	1	27-Jun-14	15	1	15	2	1	3	1	4	2	2	1
8244	101	1	14-Sep-14	12	1	15	2	1	3	1	4	2	1	2
8245	101	1	6-Jun-13	16	1	15	2	1	3	1	4	2	1	4
8246	101	1	3-Dec-13	10	1	15	2	1	3	1	4	2	5	1
8247	101	1	24-May-13	15	1	15	2	1	3	1	4	2	5	1
8248	101	1	12-Apr-13	11	1	15	2	1	3	1	4	2	1	1
8249	201	1	22-Sep-13	17	1	15	2	1	3	1	4	2	1	1
8250	101	1	27-May-13	18	1	15	2	1	3	1	4	2	1	1
8251	101	1	19-Mar-13	8	1	15	2	1	3	1	4	2	4	4
8252	101	1	8-Jun-13	16	1	15	2	1	3	1	4	2	2	1
8253	101	1	1-May-13	2	1	15	2	1	3	1	4	2	1	2
8254	101	1	30-Oct-12	16	1	15	2	1	3	1	4	2	1	2
8255	101	1	22-Jun-12	17	1	15	2	1	3	1	4	2	1	4
8256	101	1	12-Nov-12	15	1	15	2	1	3	1	4	2	5	1
8257	101	1	4-May-12	18	1	15	2	1	3	1	4	2	1	1
8258	101	1	4-May-12	18	1	15	2	1	3	1	4	2	1	1
8259	101	1	19-Jan-12	10	1	15	2	1	3	1	4	2	1	1
8260	101	1	29-Nov-12	16	1	15	2	1	3	1	4	2	1	1
8261	101	1	19-Jun-12	19	1	15	2	1	3	1	4	2	1	1
8262	101	1	22-Sep-11	17	1	15	2	1	3	1	4	2	5	1
8263	101	1	7-Mar-11	18	1	15	2	2	3	1	4	2	1	1
8264	101	1	26-Sep-11	9	1	15	2	1	3	1	4	2	5	1
8265	101	1	5-Jul-11	17	1	15	2	1	3	1	4	2	5	1
8266	101	1	6-May-11	15	1	15	2	2	3	1	4	2	5	1
8267	201	1	4-Jul-11	16	1	15	2	2	3	1	4	2	1	1
8268	101	1	15-Sep-11	20	1	15	2	1	3	1	4	2	1	1
8269	101	1	22-Jun-11	12	1	15	2	1	3	1	4	2	1	1
8270	101	1	14-Oct-11	18	1	15	2	1	3	1	4	2	1	1
8271	101	1	17-Aug-11	1	1	15	2	1	3	1	4	2	1	1
8272	101	1	31-Jan-11	13	1	15	2	1	3	1	4	2	1	1
8273	101	1	8-Dec-11	18	1	15	2	1	3	1	4	2	5	1
8274	101	1	16-Dec-11	17	1	15	2	1	3	1	4	2	4	1
8275	101	1	8-Nov-11	10	1	15	2	1	3	1	4	2	5	1
8276	101	1	18-Mar-10	12	1	15	2	1	3	1	4	2	5	1
8277	101	1	18-Jan-10	15	1	15	2	1	3	1	4	2	1	1
8278	101	1	8-Jan-10	15	1	15	2	1	3	1	4	2	2	1
8279	101	1	10-Jun-10	11	1	15	2	1	3	1	4	2	1	2
8280	101	1	14-Apr-10	12	1	15	2	1	3	1	4	2	6	1
8281	101	1	11-Jul-10	21	1	15	2	1	3	1	4	2	1	2
8282	101	1	17-Nov-10	8	1	15	2	1	3	1	4	2	5	1
8283	201	1	29-Nov-10	16	1	15	2	2	3	1	4	2	4	1
8284	101	1	30-Mar-10	18	1	15	2	1	3	1	4	2	4	1
8285	101	1	23-Jul-10	17	1	15	2	1	3	1	4	2	2	1
8286	101	1	8-Jun-10	9	1	15	2	1	3	1	4	2	2	1
8287	101	1	9-Jul-10	16	1	15	2	1	3	1	4	2	4	1
8288	101	1	7-Aug-10	20	1	15	2	1	3	1	4	2	4	1
8289	101	1	27-May-10	15	1	15	2	2	3	1	4	2	1	1
8290	101	1	23-Jan-10	21	1	15	2	1	3	1	4	2	1	1
8291	101	1	9-May-10	17	1	15	2	1	3	1	4	2	1	1
8292	201	1	6-May-09	9	1	15	2	2	3	1	4	2	6	1
8293	101	1	30-Jun-09	12	1	15	2	2	3	1	4	2	6	2
8294	101	1	15-Apr-09	18	1	15	2	1	3	1	4	2	1	1
8295	101	1	30-Oct-09	11	1	15	2	1	3	1	4	2	1	1
8296	101	1	11-Nov-09	18	1	15	2	1	3	1	4	2	1	1
8297	101	1	30-Apr-09	16	1	15	2	1	3	1	4	2	1	1
8298	101	1	20-Apr-09	17	1	15	2	1	3	1	4	2	1	1
8299	101	1	26-Nov-09	18	1	15	2	1	3	1	4	2	1	1
8300	201	1	17-Oct-09	20	1	15	2	3	3	1	4	2	4	1
8301	101	1	4-Jan-08	17	1	15	2	1	3	1	4	2	5	1

8302	101	1	12-May-08	12	1	15	2	1	3	1	4	2	5	1
8303	101	1	1-Apr-08	11	1	15	2	1	3	1	4	2	4	1
8304	101	1	7-Mar-08	12	1	15	2	1	3	1	4	2	4	1
8305	101	1	16-May-08	17	1	15	2	1	3	1	4	2	4	1
8306	201	1	20-Oct-08	18	1	15	2	1	3	1	4	2	1	1
8307	101	1	7-Mar-08	12	1	15	2	1	3	1	4	2	1	1
8308	201	1	8-May-08	19	1	15	2	1	3	1	4	2	1	1
8309	101	1	27-Sep-08	18	1	15	2	1	3	1	4	2	2	4
8310	101	1	3-Dec-08	22	1	15	2	1	3	1	4	2	4	1
8311	101	1	29-Aug-08	22	1	15	2	1	3	1	4	2	6	1
8312	101	1	29-Feb-08	18	1	15	2	1	3	1	4	2	1	1
8313	201	1	29-Sep-08	17	1	15	2	2	3	1	4	2	1	1
8314	101	1	17-Aug-08	22	1	15	2	1	3	1	4	2	4	1
8315	101	1	18-Dec-07	17	1	15	2	1	3	1	4	2	5	1
8316	201	1	9-Nov-07	14	1	15	2	2	3	1	4	2	4	1
8317	101	1	10-Nov-07	20	1	15	2	3	3	1	4	2	4	1
8318	101	1	12-Oct-07	16	1	15	2	1	3	1	4	2	5	1
8319	101	1	14-Dec-07	15	1	15	2	1	3	1	4	2	3	1
8320	101	1	18-Jan-07	17	1	15	2	1	3	1	4	2	4	1
8321	101	1	18-May-07	17	1	15	2	1	3	1	4	2	5	2
8322	101	1	30-May-07	16	1	15	2	1	3	1	4	2	5	1
8323	101	1	10-Dec-07	18	1	15	2	1	3	1	4	2	1	1
8324	101	1	10-Jul-07	15	1	15	2	1	3	1	4	2	2	1
8325	101	1	8-Aug-07	2	1	15	2	1	3	1	4	2	1	1
8326	201	1	14-Dec-07	23	1	15	2	3	3	1	4	2	4	2
8327	101	1	5-Jul-07	22	1	15	2	1	3	1	4	2	1	2
8328	201	1	22-Nov-06	15	1	15	2	3	3	1	4	2	1	1
8329	201	1	21-Nov-06	8	1	15	2	1	3	1	4	2	1	1
8330	101	1	12-Jan-06	11	1	15	2	1	3	1	4	2	1	1
8331	101	1	14-Oct-06	17	1	15	2	1	3	1	4	2	6	1
8332	101	1	10-May-06	15	1	15	2	1	3	1	4	2	1	1
8333	101	1	18-Dec-06	10	1	15	2	2	3	1	4	2	1	1
8334	101	1	21-Apr-06	9	1	15	2	1	3	1	4	2	1	1
8335	101	1	3-Jan-06	12	1	15	2	2	3	1	4	2	4	1
8336	101	1	23-Feb-06	17	1	15	2	1	3	1	4	2	4	2
8337	101	1	25-Apr-06	15	1	15	2	1	3	1	4	2	5	1
8338	101	1	7-Nov-06	17	1	15	2	1	3	1	4	2	1	1
8339	201	1	28-May-06	20	1	15	2	2	3	1	4	2	4	1
8340	201	1	30-Aug-06	1	1	15	2	1	3	1	4	2	1	1
8341	101	1	4-May-12	18	1	15	2	1	3	1	3	2	1	1
8342	101	1	19-Dec-15	13	1	15	2	1	3	1	6	2	5	2
8343	101	1	27-Jun-14	16	1	15	2	1	3	1	6	2	2	1
8344	101	1	25-Mar-10	17	1	15	2	1	3	1	6	2	2	1
8345	201	1	4-Sep-10	22	1	15	2	2	3	1	6	2	2	1
8346	101	1	20-Oct-09	11	1	15	2	1	3	1	6	2	1	1
8347	201	1	4-Aug-09	4	1	15	2	1	3	1	6	2	3	2
8348	201	1	31-Dec-08	11	1	15	2	2	3	1	6	2	4	2
8349	101	1	23-Feb-08	23	1	15	2	2	3	1	6	2	2	2
8350	101	1	16-Oct-06	18	1	15	2	1	3	1	6	2	2	1
8351	101	1	24-Oct-08	19	1	15	2	1	3	1	13	2	3	1
8352	101	1	30-Mar-17	22	1	15	2	1	3	1	13	2	6	1
8353	101	1	16-Jun-14	16	2	15	2	1	3	1	4	2	1	1
8354	101	1	8-Jul-12	23	2	15	2	1	3	1	4	2	1	1
8355	101	1	15-Sep-10	16	2	15	2	1	3	1	4	2	1	1
8356	101	1	3-Jun-10	10	2	15	2	1	3	1	4	2	1	1
8357	101	1	10-Sep-10	18	2	15	2	1	3	1	4	2	1	1
8358	101	1	14-Apr-09	19	2	15	2	1	3	1	4	2	4	1
8359	101	1	19-Dec-08	18	2	15	2	1	3	1	4	2	1	1
8360	101	1	3-Oct-08	21	2	15	2	1	3	1	4	2	4	1
8361	101	1	26-Sep-07	10	2	15	2	1	3	1	4	2	1	1
8362	101	1	29-Oct-07	17	2	15	2	1	3	1	4	2	5	1
8363	101	1	28-Jul-07	8	2	15	2	1	3	1	4	2	5	1
8364	101	1	27-Oct-06	17	2	15	2	1	3	1	4	2	4	1
8365	101	1	12-Nov-06	11	2	15	2	1	3	1	4	2	1	1
8366	101	1	5-Jun-17	17	2	15	2	1	3	1	4	2	2	1
8367	101	1	22-Apr-09	10	2	15	2	1	3	1	11	2	3	1
8368	201	1	8-Oct-07	21	2	15	2	3	3	1	3	2	1	2
8369	202	0	16-Nov-12	12	1	16	2	1	3	1	6	2	1	1
8370	202	0	12-Nov-14	21	1	16	2	1	3	1	4	2	5	2
8371	202	0	28-Aug-13	19	1	16	2	1	3	1	6	2	1	1
8372	202	0	31-May-17	23	1	16	2	2	3	1	6	2	4	1
8373	202	0	13-Jul-16	10	1	16	2	3	3	1	12	2	2	1
8374	202	0	13-Mar-10	20	1	16	2	1	3	1	13	2	1	1
8375	202	0	21-Dec-09	13	1	16	2	1	3	1	13	2	6	1
8376	202	0	17-May-08	18	1	16	2	3	3	1	13	2	4	2
8377	202	0	3-Apr-06	23	1	16	2	1	3	1	13	2	2	1
8378	202	0	26-Oct-16	12	1	16	2	1	3	1	4	2	5	1
8379	202	0	31-Aug-16	22	1	16	2	3	3	1	4	2	4	2
8380	202	0	11-Jan-16	11	1	16	2	1	3	1	4	2	1	2
8381	202	0	29-May-16	0	1	16	2	1	3	1	4	2	5	1
8382	202	0	23-Sep-15	20	1	16	2	1	3	1	4	2	1	1
8383	202	0	27-Nov-15	11	1	16	2	1	3	1	4	2	5	1
8384	202	0	17-Sep-15	9	1	16	2	1	3	1	4	2	1	1
8385	202	0	10-Oct-15	16	1	16	2	1	3	1	4	2	5	1
8386	202	0	27-Nov-15	11	1	16	2	1	3	1	4	2	5	1
8387	202	0	12-Jun-15	12	1	16	2	1	3	1	4	2	1	1
8388	202	0	15-Oct-15	16	1	16	2	1	3	1	4	2	4	1
8389	202	0	9-Jul-15	21	1	16	2	3	3	1	4	2	5	1
8390	202	0	25-Jun-15	18	1	16	2	1	3	1	4	2	1	1
8391	202	0	3-Jul-14	21	1	16	2	1	3	1	4	2	1	1
8392	202	0	19-Jul-14	16	1	16	2	1	3	1	4	2	5	1
8393	202	0	15-Sep-14	17	1	16	2	2	3	1	4	2	5	1
8394	202	0	15-Sep-14	17	1	16	2	2	3	1	4	2	5	1
8395	202	0	20-Nov-14	12	1	16	2	1	3	1	4	2	1	1
8396	202	0	13-Jun-14	16	1	16	2	1	3	1	4	2	1	1
8397	202	0	22-Apr-13	18	1	16	2	2	3	1	4	2	4	2
8398	202	0	21-Mar-13	11	1	16	2	1	3	1	4	2	1	1
8399	202	0	20-May-13	15	1	16	2	1	3	1	4	2	4	1
8400	202	0	3-Feb-13	11	1	16	2	1	3	1	4	2	1	1
8401	202	0	21-Mar-13	2	1	16	2	1	3	1	4	2	1	1
8402	202	0	13-Apr-13	22	1	16	2	3	3	1	4	2	1	1
8403	202	0	15-Nov-13	2	1	16	2	2	3	1	4	2	1	2
8404	202	0	1-Oct-13	6	1	16	2	1	3	1	4	2	1	1
8405	202	0	18-Dec-13	22	1	16	2	3	3	1	4	2	1	1
8406	202	0	8-Mar-13	23	1	16	2	1	3	1	4	2	1	1
8407	202	0	12-Sep-12	9	1	16	2	1	3	1	4	2	5	2
8408	202	0	2-May-12	7	1	16	2	1	3	1	4	2	1	2
8409	202	0	20-Dec-12	12	1	16	2	1	3	1	4	2	1	1
8410	202	0	27-Oct-11	18	1	16	2	1	3	1	4	2	6	1
8411	202	0	7-Jan-11	16	1	16	2	1	3	1	4	2	1	2
8412	202	0	11-Nov-11	22	1	16	2	1	3	1	4	2	1	2
8413	202	0	3-Jun-11	17	1	16	2	1	3	1	4	2	1	1
8414	202	0	8-Sep-10	0	1	16	2	1	3	1	4	2	1	1
8415	202	0	4-Nov-10	10	1	16	2	1	3	1	4	2	1	1
8416	202	0	2-Nov-10	16	1	16	2	1	3	1	4	2	1	1
8417	202	0	4-Mar-10	14	1	16	2	2	3	1	4	2	1	2
8418	202	0	28-Jun-10	19	1	16	2	1	3	1	4	2	6	2
8419	202	0	30-Aug-10	12	1	16	2	2	3	1	4	2	1	1
8420	202	0	11-Dec-09	18	1	16	2	1	3	1	4	2	6	1
8421	202	0	18-Nov-09	12	1	16	2	1	3	1	4	2	1	1
8422	202	0	18-Nov-09	12	1	16	2	1	3	1	4	2	1	1
8423	202	0	5-Nov-09	16	1	16	2	1	3	1	4	2	6	2
8424	202	0	29-Sep-09	20	1	16	2	1	3	1	4	2	4	1
8425	202	0	20-Oct-09	13	1	16	2	1	3	1	4	2	1	1

8426	202	0	11-May-09	8	1	16	2	1	3	1	4	2	2	1
8427	202	0	10-Mar-09	18	1	16	2	1	3	1	4	2	1	1
8428	202	0	20-Mar-08	17	1	16	2	1	3	1	4	2	1	2
8429	202	0	12-Sep-08	17	1	16	2	1	3	1	4	2	1	1
8430	202	0	9-May-08	20	1	16	2	1	3	1	4	2	4	1
8431	202	0	21-Jan-08	17	1	16	2	1	3	1	4	2	6	1
8432	202	0	20-Sep-08	2	1	16	2	3	3	1	4	2	6	2
8433	202	0	17-Nov-08	15	1	16	2	1	3	1	4	2	1	2
8434	202	0	5-Jun-08	19	1	16	2	1	3	1	4	2	4	1
8435	202	0	23-Oct-08	11	1	16	2	1	3	1	4	2	2	1
8436	202	0	3-Oct-08	22	1	16	2	1	3	1	4	2	4	1
8437	202	0	19-Dec-08	18	1	16	2	1	3	1	4	2	1	1
8438	202	0	28-Feb-08	17	1	16	2	1	3	1	4	2	4	1
8439	202	0	16-Jul-07	12	1	16	2	1	3	1	4	2	4	1
8440	202	0	18-Dec-07	21	1	16	2	1	3	1	4	2	3	1
8441	202	0	18-Oct-07	13	1	16	2	1	3	1	4	2	5	1
8442	202	0	18-Sep-07	9	1	16	2	1	3	1	4	2	6	1
8443	202	0	18-Oct-07	21	1	16	2	1	3	1	4	2	7	1
8444	202	0	4-Nov-07	22	1	16	2	1	3	1	4	2	1	1
8445	202	0	14-Nov-07	23	1	16	2	1	3	1	4	2	6	1
8446	202	0	22-Feb-07	10	1	16	2	3	3	1	4	2	1	1
8447	202	0	18-Dec-07	22	1	16	2	2	3	1	4	2	6	1
8448	202	0	18-Dec-07	23	1	16	2	1	3	1	4	2	6	1
8449	202	0	15-Oct-07	9	1	16	2	1	3	1	4	2	2	1
8450	202	0	24-Jan-07	11	1	16	2	1	3	1	4	2	5	1
8451	202	0	14-Nov-07	22	1	16	2	1	3	1	4	2	4	1
8452	202	0	4-Nov-07	22	1	16	2	1	3	1	4	2	1	1
8453	202	0	3-Dec-07	12	1	16	2	1	3	1	4	2	4	1
8454	202	0	23-Apr-07	1	1	16	2	1	3	1	4	2	1	1
8455	202	0	25-Apr-06	7	1	16	2	1	3	1	4	2	2	1
8456	202	0	26-Dec-06	19	1	16	2	1	3	1	4	2	1	2
8457	202	0	10-May-06	18	1	16	2	1	3	1	4	2	1	1
8458	202	0	23-Nov-06	19	1	16	2	1	3	1	4	2	6	1
8459	202	0	24-Sep-06	19	1	16	2	1	3	1	4	2	2	1
8460	202	0	29-Jan-06	2	1	16	2	1	3	1	4	2	6	1
8461	202	0	1-Jul-17	1	1	16	2	1	3	1	4	2	1	1
8462	202	0	9-Jun-17	10	1	16	2	1	3	1	4	2	2	1
8463	202	0	9-Nov-17	0	1	16	2	1	3	1	4	2	1	1
8464	202	0	27-Jul-17	15	1	16	2	1	3	1	4	2	5	2
8465	202	0	26-Dec-17	0	1	16	2	3	3	1	4	2	7	1
8466	202	0	11-Oct-12	22	1	16	2	1	3	1	11	2	2	1
8467	202	0	26-Sep-11	22	1	16	2	1	3	1	11	2	1	1
8468	202	0	14-Sep-11	19	1	16	2	1	3	1	11	2	5	2
8469	202	0	14-Sep-11	18	1	16	2	1	3	1	11	2	2	1
8470	202	0	28-Jul-11	19	1	16	2	1	3	1	11	2	3	1
8471	202	0	7-Jun-16	21	1	16	2	1	3	1	3	2	5	1
8472	202	0	9-Mar-11	13	1	16	2	1	3	1	3	2	1	1
8473	202	0	31-Oct-17	13	1	16	2	1	3	1	3	2	5	1
8474	202	0	10-Jun-11	16	1	16	2	1	3	1	6	2	1	2
8475	202	0	24-May-10	22	1	16	2	1	3	1	6	2	2	2
8476	202	0	8-Nov-09	1	1	16	2	1	3	1	6	2	2	2
8477	202	0	2-Apr-09	12	1	16	2	1	3	1	6	2	4	1
8478	202	0	2-Apr-09	18	1	16	2	1	3	1	6	2	4	1
8479	202	0	2-Apr-09	18	1	16	2	1	3	1	6	2	4	1
8480	202	0	25-Nov-08	18	1	16	2	1	3	1	6	2	6	2
8481	202	0	16-Sep-08	20	1	16	2	1	3	1	6	2	1	1
8482	202	0	24-Dec-07	16	1	16	2	1	3	1	6	2	4	1
8483	202	0	29-Aug-06	3	1	16	2	1	3	1	6	2	4	1
8484	202	0	27-Oct-09	0	1	16	2	1	3	1	13	2	7	2
8485	202	0	27-Oct-09	1	1	16	2	1	3	1	13	2	7	2
8486	202	0	27-Oct-09	0	1	16	2	1	3	1	13	2	7	2
8487	202	0	27-Oct-09	0	1	16	2	1	3	1	13	2	7	2
8488	202	0	14-Nov-07	21	1	16	2	1	3	1	13	2	5	2
8489	202	0	14-Nov-07	20	1	16	2	1	3	1	13	2	5	1
8490	202	0	30-May-17	5	2	16	2	1	3	1	4	2	1	1
8491	202	0	18-Apr-08	19	2	16	2	1	3	1	13	2	6	1
8492	202	0	11-Jan-08	16	2	16	2	1	3	1	13	2	5	1
8493	202	0	19-Dec-07	19	2	16	2	1	3	1	13	2	6	1
8494	202	0	5-Jul-16	22	2	16	2	1	3	1	4	2	1	1
8495	202	0	3-Sep-15	18	2	16	2	1	3	1	4	2	2	1
8496	202	0	19-Nov-15	20	2	16	2	1	3	1	4	2	1	1
8497	202	0	7-Nov-12	17	2	16	2	1	3	1	4	2	4	1
8498	202	0	22-Sep-11	20	2	16	2	1	3	1	4	2	6	2
8499	202	0	8-Aug-09	16	2	16	2	1	3	1	4	2	1	1
8500	202	0	25-Oct-09	21	2	16	2	1	3	1	4	2	4	2
8501	202	0	24-Nov-09	10	2	16	2	1	3	1	4	2	1	1
8502	202	0	15-Oct-15	11	2	16	2	1	3	1	11	2	2	1
8503	202	0	14-Sep-11	18	2	16	2	1	3	1	11	2	1	1
8504	202	0	7-Jun-16	21	2	16	2	1	3	1	3	2	5	1
8505	202	0	21-Sep-07	15	2	16	2	1	3	1	6	2	4	1
8506	201	1	2-Feb-12	5	1	16	2	1	3	1	6	2	1	1
8507	101	1	13-May-16	16	1	16	2	1	3	1	13	2	6	1
8508	201	1	11-Apr-11	11	1	16	2	1	3	1	13	2	5	1
8509	201	1	20-Jan-10	2	1	16	2	1	3	1	13	2	1	2
8510	201	1	22-Aug-07	21	1	16	2	1	3	1	13	2	1	2
8511	201	1	22-Aug-07	21	1	16	2	1	3	1	13	2	2	1
8512	101	1	18-Jan-16	13	1	16	2	1	3	1	4	2	5	1
8513	201	1	24-Oct-16	6	1	16	2	3	3	1	4	2	1	2
8514	101	1	14-Dec-16	21	1	16	2	1	3	1	4	2	1	1
8515	101	1	27-Nov-15	17	1	16	2	1	3	1	4	2	4	1
8516	101	1	19-Jul-15	17	1	16	2	1	3	1	4	2	2	1
8517	101	1	4-Mar-15	14	1	16	2	1	3	1	4	2	4	1
8518	101	1	20-Nov-15	15	1	16	2	1	3	1	4	2	5	1
8519	101	1	6-Jan-15	22	1	16	2	1	3	1	4	2	5	4
8520	101	1	28-Sep-15	17	1	16	2	1	3	1	4	2	1	1
8521	101	1	6-Jan-15	20	1	16	2	1	3	1	4	2	5	1
8522	101	1	2-Apr-14	17	1	16	2	1	3	1	4	2	1	1
8523	101	1	27-Feb-14	9	1	16	2	1	3	1	4	2	1	1
8524	101	1	12-Sep-14	12	1	16	2	1	3	1	4	2	1	1
8525	101	1	12-Mar-14	22	1	16	2	1	3	1	4	2	1	1
8526	101	1	25-Dec-14	16	1	16	2	1	3	1	4	2	4	1
8527	101	1	17-Apr-14	16	1	16	2	1	3	1	4	2	1	1
8528	101	1	9-Dec-13	17	1	16	2	1	3	1	4	2	5	1
8529	101	1	17-Dec-13	9	1	16	2	1	3	1	4	2	1	1
8530	101	1	24-Apr-13	19	1	16	2	1	3	1	4	2	4	1
8531	101	1	17-May-13	14	1	16	2	1	3	1	4	2	5	1
8532	101	1	14-Jul-13	2	1	16	2	1	3	1	4	2	1	1
8533	101	1	22-Mar-13	12	1	16	2	1	3	1	4	2	1	1
8534	101	1	26-Mar-12	17	1	16	2	1	3	1	4	2	5	1
8535	101	1	30-Oct-12	16	1	16	2	3	3	1	4	2	5	4
8536	101	1	19-Apr-12	17	1	16	2	1	3	1	4	2	1	1
8537	101	1	13-Sep-12	11	1	16	2	1	3	1	4	2	4	1
8538	101	1	23-Feb-12	12	1	16	2	3	3	1	4	2	1	1
8539	101	1	29-Nov-12	16	1	16	2	1	3	1	4	2	1	1
8540	101	1	4-May-12	20	1	16	2	1	3	1	4	2	1	1
8541	101	1	2-Mar-12	22	1	16	2	1	3	1	4	2	5	1
8542	201	1	14-Sep-12	17	1	16	2	2	3	1	4	2	4	1
8543	201	1	30-Aug-12	20	1	16	2	2	3	1	4	2	6	1
8544	201	1	14-Sep-12	10	1	16	2	2	3	1	4	2	1	1
8545	101	1	2-Nov-11	11	1	16	2	1	3	1	4	2	5	1
8546	101	1	4-Jun-11	2	1	16	2	1	3	1	4	2	5	1
8547	101	1	6-Jun-11	10	1	16	2	1	3	1	4	2	1	1
8548	101	1	19-Sep-11	12	1	16	2	1	3	1	4	2	2	1
8549	101	1	27-Oct-11	16	1	16	2	1	3	1	4	2	5	1

8550	101	1	6-Jul-11	17	1	16	2	1	3	1	4	2	1	1
8551	101	1	8-Dec-11	18	1	16	2	1	3	1	4	2	5	1
8552	101	1	19-Sep-11	12	1	16	2	1	3	1	4	2	1	1
8553	101	1	9-Apr-10	19	1	16	2	1	3	1	4	2	1	1
8554	101	1	11-Jul-10	15	1	16	2	1	3	1	4	2	1	1
8555	101	1	4-May-10	22	1	16	2	1	3	1	4	2	1	1
8556	101	1	25-Jun-10	19	1	16	2	1	3	1	4	2	5	2
8557	101	1	6-Oct-10	15	1	16	2	2	3	1	4	2	5	1
8558	201	1	29-Nov-10	23	1	16	2	1	3	1	4	2	1	1
8559	101	1	1-Jun-10	18	1	16	2	2	3	1	4	2	5	1
8560	101	1	23-Jan-10	21	1	16	2	1	3	1	4	2	1	1
8561	101	1	2-Apr-10	21	1	16	2	1	3	1	4	2	1	1
8562	101	1	23-Jul-10	11	1	16	2	2	3	1	4	2	4	2
8563	101	1	16-Oct-09	20	1	16	2	1	3	1	4	2	1	1
8564	101	1	8-Sep-09	23	1	16	2	1	3	1	4	2	5	1
8565	101	1	16-Sep-09	11	1	16	2	1	3	1	4	2	5	1
8566	101	1	8-Sep-09	23	1	16	2	1	3	1	4	2	1	1
8567	101	1	21-Jan-08	10	1	16	2	1	3	1	4	2	1	1
8568	101	1	27-Nov-08	19	1	16	2	1	3	1	4	2	1	1
8569	101	1	30-Jun-08	12	1	16	2	1	3	1	4	2	1	1
8570	101	1	6-Oct-08	20	1	16	2	1	3	1	4	2	1	1
8571	101	1	27-Dec-08	18	1	16	2	1	3	1	4	2	4	2
8572	101	1	17-Sep-08	19	1	16	2	1	3	1	4	2	1	1
8573	101	1	18-Feb-08	18	1	16	2	1	3	1	4	2	1	1
8574	101	1	26-Oct-08	20	1	16	2	3	3	1	4	2	1	1
8575	101	1	26-Sep-07	11	1	16	2	1	3	1	4	2	1	1
8576	101	1	13-Mar-07	16	1	16	2	1	3	1	4	2	1	2
8577	101	1	2-Jun-07	11	1	16	2	1	3	1	4	2	1	1
8578	101	1	5-Jan-07	12	1	16	2	1	3	1	4	2	1	1
8579	101	1	5-Jun-07	14	1	16	2	1	3	1	4	2	1	1
8580	101	1	28-Jun-07	16	1	16	2	1	3	1	4	2	1	1
8581	101	1	17-Apr-07	13	1	16	2	1	3	1	4	2	4	1
8582	101	1	28-Sep-07	22	1	16	2	2	3	1	4	2	2	1
8583	101	1	28-Sep-07	22	1	16	2	2	3	1	4	2	2	1
8584	101	1	28-Sep-07	22	1	16	2	1	3	1	4	2	3	1
8585	101	1	5-Dec-07	16	1	16	2	1	3	1	4	2	1	1
8586	101	1	26-Sep-07	22	1	16	2	1	3	1	4	2	1	1
8587	101	1	29-Sep-06	21	1	16	2	1	3	1	4	2	1	1
8588	101	1	23-Oct-06	12	1	16	2	1	3	1	4	2	1	1
8589	101	1	26-Oct-06	12	1	16	2	1	3	1	4	2	1	1
8590	101	1	24-Nov-06	12	1	16	2	1	3	1	4	2	5	1
8591	101	1	22-May-06	17	1	16	2	1	3	1	4	2	1	2
8592	101	1	18-Sep-06	12	1	16	2	1	3	1	4	2	1	1
8593	201	1	2-Apr-06	9	1	16	2	1	3	1	4	2	1	1
8594	101	1	6-Oct-06	3	1	16	2	1	3	1	4	2	1	2
8595	201	1	22-May-06	19	1	16	2	1	3	1	4	2	1	1
8596	101	1	15-May-06	21	1	16	2	1	3	1	4	2	4	1
8597	101	1	16-May-06	19	1	16	2	1	3	1	4	2	1	1
8598	101	1	19-Mar-17	10	1	16	2	1	3	1	4	2	5	1
8599	201	1	26-Jan-17	0	1	16	2	2	3	1	4	2	5	1
8600	101	1	25-Feb-17	23	1	16	2	1	3	1	4	2	1	1
8601	201	1	27-Nov-17	10	1	16	2	3	3	1	4	2	2	1
8602	101	1	27-Sep-10	8	1	16	2	1	3	1	11	2	2	1
8603	101	1	19-Feb-07	13	1	16	2	1	3	1	11	2	2	1
8604	101	1	7-Nov-15	18	1	16	2	1	3	1	3	2	4	1
8605	101	1	4-Nov-11	10	1	16	2	2	3	1	3	2	3	1
8606	201	1	26-Apr-08	19	1	16	2	3	3	1	3	2	1	1
8607	201	1	18-Feb-07	1	1	16	2	2	3	1	3	2	1	1
8608	101	1	4-Apr-15	0	1	16	2	1	3	1	6	2	1	2
8609	101	1	1-Dec-11	19	1	16	2	1	3	1	6	2	2	2
8610	201	1	5-Jan-11	23	1	16	2	1	3	1	6	2	1	1
8611	201	1	19-Jun-11	16	1	16	2	1	3	1	6	2	2	2
8612	201	1	15-Mar-10	23	1	16	2	1	3	1	6	2	4	1
8613	201	1	16-Mar-10	0	1	16	2	1	3	1	6	2	2	1
8614	101	1	29-Sep-07	12	1	16	2	1	3	1	6	2	3	2
8615	101	1	20-Apr-06	8	1	16	2	1	3	1	6	2	1	1
8616	201	1	8-May-06	19	1	16	2	2	3	1	6	2	1	1
8617	101	1	24-Sep-10	19	2	16	2	1	3	1	13	2	6	1
8618	101	1	2-Aug-10	23	2	16	2	1	3	1	13	2	6	1
8619	101	1	18-Aug-10	16	2	16	2	1	3	1	13	2	4	1
8620	101	1	7-Sep-15	16	2	16	2	1	3	1	4	2	5	1
8621	101	1	16-Oct-15	13	2	16	2	1	3	1	4	2	1	1
8622	101	1	14-Dec-11	17	2	16	2	1	3	1	4	2	1	1
8623	101	1	14-Jul-10	20	2	16	2	3	3	1	4	2	5	2
8624	101	1	25-Jun-10	16	2	16	2	1	3	1	4	2	1	1
8625	101	1	3-Feb-09	12	2	16	2	1	3	1	4	2	5	1
8626	201	1	19-Mar-09	18	2	16	2	1	3	1	4	2	1	1
8627	201	1	2-Jun-09	18	2	16	2	2	3	1	4	2	6	1
8628	101	1	8-Nov-06	17	2	16	2	1	3	1	4	2	1	1
8629	101	1	18-Jul-12	2	2	16	2	1	3	1	6	2	2	1
8630	101	1	27-May-07	15	2	16	2	1	3	1	6	2	2	1
8631	202	0	22-May-16	20	1	17	2	1	3	1	4	2	7	1
8632	202	0	23-Feb-12	19	1	17	2	1	3	1	4	2	1	1
8633	202	0	4-Oct-16	8	1	17	2	3	3	1	4	2	5	1
8634	202	0	24-Sep-16	11	1	17	2	1	3	1	4	2	1	1
8635	202	0	26-Feb-12	0	1	17	2	1	3	1	10	2	7	1
8636	202	0	15-May-16	2	1	17	2	1	3	1	4	2	1	2
8637	202	0	1-Jan-10	11	1	17	2	1	3	1	13	2	2	1
8638	202	0	16-Sep-09	21	1	17	2	1	3	1	13	2	1	2
8639	202	0	6-Jul-08	11	1	17	2	1	3	1	13	2	1	1
8640	202	0	19-Nov-08	17	1	17	2	1	3	1	13	2	3	1
8641	202	0	19-Nov-08	18	1	17	2	1	3	1	13	2	1	2
8642	202	0	26-Oct-16	12	1	17	2	1	3	1	4	2	5	1
8643	202	0	8-Sep-16	12	1	17	2	3	3	1	4	2	4	1
8644	202	0	24-Jun-16	15	1	17	2	2	3	1	4	2	1	1
8645	202	0	3-May-16	8	1	17	2	1	3	1	4	2	1	1
8646	202	0	3-May-16	8	1	17	2	1	3	1	4	2	5	1
8647	202	0	3-May-16	8	1	17	2	1	3	1	4	2	1	1
8648	202	0	25-Sep-15	13	1	17	2	3	3	1	4	2	5	1
8649	202	0	28-Oct-14	20	1	17	2	3	3	1	4	2	4	1
8650	202	0	25-Feb-14	21	1	17	2	1	3	1	4	2	4	1
8651	202	0	23-Aug-14	21	1	17	2	1	3	1	4	2	5	2
8652	202	0	12-Jul-14	1	1	17	2	1	3	1	4	2	1	2
8653	202	0	4-Jan-14	1	1	17	2	3	3	1	4	2	1	1
8654	202	0	15-Feb-14	2	1	17	2	1	3	1	4	2	1	1
8655	202	0	31-Oct-13	8	1	17	2	2	3	1	4	2	1	1
8656	202	0	1-Nov-13	8	1	17	2	1	3	1	4	2	5	1
8657	202	0	20-Mar-13	12	1	17	2	1	3	1	4	2	1	1
8658	202	0	25-Mar-13	20	1	17	2	1	3	1	4	2	1	2
8659	202	0	27-Mar-13	21	1	17	2	1	3	1	4	2	1	1
8660	202	0	14-Feb-13	11	1	17	2	1	3	1	4	2	2	2
8661	202	0	17-Feb-13	3	1	17	2	1	3	1	4	2	1	1
8662	202	0	30-May-13	19	1	17	2	1	3	1	4	2	1	1
8663	202	0	18-Mar-13	0	1	17	2	1	3	1	4	2	1	1
8664	202	0	18-Mar-13	0	1	17	2	1	3	1	4	2	1	1
8665	202	0	31-Oct-12	19	1	17	2	1	3	1	4	2	1	1
8666	202	0	20-Dec-12	17	1	17	2	1	3	1	4	2	5	1
8667	202	0	18-Jan-11	17	1	17	2	1	3	1	4	2	1	1
8668	202	0	11-Nov-11	22	1	17	2	1	3	1	4	2	1	2
8669	202	0	22-Sep-11	22	1	17	2	1	3	1	4	2	1	1
8670	202	0	24-Dec-09	0	1	17	2	1	3	1	4	2	1	2
8671	202	0	27-Sep-09	12	1	17	2	1	3	1	4	2	1	1
8672	202	0	5-Jan-09	16	1	17	2	1	3	1	4	2	6	1
8673	202	0	2-Apr-09	18	1	17	2	1	3	1	4	2	6	1

8674	202	0	8-May-09	12	1	17	2	1	3	1	4	2	1	2
8675	202	0	20-Jun-09	12	1	17	2	1	3	1	4	2	1	1
8676	202	0	23-Jun-09	18	1	17	2	1	3	1	4	2	1	2
8677	202	0	16-Oct-08	20	1	17	2	1	3	1	4	2	1	2
8678	202	0	12-Mar-08	10	1	17	2	1	3	1	4	2	1	1
8679	202	0	9-Jan-08	12	1	17	2	1	3	1	4	2	1	1
8680	202	0	3-Jul-08	20	1	17	2	1	3	1	4	2	4	2
8681	202	0	31-Aug-08	20	1	17	2	1	3	1	4	2	2	1
8682	202	0	27-Mar-07	12	1	17	2	1	3	1	4	2	1	1
8683	202	0	16-Oct-07	13	1	17	2	1	3	1	4	2	5	1
8684	202	0	7-Apr-07	16	1	17	2	1	3	1	4	2	5	1
8685	202	0	20-Sep-07	12	1	17	2	1	3	1	4	2	1	1
8686	202	0	26-Sep-07	23	1	17	2	1	3	1	4	2	1	1
8687	202	0	30-Oct-07	9	1	17	2	1	3	1	4	2	1	1
8688	202	0	25-Oct-07	19	1	17	2	1	3	1	4	2	5	1
8689	202	0	19-Mar-07	21	1	17	2	1	3	1	4	2	6	1
8690	202	0	7-Oct-07	23	1	17	2	1	3	1	4	2	1	1
8691	202	0	4-Nov-07	22	1	17	2	1	3	1	4	2	1	1
8692	202	0	21-Sep-07	12	1	17	2	1	3	1	4	2	1	1
8693	202	0	21-Sep-07	12	1	17	2	1	3	1	4	2	1	1
8694	202	0	9-Nov-07	14	1	17	2	1	3	1	4	2	1	1
8695	202	0	5-Apr-06	11	1	17	2	1	3	1	4	2	4	1
8696	202	0	24-Feb-06	16	1	17	2	1	3	1	4	2	1	1
8697	202	0	24-Feb-06	4	1	17	2	1	3	1	4	2	1	2
8698	202	0	12-Oct-06	20	1	17	2	1	3	1	4	2	1	1
8699	202	0	25-Apr-06	7	1	17	2	2	3	1	4	2	1	1
8700	202	0	18-Oct-06	10	1	17	2	1	3	1	4	2	4	1
8701	202	0	20-Apr-06	16	1	17	2	1	3	1	4	2	1	1
8702	202	0	27-Nov-06	1	1	17	2	3	3	1	4	2	6	2
8703	202	0	10-Jun-17	7	1	17	2	1	3	1	4	2	1	1
8704	202	0	3-Nov-17	22	1	17	2	1	3	1	4	2	4	1
8705	202	0	12-Jan-17	16	1	17	2	2	3	1	4	2	5	1
8706	202	0	19-Jun-17	5	1	17	2	2	3	1	4	2	1	1
8707	202	0	23-Mar-17	10	1	17	2	3	3	1	4	2	4	1
8708	202	0	26-Dec-17	21	1	17	2	1	3	1	4	2	5	1
8709	202	0	4-Jan-17	19	1	17	2	3	3	1	4	2	1	1
8710	202	0	14-Sep-11	19	1	17	2	1	3	1	11	2	1	2
8711	202	0	27-Oct-15	18	1	17	2	3	3	1	3	2	1	1
8712	202	0	1-Dec-09	6	1	17	2	1	3	1	3	2	4	1
8713	202	0	5-Sep-07	17	1	17	2	1	3	1	3	2	1	1
8714	202	0	21-Nov-07	9	1	17	2	1	3	1	3	2	4	2
8715	202	0	16-Nov-07	10	1	17	2	1	3	1	3	2	4	1
8716	202	0	5-Mar-17	22	1	17	2	1	3	1	3	2	5	1
8717	202	0	7-Mar-17	23	1	17	2	2	3	1	3	2	4	1
8718	202	0	25-Feb-16	3	1	17	2	1	3	1	6	2	2	1
8719	202	0	8-Nov-12	15	1	17	2	1	3	1	6	2	1	1
8720	202	0	16-Feb-12	13	1	17	2	1	3	1	6	2	1	2
8721	202	0	30-Mar-10	10	1	17	2	1	3	1	6	2	1	2
8722	202	0	25-Apr-09	3	1	17	2	1	3	1	6	2	1	1
8723	202	0	22-Jul-07	1	1	17	2	1	3	1	6	2	2	2
8724	202	0	4-Oct-06	2	1	17	2	1	3	1	6	2	2	2
8725	202	0	21-Dec-07	16	1	17	2	1	3	1	13	2	1	2
8726	202	0	26-Dec-16	11	2	17	2	2	3	1	4	2	1	2
8727	202	0	24-Apr-09	12	2	17	2	1	3	1	13	2	1	1
8728	202	0	5-Nov-13	5	2	17	2	2	3	1	4	2	1	1
8729	202	0	5-Sep-10	18	2	17	2	1	3	1	4	2	1	2
8730	202	0	19-Oct-09	12	2	17	2	1	3	1	4	2	1	1
8731	202	0	21-Mar-09	21	2	17	2	1	3	1	4	2	1	1
8732	202	0	16-Jan-08	10	2	17	2	1	3	1	4	2	4	1
8733	202	0	21-Sep-07	15	2	17	2	1	3	1	4	2	1	1
8734	202	0	14-Apr-07	23	2	17	2	3	3	1	13	2	5	1
8735	201	1	13-Nov-13	22	1	17	2	2	3	1	6	2	4	1
8736	101	1	13-Jan-06	21	1	17	2	1	3	1	13	2	5	2
8737	101	1	26-Dec-12	11	1	17	2	1	3	1	1	2	1	1
8738	101	1	11-Jan-16	22	1	17	2	1	3	1	4	2	1	1
8739	101	1	15-Jul-16	16	1	17	2	2	3	1	4	2	1	1
8740	101	1	4-Oct-16	0	1	17	2	1	3	1	4	2	1	1
8741	101	1	14-Dec-15	19	1	17	2	1	3	1	4	2	4	1
8742	101	1	27-Jan-15	3	1	17	2	2	3	1	4	2	1	1
8743	201	1	25-Jun-14	7	1	17	2	2	3	1	4	2	4	1
8744	101	1	28-Apr-14	20	1	17	2	1	3	1	4	2	1	1
8745	101	1	22-Feb-14	20	1	17	2	2	3	1	4	2	1	2
8746	101	1	16-Sep-13	14	1	17	2	1	3	1	4	2	5	1
8747	101	1	16-Sep-13	14	1	17	2	2	3	1	4	2	5	1
8748	101	1	8-Mar-13	16	1	17	2	1	3	1	4	2	1	4
8749	101	1	1-Jan-13	23	1	17	2	2	3	1	4	2	5	1
8750	101	1	22-Jun-13	23	1	17	2	1	3	1	4	2	1	1
8751	101	1	2-Oct-13	9	1	17	2	1	3	1	4	2	1	1
8752	101	1	15-Feb-13	1	1	17	2	1	3	1	4	2	1	1
8753	101	1	17-May-13	23	1	17	2	1	3	1	4	2	2	1
8754	101	1	31-Oct-12	19	1	17	2	1	3	1	4	2	1	1
8755	101	1	21-Mar-12	17	1	17	2	1	3	1	4	2	1	1
8756	101	1	30-May-12	12	1	17	2	1	3	1	4	2	1	1
8757	101	1	3-Jul-12	23	1	17	2	1	3	1	4	2	1	1
8758	101	1	2-Sep-12	0	1	17	2	3	3	1	4	2	5	2
8759	101	1	21-Sep-12	12	1	17	2	1	3	1	4	2	1	1
8760	101	1	17-May-11	16	1	17	2	2	3	1	4	2	1	1
8761	101	1	4-Mar-11	21	1	17	2	1	3	1	4	2	1	1
8762	101	1	17-Jan-11	23	1	17	2	1	3	1	4	2	1	1
8763	101	1	1-Aug-11	23	1	17	2	1	3	1	4	2	5	1
8764	101	1	10-May-11	20	1	17	2	1	3	1	4	2	5	1
8765	101	1	23-Oct-11	19	1	17	2	1	3	1	4	2	1	1
8766	101	1	23-Oct-11	10	1	17	2	1	3	1	4	2	7	2
8767	101	1	14-Jan-11	23	1	17	2	1	3	1	4	2	1	1
8768	101	1	14-Oct-11	18	1	17	2	1	3	1	4	2	2	1
8769	101	1	31-May-10	20	1	17	2	1	3	1	4	2	1	2
8770	101	1	4-Nov-10	13	1	17	2	1	3	1	4	2	5	1
8771	101	1	8-Jul-10	10	1	17	2	1	3	1	4	2	1	1
8772	101	1	8-Jul-10	10	1	17	2	1	3	1	4	2	3	1
8773	101	1	23-Oct-10	1	1	17	2	1	3	1	4	2	1	1
8774	101	1	31-Aug-10	22	1	17	2	1	3	1	4	2	1	1
8775	101	1	14-Mar-10	22	1	17	2	1	3	1	4	2	1	1
8776	201	1	4-Jun-10	18	1	17	2	2	3	1	4	2	2	1
8777	201	1	27-Jul-10	4	1	17	2	3	3	1	4	2	2	1
8778	101	1	25-Feb-09	16	1	17	2	1	3	1	4	2	4	1
8779	101	1	30-Nov-09	15	1	17	2	1	3	1	4	2	5	1
8780	101	1	8-Sep-09	23	1	17	2	1	3	1	4	2	1	1
8781	101	1	20-Jun-09	2	1	17	2	1	3	1	4	2	2	1
8782	101	1	15-Apr-08	8	1	17	2	1	3	1	4	2	4	1
8783	101	1	1-Jan-08	9	1	17	2	1	3	1	4	2	1	1
8784	101	1	18-Oct-08	1	1	17	2	1	3	1	4	2	1	1
8785	101	1	18-Oct-08	1	1	17	2	1	3	1	4	2	1	1
8786	101	1	8-Jul-08	18	1	17	2	1	3	1	4	2	1	1
8787	101	1	19-Sep-07	14	1	17	2	1	3	1	4	2	5	1
8788	201	1	16-Oct-07	20	1	17	2	2	3	1	4	2	5	1
8789	101	1	17-Mar-07	12	1	17	2	1	3	1	4	2	1	1
8790	201	1	11-Nov-07	22	1	17	2	2	3	1	4	2	5	1
8791	101	1	3-Feb-07	12	1	17	2	1	3	1	4	2	2	1
8792	101	1	12-Oct-07	16	1	17	2	1	3	1	4	2	1	1
8793	201	1	1-Aug-07	12	1	17	2	2	3	1	4	2	4	1
8794	201	1	26-Jun-07	17	1	17	2	2	3	1	4	2	1	1
8795	101	1	17-Sep-07	19	1	17	2	1	3	1	4	2	1	1
8796	101	1	15-Dec-06	1	1	17	2	1	3	1	4	2	1	2
8797	101	1	23-Aug-06	17	1	17	2	1	3	1	4	2	1	1

8798	201	1	9-Mar-06	19	1	17	2	1	3	1	4	2	1	1
8799	101	1	23-Feb-06	18	1	17	2	1	3	1	4	2	4	1
8800	101	1	11-Nov-17	22	1	17	2	1	3	1	4	2	4	1
8801	101	1	26-Sep-17	13	1	17	2	1	3	1	4	2	1	1
8802	101	1	10-Apr-17	18	1	17	2	1	3	1	4	2	1	1
8803	201	1	3-Nov-17	14	1	17	2	2	3	1	4	2	5	1
8804	101	1	31-Oct-10	22	1	17	2	1	3	1	11	2	3	1
8805	101	1	17-May-13	23	1	17	2	1	3	1	3	2	1	1
8806	201	1	22-Apr-11	17	1	17	2	3	3	1	3	2	6	2
8807	101	1	7-Jan-16	11	1	17	2	1	3	1	6	2	2	2
8808	101	1	19-Oct-12	15	1	17	2	1	3	1	6	2	4	1
8809	101	1	28-Aug-12	22	1	17	2	1	3	1	6	2	2	1
8810	201	1	5-Jan-11	22	1	17	2	3	3	1	6	2	6	1
8811	101	1	6-Feb-11	1	1	17	2	1	3	1	6	2	5	2
8812	101	1	11-Sep-11	19	1	17	2	1	3	1	6	2	2	4
8813	201	1	15-Mar-10	23	1	17	2	1	3	1	6	2	2	2
8814	101	1	25-Nov-10	16	1	17	2	1	3	1	6	2	4	1
8815	101	1	6-Oct-08	20	1	17	2	1	3	1	6	2	1	1
8816	101	1	7-Aug-07	23	1	17	2	2	3	1	6	2	1	1
8817	201	1	10-Aug-07	2	1	17	2	1	3	1	6	2	4	2
8818	201	1	3-May-13	1	1	17	2	2	3	1	13	2	6	1
8819	101	1	18-Jun-06	14	1	17	2	2	3	1	13	2	6	1
8820	201	1	30-Jul-16	15	2	17	2	1	3	1	4	2	1	1
8821	101	1	5-Jan-15	18	2	17	2	1	3	1	4	2	1	1
8822	101	1	30-Apr-14	11	2	17	2	1	3	1	4	2	1	1
8823	101	1	20-May-14	8	2	17	2	1	3	1	4	2	4	1
8824	101	1	21-May-10	16	2	17	2	1	3	1	4	2	1	1
8825	101	1	17-Mar-09	14	2	17	2	1	3	1	4	2	1	1
8826	101	1	23-Aug-09	22	2	17	2	1	3	1	4	2	4	1
8827	101	1	28-Nov-06	13	2	17	2	2	3	1	4	2	1	1
8828	201	1	19-May-08	18	2	17	2	2	3	1	6	2	4	2
8829	201	1	20-Apr-09	12	2	17	2	1	3	1	13	2	6	1
8830	101	1	22-Oct-06	9	1	11	2	1	2	1	4	2	2	1
8831	101	1	6-Dec-16	10	2	12	2	1	2	1	6	2	2	1
8832	202	0	18-Aug-17	2	1	13	2	2	2	1	6	2	2	2
8833	202	0	2-Nov-07	0	2	13	2	1	2	1	10	2	7	1
8834	202	0	11-Dec-13	14	2	13	2	1	2	1	3	2	2	2
8835	101	1	14-Mar-10	18	2	13	2	1	2	1	3	2	1	3
8836	101	1	1-Jun-12	17	1	14	2	1	2	1	6	2	2	1
8837	101	1	18-Jul-14	13	1	15	2	1	2	1	1	2	4	1
8838	101	1	10-Jun-07	20	1	15	2	1	2	1	7	2	2	1
8839	101	1	17-Oct-13	22	1	15	2	1	2	1	4	2	2	1
8840	101	1	21-Dec-06	0	1	15	2	1	2	1	4	2	1	1
8841	101	1	17-Dec-11	11	1	15	2	1	2	1	3	2	2	1
8842	101	1	23-Sep-08	18	1	15	2	1	2	1	3	2	3	1
8843	101	1	28-Nov-14	15	1	15	2	1	2	1	6	2	2	1
8844	201	1	14-Feb-14	10	2	15	2	3	2	1	3	2	3	1
8845	202	0	8-Aug-07	23	1	16	2	1	2	1	10	2	7	2
8846	202	0	2-Jul-11	2	1	16	2	2	2	1	13	2	1	1
8847	202	0	11-Nov-08	13	1	16	2	1	2	1	13	2	2	2
8848	202	0	23-Jun-13	14	1	16	2	3	2	1	4	2	2	2
8849	202	0	5-Apr-06	17	1	16	2	1	2	1	4	2	1	1
8850	202	0	14-Sep-17	10	1	16	2	2	2	1	4	2	1	1
8851	202	0	19-Jul-06	2	2	16	2	1	2	1	10	2	7	1
8852	101	1	10-Dec-11	18	1	16	2	3	2	1	1	2	2	1
8853	201	1	13-Dec-15	2	1	16	2	1	2	1	4	2	1	1
8854	101	1	22-Jun-11	11	1	16	2	1	2	1	6	2	2	1
8855	201	1	28-May-10	8	2	16	2	1	2	1	3	2	6	2
8856	202	0	15-Apr-09	23	1	17	2	2	2	1	10	2	7	2
8857	202	0	15-Apr-09	23	1	17	2	2	2	1	10	2	7	2
8858	202	0	21-Apr-09	20	1	17	2	3	2	1	10	2	7	2
8859	202	0	28-Dec-08	19	1	17	2	1	2	1	10	2	7	2
8860	202	0	18-Jun-10	17	1	17	2	1	2	1	13	2	6	1
8861	202	0	3-Jul-13	22	1	17	2	1	2	1	6	2	2	1
8862	202	0	28-Nov-07	19	1	17	2	1	2	1	6	2	2	1
8863	202	0	3-Apr-06	11	1	17	2	1	2	1	6	2	4	1
8864	202	0	11-Aug-10	12	2	17	2	1	2	1	10	2	7	2
8865	202	0	2-Sep-12	13	2	17	2	1	2	1	9	2	7	1
8866	201	1	20-Jul-08	5	1	17	2	1	2	1	10	2	7	1
8867	101	1	12-Aug-13	16	1	17	2	1	2	1	4	2	1	1
8868	101	1	7-Jul-10	8	1	17	2	1	2	1	3	2	1	2
8869	201	1	20-Mar-07	4	2	17	2	1	2	1	6	2	2	1
8870	101	1	12-Dec-16	7	1	9	2	1	1	0	3	2	3	1
8871	201	1	6-Nov-15	12	1	9	2	2	1	0	3	2	1	1
8872	101	1	16-Nov-11	11	1	9	2	1	1	0	6	2	3	1
8873	201	1	25-Feb-11	21	2	9	2	2	1	0	11	2	2	1
8874	201	1	8-Apr-10	12	2	9	2	2	1	0	3	2	1	1
8875	202	0	2-Feb-11	21	1	10	2	2	1	0	7	2	7	2
8876	202	0	30-Dec-16	16	1	10	2	1	1	0	4	2	1	1
8877	202	0	18-Sep-09	18	1	10	2	3	1	0	11	2	7	2
8878	202	0	6-Jan-16	16	1	10	2	3	1	0	3	2	3	1
8879	202	0	15-Jun-17	0	1	10	2	1	1	0	3	2	3	1
8880	202	0	31-Jan-17	16	2	10	2	3	1	0	6	2	2	1
8881	202	0	11-Dec-10	22	2	10	2	1	1	0	4	2	1	2
8882	202	0	15-Jan-14	14	2	10	2	1	1	0	11	2	3	1
8883	101	1	4-Nov-09	17	1	10	2	1	1	0	4	2	1	1
8884	201	1	3-Sep-06	16	1	10	2	1	1	0	11	2	3	1
8885	101	1	8-May-11	11	1	10	2	1	1	0	3	2	1	1
8886	101	1	1-Jan-06	15	1	10	2	1	1	0	3	2	2	1
8887	101	1	27-Nov-17	10	1	10	2	1	1	0	3	2	3	1
8888	101	1	3-Apr-11	7	2	10	2	2	1	0	11	2	3	1
8889	201	1	21-Jun-09	14	2	10	2	2	1	0	3	2	3	1
8890	202	0	5-Sep-17	14	1	11	2	2	1	0	7	2	3	1
8891	202	0	23-Jun-08	15	1	11	2	3	1	0	13	2	1	1
8892	202	0	6-Mar-17	17	1	11	2	1	1	0	4	2	1	1
8893	202	0	15-Oct-16	13	1	11	2	3	1	0	11	2	2	1
8894	202	0	26-Apr-13	22	1	11	2	1	1	0	11	2	2	1
8895	202	0	9-Oct-16	17	1	11	2	3	1	0	3	2	3	2
8896	202	0	13-Oct-15	20	1	11	2	3	1	0	3	2	2	1
8897	202	0	29-Jun-09	18	1	11	2	1	1	0	3	2	2	2
8898	202	0	28-Nov-07	12	1	11	2	1	1	0	6	2	1	2
8899	202	0	10-Sep-09	0	2	11	2	1	1	0	13	2	1	1
8900	202	0	23-Apr-10	10	2	11	2	1	1	0	4	2	1	2
8901	202	0	28-Apr-16	15	2	11	2	1	1	0	3	2	1	1
8902	202	0	26-May-14	17	2	11	2	1	1	0	3	2	3	1
8903	202	0	31-May-13	21	2	11	2	2	1	0	3	2	5	1
8904	202	0	3-Mar-10	21	2	11	2	1	1	0	3	2	1	1
8905	202	0	19-Sep-09	19	2	11	2	1	1	0	3	2	1	1
8906	201	1	3-Apr-14	14	1	11	2	3	1	0	6	2	2	1
8907	201	1	24-Apr-16	9	1	11	2	1	1	0	4	2	3	1
8908	201	1	23-Jun-08	15	1	11	2	3	1	0	13	2	1	1
8909	201	1	31-May-10	12	1	11	2	1	1	0	1	2	3	1
8910	101	1	21-Aug-06	21	1	11	2	1	1	0	1	2	3	1
8911	101	1	21-Aug-06	21	1	11	2	1	1	0	1	2	1	2
8912	101	1	21-Aug-06	21	1	11	2	1	1	0	1	2	3	1
8913	201	1	19-Apr-15	20	1	11	2	3	1	0	4	2	3	1
8914	101	1	28-Oct-13	14	1	11	2	1	1	0	4	2	2	1
8915	101	1	16-Oct-12	17	1	11	2	1	1	0	4	2	3	1
8916	201	1	28-Oct-11	10	1	11	2	3	1	0	4	2	1	1
8917	101	1	15-Dec-11	12	1	11	2	1	1	0	4	2	2	1
8918	201	1	9-Dec-10	14	1	11	2	3	1	0	4	2	2	1
8919	201	1	11-Dec-10	9	1	11	2	3	1	0	4	2	2	1
8920	201	1	9-Dec-10	16	1	11	2	3	1	0	4	2	3	1
8921	101	1	18-Aug-10	18	1	11	2	3	1	0	4	2	7	2

8922	201	1	17-Apr-09	13	1	11	2	2	1	0	4	2	2	1
8923	101	1	21-Apr-07	14	1	11	2	1	1	0	4	2	4	1
8924	201	1	11-Jan-15	16	1	11	2	2	1	0	11	2	4	1
8925	201	1	31-Dec-10	13	1	11	2	1	1	0	11	2	3	1
8926	101	1	2-Feb-06	13	1	11	2	1	1	0	11	2	2	1
8927	201	1	16-Sep-17	9	1	11	2	1	1	0	11	2	3	1
8928	201	1	7-Apr-16	13	1	11	2	2	1	0	3	2	3	1
8929	201	1	21-Nov-16	16	1	11	2	1	1	0	3	2	3	1
8930	201	1	22-Mar-16	14	1	11	2	2	1	0	3	2	1	1
8931	101	1	12-Mar-15	16	1	11	2	1	1	0	3	2	3	1
8932	201	1	21-Apr-14	18	1	11	2	3	1	0	3	2	2	1
8933	201	1	12-Nov-14	10	1	11	2	3	1	0	3	2	1	1
8934	201	1	3-Apr-14	12	1	11	2	2	1	0	3	2	1	1
8935	101	1	5-Nov-13	11	1	11	2	1	1	0	3	2	4	1
8936	201	1	16-May-13	15	1	11	2	2	1	0	3	2	2	1
8937	101	1	19-Mar-13	11	1	11	2	1	1	0	3	2	1	1
8938	201	1	26-Sep-13	9	1	11	2	2	1	0	3	2	1	1
8939	101	1	13-Jan-13	17	1	11	2	1	1	0	3	2	2	2
8940	201	1	6-Jan-13	10	1	11	2	3	1	0	3	2	1	1
8941	201	1	9-Jul-12	15	1	11	2	2	1	0	3	2	3	1
8942	101	1	31-Dec-12	14	1	11	2	1	1	0	3	2	6	1
8943	101	1	23-Oct-12	10	1	11	2	1	1	0	3	2	2	1
8944	201	1	23-Apr-12	10	1	11	2	2	1	0	3	2	3	1
8945	201	1	16-Mar-11	18	1	11	2	3	1	0	3	2	1	1
8946	101	1	15-Dec-11	7	1	11	2	1	1	0	3	2	1	1
8947	201	1	20-Oct-11	17	1	11	2	2	1	0	3	2	1	1
8948	201	1	1-Nov-11	21	1	11	2	3	1	0	3	2	1	1
8949	201	1	2-Nov-11	22	1	11	2	3	1	0	3	2	3	1
8950	101	1	21-Oct-10	20	1	11	2	1	1	0	3	2	2	4
8951	201	1	28-Jul-10	13	1	11	2	3	1	0	3	2	1	1
8952	201	1	30-Dec-09	12	1	11	2	2	1	0	3	2	1	1
8953	101	1	28-Sep-09	15	1	11	2	1	1	0	3	2	4	1
8954	201	1	19-Jun-09	17	1	11	2	1	1	0	3	2	2	1
8955	201	1	31-Aug-08	20	1	11	2	3	1	0	3	2	4	1
8956	201	1	21-Dec-08	17	1	11	2	1	1	0	3	2	2	2
8957	201	1	25-Sep-08	9	1	11	2	3	1	0	3	2	3	1
8958	101	1	21-Sep-07	12	1	11	2	1	1	0	3	2	3	1
8959	101	1	7-Jun-07	17	1	11	2	1	1	0	3	2	2	1
8960	101	1	28-Sep-07	12	1	11	2	1	1	0	3	2	2	1
8961	201	1	18-Aug-17	22	1	11	2	1	1	0	3	2	3	1
8962	101	1	5-Apr-15	11	1	11	2	1	1	0	6	2	1	1
8963	201	1	11-Aug-10	21	2	11	2	2	1	0	1	2	5	1
8964	201	1	11-Jul-14	17	2	11	2	3	1	0	4	2	3	1
8965	201	1	14-Jul-09	17	2	11	2	3	1	0	4	2	3	1
8966	201	1	28-Aug-14	17	2	11	2	3	1	0	11	2	2	1
8967	101	1	5-Aug-11	17	2	11	2	1	1	0	11	2	2	1
8968	101	1	21-Oct-17	19	2	11	2	1	1	0	11	2	2	1
8969	201	1	3-Feb-16	21	2	11	2	3	1	0	3	2	3	1
8970	201	1	22-Mar-16	14	2	11	2	2	1	0	3	2	3	1
8971	101	1	5-Jan-15	17	2	11	2	1	1	0	3	2	3	1
8972	101	1	18-Jul-13	9	2	11	2	1	1	0	3	2	1	4
8973	101	1	18-Jul-12	20	2	11	2	1	1	0	3	2	1	1
8974	201	1	22-Apr-11	15	2	11	2	1	1	0	3	2	2	2
8975	101	1	30-Apr-07	19	2	11	2	1	1	0	3	2	2	1
8976	202	0	12-Dec-13	14	1	12	2	1	1	0	1	2	5	1
8977	202	0	9-Jun-17	14	1	12	2	1	1	0	4	2	1	1
8978	202	0	30-Jul-10	5	1	12	2	1	1	0	10	2	7	1
8979	202	0	5-May-16	16	1	12	2	1	1	0	4	2	4	1
8980	202	0	27-Sep-16	19	1	12	2	3	1	0	4	2	3	1
8981	202	0	16-Nov-15	12	1	12	2	3	1	0	4	2	3	1
8982	202	0	25-Sep-13	18	1	12	2	1	1	0	4	2	1	1
8983	202	0	17-Sep-12	16	1	12	2	2	1	0	4	2	2	1
8984	202	0	3-Mar-17	11	1	12	2	1	1	0	4	2	2	1
8985	202	0	10-Apr-17	16	1	12	2	3	1	0	4	2	4	1
8986	202	0	25-Jun-17	13	1	12	2	1	1	0	11	2	2	1
8987	202	0	14-Nov-17	17	1	12	2	2	1	0	11	2	2	1
8988	202	0	22-Apr-17	19	1	12	2	3	1	0	11	2	3	1
8989	202	0	29-Sep-16	16	1	12	2	1	1	0	3	2	3	1
8990	202	0	24-Sep-16	14	1	12	2	1	1	0	3	2	2	1
8991	202	0	24-Mar-16	16	1	12	2	1	1	0	3	2	3	1
8992	202	0	8-Feb-16	2	1	12	2	1	1	0	3	2	1	1
8993	202	0	21-Jun-16	17	1	12	2	1	1	0	3	2	4	1
8994	202	0	8-Sep-15	15	1	12	2	1	1	0	3	2	2	2
8995	202	0	17-Sep-14	11	1	12	2	1	1	0	3	2	1	1
8996	202	0	23-Oct-14	10	1	12	2	1	1	0	3	2	2	1
8997	202	0	22-Dec-14	10	1	12	2	1	1	0	3	2	3	2
8998	202	0	29-Oct-14	13	1	12	2	1	1	0	3	2	1	1
8999	202	0	8-Oct-13	15	1	12	2	1	1	0	3	2	1	1
9000	202	0	8-Oct-13	12	1	12	2	1	1	0	3	2	2	2
9001	202	0	13-Jul-13	18	1	12	2	1	1	0	3	2	2	1
9002	202	0	27-Mar-13	20	1	12	2	1	1	0	3	2	3	1
9003	202	0	14-Apr-13	17	1	12	2	1	1	0	3	2	1	1
9004	202	0	9-May-10	8	1	12	2	1	1	0	3	2	2	2
9005	202	0	9-Nov-10	18	1	12	2	1	1	0	3	2	3	2
9006	202	0	28-Dec-10	17	1	12	2	1	1	0	3	2	2	1
9007	202	0	10-May-10	11	1	12	2	3	1	0	3	2	3	1
9008	202	0	26-May-09	20	1	12	2	1	1	0	3	2	2	1
9009	202	0	6-Nov-09	18	1	12	2	1	1	0	3	2	2	1
9010	202	0	28-Oct-08	17	1	12	2	2	1	0	3	2	7	2
9011	202	0	20-Dec-07	9	1	12	2	1	1	0	3	2	1	1
9012	202	0	17-Oct-07	16	1	12	2	1	1	0	3	2	1	1
9013	202	0	14-Sep-07	21	1	12	2	3	1	0	3	2	2	2
9014	202	0	22-Nov-07	11	1	12	2	1	1	0	3	2	2	2
9015	202	0	2-Apr-07	13	1	12	2	3	1	0	3	2	2	2
9016	202	0	3-Nov-06	9	1	12	2	1	1	0	3	2	3	1
9017	202	0	3-Mar-17	11	1	12	2	1	1	0	3	2	1	1
9018	202	0	5-Nov-17	16	1	12	2	1	1	0	3	2	2	2
9019	202	0	4-Sep-17	4	1	12	2	2	1	0	3	2	1	2
9020	202	0	13-Oct-16	14	1	12	2	1	1	0	6	2	2	1
9021	202	0	18-Aug-09	21	1	12	2	3	1	0	6	2	2	2
9022	202	0	24-May-08	15	1	12	2	1	1	0	6	2	2	1
9023	202	0	9-Nov-07	20	1	12	2	1	1	0	6	2	1	1
9024	202	0	9-Nov-17	9	1	12	2	1	1	0	6	2	5	1
9025	202	0	5-Aug-17	16	1	12	2	2	1	0	6	2	1	2
9026	202	0	12-Dec-07	10	1	12	2	1	1	0	13	2	1	1
9027	202	0	8-Jan-13	13	2	12	2	1	1	0	1	2	1	1
9028	202	0	5-Jul-17	10	2	12	2	1	1	0	4	2	2	2
9029	202	0	21-Oct-08	15	2	12	2	1	1	0	13	2	1	1
9030	202	0	5-Dec-17	16	2	12	2	2	1	0	13	2	1	1
9031	202	0	12-Jul-16	19	2	12	2	1	1	0	4	2	3	1
9032	202	0	23-Dec-13	15	2	12	2	1	1	0	4	2	1	1
9033	202	0	14-Nov-07	20	2	12	2	1	1	0	4	2	2	2
9034	202	0	19-Nov-07	11	2	12	2	1	1	0	4	2	1	2
9035	202	0	12-Apr-07	15	2	12	2	3	1	0	4	2	1	1
9036	202	0	23-Apr-16	17	2	12	2	1	1	0	11	2	2	1
9037	202	0	6-May-16	6	2	12	2	1	1	0	11	2	2	1
9038	202	0	9-Aug-08	20	2	12	2	3	1	0	11	2	3	1
9039	202	0	4-Oct-17	17	2	12	2	2	1	0	11	2	2	1
9040	202	0	22-Apr-17	19	2	12	2	1	1	0	11	2	2	1
9041	202	0	28-Sep-16	18	2	12	2	2	1	0	3	2	2	1
9042	202	0	7-Oct-15	22	2	12	2	3	1	0	3	2	3	1
9043	202	0	9-Oct-13	19	2	12	2	1	1	0	3	2	3	1
9044	202	0	13-Dec-12	15	2	12	2	1	1	0	3	2	2	1
9045	202	0	20-Aug-11	14	2	12	2	1	1	0	3	2	4	1

9046	202	0	20-May-10	7	2	12	2	1	1	0	3	2	4	2
9047	202	0	21-Aug-10	16	2	12	2	1	1	0	3	2	3	1
9048	202	0	8-Oct-08	17	2	12	2	1	1	0	3	2	2	1
9049	202	0	8-Nov-07	10	2	12	2	1	1	0	3	2	2	1
9050	202	0	24-Sep-17	18	2	12	2	1	1	0	3	2	2	1
9051	202	0	17-Oct-17	19	2	12	2	2	1	0	3	2	1	1
9052	202	0	26-Nov-17	18	2	12	2	3	1	0	3	2	2	1
9053	101	1	10-Jun-17	10	1	12	2	1	1	0	1	2	5	1
9054	101	1	19-Mar-12	21	1	12	2	1	1	0	1	2	3	1
9055	201	1	11-Jun-14	15	1	12	2	1	1	0	4	2	3	1
9056	201	1	13-Mar-12	16	1	12	2	2	1	0	3	2	3	1
9057	201	1	25-Oct-13	16	1	12	2	1	1	0	6	2	2	1
9058	201	1	10-Mar-16	11	1	12	2	2	1	0	6	2	2	1
9059	101	1	12-Oct-16	16	1	12	2	1	1	0	4	2	2	1
9060	101	1	29-Nov-12	19	1	12	2	1	1	0	7	2	3	1
9061	101	1	29-Nov-12	18	1	12	2	1	1	0	7	2	3	1
9062	101	1	27-Aug-11	16	1	12	2	1	1	0	7	2	5	1
9063	101	1	19-Nov-14	15	1	12	2	1	1	0	13	2	1	1
9064	201	1	30-Dec-10	18	1	12	2	2	1	0	13	2	4	1
9065	201	1	18-Nov-09	11	1	12	2	2	1	0	13	2	3	1
9066	201	1	27-Sep-08	15	1	12	2	2	1	0	1	2	4	1
9067	101	1	2-Jun-06	15	1	12	2	1	1	0	1	2	3	1
9068	101	1	26-Jun-11	17	1	12	2	1	1	0	1	2	1	1
9069	101	1	19-Dec-06	20	1	12	2	1	1	0	1	2	3	1
9070	101	1	24-Sep-06	15	1	12	2	1	1	0	1	2	1	1
9071	101	1	20-Mar-15	16	1	12	2	1	1	0	4	2	2	4
9072	101	1	17-Nov-15	13	1	12	2	1	1	0	4	2	2	1
9073	201	1	12-Apr-15	10	1	12	2	1	1	0	4	2	2	1
9074	101	1	23-Oct-14	17	1	12	2	1	1	0	4	2	3	1
9075	101	1	23-Jan-14	18	1	12	2	1	1	0	4	2	1	1
9076	101	1	21-Sep-14	21	1	12	2	2	1	0	4	2	3	1
9077	101	1	29-Mar-14	11	1	12	2	1	1	0	4	2	2	1
9078	101	1	16-Jan-13	13	1	12	2	1	1	0	4	2	2	1
9079	101	1	15-May-12	11	1	12	2	1	1	0	4	2	2	1
9080	101	1	11-Jun-12	10	1	12	2	1	1	0	4	2	2	1
9081	101	1	14-Sep-12	8	1	12	2	1	1	0	4	2	4	2
9082	101	1	18-Dec-12	17	1	12	2	1	1	0	4	2	1	1
9083	101	1	9-Dec-11	8	1	12	2	1	1	0	4	2	4	1
9084	101	1	1-Dec-11	11	1	12	2	1	1	0	4	2	4	1
9085	101	1	14-Jun-11	16	1	12	2	1	1	0	4	2	3	1
9086	101	1	8-Dec-11	10	1	12	2	1	1	0	4	2	1	1
9087	101	1	5-Aug-11	22	1	12	2	3	1	0	4	2	2	1
9088	101	1	2-Jun-11	18	1	12	2	1	1	0	4	2	2	1
9089	201	1	23-Jun-11	20	1	12	2	3	1	0	4	2	4	1
9090	101	1	22-Nov-10	17	1	12	2	1	1	0	4	2	4	1
9091	201	1	17-May-10	14	1	12	2	1	1	0	4	2	2	1
9092	101	1	10-Sep-10	11	1	12	2	1	1	0	4	2	2	1
9093	101	1	15-Sep-10	16	1	12	2	1	1	0	4	2	2	1
9094	101	1	3-Jun-10	16	1	12	2	1	1	0	4	2	2	1
9095	101	1	13-Sep-10	17	1	12	2	1	1	0	4	2	1	1
9096	101	1	9-Feb-08	15	1	12	2	3	1	0	4	2	4	1
9097	101	1	13-Nov-08	12	1	12	2	1	1	0	4	2	3	1
9098	201	1	18-Aug-08	17	1	12	2	3	1	0	4	2	3	1
9099	101	1	14-Jan-08	17	1	12	2	1	1	0	4	2	2	1
9100	101	1	26-Sep-07	10	1	12	2	1	1	0	4	2	1	1
9101	101	1	8-Apr-07	14	1	12	2	1	1	0	4	2	3	1
9102	101	1	15-Dec-07	21	1	12	2	1	1	0	4	2	2	1
9103	201	1	23-Sep-06	11	1	12	2	1	1	0	4	2	3	1
9104	101	1	28-Mar-17	10	1	12	2	1	1	0	4	2	7	1
9105	101	1	27-Mar-17	12	1	12	2	1	1	0	4	2	2	1
9106	101	1	16-May-17	10	1	12	2	1	1	0	4	2	2	1
9107	101	1	24-Oct-15	18	1	12	2	1	1	0	11	2	2	1
9108	101	1	15-Nov-14	19	1	12	2	1	1	0	11	2	2	1
9109	101	1	27-Jun-13	17	1	12	2	1	1	0	11	2	2	1
9110	101	1	21-Jun-12	17	1	12	2	1	1	0	11	2	2	1
9111	201	1	21-Jun-12	7	1	12	2	1	1	0	11	2	2	1
9112	101	1	5-Mar-12	12	1	12	2	1	1	0	11	2	2	1
9113	101	1	26-Sep-12	21	1	12	2	1	1	0	11	2	3	1
9114	101	1	8-Aug-12	11	1	12	2	1	1	0	11	2	2	1
9115	201	1	9-Oct-11	14	1	12	2	3	1	0	11	2	2	1
9116	201	1	17-Apr-11	19	1	12	2	3	1	0	11	2	3	1
9117	201	1	8-Jul-10	15	1	12	2	1	1	0	11	2	3	1
9118	201	1	18-Jul-09	8	1	12	2	2	1	0	11	2	3	1
9119	101	1	18-Apr-09	17	1	12	2	1	1	0	11	2	5	1
9120	201	1	11-Mar-09	14	1	12	2	3	1	0	11	2	2	1
9121	101	1	11-Apr-07	9	1	12	2	1	1	0	11	2	2	1
9122	201	1	30-Nov-17	17	1	12	2	1	1	0	11	2	2	1
9123	101	1	2-Sep-16	8	1	12	2	1	1	0	3	2	3	1
9124	101	1	9-Oct-16	17	1	12	2	1	1	0	3	2	2	2
9125	101	1	13-Dec-16	18	1	12	2	1	1	0	3	2	3	1
9126	101	1	30-Oct-16	16	1	12	2	1	1	0	3	2	3	1
9127	201	1	3-Nov-16	19	1	12	2	1	1	0	3	2	4	1
9128	101	1	23-Jul-16	13	1	12	2	1	1	0	3	2	1	1
9129	101	1	12-Dec-16	12	1	12	2	1	1	0	3	2	4	1
9130	201	1	3-Nov-16	12	1	12	2	2	1	0	3	2	4	1
9131	101	1	25-Oct-16	21	1	12	2	1	1	0	3	2	2	1
9132	101	1	12-Dec-16	11	1	12	2	1	1	0	3	2	1	1
9133	201	1	28-Oct-16	16	1	12	2	1	1	0	3	2	1	1
9134	101	1	7-Jan-16	11	1	12	2	1	1	0	3	2	4	1
9135	101	1	21-Sep-16	11	1	12	2	1	1	0	3	2	2	1
9136	101	1	26-Sep-16	13	1	12	2	1	1	0	3	2	3	1
9137	201	1	25-Aug-16	18	1	12	2	1	1	0	3	2	2	1
9138	101	1	25-Sep-16	18	1	12	2	1	1	0	3	2	2	1
9139	101	1	3-Oct-16	21	1	12	2	1	1	0	3	2	2	2
9140	201	1	28-Apr-16	11	1	12	2	2	1	0	3	2	2	1
9141	201	1	18-Jun-16	19	1	12	2	1	1	0	3	2	3	2
9142	101	1	10-Jun-15	10	1	12	2	1	1	0	3	2	2	1
9143	101	1	12-Apr-15	16	1	12	2	1	1	0	3	2	4	1
9144	201	1	26-Nov-15	10	1	12	2	2	1	0	3	2	5	1
9145	201	1	9-Oct-15	17	1	12	2	1	1	0	3	2	3	1
9146	101	1	14-Dec-15	20	1	12	2	1	1	0	3	2	2	1
9147	101	1	21-Mar-15	19	1	12	2	1	1	0	3	2	1	1
9148	101	1	5-Jan-15	16	1	12	2	1	1	0	3	2	5	1
9149	101	1	30-Jan-15	22	1	12	2	1	1	0	3	2	4	1
9150	101	1	29-Jan-15	19	1	12	2	1	1	0	3	2	2	1
9151	201	1	9-Nov-15	17	1	12	2	2	1	0	3	2	3	1
9152	201	1	29-May-15	8	1	12	2	3	1	0	3	2	2	1
9153	101	1	3-Dec-15	15	1	12	2	1	1	0	3	2	1	1
9154	101	1	7-Feb-15	16	1	12	2	1	1	0	3	2	2	1
9155	101	1	11-Dec-15	10	1	12	2	1	1	0	3	2	3	1
9156	201	1	25-Oct-15	8	1	12	2	1	1	0	3	2	3	1
9157	101	1	30-May-15	9	1	12	2	1	1	0	3	2	2	1
9158	201	1	24-Nov-15	11	1	12	2	2	1	0	3	2	4	2
9159	101	1	8-Jan-15	16	1	12	2	1	1	0	3	2	2	1
9160	101	1	30-Dec-15	11	1	12	2	1	1	0	3	2	4	1
9161	101	1	29-Sep-15	11	1	12	2	1	1	0	3	2	2	1
9162	201	1	18-Oct-14	15	1	12	2	2	1	0	3	2	2	1
9163	101	1	20-Apr-14	15	1	12	2	1	1	0	3	2	1	1
9164	201	1	24-May-14	19	1	12	2	3	1	0	3	2	1	1
9165	201	1	30-Jul-14	14	1	12	2	1	1	0	3	2	1	1
9166	101	1	23-Sep-14	12	1	12	2	3	1	0	3	2	2	1
9167	201	1	17-Apr-14	17	1	12	2	3	1	0	3	2	2	1
9168	101	1	20-Jan-14	18	1	12	2	1	1	0	3	2	2	1
9169	101	1	28-Sep-14	16	1	12	2	1	1	0	3	2	2	1

9170	201	1	1-Nov-14	17	1	12	2	1	1	0	3	2	3	1
9171	101	1	29-Nov-14	13	1	12	2	1	1	0	3	2	5	1
9172	201	1	11-Dec-14	12	1	12	2	2	1	0	3	2	5	1
9173	201	1	16-Jan-14	12	1	12	2	2	1	0	3	2	1	1
9174	201	1	12-Jan-14	9	1	12	2	2	1	0	3	2	1	1
9175	101	1	4-Jun-14	20	1	12	2	1	1	0	3	2	1	1
9176	101	1	27-Feb-14	11	1	12	2	1	1	0	3	2	2	1
9177	101	1	10-Feb-14	21	1	12	2	1	1	0	3	2	2	1
9178	101	1	10-Sep-14	17	1	12	2	1	1	0	3	2	4	1
9179	101	1	18-Nov-14	17	1	12	2	1	1	0	3	2	2	1
9180	101	1	23-Jan-14	21	1	12	2	1	1	0	3	2	2	1
9181	101	1	3-Apr-14	9	1	12	2	1	1	0	3	2	3	1
9182	101	1	2-Apr-14	20	1	12	2	1	1	0	3	2	3	1
9183	201	1	16-Jan-14	9	1	12	2	1	1	0	3	2	1	1
9184	101	1	4-Jun-14	18	1	12	2	1	1	0	3	2	1	1
9185	101	1	26-Sep-14	23	1	12	2	3	1	0	3	2	1	1
9186	101	1	25-Feb-14	18	1	12	2	1	1	0	3	2	2	1
9187	201	1	12-Nov-14	19	1	12	2	3	1	0	3	2	3	1
9188	101	1	24-Mar-14	16	1	12	2	1	1	0	3	2	2	1
9189	101	1	24-Oct-14	19	1	12	2	1	1	0	3	2	2	1
9190	101	1	9-Jun-14	17	1	12	2	1	1	0	3	2	3	1
9191	201	1	7-Jan-14	18	1	12	2	1	1	0	3	2	3	1
9192	201	1	25-Mar-14	17	1	12	2	3	1	0	3	2	2	1
9193	201	1	7-Apr-14	9	1	12	2	2	1	0	3	2	2	1
9194	101	1	16-May-13	10	1	12	2	1	1	0	3	2	1	2
9195	101	1	25-Oct-13	11	1	12	2	1	1	0	3	2	1	1
9196	101	1	7-Jan-13	17	1	12	2	3	1	0	3	2	1	1
9197	101	1	1-Jul-13	12	1	12	2	1	1	0	3	2	1	1
9198	201	1	13-May-13	18	1	12	2	2	1	0	3	2	2	2
9199	201	1	7-Nov-13	17	1	12	2	2	1	0	3	2	2	1
9200	201	1	21-Oct-13	15	1	12	2	3	1	0	3	2	2	1
9201	101	1	12-Jun-13	18	1	12	2	1	1	0	3	2	1	1
9202	101	1	28-Sep-13	20	1	12	2	1	1	0	3	2	2	1
9203	101	1	30-Apr-13	18	1	12	2	1	1	0	3	2	2	1
9204	101	1	10-Jan-13	17	1	12	2	1	1	0	3	2	3	1
9205	201	1	24-Apr-13	19	1	12	2	1	1	0	3	2	1	1
9206	101	1	23-Mar-13	23	1	12	2	1	1	0	3	2	1	1
9207	101	1	27-Nov-13	11	1	12	2	1	1	0	3	2	1	1
9208	201	1	1-Oct-13	9	1	12	2	2	1	0	3	2	1	1
9209	201	1	24-Sep-13	19	1	12	2	2	1	0	3	2	1	1
9210	101	1	9-Mar-13	20	1	12	2	1	1	0	3	2	1	1
9211	101	1	22-Mar-13	10	1	12	2	1	1	0	3	2	2	1
9212	101	1	9-Apr-13	16	1	12	2	1	1	0	3	2	2	4
9213	101	1	24-Mar-13	17	1	12	2	1	1	0	3	2	2	2
9214	101	1	12-Jul-13	10	1	12	2	1	1	0	3	2	4	1
9215	101	1	12-Dec-13	18	1	12	2	1	1	0	3	2	2	1
9216	101	1	7-Oct-13	7	1	12	2	1	1	0	3	2	3	1
9217	101	1	24-Nov-13	16	1	12	2	1	1	0	3	2	3	1
9218	101	1	23-Mar-13	9	1	12	2	1	1	0	3	2	3	2
9219	101	1	2-Feb-13	16	1	12	2	1	1	0	3	2	2	4
9220	101	1	15-May-13	18	1	12	2	1	1	0	3	2	3	1
9221	101	1	19-Jan-13	10	1	12	2	1	1	0	3	2	2	4
9222	101	1	9-Jul-13	18	1	12	2	1	1	0	3	2	3	4
9223	101	1	10-Sep-13	17	1	12	2	1	1	0	3	2	2	1
9224	101	1	2-Jul-13	16	1	12	2	1	1	0	3	2	2	1
9225	101	1	13-Apr-13	10	1	12	2	1	1	0	3	2	2	1
9226	101	1	7-Feb-13	15	1	12	2	1	1	0	3	2	2	1
9227	201	1	13-Mar-13	14	1	12	2	3	1	0	3	2	3	1
9228	101	1	20-Feb-13	17	1	12	2	1	1	0	3	2	4	1
9229	101	1	11-Feb-13	11	1	12	2	1	1	0	3	2	3	1
9230	201	1	21-Jun-13	15	1	12	2	3	1	0	3	2	3	1
9231	101	1	11-Sep-13	9	1	12	2	1	1	0	3	2	1	1
9232	101	1	3-Mar-13	19	1	12	2	1	1	0	3	2	2	1
9233	101	1	17-Mar-13	12	1	12	2	1	1	0	3	2	3	1
9234	201	1	21-Aug-13	16	1	12	2	1	1	0	3	2	2	1
9235	201	1	9-Dec-13	14	1	12	2	3	1	0	3	2	3	1
9236	101	1	26-Oct-13	12	1	12	2	1	1	0	3	2	3	1
9237	201	1	20-Mar-13	11	1	12	2	2	1	0	3	2	3	1
9238	201	1	23-Oct-13	11	1	12	2	2	1	0	3	2	2	1
9239	101	1	14-May-12	14	1	12	2	1	1	0	3	2	1	1
9240	201	1	25-Oct-12	17	1	12	2	3	1	0	3	2	1	1
9241	101	1	29-May-12	8	1	12	2	1	1	0	3	2	1	1
9242	101	1	27-Apr-12	10	1	12	2	1	1	0	3	2	1	1
9243	101	1	8-Nov-12	17	1	12	2	1	1	0	3	2	1	1
9244	101	1	6-Dec-12	9	1	12	2	1	1	0	3	2	1	1
9245	101	1	13-Sep-12	12	1	12	2	1	1	0	3	2	3	1
9246	101	1	9-Nov-12	19	1	12	2	1	1	0	3	2	3	1
9247	101	1	6-Apr-12	10	1	12	2	1	1	0	3	2	6	1
9248	101	1	21-Nov-12	11	1	12	2	1	1	0	3	2	3	1
9249	201	1	28-Dec-12	18	1	12	2	2	1	0	3	2	3	1
9250	201	1	25-May-12	16	1	12	2	3	1	0	3	2	5	1
9251	101	1	5-Nov-12	17	1	12	2	1	1	0	3	2	4	1
9252	101	1	24-Apr-12	13	1	12	2	1	1	0	3	2	1	1
9253	101	1	14-Sep-12	16	1	12	2	1	1	0	3	2	1	1
9254	101	1	1-Dec-12	17	1	12	2	1	1	0	3	2	1	1
9255	101	1	10-Nov-12	10	1	12	2	1	1	0	3	2	1	1
9256	101	1	17-Mar-12	10	1	12	2	1	1	0	3	2	7	4
9257	101	1	2-Nov-12	18	1	12	2	1	1	0	3	2	2	4
9258	101	1	15-Jun-12	17	1	12	2	1	1	0	3	2	1	1
9259	101	1	23-Dec-12	14	1	12	2	1	1	0	3	2	4	1
9260	101	1	2-Jul-12	18	1	12	2	1	1	0	3	2	3	1
9261	101	1	20-Dec-12	17	1	12	2	1	1	0	3	2	2	1
9262	101	1	30-Mar-12	10	1	12	2	1	1	0	3	2	3	1
9263	101	1	21-Nov-12	12	1	12	2	1	1	0	3	2	3	1
9264	101	1	5-Mar-12	21	1	12	2	1	1	0	3	2	2	1
9265	101	1	25-Sep-12	16	1	12	2	1	1	0	3	2	2	1
9266	101	1	1-Apr-12	20	1	12	2	1	1	0	3	2	3	1
9267	101	1	10-May-12	9	1	12	2	1	1	0	3	2	2	1
9268	101	1	29-Nov-12	16	1	12	2	1	1	0	3	2	2	1
9269	101	1	24-Dec-12	17	1	12	2	1	1	0	3	2	2	1
9270	101	1	19-Feb-12	18	1	12	2	1	1	0	3	2	2	1
9271	101	1	4-May-12	20	1	12	2	1	1	0	3	2	2	1
9272	101	1	16-Dec-12	7	1	12	2	1	1	0	3	2	2	1
9273	101	1	28-Dec-12	17	1	12	2	1	1	0	3	2	2	1
9274	101	1	21-Sep-12	7	1	12	2	1	1	0	3	2	3	1
9275	101	1	26-Jul-12	8	1	12	2	1	1	0	3	2	3	1
9276	101	1	20-Jun-12	16	1	12	2	1	1	0	3	2	3	1
9277	201	1	5-Nov-12	14	1	12	2	2	1	0	3	2	4	1
9278	101	1	21-Jun-12	17	1	12	2	1	1	0	3	2	3	1
9279	101	1	23-Nov-12	17	1	12	2	1	1	0	3	2	3	1
9280	101	1	10-Sep-12	17	1	12	2	1	1	0	3	2	3	1
9281	201	1	14-Sep-12	18	1	12	2	2	1	0	3	2	1	1
9282	101	1	17-May-12	15	1	12	2	2	1	0	3	2	2	1
9283	101	1	29-Sep-12	18	1	12	2	1	1	0	3	2	3	1
9284	201	1	27-Jun-12	18	1	12	2	2	1	0	3	2	3	1
9285	101	1	6-Dec-12	11	1	12	2	1	1	0	3	2	3	4
9286	101	1	2-Mar-12	18	1	12	2	1	1	0	3	2	2	4
9287	201	1	30-Nov-12	12	1	12	2	1	1	0	3	2	2	1
9288	101	1	18-May-12	17	1	12	2	1	1	0	3	2	1	1
9289	101	1	23-Aug-11	22	1	12	2	1	1	0	3	2	2	1
9290	101	1	10-Oct-11	16	1	12	2	1	1	0	3	2	3	1
9291	101	1	16-Nov-11	16	1	12	2	1	1	0	3	2	2	4
9292	201	1	18-Jul-11	15	1	12	2	1	1	0	3	2	2	1
9293	101	1	3-Mar-11	10	1	12	2	1	1	0	3	2	2	1

9294	201	1	31-Mar-11	16	1	12	2	1	1	0	3	2	4	1
9295	101	1	26-Jan-11	19	1	12	2	2	1	0	3	2	1	1
9296	101	1	15-Nov-11	14	1	12	2	1	1	0	3	2	1	1
9297	101	1	10-Mar-11	17	1	12	2	1	1	0	3	2	2	1
9298	101	1	15-Sep-11	16	1	12	2	3	1	0	3	2	1	1
9299	101	1	13-Dec-11	16	1	12	2	1	1	0	3	2	1	1
9300	101	1	7-Apr-11	17	1	12	2	1	1	0	3	2	1	1
9301	101	1	19-Sep-11	20	1	12	2	1	1	0	3	2	2	1
9302	101	1	14-Mar-11	10	1	12	2	1	1	0	3	2	2	1
9303	101	1	10-Jan-11	10	1	12	2	1	1	0	3	2	2	1
9304	101	1	11-Oct-11	18	1	12	2	1	1	0	3	2	2	1
9305	101	1	1-Nov-11	21	1	12	2	1	1	0	3	2	3	1
9306	101	1	5-Nov-11	8	1	12	2	1	1	0	3	2	2	1
9307	101	1	21-Nov-11	18	1	12	2	1	1	0	3	2	1	1
9308	101	1	9-Oct-11	20	1	12	2	1	1	0	3	2	3	1
9309	101	1	9-Dec-11	19	1	12	2	1	1	0	3	2	2	1
9310	101	1	9-Oct-11	18	1	12	2	1	1	0	3	2	4	1
9311	101	1	17-Jun-11	16	1	12	2	1	1	0	3	2	3	1
9312	101	1	9-Sep-11	19	1	12	2	1	1	0	3	2	2	1
9313	201	1	16-Mar-11	19	1	12	2	1	1	0	3	2	1	1
9314	201	1	26-Sep-11	10	1	12	2	3	1	0	3	2	2	1
9315	101	1	23-Feb-11	10	1	12	2	1	1	0	3	2	2	1
9316	201	1	3-Nov-11	13	1	12	2	1	1	0	3	2	3	1
9317	101	1	29-Mar-10	11	1	12	2	1	1	0	3	2	1	2
9318	101	1	8-Dec-10	18	1	12	2	1	1	0	3	2	1	1
9319	101	1	18-Oct-10	17	1	12	2	1	1	0	3	2	2	1
9320	201	1	31-Mar-10	17	1	12	2	1	1	0	3	2	2	1
9321	101	1	13-Dec-10	11	1	12	2	1	1	0	3	2	2	1
9322	101	1	2-Apr-10	15	1	12	2	1	1	0	3	2	3	1
9323	101	1	28-May-10	17	1	12	2	1	1	0	3	2	3	1
9324	101	1	11-Aug-10	20	1	12	2	1	1	0	3	2	2	1
9325	101	1	24-Dec-10	16	1	12	2	1	1	0	3	2	4	1
9326	201	1	11-Oct-10	23	1	12	2	2	1	0	3	2	5	1
9327	101	1	30-Sep-10	19	1	12	2	1	1	0	3	2	4	1
9328	101	1	24-Jun-10	7	1	12	2	1	1	0	3	2	4	1
9329	101	1	8-Oct-10	16	1	12	2	1	1	0	3	2	2	1
9330	101	1	12-May-10	15	1	12	2	1	1	0	3	2	2	1
9331	101	1	2-Nov-10	12	1	12	2	1	1	0	3	2	2	1
9332	101	1	12-Dec-10	20	1	12	2	1	1	0	3	2	2	1
9333	101	1	19-Jun-10	12	1	12	2	1	1	0	3	2	2	1
9334	101	1	18-Nov-10	16	1	12	2	1	1	0	3	2	2	1
9335	101	1	24-May-10	16	1	12	2	1	1	0	3	2	3	1
9336	101	1	20-Sep-10	16	1	12	2	1	1	0	3	2	3	1
9337	101	1	7-May-10	18	1	12	2	1	1	0	3	2	3	1
9338	101	1	24-Dec-10	12	1	12	2	1	1	0	3	2	3	1
9339	101	1	19-Oct-10	16	1	12	2	1	1	0	3	2	3	1
9340	101	1	30-Apr-10	16	1	12	2	1	1	0	3	2	3	1
9341	101	1	1-Apr-10	17	1	12	2	1	1	0	3	2	3	1
9342	101	1	27-Oct-10	17	1	12	2	1	1	0	3	2	2	1
9343	201	1	22-Dec-09	12	1	12	2	2	1	0	3	2	4	1
9344	101	1	24-Apr-09	15	1	12	2	1	1	0	3	2	4	1
9345	101	1	20-Oct-09	16	1	12	2	1	1	0	3	2	2	1
9346	101	1	10-Dec-09	8	1	12	2	1	1	0	3	2	2	1
9347	101	1	9-Dec-09	8	1	12	2	1	1	0	3	2	2	1
9348	101	1	23-Dec-09	12	1	12	2	1	1	0	3	2	1	1
9349	201	1	15-Apr-09	12	1	12	2	1	1	0	3	2	2	2
9350	101	1	17-Aug-09	8	1	12	2	1	1	0	3	2	2	1
9351	101	1	27-Aug-09	11	1	12	2	1	1	0	3	2	1	1
9352	201	1	17-May-09	12	1	12	2	3	1	0	3	2	2	1
9353	101	1	30-Oct-09	12	1	12	2	1	1	0	3	2	2	1
9354	101	1	28-Oct-09	10	1	12	2	1	1	0	3	2	2	1
9355	201	1	1-Feb-09	19	1	12	2	1	1	0	3	2	2	1
9356	101	1	9-Mar-09	17	1	12	2	1	1	0	3	2	2	1
9357	101	1	4-Jun-09	17	1	12	2	1	1	0	3	2	3	1
9358	101	1	10-Oct-08	14	1	12	2	1	1	0	3	2	1	1
9359	101	1	5-Sep-08	14	1	12	2	1	1	0	3	2	1	1
9360	201	1	23-Dec-08	16	1	12	2	2	1	0	3	2	7	1
9361	101	1	3-Dec-08	16	1	12	2	1	1	0	3	2	2	1
9362	101	1	24-Mar-08	12	1	12	2	1	1	0	3	2	3	1
9363	101	1	9-Dec-08	12	1	12	2	1	1	0	3	2	1	1
9364	101	1	23-Sep-08	20	1	12	2	1	1	0	3	2	1	1
9365	101	1	31-Oct-08	17	1	12	2	1	1	0	3	2	1	1
9366	201	1	30-Apr-08	20	1	12	2	1	1	0	3	2	2	1
9367	101	1	3-Sep-08	20	1	12	2	1	1	0	3	2	3	1
9368	101	1	9-May-08	21	1	12	2	1	1	0	3	2	3	1
9369	201	1	9-Aug-08	23	1	12	2	3	1	0	3	2	2	2
9370	201	1	3-Aug-08	21	1	12	2	1	1	0	3	2	2	2
9371	101	1	5-Sep-08	14	1	12	2	1	1	0	3	2	2	1
9372	101	1	6-Sep-08	19	1	12	2	1	1	0	3	2	2	1
9373	101	1	1-Apr-08	17	1	12	2	1	1	0	3	2	2	4
9374	101	1	17-Nov-08	11	1	12	2	1	1	0	3	2	3	1
9375	201	1	3-Jan-08	17	1	12	2	1	1	0	3	2	2	1
9376	101	1	23-Dec-08	20	1	12	2	1	1	0	3	2	2	2
9377	101	1	3-Sep-08	12	1	12	2	1	1	0	3	2	2	1
9378	101	1	21-May-08	11	1	12	2	1	1	0	3	2	2	1
9379	101	1	13-Mar-08	20	1	12	2	1	1	0	3	2	3	2
9380	101	1	31-Oct-08	20	1	12	2	1	1	0	3	2	2	1
9381	101	1	24-Mar-08	16	1	12	2	1	1	0	3	2	2	1
9382	201	1	28-Oct-08	21	1	12	2	3	1	0	3	2	2	1
9383	101	1	6-Jun-07	12	1	12	2	1	1	0	3	2	2	2
9384	101	1	10-Dec-07	18	1	12	2	1	1	0	3	2	2	1
9385	101	1	10-Aug-07	10	1	12	2	1	1	0	3	2	3	1
9386	101	1	3-Jun-07	22	1	12	2	3	1	0	3	2	2	1
9387	201	1	29-May-07	19	1	12	2	1	1	0	3	2	3	1
9388	201	1	23-Aug-07	19	1	12	2	1	1	0	3	2	2	2
9389	101	1	13-Feb-07	20	1	12	2	1	1	0	3	2	4	1
9390	201	1	19-Apr-07	10	1	12	2	2	1	0	3	2	5	1
9391	101	1	19-Dec-07	17	1	12	2	1	1	0	3	2	2	1
9392	101	1	23-May-06	14	1	12	2	1	1	0	3	2	2	1
9393	101	1	31-Oct-06	15	1	12	2	1	1	0	3	2	1	1
9394	101	1	12-Jun-06	12	1	12	2	1	1	0	3	2	3	1
9395	101	1	31-Dec-06	9	1	12	2	1	1	0	3	2	1	1
9396	101	1	7-Sep-06	17	1	12	2	1	1	0	3	2	1	1
9397	101	1	19-Dec-06	18	1	12	2	1	1	0	3	2	2	1
9398	101	1	5-Dec-06	15	1	12	2	1	1	0	3	2	3	1
9399	101	1	5-Dec-06	9	1	12	2	1	1	0	3	2	3	1
9400	101	1	15-Aug-06	21	1	12	2	1	1	0	3	2	1	1
9401	101	1	25-Aug-06	21	1	12	2	1	1	0	3	2	3	1
9402	101	1	31-Jul-06	22	1	12	2	1	1	0	3	2	1	1
9403	101	1	11-Feb-06	16	1	12	2	2	1	0	3	2	3	1
9404	201	1	8-Dec-06	15	1	12	2	2	1	0	3	2	2	1
9405	101	1	8-Nov-06	16	1	12	2	1	1	0	3	2	3	1
9406	101	1	13-Nov-06	10	1	12	2	1	1	0	3	2	2	1
9407	101	1	20-Oct-06	17	1	12	2	1	1	0	3	2	2	1
9408	201	1	13-Sep-17	18	1	12	2	1	1	0	3	2	5	1
9409	101	1	19-May-17	21	1	12	2	1	1	0	3	2	4	1
9410	101	1	7-Mar-17	15	1	12	2	1	1	0	3	2	3	1
9411	101	1	27-Jun-17	17	1	12	2	1	1	0	3	2	3	1
9412	201	1	26-Apr-17	8	1	12	2	1	1	0	3	2	3	1
9413	101	1	7-Apr-17	8	1	12	2	1	1	0	3	2	2	1
9414	101	1	19-May-17	21	1	12	2	1	1	0	3	2	2	1
9415	101	1	18-Apr-17	17	1	12	2	1	1	0	3	2	2	1
9416	101	1	19-May-17	10	1	12	2	1	1	0	3	2	2	1
9417	101	1	5-Jan-17	15	1	12	2	1	1	0	3	2	2	1

9418	101	1	14-Aug-17	21	1	12	2	1	1	0	3	2	1	1
9419	101	1	19-Sep-17	9	1	12	2	1	1	0	3	2	1	1
9420	101	1	29-Nov-17	16	1	12	2	1	1	0	3	2	1	1
9421	201	1	9-Nov-17	11	1	12	2	1	1	0	3	2	1	1
9422	201	1	10-Apr-17	16	1	12	2	1	1	0	3	2	3	2
9423	201	1	23-Jun-17	18	1	12	2	2	1	0	3	2	3	1
9424	201	1	16-Jun-17	20	1	12	2	2	1	0	3	2	2	1
9425	101	1	19-Aug-17	13	1	12	2	1	1	0	3	2	4	1
9426	101	1	10-Oct-17	17	1	12	2	1	1	0	3	2	4	1
9427	101	1	15-Sep-17	18	1	12	2	1	1	0	3	2	2	1
9428	101	1	20-Sep-17	10	1	12	2	1	1	0	3	2	1	1
9429	101	1	9-Nov-17	17	1	12	2	1	1	0	3	2	1	1
9430	101	1	19-Mar-15	10	1	12	2	1	1	0	6	2	2	1
9431	101	1	1-Jul-14	17	1	12	2	1	1	0	6	2	2	1
9432	101	1	27-Dec-13	17	1	12	2	1	1	0	6	2	1	1
9433	101	1	29-Oct-12	14	1	12	2	1	1	0	6	2	2	1
9434	101	1	18-May-12	12	1	12	2	1	1	0	6	2	2	1
9435	101	1	21-Oct-12	15	1	12	2	1	1	0	6	2	3	1
9436	101	1	3-Mar-11	21	1	12	2	1	1	0	6	2	2	1
9437	101	1	29-Jun-11	17	1	12	2	1	1	0	6	2	3	1
9438	101	1	24-Dec-09	15	1	12	2	1	1	0	6	2	2	1
9439	101	1	27-Mar-07	10	1	12	2	1	1	0	6	2	2	1
9440	101	1	28-Sep-06	11	1	12	2	1	1	0	6	2	2	1
9441	101	1	30-Jan-17	15	1	12	2	1	1	0	6	2	3	1
9442	101	1	31-Jan-17	21	1	12	2	1	1	0	6	2	2	1
9443	101	1	23-Aug-17	20	1	12	2	1	1	0	6	2	3	1
9444	101	1	9-Nov-16	12	1	12	2	1	1	0	13	2	2	1
9445	101	1	1-Jun-12	16	1	12	2	1	1	0	13	2	3	1
9446	201	1	8-Mar-11	19	1	12	2	1	1	0	13	2	1	1
9447	101	1	3-Jun-12	13	2	12	2	1	1	0	1	2	2	1
9448	201	1	5-Oct-12	17	2	12	2	1	1	0	3	2	3	1
9449	101	1	2-Aug-09	8	2	12	2	1	1	0	7	2	2	1
9450	201	1	25-Dec-08	12	2	12	2	1	1	0	13	2	1	1
9451	201	1	4-Sep-10	14	2	12	2	1	1	0	1	2	1	1
9452	201	1	9-Jun-11	18	2	12	2	1	1	0	4	2	2	1
9453	101	1	4-Jun-10	10	2	12	2	1	1	0	4	2	1	1
9454	101	1	27-May-08	20	2	12	2	1	1	0	4	2	2	1
9455	101	1	9-May-08	10	2	12	2	1	1	0	4	2	1	1
9456	201	1	7-Aug-08	18	2	12	2	2	1	0	4	2	3	1
9457	101	1	12-Jan-07	17	2	12	2	1	1	0	4	2	2	1
9458	101	1	12-Apr-07	15	2	12	2	1	1	0	4	2	1	1
9459	101	1	5-Jul-07	19	2	12	2	1	1	0	4	2	2	1
9460	101	1	10-Mar-06	19	2	12	2	1	1	0	4	2	2	1
9461	101	1	25-Oct-12	10	2	12	2	1	1	0	11	2	2	1
9462	101	1	1-Feb-12	8	2	12	2	1	1	0	11	2	3	1
9463	201	1	31-Dec-12	16	2	12	2	1	1	0	11	2	2	1
9464	101	1	31-Oct-11	17	2	12	2	1	1	0	11	2	2	1
9465	101	1	22-Oct-11	17	2	12	2	1	1	0	11	2	2	1
9466	101	1	17-Oct-11	12	2	12	2	1	1	0	11	2	2	1
9467	101	1	25-Oct-11	18	2	12	2	1	1	0	11	2	2	1
9468	101	1	5-Mar-10	20	2	12	2	1	1	0	11	2	4	1
9469	101	1	27-Aug-10	10	2	12	2	1	1	0	11	2	3	1
9470	201	1	25-Feb-09	18	2	12	2	1	1	0	11	2	2	1
9471	101	1	21-Oct-17	10	2	12	2	1	1	0	11	2	2	1
9472	101	1	11-Sep-17	19	2	12	2	1	1	0	11	2	2	1
9473	101	1	20-Oct-17	19	2	12	2	1	1	0	11	2	1	1
9474	101	1	12-Dec-16	15	2	12	2	1	1	0	3	2	1	1
9475	101	1	4-Nov-16	15	2	12	2	1	1	0	3	2	3	1
9476	101	1	22-Dec-15	21	2	12	2	1	1	0	3	2	1	2
9477	101	1	6-Jan-15	17	2	12	2	1	1	0	3	2	4	1
9478	101	1	12-Jan-15	17	2	12	2	1	1	0	3	2	1	1
9479	101	1	26-Apr-15	21	2	12	2	1	1	0	3	2	4	1
9480	101	1	14-Jan-15	12	2	12	2	1	1	0	3	2	4	1
9481	101	1	15-Nov-15	16	2	12	2	1	1	0	3	2	2	1
9482	101	1	14-Sep-15	17	2	12	2	1	1	0	3	2	5	1
9483	101	1	30-Nov-15	20	2	12	2	1	1	0	3	2	2	1
9484	101	1	23-Oct-15	17	2	12	2	1	1	0	3	2	1	1
9485	101	1	14-Nov-15	14	2	12	2	1	1	0	3	2	2	1
9486	201	1	1-Jun-15	11	2	12	2	3	1	0	3	2	2	1
9487	101	1	25-Jun-14	16	2	12	2	1	1	0	3	2	5	1
9488	201	1	22-Oct-14	20	2	12	2	2	1	0	3	2	3	1
9489	101	1	24-Sep-14	20	2	12	2	1	1	0	3	2	2	1
9490	101	1	15-Nov-14	8	2	12	2	1	1	0	3	2	3	1
9491	101	1	27-Nov-14	18	2	12	2	1	1	0	3	2	3	1
9492	101	1	9-Dec-14	16	2	12	2	1	1	0	3	2	3	1
9493	201	1	16-Mar-13	23	2	12	2	1	1	0	3	2	2	1
9494	101	1	19-Mar-13	12	2	12	2	1	1	0	3	2	3	1
9495	101	1	27-Mar-13	9	2	12	2	1	1	0	3	2	3	1
9496	201	1	30-Aug-13	11	2	12	2	3	1	0	3	2	5	1
9497	101	1	3-Sep-13	12	2	12	2	1	1	0	3	2	4	1
9498	101	1	8-Jun-13	21	2	12	2	1	1	0	3	2	2	1
9499	101	1	11-Mar-13	11	2	12	2	1	1	0	3	2	3	1
9500	101	1	20-Sep-13	8	2	12	2	1	1	0	3	2	2	1
9501	101	1	25-Apr-13	16	2	12	2	1	1	0	3	2	3	1
9502	101	1	13-Apr-13	7	2	12	2	1	1	0	3	2	3	1
9503	101	1	18-May-13	16	2	12	2	1	1	0	3	2	3	1
9504	101	1	30-Jan-13	12	2	12	2	1	1	0	3	2	3	1
9505	101	1	5-Nov-13	15	2	12	2	1	1	0	3	2	2	1
9506	101	1	3-Jun-13	17	2	12	2	1	1	0	3	2	2	1
9507	101	1	29-Mar-13	16	2	12	2	1	1	0	3	2	3	1
9508	101	1	22-Oct-12	11	2	12	2	1	1	0	3	2	1	1
9509	101	1	16-Dec-12	17	2	12	2	1	1	0	3	2	3	1
9510	101	1	28-Jul-12	17	2	12	2	1	1	0	3	2	5	1
9511	101	1	10-Nov-12	12	2	12	2	1	1	0	3	2	1	1
9512	101	1	27-Oct-12	21	2	12	2	1	1	0	3	2	2	1
9513	101	1	22-May-12	11	2	12	2	1	1	0	3	2	2	1
9514	101	1	29-Dec-12	17	2	12	2	1	1	0	3	2	3	1
9515	101	1	14-Dec-12	16	2	12	2	1	1	0	3	2	2	2
9516	101	1	21-Nov-12	16	2	12	2	1	1	0	3	2	3	1
9517	101	1	6-Feb-12	0	2	12	2	1	1	0	3	2	2	1
9518	101	1	11-Oct-11	13	2	12	2	1	1	0	3	2	1	1
9519	101	1	21-Sep-11	6	2	12	2	1	1	0	3	2	1	1
9520	201	1	9-Jun-11	11	2	12	2	2	1	0	3	2	2	1
9521	101	1	24-Jul-11	19	2	12	2	1	1	0	3	2	1	1
9522	201	1	7-Jan-11	10	2	12	2	2	1	0	3	2	2	1
9523	101	1	10-May-11	7	2	12	2	1	1	0	3	2	3	1
9524	101	1	31-Jan-11	21	2	12	2	1	1	0	3	2	2	1
9525	101	1	27-Oct-11	10	2	12	2	1	1	0	3	2	2	1
9526	101	1	21-Dec-11	18	2	12	2	1	1	0	3	2	3	1
9527	201	1	8-Feb-11	11	2	12	2	3	1	0	3	2	4	1
9528	101	1	12-Jun-11	17	2	12	2	1	1	0	3	2	3	1
9529	101	1	19-Mar-10	12	2	12	2	1	1	0	3	2	2	1
9530	201	1	12-Aug-10	10	2	12	2	2	1	0	3	2	2	1
9531	101	1	25-Mar-10	16	2	12	2	1	1	0	3	2	4	1
9532	101	1	21-Jan-10	17	2	12	2	1	1	0	3	2	2	4
9533	101	1	11-Mar-10	8	2	12	2	1	1	0	3	2	3	1
9534	101	1	16-Dec-09	12	2	12	2	1	1	0	3	2	1	1
9535	101	1	10-Nov-09	16	2	12	2	1	1	0	3	2	1	1
9536	101	1	6-Sep-09	22	2	12	2	1	1	0	3	2	4	1
9537	101	1	2-Aug-09	17	2	12	2	1	1	0	3	2	1	1
9538	201	1	24-Nov-09	14	2	12	2	1	1	0	3	2	3	1
9539	101	1	28-Sep-08	21	2	12	2	2	1	0	3	2	1	1
9540	201	1	26-Mar-08	19	2	12	2	1	1	0	3	2	2	1
9541	201	1	29-Oct-08	17	2	12	2	3	1	0	3	2	5	2

9542	101	1	28-Oct-08	17	2	12	2	1	1	0	3	2	2	1
9543	101	1	6-Dec-08	22	2	12	2	2	1	0	3	2	2	1
9544	101	1	3-Apr-07	16	2	12	2	1	1	0	3	2	2	1
9545	101	1	13-Aug-07	17	2	12	2	1	1	0	3	2	2	1
9546	201	1	25-May-07	20	2	12	2	1	1	0	3	2	2	1
9547	101	1	1-Nov-07	12	2	12	2	1	1	0	3	2	2	1
9548	101	1	26-Apr-07	17	2	12	2	1	1	0	3	2	2	1
9549	101	1	18-Oct-06	12	2	12	2	1	1	0	3	2	2	1
9550	101	1	30-Sep-06	13	2	12	2	1	1	0	3	2	3	1
9551	101	1	11-May-06	19	2	12	2	1	1	0	3	2	3	1
9552	201	1	6-Dec-06	15	2	12	2	2	1	0	3	2	3	1
9553	101	1	15-Aug-06	20	2	12	2	1	1	0	3	2	1	1
9554	101	1	23-Mar-17	15	2	12	2	1	1	0	3	2	5	1
9555	101	1	24-Jan-17	14	2	12	2	1	1	0	3	2	3	1
9556	101	1	15-Nov-17	17	2	12	2	1	1	0	3	2	3	1
9557	101	1	30-Dec-16	18	2	12	2	1	1	0	3	2	2	1
9558	101	1	11-Aug-17	7	2	12	2	1	1	0	3	2	2	2
9559	201	1	26-Sep-17	20	2	12	2	3	1	0	3	2	3	1
9560	201	1	27-Dec-17	16	2	12	2	1	1	0	3	2	3	1
9561	101	1	21-Apr-16	7	2	12	2	1	1	0	6	2	3	1
9562	101	1	30-Aug-13	10	2	12	2	1	1	0	6	2	3	1
9563	101	1	29-May-12	14	2	12	2	1	1	0	6	2	2	1
9564	101	1	7-Jul-10	13	2	12	2	1	1	0	6	2	3	1
9565	101	1	3-Jun-09	11	2	12	2	1	1	0	6	2	3	1
9566	202	0	9-Sep-13	21	1	13	2	1	1	0	6	2	3	1
9567	202	0	24-Oct-14	16	1	13	2	1	1	0	4	2	1	1
9568	202	0	23-Nov-12	16	1	13	2	1	1	0	6	2	2	2
9569	202	0	30-Jun-17	18	1	13	2	1	1	0	6	2	2	1
9570	202	0	17-Nov-16	21	1	13	2	1	1	0	4	2	2	1
9571	202	0	23-Aug-17	0	1	13	2	1	1	0	6	2	3	1
9572	202	0	8-Jul-16	14	1	13	2	2	1	0	10	2	6	1
9573	202	0	10-Sep-15	11	1	13	2	1	1	0	13	2	1	1
9574	202	0	8-May-07	17	1	13	2	1	1	0	13	2	1	1
9575	202	0	9-Aug-17	10	1	13	2	1	1	0	13	2	1	1
9576	202	0	5-Nov-08	17	1	13	2	1	1	0	1	2	6	2
9577	202	0	7-Dec-16	11	1	13	2	1	1	0	4	2	4	1
9578	202	0	21-Nov-16	8	1	13	2	2	1	0	4	2	1	1
9579	202	0	12-May-16	10	1	13	2	3	1	0	4	2	3	1
9580	202	0	28-Oct-16	7	1	13	2	2	1	0	4	2	1	1
9581	202	0	23-Jun-15	22	1	13	2	3	1	0	4	2	6	1
9582	202	0	17-Dec-15	14	1	13	2	1	1	0	4	2	1	1
9583	202	0	31-Oct-15	17	1	13	2	1	1	0	4	2	1	1
9584	202	0	6-Nov-14	11	1	13	2	1	1	0	4	2	1	1
9585	202	0	5-Jul-13	9	1	13	2	3	1	0	4	2	2	2
9586	202	0	6-Dec-13	16	1	13	2	1	1	0	4	2	1	2
9587	202	0	9-Oct-13	17	1	13	2	1	1	0	4	2	1	1
9588	202	0	30-May-13	18	1	13	2	1	1	0	4	2	2	1
9589	202	0	27-Jun-13	16	1	13	2	1	1	0	4	2	3	1
9590	202	0	5-Jul-10	12	1	13	2	2	1	0	4	2	1	1
9591	202	0	26-Mar-09	8	1	13	2	1	1	0	4	2	7	1
9592	202	0	22-Jun-09	17	1	13	2	1	1	0	4	2	1	2
9593	202	0	25-Dec-09	17	1	13	2	1	1	0	4	2	2	1
9594	202	0	9-May-09	12	1	13	2	3	1	0	4	2	7	2
9595	202	0	18-Sep-09	9	1	13	2	1	1	0	4	2	7	1
9596	202	0	28-Nov-07	12	1	13	2	1	1	0	4	2	1	2
9597	202	0	22-Mar-17	10	1	13	2	1	1	0	4	2	4	1
9598	202	0	30-Sep-17	8	1	13	2	1	1	0	4	2	2	1
9599	202	0	11-Oct-17	15	1	13	2	1	1	0	4	2	1	1
9600	202	0	27-Aug-16	13	1	13	2	3	1	0	11	2	3	1
9601	202	0	10-Jul-16	9	1	13	2	3	1	0	11	2	3	1
9602	202	0	8-Jan-13	12	1	13	2	1	1	0	11	2	1	1
9603	202	0	7-Sep-11	19	1	13	2	1	1	0	11	2	3	1
9604	202	0	9-Jun-10	14	1	13	2	1	1	0	11	2	3	2
9605	202	0	23-Sep-17	22	1	13	2	2	1	0	11	2	3	1
9606	202	0	23-Feb-17	0	1	13	2	3	1	0	11	2	3	2
9607	202	0	22-Dec-17	11	1	13	2	1	1	0	11	2	2	1
9608	202	0	20-Dec-16	16	1	13	2	3	1	0	3	2	3	1
9609	202	0	21-Dec-16	15	1	13	2	1	1	0	3	2	2	1
9610	202	0	25-Mar-16	20	1	13	2	1	1	0	3	2	2	2
9611	202	0	14-Jan-16	10	1	13	2	1	1	0	3	2	1	1
9612	202	0	29-Dec-16	15	1	13	2	1	1	0	3	2	1	1
9613	202	0	31-May-16	9	1	13	2	3	1	0	3	2	2	1
9614	202	0	6-May-16	16	1	13	2	3	1	0	3	2	1	1
9615	202	0	21-Dec-16	8	1	13	2	1	1	0	3	2	1	1
9616	202	0	28-Dec-16	19	1	13	2	3	1	0	3	2	4	1
9617	202	0	9-Sep-16	17	1	13	2	1	1	0	3	2	1	1
9618	202	0	24-Oct-16	18	1	13	2	3	1	0	3	2	1	1
9619	202	0	10-Jul-16	22	1	13	2	1	1	0	3	2	1	1
9620	202	0	30-Jun-16	8	1	13	2	2	1	0	3	2	2	1
9621	202	0	1-Jan-16	22	1	13	2	1	1	0	3	2	2	1
9622	202	0	2-Nov-15	20	1	13	2	1	1	0	3	2	3	1
9623	202	0	29-May-15	15	1	13	2	3	1	0	3	2	4	1
9624	202	0	24-Dec-15	14	1	13	2	1	1	0	3	2	4	1
9625	202	0	6-Jun-15	15	1	13	2	1	1	0	3	2	1	1
9626	202	0	25-Nov-15	17	1	13	2	1	1	0	3	2	2	1
9627	202	0	2-Jul-15	18	1	13	2	1	1	0	3	2	2	2
9628	202	0	31-May-14	15	1	13	2	1	1	0	3	2	1	1
9629	202	0	14-Mar-14	16	1	13	2	3	1	0	3	2	1	1
9630	202	0	19-Dec-14	11	1	13	2	1	1	0	3	2	1	1
9631	202	0	3-Sep-14	12	1	13	2	2	1	0	3	2	4	1
9632	202	0	2-Mar-14	18	1	13	2	1	1	0	3	2	2	1
9633	202	0	16-May-14	13	1	13	2	3	1	0	3	2	3	1
9634	202	0	22-Jun-14	22	1	13	2	1	1	0	3	2	3	1
9635	202	0	15-May-14	18	1	13	2	2	1	0	3	2	4	2
9636	202	0	12-Nov-14	16	1	13	2	1	1	0	3	2	1	1
9637	202	0	29-May-14	18	1	13	2	1	1	0	3	2	1	1
9638	202	0	27-May-14	17	1	13	2	1	1	0	3	2	1	1
9639	202	0	23-Dec-14	20	1	13	2	1	1	0	3	2	3	2
9640	202	0	14-Oct-14	20	1	13	2	1	1	0	3	2	2	1
9641	202	0	19-Nov-14	16	1	13	2	1	1	0	3	2	3	1
9642	202	0	24-Oct-14	13	1	13	2	1	1	0	3	2	1	1
9643	202	0	9-Jul-13	8	1	13	2	1	1	0	3	2	1	1
9644	202	0	13-May-13	9	1	13	2	1	1	0	3	2	3	2
9645	202	0	30-Sep-13	11	1	13	2	1	1	0	3	2	5	2
9646	202	0	5-Jul-13	14	1	13	2	2	1	0	3	2	4	1
9647	202	0	12-Dec-13	18	1	13	2	1	1	0	3	2	2	2
9648	202	0	19-May-13	15	1	13	2	1	1	0	3	2	3	2
9649	202	0	11-Sep-13	16	1	13	2	1	1	0	3	2	2	2
9650	202	0	6-Jun-13	15	1	13	2	1	1	0	3	2	2	1
9651	202	0	6-Mar-13	21	1	13	2	1	1	0	3	2	1	1
9652	202	0	22-Nov-13	17	1	13	2	1	1	0	3	2	2	2
9653	202	0	21-Nov-13	17	1	13	2	1	1	0	3	2	2	2
9654	202	0	21-Mar-13	9	1	13	2	1	1	0	3	2	3	1
9655	202	0	26-Dec-12	10	1	13	2	1	1	0	3	2	2	1
9656	202	0	12-Nov-12	15	1	13	2	3	1	0	3	2	1	2
9657	202	0	15-Oct-12	16	1	13	2	1	1	0	3	2	3	1
9658	202	0	14-Nov-12	17	1	13	2	1	1	0	3	2	2	1
9659	202	0	20-Nov-12	14	1	13	2	1	1	0	3	2	2	2
9660	202	0	6-Apr-11	16	1	13	2	1	1	0	3	2	1	2
9661	202	0	16-Mar-11	15	1	13	2	1	1	0	3	2	2	1
9662	202	0	15-Apr-11	21	1	13	2	2	1	0	3	2	1	2
9663	202	0	4-Jul-11	22	1	13	2	1	1	0	3	2	3	1
9664	202	0	17-Nov-10	20	1	13	2	1	1	0	3	2	1	2
9665	202	0	6-Aug-10	22	1	13	2	1	1	0	3	2	2	2

9666	202	0	26-Dec-10	16	1	13	2	3	1	0	3	2	3	2
9667	202	0	13-May-10	9	1	13	2	1	1	0	3	2	1	1
9668	202	0	21-Oct-09	7	1	13	2	1	1	0	3	2	4	1
9669	202	0	29-Mar-09	11	1	13	2	2	1	0	3	2	2	2
9670	202	0	22-Apr-09	12	1	13	2	1	1	0	3	2	2	1
9671	202	0	22-Jun-09	15	1	13	2	2	1	0	3	2	2	2
9672	202	0	18-Dec-08	22	1	13	2	1	1	0	3	2	1	1
9673	202	0	14-Mar-08	17	1	13	2	1	1	0	3	2	1	1
9674	202	0	25-Aug-08	17	1	13	2	1	1	0	3	2	1	1
9675	202	0	8-Jan-08	19	1	13	2	3	1	0	3	2	3	1
9676	202	0	14-Nov-08	21	1	13	2	3	1	0	3	2	2	1
9677	202	0	9-Oct-08	15	1	13	2	3	1	0	3	2	1	1
9678	202	0	18-Apr-08	16	1	13	2	3	1	0	3	2	2	2
9679	202	0	16-Dec-08	10	1	13	2	1	1	0	3	2	3	1
9680	202	0	19-Jun-07	15	1	13	2	1	1	0	3	2	1	1
9681	202	0	28-Sep-07	15	1	13	2	1	1	0	3	2	6	1
9682	202	0	13-Oct-07	23	1	13	2	1	1	0	3	2	2	1
9683	202	0	13-Mar-07	19	1	13	2	1	1	0	3	2	2	2
9684	202	0	20-Nov-07	17	1	13	2	1	1	0	3	2	3	1
9685	202	0	22-Jun-07	17	1	13	2	2	1	0	3	2	2	1
9686	202	0	1-Jun-07	11	1	13	2	1	1	0	3	2	2	2
9687	202	0	30-Apr-07	13	1	13	2	1	1	0	3	2	1	2
9688	202	0	16-Apr-07	16	1	13	2	1	1	0	3	2	1	1
9689	202	0	12-Jun-07	13	1	13	2	1	1	0	3	2	3	1
9690	202	0	1-Mar-06	20	1	13	2	1	1	0	3	2	1	1
9691	202	0	12-May-06	17	1	13	2	1	1	0	3	2	2	2
9692	202	0	4-Jan-06	20	1	13	2	1	1	0	3	2	4	1
9693	202	0	2-May-06	18	1	13	2	1	1	0	3	2	2	1
9694	202	0	18-Sep-17	9	1	13	2	1	1	0	3	2	4	1
9695	202	0	15-Oct-17	14	1	13	2	1	1	0	3	2	3	1
9696	202	0	25-Sep-17	22	1	13	2	1	1	0	3	2	1	1
9697	202	0	21-Mar-17	15	1	13	2	1	1	0	3	2	1	1
9698	202	0	29-May-17	13	1	13	2	1	1	0	3	2	1	1
9699	202	0	8-May-17	0	1	13	2	1	1	0	3	2	1	1
9700	202	0	14-Apr-17	18	1	13	2	1	1	0	3	2	2	2
9701	202	0	31-Jan-17	12	1	13	2	2	1	0	3	2	3	1
9702	202	0	20-Mar-17	15	1	13	2	2	1	0	3	2	3	1
9703	202	0	20-Feb-17	18	1	13	2	2	1	0	3	2	3	1
9704	202	0	4-May-17	9	1	13	2	2	1	0	3	2	1	1
9705	202	0	23-Mar-17	9	1	13	2	3	1	0	3	2	2	1
9706	202	0	24-Mar-17	18	1	13	2	3	1	0	3	2	2	1
9707	202	0	12-Sep-17	11	1	13	2	3	1	0	3	2	1	1
9708	202	0	11-Dec-17	17	1	13	2	3	1	0	3	2	2	1
9709	202	0	8-Jun-13	14	1	13	2	1	1	0	6	2	2	2
9710	202	0	23-Apr-13	18	1	13	2	1	1	0	6	2	2	1
9711	202	0	3-Jul-11	9	1	13	2	1	1	0	6	2	2	1
9712	202	0	30-Dec-11	21	1	13	2	1	1	0	6	2	3	1
9713	202	0	7-Oct-08	17	1	13	2	1	1	0	6	2	1	1
9714	202	0	12-Jul-08	19	1	13	2	1	1	0	6	2	2	1
9715	202	0	2-Nov-06	16	1	13	2	1	1	0	6	2	2	1
9716	202	0	23-Nov-16	9	1	13	2	1	1	0	13	2	1	1
9717	202	0	28-Nov-10	15	1	13	2	1	1	0	13	2	1	1
9718	202	0	12-Apr-17	16	2	13	2	1	1	0	4	2	1	1
9719	202	0	8-Jul-16	14	2	13	2	1	1	0	10	2	6	2
9720	202	0	16-May-06	15	2	13	2	1	1	0	13	2	7	1
9721	202	0	30-May-17	9	2	13	2	1	1	0	13	2	1	1
9722	202	0	5-Nov-08	17	2	13	2	1	1	0	1	2	2	2
9723	202	0	27-Mar-16	18	2	13	2	3	1	0	4	2	2	1
9724	202	0	24-Oct-14	15	2	13	2	1	1	0	4	2	1	1
9725	202	0	14-Oct-14	16	2	13	2	1	1	0	4	2	1	1
9726	202	0	8-Jan-13	14	2	13	2	1	1	0	4	2	2	1
9727	202	0	19-Dec-12	17	2	13	2	3	1	0	4	2	2	1
9728	202	0	5-Jul-11	20	2	13	2	1	1	0	4	2	5	1
9729	202	0	21-Sep-07	20	2	13	2	1	1	0	4	2	4	1
9730	202	0	2-Nov-06	19	2	13	2	1	1	0	4	2	1	1
9731	202	0	8-Oct-06	15	2	13	2	1	1	0	4	2	3	1
9732	202	0	26-Jul-14	14	2	13	2	1	1	0	11	2	5	1
9733	202	0	23-Aug-14	19	2	13	2	1	1	0	11	2	2	1
9734	202	0	10-Jun-13	9	2	13	2	1	1	0	11	2	2	1
9735	202	0	13-Jan-10	20	2	13	2	1	1	0	11	2	3	2
9736	202	0	16-Jun-17	20	2	13	2	1	1	0	11	2	3	1
9737	202	0	19-Feb-17	21	2	13	2	3	1	0	11	2	2	1
9738	202	0	16-Mar-16	16	2	13	2	1	1	0	3	2	1	1
9739	202	0	5-Apr-16	16	2	13	2	1	1	0	3	2	2	1
9740	202	0	16-Sep-15	19	2	13	2	1	1	0	3	2	3	1
9741	202	0	1-May-14	15	2	13	2	1	1	0	3	2	3	1
9742	202	0	21-Nov-14	10	2	13	2	1	1	0	3	2	5	1
9743	202	0	12-Nov-13	21	2	13	2	1	1	0	3	2	1	1
9744	202	0	15-Nov-13	17	2	13	2	1	1	0	3	2	4	1
9745	202	0	24-Jun-13	14	2	13	2	1	1	0	3	2	4	1
9746	202	0	18-Jun-13	18	2	13	2	3	1	0	3	2	3	1
9747	202	0	27-Dec-12	17	2	13	2	1	1	0	3	2	1	1
9748	202	0	5-Dec-12	19	2	13	2	3	1	0	3	2	2	1
9749	202	0	28-Aug-12	16	2	13	2	1	1	0	3	2	3	1
9750	202	0	6-Nov-11	20	2	13	2	1	1	0	3	2	5	2
9751	202	0	28-Oct-11	12	2	13	2	1	1	0	3	2	1	2
9752	202	0	2-Dec-10	18	2	13	2	1	1	0	3	2	3	1
9753	202	0	9-Dec-10	9	2	13	2	1	1	0	3	2	2	1
9754	202	0	4-Mar-08	8	2	13	2	1	1	0	3	2	4	1
9755	202	0	24-Nov-08	9	2	13	2	1	1	0	3	2	1	2
9756	202	0	27-Dec-07	10	2	13	2	1	1	0	3	2	1	1
9757	202	0	26-Jun-07	16	2	13	2	1	1	0	3	2	2	2
9758	202	0	25-Dec-06	17	2	13	2	1	1	0	3	2	4	1
9759	202	0	22-Nov-06	17	2	13	2	1	1	0	3	2	3	1
9760	202	0	17-Oct-17	20	2	13	2	1	1	0	3	2	4	1
9761	202	0	29-Aug-17	11	2	13	2	2	1	0	3	2	5	1
9762	202	0	11-Dec-17	11	2	13	2	2	1	0	3	2	4	1
9763	202	0	2-Oct-07	14	2	13	2	1	1	0	6	2	3	1
9764	101	1	26-Jan-13	15	1	13	2	1	1	0	1	2	1	1
9765	201	1	10-Dec-13	11	1	13	2	2	1	0	1	2	2	1
9766	101	1	25-Oct-17	18	1	13	2	1	1	0	1	2	3	1
9767	101	1	10-Jul-16	19	1	13	2	1	1	0	3	2	2	1
9768	201	1	10-Jan-17	19	1	13	2	3	1	0	3	2	5	1
9769	201	1	5-May-17	17	1	13	2	1	1	0	1	2	1	1
9770	101	1	24-Apr-12	17	1	13	2	1	1	0	6	2	2	1
9771	201	1	22-Jan-16	10	1	13	2	2	1	0	4	2	1	1
9772	201	1	18-Apr-15	17	1	13	2	3	1	0	6	2	3	1
9773	201	1	14-Dec-16	10	1	13	2	1	1	0	3	2	2	1
9774	201	1	19-Sep-12	15	1	13	2	1	1	0	13	2	2	1
9775	101	1	12-Apr-13	15	1	13	2	1	1	0	6	2	2	1
9776	101	1	17-Jan-12	19	1	13	2	1	1	0	4	2	2	1
9777	201	1	11-Jun-12	11	1	13	2	2	1	0	4	2	1	1
9778	101	1	3-Sep-12	22	1	13	2	1	1	0	7	2	5	1
9779	101	1	22-Nov-10	10	1	13	2	1	1	0	7	2	4	1
9780	101	1	24-Jun-06	19	1	13	2	1	1	0	7	2	4	1
9781	201	1	31-May-12	10	1	13	2	1	1	0	13	2	2	1
9782	201	1	22-Sep-12	15	1	13	2	3	1	0	13	2	2	1
9783	101	1	29-Apr-06	10	1	13	2	1	1	0	13	2	1	2
9784	101	1	13-Jan-06	17	1	13	2	1	1	0	13	2	3	1
9785	101	1	19-Oct-17	16	1	13	2	1	1	0	13	2	2	1
9786	101	1	6-Jul-07	22	1	13	2	1	1	0	1	2	3	1
9787	101	1	10-Apr-12	11	1	13	2	1	1	0	1	2	2	4
9788	101	1	22-Nov-11	16	1	13	2	1	1	0	1	2	1	1
9789	101	1	19-Oct-16	20	1	13	2	1	1	0	4	2	3	1

9790	101	1	6-Jun-16	14	1	13	2	1	1	0	4	2	1	1
9791	101	1	18-Jun-16	16	1	13	2	1	1	0	4	2	2	1
9792	101	1	17-Apr-15	17	1	13	2	1	1	0	4	2	4	1
9793	201	1	8-Aug-15	11	1	13	2	3	1	0	4	2	2	1
9794	101	1	7-May-14	13	1	13	2	1	1	0	4	2	2	1
9795	101	1	7-Nov-14	17	1	13	2	1	1	0	4	2	2	1
9796	101	1	8-Jan-14	17	1	13	2	1	1	0	4	2	4	1
9797	101	1	13-Oct-14	12	1	13	2	1	1	0	4	2	4	1
9798	101	1	15-Apr-14	19	1	13	2	1	1	0	4	2	1	1
9799	101	1	29-May-14	17	1	13	2	1	1	0	4	2	1	1
9800	101	1	17-Jun-14	18	1	13	2	1	1	0	4	2	1	1
9801	101	1	24-Apr-14	7	1	13	2	1	1	0	4	2	2	1
9802	101	1	16-Apr-14	20	1	13	2	1	1	0	4	2	2	1
9803	101	1	11-Sep-14	16	1	13	2	1	1	0	4	2	3	1
9804	201	1	8-Dec-14	14	1	13	2	3	1	0	4	2	2	1
9805	101	1	10-Sep-14	1	1	13	2	1	1	0	4	2	2	1
9806	101	1	31-Oct-14	11	1	13	2	1	1	0	4	2	1	1
9807	101	1	28-Nov-14	16	1	13	2	1	1	0	4	2	2	1
9808	101	1	26-Apr-14	18	1	13	2	1	1	0	4	2	2	1
9809	101	1	24-Jun-14	16	1	13	2	1	1	0	4	2	4	1
9810	101	1	25-Dec-13	15	1	13	2	1	1	0	4	2	5	1
9811	201	1	1-Aug-13	14	1	13	2	2	1	0	4	2	3	1
9812	101	1	12-Sep-13	12	1	13	2	1	1	0	4	2	4	1
9813	101	1	16-May-13	10	1	13	2	1	1	0	4	2	2	1
9814	101	1	25-Jun-13	18	1	13	2	1	1	0	4	2	2	1
9815	101	1	24-Mar-13	8	1	13	2	1	1	0	4	2	2	1
9816	101	1	5-Nov-13	16	1	13	2	1	1	0	4	2	2	1
9817	101	1	1-May-12	16	1	13	2	1	1	0	4	2	3	4
9818	101	1	29-Jun-12	12	1	13	2	1	1	0	4	2	2	1
9819	101	1	31-Mar-12	10	1	13	2	1	1	0	4	2	1	1
9820	101	1	6-Nov-12	21	1	13	2	1	1	0	4	2	3	1
9821	101	1	13-Mar-12	17	1	13	2	1	1	0	4	2	2	1
9822	101	1	9-May-12	19	1	13	2	1	1	0	4	2	3	1
9823	101	1	24-May-12	17	1	13	2	1	1	0	4	2	2	1
9824	101	1	1-Jul-12	17	1	13	2	1	1	0	4	2	2	1
9825	101	1	12-Jun-12	12	1	13	2	1	1	0	4	2	2	1
9826	101	1	21-Feb-12	11	1	13	2	2	1	0	4	2	2	4
9827	101	1	24-Oct-11	17	1	13	2	1	1	0	4	2	7	1
9828	101	1	23-Jun-11	11	1	13	2	1	1	0	4	2	3	1
9829	101	1	1-Dec-11	12	1	13	2	1	1	0	4	2	3	1
9830	101	1	24-May-11	11	1	13	2	1	1	0	4	2	3	1
9831	101	1	22-Oct-11	16	1	13	2	1	1	0	4	2	4	1
9832	101	1	16-Dec-11	16	1	13	2	1	1	0	4	2	1	1
9833	101	1	27-Oct-11	20	1	13	2	1	1	0	4	2	2	1
9834	101	1	11-May-11	17	1	13	2	1	1	0	4	2	4	1
9835	101	1	21-Oct-11	10	1	13	2	1	1	0	4	2	3	1
9836	101	1	13-Apr-10	11	1	13	2	1	1	0	4	2	1	1
9837	101	1	21-Dec-10	21	1	13	2	1	1	0	4	2	1	1
9838	101	1	12-May-10	19	1	13	2	1	1	0	4	2	1	1
9839	101	1	4-Dec-10	13	1	13	2	1	1	0	4	2	4	1
9840	101	1	28-Nov-10	16	1	13	2	1	1	0	4	2	4	1
9841	101	1	8-Jun-10	12	1	13	2	1	1	0	4	2	2	1
9842	101	1	4-Jun-10	10	1	13	2	1	1	0	4	2	2	4
9843	101	1	6-Sep-10	18	1	13	2	1	1	0	4	2	3	1
9844	101	1	9-Apr-10	17	1	13	2	1	1	0	4	2	2	1
9845	101	1	4-Nov-09	12	1	13	2	1	1	0	4	2	4	1
9846	101	1	26-Apr-09	18	1	13	2	1	1	0	4	2	4	1
9847	101	1	12-Mar-09	14	1	13	2	1	1	0	4	2	1	1
9848	101	1	24-Dec-09	17	1	13	2	1	1	0	4	2	4	1
9849	101	1	18-Dec-09	12	1	13	2	1	1	0	4	2	1	1
9850	101	1	19-Feb-09	12	1	13	2	1	1	0	4	2	3	1
9851	101	1	29-Oct-09	18	1	13	2	1	1	0	4	2	2	1
9852	101	1	19-May-09	10	1	13	2	1	1	0	4	2	4	1
9853	101	1	11-Nov-09	16	1	13	2	1	1	0	4	2	2	1
9854	101	1	15-May-09	21	1	13	2	1	1	0	4	2	2	1
9855	101	1	20-Apr-09	18	1	13	2	1	1	0	4	2	2	1
9856	101	1	17-Oct-08	11	1	13	2	1	1	0	4	2	1	1
9857	101	1	6-May-08	15	1	13	2	1	1	0	4	2	2	1
9858	101	1	15-May-08	10	1	13	2	1	1	0	4	2	2	4
9859	101	1	13-Sep-08	15	1	13	2	1	1	0	4	2	1	2
9860	101	1	13-Nov-08	16	1	13	2	1	1	0	4	2	2	1
9861	201	1	15-Apr-08	16	1	13	2	1	1	0	4	2	2	1
9862	201	1	15-Nov-07	16	1	13	2	1	1	0	4	2	1	1
9863	101	1	21-May-07	11	1	13	2	1	1	0	4	2	2	1
9864	101	1	8-Jan-07	21	1	13	2	1	1	0	4	2	2	1
9865	101	1	30-Nov-07	18	1	13	2	1	1	0	4	2	4	1
9866	201	1	1-Jun-07	17	1	13	2	1	1	0	4	2	3	1
9867	101	1	25-Jul-07	16	1	13	2	1	1	0	4	2	2	1
9868	101	1	25-Oct-07	12	1	13	2	1	1	0	4	2	2	1
9869	101	1	31-Oct-06	18	1	13	2	1	1	0	4	2	1	1
9870	101	1	5-Feb-06	15	1	13	2	1	1	0	4	2	1	1
9871	101	1	29-Sep-06	11	1	13	2	1	1	0	4	2	1	1
9872	101	1	11-Sep-06	16	1	13	2	1	1	0	4	2	4	1
9873	101	1	25-Jun-06	17	1	13	2	1	1	0	4	2	2	1
9874	201	1	16-Sep-06	8	1	13	2	1	1	0	4	2	3	1
9875	101	1	24-Mar-17	12	1	13	2	1	1	0	4	2	2	1
9876	101	1	19-Sep-17	11	1	13	2	1	1	0	4	2	1	1
9877	201	1	8-Dec-17	13	1	13	2	2	1	0	4	2	1	1
9878	201	1	9-Apr-17	1	1	13	2	2	1	0	4	2	1	2
9879	201	1	27-Nov-17	17	1	13	2	3	1	0	4	2	5	1
9880	101	1	13-Nov-17	12	1	13	2	1	1	0	4	2	4	1
9881	101	1	19-May-16	17	1	13	2	1	1	0	11	2	1	1
9882	101	1	3-Oct-16	21	1	13	2	1	1	0	11	2	2	1
9883	201	1	27-Nov-16	17	1	13	2	1	1	0	11	2	2	1
9884	201	1	19-Jun-16	13	1	13	2	2	1	0	11	2	2	1
9885	201	1	22-May-15	15	1	13	2	2	1	0	11	2	1	1
9886	201	1	23-Jun-15	20	1	13	2	2	1	0	11	2	2	1
9887	101	1	6-Nov-15	15	1	13	2	1	1	0	11	2	2	1
9888	101	1	3-Dec-15	14	1	13	2	1	1	0	11	2	2	1
9889	201	1	14-May-14	14	1	13	2	3	1	0	11	2	3	1
9890	101	1	13-Dec-14	19	1	13	2	1	1	0	11	2	3	1
9891	101	1	30-Jan-14	17	1	13	2	1	1	0	11	2	2	1
9892	201	1	5-Apr-14	23	1	13	2	3	1	0	11	2	2	1
9893	101	1	17-Jun-14	10	1	13	2	1	1	0	11	2	2	1
9894	101	1	27-Mar-14	10	1	13	2	1	1	0	11	2	2	1
9895	101	1	16-Jan-14	20	1	13	2	1	1	0	11	2	3	1
9896	201	1	8-Feb-14	18	1	13	2	3	1	0	11	2	3	1
9897	201	1	18-Feb-13	9	1	13	2	3	1	0	11	2	2	1
9898	101	1	9-Nov-13	9	1	13	2	1	1	0	11	2	2	1
9899	101	1	29-Mar-13	19	1	13	2	1	1	0	11	2	2	1
9900	101	1	26-Nov-13	21	1	13	2	1	1	0	11	2	2	1
9901	101	1	9-Sep-13	20	1	13	2	1	1	0	11	2	3	1
9902	201	1	19-Nov-13	10	1	13	2	2	1	0	11	2	2	1
9903	101	1	19-Nov-12	19	1	13	2	1	1	0	11	2	3	1
9904	101	1	19-Nov-12	17	1	13	2	1	1	0	11	2	2	1
9905	101	1	28-Oct-12	10	1	13	2	1	1	0	11	2	2	1
9906	101	1	18-Jun-12	19	1	13	2	3	1	0	11	2	2	1
9907	201	1	8-Jun-12	19	1	13	2	2	1	0	11	2	3	1
9908	101	1	30-Mar-12	13	1	13	2	3	1	0	11	2	3	1
9909	101	1	24-Apr-12	21	1	13	2	1	1	0	11	2	3	1
9910	101	1	2-Oct-12	11	1	13	2	1	1	0	11	2	3	1
9911	101	1	14-May-11	10	1	13	2	1	1	0	11	2	1	1
9912	201	1	28-Jul-11	14	1	13	2	2	1	0	11	2	3	1
9913	101	1	20-Aug-11	19	1	13	2	1	1	0	11	2	3	1

9914	101	1	27-Feb-10	20	1	13	2	1	1	0	11	2	3	1
9915	101	1	11-Dec-10	16	1	13	2	3	1	0	11	2	3	1
9916	101	1	11-Jul-10	19	1	13	2	1	1	0	11	2	3	1
9917	101	1	3-Oct-10	21	1	13	2	1	1	0	11	2	3	1
9918	101	1	29-Apr-09	17	1	13	2	1	1	0	11	2	2	1
9919	201	1	17-Nov-08	14	1	13	2	2	1	0	11	2	2	1
9920	101	1	12-Sep-08	21	1	13	2	1	1	0	11	2	2	1
9921	201	1	17-Sep-17	10	1	13	2	1	1	0	11	2	3	1
9922	101	1	15-Jul-17	14	1	13	2	1	1	0	11	2	2	1
9923	201	1	22-Jun-17	21	1	13	2	1	1	0	11	2	2	1
9924	101	1	13-Dec-17	13	1	13	2	1	1	0	11	2	2	1
9925	201	1	30-Jun-16	22	1	13	2	2	1	0	3	2	1	1
9926	101	1	23-Mar-16	13	1	13	2	1	1	0	3	2	2	1
9927	101	1	31-May-16	19	1	13	2	1	1	0	3	2	3	1
9928	101	1	24-Jun-16	13	1	13	2	1	1	0	3	2	2	1
9929	201	1	27-Jun-16	10	1	13	2	2	1	0	3	2	3	1
9930	101	1	5-May-16	17	1	13	2	1	1	0	3	2	5	1
9931	201	1	1-Nov-16	10	1	13	2	1	1	0	3	2	2	1
9932	201	1	6-Oct-16	16	1	13	2	2	1	0	3	2	3	1
9933	101	1	3-Apr-16	13	1	13	2	1	1	0	3	2	3	1
9934	101	1	25-May-16	20	1	13	2	1	1	0	3	2	2	4
9935	101	1	27-Apr-16	17	1	13	2	1	1	0	3	2	4	1
9936	101	1	29-Mar-16	12	1	13	2	1	1	0	3	2	2	1
9937	101	1	27-Jul-16	20	1	13	2	1	1	0	3	2	1	1
9938	101	1	6-Feb-16	20	1	13	2	1	1	0	3	2	2	2
9939	101	1	15-Jan-16	10	1	13	2	1	1	0	3	2	2	4
9940	101	1	13-Apr-16	12	1	13	2	1	1	0	3	2	2	1
9941	201	1	18-Mar-16	16	1	13	2	3	1	0	3	2	2	1
9942	101	1	29-May-16	18	1	13	2	1	1	0	3	2	2	4
9943	101	1	31-Mar-16	17	1	13	2	1	1	0	3	2	5	1
9944	101	1	10-Jul-16	13	1	13	2	1	1	0	3	2	2	1
9945	101	1	21-May-16	18	1	13	2	1	1	0	3	2	3	1
9946	101	1	14-Mar-16	17	1	13	2	1	1	0	3	2	3	1
9947	101	1	13-May-16	20	1	13	2	1	1	0	3	2	3	1
9948	101	1	5-Jul-16	17	1	13	2	1	1	0	3	2	3	1
9949	101	1	14-May-16	11	1	13	2	1	1	0	3	2	2	1
9950	101	1	5-Jul-16	18	1	13	2	1	1	0	3	2	2	1
9951	101	1	4-Mar-16	15	1	13	2	1	1	0	3	2	2	1
9952	101	1	10-Apr-16	15	1	13	2	1	1	0	3	2	4	1
9953	101	1	7-Aug-16	19	1	13	2	1	1	0	3	2	2	2
9954	101	1	15-Jul-16	16	1	13	2	1	1	0	3	2	4	1
9955	101	1	4-Mar-16	18	1	13	2	1	1	0	3	2	2	1
9956	101	1	7-Apr-16	19	1	13	2	1	1	0	3	2	4	1
9957	101	1	19-Jun-16	16	1	13	2	1	1	0	3	2	2	1
9958	101	1	24-Dec-16	12	1	13	2	1	1	0	3	2	3	1
9959	201	1	8-Dec-16	18	1	13	2	1	1	0	3	2	3	1
9960	201	1	17-Apr-16	12	1	13	2	2	1	0	3	2	3	1
9961	201	1	8-Mar-16	10	1	13	2	1	1	0	3	2	2	1
9962	201	1	28-Apr-16	16	1	13	2	2	1	0	3	2	1	1
9963	201	1	5-Jan-16	19	1	13	2	2	1	0	3	2	2	1
9964	201	1	1-Nov-16	18	1	13	2	2	1	0	3	2	3	1
9965	201	1	22-Sep-16	17	1	13	2	1	1	0	3	2	4	1
9966	201	1	9-Nov-16	9	1	13	2	1	1	0	3	2	2	1
9967	201	1	11-Oct-16	16	1	13	2	1	1	0	3	2	4	1
9968	201	1	2-Jun-16	17	1	13	2	1	1	0	3	2	3	2
9969	201	1	22-Mar-16	12	1	13	2	2	1	0	3	2	3	1
9970	201	1	15-Oct-16	16	1	13	2	2	1	0	3	2	3	1
9971	101	1	2-Aug-16	19	1	13	2	1	1	0	3	2	2	2
9972	101	1	10-Apr-15	11	1	13	2	1	1	0	3	2	3	1
9973	101	1	6-Oct-15	18	1	13	2	1	1	0	3	2	1	1
9974	101	1	2-Jun-15	16	1	13	2	1	1	0	3	2	3	1
9975	201	1	1-Dec-15	16	1	13	2	2	1	0	3	2	3	1
9976	201	1	16-Dec-15	12	1	13	2	2	1	0	3	2	1	1
9977	201	1	16-Oct-15	15	1	13	2	2	1	0	3	2	1	1
9978	201	1	30-Jan-15	16	1	13	2	2	1	0	3	2	3	1
9979	101	1	28-Apr-15	15	1	13	2	1	1	0	3	2	2	1
9980	201	1	28-Apr-15	15	1	13	2	1	1	0	3	2	2	1
9981	201	1	14-Jan-15	17	1	13	2	2	1	0	3	2	2	1
9982	201	1	2-Jun-15	19	1	13	2	1	1	0	3	2	2	1
9983	201	1	28-Sep-15	9	1	13	2	2	1	0	3	2	3	1
9984	101	1	8-Apr-15	15	1	13	2	1	1	0	3	2	2	1
9985	201	1	21-Oct-15	11	1	13	2	1	1	0	3	2	2	1
9986	101	1	14-Apr-15	15	1	13	2	1	1	0	3	2	2	1
9987	101	1	20-Nov-15	18	1	13	2	1	1	0	3	2	2	1
9988	101	1	11-May-15	19	1	13	2	1	1	0	3	2	2	1
9989	101	1	6-May-15	19	1	13	2	1	1	0	3	2	1	1
9990	101	1	3-Jul-15	16	1	13	2	1	1	0	3	2	2	1
9991	101	1	7-Jan-15	12	1	13	2	1	1	0	3	2	2	1
9992	101	1	16-Mar-15	16	1	13	2	1	1	0	3	2	2	1
9993	101	1	18-Jun-15	12	1	13	2	1	1	0	3	2	3	1
9994	201	1	23-Oct-15	17	1	13	2	2	1	0	3	2	3	1
9995	201	1	11-Jul-15	12	1	13	2	1	1	0	3	2	2	1
9996	101	1	13-Nov-15	16	1	13	2	1	1	0	3	2	2	1
9997	101	1	11-Dec-15	10	1	13	2	1	1	0	3	2	3	1
9998	101	1	2-Oct-15	16	1	13	2	1	1	0	3	2	1	1
9999	201	1	20-Apr-15	18	1	13	2	2	1	0	3	2	5	1
10000	101	1	25-Nov-15	20	1	13	2	1	1	0	3	2	2	1
10001	101	1	17-Dec-15	16	1	13	2	1	1	0	3	2	3	1
10002	201	1	20-Apr-15	15	1	13	2	2	1	0	3	2	3	1
10003	101	1	28-May-15	17	1	13	2	1	1	0	3	2	2	4
10004	101	1	28-Dec-15	20	1	13	2	1	1	0	3	2	2	1
10005	101	1	22-Oct-15	16	1	13	2	1	1	0	3	2	3	1
10006	101	1	25-May-15	16	1	13	2	1	1	0	3	2	3	1
10007	101	1	11-Nov-15	16	1	13	2	1	1	0	3	2	2	1
10008	101	1	17-Sep-15	10	1	13	2	1	1	0	3	2	2	1
10009	201	1	19-Oct-15	19	1	13	2	2	1	0	3	2	2	1
10010	101	1	1-Feb-15	19	1	13	2	1	1	0	3	2	2	1
10011	101	1	30-Nov-15	9	1	13	2	1	1	0	3	2	1	1
10012	101	1	3-Apr-15	17	1	13	2	1	1	0	3	2	3	1
10013	201	1	8-Jun-15	15	1	13	2	1	1	0	3	2	1	1
10014	201	1	20-Nov-15	17	1	13	2	2	1	0	3	2	2	1
10015	201	1	16-Jun-15	12	1	13	2	2	1	0	3	2	4	1
10016	201	1	21-May-15	10	1	13	2	2	1	0	3	2	3	1
10017	201	1	27-Apr-15	10	1	13	2	1	1	0	3	2	2	1
10018	201	1	20-Mar-15	14	1	13	2	3	1	0	3	2	2	1
10019	101	1	2-Nov-15	18	1	13	2	1	1	0	3	2	3	1
10020	201	1	25-May-15	11	1	13	2	3	1	0	3	2	3	2
10021	201	1	12-Jun-15	19	1	13	2	2	1	0	3	2	2	1
10022	201	1	20-Jan-15	10	1	13	2	2	1	0	3	2	2	1
10023	101	1	26-Oct-14	19	1	13	2	1	1	0	3	2	4	1
10024	101	1	16-Dec-14	17	1	13	2	1	1	0	3	2	1	1
10025	201	1	24-Dec-14	14	1	13	2	1	1	0	3	2	1	1
10026	101	1	16-Jan-14	8	1	13	2	1	1	0	3	2	1	1
10027	101	1	3-Apr-14	12	1	13	2	1	1	0	3	2	1	1
10028	101	1	4-Nov-14	9	1	13	2	1	1	0	3	2	3	1
10029	101	1	28-Nov-14	10	1	13	2	1	1	0	3	2	4	1
10030	101	1	26-Jun-14	18	1	13	2	1	1	0	3	2	2	1
10031	101	1	16-Oct-14	12	1	13	2	1	1	0	3	2	2	1
10032	201	1	17-Apr-14	17	1	13	2	3	1	0	3	2	2	1
10033	101	1	9-Jun-14	17	1	13	2	1	1	0	3	2	2	1
10034	101	1	28-Oct-14	17	1	13	2	1	1	0	3	2	3	1
10035	101	1	28-May-14	9	1	13	2	1	1	0	3	2	4	1
10036	201	1	26-Sep-14	17	1	13	2	2	1	0	3	2	3	1
10037	101	1	17-Jun-14	11	1	13	2	1	1	0	3	2	4	1

10038	101	1	2-Nov-14	13	1	13	2	1	1	0	3	2	5	1
10039	101	1	15-Nov-14	12	1	13	2	1	1	0	3	2	5	1
10040	101	1	8-Jan-14	7	1	13	2	1	1	0	3	2	5	1
10041	101	1	21-Jan-14	18	1	13	2	1	1	0	3	2	1	1
10042	101	1	14-Jul-14	18	1	13	2	1	1	0	3	2	1	1
10043	101	1	27-Apr-14	20	1	13	2	1	1	0	3	2	1	1
10044	201	1	22-May-14	22	1	13	2	1	1	0	3	2	1	1
10045	101	1	24-Oct-14	20	1	13	2	1	1	0	3	2	3	1
10046	101	1	23-Jan-14	19	1	13	2	1	1	0	3	2	5	1
10047	201	1	29-Oct-14	21	1	13	2	2	1	0	3	2	3	1
10048	101	1	26-Apr-14	19	1	13	2	1	1	0	3	2	2	1
10049	101	1	25-Sep-14	16	1	13	2	1	1	0	3	2	4	1
10050	101	1	11-Oct-14	23	1	13	2	1	1	0	3	2	1	1
10051	101	1	3-Nov-14	14	1	13	2	1	1	0	3	2	2	2
10052	101	1	23-Feb-14	19	1	13	2	1	1	0	3	2	1	1
10053	201	1	16-Jul-14	1	1	13	2	2	1	0	3	2	1	1
10054	101	1	31-Oct-14	21	1	13	2	1	1	0	3	2	2	1
10055	101	1	24-Nov-14	16	1	13	2	1	1	0	3	2	2	1
10056	101	1	7-Mar-14	18	1	13	2	1	1	0	3	2	2	1
10057	101	1	18-Mar-14	12	1	13	2	1	1	0	3	2	2	2
10058	201	1	2-Sep-14	14	1	13	2	3	1	0	3	2	1	1
10059	101	1	23-Jan-14	20	1	13	2	1	1	0	3	2	2	1
10060	101	1	6-Sep-14	22	1	13	2	1	1	0	3	2	2	1
10061	101	1	2-Sep-14	16	1	13	2	1	1	0	3	2	3	1
10062	201	1	13-Jul-14	16	1	13	2	2	1	0	3	2	3	1
10063	101	1	28-Aug-14	13	1	13	2	1	1	0	3	2	3	1
10064	201	1	21-Apr-14	9	1	13	2	3	1	0	3	2	5	2
10065	101	1	4-May-14	11	1	13	2	1	1	0	3	2	3	1
10066	101	1	16-Sep-14	20	1	13	2	1	1	0	3	2	1	1
10067	201	1	14-Oct-14	16	1	13	2	3	1	0	3	2	4	1
10068	101	1	11-Apr-14	16	1	13	2	1	1	0	3	2	3	1
10069	101	1	27-Feb-14	19	1	13	2	1	1	0	3	2	3	1
10070	101	1	23-Nov-14	19	1	13	2	1	1	0	3	2	3	1
10071	101	1	7-Apr-14	19	1	13	2	1	1	0	3	2	3	1
10072	101	1	26-Sep-14	13	1	13	2	1	1	0	3	2	2	1
10073	201	1	4-Jan-14	9	1	13	2	2	1	0	3	2	2	1
10074	101	1	28-Feb-14	17	1	13	2	2	1	0	3	2	2	4
10075	201	1	16-Feb-14	13	1	13	2	1	1	0	3	2	3	1
10076	101	1	28-Jun-14	19	1	13	2	1	1	0	3	2	2	1
10077	101	1	27-Apr-14	19	1	13	2	1	1	0	3	2	2	1
10078	201	1	28-May-14	18	1	13	2	2	1	0	3	2	2	1
10079	101	1	21-Mar-14	11	1	13	2	1	1	0	3	2	3	1
10080	201	1	14-Apr-14	10	1	13	2	2	1	0	3	2	2	1
10081	201	1	13-Nov-14	16	1	13	2	1	1	0	3	2	3	2
10082	201	1	11-Dec-14	16	1	13	2	3	1	0	3	2	3	1
10083	101	1	11-Mar-14	17	1	13	2	1	1	0	3	2	2	1
10084	201	1	20-May-14	9	1	13	2	1	1	0	3	2	2	1
10085	101	1	9-Apr-14	14	1	13	2	1	1	0	3	2	2	2
10086	201	1	25-Feb-14	16	1	13	2	2	1	0	3	2	2	1
10087	201	1	26-Feb-14	11	1	13	2	1	1	0	3	2	2	1
10088	201	1	23-Apr-14	18	1	13	2	3	1	0	3	2	2	1
10089	101	1	4-Nov-14	17	1	13	2	1	1	0	3	2	3	1
10090	201	1	7-Jan-14	9	1	13	2	1	1	0	3	2	3	1
10091	101	1	11-Nov-14	17	1	13	2	1	1	0	3	2	4	1
10092	101	1	20-Nov-14	18	1	13	2	1	1	0	3	2	2	1
10093	101	1	9-Oct-14	10	1	13	2	1	1	0	3	2	2	1
10094	201	1	10-Oct-14	19	1	13	2	2	1	0	3	2	3	1
10095	201	1	28-Feb-13	9	1	13	2	1	1	0	3	2	1	1
10096	201	1	21-Oct-13	15	1	13	2	3	1	0	3	2	4	1
10097	201	1	11-Mar-13	15	1	13	2	2	1	0	3	2	2	1
10098	101	1	14-Mar-13	12	1	13	2	1	1	0	3	2	1	1
10099	101	1	13-Sep-13	7	1	13	2	1	1	0	3	2	1	1
10100	201	1	5-Dec-13	9	1	13	2	1	1	0	3	2	1	1
10101	101	1	23-Oct-13	18	1	13	2	1	1	0	3	2	2	1
10102	101	1	8-Jan-13	17	1	13	2	1	1	0	3	2	2	1
10103	101	1	18-Dec-13	12	1	13	2	1	1	0	3	2	3	1
10104	101	1	27-Sep-13	16	1	13	2	1	1	0	3	2	3	4
10105	101	1	5-Jan-13	14	1	13	2	1	1	0	3	2	3	1
10106	101	1	28-Nov-13	13	1	13	2	1	1	0	3	2	3	1
10107	101	1	2-Apr-13	10	1	13	2	1	1	0	3	2	2	1
10108	201	1	29-Sep-13	17	1	13	2	2	1	0	3	2	2	2
10109	101	1	7-Mar-13	18	1	13	2	1	1	0	3	2	2	1
10110	101	1	9-Jun-13	9	1	13	2	1	1	0	3	2	3	1
10111	101	1	19-Oct-13	10	1	13	2	1	1	0	3	2	4	1
10112	101	1	27-Dec-13	16	1	13	2	1	1	0	3	2	4	1
10113	101	1	20-Oct-13	17	1	13	2	1	1	0	3	2	1	2
10114	101	1	4-Apr-13	17	1	13	2	1	1	0	3	2	1	1
10115	101	1	21-Jun-13	22	1	13	2	3	1	0	3	2	1	1
10116	201	1	23-Mar-13	20	1	13	2	3	1	0	3	2	5	1
10117	201	1	25-Feb-13	19	1	13	2	2	1	0	3	2	1	1
10118	101	1	18-May-13	14	1	13	2	3	1	0	3	2	1	2
10119	101	1	9-Jan-13	11	1	13	2	1	1	0	3	2	5	1
10120	101	1	20-Nov-13	16	1	13	2	1	1	0	3	2	1	1
10121	101	1	14-Nov-13	16	1	13	2	1	1	0	3	2	1	1
10122	101	1	20-Nov-13	21	1	13	2	1	1	0	3	2	1	1
10123	101	1	24-Apr-13	10	1	13	2	1	1	0	3	2	4	1
10124	101	1	8-Sep-13	15	1	13	2	1	1	0	3	2	4	1
10125	101	1	31-May-13	20	1	13	2	1	1	0	3	2	2	1
10126	101	1	9-Jul-13	18	1	13	2	1	1	0	3	2	2	4
10127	101	1	8-Jun-13	17	1	13	2	1	1	0	3	2	2	1
10128	101	1	1-Mar-13	15	1	13	2	1	1	0	3	2	3	1
10129	101	1	25-Feb-13	16	1	13	2	1	1	0	3	2	3	1
10130	201	1	20-May-13	16	1	13	2	1	1	0	3	2	2	1
10131	101	1	16-Jun-13	19	1	13	2	1	1	0	3	2	2	1
10132	201	1	23-May-13	22	1	13	2	3	1	0	3	2	2	1
10133	101	1	17-Feb-13	10	1	13	2	1	1	0	3	2	3	1
10134	201	1	11-Aug-13	15	1	13	2	1	1	0	3	2	3	1
10135	101	1	15-Nov-13	18	1	13	2	1	1	0	3	2	3	1
10136	201	1	9-Oct-13	19	1	13	2	1	1	0	3	2	3	1
10137	101	1	23-Nov-13	12	1	13	2	1	1	0	3	2	2	1
10138	101	1	11-Jun-13	8	1	13	2	1	1	0	3	2	2	1
10139	101	1	4-Mar-13	12	1	13	2	1	1	0	3	2	2	1
10140	101	1	7-Mar-13	19	1	13	2	1	1	0	3	2	2	1
10141	101	1	24-Nov-13	15	1	13	2	1	1	0	3	2	2	1
10142	101	1	11-Jun-13	10	1	13	2	1	1	0	3	2	2	1
10143	101	1	9-Sep-13	21	1	13	2	1	1	0	3	2	2	1
10144	101	1	14-Mar-13	17	1	13	2	1	1	0	3	2	1	1
10145	101	1	21-Apr-13	9	1	13	2	1	1	0	3	2	2	1
10146	101	1	4-Nov-13	20	1	13	2	1	1	0	3	2	2	1
10147	201	1	19-Feb-13	20	1	13	2	3	1	0	3	2	1	2
10148	101	1	4-May-13	18	1	13	2	1	1	0	3	2	3	1
10149	101	1	28-Nov-13	16	1	13	2	1	1	0	3	2	3	1
10150	101	1	16-Oct-13	15	1	13	2	1	1	0	3	2	3	1
10151	101	1	20-Jun-13	16	1	13	2	1	1	0	3	2	3	1
10152	101	1	23-Nov-13	11	1	13	2	1	1	0	3	2	2	1
10153	101	1	14-May-13	17	1	13	2	1	1	0	3	2	5	4
10154	101	1	26-Jun-13	12	1	13	2	1	1	0	3	2	3	4
10155	101	1	15-Jun-13	17	1	13	2	1	1	0	3	2	2	1
10156	101	1	18-Oct-13	18	1	13	2	1	1	0	3	2	2	1
10157	101	1	18-Feb-13	16	1	13	2	1	1	0	3	2	2	1
10158	101	1	20-Apr-13	19	1	13	2	1	1	0	3	2	2	1
10159	101	1	22-Mar-13	22	1	13	2	1	1	0	3	2	2	4
10160	201	1	6-Nov-13	17	1	13	2	1	1	0	3	2	1	1
10161	101	1	20-May-13	16	1	13	2	1	1	0	3	2	2	4

10162	201	1	7-Jan-13	17	1	13	2	2	1	0	3	2	3	1
10163	101	1	26-Sep-13	10	1	13	2	1	1	0	3	2	3	2
10164	201	1	30-Jun-13	10	1	13	2	2	1	0	3	2	2	2
10165	101	1	5-Jun-13	10	1	13	2	1	1	0	3	2	3	4
10166	101	1	7-Dec-12	11	1	13	2	1	1	0	3	2	3	1
10167	101	1	7-Nov-12	9	1	13	2	3	1	0	3	2	4	4
10168	201	1	14-Jun-12	11	1	13	2	1	1	0	3	2	6	1
10169	101	1	24-Oct-12	15	1	13	2	1	1	0	3	2	1	1
10170	101	1	8-Mar-12	14	1	13	2	1	1	0	3	2	1	1
10171	201	1	18-Jun-12	14	1	13	2	2	1	0	3	2	4	1
10172	101	1	29-Feb-12	10	1	13	2	1	1	0	3	2	4	2
10173	101	1	14-Dec-12	9	1	13	2	1	1	0	3	2	3	2
10174	101	1	27-Apr-12	15	1	13	2	1	1	0	3	2	2	4
10175	101	1	2-Mar-12	16	1	13	2	1	1	0	3	2	2	4
10176	101	1	16-Nov-12	12	1	13	2	1	1	0	3	2	3	1
10177	101	1	22-Mar-12	16	1	13	2	1	1	0	3	2	2	1
10178	101	1	5-Mar-12	18	1	13	2	1	1	0	3	2	3	1
10179	101	1	12-Feb-12	15	1	13	2	1	1	0	3	2	5	1
10180	201	1	10-Nov-12	18	1	13	2	2	1	0	3	2	2	1
10181	201	1	27-May-12	23	1	13	2	2	1	0	3	2	2	1
10182	101	1	16-Mar-12	15	1	13	2	1	1	0	3	2	2	1
10183	101	1	11-Nov-12	17	1	13	2	1	1	0	3	2	1	1
10184	101	1	10-Nov-12	21	1	13	2	3	1	0	3	2	5	1
10185	101	1	22-Nov-12	7	1	13	2	1	1	0	3	2	5	1
10186	101	1	31-Mar-12	10	1	13	2	1	1	0	3	2	1	4
10187	101	1	5-Nov-12	16	1	13	2	1	1	0	3	2	1	1
10188	101	1	8-Oct-12	10	1	13	2	1	1	0	3	2	1	1
10189	101	1	15-May-12	18	1	13	2	1	1	0	3	2	1	2
10190	101	1	9-May-12	14	1	13	2	1	1	0	3	2	1	1
10191	101	1	4-Feb-12	16	1	13	2	1	1	0	3	2	1	1
10192	101	1	21-Jun-12	20	1	13	2	1	1	0	3	2	1	1
10193	101	1	13-Feb-12	20	1	13	2	1	1	0	3	2	1	1
10194	101	1	6-Sep-12	15	1	13	2	1	1	0	3	2	2	1
10195	101	1	23-May-12	17	1	13	2	1	1	0	3	2	2	1
10196	101	1	1-Nov-12	16	1	13	2	1	1	0	3	2	3	1
10197	101	1	19-Aug-12	7	1	13	2	1	1	0	3	2	3	1
10198	201	1	10-Oct-12	21	1	13	2	3	1	0	3	2	2	1
10199	101	1	30-Dec-12	17	1	13	2	1	1	0	3	2	3	1
10200	101	1	25-Apr-12	19	1	13	2	1	1	0	3	2	3	1
10201	101	1	11-Sep-12	17	1	13	2	1	1	0	3	2	5	1
10202	101	1	30-Aug-12	10	1	13	2	1	1	0	3	2	2	1
10203	101	1	23-Jun-12	16	1	13	2	1	1	0	3	2	1	2
10204	101	1	13-Oct-12	21	1	13	2	1	1	0	3	2	2	1
10205	101	1	1-Aug-12	11	1	13	2	1	1	0	3	2	2	1
10206	101	1	19-Mar-12	17	1	13	2	1	1	0	3	2	2	1
10207	101	1	16-Mar-12	11	1	13	2	1	1	0	3	2	2	1
10208	101	1	24-Mar-12	10	1	13	2	1	1	0	3	2	2	1
10209	101	1	12-Nov-12	15	1	13	2	1	1	0	3	2	2	1
10210	201	1	18-Jun-12	12	1	13	2	2	1	0	3	2	2	1
10211	101	1	15-Jun-12	16	1	13	2	1	1	0	3	2	3	1
10212	101	1	7-Sep-12	20	1	13	2	1	1	0	3	2	3	1
10213	101	1	25-Sep-12	10	1	13	2	1	1	0	3	2	2	1
10214	201	1	3-Dec-12	17	1	13	2	3	1	0	3	2	3	1
10215	101	1	3-Sep-12	15	1	13	2	1	1	0	3	2	2	1
10216	201	1	30-May-12	13	1	13	2	1	1	0	3	2	5	1
10217	101	1	2-Jul-12	16	1	13	2	1	1	0	3	2	3	1
10218	101	1	3-Jul-12	16	1	13	2	1	1	0	3	2	3	1
10219	201	1	7-Dec-12	14	1	13	2	1	1	0	3	2	1	1
10220	201	1	4-May-12	15	1	13	2	1	1	0	3	2	1	1
10221	101	1	19-Jun-12	12	1	13	2	2	1	0	3	2	3	1
10222	101	1	28-Oct-12	10	1	13	2	1	1	0	3	2	3	1
10223	201	1	11-Oct-12	9	1	13	2	2	1	0	3	2	2	2
10224	101	1	23-Nov-12	16	1	13	2	1	1	0	3	2	2	4
10225	101	1	12-Apr-12	15	1	13	2	1	1	0	3	2	2	4
10226	201	1	29-Jan-12	13	1	13	2	1	1	0	3	2	3	1
10227	201	1	24-Sep-12	10	1	13	2	3	1	0	3	2	2	1
10228	201	1	11-Oct-12	16	1	13	2	2	1	0	3	2	2	1
10229	101	1	19-Jun-12	16	1	13	2	1	1	0	3	2	3	1
10230	101	1	22-May-12	16	1	13	2	1	1	0	3	2	5	1
10231	101	1	21-Apr-12	15	1	13	2	1	1	0	3	2	2	4
10232	101	1	20-Feb-11	10	1	13	2	1	1	0	3	2	3	1
10233	101	1	3-May-11	16	1	13	2	1	1	0	3	2	2	1
10234	101	1	29-Dec-11	18	1	13	2	1	1	0	3	2	1	1
10235	201	1	14-Oct-11	18	1	13	2	1	1	0	3	2	1	1
10236	101	1	28-Jun-11	12	1	13	2	1	1	0	3	2	1	1
10237	201	1	11-Oct-11	10	1	13	2	3	1	0	3	2	1	1
10238	101	1	22-Nov-11	20	1	13	2	1	1	0	3	2	2	1
10239	101	1	29-Sep-11	10	1	13	2	1	1	0	3	2	2	1
10240	101	1	29-Dec-11	20	1	13	2	1	1	0	3	2	5	1
10241	101	1	3-Dec-11	10	1	13	2	1	1	0	3	2	7	4
10242	201	1	13-Apr-11	18	1	13	2	1	1	0	3	2	6	1
10243	101	1	4-Sep-11	16	1	13	2	1	1	0	3	2	2	1
10244	101	1	9-Jan-11	21	1	13	2	1	1	0	3	2	1	1
10245	101	1	10-Mar-11	16	1	13	2	1	1	0	3	2	2	1
10246	101	1	22-Sep-11	11	1	13	2	1	1	0	3	2	3	1
10247	101	1	28-Apr-11	17	1	13	2	1	1	0	3	2	3	1
10248	101	1	30-Sep-11	7	1	13	2	1	1	0	3	2	3	1
10249	101	1	14-Jul-11	22	1	13	2	1	1	0	3	2	2	1
10250	101	1	26-Jan-11	17	1	13	2	1	1	0	3	2	3	1
10251	101	1	29-Nov-11	19	1	13	2	1	1	0	3	2	3	1
10252	101	1	8-Nov-11	17	1	13	2	1	1	0	3	2	2	1
10253	101	1	17-Feb-11	22	1	13	2	1	1	0	3	2	2	1
10254	101	1	20-Nov-11	16	1	13	2	1	1	0	3	2	2	1
10255	101	1	15-Apr-11	15	1	13	2	1	1	0	3	2	2	1
10256	101	1	8-Sep-11	12	1	13	2	1	1	0	3	2	2	1
10257	101	1	15-Oct-11	19	1	13	2	1	1	0	3	2	2	4
10258	201	1	31-Aug-11	16	1	13	2	4	1	0	3	2	2	2
10259	101	1	31-Dec-11	11	1	13	2	1	1	0	3	2	2	1
10260	101	1	8-Dec-11	17	1	13	2	1	1	0	3	2	2	1
10261	101	1	27-May-11	14	1	13	2	1	1	0	3	2	2	1
10262	101	1	17-Sep-11	12	1	13	2	1	1	0	3	2	4	1
10263	201	1	11-Mar-11	22	1	13	2	3	1	0	3	2	2	1
10264	101	1	6-May-11	23	1	13	2	1	1	0	3	2	2	1
10265	201	1	31-Jul-11	10	1	13	2	1	1	0	3	2	3	1
10266	101	1	3-Dec-11	17	1	13	2	1	1	0	3	2	4	1
10267	101	1	19-May-11	23	1	13	2	1	1	0	3	2	2	2
10268	101	1	4-Jan-11	16	1	13	2	1	1	0	3	2	3	1
10269	201	1	24-Jan-11	20	1	13	2	1	1	0	3	2	3	1
10270	101	1	17-Sep-11	21	1	13	2	2	1	0	3	2	2	1
10271	101	1	2-Jan-11	17	1	13	2	1	1	0	3	2	2	1
10272	101	1	9-Aug-11	22	1	13	2	1	1	0	3	2	3	1
10273	101	1	28-Oct-11	17	1	13	2	1	1	0	3	2	2	1
10274	101	1	14-Apr-11	17	1	13	2	1	1	0	3	2	2	1
10275	101	1	20-Jul-11	18	1	13	2	1	1	0	3	2	3	1
10276	101	1	4-Jul-11	21	1	13	2	1	1	0	3	2	2	1
10277	101	1	25-Feb-11	10	1	13	2	1	1	0	3	2	2	1
10278	201	1	14-Dec-11	16	1	13	2	2	1	0	3	2	1	1
10279	201	1	8-Jun-11	14	1	13	2	1	1	0	3	2	1	1
10280	101	1	24-Oct-11	11	1	13	2	1	1	0	3	2	1	1
10281	201	1	22-Nov-11	9	1	13	2	3	1	0	3	2	4	1
10282	101	1	4-Nov-11	12	1	13	2	1	1	0	3	2	2	4
10283	201	1	8-Jun-11	16	1	13	2	1	1	0	3	2	2	1
10284	101	1	23-May-11	11	1	13	2	1	1	0	3	2	2	1
10285	101	1	21-Feb-11	15	1	13	2	1	1	0	3	2	2	4

10286	101	1	7-May-11	9	1	13	2	2	1	0	3	2	2	1
10287	101	1	22-Apr-11	16	1	13	2	2	1	0	3	2	4	1
10288	101	1	11-Mar-11	12	1	13	2	1	1	0	3	2	2	1
10289	201	1	8-Feb-11	11	1	13	2	3	1	0	3	2	1	1
10290	201	1	13-Aug-11	14	1	13	2	3	1	0	3	2	3	1
10291	101	1	13-May-10	10	1	13	2	1	1	0	3	2	5	2
10292	101	1	10-Mar-10	10	1	13	2	1	1	0	3	2	1	2
10293	201	1	7-Apr-10	8	1	13	2	2	1	0	3	2	1	2
10294	201	1	20-Apr-10	16	1	13	2	1	1	0	3	2	1	1
10295	201	1	1-Dec-10	7	1	13	2	2	1	0	3	2	1	1
10296	101	1	17-Jun-10	10	1	13	2	1	1	0	3	2	1	1
10297	101	1	26-Feb-10	8	1	13	2	1	1	0	3	2	3	2
10298	101	1	25-May-10	16	1	13	2	1	1	0	3	2	2	2
10299	201	1	23-Nov-10	22	1	13	2	1	1	0	3	2	3	1
10300	101	1	21-Jun-10	17	1	13	2	1	1	0	3	2	5	1
10301	101	1	21-Apr-10	17	1	13	2	1	1	0	3	2	1	1
10302	101	1	11-Oct-10	15	1	13	2	1	1	0	3	2	2	1
10303	201	1	1-Oct-10	7	1	13	2	3	1	0	3	2	3	1
10304	101	1	7-Jan-10	16	1	13	2	1	1	0	3	2	2	1
10305	101	1	13-Jun-10	17	1	13	2	1	1	0	3	2	3	1
10306	101	1	26-Dec-10	14	1	13	2	1	1	0	3	2	1	1
10307	101	1	26-Nov-10	17	1	13	2	1	1	0	3	2	3	1
10308	101	1	9-Apr-10	16	1	13	2	1	1	0	3	2	1	1
10309	101	1	5-Oct-10	17	1	13	2	1	1	0	3	2	2	2
10310	101	1	10-Dec-10	12	1	13	2	2	1	0	3	2	2	1
10311	101	1	5-Nov-10	17	1	13	2	1	1	0	3	2	2	1
10312	101	1	24-Apr-10	20	1	13	2	1	1	0	3	2	2	1
10313	101	1	23-Dec-10	20	1	13	2	1	1	0	3	2	2	4
10314	101	1	9-Apr-10	18	1	13	2	1	1	0	3	2	1	1
10315	101	1	8-Sep-10	19	1	13	2	1	1	0	3	2	2	1
10316	101	1	11-Nov-10	17	1	13	2	1	1	0	3	2	2	1
10317	101	1	26-Jul-10	22	1	13	2	2	1	0	3	2	1	1
10318	101	1	4-May-10	17	1	13	2	1	1	0	3	2	1	1
10319	101	1	18-Dec-10	21	1	13	2	1	1	0	3	2	1	1
10320	101	1	1-Mar-10	19	1	13	2	1	1	0	3	2	3	1
10321	101	1	2-Sep-10	18	1	13	2	1	1	0	3	2	3	4
10322	101	1	24-Nov-10	17	1	13	2	1	1	0	3	2	3	1
10323	101	1	6-Dec-10	12	1	13	2	1	1	0	3	2	3	1
10324	101	1	24-May-10	17	1	13	2	1	1	0	3	2	2	1
10325	101	1	7-Dec-10	16	1	13	2	1	1	0	3	2	2	1
10326	101	1	9-Nov-10	19	1	13	2	1	1	0	3	2	3	1
10327	101	1	9-Dec-10	10	1	13	2	1	1	0	3	2	1	1
10328	201	1	15-Jul-10	15	1	13	2	1	1	0	3	2	3	2
10329	101	1	23-Mar-10	15	1	13	2	1	1	0	3	2	3	1
10330	101	1	20-Feb-10	17	1	13	2	1	1	0	3	2	2	1
10331	201	1	10-Nov-10	14	1	13	2	2	1	0	3	2	2	1
10332	201	1	27-Oct-10	20	1	13	2	2	1	0	3	2	4	2
10333	101	1	25-Dec-09	10	1	13	2	1	1	0	3	2	3	1
10334	101	1	13-Jul-09	9	1	13	2	1	1	0	3	2	1	1
10335	101	1	10-Mar-09	9	1	13	2	1	1	0	3	2	1	1
10336	101	1	19-Feb-09	17	1	13	2	1	1	0	3	2	2	1
10337	201	1	29-Dec-09	20	1	13	2	1	1	0	3	2	3	1
10338	201	1	25-Dec-09	19	1	13	2	1	1	0	3	2	2	4
10339	101	1	2-Apr-09	12	1	13	2	2	1	0	3	2	2	1
10340	101	1	18-Jun-09	14	1	13	2	1	1	0	3	2	2	1
10341	101	1	2-Nov-09	18	1	13	2	2	1	0	3	2	5	1
10342	101	1	18-Aug-09	16	1	13	2	1	1	0	3	2	1	2
10343	101	1	9-Feb-09	18	1	13	2	1	1	0	3	2	1	1
10344	201	1	6-Jan-09	16	1	13	2	1	1	0	3	2	1	1
10345	101	1	18-Nov-09	17	1	13	2	1	1	0	3	2	1	1
10346	101	1	23-Dec-09	12	1	13	2	1	1	0	3	2	2	1
10347	201	1	21-Nov-09	17	1	13	2	1	1	0	3	2	2	4
10348	101	1	6-Sep-09	20	1	13	2	1	1	0	3	2	2	1
10349	201	1	22-Dec-09	17	1	13	2	2	1	0	3	2	1	1
10350	101	1	16-Dec-09	18	1	13	2	1	1	0	3	2	2	1
10351	201	1	18-Oct-09	21	1	13	2	2	1	0	3	2	2	2
10352	101	1	10-Nov-09	17	1	13	2	1	1	0	3	2	3	1
10353	101	1	1-Apr-09	17	1	13	2	1	1	0	3	2	1	1
10354	101	1	4-Sep-09	14	1	13	2	1	1	0	3	2	2	1
10355	101	1	30-May-09	17	1	13	2	1	1	0	3	2	3	1
10356	101	1	14-Aug-09	20	1	13	2	1	1	0	3	2	2	1
10357	101	1	19-Apr-09	6	1	13	2	3	1	0	3	2	2	1
10358	201	1	5-May-09	19	1	13	2	1	1	0	3	2	2	1
10359	101	1	24-Nov-09	16	1	13	2	1	1	0	3	2	2	1
10360	101	1	29-Apr-09	17	1	13	2	1	1	0	3	2	3	1
10361	201	1	19-May-09	8	1	13	2	3	1	0	3	2	2	1
10362	101	1	6-Nov-09	15	1	13	2	1	1	0	3	2	3	1
10363	101	1	4-Oct-09	20	1	13	2	2	1	0	3	2	2	4
10364	201	1	18-Sep-08	10	1	13	2	3	1	0	3	2	2	1
10365	201	1	4-Sep-08	13	1	13	2	3	1	0	3	2	2	1
10366	101	1	13-Nov-08	20	1	13	2	1	1	0	3	2	3	1
10367	201	1	23-Oct-08	12	1	13	2	1	1	0	3	2	4	1
10368	101	1	11-Sep-08	10	1	13	2	1	1	0	3	2	4	1
10369	201	1	23-Dec-08	17	1	13	2	2	1	0	3	2	6	1
10370	201	1	30-Oct-08	20	1	13	2	3	1	0	3	2	2	1
10371	201	1	10-Jun-08	16	1	13	2	1	1	0	3	2	2	1
10372	101	1	12-Mar-08	11	1	13	2	1	1	0	3	2	2	1
10373	101	1	28-Nov-08	19	1	13	2	1	1	0	3	2	3	1
10374	101	1	18-Nov-08	15	1	13	2	1	1	0	3	2	4	2
10375	201	1	30-Oct-08	18	1	13	2	1	1	0	3	2	2	1
10376	201	1	30-Oct-08	19	1	13	2	1	1	0	3	2	3	1
10377	101	1	15-Dec-08	12	1	13	2	1	1	0	3	2	2	1
10378	101	1	25-Jul-08	20	1	13	2	2	1	0	3	2	1	1
10379	101	1	6-Sep-08	22	1	13	2	1	1	0	3	2	2	1
10380	101	1	20-Jan-08	9	1	13	2	1	1	0	3	2	1	1
10381	201	1	16-Aug-08	21	1	13	2	1	1	0	3	2	1	1
10382	201	1	5-Nov-08	18	1	13	2	3	1	0	3	2	2	1
10383	101	1	6-Sep-08	19	1	13	2	1	1	0	3	2	2	1
10384	101	1	25-Sep-08	22	1	13	2	1	1	0	3	2	2	1
10385	101	1	25-Dec-08	18	1	13	2	1	1	0	3	2	2	4
10386	101	1	17-Aug-08	22	1	13	2	1	1	0	3	2	2	1
10387	101	1	1-Aug-08	19	1	13	2	1	1	0	3	2	1	1
10388	101	1	16-Aug-08	18	1	13	2	2	1	0	3	2	1	1
10389	101	1	18-Mar-08	18	1	13	2	1	1	0	3	2	2	1
10390	101	1	24-Apr-08	17	1	13	2	1	1	0	3	2	2	2
10391	101	1	17-Jun-08	17	1	13	2	1	1	0	3	2	3	1
10392	201	1	27-Oct-08	15	1	13	2	2	1	0	3	2	1	1
10393	101	1	28-Sep-08	13	1	13	2	1	1	0	3	2	1	1
10394	101	1	5-Nov-08	14	1	13	2	1	1	0	3	2	1	1
10395	101	1	16-Apr-08	17	1	13	2	1	1	0	3	2	2	2
10396	201	1	17-May-08	18	1	13	2	1	1	0	3	2	3	2
10397	101	1	19-Sep-08	17	1	13	2	2	1	0	3	2	2	1
10398	101	1	8-Apr-08	17	1	13	2	1	1	0	3	2	2	1
10399	201	1	17-Nov-08	18	1	13	2	2	1	0	3	2	2	1
10400	101	1	14-Jan-08	16	1	13	2	1	1	0	3	2	2	2
10401	201	1	18-Jun-08	13	1	13	2	1	1	0	3	2	2	1
10402	101	1	13-May-08	17	1	13	2	1	1	0	3	2	2	1
10403	101	1	18-Dec-08	16	1	13	2	2	1	0	3	2	2	2
10404	201	1	18-Sep-07	8	1	13	2	1	1	0	3	2	1	1
10405	101	1	19-Oct-07	18	1	13	2	1	1	0	3	2	1	2
10406	201	1	3-Sep-07	10	1	13	2	1	1	0	3	2	2	1
10407	101	1	21-May-07	14	1	13	2	1	1	0	3	2	1	1
10408	201	1	14-Jul-07	12	1	13	2	1	1	0	3	2	2	1
10409	201	1	15-May-07	12	1	13	2	3	1	0	3	2	2	1

10410	201	1	20-Sep-07	23	1	13	2	1	1	0	3	2	3	1
10411	101	1	28-May-07	15	1	13	2	1	1	0	3	2	2	1
10412	101	1	3-Jul-07	16	1	13	2	1	1	0	3	2	3	1
10413	101	1	13-Dec-07	18	1	13	2	1	1	0	3	2	3	1
10414	101	1	3-Oct-07	16	1	13	2	1	1	0	3	2	2	1
10415	101	1	29-Nov-07	17	1	13	2	1	1	0	3	2	1	1
10416	101	1	27-May-07	10	1	13	2	1	1	0	3	2	3	1
10417	101	1	18-Nov-07	18	1	13	2	1	1	0	3	2	3	1
10418	101	1	2-Aug-07	19	1	13	2	1	1	0	3	2	2	1
10419	101	1	9-Sep-07	16	1	13	2	1	1	0	3	2	2	1
10420	201	1	24-Apr-07	18	1	13	2	1	1	0	3	2	3	2
10421	101	1	26-Aug-07	13	1	13	2	1	1	0	3	2	2	1
10422	101	1	9-Apr-07	17	1	13	2	1	1	0	3	2	2	1
10423	101	1	30-Sep-07	20	1	13	2	1	1	0	3	2	2	1
10424	101	1	2-Sep-07	11	1	13	2	1	1	0	3	2	3	1
10425	101	1	17-May-07	17	1	13	2	1	1	0	3	2	1	1
10426	201	1	1-Jun-07	16	1	13	2	1	1	0	3	2	2	1
10427	201	1	26-Mar-07	16	1	13	2	2	1	0	3	2	2	1
10428	101	1	12-Jun-07	22	1	13	2	1	1	0	3	2	3	1
10429	201	1	14-Jul-07	14	1	13	2	2	1	0	3	2	2	1
10430	201	1	26-Jun-07	14	1	13	2	1	1	0	3	2	3	1
10431	101	1	28-Mar-07	17	1	13	2	1	1	0	3	2	2	1
10432	101	1	5-Dec-07	17	1	13	2	1	1	0	3	2	2	1
10433	101	1	11-May-07	10	1	13	2	1	1	0	3	2	2	1
10434	101	1	5-Jul-07	17	1	13	2	1	1	0	3	2	2	1
10435	101	1	29-Sep-07	17	1	13	2	1	1	0	3	2	3	1
10436	201	1	2-Sep-07	9	1	13	2	2	1	0	3	2	3	1
10437	101	1	21-Oct-06	8	1	13	2	1	1	0	3	2	1	1
10438	101	1	21-Feb-06	17	1	13	2	1	1	0	3	2	4	1
10439	101	1	30-Oct-06	11	1	13	2	1	1	0	3	2	4	1
10440	101	1	27-Nov-06	16	1	13	2	1	1	0	3	2	1	1
10441	101	1	29-Nov-06	17	1	13	2	3	1	0	3	2	2	1
10442	101	1	30-Mar-06	7	1	13	2	1	1	0	3	2	2	2
10443	201	1	5-Apr-06	19	1	13	2	1	1	0	3	2	3	1
10444	101	1	11-Dec-06	15	1	13	2	1	1	0	3	2	1	1
10445	201	1	21-Sep-06	12	1	13	2	2	1	0	3	2	2	1
10446	101	1	5-Dec-06	11	1	13	2	1	1	0	3	2	2	2
10447	101	1	29-Apr-06	9	1	13	2	1	1	0	3	2	2	1
10448	101	1	14-Sep-06	15	1	13	2	1	1	0	3	2	2	1
10449	101	1	18-Aug-06	17	1	13	2	1	1	0	3	2	1	1
10450	101	1	6-Feb-06	15	1	13	2	1	1	0	3	2	2	2
10451	101	1	31-Oct-06	16	1	13	2	1	1	0	3	2	2	1
10452	201	1	26-Jul-06	16	1	13	2	3	1	0	3	2	1	1
10453	201	1	5-May-06	17	1	13	2	2	1	0	3	2	2	1
10454	101	1	8-Dec-06	16	1	13	2	1	1	0	3	2	2	1
10455	101	1	15-Aug-06	10	1	13	2	1	1	0	3	2	3	1
10456	101	1	8-Sep-06	11	1	13	2	1	1	0	3	2	2	1
10457	101	1	25-May-06	12	1	13	2	1	1	0	3	2	1	1
10458	101	1	21-Jul-06	11	1	13	2	3	1	0	3	2	2	1
10459	201	1	30-Nov-06	19	1	13	2	2	1	0	3	2	2	1
10460	201	1	31-Aug-06	16	1	13	2	1	1	0	3	2	3	1
10461	101	1	15-Feb-06	14	1	13	2	1	1	0	3	2	2	1
10462	101	1	19-Oct-06	14	1	13	2	1	1	0	3	2	3	1
10463	101	1	3-Jul-06	20	1	13	2	1	1	0	3	2	2	1
10464	101	1	17-Nov-17	23	1	13	2	1	1	0	3	2	5	1
10465	101	1	29-Mar-17	12	1	13	2	1	1	0	3	2	4	1
10466	101	1	27-Apr-17	15	1	13	2	1	1	0	3	2	3	1
10467	101	1	17-Feb-17	17	1	13	2	1	1	0	3	2	3	1
10468	101	1	18-Apr-17	11	1	13	2	1	1	0	3	2	3	1
10469	101	1	5-Jan-17	11	1	13	2	1	1	0	3	2	3	1
10470	101	1	27-Feb-17	13	1	13	2	1	1	0	3	2	3	1
10471	201	1	29-Sep-17	17	1	13	2	1	1	0	3	2	3	1
10472	201	1	17-Jun-17	19	1	13	2	1	1	0	3	2	3	1
10473	201	1	30-Aug-17	18	1	13	2	1	1	0	3	2	3	1
10474	101	1	14-Jun-17	16	1	13	2	1	1	0	3	2	2	1
10475	101	1	28-Apr-17	22	1	13	2	1	1	0	3	2	2	1
10476	101	1	6-Jan-17	9	1	13	2	1	1	0	3	2	2	1
10477	101	1	28-Feb-17	17	1	13	2	1	1	0	3	2	2	1
10478	101	1	26-Sep-17	12	1	13	2	1	1	0	3	2	2	1
10479	101	1	20-Sep-17	21	1	13	2	1	1	0	3	2	2	1
10480	201	1	18-May-17	19	1	13	2	1	1	0	3	2	2	1
10481	201	1	17-May-17	11	1	13	2	1	1	0	3	2	2	1
10482	201	1	27-Apr-17	18	1	13	2	1	1	0	3	2	2	1
10483	101	1	12-Feb-17	10	1	13	2	1	1	0	3	2	1	1
10484	101	1	24-May-17	7	1	13	2	1	1	0	3	2	1	1
10485	101	1	17-Apr-17	17	1	13	2	1	1	0	3	2	1	1
10486	101	1	27-Jan-17	15	1	13	2	1	1	0	3	2	1	1
10487	201	1	9-Jun-17	9	1	13	2	1	1	0	3	2	1	1
10488	101	1	19-May-17	21	1	13	2	1	1	0	3	2	3	2
10489	101	1	24-Aug-17	19	1	13	2	1	1	0	3	2	2	2
10490	101	1	10-Apr-17	13	1	13	2	1	1	0	3	2	2	2
10491	101	1	16-May-17	17	1	13	2	1	1	0	3	2	2	2
10492	201	1	23-Nov-17	16	1	13	2	2	1	0	3	2	5	1
10493	201	1	28-Apr-17	18	1	13	2	2	1	0	3	2	4	1
10494	101	1	14-Apr-17	8	1	13	2	2	1	0	3	2	3	1
10495	201	1	30-May-17	8	1	13	2	2	1	0	3	2	3	1
10496	201	1	27-Apr-17	15	1	13	2	2	1	0	3	2	2	1
10497	201	1	20-Feb-17	17	1	13	2	2	1	0	3	2	2	1
10498	201	1	20-Oct-17	20	1	13	2	2	1	0	3	2	2	1
10499	201	1	17-Feb-17	18	1	13	2	2	1	0	3	2	2	2
10500	201	1	18-Sep-17	15	1	13	2	3	1	0	3	2	2	1
10501	101	1	20-Oct-17	17	1	13	2	1	1	0	3	2	3	1
10502	101	1	19-Oct-17	12	1	13	2	1	1	0	3	2	2	1
10503	101	1	21-Nov-17	19	1	13	2	1	1	0	3	2	2	1
10504	101	1	22-Nov-17	16	1	13	2	1	1	0	3	2	1	1
10505	201	1	18-Dec-17	12	1	13	2	3	1	0	3	2	5	1
10506	101	1	9-Jun-16	23	1	13	2	1	1	0	6	2	2	4
10507	101	1	27-Mar-15	11	1	13	2	1	1	0	6	2	2	1
10508	101	1	21-May-15	16	1	13	2	1	1	0	6	2	2	1
10509	101	1	2-Apr-15	17	1	13	2	1	1	0	6	2	2	1
10510	101	1	8-Feb-15	12	1	13	2	1	1	0	6	2	2	1
10511	101	1	24-Sep-15	9	1	13	2	1	1	0	6	2	2	1
10512	101	1	18-Feb-14	12	1	13	2	1	1	0	6	2	2	1
10513	101	1	25-Apr-14	10	1	13	2	1	1	0	6	2	2	1
10514	101	1	13-Oct-14	10	1	13	2	1	1	0	6	2	2	1
10515	101	1	28-Oct-14	10	1	13	2	1	1	0	6	2	2	1
10516	101	1	26-Jul-14	17	1	13	2	3	1	0	6	2	3	1
10517	101	1	17-Oct-13	17	1	13	2	1	1	0	6	2	2	1
10518	101	1	14-Jun-13	17	1	13	2	1	1	0	6	2	2	1
10519	101	1	18-Jun-13	17	1	13	2	3	1	0	6	2	2	4
10520	101	1	18-Jun-13	17	1	13	2	1	1	0	6	2	2	1
10521	101	1	15-Jul-13	22	1	13	2	3	1	0	6	2	3	1
10522	101	1	8-Aug-13	19	1	13	2	1	1	0	6	2	2	1
10523	101	1	18-Oct-13	17	1	13	2	1	1	0	6	2	3	1
10524	101	1	11-Nov-12	18	1	13	2	1	1	0	6	2	3	1
10525	101	1	12-Oct-12	19	1	13	2	1	1	0	6	2	2	1
10526	101	1	12-Apr-12	10	1	13	2	1	1	0	6	2	2	1
10527	101	1	4-Jul-12	17	1	13	2	1	1	0	6	2	2	1
10528	101	1	9-Jun-12	21	1	13	2	1	1	0	6	2	2	1
10529	101	1	6-Jul-11	18	1	13	2	2	1	0	6	2	1	1
10530	101	1	14-Nov-11	17	1	13	2	1	1	0	6	2	2	1
10531	201	1	28-Sep-11	10	1	13	2	3	1	0	6	2	3	1
10532	101	1	31-Dec-11	19	1	13	2	1	1	0	6	2	2	1
10533	101	1	17-Feb-11	21	1	13	2	1	1	0	6	2	1	1

10534	101	1	8-Nov-11	12	1	13	2	1	1	0	6	2	3	1
10535	101	1	9-Dec-11	14	1	13	2	1	1	0	6	2	1	1
10536	201	1	25-May-10	20	1	13	2	2	1	0	6	2	2	1
10537	101	1	15-Oct-10	11	1	13	2	1	1	0	6	2	2	1
10538	101	1	26-Nov-10	10	1	13	2	1	1	0	6	2	2	1
10539	101	1	30-Jun-10	10	1	13	2	1	1	0	6	2	2	4
10540	101	1	26-Apr-10	11	1	13	2	1	1	0	6	2	2	1
10541	101	1	13-Oct-10	14	1	13	2	1	1	0	6	2	2	1
10542	101	1	26-Oct-10	16	1	13	2	2	1	0	6	2	3	1
10543	101	1	25-Oct-10	22	1	13	2	1	1	0	6	2	3	1
10544	101	1	13-Apr-10	16	1	13	2	1	1	0	6	2	3	1
10545	101	1	1-Jul-08	12	1	13	2	1	1	0	6	2	4	1
10546	101	1	21-Oct-08	16	1	13	2	1	1	0	6	2	2	1
10547	101	1	12-Nov-08	10	1	13	2	1	1	0	6	2	2	1
10548	101	1	2-Sep-08	13	1	13	2	1	1	0	6	2	2	1
10549	101	1	29-Oct-07	18	1	13	2	1	1	0	6	2	2	1
10550	101	1	30-Oct-06	11	1	13	2	1	1	0	6	2	2	1
10551	101	1	6-Apr-06	18	1	13	2	1	1	0	6	2	2	1
10552	201	1	15-Nov-06	12	1	13	2	3	1	0	6	2	2	1
10553	101	1	14-Mar-06	18	1	13	2	1	1	0	6	2	3	1
10554	101	1	3-Jan-12	7	1	13	2	1	1	0	13	2	2	1
10555	201	1	10-Jul-17	15	2	13	2	3	1	0	6	2	2	1
10556	201	1	25-Jul-12	20	2	13	2	2	1	0	3	2	1	1
10557	101	1	19-Oct-17	21	2	13	2	1	1	0	3	2	6	1
10558	201	1	17-Apr-17	13	2	13	2	1	1	0	4	2	2	1
10559	201	1	22-Aug-16	7	2	13	2	3	1	0	7	2	3	1
10560	101	1	19-Nov-14	14	2	13	2	1	1	0	13	2	3	1
10561	101	1	22-Jan-13	16	2	13	2	1	1	0	13	2	1	1
10562	201	1	19-Jun-09	11	2	13	2	1	1	0	13	2	4	1
10563	101	1	7-Jan-15	13	2	13	2	1	1	0	9	2	2	1
10564	101	1	15-Apr-13	18	2	13	2	1	1	0	1	2	2	1
10565	101	1	9-Jan-16	16	2	13	2	1	1	0	4	2	1	1
10566	101	1	17-Feb-15	18	2	13	2	1	1	0	4	2	3	1
10567	101	1	6-May-15	18	2	13	2	1	1	0	4	2	3	1
10568	101	1	31-Dec-14	17	2	13	2	1	1	0	4	2	1	1
10569	201	1	3-Jul-14	18	2	13	2	3	1	0	4	2	2	1
10570	101	1	31-Mar-13	14	2	13	2	1	1	0	4	2	1	1
10571	101	1	9-Aug-13	23	2	13	2	1	1	0	4	2	1	1
10572	201	1	20-Apr-13	18	2	13	2	3	1	0	4	2	3	1
10573	101	1	19-Oct-13	20	2	13	2	1	1	0	4	2	3	1
10574	101	1	29-Oct-12	11	2	13	2	1	1	0	4	2	1	1
10575	101	1	5-Jun-12	16	2	13	2	1	1	0	4	2	3	1
10576	101	1	9-May-12	16	2	13	2	1	1	0	4	2	1	1
10577	101	1	26-Aug-12	11	2	13	2	1	1	0	4	2	2	1
10578	101	1	2-May-11	9	2	13	2	1	1	0	4	2	2	1
10579	201	1	30-Nov-10	15	2	13	2	3	1	0	4	2	1	1
10580	101	1	10-Mar-10	12	2	13	2	1	1	0	4	2	1	1
10581	101	1	18-Nov-10	10	2	13	2	1	1	0	4	2	2	1
10582	101	1	30-Mar-10	16	2	13	2	1	1	0	4	2	2	1
10583	101	1	31-Oct-08	21	2	13	2	1	1	0	4	2	3	1
10584	101	1	30-Jun-07	12	2	13	2	1	1	0	4	2	2	1
10585	101	1	22-Aug-07	17	2	13	2	1	1	0	4	2	3	1
10586	101	1	24-Aug-06	12	2	13	2	1	1	0	4	2	2	1
10587	201	1	19-Oct-06	16	2	13	2	2	1	0	4	2	4	1
10588	201	1	2-Jan-16	9	2	13	2	2	1	0	11	2	2	1
10589	201	1	23-Mar-16	22	2	13	2	1	1	0	11	2	2	1
10590	101	1	8-Jun-16	19	2	13	2	1	1	0	11	2	2	1
10591	101	1	19-Dec-15	16	2	13	2	1	1	0	11	2	2	1
10592	101	1	10-Feb-15	20	2	13	2	1	1	0	11	2	3	1
10593	101	1	27-Sep-14	17	2	13	2	1	1	0	11	2	2	1
10594	101	1	4-Apr-14	19	2	13	2	1	1	0	11	2	2	1
10595	101	1	17-Jan-14	12	2	13	2	1	1	0	11	2	2	1
10596	201	1	7-May-13	7	2	13	2	2	1	0	11	2	3	1
10597	101	1	11-Jan-13	21	2	13	2	1	1	0	11	2	1	1
10598	101	1	20-Jan-13	16	2	13	2	1	1	0	11	2	2	1
10599	101	1	3-Apr-13	22	2	13	2	1	1	0	11	2	2	1
10600	101	1	31-Jul-13	16	2	13	2	2	1	0	11	2	2	1
10601	201	1	25-Nov-13	21	2	13	2	1	1	0	11	2	3	1
10602	101	1	17-Dec-13	20	2	13	2	1	1	0	11	2	3	1
10603	101	1	12-May-12	17	2	13	2	1	1	0	11	2	2	1
10604	101	1	1-Nov-12	9	2	13	2	1	1	0	11	2	3	1
10605	101	1	19-Oct-12	11	2	13	2	1	1	0	11	2	2	1
10606	101	1	13-Jul-12	13	2	13	2	1	1	0	11	2	2	1
10607	101	1	1-Jun-12	22	2	13	2	1	1	0	11	2	3	2
10608	101	1	4-Feb-12	17	2	13	2	1	1	0	11	2	2	1
10609	101	1	9-Aug-12	22	2	13	2	1	1	0	11	2	3	1
10610	101	1	1-Jul-11	18	2	13	2	1	1	0	11	2	2	1
10611	101	1	23-Sep-11	22	2	13	2	2	1	0	11	2	3	1
10612	101	1	23-Jan-10	18	2	13	2	1	1	0	11	2	2	1
10613	201	1	6-Dec-09	12	2	13	2	1	1	0	11	2	3	1
10614	101	1	17-Sep-09	2	2	13	2	3	1	0	11	2	3	2
10615	201	1	27-Jan-09	12	2	13	2	3	1	0	11	2	2	1
10616	101	1	21-Jul-09	23	2	13	2	1	1	0	11	2	3	1
10617	201	1	13-Aug-09	23	2	13	2	1	1	0	11	2	3	1
10618	201	1	28-Jul-17	19	2	13	2	1	1	0	11	2	3	1
10619	101	1	24-Mar-16	11	2	13	2	1	1	0	3	2	4	1
10620	101	1	6-Oct-16	20	2	13	2	1	1	0	3	2	2	1
10621	101	1	27-Apr-16	17	2	13	2	1	1	0	3	2	2	4
10622	101	1	21-Apr-16	11	2	13	2	1	1	0	3	2	3	1
10623	101	1	8-Jan-16	11	2	13	2	1	1	0	3	2	2	1
10624	101	1	31-Aug-16	15	2	13	2	1	1	0	3	2	3	1
10625	201	1	2-Mar-16	18	2	13	2	3	1	0	3	2	3	1
10626	101	1	2-Mar-16	10	2	13	2	1	1	0	3	2	2	4
10627	201	1	25-Mar-16	10	2	13	2	2	1	0	3	2	3	1
10628	101	1	23-Nov-15	16	2	13	2	1	1	0	3	2	3	1
10629	101	1	9-Dec-15	11	2	13	2	1	1	0	3	2	3	1
10630	201	1	21-Apr-15	17	2	13	2	2	1	0	3	2	4	1
10631	101	1	21-May-15	19	2	13	2	1	1	0	3	2	1	1
10632	101	1	26-Oct-15	7	2	13	2	1	1	0	3	2	3	1
10633	201	1	5-Sep-15	15	2	13	2	1	1	0	3	2	1	1
10634	101	1	21-Mar-15	19	2	13	2	1	1	0	3	2	2	2
10635	201	1	3-May-15	20	2	13	2	2	1	0	3	2	3	1
10636	101	1	25-Dec-15	20	2	13	2	1	1	0	3	2	2	1
10637	101	1	25-Apr-15	18	2	13	2	1	1	0	3	2	2	1
10638	101	1	21-Apr-15	15	2	13	2	1	1	0	3	2	3	1
10639	101	1	3-Mar-15	16	2	13	2	1	1	0	3	2	2	1
10640	201	1	28-Oct-15	19	2	13	2	1	1	0	3	2	2	1
10641	101	1	15-May-15	13	2	13	2	1	1	0	3	2	3	1
10642	201	1	27-Jan-15	18	2	13	2	2	1	0	3	2	3	1
10643	101	1	23-Jan-15	17	2	13	2	1	1	0	3	2	3	1
10644	201	1	30-Nov-15	17	2	13	2	1	1	0	3	2	3	1
10645	101	1	27-May-15	17	2	13	2	1	1	0	3	2	3	1
10646	101	1	12-Apr-15	17	2	13	2	1	1	0	3	2	2	1
10647	101	1	15-Feb-15	9	2	13	2	1	1	0	3	2	3	1
10648	101	1	18-Jun-14	18	2	13	2	1	1	0	3	2	3	1
10649	201	1	19-May-14	13	2	13	2	2	1	0	3	2	4	1
10650	201	1	29-Apr-14	20	2	13	2	3	1	0	3	2	2	1
10651	101	1	21-Feb-14	15	2	13	2	1	1	0	3	2	2	1
10652	101	1	24-Mar-14	10	2	13	2	1	1	0	3	2	3	1
10653	101	1	25-Nov-14	11	2	13	2	1	1	0	3	2	5	1
10654	101	1	14-Feb-14	17	2	13	2	1	1	0	3	2	5	1
10655	101	1	11-Jan-14	12	2	13	2	1	1	0	3	2	1	1
10656	101	1	12-Dec-14	8	2	13	2	1	1	0	3	2	1	1
10657	101	1	12-Sep-14	17	2	13	2	1	1	0	3	2	4	1

10658	101	1	29-Oct-14	8	2	13	2	1	1	0	3	2	2	1
10659	101	1	20-May-14	9	2	13	2	1	1	0	3	2	2	1
10660	101	1	17-Jan-14	16	2	13	2	1	1	0	3	2	3	1
10661	101	1	19-May-14	10	2	13	2	1	1	0	3	2	3	1
10662	101	1	30-Dec-14	11	2	13	2	1	1	0	3	2	3	1
10663	201	1	13-May-14	9	2	13	2	1	1	0	3	2	3	1
10664	201	1	23-Oct-14	9	2	13	2	1	1	0	3	2	4	1
10665	101	1	13-Oct-14	11	2	13	2	1	1	0	3	2	2	1
10666	201	1	18-Nov-13	18	2	13	2	2	1	0	3	2	2	1
10667	101	1	17-May-13	16	2	13	2	1	1	0	3	2	1	1
10668	101	1	4-Dec-13	16	2	13	2	1	1	0	3	2	2	2
10669	201	1	27-Nov-13	10	2	13	2	1	1	0	3	2	2	2
10670	101	1	9-May-13	15	2	13	2	1	1	0	3	2	1	1
10671	101	1	15-Oct-13	11	2	13	2	1	1	0	3	2	2	1
10672	201	1	20-May-13	16	2	13	2	2	1	0	3	2	2	1
10673	201	1	5-Jun-13	15	2	13	2	3	1	0	3	2	3	1
10674	101	1	6-Nov-13	16	2	13	2	1	1	0	3	2	3	1
10675	201	1	3-Oct-13	15	2	13	2	2	1	0	3	2	5	1
10676	101	1	30-Sep-13	17	2	13	2	1	1	0	3	2	5	1
10677	101	1	18-Apr-13	21	2	13	2	1	1	0	3	2	1	1
10678	101	1	16-Nov-13	15	2	13	2	1	1	0	3	2	1	1
10679	101	1	14-Oct-13	17	2	13	2	1	1	0	3	2	3	1
10680	101	1	9-Nov-13	20	2	13	2	1	1	0	3	2	3	1
10681	201	1	27-Apr-13	19	2	13	2	3	1	0	3	2	3	1
10682	101	1	2-Dec-13	20	2	13	2	1	1	0	3	2	2	1
10683	201	1	18-Dec-13	9	2	13	2	1	1	0	3	2	2	1
10684	101	1	6-Nov-13	17	2	13	2	1	1	0	3	2	2	1
10685	101	1	18-Oct-13	17	2	13	2	1	1	0	3	2	2	2
10686	201	1	23-Apr-13	15	2	13	2	1	1	0	3	2	1	1
10687	101	1	26-Jun-13	11	2	13	2	1	1	0	3	2	2	1
10688	201	1	22-Apr-13	14	2	13	2	1	1	0	3	2	3	1
10689	101	1	16-Sep-13	16	2	13	2	1	1	0	3	2	2	1
10690	201	1	6-Sep-13	16	2	13	2	2	1	0	3	2	3	1
10691	201	1	23-Oct-13	9	2	13	2	1	1	0	3	2	2	1
10692	101	1	28-Apr-12	17	2	13	2	1	1	0	3	2	4	1
10693	101	1	13-May-12	17	2	13	2	1	1	0	3	2	3	1
10694	101	1	14-Dec-12	17	2	13	2	1	1	0	3	2	4	1
10695	101	1	17-Sep-12	17	2	13	2	1	1	0	3	2	5	1
10696	101	1	12-Jan-12	17	2	13	2	1	1	0	3	2	5	1
10697	101	1	29-Dec-12	12	2	13	2	1	1	0	3	2	1	1
10698	101	1	12-May-12	16	2	13	2	1	1	0	3	2	3	1
10699	101	1	2-Sep-12	21	2	13	2	1	1	0	3	2	3	1
10700	101	1	7-Aug-12	10	2	13	2	1	1	0	3	2	3	1
10701	101	1	14-Jan-12	10	2	13	2	1	1	0	3	2	3	1
10702	101	1	9-Oct-12	17	2	13	2	1	1	0	3	2	3	1
10703	101	1	24-Apr-12	17	2	13	2	1	1	0	3	2	2	1
10704	101	1	18-Mar-12	12	2	13	2	1	1	0	3	2	2	1
10705	101	1	20-Sep-12	15	2	13	2	1	1	0	3	2	3	1
10706	101	1	5-Mar-12	16	2	13	2	1	1	0	3	2	3	1
10707	201	1	29-Sep-12	16	2	13	2	1	1	0	3	2	4	1
10708	201	1	17-Oct-12	13	2	13	2	1	1	0	3	2	2	1
10709	101	1	2-Mar-12	16	2	13	2	1	1	0	3	2	3	1
10710	101	1	21-Nov-11	11	2	13	2	1	1	0	3	2	1	1
10711	101	1	22-May-11	17	2	13	2	1	1	0	3	2	2	1
10712	101	1	14-Nov-11	8	2	13	2	1	1	0	3	2	3	1
10713	101	1	15-Dec-11	18	2	13	2	1	1	0	3	2	1	1
10714	101	1	23-Jan-11	13	2	13	2	1	1	0	3	2	2	1
10715	101	1	13-Oct-11	14	2	13	2	1	1	0	3	2	3	1
10716	101	1	27-Oct-11	16	2	13	2	1	1	0	3	2	2	1
10717	201	1	8-Feb-11	11	2	13	2	3	1	0	3	2	1	1
10718	101	1	26-Nov-10	17	2	13	2	1	1	0	3	2	4	1
10719	201	1	5-Aug-10	18	2	13	2	2	1	0	3	2	3	1
10720	201	1	28-Oct-10	8	2	13	2	1	1	0	3	2	2	1
10721	201	1	24-May-10	12	2	13	2	2	1	0	3	2	3	1
10722	101	1	11-Dec-10	10	2	13	2	1	1	0	3	2	3	1
10723	101	1	17-Aug-10	17	2	13	2	1	1	0	3	2	2	1
10724	101	1	12-Jul-10	8	2	13	2	1	1	0	3	2	2	1
10725	101	1	18-Oct-10	16	2	13	2	1	1	0	3	2	2	1
10726	101	1	29-Oct-10	18	2	13	2	1	1	0	3	2	2	1
10727	101	1	22-Mar-10	17	2	13	2	1	1	0	3	2	3	1
10728	201	1	20-Jan-09	10	2	13	2	1	1	0	3	2	4	1
10729	201	1	20-Mar-09	7	2	13	2	1	1	0	3	2	5	1
10730	101	1	3-Mar-09	19	2	13	2	1	1	0	3	2	3	1
10731	101	1	19-Mar-09	13	2	13	2	1	1	0	3	2	2	1
10732	101	1	3-Sep-09	19	2	13	2	1	1	0	3	2	3	1
10733	201	1	13-Oct-09	14	2	13	2	2	1	0	3	2	1	1
10734	101	1	6-Dec-08	10	2	13	2	1	1	0	3	2	1	4
10735	201	1	24-Apr-08	19	2	13	2	2	1	0	3	2	1	1
10736	101	1	17-Mar-08	17	2	13	2	1	1	0	3	2	3	1
10737	101	1	3-Jun-08	16	2	13	2	1	1	0	3	2	2	1
10738	101	1	1-Jun-08	10	2	13	2	1	1	0	3	2	3	1
10739	101	1	4-Dec-08	11	2	13	2	1	1	0	3	2	2	1
10740	101	1	31-Oct-08	19	2	13	2	1	1	0	3	2	2	1
10741	101	1	15-Nov-08	8	2	13	2	1	1	0	3	2	1	4
10742	201	1	1-Sep-08	11	2	13	2	3	1	0	3	2	2	1
10743	101	1	6-Sep-08	18	2	13	2	1	1	0	3	2	3	1
10744	101	1	8-Dec-08	9	2	13	2	1	1	0	3	2	3	1
10745	101	1	27-Dec-07	12	2	13	2	4	1	0	3	2	1	1
10746	101	1	16-Mar-07	18	2	13	2	1	1	0	3	2	2	1
10747	101	1	7-Feb-07	17	2	13	2	1	1	0	3	2	2	1
10748	201	1	16-May-07	16	2	13	2	1	1	0	3	2	4	1
10749	101	1	10-Jun-07	15	2	13	2	1	1	0	3	2	3	1
10750	101	1	30-Oct-06	11	2	13	2	1	1	0	3	2	4	1
10751	201	1	17-May-06	9	2	13	2	1	1	0	3	2	1	2
10752	101	1	2-Jun-06	9	2	13	2	1	1	0	3	2	1	1
10753	101	1	15-Feb-06	17	2	13	2	1	1	0	3	2	3	1
10754	101	1	6-Dec-06	17	2	13	2	1	1	0	3	2	2	1
10755	101	1	20-Nov-06	16	2	13	2	1	1	0	3	2	2	1
10756	201	1	23-Jul-06	19	2	13	2	1	1	0	3	2	3	1
10757	101	1	23-Nov-06	16	2	13	2	1	1	0	3	2	3	1
10758	201	1	20-May-06	11	2	13	2	1	1	0	3	2	3	2
10759	101	1	24-Feb-17	17	2	13	2	1	1	0	3	2	3	1
10760	101	1	22-Mar-17	16	2	13	2	1	1	0	3	2	3	1
10761	101	1	6-May-17	11	2	13	2	1	1	0	3	2	3	1
10762	101	1	19-Feb-17	14	2	13	2	1	1	0	3	2	3	1
10763	101	1	23-Nov-17	16	2	13	2	1	1	0	3	2	3	1
10764	101	1	9-Dec-17	12	2	13	2	1	1	0	3	2	3	1
10765	101	1	31-Jan-17	20	2	13	2	1	1	0	3	2	3	1
10766	201	1	17-Sep-17	19	2	13	2	1	1	0	3	2	3	1
10767	201	1	19-Jul-17	12	2	13	2	1	1	0	3	2	2	1
10768	101	1	25-Apr-17	1	2	13	2	1	1	0	3	2	1	1
10769	101	1	5-Jun-17	8	2	13	2	1	1	0	3	2	1	1
10770	201	1	16-Aug-17	19	2	13	2	3	1	0	3	2	2	1
10771	101	1	21-Jan-17	16	2	13	2	4	1	0	3	2	3	1
10772	101	1	14-Dec-17	18	2	13	2	1	1	0	3	2	3	1
10773	101	1	14-Oct-17	11	2	13	2	1	1	0	3	2	3	1
10774	101	1	19-Sep-17	17	2	13	2	1	1	0	3	2	2	1
10775	101	1	3-Sep-14	16	2	13	2	1	1	0	6	2	3	1
10776	101	1	28-Mar-13	17	2	13	2	1	1	0	6	2	2	1
10777	101	1	28-Jul-13	16	2	13	2	1	1	0	6	2	3	1
10778	101	1	21-May-12	12	2	13	2	1	1	0	6	2	3	1
10779	201	1	13-Oct-09	11	2	13	2	1	1	0	6	2	2	1
10780	101	1	12-Jun-17	15	2	13	2	1	1	0	6	2	3	1
10781	101	1	18-Dec-11	10	2	13	2	1	1	0	13	2	4	1

10782	202	0	20-Jul-13	17	1	14	2	1	1	0	1	2	3	1
10783	202	0	28-Sep-17	18	1	14	2	1	1	0	1	2	3	1
10784	202	0	26-Sep-16	16	1	14	2	2	1	0	4	2	1	1
10785	202	0	23-Apr-12	0	1	14	2	1	1	0	10	2	7	2
10786	202	0	23-Jul-16	15	1	14	2	1	1	0	4	2	3	1
10787	202	0	14-May-13	16	1	14	2	1	1	0	3	2	2	1
10788	202	0	17-Sep-13	21	1	14	2	2	1	0	6	2	2	1
10789	202	0	2-Jun-17	10	1	14	2	3	1	0	4	2	1	1
10790	202	0	22-Sep-08	10	1	14	2	1	1	0	10	2	7	2
10791	202	0	18-Aug-14	15	1	14	2	3	1	0	7	2	4	1
10792	202	0	16-May-13	11	1	14	2	1	1	0	7	2	3	2
10793	202	0	18-Nov-16	8	1	14	2	1	1	0	13	2	1	1
10794	202	0	1-Apr-13	16	1	14	2	1	1	0	13	2	4	1
10795	202	0	10-Nov-08	14	1	14	2	1	1	0	13	2	3	1
10796	202	0	30-May-17	11	1	14	2	1	1	0	13	2	1	1
10797	202	0	4-Nov-12	17	1	14	2	1	1	0	1	2	5	1
10798	202	0	1-Nov-11	18	1	14	2	1	1	0	1	2	5	1
10799	202	0	15-Jan-11	19	1	14	2	1	1	0	1	2	1	2
10800	202	0	13-Apr-10	7	1	14	2	2	1	0	1	2	3	2
10801	202	0	7-Apr-09	8	1	14	2	3	1	0	1	2	6	2
10802	202	0	26-Dec-16	18	1	14	2	1	1	0	4	2	2	1
10803	202	0	16-May-16	21	1	14	2	3	1	0	4	2	1	1
10804	202	0	2-Oct-16	10	1	14	2	2	1	0	4	2	1	1
10805	202	0	6-Sep-15	21	1	14	2	1	1	0	4	2	1	1
10806	202	0	31-Dec-15	17	1	14	2	1	1	0	4	2	1	1
10807	202	0	24-Oct-15	22	1	14	2	2	1	0	4	2	1	1
10808	202	0	19-Apr-14	9	1	14	2	1	1	0	4	2	2	1
10809	202	0	17-Dec-14	21	1	14	2	1	1	0	4	2	1	1
10810	202	0	2-Jan-14	19	1	14	2	1	1	0	4	2	1	1
10811	202	0	30-Apr-14	17	1	14	2	1	1	0	4	2	1	1
10812	202	0	30-May-14	12	1	14	2	1	1	0	4	2	1	1
10813	202	0	16-Dec-14	7	1	14	2	2	1	0	4	2	2	1
10814	202	0	22-May-14	1	1	14	2	1	1	0	4	2	1	2
10815	202	0	27-Feb-14	19	1	14	2	1	1	0	4	2	2	1
10816	202	0	24-Mar-14	12	1	14	2	1	1	0	4	2	3	1
10817	202	0	25-Nov-13	20	1	14	2	1	1	0	4	2	1	1
10818	202	0	16-Apr-13	11	1	14	2	1	1	0	4	2	1	1
10819	202	0	11-Sep-13	23	1	14	2	1	1	0	4	2	1	1
10820	202	0	5-Jun-13	19	1	14	2	2	1	0	4	2	1	1
10821	202	0	3-Jun-13	22	1	14	2	1	1	0	4	2	3	2
10822	202	0	27-Sep-12	13	1	14	2	1	1	0	4	2	4	1
10823	202	0	22-Mar-11	8	1	14	2	1	1	0	4	2	1	2
10824	202	0	14-Dec-11	14	1	14	2	1	1	0	4	2	1	1
10825	202	0	15-Aug-11	21	1	14	2	1	1	0	4	2	1	1
10826	202	0	23-Dec-11	15	1	14	2	1	1	0	4	2	1	1
10827	202	0	21-Oct-10	11	1	14	2	1	1	0	4	2	7	1
10828	202	0	5-Nov-10	18	1	14	2	2	1	0	4	2	1	2
10829	202	0	21-Oct-10	12	1	14	2	3	1	0	4	2	1	1
10830	202	0	3-Jun-10	11	1	14	2	3	1	0	4	2	2	2
10831	202	0	30-Jan-10	19	1	14	2	3	1	0	4	2	7	2
10832	202	0	15-Sep-10	13	1	14	2	2	1	0	4	2	6	1
10833	202	0	15-Sep-10	10	1	14	2	1	1	0	4	2	5	1
10834	202	0	27-Sep-09	18	1	14	2	1	1	0	4	2	6	1
10835	202	0	27-Mar-09	21	1	14	2	1	1	0	4	2	1	1
10836	202	0	31-Aug-09	18	1	14	2	1	1	0	4	2	1	2
10837	202	0	28-Sep-08	17	1	14	2	1	1	0	4	2	6	1
10838	202	0	2-Dec-08	18	1	14	2	1	1	0	4	2	1	1
10839	202	0	14-Jan-08	17	1	14	2	1	1	0	4	2	1	1
10840	202	0	20-Sep-07	15	1	14	2	1	1	0	4	2	4	1
10841	202	0	22-Nov-07	15	1	14	2	1	1	0	4	2	1	2
10842	202	0	3-Sep-07	13	1	14	2	1	1	0	4	2	7	2
10843	202	0	23-Nov-07	20	1	14	2	1	1	0	4	2	1	1
10844	202	0	20-Jun-07	10	1	14	2	1	1	0	4	2	1	1
10845	202	0	29-Apr-06	16	1	14	2	1	1	0	4	2	2	1
10846	202	0	19-Dec-06	8	1	14	2	1	1	0	4	2	3	1
10847	202	0	30-Dec-06	9	1	14	2	1	1	0	4	2	1	1
10848	202	0	29-May-06	19	1	14	2	1	1	0	4	2	1	1
10849	202	0	27-Mar-06	18	1	14	2	1	1	0	4	2	3	1
10850	202	0	24-Jan-17	11	1	14	2	1	1	0	4	2	1	1
10851	202	0	11-Apr-17	9	1	14	2	2	1	0	4	2	4	1
10852	202	0	19-Aug-17	18	1	14	2	2	1	0	4	2	3	2
10853	202	0	19-Oct-17	21	1	14	2	3	1	0	4	2	1	1
10854	202	0	9-Apr-16	14	1	14	2	2	1	0	11	2	2	1
10855	202	0	1-Dec-16	19	1	14	2	3	1	0	11	2	2	1
10856	202	0	17-Jun-15	6	1	14	2	1	1	0	11	2	2	1
10857	202	0	6-Nov-15	16	1	14	2	1	1	0	11	2	3	1
10858	202	0	10-Jul-12	20	1	14	2	1	1	0	11	2	2	2
10859	202	0	7-Nov-11	16	1	14	2	1	1	0	11	2	3	2
10860	202	0	16-Sep-11	13	1	14	2	2	1	0	11	2	3	2
10861	202	0	18-Jul-11	19	1	14	2	1	1	0	11	2	3	1
10862	202	0	17-Jan-10	15	1	14	2	1	1	0	11	2	3	2
10863	202	0	27-Nov-10	19	1	14	2	1	1	0	11	2	1	2
10864	202	0	3-May-08	21	1	14	2	1	1	0	11	2	5	1
10865	202	0	16-Sep-07	20	1	14	2	2	1	0	11	2	3	2
10866	202	0	28-Oct-17	15	1	14	2	1	1	0	11	2	3	1
10867	202	0	22-Dec-17	19	1	14	2	2	1	0	11	2	3	1
10868	202	0	23-Apr-16	22	1	14	2	3	1	0	3	2	1	1
10869	202	0	20-Oct-16	17	1	14	2	1	1	0	3	2	3	1
10870	202	0	11-Jan-16	18	1	14	2	3	1	0	3	2	3	1
10871	202	0	15-Jun-16	16	1	14	2	3	1	0	3	2	1	1
10872	202	0	17-May-16	19	1	14	2	1	1	0	3	2	2	2
10873	202	0	25-Dec-16	20	1	14	2	3	1	0	3	2	2	1
10874	202	0	6-Jun-16	20	1	14	2	1	1	0	3	2	2	1
10875	202	0	14-Nov-16	14	1	14	2	1	1	0	3	2	2	1
10876	202	0	14-Feb-16	19	1	14	2	1	1	0	3	2	3	1
10877	202	0	17-Nov-16	15	1	14	2	1	1	0	3	2	1	1
10878	202	0	21-Sep-16	19	1	14	2	3	1	0	3	2	4	2
10879	202	0	13-Jan-16	13	1	14	2	1	1	0	3	2	3	1
10880	202	0	24-Jun-16	16	1	14	2	2	1	0	3	2	2	1
10881	202	0	25-Nov-16	16	1	14	2	2	1	0	3	2	3	4
10882	202	0	11-Nov-15	18	1	14	2	2	1	0	3	2	1	1
10883	202	0	18-Dec-14	17	1	14	2	3	1	0	3	2	3	2
10884	202	0	17-Oct-14	9	1	14	2	1	1	0	3	2	4	1
10885	202	0	27-Oct-14	14	1	14	2	1	1	0	3	2	1	1
10886	202	0	29-Sep-14	15	1	14	2	1	1	0	3	2	1	1
10887	202	0	27-Oct-14	17	1	14	2	1	1	0	3	2	1	1
10888	202	0	5-Dec-14	16	1	14	2	3	1	0	3	2	5	1
10889	202	0	11-May-14	16	1	14	2	1	1	0	3	2	1	1
10890	202	0	25-Nov-14	21	1	14	2	1	1	0	3	2	2	2
10891	202	0	23-May-14	18	1	14	2	1	1	0	3	2	2	2
10892	202	0	21-May-14	16	1	14	2	1	1	0	3	2	2	1
10893	202	0	3-Jan-14	17	1	14	2	1	1	0	3	2	3	1
10894	202	0	28-Sep-14	16	1	14	2	1	1	0	3	2	3	2
10895	202	0	10-Mar-14	17	1	14	2	1	1	0	3	2	1	1
10896	202	0	19-Jun-14	18	1	14	2	1	1	0	3	2	3	1
10897	202	0	6-Sep-14	22	1	14	2	1	1	0	3	2	2	1
10898	202	0	8-Dec-14	18	1	14	2	1	1	0	3	2	2	1
10899	202	0	12-Jun-14	17	1	14	2	1	1	0	3	2	2	2
10900	202	0	25-Oct-14	20	1	14	2	1	1	0	3	2	2	2
10901	202	0	26-May-14	18	1	14	2	1	1	0	3	2	3	1
10902	202	0	15-Oct-14	20	1	14	2	2	1	0	3	2	2	1
10903	202	0	28-Nov-14	13	1	14	2	1	1	0	3	2	3	1
10904	202	0	28-Sep-13	17	1	14	2	1	1	0	3	2	2	1
10905	202	0	6-Dec-13	18	1	14	2	3	1	0	3	2	1	1

10906	202	0	24-Nov-13	0	1	14	2	1	1	0	3	2	3	2
10907	202	0	23-Jul-13	22	1	14	2	1	1	0	3	2	1	1
10908	202	0	21-Sep-13	15	1	14	2	1	1	0	3	2	5	1
10909	202	0	17-Dec-13	21	1	14	2	2	1	0	3	2	4	2
10910	202	0	5-Dec-13	15	1	14	2	1	1	0	3	2	2	2
10911	202	0	11-Oct-13	22	1	14	2	3	1	0	3	2	2	1
10912	202	0	27-May-13	18	1	14	2	1	1	0	3	2	2	1
10913	202	0	5-Dec-13	21	1	14	2	1	1	0	3	2	2	1
10914	202	0	19-Apr-13	22	1	14	2	1	1	0	3	2	1	1
10915	202	0	4-Dec-13	7	1	14	2	1	1	0	3	2	5	1
10916	202	0	16-Feb-13	19	1	14	2	1	1	0	3	2	1	2
10917	202	0	20-Sep-13	1	1	14	2	1	1	0	3	2	1	1
10918	202	0	27-Sep-13	17	1	14	2	1	1	0	3	2	2	1
10919	202	0	12-Jun-13	12	1	14	2	1	1	0	3	2	1	1
10920	202	0	3-Dec-13	19	1	14	2	1	1	0	3	2	3	1
10921	202	0	13-Apr-13	17	1	14	2	3	1	0	3	2	2	1
10922	202	0	29-Apr-13	18	1	14	2	1	1	0	3	2	3	2
10923	202	0	29-Apr-13	17	1	14	2	1	1	0	3	2	3	1
10924	202	0	10-Nov-13	15	1	14	2	1	1	0	3	2	3	1
10925	202	0	17-Sep-13	21	1	14	2	3	1	0	3	2	4	1
10926	202	0	3-Nov-13	8	1	14	2	1	1	0	3	2	1	1
10927	202	0	2-Dec-13	16	1	14	2	1	1	0	3	2	3	1
10928	202	0	25-Oct-13	17	1	14	2	2	1	0	3	2	2	2
10929	202	0	20-Nov-13	18	1	14	2	1	1	0	3	2	3	1
10930	202	0	7-Jun-13	19	1	14	2	1	1	0	3	2	2	1
10931	202	0	11-Mar-13	10	1	14	2	1	1	0	3	2	2	1
10932	202	0	16-Sep-13	15	1	14	2	1	1	0	3	2	2	1
10933	202	0	18-Oct-13	10	1	14	2	1	1	0	3	2	2	1
10934	202	0	7-Nov-13	15	1	14	2	1	1	0	3	2	3	1
10935	202	0	12-Feb-13	21	1	14	2	1	1	0	3	2	2	1
10936	202	0	20-Dec-12	12	1	14	2	1	1	0	3	2	1	1
10937	202	0	10-Jul-12	21	1	14	2	2	1	0	3	2	1	2
10938	202	0	20-Aug-12	21	1	14	2	1	1	0	3	2	1	1
10939	202	0	14-Sep-12	19	1	14	2	1	1	0	3	2	3	1
10940	202	0	28-Jun-12	19	1	14	2	1	1	0	3	2	2	1
10941	202	0	19-Jul-12	23	1	14	2	1	1	0	3	2	2	1
10942	202	0	12-Oct-12	12	1	14	2	3	1	0	3	2	6	1
10943	202	0	28-Sep-12	15	1	14	2	1	1	0	3	2	3	2
10944	202	0	14-Dec-12	15	1	14	2	1	1	0	3	2	2	1
10945	202	0	28-Sep-12	9	1	14	2	1	1	0	3	2	3	1
10946	202	0	7-Nov-12	18	1	14	2	1	1	0	3	2	3	1
10947	202	0	30-Dec-11	16	1	14	2	1	1	0	3	2	2	1
10948	202	0	22-Mar-11	7	1	14	2	1	1	0	3	2	2	1
10949	202	0	6-May-11	18	1	14	2	1	1	0	3	2	4	1
10950	202	0	4-Apr-11	22	1	14	2	1	1	0	3	2	3	1
10951	202	0	5-Aug-11	22	1	14	2	1	1	0	3	2	1	1
10952	202	0	27-Apr-11	19	1	14	2	1	1	0	3	2	3	1
10953	202	0	21-Oct-11	8	1	14	2	1	1	0	3	2	2	1
10954	202	0	31-Dec-10	9	1	14	2	1	1	0	3	2	1	1
10955	202	0	6-Jul-10	21	1	14	2	3	1	0	3	2	2	2
10956	202	0	17-Nov-10	14	1	14	2	1	1	0	3	2	1	1
10957	202	0	5-Apr-10	17	1	14	2	3	1	0	3	2	1	2
10958	202	0	30-Nov-10	17	1	14	2	1	1	0	3	2	2	2
10959	202	0	30-Apr-10	17	1	14	2	1	1	0	3	2	1	1
10960	202	0	26-May-10	18	1	14	2	1	1	0	3	2	2	1
10961	202	0	19-Mar-09	15	1	14	2	1	1	0	3	2	1	1
10962	202	0	10-Jun-09	20	1	14	2	3	1	0	3	2	2	2
10963	202	0	19-Mar-09	16	1	14	2	1	1	0	3	2	7	2
10964	202	0	19-Mar-09	19	1	14	2	3	1	0	3	2	3	2
10965	202	0	17-Apr-09	20	1	14	2	2	1	0	3	2	2	2
10966	202	0	20-Nov-09	13	1	14	2	1	1	0	3	2	1	1
10967	202	0	28-Apr-09	12	1	14	2	1	1	0	3	2	2	2
10968	202	0	25-Apr-09	18	1	14	2	1	1	0	3	2	2	2
10969	202	0	10-Nov-09	15	1	14	2	1	1	0	3	2	2	2
10970	202	0	15-Oct-08	16	1	14	2	1	1	0	3	2	2	1
10971	202	0	13-Nov-08	9	1	14	2	1	1	0	3	2	1	1
10972	202	0	20-Jul-08	19	1	14	2	1	1	0	3	2	3	2
10973	202	0	18-Oct-08	15	1	14	2	1	1	0	3	2	3	2
10974	202	0	20-May-08	21	1	14	2	1	1	0	3	2	1	1
10975	202	0	18-Jun-08	14	1	14	2	1	1	0	3	2	1	1
10976	202	0	5-Mar-08	11	1	14	2	1	1	0	3	2	2	1
10977	202	0	23-Oct-08	18	1	14	2	1	1	0	3	2	2	1
10978	202	0	16-Oct-08	21	1	14	2	1	1	0	3	2	2	1
10979	202	0	10-Sep-08	20	1	14	2	1	1	0	3	2	2	2
10980	202	0	25-Oct-08	16	1	14	2	1	1	0	3	2	2	2
10981	202	0	5-Jan-08	17	1	14	2	1	1	0	3	2	2	2
10982	202	0	25-Oct-08	14	1	14	2	1	1	0	3	2	1	2
10983	202	0	20-Jun-08	20	1	14	2	2	1	0	3	2	2	2
10984	202	0	19-Sep-08	12	1	14	2	1	1	0	3	2	2	1
10985	202	0	4-May-08	17	1	14	2	1	1	0	3	2	3	1
10986	202	0	24-Nov-08	19	1	14	2	2	1	0	3	2	2	2
10987	202	0	15-Apr-08	10	1	14	2	1	1	0	3	2	1	1
10988	202	0	25-Nov-08	21	1	14	2	1	1	0	3	2	2	1
10989	202	0	14-Nov-08	11	1	14	2	1	1	0	3	2	2	1
10990	202	0	15-May-08	15	1	14	2	1	1	0	3	2	3	2
10991	202	0	2-Aug-08	20	1	14	2	1	1	0	3	2	3	2
10992	202	0	30-Jun-07	17	1	14	2	2	1	0	3	2	2	1
10993	202	0	3-Jul-07	17	1	14	2	1	1	0	3	2	1	1
10994	202	0	25-Sep-07	17	1	14	2	1	1	0	3	2	3	1
10995	202	0	31-Mar-07	21	1	14	2	1	1	0	3	2	3	2
10996	202	0	31-Aug-07	20	1	14	2	1	1	0	3	2	4	1
10997	202	0	15-Mar-07	11	1	14	2	1	1	0	3	2	1	1
10998	202	0	23-Oct-07	19	1	14	2	1	1	0	3	2	2	1
10999	202	0	20-Nov-07	16	1	14	2	1	1	0	3	2	4	2
11000	202	0	17-Oct-07	16	1	14	2	1	1	0	3	2	1	1
11001	202	0	9-Nov-07	17	1	14	2	1	1	0	3	2	2	1
11002	202	0	10-Oct-07	14	1	14	2	1	1	0	3	2	2	2
11003	202	0	19-Feb-07	11	1	14	2	1	1	0	3	2	2	2
11004	202	0	11-Apr-06	10	1	14	2	1	1	0	3	2	1	1
11005	202	0	30-Nov-06	21	1	14	2	1	1	0	3	2	1	1
11006	202	0	23-Aug-06	6	1	14	2	1	1	0	3	2	2	2
11007	202	0	31-Oct-06	17	1	14	2	1	1	0	3	2	2	2
11008	202	0	12-Nov-06	20	1	14	2	1	1	0	3	2	3	2
11009	202	0	5-Mar-17	17	1	14	2	1	1	0	3	2	3	1
11010	202	0	9-Nov-17	18	1	14	2	1	1	0	3	2	3	1
11011	202	0	18-Nov-17	22	1	14	2	1	1	0	3	2	2	1
11012	202	0	7-Dec-17	17	1	14	2	1	1	0	3	2	2	1
11013	202	0	1-May-17	15	1	14	2	1	1	0	3	2	1	1
11014	202	0	4-Jan-17	14	1	14	2	1	1	0	3	2	1	1
11015	202	0	7-Sep-17	22	1	14	2	1	1	0	3	2	1	1
11016	202	0	7-Dec-17	16	1	14	2	1	1	0	3	2	4	2
11017	202	0	25-Nov-17	20	1	14	2	1	1	0	3	2	2	2
11018	202	0	29-Sep-17	10	1	14	2	2	1	0	3	2	4	1
11019	202	0	12-Jan-17	9	1	14	2	2	1	0	3	2	3	1
11020	202	0	18-Mar-17	8	1	14	2	2	1	0	3	2	1	1
11021	202	0	22-Mar-17	14	1	14	2	3	1	0	3	2	3	1
11022	202	0	14-Dec-17	17	1	14	2	1	1	0	3	2	1	1
11023	202	0	9-May-13	20	1	14	2	2	1	0	6	2	2	2
11024	202	0	18-Nov-12	12	1	14	2	1	1	0	6	2	2	1
11025	202	0	19-Aug-12	20	1	14	2	1	1	0	6	2	2	2
11026	202	0	29-Sep-11	12	1	14	2	1	1	0	6	2	3	1
11027	202	0	7-Apr-11	9	1	14	2	1	1	0	6	2	3	1
11028	202	0	14-Mar-11	16	1	14	2	1	1	0	6	2	2	1
11029	202	0	26-Apr-10	11	1	14	2	1	1	0	6	2	1	1

11030	202	0	21-Feb-10	14	1	14	2	1	1	0	6	2	2	4
11031	202	0	12-Jun-10	11	1	14	2	1	1	0	6	2	2	1
11032	202	0	1-Jun-10	9	1	14	2	1	1	0	6	2	3	1
11033	202	0	4-Mar-10	1	1	14	2	1	1	0	6	2	2	2
11034	202	0	25-Dec-09	23	1	14	2	1	1	0	6	2	1	1
11035	202	0	23-Oct-09	18	1	14	2	1	1	0	6	2	4	1
11036	202	0	21-Oct-08	15	1	14	2	1	1	0	6	2	1	1
11037	202	0	28-Apr-08	17	1	14	2	1	1	0	6	2	2	1
11038	202	0	30-Oct-08	22	1	14	2	1	1	0	6	2	3	1
11039	202	0	2-May-08	20	1	14	2	2	1	0	6	2	2	1
11040	202	0	18-Sep-08	18	1	14	2	1	1	0	6	2	2	2
11041	202	0	22-May-07	21	1	14	2	1	1	0	6	2	4	1
11042	202	0	3-Dec-07	14	1	14	2	1	1	0	6	2	2	1
11043	202	0	20-Jul-06	16	1	14	2	2	1	0	6	2	1	1
11044	202	0	3-Jan-06	10	1	14	2	1	1	0	6	2	2	1
11045	202	0	5-Oct-09	12	1	14	2	1	1	0	13	2	3	2
11046	202	0	27-May-08	18	1	14	2	2	1	0	13	2	1	1
11047	202	0	5-Jul-06	12	1	14	2	1	1	0	13	2	6	1
11048	202	0	13-Dec-14	15	2	14	2	1	1	0	1	2	3	1
11049	202	0	8-Jan-13	23	2	14	2	1	1	0	1	2	2	2
11050	202	0	6-Mar-13	9	2	14	2	2	1	0	4	2	1	1
11051	202	0	25-Mar-16	11	2	14	2	1	1	0	4	2	4	1
11052	202	0	19-Nov-14	15	2	14	2	1	1	0	3	2	1	1
11053	202	0	17-Jun-15	18	2	14	2	1	1	0	6	2	2	1
11054	202	0	6-Jul-17	20	2	14	2	2	1	0	6	2	2	1
11055	202	0	20-Aug-17	19	2	14	2	2	1	0	6	2	2	1
11056	202	0	7-Sep-16	12	2	14	2	1	1	0	6	2	2	1
11057	202	0	26-Apr-14	3	2	14	2	1	1	0	6	2	3	1
11058	202	0	28-Dec-12	0	2	14	2	3	1	0	7	2	5	2
11059	202	0	8-Apr-08	14	2	14	2	1	1	0	13	2	1	1
11060	202	0	3-Jul-07	10	2	14	2	1	1	0	13	2	1	1
11061	202	0	1-Jun-07	11	2	14	2	1	1	0	13	2	1	1
11062	202	0	9-Oct-06	11	2	14	2	2	1	0	13	2	1	1
11063	202	0	9-Sep-16	14	2	14	2	1	1	0	4	2	1	1
11064	202	0	20-Oct-16	16	2	14	2	1	1	0	4	2	1	1
11065	202	0	30-Mar-16	17	2	14	2	2	1	0	4	2	1	2
11066	202	0	9-May-13	9	2	14	2	3	1	0	4	2	1	1
11067	202	0	9-Dec-09	7	2	14	2	1	1	0	4	2	2	1
11068	202	0	4-May-08	17	2	14	2	1	1	0	4	2	1	1
11069	202	0	12-Jun-08	17	2	14	2	1	1	0	4	2	1	1
11070	202	0	3-Nov-06	17	2	14	2	1	1	0	4	2	1	1
11071	202	0	6-Feb-16	10	2	14	2	3	1	0	11	2	3	1
11072	202	0	12-Aug-14	19	2	14	2	1	1	0	11	2	3	1
11073	202	0	21-Jul-13	9	2	14	2	1	1	0	11	2	3	1
11074	202	0	1-Jul-11	17	2	14	2	1	1	0	11	2	7	1
11075	202	0	1-Jul-11	18	2	14	2	2	1	0	11	2	4	2
11076	202	0	24-Jun-17	12	2	14	2	1	1	0	11	2	3	1
11077	202	0	16-Nov-17	9	2	14	2	1	1	0	11	2	2	1
11078	202	0	19-Sep-16	20	2	14	2	2	1	0	3	2	3	1
11079	202	0	8-Sep-16	7	2	14	2	1	1	0	3	2	2	2
11080	202	0	7-Apr-16	17	2	14	2	3	1	0	3	2	3	1
11081	202	0	13-Dec-16	16	2	14	2	3	1	0	3	2	3	1
11082	202	0	18-Nov-16	16	2	14	2	1	1	0	3	2	3	1
11083	202	0	14-Dec-16	15	2	14	2	1	1	0	3	2	2	1
11084	202	0	2-Dec-15	23	2	14	2	1	1	0	3	2	2	1
11085	202	0	9-Jul-15	10	2	14	2	1	1	0	3	2	1	1
11086	202	0	18-Jun-15	18	2	14	2	1	1	0	3	2	5	1
11087	202	0	12-Nov-14	0	2	14	2	1	1	0	3	2	1	1
11088	202	0	28-Apr-14	16	2	14	2	1	1	0	3	2	1	1
11089	202	0	29-Aug-14	21	2	14	2	2	1	0	3	2	1	1
11090	202	0	15-Oct-14	23	2	14	2	1	1	0	3	2	1	1
11091	202	0	15-May-14	1	2	14	2	1	1	0	3	2	2	2
11092	202	0	16-Apr-14	12	2	14	2	1	1	0	3	2	3	1
11093	202	0	5-Nov-13	14	2	14	2	1	1	0	3	2	3	1
11094	202	0	16-Apr-13	18	2	14	2	1	1	0	3	2	3	1
11095	202	0	2-Dec-13	17	2	14	2	1	1	0	3	2	4	1
11096	202	0	11-Oct-12	7	2	14	2	1	1	0	3	2	5	1
11097	202	0	24-Jan-12	0	2	14	2	2	1	0	3	2	3	2
11098	202	0	7-Mar-11	11	2	14	2	1	1	0	3	2	6	2
11099	202	0	14-Dec-10	18	2	14	2	1	1	0	3	2	1	1
11100	202	0	9-Mar-10	9	2	14	2	1	1	0	3	2	3	1
11101	202	0	21-May-09	15	2	14	2	1	1	0	3	2	3	1
11102	202	0	25-Aug-09	11	2	14	2	1	1	0	3	2	1	1
11103	202	0	6-Oct-09	21	2	14	2	1	1	0	3	2	4	1
11104	202	0	6-Oct-09	20	2	14	2	1	1	0	3	2	4	1
11105	202	0	30-May-08	13	2	14	2	1	1	0	3	2	3	1
11106	202	0	23-May-08	7	2	14	2	1	1	0	3	2	3	1
11107	202	0	29-Apr-08	18	2	14	2	1	1	0	3	2	1	1
11108	202	0	9-Dec-07	1	2	14	2	3	1	0	3	2	1	1
11109	202	0	25-May-06	21	2	14	2	1	1	0	3	2	1	1
11110	202	0	2-Mar-17	17	2	14	2	1	1	0	3	2	5	1
11111	202	0	7-Jan-10	11	2	14	2	1	1	0	6	2	2	1
11112	202	0	28-May-10	16	2	14	2	1	1	0	6	2	2	1
11113	202	0	18-Apr-10	11	2	14	2	1	1	0	6	2	3	2
11114	202	0	26-Dec-09	17	2	14	2	1	1	0	6	2	1	2
11115	202	0	3-Sep-08	10	2	14	2	1	1	0	6	2	2	1
11116	202	0	22-Nov-07	20	2	14	2	3	1	0	6	2	1	1
11117	202	0	17-Jun-06	22	2	14	2	1	1	0	6	2	3	1
11118	202	0	1-Nov-08	12	2	14	2	1	1	0	13	2	3	2
11119	201	1	21-Sep-16	20	1	14	2	1	1	0	1	2	2	1
11120	201	1	9-May-14	14	1	14	2	1	1	0	1	2	1	2
11121	101	1	1-Dec-13	2	1	14	2	1	1	0	1	2	1	1
11122	101	1	3-Jul-13	19	1	14	2	1	1	0	1	2	5	1
11123	201	1	4-Jan-17	20	1	14	2	1	1	0	1	2	5	1
11124	201	1	14-Dec-17	11	1	14	2	1	1	0	4	2	1	1
11125	201	1	4-May-16	13	1	14	2	2	1	0	6	2	2	1
11126	201	1	28-Apr-15	8	1	14	2	1	1	0	6	2	2	1
11127	101	1	20-Jan-17	18	1	14	2	1	1	0	3	2	1	1
11128	201	1	23-Apr-13	10	1	14	2	1	1	0	6	2	1	1
11129	201	1	22-Jul-17	21	1	14	2	2	1	0	6	2	3	1
11130	201	1	16-Feb-17	18	1	14	2	2	1	0	6	2	3	1
11131	201	1	15-Jan-12	21	1	14	2	3	1	0	6	2	3	1
11132	201	1	19-Sep-16	11	1	14	2	1	1	0	6	2	3	1
11133	101	1	6-Mar-15	15	1	14	2	1	1	0	7	2	4	1
11134	101	1	8-Jun-16	9	1	14	2	1	1	0	6	2	2	1
11135	101	1	7-Nov-17	15	1	14	2	1	1	0	6	2	2	1
11136	201	1	11-Dec-16	18	1	14	2	2	1	0	4	2	1	1
11137	201	1	27-Jul-16	9	1	14	2	1	1	0	3	2	3	1
11138	101	1	13-Mar-15	13	1	14	2	1	1	0	6	2	2	1
11139	201	1	25-Oct-17	14	1	14	2	3	1	0	6	2	2	1
11140	101	1	2-Apr-15	10	1	14	2	1	1	0	6	2	2	1
11141	201	1	8-May-12	15	1	14	2	3	1	0	6	2	2	1
11142	201	1	10-Sep-12	18	1	14	2	2	1	0	7	2	2	1
11143	101	1	18-Feb-15	20	1	14	2	1	1	0	7	2	2	2
11144	201	1	30-Oct-13	18	1	14	2	1	1	0	7	2	3	1
11145	101	1	24-Apr-09	7	1	14	2	1	1	0	7	2	1	1
11146	101	1	6-Mar-06	12	1	14	2	1	1	0	7	2	3	1
11147	101	1	4-Dec-14	15	1	14	2	1	1	0	13	2	4	1
11148	101	1	27-Apr-13	17	1	14	2	1	1	0	13	2	4	2
11149	201	1	24-Aug-12	18	1	14	2	1	1	0	13	2	3	2
11150	101	1	8-May-11	16	1	14	2	2	1	0	13	2	3	4
11151	101	1	22-Nov-11	15	1	14	2	1	1	0	13	2	3	4
11152	101	1	30-Mar-10	18	1	14	2	3	1	0	13	2	3	1
11153	101	1	8-Jan-10	15	1	14	2	1	1	0	13	2	4	1

11154	201	1	23-Oct-09	12	1	14	2	1	1	0	13	2	1	1
11155	101	1	1-Apr-09	14	1	14	2	1	1	0	13	2	2	1
11156	101	1	31-Mar-07	8	1	14	2	1	1	0	13	2	2	1
11157	101	1	28-Mar-06	16	1	14	2	1	1	0	13	2	2	1
11158	101	1	7-Dec-17	1	1	14	2	1	1	0	13	2	4	1
11159	101	1	3-Mar-17	18	1	14	2	1	1	0	13	2	2	1
11160	101	1	2-Oct-17	10	1	14	2	1	1	0	13	2	2	1
11161	101	1	4-May-08	18	1	14	2	1	1	0	1	2	1	1
11162	101	1	27-Mar-08	8	1	14	2	1	1	0	1	2	3	1
11163	101	1	21-Aug-06	9	1	14	2	2	1	0	1	2	3	2
11164	101	1	6-Dec-15	21	1	14	2	1	1	0	5	2	5	4
11165	101	1	29-May-09	17	1	14	2	1	1	0	1	2	4	1
11166	101	1	23-Mar-09	11	1	14	2	3	1	0	1	2	2	1
11167	101	1	31-Mar-16	17	1	14	2	1	1	0	4	2	2	1
11168	101	1	16-Jan-16	12	1	14	2	1	1	0	4	2	4	1
11169	201	1	16-May-16	12	1	14	2	2	1	0	4	2	3	1
11170	201	1	25-Sep-15	9	1	14	2	1	1	0	4	2	2	1
11171	201	1	13-Jan-15	18	1	14	2	2	1	0	4	2	2	1
11172	201	1	15-Sep-15	10	1	14	2	3	1	0	4	2	1	1
11173	201	1	5-Jan-15	15	1	14	2	1	1	0	4	2	2	1
11174	101	1	29-May-14	16	1	14	2	1	1	0	4	2	1	1
11175	201	1	6-May-14	15	1	14	2	2	1	0	4	2	2	1
11176	201	1	20-Jul-14	18	1	14	2	3	1	0	4	2	2	1
11177	101	1	16-Nov-14	11	1	14	2	1	1	0	4	2	3	1
11178	101	1	26-Jul-14	18	1	14	2	1	1	0	4	2	2	1
11179	201	1	1-Jan-14	15	1	14	2	1	1	0	4	2	3	2
11180	101	1	5-Jan-13	16	1	14	2	1	1	0	4	2	2	1
11181	101	1	29-Sep-13	15	1	14	2	1	1	0	4	2	1	1
11182	101	1	4-Oct-13	16	1	14	2	1	1	0	4	2	1	1
11183	201	1	19-Apr-13	15	1	14	2	3	1	0	4	2	1	1
11184	101	1	24-Oct-13	17	1	14	2	1	1	0	4	2	1	1
11185	101	1	10-Jan-13	18	1	14	2	1	1	0	4	2	1	1
11186	101	1	12-Mar-13	10	1	14	2	1	1	0	4	2	2	1
11187	101	1	9-Aug-13	12	1	14	2	1	1	0	4	2	2	1
11188	101	1	15-Feb-12	17	1	14	2	1	1	0	4	2	1	1
11189	101	1	12-Nov-12	13	1	14	2	1	1	0	4	2	1	4
11190	101	1	29-Oct-12	17	1	14	2	1	1	0	4	2	7	4
11191	101	1	18-Dec-12	10	1	14	2	1	1	0	4	2	3	1
11192	101	1	26-Dec-12	16	1	14	2	1	1	0	4	2	3	4
11193	101	1	4-Jul-12	16	1	14	2	1	1	0	4	2	5	1
11194	101	1	9-Oct-12	15	1	14	2	1	1	0	4	2	1	1
11195	101	1	21-Sep-12	12	1	14	2	1	1	0	4	2	1	1
11196	101	1	2-Jun-12	17	1	14	2	1	1	0	4	2	1	1
11197	201	1	19-Dec-12	10	1	14	2	3	1	0	4	2	4	1
11198	201	1	17-Dec-12	10	1	14	2	2	1	0	4	2	4	1
11199	101	1	26-Jun-12	16	1	14	2	1	1	0	4	2	3	4
11200	201	1	2-Mar-11	14	1	14	2	1	1	0	4	2	1	1
11201	101	1	7-Nov-11	11	1	14	2	1	1	0	4	2	3	1
11202	101	1	1-Nov-11	14	1	14	2	1	1	0	4	2	4	1
11203	101	1	15-Dec-11	10	1	14	2	1	1	0	4	2	2	1
11204	101	1	20-Nov-11	9	1	14	2	1	1	0	4	2	3	1
11205	101	1	10-May-11	17	1	14	2	1	1	0	4	2	2	1
11206	101	1	2-Jun-11	17	1	14	2	1	1	0	4	2	2	1
11207	101	1	10-Nov-11	17	1	14	2	1	1	0	4	2	3	1
11208	101	1	25-Apr-11	11	1	14	2	1	1	0	4	2	2	1
11209	201	1	4-Nov-10	18	1	14	2	3	1	0	4	2	4	1
11210	101	1	4-Nov-10	15	1	14	2	1	1	0	4	2	4	1
11211	101	1	4-Mar-10	16	1	14	2	1	1	0	4	2	3	1
11212	101	1	18-Aug-10	18	1	14	2	1	1	0	4	2	3	1
11213	201	1	3-Mar-09	11	1	14	2	3	1	0	4	2	1	1
11214	101	1	26-May-09	16	1	14	2	1	1	0	4	2	4	1
11215	101	1	17-May-09	15	1	14	2	1	1	0	4	2	7	4
11216	101	1	25-Jun-09	8	1	14	2	1	1	0	4	2	1	4
11217	101	1	25-Mar-09	10	1	14	2	1	1	0	4	2	4	1
11218	101	1	11-Sep-09	16	1	14	2	1	1	0	4	2	2	1
11219	101	1	10-Mar-09	11	1	14	2	3	1	0	4	2	2	1
11220	101	1	10-Dec-09	11	1	14	2	1	1	0	4	2	2	1
11221	101	1	15-Apr-09	10	1	14	2	1	1	0	4	2	2	1
11222	101	1	4-Dec-09	16	1	14	2	1	1	0	4	2	2	4
11223	101	1	17-Feb-09	17	1	14	2	1	1	0	4	2	2	1
11224	101	1	25-Sep-09	17	1	14	2	1	1	0	4	2	2	1
11225	101	1	25-Feb-09	11	1	14	2	1	1	0	4	2	2	1
11226	101	1	9-Jun-09	10	1	14	2	1	1	0	4	2	1	1
11227	201	1	2-Mar-09	17	1	14	2	3	1	0	4	2	1	1
11228	101	1	20-Sep-08	15	1	14	2	1	1	0	4	2	3	1
11229	101	1	6-Jun-08	19	1	14	2	1	1	0	4	2	2	1
11230	201	1	3-Oct-08	14	1	14	2	3	1	0	4	2	1	1
11231	101	1	26-Dec-08	10	1	14	2	1	1	0	4	2	3	1
11232	201	1	22-Jun-08	1	1	14	2	2	1	0	4	2	2	1
11233	101	1	17-Apr-08	20	1	14	2	1	1	0	4	2	2	1
11234	201	1	17-Oct-08	11	1	14	2	2	1	0	4	2	2	1
11235	101	1	28-Nov-07	20	1	14	2	1	1	0	4	2	1	2
11236	101	1	14-Sep-07	15	1	14	2	1	1	0	4	2	2	1
11237	201	1	8-Nov-07	16	1	14	2	1	1	0	4	2	2	1
11238	101	1	17-Dec-07	9	1	14	2	1	1	0	4	2	2	1
11239	101	1	22-Aug-07	22	1	14	2	1	1	0	4	2	4	1
11240	101	1	18-Jun-07	15	1	14	2	1	1	0	4	2	1	1
11241	201	1	1-Oct-06	17	1	14	2	2	1	0	4	2	3	1
11242	101	1	31-Oct-06	13	1	14	2	4	1	0	4	2	2	1
11243	101	1	29-Oct-06	17	1	14	2	1	1	0	4	2	1	1
11244	101	1	7-Apr-06	11	1	14	2	1	1	0	4	2	2	1
11245	101	1	19-Oct-06	16	1	14	2	1	1	0	4	2	4	1
11246	101	1	26-May-06	15	1	14	2	1	1	0	4	2	1	1
11247	101	1	5-Jan-06	12	1	14	2	1	1	0	4	2	1	1
11248	101	1	14-Sep-06	16	1	14	2	1	1	0	4	2	2	1
11249	101	1	28-Sep-06	14	1	14	2	1	1	0	4	2	7	1
11250	101	1	1-Aug-17	19	1	14	2	1	1	0	4	2	4	1
11251	101	1	2-Mar-17	18	1	14	2	1	1	0	4	2	3	1
11252	201	1	2-Nov-17	12	1	14	2	1	1	0	4	2	2	1
11253	201	1	6-Jul-17	9	1	14	2	2	1	0	4	2	2	1
11254	201	1	13-Apr-17	21	1	14	2	3	1	0	4	2	2	1
11255	101	1	21-Sep-17	19	1	14	2	1	1	0	4	2	1	1
11256	101	1	22-Aug-16	19	1	14	2	1	1	0	11	2	2	1
11257	101	1	15-Jul-16	8	1	14	2	1	1	0	11	2	3	1
11258	101	1	26-Dec-15	18	1	14	2	1	1	0	11	2	2	1
11259	201	1	14-Aug-15	17	1	14	2	2	1	0	11	2	3	1
11260	201	1	14-Oct-15	16	1	14	2	1	1	0	11	2	3	1
11261	201	1	19-Aug-15	11	1	14	2	1	1	0	11	2	2	1
11262	101	1	9-Jan-15	18	1	14	2	1	1	0	11	2	2	1
11263	101	1	4-Oct-15	16	1	14	2	1	1	0	11	2	2	1
11264	201	1	4-Jun-14	16	1	14	2	1	1	0	11	2	2	1
11265	101	1	26-Oct-14	12	1	14	2	1	1	0	11	2	2	1
11266	101	1	6-Nov-14	14	1	14	2	1	1	0	11	2	2	1
11267	101	1	28-Jun-14	16	1	14	2	3	1	0	11	2	2	1
11268	201	1	14-Aug-13	10	1	14	2	2	1	0	11	2	2	1
11269	201	1	4-Aug-13	11	1	14	2	1	1	0	11	2	3	1
11270	201	1	16-Feb-13	15	1	14	2	2	1	0	11	2	3	1
11271	101	1	22-Aug-13	22	1	14	2	1	1	0	11	2	3	2
11272	101	1	3-Jun-13	14	1	14	2	3	1	0	11	2	2	1
11273	201	1	15-Aug-13	14	1	14	2	1	1	0	11	2	2	1
11274	101	1	18-Sep-12	13	1	14	2	1	1	0	11	2	2	1
11275	101	1	25-Oct-12	9	1	14	2	1	1	0	11	2	2	1
11276	201	1	10-Jun-12	15	1	14	2	1	1	0	11	2	3	1
11277	101	1	30-Apr-12	22	1	14	2	1	1	0	11	2	2	1

11278	101	1	14-Jul-12	22	1	14	2	1	1	0	11	2	3	1
11279	101	1	14-Dec-12	15	1	14	2	2	1	0	11	2	4	1
11280	101	1	23-Sep-12	15	1	14	2	1	1	0	11	2	2	1
11281	101	1	29-Sep-12	22	1	14	2	1	1	0	11	2	2	1
11282	101	1	11-Oct-11	14	1	14	2	1	1	0	11	2	2	1
11283	101	1	25-Jun-11	23	1	14	2	1	1	0	11	2	3	1
11284	101	1	29-Sep-10	17	1	14	2	1	1	0	11	2	5	1
11285	201	1	19-Sep-10	15	1	14	2	1	1	0	11	2	3	1
11286	201	1	23-Jul-10	15	1	14	2	2	1	0	11	2	2	1
11287	201	1	28-Jan-10	12	1	14	2	1	1	0	11	2	2	1
11288	201	1	11-May-10	17	1	14	2	1	1	0	11	2	3	1
11289	101	1	8-Jan-10	20	1	14	2	2	1	0	11	2	2	1
11290	101	1	15-Aug-10	19	1	14	2	1	1	0	11	2	2	1
11291	101	1	15-Aug-10	17	1	14	2	1	1	0	11	2	3	1
11292	101	1	21-Feb-10	15	1	14	2	3	1	0	11	2	2	1
11293	101	1	6-Sep-09	14	1	14	2	1	1	0	11	2	3	1
11294	101	1	1-Sep-09	16	1	14	2	1	1	0	11	2	3	1
11295	101	1	10-Apr-09	10	1	14	2	1	1	0	11	2	2	1
11296	101	1	17-Oct-08	10	1	14	2	1	1	0	11	2	5	1
11297	201	1	29-Apr-08	18	1	14	2	1	1	0	11	2	2	1
11298	201	1	22-Apr-08	19	1	14	2	1	1	0	11	2	2	1
11299	101	1	26-Sep-06	12	1	14	2	3	1	0	11	2	1	1
11300	201	1	15-Sep-06	21	1	14	2	3	1	0	11	2	3	1
11301	201	1	28-Aug-06	14	1	14	2	1	1	0	11	2	3	1
11302	101	1	31-Mar-17	19	1	14	2	1	1	0	11	2	2	1
11303	201	1	28-Dec-17	10	1	14	2	3	1	0	11	2	2	1
11304	101	1	20-Apr-16	20	1	14	2	1	1	0	3	2	1	1
11305	101	1	19-Oct-16	17	1	14	2	1	1	0	3	2	2	1
11306	101	1	17-Dec-16	8	1	14	2	1	1	0	3	2	3	4
11307	201	1	27-Dec-16	14	1	14	2	1	1	0	3	2	3	1
11308	201	1	6-Apr-16	14	1	14	2	1	1	0	3	2	1	1
11309	201	1	25-Apr-16	18	1	14	2	2	1	0	3	2	2	1
11310	101	1	13-Jul-16	19	1	14	2	1	1	0	3	2	2	1
11311	101	1	17-Mar-16	11	1	14	2	1	1	0	3	2	2	2
11312	101	1	7-Jun-16	8	1	14	2	1	1	0	3	2	5	1
11313	101	1	31-May-16	20	1	14	2	1	1	0	3	2	3	1
11314	201	1	5-May-16	22	1	14	2	1	1	0	3	2	2	1
11315	101	1	4-Aug-16	12	1	14	2	1	1	0	3	2	3	1
11316	101	1	12-Mar-16	14	1	14	2	1	1	0	3	2	1	1
11317	101	1	10-Mar-16	17	1	14	2	1	1	0	3	2	1	1
11318	201	1	13-Jan-16	9	1	14	2	2	1	0	3	2	1	2
11319	101	1	7-Mar-16	12	1	14	2	1	1	0	3	2	2	1
11320	101	1	28-Jan-16	12	1	14	2	1	1	0	3	2	3	1
11321	101	1	19-Apr-16	21	1	14	2	1	1	0	3	2	3	1
11322	101	1	13-Jun-16	17	1	14	2	1	1	0	3	2	2	1
11323	101	1	4-May-16	11	1	14	2	1	1	0	3	2	3	1
11324	101	1	3-Jun-16	18	1	14	2	1	1	0	3	2	2	1
11325	101	1	25-Jul-16	14	1	14	2	1	1	0	3	2	2	1
11326	101	1	24-Mar-16	10	1	14	2	1	1	0	3	2	1	1
11327	101	1	11-May-16	15	1	14	2	1	1	0	3	2	3	1
11328	101	1	6-Oct-16	17	1	14	2	1	1	0	3	2	2	1
11329	101	1	15-Dec-16	21	1	14	2	1	1	0	3	2	3	1
11330	201	1	28-Nov-16	16	1	14	2	2	1	0	3	2	2	1
11331	101	1	4-Aug-16	17	1	14	2	1	1	0	3	2	3	1
11332	101	1	23-Jun-16	16	1	14	2	1	1	0	3	2	3	4
11333	101	1	9-Jun-16	15	1	14	2	1	1	0	3	2	3	1
11334	201	1	9-Jun-16	18	1	14	2	2	1	0	3	2	3	1
11335	101	1	28-Apr-16	17	1	14	2	1	1	0	3	2	2	1
11336	201	1	5-May-16	10	1	14	2	1	1	0	3	2	2	1
11337	201	1	3-May-16	14	1	14	2	3	1	0	3	2	1	1
11338	101	1	10-Jan-16	17	1	14	2	1	1	0	3	2	1	1
11339	201	1	21-Jan-16	17	1	14	2	1	1	0	3	2	3	1
11340	201	1	25-Jun-16	9	1	14	2	1	1	0	3	2	1	1
11341	201	1	8-Dec-16	9	1	14	2	1	1	0	3	2	2	1
11342	201	1	14-Mar-16	10	1	14	2	1	1	0	3	2	2	1
11343	201	1	21-Apr-16	17	1	14	2	3	1	0	3	2	1	1
11344	101	1	30-May-16	17	1	14	2	1	1	0	3	2	2	1
11345	101	1	4-Dec-16	18	1	14	2	1	1	0	3	2	2	4
11346	201	1	4-Jan-16	15	1	14	2	1	1	0	3	2	2	1
11347	101	1	17-Apr-15	17	1	14	2	1	1	0	3	2	2	2
11348	101	1	23-Mar-15	16	1	14	2	1	1	0	3	2	3	1
11349	101	1	22-Apr-15	12	1	14	2	1	1	0	3	2	3	4
11350	101	1	13-Apr-15	10	1	14	2	1	1	0	3	2	2	4
11351	201	1	28-Oct-15	17	1	14	2	1	1	0	3	2	3	1
11352	101	1	6-Mar-15	9	1	14	2	1	1	0	3	2	3	1
11353	101	1	11-Dec-15	9	1	14	2	1	1	0	3	2	1	1
11354	101	1	18-Dec-15	17	1	14	2	1	1	0	3	2	2	1
11355	101	1	13-May-15	15	1	14	2	1	1	0	3	2	3	1
11356	101	1	8-Apr-15	14	1	14	2	1	1	0	3	2	1	1
11357	101	1	29-May-15	14	1	14	2	1	1	0	3	2	3	1
11358	201	1	13-Feb-15	20	1	14	2	2	1	0	3	2	5	1
11359	201	1	5-Oct-15	20	1	14	2	1	1	0	3	2	4	1
11360	201	1	1-Jun-15	19	1	14	2	3	1	0	3	2	2	1
11361	101	1	11-Jan-15	10	1	14	2	1	1	0	3	2	1	1
11362	101	1	24-Jan-15	15	1	14	2	1	1	0	3	2	1	2
11363	201	1	27-Sep-15	23	1	14	2	1	1	0	3	2	1	1
11364	101	1	30-May-15	18	1	14	2	1	1	0	3	2	1	1
11365	101	1	1-Apr-15	18	1	14	2	1	1	0	3	2	2	1
11366	101	1	21-Apr-15	18	1	14	2	1	1	0	3	2	2	1
11367	101	1	14-Dec-15	20	1	14	2	1	1	0	3	2	2	1
11368	101	1	2-May-15	12	1	14	2	1	1	0	3	2	3	1
11369	101	1	10-May-15	13	1	14	2	1	1	0	3	2	2	1
11370	101	1	2-Apr-15	16	1	14	2	1	1	0	3	2	3	2
11371	101	1	31-Oct-15	22	1	14	2	1	1	0	3	2	2	1
11372	101	1	17-Dec-15	16	1	14	2	1	1	0	3	2	4	1
11373	101	1	23-Dec-15	19	1	14	2	1	1	0	3	2	3	1
11374	101	1	15-May-15	15	1	14	2	1	1	0	3	2	5	1
11375	201	1	21-Aug-15	19	1	14	2	1	1	0	3	2	2	1
11376	101	1	11-Jan-15	17	1	14	2	1	1	0	3	2	2	1
11377	201	1	24-Sep-15	13	1	14	2	2	1	0	3	2	2	1
11378	101	1	28-Dec-15	11	1	14	2	1	1	0	3	2	3	1
11379	101	1	20-Sep-15	15	1	14	2	1	1	0	3	2	3	1
11380	101	1	6-Nov-15	11	1	14	2	1	1	0	3	2	3	1
11381	101	1	2-Mar-15	11	1	14	2	1	1	0	3	2	1	2
11382	101	1	9-Jun-15	15	1	14	2	1	1	0	3	2	1	1
11383	201	1	2-Sep-15	18	1	14	2	2	1	0	3	2	1	1
11384	101	1	10-Sep-15	21	1	14	2	1	1	0	3	2	3	4
11385	101	1	20-Apr-15	11	1	14	2	1	1	0	3	2	3	1
11386	101	1	8-Apr-15	12	1	14	2	1	1	0	3	2	3	1
11387	101	1	12-Jun-15	20	1	14	2	1	1	0	3	2	3	1
11388	101	1	22-Dec-15	17	1	14	2	1	1	0	3	2	3	1
11389	201	1	19-Oct-15	16	1	14	2	1	1	0	3	2	2	2
11390	201	1	22-Apr-15	17	1	14	2	2	1	0	3	2	2	1
11391	101	1	27-Mar-15	18	1	14	2	1	1	0	3	2	3	1
11392	101	1	7-Mar-15	18	1	14	2	1	1	0	3	2	3	1
11393	201	1	11-Jan-15	11	1	14	2	2	1	0	3	2	3	1
11394	101	1	20-Apr-15	11	1	14	2	1	1	0	3	2	3	1
11395	101	1	27-Apr-15	17	1	14	2	1	1	0	3	2	4	1
11396	101	1	3-Mar-15	19	1	14	2	1	1	0	3	2	2	1
11397	101	1	2-Mar-15	20	1	14	2	1	1	0	3	2	2	1
11398	101	1	25-Dec-15	17	1	14	2	1	1	0	3	2	2	1
11399	201	1	24-Oct-15	11	1	14	2	2	1	0	3	2	3	1
11400	101	1	10-Jun-15	15	1	14	2	1	1	0	3	2	2	1
11401	201	1	11-Sep-15	18	1	14	2	1	1	0	3	2	1	1

11402	201	1	23-Jan-15	17	1	14	2	2	1	0	3	2	1	1
11403	201	1	23-Sep-15	15	1	14	2	2	1	0	3	2	1	1
11404	201	1	14-Sep-15	8	1	14	2	1	1	0	3	2	3	1
11405	201	1	8-May-15	16	1	14	2	1	1	0	3	2	4	1
11406	201	1	7-Mar-15	10	1	14	2	3	1	0	3	2	2	1
11407	101	1	12-May-15	11	1	14	2	1	1	0	3	2	3	1
11408	101	1	6-Feb-15	10	1	14	2	1	1	0	3	2	3	1
11409	201	1	25-Nov-15	17	1	14	2	1	1	0	3	2	4	1
11410	201	1	21-Oct-15	15	1	14	2	1	1	0	3	2	2	1
11411	101	1	20-Jan-15	10	1	14	2	1	1	0	3	2	2	1
11412	201	1	16-Nov-15	17	1	14	2	1	1	0	3	2	4	2
11413	101	1	28-Aug-15	12	1	14	2	1	1	0	3	2	3	1
11414	201	1	16-Sep-15	20	1	14	2	3	1	0	3	2	3	1
11415	201	1	15-Oct-14	15	1	14	2	3	1	0	3	2	1	2
11416	201	1	9-Jan-14	16	1	14	2	2	1	0	3	2	2	1
11417	201	1	14-Oct-14	19	1	14	2	2	1	0	3	2	3	1
11418	101	1	21-Apr-14	21	1	14	2	1	1	0	3	2	5	1
11419	201	1	18-Feb-14	9	1	14	2	3	1	0	3	2	1	1
11420	201	1	14-Apr-14	13	1	14	2	2	1	0	3	2	1	1
11421	101	1	14-Aug-14	15	1	14	2	1	1	0	3	2	1	1
11422	201	1	9-Jun-14	14	1	14	2	2	1	0	3	2	4	1
11423	101	1	4-Nov-14	10	1	14	2	1	1	0	3	2	1	1
11424	201	1	20-Feb-14	18	1	14	2	3	1	0	3	2	3	1
11425	201	1	4-Nov-14	11	1	14	2	1	1	0	3	2	2	1
11426	101	1	16-Apr-14	16	1	14	2	1	1	0	3	2	2	1
11427	101	1	23-Oct-14	17	1	14	2	1	1	0	3	2	3	1
11428	101	1	14-Mar-14	11	1	14	2	1	1	0	3	2	1	1
11429	201	1	6-May-14	18	1	14	2	2	1	0	3	2	1	1
11430	101	1	18-Mar-14	19	1	14	2	1	1	0	3	2	2	1
11431	201	1	12-Dec-14	17	1	14	2	2	1	0	3	2	4	1
11432	201	1	14-Oct-14	14	1	14	2	2	1	0	3	2	3	1
11433	201	1	15-Sep-14	15	1	14	2	2	1	0	3	2	3	1
11434	101	1	24-Sep-14	14	1	14	2	1	1	0	3	2	5	1
11435	101	1	23-Apr-14	19	1	14	2	1	1	0	3	2	1	1
11436	101	1	18-Apr-14	19	1	14	2	1	1	0	3	2	1	1
11437	101	1	21-Apr-14	12	1	14	2	1	1	0	3	2	2	1
11438	201	1	26-Jul-14	12	1	14	2	3	1	0	3	2	1	1
11439	101	1	26-Jun-14	10	1	14	2	1	1	0	3	2	1	1
11440	101	1	30-Nov-14	14	1	14	2	1	1	0	3	2	1	1
11441	101	1	7-Jul-14	20	1	14	2	1	1	0	3	2	1	1
11442	101	1	11-Jun-14	17	1	14	2	1	1	0	3	2	2	1
11443	101	1	10-Nov-14	11	1	14	2	1	1	0	3	2	3	1
11444	101	1	7-Jun-14	21	1	14	2	1	1	0	3	2	2	1
11445	101	1	17-Mar-14	19	1	14	2	1	1	0	3	2	2	1
11446	101	1	10-Apr-14	19	1	14	2	1	1	0	3	2	2	1
11447	101	1	24-Oct-14	17	1	14	2	1	1	0	3	2	2	2
11448	101	1	3-Dec-14	11	1	14	2	1	1	0	3	2	2	1
11449	101	1	3-Jul-14	16	1	14	2	1	1	0	3	2	2	1
11450	101	1	9-Dec-14	19	1	14	2	1	1	0	3	2	2	1
11451	101	1	31-Jan-14	9	1	14	2	1	1	0	3	2	2	1
11452	201	1	3-Mar-14	17	1	14	2	3	1	0	3	2	3	1
11453	101	1	8-Oct-14	16	1	14	2	2	1	0	3	2	3	1
11454	101	1	11-Aug-14	16	1	14	2	1	1	0	3	2	3	1
11455	101	1	10-May-14	19	1	14	2	1	1	0	3	2	1	1
11456	101	1	10-Nov-14	16	1	14	2	1	1	0	3	2	3	1
11457	101	1	24-May-14	19	1	14	2	1	1	0	3	2	3	1
11458	201	1	3-Jul-14	21	1	14	2	3	1	0	3	2	3	1
11459	101	1	24-Jan-14	22	1	14	2	1	1	0	3	2	2	1
11460	101	1	31-Dec-14	7	1	14	2	1	1	0	3	2	2	1
11461	101	1	11-Jul-14	22	1	14	2	1	1	0	3	2	4	1
11462	101	1	3-Sep-14	19	1	14	2	1	1	0	3	2	2	1
11463	201	1	11-Jun-14	16	1	14	2	1	1	0	3	2	5	1
11464	201	1	14-Feb-14	16	1	14	2	1	1	0	3	2	2	1
11465	101	1	3-Jul-14	13	1	14	2	1	1	0	3	2	1	1
11466	201	1	14-Mar-14	11	1	14	2	2	1	0	3	2	3	2
11467	101	1	7-Jul-14	16	1	14	2	1	1	0	3	2	2	1
11468	101	1	28-Oct-14	17	1	14	2	1	1	0	3	2	2	4
11469	101	1	25-Nov-14	18	1	14	2	1	1	0	3	2	3	1
11470	101	1	28-Jan-14	15	1	14	2	1	1	0	3	2	3	4
11471	201	1	2-Oct-14	18	1	14	2	2	1	0	3	2	4	2
11472	101	1	1-Jun-14	15	1	14	2	1	1	0	3	2	3	2
11473	101	1	14-Nov-14	18	1	14	2	1	1	0	3	2	3	1
11474	101	1	27-Feb-14	18	1	14	2	1	1	0	3	2	3	1
11475	201	1	27-Nov-14	18	1	14	2	2	1	0	3	2	3	1
11476	101	1	9-Dec-14	11	1	14	2	1	1	0	3	2	3	1
11477	101	1	21-Apr-14	18	1	14	2	1	1	0	3	2	2	1
11478	201	1	21-Jun-14	17	1	14	2	3	1	0	3	2	3	1
11479	101	1	26-Jul-14	21	1	14	2	1	1	0	3	2	3	1
11480	101	1	17-Apr-14	16	1	14	2	1	1	0	3	2	3	1
11481	101	1	9-Mar-14	19	1	14	2	1	1	0	3	2	2	1
11482	101	1	27-Nov-14	13	1	14	2	1	1	0	3	2	1	2
11483	201	1	21-Feb-14	10	1	14	2	2	1	0	3	2	1	1
11484	201	1	27-Feb-14	16	1	14	2	1	1	0	3	2	3	1
11485	101	1	30-Aug-14	11	1	14	2	1	1	0	3	2	4	1
11486	201	1	28-Apr-14	10	1	14	2	1	1	0	3	2	2	1
11487	201	1	18-Sep-14	10	1	14	2	1	1	0	3	2	3	1
11488	201	1	21-Jun-14	19	1	14	2	3	1	0	3	2	3	1
11489	101	1	26-Mar-14	17	1	14	2	1	1	0	3	2	3	1
11490	201	1	26-Nov-14	15	1	14	2	3	1	0	3	2	3	1
11491	201	1	29-Oct-14	14	1	14	2	2	1	0	3	2	3	1
11492	201	1	8-Oct-14	18	1	14	2	3	1	0	3	2	2	1
11493	101	1	1-Dec-14	17	1	14	2	1	1	0	3	2	3	1
11494	201	1	22-Oct-14	9	1	14	2	1	1	0	3	2	2	1
11495	201	1	5-Sep-14	14	1	14	2	2	1	0	3	2	3	1
11496	201	1	24-May-14	18	1	14	2	3	1	0	3	2	1	1
11497	201	1	30-Apr-14	17	1	14	2	3	1	0	3	2	2	1
11498	201	1	13-Aug-14	10	1	14	2	2	1	0	3	2	3	1
11499	201	1	28-Jun-13	12	1	14	2	2	1	0	3	2	2	1
11500	201	1	2-Dec-13	11	1	14	2	1	1	0	3	2	2	1
11501	201	1	15-Nov-13	20	1	14	2	3	1	0	3	2	3	1
11502	101	1	12-Dec-13	15	1	14	2	1	1	0	3	2	4	1
11503	101	1	19-Dec-13	9	1	14	2	1	1	0	3	2	1	1
11504	201	1	20-Oct-13	15	1	14	2	2	1	0	3	2	1	1
11505	101	1	27-Mar-13	16	1	14	2	1	1	0	3	2	5	1
11506	201	1	3-Jul-13	21	1	14	2	1	1	0	3	2	2	1
11507	101	1	28-Mar-13	12	1	14	2	1	1	0	3	2	2	1
11508	101	1	15-May-13	17	1	14	2	1	1	0	3	2	2	1
11509	101	1	17-Oct-13	15	1	14	2	2	1	0	3	2	1	1
11510	201	1	28-Nov-13	10	1	14	2	2	1	0	3	2	2	1
11511	101	1	15-Oct-13	10	1	14	2	1	1	0	3	2	2	1
11512	101	1	3-Jun-13	12	1	14	2	1	1	0	3	2	2	1
11513	201	1	13-May-13	21	1	14	2	3	1	0	3	2	2	1
11514	101	1	27-Jul-13	20	1	14	2	1	1	0	3	2	4	1
11515	101	1	28-Feb-13	19	1	14	2	1	1	0	3	2	3	1
11516	101	1	9-Feb-13	21	1	14	2	1	1	0	3	2	3	1
11517	101	1	26-Feb-13	15	1	14	2	1	1	0	3	2	5	1
11518	101	1	26-Feb-13	15	1	14	2	1	1	0	3	2	5	1
11519	101	1	16-Sep-13	17	1	14	2	1	1	0	3	2	5	1
11520	101	1	10-Oct-13	17	1	14	2	1	1	0	3	2	1	2
11521	101	1	29-Sep-13	14	1	14	2	1	1	0	3	2	1	1
11522	201	1	9-Oct-13	19	1	14	2	1	1	0	3	2	1	1
11523	101	1	19-Apr-13	19	1	14	2	1	1	0	3	2	1	1
11524	101	1	12-Dec-13	21	1	14	2	1	1	0	3	2	1	1
11525	101	1	16-Nov-13	19	1	14	2	1	1	0	3	2	4	1

11526	101	1	24-Jun-13	21	1	14	2	1	1	0	3	2	3	1
11527	101	1	20-Jan-13	19	1	14	2	1	1	0	3	2	2	4
11528	101	1	28-Apr-13	19	1	14	2	1	1	0	3	2	2	1
11529	201	1	9-Nov-13	16	1	14	2	3	1	0	3	2	2	1
11530	101	1	9-Dec-13	19	1	14	2	1	1	0	3	2	2	1
11531	101	1	12-Feb-13	13	1	14	2	3	1	0	3	2	2	1
11532	101	1	16-May-13	16	1	14	2	1	1	0	3	2	2	1
11533	101	1	8-Jun-13	18	1	14	2	1	1	0	3	2	2	1
11534	101	1	11-Mar-13	18	1	14	2	1	1	0	3	2	2	1
11535	101	1	20-Nov-13	16	1	14	2	1	1	0	3	2	3	1
11536	101	1	24-Apr-13	19	1	14	2	1	1	0	3	2	3	1
11537	101	1	28-Jun-13	18	1	14	2	1	1	0	3	2	3	1
11538	101	1	15-Apr-13	18	1	14	2	1	1	0	3	2	2	1
11539	201	1	26-May-13	17	1	14	2	1	1	0	3	2	3	1
11540	101	1	19-Sep-13	22	1	14	2	1	1	0	3	2	2	1
11541	101	1	7-Apr-13	19	1	14	2	1	1	0	3	2	3	1
11542	101	1	8-May-13	7	1	14	2	1	1	0	3	2	3	1
11543	101	1	24-Nov-13	17	1	14	2	1	1	0	3	2	3	1
11544	101	1	5-Sep-13	17	1	14	2	1	1	0	3	2	2	1
11545	101	1	12-Apr-13	14	1	14	2	1	1	0	3	2	2	2
11546	101	1	6-May-13	18	1	14	2	1	1	0	3	2	2	1
11547	101	1	3-Mar-13	16	1	14	2	1	1	0	3	2	2	1
11548	201	1	27-Oct-13	11	1	14	2	2	1	0	3	2	2	1
11549	101	1	21-May-13	19	1	14	2	1	1	0	3	2	1	1
11550	201	1	17-Sep-13	15	1	14	2	2	1	0	3	2	1	1
11551	201	1	17-Dec-13	16	1	14	2	2	1	0	3	2	1	1
11552	101	1	14-Nov-13	16	1	14	2	1	1	0	3	2	1	2
11553	201	1	12-Apr-13	18	1	14	2	3	1	0	3	2	4	1
11554	101	1	27-Jun-13	11	1	14	2	2	1	0	3	2	4	1
11555	101	1	31-Oct-13	18	1	14	2	1	1	0	3	2	2	1
11556	201	1	14-Jan-13	19	1	14	2	2	1	0	3	2	3	2
11557	101	1	18-Oct-13	16	1	14	2	1	1	0	3	2	3	1
11558	101	1	20-Feb-13	18	1	14	2	1	1	0	3	2	3	1
11559	101	1	27-Apr-13	18	1	14	2	1	1	0	3	2	3	1
11560	101	1	10-Jun-13	19	1	14	2	1	1	0	3	2	2	1
11561	101	1	15-May-13	18	1	14	2	1	1	0	3	2	2	1
11562	101	1	22-Oct-13	14	1	14	2	1	1	0	3	2	4	1
11563	101	1	16-Oct-13	14	1	14	2	1	1	0	3	2	3	4
11564	101	1	23-Apr-13	17	1	14	2	1	1	0	3	2	3	1
11565	101	1	13-Sep-13	15	1	14	2	1	1	0	3	2	3	1
11566	201	1	7-Jan-13	19	1	14	2	2	1	0	3	2	1	1
11567	201	1	23-Apr-13	14	1	14	2	1	1	0	3	2	1	1
11568	201	1	27-Jan-13	14	1	14	2	2	1	0	3	2	1	1
11569	201	1	17-Nov-13	17	1	14	2	3	1	0	3	2	2	1
11570	201	1	16-Oct-13	9	1	14	2	2	1	0	3	2	2	1
11571	201	1	13-Mar-13	15	1	14	2	1	1	0	3	2	4	1
11572	101	1	8-Jun-13	16	1	14	2	1	1	0	3	2	2	4
11573	101	1	4-Jan-13	20	1	14	2	1	1	0	3	2	2	4
11574	201	1	20-Nov-13	18	1	14	2	3	1	0	3	2	2	1
11575	201	1	1-Jul-13	13	1	14	2	2	1	0	3	2	3	1
11576	201	1	25-Nov-13	21	1	14	2	2	1	0	3	2	5	1
11577	201	1	29-Oct-12	18	1	14	2	1	1	0	3	2	2	2
11578	101	1	14-Mar-12	10	1	14	2	1	1	0	3	2	2	1
11579	201	1	26-Dec-12	14	1	14	2	1	1	0	3	2	3	1
11580	101	1	24-Oct-12	16	1	14	2	1	1	0	3	2	3	1
11581	101	1	26-Apr-12	9	1	14	2	1	1	0	3	2	2	4
11582	101	1	14-Dec-12	10	1	14	2	1	1	0	3	2	3	1
11583	101	1	12-Apr-12	11	1	14	2	1	1	0	3	2	3	1
11584	101	1	14-Dec-12	17	1	14	2	2	1	0	3	2	2	4
11585	101	1	13-Dec-12	17	1	14	2	1	1	0	3	2	2	1
11586	101	1	2-Nov-12	14	1	14	2	1	1	0	3	2	2	4
11587	101	1	10-Apr-12	15	1	14	2	2	1	0	3	2	2	1
11588	201	1	14-Feb-12	15	1	14	2	2	1	0	3	2	2	1
11589	101	1	24-Feb-12	17	1	14	2	1	1	0	3	2	2	1
11590	201	1	18-Dec-12	20	1	14	2	2	1	0	3	2	3	1
11591	101	1	5-Nov-12	7	1	14	2	1	1	0	3	2	5	1
11592	101	1	5-Jun-12	10	1	14	2	1	1	0	3	2	5	1
11593	101	1	7-May-12	19	1	14	2	1	1	0	3	2	5	1
11594	101	1	29-Jul-12	10	1	14	2	1	1	0	3	2	1	2
11595	101	1	22-Mar-12	16	1	14	2	1	1	0	3	2	1	1
11596	101	1	20-Apr-12	16	1	14	2	1	1	0	3	2	1	1
11597	101	1	10-Aug-12	7	1	14	2	1	1	0	3	2	1	1
11598	101	1	16-Nov-12	17	1	14	2	1	1	0	3	2	2	1
11599	101	1	23-Nov-12	19	1	14	2	1	1	0	3	2	2	4
11600	101	1	14-Apr-12	10	1	14	2	1	1	0	3	2	3	1
11601	101	1	16-Oct-12	20	1	14	2	1	1	0	3	2	3	1
11602	101	1	25-Mar-12	15	1	14	2	1	1	0	3	2	2	1
11603	101	1	8-Jun-12	19	1	14	2	1	1	0	3	2	2	1
11604	101	1	27-Jun-12	18	1	14	2	1	1	0	3	2	2	4
11605	101	1	19-Nov-12	10	1	14	2	1	1	0	3	2	6	1
11606	101	1	3-Dec-12	17	1	14	2	1	1	0	3	2	3	1
11607	101	1	2-Apr-12	18	1	14	2	1	1	0	3	2	3	1
11608	101	1	16-Jun-12	18	1	14	2	1	1	0	3	2	3	1
11609	101	1	14-Oct-12	17	1	14	2	1	1	0	3	2	3	1
11610	101	1	20-Jun-12	19	1	14	2	1	1	0	3	2	2	1
11611	101	1	20-Jun-12	16	1	14	2	1	1	0	3	2	4	1
11612	101	1	22-Aug-12	21	1	14	2	1	1	0	3	2	3	1
11613	201	1	19-Jul-12	15	1	14	2	3	1	0	3	2	3	1
11614	101	1	1-Nov-12	17	1	14	2	1	1	0	3	2	1	1
11615	101	1	5-Jun-12	13	1	14	2	1	1	0	3	2	2	4
11616	101	1	16-Nov-12	20	1	14	2	1	1	0	3	2	2	1
11617	101	1	13-Dec-12	13	1	14	2	1	1	0	3	2	2	1
11618	101	1	25-Mar-12	8	1	14	2	1	1	0	3	2	3	1
11619	101	1	29-Apr-12	16	1	14	2	1	1	0	3	2	2	1
11620	101	1	26-Nov-12	20	1	14	2	1	1	0	3	2	2	1
11621	101	1	6-Jan-12	22	1	14	2	1	1	0	3	2	2	1
11622	201	1	21-Nov-12	10	1	14	2	2	1	0	3	2	1	1
11623	201	1	21-Nov-12	14	1	14	2	2	1	0	3	2	1	1
11624	201	1	10-May-12	9	1	14	2	2	1	0	3	2	4	1
11625	101	1	2-Jun-12	10	1	14	2	1	1	0	3	2	2	1
11626	101	1	12-Oct-12	17	1	14	2	2	1	0	3	2	3	1
11627	101	1	20-Nov-12	12	1	14	2	1	1	0	3	2	3	1
11628	101	1	11-Nov-12	19	1	14	2	1	1	0	3	2	3	1
11629	101	1	27-Dec-12	17	1	14	2	1	1	0	3	2	3	2
11630	201	1	30-May-12	11	1	14	2	2	1	0	3	2	2	1
11631	101	1	29-Sep-12	16	1	14	2	1	1	0	3	2	2	1
11632	101	1	24-Oct-12	17	1	14	2	1	1	0	3	2	2	1
11633	101	1	17-May-12	14	1	14	2	1	1	0	3	2	2	1
11634	101	1	30-Jul-12	21	1	14	2	1	1	0	3	2	3	1
11635	101	1	27-Aug-12	14	1	14	2	1	1	0	3	2	3	1
11636	101	1	17-Oct-12	16	1	14	2	1	1	0	3	2	3	1
11637	101	1	20-Aug-12	16	1	14	2	1	1	0	3	2	3	1
11638	101	1	29-Jun-12	11	1	14	2	1	1	0	3	2	2	1
11639	101	1	7-Jun-12	13	1	14	2	1	1	0	3	2	3	1
11640	201	1	29-Sep-12	17	1	14	2	1	1	0	3	2	4	1
11641	201	1	17-Jan-12	11	1	14	2	1	1	0	3	2	2	1
11642	101	1	28-Dec-12	17	1	14	2	1	1	0	3	2	5	1
11643	201	1	19-Dec-12	13	1	14	2	2	1	0	3	2	1	1
11644	101	1	24-Oct-12	16	1	14	2	1	1	0	3	2	3	1
11645	101	1	12-Dec-12	16	1	14	2	1	1	0	3	2	2	1
11646	201	1	15-Sep-12	17	1	14	2	2	1	0	3	2	2	1
11647	201	1	27-Mar-12	12	1	14	2	1	1	0	3	2	2	2
11648	101	1	16-Feb-12	17	1	14	2	1	1	0	3	2	2	4
11649	101	1	11-Apr-12	17	1	14	2	1	1	0	3	2	2	4

11650	201	1	22-Oct-12	18	1	14	2	3	1	0	3	2	3	2
11651	201	1	8-Dec-12	15	1	14	2	2	1	0	3	2	2	2
11652	101	1	16-Feb-12	17	1	14	2	1	1	0	3	2	2	1
11653	101	1	24-Sep-12	15	1	14	2	1	1	0	3	2	2	4
11654	101	1	17-Jul-12	20	1	14	2	1	1	0	3	2	2	1
11655	101	1	24-Aug-12	21	1	14	2	3	1	0	3	2	2	1
11656	101	1	25-May-12	16	1	14	2	1	1	0	3	2	3	4
11657	101	1	28-Sep-12	16	1	14	2	1	1	0	3	2	4	1
11658	201	1	16-Oct-12	14	1	14	2	1	1	0	3	2	2	1
11659	201	1	10-Aug-11	9	1	14	2	1	1	0	3	2	4	1
11660	101	1	16-Sep-11	17	1	14	2	1	1	0	3	2	1	4
11661	201	1	11-Jun-11	17	1	14	2	1	1	0	3	2	1	1
11662	101	1	4-May-11	12	1	14	2	1	1	0	3	2	1	1
11663	101	1	19-Oct-11	14	1	14	2	1	1	0	3	2	4	1
11664	101	1	4-May-11	17	1	14	2	1	1	0	3	2	2	4
11665	101	1	28-Nov-11	17	1	14	2	1	1	0	3	2	1	1
11666	101	1	29-Dec-11	15	1	14	2	1	1	0	3	2	3	1
11667	101	1	28-Jun-11	20	1	14	2	1	1	0	3	2	2	1
11668	101	1	20-Sep-11	12	1	14	2	1	1	0	3	2	2	1
11669	101	1	20-Sep-11	14	1	14	2	1	1	0	3	2	2	1
11670	101	1	11-Nov-11	11	1	14	2	1	1	0	3	2	2	1
11671	201	1	17-Aug-11	21	1	14	2	2	1	0	3	2	6	1
11672	101	1	10-Oct-11	11	1	14	2	1	1	0	3	2	4	1
11673	101	1	28-Nov-11	10	1	14	2	1	1	0	3	2	5	1
11674	101	1	5-Sep-11	18	1	14	2	1	1	0	3	2	1	1
11675	101	1	6-Nov-11	10	1	14	2	1	1	0	3	2	6	1
11676	101	1	16-Jun-11	13	1	14	2	1	1	0	3	2	3	1
11677	101	1	6-Oct-11	21	1	14	2	3	1	0	3	2	5	1
11678	101	1	17-Jul-11	15	1	14	2	2	1	0	3	2	3	1
11679	101	1	6-Nov-11	0	1	14	2	1	1	0	3	2	3	1
11680	101	1	7-Oct-11	11	1	14	2	1	1	0	3	2	3	1
11681	101	1	4-Sep-11	20	1	14	2	1	1	0	3	2	2	1
11682	101	1	26-Sep-11	17	1	14	2	1	1	0	3	2	2	4
11683	101	1	15-Dec-11	15	1	14	2	1	1	0	3	2	3	4
11684	101	1	7-May-11	10	1	14	2	1	1	0	3	2	3	1
11685	101	1	10-May-11	17	1	14	2	1	1	0	3	2	1	1
11686	101	1	19-Apr-11	19	1	14	2	1	1	0	3	2	2	1
11687	101	1	16-Jul-11	19	1	14	2	1	1	0	3	2	2	2
11688	101	1	12-Jun-11	17	1	14	2	1	1	0	3	2	3	1
11689	101	1	21-Oct-11	10	1	14	2	1	1	0	3	2	3	1
11690	101	1	24-Feb-11	16	1	14	2	1	1	0	3	2	3	1
11691	101	1	15-Mar-11	17	1	14	2	1	1	0	3	2	3	1
11692	101	1	2-Apr-11	18	1	14	2	1	1	0	3	2	2	1
11693	101	1	27-Oct-11	18	1	14	2	1	1	0	3	2	2	1
11694	101	1	26-Nov-11	11	1	14	2	1	1	0	3	2	4	4
11695	101	1	27-Apr-11	11	1	14	2	1	1	0	3	2	2	2
11696	101	1	17-Sep-11	16	1	14	2	1	1	0	3	2	2	1
11697	101	1	11-May-11	8	1	14	2	1	1	0	3	2	2	1
11698	101	1	10-Apr-11	10	1	14	2	1	1	0	3	2	3	1
11699	201	1	17-Sep-11	20	1	14	2	2	1	0	3	2	4	1
11700	201	1	18-Oct-11	20	1	14	2	1	1	0	3	2	5	1
11701	201	1	4-Nov-11	8	1	14	2	1	1	0	3	2	1	1
11702	201	1	5-Dec-11	17	1	14	2	1	1	0	3	2	1	1
11703	101	1	19-Dec-11	17	1	14	2	1	1	0	3	2	4	1
11704	201	1	19-Oct-11	20	1	14	2	2	1	0	3	2	1	1
11705	101	1	8-Mar-11	10	1	14	2	1	1	0	3	2	2	1
11706	101	1	5-Jul-11	18	1	14	2	1	1	0	3	2	2	1
11707	101	1	15-Apr-11	19	1	14	2	1	1	0	3	2	3	2
11708	101	1	3-Nov-11	19	1	14	2	1	1	0	3	2	2	1
11709	101	1	2-Nov-10	17	1	14	2	1	1	0	3	2	5	1
11710	101	1	11-Jul-10	18	1	14	2	1	1	0	3	2	4	1
11711	101	1	9-Nov-10	12	1	14	2	1	1	0	3	2	1	1
11712	201	1	1-Dec-10	7	1	14	2	2	1	0	3	2	1	1
11713	101	1	28-May-10	11	1	14	2	1	1	0	3	2	2	1
11714	101	1	6-Dec-10	12	1	14	2	1	1	0	3	2	3	1
11715	101	1	16-Apr-10	11	1	14	2	1	1	0	3	2	3	1
11716	201	1	24-Mar-10	18	1	14	2	1	1	0	3	2	7	2
11717	101	1	31-Dec-10	18	1	14	2	1	1	0	3	2	2	1
11718	201	1	13-Oct-10	16	1	14	2	1	1	0	3	2	3	1
11719	101	1	16-Oct-10	19	1	14	2	1	1	0	3	2	4	1
11720	101	1	6-Nov-10	17	1	14	2	3	1	0	3	2	1	2
11721	101	1	14-Jul-10	23	1	14	2	1	1	0	3	2	1	1
11722	101	1	6-Dec-10	16	1	14	2	1	1	0	3	2	3	1
11723	101	1	7-May-10	19	1	14	2	1	1	0	3	2	2	4
11724	101	1	13-Dec-10	10	1	14	2	1	1	0	3	2	3	1
11725	101	1	8-Dec-10	19	1	14	2	1	1	0	3	2	2	1
11726	201	1	6-Feb-10	21	1	14	2	2	1	0	3	2	3	1
11727	101	1	25-Apr-10	13	1	14	2	1	1	0	3	2	3	1
11728	101	1	11-Nov-10	14	1	14	2	1	1	0	3	2	2	1
11729	101	1	6-Oct-10	18	1	14	2	1	1	0	3	2	3	1
11730	101	1	20-Nov-10	17	1	14	2	1	1	0	3	2	3	1
11731	101	1	3-Sep-10	13	1	14	2	2	1	0	3	2	2	1
11732	101	1	7-Jan-10	7	1	14	2	2	1	0	3	2	2	1
11733	201	1	19-May-10	20	1	14	2	1	1	0	3	2	2	1
11734	101	1	3-Aug-10	22	1	14	2	2	1	0	3	2	3	1
11735	101	1	30-Aug-10	21	1	14	2	1	1	0	3	2	2	1
11736	101	1	27-Mar-10	17	1	14	2	1	1	0	3	2	3	1
11737	101	1	15-Jan-10	16	1	14	2	1	1	0	3	2	3	1
11738	101	1	30-Sep-10	11	1	14	2	1	1	0	3	2	3	1
11739	101	1	19-Mar-10	20	1	14	2	1	1	0	3	2	3	2
11740	101	1	3-Mar-10	17	1	14	2	1	1	0	3	2	2	1
11741	101	1	13-Apr-10	17	1	14	2	1	1	0	3	2	2	1
11742	101	1	17-Dec-10	16	1	14	2	1	1	0	3	2	3	1
11743	101	1	17-Oct-10	17	1	14	2	1	1	0	3	2	3	1
11744	101	1	29-Oct-10	17	1	14	2	1	1	0	3	2	2	1
11745	201	1	12-May-10	11	1	14	2	1	1	0	3	2	3	1
11746	101	1	17-Oct-10	19	1	14	2	1	1	0	3	2	3	1
11747	101	1	5-May-10	11	1	14	2	1	1	0	3	2	3	1
11748	101	1	8-May-10	17	1	14	2	1	1	0	3	2	3	1
11749	201	1	8-Jun-10	20	1	14	2	1	1	0	3	2	2	1
11750	101	1	31-Oct-10	8	1	14	2	1	1	0	3	2	2	1
11751	101	1	14-May-10	8	1	14	2	1	1	0	3	2	2	1
11752	101	1	8-Sep-10	17	1	14	2	1	1	0	3	2	3	1
11753	101	1	30-Apr-10	20	1	14	2	1	1	0	3	2	4	1
11754	201	1	30-Nov-10	14	1	14	2	3	1	0	3	2	2	1
11755	201	1	23-Nov-09	9	1	14	2	2	1	0	3	2	1	1
11756	101	1	30-Sep-09	16	1	14	2	1	1	0	3	2	1	1
11757	101	1	21-Apr-09	9	1	14	2	1	1	0	3	2	5	1
11758	201	1	15-Oct-09	22	1	14	2	2	1	0	3	2	3	1
11759	101	1	5-Sep-09	17	1	14	2	2	1	0	3	2	2	2
11760	201	1	18-Nov-09	22	1	14	2	1	1	0	3	2	2	1
11761	101	1	9-Oct-09	19	1	14	2	2	1	0	3	2	3	4
11762	101	1	12-Oct-09	17	1	14	2	1	1	0	3	2	2	1
11763	201	1	21-Apr-09	19	1	14	2	2	1	0	3	2	2	1
11764	101	1	2-Apr-09	17	1	14	2	1	1	0	3	2	3	1
11765	101	1	14-Aug-09	12	1	14	2	1	1	0	3	2	5	1
11766	101	1	31-Jul-09	12	1	14	2	1	1	0	3	2	5	1
11767	101	1	13-Aug-09	8	1	14	2	1	1	0	3	2	4	1
11768	201	1	22-Apr-09	20	1	14	2	3	1	0	3	2	2	1
11769	101	1	24-Dec-09	17	1	14	2	1	1	0	3	2	2	1
11770	101	1	17-Feb-09	10	1	14	2	1	1	0	3	2	2	1
11771	201	1	19-Jul-09	18	1	14	2	1	1	0	3	2	3	1
11772	201	1	22-Feb-09	22	1	14	2	3	1	0	3	2	3	1
11773	101	1	4-May-09	18	1	14	2	1	1	0	3	2	3	1

11774	101	1	23-Jun-09	10	1	14	2	1	1	0	3	2	3	1
11775	201	1	15-Jul-09	17	1	14	2	1	1	0	3	2	3	1
11776	101	1	27-Apr-09	16	1	14	2	1	1	0	3	2	1	1
11777	201	1	23-Mar-09	15	1	14	2	1	1	0	3	2	4	1
11778	101	1	6-Apr-09	8	1	14	2	1	1	0	3	2	3	1
11779	101	1	20-Nov-09	16	1	14	2	1	1	0	3	2	2	1
11780	101	1	30-Apr-09	17	1	14	2	1	1	0	3	2	2	4
11781	101	1	30-Apr-09	17	1	14	2	1	1	0	3	2	2	1
11782	101	1	6-Feb-09	10	1	14	2	1	1	0	3	2	2	1
11783	101	1	19-Mar-09	18	1	14	2	1	1	0	3	2	2	1
11784	101	1	15-Apr-09	11	1	14	2	1	1	0	3	2	3	1
11785	101	1	12-Oct-09	18	1	14	2	1	1	0	3	2	4	1
11786	101	1	8-Mar-09	17	1	14	2	1	1	0	3	2	2	1
11787	201	1	2-Jul-09	20	1	14	2	1	1	0	3	2	5	1
11788	101	1	22-Jul-09	12	1	14	2	1	1	0	3	2	1	1
11789	201	1	28-May-09	16	1	14	2	1	1	0	3	2	2	1
11790	101	1	30-Oct-09	17	1	14	2	2	1	0	3	2	3	1
11791	101	1	24-Feb-09	8	1	14	2	1	1	0	3	2	3	1
11792	101	1	24-Jan-09	10	1	14	2	1	1	0	3	2	2	1
11793	101	1	23-Dec-09	18	1	14	2	1	1	0	3	2	2	1
11794	201	1	26-Feb-09	11	1	14	2	1	1	0	3	2	2	1
11795	101	1	18-Nov-09	17	1	14	2	1	1	0	3	2	2	1
11796	201	1	14-Jan-09	16	1	14	2	1	1	0	3	2	3	1
11797	101	1	26-Oct-09	16	1	14	2	1	1	0	3	2	2	1
11798	101	1	22-Apr-09	10	1	14	2	1	1	0	3	2	2	4
11799	101	1	28-Apr-09	17	1	14	2	1	1	0	3	2	2	2
11800	101	1	25-Feb-09	17	1	14	2	1	1	0	3	2	3	1
11801	201	1	16-Oct-09	12	1	14	2	2	1	0	3	2	3	1
11802	201	1	5-Mar-08	17	1	14	2	1	1	0	3	2	3	2
11803	101	1	23-Oct-08	7	1	14	2	1	1	0	3	2	1	1
11804	101	1	28-Oct-08	18	1	14	2	1	1	0	3	2	1	1
11805	201	1	9-Sep-08	18	1	14	2	1	1	0	3	2	1	1
11806	101	1	22-Apr-08	16	1	14	2	1	1	0	3	2	1	1
11807	101	1	23-Dec-08	10	1	14	2	1	1	0	3	2	3	1
11808	101	1	18-Apr-08	10	1	14	2	1	1	0	3	2	3	1
11809	101	1	29-Aug-08	12	1	14	2	1	1	0	3	2	2	1
11810	101	1	28-Apr-08	12	1	14	2	1	1	0	3	2	2	1
11811	201	1	4-Dec-08	20	1	14	2	1	1	0	3	2	3	1
11812	201	1	21-Nov-08	13	1	14	2	3	1	0	3	2	7	1
11813	201	1	25-Nov-08	15	1	14	2	3	1	0	3	2	2	1
11814	201	1	3-Oct-08	10	1	14	2	3	1	0	3	2	1	1
11815	101	1	28-Sep-08	22	1	14	2	1	1	0	3	2	1	1
11816	101	1	7-Nov-08	19	1	14	2	1	1	0	3	2	5	1
11817	201	1	23-Oct-08	19	1	14	2	1	1	0	3	2	1	1
11818	101	1	5-May-08	8	1	14	2	1	1	0	3	2	5	1
11819	101	1	5-May-08	16	1	14	2	1	1	0	3	2	2	4
11820	201	1	26-Apr-08	21	1	14	2	3	1	0	3	2	2	1
11821	101	1	5-Nov-08	18	1	14	2	1	1	0	3	2	3	1
11822	101	1	27-Apr-08	18	1	14	2	1	1	0	3	2	2	2
11823	101	1	18-Nov-08	19	1	14	2	1	1	0	3	2	2	2
11824	201	1	18-Nov-08	18	1	14	2	1	1	0	3	2	2	1
11825	101	1	10-Apr-08	18	1	14	2	1	1	0	3	2	3	1
11826	101	1	26-Aug-08	16	1	14	2	1	1	0	3	2	3	2
11827	101	1	30-Sep-08	21	1	14	2	1	1	0	3	2	3	1
11828	101	1	14-Nov-08	18	1	14	2	1	1	0	3	2	3	1
11829	101	1	12-May-08	17	1	14	2	1	1	0	3	2	2	2
11830	101	1	9-Dec-08	17	1	14	2	1	1	0	3	2	2	1
11831	101	1	17-Sep-08	18	1	14	2	1	1	0	3	2	3	1
11832	101	1	24-Nov-08	12	1	14	2	1	1	0	3	2	3	1
11833	101	1	8-Apr-08	17	1	14	2	1	1	0	3	2	2	1
11834	101	1	14-Mar-08	10	1	14	2	1	1	0	3	2	3	1
11835	201	1	27-Feb-08	18	1	14	2	1	1	0	3	2	7	1
11836	201	1	17-Nov-08	22	1	14	2	2	1	0	3	2	5	1
11837	201	1	6-Mar-08	18	1	14	2	2	1	0	3	2	3	1
11838	201	1	19-Jun-08	12	1	14	2	2	1	0	3	2	2	1
11839	101	1	28-Nov-08	17	1	14	2	1	1	0	3	2	2	1
11840	201	1	14-Oct-08	17	1	14	2	3	1	0	3	2	2	1
11841	101	1	4-Oct-07	19	1	14	2	1	1	0	3	2	4	1
11842	101	1	28-Jun-07	9	1	14	2	1	1	0	3	2	1	2
11843	101	1	28-Sep-07	16	1	14	2	1	1	0	3	2	1	1
11844	101	1	30-Mar-07	16	1	14	2	1	1	0	3	2	1	1
11845	101	1	2-Apr-07	11	1	14	2	1	1	0	3	2	1	1
11846	101	1	9-May-07	17	1	14	2	1	1	0	3	2	3	1
11847	101	1	12-Nov-07	17	1	14	2	1	1	0	3	2	2	4
11848	101	1	8-May-07	18	1	14	2	1	1	0	3	2	2	2
11849	101	1	19-Apr-07	10	1	14	2	1	1	0	3	2	3	1
11850	101	1	31-Oct-07	22	1	14	2	1	1	0	3	2	2	1
11851	101	1	9-Nov-07	13	1	14	2	1	1	0	3	2	2	2
11852	101	1	12-Dec-07	8	1	14	2	1	1	0	3	2	3	1
11853	101	1	6-Dec-07	18	1	14	2	1	1	0	3	2	2	1
11854	101	1	21-Sep-07	9	1	14	2	1	1	0	3	2	3	1
11855	201	1	4-Jan-07	13	1	14	2	1	1	0	3	2	3	1
11856	201	1	18-Dec-07	21	1	14	2	1	1	0	3	2	1	1
11857	101	1	27-Oct-07	22	1	14	2	1	1	0	3	2	3	1
11858	101	1	9-Oct-07	17	1	14	2	1	1	0	3	2	2	1
11859	101	1	27-Nov-07	18	1	14	2	1	1	0	3	2	2	1
11860	101	1	11-Dec-07	15	1	14	2	1	1	0	3	2	4	1
11861	101	1	27-Jul-07	16	1	14	2	3	1	0	3	2	2	1
11862	101	1	9-Oct-07	19	1	14	2	1	1	0	3	2	2	1
11863	101	1	26-Jan-07	15	1	14	2	1	1	0	3	2	2	1
11864	101	1	9-Nov-07	17	1	14	2	1	1	0	3	2	2	2
11865	101	1	8-Mar-07	16	1	14	2	1	1	0	3	2	3	1
11866	201	1	25-Apr-07	16	1	14	2	1	1	0	3	2	1	1
11867	201	1	27-Jun-07	16	1	14	2	1	1	0	3	2	4	1
11868	101	1	6-Jun-07	11	1	14	2	1	1	0	3	2	2	1
11869	201	1	26-Jun-07	14	1	14	2	1	1	0	3	2	4	1
11870	101	1	14-Mar-07	17	1	14	2	1	1	0	3	2	2	1
11871	201	1	5-Jun-07	17	1	14	2	3	1	0	3	2	2	1
11872	201	1	10-Oct-07	19	1	14	2	1	1	0	3	2	3	2
11873	201	1	30-Mar-07	15	1	14	2	1	1	0	3	2	2	2
11874	201	1	21-Dec-07	16	1	14	2	1	1	0	3	2	2	1
11875	101	1	13-Dec-07	18	1	14	2	1	1	0	3	2	3	1
11876	101	1	22-Aug-07	21	1	14	2	1	1	0	3	2	1	2
11877	101	1	10-Nov-06	21	1	14	2	1	1	0	3	2	3	1
11878	101	1	21-Nov-06	12	1	14	2	1	1	0	3	2	1	1
11879	201	1	9-Sep-06	13	1	14	2	1	1	0	3	2	3	1
11880	101	1	28-Dec-06	9	1	14	2	1	1	0	3	2	3	2
11881	101	1	15-Jun-06	20	1	14	2	1	1	0	3	2	2	1
11882	201	1	25-Aug-06	12	1	14	2	1	1	0	3	2	1	1
11883	101	1	13-Sep-06	12	1	14	2	1	1	0	3	2	1	1
11884	101	1	25-Dec-06	13	1	14	2	1	1	0	3	2	2	1
11885	101	1	19-Nov-06	17	1	14	2	1	1	0	3	2	2	1
11886	101	1	15-Jun-06	17	1	14	2	1	1	0	3	2	1	1
11887	101	1	24-Jul-06	23	1	14	2	2	1	0	3	2	1	1
11888	101	1	27-Aug-06	9	1	14	2	1	1	0	3	2	3	1
11889	101	1	28-Nov-06	17	1	14	2	1	1	0	3	2	2	1
11890	101	1	23-Nov-06	16	1	14	2	1	1	0	3	2	2	1
11891	101	1	26-Oct-06	16	1	14	2	1	1	0	3	2	5	1
11892	201	1	22-Feb-06	13	1	14	2	2	1	0	3	2	1	1
11893	201	1	7-Jul-06	11	1	14	2	1	1	0	3	2	1	1
11894	201	1	22-Jun-06	11	1	14	2	1	1	0	3	2	7	1
11895	101	1	18-Oct-06	17	1	14	2	1	1	0	3	2	3	1
11896	101	1	9-May-06	17	1	14	2	1	1	0	3	2	2	2
11897	101	1	2-Mar-06	11	1	14	2	1	1	0	3	2	2	1

11898	101	1	13-Sep-06	6	1	14	2	1	1	0	3	2	2	1
11899	201	1	18-Sep-06	17	1	14	2	1	1	0	3	2	3	1
11900	101	1	6-Dec-06	18	1	14	2	1	1	0	3	2	3	1
11901	101	1	8-Sep-06	12	1	14	2	1	1	0	3	2	3	1
11902	201	1	6-Jul-06	19	1	14	2	1	1	0	3	2	3	1
11903	201	1	18-Sep-06	15	1	14	2	1	1	0	3	2	3	1
11904	101	1	10-Jan-06	18	1	14	2	1	1	0	3	2	2	1
11905	101	1	20-Apr-06	17	1	14	2	1	1	0	3	2	2	1
11906	101	1	23-Sep-06	13	1	14	2	1	1	0	3	2	2	1
11907	201	1	7-Nov-06	17	1	14	2	1	1	0	3	2	2	1
11908	101	1	8-Dec-06	11	1	14	2	1	1	0	3	2	3	1
11909	101	1	3-Sep-06	16	1	14	2	1	1	0	3	2	2	1
11910	101	1	19-Sep-06	18	1	14	2	1	1	0	3	2	2	1
11911	101	1	20-Apr-17	21	1	14	2	1	1	0	3	2	5	1
11912	101	1	15-Apr-17	18	1	14	2	1	1	0	3	2	2	1
11913	101	1	17-Feb-17	15	1	14	2	1	1	0	3	2	3	2
11914	201	1	17-Nov-17	23	1	14	2	1	1	0	3	2	5	1
11915	101	1	25-Jun-17	17	1	14	2	1	1	0	3	2	4	1
11916	201	1	16-Feb-17	17	1	14	2	1	1	0	3	2	4	1
11917	201	1	10-Jul-17	19	1	14	2	1	1	0	3	2	4	1
11918	101	1	14-Apr-17	17	1	14	2	1	1	0	3	2	3	1
11919	101	1	1-Aug-17	19	1	14	2	1	1	0	3	2	3	1
11920	101	1	9-Jul-17	12	1	14	2	1	1	0	3	2	3	1
11921	101	1	12-Feb-17	18	1	14	2	1	1	0	3	2	3	1
11922	101	1	24-Feb-17	17	1	14	2	1	1	0	3	2	3	1
11923	101	1	25-Aug-17	19	1	14	2	1	1	0	3	2	3	1
11924	101	1	11-Apr-17	6	1	14	2	1	1	0	3	2	3	1
11925	201	1	13-Jan-17	14	1	14	2	1	1	0	3	2	3	1
11926	201	1	1-Jul-17	21	1	14	2	1	1	0	3	2	3	1
11927	101	1	9-Feb-17	11	1	14	2	1	1	0	3	2	2	1
11928	101	1	11-Nov-17	10	1	14	2	1	1	0	3	2	2	1
11929	201	1	7-Sep-17	21	1	14	2	1	1	0	3	2	2	1
11930	101	1	18-Mar-17	15	1	14	2	1	1	0	3	2	1	1
11931	101	1	6-May-17	18	1	14	2	1	1	0	3	2	1	1
11932	101	1	25-Feb-17	12	1	14	2	1	1	0	3	2	1	1
11933	101	1	11-Apr-17	14	1	14	2	1	1	0	3	2	1	1
11934	201	1	25-May-17	19	1	14	2	1	1	0	3	2	1	1
11935	101	1	17-Apr-17	17	1	14	2	1	1	0	3	2	1	2
11936	201	1	19-Sep-17	18	1	14	2	1	1	0	3	2	3	2
11937	101	1	16-Nov-17	16	1	14	2	1	1	0	3	2	2	4
11938	201	1	5-Apr-17	9	1	14	2	2	1	0	3	2	3	1
11939	201	1	25-Oct-17	8	1	14	2	2	1	0	3	2	3	1
11940	201	1	29-Jun-17	20	1	14	2	2	1	0	3	2	3	1
11941	201	1	20-Nov-17	11	1	14	2	2	1	0	3	2	3	1
11942	201	1	10-Sep-17	12	1	14	2	2	1	0	3	2	2	1
11943	201	1	7-Jan-17	19	1	14	2	2	1	0	3	2	2	1
11944	201	1	2-May-17	10	1	14	2	3	1	0	3	2	2	1
11945	201	1	13-Apr-17	21	1	14	2	3	1	0	3	2	1	1
11946	101	1	1-Sep-17	10	1	14	2	1	1	0	3	2	5	1
11947	101	1	24-Nov-17	20	1	14	2	1	1	0	3	2	5	1
11948	101	1	20-Oct-17	21	1	14	2	1	1	0	3	2	4	1
11949	101	1	19-Oct-17	18	1	14	2	1	1	0	3	2	3	1
11950	101	1	16-Dec-17	18	1	14	2	1	1	0	3	2	2	1
11951	101	1	4-Dec-17	19	1	14	2	1	1	0	3	2	2	1
11952	201	1	26-Dec-17	21	1	14	2	2	1	0	3	2	2	1
11953	201	1	5-May-16	11	1	14	2	2	1	0	6	2	2	1
11954	101	1	15-Jun-16	12	1	14	2	1	1	0	6	2	2	1
11955	101	1	15-Apr-16	16	1	14	2	1	1	0	6	2	2	1
11956	101	1	7-Mar-16	14	1	14	2	1	1	0	6	2	2	1
11957	101	1	23-Apr-15	8	1	14	2	1	1	0	6	2	2	1
11958	101	1	5-Jun-15	19	1	14	2	1	1	0	6	2	2	1
11959	201	1	30-Sep-14	15	1	14	2	2	1	0	6	2	2	1
11960	101	1	31-Mar-14	18	1	14	2	1	1	0	6	2	2	1
11961	101	1	26-Oct-14	18	1	14	2	1	1	0	6	2	2	1
11962	101	1	26-Nov-14	15	1	14	2	1	1	0	6	2	2	1
11963	101	1	26-Jul-14	17	1	14	2	3	1	0	6	2	3	1
11964	101	1	15-Aug-14	14	1	14	2	1	1	0	6	2	2	1
11965	101	1	26-Apr-14	23	1	14	2	1	1	0	6	2	2	1
11966	201	1	16-Oct-14	17	1	14	2	2	1	0	6	2	2	2
11967	101	1	1-Mar-13	17	1	14	2	1	1	0	6	2	2	1
11968	101	1	24-Dec-13	15	1	14	2	1	1	0	6	2	2	1
11969	101	1	22-Nov-13	16	1	14	2	2	1	0	6	2	2	2
11970	101	1	12-Jun-13	16	1	14	2	3	1	0	6	2	3	1
11971	101	1	7-Feb-13	23	1	14	2	3	1	0	6	2	3	4
11972	201	1	27-Feb-13	15	1	14	2	3	1	0	6	2	2	1
11973	101	1	1-Apr-12	17	1	14	2	1	1	0	6	2	2	1
11974	101	1	18-May-12	8	1	14	2	1	1	0	6	2	2	1
11975	101	1	6-Jun-12	17	1	14	2	1	1	0	6	2	3	1
11976	101	1	24-Feb-12	8	1	14	2	1	1	0	6	2	3	1
11977	101	1	3-Nov-11	14	1	14	2	1	1	0	6	2	2	1
11978	101	1	24-Oct-11	18	1	14	2	1	1	0	6	2	2	1
11979	201	1	3-Feb-11	14	1	14	2	1	1	0	6	2	2	1
11980	101	1	1-Mar-11	18	1	14	2	1	1	0	6	2	2	1
11981	101	1	23-Mar-11	16	1	14	2	1	1	0	6	2	2	1
11982	101	1	14-Dec-10	9	1	14	2	1	1	0	6	2	2	1
11983	101	1	8-Mar-10	11	1	14	2	1	1	0	6	2	2	1
11984	101	1	5-Jul-10	15	1	14	2	3	1	0	6	2	1	1
11985	101	1	4-Jun-10	14	1	14	2	1	1	0	6	2	2	1
11986	201	1	29-Mar-09	9	1	14	2	3	1	0	6	2	4	1
11987	201	1	21-Mar-09	14	1	14	2	3	1	0	6	2	2	1
11988	201	1	21-Feb-09	13	1	14	2	3	1	0	6	2	3	1
11989	101	1	8-Sep-08	23	1	14	2	1	1	0	6	2	2	1
11990	101	1	18-Dec-08	13	1	14	2	1	1	0	6	2	2	1
11991	201	1	4-Sep-07	13	1	14	2	1	1	0	6	2	2	1
11992	101	1	17-Mar-07	21	1	14	2	1	1	0	6	2	2	1
11993	101	1	21-Dec-06	11	1	14	2	1	1	0	6	2	1	1
11994	101	1	21-Aug-06	22	1	14	2	1	1	0	6	2	3	1
11995	101	1	12-Apr-17	22	1	14	2	1	1	0	6	2	3	1
11996	101	1	22-Jun-17	14	1	14	2	1	1	0	6	2	2	1
11997	101	1	16-Sep-11	15	1	14	2	1	1	0	13	2	5	1
11998	101	1	8-Mar-17	8	1	14	2	1	1	0	13	2	3	2
11999	101	1	11-Apr-13	16	2	14	2	1	1	0	4	2	1	1
12000	201	1	14-Mar-17	18	2	14	2	1	1	0	4	2	2	1
12001	201	1	14-Dec-16	13	2	14	2	1	1	0	4	2	4	1
12002	201	1	15-May-12	13	2	14	2	1	1	0	4	2	1	1
12003	201	1	22-Aug-16	7	2	14	2	3	1	0	7	2	3	1
12004	101	1	29-Apr-16	16	2	14	2	1	1	0	13	2	3	1
12005	101	1	28-Nov-17	15	2	14	2	1	1	0	13	2	3	1
12006	201	1	2-Mar-10	22	2	14	2	1	1	0	1	2	2	1
12007	201	1	2-Nov-10	15	2	14	2	2	1	0	1	2	6	1
12008	101	1	16-Mar-15	14	2	14	2	1	1	0	4	2	1	1
12009	201	1	16-Dec-15	12	2	14	2	2	1	0	4	2	4	1
12010	101	1	5-Sep-14	11	2	14	2	1	1	0	4	2	1	1
12011	201	1	26-Dec-13	11	2	14	2	1	1	0	4	2	2	1
12012	101	1	31-May-13	10	2	14	2	3	1	0	4	2	1	1
12013	201	1	10-Sep-13	17	2	14	2	3	1	0	4	2	3	1
12014	101	1	14-May-13	23	2	14	2	3	1	0	4	2	2	4
12015	101	1	11-Nov-13	15	2	14	2	1	1	0	4	2	4	1
12016	101	1	30-Oct-12	14	2	14	2	1	1	0	4	2	3	1
12017	101	1	9-Feb-12	19	2	14	2	1	1	0	4	2	2	1
12018	101	1	10-Oct-12	12	2	14	2	1	1	0	4	2	3	1
12019	101	1	9-Oct-12	20	2	14	2	1	1	0	4	2	1	1
12020	101	1	3-Jun-11	18	2	14	2	1	1	0	4	2	1	1
12021	101	1	11-May-11	16	2	14	2	1	1	0	4	2	2	1

12022	101	1	5-Aug-10	19	2	14	2	1	1	0	4	2	3	1
12023	101	1	20-Sep-10	17	2	14	2	1	1	0	4	2	1	2
12024	101	1	26-May-10	15	2	14	2	1	1	0	4	2	2	1
12025	201	1	20-Oct-09	21	2	14	2	1	1	0	4	2	2	1
12026	101	1	29-Feb-08	20	2	14	2	1	1	0	4	2	2	1
12027	101	1	11-Apr-08	14	2	14	2	1	1	0	4	2	3	1
12028	101	1	5-Jan-07	7	2	14	2	1	1	0	4	2	1	1
12029	101	1	11-Jun-07	15	2	14	2	1	1	0	4	2	1	1
12030	101	1	31-Oct-07	9	2	14	2	1	1	0	4	2	1	1
12031	101	1	16-Nov-06	14	2	14	2	1	1	0	4	2	1	1
12032	101	1	15-Sep-06	12	2	14	2	1	1	0	4	2	4	1
12033	201	1	30-Oct-16	19	2	14	2	1	1	0	11	2	2	1
12034	101	1	9-Jul-16	7	2	14	2	1	1	0	11	2	3	1
12035	101	1	30-Oct-16	17	2	14	2	1	1	0	11	2	2	1
12036	101	1	29-May-16	19	2	14	2	1	1	0	11	2	2	1
12037	201	1	25-Jul-15	18	2	14	2	3	1	0	11	2	1	1
12038	201	1	27-Aug-15	12	2	14	2	2	1	0	11	2	3	1
12039	201	1	20-Dec-14	15	2	14	2	1	1	0	11	2	2	1
12040	201	1	31-May-14	19	2	14	2	3	1	0	11	2	3	1
12041	101	1	19-Aug-14	19	2	14	2	1	1	0	11	2	2	1
12042	201	1	24-Oct-13	7	2	14	2	1	1	0	11	2	2	1
12043	201	1	11-Oct-13	7	2	14	2	2	1	0	11	2	2	1
12044	101	1	1-Mar-13	21	2	14	2	1	1	0	11	2	2	1
12045	101	1	4-Aug-12	19	2	14	2	2	1	0	11	2	2	1
12046	101	1	21-Apr-12	19	2	14	2	1	1	0	11	2	3	1
12047	101	1	19-Nov-12	20	2	14	2	1	1	0	11	2	3	1
12048	101	1	17-Oct-12	7	2	14	2	1	1	0	11	2	2	1
12049	201	1	17-Oct-11	16	2	14	2	2	1	0	11	2	3	1
12050	101	1	6-Oct-11	10	2	14	2	1	1	0	11	2	2	1
12051	101	1	23-Aug-11	23	2	14	2	2	1	0	11	2	3	1
12052	101	1	20-Nov-11	16	2	14	2	1	1	0	11	2	2	1
12053	201	1	25-Oct-10	9	2	14	2	1	1	0	11	2	2	1
12054	101	1	24-Sep-10	23	2	14	2	1	1	0	11	2	5	1
12055	101	1	10-May-09	18	2	14	2	2	1	0	11	2	3	1
12056	101	1	10-Apr-08	7	2	14	2	1	1	0	11	2	3	1
12057	201	1	18-Aug-07	18	2	14	2	2	1	0	11	2	2	1
12058	101	1	5-Feb-17	12	2	14	2	1	1	0	11	2	3	1
12059	101	1	8-Aug-17	19	2	14	2	1	1	0	11	2	3	1
12060	201	1	5-Dec-17	14	2	14	2	2	1	0	11	2	3	1
12061	201	1	3-Nov-17	14	2	14	2	2	1	0	11	2	2	1
12062	101	1	19-Nov-17	19	2	14	2	1	1	0	11	2	2	1
12063	201	1	21-Jan-16	17	2	14	2	1	1	0	3	2	3	1
12064	101	1	18-Apr-16	9	2	14	2	1	1	0	3	2	3	1
12065	101	1	21-Jun-16	12	2	14	2	1	1	0	3	2	3	1
12066	101	1	2-Dec-16	18	2	14	2	1	1	0	3	2	1	1
12067	101	1	14-May-16	16	2	14	2	1	1	0	3	2	3	1
12068	101	1	8-Jun-16	17	2	14	2	1	1	0	3	2	2	1
12069	101	1	10-Feb-16	21	2	14	2	1	1	0	3	2	3	1
12070	101	1	9-May-16	17	2	14	2	1	1	0	3	2	3	1
12071	101	1	21-Apr-16	13	2	14	2	1	1	0	3	2	2	1
12072	201	1	7-Dec-16	11	2	14	2	1	1	0	3	2	3	1
12073	101	1	13-Aug-16	15	2	14	2	1	1	0	3	2	3	1
12074	101	1	30-Oct-16	16	2	14	2	1	1	0	3	2	2	1
12075	201	1	14-May-16	16	2	14	2	1	1	0	3	2	1	1
12076	201	1	31-Mar-16	18	2	14	2	2	1	0	3	2	3	1
12077	201	1	9-Nov-16	15	2	14	2	1	1	0	3	2	2	1
12078	101	1	30-May-15	12	2	14	2	1	1	0	3	2	3	1
12079	101	1	16-Mar-15	7	2	14	2	1	1	0	3	2	3	1
12080	101	1	22-Jan-15	15	2	14	2	1	1	0	3	2	1	1
12081	101	1	15-Dec-15	9	2	14	2	1	1	0	3	2	1	1
12082	101	1	30-Dec-15	12	2	14	2	1	1	0	3	2	3	1
12083	101	1	10-Jan-15	8	2	14	2	1	1	0	3	2	1	1
12084	101	1	20-May-15	20	2	14	2	1	1	0	3	2	4	1
12085	201	1	2-Feb-15	21	2	14	2	1	1	0	3	2	2	1
12086	101	1	27-Nov-15	11	2	14	2	1	1	0	3	2	3	1
12087	201	1	26-Sep-15	21	2	14	2	3	1	0	3	2	3	1
12088	201	1	15-Sep-15	13	2	14	2	3	1	0	3	2	3	1
12089	101	1	28-Sep-15	7	2	14	2	1	1	0	3	2	3	1
12090	201	1	8-Nov-15	18	2	14	2	3	1	0	3	2	3	1
12091	201	1	12-Oct-15	16	2	14	2	1	1	0	3	2	3	1
12092	201	1	30-Apr-15	16	2	14	2	2	1	0	3	2	5	1
12093	201	1	23-Jul-15	9	2	14	2	2	1	0	3	2	3	1
12094	201	1	26-Mar-15	16	2	14	2	1	1	0	3	2	2	1
12095	101	1	9-Jan-15	15	2	14	2	1	1	0	3	2	3	1
12096	101	1	24-Feb-14	10	2	14	2	1	1	0	3	2	3	1
12097	101	1	21-Feb-14	11	2	14	2	1	1	0	3	2	3	1
12098	201	1	21-Jan-14	19	2	14	2	3	1	0	3	2	3	1
12099	201	1	16-Feb-14	18	2	14	2	2	1	0	3	2	1	1
12100	101	1	16-Jun-14	21	2	14	2	1	1	0	3	2	4	1
12101	101	1	27-Nov-14	18	2	14	2	1	1	0	3	2	1	1
12102	101	1	12-Nov-14	7	2	14	2	1	1	0	3	2	1	1
12103	201	1	12-Nov-14	2	2	14	2	2	1	0	3	2	1	1
12104	101	1	21-Jul-14	11	2	14	2	2	1	0	3	2	2	1
12105	101	1	13-Feb-14	18	2	14	2	1	1	0	3	2	3	1
12106	101	1	31-Dec-14	18	2	14	2	1	1	0	3	2	4	1
12107	201	1	1-Mar-14	18	2	14	2	1	1	0	3	2	4	1
12108	101	1	17-Apr-14	17	2	14	2	1	1	0	3	2	3	1
12109	201	1	15-Feb-14	7	2	14	2	1	1	0	3	2	2	1
12110	101	1	16-Apr-14	10	2	14	2	1	1	0	3	2	3	1
12111	201	1	1-Nov-14	10	2	14	2	2	1	0	3	2	1	1
12112	101	1	25-Mar-14	17	2	14	2	1	1	0	3	2	3	1
12113	201	1	1-Dec-14	9	2	14	2	1	1	0	3	2	3	1
12114	201	1	18-Dec-13	12	2	14	2	3	1	0	3	2	1	1
12115	101	1	5-Sep-13	18	2	14	2	1	1	0	3	2	4	1
12116	201	1	26-Dec-13	5	2	14	2	1	1	0	3	2	4	1
12117	101	1	24-Oct-13	22	2	14	2	1	1	0	3	2	1	1
12118	201	1	11-Feb-13	22	2	14	2	3	1	0	3	2	2	1
12119	201	1	16-Nov-13	15	2	14	2	3	1	0	3	2	4	1
12120	201	1	30-Aug-13	12	2	14	2	3	1	0	3	2	5	1
12121	101	1	5-Aug-13	21	2	14	2	1	1	0	3	2	5	1
12122	101	1	9-Dec-13	23	2	14	2	1	1	0	3	2	1	1
12123	101	1	31-Aug-13	12	2	14	2	1	1	0	3	2	1	1
12124	101	1	23-Sep-13	8	2	14	2	1	1	0	3	2	3	1
12125	101	1	13-Feb-13	16	2	14	2	1	1	0	3	2	3	1
12126	101	1	10-Nov-13	11	2	14	2	1	1	0	3	2	3	1
12127	101	1	17-Sep-13	20	2	14	2	1	1	0	3	2	3	1
12128	101	1	5-Jan-13	17	2	14	2	1	1	0	3	2	3	1
12129	101	1	22-Jul-13	15	2	14	2	3	1	0	3	2	3	4
12130	101	1	13-Oct-13	11	2	14	2	1	1	0	3	2	3	1
12131	101	1	23-Apr-13	17	2	14	2	1	1	0	3	2	3	1
12132	201	1	31-Oct-13	15	2	14	2	3	1	0	3	2	3	1
12133	201	1	16-Oct-13	16	2	14	2	1	1	0	3	2	2	1
12134	201	1	1-Apr-13	12	2	14	2	2	1	0	3	2	2	1
12135	101	1	13-Apr-12	17	2	14	2	1	1	0	3	2	1	1
12136	201	1	21-Sep-12	10	2	14	2	1	1	0	3	2	4	1
12137	101	1	27-Dec-12	10	2	14	2	1	1	0	3	2	1	2
12138	201	1	19-Nov-12	10	2	14	2	3	1	0	3	2	2	1
12139	201	1	30-Nov-12	15	2	14	2	2	1	0	3	2	1	1
12140	101	1	23-May-12	17	2	14	2	1	1	0	3	2	2	1
12141	101	1	16-Dec-12	18	2	14	2	1	1	0	3	2	5	1
12142	101	1	26-Nov-12	19	2	14	2	1	1	0	3	2	1	1
12143	101	1	16-May-12	9	2	14	2	1	1	0	3	2	4	1
12144	101	1	20-Feb-12	22	2	14	2	1	1	0	3	2	3	1
12145	101	1	24-Apr-12	10	2	14	2	1	1	0	3	2	3	1

12146	101	1	29-Nov-12	10	2	14	2	1	1	0	3	2	3	1
12147	201	1	8-Aug-12	15	2	14	2	2	1	0	3	2	2	1
12148	101	1	1-Apr-12	10	2	14	2	1	1	0	3	2	3	1
12149	101	1	27-Aug-12	17	2	14	2	1	1	0	3	2	3	1
12150	201	1	8-May-12	15	2	14	2	1	1	0	3	2	5	1
12151	101	1	23-May-12	19	2	14	2	1	1	0	3	2	3	1
12152	101	1	10-Nov-12	9	2	14	2	1	1	0	3	2	4	1
12153	101	1	23-May-12	16	2	14	2	1	1	0	3	2	4	1
12154	201	1	22-Oct-12	18	2	14	2	1	1	0	3	2	5	1
12155	201	1	23-Aug-11	20	2	14	2	1	1	0	3	2	1	1
12156	101	1	22-Jul-11	14	2	14	2	1	1	0	3	2	1	2
12157	101	1	16-Nov-11	18	2	14	2	1	1	0	3	2	2	1
12158	101	1	28-May-11	23	2	14	2	1	1	0	3	2	5	1
12159	101	1	27-Jul-11	17	2	14	2	1	1	0	3	2	3	1
12160	101	1	19-Jan-11	17	2	14	2	1	1	0	3	2	3	1
12161	101	1	16-Oct-11	10	2	14	2	1	1	0	3	2	3	1
12162	101	1	23-Sep-11	16	2	14	2	1	1	0	3	2	3	1
12163	201	1	11-Mar-11	15	2	14	2	1	1	0	3	2	1	1
12164	201	1	14-Dec-11	9	2	14	2	2	1	0	3	2	1	1
12165	101	1	11-Nov-11	10	2	14	2	1	1	0	3	2	2	1
12166	201	1	18-Jan-10	23	2	14	2	1	1	0	3	2	3	1
12167	101	1	22-Apr-10	15	2	14	2	1	1	0	3	2	3	1
12168	201	1	3-Oct-10	15	2	14	2	1	1	0	3	2	5	1
12169	101	1	18-Apr-10	18	2	14	2	1	1	0	3	2	1	1
12170	101	1	28-Feb-10	7	2	14	2	1	1	0	3	2	3	1
12171	201	1	16-Dec-10	11	2	14	2	3	1	0	3	2	1	1
12172	101	1	21-Oct-10	18	2	14	2	1	1	0	3	2	2	4
12173	101	1	2-May-10	16	2	14	2	1	1	0	3	2	3	1
12174	101	1	16-Mar-10	10	2	14	2	1	1	0	3	2	3	1
12175	101	1	13-May-10	17	2	14	2	1	1	0	3	2	3	1
12176	101	1	17-Dec-10	10	2	14	2	1	1	0	3	2	3	1
12177	101	1	13-May-10	10	2	14	2	1	1	0	3	2	5	1
12178	101	1	14-May-10	16	2	14	2	1	1	0	3	2	3	4
12179	101	1	23-Apr-10	11	2	14	2	1	1	0	3	2	3	1
12180	101	1	9-Dec-10	12	2	14	2	1	1	0	3	2	3	1
12181	101	1	20-Mar-09	1	2	14	2	1	1	0	3	2	3	1
12182	101	1	1-May-09	20	2	14	2	2	1	0	3	2	1	1
12183	101	1	27-Feb-09	13	2	14	2	1	1	0	3	2	1	1
12184	101	1	4-Aug-09	20	2	14	2	1	1	0	3	2	1	1
12185	101	1	22-Jul-08	21	2	14	2	1	1	0	3	2	3	4
12186	101	1	23-Sep-08	17	2	14	2	2	1	0	3	2	4	1
12187	201	1	29-Jul-08	19	2	14	2	2	1	0	3	2	1	2
12188	101	1	4-Nov-08	17	2	14	2	1	1	0	3	2	1	1
12189	101	1	12-Apr-08	10	2	14	2	1	1	0	3	2	3	1
12190	101	1	18-Oct-08	8	2	14	2	1	1	0	3	2	3	2
12191	101	1	15-May-08	9	2	14	2	1	1	0	3	2	2	1
12192	201	1	21-May-08	12	2	14	2	1	1	0	3	2	1	1
12193	101	1	21-May-08	12	2	14	2	1	1	0	3	2	1	2
12194	201	1	2-Nov-08	9	2	14	2	3	1	0	3	2	2	1
12195	101	1	4-Oct-08	1	2	14	2	1	1	0	3	2	2	1
12196	101	1	17-Aug-08	20	2	14	2	1	1	0	3	2	3	1
12197	101	1	19-Apr-08	16	2	14	2	1	1	0	3	2	3	1
12198	201	1	27-Oct-08	15	2	14	2	3	1	0	3	2	2	1
12199	101	1	27-Sep-07	11	2	14	2	1	1	0	3	2	1	1
12200	201	1	23-Feb-07	19	2	14	2	3	1	0	3	2	2	1
12201	101	1	30-Oct-07	23	2	14	2	1	1	0	3	2	2	1
12202	101	1	9-Apr-07	16	2	14	2	1	1	0	3	2	3	1
12203	101	1	10-May-07	20	2	14	2	1	1	0	3	2	2	1
12204	101	1	16-Mar-07	18	2	14	2	1	1	0	3	2	3	1
12205	101	1	7-Oct-07	9	2	14	2	1	1	0	3	2	6	1
12206	201	1	8-Jan-07	8	2	14	2	3	1	0	3	2	3	1
12207	101	1	24-Sep-06	9	2	14	2	1	1	0	3	2	3	1
12208	201	1	8-Oct-06	20	2	14	2	3	1	0	3	2	2	1
12209	101	1	29-Jul-06	22	2	14	2	1	1	0	3	2	3	1
12210	101	1	15-Aug-06	17	2	14	2	1	1	0	3	2	2	1
12211	101	1	28-Dec-06	15	2	14	2	1	1	0	3	2	2	1
12212	201	1	23-Nov-06	15	2	14	2	2	1	0	3	2	3	1
12213	101	1	10-Mar-06	20	2	14	2	1	1	0	3	2	3	1
12214	101	1	24-Oct-06	17	2	14	2	1	1	0	3	2	5	1
12215	101	1	24-Oct-06	18	2	14	2	1	1	0	3	2	5	1
12216	101	1	10-Nov-06	10	2	14	2	1	1	0	3	2	3	1
12217	101	1	27-Feb-06	16	2	14	2	1	1	0	3	2	2	1
12218	101	1	12-Jan-06	9	2	14	2	1	1	0	3	2	1	1
12219	201	1	1-Aug-06	16	2	14	2	1	1	0	3	2	3	1
12220	201	1	1-Mar-17	15	2	14	2	1	1	0	3	2	5	1
12221	101	1	30-Jan-17	18	2	14	2	1	1	0	3	2	3	1
12222	101	1	12-Jan-17	11	2	14	2	1	1	0	3	2	3	1
12223	101	1	4-Jan-17	16	2	14	2	1	1	0	3	2	3	1
12224	101	1	9-Dec-17	16	2	14	2	1	1	0	3	2	3	1
12225	101	1	23-Nov-17	16	2	14	2	1	1	0	3	2	3	1
12226	101	1	25-May-17	12	2	14	2	1	1	0	3	2	2	1
12227	101	1	16-Mar-17	15	2	14	2	1	1	0	3	2	2	1
12228	201	1	6-Apr-17	16	2	14	2	1	1	0	3	2	2	1
12229	101	1	11-Mar-17	17	2	14	2	1	1	0	3	2	1	1
12230	101	1	20-Oct-17	20	2	14	2	1	1	0	3	2	3	1
12231	101	1	19-Sep-17	17	2	14	2	1	1	0	3	2	2	1
12232	201	1	27-Dec-17	17	2	14	2	2	1	0	3	2	2	1
12233	101	1	10-Nov-16	20	2	14	2	1	1	0	6	2	2	1
12234	101	1	23-Dec-16	21	2	14	2	1	1	0	6	2	2	1
12235	201	1	8-Feb-15	9	2	14	2	1	1	0	6	2	3	1
12236	101	1	31-Mar-14	17	2	14	2	1	1	0	6	2	2	1
12237	101	1	26-Nov-14	11	2	14	2	1	1	0	6	2	2	1
12238	101	1	17-Mar-14	16	2	14	2	1	1	0	6	2	3	1
12239	101	1	11-Sep-13	9	2	14	2	1	1	0	6	2	2	1
12240	101	1	12-Aug-13	22	2	14	2	1	1	0	6	2	3	1
12241	101	1	9-Jun-12	17	2	14	2	1	1	0	6	2	3	1
12242	201	1	25-Mar-12	18	2	14	2	3	1	0	6	2	2	1
12243	201	1	14-Jun-11	13	2	14	2	2	1	0	6	2	2	1
12244	101	1	29-Aug-11	10	2	14	2	2	1	0	6	2	3	1
12245	101	1	10-Sep-10	16	2	14	2	1	1	0	6	2	2	1
12246	101	1	8-May-10	10	2	14	2	1	1	0	6	2	3	1
12247	101	1	26-Nov-09	12	2	14	2	1	1	0	6	2	3	1
12248	101	1	18-Sep-08	23	2	14	2	1	1	0	6	2	3	1
12249	101	1	30-Sep-06	16	2	14	2	1	1	0	6	2	2	1
12250	101	1	5-Dec-17	20	2	14	2	1	1	0	6	2	3	1
12251	101	1	4-May-11	10	2	14	2	1	1	0	13	2	3	1
12252	202	0	28-Mar-16	11	1	15	2	1	1	0	1	2	2	1
12253	202	0	12-Jul-13	18	1	15	2	1	1	0	1	2	5	2
12254	202	0	12-Jul-13	18	1	15	2	1	1	0	1	2	3	1
12255	202	0	28-Nov-14	16	1	15	2	1	1	0	4	2	1	1
12256	202	0	12-Oct-17	0	1	15	2	2	1	0	10	2	6	2
12257	202	0	29-Sep-14	16	1	15	2	1	1	0	4	2	2	2
12258	202	0	16-Feb-12	12	1	15	2	1	1	0	4	2	2	1
12259	202	0	17-May-13	17	1	15	2	1	1	0	3	2	2	2
12260	202	0	5-Dec-16	22	1	15	2	1	1	0	6	2	2	1
12261	202	0	19-Jun-15	18	1	15	2	1	1	0	6	2	2	1
12262	202	0	29-Sep-15	22	1	15	2	1	1	0	6	2	2	1
12263	202	0	29-Sep-15	19	1	15	2	1	1	0	6	2	2	1
12264	202	0	16-Aug-13	11	1	15	2	3	1	0	6	2	2	2
12265	202	0	10-Aug-16	12	1	15	2	3	1	0	6	2	3	1
12266	202	0	6-Aug-13	16	1	15	2	1	1	0	6	2	3	1
12267	202	0	10-May-13	1	1	15	2	1	1	0	6	2	2	1
12268	202	0	7-Sep-12	17	1	15	2	1	1	0	6	2	2	1
12269	202	0	25-Mar-13	0	1	15	2	1	1	0	10	2	7	1

12270	202	0	30-Nov-09	23	1	15	2	1	1	0	10	2	7	2
12271	202	0	16-May-08	1	1	15	2	1	1	0	10	2	7	2
12272	202	0	15-Sep-15	15	1	15	2	1	1	0	7	2	1	1
12273	202	0	7-Aug-10	11	1	15	2	1	1	0	7	2	2	1
12274	202	0	17-Sep-07	17	1	15	2	1	1	0	7	2	2	1
12275	202	0	22-Mar-13	12	1	15	2	1	1	0	13	2	4	1
12276	202	0	1-Oct-10	3	1	15	2	1	1	0	13	2	1	1
12277	202	0	13-Mar-10	14	1	15	2	1	1	0	13	2	3	1
12278	202	0	9-Oct-09	17	1	15	2	1	1	0	13	2	1	2
12279	202	0	5-Sep-09	19	1	15	2	1	1	0	13	2	1	2
12280	202	0	22-Jul-08	18	1	15	2	1	1	0	13	2	1	1
12281	202	0	28-Dec-07	21	1	15	2	3	1	0	13	2	2	2
12282	202	0	27-Dec-07	17	1	15	2	1	1	0	13	2	2	1
12283	202	0	19-Oct-07	17	1	15	2	1	1	0	13	2	1	1
12284	202	0	17-Nov-07	12	1	15	2	1	1	0	13	2	1	1
12285	202	0	7-Jan-11	21	1	15	2	1	1	0	1	2	2	2
12286	202	0	9-Jul-10	9	1	15	2	2	1	0	1	2	2	2
12287	202	0	5-Nov-08	17	1	15	2	1	1	0	1	2	6	2
12288	202	0	20-May-16	20	1	15	2	1	1	0	4	2	1	1
12289	202	0	6-Jan-16	18	1	15	2	1	1	0	4	2	2	2
12290	202	0	2-Nov-16	20	1	15	2	1	1	0	4	2	2	1
12291	202	0	12-Oct-15	19	1	15	2	1	1	0	4	2	2	1
12292	202	0	12-Oct-15	20	1	15	2	1	1	0	4	2	1	1
12293	202	0	14-Dec-15	18	1	15	2	1	1	0	4	2	1	1
12294	202	0	13-Oct-15	11	1	15	2	2	1	0	4	2	2	1
12295	202	0	5-Jun-15	17	1	15	2	1	1	0	4	2	2	2
12296	202	0	30-Mar-14	11	1	15	2	1	1	0	4	2	1	1
12297	202	0	22-Apr-14	10	1	15	2	1	1	0	4	2	2	1
12298	202	0	24-Nov-14	17	1	15	2	1	1	0	4	2	1	1
12299	202	0	29-Apr-14	10	1	15	2	1	1	0	4	2	4	1
12300	202	0	24-Mar-14	8	1	15	2	1	1	0	4	2	1	1
12301	202	0	15-Nov-14	16	1	15	2	1	1	0	4	2	1	1
12302	202	0	10-Nov-14	17	1	15	2	1	1	0	4	2	1	1
12303	202	0	6-Aug-14	20	1	15	2	1	1	0	4	2	2	1
12304	202	0	10-Dec-14	13	1	15	2	1	1	0	4	2	1	1
12305	202	0	2-Apr-13	16	1	15	2	1	1	0	4	2	4	1
12306	202	0	28-Mar-13	11	1	15	2	1	1	0	4	2	2	1
12307	202	0	1-Sep-13	22	1	15	2	1	1	0	4	2	1	1
12308	202	0	30-May-13	20	1	15	2	1	1	0	4	2	1	1
12309	202	0	26-Feb-13	14	1	15	2	1	1	0	4	2	1	1
12310	202	0	4-Jul-13	17	1	15	2	1	1	0	4	2	5	1
12311	202	0	18-Jun-12	20	1	15	2	1	1	0	4	2	1	1
12312	202	0	4-Jun-12	18	1	15	2	1	1	0	4	2	3	1
12313	202	0	30-Dec-11	10	1	15	2	1	1	0	4	2	6	2
12314	202	0	14-Mar-11	10	1	15	2	1	1	0	4	2	2	2
12315	202	0	14-Dec-11	15	1	15	2	1	1	0	4	2	1	1
12316	202	0	4-Jan-10	17	1	15	2	1	1	0	4	2	4	1
12317	202	0	3-Oct-10	16	1	15	2	2	1	0	4	2	6	2
12318	202	0	4-Mar-10	18	1	15	2	1	1	0	4	2	1	1
12319	202	0	9-Jan-10	12	1	15	2	1	1	0	4	2	1	1
12320	202	0	8-Nov-10	17	1	15	2	1	1	0	4	2	1	2
12321	202	0	7-Sep-10	7	1	15	2	1	1	0	4	2	2	1
12322	202	0	4-Apr-10	20	1	15	2	2	1	0	4	2	1	2
12323	202	0	20-Dec-09	22	1	15	2	1	1	0	4	2	1	1
12324	202	0	30-Apr-09	17	1	15	2	1	1	0	4	2	1	1
12325	202	0	21-Mar-09	21	1	15	2	1	1	0	4	2	1	1
12326	202	0	1-Jul-08	10	1	15	2	1	1	0	4	2	1	1
12327	202	0	12-Jun-08	23	1	15	2	1	1	0	4	2	1	1
12328	202	0	22-May-08	17	1	15	2	3	1	0	4	2	1	1
12329	202	0	23-May-08	10	1	15	2	1	1	0	4	2	4	1
12330	202	0	5-Aug-08	15	1	15	2	1	1	0	4	2	1	1
12331	202	0	24-Sep-08	16	1	15	2	1	1	0	4	2	2	1
12332	202	0	6-Jul-07	14	1	15	2	1	1	0	4	2	1	1
12333	202	0	13-Dec-07	9	1	15	2	1	1	0	4	2	1	1
12334	202	0	5-Jul-07	17	1	15	2	1	1	0	4	2	1	1
12335	202	0	29-Jun-06	17	1	15	2	1	1	0	4	2	1	1
12336	202	0	24-Jun-06	18	1	15	2	1	1	0	4	2	4	1
12337	202	0	3-Nov-17	18	1	15	2	2	1	0	4	2	1	1
12338	202	0	27-Dec-17	19	1	15	2	1	1	0	4	2	4	1
12339	202	0	22-Nov-16	13	1	15	2	3	1	0	11	2	3	1
12340	202	0	9-Apr-16	21	1	15	2	3	1	0	11	2	3	1
12341	202	0	14-Oct-16	20	1	15	2	3	1	0	11	2	2	1
12342	202	0	20-Oct-16	7	1	15	2	3	1	0	11	2	2	1
12343	202	0	21-May-13	22	1	15	2	3	1	0	11	2	3	2
12344	202	0	9-May-13	14	1	15	2	1	1	0	11	2	3	1
12345	202	0	3-Oct-13	21	1	15	2	1	1	0	11	2	3	1
12346	202	0	29-Nov-10	16	1	15	2	1	1	0	11	2	2	1
12347	202	0	22-Aug-10	8	1	15	2	1	1	0	11	2	3	2
12348	202	0	15-Feb-08	18	1	15	2	3	1	0	11	2	2	1
12349	202	0	31-Jul-08	20	1	15	2	1	1	0	11	2	2	1
12350	202	0	13-Oct-16	11	1	15	2	1	1	0	3	2	4	1
12351	202	0	9-Oct-16	0	1	15	2	1	1	0	3	2	2	1
12352	202	0	18-May-16	11	1	15	2	3	1	0	3	2	2	1
12353	202	0	18-Mar-16	1	1	15	2	1	1	0	3	2	1	2
12354	202	0	21-Oct-16	15	1	15	2	3	1	0	3	2	2	1
12355	202	0	21-Jun-16	22	1	15	2	1	1	0	3	2	1	1
12356	202	0	22-Dec-16	16	1	15	2	3	1	0	3	2	1	1
12357	202	0	29-Jan-16	13	1	15	2	1	1	0	3	2	1	1
12358	202	0	27-Dec-16	19	1	15	2	1	1	0	3	2	2	1
12359	202	0	11-Feb-16	18	1	15	2	1	1	0	3	2	2	1
12360	202	0	11-Dec-16	17	1	15	2	3	1	0	3	2	1	1
12361	202	0	30-May-16	17	1	15	2	1	1	0	3	2	3	1
12362	202	0	20-Sep-16	6	1	15	2	1	1	0	3	2	3	1
12363	202	0	6-Jan-16	18	1	15	2	1	1	0	3	2	3	1
12364	202	0	28-Apr-16	19	1	15	2	2	1	0	3	2	2	1
12365	202	0	23-Dec-16	12	1	15	2	1	1	0	3	2	3	1
12366	202	0	27-Apr-16	18	1	15	2	3	1	0	3	2	2	1
12367	202	0	11-Dec-16	19	1	15	2	1	1	0	3	2	1	1
12368	202	0	28-Jun-16	20	1	15	2	1	1	0	3	2	3	1
12369	202	0	5-Jan-16	11	1	15	2	1	1	0	3	2	1	1
12370	202	0	11-Dec-16	19	1	15	2	1	1	0	3	2	1	1
12371	202	0	25-Feb-16	17	1	15	2	2	1	0	3	2	3	1
12372	202	0	21-Jun-16	18	1	15	2	1	1	0	3	2	2	4
12373	202	0	27-Sep-16	19	1	15	2	3	1	0	3	2	3	1
12374	202	0	18-Nov-16	17	1	15	2	2	1	0	3	2	3	2
12375	202	0	18-Nov-15	12	1	15	2	2	1	0	3	2	4	1
12376	202	0	28-May-15	12	1	15	2	1	1	0	3	2	2	1
12377	202	0	25-Nov-15	15	1	15	2	3	1	0	3	2	5	1
12378	202	0	26-Nov-15	13	1	15	2	1	1	0	3	2	3	1
12379	202	0	10-Dec-15	15	1	15	2	1	1	0	3	2	5	1
12380	202	0	25-May-15	17	1	15	2	1	1	0	3	2	3	1
12381	202	0	31-Jul-15	19	1	15	2	2	1	0	3	2	3	1
12382	202	0	24-Dec-15	17	1	15	2	1	1	0	3	2	1	1
12383	202	0	11-Jun-15	18	1	15	2	1	1	0	3	2	1	1
12384	202	0	12-Oct-15	15	1	15	2	3	1	0	3	2	3	1
12385	202	0	13-May-15	16	1	15	2	1	1	0	3	2	3	1
12386	202	0	29-Dec-15	16	1	15	2	1	1	0	3	2	2	1
12387	202	0	19-Dec-15	12	1	15	2	3	1	0	3	2	3	2
12388	202	0	30-Dec-15	10	1	15	2	1	1	0	3	2	3	1
12389	202	0	22-May-14	10	1	15	2	1	1	0	3	2	4	1
12390	202	0	11-Nov-14	18	1	15	2	1	1	0	3	2	3	1
12391	202	0	8-May-14	22	1	15	2	1	1	0	3	2	1	1
12392	202	0	21-May-14	17	1	15	2	1	1	0	3	2	3	1
12393	202	0	25-Sep-14	12	1	15	2	1	1	0	3	2	1	1

12394	202	0	11-Sep-14	16	1	15	2	1	1	0	3	2	1	1
12395	202	0	10-Sep-14	12	1	15	2	1	1	0	3	2	1	1
12396	202	0	3-Nov-14	18	1	15	2	2	1	0	3	2	2	1
12397	202	0	12-Nov-14	17	1	15	2	1	1	0	3	2	2	2
12398	202	0	21-May-14	15	1	15	2	1	1	0	3	2	3	2
12399	202	0	28-Apr-14	16	1	15	2	3	1	0	3	2	1	1
12400	202	0	31-May-14	2	1	15	2	1	1	0	3	2	2	2
12401	202	0	15-Sep-14	18	1	15	2	2	1	0	3	2	2	1
12402	202	0	8-Apr-14	6	1	15	2	1	1	0	3	2	3	1
12403	202	0	14-Jul-14	16	1	15	2	3	1	0	3	2	2	2
12404	202	0	28-Jan-14	21	1	15	2	1	1	0	3	2	5	1
12405	202	0	21-Nov-14	12	1	15	2	1	1	0	3	2	1	1
12406	202	0	11-Oct-14	21	1	15	2	1	1	0	3	2	3	1
12407	202	0	20-Nov-14	17	1	15	2	1	1	0	3	2	2	1
12408	202	0	26-Nov-14	11	1	15	2	1	1	0	3	2	3	2
12409	202	0	6-May-14	20	1	15	2	1	1	0	3	2	4	1
12410	202	0	18-May-14	8	1	15	2	1	1	0	3	2	1	1
12411	202	0	28-Nov-14	16	1	15	2	1	1	0	3	2	3	1
12412	202	0	27-Dec-14	17	1	15	2	1	1	0	3	2	3	2
12413	202	0	20-Apr-14	19	1	15	2	2	1	0	3	2	3	1
12414	202	0	22-Apr-14	17	1	15	2	1	1	0	3	2	3	1
12415	202	0	10-Jun-14	14	1	15	2	1	1	0	3	2	3	1
12416	202	0	25-Apr-14	17	1	15	2	1	1	0	3	2	2	2
12417	202	0	29-Apr-14	20	1	15	2	1	1	0	3	2	3	1
12418	202	0	17-May-14	13	1	15	2	1	1	0	3	2	3	1
12419	202	0	11-Apr-14	7	1	15	2	1	1	0	3	2	3	1
12420	202	0	15-Apr-14	19	1	15	2	1	1	0	3	2	3	1
12421	202	0	9-Sep-13	19	1	15	2	1	1	0	3	2	3	1
12422	202	0	15-Jul-13	19	1	15	2	3	1	0	3	2	2	1
12423	202	0	31-Oct-13	20	1	15	2	2	1	0	3	2	3	1
12424	202	0	6-Apr-13	16	1	15	2	3	1	0	3	2	2	2
12425	202	0	5-Nov-13	17	1	15	2	1	1	0	3	2	1	1
12426	202	0	21-Nov-13	16	1	15	2	1	1	0	3	2	1	1
12427	202	0	19-Sep-13	16	1	15	2	1	1	0	3	2	1	1
12428	202	0	7-Dec-13	22	1	15	2	2	1	0	3	2	1	2
12429	202	0	23-Nov-13	22	1	15	2	2	1	0	3	2	1	1
12430	202	0	12-Sep-13	21	1	15	2	1	1	0	3	2	2	1
12431	202	0	19-Dec-13	18	1	15	2	2	1	0	3	2	3	2
12432	202	0	19-Feb-13	23	1	15	2	1	1	0	3	2	1	1
12433	202	0	12-Aug-13	22	1	15	2	1	1	0	3	2	1	2
12434	202	0	11-Mar-13	18	1	15	2	3	1	0	3	2	3	1
12435	202	0	9-Aug-13	18	1	15	2	2	1	0	3	2	3	1
12436	202	0	8-Apr-13	17	1	15	2	1	1	0	3	2	2	2
12437	202	0	4-Dec-13	13	1	15	2	1	1	0	3	2	1	2
12438	202	0	10-Dec-13	0	1	15	2	1	1	0	3	2	4	2
12439	202	0	21-Oct-13	19	1	15	2	1	1	0	3	2	5	2
12440	202	0	25-Nov-13	19	1	15	2	2	1	0	3	2	3	1
12441	202	0	8-Oct-13	18	1	15	2	1	1	0	3	2	2	1
12442	202	0	14-Mar-13	16	1	15	2	1	1	0	3	2	2	2
12443	202	0	14-Oct-13	19	1	15	2	1	1	0	3	2	2	1
12444	202	0	26-Dec-13	10	1	15	2	1	1	0	3	2	1	1
12445	202	0	27-Sep-13	17	1	15	2	1	1	0	3	2	3	1
12446	202	0	15-Mar-13	3	1	15	2	1	1	0	3	2	4	1
12447	202	0	18-Oct-13	10	1	15	2	1	1	0	3	2	4	2
12448	202	0	11-Dec-13	17	1	15	2	1	1	0	3	2	3	1
12449	202	0	26-Nov-12	16	1	15	2	1	1	0	3	2	4	1
12450	202	0	25-Dec-12	18	1	15	2	1	1	0	3	2	1	1
12451	202	0	5-Dec-12	17	1	15	2	1	1	0	3	2	1	1
12452	202	0	18-Jun-12	18	1	15	2	1	1	0	3	2	1	1
12453	202	0	30-Mar-12	17	1	15	2	1	1	0	3	2	2	1
12454	202	0	24-Sep-12	17	1	15	2	1	1	0	3	2	3	2
12455	202	0	25-Feb-12	21	1	15	2	1	1	0	3	2	1	2
12456	202	0	9-Dec-12	12	1	15	2	3	1	0	3	2	2	1
12457	202	0	9-Oct-12	19	1	15	2	1	1	0	3	2	4	2
12458	202	0	20-Dec-12	13	1	15	2	2	1	0	3	2	1	1
12459	202	0	29-Nov-12	11	1	15	2	3	1	0	3	2	2	1
12460	202	0	6-Dec-12	15	1	15	2	1	1	0	3	2	3	2
12461	202	0	29-Mar-12	13	1	15	2	1	1	0	3	2	1	2
12462	202	0	29-Apr-11	10	1	15	2	1	1	0	3	2	1	1
12463	202	0	22-Dec-11	11	1	15	2	1	1	0	3	2	5	1
12464	202	0	12-Apr-11	12	1	15	2	1	1	0	3	2	2	2
12465	202	0	26-Oct-11	20	1	15	2	1	1	0	3	2	3	1
12466	202	0	7-Apr-11	20	1	15	2	1	1	0	3	2	2	1
12467	202	0	17-Nov-11	20	1	15	2	1	1	0	3	2	1	2
12468	202	0	7-Aug-11	3	1	15	2	1	1	0	3	2	2	1
12469	202	0	7-Apr-11	17	1	15	2	1	1	0	3	2	3	2
12470	202	0	29-Dec-11	10	1	15	2	1	1	0	3	2	3	1
12471	202	0	4-Mar-11	17	1	15	2	2	1	0	3	2	3	1
12472	202	0	11-Jun-10	17	1	15	2	2	1	0	3	2	1	1
12473	202	0	15-May-10	17	1	15	2	1	1	0	3	2	6	1
12474	202	0	9-Sep-10	10	1	15	2	1	1	0	3	2	6	1
12475	202	0	26-May-10	0	1	15	2	1	1	0	3	2	1	2
12476	202	0	10-Jul-10	13	1	15	2	1	1	0	3	2	1	1
12477	202	0	27-Mar-10	17	1	15	2	1	1	0	3	2	3	1
12478	202	0	10-Jun-10	16	1	15	2	1	1	0	3	2	1	1
12479	202	0	30-May-10	14	1	15	2	1	1	0	3	2	3	1
12480	202	0	12-Dec-09	15	1	15	2	1	1	0	3	2	1	2
12481	202	0	14-May-09	18	1	15	2	1	1	0	3	2	1	2
12482	202	0	29-Dec-09	18	1	15	2	1	1	0	3	2	4	1
12483	202	0	18-Dec-09	18	1	15	2	1	1	0	3	2	3	1
12484	202	0	8-May-09	17	1	15	2	1	1	0	3	2	3	1
12485	202	0	22-Dec-09	16	1	15	2	1	1	0	3	2	2	2
12486	202	0	16-Oct-09	22	1	15	2	1	1	0	3	2	2	2
12487	202	0	9-Nov-09	18	1	15	2	1	1	0	3	2	2	1
12488	202	0	26-May-09	17	1	15	2	1	1	0	3	2	3	1
12489	202	0	30-Jun-09	17	1	15	2	1	1	0	3	2	1	1
12490	202	0	14-Nov-09	16	1	15	2	1	1	0	3	2	2	2
12491	202	0	8-Feb-08	9	1	15	2	1	1	0	3	2	2	2
12492	202	0	23-Jan-08	14	1	15	2	1	1	0	3	2	1	2
12493	202	0	11-Dec-08	23	1	15	2	1	1	0	3	2	2	1
12494	202	0	27-Sep-08	22	1	15	2	1	1	0	3	2	3	1
12495	202	0	5-Mar-08	17	1	15	2	1	1	0	3	2	2	2
12496	202	0	29-May-08	20	1	15	2	1	1	0	3	2	3	1
12497	202	0	9-Jan-08	17	1	15	2	1	1	0	3	2	2	1
12498	202	0	24-Oct-08	11	1	15	2	1	1	0	3	2	2	2
12499	202	0	26-Aug-08	21	1	15	2	1	1	0	3	2	3	1
12500	202	0	23-Sep-08	19	1	15	2	1	1	0	3	2	7	1
12501	202	0	10-Oct-08	18	1	15	2	1	1	0	3	2	1	1
12502	202	0	23-May-07	11	1	15	2	1	1	0	3	2	1	1
12503	202	0	23-Aug-07	19	1	15	2	3	1	0	3	2	2	2
12504	202	0	20-Apr-07	11	1	15	2	1	1	0	3	2	1	1
12505	202	0	5-Jun-07	18	1	15	2	1	1	0	3	2	2	1
12506	202	0	4-Sep-07	21	1	15	2	1	1	0	3	2	2	1
12507	202	0	15-Mar-07	21	1	15	2	1	1	0	3	2	2	1
12508	202	0	21-Jan-07	16	1	15	2	3	1	0	3	2	3	2
12509	202	0	23-Aug-07	16	1	15	2	1	1	0	3	2	3	2
12510	202	0	24-Jun-07	18	1	15	2	1	1	0	3	2	1	1
12511	202	0	13-Nov-07	18	1	15	2	1	1	0	3	2	2	1
12512	202	0	2-Jan-07	17	1	15	2	1	1	0	3	2	3	1
12513	202	0	11-Nov-07	17	1	15	2	1	1	0	3	2	1	2
12514	202	0	23-Oct-07	17	1	15	2	1	1	0	3	2	3	1
12515	202	0	25-Dec-07	17	1	15	2	1	1	0	3	2	2	1
12516	202	0	1-Jun-06	17	1	15	2	1	1	0	3	2	2	2
12517	202	0	12-Dec-06	19	1	15	2	1	1	0	3	2	2	2

12518	202	0	12-Oct-06	15	1	15	2	1	1	0	3	2	1	1
12519	202	0	5-May-06	14	1	15	2	1	1	0	3	2	1	1
12520	202	0	28-Jan-06	22	1	15	2	1	1	0	3	2	3	2
12521	202	0	31-May-06	16	1	15	2	1	1	0	3	2	2	1
12522	202	0	25-Feb-06	16	1	15	2	1	1	0	3	2	1	1
12523	202	0	11-Jan-06	18	1	15	2	1	1	0	3	2	1	1
12524	202	0	5-Dec-06	19	1	15	2	1	1	0	3	2	3	1
12525	202	0	29-Sep-06	10	1	15	2	1	1	0	3	2	2	2
12526	202	0	24-Oct-06	11	1	15	2	1	1	0	3	2	2	2
12527	202	0	3-Jan-06	11	1	15	2	1	1	0	3	2	2	2
12528	202	0	30-Nov-17	17	1	15	2	1	1	0	3	2	1	1
12529	202	0	19-Oct-17	19	1	15	2	2	1	0	3	2	3	1
12530	202	0	22-Apr-17	20	1	15	2	1	1	0	3	2	5	1
12531	202	0	7-Mar-17	10	1	15	2	1	1	0	3	2	3	1
12532	202	0	22-Sep-17	14	1	15	2	1	1	0	3	2	3	1
12533	202	0	10-Apr-17	12	1	15	2	1	1	0	3	2	2	1
12534	202	0	18-Apr-17	9	1	15	2	1	1	0	3	2	1	1
12535	202	0	20-Nov-17	10	1	15	2	1	1	0	3	2	1	1
12536	202	0	22-Jun-17	8	1	15	2	1	1	0	3	2	1	1
12537	202	0	11-Jan-17	14	1	15	2	1	1	0	3	2	2	2
12538	202	0	10-Nov-17	15	1	15	2	2	1	0	3	2	5	1
12539	202	0	3-Aug-17	3	1	15	2	2	1	0	3	2	1	1
12540	202	0	10-Apr-17	20	1	15	2	3	1	0	3	2	3	1
12541	202	0	4-Jan-17	22	1	15	2	3	1	0	3	2	3	1
12542	202	0	21-Dec-17	16	1	15	2	1	1	0	3	2	3	1
12543	202	0	21-Aug-16	20	1	15	2	1	1	0	6	2	2	1
12544	202	0	10-Nov-15	18	1	15	2	3	1	0	6	2	2	1
12545	202	0	24-Nov-14	16	1	15	2	3	1	0	6	2	3	1
12546	202	0	29-May-13	15	1	15	2	1	1	0	6	2	2	1
12547	202	0	27-Apr-13	15	1	15	2	1	1	0	6	2	3	1
12548	202	0	29-Jun-12	10	1	15	2	1	1	0	6	2	2	2
12549	202	0	27-Dec-12	15	1	15	2	1	1	0	6	2	2	1
12550	202	0	15-Dec-11	12	1	15	2	1	1	0	6	2	2	1
12551	202	0	7-Aug-10	16	1	15	2	1	1	0	6	2	2	2
12552	202	0	8-May-09	12	1	15	2	1	1	0	6	2	2	1
12553	202	0	24-Dec-09	13	1	15	2	1	1	0	6	2	2	2
12554	202	0	26-Nov-09	14	1	15	2	1	1	0	6	2	2	2
12555	202	0	29-Aug-09	2	1	15	2	2	1	0	6	2	3	1
12556	202	0	16-Oct-08	14	1	15	2	1	1	0	6	2	6	2
12557	202	0	16-Oct-08	14	1	15	2	1	1	0	6	2	2	2
12558	202	0	8-Oct-08	13	1	15	2	1	1	0	6	2	2	2
12559	202	0	31-Aug-08	12	1	15	2	1	1	0	6	2	3	1
12560	202	0	25-Feb-08	18	1	15	2	1	1	0	6	2	1	1
12561	202	0	16-Oct-08	13	1	15	2	1	1	0	6	2	2	1
12562	202	0	15-Mar-07	17	1	15	2	1	1	0	6	2	2	1
12563	202	0	18-Oct-07	19	1	15	2	1	1	0	6	2	3	1
12564	202	0	16-Aug-07	16	1	15	2	1	1	0	6	2	3	1
12565	202	0	6-Nov-06	17	1	15	2	1	1	0	6	2	3	1
12566	202	0	17-Nov-16	15	1	15	2	1	1	0	13	2	1	1
12567	202	0	20-Feb-11	17	1	15	2	1	1	0	13	2	1	1
12568	202	0	5-Mar-08	20	1	15	2	1	1	0	13	2	7	1
12569	202	0	1-Aug-07	18	1	15	2	1	1	0	13	2	1	1
12570	202	0	8-Apr-07	8	1	15	2	1	1	0	13	2	4	1
12571	202	0	10-Feb-13	19	2	15	2	3	1	0	1	2	3	1
12572	202	0	8-May-13	19	2	15	2	1	1	0	1	2	3	1
12573	202	0	29-Jul-13	23	2	15	2	2	1	0	1	2	3	1
12574	202	0	22-Nov-17	15	2	15	2	3	1	0	6	2	2	1
12575	202	0	12-Jan-12	9	2	15	2	1	1	0	6	2	2	1
12576	202	0	2-Jun-14	20	2	15	2	1	1	0	6	2	3	2
12577	202	0	17-Jul-14	2	2	15	2	1	1	0	10	2	7	1
12578	202	0	7-May-13	18	2	15	2	1	1	0	6	2	3	1
12579	202	0	1-Jul-12	16	2	15	2	3	1	0	10	2	7	2
12580	202	0	1-Jul-12	16	2	15	2	3	1	0	10	2	7	2
12581	202	0	18-Oct-11	18	2	15	2	1	1	0	10	2	7	2
12582	202	0	8-Sep-10	17	2	15	2	1	1	0	7	2	2	2
12583	202	0	29-Mar-16	18	2	15	2	3	1	0	13	2	5	2
12584	202	0	22-May-15	8	2	15	2	1	1	0	13	2	1	1
12585	202	0	24-Feb-06	16	2	15	2	1	1	0	13	2	1	2
12586	202	0	17-Sep-10	14	2	15	2	1	1	0	1	2	1	2
12587	202	0	26-Dec-09	16	2	15	2	1	1	0	1	2	3	2
12588	202	0	30-May-08	14	2	15	2	1	1	0	1	2	1	2
12589	202	0	7-Feb-07	18	2	15	2	1	1	0	1	2	1	1
12590	202	0	5-Nov-08	17	2	15	2	1	1	0	1	2	1	2
12591	202	0	5-Nov-08	17	2	15	2	1	1	0	1	2	7	2
12592	202	0	28-Sep-16	16	2	15	2	3	1	0	4	2	1	1
12593	202	0	8-May-13	20	2	15	2	1	1	0	4	2	3	1
12594	202	0	5-Apr-13	16	2	15	2	1	1	0	4	2	4	1
12595	202	0	11-Mar-11	15	2	15	2	1	1	0	4	2	1	1
12596	202	0	18-Apr-08	14	2	15	2	1	1	0	4	2	2	1
12597	202	0	27-Oct-07	22	2	15	2	1	1	0	4	2	1	1
12598	202	0	9-Oct-07	11	2	15	2	1	1	0	4	2	3	1
12599	202	0	2-Jul-06	19	2	15	2	1	1	0	4	2	3	1
12600	202	0	29-Dec-06	11	2	15	2	1	1	0	4	2	1	1
12601	202	0	24-Nov-17	13	2	15	2	1	1	0	4	2	1	1
12602	202	0	14-Oct-15	21	2	15	2	1	1	0	11	2	2	1
12603	202	0	20-Jun-14	22	2	15	2	2	1	0	11	2	3	2
12604	202	0	27-Dec-13	17	2	15	2	1	1	0	11	2	2	1
12605	202	0	8-Sep-13	11	2	15	2	1	1	0	11	2	2	1
12606	202	0	17-Jun-12	22	2	15	2	1	1	0	11	2	3	1
12607	202	0	23-Jun-12	13	2	15	2	1	1	0	11	2	2	1
12608	202	0	22-Jun-12	16	2	15	2	1	1	0	11	2	2	2
12609	202	0	25-Oct-10	10	2	15	2	1	1	0	11	2	3	2
12610	202	0	9-Oct-09	12	2	15	2	1	1	0	11	2	1	2
12611	202	0	30-Oct-07	11	2	15	2	1	1	0	11	2	2	1
12612	202	0	26-Dec-16	10	2	15	2	1	1	0	3	2	3	1
12613	202	0	14-Dec-16	17	2	15	2	1	1	0	3	2	3	1
12614	202	0	14-Apr-16	23	2	15	2	1	1	0	3	2	4	1
12615	202	0	1-May-16	21	2	15	2	1	1	0	3	2	1	1
12616	202	0	13-Dec-16	12	2	15	2	1	1	0	3	2	2	1
12617	202	0	19-May-15	12	2	15	2	3	1	0	3	2	4	1
12618	202	0	14-Oct-15	18	2	15	2	1	1	0	3	2	3	1
12619	202	0	28-Oct-15	5	2	15	2	2	1	0	3	2	3	1
12620	202	0	27-Nov-15	12	2	15	2	1	1	0	3	2	5	1
12621	202	0	15-Feb-14	15	2	15	2	1	1	0	3	2	1	1
12622	202	0	20-Oct-14	21	2	15	2	1	1	0	3	2	1	1
12623	202	0	18-Nov-14	15	2	15	2	2	1	0	3	2	3	1
12624	202	0	2-Dec-14	11	2	15	2	1	1	0	3	2	2	2
12625	202	0	14-Mar-14	13	2	15	2	1	1	0	3	2	1	1
12626	202	0	17-Feb-14	13	2	15	2	1	1	0	3	2	4	1
12627	202	0	17-May-14	16	2	15	2	1	1	0	3	2	3	1
12628	202	0	31-Oct-13	19	2	15	2	1	1	0	3	2	5	1
12629	202	0	20-Dec-13	1	2	15	2	3	1	0	3	2	1	1
12630	202	0	7-Dec-13	17	2	15	2	2	1	0	3	2	3	1
12631	202	0	25-Nov-13	19	2	15	2	1	1	0	3	2	2	1
12632	202	0	2-Jun-13	18	2	15	2	1	1	0	3	2	7	1
12633	202	0	29-May-12	17	2	15	2	1	1	0	3	2	1	1
12634	202	0	22-Nov-12	21	2	15	2	1	1	0	3	2	1	1
12635	202	0	18-Oct-12	23	2	15	2	1	1	0	3	2	3	2
12636	202	0	28-Aug-12	23	2	15	2	1	1	0	3	2	1	1
12637	202	0	10-Oct-12	17	2	15	2	1	1	0	3	2	4	2
12638	202	0	30-Jun-11	17	2	15	2	1	1	0	3	2	1	2
12639	202	0	20-Jun-11	18	2	15	2	1	1	0	3	2	5	1
12640	202	0	28-Oct-11	10	2	15	2	1	1	0	3	2	3	2
12641	202	0	4-Jan-11	18	2	15	2	1	1	0	3	2	3	1

12642	202	0	20-Sep-10	18	2	15	2	1	1	0	3	2	1	2
12643	202	0	28-Mar-10	17	2	15	2	1	1	0	3	2	1	2
12644	202	0	25-Jan-10	12	2	15	2	1	1	0	3	2	1	1
12645	202	0	20-Dec-10	17	2	15	2	1	1	0	3	2	3	1
12646	202	0	29-Nov-10	15	2	15	2	1	1	0	3	2	3	1
12647	202	0	18-Sep-10	17	2	15	2	1	1	0	3	2	3	1
12648	202	0	29-Nov-10	15	2	15	2	1	1	0	3	2	3	1
12649	202	0	4-Jan-09	18	2	15	2	1	1	0	3	2	1	2
12650	202	0	25-Nov-09	18	2	15	2	1	1	0	3	2	3	1
12651	202	0	19-Sep-08	11	2	15	2	1	1	0	3	2	3	1
12652	202	0	10-Oct-08	16	2	15	2	1	1	0	3	2	3	2
12653	202	0	19-Apr-07	10	2	15	2	1	1	0	3	2	3	1
12654	202	0	25-Nov-07	10	2	15	2	1	1	0	3	2	1	1
12655	202	0	9-Jan-06	22	2	15	2	2	1	0	3	2	2	1
12656	202	0	21-Dec-06	7	2	15	2	1	1	0	3	2	7	1
12657	202	0	19-Dec-06	8	2	15	2	1	1	0	3	2	2	1
12658	202	0	6-Mar-17	21	2	15	2	1	1	0	3	2	2	1
12659	202	0	20-Apr-17	11	2	15	2	3	1	0	3	2	3	1
12660	202	0	13-Apr-17	16	2	15	2	1	1	0	3	2	3	1
12661	202	0	5-Jul-17	19	2	15	2	1	1	0	3	2	3	1
12662	202	0	9-Nov-17	0	2	15	2	1	1	0	3	2	3	1
12663	202	0	15-Nov-17	0	2	15	2	1	1	0	3	2	1	1
12664	202	0	17-Oct-17	20	2	15	2	1	1	0	3	2	1	1
12665	202	0	29-Sep-17	16	2	15	2	2	1	0	3	2	1	1
12666	202	0	12-Dec-17	11	2	15	2	1	1	0	3	2	2	1
12667	202	0	11-Dec-17	18	2	15	2	1	1	0	3	2	1	1
12668	202	0	4-Nov-15	11	2	15	2	3	1	0	6	2	2	1
12669	202	0	22-Sep-15	23	2	15	2	2	1	0	6	2	3	1
12670	202	0	22-Jul-14	18	2	15	2	1	1	0	6	2	3	1
12671	202	0	1-Mar-14	15	2	15	2	1	1	0	6	2	2	1
12672	202	0	3-Nov-11	22	2	15	2	1	1	0	6	2	3	2
12673	202	0	11-Nov-10	23	2	15	2	1	1	0	6	2	2	1
12674	202	0	28-Sep-09	23	2	15	2	1	1	0	6	2	2	1
12675	202	0	4-Dec-07	14	2	15	2	1	1	0	6	2	3	2
12676	201	1	31-Jul-15	7	1	15	2	1	1	0	1	2	2	1
12677	101	1	9-Mar-13	18	1	15	2	1	1	0	1	2	4	1
12678	201	1	17-Dec-14	23	1	15	2	1	1	0	3	2	1	1
12679	201	1	13-Nov-16	17	1	15	2	1	1	0	3	2	3	1
12680	201	1	26-May-17	19	1	15	2	1	1	0	6	2	2	1
12681	201	1	31-May-12	17	1	15	2	2	1	0	4	2	2	1
12682	201	1	20-Feb-17	14	1	15	2	2	1	0	6	2	2	1
12683	201	1	14-Jul-16	10	1	15	2	1	1	0	6	2	3	1
12684	201	1	22-Oct-14	8	1	15	2	1	1	0	4	2	2	1
12685	201	1	24-Jul-17	13	1	15	2	1	1	0	4	2	2	1
12686	201	1	5-Nov-16	10	1	15	2	1	1	0	4	2	2	1
12687	201	1	25-Nov-13	16	1	15	2	3	1	0	6	2	2	1
12688	201	1	7-Apr-12	11	1	15	2	3	1	0	4	2	1	1
12689	201	1	6-May-17	12	1	15	2	1	1	0	3	2	4	1
12690	201	1	23-Jan-12	19	1	15	2	2	1	0	5	2	2	1
12691	201	1	24-Nov-14	13	1	15	2	1	1	0	6	2	2	1
12692	201	1	25-Oct-16	17	1	15	2	1	1	0	3	2	3	1
12693	201	1	1-Apr-16	15	1	15	2	1	1	0	4	2	2	1
12694	201	1	6-Mar-17	15	1	15	2	1	1	0	6	2	2	1
12695	201	1	26-Oct-17	19	1	15	2	2	1	0	4	2	2	2
12696	101	1	30-Jun-16	21	1	15	2	1	1	0	1	2	3	1
12697	201	1	14-Sep-17	20	1	15	2	2	1	0	3	2	2	1
12698	201	1	9-Jul-16	15	1	15	2	1	1	0	4	2	5	1
12699	101	1	13-Apr-13	18	1	15	2	1	1	0	6	2	3	1
12700	201	1	3-Nov-17	15	1	15	2	3	1	0	3	2	2	1
12701	201	1	12-Aug-16	11	1	15	2	2	1	0	6	2	2	1
12702	101	1	29-May-17	17	1	15	2	1	1	0	3	2	5	1
12703	101	1	31-Oct-07	10	1	15	2	1	1	0	8	2	4	1
12704	201	1	26-Sep-13	6	1	15	2	1	1	0	7	2	5	1
12705	101	1	5-Dec-12	17	1	15	2	1	1	0	7	2	1	1
12706	101	1	19-Mar-10	17	1	15	2	1	1	0	7	2	1	1
12707	101	1	9-Feb-08	21	1	15	2	2	1	0	7	2	1	1
12708	101	1	1-Jul-11	18	1	15	2	1	1	0	13	2	4	1
12709	101	1	25-Oct-10	14	1	15	2	1	1	0	13	2	3	1
12710	101	1	4-May-09	12	1	15	2	1	1	0	13	2	2	1
12711	101	1	8-Nov-06	16	1	15	2	1	1	0	13	2	4	2
12712	101	1	18-Sep-06	15	1	15	2	1	1	0	13	2	2	1
12713	201	1	12-Sep-11	1	1	15	2	2	1	0	1	2	1	1
12714	101	1	8-Jun-08	17	1	15	2	1	1	0	1	2	4	1
12715	201	1	15-Jan-11	11	1	15	2	2	1	0	1	2	6	1
12716	201	1	21-Aug-10	16	1	15	2	3	1	0	1	2	2	2
12717	101	1	3-Jun-16	11	1	15	2	1	1	0	4	2	4	1
12718	101	1	25-Feb-16	14	1	15	2	1	1	0	4	2	1	1
12719	101	1	13-Oct-16	3	1	15	2	1	1	0	4	2	1	1
12720	101	1	15-Jun-16	21	1	15	2	1	1	0	4	2	2	1
12721	101	1	5-Jan-16	17	1	15	2	1	1	0	4	2	1	1
12722	101	1	21-Apr-16	17	1	15	2	1	1	0	4	2	1	1
12723	201	1	14-Jan-16	17	1	15	2	1	1	0	4	2	1	1
12724	201	1	14-Oct-15	17	1	15	2	1	1	0	4	2	3	1
12725	201	1	26-Sep-15	4	1	15	2	3	1	0	4	2	5	2
12726	101	1	25-Feb-15	19	1	15	2	1	1	0	4	2	1	1
12727	101	1	18-Jan-14	16	1	15	2	1	1	0	4	2	3	1
12728	101	1	30-Apr-14	11	1	15	2	1	1	0	4	2	1	1
12729	101	1	7-Jul-14	17	1	15	2	1	1	0	4	2	1	1
12730	201	1	22-Mar-14	18	1	15	2	1	1	0	4	2	2	1
12731	101	1	25-Apr-14	17	1	15	2	1	1	0	4	2	2	1
12732	101	1	9-May-14	18	1	15	2	1	1	0	4	2	3	1
12733	201	1	26-Dec-14	19	1	15	2	3	1	0	4	2	5	1
12734	201	1	16-Nov-14	22	1	15	2	3	1	0	4	2	5	1
12735	101	1	2-Dec-14	12	1	15	2	1	1	0	4	2	1	1
12736	101	1	16-Dec-14	2	1	15	2	1	1	0	4	2	1	1
12737	201	1	18-Jan-14	20	1	15	2	3	1	0	4	2	3	1
12738	201	1	14-Sep-14	13	1	15	2	3	1	0	4	2	2	1
12739	101	1	7-Mar-13	17	1	15	2	3	1	0	4	2	1	1
12740	101	1	20-May-13	15	1	15	2	1	1	0	4	2	2	1
12741	101	1	10-Mar-13	18	1	15	2	1	1	0	4	2	7	4
12742	101	1	21-Mar-13	23	1	15	2	1	1	0	4	2	1	1
12743	101	1	26-Oct-13	16	1	15	2	1	1	0	4	2	2	1
12744	201	1	19-Oct-13	21	1	15	2	3	1	0	4	2	3	1
12745	101	1	24-Nov-13	16	1	15	2	2	1	0	4	2	4	1
12746	101	1	7-Jun-13	17	1	15	2	1	1	0	4	2	4	1
12747	101	1	18-Mar-12	16	1	15	2	1	1	0	4	2	1	1
12748	101	1	14-Sep-12	17	1	15	2	1	1	0	4	2	1	1
12749	101	1	1-May-12	16	1	15	2	1	1	0	4	2	4	1
12750	101	1	1-Apr-12	16	1	15	2	1	1	0	4	2	7	1
12751	101	1	26-Feb-12	17	1	15	2	1	1	0	4	2	1	1
12752	101	1	30-Jul-12	22	1	15	2	1	1	0	4	2	1	1
12753	201	1	24-Sep-12	17	1	15	2	2	1	0	4	2	1	1
12754	101	1	7-Feb-12	16	1	15	2	1	1	0	4	2	1	2
12755	101	1	24-Jun-12	12	1	15	2	1	1	0	4	2	2	2
12756	101	1	1-Aug-12	18	1	15	2	2	1	0	4	2	1	1
12757	201	1	31-Oct-12	17	1	15	2	3	1	0	4	2	1	1
12758	101	1	10-May-12	11	1	15	2	1	1	0	4	2	2	1
12759	101	1	12-Mar-12	16	1	15	2	1	1	0	4	2	3	1
12760	101	1	14-Jun-12	16	1	15	2	1	1	0	4	2	2	1
12761	201	1	11-Apr-11	12	1	15	2	1	1	0	4	2	1	1
12762	101	1	19-Sep-11	10	1	15	2	1	1	0	4	2	1	2
12763	101	1	13-May-11	21	1	15	2	1	1	0	4	2	1	1
12764	101	1	7-May-11	18	1	15	2	1	1	0	4	2	2	1
12765	101	1	20-Dec-11	11	1	15	2	1	1	0	4	2	4	2

12766	201	1	21-Jun-11	10	1	15	2	1	1	0	4	2	2	1
12767	101	1	10-Sep-10	17	1	15	2	1	1	0	4	2	1	1
12768	101	1	9-Mar-10	12	1	15	2	1	1	0	4	2	2	1
12769	101	1	11-Nov-10	12	1	15	2	1	1	0	4	2	1	1
12770	101	1	4-Jan-10	9	1	15	2	1	1	0	4	2	3	1
12771	101	1	21-Oct-10	20	1	15	2	1	1	0	4	2	2	1
12772	101	1	17-Feb-10	10	1	15	2	1	1	0	4	2	2	1
12773	101	1	12-Nov-10	16	1	15	2	1	1	0	4	2	2	1
12774	101	1	31-Oct-10	16	1	15	2	1	1	0	4	2	2	1
12775	101	1	18-Mar-10	17	1	15	2	1	1	0	4	2	2	4
12776	101	1	2-Dec-10	11	1	15	2	1	1	0	4	2	2	1
12777	201	1	5-Dec-09	18	1	15	2	1	1	0	4	2	3	2
12778	201	1	14-Sep-09	14	1	15	2	1	1	0	4	2	1	1
12779	101	1	10-Sep-09	16	1	15	2	1	1	0	4	2	1	1
12780	201	1	1-Dec-09	9	1	15	2	1	1	0	4	2	4	1
12781	101	1	17-Feb-09	11	1	15	2	1	1	0	4	2	1	1
12782	101	1	24-Dec-09	23	1	15	2	2	1	0	4	2	3	1
12783	101	1	28-Dec-09	18	1	15	2	1	1	0	4	2	2	1
12784	101	1	14-May-09	19	1	15	2	1	1	0	4	2	1	1
12785	201	1	6-Mar-09	17	1	15	2	1	1	0	4	2	2	1
12786	101	1	15-Jun-08	14	1	15	2	1	1	0	4	2	3	1
12787	101	1	29-Sep-08	12	1	15	2	1	1	0	4	2	3	1
12788	101	1	27-Mar-08	19	1	15	2	1	1	0	4	2	7	4
12789	101	1	29-Jul-08	17	1	15	2	1	1	0	4	2	1	1
12790	101	1	14-Mar-08	14	1	15	2	1	1	0	4	2	1	1
12791	101	1	20-Nov-08	16	1	15	2	1	1	0	4	2	4	1
12792	201	1	8-Sep-08	20	1	15	2	3	1	0	4	2	3	1
12793	101	1	30-Dec-07	8	1	15	2	1	1	0	4	2	1	1
12794	101	1	12-Oct-07	10	1	15	2	1	1	0	4	2	3	1
12795	101	1	11-Oct-07	17	1	15	2	1	1	0	4	2	1	1
12796	101	1	11-Dec-07	16	1	15	2	1	1	0	4	2	1	1
12797	101	1	18-Apr-07	17	1	15	2	3	1	0	4	2	4	1
12798	101	1	18-Aug-07	18	1	15	2	1	1	0	4	2	1	1
12799	101	1	1-Jan-07	9	1	15	2	1	1	0	4	2	1	1
12800	101	1	31-Oct-06	19	1	15	2	1	1	0	4	2	1	1
12801	101	1	13-Oct-06	17	1	15	2	1	1	0	4	2	4	1
12802	101	1	13-Oct-06	17	1	15	2	1	1	0	4	2	1	1
12803	201	1	23-Feb-06	19	1	15	2	1	1	0	4	2	4	1
12804	101	1	20-Sep-06	13	1	15	2	1	1	0	4	2	2	1
12805	101	1	13-Apr-06	16	1	15	2	1	1	0	4	2	2	1
12806	201	1	27-Jun-16	15	1	15	2	2	1	0	11	2	5	1
12807	201	1	5-Dec-16	11	1	15	2	2	1	0	11	2	2	1
12808	101	1	30-May-16	19	1	15	2	1	1	0	11	2	3	1
12809	101	1	3-Jun-16	17	1	15	2	1	1	0	11	2	2	1
12810	101	1	16-May-16	20	1	15	2	1	1	0	11	2	3	1
12811	101	1	31-Mar-16	18	1	15	2	1	1	0	11	2	2	1
12812	101	1	12-Nov-16	19	1	15	2	1	1	0	11	2	2	1
12813	101	1	28-Apr-15	17	1	15	2	1	1	0	11	2	2	1
12814	101	1	3-Jan-15	11	1	15	2	1	1	0	11	2	3	1
12815	201	1	9-Feb-15	16	1	15	2	1	1	0	11	2	2	1
12816	101	1	30-Dec-15	11	1	15	2	1	1	0	11	2	2	1
12817	201	1	25-May-14	21	1	15	2	2	1	0	11	2	2	1
12818	201	1	7-Jun-14	21	1	15	2	1	1	0	11	2	2	1
12819	201	1	27-Feb-14	10	1	15	2	3	1	0	11	2	2	1
12820	101	1	11-Oct-14	21	1	15	2	1	1	0	11	2	2	1
12821	101	1	28-Jan-14	14	1	15	2	2	1	0	11	2	2	1
12822	201	1	18-Feb-14	10	1	15	2	3	1	0	11	2	2	1
12823	101	1	27-Sep-14	19	1	15	2	1	1	0	11	2	2	1
12824	201	1	16-Jan-13	10	1	15	2	3	1	0	11	2	2	1
12825	201	1	12-Mar-13	17	1	15	2	2	1	0	11	2	3	1
12826	101	1	25-Jan-13	19	1	15	2	1	1	0	11	2	3	1
12827	101	1	21-Jun-13	23	1	15	2	2	1	0	11	2	3	1
12828	101	1	26-Nov-13	17	1	15	2	2	1	0	11	2	2	1
12829	101	1	30-May-12	17	1	15	2	1	1	0	11	2	1	1
12830	101	1	7-Apr-12	15	1	15	2	1	1	0	11	2	2	1
12831	101	1	21-Mar-12	17	1	15	2	1	1	0	11	2	2	1
12832	201	1	25-Jan-12	20	1	15	2	3	1	0	11	2	2	1
12833	201	1	7-Jul-12	15	1	15	2	3	1	0	11	2	3	1
12834	101	1	1-Feb-12	12	1	15	2	1	1	0	11	2	2	2
12835	201	1	18-Jan-12	23	1	15	2	2	1	0	11	2	3	1
12836	101	1	21-Jun-11	16	1	15	2	1	1	0	11	2	3	1
12837	201	1	22-Feb-11	10	1	15	2	1	1	0	11	2	2	1
12838	101	1	2-Dec-11	7	1	15	2	1	1	0	11	2	2	1
12839	201	1	21-Dec-11	7	1	15	2	1	1	0	11	2	2	1
12840	101	1	30-Apr-10	14	1	15	2	1	1	0	11	2	3	1
12841	201	1	15-Aug-10	8	1	15	2	2	1	0	11	2	2	1
12842	201	1	23-Nov-10	16	1	15	2	2	1	0	11	2	2	1
12843	201	1	1-Jan-10	9	1	15	2	3	1	0	11	2	3	1
12844	101	1	13-Oct-10	21	1	15	2	1	1	0	11	2	2	1
12845	101	1	14-Mar-10	8	1	15	2	1	1	0	11	2	2	1
12846	101	1	28-Feb-09	14	1	15	2	1	1	0	11	2	2	1
12847	201	1	29-Jul-09	7	1	15	2	3	1	0	11	2	3	1
12848	101	1	24-Apr-09	15	1	15	2	1	1	0	11	2	2	1
12849	101	1	24-Apr-09	9	1	15	2	1	1	0	11	2	2	1
12850	201	1	11-May-08	20	1	15	2	1	1	0	11	2	2	1
12851	101	1	27-Feb-17	12	1	15	2	1	1	0	11	2	2	1
12852	101	1	26-Jan-17	23	1	15	2	1	1	0	11	2	2	1
12853	201	1	22-Oct-17	18	1	15	2	1	1	0	11	2	3	1
12854	201	1	12-Nov-17	7	1	15	2	1	1	0	11	2	2	1
12855	201	1	16-Sep-17	18	1	15	2	2	1	0	11	2	2	1
12856	201	1	20-Dec-17	7	1	15	2	1	1	0	11	2	2	1
12857	201	1	20-Dec-17	7	1	15	2	2	1	0	11	2	3	1
12858	201	1	8-Nov-16	13	1	15	2	2	1	0	3	2	1	1
12859	101	1	9-Mar-16	10	1	15	2	1	1	0	3	2	1	1
12860	101	1	19-Oct-16	16	1	15	2	1	1	0	3	2	4	1
12861	201	1	17-May-16	10	1	15	2	2	1	0	3	2	5	1
12862	201	1	18-Mar-16	14	1	15	2	1	1	0	3	2	3	1
12863	201	1	24-Aug-16	9	1	15	2	1	1	0	3	2	1	1
12864	201	1	20-Sep-16	17	1	15	2	1	1	0	3	2	2	1
12865	201	1	12-Jun-16	21	1	15	2	1	1	0	3	2	2	1
12866	101	1	7-Mar-16	21	1	15	2	1	1	0	3	2	2	1
12867	101	1	17-Apr-16	12	1	15	2	1	1	0	3	2	3	1
12868	201	1	20-Apr-16	1	1	15	2	2	1	0	3	2	4	1
12869	101	1	5-Apr-16	21	1	15	2	1	1	0	3	2	1	1
12870	201	1	18-Mar-16	21	1	15	2	2	1	0	3	2	4	1
12871	101	1	31-Mar-16	18	1	15	2	1	1	0	3	2	1	4
12872	201	1	6-Feb-16	17	1	15	2	3	1	0	3	2	1	1
12873	101	1	2-Jan-16	12	1	15	2	1	1	0	3	2	2	1
12874	201	1	10-Jul-16	10	1	15	2	1	1	0	3	2	2	1
12875	101	1	27-Jul-16	10	1	15	2	1	1	0	3	2	3	1
12876	101	1	7-Apr-16	11	1	15	2	2	1	0	3	2	1	1
12877	101	1	16-Apr-16	19	1	15	2	1	1	0	3	2	1	1
12878	101	1	13-Jan-16	12	1	15	2	1	1	0	3	2	2	1
12879	101	1	17-Dec-16	8	1	15	2	1	1	0	3	2	3	1
12880	101	1	12-Apr-16	13	1	15	2	1	1	0	3	2	3	1
12881	101	1	6-Aug-16	17	1	15	2	1	1	0	3	2	2	1
12882	101	1	11-Aug-16	16	1	15	2	1	1	0	3	2	5	1
12883	101	1	28-Dec-16	17	1	15	2	3	1	0	3	2	4	2
12884	101	1	13-May-16	19	1	15	2	1	1	0	3	2	1	1
12885	101	1	3-Jun-16	12	1	15	2	1	1	0	3	2	1	1
12886	201	1	17-Jun-16	14	1	15	2	3	1	0	3	2	1	1
12887	101	1	10-May-16	16	1	15	2	1	1	0	3	2	2	1
12888	201	1	18-Nov-16	11	1	15	2	1	1	0	3	2	4	2
12889	201	1	6-Apr-16	12	1	15	2	1	1	0	3	2	2	2

12890	101	1	8-Oct-16	17	1	15	2	1	1	0	3	2	3	1
12891	201	1	11-Apr-16	16	1	15	2	1	1	0	3	2	2	1
12892	101	1	25-Oct-16	18	1	15	2	1	1	0	3	2	3	1
12893	101	1	24-Jun-16	12	1	15	2	1	1	0	3	2	3	1
12894	101	1	4-Mar-16	18	1	15	2	1	1	0	3	2	2	1
12895	101	1	7-Aug-16	10	1	15	2	1	1	0	3	2	3	1
12896	101	1	12-Nov-16	17	1	15	2	1	1	0	3	2	3	1
12897	101	1	24-Mar-16	9	1	15	2	1	1	0	3	2	4	1
12898	101	1	7-Apr-16	19	1	15	2	1	1	0	3	2	2	1
12899	101	1	14-Aug-16	21	1	15	2	1	1	0	3	2	3	1
12900	101	1	9-Nov-16	13	1	15	2	1	1	0	3	2	3	1
12901	201	1	31-Mar-16	18	1	15	2	1	1	0	3	2	4	1
12902	201	1	29-Dec-16	16	1	15	2	1	1	0	3	2	1	1
12903	201	1	12-Dec-16	10	1	15	2	1	1	0	3	2	4	1
12904	101	1	18-Apr-16	11	1	15	2	1	1	0	3	2	3	4
12905	201	1	12-May-16	17	1	15	2	1	1	0	3	2	3	1
12906	101	1	31-Mar-16	19	1	15	2	1	1	0	3	2	3	1
12907	201	1	18-May-16	17	1	15	2	2	1	0	3	2	2	1
12908	201	1	9-Jun-16	15	1	15	2	2	1	0	3	2	2	1
12909	201	1	18-Jun-16	11	1	15	2	3	1	0	3	2	2	1
12910	201	1	26-Sep-16	16	1	15	2	3	1	0	3	2	2	1
12911	201	1	26-May-16	7	1	15	2	2	1	0	3	2	2	1
12912	101	1	20-Dec-16	8	1	15	2	1	1	0	3	2	3	1
12913	201	1	11-Nov-16	17	1	15	2	2	1	0	3	2	2	1
12914	101	1	12-Mar-15	15	1	15	2	1	1	0	3	2	1	1
12915	201	1	16-Oct-15	9	1	15	2	2	1	0	3	2	1	1
12916	101	1	17-Apr-15	11	1	15	2	1	1	0	3	2	3	1
12917	101	1	16-Jan-15	12	1	15	2	1	1	0	3	2	1	1
12918	201	1	28-Mar-15	13	1	15	2	2	1	0	3	2	5	1
12919	101	1	6-Jan-15	10	1	15	2	1	1	0	3	2	1	1
12920	101	1	7-Apr-15	15	1	15	2	1	1	0	3	2	1	1
12921	201	1	19-Jan-15	9	1	15	2	2	1	0	3	2	3	1
12922	101	1	8-Apr-15	13	1	15	2	1	1	0	3	2	2	1
12923	101	1	27-May-15	10	1	15	2	1	1	0	3	2	3	1
12924	201	1	10-Jun-15	11	1	15	2	2	1	0	3	2	2	1
12925	101	1	27-May-15	10	1	15	2	1	1	0	3	2	2	4
12926	101	1	22-Jan-15	18	1	15	2	1	1	0	3	2	1	1
12927	201	1	21-Oct-15	17	1	15	2	2	1	0	3	2	1	1
12928	101	1	5-Jan-15	18	1	15	2	1	1	0	3	2	1	1
12929	101	1	21-May-15	18	1	15	2	1	1	0	3	2	1	1
12930	101	1	22-Jun-15	17	1	15	2	1	1	0	3	2	4	1
12931	201	1	1-Dec-15	16	1	15	2	2	1	0	3	2	3	1
12932	201	1	19-Dec-15	1	1	15	2	2	1	0	3	2	2	1
12933	101	1	18-Dec-15	23	1	15	2	1	1	0	3	2	3	1
12934	101	1	22-Jul-15	18	1	15	2	1	1	0	3	2	3	1
12935	101	1	4-Jan-15	19	1	15	2	1	1	0	3	2	2	1
12936	101	1	28-Sep-15	16	1	15	2	1	1	0	3	2	2	4
12937	101	1	24-Mar-15	17	1	15	2	1	1	0	3	2	3	1
12938	101	1	1-May-15	9	1	15	2	1	1	0	3	2	3	1
12939	101	1	1-Dec-15	12	1	15	2	1	1	0	3	2	3	4
12940	201	1	7-May-15	19	1	15	2	1	1	0	3	2	5	1
12941	101	1	28-Dec-15	20	1	15	2	1	1	0	3	2	3	1
12942	101	1	21-Nov-15	19	1	15	2	1	1	0	3	2	1	1
12943	101	1	17-Dec-15	20	1	15	2	1	1	0	3	2	1	1
12944	201	1	13-Oct-15	12	1	15	2	1	1	0	3	2	1	1
12945	101	1	7-Jan-15	17	1	15	2	1	1	0	3	2	3	1
12946	101	1	7-Apr-15	18	1	15	2	1	1	0	3	2	3	1
12947	101	1	18-Aug-15	19	1	15	2	1	1	0	3	2	3	1
12948	101	1	19-Oct-15	18	1	15	2	1	1	0	3	2	3	2
12949	201	1	13-Aug-15	16	1	15	2	1	1	0	3	2	2	1
12950	201	1	20-May-15	16	1	15	2	1	1	0	3	2	2	1
12951	101	1	29-Nov-15	17	1	15	2	1	1	0	3	2	2	1
12952	101	1	23-Sep-15	18	1	15	2	1	1	0	3	2	3	1
12953	101	1	25-Mar-15	16	1	15	2	1	1	0	3	2	3	1
12954	101	1	3-Jun-15	17	1	15	2	1	1	0	3	2	3	4
12955	101	1	10-Sep-15	16	1	15	2	1	1	0	3	2	2	1
12956	101	1	11-May-15	17	1	15	2	1	1	0	3	2	3	1
12957	101	1	21-Nov-15	11	1	15	2	1	1	0	3	2	3	1
12958	101	1	25-Nov-15	17	1	15	2	1	1	0	3	2	3	1
12959	101	1	17-Feb-15	17	1	15	2	1	1	0	3	2	3	1
12960	101	1	8-Dec-15	16	1	15	2	1	1	0	3	2	3	1
12961	101	1	20-Nov-15	17	1	15	2	1	1	0	3	2	4	1
12962	101	1	17-Dec-15	18	1	15	2	1	1	0	3	2	2	1
12963	101	1	12-Jun-15	15	1	15	2	1	1	0	3	2	2	1
12964	101	1	4-May-15	18	1	15	2	1	1	0	3	2	2	4
12965	101	1	25-Apr-15	16	1	15	2	1	1	0	3	2	2	2
12966	101	1	3-Apr-15	16	1	15	2	1	1	0	3	2	1	1
12967	201	1	13-May-15	19	1	15	2	2	1	0	3	2	1	1
12968	201	1	1-May-15	9	1	15	2	1	1	0	3	2	1	1
12969	101	1	3-Apr-15	17	1	15	2	1	1	0	3	2	3	1
12970	201	1	27-May-15	17	1	15	2	1	1	0	3	2	3	1
12971	101	1	10-Jan-15	17	1	15	2	1	1	0	3	2	3	1
12972	201	1	20-Nov-15	17	1	15	2	2	1	0	3	2	2	1
12973	201	1	17-Sep-15	14	1	15	2	2	1	0	3	2	3	1
12974	201	1	27-Jan-15	18	1	15	2	2	1	0	3	2	3	1
12975	201	1	24-Nov-15	14	1	15	2	2	1	0	3	2	4	1
12976	201	1	12-Oct-15	14	1	15	2	1	1	0	3	2	2	1
12977	101	1	31-Mar-15	10	1	15	2	1	1	0	3	2	3	1
12978	201	1	6-May-14	10	1	15	2	2	1	0	3	2	1	1
12979	101	1	20-May-14	0	1	15	2	1	1	0	3	2	5	1
12980	101	1	30-Dec-14	10	1	15	2	1	1	0	3	2	1	2
12981	101	1	25-Feb-14	15	1	15	2	1	1	0	3	2	1	2
12982	201	1	22-Dec-14	10	1	15	2	1	1	0	3	2	5	1
12983	201	1	20-Feb-14	19	1	15	2	3	1	0	3	2	3	2
12984	201	1	15-May-14	10	1	15	2	2	1	0	3	2	5	1
12985	201	1	17-Apr-14	18	1	15	2	3	1	0	3	2	1	1
12986	101	1	7-Dec-14	16	1	15	2	1	1	0	3	2	7	1
12987	201	1	15-Dec-14	8	1	15	2	1	1	0	3	2	4	1
12988	201	1	17-Apr-14	18	1	15	2	3	1	0	3	2	3	1
12989	101	1	19-Apr-14	17	1	15	2	1	1	0	3	2	3	1
12990	201	1	6-Oct-14	23	1	15	2	2	1	0	3	2	1	1
12991	101	1	23-Oct-14	14	1	15	2	1	1	0	3	2	5	1
12992	201	1	3-Apr-14	14	1	15	2	1	1	0	3	2	2	1
12993	101	1	17-Dec-14	17	1	15	2	1	1	0	3	2	3	1
12994	101	1	20-May-14	9	1	15	2	1	1	0	3	2	3	1
12995	101	1	6-Nov-14	10	1	15	2	1	1	0	3	2	3	1
12996	201	1	29-Apr-14	11	1	15	2	3	1	0	3	2	3	1
12997	101	1	23-Oct-14	12	1	15	2	1	1	0	3	2	4	1
12998	201	1	17-Apr-14	19	1	15	2	3	1	0	3	2	3	2
12999	201	1	5-Mar-14	18	1	15	2	3	1	0	3	2	2	1
13000	101	1	18-Oct-14	16	1	15	2	1	1	0	3	2	4	1
13001	101	1	14-Apr-14	13	1	15	2	1	1	0	3	2	5	1
13002	101	1	4-May-14	19	1	15	2	1	1	0	3	2	5	1
13003	101	1	29-Sep-14	16	1	15	2	1	1	0	3	2	5	1
13004	101	1	15-May-14	8	1	15	2	1	1	0	3	2	5	1
13005	101	1	9-Jul-14	1	1	15	2	2	1	0	3	2	1	1
13006	101	1	2-Jul-14	19	1	15	2	1	1	0	3	2	1	1
13007	101	1	9-Jan-14	10	1	15	2	1	1	0	3	2	4	1
13008	101	1	30-Apr-14	20	1	15	2	1	1	0	3	2	2	1
13009	101	1	14-Nov-14	20	1	15	2	1	1	0	3	2	3	1
13010	101	1	17-Jun-14	11	1	15	2	1	1	0	3	2	2	1
13011	101	1	2-Apr-14	22	1	15	2	1	1	0	3	2	2	4
13012	101	1	2-Apr-14	11	1	15	2	1	1	0	3	2	2	4
13013	101	1	16-May-14	16	1	15	2	1	1	0	3	2	1	2

13014	101	1	18-Nov-14	12	1	15	2	1	1	0	3	2	3	1
13015	201	1	31-Mar-14	17	1	15	2	3	1	0	3	2	3	2
13016	101	1	28-Oct-14	8	1	15	2	1	1	0	3	2	3	2
13017	101	1	27-Nov-14	17	1	15	2	1	1	0	3	2	2	1
13018	101	1	30-Apr-14	12	1	15	2	1	1	0	3	2	2	1
13019	101	1	26-May-14	17	1	15	2	1	1	0	3	2	2	1
13020	101	1	14-Oct-14	19	1	15	2	1	1	0	3	2	3	1
13021	101	1	29-Oct-14	20	1	15	2	1	1	0	3	2	3	1
13022	101	1	11-Dec-14	16	1	15	2	1	1	0	3	2	1	4
13023	201	1	28-Aug-14	11	1	15	2	1	1	0	3	2	5	1
13024	201	1	23-Apr-14	20	1	15	2	3	1	0	3	2	3	1
13025	101	1	28-Mar-14	14	1	15	2	1	1	0	3	2	3	1
13026	101	1	12-Dec-14	16	1	15	2	1	1	0	3	2	3	1
13027	201	1	4-Nov-14	10	1	15	2	1	1	0	3	2	2	1
13028	101	1	8-Mar-14	10	1	15	2	1	1	0	3	2	3	1
13029	201	1	16-Oct-14	19	1	15	2	1	1	0	3	2	3	1
13030	101	1	10-Apr-14	16	1	15	2	1	1	0	3	2	4	1
13031	101	1	18-Dec-14	18	1	15	2	1	1	0	3	2	2	1
13032	101	1	14-Sep-14	15	1	15	2	1	1	0	3	2	2	1
13033	201	1	23-Feb-14	14	1	15	2	3	1	0	3	2	3	2
13034	101	1	25-Sep-14	18	1	15	2	1	1	0	3	2	3	1
13035	101	1	18-Sep-14	17	1	15	2	1	1	0	3	2	3	1
13036	101	1	17-Jan-14	19	1	15	2	1	1	0	3	2	3	1
13037	101	1	21-Jun-14	21	1	15	2	1	1	0	3	2	3	1
13038	201	1	3-Sep-14	11	1	15	2	1	1	0	3	2	3	2
13039	101	1	4-Nov-14	10	1	15	2	1	1	0	3	2	3	4
13040	201	1	17-Apr-14	15	1	15	2	2	1	0	3	2	3	1
13041	101	1	30-May-14	17	1	15	2	1	1	0	3	2	2	1
13042	101	1	13-Jan-14	22	1	15	2	1	1	0	3	2	2	1
13043	201	1	29-Mar-14	15	1	15	2	2	1	0	3	2	3	1
13044	101	1	19-Mar-14	10	1	15	2	1	1	0	3	2	3	1
13045	201	1	16-Sep-14	20	1	15	2	3	1	0	3	2	4	1
13046	201	1	23-Apr-14	8	1	15	2	1	1	0	3	2	5	1
13047	101	1	1-Dec-14	10	1	15	2	1	1	0	3	2	1	2
13048	101	1	3-Mar-14	17	1	15	2	1	1	0	3	2	3	1
13049	101	1	15-Jun-14	9	1	15	2	1	1	0	3	2	3	1
13050	101	1	21-Feb-14	18	1	15	2	1	1	0	3	2	3	1
13051	201	1	25-Mar-14	16	1	15	2	1	1	0	3	2	2	1
13052	201	1	14-Nov-14	19	1	15	2	1	1	0	3	2	4	2
13053	201	1	29-Apr-14	17	1	15	2	3	1	0	3	2	2	1
13054	201	1	5-May-14	18	1	15	2	2	1	0	3	2	3	1
13055	201	1	17-Jan-14	10	1	15	2	2	1	0	3	2	2	2
13056	101	1	6-May-14	17	1	15	2	1	1	0	3	2	3	1
13057	101	1	11-Dec-14	18	1	15	2	1	1	0	3	2	3	1
13058	101	1	16-Dec-14	15	1	15	2	1	1	0	3	2	2	1
13059	101	1	27-Jan-14	20	1	15	2	1	1	0	3	2	2	1
13060	201	1	12-Sep-14	17	1	15	2	3	1	0	3	2	2	1
13061	201	1	8-Jun-13	11	1	15	2	2	1	0	3	2	1	1
13062	201	1	12-Oct-13	11	1	15	2	2	1	0	3	2	3	1
13063	101	1	28-Dec-13	12	1	15	2	1	1	0	3	2	3	1
13064	101	1	23-May-13	10	1	15	2	1	1	0	3	2	2	1
13065	101	1	1-Feb-13	18	1	15	2	1	1	0	3	2	2	1
13066	101	1	18-Nov-13	21	1	15	2	1	1	0	3	2	3	1
13067	101	1	29-Nov-13	10	1	15	2	1	1	0	3	2	2	1
13068	101	1	28-Sep-13	18	1	15	2	1	1	0	3	2	2	4
13069	201	1	20-Oct-13	14	1	15	2	1	1	0	3	2	3	1
13070	101	1	16-Nov-13	21	1	15	2	1	1	0	3	2	4	1
13071	101	1	8-Sep-13	16	1	15	2	1	1	0	3	2	1	1
13072	101	1	9-Oct-13	21	1	15	2	1	1	0	3	2	1	1
13073	101	1	9-Dec-13	14	1	15	2	1	1	0	3	2	1	1
13074	101	1	31-Dec-13	16	1	15	2	1	1	0	3	2	1	2
13075	101	1	30-Sep-13	22	1	15	2	1	1	0	3	2	1	1
13076	101	1	19-Dec-13	20	1	15	2	1	1	0	3	2	2	1
13077	201	1	6-Dec-13	13	1	15	2	2	1	0	3	2	1	1
13078	101	1	19-Mar-13	9	1	15	2	1	1	0	3	2	1	1
13079	101	1	6-May-13	20	1	15	2	1	1	0	3	2	1	1
13080	101	1	28-Aug-13	23	1	15	2	1	1	0	3	2	1	1
13081	201	1	28-Jun-13	10	1	15	2	2	1	0	3	2	1	1
13082	101	1	20-Sep-13	22	1	15	2	1	1	0	3	2	1	2
13083	201	1	1-Mar-13	19	1	15	2	1	1	0	3	2	4	1
13084	101	1	1-Nov-13	23	1	15	2	1	1	0	3	2	2	1
13085	101	1	12-Jul-13	10	1	15	2	1	1	0	3	2	2	1
13086	101	1	28-Aug-13	20	1	15	2	1	1	0	3	2	3	1
13087	201	1	18-Sep-13	21	1	15	2	3	1	0	3	2	3	1
13088	101	1	12-Dec-13	18	1	15	2	1	1	0	3	2	3	1
13089	101	1	15-Oct-13	2	1	15	2	2	1	0	3	2	1	1
13090	101	1	15-Sep-13	21	1	15	2	1	1	0	3	2	3	1
13091	101	1	10-Jan-13	17	1	15	2	1	1	0	3	2	2	1
13092	101	1	21-May-13	12	1	15	2	1	1	0	3	2	3	1
13093	101	1	26-Mar-13	12	1	15	2	1	1	0	3	2	3	1
13094	101	1	9-Jan-13	11	1	15	2	1	1	0	3	2	3	1
13095	101	1	2-Dec-13	16	1	15	2	1	1	0	3	2	3	1
13096	101	1	27-May-13	11	1	15	2	1	1	0	3	2	2	2
13097	101	1	29-Mar-13	19	1	15	2	1	1	0	3	2	2	1
13098	101	1	21-Apr-13	10	1	15	2	1	1	0	3	2	2	4
13099	201	1	24-Sep-13	19	1	15	2	2	1	0	3	2	3	1
13100	101	1	24-Feb-13	20	1	15	2	1	1	0	3	2	2	1
13101	101	1	31-Dec-13	12	1	15	2	1	1	0	3	2	2	1
13102	101	1	5-Nov-13	16	1	15	2	1	1	0	3	2	2	1
13103	101	1	31-May-13	10	1	15	2	1	1	0	3	2	2	1
13104	101	1	19-Oct-13	11	1	15	2	1	1	0	3	2	2	1
13105	101	1	12-Mar-13	19	1	15	2	1	1	0	3	2	3	1
13106	101	1	9-Oct-13	20	1	15	2	1	1	0	3	2	3	1
13107	101	1	19-Oct-13	15	1	15	2	1	1	0	3	2	3	1
13108	101	1	4-Mar-13	10	1	15	2	1	1	0	3	2	2	1
13109	101	1	5-Jan-13	19	1	15	2	1	1	0	3	2	3	1
13110	101	1	27-Apr-13	16	1	15	2	1	1	0	3	2	5	1
13111	101	1	9-Oct-13	19	1	15	2	1	1	0	3	2	1	1
13112	201	1	24-Nov-13	9	1	15	2	3	1	0	3	2	5	1
13113	201	1	15-Mar-13	16	1	15	2	3	1	0	3	2	1	1
13114	201	1	5-Sep-13	11	1	15	2	1	1	0	3	2	5	1
13115	101	1	6-Apr-13	16	1	15	2	1	1	0	3	2	3	1
13116	101	1	15-Mar-13	17	1	15	2	1	1	0	3	2	3	1
13117	101	1	7-Mar-13	18	1	15	2	1	1	0	3	2	3	1
13118	101	1	14-Nov-13	16	1	15	2	1	1	0	3	2	2	1
13119	101	1	14-Sep-13	18	1	15	2	2	1	0	3	2	3	1
13120	101	1	7-Jun-13	16	1	15	2	1	1	0	3	2	2	1
13121	201	1	1-Feb-13	18	1	15	2	3	1	0	3	2	3	1
13122	101	1	6-May-13	17	1	15	2	1	1	0	3	2	3	1
13123	101	1	7-Sep-13	16	1	15	2	1	1	0	3	2	3	1
13124	101	1	29-Apr-13	10	1	15	2	1	1	0	3	2	2	1
13125	201	1	5-Jul-13	17	1	15	2	3	1	0	3	2	2	1
13126	201	1	5-Oct-13	12	1	15	2	3	1	0	3	2	2	1
13127	201	1	20-May-13	8	1	15	2	2	1	0	3	2	2	1
13128	101	1	21-Oct-13	16	1	15	2	2	1	0	3	2	3	1
13129	101	1	18-Mar-13	17	1	15	2	1	1	0	3	2	2	1
13130	201	1	6-Mar-13	14	1	15	2	1	1	0	3	2	3	1
13131	201	1	23-Apr-13	10	1	15	2	1	1	0	3	2	2	1
13132	101	1	18-Nov-13	11	1	15	2	1	1	0	3	2	2	1
13133	101	1	4-Jan-13	17	1	15	2	1	1	0	3	2	3	4
13134	101	1	1-Dec-13	17	1	15	2	1	1	0	3	2	3	1
13135	101	1	27-Nov-13	16	1	15	2	1	1	0	3	2	3	1
13136	201	1	28-Apr-13	15	1	15	2	1	1	0	3	2	3	1
13137	201	1	25-Nov-13	21	1	15	2	1	1	0	3	2	7	2

13138	201	1	25-Oct-12	11	1	15	2	4	1	0	3	2	1	1
13139	201	1	24-May-12	17	1	15	2	2	1	0	3	2	1	1
13140	201	1	25-May-12	15	1	15	2	3	1	0	3	2	1	1
13141	201	1	11-May-12	14	1	15	2	1	1	0	3	2	1	1
13142	101	1	29-May-12	15	1	15	2	1	1	0	3	2	1	1
13143	101	1	22-Feb-12	14	1	15	2	1	1	0	3	2	1	1
13144	201	1	25-Sep-12	15	1	15	2	1	1	0	3	2	1	1
13145	201	1	6-Jun-12	19	1	15	2	2	1	0	3	2	2	1
13146	201	1	21-Dec-12	15	1	15	2	3	1	0	3	2	2	1
13147	101	1	19-Jun-12	17	1	15	2	1	1	0	3	2	2	2
13148	101	1	12-Sep-12	18	1	15	2	1	1	0	3	2	2	1
13149	101	1	20-Mar-12	10	1	15	2	1	1	0	3	2	2	1
13150	101	1	9-May-12	13	1	15	2	1	1	0	3	2	2	1
13151	101	1	26-Dec-12	15	1	15	2	1	1	0	3	2	1	1
13152	101	1	14-Sep-12	17	1	15	2	1	1	0	3	2	2	1
13153	101	1	27-Apr-12	12	1	15	2	1	1	0	3	2	3	1
13154	201	1	8-Jun-12	12	1	15	2	2	1	0	3	2	3	1
13155	201	1	16-Aug-12	10	1	15	2	3	1	0	3	2	3	2
13156	101	1	3-Jul-12	17	1	15	2	2	1	0	3	2	1	1
13157	101	1	20-Mar-12	20	1	15	2	1	1	0	3	2	2	1
13158	101	1	7-May-12	16	1	15	2	1	1	0	3	2	5	1
13159	101	1	6-Sep-12	15	1	15	2	1	1	0	3	2	1	1
13160	101	1	14-Apr-12	20	1	15	2	1	1	0	3	2	1	1
13161	101	1	1-Nov-12	22	1	15	2	1	1	0	3	2	1	1
13162	101	1	11-Sep-12	20	1	15	2	1	1	0	3	2	4	1
13163	101	1	1-Nov-12	10	1	15	2	1	1	0	3	2	4	1
13164	101	1	1-Sep-12	9	1	15	2	1	1	0	3	2	2	1
13165	101	1	6-May-12	19	1	15	2	2	1	0	3	2	1	1
13166	101	1	21-Sep-12	8	1	15	2	1	1	0	3	2	2	1
13167	101	1	25-May-12	17	1	15	2	1	1	0	3	2	3	1
13168	101	1	13-Oct-12	21	1	15	2	1	1	0	3	2	3	1
13169	101	1	14-Apr-12	11	1	15	2	1	1	0	3	2	2	1
13170	101	1	13-Dec-12	11	1	15	2	1	1	0	3	2	2	1
13171	101	1	14-May-12	18	1	15	2	1	1	0	3	2	1	1
13172	101	1	13-Oct-12	21	1	15	2	1	1	0	3	2	3	1
13173	101	1	14-Nov-12	11	1	15	2	1	1	0	3	2	4	1
13174	101	1	6-Jun-12	10	1	15	2	1	1	0	3	2	1	1
13175	101	1	8-Oct-12	15	1	15	2	1	1	0	3	2	1	1
13176	201	1	19-Aug-12	21	1	15	2	2	1	0	3	2	2	1
13177	101	1	18-Aug-12	17	1	15	2	1	1	0	3	2	3	1
13178	201	1	7-Jun-12	15	1	15	2	1	1	0	3	2	3	1
13179	101	1	4-Nov-12	17	1	15	2	1	1	0	3	2	3	1
13180	101	1	14-Dec-12	17	1	15	2	1	1	0	3	2	3	1
13181	101	1	16-May-12	11	1	15	2	1	1	0	3	2	4	1
13182	101	1	26-Sep-12	18	1	15	2	1	1	0	3	2	3	1
13183	101	1	11-Dec-12	16	1	15	2	1	1	0	3	2	2	1
13184	201	1	19-Aug-12	18	1	15	2	2	1	0	3	2	3	1
13185	101	1	7-Apr-12	17	1	15	2	1	1	0	3	2	2	1
13186	101	1	26-Sep-12	16	1	15	2	1	1	0	3	2	3	1
13187	201	1	14-Jun-12	11	1	15	2	2	1	0	3	2	3	1
13188	201	1	18-Jun-12	10	1	15	2	1	1	0	3	2	1	1
13189	201	1	13-Jun-12	9	1	15	2	2	1	0	3	2	1	1
13190	201	1	24-Mar-12	11	1	15	2	1	1	0	3	2	1	1
13191	201	1	18-Oct-12	11	1	15	2	1	1	0	3	2	1	1
13192	201	1	20-Nov-12	9	1	15	2	1	1	0	3	2	3	1
13193	201	1	30-Nov-12	9	1	15	2	3	1	0	3	2	2	1
13194	201	1	21-Apr-12	19	1	15	2	1	1	0	3	2	2	1
13195	101	1	8-May-12	17	1	15	2	1	1	0	3	2	2	1
13196	101	1	28-Apr-12	17	1	15	2	1	1	0	3	2	3	1
13197	201	1	22-Oct-12	16	1	15	2	2	1	0	3	2	3	2
13198	201	1	3-May-12	15	1	15	2	2	1	0	3	2	2	1
13199	101	1	16-Oct-12	17	1	15	2	1	1	0	3	2	2	1
13200	101	1	13-Jun-12	16	1	15	2	1	1	0	3	2	2	4
13201	201	1	11-Mar-11	10	1	15	2	3	1	0	3	2	3	1
13202	101	1	28-May-11	17	1	15	2	2	1	0	3	2	1	1
13203	201	1	17-Mar-11	13	1	15	2	1	1	0	3	2	1	1
13204	101	1	21-Sep-11	10	1	15	2	1	1	0	3	2	1	1
13205	101	1	16-Dec-11	22	1	15	2	1	1	0	3	2	3	1
13206	201	1	18-Feb-11	21	1	15	2	1	1	0	3	2	3	2
13207	201	1	28-Mar-11	21	1	15	2	1	1	0	3	2	2	1
13208	101	1	27-Nov-11	11	1	15	2	1	1	0	3	2	3	1
13209	101	1	5-Nov-11	19	1	15	2	1	1	0	3	2	5	1
13210	101	1	25-Oct-11	18	1	15	2	1	1	0	3	2	1	1
13211	101	1	30-Apr-11	10	1	15	2	1	1	0	3	2	1	1
13212	101	1	27-Oct-11	17	1	15	2	2	1	0	3	2	1	1
13213	101	1	29-Jan-11	18	1	15	2	1	1	0	3	2	1	1
13214	201	1	24-Apr-11	14	1	15	2	2	1	0	3	2	1	1
13215	201	1	14-Jul-11	13	1	15	2	3	1	0	3	2	2	2
13216	101	1	28-Jul-11	0	1	15	2	1	1	0	3	2	2	1
13217	101	1	3-Dec-11	23	1	15	2	1	1	0	3	2	2	1
13218	101	1	28-Aug-11	17	1	15	2	1	1	0	3	2	2	4
13219	101	1	31-Dec-11	10	1	15	2	1	1	0	3	2	2	1
13220	101	1	15-Mar-11	8	1	15	2	1	1	0	3	2	2	1
13221	101	1	19-Sep-11	18	1	15	2	1	1	0	3	2	3	1
13222	101	1	6-Jan-11	17	1	15	2	1	1	0	3	2	3	1
13223	101	1	4-Mar-11	17	1	15	2	1	1	0	3	2	3	1
13224	101	1	30-May-11	15	1	15	2	1	1	0	3	2	4	1
13225	101	1	18-Apr-11	12	1	15	2	1	1	0	3	2	3	2
13226	101	1	1-May-11	15	1	15	2	2	1	0	3	2	3	1
13227	101	1	31-Oct-11	10	1	15	2	1	1	0	3	2	3	1
13228	101	1	30-Mar-11	20	1	15	2	1	1	0	3	2	3	1
13229	101	1	21-Dec-11	16	1	15	2	1	1	0	3	2	3	1
13230	101	1	14-Jun-11	18	1	15	2	1	1	0	3	2	3	1
13231	101	1	20-May-11	20	1	15	2	1	1	0	3	2	2	1
13232	101	1	24-Jul-11	10	1	15	2	1	1	0	3	2	3	1
13233	101	1	5-Dec-11	17	1	15	2	1	1	0	3	2	2	1
13234	101	1	15-Sep-11	16	1	15	2	1	1	0	3	2	3	1
13235	101	1	24-Nov-11	17	1	15	2	1	1	0	3	2	3	1
13236	101	1	4-Jan-11	17	1	15	2	1	1	0	3	2	3	1
13237	101	1	27-Oct-11	11	1	15	2	1	1	0	3	2	2	4
13238	101	1	22-Nov-11	20	1	15	2	1	1	0	3	2	2	1
13239	101	1	7-May-11	9	1	15	2	2	1	0	3	2	2	1
13240	101	1	17-May-11	17	1	15	2	1	1	0	3	2	2	1
13241	101	1	9-Jun-11	16	1	15	2	1	1	0	3	2	2	1
13242	101	1	28-Feb-11	15	1	15	2	1	1	0	3	2	3	1
13243	201	1	26-Jun-11	8	1	15	2	1	1	0	3	2	1	1
13244	201	1	6-Sep-11	9	1	15	2	1	1	0	3	2	5	1
13245	201	1	20-Mar-11	15	1	15	2	1	1	0	3	2	1	1
13246	201	1	9-Dec-11	14	1	15	2	1	1	0	3	2	1	1
13247	101	1	25-Oct-11	10	1	15	2	1	1	0	3	2	1	1
13248	201	1	30-May-11	19	1	15	2	1	1	0	3	2	1	1
13249	101	1	30-Oct-11	18	1	15	2	1	1	0	3	2	4	1
13250	101	1	25-Nov-11	10	1	15	2	1	1	0	3	2	2	1
13251	201	1	14-Jun-11	20	1	15	2	1	1	0	3	2	1	1
13252	201	1	8-Sep-11	18	1	15	2	2	1	0	3	2	2	1
13253	101	1	16-Apr-11	19	1	15	2	1	1	0	3	2	3	1
13254	101	1	2-Jun-11	12	1	15	2	1	1	0	3	2	2	1
13255	101	1	19-Oct-11	17	1	15	2	1	1	0	3	2	2	1
13256	201	1	18-Feb-11	21	1	15	2	2	1	0	3	2	1	1
13257	201	1	30-Dec-10	17	1	15	2	2	1	0	3	2	6	1
13258	101	1	12-Nov-10	13	1	15	2	1	1	0	3	2	4	1
13259	201	1	14-Jul-10	10	1	15	2	3	1	0	3	2	6	2
13260	101	1	9-Jun-10	10	1	15	2	1	1	0	3	2	1	2
13261	201	1	11-Aug-10	15	1	15	2	3	1	0	3	2	2	1

13262	201	1	27-Aug-10	13	1	15	2	2	1	0	3	2	1	1
13263	101	1	7-Jan-10	19	1	15	2	1	1	0	3	2	1	1
13264	101	1	7-May-10	17	1	15	2	1	1	0	3	2	3	1
13265	201	1	30-Dec-10	15	1	15	2	2	1	0	3	2	3	1
13266	101	1	21-Dec-10	17	1	15	2	1	1	0	3	2	3	1
13267	201	1	29-Nov-10	3	1	15	2	3	1	0	3	2	2	1
13268	101	1	29-Sep-10	10	1	15	2	1	1	0	3	2	3	1
13269	201	1	5-Jun-10	14	1	15	2	1	1	0	3	2	4	1
13270	101	1	8-Feb-10	12	1	15	2	1	1	0	3	2	1	1
13271	101	1	24-Sep-10	22	1	15	2	1	1	0	3	2	6	1
13272	101	1	19-Mar-10	10	1	15	2	1	1	0	3	2	2	1
13273	201	1	25-Oct-10	18	1	15	2	1	1	0	3	2	1	1
13274	101	1	14-Dec-10	1	1	15	2	1	1	0	3	2	1	1
13275	101	1	17-Oct-10	21	1	15	2	1	1	0	3	2	3	1
13276	101	1	9-Apr-10	16	1	15	2	1	1	0	3	2	3	4
13277	101	1	9-Nov-10	12	1	15	2	1	1	0	3	2	3	1
13278	101	1	29-Sep-10	10	1	15	2	1	1	0	3	2	1	1
13279	101	1	1-Aug-10	18	1	15	2	1	1	0	3	2	2	1
13280	101	1	30-Oct-10	17	1	15	2	1	1	0	3	2	2	4
13281	101	1	24-Mar-10	10	1	15	2	1	1	0	3	2	2	1
13282	201	1	1-Nov-10	21	1	15	2	2	1	0	3	2	3	1
13283	101	1	26-May-10	17	1	15	2	1	1	0	3	2	3	1
13284	101	1	23-Jan-10	21	1	15	2	1	1	0	3	2	3	1
13285	101	1	3-Dec-10	18	1	15	2	1	1	0	3	2	2	1
13286	101	1	18-Oct-10	21	1	15	2	1	1	0	3	2	4	1
13287	101	1	24-Nov-10	17	1	15	2	1	1	0	3	2	3	1
13288	101	1	3-May-10	10	1	15	2	1	1	0	3	2	4	1
13289	101	1	14-Oct-10	17	1	15	2	1	1	0	3	2	3	1
13290	101	1	23-Apr-10	21	1	15	2	1	1	0	3	2	3	1
13291	101	1	14-Mar-10	16	1	15	2	1	1	0	3	2	2	1
13292	101	1	6-Jul-10	19	1	15	2	2	1	0	3	2	3	4
13293	101	1	7-Dec-10	10	1	15	2	1	1	0	3	2	2	1
13294	201	1	27-Mar-10	14	1	15	2	2	1	0	3	2	2	1
13295	101	1	31-Jul-10	10	1	15	2	1	1	0	3	2	3	1
13296	101	1	14-Jun-10	17	1	15	2	1	1	0	3	2	3	4
13297	101	1	27-Jan-10	17	1	15	2	1	1	0	3	2	2	1
13298	101	1	14-Jan-10	16	1	15	2	1	1	0	3	2	2	1
13299	101	1	10-Jul-10	11	1	15	2	1	1	0	3	2	2	4
13300	101	1	1-May-10	18	1	15	2	1	1	0	3	2	2	4
13301	201	1	15-Oct-10	17	1	15	2	1	1	0	3	2	3	1
13302	101	1	16-Apr-10	15	1	15	2	3	1	0	3	2	2	4
13303	101	1	11-Nov-10	17	1	15	2	1	1	0	3	2	3	1
13304	201	1	17-Oct-10	6	1	15	2	3	1	0	3	2	2	1
13305	201	1	12-Dec-10	22	1	15	2	2	1	0	3	2	5	1
13306	201	1	12-Dec-10	22	1	15	2	1	1	0	3	2	5	1
13307	201	1	15-Aug-10	15	1	15	2	1	1	0	3	2	3	1
13308	101	1	6-Apr-10	18	1	15	2	1	1	0	3	2	2	1
13309	101	1	12-Mar-09	11	1	15	2	1	1	0	3	2	2	1
13310	101	1	17-Nov-09	8	1	15	2	1	1	0	3	2	3	1
13311	101	1	18-Sep-09	11	1	15	2	1	1	0	3	2	5	1
13312	101	1	4-Mar-09	14	1	15	2	1	1	0	3	2	1	1
13313	101	1	2-Jul-09	17	1	15	2	1	1	0	3	2	3	4
13314	101	1	20-Feb-09	16	1	15	2	1	1	0	3	2	3	1
13315	201	1	10-Jun-09	12	1	15	2	1	1	0	3	2	2	1
13316	101	1	12-Nov-09	17	1	15	2	3	1	0	3	2	3	1
13317	101	1	26-May-09	12	1	15	2	1	1	0	3	2	2	1
13318	101	1	29-Sep-09	14	1	15	2	1	1	0	3	2	2	1
13319	101	1	23-Mar-09	13	1	15	2	2	1	0	3	2	1	1
13320	101	1	10-Sep-09	16	1	15	2	1	1	0	3	2	2	1
13321	201	1	17-Jan-09	17	1	15	2	2	1	0	3	2	2	1
13322	101	1	9-Dec-09	12	1	15	2	3	1	0	3	2	2	4
13323	101	1	9-Jun-09	15	1	15	2	1	1	0	3	2	3	1
13324	101	1	1-Apr-09	12	1	15	2	1	1	0	3	2	3	1
13325	101	1	16-Jul-09	18	1	15	2	1	1	0	3	2	2	1
13326	101	1	9-Jul-09	23	1	15	2	1	1	0	3	2	1	1
13327	101	1	14-Apr-09	13	1	15	2	1	1	0	3	2	1	1
13328	101	1	19-Aug-09	8	1	15	2	1	1	0	3	2	2	4
13329	101	1	6-Nov-09	9	1	15	2	1	1	0	3	2	2	2
13330	101	1	20-Nov-09	10	1	15	2	1	1	0	3	2	3	1
13331	201	1	22-Apr-09	15	1	15	2	1	1	0	3	2	3	1
13332	201	1	28-Oct-09	18	1	15	2	1	1	0	3	2	2	1
13333	101	1	12-Jun-09	23	1	15	2	1	1	0	3	2	1	1
13334	101	1	6-Nov-09	18	1	15	2	1	1	0	3	2	2	1
13335	101	1	29-Jun-09	17	1	15	2	1	1	0	3	2	3	2
13336	201	1	25-Feb-09	18	1	15	2	1	1	0	3	2	2	1
13337	101	1	20-Apr-09	10	1	15	2	1	1	0	3	2	2	1
13338	201	1	21-Apr-09	17	1	15	2	1	1	0	3	2	2	1
13339	101	1	24-Jun-09	18	1	15	2	1	1	0	3	2	3	2
13340	101	1	17-Dec-09	14	1	15	2	1	1	0	3	2	3	1
13341	101	1	27-May-09	17	1	15	2	1	1	0	3	2	3	1
13342	101	1	1-May-09	17	1	15	2	2	1	0	3	2	2	4
13343	201	1	6-Mar-09	18	1	15	2	1	1	0	3	2	2	1
13344	101	1	19-Nov-09	19	1	15	2	1	1	0	3	2	3	1
13345	101	1	27-Sep-09	17	1	15	2	1	1	0	3	2	2	1
13346	101	1	30-Apr-09	19	1	15	2	1	1	0	3	2	2	1
13347	101	1	27-Nov-09	10	1	15	2	1	1	0	3	2	4	4
13348	201	1	27-Jun-09	17	1	15	2	3	1	0	3	2	3	1
13349	201	1	30-Mar-09	11	1	15	2	1	1	0	3	2	2	1
13350	101	1	6-Nov-09	17	1	15	2	1	1	0	3	2	2	1
13351	101	1	12-May-09	19	1	15	2	1	1	0	3	2	3	1
13352	101	1	12-Jun-09	16	1	15	2	1	1	0	3	2	3	1
13353	101	1	4-Apr-09	18	1	15	2	1	1	0	3	2	2	4
13354	101	1	7-Apr-09	10	1	15	2	1	1	0	3	2	3	1
13355	201	1	10-Dec-09	22	1	15	2	2	1	0	3	2	1	1
13356	101	1	1-Nov-09	15	1	15	2	1	1	0	3	2	3	1
13357	201	1	13-Jun-08	16	1	15	2	1	1	0	3	2	1	1
13358	201	1	16-Oct-08	11	1	15	2	2	1	0	3	2	1	2
13359	101	1	26-Feb-08	10	1	15	2	2	1	0	3	2	1	1
13360	101	1	6-Mar-08	17	1	15	2	1	1	0	3	2	1	1
13361	101	1	28-Sep-08	15	1	15	2	2	1	0	3	2	1	1
13362	201	1	30-Apr-08	19	1	15	2	1	1	0	3	2	3	1
13363	101	1	19-Sep-08	17	1	15	2	1	1	0	3	2	3	1
13364	101	1	20-Mar-08	19	1	15	2	1	1	0	3	2	3	1
13365	201	1	4-Nov-08	11	1	15	2	2	1	0	3	2	2	1
13366	101	1	15-May-08	19	1	15	2	1	1	0	3	2	2	1
13367	201	1	5-Mar-08	16	1	15	2	1	1	0	3	2	2	2
13368	101	1	16-Dec-08	18	1	15	2	1	1	0	3	2	3	1
13369	101	1	5-Dec-08	16	1	15	2	1	1	0	3	2	3	1
13370	101	1	26-Feb-08	14	1	15	2	1	1	0	3	2	2	1
13371	201	1	5-Aug-08	13	1	15	2	1	1	0	3	2	2	1
13372	101	1	16-Apr-08	18	1	15	2	1	1	0	3	2	2	4
13373	101	1	11-Jan-08	20	1	15	2	1	1	0	3	2	3	4
13374	201	1	22-Apr-08	12	1	15	2	1	1	0	3	2	3	1
13375	201	1	20-May-08	13	1	15	2	1	1	0	3	2	1	1
13376	101	1	3-Jun-08	17	1	15	2	1	1	0	3	2	4	1
13377	101	1	23-Jun-08	16	1	15	2	1	1	0	3	2	3	1
13378	101	1	29-Feb-08	20	1	15	2	1	1	0	3	2	2	1
13379	101	1	8-Apr-08	10	1	15	2	1	1	0	3	2	4	1
13380	101	1	27-May-08	12	1	15	2	1	1	0	3	2	3	1
13381	101	1	11-Oct-08	10	1	15	2	1	1	0	3	2	2	1
13382	201	1	29-Oct-08	18	1	15	2	2	1	0	3	2	3	1
13383	101	1	3-Jul-08	21	1	15	2	1	1	0	3	2	2	1
13384	101	1	20-Apr-08	10	1	15	2	1	1	0	3	2	2	4
13385	201	1	19-Sep-08	18	1	15	2	1	1	0	3	2	2	1

13386	101	1	1-Apr-08	13	1	15	2	1	1	0	3	2	2	1
13387	101	1	17-May-08	19	1	15	2	1	1	0	3	2	2	1
13388	101	1	1-Nov-08	8	1	15	2	1	1	0	3	2	3	1
13389	101	1	5-Jan-08	17	1	15	2	1	1	0	3	2	2	1
13390	101	1	18-Mar-08	19	1	15	2	1	1	0	3	2	4	1
13391	101	1	9-Aug-08	10	1	15	2	1	1	0	3	2	3	1
13392	101	1	23-Oct-08	17	1	15	2	1	1	0	3	2	3	1
13393	101	1	15-Aug-08	20	1	15	2	1	1	0	3	2	3	1
13394	201	1	18-Aug-08	21	1	15	2	3	1	0	3	2	3	1
13395	101	1	24-May-08	17	1	15	2	1	1	0	3	2	2	1
13396	101	1	11-Oct-08	16	1	15	2	1	1	0	3	2	3	1
13397	101	1	27-May-08	18	1	15	2	1	1	0	3	2	2	1
13398	101	1	11-Mar-08	18	1	15	2	1	1	0	3	2	3	1
13399	101	1	20-Mar-08	19	1	15	2	1	1	0	3	2	3	1
13400	101	1	11-Nov-08	17	1	15	2	1	1	0	3	2	2	1
13401	201	1	18-Apr-08	10	1	15	2	2	1	0	3	2	2	1
13402	101	1	8-May-08	16	1	15	2	1	1	0	3	2	2	1
13403	201	1	20-Mar-08	18	1	15	2	1	1	0	3	2	3	1
13404	101	1	10-Jan-08	16	1	15	2	1	1	0	3	2	2	1
13405	101	1	13-Nov-08	16	1	15	2	1	1	0	3	2	3	1
13406	101	1	18-Apr-08	18	1	15	2	1	1	0	3	2	2	1
13407	101	1	4-Sep-08	17	1	15	2	1	1	0	3	2	2	1
13408	101	1	4-Nov-08	17	1	15	2	1	1	0	3	2	2	1
13409	201	1	12-May-08	15	1	15	2	3	1	0	3	2	3	1
13410	201	1	27-Sep-07	18	1	15	2	1	1	0	3	2	2	1
13411	101	1	24-Nov-07	11	1	15	2	1	1	0	3	2	1	1
13412	101	1	11-Jul-07	8	1	15	2	1	1	0	3	2	1	1
13413	101	1	15-Nov-07	17	1	15	2	1	1	0	3	2	2	1
13414	201	1	24-Aug-07	19	1	15	2	2	1	0	3	2	3	1
13415	201	1	23-Apr-07	19	1	15	2	1	1	0	3	2	3	1
13416	101	1	27-Nov-07	14	1	15	2	1	1	0	3	2	2	4
13417	101	1	25-Dec-07	10	1	15	2	4	1	0	3	2	3	1
13418	101	1	24-Oct-07	18	1	15	2	1	1	0	3	2	2	1
13419	201	1	29-Mar-07	15	1	15	2	1	1	0	3	2	6	1
13420	101	1	7-Sep-07	18	1	15	2	1	1	0	3	2	1	1
13421	201	1	24-Dec-07	20	1	15	2	2	1	0	3	2	3	1
13422	101	1	28-May-07	17	1	15	2	1	1	0	3	2	3	1
13423	101	1	16-Nov-07	16	1	15	2	1	1	0	3	2	2	1
13424	101	1	11-Feb-07	18	1	15	2	1	1	0	3	2	3	2
13425	101	1	21-Sep-07	16	1	15	2	1	1	0	3	2	3	1
13426	101	1	14-Apr-07	16	1	15	2	1	1	0	3	2	3	1
13427	101	1	13-Jan-07	16	1	15	2	1	1	0	3	2	5	1
13428	201	1	25-Oct-07	17	1	15	2	2	1	0	3	2	4	1
13429	101	1	30-Nov-07	19	1	15	2	1	1	0	3	2	3	1
13430	201	1	29-Sep-07	12	1	15	2	1	1	0	3	2	2	1
13431	201	1	20-Dec-07	16	1	15	2	2	1	0	3	2	2	2
13432	101	1	26-Oct-07	9	1	15	2	1	1	0	3	2	3	1
13433	101	1	26-Mar-07	17	1	15	2	1	1	0	3	2	2	1
13434	101	1	10-Dec-07	18	1	15	2	1	1	0	3	2	3	1
13435	101	1	5-Mar-07	17	1	15	2	1	1	0	3	2	3	1
13436	101	1	15-May-07	18	1	15	2	1	1	0	3	2	3	1
13437	201	1	25-Mar-07	17	1	15	2	1	1	0	3	2	2	2
13438	101	1	10-Sep-07	15	1	15	2	3	1	0	3	2	3	1
13439	101	1	11-May-07	15	1	15	2	1	1	0	3	2	3	1
13440	201	1	30-Jul-07	12	1	15	2	3	1	0	3	2	2	2
13441	101	1	22-Dec-06	19	1	15	2	1	1	0	3	2	7	1
13442	101	1	23-May-06	10	1	15	2	1	1	0	3	2	1	1
13443	101	1	4-Jul-06	10	1	15	2	1	1	0	3	2	1	1
13444	101	1	2-Jun-06	7	1	15	2	1	1	0	3	2	3	1
13445	101	1	28-Jul-06	21	1	15	2	1	1	0	3	2	3	1
13446	201	1	10-May-06	22	1	15	2	1	1	0	3	2	3	1
13447	101	1	31-Oct-06	17	1	15	2	1	1	0	3	2	2	1
13448	101	1	2-Oct-06	20	1	15	2	1	1	0	3	2	1	1
13449	101	1	28-Sep-06	17	1	15	2	1	1	0	3	2	4	2
13450	101	1	26-Dec-06	17	1	15	2	1	1	0	3	2	2	2
13451	101	1	29-Mar-06	14	1	15	2	1	1	0	3	2	2	2
13452	101	1	11-Mar-06	16	1	15	2	2	1	0	3	2	2	1
13453	101	1	11-Dec-06	11	1	15	2	1	1	0	3	2	2	1
13454	101	1	17-Oct-06	18	1	15	2	1	1	0	3	2	2	1
13455	201	1	1-Jan-06	9	1	15	2	1	1	0	3	2	5	1
13456	201	1	27-Jun-06	18	1	15	2	1	1	0	3	2	1	1
13457	201	1	27-Nov-06	9	1	15	2	2	1	0	3	2	1	1
13458	201	1	21-Mar-06	14	1	15	2	3	1	0	3	2	1	1
13459	101	1	7-Jun-06	17	1	15	2	1	1	0	3	2	3	1
13460	101	1	10-Nov-06	17	1	15	2	1	1	0	3	2	3	1
13461	101	1	16-May-06	10	1	15	2	1	1	0	3	2	4	1
13462	201	1	3-Nov-06	12	1	15	2	1	1	0	3	2	2	1
13463	101	1	30-Apr-06	15	1	15	2	1	1	0	3	2	2	1
13464	201	1	19-Oct-06	19	1	15	2	1	1	0	3	2	2	1
13465	201	1	18-Sep-06	19	1	15	2	1	1	0	3	2	3	1
13466	201	1	8-Oct-06	20	1	15	2	1	1	0	3	2	2	1
13467	101	1	24-Oct-06	18	1	15	2	1	1	0	3	2	2	1
13468	101	1	22-Jun-17	17	1	15	2	1	1	0	3	2	4	1
13469	101	1	29-Nov-17	12	1	15	2	1	1	0	3	2	3	1
13470	201	1	7-Apr-17	18	1	15	2	1	1	0	3	2	3	1
13471	101	1	13-Mar-17	9	1	15	2	1	1	0	3	2	2	1
13472	101	1	9-Jul-17	15	1	15	2	1	1	0	3	2	2	1
13473	101	1	16-Aug-17	19	1	15	2	1	1	0	3	2	1	1
13474	101	1	13-Feb-17	17	1	15	2	1	1	0	3	2	1	1
13475	101	1	8-Sep-17	20	1	15	2	1	1	0	3	2	1	1
13476	101	1	17-Feb-17	18	1	15	2	1	1	0	3	2	4	2
13477	101	1	11-Mar-17	10	1	15	2	1	1	0	3	2	3	2
13478	101	1	5-Sep-17	11	1	15	2	1	1	0	3	2	2	2
13479	201	1	28-Sep-17	16	1	15	2	2	1	0	3	2	2	1
13480	101	1	24-Nov-17	19	1	15	2	1	1	0	3	2	5	1
13481	101	1	10-Oct-17	22	1	15	2	1	1	0	3	2	1	1
13482	201	1	11-Oct-17	17	1	15	2	1	1	0	3	2	5	1
13483	101	1	28-Apr-17	13	1	15	2	1	1	0	3	2	4	1
13484	101	1	8-Feb-17	16	1	15	2	1	1	0	3	2	4	1
13485	101	1	19-May-17	17	1	15	2	1	1	0	3	2	3	1
13486	101	1	6-Apr-17	17	1	15	2	1	1	0	3	2	3	1
13487	101	1	15-Mar-17	16	1	15	2	1	1	0	3	2	3	1
13488	201	1	25-Sep-17	17	1	15	2	1	1	0	3	2	3	1
13489	201	1	17-Aug-17	9	1	15	2	1	1	0	3	2	3	1
13490	201	1	2-Oct-17	8	1	15	2	1	1	0	3	2	3	1
13491	101	1	3-May-17	17	1	15	2	1	1	0	3	2	2	1
13492	101	1	19-Feb-17	12	1	15	2	1	1	0	3	2	2	1
13493	101	1	23-Mar-17	16	1	15	2	1	1	0	3	2	2	1
13494	101	1	29-Aug-17	18	1	15	2	1	1	0	3	2	2	1
13495	201	1	12-Mar-17	14	1	15	2	1	1	0	3	2	2	1
13496	201	1	9-Nov-17	17	1	15	2	1	1	0	3	2	2	1
13497	101	1	25-May-17	19	1	15	2	1	1	0	3	2	1	1
13498	201	1	13-Mar-17	8	1	15	2	1	1	0	3	2	1	1
13499	201	1	22-Jan-17	21	1	15	2	1	1	0	3	2	1	1
13500	101	1	20-Jun-17	8	1	15	2	1	1	0	3	2	5	2
13501	101	1	30-May-17	18	1	15	2	1	1	0	3	2	4	2
13502	101	1	10-Mar-17	15	1	15	2	1	1	0	3	2	2	4
13503	201	1	19-Sep-17	19	1	15	2	3	1	0	3	2	2	1
13504	201	1	27-Nov-17	20	1	15	2	3	1	0	3	2	2	1
13505	201	1	24-Aug-17	23	1	15	2	3	1	0	3	2	2	1
13506	201	1	10-Nov-17	19	1	15	2	3	1	0	3	2	1	1
13507	201	1	9-May-17	10	1	15	2	2	1	0	3	2	3	1
13508	101	1	19-Oct-17	15	1	15	2	1	1	0	3	2	3	1
13509	101	1	3-Sep-17	14	1	15	2	1	1	0	3	2	3	1

13510	101	1	14-Dec-17	19	1	15	2	1	1	0	3	2	2	1
13511	101	1	15-Oct-17	21	1	15	2	1	1	0	3	2	4	2
13512	101	1	18-Sep-17	21	1	15	2	1	1	0	3	2	3	2
13513	201	1	20-Dec-17	17	1	15	2	2	1	0	3	2	3	1
13514	101	1	18-Sep-17	9	1	15	2	3	1	0	3	2	5	1
13515	101	1	10-Jun-16	11	1	15	2	1	1	0	6	2	2	1
13516	101	1	6-May-15	15	1	15	2	1	1	0	6	2	2	1
13517	101	1	16-Mar-15	16	1	15	2	1	1	0	6	2	3	1
13518	201	1	7-Mar-15	0	1	15	2	2	1	0	6	2	1	1
13519	101	1	20-Dec-15	17	1	15	2	1	1	0	6	2	2	1
13520	101	1	3-Apr-15	1	1	15	2	1	1	0	6	2	2	1
13521	101	1	6-Mar-14	8	1	15	2	1	1	0	6	2	2	1
13522	101	1	25-Feb-14	20	1	15	2	1	1	0	6	2	1	1
13523	101	1	19-Jul-14	14	1	15	2	1	1	0	6	2	2	4
13524	101	1	26-Feb-13	16	1	15	2	3	1	0	6	2	2	1
13525	201	1	29-Jul-13	19	1	15	2	1	1	0	6	2	1	1
13526	101	1	17-Apr-13	19	1	15	2	1	1	0	6	2	2	2
13527	101	1	3-Dec-12	18	1	15	2	2	1	0	6	2	2	1
13528	101	1	28-Oct-12	16	1	15	2	1	1	0	6	2	2	2
13529	101	1	14-Jun-12	12	1	15	2	1	1	0	6	2	2	1
13530	201	1	23-Aug-11	14	1	15	2	1	1	0	6	2	4	1
13531	101	1	30-Nov-11	1	1	15	2	1	1	0	6	2	3	1
13532	201	1	12-Jul-11	7	1	15	2	1	1	0	6	2	3	1
13533	101	1	18-Oct-11	18	1	15	2	1	1	0	6	2	2	1
13534	101	1	22-May-11	15	1	15	2	1	1	0	6	2	3	1
13535	101	1	18-Aug-11	16	1	15	2	1	1	0	6	2	3	1
13536	101	1	17-Nov-11	21	1	15	2	3	1	0	6	2	2	1
13537	201	1	21-Jun-11	10	1	15	2	1	1	0	6	2	3	1
13538	101	1	1-Jun-10	10	1	15	2	1	1	0	6	2	2	1
13539	101	1	24-Apr-10	10	1	15	2	1	1	0	6	2	2	1
13540	101	1	24-May-10	14	1	15	2	1	1	0	6	2	2	1
13541	201	1	11-Aug-10	10	1	15	2	3	1	0	6	2	2	1
13542	101	1	1-Nov-10	19	1	15	2	1	1	0	6	2	1	1
13543	201	1	28-Apr-10	16	1	15	2	3	1	0	6	2	2	1
13544	101	1	20-Sep-10	9	1	15	2	1	1	0	6	2	2	1
13545	101	1	27-Aug-10	4	1	15	2	1	1	0	6	2	3	1
13546	101	1	29-Aug-10	17	1	15	2	1	1	0	6	2	3	1
13547	101	1	20-Oct-09	8	1	15	2	1	1	0	6	2	2	1
13548	201	1	22-Mar-09	15	1	15	2	1	1	0	6	2	3	1
13549	201	1	16-Dec-09	14	1	15	2	1	1	0	6	2	3	1
13550	101	1	15-Feb-09	17	1	15	2	1	1	0	6	2	3	1
13551	101	1	14-Aug-09	18	1	15	2	1	1	0	6	2	2	1
13552	201	1	5-Jun-08	10	1	15	2	1	1	0	6	2	2	1
13553	101	1	6-Mar-08	16	1	15	2	1	1	0	6	2	4	1
13554	101	1	8-Oct-07	8	1	15	2	1	1	0	6	2	2	1
13555	101	1	3-Apr-07	16	1	15	2	1	1	0	6	2	2	1
13556	101	1	29-May-07	12	1	15	2	1	1	0	6	2	2	1
13557	101	1	19-Jun-07	20	1	15	2	1	1	0	6	2	3	1
13558	101	1	5-Dec-07	16	1	15	2	1	1	0	6	2	2	1
13559	101	1	3-Nov-06	9	1	15	2	1	1	0	6	2	2	1
13560	101	1	7-Jun-06	18	1	15	2	1	1	0	6	2	2	1
13561	101	1	23-Feb-06	17	1	15	2	1	1	0	6	2	2	1
13562	101	1	27-Jul-06	0	1	15	2	1	1	0	6	2	3	1
13563	101	1	19-Jul-06	14	1	15	2	1	1	0	6	2	2	1
13564	101	1	26-Sep-17	11	1	15	2	1	1	0	6	2	2	1
13565	101	1	13-May-15	21	1	15	2	1	1	0	13	2	4	2
13566	101	1	15-Jan-15	16	1	15	2	1	1	0	13	2	2	1
13567	101	1	15-Jan-15	15	1	15	2	1	1	0	13	2	4	1
13568	101	1	28-Mar-12	7	1	15	2	1	1	0	13	2	2	1
13569	201	1	22-Dec-10	18	1	15	2	1	1	0	13	2	3	1
13570	201	1	22-Feb-08	19	1	15	2	1	1	0	13	2	3	1
13571	101	1	24-Oct-06	17	1	15	2	1	1	0	13	2	3	1
13572	201	1	20-Mar-17	9	2	15	2	2	1	0	6	2	3	1
13573	201	1	1-May-13	20	2	15	2	2	1	0	3	2	2	1
13574	201	1	28-Dec-17	3	2	15	2	3	1	0	6	2	3	1
13575	201	1	23-Aug-17	12	2	15	2	2	1	0	6	2	1	1
13576	201	1	29-Sep-14	11	2	15	2	2	1	0	6	2	2	1
13577	201	1	26-Feb-17	18	2	15	2	1	1	0	3	2	3	1
13578	201	1	5-Dec-08	20	2	15	2	1	1	0	10	2	7	2
13579	101	1	7-Sep-15	18	2	15	2	1	1	0	7	2	3	1
13580	101	1	9-Sep-15	20	2	15	2	1	1	0	7	2	3	1
13581	101	1	19-Jan-13	6	2	15	2	1	1	0	7	2	4	1
13582	201	1	28-Jun-12	11	2	15	2	1	1	0	7	2	3	1
13583	201	1	14-Jun-11	20	2	15	2	3	1	0	7	2	2	1
13584	101	1	8-May-11	16	2	15	2	2	1	0	13	2	3	4
13585	101	1	7-Apr-11	14	2	15	2	1	1	0	13	2	4	1
13586	101	1	6-Sep-10	11	2	15	2	1	1	0	13	2	3	1
13587	201	1	12-Apr-10	19	2	15	2	1	1	0	13	2	2	1
13588	101	1	18-Mar-08	10	2	15	2	1	1	0	13	2	3	1
13589	101	1	10-Mar-07	10	2	15	2	1	1	0	13	2	3	1
13590	201	1	20-Sep-10	2	2	15	2	3	1	0	1	2	3	1
13591	101	1	13-Mar-16	10	2	15	2	1	1	0	4	2	2	1
13592	101	1	13-Oct-14	16	2	15	2	1	1	0	4	2	1	1
13593	201	1	16-Jun-14	21	2	15	2	3	1	0	4	2	1	1
13594	201	1	15-Aug-14	18	2	15	2	3	1	0	4	2	1	1
13595	201	1	30-Jan-14	20	2	15	2	3	1	0	4	2	1	1
13596	101	1	30-Oct-14	20	2	15	2	1	1	0	4	2	2	1
13597	101	1	23-Jul-14	11	2	15	2	1	1	0	4	2	4	1
13598	101	1	23-Jun-12	22	2	15	2	1	1	0	4	2	1	1
13599	101	1	3-Jun-12	20	2	15	2	1	1	0	4	2	2	1
13600	101	1	13-Mar-11	18	2	15	2	1	1	0	4	2	2	1
13601	101	1	7-Dec-10	11	2	15	2	1	1	0	4	2	4	1
13602	101	1	7-Sep-09	18	2	15	2	1	1	0	4	2	1	1
13603	101	1	25-Mar-09	18	2	15	2	1	1	0	4	2	1	1
13604	101	1	7-Nov-09	17	2	15	2	1	1	0	4	2	1	1
13605	101	1	18-Mar-08	19	2	15	2	1	1	0	4	2	4	1
13606	101	1	21-Nov-07	22	2	15	2	1	1	0	4	2	2	1
13607	101	1	3-Feb-06	20	2	15	2	1	1	0	4	2	1	1
13608	101	1	12-Jun-17	17	2	15	2	1	1	0	4	2	1	1
13609	101	1	10-Sep-17	13	2	15	2	1	1	0	4	2	3	2
13610	201	1	23-Jul-16	12	2	15	2	1	1	0	11	2	2	1
13611	101	1	17-Sep-16	19	2	15	2	1	1	0	11	2	3	1
13612	101	1	28-Jun-16	18	2	15	2	1	1	0	11	2	2	1
13613	201	1	28-Oct-16	15	2	15	2	2	1	0	11	2	2	1
13614	101	1	2-Sep-15	19	2	15	2	1	1	0	11	2	3	1
13615	101	1	8-Mar-15	12	2	15	2	1	1	0	11	2	2	1
13616	101	1	5-Dec-15	11	2	15	2	1	1	0	11	2	2	1
13617	101	1	29-Sep-15	8	2	15	2	1	1	0	11	2	3	1
13618	101	1	5-Sep-14	20	2	15	2	1	1	0	11	2	3	1
13619	201	1	28-Jul-13	19	2	15	2	1	1	0	11	2	2	1
13620	201	1	1-Aug-13	21	2	15	2	2	1	0	11	2	2	1
13621	101	1	31-Mar-13	22	2	15	2	1	1	0	11	2	2	1
13622	101	1	17-Sep-13	11	2	15	2	1	1	0	11	2	2	1
13623	101	1	27-Aug-13	21	2	15	2	1	1	0	11	2	2	1
13624	101	1	9-Mar-13	11	2	15	2	1	1	0	11	2	2	1
13625	101	1	6-Jan-13	7	2	15	2	1	1	0	11	2	3	1
13626	101	1	31-Aug-12	21	2	15	2	1	1	0	11	2	3	1
13627	101	1	16-Feb-12	17	2	15	2	1	1	0	11	2	2	1
13628	101	1	14-May-12	11	2	15	2	1	1	0	11	2	2	1
13629	101	1	18-Sep-11	19	2	15	2	1	1	0	11	2	2	1
13630	101	1	3-Sep-10	9	2	15	2	1	1	0	11	2	3	1
13631	101	1	26-May-10	13	2	15	2	1	1	0	11	2	1	1
13632	101	1	18-Sep-10	17	2	15	2	1	1	0	11	2	2	1
13633	201	1	2-Jun-06	18	2	15	2	1	1	0	11	2	1	1

13634	201	1	2-Nov-06	12	2	15	2	1	1	0	11	2	3	1
13635	101	1	7-May-17	18	2	15	2	1	1	0	11	2	3	1
13636	101	1	21-Nov-17	17	2	15	2	1	1	0	11	2	4	1
13637	101	1	19-Feb-17	12	2	15	2	1	1	0	11	2	3	1
13638	101	1	15-Jan-16	17	2	15	2	1	1	0	3	2	4	1
13639	101	1	25-Apr-16	6	2	15	2	1	1	0	3	2	1	1
13640	101	1	3-Apr-16	14	2	15	2	1	1	0	3	2	5	1
13641	101	1	5-Dec-16	19	2	15	2	1	1	0	3	2	1	1
13642	201	1	5-Feb-16	13	2	15	2	2	1	0	3	2	1	1
13643	101	1	30-Jun-16	12	2	15	2	1	1	0	3	2	2	1
13644	201	1	13-Dec-16	19	2	15	2	1	1	0	3	2	1	1
13645	101	1	23-Dec-16	15	2	15	2	1	1	0	3	2	3	1
13646	101	1	30-Jan-16	14	2	15	2	1	1	0	3	2	2	1
13647	101	1	17-Dec-16	13	2	15	2	1	1	0	3	2	3	1
13648	101	1	27-Feb-16	20	2	15	2	1	1	0	3	2	3	2
13649	101	1	17-Mar-16	11	2	15	2	1	1	0	3	2	4	1
13650	101	1	8-Jun-16	17	2	15	2	1	1	0	3	2	3	1
13651	201	1	9-Sep-16	15	2	15	2	2	1	0	3	2	2	1
13652	201	1	10-Mar-15	16	2	15	2	1	1	0	3	2	2	1
13653	101	1	3-Dec-15	8	2	15	2	1	1	0	3	2	4	1
13654	101	1	28-Aug-15	19	2	15	2	1	1	0	3	2	3	1
13655	101	1	27-Oct-15	7	2	15	2	1	1	0	3	2	2	1
13656	101	1	8-Dec-15	19	2	15	2	1	1	0	3	2	3	1
13657	101	1	6-May-15	18	2	15	2	1	1	0	3	2	3	1
13658	201	1	7-Jan-15	23	2	15	2	3	1	0	3	2	3	1
13659	101	1	15-Sep-15	18	2	15	2	1	1	0	3	2	4	1
13660	101	1	6-Feb-15	16	2	15	2	1	1	0	3	2	3	1
13661	101	1	25-Nov-15	21	2	15	2	1	1	0	3	2	3	1
13662	201	1	23-Mar-15	13	2	15	2	2	1	0	3	2	1	1
13663	201	1	29-May-14	11	2	15	2	2	1	0	3	2	2	1
13664	201	1	26-Feb-14	14	2	15	2	1	1	0	3	2	3	1
13665	201	1	4-Jan-14	9	2	15	2	1	1	0	3	2	3	1
13666	101	1	10-Mar-14	17	2	15	2	1	1	0	3	2	4	1
13667	201	1	24-Dec-14	17	2	15	2	2	1	0	3	2	4	1
13668	101	1	1-Sep-14	18	2	15	2	1	1	0	3	2	3	1
13669	201	1	5-Mar-14	11	2	15	2	3	1	0	3	2	3	1
13670	101	1	20-May-14	10	2	15	2	1	1	0	3	2	3	1
13671	101	1	18-May-14	12	2	15	2	1	1	0	3	2	2	1
13672	101	1	29-Mar-14	9	2	15	2	1	1	0	3	2	1	1
13673	201	1	17-Aug-14	19	2	15	2	3	1	0	3	2	3	1
13674	101	1	13-Oct-14	18	2	15	2	1	1	0	3	2	3	1
13675	101	1	3-Sep-14	17	2	15	2	1	1	0	3	2	3	1
13676	201	1	16-Jul-14	10	2	15	2	2	1	0	3	2	2	1
13677	101	1	7-Jul-14	18	2	15	2	1	1	0	3	2	1	1
13678	101	1	8-Jan-14	16	2	15	2	1	1	0	3	2	1	1
13679	101	1	8-Sep-14	17	2	15	2	1	1	0	3	2	3	1
13680	101	1	5-Apr-14	1	2	15	2	1	1	0	3	2	3	1
13681	101	1	26-Sep-14	19	2	15	2	1	1	0	3	2	3	1
13682	201	1	27-Jul-14	21	2	15	2	2	1	0	3	2	3	1
13683	101	1	17-Mar-14	19	2	15	2	1	1	0	3	2	3	1
13684	101	1	13-Mar-14	14	2	15	2	1	1	0	3	2	2	1
13685	201	1	19-May-14	11	2	15	2	1	1	0	3	2	3	1
13686	201	1	26-Feb-14	16	2	15	2	1	1	0	3	2	4	1
13687	101	1	6-Mar-14	10	2	15	2	1	1	0	3	2	3	1
13688	201	1	27-Nov-14	17	2	15	2	2	1	0	3	2	3	1
13689	101	1	11-Oct-14	11	2	15	2	1	1	0	3	2	3	1
13690	101	1	25-Feb-14	17	2	15	2	1	1	0	3	2	3	1
13691	101	1	15-Mar-14	20	2	15	2	1	1	0	3	2	1	1
13692	201	1	7-Nov-14	11	2	15	2	1	1	0	3	2	3	1
13693	101	1	26-Sep-13	18	2	15	2	1	1	0	3	2	5	1
13694	101	1	4-Jun-13	23	2	15	2	1	1	0	3	2	1	2
13695	101	1	27-Nov-13	18	2	15	2	1	1	0	3	2	3	4
13696	101	1	22-Dec-13	16	2	15	2	1	1	0	3	2	3	1
13697	101	1	26-Sep-13	22	2	15	2	1	1	0	3	2	4	1
13698	201	1	29-Oct-13	9	2	15	2	2	1	0	3	2	4	1
13699	101	1	14-Sep-13	22	2	15	2	1	1	0	3	2	1	1
13700	101	1	12-Dec-13	20	2	15	2	1	1	0	3	2	2	1
13701	101	1	5-Nov-13	16	2	15	2	1	1	0	3	2	4	1
13702	101	1	18-Oct-13	17	2	15	2	1	1	0	3	2	2	1
13703	201	1	26-Jan-13	15	2	15	2	2	1	0	3	2	2	1
13704	201	1	10-Feb-13	10	2	15	2	3	1	0	3	2	2	1
13705	101	1	26-Sep-13	22	2	15	2	1	1	0	3	2	3	1
13706	101	1	24-Apr-13	7	2	15	2	1	1	0	3	2	3	1
13707	201	1	27-Sep-13	19	2	15	2	2	1	0	3	2	5	1
13708	201	1	10-Aug-13	20	2	15	2	1	1	0	3	2	1	1
13709	101	1	15-Nov-13	18	2	15	2	1	1	0	3	2	3	1
13710	101	1	30-Oct-13	16	2	15	2	1	1	0	3	2	3	1
13711	101	1	11-Mar-13	17	2	15	2	1	1	0	3	2	2	1
13712	201	1	19-Dec-13	12	2	15	2	1	1	0	3	2	2	1
13713	101	1	26-Jun-13	11	2	15	2	1	1	0	3	2	4	1
13714	101	1	22-Nov-13	10	2	15	2	1	1	0	3	2	4	1
13715	201	1	20-Oct-13	16	2	15	2	2	1	0	3	2	3	1
13716	101	1	17-Dec-13	11	2	15	2	1	1	0	3	2	2	1
13717	201	1	29-Oct-13	14	2	15	2	1	1	0	3	2	2	1
13718	201	1	16-Apr-13	21	2	15	2	3	1	0	3	2	2	1
13719	201	1	9-Apr-12	16	2	15	2	1	1	0	3	2	1	1
13720	101	1	21-Dec-12	22	2	15	2	1	1	0	3	2	1	1
13721	101	1	4-Dec-12	17	2	15	2	1	1	0	3	2	3	1
13722	201	1	14-Oct-12	18	2	15	2	3	1	0	3	2	3	2
13723	201	1	5-Jan-12	20	2	15	2	1	1	0	3	2	3	1
13724	101	1	31-Jul-12	22	2	15	2	1	1	0	3	2	1	1
13725	101	1	10-Apr-12	18	2	15	2	1	1	0	3	2	5	1
13726	101	1	5-Dec-12	21	2	15	2	1	1	0	3	2	1	1
13727	101	1	28-Oct-12	7	2	15	2	1	1	0	3	2	3	1
13728	101	1	10-Sep-12	18	2	15	2	1	1	0	3	2	1	1
13729	101	1	12-Dec-12	8	2	15	2	1	1	0	3	2	1	1
13730	101	1	2-Mar-12	15	2	15	2	1	1	0	3	2	4	1
13731	201	1	11-Apr-12	11	2	15	2	1	1	0	3	2	1	1
13732	101	1	23-Apr-12	17	2	15	2	1	1	0	3	2	3	1
13733	101	1	28-Apr-12	17	2	15	2	1	1	0	3	2	3	1
13734	101	1	23-Apr-12	17	2	15	2	1	1	0	3	2	3	1
13735	101	1	1-Dec-11	12	2	15	2	1	1	0	3	2	5	1
13736	101	1	29-Apr-11	16	2	15	2	1	1	0	3	2	1	1
13737	201	1	8-Jun-11	10	2	15	2	1	1	0	3	2	2	1
13738	101	1	6-May-11	11	2	15	2	1	1	0	3	2	1	1
13739	101	1	7-Jan-11	22	2	15	2	1	1	0	3	2	1	1
13740	101	1	6-Mar-11	18	2	15	2	1	1	0	3	2	1	1
13741	201	1	27-Feb-11	14	2	15	2	3	1	0	3	2	3	1
13742	201	1	13-May-11	21	2	15	2	1	1	0	3	2	2	2
13743	101	1	7-Dec-11	19	2	15	2	1	1	0	3	2	2	4
13744	201	1	14-Nov-11	14	2	15	2	1	1	0	3	2	3	1
13745	101	1	22-Feb-11	12	2	15	2	1	1	0	3	2	3	1
13746	101	1	16-Oct-11	12	2	15	2	1	1	0	3	2	3	1
13747	201	1	21-Nov-11	9	2	15	2	3	1	0	3	2	3	1
13748	101	1	22-May-11	10	2	15	2	1	1	0	3	2	4	1
13749	101	1	1-Dec-11	16	2	15	2	1	1	0	3	2	4	2
13750	101	1	4-Sep-11	18	2	15	2	1	1	0	3	2	5	1
13751	201	1	14-Dec-11	9	2	15	2	2	1	0	3	2	1	1
13752	201	1	4-Nov-11	13	2	15	2	1	1	0	3	2	1	1
13753	201	1	2-Nov-11	16	2	15	2	2	1	0	3	2	3	1
13754	101	1	30-Jan-10	7	2	15	2	1	1	0	3	2	3	1
13755	101	1	14-Apr-10	17	2	15	2	1	1	0	3	2	3	1
13756	101	1	31-Oct-10	9	2	15	2	1	1	0	3	2	3	1
13757	101	1	2-Jul-10	10	2	15	2	1	1	0	3	2	2	1

13758	101	1	5-Mar-10	19	2	15	2	1	1	0	3	2	5	1
13759	101	1	6-May-10	8	2	15	2	1	1	0	3	2	5	1
13760	101	1	4-May-10	10	2	15	2	1	1	0	3	2	3	1
13761	201	1	27-Mar-10	14	2	15	2	2	1	0	3	2	3	1
13762	101	1	9-Apr-10	16	2	15	2	1	1	0	3	2	3	1
13763	101	1	3-Dec-10	16	2	15	2	1	1	0	3	2	4	1
13764	101	1	23-Apr-10	22	2	15	2	1	1	0	3	2	3	1
13765	101	1	16-Mar-10	10	2	15	2	1	1	0	3	2	4	4
13766	101	1	14-Dec-10	11	2	15	2	1	1	0	3	2	1	1
13767	101	1	26-Oct-10	17	2	15	2	1	1	0	3	2	1	1
13768	201	1	18-Jul-10	22	2	15	2	2	1	0	3	2	4	2
13769	101	1	17-Mar-10	18	2	15	2	2	1	0	3	2	3	1
13770	101	1	30-Dec-09	17	2	15	2	1	1	0	3	2	4	1
13771	201	1	21-Apr-09	12	2	15	2	1	1	0	3	2	1	1
13772	101	1	7-Apr-09	18	2	15	2	2	1	0	3	2	3	1
13773	201	1	9-Feb-09	20	2	15	2	3	1	0	3	2	5	1
13774	101	1	23-Sep-09	20	2	15	2	1	1	0	3	2	3	1
13775	201	1	16-Aug-09	21	2	15	2	2	1	0	3	2	3	1
13776	101	1	2-Aug-09	7	2	15	2	2	1	0	3	2	3	1
13777	101	1	24-Apr-09	15	2	15	2	1	1	0	3	2	3	1
13778	101	1	23-Feb-09	14	2	15	2	1	1	0	3	2	4	1
13779	101	1	14-Apr-09	10	2	15	2	2	1	0	3	2	3	1
13780	101	1	6-Feb-09	16	2	15	2	1	1	0	3	2	2	1
13781	101	1	28-Apr-09	14	2	15	2	1	1	0	3	2	3	1
13782	101	1	2-Mar-09	13	2	15	2	1	1	0	3	2	3	1
13783	101	1	19-Oct-08	17	2	15	2	1	1	0	3	2	1	1
13784	101	1	17-Nov-08	10	2	15	2	1	1	0	3	2	3	1
13785	101	1	2-Sep-08	18	2	15	2	1	1	0	3	2	3	1
13786	101	1	22-Apr-08	11	2	15	2	1	1	0	3	2	3	1
13787	101	1	12-Dec-08	17	2	15	2	1	1	0	3	2	3	1
13788	101	1	20-Mar-08	16	2	15	2	1	1	0	3	2	3	1
13789	101	1	2-Apr-07	8	2	15	2	1	1	0	3	2	4	1
13790	101	1	22-Jun-07	19	2	15	2	1	1	0	3	2	3	1
13791	101	1	17-Mar-07	18	2	15	2	1	1	0	3	2	2	1
13792	101	1	9-May-07	21	2	15	2	1	1	0	3	2	3	1
13793	101	1	18-Oct-07	19	2	15	2	1	1	0	3	2	3	4
13794	101	1	18-Jul-06	20	2	15	2	1	1	0	3	2	3	1
13795	101	1	16-Nov-06	19	2	15	2	1	1	0	3	2	3	1
13796	101	1	26-Dec-06	17	2	15	2	1	1	0	3	2	3	1
13797	101	1	25-Apr-06	10	2	15	2	1	1	0	3	2	4	1
13798	101	1	10-Apr-06	15	2	15	2	1	1	0	3	2	4	1
13799	101	1	1-Nov-06	18	2	15	2	1	1	0	3	2	2	1
13800	101	1	18-Sep-06	10	2	15	2	1	1	0	3	2	3	1
13801	201	1	1-Aug-06	14	2	15	2	2	1	0	3	2	1	1
13802	101	1	15-Nov-17	20	2	15	2	1	1	0	3	2	7	1
13803	201	1	11-Nov-17	21	2	15	2	2	1	0	3	2	3	1
13804	101	1	9-Jun-17	8	2	15	2	1	1	0	3	2	5	1
13805	101	1	2-Jun-17	21	2	15	2	1	1	0	3	2	3	1
13806	201	1	25-Jan-17	17	2	15	2	1	1	0	3	2	3	1
13807	201	1	17-Mar-17	9	2	15	2	1	1	0	3	2	3	1
13808	201	1	10-Oct-17	20	2	15	2	1	1	0	3	2	3	1
13809	101	1	28-Nov-17	16	2	15	2	1	1	0	3	2	1	1
13810	201	1	21-Oct-17	17	2	15	2	2	1	0	3	2	2	1
13811	201	1	30-Oct-17	20	2	15	2	3	1	0	3	2	3	1
13812	101	1	27-Oct-17	22	2	15	2	1	1	0	3	2	3	1
13813	101	1	1-Dec-17	14	2	15	2	1	1	0	3	2	3	1
13814	101	1	3-May-15	12	2	15	2	1	1	0	6	2	2	1
13815	101	1	29-May-12	11	2	15	2	1	1	0	6	2	2	1
13816	101	1	11-Sep-12	13	2	15	2	1	1	0	6	2	2	1
13817	101	1	2-Sep-11	13	2	15	2	1	1	0	6	2	2	1
13818	101	1	24-Dec-11	17	2	15	2	1	1	0	6	2	2	1
13819	101	1	13-Oct-11	12	2	15	2	1	1	0	6	2	2	1
13820	101	1	18-Nov-11	14	2	15	2	1	1	0	6	2	3	1
13821	101	1	28-Oct-09	13	2	15	2	1	1	0	6	2	2	1
13822	101	1	23-Oct-08	15	2	15	2	1	1	0	6	2	1	1
13823	101	1	1-Nov-08	12	2	15	2	1	1	0	6	2	3	1
13824	101	1	17-Apr-17	16	2	15	2	1	1	0	6	2	3	1
13825	101	1	17-Apr-12	16	2	15	2	1	1	0	13	2	3	1
13826	202	0	20-Oct-15	13	1	16	2	1	1	0	1	2	1	2
13827	202	0	3-Jun-17	3	1	16	2	1	1	0	1	2	6	1
13828	202	0	4-Oct-14	11	1	16	2	1	1	0	6	2	3	1
13829	202	0	10-May-13	1	1	16	2	1	1	0	4	2	3	1
13830	202	0	5-Oct-14	18	1	16	2	1	1	0	6	2	2	1
13831	202	0	6-Sep-16	20	1	16	2	1	1	0	6	2	2	1
13832	202	0	2-Dec-14	21	1	16	2	3	1	0	4	2	1	1
13833	202	0	29-May-13	13	1	16	2	1	1	0	6	2	3	1
13834	202	0	22-Dec-15	16	1	16	2	1	1	0	4	2	1	1
13835	202	0	25-May-17	18	1	16	2	1	1	0	3	2	2	1
13836	202	0	8-Oct-12	17	1	16	2	1	1	0	6	2	1	2
13837	202	0	4-Aug-16	11	1	16	2	3	1	0	4	2	2	2
13838	202	0	17-Feb-17	3	1	16	2	2	1	0	6	2	2	4
13839	202	0	1-Nov-14	20	1	16	2	3	1	0	6	2	3	1
13840	202	0	1-Jun-12	11	1	16	2	1	1	0	6	2	3	1
13841	202	0	13-Oct-17	20	1	16	2	3	1	0	6	2	2	1
13842	202	0	6-May-14	17	1	16	2	1	1	0	6	2	2	1
13843	202	0	20-Nov-14	14	1	16	2	1	1	0	4	2	1	1
13844	202	0	9-Jan-16	3	1	16	2	1	1	0	6	2	2	1
13845	202	0	29-Jun-15	2	1	16	2	1	1	0	6	2	3	1
13846	202	0	23-Sep-14	18	1	16	2	1	1	0	6	2	2	2
13847	202	0	10-May-13	11	1	16	2	3	1	0	6	2	2	1
13848	202	0	20-Jun-17	21	1	16	2	1	1	0	6	2	2	1
13849	202	0	19-Aug-17	7	1	16	2	2	1	0	6	2	7	1
13850	202	0	26-Dec-17	0	1	16	2	2	1	0	4	2	2	1
13851	202	0	24-Sep-15	18	1	16	2	1	1	0	4	2	1	1
13852	202	0	3-Oct-14	22	1	16	2	1	1	0	10	2	7	1
13853	202	0	7-Apr-12	21	1	16	2	2	1	0	10	2	7	2
13854	202	0	27-Nov-09	21	1	16	2	1	1	0	10	2	7	1
13855	202	0	27-Nov-09	21	1	16	2	1	1	0	10	2	7	1
13856	202	0	13-Oct-08	13	1	16	2	1	1	0	10	2	6	2
13857	202	0	30-Jul-16	22	1	16	2	1	1	0	7	2	6	2
13858	202	0	1-Aug-14	15	1	16	2	1	1	0	7	2	2	1
13859	202	0	21-Jan-10	7	1	16	2	1	1	0	7	2	3	1
13860	202	0	24-Mar-10	11	1	16	2	1	1	0	7	2	2	1
13861	202	0	17-Jun-07	23	1	16	2	1	1	0	7	2	4	1
13862	202	0	1-Jun-15	20	1	16	2	2	1	0	13	2	3	1
13863	202	0	18-Dec-14	9	1	16	2	1	1	0	13	2	1	1
13864	202	0	1-Oct-10	3	1	16	2	1	1	0	13	2	1	1
13865	202	0	4-Jan-10	8	1	16	2	1	1	0	13	2	3	1
13866	202	0	10-Mar-08	9	1	16	2	1	1	0	13	2	1	1
13867	202	0	8-Oct-08	18	1	16	2	1	1	0	13	2	1	1
13868	202	0	15-Aug-08	23	1	16	2	2	1	0	13	2	7	2
13869	202	0	15-Aug-07	23	1	16	2	1	1	0	13	2	7	1
13870	202	0	23-Mar-07	12	1	16	2	1	1	0	13	2	2	1
13871	202	0	14-Jan-07	6	1	16	2	1	1	0	13	2	1	1
13872	202	0	18-Jun-07	17	1	16	2	1	1	0	13	2	1	1
13873	202	0	30-Jul-08	21	1	16	2	1	1	0	1	2	2	1
13874	202	0	5-Nov-08	17	1	16	2	1	1	0	1	2	7	2
13875	202	0	21-Feb-07	14	1	16	2	1	1	0	1	2	1	2
13876	202	0	16-Nov-16	17	1	16	2	2	1	0	4	2	1	1
13877	202	0	13-May-15	14	1	16	2	1	1	0	4	2	1	1
13878	202	0	23-Oct-15	18	1	16	2	2	1	0	4	2	3	1
13879	202	0	26-May-15	23	1	16	2	1	1	0	4	2	2	1
13880	202	0	24-Sep-15	21	1	16	2	1	1	0	4	2	5	1
13881	202	0	26-May-15	15	1	16	2	1	1	0	4	2	1	1

13882	202	0	20-May-14	8	1	16	2	1	1	0	4	2	4	1
13883	202	0	20-Nov-14	10	1	16	2	1	1	0	4	2	1	1
13884	202	0	29-May-14	17	1	16	2	1	1	0	4	2	3	1
13885	202	0	9-Apr-14	16	1	16	2	1	1	0	4	2	1	1
13886	202	0	5-Jun-14	20	1	16	2	1	1	0	4	2	2	1
13887	202	0	29-Jul-14	3	1	16	2	1	1	0	4	2	1	1
13888	202	0	8-Aug-14	19	1	16	2	1	1	0	4	2	2	1
13889	202	0	4-Apr-14	19	1	16	2	1	1	0	4	2	4	1
13890	202	0	4-Jun-14	19	1	16	2	1	1	0	4	2	2	1
13891	202	0	17-Apr-14	23	1	16	2	1	1	0	4	2	3	1
13892	202	0	9-Sep-13	19	1	16	2	1	1	0	4	2	1	1
13893	202	0	14-Jan-13	19	1	16	2	1	1	0	4	2	2	2
13894	202	0	30-Jul-13	8	1	16	2	1	1	0	4	2	3	1
13895	202	0	17-Jun-13	14	1	16	2	1	1	0	4	2	3	1
13896	202	0	9-May-13	19	1	16	2	1	1	0	4	2	2	1
13897	202	0	21-May-13	17	1	16	2	1	1	0	4	2	1	1
13898	202	0	19-Apr-13	13	1	16	2	1	1	0	4	2	3	1
13899	202	0	24-Apr-12	14	1	16	2	1	1	0	4	2	1	1
13900	202	0	5-Jun-12	18	1	16	2	1	1	0	4	2	1	2
13901	202	0	5-May-12	20	1	16	2	2	1	0	4	2	2	1
13902	202	0	6-Dec-12	12	1	16	2	1	1	0	4	2	1	1
13903	202	0	17-May-11	17	1	16	2	1	1	0	4	2	4	1
13904	202	0	17-May-11	17	1	16	2	1	1	0	4	2	2	1
13905	202	0	23-Dec-11	14	1	16	2	1	1	0	4	2	5	1
13906	202	0	21-Oct-11	17	1	16	2	1	1	0	4	2	6	1
13907	202	0	30-Mar-10	10	1	16	2	1	1	0	4	2	1	2
13908	202	0	30-Nov-09	23	1	16	2	1	1	0	4	2	1	1
13909	202	0	21-Dec-09	9	1	16	2	1	1	0	4	2	6	1
13910	202	0	13-Oct-09	22	1	16	2	1	1	0	4	2	6	1
13911	202	0	16-Aug-09	21	1	16	2	1	1	0	4	2	3	1
13912	202	0	27-Aug-09	17	1	16	2	1	1	0	4	2	1	2
13913	202	0	16-Jul-08	15	1	16	2	1	1	0	4	2	6	1
13914	202	0	12-Jun-08	22	1	16	2	2	1	0	4	2	1	1
13915	202	0	26-Aug-08	14	1	16	2	1	1	0	4	2	1	1
13916	202	0	11-Nov-08	13	1	16	2	3	1	0	4	2	2	1
13917	202	0	23-Oct-08	16	1	16	2	1	1	0	4	2	1	1
13918	202	0	7-Sep-08	23	1	16	2	1	1	0	4	2	2	2
13919	202	0	9-May-08	20	1	16	2	1	1	0	4	2	1	2
13920	202	0	19-Jun-08	17	1	16	2	1	1	0	4	2	1	1
13921	202	0	9-Sep-08	17	1	16	2	1	1	0	4	2	1	2
13922	202	0	14-Nov-08	16	1	16	2	1	1	0	4	2	1	1
13923	202	0	27-Oct-08	18	1	16	2	1	1	0	4	2	7	1
13924	202	0	31-Aug-07	21	1	16	2	1	1	0	4	2	1	1
13925	202	0	7-Dec-07	16	1	16	2	1	1	0	4	2	2	1
13926	202	0	7-Dec-07	16	1	16	2	1	1	0	4	2	2	1
13927	202	0	18-Nov-07	19	1	16	2	1	1	0	4	2	3	1
13928	202	0	19-Jul-07	22	1	16	2	2	1	0	4	2	2	2
13929	202	0	6-May-07	17	1	16	2	1	1	0	4	2	1	1
13930	202	0	22-Oct-07	17	1	16	2	1	1	0	4	2	1	1
13931	202	0	11-May-07	8	1	16	2	1	1	0	4	2	1	1
13932	202	0	4-Jan-07	12	1	16	2	1	1	0	4	2	2	1
13933	202	0	1-Mar-06	13	1	16	2	1	1	0	4	2	1	1
13934	202	0	21-Dec-06	21	1	16	2	1	1	0	4	2	1	1
13935	202	0	28-Feb-06	11	1	16	2	1	1	0	4	2	1	1
13936	202	0	30-Apr-06	10	1	16	2	1	1	0	4	2	6	1
13937	202	0	6-Apr-06	8	1	16	2	1	1	0	4	2	6	1
13938	202	0	16-May-17	21	1	16	2	1	1	0	4	2	1	2
13939	202	0	24-Jan-17	11	1	16	2	2	1	0	4	2	1	1
13940	202	0	2-Jun-17	20	1	16	2	1	1	0	4	2	3	1
13941	202	0	5-Apr-17	19	1	16	2	1	1	0	4	2	4	2
13942	202	0	19-Nov-17	20	1	16	2	2	1	0	4	2	1	1
13943	202	0	1-Nov-16	17	1	16	2	1	1	0	11	2	3	1
13944	202	0	24-Sep-14	18	1	16	2	1	1	0	11	2	2	1
13945	202	0	19-May-13	21	1	16	2	1	1	0	11	2	2	1
13946	202	0	17-Aug-12	14	1	16	2	1	1	0	11	2	3	2
13947	202	0	5-Feb-10	11	1	16	2	1	1	0	11	2	3	1
13948	202	0	13-Oct-07	0	1	16	2	1	1	0	11	2	2	1
13949	202	0	10-Jul-07	19	1	16	2	3	1	0	11	2	3	2
13950	202	0	31-Jul-07	21	1	16	2	1	1	0	11	2	4	1
13951	202	0	19-Feb-16	12	1	16	2	3	1	0	3	2	1	1
13952	202	0	21-Apr-16	19	1	16	2	1	1	0	3	2	1	1
13953	202	0	9-Apr-16	21	1	16	2	1	1	0	3	2	1	2
13954	202	0	1-Aug-16	17	1	16	2	2	1	0	3	2	4	1
13955	202	0	9-Dec-16	19	1	16	2	3	1	0	3	2	2	1
13956	202	0	26-Jul-16	1	1	16	2	3	1	0	3	2	1	1
13957	202	0	4-May-16	20	1	16	2	3	1	0	3	2	3	1
13958	202	0	29-Aug-16	16	1	16	2	1	1	0	3	2	2	1
13959	202	0	15-Nov-16	18	1	16	2	2	1	0	3	2	3	1
13960	202	0	16-Jun-16	19	1	16	2	1	1	0	3	2	7	1
13961	202	0	10-Oct-16	11	1	16	2	3	1	0	3	2	2	4
13962	202	0	1-Sep-15	17	1	16	2	2	1	0	3	2	5	1
13963	202	0	24-Sep-15	17	1	16	2	2	1	0	3	2	2	1
13964	202	0	8-Oct-15	15	1	16	2	3	1	0	3	2	1	2
13965	202	0	10-Jul-15	18	1	16	2	1	1	0	3	2	3	1
13966	202	0	6-Dec-15	15	1	16	2	1	1	0	3	2	3	1
13967	202	0	17-Dec-15	18	1	16	2	1	1	0	3	2	3	1
13968	202	0	4-Jul-14	10	1	16	2	1	1	0	3	2	1	1
13969	202	0	18-Dec-14	17	1	16	2	3	1	0	3	2	1	1
13970	202	0	23-Sep-14	18	1	16	2	1	1	0	3	2	1	1
13971	202	0	25-Apr-14	15	1	16	2	1	1	0	3	2	1	1
13972	202	0	10-Sep-14	15	1	16	2	3	1	0	3	2	5	1
13973	202	0	5-Nov-14	17	1	16	2	1	1	0	3	2	1	1
13974	202	0	19-Mar-14	21	1	16	2	1	1	0	3	2	1	1
13975	202	0	23-Nov-14	22	1	16	2	1	1	0	3	2	1	1
13976	202	0	29-Jul-14	9	1	16	2	1	1	0	3	2	5	2
13977	202	0	5-Oct-14	1	1	16	2	1	1	0	3	2	5	1
13978	202	0	5-Oct-14	2	1	16	2	1	1	0	3	2	5	1
13979	202	0	16-Jun-14	19	1	16	2	1	1	0	3	2	4	2
13980	202	0	23-Apr-14	1	1	16	2	1	1	0	3	2	3	1
13981	202	0	15-Oct-14	18	1	16	2	3	1	0	3	2	1	1
13982	202	0	23-Jul-14	17	1	16	2	3	1	0	3	2	2	1
13983	202	0	28-May-14	20	1	16	2	1	1	0	3	2	1	1
13984	202	0	21-Feb-14	16	1	16	2	1	1	0	3	2	3	1
13985	202	0	2-Sep-14	17	1	16	2	2	1	0	3	2	3	1
13986	202	0	21-Nov-14	15	1	16	2	1	1	0	3	2	2	2
13987	202	0	12-Jun-14	18	1	16	2	1	1	0	3	2	3	1
13988	202	0	27-Mar-14	5	1	16	2	1	1	0	3	2	2	1
13989	202	0	15-Jun-14	20	1	16	2	1	1	0	3	2	2	2
13990	202	0	4-Sep-14	18	1	16	2	1	1	0	3	2	3	1
13991	202	0	20-Mar-14	20	1	16	2	1	1	0	3	2	3	1
13992	202	0	28-Nov-14	18	1	16	2	1	1	0	3	2	2	2
13993	202	0	12-Dec-14	12	1	16	2	1	1	0	3	2	2	1
13994	202	0	19-Jun-14	17	1	16	2	2	1	0	3	2	2	1
13995	202	0	19-Apr-14	17	1	16	2	1	1	0	3	2	3	1
13996	202	0	22-Apr-14	18	1	16	2	1	1	0	3	2	2	2
13997	202	0	28-Mar-13	11	1	16	2	1	1	0	3	2	4	1
13998	202	0	13-Dec-13	18	1	16	2	1	1	0	3	2	1	1
13999	202	0	11-Nov-13	21	1	16	2	1	1	0	3	2	4	1
14000	202	0	21-Sep-13	19	1	16	2	2	1	0	3	2	2	2
14001	202	0	24-Sep-13	20	1	16	2	1	1	0	3	2	2	2
14002	202	0	14-May-13	9	1	16	2	1	1	0	3	2	2	1
14003	202	0	12-Nov-13	3	1	16	2	1	1	0	3	2	1	1
14004	202	0	28-Oct-13	21	1	16	2	1	1	0	3	2	2	1
14005	202	0	25-Sep-13	19	1	16	2	1	1	0	3	2	3	1

14006	202	0	16-Jun-13	7	1	16	2	1	1	0	3	2	2	1
14007	202	0	20-Aug-13	4	1	16	2	1	1	0	3	2	2	1
14008	202	0	17-Jun-13	19	1	16	2	1	1	0	3	2	2	1
14009	202	0	8-Aug-13	19	1	16	2	1	1	0	3	2	4	1
14010	202	0	31-May-13	9	1	16	2	1	1	0	3	2	3	1
14011	202	0	29-Sep-13	17	1	16	2	1	1	0	3	2	2	1
14012	202	0	18-Oct-13	18	1	16	2	1	1	0	3	2	2	1
14013	202	0	8-Sep-13	10	1	16	2	1	1	0	3	2	3	2
14014	202	0	1-Jun-13	11	1	16	2	1	1	0	3	2	1	1
14015	202	0	26-Nov-13	18	1	16	2	1	1	0	3	2	1	1
14016	202	0	19-Dec-13	17	1	16	2	1	1	0	3	2	3	2
14017	202	0	10-Dec-13	11	1	16	2	1	1	0	3	2	3	1
14018	202	0	5-Jun-13	10	1	16	2	1	1	0	3	2	3	1
14019	202	0	19-Feb-13	18	1	16	2	1	1	0	3	2	4	1
14020	202	0	9-Jun-13	19	1	16	2	3	1	0	3	2	2	1
14021	202	0	29-Oct-13	17	1	16	2	3	1	0	3	2	2	1
14022	202	0	25-Mar-13	20	1	16	2	1	1	0	3	2	2	2
14023	202	0	27-Oct-13	10	1	16	2	1	1	0	3	2	3	1
14024	202	0	5-Jul-13	14	1	16	2	1	1	0	3	2	2	1
14025	202	0	16-May-12	0	1	16	2	1	1	0	3	2	7	1
14026	202	0	30-Oct-12	15	1	16	2	1	1	0	3	2	2	1
14027	202	0	20-Mar-12	18	1	16	2	1	1	0	3	2	6	2
14028	202	0	18-Dec-12	12	1	16	2	1	1	0	3	2	1	1
14029	202	0	31-May-12	19	1	16	2	1	1	0	3	2	3	2
14030	202	0	24-Sep-12	17	1	16	2	1	1	0	3	2	2	1
14031	202	0	23-Jun-12	3	1	16	2	1	1	0	3	2	3	1
14032	202	0	3-Dec-12	12	1	16	2	1	1	0	3	2	2	1
14033	202	0	17-Apr-12	13	1	16	2	1	1	0	3	2	1	2
14034	202	0	29-Nov-12	18	1	16	2	1	1	0	3	2	1	1
14035	202	0	23-Oct-12	11	1	16	2	1	1	0	3	2	2	2
14036	202	0	1-Mar-12	16	1	16	2	1	1	0	3	2	3	2
14037	202	0	21-Nov-11	23	1	16	2	1	1	0	3	2	2	1
14038	202	0	16-Mar-11	19	1	16	2	1	1	0	3	2	2	1
14039	202	0	3-Nov-11	17	1	16	2	1	1	0	3	2	1	2
14040	202	0	16-Jun-11	17	1	16	2	1	1	0	3	2	1	2
14041	202	0	19-Dec-11	18	1	16	2	1	1	0	3	2	1	1
14042	202	0	14-Jun-11	12	1	16	2	1	1	0	3	2	1	2
14043	202	0	13-Aug-11	3	1	16	2	1	1	0	3	2	2	2
14044	202	0	3-Sep-11	15	1	16	2	1	1	0	3	2	3	2
14045	202	0	10-Jul-11	22	1	16	2	3	1	0	3	2	2	2
14046	202	0	2-Apr-11	12	1	16	2	1	1	0	3	2	2	1
14047	202	0	7-May-11	18	1	16	2	3	1	0	3	2	2	1
14048	202	0	3-Jun-10	17	1	16	2	1	1	0	3	2	3	2
14049	202	0	29-Oct-10	14	1	16	2	1	1	0	3	2	1	1
14050	202	0	19-Jan-10	21	1	16	2	2	1	0	3	2	1	2
14051	202	0	13-Apr-10	11	1	16	2	1	1	0	3	2	1	1
14052	202	0	20-Oct-10	20	1	16	2	1	1	0	3	2	3	2
14053	202	0	5-Nov-10	17	1	16	2	2	1	0	3	2	1	2
14054	202	0	6-Sep-10	12	1	16	2	1	1	0	3	2	2	1
14055	202	0	30-Mar-10	21	1	16	2	3	1	0	3	2	2	2
14056	202	0	19-Sep-10	18	1	16	2	1	1	0	3	2	2	2
14057	202	0	11-Sep-10	12	1	16	2	1	1	0	3	2	3	2
14058	202	0	8-Jan-10	23	1	16	2	3	1	0	3	2	6	1
14059	202	0	1-Oct-10	15	1	16	2	1	1	0	3	2	2	2
14060	202	0	26-Nov-10	17	1	16	2	2	1	0	3	2	3	2
14061	202	0	26-Nov-10	17	1	16	2	2	1	0	3	2	3	2
14062	202	0	12-Mar-10	17	1	16	2	1	1	0	3	2	4	1
14063	202	0	29-Oct-10	17	1	16	2	1	1	0	3	2	2	2
14064	202	0	21-Jan-10	19	1	16	2	1	1	0	3	2	3	2
14065	202	0	24-Dec-10	14	1	16	2	1	1	0	3	2	2	2
14066	202	0	25-Dec-10	20	1	16	2	1	1	0	3	2	1	2
14067	202	0	10-Sep-09	17	1	16	2	1	1	0	3	2	3	1
14068	202	0	12-Jun-09	8	1	16	2	1	1	0	3	2	1	1
14069	202	0	31-May-09	17	1	16	2	1	1	0	3	2	7	1
14070	202	0	4-Sep-09	9	1	16	2	1	1	0	3	2	1	1
14071	202	0	27-Nov-09	10	1	16	2	1	1	0	3	2	7	1
14072	202	0	27-Nov-09	10	1	16	2	1	1	0	3	2	7	1
14073	202	0	30-Jul-09	17	1	16	2	1	1	0	3	2	2	1
14074	202	0	16-Sep-09	23	1	16	2	1	1	0	3	2	2	1
14075	202	0	11-Dec-09	11	1	16	2	1	1	0	3	2	2	2
14076	202	0	19-Dec-09	22	1	16	2	1	1	0	3	2	1	1
14077	202	0	22-Apr-09	17	1	16	2	1	1	0	3	2	2	2
14078	202	0	3-Apr-09	10	1	16	2	1	1	0	3	2	3	2
14079	202	0	21-Oct-09	20	1	16	2	1	1	0	3	2	2	1
14080	202	0	9-Nov-09	18	1	16	2	1	1	0	3	2	2	1
14081	202	0	1-Jun-09	18	1	16	2	1	1	0	3	2	3	1
14082	202	0	25-Apr-08	18	1	16	2	1	1	0	3	2	1	1
14083	202	0	24-Nov-08	16	1	16	2	3	1	0	3	2	1	1
14084	202	0	19-Apr-08	14	1	16	2	1	1	0	3	2	1	1
14085	202	0	12-Mar-08	16	1	16	2	1	1	0	3	2	2	2
14086	202	0	21-Apr-08	7	1	16	2	1	1	0	3	2	2	2
14087	202	0	26-Nov-08	19	1	16	2	1	1	0	3	2	3	2
14088	202	0	11-May-08	6	1	16	2	2	1	0	3	2	2	2
14089	202	0	13-Sep-08	22	1	16	2	1	1	0	3	2	1	1
14090	202	0	19-Aug-08	6	1	16	2	1	1	0	3	2	2	2
14091	202	0	21-Apr-08	18	1	16	2	1	1	0	3	2	3	1
14092	202	0	21-Oct-08	18	1	16	2	3	1	0	3	2	2	2
14093	202	0	10-May-07	8	1	16	2	1	1	0	3	2	1	1
14094	202	0	3-May-07	12	1	16	2	3	1	0	3	2	2	1
14095	202	0	13-Nov-07	10	1	16	2	1	1	0	3	2	3	2
14096	202	0	2-Jun-07	10	1	16	2	1	1	0	3	2	4	1
14097	202	0	19-Apr-07	17	1	16	2	1	1	0	3	2	1	1
14098	202	0	19-Apr-07	19	1	16	2	1	1	0	3	2	1	1
14099	202	0	6-Nov-07	17	1	16	2	1	1	0	3	2	3	1
14100	202	0	14-Mar-07	17	1	16	2	1	1	0	3	2	2	1
14101	202	0	7-Dec-07	17	1	16	2	1	1	0	3	2	1	1
14102	202	0	24-Oct-07	19	1	16	2	1	1	0	3	2	1	1
14103	202	0	9-Jan-07	19	1	16	2	1	1	0	3	2	2	2
14104	202	0	30-Mar-07	19	1	16	2	1	1	0	3	2	2	1
14105	202	0	25-Mar-07	18	1	16	2	1	1	0	3	2	3	1
14106	202	0	19-Jun-07	17	1	16	2	1	1	0	3	2	2	2
14107	202	0	17-Nov-07	12	1	16	2	1	1	0	3	2	3	1
14108	202	0	4-Dec-07	17	1	16	2	1	1	0	3	2	2	1
14109	202	0	8-Oct-07	18	1	16	2	1	1	0	3	2	1	1
14110	202	0	4-Jan-06	16	1	16	2	2	1	0	3	2	1	1
14111	202	0	14-Jan-06	12	1	16	2	1	1	0	3	2	2	1
14112	202	0	28-Nov-06	16	1	16	2	1	1	0	3	2	2	2
14113	202	0	28-Feb-06	11	1	16	2	1	1	0	3	2	1	1
14114	202	0	29-Sep-06	18	1	16	2	1	1	0	3	2	4	1
14115	202	0	10-Apr-06	13	1	16	2	1	1	0	3	2	2	2
14116	202	0	1-Jun-06	17	1	16	2	3	1	0	3	2	2	2
14117	202	0	5-Dec-06	19	1	16	2	1	1	0	3	2	1	1
14118	202	0	13-Oct-06	17	1	16	2	1	1	0	3	2	2	2
14119	202	0	28-Nov-06	16	1	16	2	1	1	0	3	2	3	1
14120	202	0	19-Mar-06	15	1	16	2	1	1	0	3	2	1	1
14121	202	0	19-May-06	2	1	16	2	2	1	0	3	2	2	2
14122	202	0	9-Jan-17	17	1	16	2	1	1	0	3	2	5	1
14123	202	0	17-Oct-17	16	1	16	2	1	1	0	3	2	3	1
14124	202	0	17-Feb-17	16	1	16	2	1	1	0	3	2	3	1
14125	202	0	9-Oct-17	9	1	16	2	1	1	0	3	2	1	1
14126	202	0	12-Aug-17	18	1	16	2	2	1	0	3	2	4	1
14127	202	0	9-Mar-17	0	1	16	2	2	1	0	3	2	2	1
14128	202	0	5-Feb-17	19	1	16	2	2	1	0	3	2	3	2
14129	202	0	30-Mar-17	17	1	16	2	3	1	0	3	2	3	1

14130	202	0	8-Sep-17	16	1	16	2	3	1	0	3	2	3	1
14131	202	0	15-Aug-17	2	1	16	2	3	1	0	3	2	2	1
14132	202	0	21-Nov-17	18	1	16	2	3	1	0	3	2	1	1
14133	202	0	9-Jan-17	17	1	16	2	3	1	0	3	2	1	1
14134	202	0	25-Mar-17	9	1	16	2	3	1	0	3	2	1	1
14135	202	0	29-May-17	13	1	16	2	1	1	0	3	2	3	1
14136	202	0	14-Sep-17	17	1	16	2	1	1	0	3	2	3	1
14137	202	0	1-Feb-17	16	1	16	2	1	1	0	3	2	1	1
14138	202	0	20-Aug-17	16	1	16	2	1	1	0	3	2	1	1
14139	202	0	3-May-17	20	1	16	2	2	1	0	3	2	1	1
14140	202	0	6-Apr-17	16	1	16	2	3	1	0	3	2	3	2
14141	202	0	28-Apr-16	14	1	16	2	1	1	0	6	2	2	2
14142	202	0	17-May-16	11	1	16	2	1	1	0	6	2	3	1
14143	202	0	25-Sep-15	10	1	16	2	1	1	0	6	2	2	1
14144	202	0	10-Oct-13	14	1	16	2	2	1	0	6	2	2	1
14145	202	0	8-Jul-13	22	1	16	2	1	1	0	6	2	2	1
14146	202	0	28-Oct-12	13	1	16	2	1	1	0	6	2	2	1
14147	202	0	4-Dec-12	19	1	16	2	1	1	0	6	2	2	1
14148	202	0	5-Mar-12	22	1	16	2	1	1	0	6	2	2	2
14149	202	0	11-May-10	21	1	16	2	1	1	0	6	2	2	1
14150	202	0	27-Oct-09	9	1	16	2	1	1	0	6	2	1	1
14151	202	0	27-Oct-09	9	1	16	2	1	1	0	6	2	1	1
14152	202	0	8-Aug-09	10	1	16	2	1	1	0	6	2	2	1
14153	202	0	8-Aug-09	10	1	16	2	1	1	0	6	2	2	1
14154	202	0	21-Mar-08	10	1	16	2	1	1	0	6	2	1	2
14155	202	0	6-Nov-08	15	1	16	2	2	1	0	6	2	2	1
14156	202	0	6-Jun-08	14	1	16	2	1	1	0	6	2	2	1
14157	202	0	14-Apr-08	22	1	16	2	1	1	0	6	2	3	1
14158	202	0	7-Sep-08	22	1	16	2	1	1	0	6	2	2	2
14159	202	0	4-Jan-08	17	1	16	2	1	1	0	6	2	2	1
14160	202	0	6-Sep-07	0	1	16	2	2	1	0	6	2	2	2
14161	202	0	28-Sep-07	20	1	16	2	1	1	0	6	2	2	2
14162	202	0	30-Oct-07	16	1	16	2	1	1	0	6	2	2	1
14163	202	0	11-May-07	1	1	16	2	1	1	0	6	2	2	1
14164	202	0	28-Mar-07	10	1	16	2	1	1	0	6	2	2	1
14165	202	0	14-Oct-07	17	1	16	2	1	1	0	6	2	1	1
14166	202	0	27-Jun-07	16	1	16	2	1	1	0	6	2	2	1
14167	202	0	7-Oct-07	13	1	16	2	1	1	0	6	2	2	1
14168	202	0	6-Sep-07	8	1	16	2	1	1	0	6	2	3	1
14169	202	0	5-Jul-07	20	1	16	2	1	1	0	6	2	1	2
14170	202	0	14-Sep-07	2	1	16	2	1	1	0	6	2	1	1
14171	202	0	19-May-07	17	1	16	2	1	1	0	6	2	2	1
14172	202	0	19-Jun-07	1	1	16	2	1	1	0	6	2	1	2
14173	202	0	17-Apr-07	16	1	16	2	1	1	0	6	2	3	1
14174	202	0	6-Dec-06	17	1	16	2	1	1	0	6	2	2	1
14175	202	0	12-Nov-17	20	1	16	2	3	1	0	6	2	2	1
14176	202	0	1-Sep-17	20	1	16	2	1	1	0	6	2	2	1
14177	202	0	28-Nov-11	12	1	16	2	3	1	0	13	2	4	1
14178	202	0	15-Sep-08	0	1	16	2	1	1	0	13	2	3	1
14179	202	0	31-Oct-08	9	1	16	2	1	1	0	13	2	1	2
14180	202	0	17-Jan-07	11	1	16	2	1	1	0	13	2	1	1
14181	202	0	10-Sep-13	0	2	16	2	1	1	0	10	2	6	1
14182	202	0	24-Jun-16	19	2	16	2	1	1	0	4	2	3	1
14183	202	0	10-Oct-16	10	2	16	2	3	1	0	10	2	6	2
14184	202	0	27-Mar-12	21	2	16	2	3	1	0	6	2	2	1
14185	202	0	9-Jan-12	18	2	16	2	1	1	0	6	2	2	2
14186	202	0	22-Jun-17	0	2	16	2	1	1	0	6	2	2	1
14187	202	0	9-Dec-17	17	2	16	2	1	1	0	11	2	2	1
14188	202	0	18-Oct-11	18	2	16	2	1	1	0	10	2	7	2
14189	202	0	15-Feb-07	0	2	16	2	1	1	0	10	2	7	2
14190	202	0	18-Dec-06	23	2	16	2	1	1	0	10	2	3	2
14191	202	0	12-Jul-09	13	2	16	2	1	1	0	7	2	3	1
14192	202	0	4-Jul-12	1	2	16	2	1	1	0	13	2	1	1
14193	202	0	11-Nov-09	18	2	16	2	1	1	0	13	2	3	1
14194	202	0	5-Dec-07	23	2	16	2	1	1	0	13	2	6	1
14195	202	0	6-Dec-17	15	2	16	2	1	1	0	13	2	1	1
14196	202	0	7-Nov-10	18	2	16	2	1	1	0	1	2	6	1
14197	202	0	19-Dec-09	1	2	16	2	1	1	0	1	2	3	2
14198	202	0	18-Jun-07	19	2	16	2	1	1	0	1	2	1	2
14199	202	0	5-Nov-08	17	2	16	2	1	1	0	1	2	1	2
14200	202	0	12-Oct-16	0	2	16	2	1	1	0	4	2	1	1
14201	202	0	12-May-16	17	2	16	2	1	1	0	4	2	2	1
14202	202	0	15-Oct-14	17	2	16	2	3	1	0	4	2	3	1
14203	202	0	3-Dec-13	17	2	16	2	1	1	0	4	2	1	1
14204	202	0	11-Nov-11	17	2	16	2	1	1	0	4	2	2	2
14205	202	0	22-Jul-08	19	2	16	2	3	1	0	4	2	3	1
14206	202	0	14-Mar-07	17	2	16	2	1	1	0	4	2	2	1
14207	202	0	7-Nov-17	23	2	16	2	1	1	0	4	2	2	1
14208	202	0	21-Aug-16	17	2	16	2	2	1	0	11	2	3	1
14209	202	0	1-Jul-16	10	2	16	2	3	1	0	11	2	3	1
14210	202	0	10-Jul-15	23	2	16	2	2	1	0	11	2	2	2
14211	202	0	3-Oct-14	10	2	16	2	2	1	0	11	2	2	1
14212	202	0	28-May-09	19	2	16	2	1	1	0	11	2	2	1
14213	202	0	18-Aug-07	22	2	16	2	1	1	0	11	2	3	1
14214	202	0	30-May-17	9	2	16	2	1	1	0	11	2	2	1
14215	202	0	20-Jun-17	11	2	16	2	2	1	0	11	2	3	1
14216	202	0	16-Aug-17	11	2	16	2	2	1	0	11	2	1	1
14217	202	0	19-Apr-16	0	2	16	2	2	1	0	3	2	1	1
14218	202	0	25-Jan-16	7	2	16	2	1	1	0	3	2	4	1
14219	202	0	17-Dec-16	20	2	16	2	1	1	0	3	2	3	1
14220	202	0	17-Jun-16	12	2	16	2	1	1	0	3	2	2	2
14221	202	0	14-May-15	12	2	16	2	3	1	0	3	2	1	1
14222	202	0	24-Oct-15	11	2	16	2	1	1	0	3	2	3	1
14223	202	0	8-May-14	21	2	16	2	1	1	0	3	2	4	1
14224	202	0	18-Nov-14	3	2	16	2	1	1	0	3	2	1	1
14225	202	0	23-Nov-14	4	2	16	2	3	1	0	3	2	5	1
14226	202	0	4-May-14	8	2	16	2	1	1	0	3	2	4	1
14227	202	0	3-Aug-14	19	2	16	2	2	1	0	3	2	3	1
14228	202	0	3-May-14	15	2	16	2	1	1	0	3	2	3	1
14229	202	0	9-Apr-14	14	2	16	2	1	1	0	3	2	4	1
14230	202	0	7-Aug-13	1	2	16	2	1	1	0	3	2	1	2
14231	202	0	28-Oct-13	16	2	16	2	1	1	0	3	2	4	1
14232	202	0	25-Nov-13	17	2	16	2	1	1	0	3	2	2	1
14233	202	0	19-Dec-13	17	2	16	2	2	1	0	3	2	1	1
14234	202	0	25-Sep-13	16	2	16	2	1	1	0	3	2	3	1
14235	202	0	6-Apr-13	16	2	16	2	3	1	0	3	2	3	1
14236	202	0	6-Oct-13	17	2	16	2	1	1	0	3	2	2	1
14237	202	0	27-Oct-12	15	2	16	2	3	1	0	3	2	4	2
14238	202	0	27-Oct-12	15	2	16	2	3	1	0	3	2	4	2
14239	202	0	23-Nov-12	17	2	16	2	1	1	0	3	2	3	1
14240	202	0	3-Sep-12	22	2	16	2	1	1	0	3	2	3	2
14241	202	0	22-Oct-12	17	2	16	2	2	1	0	3	2	3	2
14242	202	0	10-Oct-11	19	2	16	2	1	1	0	3	2	6	2
14243	202	0	13-Nov-11	2	2	16	2	1	1	0	3	2	2	2
14244	202	0	12-Sep-11	12	2	16	2	1	1	0	3	2	2	2
14245	202	0	23-Feb-10	23	2	16	2	1	1	0	3	2	2	2
14246	202	0	29-Oct-09	17	2	16	2	3	1	0	3	2	1	1
14247	202	0	29-Sep-09	16	2	16	2	1	1	0	3	2	2	2
14248	202	0	11-Nov-09	18	2	16	2	1	1	0	3	2	3	1
14249	202	0	12-Jul-08	19	2	16	2	3	1	0	3	2	2	1
14250	202	0	23-May-08	7	2	16	2	1	1	0	3	2	3	1
14251	202	0	23-Jul-07	22	2	16	2	1	1	0	3	2	7	2
14252	202	0	16-Nov-07	10	2	16	2	1	1	0	3	2	3	1
14253	202	0	4-Nov-06	16	2	16	2	1	1	0	3	2	1	1

14254	202	0	20-Jul-06	21	2	16	2	1	1	0	3	2	2	1
14255	202	0	16-Jun-17	19	2	16	2	1	1	0	3	2	3	1
14256	202	0	29-May-17	13	2	16	2	1	1	0	3	2	3	1
14257	202	0	1-Nov-17	17	2	16	2	2	1	0	3	2	2	1
14258	202	0	25-Sep-17	11	2	16	2	2	1	0	3	2	1	1
14259	202	0	29-Sep-17	17	2	16	2	3	1	0	3	2	1	1
14260	202	0	13-May-11	17	2	16	2	1	1	0	6	2	2	1
14261	202	0	22-Feb-11	21	2	16	2	1	1	0	6	2	7	1
14262	202	0	8-Aug-10	1	2	16	2	1	1	0	6	2	3	2
14263	202	0	29-Oct-08	12	2	16	2	1	1	0	6	2	2	1
14264	202	0	19-Mar-08	19	2	16	2	1	1	0	6	2	3	2
14265	202	0	26-May-08	19	2	16	2	1	1	0	6	2	3	1
14266	202	0	13-Jul-07	8	2	16	2	1	1	0	13	2	1	1
14267	202	0	3-Jun-07	17	2	16	2	1	1	0	13	2	4	1
14268	101	1	16-Oct-13	17	1	16	2	1	1	0	1	2	5	1
14269	201	1	13-Aug-17	13	1	16	2	2	1	0	1	2	3	1
14270	201	1	29-Sep-16	20	1	16	2	1	1	0	4	2	3	1
14271	201	1	1-May-12	15	1	16	2	3	1	0	6	2	2	1
14272	101	1	7-Dec-14	7	1	16	2	1	1	0	6	2	3	1
14273	201	1	14-Apr-17	16	1	16	2	2	1	0	6	2	2	1
14274	201	1	28-Jun-12	17	1	16	2	2	1	0	3	2	1	1
14275	101	1	27-Nov-17	16	1	16	2	1	1	0	6	2	2	1
14276	201	1	8-Feb-16	19	1	16	2	1	1	0	4	2	1	1
14277	201	1	9-Jul-14	11	1	16	2	2	1	0	4	2	2	1
14278	201	1	13-May-14	11	1	16	2	2	1	0	6	2	2	1
14279	201	1	6-Aug-13	13	1	16	2	2	1	0	6	2	2	1
14280	201	1	6-Nov-15	17	1	16	2	2	1	0	6	2	2	1
14281	101	1	12-Jun-13	23	1	16	2	1	1	0	4	2	2	1
14282	201	1	1-Jun-12	20	1	16	2	2	1	0	6	2	2	1
14283	201	1	28-Aug-16	8	1	16	2	2	1	0	4	2	3	1
14284	201	1	20-Oct-17	16	1	16	2	2	1	0	6	2	2	1
14285	201	1	30-Mar-15	10	1	16	2	1	1	0	6	2	2	1
14286	201	1	2-Apr-15	19	1	16	2	3	1	0	4	2	2	2
14287	101	1	9-Apr-17	20	1	16	2	1	1	0	6	2	2	1
14288	201	1	17-Aug-16	8	1	16	2	1	1	0	6	2	3	1
14289	201	1	13-Feb-14	18	1	16	2	1	1	0	3	2	1	1
14290	201	1	12-Oct-16	13	1	16	2	3	1	0	6	2	3	1
14291	201	1	1-Jul-17	11	1	16	2	1	1	0	6	2	3	1
14292	101	1	12-May-17	12	1	16	2	1	1	0	3	2	2	1
14293	101	1	10-Jun-11	11	1	16	2	1	1	0	7	2	2	1
14294	101	1	6-Apr-10	20	1	16	2	1	1	0	7	2	4	2
14295	101	1	20-Oct-09	9	1	16	2	1	1	0	7	2	3	1
14296	101	1	14-May-17	16	1	16	2	1	1	0	7	2	2	1
14297	101	1	19-Feb-14	17	1	16	2	1	1	0	13	2	4	1
14298	101	1	14-Dec-12	10	1	16	2	1	1	0	13	2	3	4
14299	201	1	21-May-12	19	1	16	2	1	1	0	13	2	1	1
14300	101	1	2-May-11	10	1	16	2	1	1	0	13	2	3	1
14301	101	1	3-Aug-10	20	1	16	2	1	1	0	13	2	3	1
14302	101	1	8-Sep-10	17	1	16	2	1	1	0	13	2	1	1
14303	201	1	9-Apr-10	16	1	16	2	2	1	0	13	2	4	1
14304	101	1	17-Jun-09	18	1	16	2	1	1	0	13	2	1	1
14305	101	1	30-Oct-09	9	1	16	2	1	1	0	13	2	4	1
14306	201	1	2-Sep-09	22	1	16	2	1	1	0	13	2	2	2
14307	101	1	11-Jan-08	18	1	16	2	1	1	0	13	2	4	1
14308	201	1	4-Apr-06	21	1	16	2	1	1	0	13	2	3	1
14309	101	1	31-Jan-06	17	1	16	2	1	1	0	13	2	3	1
14310	101	1	4-Apr-06	17	1	16	2	1	1	0	13	2	1	1
14311	101	1	14-Apr-17	20	1	16	2	1	1	0	13	2	3	1
14312	201	1	13-Sep-10	13	1	16	2	2	1	0	1	2	5	1
14313	201	1	4-Sep-10	19	1	16	2	1	1	0	1	2	5	1
14314	101	1	2-Aug-08	15	1	16	2	1	1	0	1	2	1	1
14315	101	1	14-Oct-08	12	1	16	2	1	1	0	1	2	1	2
14316	101	1	24-Feb-11	21	1	16	2	1	1	0	1	2	1	1
14317	201	1	30-Dec-10	18	1	16	2	2	1	0	1	2	2	1
14318	101	1	24-Mar-16	6	1	16	2	1	1	0	4	2	3	2
14319	101	1	10-Jun-16	21	1	16	2	1	1	0	4	2	1	1
14320	101	1	28-Oct-16	20	1	16	2	1	1	0	4	2	3	1
14321	101	1	6-Oct-15	17	1	16	2	1	1	0	4	2	4	4
14322	101	1	23-Apr-15	18	1	16	2	1	1	0	4	2	1	1
14323	101	1	25-Mar-15	16	1	16	2	1	1	0	4	2	3	1
14324	101	1	27-Mar-15	20	1	16	2	1	1	0	4	2	2	1
14325	201	1	15-Apr-15	17	1	16	2	3	1	0	4	2	2	1
14326	101	1	10-Jul-15	18	1	16	2	1	1	0	4	2	2	1
14327	201	1	9-Jun-15	17	1	16	2	1	1	0	4	2	2	1
14328	101	1	15-Sep-14	12	1	16	2	1	1	0	4	2	4	1
14329	201	1	24-Feb-14	15	1	16	2	1	1	0	4	2	1	1
14330	201	1	12-Dec-14	10	1	16	2	2	1	0	4	2	2	1
14331	101	1	12-Jun-14	15	1	16	2	1	1	0	4	2	2	2
14332	101	1	13-Jun-14	19	1	16	2	1	1	0	4	2	2	1
14333	101	1	14-Dec-14	18	1	16	2	1	1	0	4	2	4	1
14334	101	1	17-Feb-14	17	1	16	2	1	1	0	4	2	4	1
14335	101	1	11-Apr-14	23	1	16	2	1	1	0	4	2	1	1
14336	101	1	13-May-14	20	1	16	2	1	1	0	4	2	2	2
14337	101	1	13-Sep-14	19	1	16	2	1	1	0	4	2	3	1
14338	201	1	15-Aug-14	22	1	16	2	3	1	0	4	2	3	1
14339	101	1	28-Feb-14	16	1	16	2	1	1	0	4	2	4	1
14340	101	1	18-Oct-14	16	1	16	2	1	1	0	4	2	4	1
14341	201	1	26-Apr-14	16	1	16	2	2	1	0	4	2	1	1
14342	101	1	24-Jun-14	18	1	16	2	1	1	0	4	2	2	4
14343	201	1	27-Jan-14	13	1	16	2	2	1	0	4	2	1	1
14344	101	1	11-Nov-13	22	1	16	2	1	1	0	4	2	4	1
14345	101	1	18-Dec-13	10	1	16	2	1	1	0	4	2	2	1
14346	201	1	6-Mar-13	11	1	16	2	3	1	0	4	2	2	1
14347	101	1	1-Feb-13	11	1	16	2	1	1	0	4	2	1	1
14348	101	1	4-Jul-13	11	1	16	2	1	1	0	4	2	3	1
14349	101	1	26-Jun-13	23	1	16	2	1	1	0	4	2	2	4
14350	201	1	4-Jan-13	21	1	16	2	3	1	0	4	2	3	1
14351	101	1	4-Jul-13	22	1	16	2	1	1	0	4	2	2	1
14352	101	1	24-Jun-13	17	1	16	2	1	1	0	4	2	2	1
14353	101	1	23-Apr-13	17	1	16	2	1	1	0	4	2	1	1
14354	101	1	12-Jun-13	20	1	16	2	1	1	0	4	2	1	1
14355	101	1	27-Jun-13	20	1	16	2	1	1	0	4	2	2	1
14356	101	1	2-Jul-13	10	1	16	2	1	1	0	4	2	2	1
14357	101	1	25-Jun-13	16	1	16	2	1	1	0	4	2	2	4
14358	101	1	26-Nov-12	11	1	16	2	1	1	0	4	2	2	1
14359	101	1	2-Jun-12	6	1	16	2	1	1	0	4	2	2	1
14360	101	1	2-Sep-12	5	1	16	2	1	1	0	4	2	2	4
14361	101	1	4-Mar-12	3	1	16	2	1	1	0	4	2	2	4
14362	101	1	15-Mar-12	23	1	16	2	1	1	0	4	2	2	1
14363	101	1	26-Apr-12	17	1	16	2	1	1	0	4	2	4	1
14364	101	1	18-Sep-12	16	1	16	2	1	1	0	4	2	4	1
14365	101	1	11-Apr-12	16	1	16	2	1	1	0	4	2	1	1
14366	101	1	5-Jan-11	15	1	16	2	1	1	0	4	2	1	1
14367	101	1	8-Dec-11	11	1	16	2	1	1	0	4	2	2	1
14368	101	1	3-Mar-11	20	1	16	2	1	1	0	4	2	1	1
14369	101	1	12-Aug-11	10	1	16	2	1	1	0	4	2	3	1
14370	101	1	22-Mar-11	19	1	16	2	1	1	0	4	2	1	1
14371	101	1	15-Nov-10	17	1	16	2	1	1	0	4	2	2	1
14372	101	1	16-Apr-10	18	1	16	2	1	1	0	4	2	4	1
14373	101	1	9-Jan-10	12	1	16	2	1	1	0	4	2	2	1
14374	201	1	2-Jun-10	16	1	16	2	2	1	0	4	2	2	1
14375	101	1	22-Jul-10	12	1	16	2	1	1	0	4	2	1	1
14376	101	1	31-Jul-10	16	1	16	2	1	1	0	4	2	1	1
14377	201	1	20-Jan-10	20	1	16	2	1	1	0	4	2	1	1

14378	201	1	7-Sep-10	22	1	16	2	1	1	0	4	2	3	1
14379	101	1	25-Dec-10	18	1	16	2	1	1	0	4	2	2	1
14380	101	1	3-Jun-10	17	1	16	2	1	1	0	4	2	1	1
14381	101	1	20-Jan-10	18	1	16	2	1	1	0	4	2	2	1
14382	101	1	18-Jun-10	17	1	16	2	1	1	0	4	2	3	1
14383	101	1	22-Dec-10	23	1	16	2	3	1	0	4	2	1	1
14384	101	1	27-Apr-10	17	1	16	2	1	1	0	4	2	3	1
14385	201	1	20-Apr-10	10	1	16	2	1	1	0	4	2	1	1
14386	101	1	12-Oct-09	11	1	16	2	1	1	0	4	2	1	2
14387	101	1	15-Apr-09	17	1	16	2	1	1	0	4	2	2	1
14388	101	1	1-Oct-09	8	1	16	2	1	1	0	4	2	5	1
14389	201	1	6-Mar-09	18	1	16	2	2	1	0	4	2	4	2
14390	201	1	6-Mar-09	8	1	16	2	1	1	0	4	2	2	1
14391	101	1	24-Mar-09	17	1	16	2	1	1	0	4	2	1	1
14392	101	1	6-Mar-09	17	1	16	2	1	1	0	4	2	1	1
14393	101	1	9-Oct-09	17	1	16	2	1	1	0	4	2	1	1
14394	101	1	25-Feb-09	23	1	16	2	1	1	0	4	2	1	1
14395	101	1	20-Mar-08	14	1	16	2	2	1	0	4	2	2	1
14396	201	1	16-Dec-08	21	1	16	2	1	1	0	4	2	2	1
14397	101	1	12-Dec-08	17	1	16	2	1	1	0	4	2	4	1
14398	101	1	24-Mar-08	18	1	16	2	1	1	0	4	2	4	2
14399	101	1	10-Mar-08	15	1	16	2	1	1	0	4	2	2	1
14400	101	1	17-Sep-07	18	1	16	2	1	1	0	4	2	1	1
14401	101	1	6-Apr-07	9	1	16	2	2	1	0	4	2	1	2
14402	201	1	23-Mar-07	15	1	16	2	1	1	0	4	2	1	1
14403	201	1	8-Oct-07	4	1	16	2	2	1	0	4	2	1	1
14404	101	1	14-Sep-07	18	1	16	2	1	1	0	4	2	3	1
14405	201	1	22-Nov-07	9	1	16	2	2	1	0	4	2	1	1
14406	101	1	13-Apr-07	17	1	16	2	1	1	0	4	2	1	1
14407	101	1	23-Jun-06	0	1	16	2	1	1	0	4	2	4	1
14408	101	1	27-Sep-06	21	1	16	2	1	1	0	4	2	4	1
14409	101	1	15-Sep-06	18	1	16	2	1	1	0	4	2	1	1
14410	101	1	29-Jun-06	17	1	16	2	1	1	0	4	2	4	1
14411	101	1	17-Dec-06	15	1	16	2	1	1	0	4	2	3	1
14412	101	1	25-Apr-06	20	1	16	2	1	1	0	4	2	2	1
14413	101	1	5-Apr-06	18	1	16	2	1	1	0	4	2	7	1
14414	101	1	22-Mar-17	12	1	16	2	1	1	0	4	2	3	1
14415	101	1	6-Apr-17	19	1	16	2	1	1	0	4	2	3	1
14416	101	1	7-Apr-16	4	1	16	2	1	1	0	11	2	2	1
14417	101	1	18-Dec-16	14	1	16	2	1	1	0	11	2	3	1
14418	101	1	6-Nov-16	22	1	16	2	1	1	0	11	2	3	1
14419	201	1	4-Aug-16	17	1	16	2	2	1	0	11	2	2	1
14420	101	1	19-Feb-16	20	1	16	2	1	1	0	11	2	2	1
14421	101	1	5-Jun-16	7	1	16	2	1	1	0	11	2	2	1
14422	101	1	7-Feb-16	13	1	16	2	1	1	0	11	2	3	1
14423	101	1	5-Jun-16	16	1	16	2	1	1	0	11	2	3	1
14424	201	1	31-May-15	16	1	16	2	2	1	0	11	2	1	1
14425	101	1	8-May-15	14	1	16	2	1	1	0	11	2	3	2
14426	101	1	28-Jul-15	17	1	16	2	1	1	0	11	2	2	1
14427	101	1	1-Feb-15	18	1	16	2	3	1	0	11	2	3	1
14428	101	1	25-Dec-15	21	1	16	2	1	1	0	11	2	2	1
14429	201	1	27-Jul-14	19	1	16	2	3	1	0	11	2	2	1
14430	201	1	11-Jun-13	18	1	16	2	1	1	0	11	2	3	1
14431	101	1	14-Nov-13	19	1	16	2	1	1	0	11	2	2	1
14432	101	1	18-Jun-12	18	1	16	2	1	1	0	11	2	5	1
14433	101	1	23-Mar-12	17	1	16	2	1	1	0	11	2	2	2
14434	101	1	16-Oct-12	12	1	16	2	1	1	0	11	2	1	1
14435	101	1	30-Jun-12	12	1	16	2	1	1	0	11	2	3	1
14436	101	1	14-Nov-12	19	1	16	2	1	1	0	11	2	3	1
14437	101	1	12-Dec-11	12	1	16	2	1	1	0	11	2	4	1
14438	201	1	11-Apr-11	15	1	16	2	1	1	0	11	2	3	1
14439	101	1	12-Jul-11	12	1	16	2	1	1	0	11	2	1	1
14440	101	1	17-Nov-11	18	1	16	2	1	1	0	11	2	2	1
14441	201	1	28-Oct-10	9	1	16	2	2	1	0	11	2	2	1
14442	201	1	20-Apr-10	9	1	16	2	1	1	0	11	2	3	1
14443	101	1	23-Sep-10	19	1	16	2	1	1	0	11	2	2	1
14444	101	1	11-Jun-10	20	1	16	2	1	1	0	11	2	2	1
14445	201	1	9-May-09	19	1	16	2	3	1	0	11	2	2	1
14446	201	1	13-Jul-09	21	1	16	2	1	1	0	11	2	3	1
14447	201	1	29-Sep-07	20	1	16	2	1	1	0	11	2	3	1
14448	201	1	19-Oct-06	20	1	16	2	2	1	0	11	2	2	1
14449	201	1	17-Sep-06	10	1	16	2	3	1	0	11	2	2	1
14450	201	1	16-Oct-06	12	1	16	2	1	1	0	11	2	3	1
14451	201	1	5-Mar-17	11	1	16	2	2	1	0	11	2	3	1
14452	201	1	2-Jul-17	11	1	16	2	3	1	0	11	2	2	1
14453	101	1	10-Jan-16	17	1	16	2	1	1	0	3	2	1	1
14454	101	1	19-Oct-16	17	1	16	2	1	1	0	3	2	2	1
14455	201	1	5-Apr-16	18	1	16	2	2	1	0	3	2	1	1
14456	201	1	12-Jan-16	13	1	16	2	2	1	0	3	2	3	1
14457	101	1	11-Mar-16	12	1	16	2	1	1	0	3	2	2	1
14458	101	1	22-Mar-16	18	1	16	2	1	1	0	3	2	3	1
14459	101	1	7-Aug-16	22	1	16	2	1	1	0	3	2	3	4
14460	101	1	7-May-16	1	1	16	2	1	1	0	3	2	5	1
14461	101	1	23-Feb-16	1	1	16	2	1	1	0	3	2	1	1
14462	201	1	26-Mar-16	20	1	16	2	2	1	0	3	2	3	1
14463	101	1	29-May-16	15	1	16	2	1	1	0	3	2	3	1
14464	101	1	1-Nov-16	16	1	16	2	1	1	0	3	2	2	1
14465	101	1	6-Aug-16	16	1	16	2	1	1	0	3	2	1	1
14466	101	1	8-Apr-16	17	1	16	2	1	1	0	3	2	2	1
14467	101	1	20-Apr-16	16	1	16	2	1	1	0	3	2	4	1
14468	201	1	4-Jan-16	15	1	16	2	1	1	0	3	2	1	1
14469	201	1	23-Feb-16	14	1	16	2	1	1	0	3	2	1	1
14470	201	1	1-Jan-16	12	1	16	2	2	1	0	3	2	3	1
14471	101	1	9-Dec-16	19	1	16	2	1	1	0	3	2	3	1
14472	101	1	10-Jan-16	9	1	16	2	1	1	0	3	2	3	1
14473	101	1	28-Jun-16	17	1	16	2	1	1	0	3	2	3	1
14474	101	1	4-Nov-16	14	1	16	2	1	1	0	3	2	3	1
14475	101	1	19-Dec-16	19	1	16	2	1	1	0	3	2	7	1
14476	101	1	9-Dec-16	19	1	16	2	1	1	0	3	2	2	1
14477	201	1	31-Mar-16	21	1	16	2	1	1	0	3	2	3	1
14478	101	1	7-Apr-16	15	1	16	2	1	1	0	3	2	3	4
14479	201	1	18-Nov-16	9	1	16	2	2	1	0	3	2	3	2
14480	101	1	30-Dec-16	16	1	16	2	1	1	0	3	2	3	1
14481	201	1	29-Jun-16	10	1	16	2	1	1	0	3	2	3	1
14482	101	1	20-Apr-16	19	1	16	2	1	1	0	3	2	2	1
14483	101	1	6-May-16	19	1	16	2	1	1	0	3	2	2	1
14484	101	1	26-Apr-16	17	1	16	2	1	1	0	3	2	3	1
14485	201	1	22-Apr-16	14	1	16	2	3	1	0	3	2	1	1
14486	101	1	13-May-16	20	1	16	2	1	1	0	3	2	1	1
14487	201	1	23-Jan-16	10	1	16	2	2	1	0	3	2	4	1
14488	201	1	6-Sep-16	11	1	16	2	1	1	0	3	2	3	1
14489	101	1	4-Nov-16	14	1	16	2	1	1	0	3	2	3	1
14490	201	1	16-Apr-16	15	1	16	2	1	1	0	3	2	5	1
14491	201	1	16-Apr-16	13	1	16	2	3	1	0	3	2	2	1
14492	201	1	8-Apr-16	18	1	16	2	2	1	0	3	2	2	1
14493	201	1	1-Dec-16	18	1	16	2	1	1	0	3	2	3	1
14494	101	1	4-Jan-16	14	1	16	2	1	1	0	3	2	2	1
14495	201	1	13-Oct-16	19	1	16	2	1	1	0	3	2	2	1
14496	101	1	1-Mar-16	13	1	16	2	1	1	0	3	2	3	1
14497	201	1	15-Dec-16	17	1	16	2	1	1	0	3	2	3	1
14498	201	1	10-Sep-16	12	1	16	2	2	1	0	3	2	1	1
14499	101	1	1-May-15	10	1	16	2	1	1	0	3	2	3	1
14500	201	1	12-Aug-15	16	1	16	2	3	1	0	3	2	3	1
14501	201	1	4-Mar-15	17	1	16	2	2	1	0	3	2	4	1

14502	201	1	16-Apr-15	10	1	16	2	1	1	0	3	2	3	1
14503	101	1	24-Mar-15	12	1	16	2	1	1	0	3	2	3	1
14504	101	1	13-Nov-15	18	1	16	2	1	1	0	3	2	3	1
14505	101	1	5-Dec-15	9	1	16	2	3	1	0	3	2	2	4
14506	201	1	2-Apr-15	20	1	16	2	1	1	0	3	2	5	1
14507	101	1	4-Apr-15	18	1	16	2	1	1	0	3	2	3	1
14508	201	1	21-Apr-15	16	1	16	2	1	1	0	3	2	3	1
14509	201	1	8-Jun-15	18	1	16	2	2	1	0	3	2	2	1
14510	101	1	3-Oct-15	7	1	16	2	1	1	0	3	2	2	1
14511	101	1	24-Jan-15	11	1	16	2	1	1	0	3	2	5	1
14512	101	1	31-Jan-15	22	1	16	2	1	1	0	3	2	1	1
14513	101	1	23-May-15	17	1	16	2	1	1	0	3	2	4	1
14514	201	1	24-Feb-15	12	1	16	2	1	1	0	3	2	1	1
14515	201	1	23-Feb-15	8	1	16	2	1	1	0	3	2	1	1
14516	101	1	20-Jan-15	17	1	16	2	1	1	0	3	2	1	1
14517	101	1	25-Mar-15	17	1	16	2	1	1	0	3	2	1	1
14518	101	1	3-Aug-15	16	1	16	2	1	1	0	3	2	3	1
14519	101	1	4-Feb-15	17	1	16	2	1	1	0	3	2	3	1
14520	101	1	8-Oct-15	21	1	16	2	1	1	0	3	2	2	1
14521	201	1	10-Jun-15	18	1	16	2	2	1	0	3	2	1	1
14522	101	1	11-Apr-15	1	1	16	2	1	1	0	3	2	1	1
14523	201	1	19-Apr-15	14	1	16	2	2	1	0	3	2	2	1
14524	101	1	5-Feb-15	16	1	16	2	1	1	0	3	2	3	1
14525	101	1	30-Jul-15	12	1	16	2	1	1	0	3	2	3	1
14526	201	1	1-Apr-15	20	1	16	2	1	1	0	3	2	1	1
14527	101	1	20-Apr-15	12	1	16	2	1	1	0	3	2	1	1
14528	101	1	25-Sep-15	17	1	16	2	1	1	0	3	2	1	1
14529	101	1	5-Nov-15	19	1	16	2	1	1	0	3	2	1	1
14530	201	1	13-Mar-15	20	1	16	2	2	1	0	3	2	1	1
14531	101	1	17-Sep-15	17	1	16	2	1	1	0	3	2	3	2
14532	101	1	25-Apr-15	14	1	16	2	1	1	0	3	2	1	1
14533	101	1	12-Mar-15	16	1	16	2	1	1	0	3	2	3	1
14534	101	1	18-May-15	17	1	16	2	1	1	0	3	2	3	1
14535	201	1	30-Sep-15	18	1	16	2	1	1	0	3	2	3	1
14536	201	1	17-Oct-15	19	1	16	2	1	1	0	3	2	2	1
14537	101	1	21-Apr-15	16	1	16	2	1	1	0	3	2	3	1
14538	101	1	30-Jul-15	17	1	16	2	1	1	0	3	2	3	1
14539	101	1	19-Jun-15	19	1	16	2	1	1	0	3	2	2	1
14540	201	1	30-May-15	1	1	16	2	3	1	0	3	2	3	1
14541	101	1	20-Apr-15	17	1	16	2	1	1	0	3	2	3	4
14542	201	1	2-Nov-15	19	1	16	2	1	1	0	3	2	2	1
14543	101	1	15-May-15	11	1	16	2	1	1	0	3	2	3	1
14544	101	1	22-Apr-15	18	1	16	2	1	1	0	3	2	3	1
14545	101	1	12-Jun-15	18	1	16	2	1	1	0	3	2	3	1
14546	101	1	18-Mar-15	11	1	16	2	1	1	0	3	2	3	1
14547	101	1	4-May-15	17	1	16	2	1	1	0	3	2	3	1
14548	101	1	2-Dec-15	17	1	16	2	1	1	0	3	2	2	1
14549	101	1	4-May-15	17	1	16	2	1	1	0	3	2	3	1
14550	201	1	30-Aug-15	14	1	16	2	1	1	0	3	2	2	1
14551	101	1	22-Apr-15	16	1	16	2	1	1	0	3	2	3	1
14552	101	1	2-Apr-15	17	1	16	2	1	1	0	3	2	3	1
14553	201	1	17-Nov-15	19	1	16	2	2	1	0	3	2	4	1
14554	201	1	20-May-15	12	1	16	2	2	1	0	3	2	1	1
14555	201	1	16-Oct-15	15	1	16	2	2	1	0	3	2	3	2
14556	101	1	26-May-15	14	1	16	2	1	1	0	3	2	2	1
14557	201	1	23-Sep-15	18	1	16	2	1	1	0	3	2	2	1
14558	201	1	11-Nov-15	17	1	16	2	1	1	0	3	2	3	1
14559	201	1	4-Dec-15	10	1	16	2	2	1	0	3	2	1	1
14560	201	1	28-Sep-15	11	1	16	2	1	1	0	3	2	4	2
14561	201	1	18-Apr-15	18	1	16	2	1	1	0	3	2	3	1
14562	101	1	23-May-14	21	1	16	2	1	1	0	3	2	1	1
14563	201	1	15-Jul-14	9	1	16	2	1	1	0	3	2	1	1
14564	101	1	14-May-14	23	1	16	2	1	1	0	3	2	1	1
14565	101	1	7-Mar-14	8	1	16	2	1	1	0	3	2	1	1
14566	101	1	8-Jul-14	17	1	16	2	1	1	0	3	2	3	1
14567	101	1	11-Dec-14	15	1	16	2	1	1	0	3	2	1	1
14568	101	1	8-Jan-14	20	1	16	2	1	1	0	3	2	1	1
14569	201	1	21-Nov-14	17	1	16	2	2	1	0	3	2	3	1
14570	101	1	15-Jan-14	10	1	16	2	1	1	0	3	2	3	1
14571	201	1	15-Apr-14	8	1	16	2	1	1	0	3	2	3	1
14572	101	1	21-Feb-14	13	1	16	2	1	1	0	3	2	1	1
14573	101	1	4-Sep-14	18	1	16	2	1	1	0	3	2	1	1
14574	201	1	21-Jan-14	21	1	16	2	3	1	0	3	2	5	1
14575	101	1	27-Feb-14	16	1	16	2	1	1	0	3	2	2	1
14576	101	1	30-Oct-14	19	1	16	2	1	1	0	3	2	1	1
14577	101	1	14-Jul-14	16	1	16	2	1	1	0	3	2	1	1
14578	101	1	20-Mar-14	11	1	16	2	1	1	0	3	2	3	1
14579	101	1	8-Nov-14	19	1	16	2	1	1	0	3	2	3	1
14580	201	1	5-Oct-14	9	1	16	2	1	1	0	3	2	2	1
14581	201	1	12-May-14	17	1	16	2	2	1	0	3	2	2	1
14582	101	1	17-Jun-14	18	1	16	2	1	1	0	3	2	3	1
14583	101	1	17-Oct-14	19	1	16	2	1	1	0	3	2	3	1
14584	101	1	7-Dec-14	9	1	16	2	1	1	0	3	2	1	2
14585	201	1	2-Feb-14	18	1	16	2	3	1	0	3	2	3	1
14586	101	1	10-Jul-14	19	1	16	2	1	1	0	3	2	3	1
14587	201	1	9-Feb-14	10	1	16	2	3	1	0	3	2	3	1
14588	201	1	26-Nov-14	10	1	16	2	2	1	0	3	2	3	2
14589	201	1	5-Sep-14	22	1	16	2	2	1	0	3	2	3	1
14590	101	1	6-Jan-14	8	1	16	2	1	1	0	3	2	3	1
14591	101	1	19-Apr-14	20	1	16	2	1	1	0	3	2	1	1
14592	201	1	22-May-14	16	1	16	2	1	1	0	3	2	1	1
14593	201	1	27-Apr-14	16	1	16	2	2	1	0	3	2	1	1
14594	101	1	14-Nov-14	13	1	16	2	1	1	0	3	2	3	1
14595	101	1	17-Apr-14	16	1	16	2	1	1	0	3	2	3	1
14596	101	1	9-Sep-14	16	1	16	2	1	1	0	3	2	3	1
14597	101	1	29-Oct-14	18	1	16	2	1	1	0	3	2	3	1
14598	101	1	16-Dec-14	18	1	16	2	1	1	0	3	2	3	1
14599	101	1	3-Apr-14	19	1	16	2	1	1	0	3	2	2	1
14600	201	1	3-Dec-14	19	1	16	2	1	1	0	3	2	3	1
14601	101	1	14-May-14	12	1	16	2	1	1	0	3	2	3	1
14602	101	1	9-May-14	15	1	16	2	1	1	0	3	2	2	1
14603	101	1	14-Mar-14	16	1	16	2	1	1	0	3	2	3	1
14604	101	1	26-Feb-14	13	1	16	2	1	1	0	3	2	3	1
14605	101	1	8-Oct-14	18	1	16	2	1	1	0	3	2	3	1
14606	101	1	9-Dec-14	19	1	16	2	1	1	0	3	2	3	1
14607	201	1	17-Apr-14	15	1	16	2	3	1	0	3	2	2	1
14608	201	1	9-Apr-14	10	1	16	2	3	1	0	3	2	4	1
14609	101	1	16-Oct-14	20	1	16	2	1	1	0	3	2	4	1
14610	201	1	21-Dec-14	14	1	16	2	2	1	0	3	2	5	1
14611	201	1	4-Mar-14	14	1	16	2	1	1	0	3	2	1	2
14612	201	1	7-Mar-14	14	1	16	2	2	1	0	3	2	2	1
14613	101	1	22-May-14	18	1	16	2	1	1	0	3	2	3	1
14614	101	1	25-Mar-14	17	1	16	2	1	1	0	3	2	3	1
14615	201	1	16-Apr-14	14	1	16	2	2	1	0	3	2	2	1
14616	101	1	3-Mar-14	17	1	16	2	1	1	0	3	2	2	2
14617	201	1	26-Nov-14	14	1	16	2	2	1	0	3	2	3	1
14618	101	1	20-Nov-14	10	1	16	2	1	1	0	3	2	3	1
14619	101	1	21-Apr-14	17	1	16	2	1	1	0	3	2	3	1
14620	201	1	7-Mar-14	13	1	16	2	1	1	0	3	2	4	1
14621	201	1	4-Jan-14	11	1	16	2	1	1	0	3	2	2	2
14622	201	1	29-Oct-14	14	1	16	2	1	1	0	3	2	2	1
14623	201	1	26-Oct-14	9	1	16	2	2	1	0	3	2	3	1
14624	201	1	17-Dec-14	13	1	16	2	1	1	0	3	2	3	1
14625	201	1	22-Feb-13	20	1	16	2	2	1	0	3	2	1	1

14626	201	1	5-Oct-13	18	1	16	2	1	1	0	3	2	2	1
14627	101	1	22-Apr-13	9	1	16	2	1	1	0	3	2	3	1
14628	201	1	17-Dec-13	10	1	16	2	1	1	0	3	2	4	1
14629	101	1	14-May-13	19	1	16	2	2	1	0	3	2	2	4
14630	201	1	23-Oct-13	8	1	16	2	2	1	0	3	2	3	1
14631	101	1	26-Jun-13	16	1	16	2	1	1	0	3	2	3	1
14632	101	1	24-Jan-13	13	1	16	2	1	1	0	3	2	1	1
14633	101	1	20-Feb-13	20	1	16	2	1	1	0	3	2	5	1
14634	101	1	21-Mar-13	22	1	16	2	1	1	0	3	2	5	1
14635	101	1	22-Mar-13	10	1	16	2	1	1	0	3	2	5	2
14636	101	1	31-Oct-13	20	1	16	2	1	1	0	3	2	1	1
14637	101	1	1-Jun-13	11	1	16	2	1	1	0	3	2	2	4
14638	101	1	4-Mar-13	12	1	16	2	1	1	0	3	2	3	1
14639	101	1	12-Mar-13	12	1	16	2	1	1	0	3	2	3	4
14640	201	1	13-Aug-13	21	1	16	2	2	1	0	3	2	1	1
14641	101	1	28-Apr-13	12	1	16	2	1	1	0	3	2	5	1
14642	101	1	21-Jul-13	21	1	16	2	1	1	0	3	2	2	1
14643	101	1	20-Sep-13	22	1	16	2	1	1	0	3	2	3	1
14644	101	1	24-Mar-13	16	1	16	2	1	1	0	3	2	3	1
14645	101	1	10-Oct-13	18	1	16	2	1	1	0	3	2	2	2
14646	101	1	18-Nov-13	20	1	16	2	1	1	0	3	2	3	1
14647	101	1	28-May-13	17	1	16	2	1	1	0	3	2	3	1
14648	101	1	4-Jul-13	20	1	16	2	1	1	0	3	2	3	1
14649	201	1	15-Dec-13	9	1	16	2	1	1	0	3	2	2	1
14650	201	1	31-Dec-13	12	1	16	2	2	1	0	3	2	2	1
14651	101	1	2-Jun-13	17	1	16	2	1	1	0	3	2	2	1
14652	201	1	15-Mar-13	21	1	16	2	3	1	0	3	2	2	1
14653	101	1	1-Jun-13	7	1	16	2	1	1	0	3	2	2	1
14654	101	1	4-Mar-13	13	1	16	2	1	1	0	3	2	3	1
14655	101	1	26-Mar-13	16	1	16	2	1	1	0	3	2	3	1
14656	101	1	29-Nov-13	20	1	16	2	1	1	0	3	2	1	1
14657	201	1	26-Aug-13	10	1	16	2	1	1	0	3	2	1	1
14658	101	1	28-Dec-13	11	1	16	2	1	1	0	3	2	4	1
14659	101	1	22-Oct-13	12	1	16	2	1	1	0	3	2	3	1
14660	101	1	5-Oct-13	10	1	16	2	1	1	0	3	2	3	1
14661	101	1	12-Sep-13	17	1	16	2	1	1	0	3	2	3	1
14662	101	1	16-Mar-13	14	1	16	2	1	1	0	3	2	3	1
14663	101	1	10-May-13	16	1	16	2	1	1	0	3	2	3	1
14664	101	1	21-Nov-13	21	1	16	2	3	1	0	3	2	2	1
14665	201	1	25-Aug-13	19	1	16	2	3	1	0	3	2	2	1
14666	101	1	17-Sep-13	19	1	16	2	3	1	0	3	2	2	4
14667	101	1	5-Jul-13	16	1	16	2	2	1	0	3	2	2	1
14668	101	1	20-Nov-13	17	1	16	2	1	1	0	3	2	3	1
14669	101	1	3-Dec-13	18	1	16	2	1	1	0	3	2	2	1
14670	201	1	30-Nov-13	20	1	16	2	2	1	0	3	2	2	1
14671	101	1	21-Nov-13	8	1	16	2	1	1	0	3	2	3	1
14672	201	1	30-Nov-13	19	1	16	2	3	1	0	3	2	3	1
14673	101	1	7-Mar-13	16	1	16	2	1	1	0	3	2	4	1
14674	101	1	14-Dec-13	21	1	16	2	1	1	0	3	2	3	1
14675	101	1	3-Dec-13	12	1	16	2	1	1	0	3	2	3	1
14676	101	1	5-May-13	17	1	16	2	1	1	0	3	2	3	1
14677	101	1	27-Sep-13	16	1	16	2	1	1	0	3	2	2	1
14678	101	1	26-Feb-13	16	1	16	2	1	1	0	3	2	3	4
14679	101	1	30-May-13	18	1	16	2	1	1	0	3	2	2	1
14680	101	1	19-Jun-13	19	1	16	2	1	1	0	3	2	2	1
14681	101	1	6-Nov-13	18	1	16	2	1	1	0	3	2	3	1
14682	101	1	30-Nov-13	20	1	16	2	1	1	0	3	2	3	1
14683	201	1	16-Jun-13	15	1	16	2	2	1	0	3	2	2	2
14684	101	1	21-Jul-13	17	1	16	2	1	1	0	3	2	1	1
14685	201	1	23-Oct-13	9	1	16	2	2	1	0	3	2	1	1
14686	201	1	13-Mar-13	16	1	16	2	1	1	0	3	2	1	1
14687	201	1	18-Oct-13	18	1	16	2	2	1	0	3	2	2	1
14688	101	1	1-Dec-13	16	1	16	2	1	1	0	3	2	3	1
14689	101	1	29-Nov-13	18	1	16	2	1	1	0	3	2	3	1
14690	201	1	18-Mar-13	14	1	16	2	2	1	0	3	2	3	1
14691	201	1	29-May-13	17	1	16	2	1	1	0	3	2	1	1
14692	201	1	18-Nov-13	15	1	16	2	2	1	0	3	2	3	1
14693	201	1	14-Mar-13	15	1	16	2	2	1	0	3	2	2	1
14694	201	1	2-Dec-13	17	1	16	2	2	1	0	3	2	2	1
14695	201	1	6-Aug-13	10	1	16	2	1	1	0	3	2	1	1
14696	101	1	5-May-12	21	1	16	2	1	1	0	3	2	3	1
14697	201	1	18-Oct-12	23	1	16	2	1	1	0	3	2	4	1
14698	101	1	14-Mar-12	13	1	16	2	1	1	0	3	2	1	1
14699	101	1	2-Nov-12	9	1	16	2	2	1	0	3	2	4	1
14700	201	1	26-Mar-12	10	1	16	2	3	1	0	3	2	3	2
14701	201	1	8-Sep-12	0	1	16	2	2	1	0	3	2	2	1
14702	201	1	5-Jun-12	21	1	16	2	2	1	0	3	2	2	1
14703	101	1	30-Nov-12	9	1	16	2	1	1	0	3	2	1	1
14704	101	1	29-Nov-12	0	1	16	2	3	1	0	3	2	3	1
14705	201	1	18-Dec-12	16	1	16	2	2	1	0	3	2	2	1
14706	201	1	17-Jul-12	4	1	16	2	2	1	0	3	2	3	1
14707	101	1	10-Apr-12	15	1	16	2	1	1	0	3	2	1	1
14708	101	1	8-Nov-12	15	1	16	2	1	1	0	3	2	3	1
14709	101	1	5-Jul-12	17	1	16	2	1	1	0	3	2	1	1
14710	101	1	6-Jun-12	18	1	16	2	1	1	0	3	2	5	1
14711	101	1	19-Apr-12	16	1	16	2	1	1	0	3	2	1	1
14712	101	1	16-Mar-12	22	1	16	2	1	1	0	3	2	1	1
14713	101	1	20-May-12	22	1	16	2	1	1	0	3	2	2	1
14714	101	1	14-Jul-12	23	1	16	2	1	1	0	3	2	2	1
14715	101	1	3-Nov-12	1	1	16	2	1	1	0	3	2	5	1
14716	101	1	24-Mar-12	8	1	16	2	1	1	0	3	2	3	1
14717	101	1	2-Dec-12	2	1	16	2	1	1	0	3	2	3	1
14718	101	1	15-Nov-12	12	1	16	2	1	1	0	3	2	3	1
14719	101	1	5-Nov-12	16	1	16	2	1	1	0	3	2	1	1
14720	101	1	26-Nov-12	12	1	16	2	1	1	0	3	2	3	1
14721	201	1	2-Oct-12	18	1	16	2	2	1	0	3	2	2	1
14722	101	1	9-May-12	18	1	16	2	1	1	0	3	2	2	1
14723	201	1	30-Sep-12	17	1	16	2	1	1	0	3	2	2	2
14724	101	1	25-May-12	19	1	16	2	1	1	0	3	2	3	1
14725	101	1	6-Dec-12	13	1	16	2	1	1	0	3	2	3	1
14726	101	1	26-Jun-12	4	1	16	2	1	1	0	3	2	3	1
14727	101	1	18-Jun-12	17	1	16	2	1	1	0	3	2	2	4
14728	201	1	13-Aug-12	18	1	16	2	2	1	0	3	2	1	1
14729	201	1	24-Nov-12	17	1	16	2	2	1	0	3	2	1	1
14730	101	1	7-Oct-12	10	1	16	2	1	1	0	3	2	4	1
14731	101	1	21-Sep-12	18	1	16	2	1	1	0	3	2	2	4
14732	101	1	6-Dec-12	17	1	16	2	1	1	0	3	2	3	1
14733	101	1	10-Feb-12	21	1	16	2	1	1	0	3	2	3	1
14734	201	1	7-Aug-12	8	1	16	2	2	1	0	3	2	3	1
14735	101	1	31-Mar-12	22	1	16	2	1	1	0	3	2	3	1
14736	101	1	19-Mar-12	17	1	16	2	1	1	0	3	2	2	1
14737	201	1	23-Dec-12	16	1	16	2	3	1	0	3	2	2	1
14738	101	1	15-Nov-12	16	1	16	2	1	1	0	3	2	3	1
14739	101	1	13-Dec-12	11	1	16	2	1	1	0	3	2	3	1
14740	101	1	28-Feb-12	17	1	16	2	1	1	0	3	2	3	1
14741	101	1	15-May-12	17	1	16	2	1	1	0	3	2	3	1
14742	101	1	5-Mar-12	10	1	16	2	1	1	0	3	2	3	1
14743	101	1	15-Nov-12	16	1	16	2	1	1	0	3	2	3	1
14744	101	1	20-Mar-12	17	1	16	2	1	1	0	3	2	3	1
14745	101	1	10-Apr-12	20	1	16	2	1	1	0	3	2	3	1
14746	101	1	22-Jun-12	16	1	16	2	1	1	0	3	2	3	1
14747	101	1	21-Nov-12	18	1	16	2	1	1	0	3	2	1	1
14748	101	1	4-Feb-12	17	1	16	2	1	1	0	3	2	2	4
14749	101	1	15-Nov-12	16	1	16	2	1	1	0	3	2	3	1

14750	201	1	11-Oct-12	10	1	16	2	2	1	0	3	2	4	1
14751	201	1	24-Oct-12	11	1	16	2	2	1	0	3	2	2	1
14752	201	1	12-Jan-12	9	1	16	2	2	1	0	3	2	1	1
14753	201	1	11-Oct-12	16	1	16	2	1	1	0	3	2	1	1
14754	201	1	16-Oct-12	18	1	16	2	1	1	0	3	2	1	1
14755	101	1	10-Jul-12	17	1	16	2	1	1	0	3	2	3	1
14756	201	1	28-Apr-12	14	1	16	2	3	1	0	3	2	3	1
14757	201	1	14-Dec-12	12	1	16	2	2	1	0	3	2	2	1
14758	101	1	29-Mar-12	10	1	16	2	1	1	0	3	2	3	1
14759	201	1	5-Dec-12	11	1	16	2	2	1	0	3	2	1	1
14760	201	1	5-Dec-12	11	1	16	2	2	1	0	3	2	1	1
14761	201	1	30-Dec-12	17	1	16	2	1	1	0	3	2	2	1
14762	101	1	13-Nov-12	16	1	16	2	1	1	0	3	2	2	1
14763	101	1	18-Mar-12	7	1	16	2	1	1	0	3	2	3	1
14764	201	1	3-Aug-12	18	1	16	2	1	1	0	3	2	3	1
14765	201	1	20-Apr-11	22	1	16	2	1	1	0	3	2	3	2
14766	101	1	15-Nov-11	13	1	16	2	1	1	0	3	2	4	1
14767	101	1	9-Dec-11	11	1	16	2	1	1	0	3	2	3	1
14768	101	1	26-Nov-11	16	1	16	2	1	1	0	3	2	3	1
14769	101	1	20-Apr-11	12	1	16	2	1	1	0	3	2	2	1
14770	201	1	24-Jan-11	22	1	16	2	1	1	0	3	2	2	1
14771	101	1	1-Jul-11	20	1	16	2	1	1	0	3	2	3	4
14772	201	1	22-Apr-11	18	1	16	2	1	1	0	3	2	1	2
14773	201	1	28-Nov-11	9	1	16	2	3	1	0	3	2	3	2
14774	101	1	24-Nov-11	12	1	16	2	1	1	0	3	2	2	1
14775	101	1	10-Dec-11	11	1	16	2	1	1	0	3	2	5	1
14776	101	1	18-Oct-11	19	1	16	2	2	1	0	3	2	3	1
14777	101	1	2-Dec-11	15	1	16	2	1	1	0	3	2	2	1
14778	101	1	17-Apr-11	10	1	16	2	1	1	0	3	2	3	1
14779	101	1	28-Feb-11	20	1	16	2	1	1	0	3	2	3	1
14780	101	1	16-Jun-11	17	1	16	2	1	1	0	3	2	2	1
14781	201	1	17-Apr-11	11	1	16	2	2	1	0	3	2	2	2
14782	101	1	5-May-11	17	1	16	2	1	1	0	3	2	1	1
14783	101	1	8-Jul-11	20	1	16	2	3	1	0	3	2	3	1
14784	201	1	24-Jul-11	2	1	16	2	1	1	0	3	2	3	1
14785	101	1	13-Jun-11	10	1	16	2	1	1	0	3	2	1	1
14786	101	1	24-Oct-11	18	1	16	2	1	1	0	3	2	1	1
14787	101	1	11-Apr-11	18	1	16	2	1	1	0	3	2	4	1
14788	101	1	31-Mar-11	18	1	16	2	1	1	0	3	2	3	4
14789	101	1	11-Aug-11	17	1	16	2	1	1	0	3	2	3	1
14790	101	1	28-Mar-11	17	1	16	2	2	1	0	3	2	3	1
14791	101	1	4-Mar-11	18	1	16	2	1	1	0	3	2	3	1
14792	101	1	12-Dec-11	18	1	16	2	1	1	0	3	2	4	1
14793	101	1	12-Aug-11	22	1	16	2	1	1	0	3	2	3	2
14794	101	1	8-Dec-11	17	1	16	2	1	1	0	3	2	2	1
14795	101	1	31-Mar-11	15	1	16	2	1	1	0	3	2	3	1
14796	101	1	21-Oct-11	18	1	16	2	1	1	0	3	2	3	1
14797	101	1	5-Aug-11	21	1	16	2	1	1	0	3	2	2	1
14798	101	1	23-Dec-11	17	1	16	2	1	1	0	3	2	3	4
14799	101	1	20-Dec-11	17	1	16	2	1	1	0	3	2	3	1
14800	201	1	22-Jan-11	16	1	16	2	1	1	0	3	2	1	1
14801	201	1	21-Feb-11	11	1	16	2	1	1	0	3	2	1	1
14802	101	1	15-Dec-11	12	1	16	2	1	1	0	3	2	3	2
14803	201	1	7-Dec-11	9	1	16	2	2	1	0	3	2	1	1
14804	101	1	25-Mar-11	20	1	16	2	1	1	0	3	2	3	1
14805	201	1	16-Sep-11	21	1	16	2	1	1	0	3	2	3	1
14806	101	1	15-Nov-11	16	1	16	2	1	1	0	3	2	3	1
14807	201	1	26-Dec-11	16	1	16	2	1	1	0	3	2	3	2
14808	101	1	16-Sep-11	10	1	16	2	1	1	0	3	2	1	1
14809	201	1	12-Aug-11	10	1	16	2	2	1	0	3	2	2	1
14810	201	1	10-May-11	17	1	16	2	1	1	0	3	2	2	1
14811	201	1	19-Jul-11	19	1	16	2	1	1	0	3	2	2	1
14812	201	1	27-Feb-11	13	1	16	2	1	1	0	3	2	2	1
14813	101	1	12-Oct-10	18	1	16	2	1	1	0	3	2	2	1
14814	201	1	18-Mar-10	16	1	16	2	1	1	0	3	2	6	1
14815	201	1	27-Mar-10	14	1	16	2	2	1	0	3	2	1	1
14816	101	1	6-Jun-10	10	1	16	2	1	1	0	3	2	4	2
14817	101	1	19-Nov-10	17	1	16	2	1	1	0	3	2	5	1
14818	201	1	14-May-10	18	1	16	2	1	1	0	3	2	5	1
14819	101	1	3-Mar-10	17	1	16	2	1	1	0	3	2	1	1
14820	201	1	11-May-10	11	1	16	2	2	1	0	3	2	3	1
14821	201	1	21-May-10	14	1	16	2	2	1	0	3	2	3	1
14822	101	1	11-May-10	12	1	16	2	1	1	0	3	2	3	1
14823	201	1	10-Nov-10	21	1	16	2	1	1	0	3	2	5	1
14824	201	1	6-Dec-10	18	1	16	2	2	1	0	3	2	1	1
14825	101	1	16-Sep-10	15	1	16	2	1	1	0	3	2	3	1
14826	101	1	18-Oct-10	18	1	16	2	1	1	0	3	2	2	1
14827	101	1	11-Mar-10	19	1	16	2	1	1	0	3	2	2	1
14828	101	1	9-Apr-10	16	1	16	2	1	1	0	3	2	4	1
14829	101	1	14-Jun-10	19	1	16	2	1	1	0	3	2	3	1
14830	101	1	2-Jan-10	22	1	16	2	1	1	0	3	2	3	1
14831	101	1	8-Jun-10	19	1	16	2	1	1	0	3	2	1	1
14832	101	1	9-Jul-10	14	1	16	2	1	1	0	3	2	3	1
14833	101	1	29-Nov-10	16	1	16	2	1	1	0	3	2	3	1
14834	101	1	19-Nov-10	19	1	16	2	1	1	0	3	2	3	1
14835	101	1	17-Nov-10	17	1	16	2	1	1	0	3	2	3	1
14836	101	1	9-Mar-10	16	1	16	2	1	1	0	3	2	3	1
14837	101	1	6-Dec-10	17	1	16	2	1	1	0	3	2	3	1
14838	101	1	5-Mar-10	17	1	16	2	1	1	0	3	2	2	1
14839	101	1	8-Oct-10	17	1	16	2	1	1	0	3	2	2	1
14840	101	1	21-Sep-10	10	1	16	2	3	1	0	3	2	3	1
14841	101	1	17-May-10	12	1	16	2	1	1	0	3	2	7	1
14842	201	1	17-Jul-10	21	1	16	2	3	1	0	3	2	1	1
14843	201	1	15-Nov-10	16	1	16	2	2	1	0	3	2	3	1
14844	201	1	21-Aug-10	13	1	16	2	3	1	0	3	2	2	1
14845	101	1	2-Nov-10	10	1	16	2	1	1	0	3	2	3	1
14846	101	1	18-Dec-10	9	1	16	2	1	1	0	3	2	3	1
14847	201	1	15-May-10	16	1	16	2	1	1	0	3	2	1	1
14848	101	1	6-Jul-10	17	1	16	2	1	1	0	3	2	1	1
14849	101	1	25-Dec-09	10	1	16	2	1	1	0	3	2	5	1
14850	201	1	17-Sep-09	18	1	16	2	2	1	0	3	2	6	1
14851	101	1	19-Feb-09	17	1	16	2	1	1	0	3	2	1	1
14852	101	1	14-Oct-09	16	1	16	2	1	1	0	3	2	3	2
14853	101	1	19-Feb-09	16	1	16	2	1	1	0	3	2	3	1
14854	201	1	25-Jun-09	12	1	16	2	2	1	0	3	2	3	1
14855	101	1	31-Mar-09	20	1	16	2	1	1	0	3	2	1	1
14856	101	1	23-Mar-09	17	1	16	2	1	1	0	3	2	2	1
14857	101	1	17-Mar-09	21	1	16	2	1	1	0	3	2	2	2
14858	101	1	16-Apr-09	17	1	16	2	1	1	0	3	2	3	1
14859	101	1	28-Oct-09	19	1	16	2	1	1	0	3	2	1	1
14860	101	1	12-Sep-09	8	1	16	2	1	1	0	3	2	1	1
14861	101	1	1-Nov-09	19	1	16	2	1	1	0	3	2	3	1
14862	101	1	26-Nov-09	16	1	16	2	1	1	0	3	2	2	1
14863	101	1	24-Apr-09	10	1	16	2	1	1	0	3	2	3	1
14864	101	1	26-Oct-09	8	1	16	2	1	1	0	3	2	2	1
14865	101	1	9-Jul-09	22	1	16	2	1	1	0	3	2	2	1
14866	101	1	9-Dec-09	15	1	16	2	4	1	0	3	2	3	1
14867	101	1	10-Feb-09	17	1	16	2	1	1	0	3	2	3	1
14868	101	1	18-Feb-09	17	1	16	2	1	1	0	3	2	3	1
14869	101	1	16-Apr-09	17	1	16	2	2	1	0	3	2	3	1
14870	101	1	20-Apr-09	11	1	16	2	2	1	0	3	2	2	4
14871	101	1	22-Jul-09	16	1	16	2	1	1	0	3	2	4	2
14872	101	1	26-Nov-09	21	1	16	2	1	1	0	3	2	2	1
14873	201	1	28-Jun-09	17	1	16	2	2	1	0	3	2	3	1

14874	201	1	13-Nov-09	10	1	16	2	1	1	0	3	2	5	1
14875	201	1	5-Jul-09	8	1	16	2	1	1	0	3	2	3	1
14876	201	1	25-Sep-09	12	1	16	2	1	1	0	3	2	1	1
14877	101	1	28-Apr-09	17	1	16	2	2	1	0	3	2	4	4
14878	101	1	7-May-09	18	1	16	2	1	1	0	3	2	3	4
14879	201	1	20-Mar-09	18	1	16	2	2	1	0	3	2	2	1
14880	101	1	22-May-09	12	1	16	2	2	1	0	3	2	2	4
14881	201	1	23-Nov-08	18	1	16	2	1	1	0	3	2	2	1
14882	201	1	21-Feb-08	18	1	16	2	1	1	0	3	2	2	1
14883	101	1	12-Nov-08	18	1	16	2	1	1	0	3	2	1	1
14884	201	1	16-May-08	19	1	16	2	1	1	0	3	2	2	2
14885	201	1	26-Nov-08	11	1	16	2	1	1	0	3	2	3	1
14886	101	1	4-Nov-08	10	1	16	2	1	1	0	3	2	1	1
14887	101	1	23-Jan-08	10	1	16	2	1	1	0	3	2	1	1
14888	101	1	12-Dec-08	12	1	16	2	1	1	0	3	2	1	1
14889	201	1	13-Nov-08	12	1	16	2	3	1	0	3	2	2	1
14890	201	1	10-Sep-08	17	1	16	2	1	1	0	3	2	2	1
14891	101	1	20-Apr-08	10	1	16	2	1	1	0	3	2	2	1
14892	201	1	13-Dec-08	8	1	16	2	3	1	0	3	2	1	1
14893	101	1	19-Jun-08	16	1	16	2	1	1	0	3	2	1	1
14894	201	1	31-Dec-08	12	1	16	2	2	1	0	3	2	4	1
14895	101	1	23-May-08	18	1	16	2	1	1	0	3	2	3	1
14896	101	1	26-Dec-08	17	1	16	2	1	1	0	3	2	5	1
14897	101	1	16-Apr-08	17	1	16	2	1	1	0	3	2	2	2
14898	101	1	10-Jun-08	11	1	16	2	1	1	0	3	2	3	1
14899	201	1	7-Nov-08	18	1	16	2	1	1	0	3	2	3	1
14900	101	1	27-May-08	19	1	16	2	1	1	0	3	2	2	1
14901	101	1	19-Oct-08	11	1	16	2	1	1	0	3	2	3	1
14902	101	1	12-Dec-08	13	1	16	2	1	1	0	3	2	3	1
14903	101	1	18-Nov-08	17	1	16	2	1	1	0	3	2	3	1
14904	101	1	10-Jan-08	18	1	16	2	1	1	0	3	2	3	1
14905	101	1	28-Feb-08	18	1	16	2	1	1	0	3	2	3	1
14906	101	1	1-Mar-08	22	1	16	2	1	1	0	3	2	2	1
14907	201	1	7-Dec-08	12	1	16	2	1	1	0	3	2	6	1
14908	201	1	28-Nov-08	8	1	16	2	2	1	0	3	2	6	1
14909	201	1	27-Jul-08	16	1	16	2	1	1	0	3	2	4	1
14910	101	1	23-May-08	10	1	16	2	1	1	0	3	2	2	1
14911	101	1	14-Oct-08	17	1	16	2	1	1	0	3	2	3	1
14912	101	1	10-Sep-08	17	1	16	2	1	1	0	3	2	3	1
14913	101	1	20-Feb-08	18	1	16	2	1	1	0	3	2	3	4
14914	201	1	5-Aug-08	19	1	16	2	3	1	0	3	2	3	1
14915	201	1	24-Dec-08	17	1	16	2	1	1	0	3	2	1	1
14916	201	1	28-Nov-08	18	1	16	2	2	1	0	3	2	3	2
14917	201	1	12-Dec-08	17	1	16	2	1	1	0	3	2	3	1
14918	101	1	26-Mar-08	15	1	16	2	1	1	0	3	2	2	1
14919	101	1	21-May-08	10	1	16	2	1	1	0	3	2	1	1
14920	101	1	12-May-08	17	1	16	2	1	1	0	3	2	2	1
14921	201	1	29-Oct-07	4	1	16	2	1	1	0	3	2	4	2
14922	101	1	11-Oct-07	15	1	16	2	1	1	0	3	2	3	2
14923	101	1	26-Mar-07	18	1	16	2	1	1	0	3	2	4	1
14924	101	1	20-Jan-07	9	1	16	2	1	1	0	3	2	1	1
14925	201	1	2-Jul-07	17	1	16	2	1	1	0	3	2	1	1
14926	101	1	8-Oct-07	8	1	16	2	1	1	0	3	2	1	1
14927	201	1	20-Jan-07	16	1	16	2	1	1	0	3	2	2	2
14928	201	1	18-Sep-07	23	1	16	2	1	1	0	3	2	1	1
14929	101	1	25-Apr-07	13	1	16	2	1	1	0	3	2	4	1
14930	101	1	23-Nov-07	13	1	16	2	1	1	0	3	2	3	1
14931	101	1	19-Apr-07	20	1	16	2	1	1	0	3	2	3	1
14932	201	1	4-Apr-07	20	1	16	2	2	1	0	3	2	3	1
14933	201	1	15-Jul-07	21	1	16	2	2	1	0	3	2	1	1
14934	101	1	27-Sep-07	11	1	16	2	1	1	0	3	2	7	1
14935	201	1	23-Oct-07	21	1	16	2	1	1	0	3	2	1	1
14936	101	1	14-Sep-07	14	1	16	2	1	1	0	3	2	1	1
14937	201	1	16-Jun-07	23	1	16	2	1	1	0	3	2	1	1
14938	201	1	19-Jan-07	12	1	16	2	2	1	0	3	2	4	1
14939	201	1	31-Mar-07	18	1	16	2	3	1	0	3	2	2	1
14940	101	1	6-Apr-07	12	1	16	2	1	1	0	3	2	2	1
14941	101	1	9-Jul-07	18	1	16	2	1	1	0	3	2	2	1
14942	101	1	13-Dec-07	19	1	16	2	1	1	0	3	2	3	1
14943	101	1	19-Apr-07	15	1	16	2	1	1	0	3	2	3	1
14944	101	1	19-Aug-07	10	1	16	2	1	1	0	3	2	3	2
14945	201	1	5-Nov-07	16	1	16	2	3	1	0	3	2	2	1
14946	201	1	1-Mar-07	11	1	16	2	1	1	0	3	2	2	1
14947	201	1	21-Apr-07	16	1	16	2	1	1	0	3	2	2	1
14948	201	1	19-Sep-07	11	1	16	2	1	1	0	3	2	3	1
14949	101	1	15-Oct-07	22	1	16	2	1	1	0	3	2	2	1
14950	201	1	26-Apr-07	23	1	16	2	3	1	0	3	2	3	1
14951	201	1	24-Sep-06	15	1	16	2	1	1	0	3	2	1	1
14952	201	1	3-Mar-06	18	1	16	2	3	1	0	3	2	5	1
14953	101	1	29-Sep-06	8	1	16	2	1	1	0	3	2	1	1
14954	201	1	2-Nov-06	18	1	16	2	1	1	0	3	2	1	1
14955	101	1	7-Mar-06	9	1	16	2	1	1	0	3	2	1	1
14956	101	1	8-Nov-06	18	1	16	2	1	1	0	3	2	1	1
14957	101	1	2-Jun-06	16	1	16	2	3	1	0	3	2	1	1
14958	101	1	23-May-06	20	1	16	2	1	1	0	3	2	4	1
14959	201	1	21-Jul-06	19	1	16	2	1	1	0	3	2	1	1
14960	201	1	19-Oct-06	22	1	16	2	1	1	0	3	2	1	1
14961	101	1	18-Oct-06	7	1	16	2	1	1	0	3	2	3	1
14962	201	1	27-Nov-06	15	1	16	2	3	1	0	3	2	6	1
14963	101	1	6-Aug-06	11	1	16	2	1	1	0	3	2	1	1
14964	101	1	15-Dec-06	17	1	16	2	1	1	0	3	2	3	1
14965	101	1	18-Feb-06	18	1	16	2	1	1	0	3	2	2	1
14966	101	1	22-Mar-06	16	1	16	2	1	1	0	3	2	2	1
14967	201	1	21-Mar-06	14	1	16	2	3	1	0	3	2	4	1
14968	201	1	22-Feb-06	18	1	16	2	2	1	0	3	2	3	1
14969	101	1	3-Mar-06	17	1	16	2	1	1	0	3	2	3	1
14970	201	1	22-Mar-06	19	1	16	2	2	1	0	3	2	4	1
14971	101	1	3-Jan-06	20	1	16	2	1	1	0	3	2	2	1
14972	101	1	20-Dec-06	16	1	16	2	1	1	0	3	2	2	1
14973	101	1	19-May-06	17	1	16	2	1	1	0	3	2	3	1
14974	201	1	28-Dec-06	12	1	16	2	1	1	0	3	2	2	1
14975	201	1	17-Sep-06	19	1	16	2	1	1	0	3	2	4	1
14976	101	1	26-May-06	17	1	16	2	1	1	0	3	2	3	1
14977	101	1	22-Sep-06	14	1	16	2	3	1	0	3	2	1	1
14978	101	1	26-Dec-06	17	1	16	2	1	1	0	3	2	3	1
14979	101	1	22-Dec-06	16	1	16	2	1	1	0	3	2	3	1
14980	101	1	17-Jun-06	21	1	16	2	1	1	0	3	2	2	1
14981	201	1	15-Jun-17	21	1	16	2	1	1	0	3	2	4	1
14982	101	1	17-Apr-17	19	1	16	2	1	1	0	3	2	3	1
14983	101	1	21-Mar-17	16	1	16	2	1	1	0	3	2	3	1
14984	101	1	23-Feb-17	19	1	16	2	1	1	0	3	2	3	1
14985	101	1	3-May-17	15	1	16	2	1	1	0	3	2	3	1
14986	101	1	14-Jul-17	18	1	16	2	1	1	0	3	2	3	1
14987	201	1	16-Nov-17	19	1	16	2	1	1	0	3	2	3	1
14988	201	1	20-Feb-17	17	1	16	2	1	1	0	3	2	3	1
14989	101	1	20-Mar-17	14	1	16	2	1	1	0	3	2	2	1
14990	101	1	24-Feb-17	13	1	16	2	1	1	0	3	2	2	1
14991	101	1	23-Mar-17	20	1	16	2	1	1	0	3	2	2	1
14992	101	1	27-Apr-17	16	1	16	2	1	1	0	3	2	2	1
14993	101	1	18-Feb-17	12	1	16	2	1	1	0	3	2	2	1
14994	101	1	23-Aug-17	20	1	16	2	1	1	0	3	2	2	1
14995	101	1	9-Mar-17	16	1	16	2	1	1	0	3	2	2	1
14996	101	1	5-Apr-17	18	1	16	2	1	1	0	3	2	2	1
14997	101	1	25-Jan-17	15	1	16	2	1	1	0	3	2	2	1

14998	201	1	27-Sep-17	9	1	16	2	1	1	0	3	2	2	1
14999	201	1	7-May-17	11	1	16	2	1	1	0	3	2	2	1
15000	101	1	7-Jun-17	21	1	16	2	1	1	0	3	2	1	1
15001	101	1	17-Apr-17	16	1	16	2	1	1	0	3	2	1	1
15002	101	1	1-Apr-17	18	1	16	2	1	1	0	3	2	1	1
15003	101	1	30-Oct-17	20	1	16	2	1	1	0	3	2	4	1
15004	101	1	21-Dec-17	12	1	16	2	1	1	0	3	2	4	1
15005	101	1	31-Oct-17	19	1	16	2	1	1	0	3	2	3	1
15006	101	1	27-Oct-17	17	1	16	2	1	1	0	3	2	3	1
15007	101	1	28-Aug-17	12	1	16	2	1	1	0	3	2	3	1
15008	101	1	13-Oct-17	10	1	16	2	1	1	0	3	2	2	1
15009	101	1	22-Dec-17	18	1	16	2	1	1	0	3	2	2	1
15010	101	1	10-Oct-17	21	1	16	2	1	1	0	3	2	2	1
15011	101	1	14-Oct-17	15	1	16	2	1	1	0	3	2	3	2
15012	101	1	23-Sep-17	16	1	16	2	1	1	0	3	2	2	2
15013	101	1	28-Nov-17	16	1	16	2	1	1	0	3	2	2	2
15014	101	1	10-Jun-17	10	1	16	2	1	1	0	3	2	7	1
15015	101	1	31-Oct-17	10	1	16	2	1	1	0	3	2	5	1
15016	101	1	21-Apr-17	20	1	16	2	1	1	0	3	2	5	1
15017	101	1	5-Dec-17	20	1	16	2	1	1	0	3	2	4	1
15018	101	1	24-Apr-17	23	1	16	2	1	1	0	3	2	3	1
15019	101	1	20-Apr-17	8	1	16	2	1	1	0	3	2	3	1
15020	201	1	18-Apr-17	9	1	16	2	1	1	0	3	2	3	1
15021	201	1	27-Nov-17	18	1	16	2	1	1	0	3	2	3	1
15022	101	1	6-May-17	18	1	16	2	1	1	0	3	2	2	1
15023	101	1	11-Nov-17	10	1	16	2	1	1	0	3	2	1	1
15024	201	1	30-Nov-17	9	1	16	2	1	1	0	3	2	1	1
15025	201	1	2-Apr-17	17	1	16	2	2	1	0	3	2	3	1
15026	201	1	21-Apr-17	16	1	16	2	2	1	0	3	2	3	1
15027	201	1	14-Nov-17	11	1	16	2	2	1	0	3	2	1	1
15028	101	1	13-Oct-17	3	1	16	2	1	1	0	3	2	2	1
15029	101	1	18-Dec-16	22	1	16	2	1	1	0	6	2	2	1
15030	101	1	8-Apr-16	13	1	16	2	1	1	0	6	2	2	1
15031	101	1	10-Jun-16	12	1	16	2	1	1	0	6	2	2	1
15032	101	1	15-May-16	23	1	16	2	1	1	0	6	2	2	4
15033	101	1	24-Oct-15	15	1	16	2	1	1	0	6	2	2	1
15034	101	1	12-May-15	19	1	16	2	1	1	0	6	2	3	1
15035	101	1	20-Jun-15	2	1	16	2	1	1	0	6	2	2	1
15036	101	1	26-Jan-15	16	1	16	2	1	1	0	6	2	1	1
15037	101	1	28-Nov-14	11	1	16	2	1	1	0	6	2	2	1
15038	101	1	9-Jun-14	19	1	16	2	1	1	0	6	2	2	1
15039	201	1	7-Oct-14	19	1	16	2	2	1	0	6	2	3	1
15040	101	1	11-Aug-14	7	1	16	2	1	1	0	6	2	2	1
15041	101	1	31-Oct-13	12	1	16	2	1	1	0	6	2	2	1
15042	101	1	11-Aug-13	19	1	16	2	1	1	0	6	2	3	1
15043	101	1	27-Mar-13	8	1	16	2	1	1	0	6	2	2	1
15044	101	1	24-Feb-13	22	1	16	2	1	1	0	6	2	2	1
15045	201	1	26-Dec-13	12	1	16	2	1	1	0	6	2	2	1
15046	101	1	28-Nov-13	17	1	16	2	1	1	0	6	2	2	1
15047	101	1	24-May-13	16	1	16	2	1	1	0	6	2	2	1
15048	101	1	19-Jun-12	16	1	16	2	1	1	0	6	2	2	2
15049	101	1	7-Mar-12	9	1	16	2	1	1	0	6	2	2	1
15050	101	1	5-Sep-12	22	1	16	2	1	1	0	6	2	2	1
15051	101	1	6-Jul-12	14	1	16	2	1	1	0	6	2	1	1
15052	101	1	13-Jan-12	9	1	16	2	1	1	0	6	2	2	1
15053	101	1	1-Sep-12	17	1	16	2	1	1	0	6	2	2	1
15054	101	1	27-Jun-11	9	1	16	2	1	1	0	6	2	2	1
15055	101	1	17-Nov-11	9	1	16	2	1	1	0	6	2	2	1
15056	101	1	1-Mar-11	17	1	16	2	1	1	0	6	2	2	1
15057	101	1	26-Nov-11	16	1	16	2	1	1	0	6	2	2	1
15058	101	1	9-Jul-11	17	1	16	2	1	1	0	6	2	3	1
15059	101	1	10-Jun-11	10	1	16	2	1	1	0	6	2	2	1
15060	201	1	9-Jan-11	16	1	16	2	1	1	0	6	2	3	1
15061	201	1	7-Dec-11	19	1	16	2	1	1	0	6	2	3	2
15062	201	1	2-May-11	16	1	16	2	1	1	0	6	2	2	1
15063	201	1	9-Nov-10	17	1	16	2	1	1	0	6	2	2	1
15064	101	1	8-Mar-10	11	1	16	2	1	1	0	6	2	2	1
15065	101	1	14-Mar-10	14	1	16	2	1	1	0	6	2	2	1
15066	101	1	28-Apr-10	17	1	16	2	1	1	0	6	2	2	1
15067	101	1	22-Apr-10	17	1	16	2	1	1	0	6	2	2	1
15068	101	1	1-Sep-10	20	1	16	2	1	1	0	6	2	2	1
15069	101	1	4-Jan-10	16	1	16	2	1	1	0	6	2	3	1
15070	101	1	9-Nov-10	16	1	16	2	1	1	0	6	2	2	1
15071	101	1	27-Apr-10	17	1	16	2	1	1	0	6	2	2	1
15072	101	1	17-Sep-10	2	1	16	2	3	1	0	6	2	2	1
15073	201	1	26-Jul-10	11	1	16	2	2	1	0	6	2	2	1
15074	101	1	19-Nov-10	9	1	16	2	1	1	0	6	2	2	1
15075	201	1	13-Oct-09	7	1	16	2	1	1	0	6	2	2	1
15076	101	1	14-Apr-09	19	1	16	2	1	1	0	6	2	1	1
15077	201	1	27-Sep-09	16	1	16	2	2	1	0	6	2	2	1
15078	101	1	22-Oct-09	16	1	16	2	1	1	0	6	2	2	1
15079	101	1	12-Oct-09	13	1	16	2	1	1	0	6	2	2	1
15080	201	1	28-Jul-09	14	1	16	2	3	1	0	6	2	2	1
15081	101	1	29-Apr-08	18	1	16	2	1	1	0	6	2	2	1
15082	201	1	27-Jul-08	12	1	16	2	2	1	0	6	2	2	2
15083	101	1	8-Oct-08	16	1	16	2	1	1	0	6	2	2	1
15084	101	1	11-Dec-08	10	1	16	2	1	1	0	6	2	2	1
15085	101	1	21-Apr-08	18	1	16	2	2	1	0	6	2	2	1
15086	101	1	7-Jan-07	22	1	16	2	1	1	0	6	2	1	1
15087	101	1	22-Dec-07	14	1	16	2	1	1	0	6	2	2	1
15088	201	1	21-Sep-07	9	1	16	2	1	1	0	6	2	2	1
15089	101	1	29-May-07	17	1	16	2	1	1	0	6	2	2	1
15090	101	1	1-Nov-06	21	1	16	2	1	1	0	6	2	2	1
15091	101	1	13-Mar-06	17	1	16	2	1	1	0	6	2	2	1
15092	101	1	26-Nov-06	23	1	16	2	1	1	0	6	2	2	1
15093	201	1	9-Jan-06	17	1	16	2	1	1	0	6	2	2	1
15094	101	1	30-Nov-06	9	1	16	2	1	1	0	6	2	3	1
15095	101	1	2-Feb-06	12	1	16	2	2	1	0	6	2	3	1
15096	101	1	27-Mar-17	13	1	16	2	1	1	0	6	2	3	1
15097	101	1	5-Jul-17	19	1	16	2	1	1	0	6	2	2	1
15098	101	1	27-Mar-17	18	1	16	2	1	1	0	6	2	2	1
15099	101	1	31-Oct-17	10	1	16	2	1	1	0	6	2	3	1
15100	101	1	9-Mar-16	16	1	16	2	1	1	0	13	2	3	1
15101	101	1	2-Mar-11	16	1	16	2	1	1	0	13	2	4	1
15102	201	1	18-Jul-07	20	1	16	2	3	1	0	13	2	1	1
15103	101	1	24-Oct-06	9	1	16	2	1	1	0	13	2	3	1
15104	201	1	19-Jun-14	1	2	16	2	3	1	0	1	2	5	1
15105	101	1	7-Mar-13	17	2	16	2	1	1	0	4	2	1	1
15106	101	1	20-Jan-17	20	2	16	2	1	1	0	3	2	3	1
15107	101	1	24-Mar-15	20	2	16	2	1	1	0	6	2	2	1
15108	201	1	9-Sep-11	11	2	16	2	1	1	0	13	2	2	1
15109	201	1	20-Jun-10	18	2	16	2	3	1	0	13	2	3	1
15110	101	1	9-Mar-13	14	2	16	2	1	1	0	1	2	3	1
15111	201	1	30-Dec-10	19	2	16	2	3	1	0	1	2	1	1
15112	101	1	7-Jul-14	17	2	16	2	1	1	0	4	2	1	1
15113	101	1	18-Jun-14	10	2	16	2	1	1	0	4	2	1	1
15114	101	1	10-Jul-14	23	2	16	2	3	1	0	4	2	2	1
15115	201	1	23-Feb-14	14	2	16	2	1	1	0	4	2	2	1
15116	201	1	7-Sep-13	21	2	16	2	3	1	0	4	2	5	1
15117	101	1	30-Jan-13	15	2	16	2	4	1	0	4	2	2	1
15118	101	1	29-Oct-13	17	2	16	2	1	1	0	4	2	2	1
15119	101	1	7-Apr-12	18	2	16	2	1	1	0	4	2	1	1
15120	101	1	4-Mar-12	17	2	16	2	1	1	0	4	2	2	1
15121	101	1	23-Dec-12	16	2	16	2	1	1	0	4	2	2	1

15122	101	1	6-Mar-11	10	2	16	2	1	1	0	4	2	1	1
15123	201	1	17-Apr-11	20	2	16	2	2	1	0	4	2	2	1
15124	101	1	11-Nov-11	22	2	16	2	3	1	0	4	2	2	1
15125	101	1	3-Mar-10	16	2	16	2	1	1	0	4	2	1	2
15126	201	1	29-Nov-10	14	2	16	2	1	1	0	4	2	1	1
15127	201	1	4-Jun-10	18	2	16	2	2	1	0	4	2	1	1
15128	101	1	2-May-10	20	2	16	2	1	1	0	4	2	1	1
15129	101	1	2-Feb-09	18	2	16	2	1	1	0	4	2	2	1
15130	101	1	27-Mar-08	18	2	16	2	1	1	0	4	2	3	1
15131	101	1	18-Apr-08	12	2	16	2	1	1	0	4	2	2	1
15132	201	1	7-Aug-07	10	2	16	2	2	1	0	4	2	2	1
15133	101	1	27-Nov-07	18	2	16	2	1	1	0	4	2	1	1
15134	101	1	11-Dec-07	19	2	16	2	2	1	0	4	2	1	1
15135	101	1	27-Dec-06	21	2	16	2	1	1	0	4	2	2	1
15136	201	1	15-Oct-06	20	2	16	2	2	1	0	4	2	2	1
15137	101	1	15-Aug-06	16	2	16	2	3	1	0	4	2	1	1
15138	101	1	25-Sep-06	17	2	16	2	1	1	0	4	2	2	1
15139	101	1	9-Mar-17	18	2	16	2	1	1	0	4	2	4	1
15140	101	1	13-Aug-17	15	2	16	2	1	1	0	4	2	1	1
15141	201	1	7-Nov-16	19	2	16	2	1	1	0	11	2	3	1
15142	101	1	18-Jun-16	20	2	16	2	1	1	0	11	2	2	1
15143	101	1	3-Aug-16	15	2	16	2	1	1	0	11	2	3	1
15144	101	1	6-Aug-16	19	2	16	2	1	1	0	11	2	3	1
15145	101	1	1-Jul-16	11	2	16	2	1	1	0	11	2	3	1
15146	101	1	3-Aug-16	12	2	16	2	1	1	0	11	2	2	1
15147	201	1	12-Aug-15	13	2	16	2	1	1	0	11	2	2	1
15148	101	1	18-Mar-15	12	2	16	2	1	1	0	11	2	3	1
15149	201	1	23-Mar-14	5	2	16	2	2	1	0	11	2	1	1
15150	101	1	26-Dec-14	9	2	16	2	1	1	0	11	2	2	1
15151	101	1	15-Nov-14	18	2	16	2	1	1	0	11	2	3	1
15152	101	1	18-Jul-14	19	2	16	2	1	1	0	11	2	3	1
15153	101	1	10-Dec-14	17	2	16	2	1	1	0	11	2	2	1
15154	201	1	1-Apr-14	11	2	16	2	1	1	0	11	2	3	1
15155	101	1	19-Jul-14	13	2	16	2	1	1	0	11	2	3	1
15156	201	1	23-Nov-13	22	2	16	2	1	1	0	11	2	2	1
15157	201	1	19-Feb-13	10	2	16	2	3	1	0	11	2	2	1
15158	101	1	10-Nov-13	0	2	16	2	1	1	0	11	2	2	1
15159	101	1	23-Jul-12	16	2	16	2	1	1	0	11	2	1	1
15160	101	1	20-Feb-12	19	2	16	2	1	1	0	11	2	2	1
15161	101	1	12-Feb-12	11	2	16	2	1	1	0	11	2	2	1
15162	101	1	2-Sep-12	19	2	16	2	1	1	0	11	2	3	1
15163	201	1	5-Apr-11	10	2	16	2	1	1	0	11	2	2	1
15164	101	1	13-Oct-11	17	2	16	2	1	1	0	11	2	3	1
15165	101	1	31-Jul-11	16	2	16	2	2	1	0	11	2	2	1
15166	201	1	18-Jun-11	16	2	16	2	1	1	0	11	2	3	1
15167	201	1	10-Jun-10	10	2	16	2	3	1	0	11	2	2	1
15168	201	1	5-Sep-10	19	2	16	2	3	1	0	11	2	3	1
15169	201	1	12-Oct-09	8	2	16	2	2	1	0	11	2	3	1
15170	101	1	2-Nov-08	15	2	16	2	1	1	0	11	2	2	1
15171	101	1	21-Oct-08	10	2	16	2	1	1	0	11	2	3	1
15172	201	1	27-Oct-07	10	2	16	2	1	1	0	11	2	3	1
15173	101	1	23-Jul-06	10	2	16	2	1	1	0	11	2	2	2
15174	201	1	17-Sep-06	20	2	16	2	1	1	0	11	2	2	1
15175	201	1	27-Jun-17	13	2	16	2	1	1	0	11	2	3	1
15176	101	1	5-Feb-17	12	2	16	2	1	1	0	11	2	2	1
15177	201	1	11-Sep-17	14	2	16	2	2	1	0	11	2	2	1
15178	201	1	2-Jan-17	10	2	16	2	2	1	0	11	2	1	1
15179	101	1	6-Nov-17	10	2	16	2	1	1	0	11	2	3	1
15180	101	1	19-Nov-17	19	2	16	2	1	1	0	11	2	2	1
15181	101	1	8-Dec-16	21	2	16	2	1	1	0	3	2	3	1
15182	101	1	20-Jan-16	20	2	16	2	1	1	0	3	2	1	1
15183	101	1	19-Mar-16	17	2	16	2	1	1	0	3	2	3	1
15184	101	1	5-Apr-16	10	2	16	2	1	1	0	3	2	3	1
15185	101	1	22-Feb-16	18	2	16	2	1	1	0	3	2	1	1
15186	101	1	1-Mar-16	13	2	16	2	1	1	0	3	2	3	1
15187	101	1	1-May-16	18	2	16	2	1	1	0	3	2	3	1
15188	101	1	13-Jan-16	15	2	16	2	1	1	0	3	2	3	1
15189	101	1	15-Dec-16	16	2	16	2	2	1	0	3	2	3	1
15190	101	1	19-Mar-16	1	2	16	2	1	1	0	3	2	2	1
15191	101	1	16-Dec-16	14	2	16	2	1	1	0	3	2	3	1
15192	201	1	16-Feb-16	20	2	16	2	2	1	0	3	2	3	1
15193	101	1	4-Nov-16	15	2	16	2	1	1	0	3	2	3	1
15194	101	1	25-Oct-16	20	2	16	2	1	1	0	3	2	3	1
15195	101	1	28-Mar-16	17	2	16	2	1	1	0	3	2	3	1
15196	101	1	8-Dec-16	17	2	16	2	1	1	0	3	2	3	1
15197	101	1	4-Nov-16	16	2	16	2	1	1	0	3	2	5	4
15198	201	1	2-Oct-16	17	2	16	2	2	1	0	3	2	1	1
15199	201	1	29-Apr-16	16	2	16	2	3	1	0	3	2	3	1
15200	101	1	28-Nov-15	23	2	16	2	1	1	0	3	2	1	1
15201	101	1	15-Dec-15	17	2	16	2	1	1	0	3	2	3	1
15202	101	1	24-Jan-15	12	2	16	2	1	1	0	3	2	7	1
15203	101	1	27-Oct-15	21	2	16	2	1	1	0	3	2	1	1
15204	101	1	20-Nov-15	18	2	16	2	1	1	0	3	2	3	1
15205	101	1	28-Mar-15	18	2	16	2	1	1	0	3	2	3	1
15206	101	1	12-Dec-15	10	2	16	2	1	1	0	3	2	2	1
15207	101	1	21-Dec-15	16	2	16	2	1	1	0	3	2	3	1
15208	101	1	20-May-15	18	2	16	2	1	1	0	3	2	3	1
15209	201	1	3-Sep-15	9	2	16	2	2	1	0	3	2	5	1
15210	201	1	26-Jun-15	7	2	16	2	1	1	0	3	2	3	1
15211	201	1	21-Jan-15	16	2	16	2	2	1	0	3	2	3	1
15212	101	1	24-Nov-14	11	2	16	2	1	1	0	3	2	1	2
15213	101	1	2-Jul-14	17	2	16	2	1	1	0	3	2	5	1
15214	201	1	20-Aug-14	23	2	16	2	3	1	0	3	2	2	1
15215	201	1	23-Jun-14	19	2	16	2	3	1	0	3	2	3	1
15216	101	1	1-Jul-14	8	2	16	2	1	1	0	3	2	3	1
15217	101	1	12-May-14	12	2	16	2	1	1	0	3	2	2	1
15218	201	1	13-Aug-14	19	2	16	2	2	1	0	3	2	1	1
15219	101	1	21-Dec-14	22	2	16	2	1	1	0	3	2	1	1
15220	101	1	4-Nov-14	7	2	16	2	1	1	0	3	2	1	1
15221	201	1	29-Apr-14	19	2	16	2	3	1	0	3	2	4	1
15222	101	1	29-Apr-14	18	2	16	2	1	1	0	3	2	3	1
15223	101	1	15-May-14	20	2	16	2	1	1	0	3	2	3	1
15224	201	1	3-Oct-14	17	2	16	2	2	1	0	3	2	2	1
15225	201	1	21-Jun-14	15	2	16	2	1	1	0	3	2	3	1
15226	101	1	15-Mar-14	19	2	16	2	1	1	0	3	2	3	1
15227	101	1	18-Nov-14	16	2	16	2	1	1	0	3	2	3	1
15228	201	1	27-Nov-14	13	2	16	2	2	1	0	3	2	3	1
15229	201	1	11-Mar-13	9	2	16	2	2	1	0	3	2	3	1
15230	201	1	4-Sep-13	17	2	16	2	2	1	0	3	2	1	1
15231	101	1	25-Feb-13	16	2	16	2	1	1	0	3	2	3	1
15232	101	1	28-Feb-13	16	2	16	2	1	1	0	3	2	4	1
15233	201	1	19-May-13	20	2	16	2	3	1	0	3	2	5	1
15234	101	1	31-Oct-13	20	2	16	2	1	1	0	3	2	1	1
15235	201	1	20-Dec-13	18	2	16	2	2	1	0	3	2	4	1
15236	101	1	16-Mar-13	20	2	16	2	1	1	0	3	2	5	1
15237	101	1	18-Oct-13	20	2	16	2	1	1	0	3	2	2	1
15238	101	1	15-Jun-13	7	2	16	2	1	1	0	3	2	2	1
15239	101	1	18-May-13	16	2	16	2	1	1	0	3	2	3	1
15240	101	1	24-Apr-13	10	2	16	2	1	1	0	3	2	3	1
15241	101	1	1-Dec-13	15	2	16	2	1	1	0	3	2	2	1
15242	201	1	16-Oct-13	11	2	16	2	2	1	0	3	2	5	1
15243	201	1	26-Jul-13	17	2	16	2	3	1	0	3	2	1	1
15244	201	1	20-Jan-13	20	2	16	2	2	1	0	3	2	1	1
15245	101	1	13-Nov-13	10	2	16	2	1	1	0	3	2	1	2

15246	201	1	1-Jun-13	15	2	16	2	1	1	0	3	2	3	1
15247	201	1	20-Jan-13	10	2	16	2	2	1	0	3	2	2	1
15248	201	1	4-Dec-12	19	2	16	2	1	1	0	3	2	2	1
15249	101	1	12-Feb-12	21	2	16	2	1	1	0	3	2	1	1
15250	101	1	22-Feb-12	23	2	16	2	1	1	0	3	2	3	1
15251	101	1	16-Nov-12	7	2	16	2	1	1	0	3	2	3	1
15252	201	1	6-Nov-12	21	2	16	2	2	1	0	3	2	3	1
15253	101	1	15-Jan-12	11	2	16	2	1	1	0	3	2	2	1
15254	101	1	30-Sep-12	16	2	16	2	1	1	0	3	2	5	1
15255	101	1	1-Mar-12	23	2	16	2	1	1	0	3	2	1	1
15256	101	1	16-Dec-12	2	2	16	2	1	1	0	3	2	1	1
15257	101	1	5-Apr-12	17	2	16	2	1	1	0	3	2	4	1
15258	101	1	5-Sep-12	11	2	16	2	1	1	0	3	2	3	4
15259	101	1	4-Jan-12	13	2	16	2	1	1	0	3	2	3	1
15260	201	1	22-Nov-12	17	2	16	2	2	1	0	3	2	3	1
15261	101	1	2-Nov-12	15	2	16	2	1	1	0	3	2	3	4
15262	201	1	28-Apr-12	16	2	16	2	3	1	0	3	2	1	1
15263	101	1	26-Jun-12	17	2	16	2	1	1	0	3	2	2	4
15264	101	1	11-Jun-11	13	2	16	2	1	1	0	3	2	1	1
15265	101	1	15-Mar-11	19	2	16	2	1	1	0	3	2	3	1
15266	201	1	14-Jul-11	10	2	16	2	3	1	0	3	2	1	1
15267	101	1	5-Feb-11	6	2	16	2	1	1	0	3	2	1	1
15268	201	1	5-Sep-11	12	2	16	2	3	1	0	3	2	4	1
15269	201	1	8-Jul-11	17	2	16	2	1	1	0	3	2	3	1
15270	201	1	25-Jun-11	19	2	16	2	3	1	0	3	2	5	1
15271	101	1	12-Jun-11	12	2	16	2	1	1	0	3	2	2	1
15272	201	1	24-Nov-10	20	2	16	2	1	1	0	3	2	1	1
15273	101	1	18-Apr-10	17	2	16	2	1	1	0	3	2	3	1
15274	101	1	6-Jul-10	20	2	16	2	1	1	0	3	2	3	4
15275	101	1	30-Sep-10	19	2	16	2	1	1	0	3	2	3	2
15276	101	1	5-Aug-10	12	2	16	2	3	1	0	3	2	3	1
15277	201	1	1-Nov-10	21	2	16	2	3	1	0	3	2	1	1
15278	101	1	19-Jan-10	13	2	16	2	1	1	0	3	2	1	1
15279	101	1	22-Oct-10	16	2	16	2	1	1	0	3	2	3	1
15280	201	1	20-Nov-10	19	2	16	2	3	1	0	3	2	4	1
15281	201	1	10-Feb-10	20	2	16	2	3	1	0	3	2	2	1
15282	101	1	18-Dec-10	10	2	16	2	1	1	0	3	2	3	1
15283	101	1	29-Oct-10	20	2	16	2	1	1	0	3	2	3	1
15284	101	1	1-Jan-10	21	2	16	2	1	1	0	3	2	3	1
15285	101	1	16-Feb-09	16	2	16	2	1	1	0	3	2	3	1
15286	201	1	11-Oct-09	9	2	16	2	1	1	0	3	2	6	1
15287	101	1	15-Sep-09	12	2	16	2	1	1	0	3	2	1	1
15288	101	1	28-Sep-09	20	2	16	2	1	1	0	3	2	1	1
15289	201	1	5-Feb-09	21	2	16	2	1	1	0	3	2	2	1
15290	101	1	25-Feb-09	14	2	16	2	1	1	0	3	2	3	1
15291	101	1	19-Mar-09	14	2	16	2	1	1	0	3	2	3	1
15292	101	1	10-Mar-09	17	2	16	2	1	1	0	3	2	3	1
15293	101	1	14-Mar-09	18	2	16	2	1	1	0	3	2	3	1
15294	101	1	21-Oct-09	18	2	16	2	1	1	0	3	2	3	1
15295	101	1	27-Nov-09	8	2	16	2	2	1	0	3	2	3	4
15296	201	1	27-May-09	9	2	16	2	1	1	0	3	2	3	1
15297	201	1	27-Jul-08	15	2	16	2	1	1	0	3	2	3	1
15298	101	1	27-Sep-08	18	2	16	2	1	1	0	3	2	5	1
15299	101	1	21-Apr-08	17	2	16	2	1	1	0	3	2	3	1
15300	101	1	22-Oct-08	16	2	16	2	1	1	0	3	2	3	1
15301	101	1	30-Sep-08	11	2	16	2	1	1	0	3	2	2	1
15302	201	1	17-Jul-08	2	2	16	2	3	1	0	3	2	3	1
15303	201	1	11-Jun-08	9	2	16	2	3	1	0	3	2	3	1
15304	101	1	23-Feb-08	10	2	16	2	1	1	0	3	2	3	1
15305	101	1	27-Nov-08	18	2	16	2	1	1	0	3	2	1	2
15306	101	1	25-Sep-08	15	2	16	2	1	1	0	3	2	4	1
15307	101	1	18-Nov-07	19	2	16	2	1	1	0	3	2	3	1
15308	201	1	11-Mar-07	21	2	16	2	1	1	0	3	2	2	1
15309	101	1	12-Jul-07	11	2	16	2	1	1	0	3	2	3	1
15310	101	1	20-Aug-07	20	2	16	2	1	1	0	3	2	2	1
15311	101	1	9-Oct-07	18	2	16	2	1	1	0	3	2	2	1
15312	201	1	8-Oct-07	17	2	16	2	2	1	0	3	2	2	2
15313	101	1	20-Mar-07	12	2	16	2	1	1	0	3	2	3	1
15314	201	1	6-Mar-07	18	2	16	2	1	1	0	3	2	2	1
15315	101	1	30-May-07	14	2	16	2	1	1	0	3	2	2	1
15316	101	1	5-Apr-07	9	2	16	2	1	1	0	3	2	1	1
15317	201	1	16-Mar-07	20	2	16	2	1	1	0	3	2	4	1
15318	101	1	29-Dec-06	20	2	16	2	1	1	0	3	2	5	1
15319	101	1	7-Jul-06	16	2	16	2	1	1	0	3	2	2	1
15320	101	1	14-Jun-06	21	2	16	2	1	1	0	3	2	3	1
15321	101	1	21-Dec-06	10	2	16	2	2	1	0	3	2	1	1
15322	201	1	29-Dec-06	13	2	16	2	1	1	0	3	2	2	1
15323	201	1	21-Apr-06	13	2	16	2	3	1	0	3	2	2	1
15324	201	1	31-Jul-06	1	2	16	2	1	1	0	3	2	2	1
15325	101	1	6-Apr-17	12	2	16	2	1	1	0	3	2	5	1
15326	101	1	12-Apr-17	16	2	16	2	1	1	0	3	2	4	1
15327	101	1	26-Apr-17	11	2	16	2	1	1	0	3	2	3	1
15328	101	1	21-Feb-17	21	2	16	2	1	1	0	3	2	3	1
15329	101	1	26-May-17	17	2	16	2	1	1	0	3	2	3	1
15330	201	1	30-Jun-17	18	2	16	2	1	1	0	3	2	3	1
15331	101	1	2-Aug-17	16	2	16	2	1	1	0	3	2	1	1
15332	201	1	3-Jul-17	18	2	16	2	2	1	0	3	2	3	1
15333	201	1	26-Apr-17	10	2	16	2	2	1	0	3	2	1	1
15334	201	1	15-Jun-17	16	2	16	2	3	1	0	3	2	3	1
15335	101	1	3-Oct-17	19	2	16	2	1	1	0	3	2	2	1
15336	101	1	17-Dec-17	21	2	16	2	1	1	0	3	2	1	1
15337	201	1	28-Dec-17	17	2	16	2	3	1	0	3	2	3	1
15338	101	1	11-Jul-13	12	2	16	2	1	1	0	6	2	3	1
15339	201	1	7-Jun-13	17	2	16	2	2	1	0	6	2	3	1
15340	101	1	7-Nov-13	17	2	16	2	1	1	0	6	2	2	1
15341	101	1	8-May-13	12	2	16	2	1	1	0	6	2	2	1
15342	101	1	21-Jul-12	1	2	16	2	3	1	0	6	2	3	1
15343	101	1	2-Sep-11	18	2	16	2	1	1	0	6	2	3	1
15344	101	1	23-Mar-11	16	2	16	2	1	1	0	6	2	2	1
15345	101	1	8-Sep-10	11	2	16	2	1	1	0	6	2	2	1
15346	101	1	12-Mar-10	16	2	16	2	1	1	0	6	2	2	1
15347	101	1	4-Dec-10	19	2	16	2	1	1	0	6	2	2	1
15348	101	1	20-Oct-10	1	2	16	2	3	1	0	6	2	2	1
15349	101	1	10-Jan-10	20	2	16	2	1	1	0	6	2	1	1
15350	101	1	24-Nov-09	16	2	16	2	1	1	0	6	2	2	1
15351	101	1	30-Nov-08	8	2	16	2	1	1	0	6	2	3	1
15352	101	1	25-Sep-08	20	2	16	2	1	1	0	6	2	2	1
15353	101	1	30-Mar-07	14	2	16	2	1	1	0	6	2	2	1
15354	101	1	24-May-11	15	2	16	2	1	1	0	13	2	3	1
15355	202	0	18-Jan-16	11	1	17	2	1	1	0	1	2	4	1
15356	202	0	8-May-13	16	1	17	2	2	1	0	1	2	1	1
15357	202	0	3-Jun-17	3	1	17	2	1	1	0	1	2	1	1
15358	202	0	23-Feb-12	10	1	17	2	1	1	0	4	2	1	2
15359	202	0	15-Oct-16	21	1	17	2	1	1	0	10	2	6	2
15360	202	0	20-Dec-14	11	1	17	2	3	1	0	4	2	7	2
15361	202	0	18-Jun-15	19	1	17	2	2	1	0	4	2	2	1
15362	202	0	21-May-17	15	1	17	2	1	1	0	4	2	3	1
15363	202	0	23-Dec-13	9	1	17	2	1	1	0	6	2	3	1
15364	202	0	9-Aug-14	21	1	17	2	1	1	0	6	2	2	1
15365	202	0	19-Sep-13	1	1	17	2	1	1	0	6	2	1	1
15366	202	0	19-Jun-13	18	1	17	2	1	1	0	4	2	2	1
15367	202	0	17-Apr-17	18	1	17	2	1	1	0	3	2	3	1
15368	202	0	10-Aug-13	4	1	17	2	1	1	0	4	2	1	1
15369	202	0	4-Nov-16	15	1	17	2	1	1	0	6	2	2	1

15370	202	0	23-May-14	15	1	17	2	1	1	0	6	2	2	1
15371	202	0	22-Dec-16	15	1	17	2	1	1	0	6	2	2	1
15372	202	0	26-May-16	21	1	17	2	1	1	0	3	2	3	1
15373	202	0	24-Dec-17	22	1	17	2	1	1	0	4	2	2	1
15374	202	0	6-Jun-16	17	1	17	2	1	1	0	6	2	3	1
15375	202	0	2-Jun-15	22	1	17	2	1	1	0	6	2	2	1
15376	202	0	8-Jul-16	19	1	17	2	3	1	0	10	2	6	2
15377	202	0	4-Oct-16	21	1	17	2	3	1	0	10	2	6	1
15378	202	0	1-Oct-12	2	1	17	2	1	1	0	10	2	7	2
15379	202	0	12-Aug-10	23	1	17	2	1	1	0	10	2	4	2
15380	202	0	30-Nov-09	20	1	17	2	1	1	0	10	2	7	2
15381	202	0	14-Nov-17	19	1	17	2	2	1	0	10	2	6	2
15382	202	0	5-Nov-12	20	1	17	2	2	1	0	7	2	1	1
15383	202	0	21-Sep-12	18	1	17	2	3	1	0	7	2	3	1
15384	202	0	26-Feb-06	4	1	17	2	1	1	0	7	2	3	2
15385	202	0	22-Mar-13	17	1	17	2	1	1	0	13	2	3	1
15386	202	0	23-Oct-09	11	1	17	2	1	1	0	13	2	2	1
15387	202	0	2-May-08	9	1	17	2	1	1	0	13	2	1	1
15388	202	0	5-Mar-08	6	1	17	2	1	1	0	13	2	1	1
15389	202	0	1-Sep-08	17	1	17	2	1	1	0	13	2	1	1
15390	202	0	8-May-08	19	1	17	2	1	1	0	13	2	1	1
15391	202	0	9-Jul-08	18	1	17	2	1	1	0	13	2	4	1
15392	202	0	3-Apr-07	17	1	17	2	1	1	0	13	2	6	1
15393	202	0	30-Oct-07	1	1	17	2	1	1	0	13	2	6	2
15394	202	0	19-Aug-07	21	1	17	2	1	1	0	13	2	1	1
15395	202	0	1-Apr-07	18	1	17	2	1	1	0	13	2	3	2
15396	202	0	9-May-06	22	1	17	2	2	1	0	13	2	7	2
15397	202	0	19-Sep-06	18	1	17	2	1	1	0	13	2	1	1
15398	202	0	19-Dec-17	16	1	17	2	1	1	0	13	2	1	1
15399	202	0	12-Jul-08	9	1	17	2	1	1	0	1	2	6	1
15400	202	0	30-May-08	14	1	17	2	1	1	0	1	2	2	2
15401	202	0	17-Mar-16	18	1	17	2	2	1	0	4	2	1	1
15402	202	0	28-Jun-16	2	1	17	2	3	1	0	4	2	3	1
15403	202	0	25-Apr-16	17	1	17	2	2	1	0	4	2	1	1
15404	202	0	28-Sep-15	12	1	17	2	1	1	0	4	2	3	1
15405	202	0	27-Nov-14	19	1	17	2	1	1	0	4	2	4	1
15406	202	0	7-Dec-14	16	1	17	2	1	1	0	4	2	1	1
15407	202	0	28-Sep-14	8	1	17	2	3	1	0	4	2	1	1
15408	202	0	4-Dec-14	17	1	17	2	1	1	0	4	2	1	1
15409	202	0	29-Apr-14	17	1	17	2	1	1	0	4	2	2	1
15410	202	0	6-Nov-14	10	1	17	2	1	1	0	4	2	4	1
15411	202	0	23-Apr-13	18	1	17	2	3	1	0	4	2	1	1
15412	202	0	6-Sep-13	1	1	17	2	1	1	0	4	2	5	1
15413	202	0	23-Dec-13	18	1	17	2	1	1	0	4	2	1	1
15414	202	0	19-Jan-13	22	1	17	2	1	1	0	4	2	3	1
15415	202	0	13-May-13	22	1	17	2	3	1	0	4	2	3	1
15416	202	0	28-Jun-13	19	1	17	2	1	1	0	4	2	4	1
15417	202	0	25-May-13	13	1	17	2	1	1	0	4	2	7	1
15418	202	0	8-May-13	16	1	17	2	1	1	0	4	2	1	1
15419	202	0	25-Apr-13	15	1	17	2	1	1	0	4	2	4	1
15420	202	0	18-May-13	20	1	17	2	1	1	0	4	2	1	1
15421	202	0	19-Apr-12	12	1	17	2	1	1	0	4	2	1	1
15422	202	0	16-Mar-12	22	1	17	2	1	1	0	4	2	1	2
15423	202	0	28-Dec-12	14	1	17	2	1	1	0	4	2	1	1
15424	202	0	15-Dec-12	16	1	17	2	1	1	0	4	2	4	1
15425	202	0	28-Apr-11	18	1	17	2	1	1	0	4	2	1	1
15426	202	0	23-Feb-10	21	1	17	2	1	1	0	4	2	6	1
15427	202	0	7-Nov-10	19	1	17	2	1	1	0	4	2	1	2
15428	202	0	12-Jan-10	14	1	17	2	1	1	0	4	2	1	2
15429	202	0	31-Jan-10	23	1	17	2	1	1	0	4	2	6	1
15430	202	0	15-Sep-10	19	1	17	2	1	1	0	4	2	1	1
15431	202	0	5-Jan-10	22	1	17	2	1	1	0	4	2	1	1
15432	202	0	17-May-10	17	1	17	2	1	1	0	4	2	1	1
15433	202	0	13-Oct-10	18	1	17	2	1	1	0	4	2	1	2
15434	202	0	10-Nov-10	16	1	17	2	1	1	0	4	2	3	1
15435	202	0	25-Dec-09	19	1	17	2	1	1	0	4	2	1	1
15436	202	0	23-Jun-09	19	1	17	2	1	1	0	4	2	1	1
15437	202	0	8-Aug-09	15	1	17	2	1	1	0	4	2	1	1
15438	202	0	7-Nov-09	1	1	17	2	1	1	0	4	2	1	2
15439	202	0	18-Mar-09	17	1	17	2	1	1	0	4	2	1	1
15440	202	0	17-Apr-09	20	1	17	2	1	1	0	4	2	1	1
15441	202	0	22-Jun-09	17	1	17	2	1	1	0	4	2	1	1
15442	202	0	7-May-08	20	1	17	2	1	1	0	4	2	1	1
15443	202	0	29-Feb-08	22	1	17	2	1	1	0	4	2	1	1
15444	202	0	5-Sep-08	22	1	17	2	1	1	0	4	2	4	1
15445	202	0	24-Nov-08	11	1	17	2	1	1	0	4	2	2	1
15446	202	0	17-Nov-08	13	1	17	2	1	1	0	4	2	1	2
15447	202	0	27-Feb-08	10	1	17	2	1	1	0	4	2	1	1
15448	202	0	6-Sep-07	17	1	17	2	1	1	0	4	2	7	2
15449	202	0	19-Sep-07	10	1	17	2	1	1	0	4	2	1	1
15450	202	0	14-Jun-07	1	1	17	2	1	1	0	4	2	1	1
15451	202	0	31-May-07	8	1	17	2	1	1	0	4	2	2	1
15452	202	0	10-Apr-06	11	1	17	2	1	1	0	4	2	1	1
15453	202	0	8-Nov-06	13	1	17	2	1	1	0	4	2	1	1
15454	202	0	13-Jun-17	18	1	17	2	2	1	0	4	2	4	1
15455	202	0	27-Jun-17	18	1	17	2	1	1	0	4	2	2	1
15456	202	0	20-Sep-17	10	1	17	2	1	1	0	4	2	1	1
15457	202	0	25-Nov-17	0	1	17	2	1	1	0	4	2	1	1
15458	202	0	28-Apr-17	8	1	17	2	1	1	0	4	2	1	1
15459	202	0	28-Apr-17	8	1	17	2	1	1	0	4	2	1	1
15460	202	0	3-Nov-17	22	1	17	2	2	1	0	4	2	1	1
15461	202	0	29-Jul-17	9	1	17	2	2	1	0	4	2	1	1
15462	202	0	3-May-17	14	1	17	2	2	1	0	4	2	4	2
15463	202	0	9-Mar-16	20	1	17	2	1	1	0	11	2	3	1
15464	202	0	8-Jun-16	17	1	17	2	1	1	0	11	2	2	1
15465	202	0	1-Sep-16	17	1	17	2	1	1	0	11	2	3	1
15466	202	0	19-May-15	21	1	17	2	1	1	0	11	2	4	1
15467	202	0	1-May-14	23	1	17	2	1	1	0	11	2	2	1
15468	202	0	16-Jul-13	12	1	17	2	1	1	0	11	2	3	1
15469	202	0	21-Nov-13	8	1	17	2	3	1	0	11	2	3	1
15470	202	0	14-Oct-12	17	1	17	2	1	1	0	11	2	2	2
15471	202	0	4-Sep-12	22	1	17	2	1	1	0	11	2	3	1
15472	202	0	13-Aug-12	20	1	17	2	1	1	0	11	2	3	1
15473	202	0	7-Nov-10	22	1	17	2	1	1	0	11	2	3	1
15474	202	0	26-Oct-09	14	1	17	2	1	1	0	11	2	3	1
15475	202	0	24-Jul-08	18	1	17	2	1	1	0	11	2	2	1
15476	202	0	24-Jul-08	19	1	17	2	1	1	0	11	2	2	1
15477	202	0	6-Oct-07	20	1	17	2	1	1	0	11	2	6	2
15478	202	0	8-Jul-07	1	1	17	2	1	1	0	11	2	3	1
15479	202	0	2-Jun-17	8	1	17	2	1	1	0	11	2	3	1
15480	202	0	28-Oct-17	21	1	17	2	3	1	0	11	2	5	2
15481	202	0	24-Apr-16	19	1	17	2	2	1	0	3	2	1	1
15482	202	0	1-Feb-16	21	1	17	2	3	1	0	3	2	3	1
15483	202	0	21-Mar-16	22	1	17	2	2	1	0	3	2	1	2
15484	202	0	2-May-16	19	1	17	2	1	1	0	3	2	1	1
15485	202	0	3-Apr-16	15	1	17	2	1	1	0	3	2	1	1
15486	202	0	11-Feb-16	22	1	17	2	1	1	0	3	2	3	1
15487	202	0	9-Jan-16	18	1	17	2	3	1	0	3	2	3	1
15488	202	0	17-Apr-16	17	1	17	2	1	1	0	3	2	3	1
15489	202	0	11-May-16	12	1	17	2	1	1	0	3	2	2	1
15490	202	0	5-Jan-16	21	1	17	2	2	1	0	3	2	3	1
15491	202	0	20-Oct-16	11	1	17	2	2	1	0	3	2	1	1
15492	202	0	22-Mar-16	19	1	17	2	1	1	0	3	2	1	2
15493	202	0	10-Mar-16	0	1	17	2	1	1	0	3	2	1	1

15494	202	0	27-Apr-16	22	1	17	2	1	1	0	3	2	2	1
15495	202	0	30-Oct-15	14	1	17	2	1	1	0	3	2	1	1
15496	202	0	22-Aug-15	9	1	17	2	1	1	0	3	2	2	1
15497	202	0	4-Dec-15	12	1	17	2	1	1	0	3	2	2	2
15498	202	0	7-Sep-15	11	1	17	2	1	1	0	3	2	3	1
15499	202	0	13-Oct-15	17	1	17	2	1	1	0	3	2	2	1
15500	202	0	23-Dec-15	17	1	17	2	1	1	0	3	2	6	1
15501	202	0	25-Mar-14	20	1	17	2	1	1	0	3	2	1	1
15502	202	0	18-Nov-14	17	1	17	2	1	1	0	3	2	4	1
15503	202	0	12-Nov-14	22	1	17	2	1	1	0	3	2	1	2
15504	202	0	6-May-14	22	1	17	2	1	1	0	3	2	4	1
15505	202	0	7-Oct-14	22	1	17	2	1	1	0	3	2	3	1
15506	202	0	26-Mar-14	17	1	17	2	2	1	0	3	2	5	1
15507	202	0	24-Aug-14	14	1	17	2	2	1	0	3	2	3	1
15508	202	0	28-Sep-14	20	1	17	2	2	1	0	3	2	1	1
15509	202	0	28-Apr-14	17	1	17	2	1	1	0	3	2	2	1
15510	202	0	23-Nov-14	1	1	17	2	2	1	0	3	2	1	1
15511	202	0	28-Jan-14	9	1	17	2	1	1	0	3	2	3	1
15512	202	0	30-Nov-14	4	1	17	2	1	1	0	3	2	5	1
15513	202	0	14-Oct-14	17	1	17	2	1	1	0	3	2	3	1
15514	202	0	24-Sep-14	17	1	17	2	1	1	0	3	2	1	1
15515	202	0	26-Apr-14	20	1	17	2	1	1	0	3	2	4	1
15516	202	0	2-Nov-14	21	1	17	2	1	1	0	3	2	2	1
15517	202	0	13-Oct-14	19	1	17	2	1	1	0	3	2	3	1
15518	202	0	29-Dec-14	15	1	17	2	1	1	0	3	2	3	1
15519	202	0	2-Oct-14	19	1	17	2	1	1	0	3	2	1	1
15520	202	0	4-Aug-14	8	1	17	2	1	1	0	3	2	2	1
15521	202	0	5-Dec-14	16	1	17	2	1	1	0	3	2	3	1
15522	202	0	10-Dec-14	16	1	17	2	1	1	0	3	2	2	1
15523	202	0	4-Sep-14	18	1	17	2	1	1	0	3	2	2	1
15524	202	0	17-May-14	21	1	17	2	1	1	0	3	2	3	1
15525	202	0	19-Feb-14	16	1	17	2	1	1	0	3	2	2	1
15526	202	0	26-Mar-13	22	1	17	2	1	1	0	3	2	1	2
15527	202	0	15-May-13	22	1	17	2	1	1	0	3	2	2	1
15528	202	0	19-Sep-13	23	1	17	2	1	1	0	3	2	2	1
15529	202	0	7-Sep-13	22	1	17	2	1	1	0	3	2	1	1
15530	202	0	4-May-13	8	1	17	2	1	1	0	3	2	1	1
15531	202	0	5-Nov-13	18	1	17	2	1	1	0	3	2	1	1
15532	202	0	4-Jan-13	19	1	17	2	1	1	0	3	2	2	1
15533	202	0	13-Nov-13	12	1	17	2	1	1	0	3	2	2	1
15534	202	0	10-Apr-13	3	1	17	2	3	1	0	3	2	1	1
15535	202	0	25-Dec-13	16	1	17	2	1	1	0	3	2	1	2
15536	202	0	26-Jul-13	0	1	17	2	1	1	0	3	2	1	1
15537	202	0	15-Oct-13	21	1	17	2	1	1	0	3	2	1	1
15538	202	0	17-Jul-13	18	1	17	2	1	1	0	3	2	3	1
15539	202	0	30-Oct-13	11	1	17	2	1	1	0	3	2	2	1
15540	202	0	16-Sep-13	15	1	17	2	1	1	0	3	2	3	1
15541	202	0	11-Oct-13	10	1	17	2	1	1	0	3	2	3	1
15542	202	0	7-Nov-13	17	1	17	2	3	1	0	3	2	2	2
15543	202	0	13-May-13	19	1	17	2	1	1	0	3	2	3	1
15544	202	0	27-Feb-13	17	1	17	2	1	1	0	3	2	3	1
15545	202	0	2-Dec-13	17	1	17	2	1	1	0	3	2	2	1
15546	202	0	15-Mar-13	2	1	17	2	1	1	0	3	2	2	1
15547	202	0	4-Mar-13	17	1	17	2	1	1	0	3	2	1	1
15548	202	0	11-Feb-13	17	1	17	2	1	1	0	3	2	2	2
15549	202	0	13-Dec-12	11	1	17	2	1	1	0	3	2	1	1
15550	202	0	2-Jun-12	17	1	17	2	3	1	0	3	2	2	1
15551	202	0	28-Feb-12	18	1	17	2	1	1	0	3	2	2	1
15552	202	0	23-Jul-12	21	1	17	2	1	1	0	3	2	1	1
15553	202	0	21-Jun-12	16	1	17	2	1	1	0	3	2	2	2
15554	202	0	17-Oct-12	18	1	17	2	1	1	0	3	2	3	2
15555	202	0	17-Oct-12	18	1	17	2	1	1	0	3	2	3	2
15556	202	0	21-Nov-12	11	1	17	2	3	1	0	3	2	1	1
15557	202	0	14-Jun-12	18	1	17	2	1	1	0	3	2	2	2
15558	202	0	14-Jun-12	18	1	17	2	1	1	0	3	2	2	2
15559	202	0	21-Oct-12	10	1	17	2	1	1	0	3	2	3	1
15560	202	0	1-Sep-12	1	1	17	2	1	1	0	3	2	5	1
15561	202	0	12-Apr-11	11	1	17	2	1	1	0	3	2	2	1
15562	202	0	14-Dec-11	15	1	17	2	1	1	0	3	2	1	2
15563	202	0	18-Mar-10	1	1	17	2	1	1	0	3	2	4	1
15564	202	0	21-Jun-10	1	1	17	2	1	1	0	3	2	1	2
15565	202	0	19-Sep-10	12	1	17	2	1	1	0	3	2	2	1
15566	202	0	20-Jun-10	16	1	17	2	1	1	0	3	2	1	2
15567	202	0	6-Dec-10	10	1	17	2	1	1	0	3	2	1	1
15568	202	0	28-Feb-10	2	1	17	2	1	1	0	3	2	1	2
15569	202	0	25-Oct-10	19	1	17	2	1	1	0	3	2	1	1
15570	202	0	5-Dec-10	1	1	17	2	1	1	0	3	2	1	1
15571	202	0	19-Sep-10	22	1	17	2	1	1	0	3	2	3	1
15572	202	0	26-May-10	17	1	17	2	1	1	0	3	2	3	2
15573	202	0	25-May-10	18	1	17	2	1	1	0	3	2	1	2
15574	202	0	18-Sep-10	15	1	17	2	1	1	0	3	2	1	1
15575	202	0	3-Jun-10	11	1	17	2	1	1	0	3	2	2	2
15576	202	0	31-Jan-10	17	1	17	2	1	1	0	3	2	2	2
15577	202	0	12-Mar-10	20	1	17	2	1	1	0	3	2	2	1
15578	202	0	6-Jan-10	16	1	17	2	1	1	0	3	2	2	1
15579	202	0	7-Dec-09	19	1	17	2	1	1	0	3	2	1	1
15580	202	0	5-Dec-09	22	1	17	2	1	1	0	3	2	2	1
15581	202	0	18-Sep-09	13	1	17	2	2	1	0	3	2	1	1
15582	202	0	30-Mar-09	15	1	17	2	1	1	0	3	2	1	1
15583	202	0	19-May-09	22	1	17	2	1	1	0	3	2	1	1
15584	202	0	3-Nov-09	20	1	17	2	1	1	0	3	2	7	1
15585	202	0	10-Jun-09	23	1	17	2	1	1	0	3	2	1	1
15586	202	0	30-Nov-09	16	1	17	2	1	1	0	3	2	3	1
15587	202	0	2-Dec-09	12	1	17	2	2	1	0	3	2	2	1
15588	202	0	4-Jan-09	11	1	17	2	3	1	0	3	2	2	1
15589	202	0	3-Nov-09	19	1	17	2	1	1	0	3	2	1	1
15590	202	0	16-Oct-09	18	1	17	2	2	1	0	3	2	4	2
15591	202	0	21-May-09	17	1	17	2	1	1	0	3	2	1	2
15592	202	0	21-Oct-09	17	1	17	2	3	1	0	3	2	3	1
15593	202	0	8-Dec-09	17	1	17	2	1	1	0	3	2	3	1
15594	202	0	19-Sep-09	19	1	17	2	1	1	0	3	2	2	2
15595	202	0	11-Mar-08	19	1	17	2	1	1	0	3	2	2	2
15596	202	0	6-Nov-08	20	1	17	2	1	1	0	3	2	1	1
15597	202	0	16-May-08	21	1	17	2	1	1	0	3	2	1	1
15598	202	0	26-Nov-08	21	1	17	2	1	1	0	3	2	1	1
15599	202	0	19-Dec-08	16	1	17	2	1	1	0	3	2	4	2
15600	202	0	24-May-08	18	1	17	2	1	1	0	3	2	3	1
15601	202	0	25-Mar-08	17	1	17	2	3	1	0	3	2	2	1
15602	202	0	6-Oct-08	18	1	17	2	1	1	0	3	2	3	2
15603	202	0	20-Mar-08	17	1	17	2	1	1	0	3	2	3	1
15604	202	0	12-Jun-08	15	1	17	2	1	1	0	3	2	1	1
15605	202	0	21-May-08	9	1	17	2	1	1	0	3	2	2	1
15606	202	0	25-Jun-08	18	1	17	2	1	1	0	3	2	3	1
15607	202	0	8-May-08	21	1	17	2	1	1	0	3	2	2	1
15608	202	0	2-Sep-08	20	1	17	2	1	1	0	3	2	2	1
15609	202	0	12-Mar-08	19	1	17	2	1	1	0	3	2	3	1
15610	202	0	26-Nov-08	21	1	17	2	1	1	0	3	2	1	1
15611	202	0	6-Sep-08	21	1	17	2	1	1	0	3	2	2	2
15612	202	0	20-Oct-08	18	1	17	2	3	1	0	3	2	2	1
15613	202	0	17-Dec-08	18	1	17	2	1	1	0	3	2	3	2
15614	202	0	31-May-08	11	1	17	2	1	1	0	3	2	3	1
15615	202	0	11-Dec-08	17	1	17	2	1	1	0	3	2	2	2
15616	202	0	20-Feb-08	16	1	17	2	1	1	0	3	2	3	1
15617	202	0	4-Mar-08	19	1	17	2	1	1	0	3	2	4	2

15618	202	0	16-Sep-08	18	1	17	2	1	1	0	3	2	2	1
15619	202	0	27-Sep-08	9	1	17	2	1	1	0	3	2	3	1
15620	202	0	15-Jul-08	6	1	17	2	1	1	0	3	2	2	1
15621	202	0	4-May-07	13	1	17	2	1	1	0	3	2	3	2
15622	202	0	29-Jun-07	6	1	17	2	3	1	0	3	2	1	1
15623	202	0	18-Oct-07	16	1	17	2	1	1	0	3	2	2	2
15624	202	0	28-Dec-07	22	1	17	2	1	1	0	3	2	3	2
15625	202	0	8-Oct-07	20	1	17	2	1	1	0	3	2	2	1
15626	202	0	19-Apr-07	10	1	17	2	3	1	0	3	2	1	2
15627	202	0	25-Apr-07	8	1	17	2	1	1	0	3	2	3	2
15628	202	0	22-May-07	17	1	17	2	1	1	0	3	2	2	1
15629	202	0	16-Jun-07	18	1	17	2	1	1	0	3	2	1	1
15630	202	0	13-Sep-07	15	1	17	2	1	1	0	3	2	3	2
15631	202	0	30-Mar-07	17	1	17	2	1	1	0	3	2	2	1
15632	202	0	4-Aug-07	19	1	17	2	1	1	0	3	2	3	1
15633	202	0	3-Jul-07	18	1	17	2	1	1	0	3	2	2	1
15634	202	0	4-Jun-06	17	1	17	2	1	1	0	3	2	1	1
15635	202	0	9-Aug-06	18	1	17	2	3	1	0	3	2	1	1
15636	202	0	9-Nov-06	17	1	17	2	1	1	0	3	2	1	1
15637	202	0	22-Feb-06	13	1	17	2	3	1	0	3	2	2	1
15638	202	0	31-Aug-17	19	1	17	2	1	1	0	3	2	3	1
15639	202	0	27-Mar-17	17	1	17	2	1	1	0	3	2	3	1
15640	202	0	24-Nov-17	16	1	17	2	1	1	0	3	2	3	1
15641	202	0	12-Jun-17	0	1	17	2	1	1	0	3	2	2	1
15642	202	0	16-Jun-17	3	1	17	2	1	1	0	3	2	1	1
15643	202	0	9-Sep-17	1	1	17	2	1	1	0	3	2	1	1
15644	202	0	3-Aug-17	10	1	17	2	2	1	0	3	2	6	1
15645	202	0	10-Nov-17	10	1	17	2	2	1	0	3	2	5	1
15646	202	0	24-Sep-17	0	1	17	2	2	1	0	3	2	1	1
15647	202	0	19-Jun-17	0	1	17	2	3	1	0	3	2	5	1
15648	202	0	10-Mar-17	16	1	17	2	3	1	0	3	2	3	1
15649	202	0	12-Dec-17	10	1	17	2	1	1	0	3	2	3	1
15650	202	0	21-Dec-17	12	1	17	2	1	1	0	3	2	4	2
15651	202	0	5-Mar-17	19	1	17	2	1	1	0	3	2	3	1
15652	202	0	29-Apr-17	17	1	17	2	2	1	0	3	2	3	2
15653	202	0	4-Jan-17	22	1	17	2	3	1	0	3	2	1	1
15654	202	0	18-May-16	23	1	17	2	1	1	0	6	2	7	1
15655	202	0	27-Apr-13	14	1	17	2	1	1	0	6	2	2	1
15656	202	0	19-Dec-13	23	1	17	2	1	1	0	6	2	3	1
15657	202	0	12-Jun-13	23	1	17	2	3	1	0	6	2	2	1
15658	202	0	29-Sep-13	22	1	17	2	2	1	0	6	2	2	1
15659	202	0	29-Apr-13	1	1	17	2	1	1	0	6	2	3	1
15660	202	0	14-Jul-13	8	1	17	2	1	1	0	6	2	2	1
15661	202	0	28-Dec-13	21	1	17	2	1	1	0	6	2	1	1
15662	202	0	19-Jun-12	22	1	17	2	2	1	0	6	2	2	1
15663	202	0	16-Jul-10	14	1	17	2	2	1	0	6	2	2	2
15664	202	0	8-Oct-10	13	1	17	2	1	1	0	6	2	3	1
15665	202	0	5-Mar-10	23	1	17	2	1	1	0	6	2	2	1
15666	202	0	9-Sep-08	22	1	17	2	1	1	0	6	2	4	2
15667	202	0	4-Sep-08	15	1	17	2	1	1	0	6	2	4	2
15668	202	0	9-Sep-08	22	1	17	2	1	1	0	6	2	2	2
15669	202	0	11-Dec-08	19	1	17	2	1	1	0	6	2	2	1
15670	202	0	9-Jan-08	17	1	17	2	2	1	0	6	2	7	1
15671	202	0	16-Aug-07	18	1	17	2	1	1	0	6	2	1	2
15672	202	0	17-Apr-07	18	1	17	2	2	1	0	6	2	1	2
15673	202	0	15-Jul-07	0	1	17	2	1	1	0	6	2	2	2
15674	202	0	19-Jun-06	19	1	17	2	1	1	0	6	2	2	1
15675	202	0	22-May-17	20	1	17	2	1	1	0	6	2	2	1
15676	202	0	27-Oct-09	1	1	17	2	1	1	0	13	2	7	2
15677	202	0	18-Nov-08	16	1	17	2	1	1	0	13	2	1	2
15678	202	0	19-Apr-08	11	1	17	2	1	1	0	13	2	3	1
15679	202	0	22-Oct-07	22	1	17	2	1	1	0	13	2	1	1
15680	202	0	18-Aug-06	17	1	17	2	1	1	0	13	2	1	1
15681	202	0	24-Dec-06	23	1	17	2	1	1	0	13	2	2	1
15682	202	0	12-Oct-06	14	1	17	2	1	1	0	13	2	1	1
15683	202	0	14-Sep-17	15	2	17	2	2	1	0	10	2	6	1
15684	202	0	29-Sep-13	11	2	17	2	1	1	0	3	2	2	1
15685	202	0	22-Jul-11	1	2	17	2	1	1	0	10	2	7	2
15686	202	0	14-Apr-09	3	2	17	2	1	1	0	10	2	7	1
15687	202	0	3-Jan-06	22	2	17	2	2	1	0	10	2	7	1
15688	202	0	24-Sep-07	18	2	17	2	1	1	0	13	2	1	1
15689	202	0	25-Apr-07	8	2	17	2	1	1	0	13	2	1	1
15690	202	0	10-Jul-09	9	2	17	2	1	1	0	1	2	1	2
15691	202	0	5-Nov-08	17	2	17	2	1	1	0	1	2	6	2
15692	202	0	13-Oct-15	6	2	17	2	1	1	0	4	2	2	1
15693	202	0	5-Jun-15	0	2	17	2	1	1	0	4	2	3	1
15694	202	0	22-Nov-15	20	2	17	2	2	1	0	4	2	2	1
15695	202	0	10-Aug-15	10	2	17	2	1	1	0	4	2	3	1
15696	202	0	16-Nov-12	15	2	17	2	1	1	0	4	2	1	1
15697	202	0	4-Mar-11	17	2	17	2	1	1	0	4	2	2	1
15698	202	0	11-Nov-11	18	2	17	2	2	1	0	4	2	2	1
15699	202	0	28-Sep-10	20	2	17	2	1	1	0	4	2	2	1
15700	202	0	29-Sep-09	19	2	17	2	1	1	0	4	2	2	2
15701	202	0	7-Jun-08	16	2	17	2	1	1	0	4	2	2	1
15702	202	0	26-Jan-08	22	2	17	2	1	1	0	4	2	1	1
15703	202	0	26-Aug-07	15	2	17	2	1	1	0	4	2	2	2
15704	202	0	23-Oct-17	8	2	17	2	1	1	0	4	2	1	1
15705	202	0	21-Jan-17	16	2	17	2	3	1	0	4	2	4	1
15706	202	0	13-May-16	8	2	17	2	1	1	0	11	2	2	1
15707	202	0	19-Aug-15	20	2	17	2	2	1	0	11	2	3	2
15708	202	0	23-Aug-13	18	2	17	2	1	1	0	11	2	3	2
15709	202	0	25-Apr-13	22	2	17	2	2	1	0	11	2	2	1
15710	202	0	1-May-10	18	2	17	2	1	1	0	11	2	2	1
15711	202	0	12-Sep-07	13	2	17	2	1	1	0	11	2	3	1
15712	202	0	4-Jun-07	6	2	17	2	1	1	0	11	2	3	1
15713	202	0	8-Oct-17	13	2	17	2	2	1	0	11	2	2	1
15714	202	0	20-Sep-16	19	2	17	2	1	1	0	3	2	3	1
15715	202	0	29-Jan-16	8	2	17	2	3	1	0	3	2	4	1
15716	202	0	20-Nov-16	20	2	17	2	3	1	0	3	2	3	1
15717	202	0	19-Apr-16	16	2	17	2	1	1	0	3	2	1	1
15718	202	0	9-Jan-16	23	2	17	2	3	1	0	3	2	1	1
15719	202	0	2-Oct-16	12	2	17	2	1	1	0	3	2	1	2
15720	202	0	26-Jun-16	20	2	17	2	2	1	0	3	2	3	1
15721	202	0	13-Jan-16	17	2	17	2	2	1	0	3	2	3	1
15722	202	0	13-Nov-16	17	2	17	2	1	1	0	3	2	4	1
15723	202	0	25-Dec-15	9	2	17	2	1	1	0	3	2	1	1
15724	202	0	11-Nov-15	11	2	17	2	1	1	0	3	2	1	1
15725	202	0	6-Sep-15	11	2	17	2	3	1	0	3	2	3	1
15726	202	0	26-Nov-15	17	2	17	2	1	1	0	3	2	3	2
15727	202	0	11-Dec-14	16	2	17	2	1	1	0	3	2	3	1
15728	202	0	22-Jun-14	23	2	17	2	3	1	0	3	2	4	2
15729	202	0	17-Feb-14	15	2	17	2	1	1	0	3	2	1	1
15730	202	0	16-Feb-14	18	2	17	2	2	1	0	3	2	1	1
15731	202	0	13-Nov-14	15	2	17	2	1	1	0	3	2	3	1
15732	202	0	22-Oct-14	19	2	17	2	3	1	0	3	2	3	2
15733	202	0	2-Dec-14	18	2	17	2	1	1	0	3	2	3	1
15734	202	0	11-Jan-14	16	2	17	2	1	1	0	3	2	3	2
15735	202	0	9-Nov-13	12	2	17	2	1	1	0	3	2	1	1
15736	202	0	29-Jul-13	6	2	17	2	1	1	0	3	2	5	1
15737	202	0	5-Sep-13	7	2	17	2	1	1	0	3	2	1	1
15738	202	0	3-May-13	17	2	17	2	1	1	0	3	2	1	1
15739	202	0	11-Nov-13	18	2	17	2	1	1	0	3	2	1	1
15740	202	0	5-Sep-12	21	2	17	2	1	1	0	3	2	6	2
15741	202	0	24-Dec-12	13	2	17	2	1	1	0	3	2	3	1

15742	202	0	17-Nov-11	18	2	17	2	1	1	0	3	2	4	2
15743	202	0	21-Jun-10	18	2	17	2	1	1	0	3	2	1	1
15744	202	0	9-Apr-10	11	2	17	2	1	1	0	3	2	1	2
15745	202	0	17-Dec-10	15	2	17	2	3	1	0	3	2	3	2
15746	202	0	19-Apr-10	20	2	17	2	1	1	0	3	2	7	1
15747	202	0	9-Apr-10	20	2	17	2	3	1	0	3	2	3	1
15748	202	0	5-Apr-09	21	2	17	2	1	1	0	3	2	3	2
15749	202	0	23-Sep-09	18	2	17	2	1	1	0	3	2	3	1
15750	202	0	7-Nov-08	15	2	17	2	1	1	0	3	2	2	2
15751	202	0	7-May-08	11	2	17	2	1	1	0	3	2	1	2
15752	202	0	16-Jan-07	20	2	17	2	1	1	0	3	2	1	1
15753	202	0	8-Oct-07	18	2	17	2	1	1	0	3	2	2	1
15754	202	0	16-Nov-07	9	2	17	2	1	1	0	3	2	5	1
15755	202	0	7-May-07	18	2	17	2	1	1	0	3	2	3	1
15756	202	0	10-May-07	18	2	17	2	1	1	0	3	2	3	1
15757	202	0	5-May-06	14	2	17	2	1	1	0	3	2	3	1
15758	202	0	19-Jun-17	14	2	17	2	1	1	0	3	2	1	1
15759	202	0	5-Nov-17	0	2	17	2	2	1	0	3	2	2	1
15760	202	0	19-Jan-17	22	2	17	2	3	1	0	3	2	1	1
15761	202	0	22-Oct-12	21	2	17	2	3	1	0	6	2	2	2
15762	202	0	28-Sep-11	12	2	17	2	1	1	0	6	2	3	1
15763	202	0	20-Jun-10	15	2	17	2	1	1	0	6	2	2	2
15764	202	0	7-Jan-10	12	2	17	2	1	1	0	6	2	2	1
15765	202	0	30-Dec-08	12	2	17	2	1	1	0	6	2	2	2
15766	202	0	7-Jun-08	16	2	17	2	1	1	0	13	2	2	1
15767	202	0	30-Jul-08	21	2	17	2	2	1	0	13	2	1	2
15768	201	1	12-Dec-12	8	1	17	2	1	1	0	1	2	5	1
15769	201	1	19-Nov-14	16	1	17	2	3	1	0	1	2	5	1
15770	101	1	14-Oct-12	21	1	17	2	1	1	0	1	2	1	1
15771	201	1	20-Jan-17	14	1	17	2	2	1	0	1	2	2	1
15772	201	1	10-May-12	18	1	17	2	2	1	0	4	2	1	1
15773	201	1	27-Mar-13	13	1	17	2	2	1	0	6	2	2	2
15774	201	1	19-Aug-14	12	1	17	2	2	1	0	6	2	2	1
15775	101	1	14-Apr-12	13	1	17	2	1	1	0	6	2	2	1
15776	201	1	19-Jul-14	19	1	17	2	2	1	0	6	2	2	1
15777	201	1	12-Sep-17	16	1	17	2	2	1	0	4	2	2	1
15778	101	1	26-Jun-14	21	1	17	2	1	1	0	4	2	4	1
15779	201	1	27-Feb-15	23	1	17	2	1	1	0	6	2	2	1
15780	201	1	15-Jun-16	11	1	17	2	3	1	0	4	2	2	1
15781	201	1	10-Mar-16	9	1	17	2	3	1	0	4	2	2	1
15782	101	1	1-Apr-17	18	1	17	2	1	1	0	4	2	2	1
15783	201	1	17-Apr-15	15	1	17	2	3	1	0	3	2	5	1
15784	201	1	1-May-16	18	1	17	2	1	1	0	4	2	3	2
15785	201	1	7-Aug-15	17	1	17	2	3	1	0	4	2	2	1
15786	101	1	20-Nov-14	9	1	17	2	1	1	0	6	2	2	1
15787	201	1	2-Apr-13	12	1	17	2	1	1	0	3	2	3	1
15788	201	1	23-Jan-12	13	1	17	2	2	1	0	4	2	1	1
15789	101	1	2-Jan-13	16	1	17	2	1	1	0	6	2	2	1
15790	201	1	13-Jun-17	22	1	17	2	1	1	0	6	2	2	1
15791	201	1	30-Sep-17	20	1	17	2	1	1	0	3	2	3	1
15792	101	1	10-May-16	18	1	17	2	1	1	0	6	2	3	1
15793	201	1	26-May-14	14	1	17	2	1	1	0	6	2	3	1
15794	201	1	20-Jun-17	19	1	17	2	1	1	0	6	2	3	1
15795	201	1	7-Mar-16	19	1	17	2	1	1	0	7	2	2	1
15796	101	1	29-May-17	17	1	17	2	1	1	0	3	2	3	1
15797	101	1	13-May-17	15	1	17	2	1	1	0	3	2	1	1
15798	201	1	10-Jan-13	19	1	17	2	1	1	0	7	2	3	1
15799	101	1	4-Jan-11	17	1	17	2	1	1	0	7	2	3	1
15800	101	1	9-Jun-07	17	1	17	2	2	1	0	7	2	2	1
15801	101	1	27-Mar-17	17	1	17	2	1	1	0	7	2	1	1
15802	201	1	16-Jan-12	16	1	17	2	2	1	0	13	2	1	1
15803	101	1	20-Nov-08	17	1	17	2	4	1	0	13	2	3	1
15804	101	1	2-Jul-08	19	1	17	2	1	1	0	13	2	3	1
15805	101	1	6-Oct-07	17	1	17	2	1	1	0	13	2	3	1
15806	101	1	27-Oct-07	16	1	17	2	1	1	0	13	2	2	1
15807	101	1	16-Jun-06	17	1	17	2	1	1	0	13	2	3	1
15808	101	1	4-Apr-06	16	1	17	2	1	1	0	13	2	1	1
15809	101	1	20-Nov-17	22	1	17	2	1	1	0	13	2	3	1
15810	101	1	30-Jun-11	23	1	17	2	1	1	0	1	2	3	1
15811	201	1	10-Oct-10	19	1	17	2	2	1	0	1	2	1	1
15812	201	1	11-Mar-08	20	1	17	2	2	1	0	1	2	3	1
15813	201	1	11-Mar-08	20	1	17	2	2	1	0	1	2	2	1
15814	101	1	22-Apr-14	23	1	17	2	1	1	0	1	2	1	1
15815	201	1	30-Jun-09	18	1	17	2	1	1	0	1	2	4	1
15816	201	1	24-Jul-16	0	1	17	2	1	1	0	4	2	4	1
15817	101	1	20-Jan-16	18	1	17	2	1	1	0	4	2	1	1
15818	101	1	17-Sep-15	11	1	17	2	1	1	0	4	2	1	1
15819	101	1	16-Jun-15	19	1	17	2	1	1	0	4	2	3	1
15820	101	1	18-Sep-15	16	1	17	2	1	1	0	4	2	2	1
15821	101	1	18-Sep-14	18	1	17	2	1	1	0	4	2	3	1
15822	101	1	13-Feb-14	17	1	17	2	1	1	0	4	2	3	2
15823	101	1	10-Nov-14	18	1	17	2	1	1	0	4	2	2	1
15824	101	1	13-Oct-14	7	1	17	2	1	1	0	4	2	3	1
15825	101	1	5-Dec-14	12	1	17	2	1	1	0	4	2	1	1
15826	201	1	6-Nov-14	11	1	17	2	3	1	0	4	2	1	1
15827	101	1	13-Dec-14	16	1	17	2	1	1	0	4	2	4	1
15828	101	1	8-Jan-14	16	1	17	2	1	1	0	4	2	4	2
15829	101	1	24-Apr-14	14	1	17	2	1	1	0	4	2	2	1
15830	201	1	28-Aug-14	14	1	17	2	2	1	0	4	2	2	1
15831	101	1	27-Sep-14	16	1	17	2	1	1	0	4	2	2	1
15832	201	1	14-Feb-14	9	1	17	2	1	1	0	4	2	2	1
15833	101	1	16-Jun-13	10	1	17	2	1	1	0	4	2	2	1
15834	101	1	20-Nov-13	11	1	17	2	1	1	0	4	2	3	1
15835	101	1	30-Jun-13	11	1	17	2	1	1	0	4	2	2	1
15836	101	1	8-Jul-13	20	1	17	2	1	1	0	4	2	1	1
15837	101	1	5-Nov-13	16	1	17	2	1	1	0	4	2	3	1
15838	101	1	30-Mar-13	17	1	17	2	1	1	0	4	2	2	1
15839	101	1	22-Sep-13	8	1	17	2	1	1	0	4	2	2	1
15840	101	1	4-Sep-13	14	1	17	2	1	1	0	4	2	3	1
15841	101	1	11-Jun-13	10	1	17	2	1	1	0	4	2	3	1
15842	201	1	13-Mar-13	16	1	17	2	1	1	0	4	2	3	1
15843	201	1	20-Dec-12	19	1	17	2	2	1	0	4	2	2	1
15844	101	1	26-May-12	15	1	17	2	1	1	0	4	2	2	4
15845	101	1	18-Aug-12	22	1	17	2	1	1	0	4	2	1	1
15846	101	1	22-Mar-12	17	1	17	2	1	1	0	4	2	1	1
15847	101	1	16-Jul-12	17	1	17	2	1	1	0	4	2	2	1
15848	201	1	18-Aug-12	19	1	17	2	2	1	0	4	2	1	1
15849	101	1	10-Jan-12	17	1	17	2	1	1	0	4	2	1	1
15850	101	1	21-Nov-11	11	1	17	2	1	1	0	4	2	2	1
15851	101	1	23-May-11	17	1	17	2	1	1	0	4	2	1	1
15852	101	1	21-Feb-11	17	1	17	2	1	1	0	4	2	3	1
15853	101	1	9-Sep-11	18	1	17	2	1	1	0	4	2	3	1
15854	101	1	8-May-11	17	1	17	2	1	1	0	4	2	3	2
15855	201	1	16-Aug-11	13	1	17	2	2	1	0	4	2	1	1
15856	201	1	22-Nov-11	11	1	17	2	3	1	0	4	2	2	1
15857	101	1	11-Mar-11	18	1	17	2	1	1	0	4	2	4	1
15858	101	1	11-Nov-10	17	1	17	2	1	1	0	4	2	1	1
15859	101	1	13-Apr-10	17	1	17	2	1	1	0	4	2	1	1
15860	201	1	4-Jul-10	10	1	17	2	2	1	0	4	2	3	1
15861	201	1	1-Sep-10	6	1	17	2	2	1	0	4	2	3	1
15862	101	1	13-Jun-10	19	1	17	2	1	1	0	4	2	1	1
15863	101	1	25-May-10	18	1	17	2	1	1	0	4	2	4	1
15864	201	1	1-Apr-10	17	1	17	2	2	1	0	4	2	3	1
15865	201	1	10-Dec-10	14	1	17	2	1	1	0	4	2	2	1

15866	201	1	8-Sep-09	21	1	17	2	2	1	0	4	2	3	1
15867	101	1	30-Mar-09	22	1	17	2	1	1	0	4	2	1	1
15868	101	1	23-Jan-09	17	1	17	2	1	1	0	4	2	1	4
15869	101	1	27-Mar-09	18	1	17	2	1	1	0	4	2	1	1
15870	101	1	4-Jun-09	14	1	17	2	1	1	0	4	2	2	1
15871	101	1	1-Apr-09	17	1	17	2	1	1	0	4	2	4	2
15872	101	1	4-Nov-09	17	1	17	2	1	1	0	4	2	1	1
15873	101	1	12-Oct-09	18	1	17	2	1	1	0	4	2	1	1
15874	101	1	18-Feb-09	17	1	17	2	1	1	0	4	2	4	1
15875	101	1	16-Mar-09	9	1	17	2	1	1	0	4	2	2	1
15876	201	1	1-Apr-08	17	1	17	2	1	1	0	4	2	4	1
15877	101	1	1-Jul-08	21	1	17	2	1	1	0	4	2	2	1
15878	201	1	24-Feb-08	16	1	17	2	1	1	0	4	2	2	1
15879	101	1	23-Jun-08	19	1	17	2	1	1	0	4	2	2	1
15880	101	1	5-Jul-08	15	1	17	2	1	1	0	4	2	1	4
15881	101	1	20-Oct-08	17	1	17	2	1	1	0	4	2	3	1
15882	101	1	17-Feb-08	17	1	17	2	1	1	0	4	2	2	1
15883	101	1	21-Nov-08	9	1	17	2	1	1	0	4	2	3	1
15884	101	1	28-May-08	16	1	17	2	1	1	0	4	2	1	2
15885	201	1	7-Nov-08	17	1	17	2	3	1	0	4	2	3	1
15886	201	1	2-Jul-07	19	1	17	2	1	1	0	4	2	2	1
15887	101	1	19-Jun-07	15	1	17	2	1	1	0	4	2	1	1
15888	201	1	28-Jun-07	17	1	17	2	1	1	0	4	2	1	1
15889	201	1	28-Mar-07	22	1	17	2	1	1	0	4	2	4	1
15890	101	1	19-Mar-07	18	1	17	2	1	1	0	4	2	1	1
15891	101	1	3-Mar-07	17	1	17	2	1	1	0	4	2	1	1
15892	201	1	26-Mar-07	15	1	17	2	1	1	0	4	2	3	1
15893	101	1	16-Sep-07	16	1	17	2	1	1	0	4	2	4	1
15894	101	1	10-Mar-07	19	1	17	2	1	1	0	4	2	4	1
15895	101	1	24-Jun-07	17	1	17	2	1	1	0	4	2	1	1
15896	101	1	7-Nov-07	17	1	17	2	1	1	0	4	2	2	2
15897	101	1	18-Aug-07	21	1	17	2	1	1	0	4	2	1	1
15898	201	1	28-Jun-06	0	1	17	2	1	1	0	4	2	2	1
15899	201	1	21-Sep-06	10	1	17	2	1	1	0	4	2	2	1
15900	101	1	8-Oct-06	15	1	17	2	1	1	0	4	2	6	1
15901	201	1	23-Oct-06	18	1	17	2	1	1	0	4	2	2	1
15902	101	1	19-Jun-06	17	1	17	2	1	1	0	4	2	2	2
15903	201	1	2-Sep-06	20	1	17	2	3	1	0	4	2	1	1
15904	101	1	19-May-06	23	1	17	2	1	1	0	4	2	4	1
15905	101	1	29-Dec-06	17	1	17	2	3	1	0	4	2	1	1
15906	101	1	27-Sep-06	7	1	17	2	1	1	0	4	2	3	1
15907	101	1	10-Apr-17	8	1	17	2	1	1	0	4	2	5	1
15908	101	1	28-Mar-17	19	1	17	2	1	1	0	4	2	4	1
15909	101	1	8-Aug-17	21	1	17	2	1	1	0	4	2	4	1
15910	101	1	31-Mar-17	17	1	17	2	1	1	0	4	2	3	1
15911	101	1	31-Mar-17	18	1	17	2	1	1	0	4	2	3	1
15912	101	1	4-Apr-17	12	1	17	2	1	1	0	4	2	1	2
15913	101	1	30-Oct-17	16	1	17	2	1	1	0	4	2	2	1
15914	201	1	28-Jul-16	8	1	17	2	3	1	0	11	2	3	1
15915	101	1	31-Jul-16	8	1	17	2	1	1	0	11	2	2	1
15916	101	1	24-Jun-16	18	1	17	2	1	1	0	11	2	3	1
15917	101	1	9-Mar-16	14	1	17	2	1	1	0	11	2	3	1
15918	101	1	27-Feb-16	20	1	17	2	1	1	0	11	2	3	1
15919	101	1	28-May-16	10	1	17	2	1	1	0	11	2	2	1
15920	101	1	17-Feb-15	19	1	17	2	1	1	0	11	2	2	1
15921	201	1	18-Jul-15	14	1	17	2	1	1	0	11	2	3	1
15922	101	1	10-Feb-15	19	1	17	2	1	1	0	11	2	2	1
15923	101	1	13-Jan-15	10	1	17	2	1	1	0	11	2	2	1
15924	201	1	5-Jan-15	16	1	17	2	1	1	0	11	2	2	1
15925	101	1	26-Sep-14	16	1	17	2	1	1	0	11	2	2	1
15926	101	1	7-Nov-14	21	1	17	2	1	1	0	11	2	3	1
15927	201	1	21-Jan-14	19	1	17	2	1	1	0	11	2	2	1
15928	101	1	3-Feb-14	20	1	17	2	1	1	0	11	2	3	1
15929	101	1	5-Feb-14	20	1	17	2	1	1	0	11	2	2	1
15930	201	1	9-Jul-13	11	1	17	2	2	1	0	11	2	2	1
15931	201	1	21-Nov-13	17	1	17	2	1	1	0	11	2	2	1
15932	201	1	9-Jul-13	20	1	17	2	3	1	0	11	2	2	1
15933	101	1	7-Aug-13	18	1	17	2	3	1	0	11	2	3	1
15934	101	1	29-Mar-12	11	1	17	2	1	1	0	11	2	2	1
15935	101	1	18-Dec-12	13	1	17	2	1	1	0	11	2	2	1
15936	101	1	5-Jan-12	17	1	17	2	1	1	0	11	2	4	1
15937	101	1	20-May-12	11	1	17	2	1	1	0	11	2	2	1
15938	101	1	23-Nov-12	8	1	17	2	1	1	0	11	2	2	1
15939	101	1	10-Nov-12	22	1	17	2	1	1	0	11	2	2	1
15940	201	1	27-Apr-11	17	1	17	2	2	1	0	11	2	2	1
15941	201	1	12-Apr-11	21	1	17	2	2	1	0	11	2	2	1
15942	101	1	28-Oct-11	21	1	17	2	1	1	0	11	2	3	1
15943	101	1	7-Jun-11	22	1	17	2	1	1	0	11	2	3	1
15944	101	1	2-Aug-10	23	1	17	2	1	1	0	11	2	5	1
15945	201	1	28-Oct-10	9	1	17	2	2	1	0	11	2	2	1
15946	101	1	17-Nov-10	15	1	17	2	1	1	0	11	2	2	2
15947	101	1	29-Mar-10	12	1	17	2	1	1	0	11	2	5	2
15948	101	1	7-Aug-10	20	1	17	2	1	1	0	11	2	5	1
15949	201	1	21-Dec-10	9	1	17	2	2	1	0	11	2	2	1
15950	101	1	30-Aug-09	8	1	17	2	1	1	0	11	2	2	1
15951	201	1	5-Mar-08	22	1	17	2	1	1	0	11	2	3	1
15952	201	1	10-Oct-07	21	1	17	2	1	1	0	11	2	2	1
15953	101	1	10-Sep-07	12	1	17	2	1	1	0	11	2	2	1
15954	201	1	30-Nov-17	12	1	17	2	1	1	0	11	2	3	1
15955	101	1	24-Jan-17	15	1	17	2	1	1	0	11	2	2	1
15956	201	1	6-Mar-17	17	1	17	2	1	1	0	11	2	2	1
15957	201	1	30-Sep-17	14	1	17	2	2	1	0	11	2	3	1
15958	201	1	22-Apr-17	12	1	17	2	3	1	0	11	2	2	1
15959	101	1	14-Oct-17	15	1	17	2	1	1	0	11	2	3	1
15960	101	1	3-Oct-17	12	1	17	2	1	1	0	11	2	3	1
15961	101	1	3-Aug-17	23	1	17	2	1	1	0	11	2	2	1
15962	101	1	6-Nov-16	23	1	17	2	1	1	0	3	2	2	1
15963	101	1	24-Nov-16	3	1	17	2	1	1	0	3	2	1	1
15964	201	1	10-Aug-16	9	1	17	2	2	1	0	3	2	2	1
15965	201	1	30-Nov-16	11	1	17	2	2	1	0	3	2	2	1
15966	101	1	20-Jul-16	22	1	17	2	1	1	0	3	2	1	1
15967	101	1	22-Mar-16	15	1	17	2	1	1	0	3	2	3	1
15968	101	1	19-Mar-16	1	1	17	2	1	1	0	3	2	5	1
15969	101	1	21-Jul-16	12	1	17	2	1	1	0	3	2	1	1
15970	101	1	22-Mar-16	11	1	17	2	1	1	0	3	2	3	1
15971	101	1	3-Mar-16	18	1	17	2	1	1	0	3	2	5	1
15972	101	1	30-Jun-16	16	1	17	2	1	1	0	3	2	1	1
15973	101	1	26-Feb-16	16	1	17	2	1	1	0	3	2	3	1
15974	101	1	20-Feb-16	22	1	17	2	1	1	0	3	2	3	2
15975	101	1	5-May-16	19	1	17	2	1	1	0	3	2	2	1
15976	101	1	24-Mar-16	16	1	17	2	1	1	0	3	2	3	1
15977	101	1	23-Jun-16	16	1	17	2	1	1	0	3	2	4	2
15978	101	1	4-Mar-16	18	1	17	2	1	1	0	3	2	1	1
15979	201	1	24-Oct-16	11	1	17	2	2	1	0	3	2	1	1
15980	101	1	30-Oct-16	17	1	17	2	1	1	0	3	2	1	1
15981	101	1	27-Sep-16	16	1	17	2	1	1	0	3	2	1	1
15982	101	1	1-Nov-16	19	1	17	2	1	1	0	3	2	3	1
15983	201	1	6-Dec-16	15	1	17	2	3	1	0	3	2	3	1
15984	201	1	10-Mar-16	14	1	17	2	2	1	0	3	2	4	1
15985	201	1	19-Feb-16	11	1	17	2	1	1	0	3	2	2	1
15986	101	1	8-Dec-16	19	1	17	2	1	1	0	3	2	3	1
15987	101	1	8-Dec-16	18	1	17	2	1	1	0	3	2	3	1
15988	101	1	16-Apr-16	20	1	17	2	1	1	0	3	2	3	1
15989	101	1	5-Nov-16	14	1	17	2	1	1	0	3	2	4	1

15990	201	1	29-Nov-16	16	1	17	2	1	1	0	3	2	3	1
15991	101	1	9-Jul-16	20	1	17	2	1	1	0	3	2	2	2
15992	101	1	14-Aug-16	14	1	17	2	1	1	0	3	2	2	4
15993	101	1	29-Mar-16	18	1	17	2	1	1	0	3	2	3	1
15994	101	1	9-Dec-16	19	1	17	2	1	1	0	3	2	3	1
15995	101	1	14-Jul-16	19	1	17	2	1	1	0	3	2	3	1
15996	101	1	18-Jan-16	15	1	17	2	1	1	0	3	2	5	1
15997	201	1	20-May-16	13	1	17	2	3	1	0	3	2	1	1
15998	201	1	31-Dec-16	12	1	17	2	1	1	0	3	2	3	1
15999	201	1	19-Jun-16	18	1	17	2	3	1	0	3	2	3	1
16000	201	1	19-Sep-16	16	1	17	2	2	1	0	3	2	2	1
16001	101	1	12-Oct-16	19	1	17	2	1	1	0	3	2	3	1
16002	201	1	19-May-16	15	1	17	2	1	1	0	3	2	3	1
16003	101	1	28-Feb-16	19	1	17	2	1	1	0	3	2	2	1
16004	101	1	8-Apr-16	10	1	17	2	1	1	0	3	2	3	1
16005	101	1	23-Nov-15	22	1	17	2	1	1	0	3	2	3	2
16006	101	1	2-Jun-15	16	1	17	2	1	1	0	3	2	7	1
16007	201	1	25-Feb-15	18	1	17	2	1	1	0	3	2	3	1
16008	201	1	9-Nov-15	18	1	17	2	1	1	0	3	2	3	1
16009	101	1	12-Mar-15	10	1	17	2	1	1	0	3	2	4	1
16010	101	1	7-Sep-15	11	1	17	2	1	1	0	3	2	1	1
16011	201	1	2-Oct-15	10	1	17	2	3	1	0	3	2	1	1
16012	101	1	18-May-15	17	1	17	2	1	1	0	3	2	3	1
16013	201	1	14-Feb-15	14	1	17	2	3	1	0	3	2	3	1
16014	101	1	31-Jan-15	23	1	17	2	3	1	0	3	2	2	1
16015	101	1	30-Jan-15	23	1	17	2	1	1	0	3	2	3	1
16016	101	1	13-Mar-15	16	1	17	2	1	1	0	3	2	3	1
16017	101	1	19-Jul-15	17	1	17	2	1	1	0	3	2	4	1
16018	101	1	12-Dec-15	16	1	17	2	1	1	0	3	2	3	1
16019	101	1	1-May-15	11	1	17	2	1	1	0	3	2	3	1
16020	101	1	10-Dec-15	20	1	17	2	1	1	0	3	2	2	4
16021	201	1	9-Mar-15	7	1	17	2	3	1	0	3	2	1	1
16022	201	1	23-Jul-15	10	1	17	2	2	1	0	3	2	1	1
16023	101	1	20-Nov-15	16	1	17	2	1	1	0	3	2	1	1
16024	201	1	1-May-15	19	1	17	2	1	1	0	3	2	3	1
16025	101	1	20-Nov-15	20	1	17	2	1	1	0	3	2	2	1
16026	201	1	22-Jul-15	19	1	17	2	1	1	0	3	2	1	1
16027	201	1	10-Apr-15	18	1	17	2	1	1	0	3	2	2	1
16028	101	1	29-Dec-15	21	1	17	2	1	1	0	3	2	3	1
16029	101	1	22-Nov-15	12	1	17	2	1	1	0	3	2	3	1
16030	201	1	8-May-15	11	1	17	2	1	1	0	3	2	2	1
16031	101	1	27-May-15	17	1	17	2	1	1	0	3	2	3	1
16032	201	1	17-Mar-15	16	1	17	2	2	1	0	3	2	3	1
16033	101	1	23-Apr-15	19	1	17	2	1	1	0	3	2	3	1
16034	201	1	25-Jul-15	17	1	17	2	3	1	0	3	2	2	1
16035	101	1	19-Jan-15	9	1	17	2	1	1	0	3	2	1	1
16036	101	1	18-Feb-15	20	1	17	2	1	1	0	3	2	3	1
16037	201	1	8-May-14	15	1	17	2	3	1	0	3	2	1	1
16038	201	1	23-Oct-14	9	1	17	2	1	1	0	3	2	3	1
16039	201	1	25-Jun-14	16	1	17	2	3	1	0	3	2	3	1
16040	101	1	8-May-14	18	1	17	2	1	1	0	3	2	4	1
16041	101	1	24-Mar-14	17	1	17	2	1	1	0	3	2	4	1
16042	101	1	15-Jun-14	18	1	17	2	1	1	0	3	2	3	1
16043	101	1	8-May-14	21	1	17	2	1	1	0	3	2	2	1
16044	101	1	10-Mar-14	17	1	17	2	1	1	0	3	2	2	4
16045	101	1	10-Mar-14	10	1	17	2	1	1	0	3	2	2	1
16046	101	1	22-Oct-14	7	1	17	2	1	1	0	3	2	2	1
16047	101	1	10-Mar-14	15	1	17	2	1	1	0	3	2	3	1
16048	101	1	1-Oct-14	9	1	17	2	1	1	0	3	2	1	2
16049	201	1	29-Aug-14	20	1	17	2	2	1	0	3	2	5	1
16050	101	1	27-Dec-14	21	1	17	2	1	1	0	3	2	1	1
16051	101	1	18-May-14	0	1	17	2	1	1	0	3	2	1	1
16052	101	1	23-Dec-14	21	1	17	2	1	1	0	3	2	1	1
16053	101	1	24-May-14	21	1	17	2	1	1	0	3	2	4	1
16054	201	1	24-Oct-14	9	1	17	2	2	1	0	3	2	4	2
16055	101	1	16-Mar-14	15	1	17	2	1	1	0	3	2	3	1
16056	201	1	9-Jul-14	23	1	17	2	2	1	0	3	2	1	1
16057	101	1	25-Jun-14	18	1	17	2	1	1	0	3	2	3	4
16058	101	1	15-Sep-14	16	1	17	2	1	1	0	3	2	3	1
16059	201	1	11-Aug-14	19	1	17	2	3	1	0	3	2	3	2
16060	101	1	9-Nov-14	8	1	17	2	1	1	0	3	2	1	1
16061	101	1	1-May-14	17	1	17	2	1	1	0	3	2	2	1
16062	101	1	7-Oct-14	23	1	17	2	1	1	0	3	2	2	1
16063	101	1	7-Jun-14	12	1	17	2	1	1	0	3	2	3	1
16064	101	1	26-Sep-14	13	1	17	2	1	1	0	3	2	3	1
16065	101	1	4-Jun-14	15	1	17	2	1	1	0	3	2	3	1
16066	101	1	2-May-14	20	1	17	2	1	1	0	3	2	3	4
16067	201	1	28-Mar-14	18	1	17	2	2	1	0	3	2	1	2
16068	101	1	27-Oct-14	11	1	17	2	1	1	0	3	2	4	4
16069	201	1	10-May-14	14	1	17	2	3	1	0	3	2	1	1
16070	101	1	10-Nov-14	9	1	17	2	1	1	0	3	2	3	1
16071	101	1	10-Sep-14	18	1	17	2	1	1	0	3	2	3	1
16072	101	1	5-Apr-14	22	1	17	2	1	1	0	3	2	3	1
16073	101	1	19-Apr-14	22	1	17	2	1	1	0	3	2	2	1
16074	201	1	9-Apr-14	16	1	17	2	3	1	0	3	2	2	1
16075	101	1	20-Mar-14	17	1	17	2	1	1	0	3	2	2	1
16076	101	1	28-Mar-14	16	1	17	2	1	1	0	3	2	3	1
16077	101	1	16-Apr-14	9	1	17	2	1	1	0	3	2	1	1
16078	101	1	22-Apr-14	10	1	17	2	1	1	0	3	2	2	1
16079	101	1	1-May-14	10	1	17	2	1	1	0	3	2	3	1
16080	101	1	18-Jun-14	17	1	17	2	1	1	0	3	2	3	1
16081	101	1	18-Sep-14	19	1	17	2	1	1	0	3	2	3	1
16082	101	1	13-Oct-14	17	1	17	2	1	1	0	3	2	3	2
16083	201	1	24-Mar-14	15	1	17	2	2	1	0	3	2	2	1
16084	101	1	27-Feb-14	16	1	17	2	1	1	0	3	2	2	1
16085	201	1	22-Dec-14	10	1	17	2	3	1	0	3	2	2	1
16086	101	1	24-Aug-14	19	1	17	2	1	1	0	3	2	3	1
16087	201	1	26-Mar-14	12	1	17	2	1	1	0	3	2	1	1
16088	201	1	20-Apr-14	9	1	17	2	3	1	0	3	2	2	1
16089	201	1	13-May-14	10	1	17	2	1	1	0	3	2	3	1
16090	201	1	26-Nov-14	14	1	17	2	3	1	0	3	2	3	2
16091	201	1	22-Oct-14	8	1	17	2	2	1	0	3	2	2	1
16092	201	1	10-Sep-14	18	1	17	2	2	1	0	3	2	3	1
16093	101	1	23-Oct-14	16	1	17	2	1	1	0	3	2	2	1
16094	201	1	1-Jan-14	16	1	17	2	3	1	0	3	2	2	1
16095	201	1	10-Feb-14	16	1	17	2	3	1	0	3	2	3	2
16096	101	1	4-Nov-14	17	1	17	2	1	1	0	3	2	2	2
16097	101	1	25-Mar-14	17	1	17	2	1	1	0	3	2	3	1
16098	201	1	16-Apr-14	11	1	17	2	1	1	0	3	2	3	1
16099	201	1	8-Jan-14	14	1	17	2	1	1	0	3	2	2	1
16100	101	1	8-Jun-13	15	1	17	2	1	1	0	3	2	1	1
16101	101	1	24-May-13	16	1	17	2	1	1	0	3	2	3	1
16102	101	1	30-Oct-13	22	1	17	2	1	1	0	3	2	2	1
16103	101	1	29-Sep-13	11	1	17	2	1	1	0	3	2	3	1
16104	101	1	24-Mar-13	17	1	17	2	1	1	0	3	2	3	1
16105	101	1	19-Feb-13	17	1	17	2	1	1	0	3	2	3	1
16106	201	1	31-Jan-13	8	1	17	2	2	1	0	3	2	2	1
16107	101	1	10-Oct-13	12	1	17	2	1	1	0	3	2	2	1
16108	101	1	18-Nov-13	19	1	17	2	1	1	0	3	2	3	1
16109	101	1	26-Jan-13	18	1	17	2	1	1	0	3	2	1	1
16110	201	1	9-May-13	19	1	17	2	1	1	0	3	2	1	2
16111	101	1	17-Feb-13	18	1	17	2	1	1	0	3	2	1	1
16112	101	1	4-Mar-13	16	1	17	2	1	1	0	3	2	1	1
16113	201	1	9-Apr-13	21	1	17	2	2	1	0	3	2	1	1

16114	101	1	19-Jun-13	20	1	17	2	1	1	0	3	2	3	1
16115	101	1	14-Mar-13	22	1	17	2	1	1	0	3	2	3	1
16116	101	1	9-Aug-13	22	1	17	2	1	1	0	3	2	3	1
16117	101	1	21-May-13	19	1	17	2	1	1	0	3	2	2	1
16118	101	1	1-Oct-13	11	1	17	2	3	1	0	3	2	2	1
16119	101	1	29-Apr-13	12	1	17	2	1	1	0	3	2	3	1
16120	101	1	9-Nov-13	20	1	17	2	1	1	0	3	2	2	1
16121	101	1	21-Jul-13	21	1	17	2	1	1	0	3	2	2	1
16122	101	1	31-May-13	11	1	17	2	2	1	0	3	2	3	1
16123	101	1	14-Mar-13	21	1	17	2	1	1	0	3	2	3	1
16124	101	1	18-Apr-13	11	1	17	2	1	1	0	3	2	2	1
16125	101	1	28-Dec-13	22	1	17	2	1	1	0	3	2	1	1
16126	201	1	1-Mar-13	15	1	17	2	3	1	0	3	2	1	1
16127	101	1	29-Apr-13	10	1	17	2	1	1	0	3	2	2	2
16128	101	1	14-Apr-13	10	1	17	2	2	1	0	3	2	3	1
16129	101	1	15-Mar-13	17	1	17	2	1	1	0	3	2	3	1
16130	101	1	20-May-13	10	1	17	2	1	1	0	3	2	3	1
16131	101	1	17-Jan-13	18	1	17	2	1	1	0	3	2	2	1
16132	101	1	12-Aug-13	17	1	17	2	1	1	0	3	2	3	1
16133	201	1	14-Jun-13	17	1	17	2	2	1	0	3	2	1	1
16134	101	1	18-Jun-13	16	1	17	2	1	1	0	3	2	3	1
16135	101	1	16-Oct-13	16	1	17	2	1	1	0	3	2	3	1
16136	101	1	12-Apr-13	15	1	17	2	1	1	0	3	2	3	1
16137	101	1	23-Apr-13	17	1	17	2	1	1	0	3	2	3	1
16138	101	1	15-Apr-13	18	1	17	2	1	1	0	3	2	3	1
16139	101	1	21-Sep-13	11	1	17	2	1	1	0	3	2	3	1
16140	101	1	22-Jun-13	19	1	17	2	1	1	0	3	2	2	1
16141	101	1	18-Sep-13	22	1	17	2	1	1	0	3	2	1	1
16142	201	1	30-Sep-13	17	1	17	2	3	1	0	3	2	2	1
16143	101	1	25-Feb-13	17	1	17	2	1	1	0	3	2	2	2
16144	201	1	9-Oct-13	11	1	17	2	1	1	0	3	2	1	1
16145	201	1	16-Apr-13	17	1	17	2	3	1	0	3	2	1	1
16146	201	1	12-Apr-13	8	1	17	2	1	1	0	3	2	1	1
16147	201	1	17-Dec-13	20	1	17	2	3	1	0	3	2	2	1
16148	201	1	27-Apr-13	14	1	17	2	2	1	0	3	2	3	1
16149	101	1	17-May-13	19	1	17	2	1	1	0	3	2	3	1
16150	101	1	26-Feb-13	16	1	17	2	1	1	0	3	2	3	1
16151	101	1	25-Dec-13	19	1	17	2	1	1	0	3	2	2	1
16152	201	1	21-Mar-13	10	1	17	2	2	1	0	3	2	3	2
16153	101	1	25-Jun-13	17	1	17	2	1	1	0	3	2	2	4
16154	101	1	22-Mar-13	9	1	17	2	1	1	0	3	2	3	1
16155	201	1	3-Dec-12	14	1	17	2	3	1	0	3	2	1	1
16156	201	1	27-Aug-12	17	1	17	2	3	1	0	3	2	2	2
16157	101	1	19-Oct-12	16	1	17	2	1	1	0	3	2	3	1
16158	101	1	7-Nov-12	16	1	17	2	1	1	0	3	2	5	1
16159	201	1	14-May-12	18	1	17	2	1	1	0	3	2	2	1
16160	201	1	1-Dec-12	10	1	17	2	2	1	0	3	2	1	1
16161	201	1	13-Sep-12	15	1	17	2	2	1	0	3	2	3	1
16162	101	1	7-Feb-12	17	1	17	2	1	1	0	3	2	3	1
16163	101	1	16-Mar-12	17	1	17	2	1	1	0	3	2	3	1
16164	101	1	23-May-12	18	1	17	2	1	1	0	3	2	1	1
16165	101	1	10-May-12	17	1	17	2	1	1	0	3	2	5	1
16166	101	1	12-Oct-12	14	1	17	2	1	1	0	3	2	5	1
16167	101	1	5-Sep-12	14	1	17	2	1	1	0	3	2	1	1
16168	101	1	20-May-12	17	1	17	2	1	1	0	3	2	1	1
16169	101	1	16-Dec-12	15	1	17	2	1	1	0	3	2	4	1
16170	101	1	11-Jul-12	7	1	17	2	1	1	0	3	2	4	1
16171	101	1	7-Jun-12	3	1	17	2	1	1	0	3	2	3	1
16172	101	1	14-Sep-12	17	1	17	2	1	1	0	3	2	2	1
16173	101	1	5-Aug-12	17	1	17	2	1	1	0	3	2	2	1
16174	101	1	15-Sep-12	8	1	17	2	1	1	0	3	2	3	1
16175	101	1	15-Sep-12	8	1	17	2	1	1	0	3	2	3	1
16176	101	1	19-Aug-12	17	1	17	2	3	1	0	3	2	2	1
16177	101	1	28-Oct-12	10	1	17	2	1	1	0	3	2	2	4
16178	201	1	21-Mar-12	22	1	17	2	2	1	0	3	2	3	2
16179	101	1	4-Dec-12	1	1	17	2	1	1	0	3	2	3	1
16180	101	1	22-Aug-12	22	1	17	2	1	1	0	3	2	3	1
16181	101	1	15-Mar-12	18	1	17	2	1	1	0	3	2	1	1
16182	101	1	5-Dec-12	17	1	17	2	1	1	0	3	2	3	1
16183	101	1	29-May-12	17	1	17	2	1	1	0	3	2	2	1
16184	101	1	20-Nov-12	16	1	17	2	1	1	0	3	2	2	1
16185	201	1	18-Oct-12	8	1	17	2	1	1	0	3	2	1	1
16186	101	1	13-Aug-12	14	1	17	2	1	1	0	3	2	2	1
16187	101	1	19-Jun-12	17	1	17	2	1	1	0	3	2	3	1
16188	101	1	8-May-12	17	1	17	2	1	1	0	3	2	3	1
16189	201	1	29-May-12	18	1	17	2	1	1	0	3	2	3	1
16190	201	1	17-Aug-12	19	1	17	2	1	1	0	3	2	3	1
16191	101	1	8-Apr-12	10	1	17	2	1	1	0	3	2	3	1
16192	101	1	18-Dec-12	18	1	17	2	1	1	0	3	2	3	1
16193	201	1	9-Apr-12	18	1	17	2	1	1	0	3	2	3	1
16194	101	1	23-Oct-12	13	1	17	2	1	1	0	3	2	2	4
16195	101	1	26-Sep-12	17	1	17	2	1	1	0	3	2	2	1
16196	101	1	24-Mar-12	18	1	17	2	1	1	0	3	2	2	1
16197	101	1	15-May-12	7	1	17	2	1	1	0	3	2	2	1
16198	201	1	29-May-12	17	1	17	2	2	1	0	3	2	3	1
16199	201	1	23-Jun-12	16	1	17	2	2	1	0	3	2	1	2
16200	201	1	7-Dec-12	9	1	17	2	3	1	0	3	2	1	2
16201	101	1	5-Apr-12	17	1	17	2	1	1	0	3	2	1	1
16202	201	1	26-Nov-12	15	1	17	2	1	1	0	3	2	1	1
16203	201	1	4-May-12	10	1	17	2	2	1	0	3	2	3	1
16204	101	1	5-Dec-12	17	1	17	2	1	1	0	3	2	3	1
16205	201	1	26-Jun-12	10	1	17	2	1	1	0	3	2	3	1
16206	201	1	8-Jun-12	13	1	17	2	1	1	0	3	2	2	1
16207	101	1	14-Jun-12	17	1	17	2	1	1	0	3	2	3	1
16208	201	1	15-Nov-12	20	1	17	2	3	1	0	3	2	4	1
16209	201	1	4-Oct-12	10	1	17	2	2	1	0	3	2	2	2
16210	201	1	11-Jun-11	4	1	17	2	1	1	0	3	2	3	1
16211	201	1	11-May-11	14	1	17	2	1	1	0	3	2	3	1
16212	101	1	23-Feb-11	18	1	17	2	1	1	0	3	2	3	1
16213	101	1	10-Mar-11	10	1	17	2	1	1	0	3	2	1	1
16214	101	1	16-Jun-11	17	1	17	2	1	1	0	3	2	1	1
16215	101	1	16-Jun-11	17	1	17	2	1	1	0	3	2	7	1
16216	201	1	17-Dec-11	9	1	17	2	2	1	0	3	2	4	2
16217	201	1	10-Nov-11	14	1	17	2	1	1	0	3	2	2	1
16218	101	1	26-Nov-11	16	1	17	2	1	1	0	3	2	2	1
16219	201	1	27-Apr-11	17	1	17	2	2	1	0	3	2	2	1
16220	101	1	5-Aug-11	16	1	17	2	1	1	0	3	2	2	1
16221	101	1	26-Mar-11	18	1	17	2	1	1	0	3	2	1	1
16222	201	1	14-Feb-11	17	1	17	2	2	1	0	3	2	1	1
16223	201	1	30-Jul-11	6	1	17	2	3	1	0	3	2	1	1
16224	101	1	14-Dec-11	17	1	17	2	1	1	0	3	2	1	1
16225	101	1	15-Sep-11	20	1	17	2	1	1	0	3	2	3	1
16226	201	1	7-May-11	17	1	17	2	2	1	0	3	2	2	2
16227	101	1	25-Jan-11	15	1	17	2	1	1	0	3	2	3	2
16228	101	1	11-Aug-11	17	1	17	2	1	1	0	3	2	3	1
16229	101	1	28-Feb-11	10	1	17	2	1	1	0	3	2	3	1
16230	101	1	8-Nov-11	16	1	17	2	1	1	0	3	2	2	1
16231	101	1	26-May-11	17	1	17	2	1	1	0	3	2	4	1
16232	101	1	18-Jun-11	19	1	17	2	1	1	0	3	2	2	1
16233	101	1	7-Oct-11	20	1	17	2	1	1	0	3	2	2	1
16234	101	1	23-Aug-11	23	1	17	2	1	1	0	3	2	1	1
16235	101	1	19-Feb-11	14	1	17	2	3	1	0	3	2	2	1
16236	201	1	26-Oct-11	9	1	17	2	2	1	0	3	2	3	1
16237	101	1	31-Mar-11	9	1	17	2	1	1	0	3	2	2	1

16238	101	1	5-Jan-11	18	1	17	2	1	1	0	3	2	2	1
16239	101	1	13-Sep-11	17	1	17	2	1	1	0	3	2	3	1
16240	101	1	12-Apr-11	18	1	17	2	1	1	0	3	2	3	1
16241	201	1	11-Mar-11	18	1	17	2	2	1	0	3	2	3	1
16242	101	1	3-May-11	10	1	17	2	1	1	0	3	2	3	1
16243	101	1	8-Apr-11	16	1	17	2	1	1	0	3	2	3	1
16244	201	1	26-Dec-11	8	1	17	2	1	1	0	3	2	2	1
16245	101	1	12-Apr-11	11	1	17	2	1	1	0	3	2	3	1
16246	201	1	16-Aug-10	17	1	17	2	1	1	0	3	2	2	1
16247	101	1	11-May-10	22	1	17	2	1	1	0	3	2	5	1
16248	201	1	18-Jul-10	23	1	17	2	2	1	0	3	2	1	1
16249	201	1	14-May-10	20	1	17	2	1	1	0	3	2	1	1
16250	101	1	4-Jun-10	19	1	17	2	1	1	0	3	2	3	1
16251	101	1	24-May-10	1	1	17	2	1	1	0	3	2	3	1
16252	101	1	2-Mar-10	12	1	17	2	3	1	0	3	2	2	1
16253	201	1	27-Jan-10	11	1	17	2	2	1	0	3	2	2	1
16254	101	1	6-Oct-10	7	1	17	2	1	1	0	3	2	6	1
16255	201	1	30-Jun-10	23	1	17	2	3	1	0	3	2	4	1
16256	201	1	23-Nov-10	18	1	17	2	2	1	0	3	2	3	1
16257	101	1	1-Dec-10	10	1	17	2	1	1	0	3	2	3	1
16258	101	1	3-Jun-10	21	1	17	2	1	1	0	3	2	2	1
16259	101	1	27-Jun-10	9	1	17	2	1	1	0	3	2	2	1
16260	101	1	15-Jan-10	16	1	17	2	1	1	0	3	2	3	1
16261	101	1	16-Jan-10	22	1	17	2	1	1	0	3	2	1	1
16262	101	1	31-Aug-10	18	1	17	2	1	1	0	3	2	2	1
16263	201	1	1-Jun-10	9	1	17	2	2	1	0	3	2	2	1
16264	201	1	1-Oct-10	3	1	17	2	3	1	0	3	2	3	1
16265	101	1	14-Apr-10	18	1	17	2	1	1	0	3	2	1	1
16266	101	1	14-Oct-10	17	1	17	2	1	1	0	3	2	3	1
16267	201	1	6-Aug-10	20	1	17	2	1	1	0	3	2	3	1
16268	101	1	13-May-10	17	1	17	2	1	1	0	3	2	2	1
16269	101	1	4-Nov-10	16	1	17	2	1	1	0	3	2	2	1
16270	101	1	18-Dec-10	18	1	17	2	1	1	0	3	2	2	4
16271	101	1	2-May-10	12	1	17	2	1	1	0	3	2	3	1
16272	101	1	10-Sep-10	18	1	17	2	1	1	0	3	2	3	1
16273	101	1	3-Mar-10	17	1	17	2	2	1	0	3	2	5	1
16274	101	1	29-Dec-10	19	1	17	2	3	1	0	3	2	4	1
16275	101	1	14-Dec-10	11	1	17	2	1	1	0	3	2	1	1
16276	201	1	22-Dec-10	16	1	17	2	1	1	0	3	2	3	1
16277	101	1	16-Apr-10	18	1	17	2	1	1	0	3	2	3	4
16278	101	1	30-Nov-10	17	1	17	2	1	1	0	3	2	2	1
16279	101	1	11-Nov-10	17	1	17	2	1	1	0	3	2	3	1
16280	201	1	8-Oct-10	16	1	17	2	1	1	0	3	2	3	1
16281	201	1	30-Oct-10	20	1	17	2	2	1	0	3	2	2	2
16282	201	1	2-Oct-10	18	1	17	2	1	1	0	3	2	3	1
16283	101	1	9-May-09	20	1	17	2	1	1	0	3	2	1	1
16284	101	1	17-Feb-09	19	1	17	2	1	1	0	3	2	3	1
16285	101	1	20-Dec-09	20	1	17	2	1	1	0	3	2	1	1
16286	101	1	13-May-09	15	1	17	2	1	1	0	3	2	3	1
16287	101	1	13-Jul-09	21	1	17	2	1	1	0	3	2	3	1
16288	101	1	7-Sep-09	20	1	17	2	1	1	0	3	2	2	1
16289	101	1	5-May-09	20	1	17	2	2	1	0	3	2	1	1
16290	201	1	2-Jul-09	16	1	17	2	3	1	0	3	2	4	1
16291	101	1	13-Jun-09	17	1	17	2	1	1	0	3	2	1	1
16292	101	1	9-Feb-09	18	1	17	2	1	1	0	3	2	1	1
16293	101	1	17-Apr-09	20	1	17	2	1	1	0	3	2	1	1
16294	101	1	20-Apr-09	17	1	17	2	1	1	0	3	2	1	1
16295	101	1	10-Nov-09	17	1	17	2	1	1	0	3	2	2	2
16296	101	1	5-May-09	18	1	17	2	2	1	0	3	2	3	1
16297	101	1	4-Feb-09	18	1	17	2	1	1	0	3	2	3	1
16298	201	1	31-Mar-09	20	1	17	2	2	1	0	3	2	3	1
16299	101	1	16-Dec-09	17	1	17	2	1	1	0	3	2	2	1
16300	101	1	19-Mar-09	17	1	17	2	1	1	0	3	2	3	4
16301	101	1	20-Nov-09	18	1	17	2	1	1	0	3	2	3	1
16302	101	1	20-May-09	14	1	17	2	1	1	0	3	2	3	1
16303	101	1	9-Apr-09	18	1	17	2	1	1	0	3	2	4	1
16304	101	1	1-Apr-09	17	1	17	2	1	1	0	3	2	2	2
16305	101	1	10-Nov-09	15	1	17	2	1	1	0	3	2	2	1
16306	101	1	3-Mar-09	17	1	17	2	1	1	0	3	2	3	1
16307	101	1	7-Nov-09	18	1	17	2	1	1	0	3	2	3	1
16308	101	1	3-Sep-09	12	1	17	2	1	1	0	3	2	2	4
16309	201	1	22-Jan-09	18	1	17	2	1	1	0	3	2	2	1
16310	101	1	27-Apr-09	18	1	17	2	1	1	0	3	2	3	1
16311	201	1	28-May-09	16	1	17	2	1	1	0	3	2	3	1
16312	201	1	27-Apr-09	11	1	17	2	1	1	0	3	2	1	1
16313	101	1	27-Feb-09	17	1	17	2	1	1	0	3	2	3	1
16314	101	1	11-Mar-09	18	1	17	2	1	1	0	3	2	2	1
16315	101	1	2-Nov-09	7	1	17	2	1	1	0	3	2	3	1
16316	201	1	6-Oct-09	21	1	17	2	2	1	0	3	2	2	1
16317	201	1	23-Nov-08	16	1	17	2	2	1	0	3	2	4	1
16318	101	1	19-Dec-08	18	1	17	2	1	1	0	3	2	1	1
16319	101	1	1-Dec-08	13	1	17	2	1	1	0	3	2	1	1
16320	101	1	26-Feb-08	15	1	17	2	1	1	0	3	2	1	1
16321	101	1	3-Dec-08	19	1	17	2	1	1	0	3	2	3	1
16322	201	1	26-Mar-08	17	1	17	2	1	1	0	3	2	2	2
16323	101	1	1-Jul-08	12	1	17	2	1	1	0	3	2	3	2
16324	101	1	25-Sep-08	20	1	17	2	1	1	0	3	2	3	1
16325	101	1	4-Oct-08	18	1	17	2	1	1	0	3	2	2	4
16326	101	1	4-May-08	1	1	17	2	1	1	0	3	2	3	1
16327	101	1	8-May-08	11	1	17	2	1	1	0	3	2	2	1
16328	101	1	17-May-08	16	1	17	2	1	1	0	3	2	3	1
16329	101	1	11-Aug-08	23	1	17	2	3	1	0	3	2	3	1
16330	201	1	5-Dec-08	11	1	17	2	1	1	0	3	2	2	2
16331	101	1	23-Oct-08	21	1	17	2	1	1	0	3	2	3	1
16332	101	1	23-Oct-08	18	1	17	2	1	1	0	3	2	2	1
16333	101	1	2-May-08	21	1	17	2	1	1	0	3	2	1	1
16334	101	1	21-Oct-08	18	1	17	2	1	1	0	3	2	1	1
16335	101	1	11-Oct-08	15	1	17	2	1	1	0	3	2	3	1
16336	101	1	9-Dec-08	17	1	17	2	1	1	0	3	2	3	1
16337	101	1	9-Feb-08	18	1	17	2	1	1	0	3	2	3	1
16338	101	1	23-Dec-08	16	1	17	2	1	1	0	3	2	3	1
16339	101	1	30-Aug-08	19	1	17	2	1	1	0	3	2	3	1
16340	101	1	16-Sep-08	17	1	17	2	1	1	0	3	2	2	1
16341	101	1	8-Nov-08	17	1	17	2	1	1	0	3	2	3	1
16342	101	1	7-Jul-07	11	1	17	2	1	1	0	3	2	3	1
16343	101	1	28-Sep-07	17	1	17	2	1	1	0	3	2	2	1
16344	101	1	25-May-07	18	1	17	2	1	1	0	3	2	2	1
16345	101	1	27-Nov-07	22	1	17	2	2	1	0	3	2	1	1
16346	201	1	11-Nov-07	11	1	17	2	1	1	0	3	2	1	1
16347	101	1	22-Dec-07	14	1	17	2	1	1	0	3	2	5	1
16348	101	1	8-Mar-07	13	1	17	2	1	1	0	3	2	1	1
16349	101	1	22-Jul-07	22	1	17	2	1	1	0	3	2	1	1
16350	101	1	22-Nov-07	13	1	17	2	1	1	0	3	2	2	4
16351	201	1	18-Dec-07	15	1	17	2	1	1	0	3	2	3	1
16352	101	1	28-Sep-07	8	1	17	2	1	1	0	3	2	2	4
16353	101	1	23-Oct-07	12	1	17	2	1	1	0	3	2	2	1
16354	101	1	29-Dec-07	15	1	17	2	1	1	0	3	2	3	1
16355	201	1	18-Oct-07	22	1	17	2	1	1	0	3	2	4	1
16356	101	1	12-May-07	10	1	17	2	1	1	0	3	2	1	1
16357	201	1	4-Feb-07	19	1	17	2	1	1	0	3	2	2	1
16358	201	1	25-Jul-07	21	1	17	2	1	1	0	3	2	1	1
16359	101	1	8-Sep-07	17	1	17	2	1	1	0	3	2	3	1
16360	101	1	27-Nov-07	16	1	17	2	1	1	0	3	2	1	1
16361	201	1	13-Jul-07	23	1	17	2	1	1	0	3	2	3	1

16362	101	1	6-Dec-07	17	1	17	2	1	1	0	3	2	3	1
16363	101	1	29-Mar-07	17	1	17	2	1	1	0	3	2	1	1
16364	101	1	12-Mar-07	17	1	17	2	1	1	0	3	2	2	1
16365	101	1	18-Dec-07	20	1	17	2	1	1	0	3	2	3	1
16366	101	1	6-Sep-07	16	1	17	2	1	1	0	3	2	3	1
16367	101	1	23-Jul-07	21	1	17	2	1	1	0	3	2	3	1
16368	101	1	30-Nov-07	9	1	17	2	1	1	0	3	2	4	2
16369	101	1	21-Dec-07	17	1	17	2	1	1	0	3	2	2	1
16370	101	1	15-Apr-07	17	1	17	2	1	1	0	3	2	2	1
16371	101	1	26-Oct-07	17	1	17	2	1	1	0	3	2	2	1
16372	101	1	6-Sep-07	18	1	17	2	1	1	0	3	2	3	1
16373	101	1	30-Nov-07	19	1	17	2	1	1	0	3	2	3	1
16374	101	1	6-Jul-07	22	1	17	2	1	1	0	3	2	3	1
16375	101	1	5-Nov-07	16	1	17	2	1	1	0	3	2	2	1
16376	101	1	21-Sep-07	10	1	17	2	1	1	0	3	2	1	2
16377	201	1	14-May-07	13	1	17	2	1	1	0	3	2	3	1
16378	101	1	27-Nov-07	15	1	17	2	1	1	0	3	2	4	2
16379	101	1	9-Jun-07	17	1	17	2	2	1	0	3	2	3	1
16380	101	1	20-Dec-07	19	1	17	2	1	1	0	3	2	3	1
16381	101	1	28-Sep-07	16	1	17	2	1	1	0	3	2	3	1
16382	101	1	3-Nov-07	7	1	17	2	1	1	0	3	2	2	1
16383	201	1	2-Jul-07	15	1	17	2	1	1	0	3	2	2	1
16384	101	1	6-Dec-07	17	1	17	2	1	1	0	3	2	3	1
16385	201	1	3-Jul-07	15	1	17	2	1	1	0	3	2	2	1
16386	201	1	21-Mar-07	18	1	17	2	1	1	0	3	2	2	1
16387	101	1	18-Mar-06	6	1	17	2	4	1	0	3	2	1	1
16388	101	1	24-Mar-06	17	1	17	2	2	1	0	3	2	1	1
16389	101	1	7-Dec-06	22	1	17	2	1	1	0	3	2	1	1
16390	101	1	8-Sep-06	13	1	17	2	1	1	0	3	2	1	1
16391	101	1	2-Mar-06	11	1	17	2	1	1	0	3	2	7	1
16392	101	1	31-Oct-06	17	1	17	2	1	1	0	3	2	3	1
16393	201	1	3-Sep-06	21	1	17	2	1	1	0	3	2	1	1
16394	101	1	22-Mar-06	19	1	17	2	3	1	0	3	2	3	1
16395	201	1	22-Apr-06	8	1	17	2	3	1	0	3	2	3	1
16396	101	1	18-Aug-06	17	1	17	2	1	1	0	3	2	3	1
16397	201	1	8-Dec-06	17	1	17	2	2	1	0	3	2	3	2
16398	201	1	5-Mar-06	17	1	17	2	2	1	0	3	2	3	1
16399	201	1	27-Jun-06	19	1	17	2	1	1	0	3	2	3	1
16400	101	1	10-Dec-06	15	1	17	2	1	1	0	3	2	3	1
16401	101	1	30-Nov-06	21	1	17	2	1	1	0	3	2	2	1
16402	101	1	27-Sep-06	18	1	17	2	1	1	0	3	2	2	1
16403	201	1	9-Jul-06	18	1	17	2	1	1	0	3	2	1	1
16404	101	1	24-Oct-06	22	1	17	2	1	1	0	3	2	1	1
16405	201	1	30-Oct-06	17	1	17	2	1	1	0	3	2	1	1
16406	101	1	6-Dec-06	17	1	17	2	1	1	0	3	2	2	1
16407	201	1	24-Feb-06	20	1	17	2	1	1	0	3	2	3	2
16408	201	1	8-Oct-06	12	1	17	2	1	1	0	3	2	3	1
16409	101	1	29-Sep-06	17	1	17	2	1	1	0	3	2	2	1
16410	201	1	25-Apr-06	17	1	17	2	1	1	0	3	2	3	1
16411	201	1	16-Sep-06	15	1	17	2	1	1	0	3	2	4	1
16412	101	1	27-Aug-06	19	1	17	2	1	1	0	3	2	2	1
16413	101	1	17-Apr-17	19	1	17	2	1	1	0	3	2	4	1
16414	101	1	8-Feb-17	19	1	17	2	1	1	0	3	2	3	1
16415	101	1	8-Feb-17	17	1	17	2	1	1	0	3	2	3	1
16416	101	1	27-Jan-17	10	1	17	2	1	1	0	3	2	3	1
16417	101	1	29-Jun-17	16	1	17	2	1	1	0	3	2	3	1
16418	101	1	6-Apr-17	21	1	17	2	1	1	0	3	2	3	1
16419	101	1	10-Jun-17	10	1	17	2	1	1	0	3	2	3	1
16420	101	1	23-Nov-17	17	1	17	2	1	1	0	3	2	3	1
16421	101	1	19-Feb-17	12	1	17	2	1	1	0	3	2	2	1
16422	101	1	14-Feb-17	14	1	17	2	1	1	0	3	2	2	1
16423	201	1	1-Jun-17	10	1	17	2	1	1	0	3	2	2	1
16424	201	1	20-Feb-17	17	1	17	2	1	1	0	3	2	2	1
16425	101	1	18-Aug-17	14	1	17	2	1	1	0	3	2	1	1
16426	101	1	11-Feb-17	15	1	17	2	1	1	0	3	2	1	1
16427	101	1	9-Jun-17	21	1	17	2	1	1	0	3	2	1	1
16428	101	1	15-Jan-17	14	1	17	2	1	1	0	3	2	1	1
16429	101	1	8-Mar-17	17	1	17	2	1	1	0	3	2	1	1
16430	101	1	22-Mar-17	8	1	17	2	1	1	0	3	2	1	1
16431	201	1	20-Nov-17	1	1	17	2	1	1	0	3	2	1	1
16432	101	1	6-May-17	17	1	17	2	1	1	0	3	2	2	2
16433	201	1	26-Jul-17	9	1	17	2	2	1	0	3	2	3	1
16434	201	1	21-Apr-17	17	1	17	2	2	1	0	3	2	3	1
16435	201	1	8-Jun-17	16	1	17	2	2	1	0	3	2	1	1
16436	201	1	17-May-17	21	1	17	2	2	1	0	3	2	1	1
16437	201	1	24-May-17	16	1	17	2	2	1	0	3	2	1	1
16438	201	1	18-May-17	22	1	17	2	2	1	0	3	2	1	1
16439	201	1	18-Oct-17	9	1	17	2	3	1	0	3	2	3	1
16440	201	1	24-Nov-17	7	1	17	2	3	1	0	3	2	3	1
16441	101	1	3-Dec-17	9	1	17	2	1	1	0	3	2	3	1
16442	101	1	6-Dec-17	18	1	17	2	1	1	0	3	2	3	1
16443	101	1	20-Sep-17	21	1	17	2	1	1	0	3	2	2	1
16444	101	1	24-Oct-17	16	1	17	2	1	1	0	3	2	2	1
16445	101	1	6-Oct-17	19	1	17	2	1	1	0	3	2	1	1
16446	101	1	4-Nov-17	20	1	17	2	1	1	0	3	2	1	1
16447	101	1	22-Dec-17	21	1	17	2	1	1	0	3	2	1	1
16448	101	1	5-Nov-17	22	1	17	2	1	1	0	3	2	1	1
16449	101	1	8-Nov-17	19	1	17	2	1	1	0	3	2	1	1
16450	101	1	3-Nov-17	10	1	17	2	1	1	0	3	2	1	1
16451	101	1	26-Oct-17	22	1	17	2	1	1	0	3	2	5	1
16452	101	1	21-Oct-17	10	1	17	2	1	1	0	3	2	1	1
16453	101	1	9-Nov-16	9	1	17	2	1	1	0	6	2	2	1
16454	201	1	27-Jul-16	10	1	17	2	1	1	0	6	2	3	1
16455	101	1	8-Jul-16	11	1	17	2	1	1	0	6	2	3	1
16456	101	1	5-Jun-16	21	1	17	2	1	1	0	6	2	2	1
16457	101	1	9-May-16	20	1	17	2	1	1	0	6	2	5	1
16458	101	1	26-Sep-16	15	1	17	2	1	1	0	6	2	2	1
16459	101	1	14-Sep-15	15	1	17	2	1	1	0	6	2	2	1
16460	101	1	20-Jun-15	16	1	17	2	1	1	0	6	2	3	1
16461	101	1	4-May-15	18	1	17	2	1	1	0	6	2	2	1
16462	101	1	20-Sep-15	16	1	17	2	1	1	0	6	2	2	1
16463	101	1	2-Jul-15	21	1	17	2	1	1	0	6	2	2	1
16464	201	1	9-Mar-15	16	1	17	2	1	1	0	6	2	2	2
16465	101	1	4-Mar-14	21	1	17	2	1	1	0	6	2	3	1
16466	101	1	1-Sep-14	22	1	17	2	1	1	0	6	2	2	1
16467	201	1	15-Apr-13	9	1	17	2	2	1	0	6	2	1	1
16468	101	1	3-Nov-13	22	1	17	2	1	1	0	6	2	2	1
16469	101	1	25-Mar-13	8	1	17	2	1	1	0	6	2	2	1
16470	101	1	19-Jun-13	16	1	17	2	1	1	0	6	2	2	4
16471	101	1	25-Apr-13	21	1	17	2	1	1	0	6	2	3	1
16472	101	1	30-Jun-13	15	1	17	2	1	1	0	6	2	2	1
16473	201	1	15-Nov-12	10	1	17	2	2	1	0	6	2	2	1
16474	101	1	23-Apr-12	12	1	17	2	1	1	0	6	2	2	1
16475	201	1	5-Jun-12	7	1	17	2	2	1	0	6	2	2	2
16476	101	1	31-Aug-12	23	1	17	2	1	1	0	6	2	2	1
16477	101	1	28-Oct-12	17	1	17	2	1	1	0	6	2	2	1
16478	101	1	5-Jul-12	14	1	17	2	1	1	0	6	2	3	1
16479	101	1	24-Apr-11	16	1	17	2	1	1	0	6	2	2	1
16480	101	1	21-Nov-11	13	1	17	2	1	1	0	6	2	2	1
16481	101	1	25-Feb-11	8	1	17	2	1	1	0	6	2	2	1
16482	101	1	7-Oct-10	18	1	17	2	1	1	0	6	2	3	1
16483	101	1	6-May-10	14	1	17	2	1	1	0	6	2	3	1
16484	101	1	18-Nov-10	9	1	17	2	1	1	0	6	2	4	1
16485	101	1	25-Aug-10	11	1	17	2	1	1	0	6	2	2	1

16486	201	1	27-Nov-10	17	1	17	2	2	1	0	6	2	2	1
16487	201	1	31-Oct-09	20	1	17	2	1	1	0	6	2	2	1
16488	101	1	17-Dec-08	17	1	17	2	1	1	0	6	2	2	1
16489	201	1	6-Mar-08	20	1	17	2	2	1	0	6	2	2	1
16490	101	1	4-Jun-08	17	1	17	2	1	1	0	6	2	3	1
16491	101	1	27-Sep-08	16	1	17	2	1	1	0	6	2	2	1
16492	201	1	12-Sep-08	23	1	17	2	2	1	0	6	2	2	1
16493	201	1	28-Feb-08	19	1	17	2	2	1	0	6	2	3	1
16494	101	1	24-Sep-08	17	1	17	2	1	1	0	6	2	2	1
16495	101	1	9-Jan-08	20	1	17	2	1	1	0	6	2	2	1
16496	101	1	8-Apr-08	21	1	17	2	1	1	0	6	2	2	1
16497	101	1	28-Aug-08	23	1	17	2	1	1	0	6	2	2	1
16498	101	1	8-Jul-08	16	1	17	2	1	1	0	6	2	2	1
16499	101	1	19-Apr-07	14	1	17	2	1	1	0	6	2	2	1
16500	101	1	14-Jul-07	14	1	17	2	1	1	0	6	2	2	1
16501	101	1	27-Nov-07	13	1	17	2	1	1	0	6	2	2	4
16502	101	1	30-Apr-07	8	1	17	2	1	1	0	6	2	3	1
16503	101	1	22-Mar-07	12	1	17	2	1	1	0	6	2	1	1
16504	101	1	4-Mar-06	7	1	17	2	1	1	0	6	2	1	1
16505	101	1	27-Nov-06	17	1	17	2	1	1	0	6	2	2	1
16506	101	1	13-Jul-06	23	1	17	2	1	1	0	6	2	2	1
16507	101	1	25-Apr-06	23	1	17	2	1	1	0	6	2	3	1
16508	201	1	16-Aug-06	6	1	17	2	2	1	0	6	2	3	1
16509	101	1	6-May-17	19	1	17	2	1	1	0	6	2	5	1
16510	201	1	20-Feb-17	9	1	17	2	2	1	0	6	2	2	1
16511	101	1	23-Nov-17	7	1	17	2	1	1	0	6	2	2	1
16512	101	1	24-Jun-16	15	1	17	2	1	1	0	13	2	2	1
16513	201	1	20-Nov-08	16	1	17	2	1	1	0	13	2	3	2
16514	101	1	17-Jan-07	10	1	17	2	3	1	0	13	2	1	1
16515	101	1	9-May-06	4	1	17	2	1	1	0	13	2	7	1
16516	201	1	3-Feb-06	15	1	17	2	1	1	0	13	2	3	2
16517	101	1	19-Mar-06	17	1	17	2	1	1	0	13	2	2	1
16518	101	1	1-Oct-17	10	1	17	2	1	1	0	13	2	3	1
16519	101	1	30-Oct-17	21	1	17	2	1	1	0	13	2	2	1
16520	201	1	21-Sep-17	11	2	17	2	2	1	0	4	2	2	1
16521	201	1	29-Jul-12	21	2	17	2	3	1	0	3	2	1	1
16522	201	1	23-Oct-14	3	2	17	2	2	1	0	10	2	7	2
16523	201	1	23-Oct-12	17	2	17	2	3	1	0	3	2	1	1
16524	101	1	2-Jan-07	10	2	17	2	2	1	0	7	2	2	1
16525	101	1	29-Jul-16	20	2	17	2	1	1	0	13	2	4	1
16526	101	1	12-Mar-11	19	2	17	2	1	1	0	13	2	2	1
16527	101	1	20-Oct-07	9	2	17	2	1	1	0	13	2	3	1
16528	101	1	15-Feb-06	10	2	17	2	1	1	0	13	2	2	1
16529	101	1	19-Aug-06	17	2	17	2	1	1	0	1	2	1	1
16530	101	1	18-Oct-16	16	2	17	2	1	1	0	4	2	1	1
16531	101	1	21-Mar-14	21	2	17	2	3	1	0	4	2	2	1
16532	101	1	8-Jan-14	9	2	17	2	1	1	0	4	2	1	1
16533	101	1	11-Jul-13	17	2	17	2	1	1	0	4	2	3	1
16534	201	1	14-Oct-13	10	2	17	2	3	1	0	4	2	2	1
16535	101	1	23-Mar-12	17	2	17	2	1	1	0	4	2	4	1
16536	101	1	22-Mar-11	17	2	17	2	1	1	0	4	2	2	1
16537	101	1	22-Feb-11	11	2	17	2	1	1	0	4	2	1	1
16538	101	1	4-Mar-11	20	2	17	2	1	1	0	4	2	2	1
16539	101	1	31-May-10	12	2	17	2	1	1	0	4	2	1	1
16540	101	1	6-Apr-10	17	2	17	2	1	1	0	4	2	2	1
16541	101	1	16-May-09	13	2	17	2	1	1	0	4	2	2	1
16542	101	1	28-Jul-09	12	2	17	2	1	1	0	4	2	2	1
16543	101	1	7-Aug-09	1	2	17	2	1	1	0	4	2	3	1
16544	101	1	19-Mar-07	17	2	17	2	1	1	0	4	2	4	1
16545	101	1	7-Dec-07	17	2	17	2	1	1	0	4	2	1	1
16546	201	1	3-Apr-06	17	2	17	2	2	1	0	4	2	5	1
16547	101	1	19-May-06	17	2	17	2	1	1	0	4	2	1	1
16548	101	1	11-Jul-06	9	2	17	2	1	1	0	4	2	1	1
16549	201	1	17-Aug-06	9	2	17	2	2	1	0	4	2	3	1
16550	101	1	27-Sep-06	15	2	17	2	1	1	0	4	2	2	1
16551	101	1	7-Apr-17	21	2	17	2	1	1	0	4	2	3	1
16552	101	1	11-Mar-17	17	2	17	2	1	1	0	4	2	2	1
16553	201	1	30-Dec-16	9	2	17	2	2	1	0	11	2	2	1
16554	101	1	10-Nov-16	18	2	17	2	1	1	0	11	2	1	1
16555	201	1	10-Aug-16	9	2	17	2	2	1	0	11	2	2	1
16556	201	1	22-Jul-16	14	2	17	2	2	1	0	11	2	2	1
16557	201	1	19-Aug-16	16	2	17	2	3	1	0	11	2	3	1
16558	201	1	23-Oct-14	9	2	17	2	1	1	0	11	2	2	1
16559	101	1	22-Mar-14	21	2	17	2	1	1	0	11	2	2	1
16560	101	1	3-Jun-14	18	2	17	2	1	1	0	11	2	3	1
16561	201	1	17-Sep-13	14	2	17	2	3	1	0	11	2	2	1
16562	101	1	22-Oct-13	21	2	17	2	1	1	0	11	2	2	1
16563	101	1	8-Sep-13	7	2	17	2	1	1	0	11	2	1	1
16564	101	1	2-Nov-13	19	2	17	2	1	1	0	11	2	2	1
16565	101	1	29-Aug-13	17	2	17	2	2	1	0	11	2	3	1
16566	101	1	5-Jul-13	17	2	17	2	1	1	0	11	2	3	1
16567	101	1	5-Jul-13	14	2	17	2	1	1	0	11	2	2	1
16568	201	1	8-Oct-13	11	2	17	2	2	1	0	11	2	2	1
16569	101	1	6-Aug-13	13	2	17	2	3	1	0	11	2	3	1
16570	101	1	28-Apr-12	17	2	17	2	1	1	0	11	2	3	1
16571	101	1	18-Feb-12	11	2	17	2	1	1	0	11	2	2	1
16572	201	1	3-Feb-12	3	2	17	2	1	1	0	11	2	2	1
16573	101	1	10-Mar-12	10	2	17	2	1	1	0	11	2	3	1
16574	101	1	13-Sep-11	19	2	17	2	1	1	0	11	2	3	1
16575	101	1	6-Jul-11	17	2	17	2	1	1	0	11	2	3	1
16576	101	1	4-Jan-10	17	2	17	2	1	1	0	11	2	2	1
16577	201	1	8-Dec-10	16	2	17	2	2	1	0	11	2	2	1
16578	201	1	13-Aug-10	16	2	17	2	1	1	0	11	2	2	1
16579	201	1	28-Aug-10	10	2	17	2	2	1	0	11	2	3	1
16580	101	1	28-Jul-09	12	2	17	2	1	1	0	11	2	2	1
16581	101	1	17-Oct-08	12	2	17	2	1	1	0	11	2	2	1
16582	101	1	11-Mar-17	11	2	17	2	1	1	0	11	2	2	1
16583	101	1	30-Jan-17	14	2	17	2	1	1	0	11	2	2	1
16584	201	1	30-Jun-17	16	2	17	2	2	1	0	11	2	2	1
16585	201	1	9-Dec-17	10	2	17	2	3	1	0	11	2	2	1
16586	101	1	3-Oct-17	17	2	17	2	1	1	0	11	2	3	1
16587	201	1	29-Oct-17	10	2	17	2	1	1	0	11	2	2	1
16588	101	1	4-Nov-16	13	2	17	2	1	1	0	3	2	1	1
16589	201	1	30-Aug-16	21	2	17	2	3	1	0	3	2	4	1
16590	101	1	15-Jun-16	12	2	17	2	1	1	0	3	2	3	1
16591	101	1	3-Jun-16	15	2	17	2	1	1	0	3	2	2	1
16592	101	1	4-Apr-16	20	2	17	2	1	1	0	3	2	5	1
16593	201	1	8-Jun-16	15	2	17	2	1	1	0	3	2	2	1
16594	101	1	21-Jun-16	16	2	17	2	1	1	0	3	2	3	1
16595	101	1	13-Mar-16	17	2	17	2	1	1	0	3	2	5	1
16596	101	1	24-Sep-15	4	2	17	2	1	1	0	3	2	5	2
16597	101	1	23-Jan-15	17	2	17	2	1	1	0	3	2	1	1
16598	101	1	9-Nov-15	17	2	17	2	1	1	0	3	2	3	1
16599	201	1	18-Mar-15	23	2	17	2	3	1	0	3	2	1	1
16600	101	1	26-Dec-15	20	2	17	2	1	1	0	3	2	1	1
16601	101	1	30-Nov-15	17	2	17	2	1	1	0	3	2	1	1
16602	101	1	13-Oct-15	18	2	17	2	1	1	0	3	2	3	1
16603	101	1	25-Dec-15	17	2	17	2	1	1	0	3	2	3	1
16604	101	1	21-May-15	13	2	17	2	1	1	0	3	2	3	1
16605	201	1	22-Dec-15	17	2	17	2	2	1	0	3	2	3	1
16606	201	1	9-Jan-14	16	2	17	2	3	1	0	3	2	1	1
16607	101	1	7-Mar-14	16	2	17	2	1	1	0	3	2	1	1
16608	101	1	11-Oct-14	16	2	17	2	1	1	0	3	2	4	4
16609	201	1	16-Sep-14	18	2	17	2	2	1	0	3	2	3	1

16610	101	1	18-Oct-14	17	2	17	2	1	1	0	3	2	3	1
16611	201	1	2-Jun-14	15	2	17	2	3	1	0	3	2	3	1
16612	201	1	1-Mar-14	10	2	17	2	1	1	0	3	2	5	1
16613	201	1	16-May-14	14	2	17	2	2	1	0	3	2	5	1
16614	101	1	27-Apr-14	21	2	17	2	3	1	0	3	2	1	1
16615	101	1	24-Oct-14	16	2	17	2	1	1	0	3	2	3	1
16616	101	1	18-Dec-14	20	2	17	2	1	1	0	3	2	3	1
16617	101	1	5-Nov-14	14	2	17	2	1	1	0	3	2	3	1
16618	101	1	13-Dec-14	19	2	17	2	1	1	0	3	2	2	1
16619	101	1	25-May-14	9	2	17	2	1	1	0	3	2	3	1
16620	201	1	10-Feb-14	9	2	17	2	1	1	0	3	2	3	1
16621	201	1	4-Sep-14	10	2	17	2	3	1	0	3	2	3	1
16622	201	1	13-Nov-14	15	2	17	2	1	1	0	3	2	3	1
16623	201	1	15-Dec-14	9	2	17	2	1	1	0	3	2	3	1
16624	101	1	14-Dec-14	16	2	17	2	1	1	0	3	2	3	1
16625	201	1	14-May-13	15	2	17	2	2	1	0	3	2	1	1
16626	101	1	28-Sep-13	16	2	17	2	1	1	0	3	2	3	1
16627	201	1	4-Apr-13	10	2	17	2	3	1	0	3	2	4	1
16628	201	1	1-Feb-13	19	2	17	2	3	1	0	3	2	3	2
16629	101	1	2-Sep-13	8	2	17	2	1	1	0	3	2	3	1
16630	101	1	14-Mar-13	20	2	17	2	1	1	0	3	2	2	1
16631	101	1	2-Dec-13	20	2	17	2	1	1	0	3	2	3	1
16632	101	1	31-Jul-13	21	2	17	2	1	1	0	3	2	3	1
16633	101	1	29-Mar-13	10	2	17	2	1	1	0	3	2	3	1
16634	201	1	11-Nov-13	9	2	17	2	2	1	0	3	2	1	1
16635	101	1	27-Sep-13	16	2	17	2	1	1	0	3	2	5	1
16636	101	1	22-Nov-13	12	2	17	2	1	1	0	3	2	5	1
16637	201	1	11-Apr-12	11	2	17	2	2	1	0	3	2	1	1
16638	101	1	24-Feb-12	16	2	17	2	1	1	0	3	2	4	1
16639	101	1	6-Dec-12	12	2	17	2	1	1	0	3	2	3	1
16640	101	1	24-Apr-12	15	2	17	2	1	1	0	3	2	2	2
16641	201	1	14-Dec-12	14	2	17	2	1	1	0	3	2	3	1
16642	101	1	7-Dec-12	15	2	17	2	1	1	0	3	2	2	1
16643	101	1	22-Mar-12	16	2	17	2	1	1	0	3	2	2	1
16644	101	1	21-Jan-11	9	2	17	2	1	1	0	3	2	1	1
16645	101	1	21-Oct-11	16	2	17	2	1	1	0	3	2	3	1
16646	201	1	26-Jun-11	15	2	17	2	1	1	0	3	2	2	1
16647	101	1	1-Mar-11	17	2	17	2	1	1	0	3	2	3	1
16648	101	1	31-Oct-11	17	2	17	2	1	1	0	3	2	2	1
16649	101	1	19-Feb-11	6	2	17	2	1	1	0	3	2	4	1
16650	101	1	16-Dec-11	10	2	17	2	1	1	0	3	2	3	1
16651	101	1	6-Sep-11	1	2	17	2	2	1	0	3	2	3	1
16652	201	1	25-Jan-11	22	2	17	2	1	1	0	3	2	2	1
16653	101	1	29-May-11	10	2	17	2	1	1	0	3	2	4	1
16654	201	1	31-Dec-11	13	2	17	2	2	1	0	3	2	1	1
16655	201	1	30-May-11	18	2	17	2	1	1	0	3	2	6	1
16656	101	1	29-Mar-11	17	2	17	2	1	1	0	3	2	3	1
16657	201	1	19-Mar-11	17	2	17	2	2	1	0	3	2	3	1
16658	201	1	18-Dec-11	20	2	17	2	2	1	0	3	2	2	1
16659	201	1	27-Sep-11	11	2	17	2	3	1	0	3	2	2	2
16660	101	1	12-Oct-10	18	2	17	2	1	1	0	3	2	1	1
16661	101	1	27-Aug-10	9	2	17	2	1	1	0	3	2	1	1
16662	101	1	1-Aug-10	15	2	17	2	1	1	0	3	2	1	1
16663	101	1	14-Jan-10	9	2	17	2	1	1	0	3	2	2	1
16664	101	1	18-May-10	16	2	17	2	1	1	0	3	2	3	1
16665	101	1	21-Nov-10	8	2	17	2	3	1	0	3	2	3	1
16666	201	1	15-Feb-10	9	2	17	2	2	1	0	3	2	3	1
16667	101	1	3-Nov-10	19	2	17	2	1	1	0	3	2	3	1
16668	101	1	29-Aug-10	8	2	17	2	1	1	0	3	2	4	1
16669	101	1	13-Mar-10	17	2	17	2	1	1	0	3	2	2	1
16670	101	1	1-Nov-10	17	2	17	2	1	1	0	3	2	3	1
16671	101	1	14-Nov-10	17	2	17	2	1	1	0	3	2	3	1
16672	101	1	14-Dec-10	15	2	17	2	1	1	0	3	2	3	1
16673	101	1	9-Nov-10	17	2	17	2	1	1	0	3	2	3	1
16674	101	1	6-Oct-10	13	2	17	2	1	1	0	3	2	1	1
16675	201	1	20-Aug-10	20	2	17	2	1	1	0	3	2	3	1
16676	201	1	31-Jan-09	22	2	17	2	2	1	0	3	2	3	1
16677	101	1	15-Jun-09	16	2	17	2	1	1	0	3	2	3	1
16678	101	1	1-Jul-09	12	2	17	2	1	1	0	3	2	3	1
16679	201	1	19-Jun-09	15	2	17	2	2	1	0	3	2	4	1
16680	101	1	23-Aug-09	21	2	17	2	1	1	0	3	2	1	1
16681	101	1	9-Nov-09	16	2	17	2	2	1	0	3	2	1	1
16682	201	1	2-Sep-09	22	2	17	2	2	1	0	3	2	3	1
16683	201	1	19-Mar-08	13	2	17	2	1	1	0	3	2	3	1
16684	101	1	17-Apr-08	18	2	17	2	1	1	0	3	2	3	1
16685	101	1	12-May-08	21	2	17	2	1	1	0	3	2	1	1
16686	201	1	13-May-08	17	2	17	2	2	1	0	3	2	3	1
16687	101	1	22-Jun-08	12	2	17	2	1	1	0	3	2	3	1
16688	101	1	17-Dec-08	8	2	17	2	1	1	0	3	2	3	1
16689	101	1	7-Apr-08	17	2	17	2	1	1	0	3	2	2	1
16690	201	1	31-May-08	16	2	17	2	1	1	0	3	2	1	1
16691	101	1	23-Oct-08	9	2	17	2	1	1	0	3	2	4	1
16692	101	1	23-Dec-08	12	2	17	2	1	1	0	3	2	3	1
16693	201	1	20-Sep-07	18	2	17	2	1	1	0	3	2	3	1
16694	201	1	27-May-07	21	2	17	2	1	1	0	3	2	3	1
16695	101	1	24-Apr-07	19	2	17	2	1	1	0	3	2	2	1
16696	201	1	14-May-07	22	2	17	2	2	1	0	3	2	1	1
16697	101	1	24-Apr-07	20	2	17	2	1	1	0	3	2	2	1
16698	201	1	5-Aug-07	15	2	17	2	3	1	0	3	2	2	1
16699	101	1	27-Jun-07	18	2	17	2	1	1	0	3	2	2	1
16700	101	1	13-May-07	17	2	17	2	1	1	0	3	2	3	1
16701	101	1	20-Mar-07	16	2	17	2	1	1	0	3	2	3	1
16702	101	1	17-Oct-06	0	2	17	2	1	1	0	3	2	3	1
16703	101	1	9-Apr-06	15	2	17	2	1	1	0	3	2	2	1
16704	101	1	22-Mar-06	18	2	17	2	1	1	0	3	2	1	1
16705	201	1	31-Jul-06	16	2	17	2	1	1	0	3	2	1	1
16706	101	1	24-May-06	15	2	17	2	1	1	0	3	2	3	1
16707	201	1	3-Dec-06	15	2	17	2	3	1	0	3	2	3	1
16708	101	1	12-May-06	17	2	17	2	1	1	0	3	2	3	1
16709	201	1	31-Oct-06	16	2	17	2	1	1	0	3	2	2	1
16710	201	1	17-Sep-06	19	2	17	2	1	1	0	3	2	2	1
16711	201	1	5-Apr-06	23	2	17	2	2	1	0	3	2	3	1
16712	101	1	30-Mar-17	17	2	17	2	1	1	0	3	2	3	1
16713	101	1	23-Aug-17	19	2	17	2	1	1	0	3	2	2	1
16714	101	1	12-Aug-17	10	2	17	2	1	1	0	3	2	2	1
16715	101	1	23-Aug-17	18	2	17	2	1	1	0	3	2	2	1
16716	201	1	22-May-17	9	2	17	2	1	1	0	3	2	1	1
16717	101	1	10-Oct-17	18	2	17	2	1	1	0	3	2	3	1
16718	101	1	10-Nov-17	12	2	17	2	1	1	0	3	2	1	1
16719	101	1	6-Jul-16	6	2	17	2	1	1	0	6	2	3	1
16720	101	1	17-Oct-16	21	2	17	2	1	1	0	6	2	3	1
16721	201	1	20-Apr-16	8	2	17	2	2	1	0	6	2	2	1
16722	201	1	3-Aug-16	19	2	17	2	3	1	0	6	2	2	1
16723	101	1	5-Jun-16	11	2	17	2	1	1	0	6	2	2	1
16724	101	1	16-Oct-15	10	2	17	2	1	1	0	6	2	2	1
16725	101	1	1-May-15	12	2	17	2	1	1	0	6	2	3	1
16726	101	1	17-Nov-15	3	2	17	2	1	1	0	6	2	2	1
16727	101	1	10-Apr-14	23	2	17	2	1	1	0	6	2	2	1
16728	101	1	2-Jul-12	17	2	17	2	1	1	0	6	2	2	1
16729	101	1	14-May-11	12	2	17	2	1	1	0	6	2	2	1
16730	101	1	18-Apr-11	22	2	17	2	1	1	0	6	2	3	1
16731	201	1	25-May-09	15	2	17	2	1	1	0	6	2	2	1
16732	201	1	30-Sep-08	17	2	17	2	3	1	0	6	2	1	1
16733	101	1	4-Apr-08	12	2	17	2	1	1	0	6	2	2	1

16734	101	1	24-Sep-08	17	2	17	2	1	1	0	6	2	2	1
16735	101	1	18-Oct-06	13	2	17	2	1	1	0	6	2	2	1
16736	101	1	26-May-06	18	2	17	2	1	1	0	6	2	3	1
16737	101	1	18-Dec-06	10	2	17	2	1	1	0	6	2	2	1
16738	202	0	3-Mar-12	18	1	10	2	1	3	1	11	1	3	1
16739	202	0	3-Mar-12	18	1	10	2	1	3	1	11	1	3	1
16740	202	0	28-Aug-17	0	2	11	2	2	3	1	11	1	2	1
16741	202	0	5-Dec-12	19	2	12	2	1	3	1	4	1	4	1
16742	202	0	24-Jun-12	19	2	12	2	1	3	1	11	1	1	1
16743	202	0	1-Sep-11	22	2	12	2	1	3	1	11	1	2	2
16744	101	1	25-Dec-09	23	1	12	2	1	3	1	4	1	1	1
16745	101	1	7-Feb-13	20	1	12	2	1	3	1	11	1	2	1
16746	202	0	12-Oct-16	16	1	13	2	1	3	1	11	1	2	1
16747	202	0	12-Nov-13	19	1	13	2	1	3	1	11	1	2	1
16748	202	0	25-Aug-11	8	1	13	2	1	3	1	11	1	3	1
16749	202	0	10-Sep-10	21	1	13	2	1	3	1	3	1	6	1
16750	202	0	21-Jan-14	23	1	13	2	1	3	1	6	1	1	1
16751	201	1	3-Aug-16	7	1	13	2	3	3	1	4	1	2	2
16752	101	1	20-Mar-12	23	1	13	2	1	3	1	4	1	4	1
16753	101	1	15-Nov-12	20	1	13	2	1	3	1	4	1	4	1
16754	101	1	28-Dec-12	23	1	13	2	1	3	1	4	1	4	1
16755	101	1	25-Nov-09	19	1	13	2	1	3	1	4	1	1	1
16756	101	1	6-Jul-14	21	1	13	2	1	3	1	3	1	4	1
16757	101	1	10-Aug-12	10	1	13	2	2	3	1	6	1	2	1
16758	101	1	2-Jun-10	23	2	13	2	1	3	1	4	1	1	1
16759	101	1	15-Sep-08	15	2	13	2	1	3	1	4	1	2	1
16760	101	1	16-Jan-06	19	2	13	2	1	3	1	4	1	2	1
16761	101	1	7-Nov-11	17	2	13	2	1	3	1	11	1	3	1
16762	101	1	4-Nov-11	21	2	13	2	1	3	1	11	1	2	1
16763	101	1	14-Aug-10	22	2	13	2	1	3	1	11	1	3	1
16764	101	1	14-Aug-10	20	2	13	2	1	3	1	11	1	2	1
16765	101	1	16-Oct-09	9	2	13	2	1	3	1	11	1	3	1
16766	101	1	25-Aug-09	17	2	13	2	1	3	1	11	1	3	1
16767	202	0	26-Sep-16	22	1	14	2	1	3	1	4	1	1	1
16768	202	0	19-Jul-13	15	1	14	2	1	3	1	11	1	1	1
16769	202	0	25-Sep-12	14	1	14	2	1	3	1	11	1	3	1
16770	202	0	29-Oct-12	22	1	14	2	1	3	1	11	1	3	1
16771	202	0	11-Apr-17	22	1	14	2	1	3	1	11	1	2	1
16772	202	0	1-May-11	12	1	14	2	1	3	1	6	1	2	2
16773	202	0	16-Aug-12	21	2	14	2	1	3	1	11	1	4	1
16774	202	0	6-Jan-12	14	2	14	2	1	3	1	11	1	2	1
16775	101	1	15-Dec-13	3	1	14	2	1	3	1	4	1	1	1
16776	101	1	1-May-13	11	1	14	2	1	3	1	4	1	2	4
16777	101	1	31-Dec-09	20	1	14	2	3	3	1	4	1	1	1
16778	101	1	1-May-09	1	1	14	2	1	3	1	4	1	1	1
16779	101	1	19-Sep-11	15	1	14	2	1	3	1	11	1	2	1
16780	101	1	21-Nov-10	19	1	14	2	1	3	1	11	1	2	1
16781	101	1	30-Jul-09	17	1	14	2	1	3	1	11	1	2	1
16782	101	1	5-Jul-09	16	1	14	2	1	3	1	11	1	3	1
16783	101	1	3-Jun-11	20	1	14	2	1	3	1	6	1	1	1
16784	101	1	14-Jul-12	4	2	14	2	1	3	1	4	1	1	1
16785	101	1	11-Feb-10	18	2	14	2	1	3	1	11	1	3	1
16786	101	1	21-Mar-10	5	2	14	2	1	3	1	11	1	2	1
16787	101	1	10-Jun-10	20	2	14	2	1	3	1	11	1	2	1
16788	101	1	12-Jul-10	19	2	14	2	1	3	1	11	1	2	1
16789	101	1	19-Apr-10	22	2	14	2	1	3	1	11	1	3	1
16790	101	1	23-Jun-09	16	2	14	2	1	3	1	11	1	2	1
16791	202	0	24-Jul-12	18	1	15	2	3	3	1	4	1	2	1
16792	202	0	25-May-08	22	1	15	2	1	3	1	4	1	1	1
16793	202	0	4-Nov-07	20	1	15	2	1	3	1	4	1	1	2
16794	202	0	11-Mar-17	15	1	15	2	2	3	1	11	1	2	1
16795	202	0	20-Jul-09	23	2	15	2	1	3	1	4	1	6	1
16796	202	0	6-Dec-11	22	2	15	2	1	3	1	11	1	3	1
16797	202	0	4-Oct-06	12	2	15	2	1	3	1	6	1	1	2
16798	201	1	25-Jul-16	20	1	15	2	2	3	1	4	1	5	1
16799	101	1	18-Jun-09	12	1	15	2	1	3	1	4	1	1	1
16800	101	1	12-Oct-11	6	1	15	2	1	3	1	11	1	2	1
16801	101	1	17-Sep-11	7	1	15	2	1	3	1	11	1	2	1
16802	101	1	2-Aug-10	18	1	15	2	1	3	1	11	1	2	1
16803	101	1	20-Jul-09	9	1	15	2	1	3	1	11	1	2	1
16804	101	1	15-Apr-09	3	1	15	2	1	3	1	11	1	3	1
16805	201	1	3-Mar-08	12	1	15	2	1	3	1	6	1	2	1
16806	201	1	25-Jan-12	23	2	15	2	2	3	1	4	1	1	1
16807	101	1	4-Jan-08	22	2	15	2	1	3	1	4	1	2	1
16808	101	1	25-May-08	20	2	15	2	1	3	1	4	1	2	1
16809	101	1	2-Jan-11	10	2	15	2	2	3	1	11	1	2	1
16810	101	1	6-Feb-09	13	2	15	2	1	3	1	11	1	2	1
16811	101	1	30-Jul-09	18	2	15	2	1	3	1	11	1	3	1
16812	101	1	3-Aug-09	6	2	15	2	1	3	1	11	1	3	1
16813	101	1	23-Jun-09	19	2	15	2	1	3	1	11	1	2	1
16814	101	1	10-Jul-10	18	2	15	2	3	3	1	6	1	2	1
16815	202	0	21-Oct-11	2	1	16	2	1	3	1	10	1	7	2
16816	202	0	23-Sep-16	19	1	16	2	2	3	1	4	1	1	2
16817	202	0	26-Mar-16	20	1	16	2	1	3	1	11	1	2	1
16818	202	0	14-Aug-16	0	1	16	2	3	3	1	6	1	3	1
16819	202	0	17-Oct-09	21	1	16	2	1	3	1	6	1	2	1
16820	202	0	19-Sep-09	0	1	16	2	1	3	1	6	1	3	1
16821	202	0	20-Jun-16	4	2	16	2	2	3	1	6	1	4	1
16822	202	0	3-Feb-06	11	2	16	2	1	3	1	6	1	2	1
16823	101	1	11-Aug-09	1	1	16	2	1	3	1	4	1	4	2
16824	101	1	30-May-10	17	1	16	2	1	3	1	11	1	2	1
16825	101	1	3-Dec-10	8	1	16	2	1	3	1	11	1	2	1
16826	101	1	24-Aug-10	22	1	16	2	1	3	1	11	1	3	1
16827	101	1	10-Feb-10	7	1	16	2	1	3	1	11	1	3	1
16828	101	1	3-Jul-09	14	1	16	2	1	3	1	11	1	4	1
16829	101	1	31-May-09	22	1	16	2	1	3	1	11	1	2	1
16830	101	1	8-Jul-09	18	1	16	2	1	3	1	11	1	2	1
16831	101	1	7-Sep-09	1	1	16	2	1	3	1	11	1	3	1
16832	101	1	11-Jul-09	20	1	16	2	1	3	1	11	1	3	1
16833	201	1	5-Mar-07	11	1	16	2	1	3	1	11	1	2	1
16834	101	1	4-Jul-10	20	2	16	2	1	3	1	11	1	2	1
16835	202	0	24-Feb-13	13	1	17	2	2	3	1	11	1	2	1
16836	202	0	10-Mar-06	0	1	17	2	2	3	1	6	1	2	1
16837	202	0	13-Sep-13	12	2	17	2	1	3	1	10	1	6	2
16838	202	0	15-Jun-13	16	2	17	2	3	3	1	10	1	1	2
16839	202	0	21-Oct-14	14	2	17	2	1	3	1	4	1	1	1
16840	202	0	5-Sep-12	18	2	17	2	1	3	1	11	1	2	1
16841	202	0	25-Feb-12	21	2	17	2	1	3	1	11	1	3	1
16842	101	1	6-Aug-10	22	1	17	2	1	3	1	4	1	4	1
16843	101	1	24-Jan-11	23	1	17	2	1	3	1	11	1	1	1
16844	101	1	16-May-11	12	1	17	2	1	3	1	11	1	2	1
16845	101	1	11-Apr-10	19	1	17	2	1	3	1	11	1	4	1
16846	101	1	14-Aug-10	19	1	17	2	1	3	1	11	1	3	1
16847	101	1	10-Nov-10	17	1	17	2	1	3	1	11	1	2	1
16848	101	1	8-Oct-10	23	2	17	2	3	3	1	4	1	4	2
16849	101	1	28-Jan-17	21	2	17	2	3	3	1	4	1	1	1
16850	101	1	24-Jan-11	19	2	17	2	1	3	1	11	1	2	1
16851	101	1	25-Apr-10	19	2	17	2	1	3	1	11	1	2	1
16852	101	1	2-Mar-13	22	2	17	2	1	3	1	6	1	3	1
16853	101	1	24-Aug-07	2	2	17	2	3	3	1	6	1	2	4
16854	101	1	7-Dec-07	13	2	17	2	1	3	1	6	1	2	1
16855	202	0	24-Oct-09	22	1	12	2	1	2	1	10	1	7	2
16856	101	1	15-Aug-12	17	1	12	2	1	2	1	4	1	3	1
16857	202	0	29-Aug-17	15	1	13	2	1	2	1	6	1	2	1

16858	202	0	31-Jul-08	20	2	13	2	3	2	1	10	1	7	2
16859	101	1	30-Oct-17	7	2	13	2	1	2	1	6	1	2	1
16860	202	0	25-Nov-09	2	1	14	2	1	2	1	10	1	7	2
16861	202	0	11-Aug-09	23	1	14	2	1	2	1	3	1	4	2
16862	202	0	5-Sep-11	1	2	14	2	1	2	1	10	1	7	2
16863	101	1	29-Mar-12	22	1	14	2	1	2	1	6	1	2	1
16864	101	1	8-Mar-10	19	1	14	2	1	2	1	4	1	2	4
16865	101	1	29-Dec-13	11	1	14	2	1	2	1	3	1	2	1
16866	101	1	27-Jun-14	22	2	14	2	1	2	1	6	1	2	1
16867	201	1	3-May-13	7	2	14	2	1	2	1	6	1	2	1
16868	202	0	9-Apr-11	17	1	15	2	1	2	1	10	1	7	2
16869	202	0	23-Apr-10	20	1	15	2	1	2	1	10	1	7	1
16870	202	0	28-Feb-14	16	2	15	2	1	2	1	10	1	7	2
16871	202	0	19-Sep-06	21	2	15	2	1	2	1	10	1	7	1
16872	202	0	7-Mar-06	15	2	15	2	1	2	1	10	1	7	2
16873	202	0	30-May-08	13	2	15	2	1	2	1	3	1	1	2
16874	201	1	31-Aug-14	19	1	15	2	1	2	1	10	1	7	2
16875	201	1	31-Dec-07	3	1	15	2	3	2	1	10	1	7	2
16876	201	1	30-Dec-07	20	1	15	2	2	2	1	10	1	7	2
16877	201	1	8-May-13	20	1	15	2	3	2	1	6	1	2	1
16878	101	1	8-Jan-13	20	1	15	2	1	2	1	6	1	2	1
16879	101	1	9-Sep-12	16	1	15	2	1	2	1	6	1	2	1
16880	201	1	2-Jul-16	23	2	15	2	1	2	1	6	1	2	1
16881	201	1	13-Oct-15	17	2	15	2	3	2	1	10	1	6	1
16882	201	1	20-Jun-16	1	2	15	2	2	2	1	10	1	6	1
16883	201	1	16-Oct-07	23	2	15	2	1	2	1	10	1	7	2
16884	201	1	21-Oct-16	23	2	15	2	2	2	1	6	1	2	1
16885	101	1	18-Aug-11	14	2	15	2	1	2	1	6	1	2	1
16886	202	0	28-Jul-15	20	1	16	2	1	2	1	10	1	6	3
16887	202	0	3-Feb-06	22	1	16	2	1	2	1	6	1	2	1
16888	202	0	22-Jan-12	15	2	16	2	1	2	1	6	1	2	2
16889	201	1	10-May-15	20	1	16	2	2	2	1	10	1	4	1
16890	101	1	31-Jul-15	12	1	16	2	1	2	1	6	1	2	2
16891	101	1	3-Apr-15	2	1	16	2	2	2	1	6	1	2	1
16892	101	1	28-May-10	16	2	16	2	1	2	1	3	1	5	2
16893	202	0	11-Oct-17	4	2	17	2	2	2	1	10	1	7	2
16894	202	0	4-Feb-12	9	2	17	2	1	2	1	10	1	7	2
16895	202	0	21-Apr-10	2	2	17	2	1	2	1	10	1	7	2
16896	202	0	14-Jul-13	21	2	17	2	1	2	1	6	1	2	1
16897	202	0	11-Nov-08	6	2	17	2	1	2	1	6	1	2	2
16898	201	1	21-Mar-13	19	1	17	2	2	2	1	6	1	4	2
16899	101	1	29-Dec-13	1	1	17	2	3	2	1	6	1	2	1
16900	201	1	27-Mar-10	20	1	17	2	1	2	1	6	1	1	1
16901	101	1	16-Jul-07	21	1	17	2	1	2	1	6	1	2	1
16902	101	1	21-Apr-17	12	1	17	2	1	2	1	6	1	2	1
16903	201	1	21-Mar-15	20	2	17	2	3	2	1	10	1	5	1
16904	101	1	28-Nov-08	23	2	17	2	1	2	1	6	1	2	1
16905	202	0	9-Jan-12	15	1	9	2	3	1	0	11	1	4	1
16906	202	0	3-Mar-06	19	2	9	2	3	1	0	10	1	1	2
16907	202	0	3-Dec-07	12	1	10	2	3	1	0	13	1	1	1
16908	202	0	1-May-08	19	1	10	2	1	1	0	1	1	2	1
16909	202	0	17-Mar-14	21	2	10	2	1	1	0	11	1	2	1
16910	202	0	7-Aug-10	13	2	10	2	1	1	0	3	1	3	1
16911	101	1	30-Mar-08	17	1	10	2	2	1	0	11	1	3	1
16912	101	1	13-Nov-17	1	1	10	2	1	1	0	11	1	2	1
16913	202	0	2-Mar-12	19	1	11	2	1	1	0	4	1	3	1
16914	202	0	15-Sep-10	4	1	11	2	3	1	0	3	1	1	2
16915	202	0	24-Jul-09	23	1	11	2	1	1	0	3	1	2	1
16916	202	0	7-Apr-16	11	2	11	2	1	1	0	11	1	2	1
16917	201	1	14-Oct-14	19	1	11	2	2	1	0	4	1	1	1
16918	201	1	11-Mar-16	20	1	11	2	2	1	0	6	1	2	1
16919	201	1	6-Aug-12	2	1	11	2	2	1	0	4	1	1	1
16920	101	1	3-May-10	19	1	11	2	3	1	0	7	1	4	1
16921	201	1	22-Aug-07	16	1	11	2	2	1	0	7	1	4	2
16922	101	1	24-Nov-11	17	1	11	2	1	1	0	4	1	3	1
16923	201	1	20-Nov-16	19	1	11	2	1	1	0	11	1	2	1
16924	201	1	18-Feb-15	18	1	11	2	2	1	0	11	1	2	1
16925	201	1	27-Aug-15	16	1	11	2	2	1	0	11	1	2	1
16926	101	1	10-Jan-15	19	1	11	2	1	1	0	11	1	2	1
16927	201	1	17-Jan-14	8	1	11	2	3	1	0	11	1	2	1
16928	201	1	23-Jan-11	7	1	11	2	3	1	0	11	1	3	1
16929	201	1	12-Sep-11	9	1	11	2	2	1	0	11	1	2	1
16930	201	1	2-Dec-10	15	1	11	2	2	1	0	11	1	2	1
16931	201	1	7-Nov-10	14	1	11	2	1	1	0	11	1	2	1
16932	201	1	30-Nov-08	19	1	11	2	1	1	0	11	1	3	1
16933	101	1	14-Sep-07	20	1	11	2	1	1	0	11	1	2	1
16934	101	1	19-Aug-17	13	1	11	2	1	1	0	11	1	2	1
16935	101	1	24-Nov-16	3	1	11	2	1	1	0	3	1	2	1
16936	201	1	28-Apr-16	17	1	11	2	3	1	0	3	1	1	1
16937	201	1	18-Feb-15	13	1	11	2	2	1	0	3	1	1	1
16938	201	1	5-Sep-14	17	1	11	2	3	1	0	3	1	2	1
16939	201	1	9-Jun-14	23	1	11	2	3	1	0	3	1	3	1
16940	201	1	26-Dec-13	20	1	11	2	1	1	0	3	1	1	1
16941	101	1	29-Mar-11	19	1	11	2	1	1	0	3	1	2	1
16942	201	1	11-Jul-10	20	1	11	2	2	1	0	3	1	2	1
16943	201	1	21-Aug-06	20	1	11	2	3	1	0	3	1	1	1
16944	101	1	20-Jan-15	20	1	11	2	1	1	0	6	1	2	1
16945	101	1	16-Feb-13	9	2	11	2	1	1	0	11	1	3	1
16946	201	1	2-Feb-12	13	2	11	2	2	1	0	11	1	2	1
16947	201	1	2-Feb-12	12	2	11	2	3	1	0	11	1	3	1
16948	201	1	1-Aug-08	22	2	11	2	2	1	0	11	1	2	1
16949	101	1	10-Apr-08	17	2	11	2	1	1	0	11	1	2	1
16950	201	1	23-Nov-08	14	2	11	2	3	1	0	11	1	2	1
16951	101	1	18-Oct-06	14	2	11	2	1	1	0	11	1	2	1
16952	101	1	28-Nov-17	18	2	11	2	1	1	0	11	1	3	1
16953	101	1	17-Sep-17	13	2	11	2	1	1	0	11	1	2	1
16954	201	1	27-Dec-17	15	2	11	2	1	1	0	11	1	2	1
16955	101	1	16-Apr-11	17	2	11	2	1	1	0	3	1	2	1
16956	201	1	13-Feb-11	12	2	11	2	2	1	0	6	1	2	1
16957	101	1	18-Feb-06	22	2	11	2	1	1	0	6	1	2	2
16958	202	0	25-Sep-16	20	1	12	2	1	1	0	6	1	3	1
16959	202	0	19-Mar-14	20	1	12	2	3	1	0	7	1	4	1
16960	202	0	21-Mar-10	12	1	12	2	1	1	0	7	1	1	1
16961	202	0	13-Oct-16	22	1	12	2	2	1	0	4	1	3	1
16962	202	0	19-Mar-16	14	1	12	2	1	1	0	11	1	3	1
16963	202	0	12-Jan-13	8	1	12	2	2	1	0	11	1	3	1
16964	202	0	19-Sep-13	18	1	12	2	1	1	0	11	1	3	2
16965	202	0	2-Aug-13	20	1	12	2	1	1	0	11	1	2	1
16966	202	0	7-Aug-12	18	1	12	2	1	1	0	11	1	3	1
16967	202	0	3-Jul-11	13	1	12	2	1	1	0	11	1	3	2
16968	202	0	27-Oct-15	16	1	12	2	3	1	0	3	1	2	1
16969	202	0	5-May-14	16	1	12	2	2	1	0	3	1	3	1
16970	202	0	1-Jun-13	11	1	12	2	3	1	0	3	1	4	1
16971	202	0	23-Dec-10	18	1	12	2	1	1	0	3	1	5	1
16972	202	0	25-Feb-06	19	1	12	2	1	1	0	3	1	3	1
16973	202	0	24-Aug-14	21	1	12	2	3	1	0	6	1	3	1
16974	202	0	29-Aug-09	20	1	12	2	1	1	0	6	1	2	1
16975	202	0	12-Aug-17	11	2	12	2	2	1	0	6	1	3	2
16976	202	0	16-Feb-14	22	2	12	2	3	1	0	10	1	7	2
16977	202	0	14-Apr-14	18	2	12	2	1	1	0	6	1	1	1
16978	202	0	30-Nov-08	20	2	12	2	1	1	0	10	1	7	2
16979	202	0	14-Feb-10	22	2	12	2	1	1	0	11	1	3	1
16980	202	0	19-Jun-10	2	2	12	2	1	1	0	11	1	2	1
16981	202	0	10-May-14	14	2	12	2	3	1	0	3	1	1	1

16982	202	0	27-Dec-09	20	2	12	2	1	1	0	3	1	1	2
16983	202	0	20-Sep-08	21	2	12	2	1	1	0	3	1	1	2
16984	202	0	21-Dec-11	20	2	12	2	3	1	0	6	1	2	1
16985	201	1	2-Jun-14	20	1	12	2	2	1	0	1	1	3	1
16986	201	1	23-Mar-12	20	1	12	2	3	1	0	6	1	2	1
16987	201	1	22-Nov-16	18	1	12	2	3	1	0	6	1	2	1
16988	201	1	24-Nov-15	16	1	12	2	2	1	0	6	1	2	1
16989	201	1	5-Jul-17	9	1	12	2	2	1	0	6	1	2	1
16990	201	1	2-Jun-17	21	1	12	2	1	1	0	6	1	2	1
16991	201	1	23-Jul-12	22	1	12	2	3	1	0	6	1	3	1
16992	101	1	12-Dec-12	7	1	12	2	1	1	0	7	1	4	4
16993	101	1	24-May-10	22	1	12	2	1	1	0	7	1	4	1
16994	101	1	7-Oct-13	12	1	12	2	1	1	0	4	1	2	1
16995	201	1	27-Jul-13	19	1	12	2	2	1	0	4	1	3	1
16996	101	1	21-Nov-12	23	1	12	2	1	1	0	4	1	4	1
16997	101	1	20-Apr-11	18	1	12	2	1	1	0	4	1	1	1
16998	201	1	5-May-09	20	1	12	2	2	1	0	4	1	2	1
16999	201	1	11-Jul-16	13	1	12	2	2	1	0	11	1	2	1
17000	201	1	25-Aug-16	20	1	12	2	1	1	0	11	1	2	1
17001	101	1	2-Dec-16	18	1	12	2	1	1	0	11	1	2	1
17002	101	1	6-Apr-15	14	1	12	2	1	1	0	11	1	2	1
17003	201	1	21-Dec-14	4	1	12	2	2	1	0	11	1	1	1
17004	101	1	21-May-14	18	1	12	2	1	1	0	11	1	2	1
17005	101	1	27-Nov-14	17	1	12	2	1	1	0	11	1	2	1
17006	101	1	17-Feb-14	21	1	12	2	1	1	0	11	1	2	1
17007	101	1	27-Oct-14	12	1	12	2	1	1	0	11	1	2	1
17008	201	1	7-Oct-14	9	1	12	2	2	1	0	11	1	2	1
17009	101	1	3-Feb-14	22	1	12	2	1	1	0	11	1	3	1
17010	101	1	3-Apr-14	17	1	12	2	1	1	0	11	1	2	1
17011	101	1	26-Sep-14	12	1	12	2	1	1	0	11	1	2	1
17012	201	1	20-Dec-14	15	1	12	2	2	1	0	11	1	2	1
17013	201	1	9-Aug-13	10	1	12	2	3	1	0	11	1	2	1
17014	101	1	11-Jun-13	11	1	12	2	1	1	0	11	1	3	1
17015	201	1	7-Dec-13	17	1	12	2	3	1	0	11	1	2	1
17016	101	1	6-Feb-13	20	1	12	2	1	1	0	11	1	2	1
17017	101	1	8-Sep-13	19	1	12	2	1	1	0	11	1	2	1
17018	101	1	12-Apr-13	22	1	12	2	1	1	0	11	1	2	1
17019	201	1	14-Jul-12	19	1	12	2	3	1	0	11	1	3	1
17020	201	1	23-Apr-12	9	1	12	2	1	1	0	11	1	3	1
17021	101	1	28-May-12	16	1	12	2	1	1	0	11	1	3	1
17022	101	1	22-Dec-12	17	1	12	2	1	1	0	11	1	1	1
17023	201	1	8-Jun-12	19	1	12	2	2	1	0	11	1	3	1
17024	101	1	20-Dec-12	19	1	12	2	1	1	0	11	1	1	1
17025	101	1	25-Aug-12	20	1	12	2	1	1	0	11	1	2	1
17026	201	1	7-Jan-12	14	1	12	2	2	1	0	11	1	2	1
17027	101	1	30-Jul-12	15	1	12	2	1	1	0	11	1	2	1
17028	101	1	3-Apr-12	9	1	12	2	1	1	0	11	1	3	1
17029	101	1	21-Jul-12	10	1	12	2	1	1	0	11	1	3	1
17030	101	1	6-Feb-12	13	1	12	2	1	1	0	11	1	3	1
17031	101	1	8-Jun-12	22	1	12	2	1	1	0	11	1	3	1
17032	101	1	9-Mar-12	19	1	12	2	1	1	0	11	1	1	1
17033	101	1	22-Aug-12	17	1	12	2	1	1	0	11	1	2	1
17034	201	1	1-Dec-12	17	1	12	2	3	1	0	11	1	2	1
17035	101	1	20-Dec-12	16	1	12	2	1	1	0	11	1	2	1
17036	101	1	10-Feb-12	11	1	12	2	1	1	0	11	1	3	1
17037	101	1	9-Apr-12	7	1	12	2	1	1	0	11	1	2	1
17038	101	1	26-Nov-11	14	1	12	2	1	1	0	11	1	1	1
17039	101	1	26-Feb-11	10	1	12	2	1	1	0	11	1	2	1
17040	201	1	29-Oct-11	8	1	12	2	3	1	0	11	1	2	1
17041	101	1	14-Dec-11	18	1	12	2	1	1	0	11	1	2	1
17042	101	1	5-Mar-11	21	1	12	2	1	1	0	11	1	4	1
17043	101	1	25-Apr-11	10	1	12	2	2	1	0	11	1	2	1
17044	101	1	17-Dec-11	21	1	12	2	1	1	0	11	1	3	1
17045	101	1	7-Oct-10	20	1	12	2	1	1	0	11	1	2	1
17046	101	1	17-Nov-10	8	1	12	2	1	1	0	11	1	2	1
17047	101	1	4-Aug-10	13	1	12	2	1	1	0	11	1	3	1
17048	201	1	17-Aug-09	13	1	12	2	2	1	0	11	1	2	1
17049	201	1	13-May-09	10	1	12	2	1	1	0	11	1	3	1
17050	101	1	25-Jan-09	18	1	12	2	1	1	0	11	1	1	1
17051	101	1	21-Oct-09	18	1	12	2	1	1	0	11	1	2	1
17052	101	1	25-Feb-09	18	1	12	2	1	1	0	11	1	4	1
17053	101	1	28-Dec-09	21	1	12	2	1	1	0	11	1	2	1
17054	101	1	17-Feb-09	23	1	12	2	1	1	0	11	1	2	1
17055	101	1	19-Apr-09	19	1	12	2	1	1	0	11	1	2	1
17056	101	1	15-Sep-09	9	1	12	2	1	1	0	11	1	2	1
17057	101	1	12-Aug-09	17	1	12	2	1	1	0	11	1	2	1
17058	201	1	19-Aug-08	10	1	12	2	1	1	0	11	1	3	1
17059	201	1	3-Jan-08	13	1	12	2	3	1	0	11	1	2	1
17060	101	1	21-Apr-07	19	1	12	2	1	1	0	11	1	4	1
17061	101	1	14-Oct-07	18	1	12	2	1	1	0	11	1	2	1
17062	101	1	16-Jun-17	19	1	12	2	1	1	0	11	1	2	1
17063	101	1	18-Apr-17	18	1	12	2	1	1	0	11	1	2	1
17064	201	1	23-Jan-17	15	1	12	2	1	1	0	11	1	2	1
17065	201	1	5-Aug-17	18	1	12	2	2	1	0	11	1	2	1
17066	101	1	8-Oct-17	11	1	12	2	1	1	0	11	1	3	1
17067	101	1	24-Nov-16	3	1	12	2	1	1	0	3	1	2	1
17068	201	1	15-Dec-16	20	1	12	2	1	1	0	3	1	1	1
17069	201	1	16-Nov-16	21	1	12	2	2	1	0	3	1	2	1
17070	101	1	6-Jun-15	20	1	12	2	1	1	0	3	1	1	1
17071	101	1	11-Oct-14	19	1	12	2	1	1	0	3	1	1	1
17072	201	1	10-Jun-14	22	1	12	2	3	1	0	3	1	1	1
17073	201	1	24-Mar-14	9	1	12	2	2	1	0	3	1	2	1
17074	201	1	6-Dec-14	9	1	12	2	1	1	0	3	1	2	1
17075	101	1	30-Nov-14	14	1	12	2	1	1	0	3	1	2	4
17076	201	1	17-Oct-13	3	1	12	2	1	1	0	3	1	4	1
17077	201	1	16-Dec-13	0	1	12	2	2	1	0	3	1	3	1
17078	201	1	24-Dec-13	20	1	12	2	1	1	0	3	1	2	1
17079	101	1	10-Sep-12	20	1	12	2	1	1	0	3	1	1	1
17080	101	1	23-May-12	11	1	12	2	1	1	0	3	1	1	1
17081	101	1	29-Feb-12	18	1	12	2	1	1	0	3	1	4	1
17082	101	1	1-Sep-12	18	1	12	2	1	1	0	3	1	3	4
17083	101	1	20-Oct-11	11	1	12	2	1	1	0	3	1	1	1
17084	101	1	11-Sep-11	19	1	12	2	1	1	0	3	1	2	4
17085	101	1	25-Apr-11	13	1	12	2	1	1	0	3	1	2	1
17086	101	1	7-Jun-11	23	1	12	2	1	1	0	3	1	3	1
17087	101	1	28-Aug-11	20	1	12	2	1	1	0	3	1	2	1
17088	101	1	6-Sep-08	22	1	12	2	1	1	0	3	1	2	1
17089	101	1	17-Sep-07	19	1	12	2	1	1	0	3	1	5	1
17090	101	1	30-Aug-17	12	1	12	2	1	1	0	3	1	1	1
17091	101	1	17-May-14	21	1	12	2	1	1	0	6	1	2	1
17092	101	1	23-Oct-14	12	1	12	2	1	1	0	6	1	2	1
17093	101	1	1-Aug-13	21	1	12	2	1	1	0	6	1	3	2
17094	101	1	28-Sep-12	18	1	12	2	1	1	0	6	1	2	1
17095	101	1	20-Oct-12	21	1	12	2	1	1	0	6	1	3	1
17096	101	1	17-Feb-11	21	1	12	2	1	1	0	6	1	1	1
17097	201	1	23-May-11	13	1	12	2	2	1	0	6	1	2	1
17098	101	1	31-Mar-11	18	1	12	2	1	1	0	6	1	2	1
17099	201	1	6-Jan-11	8	1	12	2	2	1	0	6	1	2	1
17100	201	1	14-Jan-10	19	1	12	2	2	1	0	6	1	2	1
17101	101	1	2-Aug-10	20	1	12	2	1	1	0	6	1	2	1
17102	101	1	16-Sep-09	18	1	12	2	1	1	0	6	1	1	1
17103	101	1	28-Sep-09	19	1	12	2	1	1	0	6	1	4	1
17104	101	1	27-Jul-07	22	1	12	2	1	1	0	6	1	2	1
17105	101	1	24-Dec-06	9	1	12	2	1	1	0	6	1	3	1

17106	101	1	21-Apr-06	23	1	12	2	1	1	0	6	1	3	1
17107	201	1	28-Sep-10	13	1	12	2	2	1	0	13	1	3	1
17108	101	1	6-Sep-06	18	1	12	2	1	1	0	13	1	7	1
17109	201	1	24-Sep-14	21	2	12	2	1	1	0	6	1	2	1
17110	101	1	19-Jan-16	21	2	12	2	1	1	0	6	1	2	1
17111	101	1	8-Mar-15	13	2	12	2	1	1	0	6	1	2	1
17112	101	1	23-Aug-14	22	2	12	2	1	1	0	3	1	3	1
17113	201	1	7-Oct-14	13	2	12	2	3	1	0	7	1	2	1
17114	101	1	25-Aug-12	13	2	12	2	1	1	0	7	1	2	1
17115	201	1	8-Dec-12	14	2	12	2	2	1	0	7	1	3	1
17116	101	1	30-Oct-11	9	2	12	2	1	1	0	7	1	4	1
17117	101	1	18-Sep-10	12	2	12	2	1	1	0	7	1	2	1
17118	101	1	30-Dec-10	0	2	12	2	1	1	0	7	1	3	1
17119	101	1	12-Apr-11	21	2	12	2	3	1	0	13	1	1	2
17120	101	1	6-Jul-12	14	2	12	2	1	1	0	4	1	1	1
17121	101	1	15-Sep-12	22	2	12	2	1	1	0	4	1	2	1
17122	101	1	7-Aug-11	16	2	12	2	1	1	0	4	1	2	1
17123	101	1	23-Jul-09	21	2	12	2	1	1	0	4	1	2	1
17124	201	1	22-Mar-09	12	2	12	2	3	1	0	4	1	3	1
17125	201	1	7-Aug-16	14	2	12	2	2	1	0	11	1	2	1
17126	101	1	20-Oct-16	22	2	12	2	1	1	0	11	1	2	1
17127	101	1	26-Feb-15	20	2	12	2	1	1	0	11	1	2	1
17128	201	1	11-Oct-15	14	2	12	2	2	1	0	11	1	2	1
17129	101	1	8-Jul-15	19	2	12	2	1	1	0	11	1	2	1
17130	101	1	19-Mar-15	11	2	12	2	1	1	0	11	1	2	1
17131	201	1	13-Oct-15	16	2	12	2	1	1	0	11	1	2	1
17132	101	1	27-Oct-15	20	2	12	2	1	1	0	11	1	3	1
17133	101	1	10-Oct-14	19	2	12	2	1	1	0	11	1	1	1
17134	101	1	22-Jul-14	16	2	12	2	1	1	0	11	1	2	1
17135	201	1	14-Sep-14	9	2	12	2	2	1	0	11	1	2	1
17136	101	1	10-Feb-14	20	2	12	2	1	1	0	11	1	2	1
17137	101	1	20-Nov-13	20	2	12	2	1	1	0	11	1	2	1
17138	201	1	23-Aug-13	9	2	12	2	2	1	0	11	1	2	1
17139	101	1	17-Mar-13	20	2	12	2	1	1	0	11	1	2	1
17140	101	1	1-Mar-13	8	2	12	2	1	1	0	11	1	3	1
17141	101	1	11-Aug-12	12	2	12	2	1	1	0	11	1	5	2
17142	201	1	23-Apr-12	6	2	12	2	3	1	0	11	1	2	1
17143	101	1	12-Oct-12	15	2	12	2	1	1	0	11	1	2	1
17144	101	1	26-Feb-12	19	2	12	2	1	1	0	11	1	2	1
17145	101	1	14-Jun-12	12	2	12	2	1	1	0	11	1	2	1
17146	101	1	5-Dec-12	17	2	12	2	1	1	0	11	1	2	1
17147	101	1	26-Oct-12	12	2	12	2	1	1	0	11	1	2	1
17148	101	1	11-Sep-12	8	2	12	2	1	1	0	11	1	3	1
17149	101	1	18-Jan-12	10	2	12	2	1	1	0	11	1	2	1
17150	201	1	2-Oct-12	10	2	12	2	3	1	0	11	1	2	1
17151	101	1	23-May-12	19	2	12	2	1	1	0	11	1	2	1
17152	101	1	16-Feb-12	20	2	12	2	1	1	0	11	1	2	1
17153	201	1	13-Jul-11	13	2	12	2	2	1	0	11	1	2	1
17154	101	1	16-Jul-11	17	2	12	2	1	1	0	11	1	2	1
17155	101	1	25-Dec-11	21	2	12	2	1	1	0	11	1	2	1
17156	101	1	1-Jan-11	8	2	12	2	1	1	0	11	1	2	1
17157	101	1	7-Jul-11	15	2	12	2	1	1	0	11	1	3	1
17158	101	1	31-Mar-11	19	2	12	2	1	1	0	11	1	3	1
17159	101	1	9-Nov-11	20	2	12	2	1	1	0	11	1	3	1
17160	101	1	2-Apr-11	20	2	12	2	1	1	0	11	1	2	1
17161	101	1	25-Jun-10	23	2	12	2	1	1	0	11	1	2	1
17162	101	1	25-Oct-10	21	2	12	2	1	1	0	11	1	2	1
17163	101	1	18-Sep-10	20	2	12	2	1	1	0	11	1	3	1
17164	101	1	28-Sep-10	3	2	12	2	1	1	0	11	1	3	2
17165	101	1	25-Sep-10	20	2	12	2	1	1	0	11	1	3	1
17166	201	1	16-Aug-09	19	2	12	2	3	1	0	11	1	3	1
17167	101	1	6-Oct-09	23	2	12	2	1	1	0	11	1	2	1
17168	101	1	12-Apr-07	22	2	12	2	1	1	0	11	1	2	1
17169	101	1	7-Oct-06	18	2	12	2	1	1	0	11	1	3	1
17170	101	1	30-May-17	17	2	12	2	1	1	0	11	1	2	1
17171	101	1	12-Jun-17	12	2	12	2	1	1	0	11	1	2	1
17172	101	1	11-Jan-17	10	2	12	2	1	1	0	11	1	1	1
17173	101	1	29-Aug-17	12	2	12	2	1	1	0	11	1	3	1
17174	101	1	12-Nov-17	13	2	12	2	1	1	0	11	1	2	1
17175	101	1	12-Jul-15	0	2	12	2	1	1	0	3	1	1	2
17176	101	1	8-Feb-14	23	2	12	2	1	1	0	3	1	1	1
17177	101	1	31-Oct-13	20	2	12	2	1	1	0	3	1	3	1
17178	101	1	11-May-12	21	2	12	2	1	1	0	3	1	1	1
17179	101	1	30-Jul-12	19	2	12	2	1	1	0	3	1	1	1
17180	101	1	8-Dec-10	1	2	12	2	3	1	0	3	1	1	2
17181	201	1	10-Apr-09	19	2	12	2	3	1	0	3	1	4	1
17182	101	1	25-Sep-17	20	2	12	2	1	1	0	3	1	1	1
17183	101	1	25-Dec-14	16	2	12	2	1	1	0	6	1	2	1
17184	201	1	6-Oct-12	22	2	12	2	3	1	0	6	1	4	1
17185	101	1	14-Oct-11	18	2	12	2	1	1	0	6	1	2	1
17186	101	1	31-Jan-10	11	2	12	2	1	1	0	6	1	2	1
17187	101	1	12-Jun-08	22	2	12	2	1	1	0	6	1	3	1
17188	101	1	24-Feb-08	19	2	12	2	1	1	0	6	1	2	1
17189	101	1	17-Dec-06	9	2	12	2	1	1	0	6	1	2	1
17190	101	1	29-Oct-06	12	2	12	2	1	1	0	6	1	5	1
17191	202	0	14-Feb-14	9	1	13	2	1	1	0	10	1	7	2
17192	202	0	16-Feb-16	12	1	13	2	3	1	0	7	1	3	2
17193	202	0	20-Aug-15	17	1	13	2	3	1	0	6	1	2	1
17194	202	0	29-Feb-12	19	1	13	2	1	1	0	4	1	1	2
17195	202	0	30-Aug-17	16	1	13	2	1	1	0	4	1	3	1
17196	202	0	11-Nov-15	14	1	13	2	1	1	0	11	1	1	1
17197	202	0	14-Jul-14	20	1	13	2	1	1	0	11	1	3	1
17198	202	0	10-Jan-14	15	1	13	2	1	1	0	11	1	3	1
17199	202	0	22-Jul-13	9	1	13	2	3	1	0	11	1	3	1
17200	202	0	25-Nov-13	21	1	13	2	1	1	0	11	1	4	1
17201	202	0	2-May-12	21	1	13	2	1	1	0	11	1	3	1
17202	202	0	17-Jul-11	8	1	13	2	1	1	0	11	1	3	2
17203	202	0	14-Sep-11	15	1	13	2	1	1	0	11	1	2	1
17204	202	0	22-Jun-10	9	1	13	2	1	1	0	11	1	3	1
17205	202	0	9-Oct-09	14	1	13	2	1	1	0	11	1	2	1
17206	202	0	4-Oct-07	18	1	13	2	1	1	0	11	1	4	1
17207	202	0	29-Nov-06	17	1	13	2	3	1	0	11	1	3	1
17208	202	0	16-Jun-17	21	1	13	2	1	1	0	11	1	3	1
17209	202	0	10-May-17	17	1	13	2	1	1	0	11	1	2	1
17210	202	0	1-Feb-17	21	1	13	2	3	1	0	11	1	3	1
17211	202	0	18-Mar-17	21	1	13	2	3	1	0	11	1	2	1
17212	202	0	13-Jul-16	17	1	13	2	1	1	0	3	1	1	1
17213	202	0	18-Sep-15	15	1	13	2	1	1	0	3	1	2	1
17214	202	0	20-Jun-15	0	1	13	2	1	1	0	3	1	2	1
17215	202	0	20-Jun-15	14	1	13	2	1	1	0	3	1	3	1
17216	202	0	2-Apr-13	17	1	13	2	2	1	0	3	1	1	1
17217	202	0	22-Sep-13	21	1	13	2	1	1	0	3	1	2	2
17218	202	0	8-Sep-12	20	1	13	2	1	1	0	3	1	1	2
17219	202	0	6-Apr-12	22	1	13	2	1	1	0	3	1	2	1
17220	202	0	10-Dec-11	16	1	13	2	3	1	0	3	1	5	1
17221	202	0	20-Aug-11	20	1	13	2	3	1	0	3	1	1	2
17222	202	0	26-Jul-11	22	1	13	2	1	1	0	3	1	1	1
17223	202	0	21-Jun-10	20	1	13	2	2	1	0	3	1	6	2
17224	202	0	16-Dec-10	18	1	13	2	1	1	0	3	1	2	2
17225	202	0	23-Feb-10	17	1	13	2	1	1	0	3	1	2	2
17226	202	0	12-Jul-09	12	1	13	2	3	1	0	3	1	1	1
17227	202	0	5-Jan-14	17	1	13	2	1	1	0	6	1	1	1
17228	202	0	1-Jul-12	18	1	13	2	1	1	0	6	1	2	1
17229	202	0	19-Jun-10	10	1	13	2	1	1	0	6	1	3	1

17230	202	0	4-Jul-07	19	1	13	2	1	1	0	6	1	2	1
17231	202	0	2-Mar-07	12	1	13	2	1	1	0	6	1	2	1
17232	202	0	22-Aug-07	11	1	13	2	1	1	0	6	1	3	2
17233	202	0	26-Jan-06	14	1	13	2	1	1	0	6	1	2	1
17234	202	0	12-Feb-17	17	2	13	2	1	1	0	10	1	1	2
17235	202	0	31-May-15	18	2	13	2	1	1	0	7	1	4	1
17236	202	0	25-Jun-13	16	2	13	2	1	1	0	7	1	1	1
17237	202	0	20-Apr-10	19	2	13	2	1	1	0	7	1	3	2
17238	202	0	2-May-11	21	2	13	2	1	1	0	4	1	2	1
17239	202	0	12-Dec-09	18	2	13	2	1	1	0	4	1	2	1
17240	202	0	7-Feb-14	19	2	13	2	3	1	0	11	1	3	1
17241	202	0	19-Aug-14	9	2	13	2	3	1	0	11	1	2	1
17242	202	0	20-Aug-14	18	2	13	2	1	1	0	11	1	2	1
17243	202	0	5-Oct-13	20	2	13	2	2	1	0	11	1	3	1
17244	202	0	26-Oct-13	14	2	13	2	1	1	0	11	1	2	1
17245	202	0	24-Jan-11	0	2	13	2	1	1	0	11	1	2	1
17246	202	0	6-Sep-09	20	2	13	2	1	1	0	11	1	3	1
17247	202	0	2-Jul-17	21	2	13	2	1	1	0	11	1	2	1
17248	202	0	5-Nov-16	0	2	13	2	2	1	0	3	1	2	1
17249	202	0	6-Dec-15	19	2	13	2	1	1	0	3	1	6	1
17250	202	0	30-Oct-14	10	2	13	2	1	1	0	3	1	1	1
17251	202	0	30-May-13	9	2	13	2	1	1	0	3	1	3	1
17252	202	0	5-Oct-12	21	2	13	2	1	1	0	3	1	1	2
17253	202	0	4-Aug-10	22	2	13	2	1	1	0	3	1	1	2
17254	202	0	16-Feb-10	17	2	13	2	1	1	0	3	1	2	1
17255	202	0	27-Sep-09	18	2	13	2	1	1	0	3	1	1	1
17256	202	0	12-Apr-06	6	2	13	2	1	1	0	3	1	1	1
17257	202	0	23-Jan-13	17	2	13	2	1	1	0	6	1	2	1
17258	202	0	1-Apr-12	21	2	13	2	1	1	0	6	1	3	2
17259	202	0	22-Aug-11	16	2	13	2	1	1	0	6	1	3	1
17260	202	0	9-Aug-08	21	2	13	2	1	1	0	6	1	7	1
17261	202	0	13-Dec-07	20	2	13	2	1	1	0	6	1	3	1
17262	202	0	28-Oct-07	20	2	13	2	1	1	0	6	1	3	1
17263	202	0	24-Jun-06	1	2	13	2	1	1	0	6	1	2	1
17264	101	1	17-Jan-17	4	1	13	2	1	1	0	3	1	5	1
17265	201	1	18-Apr-15	19	1	13	2	1	1	0	6	1	3	1
17266	201	1	2-May-13	20	1	13	2	1	1	0	6	1	2	1
17267	201	1	28-Sep-12	21	1	13	2	3	1	0	6	1	2	1
17268	201	1	23-Sep-16	22	1	13	2	3	1	0	6	1	2	1
17269	201	1	15-Oct-16	10	1	13	2	2	1	0	6	1	2	1
17270	201	1	15-Feb-16	18	1	13	2	1	1	0	6	1	2	1
17271	201	1	21-Jan-17	10	1	13	2	1	1	0	6	1	2	1
17272	101	1	19-Oct-17	19	1	13	2	1	1	0	6	1	2	1
17273	101	1	5-Feb-13	22	1	13	2	1	1	0	6	1	3	1
17274	201	1	8-Sep-17	16	1	13	2	1	1	0	3	1	1	1
17275	201	1	13-Oct-16	10	1	13	2	2	1	0	7	1	4	1
17276	101	1	13-Aug-17	21	1	13	2	1	1	0	11	1	2	1
17277	101	1	27-Feb-15	14	1	13	2	3	1	0	6	1	2	1
17278	201	1	1-Nov-17	21	1	13	2	1	1	0	6	1	2	1
17279	201	1	4-Apr-12	11	1	13	2	1	1	0	6	1	3	1
17280	201	1	2-Feb-08	18	1	13	2	1	1	0	10	1	7	1
17281	201	1	12-Oct-16	15	1	13	2	3	1	0	7	1	4	1
17282	101	1	1-Jan-16	21	1	13	2	1	1	0	7	1	5	4
17283	101	1	17-Sep-16	17	1	13	2	1	1	0	7	1	2	1
17284	101	1	4-Aug-16	21	1	13	2	1	1	0	7	1	3	1
17285	101	1	11-Aug-16	18	1	13	2	1	1	0	7	1	3	1
17286	101	1	17-Feb-14	19	1	13	2	1	1	0	7	1	1	2
17287	101	1	12-Feb-14	0	1	13	2	1	1	0	7	1	5	2
17288	101	1	12-Nov-14	12	1	13	2	1	1	0	7	1	3	1
17289	101	1	9-Mar-12	20	1	13	2	1	1	0	7	1	3	1
17290	201	1	7-Jul-11	19	1	13	2	2	1	0	7	1	3	1
17291	101	1	5-Jul-10	13	1	13	2	1	1	0	7	1	4	1
17292	101	1	3-Feb-08	15	1	13	2	1	1	0	7	1	2	1
17293	101	1	9-Oct-08	22	1	13	2	1	1	0	7	1	3	1
17294	101	1	4-Dec-07	20	1	13	2	1	1	0	13	1	2	1
17295	101	1	16-Aug-06	1	1	13	2	1	1	0	13	1	2	2
17296	201	1	15-Jun-06	12	1	13	2	1	1	0	13	1	1	1
17297	101	1	5-Oct-11	17	1	13	2	1	1	0	1	1	4	2
17298	101	1	12-Mar-15	10	1	13	2	1	1	0	4	1	2	1
17299	101	1	13-Jun-14	22	1	13	2	1	1	0	4	1	2	1
17300	201	1	6-Sep-12	6	1	13	2	2	1	0	4	1	1	1
17301	201	1	20-Dec-10	18	1	13	2	2	1	0	4	1	2	1
17302	201	1	16-Jun-10	9	1	13	2	2	1	0	4	1	2	1
17303	201	1	5-Feb-09	11	1	13	2	1	1	0	4	1	1	1
17304	101	1	5-Mar-09	20	1	13	2	1	1	0	4	1	1	1
17305	201	1	9-Jan-09	15	1	13	2	1	1	0	4	1	2	1
17306	101	1	15-Apr-09	14	1	13	2	1	1	0	4	1	2	1
17307	201	1	9-Sep-09	21	1	13	2	2	1	0	4	1	2	1
17308	101	1	23-Aug-09	1	1	13	2	1	1	0	4	1	2	1
17309	101	1	13-Jul-08	8	1	13	2	1	1	0	4	1	1	1
17310	101	1	20-Feb-08	20	1	13	2	2	1	0	4	1	1	1
17311	101	1	17-Mar-07	15	1	13	2	1	1	0	4	1	1	1
17312	101	1	8-Mar-17	19	1	13	2	1	1	0	4	1	1	1
17313	101	1	20-Oct-17	21	1	13	2	1	1	0	4	1	2	1
17314	201	1	29-Jan-16	10	1	13	2	1	1	0	11	1	2	1
17315	201	1	10-Nov-16	19	1	13	2	1	1	0	11	1	1	1
17316	201	1	28-Feb-16	21	1	13	2	1	1	0	11	1	2	1
17317	101	1	31-May-16	21	1	13	2	1	1	0	11	1	3	1
17318	201	1	9-Oct-15	18	1	13	2	1	1	0	11	1	4	1
17319	101	1	19-Feb-15	13	1	13	2	1	1	0	11	1	2	1
17320	101	1	28-Dec-15	22	1	13	2	1	1	0	11	1	1	1
17321	101	1	28-Feb-15	13	1	13	2	1	1	0	11	1	2	1
17322	101	1	5-Jan-15	18	1	13	2	1	1	0	11	1	2	1
17323	101	1	17-Feb-15	15	1	13	2	1	1	0	11	1	2	1
17324	101	1	8-Feb-15	16	1	13	2	1	1	0	11	1	2	1
17325	101	1	8-Oct-15	20	1	13	2	1	1	0	11	1	3	1
17326	201	1	17-May-14	18	1	13	2	1	1	0	11	1	2	1
17327	101	1	6-Nov-14	19	1	13	2	1	1	0	11	1	2	1
17328	101	1	23-Oct-14	18	1	13	2	1	1	0	11	1	2	1
17329	101	1	2-Apr-14	12	1	13	2	1	1	0	11	1	2	1
17330	101	1	1-Jan-14	19	1	13	2	1	1	0	11	1	2	1
17331	201	1	11-Feb-13	15	1	13	2	3	1	0	11	1	3	1
17332	101	1	13-Oct-13	16	1	13	2	1	1	0	11	1	2	1
17333	101	1	13-Aug-13	14	1	13	2	2	1	0	11	1	2	1
17334	101	1	11-Oct-13	17	1	13	2	2	1	0	11	1	1	1
17335	101	1	21-Apr-13	16	1	13	2	3	1	0	11	1	3	1
17336	101	1	29-Sep-13	12	1	13	2	1	1	0	11	1	2	1
17337	101	1	29-Sep-13	14	1	13	2	1	1	0	11	1	2	2
17338	101	1	25-Apr-12	20	1	13	2	1	1	0	11	1	2	1
17339	101	1	25-May-12	23	1	13	2	1	1	0	11	1	1	1
17340	201	1	24-Jul-12	13	1	13	2	2	1	0	11	1	2	1
17341	101	1	25-Dec-12	20	1	13	2	1	1	0	11	1	2	1
17342	101	1	14-Dec-12	10	1	13	2	1	1	0	11	1	2	1
17343	101	1	10-Jun-12	19	1	13	2	1	1	0	11	1	2	1
17344	101	1	29-Dec-12	12	1	13	2	1	1	0	11	1	2	1
17345	101	1	24-Sep-12	14	1	13	2	1	1	0	11	1	3	1
17346	101	1	12-Sep-12	20	1	13	2	1	1	0	11	1	3	1
17347	101	1	2-Jul-12	12	1	13	2	1	1	0	11	1	3	1
17348	101	1	21-Oct-12	7	1	13	2	1	1	0	11	1	3	1
17349	101	1	17-Jul-12	10	1	13	2	1	1	0	11	1	3	1
17350	101	1	4-Aug-12	4	1	13	2	1	1	0	11	1	3	1
17351	101	1	4-Nov-12	7	1	13	2	1	1	0	11	1	2	1
17352	101	1	9-Feb-12	13	1	13	2	1	1	0	11	1	2	1
17353	101	1	8-May-12	12	1	13	2	1	1	0	11	1	2	1

17354	201	1	21-Jul-12	22	1	13	2	2	1	0	11	1	2	1
17355	101	1	28-Oct-11	7	1	13	2	1	1	0	11	1	1	1
17356	201	1	27-Apr-11	10	1	13	2	1	1	0	11	1	3	1
17357	201	1	1-Feb-11	15	1	13	2	2	1	0	11	1	3	1
17358	101	1	1-Sep-11	9	1	13	2	1	1	0	11	1	2	2
17359	101	1	30-May-11	12	1	13	2	1	1	0	11	1	2	1
17360	101	1	14-Aug-11	12	1	13	2	1	1	0	11	1	3	1
17361	101	1	18-Sep-11	15	1	13	2	1	1	0	11	1	3	1
17362	101	1	26-Oct-11	7	1	13	2	1	1	0	11	1	2	1
17363	101	1	23-Aug-11	16	1	13	2	1	1	0	11	1	3	1
17364	101	1	27-Nov-10	22	1	13	2	1	1	0	11	1	1	1
17365	101	1	16-May-10	19	1	13	2	1	1	0	11	1	3	1
17366	101	1	28-Oct-10	2	1	13	2	1	1	0	11	1	1	1
17367	201	1	6-Oct-10	20	1	13	2	3	1	0	11	1	2	1
17368	101	1	14-Jun-10	13	1	13	2	1	1	0	11	1	2	1
17369	101	1	10-Oct-10	18	1	13	2	1	1	0	11	1	2	1
17370	201	1	27-Jun-10	19	1	13	2	2	1	0	11	1	2	1
17371	101	1	27-Apr-10	13	1	13	2	1	1	0	11	1	2	1
17372	101	1	6-Jul-10	18	1	13	2	1	1	0	11	1	2	1
17373	101	1	8-Oct-10	8	1	13	2	1	1	0	11	1	2	1
17374	101	1	1-May-10	21	1	13	2	1	1	0	11	1	3	1
17375	201	1	25-Aug-09	18	1	13	2	2	1	0	11	1	2	1
17376	201	1	25-Aug-09	19	1	13	2	1	1	0	11	1	2	1
17377	201	1	22-Sep-09	21	1	13	2	1	1	0	11	1	2	1
17378	101	1	20-Aug-09	21	1	13	2	1	1	0	11	1	3	1
17379	101	1	23-Oct-09	17	1	13	2	1	1	0	11	1	2	1
17380	101	1	6-Aug-09	8	1	13	2	1	1	0	11	1	3	1
17381	101	1	20-Oct-09	21	1	13	2	1	1	0	11	1	2	1
17382	201	1	24-Jul-08	9	1	13	2	2	1	0	11	1	3	1
17383	201	1	27-Jun-08	16	1	13	2	2	1	0	11	1	2	1
17384	201	1	3-Jan-08	12	1	13	2	1	1	0	11	1	2	1
17385	201	1	6-Feb-08	8	1	13	2	2	1	0	11	1	2	1
17386	101	1	13-Jul-08	19	1	13	2	1	1	0	11	1	2	1
17387	201	1	9-Nov-07	20	1	13	2	3	1	0	11	1	2	1
17388	101	1	20-Jun-06	18	1	13	2	1	1	0	11	1	4	1
17389	201	1	8-Oct-17	18	1	13	2	1	1	0	11	1	3	1
17390	201	1	29-Oct-17	10	1	13	2	1	1	0	11	1	3	1
17391	101	1	17-Apr-17	17	1	13	2	1	1	0	11	1	2	1
17392	101	1	13-Sep-17	7	1	13	2	1	1	0	11	1	2	1
17393	101	1	2-Jan-17	11	1	13	2	1	1	0	11	1	2	1
17394	101	1	18-Feb-17	23	1	13	2	1	1	0	11	1	2	1
17395	201	1	7-Jan-17	10	1	13	2	1	1	0	11	1	2	1
17396	201	1	7-Aug-17	12	1	13	2	1	1	0	11	1	2	1
17397	201	1	8-Aug-17	5	1	13	2	2	1	0	11	1	3	1
17398	101	1	21-Oct-17	21	1	13	2	1	1	0	11	1	3	1
17399	101	1	18-Aug-17	12	1	13	2	1	1	0	11	1	2	1
17400	101	1	13-Sep-17	2	1	13	2	1	1	0	11	1	2	1
17401	201	1	25-Jun-16	22	1	13	2	1	1	0	3	1	2	1
17402	201	1	8-Nov-16	20	1	13	2	2	1	0	3	1	1	1
17403	201	1	19-Jan-16	12	1	13	2	1	1	0	3	1	2	1
17404	201	1	14-Jul-16	15	1	13	2	2	1	0	3	1	2	1
17405	101	1	9-Sep-15	7	1	13	2	1	1	0	3	1	1	1
17406	101	1	3-Jul-15	19	1	13	2	1	1	0	3	1	1	1
17407	101	1	4-Feb-15	3	1	13	2	1	1	0	3	1	4	1
17408	201	1	8-Sep-15	21	1	13	2	1	1	0	3	1	1	1
17409	201	1	11-Jun-15	14	1	13	2	1	1	0	3	1	1	1
17410	101	1	9-Sep-15	12	1	13	2	1	1	0	3	1	3	2
17411	201	1	2-Apr-14	20	1	13	2	2	1	0	3	1	1	1
17412	101	1	8-Dec-14	20	1	13	2	1	1	0	3	1	3	1
17413	201	1	18-Mar-14	20	1	13	2	1	1	0	3	1	2	1
17414	201	1	13-Jun-14	14	1	13	2	3	1	0	3	1	2	1
17415	101	1	20-Oct-13	19	1	13	2	1	1	0	3	1	2	2
17416	201	1	20-Nov-13	9	1	13	2	3	1	0	3	1	1	1
17417	101	1	10-Jan-13	19	1	13	2	1	1	0	3	1	1	1
17418	101	1	24-Oct-13	17	1	13	2	1	1	0	3	1	2	2
17419	201	1	31-Dec-13	12	1	13	2	2	1	0	3	1	2	1
17420	201	1	17-Nov-13	16	1	13	2	2	1	0	3	1	2	1
17421	201	1	8-Jun-12	9	1	13	2	2	1	0	3	1	3	1
17422	201	1	10-Mar-12	11	1	13	2	1	1	0	3	1	1	1
17423	201	1	19-Jun-12	8	1	13	2	1	1	0	3	1	4	1
17424	101	1	23-Jan-12	18	1	13	2	1	1	0	3	1	3	1
17425	101	1	16-Nov-12	21	1	13	2	1	1	0	3	1	3	1
17426	101	1	13-Dec-12	11	1	13	2	1	1	0	3	1	1	1
17427	101	1	9-Oct-12	20	1	13	2	1	1	0	3	1	2	1
17428	201	1	19-Aug-12	13	1	13	2	2	1	0	3	1	3	1
17429	201	1	5-Mar-12	20	1	13	2	2	1	0	3	1	4	1
17430	101	1	16-Aug-12	20	1	13	2	1	1	0	3	1	2	1
17431	101	1	22-Apr-11	16	1	13	2	1	1	0	3	1	3	1
17432	201	1	8-Feb-11	12	1	13	2	2	1	0	3	1	1	2
17433	201	1	10-Jun-11	21	1	13	2	1	1	0	3	1	3	1
17434	201	1	8-Oct-11	11	1	13	2	2	1	0	3	1	3	1
17435	201	1	15-Jun-11	20	1	13	2	1	1	0	3	1	3	1
17436	201	1	27-Apr-10	20	1	13	2	1	1	0	3	1	5	1
17437	201	1	5-Sep-10	0	1	13	2	1	1	0	3	1	1	1
17438	201	1	29-Jul-10	23	1	13	2	3	1	0	3	1	3	1
17439	201	1	22-May-10	22	1	13	2	1	1	0	3	1	4	1
17440	101	1	10-Aug-09	18	1	13	2	1	1	0	3	1	1	2
17441	101	1	28-Jan-09	22	1	13	2	1	1	0	3	1	3	1
17442	201	1	3-Nov-08	18	1	13	2	3	1	0	3	1	3	1
17443	101	1	7-Apr-08	20	1	13	2	1	1	0	3	1	2	1
17444	201	1	24-Sep-08	22	1	13	2	2	1	0	3	1	3	1
17445	201	1	24-Dec-08	20	1	13	2	3	1	0	3	1	3	1
17446	101	1	21-Aug-07	19	1	13	2	1	1	0	3	1	1	1
17447	101	1	23-Jul-07	21	1	13	2	1	1	0	3	1	1	1
17448	201	1	15-Sep-06	18	1	13	2	1	1	0	3	1	1	1
17449	201	1	24-Sep-06	23	1	13	2	1	1	0	3	1	1	1
17450	201	1	14-Sep-06	19	1	13	2	1	1	0	3	1	1	1
17451	101	1	8-Aug-06	20	1	13	2	1	1	0	3	1	2	1
17452	201	1	5-Sep-17	20	1	13	2	1	1	0	3	1	1	2
17453	101	1	7-Sep-16	18	1	13	2	1	1	0	6	1	2	1
17454	101	1	7-Jan-16	19	1	13	2	1	1	0	6	1	2	1
17455	201	1	18-Jun-15	8	1	13	2	2	1	0	6	1	2	1
17456	101	1	26-Feb-15	10	1	13	2	1	1	0	6	1	3	1
17457	101	1	13-Jun-14	17	1	13	2	1	1	0	6	1	2	1
17458	101	1	12-Aug-14	18	1	13	2	1	1	0	6	1	2	1
17459	101	1	16-Jan-13	23	1	13	2	1	1	0	6	1	3	1
17460	101	1	28-Jun-13	14	1	13	2	1	1	0	6	1	2	1
17461	101	1	31-May-13	20	1	13	2	1	1	0	6	1	2	1
17462	101	1	31-Aug-13	14	1	13	2	1	1	0	6	1	2	1
17463	101	1	22-May-13	7	1	13	2	3	1	0	6	1	2	1
17464	101	1	30-Mar-12	17	1	13	2	1	1	0	6	1	2	1
17465	101	1	28-Sep-12	17	1	13	2	2	1	0	6	1	3	1
17466	101	1	30-Mar-11	20	1	13	2	1	1	0	6	1	3	1
17467	101	1	4-Dec-11	21	1	13	2	1	1	0	6	1	2	1
17468	101	1	9-Dec-11	23	1	13	2	1	1	0	6	1	3	1
17469	101	1	16-Jun-11	17	1	13	2	1	1	0	6	1	2	1
17470	201	1	11-May-10	17	1	13	2	1	1	0	6	1	2	1
17471	101	1	13-Aug-10	12	1	13	2	1	1	0	6	1	4	1
17472	101	1	26-Jan-10	18	1	13	2	1	1	0	6	1	3	1
17473	101	1	23-May-10	16	1	13	2	1	1	0	6	1	2	1
17474	201	1	18-Mar-10	17	1	13	2	1	1	0	6	1	2	1
17475	201	1	19-Aug-09	20	1	13	2	1	1	0	6	1	2	1
17476	201	1	3-Feb-09	21	1	13	2	1	1	0	6	1	2	1
17477	201	1	15-May-09	21	1	13	2	3	1	0	6	1	3	1

17478	101	1	2-Jul-09	17	1	13	2	1	1	0	6	1	3	1
17479	101	1	17-Oct-09	17	1	13	2	1	1	0	6	1	2	1
17480	101	1	2-Aug-09	11	1	13	2	1	1	0	6	1	2	1
17481	201	1	9-May-08	18	1	13	2	2	1	0	6	1	2	1
17482	101	1	1-Jun-08	1	1	13	2	1	1	0	6	1	3	1
17483	201	1	24-May-08	16	1	13	2	2	1	0	6	1	2	1
17484	101	1	11-Nov-08	17	1	13	2	1	1	0	6	1	2	1
17485	201	1	12-May-07	21	1	13	2	1	1	0	6	1	3	1
17486	201	1	9-Jun-07	19	1	13	2	1	1	0	6	1	3	1
17487	101	1	2-Oct-07	20	1	13	2	1	1	0	6	1	3	1
17488	101	1	29-Aug-07	0	1	13	2	1	1	0	6	1	2	1
17489	101	1	26-Jul-06	20	1	13	2	1	1	0	6	1	2	1
17490	201	1	26-Oct-06	13	1	13	2	2	1	0	6	1	2	1
17491	101	1	7-Aug-06	18	1	13	2	2	1	0	6	1	2	1
17492	101	1	22-Jan-17	3	1	13	2	1	1	0	6	1	3	1
17493	101	1	23-Mar-17	19	1	13	2	1	1	0	6	1	2	1
17494	101	1	21-Jan-12	20	1	13	2	1	1	0	13	1	1	1
17495	101	1	29-May-11	21	1	13	2	1	1	0	13	1	1	1
17496	201	1	9-Nov-17	21	2	13	2	1	1	0	4	1	3	1
17497	201	1	28-Feb-12	21	2	13	2	1	1	0	10	1	7	2
17498	201	1	26-May-16	6	2	13	2	1	1	0	4	1	2	1
17499	101	1	15-Apr-17	2	2	13	2	1	1	0	6	1	2	1
17500	201	1	1-Aug-17	14	2	13	2	1	1	0	6	1	2	1
17501	201	1	20-Jun-16	18	2	13	2	1	1	0	7	1	3	1
17502	101	1	18-Dec-16	15	2	13	2	1	1	0	7	1	3	1
17503	101	1	21-Jul-11	20	2	13	2	1	1	0	7	1	3	1
17504	101	1	12-Jan-11	12	2	13	2	1	1	0	7	1	3	1
17505	101	1	20-Sep-09	11	2	13	2	2	1	0	7	1	1	1
17506	101	1	26-Oct-09	17	2	13	2	1	1	0	7	1	3	2
17507	201	1	21-Jun-08	20	2	13	2	1	1	0	7	1	3	1
17508	201	1	17-Jun-17	22	2	13	2	1	1	0	7	1	3	1
17509	201	1	27-Jul-14	21	2	13	2	2	1	0	4	1	2	1
17510	101	1	15-Nov-12	13	2	13	2	1	1	0	4	1	2	1
17511	201	1	3-Sep-09	18	2	13	2	1	1	0	4	1	2	1
17512	101	1	13-Feb-16	16	2	13	2	1	1	0	11	1	1	1
17513	101	1	28-Feb-16	22	2	13	2	1	1	0	11	1	2	1
17514	101	1	2-Jun-16	11	2	13	2	1	1	0	11	1	2	1
17515	101	1	5-Apr-16	20	2	13	2	1	1	0	11	1	3	1
17516	101	1	3-Mar-16	12	2	13	2	1	1	0	11	1	2	1
17517	101	1	9-Jul-16	16	2	13	2	1	1	0	11	1	2	1
17518	101	1	15-Jun-16	21	2	13	2	1	1	0	11	1	2	1
17519	201	1	17-Sep-15	10	2	13	2	2	1	0	11	1	2	1
17520	101	1	27-Jun-15	17	2	13	2	1	1	0	11	1	2	1
17521	101	1	17-Apr-15	12	2	13	2	1	1	0	11	1	3	1
17522	101	1	14-Jan-15	4	2	13	2	1	1	0	11	1	2	1
17523	201	1	8-Apr-14	9	2	13	2	1	1	0	11	1	2	1
17524	101	1	18-Aug-14	22	2	13	2	1	1	0	11	1	1	1
17525	101	1	18-Nov-14	19	2	13	2	1	1	0	11	1	2	1
17526	101	1	6-Mar-14	8	2	13	2	1	1	0	11	1	3	1
17527	101	1	25-Mar-14	11	2	13	2	1	1	0	11	1	2	1
17528	101	1	21-Feb-14	20	2	13	2	1	1	0	11	1	2	1
17529	101	1	26-Nov-14	20	2	13	2	1	1	0	11	1	2	1
17530	101	1	11-Jun-14	20	2	13	2	1	1	0	11	1	3	1
17531	101	1	13-Feb-14	18	2	13	2	1	1	0	11	1	2	1
17532	101	1	9-Dec-14	20	2	13	2	1	1	0	11	1	2	1
17533	201	1	27-Jul-13	0	2	13	2	3	1	0	11	1	3	1
17534	101	1	15-Mar-13	12	2	13	2	1	1	0	11	1	2	1
17535	101	1	15-Dec-13	7	2	13	2	1	1	0	11	1	2	1
17536	101	1	12-Oct-13	9	2	13	2	1	1	0	11	1	2	1
17537	101	1	1-Jun-13	14	2	13	2	2	1	0	11	1	3	1
17538	101	1	21-Sep-13	14	2	13	2	1	1	0	11	1	3	1
17539	201	1	11-Aug-13	11	2	13	2	3	1	0	11	1	3	1
17540	201	1	3-Aug-13	11	2	13	2	3	1	0	11	1	2	1
17541	101	1	18-Feb-12	19	2	13	2	1	1	0	11	1	2	1
17542	101	1	11-Nov-12	10	2	13	2	1	1	0	11	1	2	1
17543	101	1	24-Dec-12	16	2	13	2	1	1	0	11	1	2	1
17544	101	1	18-Dec-12	18	2	13	2	1	1	0	11	1	2	1
17545	201	1	9-Dec-12	18	2	13	2	2	1	0	11	1	3	1
17546	101	1	30-Dec-12	13	2	13	2	1	1	0	11	1	3	1
17547	101	1	30-Dec-12	20	2	13	2	1	1	0	11	1	2	1
17548	101	1	17-Feb-12	20	2	13	2	1	1	0	11	1	2	1
17549	201	1	8-Feb-11	10	2	13	2	3	1	0	11	1	1	1
17550	101	1	2-Aug-11	16	2	13	2	1	1	0	11	1	2	1
17551	101	1	22-Feb-11	17	2	13	2	1	1	0	11	1	3	1
17552	101	1	31-Mar-11	18	2	13	2	1	1	0	11	1	2	1
17553	201	1	21-Jan-11	7	2	13	2	3	1	0	11	1	2	1
17554	201	1	20-Nov-10	23	2	13	2	1	1	0	11	1	2	1
17555	101	1	4-May-10	12	2	13	2	1	1	0	11	1	2	1
17556	101	1	19-Dec-10	10	2	13	2	1	1	0	11	1	3	1
17557	201	1	12-Feb-10	19	2	13	2	1	1	0	11	1	3	1
17558	101	1	7-Aug-10	23	2	13	2	1	1	0	11	1	2	1
17559	201	1	9-Mar-10	13	2	13	2	1	1	0	11	1	3	1
17560	101	1	14-Oct-09	12	2	13	2	1	1	0	11	1	2	1
17561	101	1	20-Jul-09	20	2	13	2	1	1	0	11	1	2	1
17562	101	1	5-Aug-09	8	2	13	2	1	1	0	11	1	2	1
17563	201	1	17-Jul-09	9	2	13	2	2	1	0	11	1	3	1
17564	101	1	2-Aug-09	19	2	13	2	1	1	0	11	1	2	1
17565	101	1	16-Jun-09	13	2	13	2	1	1	0	11	1	3	1
17566	101	1	8-Nov-09	10	2	13	2	3	1	0	11	1	3	1
17567	201	1	13-Feb-08	17	2	13	2	3	1	0	11	1	2	1
17568	101	1	1-Mar-08	22	2	13	2	2	1	0	11	1	1	1
17569	101	1	4-May-08	18	2	13	2	1	1	0	11	1	2	1
17570	101	1	15-Jul-07	0	2	13	2	3	1	0	11	1	3	1
17571	201	1	22-May-07	20	2	13	2	3	1	0	11	1	1	1
17572	101	1	10-Feb-06	18	2	13	2	1	1	0	11	1	2	1
17573	101	1	10-Jun-17	13	2	13	2	1	1	0	11	1	3	1
17574	101	1	8-Aug-17	21	2	13	2	1	1	0	11	1	3	1
17575	201	1	21-Jan-17	16	2	13	2	1	1	0	11	1	3	1
17576	101	1	8-Sep-17	18	2	13	2	1	1	0	11	1	2	1
17577	101	1	6-Jun-17	20	2	13	2	1	1	0	11	1	2	1
17578	101	1	25-Feb-17	12	2	13	2	1	1	0	11	1	2	1
17579	101	1	9-Jul-17	17	2	13	2	1	1	0	11	1	2	1
17580	201	1	7-Jan-17	15	2	13	2	1	1	0	11	1	2	1
17581	201	1	4-Feb-17	9	2	13	2	1	1	0	11	1	1	1
17582	201	1	5-Jul-17	14	2	13	2	2	1	0	11	1	3	1
17583	201	1	25-Jan-17	8	2	13	2	2	1	0	11	1	2	1
17584	201	1	10-Nov-17	8	2	13	2	3	1	0	11	1	2	1
17585	101	1	10-Nov-17	11	2	13	2	1	1	0	11	1	3	1
17586	101	1	5-May-16	12	2	13	2	1	1	0	3	1	3	1
17587	101	1	10-Jan-15	18	2	13	2	1	1	0	3	1	1	1
17588	201	1	28-Feb-14	14	2	13	2	2	1	0	3	1	3	1
17589	101	1	29-May-13	23	2	13	2	1	1	0	3	1	1	1
17590	201	1	2-May-13	22	2	13	2	1	1	0	3	1	1	1
17591	101	1	11-Jun-13	10	2	13	2	1	1	0	3	1	3	1
17592	101	1	5-May-12	11	2	13	2	3	1	0	3	1	4	2
17593	101	1	16-Feb-12	19	2	13	2	1	1	0	3	1	4	1
17594	201	1	12-Apr-12	20	2	13	2	2	1	0	3	1	1	1
17595	101	1	4-Dec-11	10	2	13	2	1	1	0	3	1	1	1
17596	101	1	26-Jan-11	16	2	13	2	4	1	0	3	1	1	1
17597	201	1	19-Oct-10	21	2	13	2	1	1	0	3	1	3	1
17598	201	1	25-Mar-09	10	2	13	2	3	1	0	3	1	1	1
17599	201	1	19-Aug-09	2	2	13	2	2	1	0	3	1	2	1
17600	201	1	14-May-09	21	2	13	2	2	1	0	3	1	2	1
17601	201	1	3-Nov-08	17	2	13	2	3	1	0	3	1	3	1

17602	201	1	23-May-07	22	2	13	2	1	1	0	3	1	3	1
17603	101	1	10-Mar-17	18	2	13	2	1	1	0	3	1	1	1
17604	101	1	22-Sep-15	8	2	13	2	1	1	0	6	1	2	1
17605	101	1	25-Aug-14	9	2	13	2	1	1	0	6	1	2	1
17606	101	1	26-Sep-13	20	2	13	2	1	1	0	6	1	2	1
17607	101	1	12-Jan-12	11	2	13	2	1	1	0	6	1	3	1
17608	101	1	2-Oct-11	20	2	13	2	1	1	0	6	1	2	1
17609	201	1	21-Sep-09	22	2	13	2	2	1	0	6	1	2	1
17610	101	1	28-Nov-08	22	2	13	2	1	1	0	6	1	1	2
17611	101	1	13-Jun-08	21	2	13	2	1	1	0	6	1	2	1
17612	101	1	6-Aug-08	15	2	13	2	1	1	0	6	1	2	1
17613	101	1	29-Jan-07	9	2	13	2	1	1	0	6	1	2	1
17614	201	1	4-Nov-07	0	2	13	2	2	1	0	6	1	2	1
17615	201	1	23-Oct-07	17	2	13	2	1	1	0	6	1	2	1
17616	101	1	22-May-17	21	2	13	2	1	1	0	6	1	2	1
17617	101	1	26-Nov-12	12	2	13	2	1	1	0	13	1	1	1
17618	202	0	19-Oct-14	23	1	14	2	1	1	0	6	1	3	1
17619	202	0	5-Apr-12	21	1	14	2	1	1	0	6	1	2	1
17620	202	0	27-Jan-16	21	1	14	2	1	1	0	10	1	6	2
17621	202	0	28-Nov-16	0	1	14	2	1	1	0	10	1	6	2
17622	202	0	29-Jan-16	1	1	14	2	1	1	0	10	1	6	1
17623	202	0	21-Dec-17	21	1	14	2	1	1	0	10	1	6	1
17624	202	0	6-Aug-17	12	1	14	2	1	1	0	10	1	7	2
17625	202	0	22-Sep-12	23	1	14	2	1	1	0	6	1	4	1
17626	202	0	7-Apr-14	10	1	14	2	3	1	0	6	1	2	1
17627	202	0	26-Nov-14	22	1	14	2	3	1	0	6	1	2	1
17628	202	0	2-Jun-16	20	1	14	2	1	1	0	6	1	2	2
17629	202	0	18-Sep-12	12	1	14	2	3	1	0	6	1	3	2
17630	202	0	20-Aug-13	12	1	14	2	2	1	0	10	1	7	2
17631	202	0	20-Feb-11	22	1	14	2	1	1	0	10	1	7	2
17632	202	0	9-Feb-08	21	1	14	2	1	1	0	10	1	7	2
17633	202	0	9-Jul-07	14	1	14	2	1	1	0	13	1	5	1
17634	202	0	7-Mar-14	12	1	14	2	1	1	0	4	1	2	1
17635	202	0	18-Jun-13	20	1	14	2	3	1	0	4	1	1	1
17636	202	0	13-Jan-12	20	1	14	2	1	1	0	4	1	1	2
17637	202	0	18-Sep-10	20	1	14	2	1	1	0	4	1	7	2
17638	202	0	23-Mar-10	18	1	14	2	1	1	0	4	1	1	2
17639	202	0	31-Oct-17	19	1	14	2	1	1	0	4	1	2	1
17640	202	0	7-Sep-16	16	1	14	2	1	1	0	11	1	3	1
17641	202	0	6-Aug-16	9	1	14	2	1	1	0	11	1	2	1
17642	202	0	19-Jun-16	9	1	14	2	1	1	0	11	1	2	1
17643	202	0	13-Oct-16	20	1	14	2	1	1	0	11	1	2	1
17644	202	0	19-Jun-16	12	1	14	2	3	1	0	11	1	2	1
17645	202	0	20-Feb-16	22	1	14	2	3	1	0	11	1	2	1
17646	202	0	30-Dec-16	22	1	14	2	1	1	0	11	1	2	1
17647	202	0	30-May-15	2	1	14	2	1	1	0	11	1	3	1
17648	202	0	25-Jan-13	16	1	14	2	1	1	0	11	1	2	1
17649	202	0	26-Jan-13	13	1	14	2	1	1	0	11	1	3	1
17650	202	0	19-Sep-13	10	1	14	2	1	1	0	11	1	3	1
17651	202	0	25-Jul-13	2	1	14	2	1	1	0	11	1	3	1
17652	202	0	29-Aug-12	8	1	14	2	1	1	0	11	1	3	1
17653	202	0	7-Aug-12	19	1	14	2	1	1	0	11	1	2	2
17654	202	0	31-Jan-10	20	1	14	2	1	1	0	11	1	3	2
17655	202	0	9-Aug-07	15	1	14	2	1	1	0	11	1	1	1
17656	202	0	24-Aug-17	11	1	14	2	2	1	0	11	1	1	2
17657	202	0	29-Jan-16	13	1	14	2	1	1	0	3	1	1	1
17658	202	0	27-Jul-15	12	1	14	2	1	1	0	3	1	1	1
17659	202	0	9-Jan-14	10	1	14	2	2	1	0	3	1	1	1
17660	202	0	19-Jan-14	16	1	14	2	1	1	0	3	1	2	1
17661	202	0	31-May-14	16	1	14	2	3	1	0	3	1	2	2
17662	202	0	6-Apr-13	22	1	14	2	1	1	0	3	1	1	1
17663	202	0	14-May-13	20	1	14	2	1	1	0	3	1	2	2
17664	202	0	29-Oct-13	7	1	14	2	1	1	0	3	1	2	2
17665	202	0	22-Nov-13	20	1	14	2	1	1	0	3	1	2	1
17666	202	0	15-Jun-12	20	1	14	2	1	1	0	3	1	6	1
17667	202	0	23-Apr-11	12	1	14	2	1	1	0	3	1	2	2
17668	202	0	18-Feb-11	11	1	14	2	1	1	0	3	1	1	1
17669	202	0	14-Nov-11	18	1	14	2	1	1	0	3	1	2	2
17670	202	0	24-May-11	21	1	14	2	1	1	0	3	1	3	2
17671	202	0	28-Oct-10	1	1	14	2	1	1	0	3	1	5	1
17672	202	0	5-May-10	17	1	14	2	1	1	0	3	1	1	1
17673	202	0	11-Apr-10	20	1	14	2	1	1	0	3	1	1	2
17674	202	0	29-May-08	16	1	14	2	1	1	0	3	1	1	1
17675	202	0	16-Dec-08	22	1	14	2	2	1	0	3	1	3	2
17676	202	0	28-Apr-08	7	1	14	2	1	1	0	3	1	2	1
17677	202	0	13-Jul-08	19	1	14	2	1	1	0	3	1	1	1
17678	202	0	6-Nov-07	7	1	14	2	1	1	0	3	1	1	2
17679	202	0	30-Jun-07	18	1	14	2	1	1	0	3	1	2	2
17680	202	0	21-Apr-17	17	1	14	2	3	1	0	3	1	1	1
17681	202	0	8-Jan-13	15	1	14	2	1	1	0	6	1	2	1
17682	202	0	8-Mar-12	20	1	14	2	1	1	0	6	1	2	1
17683	202	0	23-Apr-11	12	1	14	2	1	1	0	6	1	3	1
17684	202	0	24-Jun-09	19	1	14	2	1	1	0	6	1	2	1
17685	202	0	14-Jan-08	19	1	14	2	1	1	0	6	1	2	1
17686	202	0	4-Dec-08	18	1	14	2	1	1	0	6	1	2	1
17687	202	0	9-Aug-08	6	1	14	2	1	1	0	6	1	3	1
17688	202	0	2-Mar-08	22	1	14	2	1	1	0	6	1	3	1
17689	202	0	30-Oct-07	9	1	14	2	1	1	0	6	1	2	1
17690	202	0	25-Jan-06	12	1	14	2	1	1	0	6	1	2	1
17691	202	0	12-Nov-06	14	1	14	2	1	1	0	6	1	3	1
17692	202	0	20-Jul-17	0	1	14	2	3	1	0	6	1	2	1
17693	202	0	17-Feb-07	12	1	14	2	1	1	0	13	1	7	1
17694	202	0	10-May-12	12	2	14	2	2	1	0	6	1	2	1
17695	202	0	22-Jan-17	16	2	14	2	1	1	0	4	1	7	1
17696	202	0	22-Dec-13	21	2	14	2	1	1	0	10	1	7	2
17697	202	0	20-Dec-13	21	2	14	2	1	1	0	10	1	7	2
17698	202	0	19-Jan-16	19	2	14	2	1	1	0	10	1	6	1
17699	202	0	28-Dec-16	19	2	14	2	3	1	0	10	1	6	2
17700	202	0	18-Mar-16	22	2	14	2	1	1	0	10	1	6	1
17701	202	0	6-Dec-15	20	2	14	2	3	1	0	10	1	6	1
17702	202	0	15-Jan-17	23	2	14	2	2	1	0	10	1	6	1
17703	202	0	21-Mar-11	6	2	14	2	1	1	0	10	1	7	2
17704	202	0	3-Jan-11	17	2	14	2	1	1	0	10	1	7	1
17705	202	0	26-Jan-10	21	2	14	2	1	1	0	10	1	7	2
17706	202	0	12-Nov-08	16	2	14	2	1	1	0	10	1	7	2
17707	202	0	27-Feb-07	12	2	14	2	1	1	0	10	1	7	1
17708	202	0	20-Mar-17	17	2	14	2	3	1	0	10	1	7	2
17709	202	0	27-Jun-16	3	2	14	2	1	1	0	7	1	1	1
17710	202	0	7-Oct-17	8	2	14	2	1	1	0	7	1	3	1
17711	202	0	28-Jan-17	8	2	14	2	1	1	0	13	1	1	1
17712	202	0	9-Mar-14	14	2	14	2	3	1	0	4	1	1	1
17713	202	0	18-Jan-10	12	2	14	2	1	1	0	4	1	1	2
17714	202	0	3-Jun-10	10	2	14	2	1	1	0	4	1	1	1
17715	202	0	13-Feb-08	21	2	14	2	1	1	0	4	1	1	1
17716	202	0	6-Feb-08	14	2	14	2	1	1	0	4	1	1	1
17717	202	0	8-Nov-08	18	2	14	2	1	1	0	4	1	2	2
17718	202	0	9-Aug-07	11	2	14	2	1	1	0	4	1	2	1
17719	202	0	9-Jul-06	18	2	14	2	1	1	0	4	1	1	1
17720	202	0	22-Oct-16	9	2	14	2	3	1	0	11	1	2	1
17721	202	0	26-Nov-16	13	2	14	2	1	1	0	11	1	2	1
17722	202	0	25-Apr-14	13	2	14	2	1	1	0	11	1	2	1
17723	202	0	2-Sep-13	9	2	14	2	3	1	0	11	1	3	1
17724	202	0	10-Dec-13	13	2	14	2	1	1	0	11	1	2	1
17725	202	0	9-Dec-11	20	2	14	2	1	1	0	11	1	2	2

17726	202	0	28-Nov-06	7	2	14	2	1	1	0	11	1	3	1
17727	202	0	28-Sep-06	17	2	14	2	1	1	0	11	1	3	1
17728	202	0	25-Sep-16	9	2	14	2	2	1	0	3	1	1	1
17729	202	0	30-Jun-16	10	2	14	2	2	1	0	3	1	1	1
17730	202	0	14-Nov-16	4	2	14	2	1	1	0	3	1	3	1
17731	202	0	1-Aug-13	20	2	14	2	3	1	0	3	1	1	1
17732	202	0	11-Feb-12	4	2	14	2	1	1	0	3	1	2	2
17733	202	0	30-May-09	18	2	14	2	1	1	0	3	1	2	2
17734	202	0	20-Dec-07	7	2	14	2	1	1	0	3	1	2	1
17735	202	0	11-Jun-07	3	2	14	2	1	1	0	3	1	4	2
17736	202	0	14-Jun-17	12	2	14	2	1	1	0	3	1	2	1
17737	202	0	9-May-09	14	2	14	2	1	1	0	6	1	2	1
17738	101	1	17-Jun-12	19	1	14	2	1	1	0	6	1	2	1
17739	201	1	6-Oct-14	19	1	14	2	2	1	0	6	1	2	1
17740	201	1	30-Mar-15	20	1	14	2	1	1	0	6	1	2	1
17741	201	1	21-Oct-15	15	1	14	2	2	1	0	6	1	2	1
17742	101	1	14-Sep-17	9	1	14	2	3	1	0	6	1	2	1
17743	201	1	21-Apr-16	20	1	14	2	1	1	0	4	1	3	1
17744	201	1	24-Aug-14	18	1	14	2	2	1	0	4	1	3	1
17745	101	1	11-Aug-17	1	1	14	2	1	1	0	11	1	3	1
17746	101	1	27-Jan-13	22	1	14	2	1	1	0	6	1	2	1
17747	201	1	8-Oct-13	23	1	14	2	2	1	0	6	1	3	2
17748	201	1	7-Jul-17	18	1	14	2	2	1	0	6	1	2	1
17749	201	1	4-May-16	20	1	14	2	1	1	0	6	1	2	1
17750	201	1	28-Oct-16	17	1	14	2	1	1	0	6	1	2	1
17751	201	1	30-Apr-13	8	1	14	2	3	1	0	6	1	1	1
17752	201	1	9-Feb-15	16	1	14	2	3	1	0	4	1	2	1
17753	101	1	17-Jan-15	14	1	14	2	1	1	0	7	1	3	1
17754	101	1	15-Feb-14	19	1	14	2	1	1	0	7	1	4	1
17755	101	1	17-Jan-14	21	1	14	2	1	1	0	7	1	3	1
17756	101	1	28-Mar-14	14	1	14	2	1	1	0	7	1	3	1
17757	101	1	5-Sep-14	11	1	14	2	1	1	0	7	1	2	1
17758	101	1	3-Jan-14	6	1	14	2	1	1	0	7	1	3	2
17759	101	1	2-Oct-14	20	1	14	2	1	1	0	7	1	3	1
17760	201	1	9-Jun-13	18	1	14	2	3	1	0	7	1	1	1
17761	101	1	20-Jan-13	21	1	14	2	1	1	0	7	1	4	1
17762	201	1	19-Sep-13	15	1	14	2	1	1	0	7	1	3	1
17763	101	1	13-Feb-13	21	1	14	2	2	1	0	7	1	2	1
17764	101	1	29-Aug-11	14	1	14	2	1	1	0	7	1	3	1
17765	101	1	20-May-09	7	1	14	2	1	1	0	7	1	3	1
17766	101	1	23-Dec-08	22	1	14	2	1	1	0	7	1	4	1
17767	101	1	9-Apr-08	18	1	14	2	1	1	0	7	1	3	1
17768	201	1	29-Aug-07	16	1	14	2	1	1	0	7	1	3	1
17769	201	1	3-Aug-07	7	1	14	2	1	1	0	7	1	2	1
17770	101	1	7-May-17	12	1	14	2	1	1	0	7	1	3	1
17771	201	1	29-Jun-07	18	1	14	2	2	1	0	13	1	3	1
17772	101	1	18-Jul-06	17	1	14	2	1	1	0	13	1	2	1
17773	201	1	28-Jun-09	15	1	14	2	2	1	0	1	1	5	1
17774	101	1	15-Jul-08	18	1	14	2	1	1	0	1	1	2	1
17775	101	1	23-Aug-09	11	1	14	2	1	1	0	1	1	3	1
17776	201	1	4-Nov-15	17	1	14	2	1	1	0	4	1	2	1
17777	101	1	18-Nov-14	21	1	14	2	1	1	0	4	1	4	1
17778	101	1	3-Jul-13	21	1	14	2	1	1	0	4	1	1	1
17779	201	1	27-Jul-13	19	1	14	2	2	1	0	4	1	2	1
17780	101	1	10-Sep-12	22	1	14	2	1	1	0	4	1	1	1
17781	201	1	2-Sep-11	11	1	14	2	3	1	0	4	1	3	2
17782	201	1	4-Nov-11	10	1	14	2	3	1	0	4	1	2	1
17783	201	1	19-Jun-11	15	1	14	2	1	1	0	4	1	3	1
17784	101	1	7-Jun-11	5	1	14	2	1	1	0	4	1	2	4
17785	101	1	4-Feb-10	13	1	14	2	1	1	0	4	1	1	1
17786	101	1	5-Jul-10	17	1	14	2	1	1	0	4	1	2	1
17787	101	1	7-Jul-10	15	1	14	2	1	1	0	4	1	2	1
17788	201	1	19-May-09	8	1	14	2	2	1	0	4	1	2	1
17789	201	1	21-Jul-09	18	1	14	2	1	1	0	4	1	2	1
17790	201	1	27-Apr-09	14	1	14	2	2	1	0	4	1	2	1
17791	101	1	11-Nov-09	5	1	14	2	1	1	0	4	1	2	1
17792	101	1	20-Mar-09	22	1	14	2	1	1	0	4	1	3	1
17793	201	1	10-Nov-08	20	1	14	2	2	1	0	4	1	1	1
17794	101	1	1-Jun-08	17	1	14	2	1	1	0	4	1	3	1
17795	101	1	5-Jun-07	13	1	14	2	1	1	0	4	1	2	1
17796	201	1	5-Jan-16	19	1	14	2	2	1	0	11	1	2	1
17797	201	1	2-Feb-16	22	1	14	2	3	1	0	11	1	2	1
17798	201	1	14-Feb-16	13	1	14	2	1	1	0	11	1	2	1
17799	201	1	20-May-16	18	1	14	2	3	1	0	11	1	2	1
17800	101	1	20-Jun-16	12	1	14	2	1	1	0	11	1	3	1
17801	101	1	27-Jun-16	7	1	14	2	1	1	0	11	1	2	1
17802	101	1	23-Jun-16	17	1	14	2	1	1	0	11	1	3	1
17803	101	1	6-Apr-15	12	1	14	2	1	1	0	11	1	2	1
17804	101	1	1-Aug-15	18	1	14	2	1	1	0	11	1	2	1
17805	101	1	4-Jan-15	19	1	14	2	1	1	0	11	1	2	1
17806	101	1	29-May-15	14	1	14	2	1	1	0	11	1	3	1
17807	101	1	25-Jan-15	8	1	14	2	3	1	0	11	1	2	1
17808	201	1	1-Apr-15	20	1	14	2	2	1	0	11	1	2	1
17809	101	1	14-Jul-15	21	1	14	2	1	1	0	11	1	2	1
17810	201	1	19-Dec-14	1	1	14	2	1	1	0	11	1	3	1
17811	201	1	7-Oct-14	19	1	14	2	2	1	0	11	1	3	1
17812	101	1	23-Dec-14	9	1	14	2	1	1	0	11	1	2	1
17813	201	1	1-Jan-14	10	1	14	2	2	1	0	11	1	2	1
17814	101	1	8-Mar-14	14	1	14	2	1	1	0	11	1	2	1
17815	101	1	27-Apr-14	20	1	14	2	1	1	0	11	1	2	1
17816	101	1	28-Jun-14	20	1	14	2	1	1	0	11	1	2	1
17817	101	1	13-Feb-14	19	1	14	2	1	1	0	11	1	3	1
17818	201	1	27-Jan-14	20	1	14	2	3	1	0	11	1	3	1
17819	101	1	29-Mar-14	10	1	14	2	1	1	0	11	1	3	1
17820	101	1	9-Dec-14	19	1	14	2	1	1	0	11	1	2	1
17821	201	1	21-Aug-13	13	1	14	2	1	1	0	11	1	3	1
17822	101	1	21-Jul-13	18	1	14	2	1	1	0	11	1	3	1
17823	101	1	31-Oct-13	18	1	14	2	1	1	0	11	1	2	1
17824	201	1	2-Dec-13	8	1	14	2	1	1	0	11	1	2	1
17825	101	1	20-Jul-13	21	1	14	2	1	1	0	11	1	2	1
17826	101	1	31-Mar-13	7	1	14	2	2	1	0	11	1	2	1
17827	101	1	7-Aug-13	20	1	14	2	1	1	0	11	1	3	1
17828	201	1	19-Jul-13	14	1	14	2	3	1	0	11	1	2	1
17829	101	1	3-Jun-13	13	1	14	2	2	1	0	11	1	2	1
17830	101	1	22-Jun-13	11	1	14	2	1	1	0	11	1	2	1
17831	101	1	26-Jul-13	8	1	14	2	2	1	0	11	1	3	1
17832	201	1	15-Jan-13	14	1	14	2	2	1	0	11	1	3	1
17833	101	1	8-Apr-13	21	1	14	2	3	1	0	11	1	3	1
17834	201	1	15-Apr-12	11	1	14	2	3	1	0	11	1	5	1
17835	101	1	15-Dec-12	8	1	14	2	1	1	0	11	1	2	1
17836	101	1	21-Oct-12	20	1	14	2	1	1	0	11	1	2	1
17837	201	1	27-Aug-12	15	1	14	2	3	1	0	11	1	3	1
17838	201	1	22-Aug-12	21	1	14	2	3	1	0	11	1	3	1
17839	101	1	19-Dec-12	10	1	14	2	1	1	0	11	1	2	1
17840	101	1	10-Jul-12	17	1	14	2	1	1	0	11	1	2	1
17841	101	1	25-Oct-12	10	1	14	2	2	1	0	11	1	3	1
17842	201	1	19-Jul-11	20	1	14	2	2	1	0	11	1	2	1
17843	201	1	2-Sep-11	11	1	14	2	1	1	0	11	1	2	1
17844	201	1	25-Sep-11	11	1	14	2	3	1	0	11	1	3	1
17845	201	1	10-Aug-11	8	1	14	2	3	1	0	11	1	2	1
17846	101	1	3-Feb-11	8	1	14	2	1	1	0	11	1	2	1
17847	101	1	25-Jan-11	10	1	14	2	1	1	0	11	1	2	1
17848	101	1	26-Jun-11	19	1	14	2	1	1	0	11	1	2	1
17849	101	1	20-Feb-11	10	1	14	2	1	1	0	11	1	2	1

17850	101	1	8-Dec-11	21	1	14	2	1	1	0	11	1	2	1
17851	201	1	2-Feb-11	9	1	14	2	3	1	0	11	1	2	1
17852	101	1	28-Jul-11	0	1	14	2	1	1	0	11	1	2	1
17853	201	1	28-May-11	0	1	14	2	2	1	0	11	1	4	1
17854	101	1	26-Jan-11	16	1	14	2	1	1	0	11	1	2	1
17855	101	1	19-Jan-11	16	1	14	2	1	1	0	11	1	2	1
17856	101	1	24-Mar-11	18	1	14	2	1	1	0	11	1	2	1
17857	101	1	1-Jan-11	10	1	14	2	1	1	0	11	1	2	1
17858	201	1	8-Mar-11	15	1	14	2	1	1	0	11	1	2	1
17859	101	1	11-Jan-11	18	1	14	2	1	1	0	11	1	2	1
17860	201	1	21-Feb-10	16	1	14	2	1	1	0	11	1	2	1
17861	201	1	24-Apr-10	12	1	14	2	1	1	0	11	1	2	1
17862	101	1	11-Aug-10	13	1	14	2	2	1	0	11	1	3	1
17863	101	1	20-Jul-10	16	1	14	2	1	1	0	11	1	2	1
17864	101	1	3-Feb-10	19	1	14	2	1	1	0	11	1	2	1
17865	201	1	25-Nov-10	8	1	14	2	1	1	0	11	1	2	1
17866	201	1	23-Aug-10	10	1	14	2	2	1	0	11	1	3	1
17867	201	1	12-Apr-10	18	1	14	2	1	1	0	11	1	3	1
17868	101	1	27-Dec-10	16	1	14	2	1	1	0	11	1	2	1
17869	101	1	22-Dec-10	20	1	14	2	3	1	0	11	1	3	1
17870	201	1	3-Dec-09	19	1	14	2	1	1	0	11	1	2	1
17871	201	1	25-Aug-09	12	1	14	2	1	1	0	11	1	3	1
17872	201	1	24-Jul-09	18	1	14	2	1	1	0	11	1	2	1
17873	101	1	23-May-09	7	1	14	2	1	1	0	11	1	2	1
17874	201	1	2-May-09	0	1	14	2	1	1	0	11	1	2	1
17875	101	1	10-May-09	17	1	14	2	1	1	0	11	1	2	1
17876	101	1	23-Jul-09	22	1	14	2	1	1	0	11	1	3	1
17877	101	1	28-Mar-09	23	1	14	2	1	1	0	11	1	2	1
17878	101	1	27-Aug-09	8	1	14	2	1	1	0	11	1	2	1
17879	101	1	4-Oct-09	20	1	14	2	1	1	0	11	1	2	1
17880	201	1	2-Nov-08	15	1	14	2	3	1	0	11	1	3	1
17881	201	1	30-Jul-08	19	1	14	2	3	1	0	11	1	2	1
17882	201	1	9-Feb-08	21	1	14	2	2	1	0	11	1	2	1
17883	101	1	30-Sep-08	20	1	14	2	1	1	0	11	1	2	1
17884	201	1	20-Aug-08	19	1	14	2	2	1	0	11	1	3	1
17885	101	1	24-Aug-08	20	1	14	2	1	1	0	11	1	3	1
17886	201	1	4-Jul-07	18	1	14	2	1	1	0	11	1	3	1
17887	101	1	2-Dec-07	8	1	14	2	1	1	0	11	1	2	1
17888	101	1	31-Mar-06	23	1	14	2	1	1	0	11	1	2	1
17889	101	1	8-Jan-17	10	1	14	2	1	1	0	11	1	2	1
17890	101	1	18-Jan-17	22	1	14	2	1	1	0	11	1	4	1
17891	201	1	17-Sep-17	9	1	14	2	1	1	0	11	1	3	1
17892	201	1	4-Jun-17	18	1	14	2	1	1	0	11	1	3	1
17893	101	1	26-Jan-17	21	1	14	2	1	1	0	11	1	2	1
17894	101	1	24-Aug-17	19	1	14	2	1	1	0	11	1	2	1
17895	101	1	14-Aug-17	12	1	14	2	1	1	0	11	1	2	1
17896	101	1	24-Mar-17	19	1	14	2	1	1	0	11	1	2	1
17897	101	1	19-Jul-17	21	1	14	2	1	1	0	11	1	2	1
17898	201	1	21-Dec-17	11	1	14	2	1	1	0	11	1	2	1
17899	101	1	15-Sep-17	20	1	14	2	2	1	0	11	1	3	1
17900	101	1	12-Sep-17	13	1	14	2	2	1	0	11	1	2	1
17901	101	1	10-Jan-16	15	1	14	2	1	1	0	3	1	5	1
17902	101	1	21-Mar-16	4	1	14	2	1	1	0	3	1	1	1
17903	101	1	18-Mar-16	21	1	14	2	1	1	0	3	1	1	1
17904	201	1	3-Jun-16	18	1	14	2	1	1	0	3	1	3	1
17905	101	1	10-Apr-15	19	1	14	2	1	1	0	3	1	4	2
17906	201	1	3-Sep-15	21	1	14	2	1	1	0	3	1	3	1
17907	101	1	5-Jan-15	18	1	14	2	1	1	0	3	1	2	1
17908	201	1	31-Aug-15	20	1	14	2	2	1	0	3	1	5	1
17909	201	1	6-Aug-15	18	1	14	2	1	1	0	3	1	2	1
17910	201	1	24-Nov-14	2	1	14	2	1	1	0	3	1	2	1
17911	201	1	23-Apr-14	6	1	14	2	2	1	0	3	1	5	1
17912	101	1	14-May-14	0	1	14	2	1	1	0	3	1	1	1
17913	201	1	28-Feb-14	11	1	14	2	2	1	0	3	1	3	1
17914	201	1	23-Oct-14	17	1	14	2	2	1	0	3	1	2	1
17915	201	1	3-May-14	15	1	14	2	2	1	0	3	1	2	1
17916	101	1	20-Sep-13	12	1	14	2	1	1	0	3	1	4	1
17917	201	1	9-Jan-13	20	1	14	2	1	1	0	3	1	1	1
17918	101	1	11-Feb-13	11	1	14	2	1	1	0	3	1	1	1
17919	101	1	18-Aug-13	18	1	14	2	1	1	0	3	1	2	4
17920	101	1	29-Jan-13	18	1	14	2	1	1	0	3	1	2	4
17921	101	1	6-Sep-13	7	1	14	2	1	1	0	3	1	3	1
17922	201	1	9-Aug-12	19	1	14	2	1	1	0	3	1	3	1
17923	201	1	25-Nov-12	17	1	14	2	2	1	0	3	1	3	1
17924	201	1	21-Sep-12	20	1	14	2	2	1	0	3	1	3	1
17925	201	1	29-Dec-12	20	1	14	2	1	1	0	3	1	5	1
17926	101	1	1-Jun-12	17	1	14	2	1	1	0	3	1	2	4
17927	201	1	10-Nov-12	20	1	14	2	2	1	0	3	1	2	1
17928	201	1	2-Jun-11	16	1	14	2	2	1	0	3	1	1	1
17929	201	1	26-Feb-11	15	1	14	2	2	1	0	3	1	1	1
17930	201	1	7-Apr-11	16	1	14	2	1	1	0	3	1	1	1
17931	101	1	1-Jan-11	18	1	14	2	1	1	0	3	1	2	4
17932	101	1	6-Feb-11	20	1	14	2	1	1	0	3	1	2	1
17933	101	1	5-Apr-10	15	1	14	2	1	1	0	3	1	1	1
17934	101	1	1-Sep-10	18	1	14	2	1	1	0	3	1	3	1
17935	201	1	18-Apr-09	9	1	14	2	2	1	0	3	1	1	1
17936	101	1	27-Nov-09	22	1	14	2	1	1	0	3	1	1	1
17937	101	1	13-Dec-09	16	1	14	2	1	1	0	3	1	2	1
17938	201	1	13-Apr-08	23	1	14	2	3	1	0	3	1	4	1
17939	101	1	27-Aug-08	16	1	14	2	1	1	0	3	1	1	4
17940	101	1	15-Oct-08	17	1	14	2	1	1	0	3	1	2	2
17941	201	1	14-Aug-08	20	1	14	2	3	1	0	3	1	3	2
17942	201	1	7-Jul-08	3	1	14	2	2	1	0	3	1	2	1
17943	201	1	14-Apr-07	20	1	14	2	2	1	0	3	1	1	1
17944	101	1	17-Mar-07	16	1	14	2	1	1	0	3	1	1	1
17945	101	1	4-Apr-07	19	1	14	2	1	1	0	3	1	4	1
17946	101	1	12-Aug-06	23	1	14	2	1	1	0	3	1	1	1
17947	201	1	24-Mar-06	22	1	14	2	3	1	0	3	1	3	1
17948	101	1	14-Aug-17	13	1	14	2	1	1	0	3	1	3	1
17949	201	1	27-Oct-17	20	1	14	2	1	1	0	3	1	2	1
17950	201	1	18-Feb-17	1	1	14	2	3	1	0	3	1	4	1
17951	101	1	17-Sep-17	13	1	14	2	1	1	0	3	1	3	1
17952	101	1	10-Jan-17	10	1	14	2	1	1	0	3	1	1	1
17953	101	1	31-Mar-16	12	1	14	2	1	1	0	6	1	1	1
17954	101	1	12-Jun-16	7	1	14	2	1	1	0	6	1	2	1
17955	201	1	16-May-16	13	1	14	2	2	1	0	6	1	2	1
17956	101	1	10-Mar-16	16	1	14	2	1	1	0	6	1	2	1
17957	101	1	12-May-15	17	1	14	2	1	1	0	6	1	2	1
17958	101	1	28-Dec-15	17	1	14	2	1	1	0	6	1	2	1
17959	101	1	9-Dec-15	21	1	14	2	1	1	0	6	1	2	1
17960	101	1	20-Jan-15	11	1	14	2	1	1	0	6	1	2	1
17961	101	1	11-Jun-14	20	1	14	2	1	1	0	6	1	2	1
17962	101	1	18-Aug-14	17	1	14	2	1	1	0	6	1	2	1
17963	101	1	28-Mar-14	8	1	14	2	1	1	0	6	1	2	1
17964	101	1	20-Aug-14	23	1	14	2	1	1	0	6	1	2	4
17965	101	1	20-May-14	16	1	14	2	1	1	0	6	1	2	1
17966	201	1	5-Mar-14	12	1	14	2	2	1	0	6	1	2	1
17967	101	1	13-Jun-14	22	1	14	2	1	1	0	6	1	3	1
17968	101	1	12-Sep-14	13	1	14	2	1	1	0	6	1	2	1
17969	101	1	1-Nov-14	8	1	14	2	1	1	0	6	1	2	1
17970	101	1	16-Dec-14	19	1	14	2	1	1	0	6	1	2	1
17971	201	1	1-May-14	11	1	14	2	1	1	0	6	1	2	1
17972	101	1	12-Apr-14	23	1	14	2	1	1	0	6	1	2	1
17973	101	1	29-May-14	21	1	14	2	1	1	0	6	1	2	1

17974	101	1	24-Jun-13	20	1	14	2	1	1	0	6	1	2	1
17975	101	1	28-Apr-13	21	1	14	2	1	1	0	6	1	2	1
17976	101	1	19-Oct-13	13	1	14	2	1	1	0	6	1	3	1
17977	101	1	27-Aug-13	21	1	14	2	1	1	0	6	1	2	1
17978	101	1	18-Mar-13	3	1	14	2	1	1	0	6	1	2	1
17979	101	1	3-Dec-13	13	1	14	2	1	1	0	6	1	2	1
17980	101	1	9-Aug-13	21	1	14	2	2	1	0	6	1	2	2
17981	101	1	8-Sep-13	15	1	14	2	1	1	0	6	1	3	1
17982	101	1	13-Aug-12	12	1	14	2	1	1	0	6	1	3	2
17983	101	1	9-Nov-12	12	1	14	2	1	1	0	6	1	2	1
17984	101	1	11-Oct-12	21	1	14	2	1	1	0	6	1	3	1
17985	101	1	23-Jul-12	23	1	14	2	1	1	0	6	1	3	1
17986	101	1	23-Jan-12	19	1	14	2	1	1	0	6	1	2	1
17987	101	1	1-Dec-12	23	1	14	2	1	1	0	6	1	2	1
17988	101	1	25-Oct-11	21	1	14	2	1	1	0	6	1	2	1
17989	201	1	4-Apr-11	8	1	14	2	1	1	0	6	1	3	1
17990	101	1	18-Sep-10	11	1	14	2	1	1	0	6	1	1	1
17991	101	1	3-Jun-10	20	1	14	2	1	1	0	6	1	2	1
17992	101	1	4-Dec-10	18	1	14	2	1	1	0	6	1	2	1
17993	101	1	19-Jul-10	15	1	14	2	1	1	0	6	1	3	1
17994	201	1	31-May-09	21	1	14	2	2	1	0	6	1	2	1
17995	101	1	3-Nov-09	9	1	14	2	1	1	0	6	1	2	1
17996	201	1	26-Jun-09	13	1	14	2	2	1	0	6	1	2	1
17997	101	1	10-Aug-09	8	1	14	2	1	1	0	6	1	2	1
17998	201	1	3-Mar-08	8	1	14	2	1	1	0	6	1	2	1
17999	101	1	26-Aug-08	14	1	14	2	1	1	0	6	1	2	1
18000	101	1	21-Dec-08	19	1	14	2	1	1	0	6	1	2	2
18001	101	1	26-Jul-08	10	1	14	2	1	1	0	6	1	2	1
18002	101	1	17-Sep-07	23	1	14	2	1	1	0	6	1	3	4
18003	101	1	24-Sep-06	20	1	14	2	1	1	0	6	1	2	1
18004	101	1	16-Aug-06	22	1	14	2	1	1	0	6	1	2	1
18005	101	1	16-May-17	8	1	14	2	1	1	0	6	1	2	1
18006	101	1	27-Sep-17	23	1	14	2	1	1	0	6	1	2	1
18007	101	1	25-Jan-16	11	1	14	2	1	1	0	13	1	4	1
18008	101	1	18-Apr-13	12	1	14	2	1	1	0	13	1	3	1
18009	101	1	28-Jun-08	10	1	14	2	1	1	0	13	1	4	1
18010	201	1	30-Mar-13	12	2	14	2	1	1	0	6	1	2	1
18011	201	1	18-Apr-17	18	2	14	2	2	1	0	6	1	2	1
18012	201	1	15-Apr-13	16	2	14	2	2	1	0	6	1	3	1
18013	201	1	29-Aug-17	11	2	14	2	3	1	0	6	1	2	1
18014	201	1	16-Aug-16	12	2	14	2	1	1	0	7	1	3	1
18015	201	1	24-Jan-16	20	2	14	2	3	1	0	7	1	3	1
18016	201	1	1-Apr-13	9	2	14	2	2	1	0	6	1	2	1
18017	101	1	24-Jun-16	19	2	14	2	1	1	0	6	1	2	1
18018	101	1	12-Aug-16	19	2	14	2	1	1	0	6	1	3	2
18019	101	1	13-May-17	19	2	14	2	1	1	0	6	1	2	1
18020	101	1	23-Feb-15	20	2	14	2	1	1	0	7	1	2	1
18021	101	1	16-Jun-14	23	2	14	2	1	1	0	7	1	3	1
18022	201	1	15-Dec-13	18	2	14	2	2	1	0	7	1	2	1
18023	101	1	1-Jun-12	22	2	14	2	1	1	0	7	1	5	4
18024	201	1	12-Jul-11	8	2	14	2	1	1	0	7	1	1	1
18025	101	1	25-Dec-10	13	2	14	2	1	1	0	7	1	2	4
18026	101	1	16-Jul-09	8	2	14	2	2	1	0	7	1	6	1
18027	101	1	16-Jul-09	7	2	14	2	1	1	0	7	1	5	1
18028	101	1	30-Jan-08	15	2	14	2	1	1	0	7	1	5	2
18029	201	1	23-Dec-06	14	2	14	2	2	1	0	7	1	2	1
18030	101	1	27-Feb-11	1	2	14	2	1	1	0	13	1	1	1
18031	201	1	15-Apr-09	8	2	14	2	1	1	0	13	1	7	1
18032	101	1	4-Sep-06	19	2	14	2	1	1	0	13	1	7	1
18033	101	1	24-Apr-15	17	2	14	2	1	1	0	4	1	2	1
18034	201	1	22-Jan-14	8	2	14	2	2	1	0	4	1	2	1
18035	201	1	2-Aug-07	18	2	14	2	2	1	0	4	1	2	1
18036	101	1	18-Sep-06	18	2	14	2	1	1	0	4	1	2	1
18037	201	1	24-Jun-16	22	2	14	2	2	1	0	11	1	2	1
18038	101	1	4-Jun-16	13	2	14	2	1	1	0	11	1	2	1
18039	101	1	4-Jun-16	13	2	14	2	1	1	0	11	1	3	1
18040	101	1	27-Jun-16	7	2	14	2	1	1	0	11	1	2	1
18041	101	1	12-Mar-16	12	2	14	2	1	1	0	11	1	2	1
18042	201	1	22-Mar-15	17	2	14	2	1	1	0	11	1	3	1
18043	201	1	15-May-15	13	2	14	2	1	1	0	11	1	2	1
18044	101	1	30-May-15	20	2	14	2	1	1	0	11	1	2	1
18045	101	1	28-Mar-15	21	2	14	2	1	1	0	11	1	2	1
18046	101	1	20-Feb-15	20	2	14	2	1	1	0	11	1	2	1
18047	201	1	17-Jul-15	17	2	14	2	2	1	0	11	1	2	1
18048	101	1	7-Jan-15	7	2	14	2	1	1	0	11	1	3	1
18049	101	1	20-May-15	18	2	14	2	1	1	0	11	1	2	1
18050	101	1	27-May-15	8	2	14	2	1	1	0	11	1	2	1
18051	101	1	1-May-15	12	2	14	2	1	1	0	11	1	3	1
18052	101	1	4-Aug-14	21	2	14	2	1	1	0	11	1	1	1
18053	201	1	8-Dec-14	14	2	14	2	1	1	0	11	1	3	1
18054	101	1	15-May-14	20	2	14	2	1	1	0	11	1	2	1
18055	101	1	3-Dec-14	12	2	14	2	1	1	0	11	1	2	1
18056	101	1	18-Jan-14	17	2	14	2	1	1	0	11	1	2	1
18057	201	1	31-May-14	22	2	14	2	2	1	0	11	1	3	1
18058	101	1	27-Oct-14	20	2	14	2	1	1	0	11	1	3	1
18059	201	1	4-Aug-14	21	2	14	2	2	1	0	11	1	3	1
18060	201	1	7-Apr-14	17	2	14	2	1	1	0	11	1	2	1
18061	201	1	21-Mar-14	17	2	14	2	1	1	0	11	1	2	1
18062	101	1	17-Dec-14	12	2	14	2	1	1	0	11	1	2	1
18063	101	1	26-Apr-14	21	2	14	2	1	1	0	11	1	2	1
18064	101	1	14-Jan-14	19	2	14	2	1	1	0	11	1	3	1
18065	101	1	14-Jun-14	16	2	14	2	1	1	0	11	1	3	1
18066	101	1	16-Nov-13	8	2	14	2	1	1	0	11	1	4	1
18067	101	1	12-Jun-13	20	2	14	2	1	1	0	11	1	3	1
18068	101	1	25-Dec-13	19	2	14	2	1	1	0	11	1	2	1
18069	101	1	21-Jun-13	21	2	14	2	1	1	0	11	1	2	1
18070	101	1	20-Aug-13	21	2	14	2	1	1	0	11	1	2	1
18071	201	1	23-Dec-13	21	2	14	2	1	1	0	11	1	2	1
18072	101	1	25-Nov-13	10	2	14	2	1	1	0	11	1	3	1
18073	101	1	25-Oct-13	19	2	14	2	2	1	0	11	1	3	1
18074	101	1	23-Jul-13	20	2	14	2	1	1	0	11	1	2	1
18075	101	1	3-Dec-13	19	2	14	2	1	1	0	11	1	2	1
18076	101	1	27-Aug-13	9	2	14	2	1	1	0	11	1	3	1
18077	101	1	2-Sep-13	7	2	14	2	1	1	0	11	1	3	1
18078	201	1	14-Jul-12	18	2	14	2	3	1	0	11	1	3	1
18079	201	1	20-Aug-12	8	2	14	2	3	1	0	11	1	2	1
18080	201	1	18-Feb-12	11	2	14	2	3	1	0	11	1	2	1
18081	201	1	22-Apr-12	12	2	14	2	2	1	0	11	1	3	1
18082	201	1	30-Jan-12	9	2	14	2	2	1	0	11	1	3	1
18083	101	1	8-Jan-12	19	2	14	2	1	1	0	11	1	1	1
18084	101	1	4-Nov-12	2	2	14	2	1	1	0	11	1	2	1
18085	101	1	3-Dec-12	19	2	14	2	1	1	0	11	1	2	1
18086	101	1	27-Feb-12	21	2	14	2	1	1	0	11	1	2	1
18087	101	1	30-May-12	17	2	14	2	1	1	0	11	1	2	1
18088	101	1	4-Aug-12	23	2	14	2	1	1	0	11	1	3	1
18089	101	1	25-Feb-12	10	2	14	2	1	1	0	11	1	3	1
18090	101	1	31-May-12	14	2	14	2	1	1	0	11	1	2	1
18091	101	1	5-Apr-12	18	2	14	2	1	1	0	11	1	2	1
18092	101	1	26-Aug-12	20	2	14	2	1	1	0	11	1	3	1
18093	101	1	5-Mar-12	17	2	14	2	1	1	0	11	1	3	1
18094	101	1	28-Mar-11	5	2	14	2	1	1	0	11	1	1	1
18095	201	1	19-Apr-11	21	2	14	2	3	1	0	11	1	3	1
18096	101	1	18-Jul-11	13	2	14	2	1	1	0	11	1	2	1
18097	101	1	9-Apr-11	15	2	14	2	1	1	0	11	1	3	1

18098	101	1	27-Sep-11	19	2	14	2	1	1	0	11	1	2	1
18099	101	1	11-Jun-11	18	2	14	2	1	1	0	11	1	5	1
18100	101	1	31-Jan-11	14	2	14	2	1	1	0	11	1	2	1
18101	101	1	27-Feb-11	14	2	14	2	1	1	0	11	1	2	1
18102	101	1	25-Oct-10	20	2	14	2	1	1	0	11	1	1	1
18103	101	1	13-Oct-10	23	2	14	2	2	1	0	11	1	3	1
18104	101	1	21-Oct-10	18	2	14	2	1	1	0	11	1	2	1
18105	101	1	1-May-10	17	2	14	2	1	1	0	11	1	2	1
18106	201	1	8-Jan-10	15	2	14	2	2	1	0	11	1	2	1
18107	101	1	7-Mar-10	10	2	14	2	1	1	0	11	1	2	1
18108	101	1	27-Feb-10	1	2	14	2	1	1	0	11	1	2	1
18109	101	1	28-Jun-10	17	2	14	2	1	1	0	11	1	2	1
18110	101	1	23-Jan-10	22	2	14	2	1	1	0	11	1	2	1
18111	201	1	23-Aug-10	10	2	14	2	3	1	0	11	1	2	1
18112	201	1	18-Oct-09	20	2	14	2	1	1	0	11	1	3	1
18113	101	1	11-Dec-09	22	2	14	2	2	1	0	11	1	2	1
18114	101	1	26-Aug-09	17	2	14	2	2	1	0	11	1	2	1
18115	101	1	30-Jul-09	20	2	14	2	1	1	0	11	1	1	1
18116	101	1	13-Sep-09	20	2	14	2	1	1	0	11	1	3	1
18117	101	1	27-Nov-09	10	2	14	2	1	1	0	11	1	2	1
18118	101	1	28-Jun-09	20	2	14	2	1	1	0	11	1	2	1
18119	101	1	23-Jun-09	15	2	14	2	1	1	0	11	1	2	1
18120	101	1	18-Feb-08	1	2	14	2	1	1	0	11	1	2	1
18121	101	1	21-Apr-08	16	2	14	2	1	1	0	11	1	2	1
18122	101	1	3-Jan-08	19	2	14	2	1	1	0	11	1	3	1
18123	101	1	18-Nov-08	22	2	14	2	1	1	0	11	1	2	1
18124	101	1	6-Apr-07	18	2	14	2	1	1	0	11	1	4	1
18125	201	1	22-Nov-07	19	2	14	2	1	1	0	11	1	2	1
18126	201	1	15-Dec-07	22	2	14	2	2	1	0	11	1	2	1
18127	201	1	29-Oct-17	10	2	14	2	1	1	0	11	1	3	1
18128	201	1	1-May-17	14	2	14	2	1	1	0	11	1	3	1
18129	101	1	18-Apr-17	17	2	14	2	1	1	0	11	1	2	1
18130	101	1	3-Aug-17	21	2	14	2	1	1	0	11	1	2	1
18131	101	1	10-Dec-17	21	2	14	2	1	1	0	11	1	2	1
18132	101	1	11-Jun-17	11	2	14	2	1	1	0	11	1	2	1
18133	101	1	13-Aug-17	13	2	14	2	1	1	0	11	1	2	1
18134	201	1	17-Oct-17	16	2	14	2	1	1	0	11	1	2	1
18135	201	1	25-Jul-17	14	2	14	2	1	1	0	11	1	2	1
18136	201	1	16-Nov-17	21	2	14	2	1	1	0	11	1	2	1
18137	201	1	10-Feb-17	8	2	14	2	2	1	0	11	1	2	1
18138	101	1	19-Sep-17	13	2	14	2	1	1	0	11	1	2	1
18139	101	1	18-Nov-17	10	2	14	2	1	1	0	11	1	2	1
18140	201	1	1-Jan-14	21	2	14	2	1	1	0	3	1	2	1
18141	201	1	23-May-14	6	2	14	2	1	1	0	3	1	3	1
18142	101	1	27-Nov-13	20	2	14	2	1	1	0	3	1	3	2
18143	101	1	6-Jun-12	17	2	14	2	1	1	0	3	1	4	4
18144	101	1	9-Aug-12	11	2	14	2	1	1	0	3	1	1	1
18145	101	1	9-Aug-12	14	2	14	2	1	1	0	3	1	1	1
18146	101	1	15-Apr-12	12	2	14	2	2	1	0	3	1	1	1
18147	201	1	12-Sep-11	13	2	14	2	1	1	0	3	1	1	1
18148	201	1	9-Jul-11	9	2	14	2	2	1	0	3	1	1	1
18149	101	1	11-May-11	18	2	14	2	1	1	0	3	1	1	1
18150	101	1	4-Apr-11	20	2	14	2	1	1	0	3	1	3	1
18151	101	1	20-Oct-11	18	2	14	2	1	1	0	3	1	3	1
18152	201	1	4-Aug-10	23	2	14	2	2	1	0	3	1	1	1
18153	201	1	3-Oct-10	8	2	14	2	2	1	0	3	1	1	1
18154	101	1	29-Apr-10	20	2	14	2	1	1	0	3	1	4	1
18155	201	1	20-Mar-10	21	2	14	2	3	1	0	3	1	3	1
18156	201	1	11-Jun-10	18	2	14	2	1	1	0	3	1	3	1
18157	101	1	20-Feb-09	19	2	14	2	1	1	0	3	1	1	2
18158	101	1	15-May-08	18	2	14	2	1	1	0	3	1	3	1
18159	101	1	6-Oct-07	18	2	14	2	1	1	0	3	1	1	2
18160	201	1	5-Apr-07	17	2	14	2	1	1	0	3	1	3	1
18161	201	1	30-Mar-07	20	2	14	2	1	1	0	3	1	1	1
18162	201	1	23-Feb-07	17	2	14	2	1	1	0	3	1	2	1
18163	101	1	17-Oct-06	19	2	14	2	1	1	0	3	1	1	1
18164	101	1	8-Apr-06	16	2	14	2	1	1	0	3	1	3	1
18165	101	1	5-Sep-17	9	2	14	2	1	1	0	3	1	3	1
18166	101	1	2-Sep-17	12	2	14	2	1	1	0	3	1	3	1
18167	101	1	21-Apr-17	10	2	14	2	1	1	0	3	1	1	1
18168	101	1	14-Mar-16	12	2	14	2	1	1	0	6	1	2	1
18169	101	1	6-Nov-15	12	2	14	2	1	1	0	6	1	2	1
18170	101	1	20-Jul-15	11	2	14	2	1	1	0	6	1	3	1
18171	101	1	15-Feb-14	9	2	14	2	1	1	0	6	1	2	1
18172	101	1	26-Oct-13	9	2	14	2	1	1	0	6	1	2	1
18173	201	1	3-May-13	7	2	14	2	1	1	0	6	1	2	1
18174	101	1	28-Feb-13	19	2	14	2	1	1	0	6	1	1	1
18175	101	1	13-Oct-13	19	2	14	2	1	1	0	6	1	2	2
18176	101	1	24-Jun-11	22	2	14	2	1	1	0	6	1	2	1
18177	101	1	20-Apr-11	17	2	14	2	1	1	0	6	1	2	1
18178	201	1	28-May-10	14	2	14	2	3	1	0	6	1	3	1
18179	201	1	19-Jun-09	22	2	14	2	3	1	0	6	1	2	1
18180	201	1	30-Jul-08	11	2	14	2	2	1	0	6	1	1	1
18181	101	1	19-Oct-08	14	2	14	2	1	1	0	6	1	3	1
18182	101	1	1-Oct-07	11	2	14	2	1	1	0	6	1	2	1
18183	101	1	11-Feb-17	17	2	14	2	1	1	0	6	1	2	1
18184	201	1	19-May-07	12	2	14	2	1	1	0	13	1	3	1
18185	202	0	13-Apr-16	22	1	15	2	1	1	0	6	1	2	1
18186	202	0	26-Jun-17	20	1	15	2	1	1	0	6	1	2	1
18187	202	0	10-Mar-16	18	1	15	2	1	1	0	6	1	1	1
18188	202	0	26-Jan-12	10	1	15	2	1	1	0	10	1	7	2
18189	202	0	26-Jun-15	21	1	15	2	1	1	0	6	1	2	1
18190	202	0	4-Oct-13	18	1	15	2	1	1	0	4	1	3	1
18191	202	0	20-Nov-15	22	1	15	2	1	1	0	6	1	2	1
18192	202	0	19-May-14	13	1	15	2	1	1	0	6	1	2	2
18193	202	0	20-Nov-16	5	1	15	2	1	1	0	10	1	6	2
18194	202	0	30-Aug-12	4	1	15	2	1	1	0	10	1	7	2
18195	202	0	1-Mar-14	16	1	15	2	1	1	0	7	1	3	1
18196	202	0	7-Dec-09	15	1	15	2	1	1	0	7	1	3	1
18197	202	0	21-Dec-08	18	1	15	2	1	1	0	13	1	7	1
18198	202	0	31-Jul-07	19	1	15	2	3	1	0	13	1	2	1
18199	202	0	10-Sep-07	14	1	15	2	1	1	0	1	1	6	1
18200	202	0	3-Jul-16	13	1	15	2	2	1	0	4	1	2	1
18201	202	0	18-May-13	17	1	15	2	2	1	0	4	1	2	1
18202	202	0	6-Jul-11	10	1	15	2	3	1	0	4	1	2	2
18203	202	0	7-Aug-10	11	1	15	2	1	1	0	4	1	2	1
18204	202	0	13-Dec-09	19	1	15	2	1	1	0	4	1	2	1
18205	202	0	27-Aug-17	7	1	15	2	1	1	0	4	1	3	1
18206	202	0	6-Dec-17	20	1	15	2	2	1	0	4	1	1	1
18207	202	0	18-Aug-16	16	1	15	2	1	1	0	11	1	3	1
18208	202	0	11-Aug-16	20	1	15	2	1	1	0	11	1	3	1
18209	202	0	30-Jun-15	1	1	15	2	2	1	0	11	1	2	1
18210	202	0	7-Sep-14	16	1	15	2	3	1	0	11	1	3	1
18211	202	0	29-Jul-14	19	1	15	2	1	1	0	11	1	3	1
18212	202	0	17-Mar-14	21	1	15	2	1	1	0	11	1	3	1
18213	202	0	14-May-14	22	1	15	2	3	1	0	11	1	3	1
18214	202	0	29-Nov-13	12	1	15	2	2	1	0	11	1	3	1
18215	202	0	2-Jan-13	13	1	15	2	3	1	0	11	1	2	1
18216	202	0	2-Jan-11	14	1	15	2	1	1	0	11	1	2	1
18217	202	0	2-Jan-11	15	1	15	2	1	1	0	11	1	3	1
18218	202	0	24-Jan-11	16	1	15	2	1	1	0	11	1	2	1
18219	202	0	9-Aug-11	23	1	15	2	1	1	0	11	1	2	2
18220	202	0	28-Mar-10	10	1	15	2	2	1	0	11	1	3	2
18221	202	0	11-Nov-09	21	1	15	2	1	1	0	11	1	3	1

18222	202	0	27-Dec-09	17	1	15	2	1	1	0	11	1	2	1
18223	202	0	27-Nov-06	4	1	15	2	1	1	0	11	1	3	1
18224	202	0	27-Apr-16	18	1	15	2	1	1	0	3	1	1	1
18225	202	0	28-Jul-15	16	1	15	2	2	1	0	3	1	2	1
18226	202	0	5-Aug-14	4	1	15	2	4	1	0	3	1	1	2
18227	202	0	3-Mar-14	19	1	15	2	1	1	0	3	1	2	2
18228	202	0	16-Sep-14	12	1	15	2	3	1	0	3	1	2	2
18229	202	0	24-Dec-14	20	1	15	2	1	1	0	3	1	2	1
18230	202	0	5-Feb-13	16	1	15	2	2	1	0	3	1	5	2
18231	202	0	15-Apr-13	8	1	15	2	1	1	0	3	1	3	1
18232	202	0	19-Nov-12	3	1	15	2	1	1	0	3	1	1	2
18233	202	0	1-Aug-12	20	1	15	2	1	1	0	3	1	2	1
18234	202	0	21-Nov-12	13	1	15	2	1	1	0	3	1	3	1
18235	202	0	1-Jul-11	19	1	15	2	1	1	0	3	1	2	2
18236	202	0	17-Feb-10	14	1	15	2	1	1	0	3	1	4	1
18237	202	0	7-Oct-10	14	1	15	2	1	1	0	3	1	1	1
18238	202	0	25-Nov-10	17	1	15	2	1	1	0	3	1	3	2
18239	202	0	11-May-10	18	1	15	2	2	1	0	3	1	3	2
18240	202	0	7-Mar-08	18	1	15	2	1	1	0	3	1	1	1
18241	202	0	31-Jan-07	20	1	15	2	2	1	0	3	1	2	1
18242	202	0	6-Dec-06	18	1	15	2	1	1	0	3	1	7	1
18243	202	0	18-Feb-17	14	1	15	2	1	1	0	3	1	1	1
18244	202	0	22-Jul-16	23	1	15	2	1	1	0	6	1	2	1
18245	202	0	25-Jun-15	8	1	15	2	1	1	0	6	1	2	1
18246	202	0	24-Dec-14	20	1	15	2	1	1	0	6	1	2	1
18247	202	0	29-Aug-13	15	1	15	2	1	1	0	6	1	3	1
18248	202	0	24-Aug-11	8	1	15	2	1	1	0	6	1	3	2
18249	202	0	6-Jun-11	19	1	15	2	1	1	0	6	1	2	2
18250	202	0	19-Aug-11	11	1	15	2	3	1	0	6	1	4	2
18251	202	0	3-Mar-11	11	1	15	2	1	1	0	6	1	3	1
18252	202	0	16-Jan-10	23	1	15	2	1	1	0	6	1	2	1
18253	202	0	31-Oct-10	15	1	15	2	1	1	0	6	1	2	1
18254	202	0	12-Sep-10	21	1	15	2	1	1	0	6	1	3	1
18255	202	0	6-Dec-10	13	1	15	2	1	1	0	6	1	2	1
18256	202	0	19-Sep-09	20	1	15	2	1	1	0	6	1	2	2
18257	202	0	4-Jul-07	13	1	15	2	1	1	0	6	1	2	2
18258	202	0	25-Feb-07	17	1	15	2	1	1	0	6	1	1	1
18259	202	0	19-May-07	17	1	15	2	1	1	0	6	1	2	1
18260	202	0	17-Jan-06	22	1	15	2	1	1	0	6	1	1	1
18261	202	0	18-Jun-11	0	1	15	2	3	1	0	13	1	2	1
18262	202	0	13-Apr-12	7	2	15	2	1	1	0	1	1	3	2
18263	202	0	2-Jan-13	2	2	15	2	1	1	0	7	1	3	2
18264	202	0	7-Dec-14	16	2	15	2	1	1	0	10	1	7	2
18265	202	0	2-Feb-12	19	2	15	2	1	1	0	10	1	7	1
18266	202	0	12-Mar-12	20	2	15	2	1	1	0	10	1	7	2
18267	202	0	12-Feb-16	9	2	15	2	2	1	0	10	1	6	2
18268	202	0	10-Aug-13	16	2	15	2	2	1	0	10	1	7	2
18269	202	0	25-May-14	17	2	15	2	1	1	0	6	1	2	1
18270	202	0	25-Feb-13	21	2	15	2	1	1	0	10	1	4	2
18271	202	0	2-Feb-11	20	2	15	2	2	1	0	10	1	7	2
18272	202	0	24-Feb-10	21	2	15	2	1	1	0	10	1	7	2
18273	202	0	31-May-10	2	2	15	2	1	1	0	10	1	7	2
18274	202	0	27-Jan-08	18	2	15	2	1	1	0	10	1	7	2
18275	202	0	22-Jan-07	19	2	15	2	1	1	0	10	1	7	2
18276	202	0	28-Jul-15	19	2	15	2	1	1	0	7	1	3	2
18277	202	0	27-Aug-07	15	2	15	2	1	1	0	7	1	2	1
18278	202	0	25-Jun-16	18	2	15	2	1	1	0	13	1	4	1
18279	202	0	21-Feb-10	12	2	15	2	1	1	0	13	1	7	2
18280	202	0	12-Jul-08	17	2	15	2	1	1	0	13	1	1	1
18281	202	0	31-Aug-13	22	2	15	2	1	1	0	4	1	3	2
18282	202	0	10-Nov-09	12	2	15	2	1	1	0	4	1	1	1
18283	202	0	11-Jan-13	15	2	15	2	1	1	0	11	1	2	1
18284	202	0	5-Sep-12	18	2	15	2	1	1	0	11	1	3	2
18285	202	0	24-Aug-17	17	2	15	2	1	1	0	11	1	3	1
18286	202	0	18-Feb-17	22	2	15	2	1	1	0	11	1	2	1
18287	202	0	19-Aug-17	10	2	15	2	2	1	0	11	1	2	1
18288	202	0	13-Sep-17	0	2	15	2	3	1	0	11	1	5	1
18289	202	0	20-Feb-16	4	2	15	2	1	1	0	3	1	1	1
18290	202	0	14-Sep-13	22	2	15	2	1	1	0	3	1	1	1
18291	202	0	11-Oct-12	7	2	15	2	3	1	0	3	1	3	1
18292	202	0	2-Jan-10	9	2	15	2	1	1	0	3	1	1	1
18293	202	0	27-Mar-10	13	2	15	2	1	1	0	3	1	1	1
18294	202	0	29-Jan-08	14	2	15	2	1	1	0	3	1	1	1
18295	202	0	24-Nov-08	8	2	15	2	1	1	0	3	1	3	1
18296	202	0	28-Oct-07	11	2	15	2	1	1	0	3	1	3	1
18297	202	0	30-Jan-17	10	2	15	2	1	1	0	3	1	4	1
18298	202	0	21-Jan-10	19	2	15	2	1	1	0	6	1	2	1
18299	202	0	26-Nov-07	10	2	15	2	1	1	0	13	1	6	1
18300	202	0	28-Dec-07	0	2	15	2	1	1	0	13	1	6	1
18301	201	1	14-Mar-17	18	1	15	2	1	1	0	4	1	3	1
18302	201	1	16-Apr-12	23	1	15	2	1	1	0	3	1	1	1
18303	201	1	25-Sep-14	19	1	15	2	2	1	0	6	1	3	1
18304	101	1	29-Jan-14	0	1	15	2	1	1	0	6	1	2	1
18305	201	1	9-Mar-17	18	1	15	2	3	1	0	6	1	2	1
18306	201	1	10-Mar-17	1	1	15	2	3	1	0	6	1	4	1
18307	201	1	7-Aug-17	9	1	15	2	3	1	0	4	1	2	1
18308	201	1	7-Nov-14	21	1	15	2	1	1	0	4	1	2	1
18309	201	1	31-May-16	7	1	15	2	1	1	0	7	1	5	1
18310	201	1	24-Jun-12	19	1	15	2	1	1	0	6	1	3	1
18311	201	1	7-Jun-15	9	1	15	2	2	1	0	6	1	2	1
18312	201	1	16-Feb-17	21	1	15	2	1	1	0	6	1	2	1
18313	101	1	25-Jun-12	22	1	15	2	2	1	0	4	1	2	1
18314	201	1	27-Jan-14	20	1	15	2	1	1	0	7	1	3	1
18315	201	1	26-May-16	20	1	15	2	1	1	0	6	1	2	1
18316	201	1	18-Apr-17	18	1	15	2	1	1	0	6	1	2	1
18317	101	1	22-Nov-14	1	1	15	2	1	1	0	6	1	2	1
18318	201	1	19-Nov-16	23	1	15	2	1	1	0	6	1	2	1
18319	201	1	30-Jun-13	3	1	15	2	2	1	0	4	1	2	2
18320	201	1	5-May-16	6	1	15	2	1	1	0	6	1	2	1
18321	201	1	9-Oct-17	1	1	15	2	2	1	0	6	1	2	1
18322	201	1	28-Nov-16	12	1	15	2	2	1	0	6	1	2	1
18323	201	1	23-Mar-14	22	1	15	2	2	1	0	6	1	2	1
18324	201	1	16-Aug-17	20	1	15	2	2	1	0	6	1	2	1
18325	201	1	28-Jan-06	16	1	15	2	2	1	0	10	1	7	2
18326	101	1	26-Mar-16	17	1	15	2	1	1	0	7	1	2	2
18327	201	1	20-Jul-16	17	1	15	2	2	1	0	7	1	3	1
18328	201	1	12-Apr-16	19	1	15	2	1	1	0	7	1	3	1
18329	201	1	31-Oct-15	20	1	15	2	1	1	0	7	1	3	1
18330	101	1	27-Jul-15	14	1	15	2	1	1	0	7	1	2	1
18331	101	1	19-May-14	22	1	15	2	2	1	0	7	1	5	1
18332	101	1	29-Aug-14	16	1	15	2	1	1	0	7	1	4	2
18333	101	1	8-Feb-14	11	1	15	2	1	1	0	7	1	3	1
18334	101	1	7-Jul-14	21	1	15	2	1	1	0	7	1	3	1
18335	201	1	7-Jun-13	10	1	15	2	3	1	0	7	1	2	1
18336	201	1	5-Oct-13	16	1	15	2	1	1	0	7	1	2	1
18337	201	1	3-Oct-13	16	1	15	2	2	1	0	7	1	3	1
18338	101	1	5-Dec-11	10	1	15	2	1	1	0	7	1	2	1
18339	201	1	14-Sep-11	15	1	15	2	1	1	0	7	1	2	1
18340	101	1	16-Jul-09	20	1	15	2	1	1	0	7	1	3	1
18341	101	1	17-Feb-06	7	1	15	2	2	1	0	7	1	4	1
18342	201	1	21-Oct-09	20	1	15	2	1	1	0	13	1	4	1
18343	201	1	23-Feb-08	20	1	15	2	1	1	0	13	1	1	2
18344	201	1	27-Jul-06	21	1	15	2	3	1	0	13	1	2	1
18345	201	1	27-Jul-06	20	1	15	2	1	1	0	13	1	3	1

18346	201	1	7-Apr-06	12	1	15	2	1	1	0	13	1	4	1
18347	201	1	2-Jan-11	14	1	15	2	2	1	0	1	1	6	1
18348	201	1	4-Aug-14	6	1	15	2	1	1	0	4	1	2	1
18349	101	1	3-Dec-13	20	1	15	2	1	1	0	4	1	3	1
18350	201	1	5-Nov-13	21	1	15	2	1	1	0	4	1	3	1
18351	201	1	12-Sep-12	14	1	15	2	2	1	0	4	1	2	1
18352	201	1	20-Aug-11	6	1	15	2	3	1	0	4	1	2	1
18353	101	1	4-Feb-07	23	1	15	2	1	1	0	4	1	4	1
18354	101	1	28-Nov-07	21	1	15	2	1	1	0	4	1	2	4
18355	201	1	20-Sep-06	22	1	15	2	1	1	0	4	1	2	1
18356	101	1	22-Sep-17	1	1	15	2	1	1	0	4	1	3	1
18357	101	1	25-Oct-17	21	1	15	2	1	1	0	4	1	2	1
18358	101	1	25-Jul-16	1	1	15	2	1	1	0	11	1	2	1
18359	201	1	25-Apr-16	6	1	15	2	3	1	0	11	1	2	1
18360	101	1	13-Apr-16	14	1	15	2	1	1	0	11	1	2	1
18361	201	1	8-Aug-16	21	1	15	2	3	1	0	11	1	2	1
18362	101	1	23-Jan-16	0	1	15	2	1	1	0	11	1	2	1
18363	201	1	2-Nov-16	7	1	15	2	2	1	0	11	1	2	1
18364	201	1	31-Mar-16	20	1	15	2	1	1	0	11	1	2	1
18365	101	1	14-Jun-16	23	1	15	2	1	1	0	11	1	3	1
18366	101	1	5-Jan-16	10	1	15	2	1	1	0	11	1	2	1
18367	201	1	31-Jul-16	18	1	15	2	1	1	0	11	1	2	1
18368	101	1	5-Jun-16	13	1	15	2	1	1	0	11	1	3	1
18369	201	1	10-Sep-16	19	1	15	2	2	1	0	11	1	3	1
18370	201	1	7-Aug-15	10	1	15	2	2	1	0	11	1	2	1
18371	101	1	2-Nov-15	20	1	15	2	1	1	0	11	1	2	1
18372	201	1	5-Oct-15	16	1	15	2	1	1	0	11	1	3	1
18373	201	1	13-Aug-15	17	1	15	2	1	1	0	11	1	1	1
18374	101	1	27-Jun-15	8	1	15	2	1	1	0	11	1	2	1
18375	101	1	30-May-15	18	1	15	2	1	1	0	11	1	1	1
18376	101	1	6-Jan-15	7	1	15	2	1	1	0	11	1	2	1
18377	101	1	10-Jan-15	10	1	15	2	1	1	0	11	1	2	1
18378	101	1	13-Dec-15	17	1	15	2	1	1	0	11	1	3	1
18379	201	1	14-Mar-15	15	1	15	2	2	1	0	11	1	3	1
18380	101	1	5-Oct-15	14	1	15	2	1	1	0	11	1	3	1
18381	101	1	22-Nov-15	8	1	15	2	1	1	0	11	1	2	1
18382	201	1	15-Nov-14	9	1	15	2	2	1	0	11	1	2	1
18383	201	1	25-May-14	20	1	15	2	1	1	0	11	1	2	1
18384	101	1	24-Jul-14	6	1	15	2	1	1	0	11	1	3	1
18385	201	1	28-Feb-14	10	1	15	2	1	1	0	11	1	2	1
18386	101	1	18-Dec-14	17	1	15	2	1	1	0	11	1	2	1
18387	101	1	11-Jan-14	17	1	15	2	1	1	0	11	1	2	1
18388	101	1	4-Jun-14	17	1	15	2	1	1	0	11	1	4	1
18389	201	1	31-Jul-14	8	1	15	2	2	1	0	11	1	3	1
18390	101	1	29-Aug-14	6	1	15	2	1	1	0	11	1	3	1
18391	201	1	4-Jul-13	9	1	15	2	2	1	0	11	1	3	1
18392	101	1	15-Oct-13	7	1	15	2	1	1	0	11	1	5	1
18393	201	1	9-Aug-13	20	1	15	2	3	1	0	11	1	2	1
18394	201	1	25-Aug-13	20	1	15	2	2	1	0	11	1	2	1
18395	201	1	10-Mar-13	23	1	15	2	1	1	0	11	1	1	1
18396	101	1	9-Apr-13	18	1	15	2	1	1	0	11	1	2	1
18397	101	1	5-Apr-13	21	1	15	2	1	1	0	11	1	2	1
18398	101	1	19-Oct-13	7	1	15	2	3	1	0	11	1	3	1
18399	101	1	19-Sep-13	10	1	15	2	1	1	0	11	1	2	1
18400	101	1	30-Apr-12	23	1	15	2	1	1	0	11	1	2	1
18401	101	1	8-Nov-12	21	1	15	2	1	1	0	11	1	2	1
18402	101	1	11-Aug-12	11	1	15	2	1	1	0	11	1	2	1
18403	101	1	11-Jul-12	8	1	15	2	1	1	0	11	1	2	1
18404	101	1	15-Feb-12	7	1	15	2	1	1	0	11	1	2	1
18405	101	1	28-Aug-12	0	1	15	2	1	1	0	11	1	3	1
18406	101	1	23-Aug-12	17	1	15	2	1	1	0	11	1	2	1
18407	101	1	16-Aug-12	10	1	15	2	1	1	0	11	1	3	1
18408	201	1	6-May-11	16	1	15	2	1	1	0	11	1	2	1
18409	101	1	23-May-11	20	1	15	2	1	1	0	11	1	1	1
18410	101	1	22-Feb-11	19	1	15	2	1	1	0	11	1	2	1
18411	101	1	29-May-11	18	1	15	2	1	1	0	11	1	3	1
18412	201	1	26-Jul-11	6	1	15	2	1	1	0	11	1	2	1
18413	101	1	6-May-11	17	1	15	2	1	1	0	11	1	2	1
18414	201	1	13-Feb-11	15	1	15	2	1	1	0	11	1	2	1
18415	101	1	2-Jul-11	14	1	15	2	1	1	0	11	1	3	1
18416	101	1	11-Sep-11	12	1	15	2	1	1	0	11	1	2	1
18417	201	1	20-Mar-11	14	1	15	2	1	1	0	11	1	2	1
18418	201	1	21-Oct-10	19	1	15	2	3	1	0	11	1	2	1
18419	201	1	8-Feb-10	12	1	15	2	1	1	0	11	1	2	1
18420	101	1	12-Mar-10	18	1	15	2	1	1	0	11	1	1	1
18421	101	1	12-Nov-10	19	1	15	2	1	1	0	11	1	2	1
18422	101	1	8-Oct-10	8	1	15	2	1	1	0	11	1	2	1
18423	101	1	23-Nov-10	12	1	15	2	1	1	0	11	1	3	1
18424	101	1	27-May-10	1	1	15	2	1	1	0	11	1	3	1
18425	101	1	13-Dec-10	12	1	15	2	1	1	0	11	1	2	1
18426	201	1	15-Nov-09	17	1	15	2	2	1	0	11	1	2	1
18427	101	1	15-Aug-09	11	1	15	2	1	1	0	11	1	1	1
18428	201	1	11-Dec-09	13	1	15	2	1	1	0	11	1	2	1
18429	201	1	11-Jan-09	7	1	15	2	1	1	0	11	1	3	1
18430	101	1	12-Nov-09	8	1	15	2	1	1	0	11	1	2	1
18431	201	1	27-Nov-09	6	1	15	2	3	1	0	11	1	2	1
18432	101	1	26-Jun-09	20	1	15	2	1	1	0	11	1	2	1
18433	201	1	13-Dec-09	11	1	15	2	2	1	0	11	1	2	1
18434	101	1	8-Jul-09	17	1	15	2	1	1	0	11	1	2	1
18435	201	1	8-May-09	23	1	15	2	3	1	0	11	1	2	1
18436	101	1	5-Jan-08	20	1	15	2	1	1	0	11	1	2	1
18437	201	1	9-Dec-07	12	1	15	2	1	1	0	11	1	3	1
18438	201	1	22-Apr-07	8	1	15	2	1	1	0	11	1	2	1
18439	101	1	29-Dec-06	18	1	15	2	1	1	0	11	1	2	1
18440	101	1	26-Nov-06	17	1	15	2	1	1	0	11	1	2	1
18441	101	1	15-Apr-17	1	1	15	2	1	1	0	11	1	3	1
18442	101	1	18-Feb-17	22	1	15	2	1	1	0	11	1	2	1
18443	201	1	29-Oct-17	11	1	15	2	3	1	0	11	1	3	1
18444	101	1	22-Apr-17	16	1	15	2	1	1	0	11	1	3	1
18445	101	1	5-Sep-17	14	1	15	2	1	1	0	11	1	3	1
18446	201	1	21-Aug-17	9	1	15	2	1	1	0	11	1	2	1
18447	201	1	1-Dec-17	19	1	15	2	2	1	0	11	1	2	1
18448	101	1	16-Dec-17	20	1	15	2	1	1	0	11	1	3	1
18449	101	1	11-Oct-17	19	1	15	2	1	1	0	11	1	2	1
18450	101	1	15-Jun-16	23	1	15	2	1	1	0	3	1	1	1
18451	101	1	31-Mar-16	20	1	15	2	1	1	0	3	1	3	1
18452	201	1	12-Sep-15	21	1	15	2	3	1	0	3	1	1	1
18453	201	1	6-May-15	2	1	15	2	2	1	0	3	1	5	1
18454	101	1	19-Sep-15	22	1	15	2	1	1	0	3	1	1	1
18455	201	1	28-Aug-15	10	1	15	2	3	1	0	3	1	1	1
18456	101	1	12-May-15	21	1	15	2	1	1	0	3	1	1	1
18457	101	1	8-Sep-15	23	1	15	2	2	1	0	3	1	4	1
18458	101	1	13-Dec-14	20	1	15	2	1	1	0	3	1	3	1
18459	201	1	5-Mar-14	2	1	15	2	2	1	0	3	1	2	2
18460	101	1	13-Apr-14	16	1	15	2	1	1	0	3	1	3	1
18461	101	1	5-Jul-13	16	1	15	2	1	1	0	3	1	4	4
18462	201	1	2-Sep-13	18	1	15	2	1	1	0	3	1	1	1
18463	101	1	10-Dec-13	16	1	15	2	1	1	0	3	1	7	1
18464	101	1	14-Jun-13	14	1	15	2	1	1	0	3	1	2	1
18465	101	1	8-Jun-13	7	1	15	2	1	1	0	3	1	3	1
18466	101	1	24-May-13	17	1	15	2	1	1	0	3	1	2	4
18467	201	1	21-Apr-13	12	1	15	2	2	1	0	3	1	2	1
18468	201	1	31-Dec-12	14	1	15	2	2	1	0	3	1	1	1
18469	101	1	17-Feb-12	20	1	15	2	1	1	0	3	1	1	1

18470	101	1	6-Feb-12	22	1	15	2	1	1	0	3	1	1	1
18471	201	1	14-Mar-12	19	1	15	2	2	1	0	3	1	3	1
18472	201	1	15-Jul-12	17	1	15	2	1	1	0	3	1	3	1
18473	201	1	27-Aug-11	23	1	15	2	1	1	0	3	1	1	1
18474	201	1	31-Mar-11	22	1	15	2	2	1	0	3	1	1	1
18475	201	1	21-Aug-11	14	1	15	2	3	1	0	3	1	2	1
18476	101	1	10-Oct-11	20	1	15	2	1	1	0	3	1	2	1
18477	101	1	20-Apr-11	15	1	15	2	3	1	0	3	1	4	1
18478	201	1	2-Oct-10	9	1	15	2	2	1	0	3	1	1	1
18479	101	1	1-Jan-10	17	1	15	2	1	1	0	3	1	1	1
18480	101	1	24-Jun-10	19	1	15	2	1	1	0	3	1	3	1
18481	201	1	8-May-09	11	1	15	2	2	1	0	3	1	3	1
18482	101	1	8-May-09	0	1	15	2	2	1	0	3	1	5	1
18483	101	1	16-May-09	19	1	15	2	1	1	0	3	1	4	1
18484	201	1	23-Nov-09	16	1	15	2	3	1	0	3	1	2	1
18485	101	1	19-Sep-09	22	1	15	2	1	1	0	3	1	3	1
18486	201	1	19-Nov-08	22	1	15	2	2	1	0	3	1	4	1
18487	201	1	24-Oct-08	18	1	15	2	3	1	0	3	1	2	1
18488	101	1	9-Sep-08	18	1	15	2	1	1	0	3	1	3	1
18489	101	1	11-Apr-08	21	1	15	2	1	1	0	3	1	2	4
18490	201	1	10-Apr-07	9	1	15	2	2	1	0	3	1	1	1
18491	101	1	19-Nov-07	22	1	15	2	1	1	0	3	1	1	1
18492	201	1	19-Jun-07	19	1	15	2	3	1	0	3	1	3	2
18493	101	1	11-Jun-07	21	1	15	2	1	1	0	3	1	3	1
18494	201	1	19-Feb-06	3	1	15	2	1	1	0	3	1	1	1
18495	201	1	1-Oct-06	14	1	15	2	2	1	0	3	1	4	1
18496	101	1	15-Aug-06	16	1	15	2	1	1	0	3	1	2	1
18497	101	1	7-Jan-17	10	1	15	2	1	1	0	3	1	3	1
18498	101	1	22-May-17	20	1	15	2	2	1	0	3	1	2	1
18499	201	1	28-Jun-17	21	1	15	2	2	1	0	3	1	2	1
18500	101	1	5-Feb-16	11	1	15	2	3	1	0	6	1	2	2
18501	101	1	6-Aug-16	17	1	15	2	1	1	0	6	1	2	1
18502	101	1	15-Apr-16	12	1	15	2	1	1	0	6	1	2	1
18503	101	1	31-Jan-16	15	1	15	2	1	1	0	6	1	2	1
18504	101	1	1-Jun-16	23	1	15	2	1	1	0	6	1	2	1
18505	101	1	31-Mar-16	18	1	15	2	1	1	0	6	1	2	1
18506	101	1	1-Jan-16	16	1	15	2	1	1	0	6	1	2	1
18507	101	1	1-May-15	17	1	15	2	1	1	0	6	1	2	1
18508	101	1	6-Feb-15	23	1	15	2	1	1	0	6	1	2	1
18509	101	1	5-May-15	10	1	15	2	1	1	0	6	1	2	1
18510	101	1	10-Sep-15	17	1	15	2	1	1	0	6	1	2	1
18511	101	1	22-Oct-15	15	1	15	2	1	1	0	6	1	2	1
18512	101	1	4-Sep-15	12	1	15	2	2	1	0	6	1	2	4
18513	201	1	26-Apr-15	11	1	15	2	2	1	0	6	1	2	1
18514	101	1	11-Jan-15	0	1	15	2	2	1	0	6	1	2	1
18515	101	1	26-Nov-15	23	1	15	2	1	1	0	6	1	2	1
18516	101	1	12-Dec-14	10	1	15	2	1	1	0	6	1	2	1
18517	101	1	6-Jul-14	1	1	15	2	2	1	0	6	1	3	2
18518	101	1	12-Aug-14	23	1	15	2	1	1	0	6	1	2	1
18519	101	1	22-Feb-14	16	1	15	2	1	1	0	6	1	2	1
18520	101	1	16-Sep-13	2	1	15	2	1	1	0	6	1	1	1
18521	101	1	21-Jul-13	19	1	15	2	1	1	0	6	1	3	1
18522	101	1	2-Sep-13	10	1	15	2	1	1	0	6	1	2	1
18523	101	1	22-Apr-13	19	1	15	2	1	1	0	6	1	2	1
18524	101	1	30-Mar-12	12	1	15	2	1	1	0	6	1	2	1
18525	101	1	28-Dec-12	22	1	15	2	1	1	0	6	1	2	1
18526	101	1	9-Mar-12	12	1	15	2	1	1	0	6	1	2	2
18527	101	1	28-Sep-12	22	1	15	2	1	1	0	6	1	2	1
18528	101	1	22-Jun-12	22	1	15	2	1	1	0	6	1	3	1
18529	201	1	29-Sep-12	16	1	15	2	1	1	0	6	1	2	1
18530	101	1	27-Feb-12	18	1	15	2	1	1	0	6	1	2	1
18531	101	1	15-Sep-12	23	1	15	2	1	1	0	6	1	3	1
18532	101	1	4-Aug-11	10	1	15	2	1	1	0	6	1	2	4
18533	101	1	9-Nov-11	22	1	15	2	1	1	0	6	1	2	1
18534	201	1	26-Apr-11	23	1	15	2	1	1	0	6	1	2	1
18535	201	1	10-Apr-11	9	1	15	2	2	1	0	6	1	2	1
18536	101	1	1-Aug-11	11	1	15	2	2	1	0	6	1	2	1
18537	201	1	17-Apr-11	9	1	15	2	2	1	0	6	1	2	1
18538	101	1	1-Aug-10	16	1	15	2	1	1	0	6	1	2	1
18539	101	1	29-Dec-10	17	1	15	2	3	1	0	6	1	2	1
18540	101	1	25-Mar-10	15	1	15	2	1	1	0	6	1	2	1
18541	101	1	20-Oct-10	7	1	15	2	1	1	0	6	1	2	1
18542	201	1	13-Apr-10	18	1	15	2	2	1	0	6	1	3	1
18543	201	1	16-Jun-10	8	1	15	2	2	1	0	6	1	2	1
18544	101	1	3-Oct-10	23	1	15	2	1	1	0	6	1	3	1
18545	101	1	9-Jul-09	15	1	15	2	1	1	0	6	1	2	4
18546	201	1	31-May-09	22	1	15	2	2	1	0	6	1	2	1
18547	201	1	19-May-09	14	1	15	2	1	1	0	6	1	2	1
18548	101	1	28-May-09	17	1	15	2	1	1	0	6	1	2	1
18549	201	1	13-Sep-09	21	1	15	2	1	1	0	6	1	3	1
18550	101	1	6-Sep-09	23	1	15	2	1	1	0	6	1	2	1
18551	101	1	21-Aug-09	3	1	15	2	3	1	0	6	1	2	1
18552	201	1	26-Oct-08	20	1	15	2	3	1	0	6	1	2	1
18553	201	1	9-Sep-08	21	1	15	2	1	1	0	6	1	2	1
18554	201	1	28-Oct-08	21	1	15	2	1	1	0	6	1	2	1
18555	101	1	9-Oct-08	21	1	15	2	1	1	0	6	1	2	1
18556	201	1	8-Jul-08	12	1	15	2	3	1	0	6	1	2	1
18557	101	1	10-Jun-08	18	1	15	2	1	1	0	6	1	3	1
18558	201	1	31-Jan-07	11	1	15	2	1	1	0	6	1	2	1
18559	201	1	20-Aug-07	23	1	15	2	1	1	0	6	1	2	1
18560	101	1	14-Jul-07	17	1	15	2	1	1	0	6	1	2	1
18561	201	1	16-Feb-07	7	1	15	2	3	1	0	6	1	3	1
18562	201	1	1-Nov-06	14	1	15	2	1	1	0	6	1	2	1
18563	101	1	17-Jul-06	21	1	15	2	1	1	0	6	1	3	1
18564	101	1	5-Aug-06	12	1	15	2	1	1	0	6	1	3	1
18565	101	1	16-May-06	19	1	15	2	1	1	0	6	1	2	1
18566	101	1	31-Jan-17	22	1	15	2	1	1	0	6	1	2	1
18567	101	1	5-Jul-17	20	1	15	2	1	1	0	6	1	2	1
18568	101	1	23-Jun-17	23	1	15	2	2	1	0	6	1	2	1
18569	101	1	27-Sep-17	22	1	15	2	1	1	0	6	1	2	1
18570	101	1	29-Nov-17	20	1	15	2	1	1	0	6	1	2	1
18571	101	1	13-Oct-17	11	1	15	2	1	1	0	6	1	4	1
18572	101	1	24-Oct-17	12	1	15	2	1	1	0	6	1	2	1
18573	101	1	22-Nov-17	11	1	15	2	1	1	0	6	1	2	1
18574	101	1	5-Mar-17	5	1	15	2	1	1	0	13	1	1	1
18575	101	1	17-Jan-17	10	1	15	2	1	1	0	13	1	4	2
18576	201	1	15-Apr-14	19	2	15	2	3	1	0	6	1	2	1
18577	101	1	4-Sep-17	19	2	15	2	1	1	0	6	1	1	1
18578	201	1	19-Aug-17	14	2	15	2	1	1	0	7	1	3	1
18579	201	1	22-Jul-16	19	2	15	2	1	1	0	6	1	2	1
18580	101	1	8-Sep-17	18	2	15	2	1	1	0	6	1	2	1
18581	101	1	13-Aug-17	22	2	15	2	1	1	0	11	1	3	1
18582	201	1	2-Feb-12	22	2	15	2	2	1	0	3	1	1	1
18583	201	1	9-Sep-14	13	2	15	2	1	1	0	6	1	3	1
18584	201	1	7-Dec-13	7	2	15	2	1	1	0	6	1	2	1
18585	201	1	4-Dec-16	18	2	15	2	1	1	0	6	1	3	1
18586	201	1	5-Feb-08	17	2	15	2	2	1	0	10	1	7	2
18587	101	1	14-Dec-15	20	2	15	2	1	1	0	7	1	2	1
18588	101	1	30-Mar-15	12	2	15	2	1	1	0	7	1	3	1
18589	101	1	1-Jan-14	16	2	15	2	2	1	0	7	1	2	1
18590	101	1	16-May-14	23	2	15	2	3	1	0	7	1	3	1
18591	201	1	21-Jun-13	10	2	15	2	3	1	0	7	1	5	1
18592	101	1	27-Oct-13	23	2	15	2	1	1	0	7	1	1	1
18593	201	1	2-Jan-12	8	2	15	2	2	1	0	7	1	2	1

18594	201	1	30-Jul-12	21	2	15	2	3	1	0	7	1	5	1
18595	101	1	21-Jul-12	21	2	15	2	1	1	0	7	1	3	1
18596	201	1	11-Jan-09	11	2	15	2	2	1	0	7	1	1	2
18597	101	1	25-Sep-08	8	2	15	2	1	1	0	7	1	2	1
18598	101	1	29-Mar-08	17	2	15	2	1	1	0	7	1	3	1
18599	101	1	13-Aug-17	1	2	15	2	1	1	0	7	1	2	1
18600	101	1	31-Aug-17	14	2	15	2	1	1	0	7	1	3	1
18601	101	1	2-May-15	22	2	15	2	1	1	0	4	1	3	1
18602	201	1	11-Aug-14	19	2	15	2	3	1	0	4	1	2	1
18603	101	1	14-Jul-11	22	2	15	2	1	1	0	4	1	1	1
18604	201	1	1-Sep-11	9	2	15	2	2	1	0	4	1	3	1
18605	101	1	16-May-10	20	2	15	2	1	1	0	4	1	1	1
18606	201	1	13-Jan-10	17	2	15	2	1	1	0	4	1	2	1
18607	201	1	1-Nov-10	21	2	15	2	3	1	0	4	1	2	2
18608	101	1	6-Oct-09	16	2	15	2	1	1	0	4	1	2	1
18609	101	1	14-May-08	18	2	15	2	1	1	0	4	1	3	1
18610	201	1	11-Nov-08	20	2	15	2	3	1	0	4	1	2	1
18611	201	1	16-Feb-16	16	2	15	2	1	1	0	11	1	3	1
18612	201	1	9-May-16	20	2	15	2	1	1	0	11	1	1	1
18613	101	1	24-May-16	18	2	15	2	1	1	0	11	1	3	1
18614	101	1	5-Jun-16	18	2	15	2	1	1	0	11	1	2	1
18615	101	1	24-May-16	10	2	15	2	1	1	0	11	1	2	1
18616	101	1	23-May-16	23	2	15	2	1	1	0	11	1	2	1
18617	101	1	4-Jul-16	23	2	15	2	1	1	0	11	1	3	1
18618	101	1	6-Feb-16	13	2	15	2	1	1	0	11	1	2	1
18619	101	1	27-Mar-16	16	2	15	2	1	1	0	11	1	2	1
18620	101	1	30-Dec-15	11	2	15	2	1	1	0	11	1	3	1
18621	101	1	9-Sep-15	18	2	15	2	1	1	0	11	1	3	1
18622	101	1	17-Sep-15	16	2	15	2	1	1	0	11	1	1	1
18623	101	1	25-May-15	20	2	15	2	1	1	0	11	1	1	1
18624	101	1	3-Apr-15	12	2	15	2	1	1	0	11	1	2	1
18625	201	1	22-Apr-15	18	2	15	2	3	1	0	11	1	2	1
18626	101	1	23-Jun-15	12	2	15	2	1	1	0	11	1	3	1
18627	101	1	11-Jul-14	20	2	15	2	1	1	0	11	1	2	1
18628	101	1	23-Jan-14	8	2	15	2	1	1	0	11	1	2	1
18629	101	1	8-Jan-14	12	2	15	2	1	1	0	11	1	2	1
18630	101	1	24-May-14	18	2	15	2	1	1	0	11	1	1	1
18631	201	1	30-Nov-14	10	2	15	2	3	1	0	11	1	2	1
18632	101	1	15-Jan-14	12	2	15	2	1	1	0	11	1	2	1
18633	101	1	21-Aug-13	19	2	15	2	1	1	0	11	1	4	1
18634	101	1	6-Feb-13	17	2	15	2	3	1	0	11	1	5	1
18635	101	1	2-Sep-13	7	2	15	2	1	1	0	11	1	3	1
18636	101	1	28-May-13	8	2	15	2	1	1	0	11	1	2	1
18637	101	1	13-Jun-13	18	2	15	2	1	1	0	11	1	2	1
18638	101	1	31-May-13	18	2	15	2	1	1	0	11	1	2	1
18639	101	1	12-Oct-13	7	2	15	2	1	1	0	11	1	3	1
18640	101	1	9-Mar-13	15	2	15	2	1	1	0	11	1	2	1
18641	101	1	29-Jul-13	7	2	15	2	1	1	0	11	1	2	1
18642	101	1	13-May-13	19	2	15	2	3	1	0	11	1	2	1
18643	101	1	17-Nov-12	2	2	15	2	3	1	0	11	1	1	1
18644	201	1	9-Feb-12	10	2	15	2	1	1	0	11	1	3	1
18645	101	1	24-Aug-12	8	2	15	2	1	1	0	11	1	1	1
18646	201	1	25-Apr-12	16	2	15	2	1	1	0	11	1	3	1
18647	101	1	19-Jun-12	20	2	15	2	1	1	0	11	1	2	1
18648	101	1	2-Feb-12	17	2	15	2	1	1	0	11	1	2	1
18649	101	1	22-May-12	12	2	15	2	1	1	0	11	1	2	1
18650	101	1	26-Aug-12	17	2	15	2	1	1	0	11	1	2	1
18651	101	1	23-Nov-12	20	2	15	2	1	1	0	11	1	2	1
18652	101	1	22-May-12	12	2	15	2	1	1	0	11	1	2	1
18653	201	1	2-Aug-11	17	2	15	2	1	1	0	11	1	2	1
18654	101	1	15-Aug-11	19	2	15	2	1	1	0	11	1	2	1
18655	101	1	20-Apr-11	12	2	15	2	1	1	0	11	1	2	1
18656	101	1	31-Jan-11	8	2	15	2	1	1	0	11	1	2	1
18657	101	1	7-Feb-11	18	2	15	2	1	1	0	11	1	2	1
18658	101	1	31-Mar-11	8	2	15	2	3	1	0	11	1	2	1
18659	101	1	6-Apr-11	8	2	15	2	1	1	0	11	1	2	1
18660	101	1	28-Jul-11	17	2	15	2	2	1	0	11	1	3	1
18661	201	1	23-Feb-11	18	2	15	2	3	1	0	11	1	3	1
18662	201	1	30-Jul-10	15	2	15	2	2	1	0	11	1	2	1
18663	201	1	5-Aug-10	13	2	15	2	3	1	0	11	1	4	1
18664	101	1	21-Aug-10	12	2	15	2	1	1	0	11	1	2	1
18665	101	1	3-Aug-10	12	2	15	2	1	1	0	11	1	2	1
18666	101	1	3-Aug-10	13	2	15	2	1	1	0	11	1	2	1
18667	101	1	17-Mar-10	20	2	15	2	1	1	0	11	1	2	1
18668	101	1	7-Apr-10	21	2	15	2	1	1	0	11	1	3	1
18669	101	1	23-Oct-10	17	2	15	2	1	1	0	11	1	2	1
18670	101	1	21-Apr-10	6	2	15	2	2	1	0	11	1	2	1
18671	101	1	22-Aug-10	20	2	15	2	1	1	0	11	1	2	1
18672	101	1	9-Jul-09	15	2	15	2	3	1	0	11	1	3	1
18673	201	1	18-Jun-09	20	2	15	2	1	1	0	11	1	7	1
18674	101	1	8-Jul-09	17	2	15	2	3	1	0	11	1	3	1
18675	201	1	27-Jun-09	8	2	15	2	2	1	0	11	1	2	1
18676	101	1	8-May-09	18	2	15	2	1	1	0	11	1	2	1
18677	101	1	15-Sep-09	21	2	15	2	1	1	0	11	1	2	1
18678	201	1	24-Mar-08	21	2	15	2	3	1	0	11	1	2	1
18679	201	1	21-Aug-08	9	2	15	2	3	1	0	11	1	2	1
18680	201	1	4-Jul-08	17	2	15	2	2	1	0	11	1	3	1
18681	201	1	16-Nov-08	14	2	15	2	3	1	0	11	1	3	1
18682	201	1	11-Apr-07	12	2	15	2	1	1	0	11	1	3	1
18683	201	1	30-Dec-07	12	2	15	2	2	1	0	11	1	3	1
18684	101	1	19-Feb-07	19	2	15	2	1	1	0	11	1	3	1
18685	201	1	20-Mar-07	19	2	15	2	1	1	0	11	1	1	1
18686	101	1	17-Jun-06	9	2	15	2	1	1	0	11	1	2	1
18687	101	1	16-Jan-17	16	2	15	2	1	1	0	11	1	3	1
18688	201	1	7-Mar-17	17	2	15	2	1	1	0	11	1	2	1
18689	101	1	1-Aug-17	20	2	15	2	1	1	0	11	1	1	1
18690	101	1	29-Oct-17	18	2	15	2	1	1	0	11	1	2	1
18691	101	1	16-Sep-17	10	2	15	2	1	1	0	11	1	2	1
18692	201	1	28-Jun-17	12	2	15	2	1	1	0	11	1	3	1
18693	101	1	10-Jul-17	10	2	15	2	1	1	0	11	1	2	1
18694	101	1	20-Feb-17	17	2	15	2	1	1	0	11	1	2	1
18695	201	1	16-Nov-17	19	2	15	2	1	1	0	11	1	2	1
18696	201	1	1-Aug-17	10	2	15	2	2	1	0	11	1	2	1
18697	201	1	5-Jun-17	18	2	15	2	2	1	0	11	1	2	1
18698	101	1	5-Feb-16	4	2	15	2	1	1	0	3	1	1	1
18699	201	1	17-Apr-15	22	2	15	2	2	1	0	3	1	4	1
18700	201	1	5-Nov-15	20	2	15	2	1	1	0	3	1	4	1
18701	201	1	9-Jan-13	22	2	15	2	3	1	0	3	1	5	1
18702	201	1	21-Apr-13	7	2	15	2	2	1	0	3	1	3	1
18703	201	1	11-Feb-13	7	2	15	2	1	1	0	3	1	3	1
18704	101	1	31-May-13	23	2	15	2	1	1	0	3	1	3	1
18705	101	1	23-Jul-13	23	2	15	2	1	1	0	3	1	3	1
18706	101	1	13-Sep-12	20	2	15	2	1	1	0	3	1	2	1
18707	101	1	19-Jul-12	23	2	15	2	1	1	0	3	1	4	1
18708	201	1	18-Mar-12	17	2	15	2	1	1	0	3	1	3	1
18709	201	1	26-Nov-11	15	2	15	2	1	1	0	3	1	1	1
18710	101	1	25-Apr-11	17	2	15	2	1	1	0	3	1	3	1
18711	101	1	4-May-11	21	2	15	2	1	1	0	3	1	1	1
18712	101	1	12-Aug-11	4	2	15	2	1	1	0	3	1	3	2
18713	101	1	19-Jun-10	16	2	15	2	1	1	0	3	1	4	1
18714	201	1	4-Jul-10	20	2	15	2	1	1	0	3	1	3	2
18715	101	1	13-Nov-10	18	2	15	2	1	1	0	3	1	2	1
18716	201	1	20-Apr-09	10	2	15	2	2	1	0	3	1	1	1
18717	101	1	20-May-09	22	2	15	2	1	1	0	3	1	2	1

18718	101	1	12-Aug-09	16	2	15	2	1	1	0	3	1	2	1
18719	101	1	28-Jul-08	21	2	15	2	3	1	0	3	1	1	2
18720	101	1	8-Jun-08	11	2	15	2	1	1	0	3	1	1	1
18721	101	1	16-Sep-07	9	2	15	2	1	1	0	3	1	1	1
18722	101	1	22-Oct-07	21	2	15	2	1	1	0	3	1	2	1
18723	101	1	12-Feb-17	3	2	15	2	1	1	0	3	1	1	1
18724	201	1	18-Feb-17	0	2	15	2	3	1	0	3	1	3	1
18725	101	1	23-Jan-16	4	2	15	2	1	1	0	6	1	2	1
18726	101	1	28-Oct-15	23	2	15	2	1	1	0	6	1	2	1
18727	101	1	1-Aug-15	16	2	15	2	1	1	0	6	1	4	1
18728	101	1	6-May-15	17	2	15	2	1	1	0	6	1	3	1
18729	201	1	19-Sep-14	20	2	15	2	2	1	0	6	1	2	1
18730	101	1	18-Oct-13	19	2	15	2	1	1	0	6	1	2	1
18731	201	1	25-Jan-13	9	2	15	2	2	1	0	6	1	2	1
18732	101	1	7-Sep-13	12	2	15	2	1	1	0	6	1	2	1
18733	101	1	6-Oct-12	9	2	15	2	1	1	0	6	1	2	1
18734	101	1	18-Oct-12	23	2	15	2	1	1	0	6	1	3	4
18735	101	1	27-Aug-11	16	2	15	2	1	1	0	6	1	3	1
18736	101	1	26-May-11	23	2	15	2	1	1	0	6	1	2	1
18737	201	1	22-Jul-10	18	2	15	2	1	1	0	6	1	2	1
18738	201	1	17-Jan-10	19	2	15	2	2	1	0	6	1	3	1
18739	101	1	9-Oct-10	23	2	15	2	1	1	0	6	1	2	1
18740	201	1	26-Nov-10	12	2	15	2	1	1	0	6	1	2	1
18741	101	1	13-Jun-08	21	2	15	2	1	1	0	6	1	2	1
18742	201	1	14-Aug-07	21	2	15	2	1	1	0	6	1	2	1
18743	201	1	16-Jan-07	7	2	15	2	1	1	0	6	1	2	1
18744	101	1	21-Dec-07	22	2	15	2	1	1	0	6	1	2	1
18745	201	1	12-Nov-06	21	2	15	2	1	1	0	6	1	2	1
18746	101	1	13-Oct-17	18	2	15	2	1	1	0	6	1	2	1
18747	202	0	29-Dec-13	15	1	16	2	2	1	0	6	1	2	1
18748	202	0	5-Dec-14	19	1	16	2	3	1	0	10	1	7	2
18749	202	0	26-Dec-14	21	1	16	2	1	1	0	10	1	6	2
18750	202	0	1-Jan-14	3	1	16	2	1	1	0	10	1	7	1
18751	202	0	17-Aug-16	0	1	16	2	1	1	0	10	1	6	2
18752	202	0	6-Nov-14	15	1	16	2	1	1	0	4	1	1	1
18753	202	0	15-Nov-12	21	1	16	2	1	1	0	6	1	1	1
18754	202	0	10-Feb-13	20	1	16	2	2	1	0	10	1	1	2
18755	202	0	19-Dec-10	21	1	16	2	1	1	0	10	1	7	1
18756	202	0	17-Oct-10	17	1	16	2	1	1	0	10	1	1	2
18757	202	0	20-Aug-10	6	1	16	2	1	1	0	10	1	7	1
18758	202	0	8-Aug-12	14	1	16	2	1	1	0	7	1	6	2
18759	202	0	16-Jul-09	20	1	16	2	1	1	0	7	1	2	1
18760	202	0	3-Apr-09	17	1	16	2	1	1	0	7	1	4	1
18761	202	0	24-Sep-08	20	1	16	2	1	1	0	13	1	1	1
18762	202	0	22-Sep-07	21	1	16	2	1	1	0	13	1	4	2
18763	202	0	13-Jan-06	23	1	16	2	1	1	0	13	1	7	1
18764	202	0	6-Aug-15	10	1	16	2	1	1	0	4	1	3	2
18765	202	0	12-Jul-13	18	1	16	2	1	1	0	4	1	2	1
18766	202	0	5-Nov-12	22	1	16	2	1	1	0	4	1	2	1
18767	202	0	27-Apr-10	17	1	16	2	1	1	0	4	1	1	1
18768	202	0	1-Dec-10	19	1	16	2	3	1	0	4	1	1	1
18769	202	0	20-Sep-10	20	1	16	2	1	1	0	4	1	3	2
18770	202	0	10-Nov-10	19	1	16	2	1	1	0	4	1	3	2
18771	202	0	1-Oct-07	11	1	16	2	2	1	0	4	1	1	1
18772	202	0	2-Sep-17	15	1	16	2	3	1	0	4	1	2	1
18773	202	0	26-Feb-16	23	1	16	2	2	1	0	11	1	2	1
18774	202	0	30-Apr-16	15	1	16	2	1	1	0	11	1	3	1
18775	202	0	14-Aug-16	15	1	16	2	1	1	0	11	1	2	1
18776	202	0	8-Jun-15	7	1	16	2	2	1	0	11	1	2	1
18777	202	0	27-Jun-15	18	1	16	2	2	1	0	11	1	3	1
18778	202	0	29-Sep-15	14	1	16	2	1	1	0	11	1	2	1
18779	202	0	14-Jun-14	21	1	16	2	1	1	0	11	1	2	1
18780	202	0	8-Jun-13	7	1	16	2	1	1	0	11	1	3	1
18781	202	0	29-Aug-13	22	1	16	2	3	1	0	11	1	3	1
18782	202	0	11-Oct-13	0	1	16	2	1	1	0	11	1	3	1
18783	202	0	26-Dec-13	13	1	16	2	1	1	0	11	1	2	1
18784	202	0	26-Apr-11	13	1	16	2	1	1	0	11	1	2	1
18785	202	0	18-Mar-11	19	1	16	2	1	1	0	11	1	2	2
18786	202	0	22-Sep-11	19	1	16	2	1	1	0	11	1	3	2
18787	202	0	5-Sep-17	12	1	16	2	2	1	0	11	1	1	1
18788	202	0	25-Jan-17	21	1	16	2	3	1	0	11	1	2	1
18789	202	0	12-Mar-16	18	1	16	2	2	1	0	3	1	2	1
18790	202	0	21-Nov-15	9	1	16	2	1	1	0	3	1	2	1
18791	202	0	16-Feb-14	5	1	16	2	1	1	0	3	1	1	1
18792	202	0	14-Jan-14	17	1	16	2	2	1	0	3	1	2	1
18793	202	0	1-Nov-14	12	1	16	2	3	1	0	3	1	1	1
18794	202	0	24-Nov-14	20	1	16	2	1	1	0	3	1	3	1
18795	202	0	29-Oct-14	22	1	16	2	2	1	0	3	1	2	1
18796	202	0	16-Nov-13	17	1	16	2	1	1	0	3	1	1	1
18797	202	0	7-Apr-13	10	1	16	2	1	1	0	3	1	4	1
18798	202	0	20-Sep-13	22	1	16	2	1	1	0	3	1	1	1
18799	202	0	14-May-12	3	1	16	2	1	1	0	3	1	1	1
18800	202	0	18-Dec-11	3	1	16	2	1	1	0	3	1	5	2
18801	202	0	26-Dec-11	22	1	16	2	1	1	0	3	1	1	2
18802	202	0	5-Jun-11	20	1	16	2	1	1	0	3	1	1	2
18803	202	0	18-Aug-10	1	1	16	2	2	1	0	3	1	6	2
18804	202	0	1-Aug-10	11	1	16	2	1	1	0	3	1	1	1
18805	202	0	5-May-10	22	1	16	2	1	1	0	3	1	1	1
18806	202	0	6-Jul-09	19	1	16	2	1	1	0	3	1	1	2
18807	202	0	29-May-09	1	1	16	2	1	1	0	3	1	3	2
18808	202	0	19-Nov-09	17	1	16	2	1	1	0	3	1	3	1
18809	202	0	1-Dec-09	10	1	16	2	1	1	0	3	1	1	2
18810	202	0	27-Nov-08	2	1	16	2	1	1	0	3	1	2	2
18811	202	0	11-Jan-08	16	1	16	2	1	1	0	3	1	1	1
18812	202	0	29-May-17	13	1	16	2	1	1	0	3	1	1	1
18813	202	0	7-Aug-16	22	1	16	2	1	1	0	6	1	4	2
18814	202	0	25-Jun-15	7	1	16	2	1	1	0	6	1	2	1
18815	202	0	16-May-13	12	1	16	2	1	1	0	6	1	2	1
18816	202	0	9-Jul-11	20	1	16	2	1	1	0	6	1	2	2
18817	202	0	27-Aug-11	17	1	16	2	1	1	0	6	1	1	2
18818	202	0	12-Dec-10	16	1	16	2	1	1	0	6	1	2	1
18819	202	0	8-May-10	9	1	16	2	1	1	0	6	1	7	1
18820	202	0	3-Apr-08	19	1	16	2	1	1	0	6	1	2	2
18821	202	0	10-Aug-08	21	1	16	2	1	1	0	6	1	2	1
18822	202	0	20-May-08	21	1	16	2	1	1	0	6	1	2	1
18823	202	0	15-Oct-07	16	1	16	2	1	1	0	6	1	2	1
18824	202	0	21-Sep-07	18	1	16	2	1	1	0	6	1	2	2
18825	202	0	21-Jul-16	8	1	16	2	3	1	0	13	1	1	1
18826	202	0	27-Jan-07	12	1	16	2	1	1	0	13	1	1	1
18827	202	0	9-May-14	20	2	16	2	1	1	0	10	1	7	2
18828	202	0	22-Dec-13	17	2	16	2	1	1	0	10	1	7	2
18829	202	0	14-Mar-12	20	2	16	2	1	1	0	10	1	7	2
18830	202	0	29-Feb-12	21	2	16	2	1	1	0	10	1	7	2
18831	202	0	7-Feb-16	0	2	16	2	3	1	0	10	1	6	2
18832	202	0	20-Feb-17	20	2	16	2	1	1	0	10	1	6	2
18833	202	0	1-Jan-16	18	2	16	2	1	1	0	7	1	3	1
18834	202	0	5-Oct-12	1	2	16	2	1	1	0	10	1	7	1
18835	202	0	3-Mar-12	23	2	16	2	1	1	0	10	1	7	1
18836	202	0	26-Feb-12	13	2	16	2	1	1	0	10	1	7	2
18837	202	0	16-Jun-13	22	2	16	2	1	1	0	6	1	2	1
18838	202	0	17-Jan-11	21	2	16	2	1	1	0	10	1	7	2
18839	202	0	15-Jul-11	0	2	16	2	1	1	0	10	1	7	2
18840	202	0	26-Jun-11	19	2	16	2	1	1	0	10	1	7	1
18841	202	0	23-Dec-09	19	2	16	2	1	1	0	10	1	7	2

18842	202	0	16-Mar-09	9	2	16	2	1	1	0	10	1	7	1
18843	202	0	18-Nov-09	19	2	16	2	1	1	0	10	1	7	2
18844	202	0	6-Sep-13	15	2	16	2	1	1	0	7	1	2	1
18845	202	0	5-May-12	13	2	16	2	1	1	0	7	1	3	1
18846	202	0	16-Feb-12	20	2	16	2	1	1	0	7	1	3	2
18847	202	0	11-Jun-11	18	2	16	2	3	1	0	7	1	2	1
18848	202	0	8-Feb-07	18	2	16	2	3	1	0	7	1	1	2
18849	202	0	3-Jun-14	16	2	16	2	1	1	0	4	1	2	2
18850	202	0	5-Aug-13	23	2	16	2	1	1	0	4	1	1	1
18851	202	0	21-Nov-10	17	2	16	2	1	1	0	4	1	2	1
18852	202	0	9-Dec-10	18	2	16	2	1	1	0	4	1	3	1
18853	202	0	8-May-09	20	2	16	2	1	1	0	4	1	2	1
18854	202	0	12-Apr-09	10	2	16	2	1	1	0	4	1	2	1
18855	202	0	13-Aug-17	17	2	16	2	3	1	0	4	1	3	1
18856	202	0	5-Jul-13	9	2	16	2	1	1	0	11	1	3	1
18857	202	0	30-Jun-12	8	2	16	2	1	1	0	11	1	3	1
18858	202	0	14-Mar-12	21	2	16	2	1	1	0	11	1	2	1
18859	202	0	22-Oct-11	19	2	16	2	1	1	0	11	1	4	2
18860	202	0	7-Jun-10	14	2	16	2	1	1	0	11	1	3	1
18861	202	0	16-Apr-08	20	2	16	2	1	1	0	11	1	3	1
18862	202	0	31-Jul-17	14	2	16	2	1	1	0	11	1	3	1
18863	202	0	1-Aug-17	23	2	16	2	1	1	0	11	1	3	1
18864	202	0	3-Feb-17	11	2	16	2	2	1	0	11	1	2	1
18865	202	0	16-Jun-17	15	2	16	2	3	1	0	11	1	5	1
18866	202	0	13-Feb-16	11	2	16	2	1	1	0	3	1	1	1
18867	202	0	1-Nov-14	2	2	16	2	1	1	0	3	1	1	1
18868	202	0	21-Dec-13	14	2	16	2	1	1	0	3	1	4	1
18869	202	0	3-Nov-13	17	2	16	2	1	1	0	3	1	3	1
18870	202	0	28-May-13	4	2	16	2	1	1	0	3	1	3	1
18871	202	0	19-Jan-12	18	2	16	2	1	1	0	3	1	1	1
18872	202	0	8-Aug-11	8	2	16	2	1	1	0	3	1	1	2
18873	202	0	25-Sep-11	12	2	16	2	1	1	0	3	1	1	1
18874	202	0	14-Apr-09	5	2	16	2	2	1	0	3	1	1	2
18875	202	0	19-May-09	19	2	16	2	1	1	0	3	1	3	1
18876	202	0	23-Jun-09	2	2	16	2	1	1	0	3	1	3	2
18877	202	0	29-May-08	6	2	16	2	1	1	0	3	1	1	1
18878	202	0	24-Oct-09	13	2	16	2	3	1	0	6	1	2	1
18879	202	0	5-Apr-08	22	2	16	2	1	1	0	6	1	3	2
18880	202	0	24-Mar-07	18	2	16	2	2	1	0	13	1	1	1
18881	101	1	27-Jul-16	18	1	16	2	1	1	0	11	1	2	1
18882	201	1	20-Apr-17	11	1	16	2	3	1	0	4	1	2	2
18883	201	1	19-Aug-12	13	1	16	2	2	1	0	6	1	2	1
18884	201	1	6-Sep-12	17	1	16	2	3	1	0	6	1	4	1
18885	201	1	27-May-16	8	1	16	2	2	1	0	6	1	2	1
18886	201	1	30-Apr-13	10	1	16	2	1	1	0	6	1	3	1
18887	201	1	12-Apr-13	18	1	16	2	3	1	0	6	1	2	1
18888	101	1	20-May-16	19	1	16	2	1	1	0	6	1	2	1
18889	201	1	3-Aug-16	2	1	16	2	2	1	0	6	1	2	1
18890	201	1	21-Mar-15	2	1	16	2	2	1	0	6	1	2	1
18891	201	1	3-Sep-17	13	1	16	2	1	1	0	6	1	2	1
18892	101	1	5-Sep-14	20	1	16	2	3	1	0	7	1	3	1
18893	201	1	10-Aug-17	10	1	16	2	3	1	0	4	1	2	1
18894	201	1	29-Aug-15	9	1	16	2	1	1	0	4	1	2	1
18895	101	1	20-May-16	20	1	16	2	1	1	0	6	1	2	1
18896	201	1	11-May-15	18	1	16	2	1	1	0	6	1	2	2
18897	201	1	21-Sep-15	12	1	16	2	3	1	0	6	1	2	1
18898	201	1	28-Nov-16	13	1	16	2	3	1	0	6	1	2	1
18899	101	1	21-Jun-12	20	1	16	2	1	1	0	6	1	3	2
18900	201	1	21-Oct-15	0	1	16	2	2	1	0	6	1	3	1
18901	201	1	8-Sep-15	20	1	16	2	1	1	0	6	1	2	1
18902	201	1	28-Jul-17	22	1	16	2	1	1	0	6	1	2	1
18903	201	1	28-Feb-08	22	1	16	2	1	1	0	10	1	7	1
18904	101	1	14-Jul-16	19	1	16	2	1	1	0	7	1	2	1
18905	201	1	4-Oct-16	15	1	16	2	3	1	0	7	1	3	1
18906	101	1	28-Jun-16	17	1	16	2	1	1	0	7	1	3	1
18907	101	1	7-Apr-16	16	1	16	2	1	1	0	7	1	3	1
18908	101	1	29-Aug-14	20	1	16	2	1	1	0	7	1	2	1
18909	201	1	5-Jan-14	16	1	16	2	2	1	0	7	1	2	1
18910	101	1	14-Nov-14	22	1	16	2	1	1	0	7	1	2	1
18911	101	1	11-Jun-13	12	1	16	2	1	1	0	7	1	2	1
18912	201	1	15-Nov-13	19	1	16	2	3	1	0	7	1	2	1
18913	201	1	24-Sep-13	11	1	16	2	2	1	0	7	1	3	1
18914	201	1	10-Sep-11	16	1	16	2	1	1	0	7	1	3	1
18915	101	1	1-Sep-11	11	1	16	2	1	1	0	7	1	2	1
18916	101	1	3-Aug-17	10	1	16	2	1	1	0	7	1	3	1
18917	101	1	7-Aug-17	15	1	16	2	1	1	0	7	1	3	1
18918	201	1	10-Sep-12	10	1	16	2	3	1	0	13	1	1	1
18919	201	1	17-Aug-10	16	1	16	2	2	1	0	1	1	2	1
18920	101	1	27-Jul-15	22	1	16	2	1	1	0	4	1	1	1
18921	201	1	12-Mar-15	19	1	16	2	1	1	0	4	1	2	1
18922	101	1	14-May-12	18	1	16	2	1	1	0	4	1	2	1
18923	101	1	20-May-11	23	1	16	2	1	1	0	4	1	1	1
18924	101	1	28-Sep-11	20	1	16	2	1	1	0	4	1	3	1
18925	201	1	2-Feb-09	17	1	16	2	2	1	0	4	1	2	2
18926	101	1	14-Jun-09	8	1	16	2	1	1	0	4	1	4	1
18927	101	1	17-Nov-09	22	1	16	2	1	1	0	4	1	2	1
18928	201	1	12-Aug-08	12	1	16	2	1	1	0	4	1	1	1
18929	201	1	24-Jul-08	7	1	16	2	2	1	0	4	1	2	1
18930	201	1	16-Jan-08	19	1	16	2	3	1	0	4	1	2	1
18931	101	1	25-Aug-17	19	1	16	2	1	1	0	4	1	2	1
18932	201	1	19-Dec-16	20	1	16	2	3	1	0	11	1	3	1
18933	201	1	13-Nov-16	16	1	16	2	2	1	0	11	1	1	1
18934	201	1	14-Jul-16	12	1	16	2	1	1	0	11	1	2	1
18935	201	1	5-Apr-16	11	1	16	2	2	1	0	11	1	2	1
18936	201	1	10-Jan-16	11	1	16	2	3	1	0	11	1	2	1
18937	201	1	17-Jan-16	0	1	16	2	1	1	0	11	1	3	1
18938	101	1	9-Dec-16	17	1	16	2	1	1	0	11	1	2	1
18939	201	1	15-Dec-16	17	1	16	2	2	1	0	11	1	3	1
18940	101	1	14-Jan-16	13	1	16	2	1	1	0	11	1	2	1
18941	101	1	28-May-16	10	1	16	2	1	1	0	11	1	2	2
18942	101	1	1-Aug-16	9	1	16	2	1	1	0	11	1	2	1
18943	101	1	2-Aug-16	19	1	16	2	1	1	0	11	1	2	1
18944	101	1	6-Nov-16	21	1	16	2	1	1	0	11	1	3	1
18945	101	1	4-Jul-16	8	1	16	2	1	1	0	11	1	2	1
18946	101	1	27-Jan-16	11	1	16	2	1	1	0	11	1	2	1
18947	201	1	19-Nov-16	14	1	16	2	1	1	0	11	1	2	1
18948	101	1	30-Jun-16	14	1	16	2	1	1	0	11	1	2	1
18949	101	1	28-Jul-16	18	1	16	2	3	1	0	11	1	2	1
18950	201	1	1-May-16	10	1	16	2	3	1	0	11	1	2	1
18951	201	1	11-Nov-16	11	1	16	2	2	1	0	11	1	3	1
18952	101	1	4-Jun-16	15	1	16	2	1	1	0	11	1	3	1
18953	101	1	30-Mar-16	21	1	16	2	1	1	0	11	1	3	1
18954	101	1	5-Aug-16	7	1	16	2	1	1	0	11	1	3	1
18955	101	1	18-Mar-15	14	1	16	2	1	1	0	11	1	2	1
18956	101	1	18-Mar-15	21	1	16	2	1	1	0	11	1	2	1
18957	201	1	5-May-15	14	1	16	2	2	1	0	11	1	3	1
18958	201	1	12-Jul-15	7	1	16	2	1	1	0	11	1	3	1
18959	101	1	1-Oct-15	13	1	16	2	1	1	0	11	1	2	1
18960	201	1	23-May-15	18	1	16	2	3	1	0	11	1	2	1
18961	101	1	8-Apr-15	13	1	16	2	1	1	0	11	1	2	1
18962	201	1	3-May-15	10	1	16	2	2	1	0	11	1	2	1
18963	101	1	7-Oct-15	14	1	16	2	1	1	0	11	1	3	1
18964	201	1	14-Jul-14	19	1	16	2	1	1	0	11	1	2	1
18965	101	1	6-Feb-14	15	1	16	2	1	1	0	11	1	2	1

18966	101	1	27-Jan-14	8	1	16	2	1	1	0	11	1	2	1
18967	101	1	29-Sep-14	18	1	16	2	1	1	0	11	1	3	1
18968	101	1	28-Oct-14	12	1	16	2	1	1	0	11	1	2	1
18969	201	1	7-Feb-14	12	1	16	2	3	1	0	11	1	2	1
18970	101	1	8-May-14	20	1	16	2	1	1	0	11	1	2	1
18971	201	1	6-Jan-14	13	1	16	2	3	1	0	11	1	2	1
18972	101	1	30-Sep-14	14	1	16	2	1	1	0	11	1	2	1
18973	201	1	27-May-13	10	1	16	2	2	1	0	11	1	2	1
18974	201	1	21-Sep-13	16	1	16	2	2	1	0	11	1	2	1
18975	101	1	25-Jul-13	13	1	16	2	1	1	0	11	1	2	1
18976	101	1	12-Sep-13	19	1	16	2	1	1	0	11	1	2	1
18977	101	1	23-Oct-13	21	1	16	2	1	1	0	11	1	3	1
18978	101	1	24-Sep-13	11	1	16	2	1	1	0	11	1	2	1
18979	101	1	3-May-13	12	1	16	2	1	1	0	11	1	2	1
18980	101	1	22-Dec-13	12	1	16	2	1	1	0	11	1	2	1
18981	201	1	22-Apr-13	20	1	16	2	1	1	0	11	1	3	1
18982	201	1	25-Nov-13	19	1	16	2	1	1	0	11	1	2	1
18983	201	1	5-Feb-13	21	1	16	2	1	1	0	11	1	2	1
18984	101	1	18-Aug-13	7	1	16	2	3	1	0	11	1	2	1
18985	201	1	8-Apr-12	17	1	16	2	3	1	0	11	1	3	1
18986	101	1	1-May-12	10	1	16	2	1	1	0	11	1	3	1
18987	101	1	9-Oct-12	21	1	16	2	1	1	0	11	1	3	1
18988	201	1	25-Feb-12	10	1	16	2	2	1	0	11	1	2	1
18989	101	1	7-Oct-12	20	1	16	2	1	1	0	11	1	2	1
18990	201	1	17-Nov-12	12	1	16	2	2	1	0	11	1	3	1
18991	101	1	7-Apr-12	18	1	16	2	1	1	0	11	1	3	1
18992	201	1	19-Mar-11	6	1	16	2	1	1	0	11	1	2	1
18993	101	1	22-Dec-11	17	1	16	2	1	1	0	11	1	5	1
18994	101	1	13-Mar-11	21	1	16	2	1	1	0	11	1	2	1
18995	101	1	10-Sep-11	13	1	16	2	1	1	0	11	1	2	1
18996	201	1	16-Sep-11	10	1	16	2	3	1	0	11	1	2	1
18997	201	1	7-Dec-11	5	1	16	2	1	1	0	11	1	2	1
18998	201	1	20-Jul-11	10	1	16	2	2	1	0	11	1	3	1
18999	201	1	16-Jul-11	23	1	16	2	1	1	0	11	1	2	1
19000	101	1	29-Nov-11	19	1	16	2	1	1	0	11	1	2	1
19001	201	1	9-Jun-11	8	1	16	2	2	1	0	11	1	3	1
19002	101	1	17-Feb-11	20	1	16	2	1	1	0	11	1	2	1
19003	101	1	10-Nov-11	17	1	16	2	1	1	0	11	1	2	1
19004	101	1	28-Dec-11	11	1	16	2	1	1	0	11	1	2	1
19005	201	1	16-Jun-10	11	1	16	2	2	1	0	11	1	2	1
19006	101	1	3-Jun-10	18	1	16	2	1	1	0	11	1	2	1
19007	101	1	7-Dec-10	20	1	16	2	1	1	0	11	1	3	1
19008	101	1	6-Feb-10	6	1	16	2	1	1	0	11	1	3	1
19009	101	1	20-Mar-10	21	1	16	2	1	1	0	11	1	2	1
19010	101	1	1-Oct-10	20	1	16	2	2	1	0	11	1	3	1
19011	101	1	21-Apr-09	16	1	16	2	1	1	0	11	1	2	1
19012	201	1	20-Jul-09	20	1	16	2	3	1	0	11	1	2	1
19013	101	1	19-Jun-09	20	1	16	2	1	1	0	11	1	3	1
19014	101	1	2-Aug-09	12	1	16	2	1	1	0	11	1	2	1
19015	101	1	27-Jun-09	17	1	16	2	1	1	0	11	1	1	1
19016	201	1	19-Mar-08	16	1	16	2	3	1	0	11	1	3	1
19017	101	1	20-Oct-08	19	1	16	2	1	1	0	11	1	2	1
19018	201	1	30-Jun-07	19	1	16	2	1	1	0	11	1	3	1
19019	101	1	22-Feb-07	16	1	16	2	1	1	0	11	1	2	1
19020	101	1	5-Jun-06	12	1	16	2	1	1	0	11	1	2	2
19021	201	1	16-Apr-06	10	1	16	2	3	1	0	11	1	2	1
19022	201	1	17-Sep-06	9	1	16	2	1	1	0	11	1	2	1
19023	101	1	7-Mar-17	18	1	16	2	1	1	0	11	1	3	1
19024	201	1	15-Jan-17	18	1	16	2	1	1	0	11	1	3	1
19025	201	1	6-Feb-17	10	1	16	2	1	1	0	11	1	2	1
19026	101	1	13-Sep-17	10	1	16	2	1	1	0	11	1	1	1
19027	201	1	22-Nov-17	0	1	16	2	2	1	0	11	1	3	1
19028	101	1	7-Dec-17	23	1	16	2	2	1	0	11	1	2	1
19029	201	1	7-Sep-17	2	1	16	2	2	1	0	11	1	3	1
19030	201	1	27-Jul-17	18	1	16	2	2	1	0	11	1	2	1
19031	201	1	2-Jan-16	5	1	16	2	3	1	0	3	1	1	1
19032	101	1	12-May-16	23	1	16	2	1	1	0	3	1	4	1
19033	201	1	23-Apr-16	20	1	16	2	3	1	0	3	1	3	1
19034	201	1	18-May-16	6	1	16	2	2	1	0	3	1	2	1
19035	101	1	10-Aug-16	15	1	16	2	1	1	0	3	1	4	2
19036	201	1	23-Apr-15	11	1	16	2	2	1	0	3	1	3	1
19037	201	1	4-Sep-15	10	1	16	2	3	1	0	3	1	2	2
19038	201	1	1-Dec-15	19	1	16	2	2	1	0	3	1	3	1
19039	201	1	26-Oct-15	15	1	16	2	2	1	0	3	1	3	1
19040	101	1	2-May-15	21	1	16	2	1	1	0	3	1	3	1
19041	201	1	27-Nov-15	22	1	16	2	2	1	0	3	1	3	1
19042	201	1	24-Oct-15	0	1	16	2	2	1	0	3	1	2	1
19043	101	1	28-Dec-15	22	1	16	2	1	1	0	3	1	2	1
19044	201	1	5-May-15	19	1	16	2	3	1	0	3	1	3	1
19045	201	1	26-Aug-14	8	1	16	2	1	1	0	3	1	3	1
19046	101	1	11-Apr-14	12	1	16	2	1	1	0	3	1	2	1
19047	201	1	14-Aug-14	7	1	16	2	2	1	0	3	1	3	1
19048	201	1	3-Mar-14	16	1	16	2	3	1	0	3	1	3	1
19049	101	1	15-Jul-14	2	1	16	2	3	1	0	3	1	2	4
19050	201	1	6-Feb-13	22	1	16	2	2	1	0	3	1	3	1
19051	201	1	1-Oct-13	9	1	16	2	3	1	0	3	1	2	1
19052	101	1	25-Apr-12	21	1	16	2	1	1	0	3	1	1	1
19053	201	1	3-Feb-12	19	1	16	2	1	1	0	3	1	1	1
19054	201	1	8-Aug-12	10	1	16	2	3	1	0	3	1	1	1
19055	101	1	25-Nov-12	8	1	16	2	1	1	0	3	1	1	1
19056	101	1	6-Jun-12	21	1	16	2	1	1	0	3	1	1	1
19057	101	1	3-Jul-12	19	1	16	2	3	1	0	3	1	4	1
19058	101	1	8-Mar-12	20	1	16	2	1	1	0	3	1	2	1
19059	201	1	13-Mar-11	23	1	16	2	1	1	0	3	1	1	1
19060	201	1	26-Jul-11	7	1	16	2	1	1	0	3	1	3	1
19061	201	1	4-Mar-11	20	1	16	2	1	1	0	3	1	3	1
19062	201	1	7-Oct-11	23	1	16	2	1	1	0	3	1	3	2
19063	201	1	12-Sep-11	10	1	16	2	3	1	0	3	1	3	1
19064	201	1	17-Jul-11	9	1	16	2	1	1	0	3	1	3	1
19065	201	1	23-May-10	16	1	16	2	2	1	0	3	1	2	1
19066	201	1	22-Jan-10	19	1	16	2	1	1	0	3	1	1	1
19067	101	1	24-Aug-10	1	1	16	2	1	1	0	3	1	1	1
19068	101	1	30-Jan-10	22	1	16	2	1	1	0	3	1	3	1
19069	101	1	21-Jul-09	20	1	16	2	1	1	0	3	1	5	1
19070	201	1	10-Apr-09	22	1	16	2	2	1	0	3	1	1	2
19071	101	1	9-Aug-09	3	1	16	2	1	1	0	3	1	1	2
19072	201	1	25-Jan-09	21	1	16	2	2	1	0	3	1	3	1
19073	101	1	7-Sep-09	19	1	16	2	1	1	0	3	1	3	1
19074	201	1	13-Sep-08	1	1	16	2	2	1	0	3	1	6	1
19075	201	1	25-Oct-08	20	1	16	2	3	1	0	3	1	3	1
19076	201	1	11-Apr-08	21	1	16	2	2	1	0	3	1	2	1
19077	201	1	29-Dec-08	22	1	16	2	3	1	0	3	1	3	1
19078	201	1	26-Sep-07	19	1	16	2	3	1	0	3	1	1	1
19079	201	1	3-Sep-07	18	1	16	2	1	1	0	3	1	3	1
19080	101	1	2-Aug-06	22	1	16	2	2	1	0	3	1	3	1
19081	201	1	26-Oct-06	22	1	16	2	1	1	0	3	1	1	1
19082	201	1	16-Sep-06	20	1	16	2	1	1	0	3	1	1	1
19083	101	1	15-Jun-16	12	1	16	2	1	1	0	6	1	2	1
19084	101	1	24-Feb-15	11	1	16	2	1	1	0	6	1	2	1
19085	101	1	24-Feb-15	19	1	16	2	1	1	0	6	1	3	1
19086	101	1	23-Oct-15	6	1	16	2	1	1	0	6	1	3	1
19087	101	1	25-May-15	11	1	16	2	1	1	0	6	1	2	1
19088	101	1	30-Sep-15	22	1	16	2	1	1	0	6	1	2	1
19089	101	1	25-Nov-15	19	1	16	2	1	1	0	6	1	2	1

19090	101	1	24-May-15	15	1	16	2	1	1	0	6	1	2	1
19091	201	1	13-Nov-15	12	1	16	2	2	1	0	6	1	2	1
19092	101	1	25-May-15	19	1	16	2	1	1	0	6	1	2	1
19093	201	1	1-Dec-15	16	1	16	2	1	1	0	6	1	2	1
19094	201	1	1-Jun-15	19	1	16	2	3	1	0	6	1	2	1
19095	101	1	5-Dec-15	11	1	16	2	1	1	0	6	1	2	4
19096	101	1	9-May-14	22	1	16	2	1	1	0	6	1	2	1
19097	201	1	20-Nov-14	9	1	16	2	3	1	0	6	1	2	1
19098	101	1	10-May-14	18	1	16	2	1	1	0	6	1	2	1
19099	101	1	19-Jun-14	7	1	16	2	1	1	0	6	1	2	4
19100	201	1	16-May-14	13	1	16	2	2	1	0	6	1	2	1
19101	101	1	16-Jul-14	16	1	16	2	1	1	0	6	1	2	1
19102	101	1	24-Aug-14	16	1	16	2	1	1	0	6	1	2	1
19103	101	1	2-Sep-14	15	1	16	2	1	1	0	6	1	2	1
19104	101	1	18-Feb-14	18	1	16	2	1	1	0	6	1	2	1
19105	101	1	10-Jan-14	17	1	16	2	1	1	0	6	1	2	1
19106	101	1	19-Mar-14	18	1	16	2	1	1	0	6	1	2	1
19107	201	1	20-May-13	20	1	16	2	2	1	0	6	1	3	1
19108	101	1	22-Oct-13	17	1	16	2	1	1	0	6	1	2	1
19109	101	1	1-Apr-13	23	1	16	2	1	1	0	6	1	2	1
19110	101	1	13-Apr-13	10	1	16	2	1	1	0	6	1	2	1
19111	101	1	17-Mar-13	13	1	16	2	2	1	0	6	1	2	1
19112	101	1	22-Jun-13	23	1	16	2	1	1	0	6	1	5	1
19113	101	1	31-Aug-13	19	1	16	2	1	1	0	6	1	2	1
19114	101	1	18-Sep-13	22	1	16	2	1	1	0	6	1	3	1
19115	101	1	14-Aug-12	8	1	16	2	3	1	0	6	1	2	4
19116	101	1	5-Sep-12	15	1	16	2	1	1	0	6	1	2	4
19117	101	1	2-Apr-12	0	1	16	2	1	1	0	6	1	3	1
19118	101	1	20-May-11	18	1	16	2	1	1	0	6	1	2	1
19119	101	1	25-Jun-11	13	1	16	2	1	1	0	6	1	2	1
19120	101	1	3-Oct-11	12	1	16	2	1	1	0	6	1	2	1
19121	201	1	12-Aug-11	9	1	16	2	2	1	0	6	1	2	1
19122	101	1	12-Jun-10	18	1	16	2	1	1	0	6	1	2	1
19123	201	1	5-Nov-10	19	1	16	2	3	1	0	6	1	2	1
19124	101	1	20-Jul-10	0	1	16	2	1	1	0	6	1	3	1
19125	101	1	1-Dec-10	15	1	16	2	1	1	0	6	1	2	1
19126	101	1	20-Jul-10	13	1	16	2	1	1	0	6	1	2	4
19127	101	1	5-Aug-10	23	1	16	2	1	1	0	6	1	3	1
19128	101	1	10-Mar-10	17	1	16	2	2	1	0	6	1	2	1
19129	101	1	7-May-10	19	1	16	2	1	1	0	6	1	2	1
19130	201	1	16-Jul-09	14	1	16	2	1	1	0	6	1	2	1
19131	201	1	1-Aug-09	1	1	16	2	3	1	0	6	1	2	1
19132	201	1	20-Nov-08	18	1	16	2	3	1	0	6	1	2	1
19133	201	1	30-Jul-08	20	1	16	2	3	1	0	6	1	2	1
19134	101	1	18-Sep-08	19	1	16	2	1	1	0	6	1	2	1
19135	201	1	5-Feb-07	22	1	16	2	1	1	0	6	1	2	1
19136	101	1	15-Oct-07	17	1	16	2	1	1	0	6	1	2	1
19137	101	1	13-Jan-07	17	1	16	2	3	1	0	6	1	2	1
19138	101	1	4-Aug-07	6	1	16	2	1	1	0	6	1	3	1
19139	101	1	5-Sep-07	10	1	16	2	1	1	0	6	1	3	1
19140	201	1	18-Jun-06	3	1	16	2	3	1	0	6	1	2	1
19141	201	1	6-Aug-06	21	1	16	2	1	1	0	6	1	2	1
19142	201	1	31-Jan-06	14	1	16	2	1	1	0	6	1	3	1
19143	201	1	7-May-06	20	1	16	2	1	1	0	6	1	2	1
19144	101	1	23-Aug-17	15	1	16	2	1	1	0	6	1	3	1
19145	101	1	7-Aug-17	15	1	16	2	1	1	0	6	1	2	1
19146	101	1	31-Oct-17	17	1	16	2	1	1	0	6	1	2	1
19147	101	1	28-Sep-17	14	1	16	2	1	1	0	6	1	2	1
19148	101	1	21-Apr-17	11	1	16	2	1	1	0	6	1	3	1
19149	201	1	19-Jun-09	18	1	16	2	1	1	0	13	1	2	1
19150	101	1	10-Sep-06	19	1	16	2	1	1	0	13	1	7	1
19151	201	1	9-May-06	15	1	16	2	2	1	0	13	1	3	2
19152	201	1	10-Oct-16	18	2	16	2	2	1	0	6	1	2	1
19153	201	1	5-Sep-15	6	2	16	2	1	1	0	6	1	2	1
19154	201	1	14-Jun-14	15	2	16	2	1	1	0	4	1	1	1
19155	201	1	5-Aug-16	16	2	16	2	1	1	0	4	1	3	1
19156	101	1	13-Aug-17	21	2	16	2	1	1	0	11	1	2	1
19157	201	1	7-May-17	6	2	16	2	3	1	0	6	1	3	1
19158	201	1	29-Nov-17	20	2	16	2	1	1	0	6	1	2	1
19159	201	1	18-Apr-15	5	2	16	2	2	1	0	6	1	2	1
19160	201	1	7-Jun-13	5	2	16	2	2	1	0	6	1	3	1
19161	201	1	6-Feb-08	20	2	16	2	3	1	0	10	1	7	2
19162	201	1	6-Jan-08	20	2	16	2	1	1	0	10	1	1	2
19163	201	1	28-Apr-16	17	2	16	2	2	1	0	7	1	4	1
19164	201	1	10-Oct-14	16	2	16	2	2	1	0	7	1	3	1
19165	201	1	7-Oct-14	20	2	16	2	2	1	0	7	1	3	1
19166	101	1	14-May-13	17	2	16	2	1	1	0	7	1	4	1
19167	201	1	29-Apr-12	19	2	16	2	2	1	0	7	1	2	1
19168	101	1	29-Feb-12	17	2	16	2	1	1	0	7	1	2	1
19169	101	1	1-Feb-11	23	2	16	2	1	1	0	7	1	3	1
19170	101	1	23-Aug-08	15	2	16	2	1	1	0	7	1	2	1
19171	101	1	24-Jul-06	20	2	16	2	3	1	0	7	1	2	1
19172	201	1	4-Aug-09	2	2	16	2	1	1	0	5	1	7	2
19173	101	1	26-Jan-14	19	2	16	2	1	1	0	4	1	3	1
19174	201	1	4-Aug-13	13	2	16	2	1	1	0	4	1	2	1
19175	101	1	2-Jan-12	2	2	16	2	1	1	0	4	1	1	1
19176	201	1	24-Aug-11	4	2	16	2	2	1	0	4	1	2	1
19177	101	1	5-Oct-10	21	2	16	2	1	1	0	4	1	2	1
19178	201	1	7-Aug-08	7	2	16	2	1	1	0	4	1	3	1
19179	201	1	20-May-07	20	2	16	2	1	1	0	4	1	2	1
19180	101	1	28-Aug-17	22	2	16	2	1	1	0	4	1	1	1
19181	201	1	7-Jul-16	7	2	16	2	1	1	0	11	1	2	1
19182	101	1	6-Aug-16	7	2	16	2	1	1	0	11	1	2	1
19183	201	1	24-Sep-16	13	2	16	2	1	1	0	11	1	2	1
19184	101	1	3-Nov-16	23	2	16	2	1	1	0	11	1	2	1
19185	101	1	8-Aug-16	21	2	16	2	1	1	0	11	1	2	1
19186	201	1	13-Feb-16	11	2	16	2	3	1	0	11	1	2	1
19187	201	1	31-Oct-16	19	2	16	2	2	1	0	11	1	2	1
19188	101	1	12-Nov-16	17	2	16	2	1	1	0	11	1	2	1
19189	101	1	25-Jun-16	18	2	16	2	1	1	0	11	1	2	1
19190	201	1	17-May-16	20	2	16	2	2	1	0	11	1	2	1
19191	201	1	22-Jul-16	8	2	16	2	2	1	0	11	1	3	1
19192	101	1	4-Oct-16	21	2	16	2	1	1	0	11	1	3	1
19193	101	1	15-Jun-16	18	2	16	2	1	1	0	11	1	3	1
19194	101	1	25-Dec-16	20	2	16	2	1	1	0	11	1	2	1
19195	101	1	6-Feb-16	9	2	16	2	1	1	0	11	1	2	1
19196	101	1	31-Mar-15	19	2	16	2	1	1	0	11	1	2	1
19197	201	1	4-Sep-15	13	2	16	2	2	1	0	11	1	3	1
19198	101	1	3-Feb-15	20	2	16	2	1	1	0	11	1	3	1
19199	201	1	26-Jan-15	0	2	16	2	3	1	0	11	1	2	1
19200	101	1	30-Jan-15	20	2	16	2	1	1	0	11	1	2	1
19201	101	1	20-Mar-15	11	2	16	2	1	1	0	11	1	2	1
19202	101	1	13-Mar-15	15	2	16	2	1	1	0	11	1	2	1
19203	101	1	12-Aug-15	12	2	16	2	1	1	0	11	1	2	1
19204	201	1	29-Jan-15	17	2	16	2	2	1	0	11	1	2	1
19205	101	1	19-Aug-15	19	2	16	2	1	1	0	11	1	2	1
19206	101	1	9-May-15	16	2	16	2	1	1	0	11	1	2	1
19207	101	1	27-Oct-14	20	2	16	2	1	1	0	11	1	2	1
19208	201	1	1-Feb-14	20	2	16	2	1	1	0	11	1	3	1
19209	101	1	17-Dec-14	16	2	16	2	1	1	0	11	1	2	1
19210	101	1	22-Dec-14	19	2	16	2	1	1	0	11	1	2	1
19211	101	1	3-Mar-14	20	2	16	2	1	1	0	11	1	2	1
19212	101	1	2-Jul-14	12	2	16	2	1	1	0	11	1	2	1
19213	101	1	30-Oct-13	10	2	16	2	1	1	0	11	1	2	1

19214	201	1	31-May-13	22	2	16	2	3	1	0	11	1	3	1
19215	201	1	23-Jul-13	21	2	16	2	3	1	0	11	1	2	1
19216	101	1	7-Jul-13	11	2	16	2	1	1	0	11	1	3	1
19217	101	1	30-May-13	20	2	16	2	1	1	0	11	1	3	1
19218	101	1	6-Jun-13	20	2	16	2	1	1	0	11	1	2	1
19219	101	1	9-Jul-13	18	2	16	2	1	1	0	11	1	2	1
19220	101	1	21-Jun-13	7	2	16	2	1	1	0	11	1	2	1
19221	101	1	22-Aug-13	8	2	16	2	3	1	0	11	1	3	1
19222	101	1	18-Aug-12	22	2	16	2	1	1	0	11	1	3	1
19223	201	1	13-Jun-12	6	2	16	2	2	1	0	11	1	3	1
19224	101	1	6-Jan-12	8	2	16	2	1	1	0	11	1	2	1
19225	101	1	22-Dec-12	17	2	16	2	1	1	0	11	1	2	1
19226	101	1	28-Jan-12	7	2	16	2	1	1	0	11	1	2	1
19227	101	1	23-Apr-12	7	2	16	2	1	1	0	11	1	2	1
19228	201	1	7-Aug-12	20	2	16	2	2	1	0	11	1	3	1
19229	101	1	5-Jun-12	10	2	16	2	1	1	0	11	1	3	1
19230	101	1	2-May-12	17	2	16	2	1	1	0	11	1	3	1
19231	201	1	9-May-11	21	2	16	2	1	1	0	11	1	2	1
19232	201	1	20-Apr-11	20	2	16	2	3	1	0	11	1	2	1
19233	101	1	13-Mar-11	16	2	16	2	1	1	0	11	1	2	1
19234	101	1	6-Jan-11	19	2	16	2	3	1	0	11	1	3	1
19235	101	1	28-Aug-11	1	2	16	2	3	1	0	11	1	3	1
19236	101	1	1-Oct-11	23	2	16	2	1	1	0	11	1	3	1
19237	101	1	10-Mar-11	14	2	16	2	1	1	0	11	1	2	1
19238	101	1	10-Mar-11	11	2	16	2	1	1	0	11	1	3	1
19239	101	1	22-Aug-11	14	2	16	2	1	1	0	11	1	2	1
19240	101	1	7-Aug-11	19	2	16	2	1	1	0	11	1	3	1
19241	101	1	11-Jul-11	3	2	16	2	2	1	0	11	1	3	1
19242	101	1	1-Jan-10	14	2	16	2	1	1	0	11	1	2	1
19243	201	1	16-Jan-10	21	2	16	2	1	1	0	11	1	2	1
19244	101	1	11-Aug-10	19	2	16	2	3	1	0	11	1	3	1
19245	101	1	13-Oct-09	19	2	16	2	1	1	0	11	1	1	1
19246	201	1	11-Aug-09	12	2	16	2	1	1	0	11	1	2	1
19247	101	1	22-Jul-09	1	2	16	2	1	1	0	11	1	3	1
19248	201	1	19-Nov-09	15	2	16	2	1	1	0	11	1	2	1
19249	101	1	4-Sep-09	22	2	16	2	1	1	0	11	1	2	1
19250	101	1	13-May-09	19	2	16	2	2	1	0	11	1	2	1
19251	101	1	18-Jul-09	10	2	16	2	1	1	0	11	1	3	1
19252	201	1	1-Jun-08	12	2	16	2	2	1	0	11	1	2	1
19253	201	1	8-Oct-08	11	2	16	2	1	1	0	11	1	3	1
19254	201	1	16-Dec-07	11	2	16	2	2	1	0	11	1	3	1
19255	201	1	11-Feb-07	23	2	16	2	2	1	0	11	1	2	1
19256	101	1	17-Nov-07	23	2	16	2	1	1	0	11	1	2	1
19257	101	1	7-Oct-07	0	2	16	2	1	1	0	11	1	3	1
19258	101	1	7-Oct-06	21	2	16	2	1	1	0	11	1	2	1
19259	201	1	17-Sep-06	19	2	16	2	1	1	0	11	1	2	1
19260	101	1	2-Aug-17	15	2	16	2	1	1	0	11	1	4	1
19261	101	1	11-Feb-17	1	2	16	2	1	1	0	11	1	3	1
19262	101	1	3-Aug-17	9	2	16	2	1	1	0	11	1	3	1
19263	101	1	7-Aug-17	20	2	16	2	1	1	0	11	1	3	1
19264	201	1	28-Jul-17	12	2	16	2	1	1	0	11	1	3	1
19265	101	1	4-Jun-17	18	2	16	2	1	1	0	11	1	2	1
19266	101	1	1-Apr-17	13	2	16	2	1	1	0	11	1	2	1
19267	101	1	23-Mar-17	18	2	16	2	1	1	0	11	1	2	1
19268	101	1	24-Mar-17	13	2	16	2	1	1	0	11	1	2	1
19269	101	1	27-Mar-17	9	2	16	2	1	1	0	11	1	2	1
19270	101	1	10-Jul-17	13	2	16	2	1	1	0	11	1	2	1
19271	201	1	13-Jun-17	20	2	16	2	1	1	0	11	1	2	1
19272	201	1	28-Apr-17	20	2	16	2	1	1	0	11	1	2	1
19273	201	1	15-Aug-17	23	2	16	2	2	1	0	11	1	3	1
19274	201	1	21-Oct-17	8	2	16	2	2	1	0	11	1	2	1
19275	101	1	31-Oct-17	13	2	16	2	1	1	0	11	1	3	1
19276	101	1	15-Oct-17	17	2	16	2	1	1	0	11	1	2	1
19277	101	1	31-Oct-17	22	2	16	2	1	1	0	11	1	2	1
19278	201	1	9-Nov-17	12	2	16	2	2	1	0	11	1	3	1
19279	201	1	27-May-17	19	2	16	2	3	1	0	11	1	3	1
19280	201	1	10-Feb-17	19	2	16	2	3	1	0	11	1	2	1
19281	101	1	15-Jul-16	20	2	16	2	1	1	0	3	1	1	1
19282	101	1	10-Dec-15	17	2	16	2	1	1	0	3	1	4	1
19283	101	1	8-May-15	22	2	16	2	1	1	0	3	1	3	1
19284	101	1	13-Apr-15	22	2	16	2	1	1	0	3	1	2	1
19285	201	1	30-Mar-14	15	2	16	2	3	1	0	3	1	1	1
19286	101	1	15-Jan-14	12	2	16	2	1	1	0	3	1	1	1
19287	101	1	2-Mar-14	0	2	16	2	3	1	0	3	1	3	4
19288	101	1	8-Jun-13	7	2	16	2	1	1	0	3	1	1	1
19289	101	1	9-Dec-13	8	2	16	2	2	1	0	3	1	1	1
19290	201	1	11-Mar-13	0	2	16	2	1	1	0	3	1	2	1
19291	201	1	23-Jul-13	15	2	16	2	1	1	0	3	1	2	1
19292	101	1	12-Jun-12	22	2	16	2	1	1	0	3	1	1	2
19293	101	1	28-May-12	16	2	16	2	1	1	0	3	1	4	1
19294	201	1	22-Aug-12	19	2	16	2	3	1	0	3	1	3	1
19295	101	1	12-Jul-11	15	2	16	2	1	1	0	3	1	1	1
19296	201	1	18-Nov-11	0	2	16	2	1	1	0	3	1	2	1
19297	101	1	10-Nov-10	1	2	16	2	1	1	0	3	1	1	1
19298	101	1	7-Sep-10	20	2	16	2	1	1	0	3	1	3	1
19299	201	1	1-Jan-09	17	2	16	2	3	1	0	3	1	3	1
19300	101	1	16-Mar-09	21	2	16	2	1	1	0	3	1	1	4
19301	101	1	26-Oct-09	23	2	16	2	1	1	0	3	1	1	1
19302	201	1	1-Aug-09	19	2	16	2	2	1	0	3	1	1	1
19303	201	1	16-Nov-08	1	2	16	2	3	1	0	3	1	1	2
19304	101	1	17-Dec-08	23	2	16	2	1	1	0	3	1	1	1
19305	101	1	7-Jun-08	9	2	16	2	1	1	0	3	1	3	1
19306	101	1	5-Feb-07	18	2	16	2	2	1	0	3	1	1	1
19307	101	1	16-Aug-07	15	2	16	2	4	1	0	3	1	3	1
19308	101	1	14-Nov-16	11	2	16	2	1	1	0	3	1	4	1
19309	101	1	12-Mar-17	23	2	16	2	1	1	0	3	1	2	1
19310	101	1	13-Jun-16	18	2	16	2	1	1	0	6	1	2	1
19311	101	1	17-Aug-15	17	2	16	2	1	1	0	6	1	3	1
19312	101	1	11-Aug-15	19	2	16	2	1	1	0	6	1	2	1
19313	101	1	12-Nov-14	17	2	16	2	1	1	0	6	1	2	1
19314	101	1	25-Sep-14	12	2	16	2	1	1	0	6	1	3	1
19315	201	1	14-Dec-13	19	2	16	2	3	1	0	6	1	2	1
19316	101	1	16-Jul-11	23	2	16	2	1	1	0	6	1	2	1
19317	201	1	12-Dec-10	22	2	16	2	1	1	0	6	1	3	1
19318	101	1	10-Jun-09	20	2	16	2	1	1	0	6	1	2	1
19319	201	1	15-Jul-08	8	2	16	2	2	1	0	6	1	2	1
19320	101	1	10-Jul-07	20	2	16	2	1	1	0	6	1	3	1
19321	101	1	8-May-06	21	2	16	2	1	1	0	6	1	2	1
19322	201	1	20-Aug-06	12	2	16	2	2	1	0	6	1	2	1
19323	101	1	8-Apr-17	16	2	16	2	1	1	0	6	1	2	1
19324	101	1	24-Nov-17	23	2	16	2	1	1	0	6	1	2	1
19325	202	0	21-Dec-15	16	1	17	2	1	1	0	4	1	2	1
19326	202	0	6-Oct-13	1	1	17	2	1	1	0	10	1	6	2
19327	202	0	18-Sep-12	18	1	17	2	1	1	0	6	1	3	1
19328	202	0	6-Feb-14	16	1	17	2	1	1	0	6	1	2	2
19329	202	0	26-Aug-13	13	1	17	2	2	1	0	10	1	7	2
19330	202	0	1-Mar-12	16	1	17	2	1	1	0	10	1	7	2
19331	202	0	24-Jan-16	19	1	17	2	3	1	0	10	1	6	2
19332	202	0	12-Mar-16	18	1	17	2	1	1	0	10	1	1	2
19333	202	0	25-Dec-15	22	1	17	2	2	1	0	4	1	2	1
19334	202	0	17-Jun-12	17	1	17	2	2	1	0	6	1	2	2
19335	202	0	19-Jun-14	13	1	17	2	1	1	0	6	1	2	2
19336	202	0	10-Aug-15	15	1	17	2	1	1	0	6	1	2	1
19337	202	0	30-Jan-16	20	1	17	2	1	1	0	6	1	2	1

19338	202	0	22-Jul-17	23	1	17	2	1	1	0	10	1	6	1
19339	202	0	30-Sep-12	15	1	17	2	1	1	0	10	1	7	2
19340	202	0	6-Apr-10	21	1	17	2	1	1	0	7	1	2	2
19341	202	0	8-Mar-06	17	1	17	2	1	1	0	7	1	1	1
19342	202	0	11-Mar-06	17	1	17	2	1	1	0	7	1	2	1
19343	202	0	27-May-17	9	1	17	2	1	1	0	7	1	3	1
19344	202	0	16-Mar-08	12	1	17	2	1	1	0	13	1	7	1
19345	202	0	7-Apr-08	8	1	17	2	1	1	0	13	1	4	1
19346	202	0	21-May-07	21	1	17	2	1	1	0	13	1	4	1
19347	202	0	29-Dec-07	18	1	17	2	1	1	0	13	1	1	1
19348	202	0	4-Sep-10	22	1	17	2	3	1	0	4	1	2	2
19349	202	0	27-May-10	20	1	17	2	1	1	0	4	1	1	1
19350	202	0	15-Apr-06	16	1	17	2	1	1	0	4	1	2	1
19351	202	0	27-Jun-16	21	1	17	2	1	1	0	11	1	3	1
19352	202	0	26-May-15	15	1	17	2	3	1	0	11	1	2	1
19353	202	0	26-Sep-14	15	1	17	2	1	1	0	11	1	2	1
19354	202	0	20-Oct-13	18	1	17	2	3	1	0	11	1	3	1
19355	202	0	6-Oct-13	4	1	17	2	1	1	0	11	1	1	1
19356	202	0	11-Jul-13	10	1	17	2	2	1	0	11	1	2	1
19357	202	0	1-May-12	2	1	17	2	1	1	0	11	1	6	2
19358	202	0	17-Mar-09	14	1	17	2	1	1	0	11	1	2	1
19359	202	0	6-Dec-17	20	1	17	2	1	1	0	11	1	3	1
19360	202	0	28-Aug-17	23	1	17	2	1	1	0	11	1	2	1
19361	202	0	24-Jan-16	22	1	17	2	1	1	0	3	1	1	1
19362	202	0	29-Apr-16	9	1	17	2	3	1	0	3	1	3	1
19363	202	0	21-Dec-13	16	1	17	2	2	1	0	3	1	1	2
19364	202	0	9-Jun-12	14	1	17	2	3	1	0	3	1	1	1
19365	202	0	9-Dec-11	20	1	17	2	1	1	0	3	1	1	2
19366	202	0	23-May-10	10	1	17	2	1	1	0	3	1	1	1
19367	202	0	2-Sep-08	22	1	17	2	1	1	0	3	1	1	1
19368	202	0	7-Mar-07	19	1	17	2	1	1	0	3	1	2	1
19369	202	0	13-Jun-07	10	1	17	2	1	1	0	3	1	2	1
19370	202	0	30-Jan-17	1	1	17	2	1	1	0	3	1	1	1
19371	202	0	19-Aug-17	21	1	17	2	3	1	0	3	1	1	1
19372	202	0	26-Jul-16	17	1	17	2	3	1	0	6	1	2	2
19373	202	0	28-Oct-13	22	1	17	2	1	1	0	6	1	2	1
19374	202	0	1-Apr-12	18	1	17	2	1	1	0	6	1	3	2
19375	202	0	9-Jan-12	20	1	17	2	1	1	0	6	1	2	2
19376	202	0	17-Nov-11	8	1	17	2	3	1	0	6	1	2	1
19377	202	0	26-Dec-11	18	1	17	2	1	1	0	6	1	2	1
19378	202	0	10-Jan-11	22	1	17	2	1	1	0	6	1	2	1
19379	202	0	12-Aug-08	21	1	17	2	1	1	0	6	1	3	2
19380	202	0	23-Oct-08	20	1	17	2	1	1	0	6	1	2	2
19381	202	0	26-Apr-06	22	1	17	2	2	1	0	6	1	2	1
19382	202	0	18-Jul-17	22	1	17	2	1	1	0	6	1	1	1
19383	202	0	3-Jul-16	8	1	17	2	1	1	0	13	1	1	1
19384	202	0	24-Dec-13	5	2	17	2	2	1	0	6	1	2	1
19385	202	0	18-Feb-14	12	2	17	2	1	1	0	10	1	7	2
19386	202	0	30-Dec-13	12	2	17	2	2	1	0	10	1	7	1
19387	202	0	29-Dec-13	17	2	17	2	1	1	0	10	1	7	1
19388	202	0	12-Mar-16	22	2	17	2	1	1	0	10	1	1	2
19389	202	0	24-May-14	18	2	17	2	1	1	0	7	1	3	1
19390	202	0	10-Sep-14	13	2	17	2	1	1	0	10	1	7	1
19391	202	0	30-Dec-14	10	2	17	2	1	1	0	6	1	2	1
19392	202	0	4-Apr-14	22	2	17	2	1	1	0	6	1	2	1
19393	202	0	19-Mar-11	16	2	17	2	1	1	0	10	1	7	2
19394	202	0	29-Jan-08	22	2	17	2	2	1	0	10	1	7	1
19395	202	0	23-Aug-07	21	2	17	2	1	1	0	10	1	6	2
19396	202	0	3-Mar-06	19	2	17	2	1	1	0	10	1	1	2
19397	202	0	3-Mar-06	12	2	17	2	1	1	0	10	1	7	2
19398	202	0	22-Mar-11	8	2	17	2	1	1	0	7	1	3	2
19399	202	0	31-Mar-09	13	2	17	2	2	1	0	7	1	7	2
19400	202	0	31-Oct-15	16	2	17	2	1	1	0	11	1	2	1
19401	202	0	23-Jul-14	12	2	17	2	3	1	0	11	1	2	1
19402	202	0	12-Jan-14	9	2	17	2	2	1	0	11	1	3	1
19403	202	0	16-Mar-13	20	2	17	2	1	1	0	11	1	3	1
19404	202	0	12-Jun-13	20	2	17	2	1	1	0	11	1	3	1
19405	202	0	18-Mar-12	14	2	17	2	1	1	0	11	1	3	2
19406	202	0	3-Apr-11	11	2	17	2	1	1	0	11	1	3	1
19407	202	0	16-Mar-10	22	2	17	2	1	1	0	11	1	2	1
19408	202	0	16-Mar-10	23	2	17	2	1	1	0	11	1	2	1
19409	202	0	10-Jul-07	6	2	17	2	1	1	0	11	1	2	1
19410	202	0	7-Oct-17	3	2	17	2	1	1	0	11	1	3	1
19411	202	0	25-Oct-16	17	2	17	2	1	1	0	3	1	3	1
19412	202	0	12-Aug-13	15	2	17	2	1	1	0	3	1	1	1
19413	202	0	25-Dec-13	18	2	17	2	1	1	0	3	1	1	2
19414	202	0	16-Jan-13	21	2	17	2	1	1	0	3	1	1	1
19415	202	0	23-Jan-13	22	2	17	2	1	1	0	3	1	1	1
19416	202	0	20-May-08	20	2	17	2	1	1	0	3	1	3	1
19417	202	0	1-Oct-08	7	2	17	2	1	1	0	3	1	1	1
19418	202	0	29-Aug-14	18	2	17	2	1	1	0	6	1	2	1
19419	202	0	10-Feb-10	15	2	17	2	1	1	0	6	1	2	1
19420	202	0	29-Sep-10	23	2	17	2	1	1	0	6	1	2	1
19421	202	0	17-Apr-10	19	2	17	2	1	1	0	6	1	2	1
19422	202	0	24-Nov-09	17	2	17	2	1	1	0	13	1	2	1
19423	101	1	22-Feb-16	14	1	17	2	1	1	0	1	1	5	1
19424	201	1	18-Jul-17	11	1	17	2	2	1	0	4	1	3	1
19425	201	1	24-Nov-14	23	1	17	2	1	1	0	6	1	2	1
19426	201	1	18-Sep-14	20	1	17	2	2	1	0	6	1	2	1
19427	101	1	20-Apr-12	5	1	17	2	3	1	0	6	1	2	1
19428	201	1	26-Jun-15	14	1	17	2	3	1	0	6	1	2	1
19429	201	1	8-Aug-17	10	1	17	2	1	1	0	6	1	2	1
19430	201	1	12-Aug-15	20	1	17	2	2	1	0	4	1	2	1
19431	201	1	1-Feb-13	18	1	17	2	3	1	0	6	1	3	1
19432	201	1	27-Feb-14	18	1	17	2	3	1	0	6	1	2	1
19433	201	1	10-Apr-13	19	1	17	2	2	1	0	6	1	2	1
19434	201	1	27-Nov-13	20	1	17	2	2	1	0	6	1	2	1
19435	201	1	30-Dec-16	18	1	17	2	1	1	0	6	1	2	1
19436	201	1	17-Apr-15	19	1	17	2	2	1	0	6	1	2	1
19437	201	1	10-Feb-15	9	1	17	2	3	1	0	6	1	2	1
19438	201	1	7-May-17	20	1	17	2	1	1	0	6	1	2	1
19439	201	1	28-May-14	12	1	17	2	3	1	0	4	1	2	1
19440	201	1	16-Sep-13	5	1	17	2	1	1	0	4	1	3	1
19441	201	1	15-Mar-15	13	1	17	2	2	1	0	6	1	2	1
19442	201	1	15-Jun-17	21	1	17	2	1	1	0	6	1	2	1
19443	201	1	20-Mar-14	20	1	17	2	1	1	0	7	1	5	1
19444	201	1	3-Apr-14	17	1	17	2	1	1	0	7	1	3	1
19445	201	1	21-Jul-15	10	1	17	2	2	1	0	6	1	2	1
19446	201	1	24-Nov-12	20	1	17	2	1	1	0	6	1	2	1
19447	201	1	10-Sep-13	17	1	17	2	2	1	0	4	1	2	1
19448	201	1	12-Jun-12	21	1	17	2	3	1	0	13	1	1	1
19449	201	1	1-Jul-16	6	1	17	2	3	1	0	6	1	2	1
19450	101	1	4-Jul-17	11	1	17	2	1	1	0	6	1	2	1
19451	201	1	26-Oct-16	20	1	17	2	1	1	0	6	1	3	1
19452	201	1	16-Aug-16	21	1	17	2	3	1	0	6	1	3	1
19453	101	1	1-Oct-17	22	1	17	2	1	1	0	6	1	3	1
19454	201	1	30-Apr-16	7	1	17	2	2	1	0	6	1	2	1
19455	101	1	15-Jun-16	18	1	17	2	1	1	0	6	1	2	1
19456	201	1	22-Jul-12	16	1	17	2	2	1	0	6	1	2	1
19457	201	1	10-Jan-12	19	1	17	2	1	1	0	6	1	2	1
19458	201	1	13-Dec-17	0	1	17	2	2	1	0	6	1	2	1
19459	201	1	5-Aug-15	17	1	17	2	2	1	0	7	1	2	1
19460	201	1	12-Sep-15	12	1	17	2	2	1	0	7	1	4	1
19461	201	1	24-Mar-14	8	1	17	2	2	1	0	7	1	5	1

19462	101	1	14-May-14	19	1	17	2	1	1	0	7	1	2	1
19463	101	1	10-Aug-14	16	1	17	2	1	1	0	7	1	2	1
19464	201	1	29-Sep-14	9	1	17	2	1	1	0	7	1	3	1
19465	201	1	2-Jul-13	11	1	17	2	1	1	0	7	1	2	1
19466	201	1	30-Jun-13	6	1	17	2	1	1	0	7	1	2	1
19467	101	1	12-Dec-12	18	1	17	2	1	1	0	7	1	2	1
19468	101	1	11-Jul-12	7	1	17	2	1	1	0	7	1	3	1
19469	101	1	19-Sep-11	23	1	17	2	1	1	0	7	1	2	1
19470	201	1	4-Nov-08	8	1	17	2	2	1	0	7	1	2	1
19471	201	1	28-Sep-07	16	1	17	2	1	1	0	7	1	1	1
19472	101	1	6-Feb-06	19	1	17	2	1	1	0	7	1	2	2
19473	101	1	8-Sep-17	21	1	17	2	1	1	0	7	1	2	1
19474	101	1	23-Jul-11	23	1	17	2	1	1	0	1	1	3	2
19475	101	1	3-Aug-10	1	1	17	2	1	1	0	1	1	2	1
19476	101	1	2-Oct-06	11	1	17	2	1	1	0	1	1	5	1
19477	201	1	10-Nov-15	11	1	17	2	1	1	0	4	1	2	1
19478	101	1	22-Apr-14	19	1	17	2	1	1	0	4	1	2	1
19479	201	1	7-Dec-14	21	1	17	2	1	1	0	4	1	2	1
19480	101	1	27-Mar-14	18	1	17	2	1	1	0	4	1	2	1
19481	101	1	19-Jun-13	17	1	17	2	1	1	0	4	1	3	1
19482	101	1	12-Jan-13	1	1	17	2	1	1	0	4	1	2	1
19483	101	1	17-Oct-12	22	1	17	2	1	1	0	4	1	3	1
19484	101	1	4-Sep-11	20	1	17	2	1	1	0	4	1	1	1
19485	201	1	26-Jul-11	3	1	17	2	1	1	0	4	1	3	2
19486	101	1	8-Sep-11	22	1	17	2	1	1	0	4	1	2	1
19487	201	1	17-Nov-11	22	1	17	2	1	1	0	4	1	2	1
19488	201	1	7-May-11	12	1	17	2	2	1	0	4	1	2	1
19489	101	1	20-Jun-10	2	1	17	2	1	1	0	4	1	1	1
19490	201	1	23-Jan-10	19	1	17	2	2	1	0	4	1	2	1
19491	201	1	14-May-10	0	1	17	2	1	1	0	4	1	2	1
19492	101	1	27-Nov-10	17	1	17	2	1	1	0	4	1	3	1
19493	201	1	23-May-09	21	1	17	2	2	1	0	4	1	4	2
19494	101	1	8-Jul-09	19	1	17	2	1	1	0	4	1	1	1
19495	201	1	18-Jun-09	15	1	17	2	2	1	0	4	1	3	2
19496	201	1	25-Nov-09	22	1	17	2	2	1	0	4	1	3	1
19497	101	1	15-Aug-09	18	1	17	2	1	1	0	4	1	2	4
19498	101	1	26-Feb-08	21	1	17	2	1	1	0	4	1	2	1
19499	201	1	1-May-07	0	1	17	2	1	1	0	4	1	1	1
19500	201	1	7-Jan-07	20	1	17	2	1	1	0	4	1	2	1
19501	101	1	25-Apr-07	19	1	17	2	1	1	0	4	1	3	1
19502	201	1	9-Oct-16	19	1	17	2	2	1	0	11	1	2	1
19503	101	1	18-Apr-16	21	1	17	2	1	1	0	11	1	2	1
19504	201	1	10-Aug-16	18	1	17	2	1	1	0	11	1	3	1
19505	101	1	28-Jul-16	18	1	17	2	1	1	0	11	1	1	1
19506	101	1	7-Feb-16	22	1	17	2	1	1	0	11	1	2	1
19507	101	1	1-Nov-16	20	1	17	2	1	1	0	11	1	2	1
19508	201	1	11-Nov-16	17	1	17	2	1	1	0	11	1	2	1
19509	101	1	6-Dec-16	12	1	17	2	1	1	0	11	1	2	1
19510	101	1	4-Aug-16	19	1	17	2	1	1	0	11	1	2	1
19511	101	1	23-May-16	14	1	17	2	1	1	0	11	1	3	1
19512	201	1	17-Jun-15	14	1	17	2	3	1	0	11	1	2	1
19513	101	1	26-Jun-15	18	1	17	2	1	1	0	11	1	2	1
19514	101	1	17-Oct-15	20	1	17	2	1	1	0	11	1	1	1
19515	101	1	22-Aug-15	16	1	17	2	1	1	0	11	1	2	1
19516	101	1	24-Feb-15	14	1	17	2	1	1	0	11	1	3	1
19517	101	1	7-Jun-15	15	1	17	2	1	1	0	11	1	2	1
19518	201	1	15-Oct-15	18	1	17	2	2	1	0	11	1	2	1
19519	201	1	5-Jun-15	7	1	17	2	2	1	0	11	1	3	1
19520	201	1	8-Sep-14	20	1	17	2	3	1	0	11	1	3	1
19521	201	1	5-Mar-14	13	1	17	2	2	1	0	11	1	3	1
19522	101	1	18-Mar-14	12	1	17	2	1	1	0	11	1	2	1
19523	201	1	4-Jan-14	15	1	17	2	1	1	0	11	1	2	1
19524	101	1	20-Dec-14	14	1	17	2	1	1	0	11	1	2	1
19525	101	1	6-Aug-14	8	1	17	2	1	1	0	11	1	3	1
19526	101	1	10-May-14	19	1	17	2	1	1	0	11	1	2	1
19527	101	1	7-Apr-14	17	1	17	2	2	1	0	11	1	2	1
19528	101	1	5-Oct-13	18	1	17	2	1	1	0	11	1	4	1
19529	201	1	2-Sep-13	6	1	17	2	1	1	0	11	1	2	1
19530	101	1	4-Aug-13	19	1	17	2	3	1	0	11	1	2	1
19531	101	1	10-Aug-13	21	1	17	2	2	1	0	11	1	3	1
19532	201	1	22-Apr-13	17	1	17	2	2	1	0	11	1	2	1
19533	101	1	3-Oct-13	2	1	17	2	1	1	0	11	1	2	1
19534	101	1	5-Oct-13	10	1	17	2	1	1	0	11	1	2	1
19535	101	1	10-Nov-13	7	1	17	2	1	1	0	11	1	2	1
19536	101	1	2-Mar-13	23	1	17	2	1	1	0	11	1	3	1
19537	201	1	29-Jul-12	10	1	17	2	3	1	0	11	1	2	1
19538	201	1	1-Aug-12	10	1	17	2	2	1	0	11	1	1	1
19539	101	1	20-Jan-12	1	1	17	2	1	1	0	11	1	2	1
19540	201	1	24-Jul-12	15	1	17	2	2	1	0	11	1	2	1
19541	201	1	21-Jul-12	15	1	17	2	2	1	0	11	1	2	1
19542	101	1	7-Jul-12	19	1	17	2	1	1	0	11	1	3	1
19543	101	1	21-Jul-12	10	1	17	2	1	1	0	11	1	3	1
19544	101	1	23-Oct-12	23	1	17	2	1	1	0	11	1	3	1
19545	101	1	15-Jan-12	16	1	17	2	1	1	0	11	1	2	1
19546	201	1	20-Jul-12	10	1	17	2	2	1	0	11	1	3	1
19547	201	1	23-Nov-11	5	1	17	2	2	1	0	11	1	3	1
19548	201	1	20-Sep-11	23	1	17	2	1	1	0	11	1	3	1
19549	201	1	16-Jul-11	19	1	17	2	1	1	0	11	1	2	1
19550	101	1	7-Feb-11	10	1	17	2	1	1	0	11	1	2	1
19551	101	1	18-Feb-11	22	1	17	2	1	1	0	11	1	2	1
19552	101	1	28-Aug-11	16	1	17	2	1	1	0	11	1	3	1
19553	101	1	19-Feb-11	16	1	17	2	1	1	0	11	1	2	1
19554	201	1	3-May-10	13	1	17	2	1	1	0	11	1	2	1
19555	101	1	23-Oct-10	19	1	17	2	1	1	0	11	1	3	1
19556	201	1	5-May-10	15	1	17	2	2	1	0	11	1	3	1
19557	101	1	20-Aug-10	12	1	17	2	1	1	0	11	1	2	1
19558	101	1	24-Feb-10	23	1	17	2	1	1	0	11	1	2	1
19559	101	1	4-Aug-10	8	1	17	2	1	1	0	11	1	2	1
19560	101	1	6-Aug-09	21	1	17	2	1	1	0	11	1	2	1
19561	201	1	17-Sep-09	20	1	17	2	2	1	0	11	1	3	1
19562	101	1	14-Nov-09	20	1	17	2	1	1	0	11	1	2	1
19563	101	1	26-Jul-09	22	1	17	2	1	1	0	11	1	2	1
19564	101	1	9-Jul-09	22	1	17	2	1	1	0	11	1	2	1
19565	101	1	22-Aug-09	23	1	17	2	1	1	0	11	1	3	1
19566	101	1	30-Oct-09	17	1	17	2	1	1	0	11	1	2	1
19567	101	1	7-Sep-09	23	1	17	2	1	1	0	11	1	3	1
19568	101	1	21-Dec-08	16	1	17	2	1	1	0	11	1	2	1
19569	201	1	4-Nov-07	17	1	17	2	2	1	0	11	1	3	1
19570	201	1	23-May-07	21	1	17	2	1	1	0	11	1	3	1
19571	101	1	19-Jul-06	20	1	17	2	1	1	0	11	1	3	1
19572	201	1	30-Jul-17	18	1	17	2	1	1	0	11	1	3	1
19573	101	1	24-Aug-17	19	1	17	2	1	1	0	11	1	2	1
19574	101	1	10-Sep-17	6	1	17	2	1	1	0	11	1	2	1
19575	101	1	19-Jan-17	20	1	17	2	1	1	0	11	1	2	1
19576	101	1	18-Jan-17	17	1	17	2	1	1	0	11	1	2	1
19577	101	1	29-Jun-17	21	1	17	2	1	1	0	11	1	2	1
19578	201	1	9-Sep-17	10	1	17	2	2	1	0	11	1	3	1
19579	201	1	9-Sep-17	18	1	17	2	2	1	0	11	1	2	1
19580	201	1	19-Oct-17	17	1	17	2	2	1	0	11	1	2	1
19581	201	1	2-Nov-17	14	1	17	2	2	1	0	11	1	2	1
19582	101	1	12-Sep-17	8	1	17	2	1	1	0	11	1	3	1
19583	101	1	1-Dec-17	7	1	17	2	1	1	0	11	1	2	1
19584	101	1	12-Oct-17	12	1	17	2	1	1	0	11	1	2	1
19585	101	1	28-Apr-17	16	1	17	2	1	1	0	11	1	2	1

19586	201	1	2-Jul-16	14	1	17	2	2	1	0	3	1	3	1
19587	201	1	24-Feb-15	17	1	17	2	1	1	0	3	1	3	1
19588	101	1	28-Sep-15	6	1	17	2	1	1	0	3	1	2	1
19589	101	1	5-May-15	20	1	17	2	1	1	0	3	1	1	1
19590	101	1	26-Sep-15	19	1	17	2	1	1	0	3	1	4	1
19591	101	1	28-May-15	16	1	17	2	1	1	0	3	1	3	1
19592	201	1	23-Jul-15	1	1	17	2	3	1	0	3	1	3	1
19593	101	1	24-Jan-15	8	1	17	2	1	1	0	3	1	3	1
19594	201	1	5-May-14	22	1	17	2	3	1	0	3	1	4	1
19595	201	1	2-Feb-14	20	1	17	2	2	1	0	3	1	2	1
19596	201	1	22-Mar-14	6	1	17	2	1	1	0	3	1	3	1
19597	101	1	24-May-13	8	1	17	2	3	1	0	3	1	5	1
19598	201	1	27-Oct-13	15	1	17	2	1	1	0	3	1	1	1
19599	101	1	13-Jun-13	18	1	17	2	1	1	0	3	1	1	1
19600	101	1	6-Feb-13	5	1	17	2	1	1	0	3	1	1	1
19601	101	1	1-Jun-13	8	1	17	2	1	1	0	3	1	1	1
19602	201	1	4-Jun-12	17	1	17	2	1	1	0	3	1	3	1
19603	101	1	10-Jan-12	23	1	17	2	3	1	0	3	1	1	1
19604	201	1	27-Mar-12	22	1	17	2	2	1	0	3	1	1	1
19605	201	1	30-Jul-11	15	1	17	2	2	1	0	3	1	1	1
19606	101	1	3-Aug-11	7	1	17	2	1	1	0	3	1	1	1
19607	201	1	5-May-11	20	1	17	2	2	1	0	3	1	2	2
19608	201	1	20-Aug-11	1	1	17	2	3	1	0	3	1	2	1
19609	201	1	9-Jul-11	8	1	17	2	2	1	0	3	1	3	1
19610	101	1	10-Jun-11	1	1	17	2	1	1	0	3	1	3	1
19611	201	1	9-Apr-11	20	1	17	2	2	1	0	3	1	2	1
19612	201	1	25-Mar-11	23	1	17	2	1	1	0	3	1	2	1
19613	201	1	13-Oct-11	0	1	17	2	1	1	0	3	1	2	1
19614	101	1	17-Oct-10	20	1	17	2	2	1	0	3	1	1	1
19615	201	1	18-Oct-10	19	1	17	2	2	1	0	3	1	3	1
19616	201	1	1-Nov-10	21	1	17	2	2	1	0	3	1	1	1
19617	101	1	19-Mar-09	22	1	17	2	1	1	0	3	1	3	4
19618	201	1	30-Jan-09	21	1	17	2	2	1	0	3	1	1	1
19619	201	1	14-Feb-07	23	1	17	2	2	1	0	3	1	1	1
19620	101	1	15-Jun-07	18	1	17	2	1	1	0	3	1	4	1
19621	101	1	10-Mar-07	19	1	17	2	1	1	0	3	1	2	1
19622	101	1	14-Nov-07	7	1	17	2	1	1	0	3	1	3	1
19623	101	1	19-Sep-17	13	1	17	2	2	1	0	3	1	2	1
19624	101	1	29-Mar-16	18	1	17	2	1	1	0	6	1	2	1
19625	101	1	17-Aug-16	16	1	17	2	1	1	0	6	1	2	1
19626	101	1	16-Mar-16	22	1	17	2	1	1	0	6	1	2	1
19627	101	1	6-May-16	22	1	17	2	1	1	0	6	1	2	1
19628	101	1	26-Apr-15	2	1	17	2	1	1	0	6	1	3	1
19629	101	1	2-Jul-15	19	1	17	2	1	1	0	6	1	2	1
19630	201	1	17-Jul-15	19	1	17	2	2	1	0	6	1	2	1
19631	101	1	12-Aug-15	11	1	17	2	1	1	0	6	1	2	1
19632	101	1	27-Mar-14	17	1	17	2	1	1	0	6	1	1	1
19633	201	1	2-Feb-14	14	1	17	2	2	1	0	6	1	1	1
19634	201	1	1-Jan-14	17	1	17	2	2	1	0	6	1	2	1
19635	101	1	18-Feb-14	9	1	17	2	1	1	0	6	1	2	1
19636	101	1	20-Sep-14	15	1	17	2	1	1	0	6	1	2	1
19637	201	1	20-Jun-14	10	1	17	2	2	1	0	6	1	2	1
19638	101	1	5-Oct-13	11	1	17	2	1	1	0	6	1	2	1
19639	101	1	18-Feb-13	18	1	17	2	1	1	0	6	1	4	1
19640	101	1	29-Jan-13	22	1	17	2	1	1	0	6	1	2	1
19641	101	1	24-Apr-13	15	1	17	2	1	1	0	6	1	2	1
19642	101	1	3-Feb-13	23	1	17	2	1	1	0	6	1	2	4
19643	201	1	10-Sep-13	1	1	17	2	2	1	0	6	1	2	1
19644	101	1	9-Mar-12	19	1	17	2	1	1	0	6	1	2	1
19645	101	1	24-Aug-12	20	1	17	2	1	1	0	6	1	2	2
19646	101	1	25-Sep-12	0	1	17	2	1	1	0	6	1	2	1
19647	101	1	30-Sep-12	19	1	17	2	1	1	0	6	1	2	1
19648	101	1	1-Jul-12	22	1	17	2	1	1	0	6	1	2	1
19649	101	1	29-Nov-12	15	1	17	2	1	1	0	6	1	2	1
19650	101	1	17-Oct-12	17	1	17	2	1	1	0	6	1	2	1
19651	101	1	20-Apr-12	22	1	17	2	1	1	0	6	1	2	1
19652	101	1	24-Jul-11	3	1	17	2	1	1	0	6	1	3	1
19653	201	1	26-Oct-11	23	1	17	2	1	1	0	6	1	3	1
19654	201	1	22-Oct-11	16	1	17	2	2	1	0	6	1	2	1
19655	101	1	30-Jan-11	23	1	17	2	1	1	0	6	1	2	1
19656	101	1	10-Sep-11	21	1	17	2	1	1	0	6	1	2	1
19657	101	1	4-Mar-11	12	1	17	2	1	1	0	6	1	2	1
19658	101	1	16-Nov-11	7	1	17	2	1	1	0	6	1	2	1
19659	101	1	14-May-11	12	1	17	2	1	1	0	6	1	2	1
19660	201	1	13-May-11	22	1	17	2	1	1	0	6	1	3	1
19661	101	1	26-Apr-11	2	1	17	2	1	1	0	6	1	3	1
19662	101	1	21-Jun-11	23	1	17	2	1	1	0	6	1	3	1
19663	101	1	19-May-11	7	1	17	2	1	1	0	6	1	2	1
19664	101	1	16-Jun-11	19	1	17	2	1	1	0	6	1	2	1
19665	101	1	22-Dec-10	21	1	17	2	2	1	0	6	1	3	2
19666	201	1	30-Mar-10	7	1	17	2	1	1	0	6	1	2	1
19667	201	1	11-Jan-10	20	1	17	2	1	1	0	6	1	3	1
19668	201	1	24-Aug-10	11	1	17	2	3	1	0	6	1	2	1
19669	201	1	22-Oct-10	13	1	17	2	2	1	0	6	1	2	2
19670	101	1	8-May-10	21	1	17	2	1	1	0	6	1	2	1
19671	201	1	12-Jun-10	11	1	17	2	1	1	0	6	1	2	1
19672	201	1	11-Mar-10	19	1	17	2	2	1	0	6	1	2	1
19673	201	1	15-Sep-10	12	1	17	2	2	1	0	6	1	2	1
19674	201	1	27-Oct-09	19	1	17	2	1	1	0	6	1	2	1
19675	101	1	27-May-09	21	1	17	2	1	1	0	6	1	1	1
19676	101	1	24-Sep-09	0	1	17	2	1	1	0	6	1	2	1
19677	101	1	24-Jul-09	11	1	17	2	1	1	0	6	1	2	1
19678	201	1	1-Oct-09	8	1	17	2	2	1	0	6	1	2	1
19679	201	1	1-Oct-09	22	1	17	2	1	1	0	6	1	2	1
19680	201	1	2-Jan-07	21	1	17	2	1	1	0	6	1	2	1
19681	101	1	21-Nov-07	21	1	17	2	1	1	0	6	1	2	1
19682	101	1	6-May-07	18	1	17	2	1	1	0	6	1	1	1
19683	201	1	9-Jun-07	9	1	17	2	1	1	0	6	1	2	1
19684	201	1	17-Aug-07	23	1	17	2	2	1	0	6	1	2	1
19685	101	1	30-Jul-07	21	1	17	2	1	1	0	6	1	2	1
19686	101	1	1-Oct-07	14	1	17	2	1	1	0	6	1	2	1
19687	101	1	12-May-07	16	1	17	2	1	1	0	6	1	2	1
19688	101	1	23-Aug-06	18	1	17	2	1	1	0	6	1	2	1
19689	201	1	24-Jul-06	14	1	17	2	2	1	0	6	1	2	1
19690	101	1	19-Jan-06	17	1	17	2	1	1	0	6	1	2	2
19691	101	1	23-Feb-06	19	1	17	2	1	1	0	6	1	2	1
19692	101	1	6-Dec-06	0	1	17	2	1	1	0	6	1	3	1
19693	101	1	1-Jan-17	1	1	17	2	1	1	0	6	1	2	1
19694	201	1	15-May-17	19	1	17	2	3	1	0	6	1	3	2
19695	101	1	13-Oct-17	19	1	17	2	1	1	0	6	1	2	1
19696	101	1	14-Mar-17	20	1	17	2	1	1	0	13	1	4	1
19697	101	1	23-Jan-17	16	2	17	2	1	1	0	11	1	3	1
19698	101	1	11-Jun-17	8	2	17	2	1	1	0	6	1	2	2
19699	201	1	24-Aug-17	13	2	17	2	2	1	0	7	1	3	1
19700	201	1	25-Aug-12	21	2	17	2	3	1	0	6	1	2	1
19701	201	1	20-Oct-16	16	2	17	2	2	1	0	4	1	2	2
19702	201	1	21-Jan-16	1	2	17	2	1	1	0	6	1	2	2
19703	201	1	6-May-17	18	2	17	2	2	1	0	3	1	4	1
19704	101	1	25-May-12	21	2	17	2	1	1	0	6	1	3	1
19705	201	1	26-Oct-14	10	2	17	2	2	1	0	4	1	2	1
19706	201	1	25-Feb-15	22	2	17	2	3	1	0	6	1	2	1
19707	201	1	18-Dec-17	21	2	17	2	3	1	0	6	1	2	1
19708	201	1	4-Jul-17	16	2	17	2	1	1	0	6	1	2	1
19709	101	1	26-May-15	11	2	17	2	1	1	0	7	1	3	1

19710	201	1	10-Jan-13	16	2	17	2	2	1	0	7	1	3	1
19711	101	1	24-Mar-13	17	2	17	2	1	1	0	7	1	4	1
19712	101	1	30-Jul-12	17	2	17	2	1	1	0	7	1	3	1
19713	101	1	1-Jul-11	12	2	17	2	1	1	0	7	1	2	1
19714	101	1	1-Oct-11	18	2	17	2	3	1	0	7	1	5	2
19715	201	1	11-Nov-09	11	2	17	2	2	1	0	7	1	1	1
19716	101	1	9-May-09	14	2	17	2	1	1	0	7	1	1	1
19717	201	1	31-Dec-08	18	2	17	2	3	1	0	7	1	2	1
19718	201	1	4-Feb-06	14	2	17	2	3	1	0	7	1	3	1
19719	101	1	16-Mar-17	21	2	17	2	1	1	0	7	1	2	1
19720	101	1	24-Oct-17	21	2	17	2	1	1	0	7	1	3	1
19721	101	1	29-Oct-17	20	2	17	2	1	1	0	7	1	2	1
19722	201	1	4-Mar-14	9	2	17	2	2	1	0	4	1	2	1
19723	201	1	23-Apr-14	9	2	17	2	2	1	0	4	1	2	1
19724	101	1	14-May-14	21	2	17	2	1	1	0	4	1	2	1
19725	101	1	5-Feb-13	19	2	17	2	1	1	0	4	1	2	1
19726	101	1	2-Sep-09	22	2	17	2	1	1	0	4	1	3	1
19727	101	1	16-Feb-07	11	2	17	2	1	1	0	4	1	2	1
19728	101	1	1-Aug-16	12	2	17	2	1	1	0	11	1	1	1
19729	201	1	24-Aug-16	17	2	17	2	2	1	0	11	1	2	1
19730	101	1	25-Dec-16	2	2	17	2	1	1	0	11	1	1	1
19731	201	1	7-Jul-16	18	2	17	2	1	1	0	11	1	5	1
19732	101	1	10-May-16	18	2	17	2	1	1	0	11	1	2	1
19733	201	1	16-Mar-16	6	2	17	2	1	1	0	11	1	3	1
19734	101	1	2-Feb-16	11	2	17	2	1	1	0	11	1	2	1
19735	101	1	19-Feb-16	20	2	17	2	1	1	0	11	1	2	1
19736	201	1	11-Nov-16	19	2	17	2	2	1	0	11	1	2	1
19737	201	1	8-May-16	17	2	17	2	1	1	0	11	1	2	1
19738	101	1	12-May-15	13	2	17	2	1	1	0	11	1	2	1
19739	101	1	11-Oct-15	17	2	17	2	1	1	0	11	1	2	1
19740	201	1	18-Aug-15	0	2	17	2	2	1	0	11	1	1	1
19741	101	1	20-Dec-15	11	2	17	2	1	1	0	11	1	2	1
19742	101	1	3-Oct-15	11	2	17	2	1	1	0	11	1	3	1
19743	201	1	13-Oct-15	19	2	17	2	2	1	0	11	1	2	1
19744	101	1	25-Feb-15	12	2	17	2	1	1	0	11	1	2	1
19745	101	1	8-Jun-15	3	2	17	2	2	1	0	11	1	2	1
19746	101	1	31-Jan-15	20	2	17	2	1	1	0	11	1	2	1
19747	101	1	28-Jun-15	19	2	17	2	1	1	0	11	1	2	1
19748	101	1	27-Oct-15	11	2	17	2	1	1	0	11	1	2	1
19749	201	1	28-Oct-14	20	2	17	2	2	1	0	11	1	2	1
19750	101	1	1-Dec-14	8	2	17	2	2	1	0	11	1	2	1
19751	101	1	31-Jan-14	8	2	17	2	1	1	0	11	1	2	1
19752	201	1	11-Feb-14	10	2	17	2	3	1	0	11	1	2	1
19753	201	1	7-Feb-14	20	2	17	2	1	1	0	11	1	2	1
19754	101	1	29-Jun-14	21	2	17	2	1	1	0	11	1	2	1
19755	101	1	29-Jun-13	11	2	17	2	1	1	0	11	1	1	1
19756	101	1	17-Jun-13	8	2	17	2	2	1	0	11	1	5	1
19757	101	1	29-Jun-13	11	2	17	2	1	1	0	11	1	1	1
19758	101	1	8-Sep-13	19	2	17	2	1	1	0	11	1	2	1
19759	101	1	3-Jan-13	17	2	17	2	1	1	0	11	1	2	1
19760	101	1	14-Jul-13	16	2	17	2	1	1	0	11	1	3	1
19761	101	1	1-Aug-13	16	2	17	2	2	1	0	11	1	3	1
19762	101	1	6-Mar-13	0	2	17	2	1	1	0	11	1	3	1
19763	201	1	9-Feb-12	9	2	17	2	2	1	0	11	1	1	1
19764	201	1	23-Aug-12	20	2	17	2	2	1	0	11	1	3	1
19765	101	1	1-Jun-12	22	2	17	2	1	1	0	11	1	3	1
19766	101	1	23-Dec-12	12	2	17	2	1	1	0	11	1	3	1
19767	101	1	18-Jul-12	23	2	17	2	1	1	0	11	1	2	1
19768	201	1	24-Aug-12	11	2	17	2	3	1	0	11	1	2	1
19769	101	1	7-May-12	20	2	17	2	1	1	0	11	1	3	1
19770	201	1	9-Sep-12	19	2	17	2	2	1	0	11	1	3	1
19771	101	1	14-Oct-12	22	2	17	2	1	1	0	11	1	2	1
19772	101	1	12-Jul-12	23	2	17	2	1	1	0	11	1	3	1
19773	101	1	16-Nov-11	22	2	17	2	1	1	0	11	1	2	1
19774	101	1	4-May-11	7	2	17	2	1	1	0	11	1	4	1
19775	101	1	14-Jul-11	12	2	17	2	1	1	0	11	1	2	1
19776	101	1	1-Dec-10	12	2	17	2	1	1	0	11	1	3	1
19777	201	1	18-May-09	11	2	17	2	2	1	0	11	1	2	1
19778	201	1	7-Jun-09	23	2	17	2	2	1	0	11	1	3	1
19779	101	1	18-Dec-09	15	2	17	2	1	1	0	11	1	3	1
19780	101	1	13-Apr-09	21	2	17	2	1	1	0	11	1	2	1
19781	201	1	1-Jul-08	8	2	17	2	1	1	0	11	1	3	1
19782	201	1	2-Jul-07	18	2	17	2	2	1	0	11	1	3	1
19783	201	1	26-Aug-07	20	2	17	2	1	1	0	11	1	2	1
19784	201	1	11-Jan-07	16	2	17	2	1	1	0	11	1	2	1
19785	201	1	4-Jun-07	22	2	17	2	1	1	0	11	1	3	1
19786	101	1	28-Nov-07	22	2	17	2	1	1	0	11	1	2	1
19787	201	1	22-Sep-07	19	2	17	2	1	1	0	11	1	3	1
19788	101	1	10-Nov-17	19	2	17	2	1	1	0	11	1	4	1
19789	101	1	3-Sep-17	20	2	17	2	1	1	0	11	1	3	1
19790	101	1	14-Jul-17	16	2	17	2	1	1	0	11	1	3	1
19791	101	1	14-May-17	21	2	17	2	1	1	0	11	1	3	1
19792	101	1	4-Aug-17	16	2	17	2	1	1	0	11	1	3	1
19793	101	1	4-Jul-17	17	2	17	2	1	1	0	11	1	2	1
19794	101	1	30-May-17	16	2	17	2	1	1	0	11	1	2	1
19795	101	1	1-Apr-17	19	2	17	2	1	1	0	11	1	2	1
19796	101	1	14-Jul-17	16	2	17	2	1	1	0	11	1	2	1
19797	101	1	4-Jul-17	21	2	17	2	1	1	0	11	1	2	1
19798	101	1	8-Feb-17	17	2	17	2	1	1	0	11	1	2	1
19799	201	1	26-Jul-17	9	2	17	2	1	1	0	11	1	2	1
19800	201	1	4-Aug-17	12	2	17	2	1	1	0	11	1	2	1
19801	201	1	30-Jun-17	2	2	17	2	1	1	0	11	1	2	1
19802	201	1	2-Aug-17	7	2	17	2	2	1	0	11	1	2	1
19803	201	1	19-Nov-17	9	2	17	2	3	1	0	11	1	3	1
19804	101	1	13-Oct-17	18	2	17	2	1	1	0	11	1	2	1
19805	101	1	28-Nov-17	19	2	17	2	1	1	0	11	1	2	1
19806	201	1	13-Dec-17	14	2	17	2	1	1	0	11	1	2	1
19807	201	1	30-Jul-16	19	2	17	2	1	1	0	3	1	1	1
19808	101	1	12-Sep-15	15	2	17	2	1	1	0	3	1	4	1
19809	101	1	23-Oct-15	6	2	17	2	1	1	0	3	1	1	1
19810	101	1	12-Oct-15	19	2	17	2	1	1	0	3	1	1	1
19811	201	1	29-Jul-14	17	2	17	2	3	1	0	3	1	1	2
19812	101	1	8-Sep-14	22	2	17	2	1	1	0	3	1	1	1
19813	101	1	6-Apr-13	16	2	17	2	1	1	0	3	1	1	1
19814	201	1	11-Mar-13	19	2	17	2	2	1	0	3	1	1	2
19815	201	1	12-Aug-13	20	2	17	2	2	1	0	3	1	3	1
19816	101	1	28-May-13	7	2	17	2	1	1	0	3	1	5	1
19817	101	1	31-Dec-12	23	2	17	2	1	1	0	3	1	3	4
19818	201	1	29-Jan-11	19	2	17	2	2	1	0	3	1	2	1
19819	201	1	9-Mar-11	20	2	17	2	1	1	0	3	1	2	1
19820	201	1	20-Jun-11	23	2	17	2	1	1	0	3	1	3	1
19821	101	1	9-Jan-10	23	2	17	2	1	1	0	3	1	1	1
19822	101	1	1-Jul-10	12	2	17	2	1	1	0	3	1	1	1
19823	201	1	6-Jun-09	10	2	17	2	1	1	0	3	1	3	1
19824	201	1	7-Feb-09	20	2	17	2	1	1	0	3	1	2	1
19825	201	1	14-Sep-08	20	2	17	2	3	1	0	3	1	3	1
19826	201	1	22-Sep-08	23	2	17	2	2	1	0	3	1	4	1
19827	101	1	29-Sep-07	14	2	17	2	1	1	0	3	1	1	1
19828	201	1	8-Apr-07	23	2	17	2	1	1	0	3	1	3	1
19829	201	1	2-Sep-06	21	2	17	2	1	1	0	3	1	1	1
19830	101	1	23-Apr-16	9	2	17	2	1	1	0	6	1	2	1
19831	101	1	10-Aug-16	11	2	17	2	1	1	0	6	1	2	1
19832	101	1	10-Aug-16	12	2	17	2	1	1	0	6	1	2	1
19833	201	1	3-Sep-16	12	2	17	2	3	1	0	6	1	1	1

19834	101	1	21-Feb-15	22	2	17	2	3	1	0	6	1	2	1
19835	101	1	11-Apr-15	14	2	17	2	1	1	0	6	1	2	1
19836	101	1	30-Dec-15	23	2	17	2	1	1	0	6	1	3	1
19837	101	1	16-May-15	22	2	17	2	1	1	0	6	1	3	2
19838	101	1	28-Apr-15	9	2	17	2	1	1	0	6	1	2	4
19839	201	1	27-May-15	3	2	17	2	3	1	0	6	1	2	1
19840	101	1	28-Apr-14	15	2	17	2	1	1	0	6	1	2	1
19841	201	1	20-Jun-14	20	2	17	2	2	1	0	6	1	2	1
19842	101	1	30-Aug-13	20	2	17	2	1	1	0	6	1	2	1
19843	101	1	24-Mar-13	19	2	17	2	1	1	0	6	1	2	1
19844	101	1	11-Sep-13	21	2	17	2	1	1	0	6	1	3	1
19845	101	1	1-Jun-13	17	2	17	2	1	1	0	6	1	2	1
19846	101	1	26-Jun-12	16	2	17	2	1	1	0	6	1	2	1
19847	101	1	17-Nov-11	14	2	17	2	1	1	0	6	1	2	1
19848	101	1	5-Oct-11	17	2	17	2	1	1	0	6	1	3	1
19849	101	1	26-May-11	2	2	17	2	1	1	0	6	1	2	1
19850	101	1	31-Oct-10	8	2	17	2	2	1	0	6	1	2	1
19851	101	1	1-Sep-10	22	2	17	2	1	1	0	6	1	2	1
19852	101	1	2-Aug-10	22	2	17	2	1	1	0	6	1	3	1
19853	201	1	11-Jul-10	3	2	17	2	2	1	0	6	1	1	1
19854	101	1	9-Jul-08	9	2	17	2	1	1	0	6	1	2	1
19855	201	1	27-Apr-07	20	2	17	2	1	1	0	6	1	2	1
19856	201	1	20-Mar-07	23	2	17	2	1	1	0	6	1	2	1
19857	101	1	26-Sep-07	10	2	17	2	1	1	0	6	1	3	1
19858	101	1	18-Dec-06	18	2	17	2	1	1	0	6	1	2	1
19859	101	1	15-Feb-17	19	2	17	2	1	1	0	6	1	2	1
19860	101	1	28-Oct-17	14	2	17	2	1	1	0	6	1	2	1
19861	101	1	11-Mar-17	3	2	17	2	1	1	0	13	1	1	1
19862	202	0	28-Aug-17	0	2	11	1	2	1	0	7	2	5	2
19863	202	0	15-May-09	16	1	15	4	1	3	1	9	5	1	1
19864	202	0	5-May-10	15	1	15	4	1	3	1	4	5	1	1
19865	202	0	21-Sep-09	12	1	15	4	1	3	1	4	5	1	1
19866	202	0	18-Oct-09	21	1	15	4	3	3	1	4	5	1	2
19867	101	1	13-Dec-13	20	1	15	4	2	3	1	4	5	5	1
19868	101	1	28-Sep-13	4	1	15	4	3	3	1	6	5	2	1
19869	101	1	25-Dec-12	17	2	15	4	1	3	1	4	5	2	2
19870	201	1	23-Jun-07	23	2	15	4	3	3	1	13	5	4	2
19871	202	0	15-Apr-11	2	1	16	4	1	3	1	4	5	1	1
19872	202	0	23-Dec-08	17	1	16	4	1	3	1	4	5	3	1
19873	202	0	17-Jul-08	0	1	16	4	1	3	1	4	5	1	1
19874	202	0	6-Jul-07	10	1	16	4	1	3	1	4	5	1	1
19875	202	0	25-Dec-07	3	2	16	4	1	3	1	6	5	1	2
19876	101	1	2-Mar-14	23	1	16	4	3	3	1	4	5	5	4
19877	101	1	5-Jun-13	1	1	16	4	1	3	1	4	5	1	1
19878	101	1	26-Oct-11	23	1	16	4	2	3	1	4	5	5	4
19879	101	1	5-Jan-13	0	2	16	4	3	3	1	6	5	2	2
19880	202	0	10-Aug-12	3	1	17	4	1	3	1	4	5	5	2
19881	202	0	1-Jan-12	4	1	17	4	2	3	1	4	5	1	2
19882	202	0	5-Jul-11	20	1	17	4	3	3	1	4	5	5	1
19883	202	0	17-Jun-06	1	1	17	4	1	3	1	6	5	5	2
19884	202	0	30-Mar-09	23	2	17	4	3	3	1	4	5	4	1
19885	201	1	3-Jul-07	23	1	17	4	3	3	1	13	5	1	1
19886	101	1	13-Dec-13	20	1	17	4	2	3	1	4	5	5	1
19887	101	1	17-Dec-12	17	2	17	4	2	3	1	4	5	1	1
19888	101	1	1-Feb-15	23	2	17	4	2	3	1	13	5	1	1
19889	101	1	15-Aug-12	1	1	15	4	2	2	1	4	5	2	1
19890	101	1	15-Jul-13	20	2	16	4	1	2	1	6	5	2	1
19891	202	0	4-Jan-07	8	2	17	4	3	2	1	3	5	3	2
19892	202	0	3-Sep-08	10	2	14	4	1	1	0	6	5	2	1
19893	101	1	23-Sep-14	14	1	14	4	2	1	0	13	5	2	2
19894	202	0	14-Apr-11	20	2	15	4	3	1	0	11	5	2	1
19895	101	1	2-Oct-17	19	1	15	4	1	1	0	11	5	2	1
19896	202	0	24-Dec-17	16	1	16	4	3	1	0	7	5	3	1
19897	202	0	2-Nov-10	23	1	16	4	1	1	0	11	5	3	2
19898	202	0	15-Sep-09	8	1	16	4	1	1	0	3	5	2	1
19899	202	0	8-Apr-09	18	1	16	4	1	1	0	3	5	1	1
19900	202	0	30-Sep-09	17	1	16	4	1	1	0	6	5	1	1
19901	202	0	11-Aug-08	12	1	16	4	3	1	0	13	5	2	2
19902	101	1	30-Nov-15	12	1	16	4	1	1	0	7	5	1	2
19903	101	1	25-Apr-11	9	1	16	4	3	1	0	3	5	3	1
19904	202	0	22-Aug-09	9	1	17	4	2	1	0	7	5	2	2
19905	202	0	17-May-09	22	1	17	4	2	1	0	13	5	7	2
19906	202	0	9-Oct-07	14	1	17	4	1	1	0	13	5	1	1
19907	202	0	3-Aug-16	2	1	17	4	2	1	0	4	5	1	2
19908	202	0	11-Dec-11	10	1	17	4	3	1	0	4	5	3	2
19909	202	0	23-Nov-08	20	1	17	4	3	1	0	4	5	3	2
19910	202	0	5-Sep-08	8	1	17	4	1	1	0	3	5	6	2
19911	202	0	20-Sep-09	16	1	17	4	3	1	0	6	5	1	1
19912	202	0	7-May-08	14	1	17	4	1	1	0	6	5	2	2
19913	202	0	16-Apr-08	11	1	17	4	2	1	0	6	5	2	1
19914	202	0	11-Apr-09	9	1	17	4	3	1	0	13	5	2	2
19915	202	0	29-Apr-08	11	1	17	4	2	1	0	13	5	2	1
19916	101	1	29-Mar-11	20	1	17	4	2	1	0	6	5	2	1
19917	201	1	18-Nov-06	23	1	17	4	3	1	0	13	5	5	1
19918	201	1	19-Feb-12	20	2	17	4	1	1	0	10	5	7	1
19919	202	0	24-Dec-08	22	1	15	4	1	3	1	4	4	6	2
19920	201	1	4-Oct-07	0	1	15	4	3	3	1	6	4	2	1
19921	202	0	30-Nov-09	4	2	16	4	1	3	1	4	4	1	2
19922	101	1	25-Oct-14	2	1	16	4	2	3	1	4	4	5	4
19923	201	1	11-Jan-17	0	1	16	4	3	3	1	4	4	5	1
19924	101	1	8-Jun-13	22	2	16	4	1	3	1	6	4	2	1
19925	202	0	2-Aug-06	21	1	17	4	3	3	1	13	4	6	1
19926	202	0	25-Dec-16	4	1	17	4	1	3	1	4	4	1	1
19927	202	0	23-Apr-12	1	1	17	4	1	3	1	6	4	4	2
19928	202	0	1-Sep-09	2	1	17	4	1	3	1	6	4	6	2
19929	202	0	1-Sep-09	2	1	17	4	1	3	1	6	4	6	2
19930	202	0	2-Jul-08	23	1	17	4	3	3	1	6	4	2	2
19931	202	0	2-Jul-08	23	1	17	4	3	3	1	6	4	6	2
19932	202	0	28-Nov-09	18	1	17	4	1	3	1	13	4	6	2
19933	202	0	17-Jul-10	2	2	17	4	1	3	1	4	4	1	1
19934	201	1	3-May-16	0	1	17	4	3	3	1	6	4	1	1
19935	201	1	28-Jun-08	23	2	15	4	3	2	1	10	4	7	1
19936	202	0	9-Dec-14	8	2	11	4	2	1	0	3	4	1	1
19937	202	0	30-Mar-11	9	1	12	4	1	1	0	13	4	1	1
19938	202	0	21-Sep-17	14	1	14	4	2	1	0	4	4	1	1
19939	202	0	24-Dec-11	20	2	14	4	1	1	0	10	4	7	1
19940	202	0	5-Jun-17	8	1	15	4	2	1	0	4	4	1	1
19941	202	0	17-Sep-08	23	2	15	4	1	1	0	10	4	7	2
19942	202	0	15-Apr-11	9	2	15	4	3	1	0	4	4	1	2
19943	202	0	6-Mar-06	10	2	15	4	3	1	0	6	4	2	1
19944	101	1	1-Jul-14	12	1	15	4	2	1	0	7	4	2	1
19945	101	1	17-Oct-09	23	1	15	4	3	1	0	6	4	2	1
19946	201	1	12-Sep-08	18	1	15	4	3	1	0	6	4	4	1
19947	202	0	6-Jun-16	9	1	16	4	2	1	0	7	4	1	1
19948	202	0	11-Apr-08	3	1	16	4	3	1	0	7	4	2	2
19949	202	0	20-Jul-15	8	1	16	4	1	1	0	13	4	1	1
19950	202	0	19-Sep-14	9	1	16	4	1	1	0	13	4	1	1
19951	202	0	15-Apr-09	16	1	16	4	3	1	0	13	4	1	1
19952	202	0	23-Mar-09	20	1	16	4	3	1	0	13	4	1	1
19953	202	0	29-Dec-08	10	1	16	4	3	1	0	4	4	2	1
19954	202	0	30-Jul-08	6	1	16	4	3	1	0	3	4	2	2
19955	202	0	21-May-16	8	1	16	4	3	1	0	6	4	1	1
19956	202	0	20-Jun-11	13	1	16	4	1	1	0	6	4	2	2
19957	202	0	26-Feb-08	3	1	16	4	1	1	0	6	4	1	1

19958	202	0	2-Aug-07	8	1	16	4	2	1	0	6	4	2	2
19959	202	0	18-Apr-06	23	1	16	4	2	1	0	13	4	1	1
19960	202	0	12-Apr-12	16	2	16	4	2	1	0	6	4	2	2
19961	201	1	17-Jan-14	9	1	16	4	3	1	0	6	4	3	1
19962	201	1	15-Sep-17	9	1	16	4	3	1	0	4	4	2	1
19963	201	1	17-Oct-09	20	1	16	4	3	1	0	10	4	7	2
19964	201	1	3-Feb-09	18	1	16	4	2	1	0	4	4	3	1
19965	201	1	24-Sep-06	19	1	16	4	3	1	0	4	4	2	1
19966	101	1	3-Jun-13	17	1	16	4	1	1	0	6	4	2	1
19967	201	1	3-Aug-11	21	1	16	4	1	1	0	6	4	6	2
19968	201	1	13-Apr-09	11	1	16	4	2	1	0	6	4	2	1
19969	201	1	6-Jul-08	14	1	16	4	2	1	0	6	4	2	1
19970	201	1	8-Oct-08	10	1	16	4	3	1	0	6	4	3	1
19971	201	1	18-Jul-12	20	2	16	4	1	1	0	10	4	7	2
19972	202	0	24-Nov-15	9	1	17	4	1	1	0	7	4	2	1
19973	202	0	21-Mar-12	19	1	17	4	3	1	0	6	4	2	2
19974	202	0	9-May-12	15	1	17	4	3	1	0	6	4	5	1
19975	202	0	12-Sep-09	9	1	17	4	3	1	0	7	4	2	2
19976	202	0	16-May-08	16	1	17	4	3	1	0	7	4	2	1
19977	202	0	18-May-17	10	1	17	4	2	1	0	7	4	1	1
19978	202	0	26-Aug-15	14	1	17	4	1	1	0	13	4	1	1
19979	202	0	17-Jul-14	11	1	17	4	3	1	0	13	4	1	1
19980	202	0	10-Jul-08	9	1	17	4	2	1	0	13	4	1	1
19981	202	0	25-May-08	15	1	17	4	3	1	0	13	4	6	2
19982	202	0	15-Dec-07	22	1	17	4	3	1	0	13	4	1	1
19983	202	0	28-Nov-09	19	1	17	4	3	1	0	1	4	3	2
19984	202	0	29-Dec-16	14	1	17	4	3	1	0	4	4	1	1
19985	202	0	10-Jan-07	19	1	17	4	3	1	0	4	4	2	1
19986	202	0	11-Jul-17	3	1	17	4	1	1	0	4	4	2	1
19987	202	0	23-Jul-12	2	1	17	4	1	1	0	6	4	2	2
19988	202	0	27-Mar-11	9	1	17	4	1	1	0	6	4	2	1
19989	202	0	28-Oct-11	15	1	17	4	3	1	0	6	4	2	2
19990	202	0	7-Nov-09	18	1	17	4	2	1	0	6	4	2	1
19991	202	0	29-Sep-15	15	2	17	4	1	1	0	6	4	2	1
19992	202	0	9-Dec-16	15	2	17	4	3	1	0	7	4	1	1
19993	202	0	10-Sep-16	14	2	17	4	1	1	0	7	4	1	1
19994	202	0	10-Sep-16	14	2	17	4	3	1	0	7	4	1	1
19995	202	0	10-Nov-14	8	2	17	4	1	1	0	13	4	1	1
19996	202	0	14-Oct-09	16	2	17	4	2	1	0	4	4	2	2
19997	202	0	6-Nov-09	21	2	17	4	3	1	0	3	4	2	2
19998	201	1	29-Jul-14	1	1	17	4	2	1	0	10	4	7	1
19999	201	1	29-Sep-14	1	1	17	4	2	1	0	10	4	7	1
20000	201	1	15-Jul-14	19	1	17	4	1	1	0	4	4	2	1
20001	201	1	10-Oct-12	17	1	17	4	3	1	0	4	4	3	2
20002	201	1	5-Jun-11	18	1	17	4	3	1	0	10	4	7	2
20003	201	1	14-Jul-11	20	1	17	4	1	1	0	7	4	6	2
20004	201	1	24-Sep-06	18	1	17	4	3	1	0	4	4	3	2
20005	201	1	27-Nov-08	15	1	17	4	3	1	0	3	4	2	2
20006	101	1	22-Apr-11	21	1	17	4	2	1	0	6	4	2	1
20007	201	1	25-Oct-11	5	1	17	4	1	1	0	6	4	2	2
20008	201	1	17-Jun-15	2	2	17	4	3	1	0	3	4	2	1
20009	202	0	21-Dec-08	17	1	15	4	1	3	1	6	3	1	2
20010	202	0	16-May-09	2	1	16	4	2	3	1	4	3	1	2
20011	202	0	15-Jun-12	0	1	16	4	3	3	1	6	3	3	2
20012	202	0	21-Jun-08	5	1	16	4	1	3	1	13	3	6	2
20013	202	0	22-Dec-12	1	1	17	4	1	3	1	4	3	1	1
20014	202	0	23-Apr-12	1	1	17	4	1	3	1	6	3	3	2
20015	202	0	8-Jun-09	3	2	17	4	2	3	1	4	3	6	2
20016	202	0	14-Oct-08	17	1	10	4	1	1	0	1	3	4	1
20017	202	0	5-Feb-11	14	2	12	4	1	1	0	1	3	6	1
20018	202	0	22-Nov-17	19	1	13	4	1	1	0	1	3	3	2
20019	202	0	16-Dec-11	7	1	13	4	1	1	0	1	3	3	1
20020	202	0	25-Oct-09	23	1	13	4	1	1	0	1	3	1	1
20021	202	0	25-Mar-09	8	1	13	4	1	1	0	1	3	1	1
20022	202	0	15-Jul-16	17	1	13	4	3	1	0	4	3	2	1
20023	202	0	13-Jul-11	20	2	13	4	3	1	0	1	3	5	1
20024	202	0	13-Dec-11	22	2	13	4	3	1	0	1	3	3	1
20025	101	1	6-Apr-13	16	1	13	4	1	1	0	1	3	2	1
20026	101	1	20-Jun-09	19	1	13	4	2	1	0	1	3	1	1
20027	202	0	22-Jan-17	18	1	14	4	2	1	0	1	3	1	1
20028	202	0	6-Feb-11	0	1	14	4	2	1	0	1	3	6	2
20029	202	0	6-Feb-11	2	1	14	4	2	1	0	1	3	6	2
20030	202	0	25-Dec-10	7	1	14	4	1	1	0	1	3	5	1
20031	202	0	25-Dec-10	7	1	14	4	1	1	0	1	3	5	1
20032	202	0	26-Nov-10	13	1	14	4	1	1	0	1	3	5	2
20033	101	1	26-Jul-10	16	1	14	4	1	1	0	3	3	2	1
20034	202	0	25-Jul-12	15	1	15	4	3	1	0	1	3	1	2
20035	202	0	20-Apr-12	19	1	15	4	1	1	0	1	3	3	2
20036	202	0	14-Jan-11	11	1	15	4	2	1	0	1	3	6	2
20037	202	0	2-May-11	1	1	15	4	2	1	0	1	3	1	2
20038	202	0	14-Jan-11	11	1	15	4	2	1	0	1	3	1	2
20039	202	0	11-Sep-11	23	1	15	4	3	1	0	1	3	3	2
20040	202	0	29-Mar-09	3	1	15	4	2	1	0	1	3	4	2
20041	202	0	23-Jul-08	21	1	15	4	1	1	0	1	3	2	1
20042	202	0	16-May-08	7	1	15	4	3	1	0	1	3	4	1
20043	202	0	23-Apr-08	7	1	15	4	3	1	0	1	3	2	2
20044	202	0	25-Apr-10	17	1	15	4	1	1	0	4	3	1	2
20045	202	0	8-Nov-12	21	1	15	4	1	1	0	6	3	3	2
20046	202	0	21-Jul-12	2	2	15	4	1	1	0	1	3	5	2
20047	202	0	12-Oct-12	2	2	15	4	1	1	0	1	3	5	2
20048	202	0	30-Dec-11	16	2	15	4	1	1	0	1	3	3	1
20049	202	0	23-Sep-10	0	2	15	4	1	1	0	1	3	6	2
20050	202	0	11-Jul-13	13	2	15	4	2	1	0	3	3	1	1
20051	201	1	28-Sep-13	0	1	15	4	3	1	0	7	3	3	1
20052	201	1	23-Aug-17	17	1	15	4	3	1	0	6	3	3	1
20053	101	1	4-Sep-10	23	1	15	4	2	1	0	3	3	2	4
20054	201	1	1-May-10	17	1	15	4	3	1	0	3	3	2	2
20055	101	1	27-Feb-15	21	2	15	4	2	1	0	1	3	3	2
20056	101	1	12-Jul-15	10	2	15	4	4	1	0	3	3	5	1
20057	202	0	20-Oct-15	9	1	16	4	3	1	0	1	3	1	1
20058	202	0	8-Aug-14	0	1	16	4	1	1	0	1	3	5	2
20059	202	0	30-Jul-14	16	1	16	4	3	1	0	1	3	3	1
20060	202	0	14-Nov-12	4	1	16	4	1	1	0	1	3	5	2
20061	202	0	21-Jul-12	2	1	16	4	1	1	0	1	3	5	2
20062	202	0	29-Jul-12	21	1	16	4	1	1	0	1	3	5	2
20063	202	0	6-Jul-12	4	1	16	4	1	1	0	1	3	2	1
20064	202	0	7-Jul-12	22	1	16	4	3	1	0	1	3	1	1
20065	202	0	22-Aug-12	1	1	16	4	1	1	0	1	3	3	2
20066	202	0	24-Jan-12	1	1	16	4	2	1	0	1	3	5	3
20067	202	0	11-Nov-12	20	1	16	4	3	1	0	1	3	1	2
20068	202	0	17-Jun-17	0	1	16	4	1	1	0	1	3	3	1
20069	202	0	21-Jul-12	3	1	16	4	1	1	0	1	3	5	2
20070	202	0	5-Aug-11	21	1	16	4	1	1	0	1	3	5	2
20071	202	0	1-Oct-11	15	1	16	4	1	1	0	1	3	3	1
20072	202	0	17-Aug-11	3	1	16	4	1	1	0	1	3	1	1
20073	202	0	27-Dec-11	19	1	16	4	3	1	0	1	3	3	1
20074	202	0	13-Sep-11	1	1	16	4	3	1	0	1	3	3	2
20075	202	0	17-Aug-11	3	1	16	4	1	1	0	1	3	2	1
20076	202	0	23-Jul-11	1	1	16	4	3	1	0	1	3	5	2
20077	202	0	6-Nov-10	4	1	16	4	3	1	0	1	3	2	1
20078	202	0	4-Oct-10	16	1	16	4	3	1	0	1	3	2	1
20079	202	0	8-Dec-10	14	1	16	4	1	1	0	1	3	3	1
20080	202	0	23-Sep-09	21	1	16	4	1	1	0	1	3	1	2
20081	202	0	20-Dec-09	0	1	16	4	1	1	0	1	3	1	1

20082	202	0	21-Mar-08	17	1	16	4	3	1	0	1	3	3	1
20083	202	0	31-Aug-08	12	1	16	4	3	1	0	1	3	2	2
20084	202	0	19-Jun-08	11	1	16	4	2	1	0	1	3	3	1
20085	202	0	3-Nov-07	3	1	16	4	1	1	0	1	3	6	2
20086	202	0	16-Aug-08	13	1	16	4	3	1	0	1	3	2	2
20087	202	0	10-Apr-12	21	1	16	4	1	1	0	3	3	3	2
20088	202	0	26-Mar-12	11	1	16	4	3	1	0	3	3	3	2
20089	202	0	17-Apr-09	23	1	16	4	2	1	0	3	3	6	2
20090	202	0	17-Apr-09	23	1	16	4	2	1	0	3	3	4	2
20091	202	0	17-Apr-09	23	1	16	4	2	1	0	3	3	7	2
20092	202	0	4-Dec-09	22	1	16	4	1	1	0	3	3	2	2
20093	202	0	24-Sep-08	23	1	16	4	1	1	0	3	3	4	1
20094	202	0	10-Apr-07	14	1	16	4	3	1	0	3	3	3	2
20095	202	0	8-Jul-12	18	2	16	4	1	1	0	1	3	5	1
20096	202	0	24-Oct-15	11	2	16	4	1	1	0	1	3	3	1
20097	202	0	13-Jul-12	22	2	16	4	3	1	0	1	3	5	1
20098	202	0	4-Apr-12	3	2	16	4	3	1	0	1	3	5	2
20099	202	0	24-Jun-12	0	2	16	4	2	1	0	1	3	3	2
20100	202	0	9-May-17	0	2	16	4	1	1	0	1	3	3	1
20101	202	0	13-Sep-11	3	2	16	4	1	1	0	1	3	5	2
20102	202	0	2-Aug-11	8	2	16	4	1	1	0	1	3	3	1
20103	202	0	7-May-11	16	2	16	4	1	1	0	1	3	2	1
20104	202	0	14-Jul-11	4	2	16	4	3	1	0	1	3	6	2
20105	202	0	17-Mar-10	2	2	16	4	1	1	0	1	3	6	1
20106	202	0	22-Nov-10	1	2	16	4	2	1	0	1	3	6	2
20107	202	0	3-Jul-09	23	2	16	4	1	1	0	1	3	6	1
20108	202	0	4-Apr-09	15	2	16	4	3	1	0	1	3	3	1
20109	202	0	9-Sep-06	13	2	16	4	3	1	0	1	3	5	1
20110	101	1	4-May-13	22	1	16	4	2	1	0	1	3	3	1
20111	101	1	4-May-13	22	1	16	4	2	1	0	1	3	1	1
20112	201	1	23-Feb-12	1	1	16	4	3	1	0	1	3	1	1
20113	101	1	17-Jul-12	23	1	16	4	1	1	0	1	3	5	2
20114	201	1	26-Dec-10	5	1	16	4	2	1	0	1	3	3	1
20115	101	1	3-Apr-09	14	1	16	4	3	1	0	1	3	3	1
20116	201	1	7-Aug-08	14	1	16	4	3	1	0	1	3	4	1
20117	101	1	9-Jan-06	8	1	16	4	1	1	0	1	3	3	1
20118	101	1	23-Oct-07	17	1	16	4	1	1	0	4	3	1	1
20119	201	1	6-Apr-10	20	1	16	4	3	1	0	3	3	1	2
20120	101	1	4-Aug-07	16	1	16	4	2	1	0	3	3	4	1
20121	101	1	19-May-13	22	2	16	4	1	1	0	7	3	3	1
20122	201	1	29-Mar-10	1	2	16	4	3	1	0	1	3	1	2
20123	202	0	25-Jul-12	15	1	17	4	2	1	0	1	3	3	2
20124	202	0	1-Nov-12	20	1	17	4	3	1	0	1	3	3	1
20125	202	0	24-Apr-12	12	1	17	4	1	1	0	1	3	2	2
20126	202	0	12-Jun-16	2	1	17	4	1	1	0	1	3	3	2
20127	202	0	29-May-16	4	1	17	4	2	1	0	1	3	2	1
20128	202	0	26-Jun-12	11	1	17	4	1	1	0	1	3	5	1
20129	202	0	13-Apr-12	19	1	17	4	1	1	0	1	3	5	1
20130	202	0	15-Dec-12	23	1	17	4	1	1	0	1	3	1	1
20131	202	0	14-Sep-12	22	1	17	4	1	1	0	1	3	5	2
20132	202	0	20-Oct-12	8	1	17	4	1	1	0	1	3	5	2
20133	202	0	22-Apr-12	16	1	17	4	1	1	0	1	3	5	2
20134	202	0	10-Aug-12	13	1	17	4	3	1	0	1	3	1	2
20135	202	0	21-Jul-12	2	1	17	4	1	1	0	1	3	1	2
20136	202	0	30-Nov-12	4	1	17	4	1	1	0	1	3	5	2
20137	202	0	18-Sep-12	20	1	17	4	1	1	0	1	3	3	2
20138	202	0	25-Jul-12	15	1	17	4	2	1	0	1	3	1	2
20139	202	0	27-Jan-12	9	1	17	4	3	1	0	1	3	1	2
20140	202	0	5-Jun-17	17	1	17	4	1	1	0	1	3	3	1
20141	202	0	9-Apr-17	14	1	17	4	3	1	0	1	3	3	1
20142	202	0	9-Nov-17	19	1	17	4	3	1	0	1	3	5	1
20143	202	0	24-Dec-17	22	1	17	4	1	1	0	1	3	7	1
20144	202	0	6-Jun-17	3	1	17	4	3	1	0	3	3	1	2
20145	202	0	20-Jul-17	20	1	17	4	1	1	0	3	3	5	1
20146	202	0	1-Dec-11	12	1	17	4	3	1	0	1	3	1	1
20147	202	0	3-Feb-11	1	1	17	4	1	1	0	1	3	1	2
20148	202	0	3-Apr-11	2	1	17	4	1	1	0	1	3	6	2
20149	202	0	24-Apr-11	21	1	17	4	1	1	0	1	3	5	1
20150	202	0	26-Oct-11	22	1	17	4	3	1	0	1	3	5	1
20151	202	0	22-Nov-11	17	1	17	4	1	1	0	1	3	5	1
20152	202	0	20-Nov-11	11	1	17	4	1	1	0	1	3	5	2
20153	202	0	20-Jan-11	21	1	17	4	1	1	0	1	3	6	2
20154	202	0	1-Dec-11	0	1	17	4	1	1	0	1	3	1	2
20155	202	0	25-Jan-11	0	1	17	4	1	1	0	1	3	1	1
20156	202	0	27-Oct-11	12	1	17	4	2	1	0	1	3	1	2
20157	202	0	14-May-11	23	1	17	4	3	1	0	1	3	1	1
20158	202	0	17-Aug-11	2	1	17	4	1	1	0	1	3	1	1
20159	202	0	16-Jan-11	3	1	17	4	1	1	0	1	3	1	1
20160	202	0	19-Jan-11	21	1	17	4	1	1	0	1	3	2	1
20161	202	0	24-Aug-11	2	1	17	4	1	1	0	1	3	3	2
20162	202	0	17-Sep-11	14	1	17	4	1	1	0	1	3	3	2
20163	202	0	17-Aug-11	1	1	17	4	1	1	0	1	3	3	1
20164	202	0	25-Mar-11	20	1	17	4	1	1	0	1	3	2	1
20165	202	0	26-Oct-11	22	1	17	4	3	1	0	1	3	3	1
20166	202	0	8-Sep-11	19	1	17	4	1	1	0	1	3	3	1
20167	202	0	20-Jun-11	7	1	17	4	1	1	0	1	3	3	2
20168	202	0	14-Jul-11	4	1	17	4	3	1	0	1	3	6	2
20169	202	0	6-Feb-11	2	1	17	4	2	1	0	1	3	4	2
20170	202	0	17-Apr-11	4	1	17	4	2	1	0	1	3	1	2
20171	202	0	2-Aug-11	16	1	17	4	3	1	0	1	3	3	2
20172	202	0	14-Sep-10	21	1	17	4	1	1	0	1	3	3	2
20173	202	0	9-Dec-10	18	1	17	4	1	1	0	1	3	3	2
20174	202	0	30-Nov-09	3	1	17	4	2	1	0	1	3	6	2
20175	202	0	3-Oct-09	1	1	17	4	1	1	0	1	3	1	1
20176	202	0	15-Apr-09	15	1	17	4	2	1	0	1	3	2	1
20177	202	0	21-Oct-08	13	1	17	4	3	1	0	1	3	2	2
20178	202	0	27-Nov-08	8	1	17	4	3	1	0	1	3	3	2
20179	202	0	16-Dec-07	17	1	17	4	2	1	0	1	3	3	1
20180	202	0	1-May-09	21	1	17	4	2	1	0	1	3	6	2
20181	202	0	17-Apr-09	23	1	17	4	2	1	0	1	3	6	2
20182	202	0	27-Mar-06	13	1	17	4	3	1	0	1	3	2	2
20183	202	0	22-May-17	0	1	17	4	1	1	0	4	3	2	1
20184	202	0	15-May-11	1	1	17	4	1	1	0	3	3	1	2
20185	202	0	30-Jul-11	1	1	17	4	1	1	0	3	3	5	1
20186	202	0	15-May-11	1	1	17	4	1	1	0	3	3	5	1
20187	202	0	15-May-11	1	1	17	4	1	1	0	3	3	1	1
20188	202	0	17-Oct-11	6	1	17	4	1	1	0	3	3	3	2
20189	202	0	24-Jul-09	21	1	17	4	3	1	0	3	3	2	1
20190	202	0	17-Oct-09	22	1	17	4	1	1	0	3	3	4	2
20191	202	0	8-May-09	20	1	17	4	2	1	0	3	3	2	2
20192	202	0	7-Jul-08	9	1	17	4	3	1	0	3	3	2	2
20193	202	0	26-Jun-17	0	1	17	4	1	1	0	3	3	6	1
20194	202	0	31-Dec-12	23	2	17	4	1	1	0	1	3	1	1
20195	202	0	17-Apr-16	4	2	17	4	3	1	0	1	3	1	2
20196	202	0	24-Jun-12	0	2	17	4	1	1	0	1	3	3	2
20197	202	0	1-Sep-12	15	2	17	4	1	1	0	1	3	3	2
20198	202	0	15-Aug-11	2	2	17	4	3	1	0	1	3	2	2
20199	202	0	13-Sep-11	1	2	17	4	1	1	0	1	3	6	2
20200	202	0	4-Dec-11	15	2	17	4	3	1	0	1	3	5	2
20201	202	0	1-Oct-11	3	2	17	4	3	1	0	1	3	3	2
20202	202	0	28-Aug-10	12	2	17	4	2	1	0	1	3	6	1
20203	202	0	6-Nov-10	23	2	17	4	2	1	0	1	3	1	2
20204	202	0	30-Apr-09	1	2	17	4	1	1	0	1	3	7	1
20205	202	0	4-Dec-09	1	2	17	4	3	1	0	1	3	3	1

20206	202	0	24-May-08	10	2	17	4	1	1	0	1	3	5	1
20207	202	0	9-May-08	18	2	17	4	3	1	0	1	3	1	1
20208	202	0	28-Jul-06	22	2	17	4	1	1	0	1	3	5	1
20209	202	0	10-Aug-08	13	2	17	4	2	1	0	4	3	3	1
20210	202	0	3-Oct-09	1	2	17	4	1	1	0	3	3	6	1
20211	202	0	27-Sep-08	21	2	17	4	1	1	0	3	3	6	1
20212	201	1	4-Dec-16	6	1	17	4	3	1	0	1	3	5	1
20213	201	1	21-Dec-14	10	1	17	4	2	1	0	1	3	1	2
20214	201	1	6-Mar-14	7	1	17	4	2	1	0	1	3	3	2
20215	201	1	28-Jul-09	4	1	17	4	1	1	0	1	3	7	2
20216	201	1	3-Oct-11	18	1	17	4	1	1	0	1	3	6	2
20217	101	1	5-Sep-15	1	1	17	4	1	1	0	3	3	5	1
20218	201	1	25-Nov-10	17	1	17	4	2	1	0	3	3	3	1
20219	201	1	24-Nov-09	20	1	17	4	3	1	0	3	3	2	2
20220	201	1	4-Apr-06	21	1	17	4	3	1	0	6	3	1	1
20221	201	1	14-Oct-06	9	1	17	4	3	1	0	6	3	3	1
20222	101	1	26-Oct-14	21	2	17	4	2	1	0	1	3	4	2
20223	201	1	6-Dec-10	6	2	17	4	1	1	0	1	3	6	1
20224	201	1	2-Aug-10	13	2	17	4	2	1	0	4	3	1	1
20225	202	0	18-May-17	0	1	14	4	3	3	1	6	2	2	1
20226	202	0	15-Dec-07	16	1	15	4	2	3	1	4	2	4	1
20227	202	0	22-Jul-14	0	1	16	4	1	3	1	6	2	5	1
20228	202	0	20-Mar-16	5	1	16	4	3	3	1	4	2	1	1
20229	202	0	24-Jan-11	19	1	16	4	2	3	1	4	2	1	2
20230	202	0	31-Jan-11	19	1	16	4	1	3	1	4	2	1	2
20231	202	0	15-Jun-10	18	1	16	4	2	3	1	4	2	7	1
20232	202	0	7-Feb-07	4	1	16	4	3	3	1	4	2	4	2
20233	202	0	18-Jul-07	1	1	16	4	1	3	1	4	2	1	1
20234	202	0	23-Sep-11	12	1	16	4	1	3	1	6	2	1	2
20235	202	0	25-Dec-11	3	1	16	4	1	3	1	6	2	1	2
20236	202	0	16-Aug-15	14	2	16	4	1	3	1	4	2	4	1
20237	202	0	10-Jun-07	2	2	16	4	1	3	1	4	2	1	1
20238	202	0	10-Nov-07	10	2	16	4	1	3	1	4	2	6	2
20239	201	1	4-Sep-14	2	1	16	4	2	3	1	4	2	1	2
20240	201	1	15-Dec-10	20	1	16	4	1	3	1	6	2	1	2
20241	202	0	22-Jun-12	3	1	17	4	1	3	1	6	2	1	2
20242	202	0	22-Apr-12	2	1	17	4	3	3	1	6	2	4	1
20243	202	0	16-Feb-17	9	1	17	4	2	3	1	12	2	1	1
20244	202	0	6-Jul-11	1	1	17	4	3	3	1	4	2	5	1
20245	202	0	20-Apr-11	23	1	17	4	1	3	1	4	2	1	2
20246	202	0	17-Jul-11	22	1	17	4	1	3	1	4	2	1	1
20247	202	0	12-Jul-08	22	1	17	4	1	3	1	4	2	6	1
20248	202	0	18-Mar-08	22	1	17	4	3	3	1	4	2	1	1
20249	202	0	12-Feb-08	23	1	17	4	1	3	1	4	2	1	1
20250	202	0	8-Dec-07	1	1	17	4	1	3	1	4	2	1	1
20251	202	0	18-Jun-07	18	1	17	4	3	3	1	4	2	1	1
20252	202	0	20-Mar-07	21	1	17	4	3	3	1	4	2	1	2
20253	202	0	19-Feb-07	1	1	17	4	1	3	1	4	2	1	1
20254	202	0	24-Sep-06	3	1	17	4	1	3	1	4	2	5	1
20255	202	0	18-Feb-11	23	1	17	4	1	3	1	6	2	5	2
20256	202	0	20-Jun-10	3	1	17	4	1	3	1	6	2	2	2
20257	202	0	2-Feb-07	1	1	17	4	3	3	1	6	2	1	1
20258	202	0	23-Feb-07	1	1	17	4	3	3	1	6	2	1	1
20259	202	0	7-Aug-06	3	1	17	4	1	3	1	6	2	4	1
20260	202	0	31-Jul-06	2	1	17	4	1	3	1	6	2	5	2
20261	202	0	3-Nov-06	0	1	17	4	3	3	1	6	2	4	2
20262	202	0	8-Oct-10	1	1	17	4	1	3	1	13	2	1	1
20263	202	0	26-Oct-12	0	2	17	4	3	3	1	4	2	6	2
20264	202	0	12-Jul-11	4	2	17	4	1	3	1	11	2	2	2
20265	202	0	13-Oct-12	2	2	17	4	3	3	1	3	2	5	2
20266	201	1	6-Dec-10	5	1	17	4	1	3	1	6	2	4	2
20267	201	1	28-Jun-06	4	1	17	4	2	3	1	6	2	2	1
20268	201	1	21-Nov-13	11	2	17	4	3	3	1	6	2	3	2
20269	202	0	2-Oct-07	1	1	16	4	1	2	1	10	2	7	2
20270	101	1	17-Aug-08	10	1	16	4	3	2	1	13	2	7	4
20271	101	1	16-Sep-12	20	1	16	4	2	2	1	6	2	2	1
20272	202	0	20-Jan-11	19	1	11	4	2	1	0	3	2	2	2
20273	202	0	3-Nov-17	14	2	11	4	3	1	0	4	2	1	1
20274	202	0	21-Jul-07	2	2	12	4	1	1	0	6	2	1	1
20275	202	0	17-Nov-16	15	1	13	4	2	1	0	4	2	1	1
20276	202	0	30-Dec-11	20	1	13	4	1	1	0	4	2	3	2
20277	202	0	6-Dec-17	11	1	13	4	2	1	0	4	2	1	1
20278	202	0	6-Sep-11	17	2	13	4	3	1	0	7	2	1	1
20279	202	0	15-Jul-16	8	2	13	4	2	1	0	13	2	1	1
20280	201	1	9-Apr-14	18	1	13	4	3	1	0	3	2	1	1
20281	101	1	30-Nov-06	17	1	13	4	1	1	0	3	2	1	1
20282	202	0	13-Jan-06	12	1	14	4	1	1	0	7	2	2	1
20283	202	0	24-Dec-11	23	1	14	4	3	1	0	1	2	1	2
20284	202	0	16-Apr-09	0	1	14	4	1	1	0	4	2	1	2
20285	202	0	12-Dec-17	11	1	14	4	3	1	0	4	2	1	1
20286	202	0	1-Jan-11	22	1	14	4	1	1	0	3	2	1	2
20287	202	0	26-Sep-08	12	1	14	4	1	1	0	3	2	1	1
20288	202	0	11-Aug-06	19	1	14	4	1	1	0	3	2	2	1
20289	202	0	9-Nov-16	8	1	14	4	1	1	0	13	2	1	1
20290	202	0	25-Oct-11	19	2	14	4	1	1	0	1	2	1	2
20291	202	0	14-May-11	23	2	14	4	1	1	0	6	2	2	1
20292	202	0	22-Jan-12	14	1	15	4	3	1	0	1	2	6	2
20293	202	0	5-Jan-16	3	1	15	4	2	1	0	4	2	4	1
20294	202	0	15-Jan-11	19	1	15	4	1	1	0	10	2	7	2
20295	202	0	29-Apr-11	10	1	15	4	2	1	0	7	2	1	2
20296	202	0	20-Jul-15	8	1	15	4	1	1	0	13	2	1	1
20297	202	0	10-Nov-14	8	1	15	4	2	1	0	13	2	1	1
20298	202	0	26-Dec-10	0	1	15	4	2	1	0	4	2	2	2
20299	202	0	17-Oct-10	20	1	15	4	1	1	0	3	2	5	2
20300	202	0	2-Nov-08	17	1	15	4	2	1	0	3	2	2	2
20301	202	0	3-Aug-11	12	1	15	4	1	1	0	6	2	2	2
20302	202	0	6-Nov-11	20	2	15	4	1	1	0	1	2	5	2
20303	202	0	6-Nov-11	20	2	15	4	1	1	0	3	2	5	2
20304	201	1	18-Jan-10	12	1	15	4	1	1	0	11	2	3	1
20305	201	1	17-Jan-14	19	1	15	4	3	1	0	3	2	5	1
20306	201	1	28-Dec-11	20	1	15	4	3	1	0	3	2	1	1
20307	101	1	18-Oct-09	18	1	15	4	3	1	0	6	2	2	1
20308	201	1	8-Aug-12	14	2	15	4	3	1	0	6	2	2	1
20309	101	1	7-Jan-06	12	2	15	4	1	1	0	3	2	2	1
20310	202	0	31-May-17	10	1	16	4	2	1	0	1	2	5	1
20311	202	0	14-Dec-16	19	1	16	4	3	1	0	10	2	7	2
20312	202	0	14-Jul-16	8	1	16	4	2	1	0	13	2	1	1
20313	202	0	14-Aug-16	9	1	16	4	1	1	0	13	2	1	1
20314	202	0	25-Oct-16	9	1	16	4	1	1	0	13	2	1	1
20315	202	0	18-Jan-11	22	1	16	4	2	1	0	4	2	1	2
20316	202	0	10-Nov-10	17	1	16	4	1	1	0	4	2	1	1
20317	202	0	24-Dec-17	14	1	16	4	1	1	0	4	2	1	1
20318	202	0	9-Nov-17	14	1	16	4	2	1	0	4	2	1	1
20319	202	0	12-Jul-12	12	1	16	4	3	1	0	3	2	5	2
20320	202	0	10-Sep-12	18	1	16	4	3	1	0	3	2	4	2
20321	202	0	27-May-08	17	1	16	4	3	1	0	3	2	2	1
20322	202	0	19-Jan-11	0	1	16	4	2	1	0	6	2	5	2
20323	202	0	28-Jul-11	19	1	16	4	1	1	0	6	2	2	1
20324	202	0	22-Dec-10	12	1	16	4	1	1	0	13	2	3	1
20325	202	0	20-Nov-16	9	2	16	4	2	1	0	13	2	1	1
20326	202	0	7-Oct-17	16	2	16	4	2	1	0	13	2	1	1
20327	202	0	1-Dec-11	0	2	16	4	1	1	0	1	2	3	1
20328	202	0	20-Feb-11	17	2	16	4	2	1	0	4	2	1	2
20329	202	0	31-May-11	10	2	16	4	1	1	0	11	2	3	1

20330	202	0	6-Sep-11	15	2	16	4	1	1	0	3	2	1	2
20331	202	0	15-Oct-06	19	2	16	4	3	1	0	3	2	2	1
20332	202	0	23-Feb-17	15	2	16	4	1	1	0	13	2	1	1
20333	101	1	15-Apr-13	22	1	16	4	1	1	0	11	2	2	1
20334	201	1	12-May-11	10	1	16	4	1	1	0	3	2	4	1
20335	101	1	12-Oct-13	22	2	16	4	1	1	0	3	2	1	1
20336	202	0	29-Feb-12	19	1	17	4	3	1	0	10	2	7	2
20337	202	0	11-Aug-11	22	1	17	4	3	1	0	10	2	7	2
20338	202	0	2-Jan-11	1	1	17	4	2	1	0	10	2	7	2
20339	202	0	19-Sep-11	0	1	17	4	3	1	0	10	2	7	2
20340	202	0	13-Feb-12	23	1	17	4	2	1	0	13	2	1	1
20341	202	0	30-May-17	0	1	17	4	2	1	0	13	2	1	1
20342	202	0	20-May-17	12	1	17	4	2	1	0	13	2	1	1
20343	202	0	20-Aug-08	22	1	17	4	3	1	0	1	2	2	1
20344	202	0	9-Oct-11	4	1	17	4	3	1	0	1	2	3	2
20345	202	0	15-Oct-11	3	1	17	4	1	1	0	1	2	6	2
20346	202	0	31-Mar-10	22	1	17	4	1	1	0	4	2	1	2
20347	202	0	17-Mar-09	13	1	17	4	2	1	0	4	2	2	2
20348	202	0	5-May-09	20	1	17	4	2	1	0	4	2	2	2
20349	202	0	25-Oct-17	14	1	17	4	2	1	0	4	2	1	1
20350	202	0	5-Jul-12	16	1	17	4	3	1	0	11	2	3	2
20351	202	0	24-Oct-14	8	1	17	4	1	1	0	3	2	1	1
20352	202	0	20-Mar-13	13	1	17	4	1	1	0	3	2	1	1
20353	202	0	21-Jul-12	0	1	17	4	3	1	0	3	2	2	2
20354	202	0	15-Jan-12	1	1	17	4	1	1	0	3	2	1	1
20355	202	0	29-Oct-11	22	1	17	4	3	1	0	3	2	5	2
20356	202	0	28-Feb-11	22	1	17	4	2	1	0	3	2	1	2
20357	202	0	27-Oct-11	0	1	17	4	2	1	0	3	2	1	2
20358	202	0	10-Nov-11	15	1	17	4	3	1	0	3	2	1	2
20359	202	0	20-Nov-10	22	1	17	4	2	1	0	3	2	2	2
20360	202	0	17-Apr-09	23	1	17	4	2	1	0	3	2	6	2
20361	202	0	1-Jun-09	1	1	17	4	1	1	0	3	2	1	2
20362	202	0	16-Jun-09	19	1	17	4	2	1	0	3	2	2	2
20363	202	0	15-Nov-08	12	1	17	4	1	1	0	3	2	2	2
20364	202	0	28-Oct-08	17	1	17	4	2	1	0	3	2	2	2
20365	202	0	14-Sep-07	9	1	17	4	3	1	0	3	2	5	2
20366	202	0	21-Feb-07	0	1	17	4	3	1	0	3	2	1	1
20367	202	0	9-Nov-06	22	1	17	4	1	1	0	3	2	7	1
20368	202	0	12-Jul-12	3	1	17	4	3	1	0	6	2	3	2
20369	202	0	10-Oct-12	0	1	17	4	3	1	0	6	2	2	2
20370	202	0	1-Jun-11	10	1	17	4	1	1	0	6	2	2	1
20371	202	0	30-Dec-11	21	1	17	4	3	1	0	6	2	6	2
20372	202	0	18-Sep-11	13	1	17	4	1	1	0	6	2	2	2
20373	202	0	20-Feb-10	3	2	17	4	1	1	0	10	2	7	2
20374	202	0	20-Aug-07	2	2	17	4	3	1	0	1	2	1	2
20375	202	0	9-Sep-11	19	2	17	4	3	1	0	1	2	1	2
20376	202	0	30-Sep-11	12	2	17	4	3	1	0	1	2	1	2
20377	202	0	9-Feb-10	0	2	17	4	3	1	0	4	2	1	1
20378	202	0	9-Aug-11	1	2	17	4	1	1	0	11	2	5	2
20379	202	0	13-Mar-16	9	2	17	4	1	1	0	3	2	1	1
20380	202	0	11-Dec-15	3	2	17	4	1	1	0	3	2	1	1
20381	202	0	6-Aug-12	3	2	17	4	1	1	0	3	2	5	2
20382	202	0	27-Aug-12	2	2	17	4	3	1	0	3	2	3	1
20383	202	0	7-Feb-11	22	2	17	4	1	1	0	3	2	1	1
20384	202	0	7-Aug-11	3	2	17	4	1	1	0	3	2	6	2
20385	202	0	28-Oct-11	12	2	17	4	3	1	0	3	2	2	2
20386	202	0	2-Nov-11	1	2	17	4	2	1	0	3	2	1	2
20387	202	0	20-Jul-11	17	2	17	4	1	1	0	3	2	3	1
20388	201	1	14-Apr-11	0	1	17	4	1	1	0	4	2	1	1
20389	201	1	11-May-10	7	1	17	4	1	1	0	4	2	3	1
20390	101	1	6-Oct-09	6	1	17	4	3	1	0	4	2	1	1
20391	101	1	26-May-13	10	1	17	4	1	1	0	3	2	1	1
20392	201	1	2-Mar-11	20	1	17	4	2	1	0	3	2	2	1
20393	201	1	6-Dec-10	13	1	17	4	2	1	0	3	2	4	4
20394	201	1	13-Nov-10	13	1	17	4	1	1	0	3	2	2	1
20395	101	1	2-Feb-07	10	1	17	4	1	1	0	3	2	4	1
20396	101	1	9-Nov-16	12	1	17	4	1	1	0	6	2	2	1
20397	101	1	3-Oct-09	18	2	17	4	3	1	0	3	2	5	1
20398	201	1	13-Jan-07	20	2	17	4	3	1	0	13	2	2	1
20399	202	0	1-Dec-08	20	2	15	4	3	3	1	10	1	7	2
20400	202	0	27-Sep-07	21	2	17	4	3	3	1	13	1	1	1
20401	201	1	26-Jan-09	4	2	17	4	3	3	1	4	1	1	1
20402	202	0	9-Jun-08	14	1	15	4	3	2	1	10	1	7	2
20403	202	0	2-Apr-09	22	1	17	4	1	2	1	10	1	7	2
20404	201	1	2-Oct-09	18	2	17	4	3	2	1	4	1	2	1
20405	201	1	17-Oct-13	2	2	17	4	2	2	1	6	1	2	1
20406	202	0	16-Feb-13	10	2	12	4	1	1	0	11	1	3	1
20407	101	1	11-Nov-17	10	1	12	4	1	1	0	11	1	2	1
20408	202	0	19-Oct-11	8	2	13	4	1	1	0	11	1	2	2
20409	202	0	23-Jun-09	11	2	13	4	2	1	0	3	1	1	2
20410	101	1	28-Nov-11	17	1	13	4	1	1	0	11	1	2	1
20411	201	1	3-May-10	8	1	13	4	3	1	0	11	1	2	1
20412	101	1	24-Nov-17	9	1	13	4	1	1	0	13	1	3	1
20413	201	1	10-Oct-09	9	2	13	4	2	1	0	11	1	2	1
20414	202	0	23-Dec-10	22	2	14	4	1	1	0	10	1	7	1
20415	202	0	28-Dec-08	13	2	14	4	1	1	0	6	1	2	2
20416	101	1	28-Apr-12	19	1	14	4	2	1	0	6	1	2	1
20417	202	0	26-Jun-13	16	1	15	4	3	1	0	1	1	1	1
20418	202	0	13-Mar-16	7	1	15	4	3	1	0	10	1	6	2
20419	202	0	1-Feb-14	22	1	15	4	1	1	0	6	1	2	1
20420	201	1	31-Jan-14	10	2	15	4	3	1	0	11	1	2	1
20421	101	1	19-Mar-11	20	2	15	4	1	1	0	6	1	3	1
20422	202	0	8-May-12	21	1	16	4	1	1	0	6	1	3	1
20423	202	0	21-Jul-16	8	1	16	4	1	1	0	13	1	1	1
20424	202	0	8-Oct-16	8	1	16	4	1	1	0	13	1	1	1
20425	202	0	16-Dec-08	21	1	16	4	3	1	0	4	1	2	2
20426	202	0	14-Aug-17	4	1	16	4	2	1	0	4	1	6	1
20427	202	0	1-Sep-12	15	1	16	4	1	1	0	11	1	2	2
20428	202	0	30-Jul-12	18	1	16	4	1	1	0	11	1	2	2
20429	202	0	24-Sep-09	22	1	16	4	3	1	0	11	1	3	2
20430	202	0	12-Jun-17	13	1	16	4	2	1	0	11	1	2	1
20431	202	0	9-May-09	14	1	16	4	2	1	0	3	1	1	2
20432	202	0	5-Sep-08	5	1	16	4	1	1	0	3	1	2	2
20433	202	0	14-Aug-06	21	1	16	4	2	1	0	3	1	2	1
20434	202	0	13-Jan-10	19	2	16	4	1	1	0	3	1	6	1
20435	201	1	24-May-13	20	1	16	4	2	1	0	6	1	5	1
20436	201	1	24-Jul-11	10	1	16	4	3	1	0	1	1	3	1
20437	201	1	11-Feb-14	22	1	16	4	3	1	0	4	1	2	1
20438	201	1	28-Jan-11	14	1	16	4	3	1	0	11	1	2	1
20439	101	1	10-Nov-17	19	1	16	4	1	1	0	6	1	2	1
20440	101	1	23-Nov-11	11	2	16	4	1	1	0	3	1	2	1
20441	202	0	2-Feb-07	23	1	17	4	2	1	0	10	1	7	1
20442	202	0	9-Aug-10	12	1	17	4	3	1	0	3	1	2	2
20443	202	0	17-Jul-15	20	2	17	4	3	1	0	4	1	1	1
20444	202	0	4-Nov-09	22	2	17	4	3	1	0	10	1	7	2
20445	202	0	4-Dec-09	20	2	17	4	1	1	0	10	1	7	2
20446	202	0	15-May-17	10	2	17	4	1	1	0	4	1	1	1
20447	202	0	9-Jun-16	18	2	17	4	1	1	0	11	1	2	1
20448	202	0	10-Jan-08	8	2	17	4	1	1	0	3	1	1	1
20449	201	1	7-Jun-14	22	1	17	4	1	1	0	4	1	2	1
20450	201	1	21-Apr-06	18	1	17	4	3	1	0	7	1	2	1
20451	101	1	17-Apr-11	1	1	17	4	3	1	0	4	1	1	1
20452	201	1	27-May-10	10	1	17	4	3	1	0	11	1	4	1
20453	201	1	26-Jan-09	17	2	17	4	3	1	0	10	1	7	2

20454	201	1	24-Dec-09	9	2	17	4	2	1	0	11	1	2	1
20455	101	1	27-Jul-09	2	2	17	4	3	1	0	6	1	3	1
20456	101	1	15-Nov-10	22	2	14	4	3	3	1	4	5	1	1
20457	202	0	2-Apr-06	2	1	16	4	1	3	1	6	5	6	2
20458	202	0	19-May-10	2	1	16	4	1	1	0	1	5	3	1
20459	202	0	19-Feb-11	1	1	16	4	1	1	0	6	5	2	2
20460	202	0	19-Feb-11	1	1	17	4	1	1	0	6	5	1	2
20461	101	1	19-Aug-06	22	1	15	4	2	3	1	6	4	2	2
20462	202	0	8-Dec-14	22	1	16	4	3	3	1	4	4	1	1
20463	202	0	22-Oct-07	0	2	17	4	2	3	1	4	4	1	1
20464	101	1	6-Sep-07	1	1	17	4	2	3	1	4	4	5	1
20465	101	1	18-Nov-06	20	1	14	4	3	1	0	6	4	2	1
20466	202	0	23-Sep-09	18	1	16	4	1	1	0	11	4	3	2
20467	202	0	24-Apr-11	22	2	17	4	3	3	1	4	3	1	2
20468	202	0	3-Nov-12	18	1	15	4	3	1	0	1	3	3	2
20469	202	0	3-Nov-12	18	1	15	4	3	1	0	1	3	3	2
20470	202	0	11-Dec-10	17	1	15	4	1	1	0	1	3	1	1
20471	202	0	4-Mar-08	4	1	15	4	1	1	0	1	3	1	2
20472	202	0	15-Oct-06	3	1	15	4	1	1	0	1	3	1	2
20473	202	0	27-Oct-07	22	2	15	4	1	1	0	1	3	1	2
20474	101	1	9-May-09	11	1	15	4	3	1	0	1	3	2	1
20475	202	0	23-Jun-10	2	1	16	4	1	1	0	1	3	6	2
20476	202	0	25-May-08	4	1	16	4	1	1	0	1	3	6	1
20477	202	0	25-May-08	4	1	16	4	1	1	0	1	3	6	1
20478	202	0	27-Oct-07	23	1	16	4	1	1	0	1	3	1	2
20479	202	0	24-Jun-07	4	1	16	4	1	1	0	1	3	1	2
20480	202	0	13-Jan-14	23	1	16	4	3	1	0	4	3	1	1
20481	202	0	21-Jun-13	12	2	16	4	3	1	0	1	3	1	2
20482	202	0	3-Aug-13	22	2	16	4	3	1	0	1	3	5	2
20483	202	0	8-Sep-11	3	2	16	4	3	1	0	1	3	6	2
20484	202	0	8-Sep-11	3	2	16	4	3	1	0	1	3	6	2
20485	202	0	12-May-09	2	2	16	4	1	1	0	1	3	3	2
20486	202	0	24-Oct-09	14	2	16	4	1	1	0	1	3	3	2
20487	202	0	7-Jan-14	17	2	16	4	2	1	0	3	3	3	1
20488	202	0	2-Sep-13	3	1	17	4	3	1	0	1	3	6	2
20489	202	0	5-May-13	3	1	17	4	1	1	0	1	3	1	1
20490	202	0	21-Mar-14	1	1	17	4	1	1	0	3	3	2	1
20491	202	0	12-Jun-10	20	1	17	4	1	1	0	3	3	6	2
20492	202	0	27-Oct-12	23	2	17	4	1	1	0	1	3	3	2
20493	202	0	27-Oct-07	23	2	17	4	2	1	0	1	3	1	2
20494	202	0	12-Sep-07	3	2	17	4	1	1	0	1	3	5	1
20495	201	1	26-Oct-08	5	1	14	4	3	3	1	6	2	1	2
20496	101	1	22-Mar-13	22	1	15	4	2	3	1	4	2	1	1
20497	202	0	11-May-11	18	1	17	4	1	3	1	6	2	5	2
20498	101	1	2-Mar-11	20	1	17	4	3	3	1	4	2	1	2
20499	202	0	22-Apr-06	2	1	16	4	1	2	1	10	2	7	2
20500	202	0	6-Jul-11	23	1	15	4	1	1	0	10	2	7	2
20501	202	0	23-Dec-07	17	1	16	4	1	1	0	3	2	1	1
20502	202	0	17-Aug-10	1	1	17	4	1	1	0	10	2	7	1
20503	202	0	17-Jul-16	16	1	15	4	1	1	0	7	1	5	2
20504	101	1	19-Jul-13	1	1	15	4	1	1	0	11	1	1	1
20505	101	1	14-Apr-13	18	1	15	4	1	1	0	6	1	2	4
20506	202	0	29-Jul-10	23	1	16	4	1	1	0	10	1	7	2
20507	202	0	28-Dec-13	4	2	16	4	3	1	0	10	1	7	1
20508	202	0	7-May-08	8	1	17	4	1	1	0	3	1	1	2
20509	201	1	25-Sep-11	11	2	17	4	3	1	0	6	1	3	1
20510	202	0	14-Sep-11	18	1	16	3	1	1	0	11	5	1	1
20511	101	1	26-Jul-10	13	1	14	3	3	3	1	4	4	1	1
20512	202	0	27-Feb-16	1	1	15	3	3	3	1	6	4	5	2
20513	202	0	30-Oct-15	2	1	16	3	3	3	1	4	4	1	1
20514	202	0	12-Sep-17	21	1	16	3	2	3	1	4	4	1	1
20515	202	0	8-Mar-17	4	1	16	3	3	3	1	4	4	4	1
20516	202	0	27-Jun-17	2	1	16	3	2	3	1	4	4	1	1
20517	202	0	3-May-16	23	1	17	3	3	3	1	6	4	2	1
20518	202	0	21-Nov-06	20	2	17	3	3	3	1	4	4	2	2
20519	201	1	15-Mar-09	23	1	17	3	3	3	1	13	4	1	1
20520	101	1	15-Nov-10	22	1	17	3	2	3	1	4	4	1	1
20521	201	1	3-Sep-08	20	1	17	3	3	3	1	4	4	1	1
20522	202	0	23-Aug-12	10	1	13	3	3	1	0	4	4	1	2
20523	202	0	24-Oct-13	16	1	14	3	3	1	0	6	4	3	1
20524	202	0	3-Aug-09	17	1	14	3	1	1	0	7	4	3	1
20525	202	0	24-Feb-11	11	1	14	3	1	1	0	3	4	3	2
20526	101	1	15-Apr-12	14	1	14	3	3	1	0	7	4	5	2
20527	202	0	23-Jul-12	8	1	15	3	1	1	0	6	4	2	2
20528	202	0	8-Jun-17	20	2	15	3	3	1	0	4	4	2	1
20529	101	1	7-Nov-11	13	1	15	3	3	1	0	4	4	2	1
20530	101	1	9-Jan-13	19	1	15	3	3	1	0	6	4	2	1
20531	201	1	2-Jun-11	13	1	15	3	3	1	0	6	4	2	1
20532	202	0	25-Feb-12	9	1	16	3	3	1	0	7	4	2	2
20533	202	0	23-Jul-16	10	1	16	3	3	1	0	4	4	2	1
20534	202	0	4-Oct-13	10	1	16	3	1	1	0	6	4	1	1
20535	202	0	3-Oct-11	19	1	16	3	1	1	0	6	4	2	2
20536	202	0	10-Dec-10	13	1	16	3	2	1	0	6	4	2	1
20537	202	0	19-Jul-10	11	1	16	3	1	1	0	6	4	2	1
20538	202	0	1-Dec-06	9	1	16	3	1	1	0	6	4	2	1
20539	202	0	29-Jul-11	4	2	16	3	1	1	0	10	4	7	2
20540	201	1	2-Sep-17	10	1	16	3	3	1	0	6	4	2	1
20541	101	1	21-Feb-08	6	1	16	3	2	1	0	4	4	2	4
20542	201	1	10-Aug-08	5	1	16	3	3	1	0	3	4	2	1
20543	202	0	11-Sep-17	0	1	17	3	2	1	0	10	4	6	2
20544	202	0	8-Jan-16	23	1	17	3	2	1	0	4	4	2	2
20545	202	0	29-Nov-16	18	1	17	3	2	1	0	4	4	3	1
20546	202	0	21-Mar-10	23	1	17	3	3	1	0	4	4	2	2
20547	202	0	10-May-07	4	1	17	3	3	1	0	11	4	7	1
20548	202	0	6-Aug-11	13	1	17	3	1	1	0	6	4	2	2
20549	202	0	2-Oct-07	21	1	17	3	3	1	0	6	4	2	2
20550	202	0	10-Jan-06	21	1	17	3	3	1	0	6	4	2	1
20551	202	0	21-Oct-13	18	2	17	3	3	1	0	4	4	2	1
20552	202	0	4-Aug-13	7	2	17	3	2	1	0	6	4	1	1
20553	202	0	7-Jun-13	12	2	17	3	3	1	0	4	4	2	2
20554	202	0	14-Mar-08	12	2	17	3	1	1	0	3	4	4	1
20555	202	0	5-Nov-11	0	2	17	3	1	1	0	6	4	1	2
20556	201	1	13-Dec-12	10	1	17	3	3	1	0	1	4	6	1
20557	201	1	5-Mar-08	2	1	17	3	3	1	0	4	4	2	1
20558	101	1	5-Mar-08	9	1	17	3	2	1	0	6	4	2	1
20559	101	1	8-Jan-10	4	2	17	3	2	1	0	3	4	2	1
20560	201	1	31-Jul-06	15	2	17	3	3	1	0	6	4	2	1
20561	202	0	16-Nov-11	21	1	15	3	1	3	1	11	3	2	1
20562	202	0	28-Oct-07	21	2	16	3	2	3	1	6	3	4	1
20563	202	0	15-Jun-15	7	1	17	3	3	3	1	6	3	4	2
20564	202	0	5-Nov-15	3	1	17	3	3	3	1	4	3	1	1
20565	202	0	7-Jul-07	1	1	17	3	1	3	1	1	3	1	1
20566	202	0	12-Sep-12	1	1	17	3	3	3	1	3	3	1	2
20567	101	1	4-Dec-11	10	1	12	3	1	1	0	1	3	3	1
20568	202	0	30-Oct-16	0	2	14	3	2	1	0	3	3	4	1
20569	202	0	22-May-09	22	2	14	3	1	1	0	1	3	6	1
20570	202	0	14-Jun-11	21	1	15	3	3	1	0	1	3	3	1
20571	202	0	10-May-12	18	2	15	3	3	1	0	3	3	5	2
20572	201	1	12-Jan-10	18	1	15	3	4	1	0	1	3	1	1
20573	101	1	21-Jul-10	3	1	15	3	2	1	0	4	3	2	1
20574	202	0	10-Apr-17	17	1	16	3	2	1	0	1	3	5	1
20575	202	0	8-Sep-13	23	1	16	3	3	1	0	1	3	5	1
20576	202	0	25-Oct-15	12	1	16	3	3	1	0	1	3	5	1
20577	202	0	8-Jan-12	21	1	16	3	1	1	0	1	3	2	2

20578	202	0	30-Sep-11	12	1	16	3	1	1	0	1	3	6	2
20579	202	0	26-Aug-11	18	1	16	3	1	1	0	1	3	6	2
20580	202	0	8-Jul-11	5	1	16	3	3	1	0	1	3	5	2
20581	202	0	25-Jun-10	22	1	16	3	1	1	0	1	3	6	1
20582	202	0	23-Sep-10	3	1	16	3	2	1	0	4	3	3	2
20583	202	0	21-Sep-10	0	1	16	3	1	1	0	6	3	1	1
20584	202	0	25-Apr-16	3	2	16	3	3	1	0	1	3	1	2
20585	202	0	18-Aug-15	23	2	16	3	1	1	0	1	3	1	1
20586	202	0	16-Oct-11	21	2	16	3	1	1	0	1	3	3	2
20587	202	0	17-Nov-11	0	2	16	3	3	1	0	1	3	5	2
20588	201	1	17-Sep-12	19	1	16	3	3	1	0	1	3	1	1
20589	201	1	14-Jun-12	20	1	16	3	3	1	0	1	3	6	1
20590	101	1	22-Jan-12	18	1	16	3	3	1	0	3	3	2	4
20591	201	1	13-Mar-16	12	2	16	3	3	1	0	1	3	5	1
20592	101	1	19-May-08	19	2	16	3	3	1	0	1	3	1	1
20593	202	0	14-Nov-13	18	1	17	3	3	1	0	1	3	5	2
20594	202	0	9-Jun-13	4	1	17	3	2	1	0	1	3	3	2
20595	202	0	31-May-15	21	1	17	3	3	1	0	1	3	5	2
20596	202	0	24-May-13	23	1	17	3	2	1	0	1	3	3	1
20597	202	0	20-Nov-13	0	1	17	3	3	1	0	1	3	1	2
20598	202	0	15-Feb-12	8	1	17	3	3	1	0	1	3	5	2
20599	202	0	5-Nov-15	3	1	17	3	3	1	0	4	3	1	1
20600	202	0	15-Oct-11	21	1	17	3	1	1	0	1	3	6	2
20601	202	0	1-May-11	18	1	17	3	3	1	0	1	3	1	2
20602	202	0	5-Jun-11	3	1	17	3	1	1	0	1	3	1	1
20603	202	0	10-Dec-11	9	1	17	3	1	1	0	1	3	3	2
20604	202	0	8-Aug-11	2	1	17	3	1	1	0	1	3	6	2
20605	202	0	8-Jun-10	0	1	17	3	1	1	0	1	3	4	1
20606	202	0	21-Oct-10	21	1	17	3	1	1	0	1	3	3	2
20607	202	0	27-May-10	22	1	17	3	1	1	0	1	3	5	1
20608	202	0	17-May-08	2	1	17	3	1	1	0	1	3	1	2
20609	202	0	8-Mar-06	22	1	17	3	2	1	0	1	3	4	1
20610	202	0	23-Sep-10	1	1	17	3	2	1	0	4	3	2	2
20611	202	0	13-Aug-15	16	1	17	3	3	1	0	3	3	3	1
20612	202	0	18-Jun-08	21	1	17	3	2	1	0	3	3	6	1
20613	202	0	31-Jul-13	18	2	17	3	3	1	0	1	3	3	1
20614	202	0	7-Jul-15	23	2	17	3	3	1	0	1	3	5	1
20615	202	0	3-Nov-13	12	2	17	3	3	1	0	1	3	5	1
20616	202	0	2-Oct-11	0	2	17	3	1	1	0	1	3	3	1
20617	202	0	22-Jul-11	17	2	17	3	1	1	0	1	3	5	1
20618	202	0	20-Nov-10	21	2	17	3	3	1	0	1	3	1	2
20619	201	1	8-Jul-13	20	1	17	3	3	1	0	1	3	1	2
20620	201	1	25-Mar-11	22	2	17	3	2	1	0	1	3	6	1
20621	101	1	14-Jul-09	1	1	14	3	3	3	1	4	2	2	1
20622	202	0	8-Nov-15	3	1	15	3	1	3	1	4	2	1	1
20623	202	0	16-Jun-15	1	1	15	3	1	3	1	4	2	1	1
20624	202	0	17-May-15	3	1	16	3	3	3	1	6	2	4	2
20625	202	0	25-Mar-13	21	1	16	3	2	3	1	4	2	1	1
20626	202	0	12-Jun-11	1	2	16	3	1	3	1	10	2	7	1
20627	202	0	16-Sep-15	1	2	16	3	1	3	1	4	2	5	1
20628	101	1	9-Sep-10	8	1	16	3	2	3	1	4	2	1	1
20629	101	1	14-Jul-09	1	1	16	3	3	3	1	4	2	1	1
20630	202	0	19-Jul-13	13	1	17	3	3	3	1	4	2	1	1
20631	202	0	21-Aug-13	17	1	17	3	3	3	1	4	2	5	1
20632	202	0	29-Jun-13	23	1	17	3	3	3	1	4	2	5	1
20633	202	0	9-Nov-12	13	1	17	3	3	3	1	4	2	4	2
20634	202	0	12-May-12	17	1	17	3	3	3	1	4	2	1	2
20635	202	0	22-Oct-14	1	1	17	3	2	3	1	6	2	4	2
20636	202	0	29-Apr-08	21	1	17	3	1	3	1	6	2	4	2
20637	202	0	10-Jun-06	8	1	17	3	3	3	1	6	2	1	2
20638	101	1	13-Sep-10	2	1	17	3	3	3	1	4	2	1	1
20639	201	1	13-Jun-09	23	1	15	3	3	2	1	6	2	2	1
20640	202	0	8-Jul-14	13	1	10	3	3	1	0	4	2	1	1
20641	202	0	10-Jan-16	16	1	12	3	3	1	0	3	2	2	2
20642	202	0	2-Oct-15	20	2	12	3	1	1	0	11	2	3	1
20643	202	0	20-Apr-16	8	2	14	3	3	1	0	3	2	3	1
20644	202	0	13-Jun-11	11	1	15	3	3	1	0	3	2	1	1
20645	202	0	28-Nov-10	22	2	15	3	3	1	0	11	2	3	2
20646	202	0	22-Aug-17	17	2	15	3	2	1	0	11	2	3	1
20647	202	0	5-Oct-16	10	1	16	3	2	1	0	6	2	2	1
20648	202	0	12-Jun-07	8	2	16	3	1	1	0	10	2	7	2
20649	202	0	12-Jun-07	8	2	16	3	2	1	0	10	2	7	2
20650	202	0	12-Jun-07	8	1	17	3	1	1	0	10	2	7	2
20651	202	0	28-Nov-12	12	1	17	3	2	1	0	1	2	3	1
20652	202	0	12-Mar-10	22	1	17	3	1	1	0	3	2	2	1
20653	202	0	21-Mar-12	20	1	17	3	1	1	0	6	2	3	2
20654	202	0	21-Oct-11	14	1	17	3	1	1	0	6	2	2	2
20655	202	0	27-Sep-17	17	1	17	3	3	1	0	6	2	2	1
20656	202	0	12-Jun-07	8	2	17	3	1	1	0	10	2	7	2
20657	101	1	25-Jun-08	20	1	17	3	2	1	0	4	2	2	4
20658	101	1	17-Nov-09	13	1	17	3	2	1	0	11	2	2	1
20659	202	0	11-Dec-14	12	2	16	3	1	3	1	4	1	2	1
20660	202	0	31-May-11	1	1	17	3	1	3	1	6	1	2	2
20661	201	1	17-Sep-06	20	2	17	3	3	2	1	6	1	2	1
20662	202	0	16-Sep-11	18	1	14	3	1	1	0	11	1	3	2
20663	101	1	28-Oct-09	1	2	15	3	1	1	0	6	1	3	1
20664	202	0	18-Jul-11	22	1	16	3	1	1	0	11	1	2	2
20665	202	0	16-Aug-07	3	1	16	3	1	1	0	6	1	6	2
20666	202	0	15-Feb-16	12	1	17	3	2	1	0	10	1	7	2
20667	101	1	28-Apr-07	17	1	6	2	1	3	1	11	5	2	1
20668	101	1	17-May-15	19	1	7	2	1	3	1	4	5	1	1
20669	201	1	30-Mar-11	15	2	7	2	1	3	1	13	5	2	1
20670	101	1	20-Aug-08	18	2	7	2	1	3	1	4	5	1	1
20671	101	1	19-Mar-11	19	1	8	2	3	3	1	13	5	4	1
20672	202	0	25-Jul-07	17	1	9	2	3	3	1	4	5	1	1
20673	101	1	24-Jul-14	17	1	9	2	1	3	1	4	5	4	1
20674	202	0	31-Jan-11	18	1	11	2	3	3	1	11	5	3	1
20675	101	1	8-Apr-11	17	1	12	2	2	3	1	13	5	1	2
20676	202	0	9-Sep-07	15	1	13	2	1	3	1	4	5	4	1
20677	202	0	24-Aug-08	8	1	5	2	1	1	0	7	5	4	2
20678	202	0	6-May-11	19	1	5	2	1	1	0	11	5	3	1
20679	202	0	25-May-08	17	1	5	2	2	1	0	3	5	3	2
20680	202	0	27-Sep-07	17	1	5	2	3	1	0	3	5	2	1
20681	202	0	9-Jul-08	20	1	5	2	3	1	0	13	5	4	2
20682	202	0	11-Nov-06	9	1	5	2	1	1	0	13	5	3	2
20683	202	0	31-Aug-17	18	2	5	2	1	1	0	4	5	1	1
20684	202	0	16-Sep-08	20	2	5	2	2	1	0	3	5	3	1
20685	101	1	3-May-07	10	1	5	2	1	1	0	7	5	3	1
20686	101	1	9-Feb-09	17	1	5	2	1	1	0	4	5	4	1
20687	101	1	1-Mar-11	15	1	5	2	1	1	0	3	5	1	1
20688	101	1	4-Oct-07	11	1	5	2	3	1	0	6	5	2	1
20689	201	1	26-Nov-11	21	2	5	2	2	1	0	3	5	1	1
20690	101	1	20-Aug-08	10	2	5	2	1	1	0	3	5	2	2
20691	101	1	27-Oct-06	12	2	5	2	1	1	0	13	5	1	1
20692	202	0	30-Oct-06	16	1	6	2	3	1	0	3	5	1	1
20693	202	0	8-May-08	14	2	6	2	1	1	0	3	5	1	2
20694	202	0	18-Jul-08	1	2	6	2	1	1	0	3	5	3	1
20695	101	1	8-Feb-09	20	1	6	2	1	1	0	4	5	1	1
20696	101	1	5-Nov-17	19	1	6	2	1	1	0	13	5	7	1
20697	101	1	4-Jul-16	18	2	6	2	1	1	0	11	5	2	1
20698	201	1	11-Feb-13	11	2	6	2	2	1	0	3	5	2	1
20699	201	1	13-Oct-09	0	2	6	2	2	1	0	13	5	3	1
20700	202	0	23-Feb-10	17	1	7	2	3	1	0	9	5	7	2
20701	202	0	1-Apr-11	19	1	7	2	1	1	0	4	5	2	1

20702	202	0	26-May-10	17	1	7	2	1	1	0	11	5	3	1
20703	202	0	2-Jul-17	17	1	7	2	1	1	0	11	5	2	1
20704	202	0	15-Aug-11	17	1	7	2	1	1	0	3	5	1	2
20705	202	0	11-Mar-10	8	1	7	2	1	1	0	3	5	2	2
20706	202	0	19-Jul-09	20	1	7	2	1	1	0	3	5	2	1
20707	202	0	26-Dec-08	21	1	7	2	1	1	0	3	5	1	1
20708	202	0	14-Dec-08	17	1	7	2	2	1	0	3	5	2	2
20709	202	0	18-Aug-07	16	1	7	2	1	1	0	3	5	3	1
20710	202	0	10-Jul-08	15	2	7	2	1	1	0	3	5	1	1
20711	201	1	9-Jul-10	20	1	7	2	3	1	0	9	5	7	2
20712	101	1	25-Mar-11	20	1	7	2	1	1	0	1	5	2	2
20713	101	1	25-Dec-14	16	1	7	2	1	1	0	4	5	2	1
20714	101	1	27-Feb-15	12	1	7	2	1	1	0	11	5	3	1
20715	101	1	1-Mar-11	10	1	7	2	1	1	0	3	5	1	1
20716	101	1	13-Mar-11	11	1	7	2	1	1	0	3	5	1	1
20717	101	1	5-Aug-13	17	1	7	2	1	1	0	6	5	3	1
20718	101	1	3-Nov-07	19	2	7	2	1	1	0	11	5	2	1
20719	101	1	5-Nov-15	18	2	7	2	1	1	0	3	5	4	2
20720	101	1	26-Jan-17	12	2	7	2	1	1	0	3	5	3	1
20721	101	1	5-Oct-06	16	2	7	2	3	1	0	6	5	3	1
20722	202	0	22-Jul-11	23	1	8	2	3	1	0	11	5	3	1
20723	202	0	5-May-08	17	1	8	2	1	1	0	3	5	4	1
20724	202	0	17-Sep-06	11	1	8	2	1	1	0	3	5	2	2
20725	202	0	12-Sep-08	17	1	8	2	3	1	0	6	5	3	2
20726	202	0	20-Oct-08	13	1	8	2	1	1	0	13	5	7	1
20727	202	0	22-Jul-07	9	1	8	2	1	1	0	13	5	7	1
20728	202	0	1-Jun-17	12	2	8	2	2	1	0	7	5	3	1
20729	202	0	16-May-11	0	2	8	2	3	1	0	11	5	5	1
20730	202	0	13-Apr-09	13	2	8	2	1	1	0	6	5	3	2
20731	101	1	12-Apr-11	21	1	8	2	3	1	0	4	5	1	1
20732	101	1	1-Oct-17	10	1	8	2	1	1	0	4	5	2	1
20733	201	1	13-Jul-10	10	1	8	2	2	1	0	11	5	2	1
20734	101	1	23-Oct-06	15	1	8	2	1	1	0	11	5	2	1
20735	101	1	17-Nov-17	19	1	8	2	1	1	0	11	5	3	1
20736	101	1	7-Apr-12	14	1	8	2	1	1	0	6	5	2	1
20737	201	1	17-Feb-12	12	1	8	2	1	1	0	13	5	1	1
20738	201	1	31-Aug-09	17	1	8	2	1	1	0	13	5	3	1
20739	101	1	17-Nov-17	19	1	8	2	1	1	0	13	5	1	1
20740	201	1	13-Mar-12	17	2	8	2	3	1	0	13	5	2	2
20741	101	1	26-Jun-09	17	2	8	2	1	1	0	4	5	4	1
20742	201	1	2-Aug-09	18	2	8	2	3	1	0	4	5	3	1
20743	101	1	4-May-06	12	2	8	2	2	1	0	3	5	2	1
20744	101	1	28-Apr-11	14	2	8	2	3	1	0	6	5	2	1
20745	101	1	8-Jul-06	17	2	8	2	1	1	0	6	5	2	2
20746	202	0	15-Aug-16	0	1	9	2	1	1	0	4	5	3	2
20747	202	0	16-Jun-09	17	1	9	2	1	1	0	3	5	2	1
20748	202	0	12-Oct-17	8	1	9	2	1	1	0	3	5	1	1
20749	202	0	25-Feb-07	19	1	9	2	2	1	0	13	5	7	2
20750	202	0	28-Feb-07	8	1	9	2	1	1	0	13	5	1	1
20751	202	0	22-Sep-07	14	1	9	2	1	1	0	13	5	7	1
20752	202	0	9-May-08	16	2	9	2	1	1	0	13	5	7	1
20753	101	1	1-Oct-17	10	1	9	2	1	1	0	4	5	2	1
20754	101	1	29-May-06	12	1	9	2	1	1	0	11	5	1	1
20755	101	1	4-Aug-13	16	1	9	2	1	1	0	3	5	3	4
20756	101	1	4-Oct-09	14	1	9	2	2	1	0	3	5	1	1
20757	101	1	23-Apr-07	12	1	9	2	3	1	0	3	5	1	1
20758	101	1	1-Feb-10	0	1	9	2	1	1	0	6	5	3	1
20759	101	1	24-Aug-09	9	1	9	2	2	1	0	6	5	3	1
20760	101	1	7-Jun-07	8	1	9	2	1	1	0	13	5	3	1
20761	101	1	16-Nov-17	9	1	9	2	1	1	0	13	5	1	1
20762	101	1	11-Oct-17	19	1	9	2	1	1	0	13	5	1	1
20763	101	1	16-Nov-17	9	1	9	2	1	1	0	13	5	1	1
20764	201	1	7-Jul-11	18	2	9	2	2	1	0	11	5	2	1
20765	202	0	21-Jul-16	22	1	10	2	3	1	0	4	5	1	1
20766	202	0	23-Apr-06	13	1	10	2	1	1	0	13	5	2	1
20767	202	0	3-Jan-10	17	1	10	2	1	1	0	4	5	1	2
20768	202	0	18-Sep-08	18	1	10	2	3	1	0	3	5	2	1
20769	202	0	12-Oct-17	19	1	10	2	1	1	0	3	5	1	1
20770	202	0	16-Dec-11	23	1	10	2	1	1	0	6	5	1	2
20771	202	0	2-Jun-11	21	2	10	2	3	1	0	13	5	1	1
20772	101	1	28-Oct-17	10	1	10	2	1	1	0	1	5	2	1
20773	101	1	17-Nov-17	18	1	10	2	1	1	0	11	5	2	1
20774	101	1	24-Jul-11	22	1	10	2	1	1	0	3	5	1	1
20775	101	1	25-May-12	7	1	10	2	1	1	0	6	5	3	1
20776	101	1	24-Mar-11	9	2	10	2	2	1	0	3	5	2	2
20777	202	0	26-Jul-16	13	1	11	2	1	1	0	13	5	1	2
20778	202	0	29-Jan-07	18	1	11	2	1	1	0	13	5	7	1
20779	202	0	25-Jul-07	17	1	11	2	3	1	0	4	5	1	1
20780	202	0	21-Mar-09	19	1	11	2	1	1	0	3	5	3	1
20781	202	0	27-Mar-08	19	1	11	2	1	1	0	3	5	1	1
20782	202	0	17-Apr-06	23	1	11	2	1	1	0	13	5	6	1
20783	202	0	26-Mar-08	10	2	11	2	3	1	0	3	5	2	1
20784	101	1	17-Aug-09	22	1	11	2	1	1	0	4	5	3	1
20785	101	1	19-Aug-15	12	1	11	2	1	1	0	3	5	4	1
20786	101	1	7-Apr-11	15	1	11	2	1	1	0	3	5	1	1
20787	201	1	8-Sep-11	23	1	11	2	1	1	0	3	5	1	1
20788	101	1	6-Aug-06	13	2	11	2	1	1	0	11	5	3	1
20789	101	1	15-Jan-17	17	2	11	2	1	1	0	11	5	2	1
20790	202	0	25-Jul-06	19	1	12	2	3	1	0	11	5	2	1
20791	202	0	12-Oct-06	15	2	12	2	1	1	0	11	5	2	2
20792	101	1	7-Feb-17	16	1	12	2	3	1	0	7	5	5	2
20793	101	1	18-Mar-11	12	1	12	2	1	1	0	3	5	1	1
20794	101	1	12-Sep-15	17	1	12	2	1	1	0	6	5	3	1
20795	101	1	4-Nov-17	8	1	12	2	1	1	0	13	5	7	1
20796	202	0	7-May-11	20	1	13	2	1	1	0	11	5	2	1
20797	202	0	12-Jul-08	17	1	13	2	1	1	0	11	5	6	2
20798	202	0	26-Aug-08	18	1	13	2	1	1	0	3	5	1	1
20799	202	0	22-Dec-08	12	1	13	2	3	1	0	3	5	3	2
20800	101	1	2-May-09	20	1	13	2	3	1	0	13	5	1	1
20801	202	0	21-Jul-17	10	1	14	2	2	1	0	6	5	2	1
20802	202	0	17-Apr-09	12	2	14	2	1	1	0	1	5	4	1
20803	202	0	24-Apr-06	14	1	15	2	2	1	0	13	5	2	1
20804	202	0	3-Mar-10	14	1	17	2	1	1	0	4	5	2	2
20805	101	1	23-Oct-11	12	2	5	2	1	3	1	11	4	4	1
20806	101	1	23-Nov-09	12	2	6	2	3	3	1	4	4	5	1
20807	202	0	13-Dec-12	16	1	7	2	3	3	1	6	4	4	1
20808	202	0	29-Dec-14	12	1	8	2	1	3	1	4	4	1	1
20809	202	0	16-Oct-13	16	1	8	2	1	3	1	10	4	5	2
20810	201	1	8-Feb-08	22	1	8	2	1	3	1	13	4	1	1
20811	101	1	15-Apr-14	16	1	8	2	1	3	1	4	4	1	1
20812	201	1	26-Jul-06	12	1	8	2	2	3	1	4	4	1	1
20813	101	1	5-Aug-15	20	1	8	2	1	3	1	3	4	1	1
20814	101	1	10-Feb-07	17	1	9	2	1	3	1	4	4	1	1
20815	101	1	17-Sep-09	21	1	9	2	1	3	1	11	4	2	1
20816	101	1	25-Jun-14	11	1	10	2	1	3	1	13	4	2	1
20817	201	1	3-Mar-09	13	1	10	2	1	3	1	13	4	1	1
20818	101	1	24-Mar-07	21	1	10	2	1	3	1	11	4	3	1
20819	202	0	8-Jun-13	16	1	11	2	3	3	1	4	4	2	1
20820	202	0	28-Dec-13	13	1	11	2	3	3	1	4	4	1	1
20821	202	0	1-May-12	21	1	11	2	3	3	1	11	4	3	2
20822	101	1	3-Jul-13	11	1	12	2	1	3	1	4	4	4	1
20823	101	1	1-Apr-06	21	1	12	2	1	3	1	4	4	4	1
20824	101	1	4-Jun-07	11	2	12	2	1	3	1	11	4	2	1
20825	202	0	1-Jul-06	17	1	13	2	1	3	1	4	4	1	1

20826	202	0	1-Sep-11	20	1	13	2	3	3	1	11	4	3	2
20827	202	0	29-Nov-10	17	1	13	2	1	3	1	11	4	3	1
20828	201	1	6-Jan-11	16	2	9	2	1	2	1	3	4	2	1
20829	202	0	11-Jul-08	21	1	13	2	1	2	1	3	4	6	1
20830	202	0	23-Jan-08	18	1	5	2	3	1	0	11	4	3	1
20831	202	0	4-Oct-07	10	1	5	2	1	1	0	11	4	3	2
20832	202	0	11-Aug-09	21	1	5	2	2	1	0	3	4	1	1
20833	202	0	22-Sep-07	15	1	5	2	1	1	0	3	4	7	1
20834	202	0	20-Jun-07	2	2	5	2	1	1	0	4	4	1	1
20835	202	0	6-Apr-17	9	2	5	2	3	1	0	11	4	2	1
20836	201	1	20-Jan-11	17	1	5	2	1	1	0	4	4	2	1
20837	201	1	9-Jul-10	12	1	5	2	1	1	0	4	4	1	1
20838	201	1	9-Dec-09	19	1	5	2	3	1	0	11	4	2	1
20839	201	1	5-Mar-08	17	1	5	2	1	1	0	11	4	2	1
20840	201	1	11-Aug-06	17	1	5	2	1	1	0	11	4	4	2
20841	201	1	26-Mar-11	19	1	5	2	3	1	0	3	4	1	2
20842	101	1	21-Jun-07	21	1	5	2	1	1	0	3	4	1	1
20843	101	1	26-Nov-06	13	1	5	2	1	1	0	3	4	2	1
20844	101	1	4-Nov-08	20	1	5	2	1	1	0	6	4	2	1
20845	201	1	17-Feb-07	16	1	5	2	1	1	0	6	4	2	1
20846	101	1	14-Aug-06	12	2	5	2	1	1	0	7	4	1	1
20847	101	1	24-Jul-11	10	2	5	2	1	1	0	11	4	1	1
20848	201	1	7-Oct-10	17	2	5	2	1	1	0	3	4	1	1
20849	201	1	19-May-07	17	2	5	2	2	1	0	6	4	1	1
20850	202	0	19-Nov-17	0	1	6	2	3	1	0	4	4	1	1
20851	202	0	17-Nov-09	14	1	6	2	1	1	0	4	4	1	1
20852	202	0	6-Nov-06	19	1	6	2	1	1	0	4	4	1	1
20853	202	0	13-Jun-16	12	1	6	2	3	1	0	3	4	1	1
20854	202	0	5-Oct-09	16	1	6	2	1	1	0	3	4	3	1
20855	202	0	29-Jul-11	5	1	6	2	1	1	0	6	4	3	2
20856	202	0	6-Apr-17	8	1	6	2	1	1	0	6	4	1	1
20857	202	0	23-Aug-09	11	2	6	2	3	1	0	11	4	2	2
20858	202	0	3-Sep-11	21	2	6	2	1	1	0	3	4	1	1
20859	202	0	17-Aug-16	17	2	6	2	1	1	0	6	4	2	2
20860	201	1	25-Jul-16	15	1	6	2	1	1	0	6	4	2	1
20861	201	1	29-Jul-16	18	1	6	2	2	1	0	3	4	2	1
20862	201	1	13-Jul-16	16	1	6	2	3	1	0	6	4	2	1
20863	201	1	29-Sep-16	18	1	6	2	1	1	0	6	4	3	1
20864	101	1	20-Aug-08	16	1	6	2	1	1	0	7	4	4	1
20865	101	1	10-Feb-07	15	1	6	2	1	1	0	7	4	2	1
20866	101	1	6-Aug-16	10	1	6	2	3	1	0	13	4	3	1
20867	201	1	3-Aug-08	19	1	6	2	1	1	0	13	4	1	1
20868	201	1	20-Mar-13	13	1	6	2	3	1	0	4	4	3	1
20869	201	1	4-Jul-10	18	1	6	2	1	1	0	4	4	2	1
20870	101	1	29-Jan-07	13	1	6	2	1	1	0	4	4	2	1
20871	201	1	12-Aug-15	16	1	6	2	2	1	0	11	4	1	1
20872	101	1	22-Oct-12	10	1	6	2	1	1	0	11	4	1	1
20873	101	1	8-May-11	16	1	6	2	2	1	0	11	4	3	1
20874	101	1	6-Aug-16	11	1	6	2	1	1	0	3	4	1	1
20875	201	1	2-Jun-16	8	1	6	2	1	1	0	3	4	3	1
20876	201	1	11-Dec-16	18	1	6	2	1	1	0	3	4	2	1
20877	201	1	14-Aug-16	21	1	6	2	1	1	0	3	4	1	1
20878	201	1	27-Jul-15	9	1	6	2	2	1	0	3	4	2	1
20879	201	1	12-May-11	22	1	6	2	3	1	0	3	4	3	1
20880	201	1	27-Aug-11	18	1	6	2	1	1	0	3	4	3	1
20881	201	1	10-Mar-08	19	1	6	2	1	1	0	3	4	1	1
20882	101	1	17-Jun-07	13	1	6	2	1	1	0	3	4	1	1
20883	101	1	1-Oct-06	19	1	6	2	1	1	0	3	4	3	1
20884	101	1	28-Aug-17	12	1	6	2	1	1	0	3	4	3	1
20885	101	1	28-Nov-08	18	1	6	2	1	1	0	6	4	2	1
20886	101	1	25-Nov-06	17	1	6	2	3	1	0	6	4	2	1
20887	101	1	20-Jan-10	20	2	6	2	1	1	0	7	4	3	1
20888	101	1	10-May-07	18	2	6	2	1	1	0	13	4	7	1
20889	101	1	7-May-08	11	2	6	2	1	1	0	4	4	2	1
20890	101	1	29-Mar-07	20	2	6	2	1	1	0	4	4	2	1
20891	201	1	1-Oct-14	11	2	6	2	1	1	0	3	4	5	1
20892	101	1	9-Dec-14	12	2	6	2	1	1	0	3	4	3	1
20893	201	1	16-Sep-11	14	2	6	2	1	1	0	3	4	1	1
20894	201	1	4-Jun-09	22	2	6	2	1	1	0	3	4	4	2
20895	101	1	21-Sep-08	18	2	6	2	1	1	0	3	4	3	1
20896	202	0	29-Nov-17	0	1	7	2	2	1	0	6	4	3	2
20897	202	0	18-Dec-16	16	1	7	2	1	1	0	4	4	2	1
20898	202	0	14-Aug-14	15	1	7	2	3	1	0	13	4	1	1
20899	202	0	22-Jul-10	15	1	7	2	1	1	0	13	4	2	1
20900	202	0	23-Feb-10	16	1	7	2	1	1	0	9	4	7	2
20901	202	0	12-Feb-13	11	1	7	2	3	1	0	11	4	2	1
20902	202	0	25-Aug-08	18	1	7	2	1	1	0	11	4	2	1
20903	202	0	2-Jan-07	15	1	7	2	1	1	0	11	4	3	2
20904	202	0	6-Jul-16	19	1	7	2	3	1	0	3	4	3	1
20905	202	0	16-Dec-10	16	1	7	2	1	1	0	3	4	2	2
20906	202	0	8-Mar-17	8	2	7	2	3	1	0	4	4	1	1
20907	202	0	5-Oct-11	20	2	7	2	1	1	0	11	4	3	2
20908	202	0	13-Aug-09	20	2	7	2	1	1	0	11	4	3	1
20909	202	0	27-May-12	17	2	7	2	1	1	0	3	4	4	2
20910	201	1	15-Jul-12	8	1	7	2	1	1	0	6	4	1	1
20911	201	1	6-Aug-15	11	1	7	2	1	1	0	4	4	1	1
20912	201	1	4-Feb-17	9	1	7	2	1	1	0	6	4	3	1
20913	201	1	18-Mar-12	18	1	7	2	2	1	0	6	4	3	1
20914	201	1	1-Nov-15	11	1	7	2	1	1	0	6	4	2	1
20915	201	1	20-Jul-08	21	1	7	2	1	1	0	7	4	7	1
20916	201	1	6-Dec-10	17	1	7	2	2	1	0	1	4	1	1
20917	201	1	10-Oct-09	20	1	7	2	2	1	0	5	4	2	2
20918	101	1	22-Jun-14	17	1	7	2	1	1	0	4	4	4	1
20919	101	1	22-Apr-14	8	1	7	2	1	1	0	4	4	4	1
20920	101	1	12-Aug-14	21	1	7	2	1	1	0	4	4	3	1
20921	101	1	10-Jul-14	12	1	7	2	1	1	0	4	4	2	1
20922	201	1	15-Apr-11	10	1	7	2	1	1	0	4	4	2	1
20923	101	1	7-May-10	16	1	7	2	1	1	0	4	4	4	1
20924	201	1	28-Aug-08	17	1	7	2	1	1	0	4	4	3	1
20925	201	1	22-Jul-08	19	1	7	2	2	1	0	4	4	1	1
20926	101	1	14-Dec-08	21	1	7	2	1	1	0	4	4	3	1
20927	101	1	30-Mar-07	21	1	7	2	1	1	0	4	4	1	1
20928	101	1	5-Oct-07	16	1	7	2	1	1	0	4	4	2	1
20929	101	1	25-May-17	14	1	7	2	1	1	0	4	4	2	1
20930	201	1	20-May-15	13	1	7	2	2	1	0	11	4	3	1
20931	201	1	5-Jul-14	18	1	7	2	3	1	0	11	4	3	1
20932	101	1	9-May-09	16	1	7	2	1	1	0	11	4	3	1
20933	201	1	24-Jul-15	9	1	7	2	1	1	0	3	4	1	1
20934	201	1	15-Jul-15	8	1	7	2	1	1	0	3	4	1	1
20935	201	1	5-Oct-15	15	1	7	2	1	1	0	3	4	3	1
20936	101	1	10-Feb-15	17	1	7	2	1	1	0	3	4	3	1
20937	201	1	27-Jun-15	16	1	7	2	1	1	0	3	4	3	1
20938	201	1	28-Jan-15	14	1	7	2	1	1	0	3	4	2	1
20939	101	1	9-Dec-14	7	1	7	2	1	1	0	3	4	5	1
20940	201	1	1-Jan-14	9	1	7	2	3	1	0	3	4	2	1
20941	101	1	15-Nov-14	16	1	7	2	1	1	0	3	4	1	2
20942	201	1	28-Mar-14	15	1	7	2	1	1	0	3	4	1	1
20943	201	1	18-Jul-14	12	1	7	2	3	1	0	3	4	1	1
20944	101	1	27-Mar-14	19	1	7	2	1	1	0	3	4	1	1
20945	101	1	24-Aug-14	20	1	7	2	1	1	0	3	4	1	1
20946	201	1	18-Jul-14	12	1	7	2	1	1	0	3	4	1	1
20947	101	1	16-Jun-14	20	1	7	2	1	1	0	3	4	1	1
20948	101	1	14-May-14	12	1	7	2	1	1	0	3	4	3	1
20949	101	1	15-May-14	17	1	7	2	1	1	0	3	4	3	1

20950	101	1	6-Sep-14	20	1	7	2	1	1	0	3	4	2	1
20951	101	1	11-Oct-14	21	1	7	2	1	1	0	3	4	2	1
20952	201	1	3-Aug-14	21	1	7	2	1	1	0	3	4	2	1
20953	101	1	14-Feb-13	15	1	7	2	3	1	0	3	4	1	1
20954	101	1	12-Feb-13	23	1	7	2	1	1	0	3	4	3	1
20955	201	1	4-Oct-12	19	1	7	2	2	1	0	3	4	2	1
20956	101	1	23-Aug-12	20	1	7	2	1	1	0	3	4	2	1
20957	101	1	4-Apr-12	10	1	7	2	1	1	0	3	4	2	1
20958	201	1	29-Dec-12	18	1	7	2	2	1	0	3	4	1	1
20959	101	1	27-Feb-11	21	1	7	2	1	1	0	3	4	1	1
20960	201	1	25-Oct-11	18	1	7	2	3	1	0	3	4	3	1
20961	201	1	6-Oct-11	8	1	7	2	2	1	0	3	4	2	1
20962	201	1	3-Mar-10	19	1	7	2	1	1	0	3	4	2	1
20963	101	1	12-Jun-17	20	1	7	2	1	1	0	3	4	1	1
20964	201	1	11-Jan-17	16	1	7	2	3	1	0	3	4	1	1
20965	101	1	29-Sep-17	15	1	7	2	2	1	0	3	4	2	4
20966	201	1	5-Nov-11	17	1	7	2	1	1	0	6	4	3	1
20967	101	1	6-Apr-11	13	1	7	2	1	1	0	6	4	1	1
20968	101	1	23-Sep-10	22	1	7	2	2	1	0	6	4	3	1
20969	101	1	30-Aug-08	17	1	7	2	1	1	0	6	4	3	1
20970	201	1	2-Nov-06	10	1	7	2	3	1	0	6	4	2	1
20971	101	1	28-Dec-13	13	2	7	2	1	1	0	7	4	3	1
20972	101	1	16-Jun-14	12	2	7	2	1	1	0	4	4	4	1
20973	101	1	15-Oct-14	19	2	7	2	1	1	0	4	4	1	1
20974	101	1	8-Feb-10	20	2	7	2	1	1	0	4	4	3	1
20975	201	1	11-Mar-09	9	2	7	2	3	1	0	4	4	1	1
20976	101	1	20-Nov-09	21	2	7	2	1	1	0	4	4	3	1
20977	101	1	1-Jul-08	20	2	7	2	1	1	0	4	4	1	1
20978	101	1	5-Jul-08	10	2	7	2	1	1	0	4	4	3	1
20979	101	1	22-Feb-16	11	2	7	2	1	1	0	11	4	3	1
20980	101	1	19-Oct-12	15	2	7	2	1	1	0	11	4	2	1
20981	101	1	17-Apr-10	17	2	7	2	3	1	0	11	4	4	1
20982	101	1	12-Jan-17	18	2	7	2	1	1	0	11	4	2	1
20983	201	1	5-Jun-17	9	2	7	2	1	1	0	11	4	2	1
20984	101	1	19-Mar-16	17	2	7	2	1	1	0	3	4	1	1
20985	101	1	9-Dec-14	15	2	7	2	1	1	0	3	4	1	1
20986	101	1	5-Jan-14	9	2	7	2	2	1	0	3	4	1	1
20987	201	1	15-Oct-14	13	2	7	2	1	1	0	3	4	2	2
20988	101	1	21-Aug-14	14	2	7	2	3	1	0	3	4	2	1
20989	101	1	7-Aug-13	18	2	7	2	1	1	0	3	4	1	2
20990	101	1	23-Sep-13	11	2	7	2	1	1	0	3	4	1	1
20991	201	1	9-Jun-12	15	2	7	2	3	1	0	3	4	1	1
20992	201	1	8-Apr-11	22	2	7	2	1	1	0	3	4	2	1
20993	201	1	5-Jul-08	13	2	7	2	1	1	0	3	4	1	1
20994	101	1	8-Apr-17	21	2	7	2	1	1	0	3	4	1	1
20995	101	1	13-May-17	14	2	7	2	1	1	0	3	4	1	1
20996	201	1	11-Aug-17	19	2	7	2	3	1	0	3	4	3	1
20997	101	1	22-Jan-17	10	2	7	2	1	1	0	6	4	3	1
20998	101	1	31-Jul-08	14	2	7	2	2	1	0	13	4	3	2
20999	202	0	23-Jul-13	20	1	8	2	3	1	0	1	4	5	1
21000	202	0	14-May-17	10	1	8	2	1	1	0	6	4	3	1
21001	202	0	3-Nov-16	12	1	8	2	2	1	0	6	4	3	1
21002	202	0	11-Apr-09	14	1	8	2	1	1	0	11	4	7	1
21003	202	0	29-Sep-08	16	1	8	2	1	1	0	11	4	4	1
21004	202	0	18-Aug-12	12	1	8	2	3	1	0	3	4	1	1
21005	202	0	30-Oct-09	20	1	8	2	1	1	0	3	4	3	1
21006	202	0	14-May-06	9	1	8	2	1	1	0	3	4	1	2
21007	202	0	9-Aug-16	12	1	8	2	1	1	0	6	4	5	2
21008	202	0	16-Jul-07	18	1	8	2	1	1	0	6	4	1	1
21009	202	0	4-Oct-06	14	2	8	2	2	1	0	6	4	1	1
21010	202	0	2-Jan-17	15	2	8	2	3	1	0	6	4	1	1
21011	201	1	20-Jan-17	17	1	8	2	1	1	0	4	4	2	1
21012	201	1	1-Feb-13	15	1	8	2	3	1	0	6	4	3	1
21013	201	1	23-Mar-14	13	1	8	2	2	1	0	4	4	2	1
21014	101	1	8-Apr-17	17	1	8	2	1	1	0	3	4	2	1
21015	201	1	6-Oct-16	19	1	8	2	1	1	0	1	4	3	1
21016	201	1	13-Aug-17	18	1	8	2	1	1	0	1	4	1	1
21017	201	1	19-Dec-11	17	1	8	2	1	1	0	7	4	3	1
21018	101	1	2-Nov-08	13	1	8	2	1	1	0	13	4	2	2
21019	101	1	31-Oct-15	17	1	8	2	1	1	0	1	4	1	1
21020	201	1	20-Sep-10	19	1	8	2	2	1	0	1	4	6	1
21021	201	1	10-Jun-15	11	1	8	2	1	1	0	4	4	2	1
21022	101	1	11-Nov-14	16	1	8	2	1	1	0	4	4	1	1
21023	101	1	25-Jul-14	17	1	8	2	3	1	0	4	4	4	1
21024	101	1	24-Nov-14	11	1	8	2	1	1	0	4	4	2	1
21025	101	1	24-May-14	21	1	8	2	1	1	0	4	4	2	1
21026	101	1	31-Dec-10	16	1	8	2	1	1	0	4	4	1	1
21027	101	1	27-Jul-16	16	1	8	2	1	1	0	11	4	3	2
21028	201	1	29-Nov-16	10	1	8	2	1	1	0	11	4	2	1
21029	101	1	29-Sep-14	12	1	8	2	1	1	0	11	4	3	2
21030	201	1	2-Jun-16	19	1	8	2	1	1	0	3	4	1	1
21031	201	1	24-Sep-16	21	1	8	2	2	1	0	3	4	1	1
21032	101	1	20-May-14	11	1	8	2	1	1	0	3	4	5	1
21033	201	1	23-Jan-14	14	1	8	2	2	1	0	3	4	1	1
21034	201	1	2-Oct-14	13	1	8	2	1	1	0	3	4	1	1
21035	201	1	29-Jun-14	17	1	8	2	3	1	0	3	4	1	1
21036	101	1	31-Jan-14	18	1	8	2	1	1	0	3	4	3	1
21037	101	1	30-Aug-13	19	1	8	2	1	1	0	3	4	5	1
21038	101	1	7-Jun-13	18	1	8	2	1	1	0	3	4	1	1
21039	101	1	26-Mar-12	17	1	8	2	1	1	0	3	4	5	1
21040	101	1	6-Sep-12	19	1	8	2	1	1	0	3	4	4	1
21041	101	1	12-Sep-11	19	1	8	2	3	1	0	3	4	3	1
21042	101	1	17-Aug-11	19	1	8	2	1	1	0	3	4	1	2
21043	201	1	31-Aug-08	18	1	8	2	3	1	0	3	4	3	1
21044	201	1	14-Aug-07	15	1	8	2	1	1	0	3	4	2	1
21045	201	1	16-Sep-17	13	1	8	2	1	1	0	3	4	3	1
21046	101	1	1-Feb-17	12	1	8	2	1	1	0	3	4	1	1
21047	101	1	17-Feb-17	19	1	8	2	1	1	0	3	4	1	1
21048	101	1	19-Sep-13	20	1	8	2	1	1	0	6	4	3	1
21049	201	1	24-Sep-10	9	1	8	2	1	1	0	6	4	3	1
21050	101	1	15-Oct-10	21	1	8	2	1	1	0	6	4	3	1
21051	201	1	23-Sep-07	23	1	8	2	2	1	0	6	4	2	1
21052	201	1	18-Dec-06	20	1	8	2	2	1	0	6	4	3	1
21053	101	1	4-Mar-07	18	2	8	2	1	1	0	4	4	1	1
21054	201	1	1-Jul-16	16	2	8	2	3	1	0	11	4	1	1
21055	101	1	21-Feb-15	10	2	8	2	1	1	0	11	4	2	1
21056	101	1	5-May-13	11	2	8	2	1	1	0	11	4	3	1
21057	101	1	13-May-15	20	2	8	2	1	1	0	3	4	2	2
21058	101	1	2-Nov-15	20	2	8	2	1	1	0	3	4	3	1
21059	101	1	31-Mar-14	20	2	8	2	1	1	0	3	4	3	1
21060	101	1	16-Jan-14	21	2	8	2	1	1	0	3	4	3	1
21061	101	1	4-Oct-13	16	2	8	2	1	1	0	3	4	1	1
21062	101	1	22-Aug-13	18	2	8	2	1	1	0	3	4	2	1
21063	201	1	18-Jan-12	13	2	8	2	2	1	0	3	4	2	1
21064	201	1	3-Aug-07	20	2	8	2	3	1	0	3	4	3	1
21065	101	1	7-Jan-17	18	2	8	2	1	1	0	3	4	1	1
21066	201	1	28-Oct-17	23	2	8	2	1	1	0	3	4	1	1
21067	101	1	12-Nov-17	11	2	8	2	1	1	0	3	4	3	1
21068	101	1	24-Jul-11	14	2	8	2	1	1	0	6	4	2	1
21069	101	1	4-May-07	13	2	8	2	1	1	0	6	4	2	1
21070	101	1	8-Jul-06	16	2	8	2	3	1	0	6	4	2	1
21071	202	0	24-Jun-07	15	1	9	2	1	1	0	9	4	7	2
21072	202	0	30-Aug-14	17	1	9	2	1	1	0	3	4	1	1
21073	202	0	21-Oct-12	19	1	9	2	3	1	0	3	4	4	1

21074	202	0	4-Dec-11	16	1	9	2	1	1	0	3	4	1	2
21075	202	0	6-Nov-09	19	1	9	2	2	1	0	3	4	1	1
21076	202	0	19-Oct-17	15	2	9	2	2	1	0	6	4	3	1
21077	202	0	4-Sep-08	8	2	9	2	1	1	0	7	4	3	1
21078	202	0	31-Jan-10	16	2	9	2	1	1	0	11	4	3	1
21079	202	0	3-Oct-16	20	2	9	2	1	1	0	3	4	3	1
21080	202	0	8-Aug-12	18	2	9	2	1	1	0	3	4	1	1
21081	202	0	29-Jan-10	20	2	9	2	1	1	0	6	4	1	1
21082	202	0	20-Jun-09	17	2	9	2	1	1	0	6	4	3	1
21083	202	0	21-May-08	17	2	9	2	2	1	0	6	4	3	1
21084	202	0	1-Aug-07	12	2	9	2	1	1	0	6	4	3	1
21085	101	1	3-Dec-15	7	1	9	2	1	1	0	7	4	4	1
21086	101	1	29-Sep-12	17	1	9	2	1	1	0	7	4	3	1
21087	101	1	16-Apr-15	10	1	9	2	1	1	0	4	4	2	1
21088	101	1	22-Dec-14	11	1	9	2	1	1	0	4	4	4	1
21089	101	1	16-Mar-14	14	1	9	2	1	1	0	4	4	1	1
21090	101	1	27-Jan-17	17	1	9	2	3	1	0	4	4	3	1
21091	201	1	12-Aug-16	13	1	9	2	2	1	0	11	4	2	1
21092	101	1	3-Sep-15	15	1	9	2	1	1	0	11	4	5	1
21093	101	1	15-Aug-15	19	1	9	2	1	1	0	11	4	3	1
21094	101	1	3-Apr-12	12	1	9	2	1	1	0	11	4	1	2
21095	101	1	12-Mar-11	20	1	9	2	1	1	0	11	4	2	1
21096	201	1	28-Feb-16	11	1	9	2	3	1	0	3	4	2	1
21097	201	1	30-Nov-15	20	1	9	2	1	1	0	3	4	1	1
21098	201	1	13-Jan-14	21	1	9	2	1	1	0	3	4	1	1
21099	101	1	16-Mar-14	17	1	9	2	1	1	0	3	4	2	1
21100	201	1	31-Jan-14	13	1	9	2	2	1	0	3	4	3	1
21101	201	1	26-Jun-13	19	1	9	2	2	1	0	3	4	1	2
21102	201	1	31-Jul-12	8	1	9	2	2	1	0	3	4	1	1
21103	101	1	22-Jun-12	12	1	9	2	1	1	0	3	4	1	1
21104	101	1	19-May-12	11	1	9	2	1	1	0	3	4	4	1
21105	101	1	21-Mar-12	20	1	9	2	1	1	0	3	4	3	1
21106	201	1	16-Oct-11	14	1	9	2	1	1	0	3	4	2	1
21107	201	1	24-Jun-11	21	1	9	2	2	1	0	3	4	1	2
21108	201	1	30-Aug-10	10	1	9	2	3	1	0	3	4	1	1
21109	101	1	29-May-17	13	1	9	2	1	1	0	3	4	5	1
21110	201	1	2-May-11	17	1	9	2	1	1	0	6	4	4	1
21111	201	1	31-Dec-10	11	1	9	2	3	1	0	6	4	2	2
21112	201	1	23-Sep-15	14	2	9	2	3	1	0	6	4	2	1
21113	201	1	7-Jan-17	10	2	9	2	1	1	0	4	4	1	1
21114	201	1	22-Sep-10	23	2	9	2	1	1	0	4	4	2	1
21115	201	1	16-Jul-08	9	2	9	2	2	1	0	4	4	3	1
21116	201	1	4-May-16	19	2	9	2	1	1	0	11	4	4	1
21117	201	1	8-Jul-15	14	2	9	2	1	1	0	11	4	3	1
21118	101	1	30-Aug-12	20	2	9	2	1	1	0	11	4	2	1
21119	201	1	29-Jan-10	15	2	9	2	2	1	0	11	4	3	1
21120	101	1	11-Jul-17	20	2	9	2	1	1	0	11	4	3	1
21121	101	1	2-Aug-17	10	2	9	2	1	1	0	11	4	1	1
21122	101	1	22-Jan-15	20	2	9	2	1	1	0	3	4	1	1
21123	101	1	1-Jun-14	17	2	9	2	2	1	0	3	4	3	1
21124	201	1	24-Oct-14	10	2	9	2	1	1	0	3	4	3	1
21125	101	1	25-Nov-13	12	2	9	2	1	1	0	3	4	3	1
21126	201	1	16-Dec-11	20	2	9	2	1	1	0	3	4	4	1
21127	101	1	3-Aug-06	13	2	9	2	1	1	0	3	4	4	1
21128	202	0	1-Dec-16	12	1	10	2	3	1	0	6	4	2	2
21129	202	0	3-Feb-13	16	1	10	2	1	1	0	7	4	3	2
21130	202	0	20-Aug-10	10	1	10	2	1	1	0	7	4	5	1
21131	202	0	24-Aug-07	16	1	10	2	1	1	0	13	4	1	1
21132	202	0	14-Nov-16	20	1	10	2	1	1	0	4	4	2	1
21133	202	0	29-May-17	13	1	10	2	1	1	0	4	4	3	2
21134	202	0	17-Oct-17	10	1	10	2	2	1	0	4	4	1	1
21135	202	0	12-Sep-14	20	1	10	2	1	1	0	11	4	3	2
21136	202	0	21-Aug-11	16	1	10	2	1	1	0	11	4	6	2
21137	202	0	9-Sep-08	11	1	10	2	1	1	0	11	4	3	1
21138	202	0	30-Oct-07	12	1	10	2	1	1	0	3	4	1	1
21139	202	0	2-May-07	9	1	10	2	1	1	0	3	4	4	1
21140	202	0	9-Apr-17	20	1	10	2	2	1	0	3	4	2	1
21141	202	0	30-Sep-15	11	2	10	2	2	1	0	13	4	1	1
21142	202	0	10-Oct-09	21	2	10	2	2	1	0	3	4	1	1
21143	201	1	24-May-16	13	1	10	2	1	1	0	6	4	2	1
21144	201	1	24-Sep-17	10	1	10	2	1	1	0	6	4	3	1
21145	201	1	26-Nov-14	9	1	10	2	1	1	0	6	4	3	1
21146	201	1	20-Nov-17	18	1	10	2	3	1	0	6	4	1	1
21147	101	1	10-Jul-14	1	1	10	2	1	1	0	4	4	4	1
21148	101	1	10-Oct-14	9	1	10	2	1	1	0	4	4	1	1
21149	101	1	15-Oct-14	19	1	10	2	1	1	0	4	4	2	1
21150	201	1	14-Oct-13	15	1	10	2	2	1	0	4	4	1	1
21151	201	1	23-May-10	14	1	10	2	1	1	0	4	4	2	1
21152	201	1	21-Mar-10	15	1	10	2	2	1	0	4	4	2	1
21153	101	1	6-Aug-09	20	1	10	2	1	1	0	4	4	1	1
21154	201	1	11-Jan-08	23	1	10	2	1	1	0	4	4	2	1
21155	101	1	24-May-16	17	1	10	2	1	1	0	11	4	1	1
21156	101	1	5-Nov-16	19	1	10	2	1	1	0	11	4	2	1
21157	201	1	10-Oct-16	19	1	10	2	2	1	0	11	4	2	1
21158	201	1	16-Oct-16	9	1	10	2	1	1	0	11	4	2	1
21159	201	1	11-Aug-15	16	1	10	2	1	1	0	11	4	1	1
21160	201	1	27-Mar-15	17	1	10	2	1	1	0	11	4	2	1
21161	201	1	1-May-14	19	1	10	2	1	1	0	11	4	2	1
21162	201	1	15-Jul-15	19	1	10	2	1	1	0	3	4	2	1
21163	101	1	31-Jan-14	18	1	10	2	1	1	0	3	4	5	1
21164	201	1	12-Jan-14	18	1	10	2	1	1	0	3	4	1	1
21165	101	1	6-Nov-14	8	1	10	2	1	1	0	3	4	3	1
21166	101	1	10-Feb-13	21	1	10	2	4	1	0	3	4	1	1
21167	101	1	1-Sep-13	18	1	10	2	1	1	0	3	4	1	1
21168	101	1	27-Jun-12	12	1	10	2	1	1	0	3	4	1	1
21169	101	1	15-Apr-12	18	1	10	2	1	1	0	3	4	3	1
21170	201	1	3-Jan-12	14	1	10	2	2	1	0	3	4	2	2
21171	201	1	25-Jun-11	8	1	10	2	3	1	0	3	4	1	1
21172	201	1	28-Apr-10	19	1	10	2	2	1	0	3	4	1	1
21173	101	1	7-Jun-09	16	1	10	2	1	1	0	3	4	3	1
21174	101	1	22-Dec-06	14	1	10	2	4	1	0	3	4	1	1
21175	101	1	2-Jul-06	16	1	10	2	1	1	0	3	4	2	1
21176	101	1	25-Jun-06	11	1	10	2	1	1	0	3	4	3	1
21177	101	1	21-Mar-06	18	1	10	2	1	1	0	3	4	2	1
21178	101	1	29-Apr-13	14	1	10	2	1	1	0	6	4	3	1
21179	101	1	27-Jun-09	22	1	10	2	1	1	0	6	4	1	1
21180	101	1	20-Aug-06	23	1	10	2	3	1	0	13	4	2	2
21181	101	1	10-Feb-15	20	2	10	2	1	1	0	4	4	1	1
21182	101	1	14-Nov-08	20	2	10	2	1	1	0	4	4	1	1
21183	101	1	19-Feb-06	19	2	10	2	1	1	0	4	4	1	1
21184	201	1	1-Sep-10	16	2	10	2	2	1	0	11	4	2	1
21185	201	1	15-Jan-17	15	2	10	2	1	1	0	11	4	4	1
21186	201	1	25-Sep-17	9	2	10	2	1	1	0	11	4	3	1
21187	101	1	11-Aug-15	20	2	10	2	1	1	0	3	4	3	1
21188	201	1	5-Sep-14	17	2	10	2	2	1	0	3	4	1	1
21189	101	1	11-Nov-14	21	2	10	2	1	1	0	3	4	2	1
21190	201	1	10-May-17	13	2	10	2	1	1	0	3	4	3	1
21191	101	1	12-Feb-17	15	2	10	2	1	1	0	3	4	1	1
21192	101	1	3-Dec-17	13	2	10	2	1	1	0	3	4	2	2
21193	101	1	20-Jun-09	17	2	10	2	1	1	0	6	4	2	1
21194	202	0	4-Apr-12	18	1	11	2	1	1	0	4	4	2	1
21195	202	0	12-Dec-08	19	1	11	2	1	1	0	7	4	7	1
21196	202	0	4-Sep-09	19	1	11	2	1	1	0	3	4	3	1
21197	202	0	13-Aug-07	13	1	11	2	1	1	0	3	4	2	2

21198	202	0	2-Dec-16	21	2	11	2	1	1	0	6	4	3	1
21199	202	0	13-Apr-08	16	2	11	2	1	1	0	4	4	4	1
21200	202	0	30-Nov-16	11	2	11	2	1	1	0	3	4	1	1
21201	201	1	8-Jul-16	15	1	11	2	2	1	0	6	4	2	1
21202	101	1	3-Oct-13	9	1	11	2	1	1	0	4	4	3	1
21203	201	1	25-Sep-11	8	1	11	2	1	1	0	7	4	2	1
21204	101	1	18-Jan-15	16	1	11	2	1	1	0	4	4	2	1
21205	101	1	2-Mar-14	20	1	11	2	1	1	0	4	4	2	1
21206	201	1	3-Mar-09	10	1	11	2	1	1	0	4	4	2	1
21207	101	1	30-Jul-08	17	1	11	2	1	1	0	4	4	3	1
21208	101	1	20-Sep-13	15	1	11	2	1	1	0	11	4	4	1
21209	201	1	7-Jul-17	16	1	11	2	1	1	0	11	4	3	1
21210	201	1	1-Jun-16	11	1	11	2	1	1	0	3	4	3	1
21211	201	1	19-Jul-16	14	1	11	2	3	1	0	3	4	3	1
21212	101	1	4-Dec-14	19	1	11	2	1	1	0	3	4	1	1
21213	201	1	1-Sep-14	19	1	11	2	2	1	0	3	4	2	1
21214	201	1	14-Jun-14	23	1	11	2	2	1	0	3	4	3	1
21215	101	1	27-Jun-13	20	1	11	2	1	1	0	3	4	4	1
21216	101	1	12-Aug-13	21	1	11	2	1	1	0	3	4	3	1
21217	101	1	7-Oct-12	17	1	11	2	1	1	0	3	4	3	1
21218	101	1	14-Jul-12	17	1	11	2	1	1	0	3	4	2	1
21219	201	1	3-Aug-12	21	1	11	2	1	1	0	3	4	2	1
21220	101	1	2-May-11	12	1	11	2	1	1	0	3	4	2	1
21221	101	1	11-Feb-11	15	1	11	2	2	1	0	3	4	2	4
21222	201	1	13-Jun-11	21	1	11	2	3	1	0	3	4	3	1
21223	201	1	3-Sep-07	1	1	11	2	2	1	0	3	4	3	1
21224	101	1	6-Feb-06	10	1	11	2	1	1	0	3	4	1	1
21225	201	1	1-Oct-11	12	1	11	2	1	1	0	6	4	3	1
21226	101	1	1-Mar-06	9	1	11	2	1	1	0	6	4	1	1
21227	101	1	1-Jun-07	9	2	11	2	1	1	0	7	4	3	1
21228	101	1	15-Jul-11	21	2	11	2	1	1	0	4	4	2	1
21229	201	1	20-Sep-10	18	2	11	2	2	1	0	4	4	3	1
21230	201	1	21-Aug-11	20	2	11	2	3	1	0	11	4	1	1
21231	101	1	6-Nov-11	20	2	11	2	1	1	0	11	4	1	1
21232	101	1	17-Apr-09	12	2	11	2	1	1	0	11	4	4	1
21233	101	1	20-Mar-17	20	2	11	2	1	1	0	11	4	2	1
21234	201	1	12-Dec-14	13	2	11	2	3	1	0	3	4	2	1
21235	101	1	6-Nov-14	12	2	11	2	1	1	0	3	4	3	1
21236	201	1	27-Sep-13	18	2	11	2	2	1	0	3	4	3	1
21237	101	1	19-Aug-12	19	2	11	2	2	1	0	3	4	2	1
21238	201	1	25-Sep-11	9	2	11	2	3	1	0	3	4	2	1
21239	101	1	14-May-17	17	2	11	2	1	1	0	3	4	4	2
21240	101	1	19-Apr-14	18	2	11	2	1	1	0	6	4	2	1
21241	101	1	5-Sep-06	21	2	11	2	1	1	0	6	4	3	1
21242	202	0	8-Aug-07	14	1	12	2	1	1	0	7	4	2	1
21243	202	0	21-Jul-11	17	1	12	2	1	1	0	4	4	1	2
21244	202	0	15-Jun-12	18	1	12	2	1	1	0	3	4	1	2
21245	202	0	25-Aug-12	16	1	12	2	3	1	0	6	4	3	2
21246	202	0	23-Apr-06	12	1	12	2	1	1	0	13	4	2	1
21247	202	0	28-Jan-08	14	2	12	2	1	1	0	4	4	2	2
21248	202	0	10-Jun-16	20	2	12	2	1	1	0	3	4	2	1
21249	201	1	3-Aug-15	0	1	12	2	1	1	0	6	4	2	1
21250	201	1	24-Sep-16	20	1	12	2	1	1	0	4	4	2	1
21251	101	1	15-Apr-14	17	1	12	2	1	1	0	4	4	1	1
21252	101	1	30-Sep-13	17	1	12	2	1	1	0	4	4	1	1
21253	201	1	2-Dec-11	15	1	12	2	1	1	0	4	4	2	1
21254	101	1	18-Sep-08	14	1	12	2	1	1	0	4	4	4	1
21255	201	1	13-Jul-07	21	1	12	2	1	1	0	4	4	2	2
21256	101	1	27-Nov-17	15	1	12	2	1	1	0	11	4	1	1
21257	201	1	27-Apr-16	22	1	12	2	2	1	0	3	4	3	1
21258	101	1	19-Oct-14	10	1	12	2	1	1	0	3	4	5	1
21259	101	1	26-Mar-14	10	1	12	2	1	1	0	3	4	1	1
21260	201	1	14-Mar-14	10	1	12	2	1	1	0	3	4	1	1
21261	101	1	27-Mar-14	20	1	12	2	1	1	0	3	4	2	1
21262	101	1	25-Apr-14	13	1	12	2	1	1	0	3	4	2	1
21263	101	1	9-Dec-14	16	1	12	2	1	1	0	3	4	2	1
21264	201	1	18-Sep-13	9	1	12	2	2	1	0	3	4	3	1
21265	101	1	24-Dec-13	18	1	12	2	1	1	0	3	4	2	1
21266	101	1	21-Jun-12	17	1	12	2	1	1	0	3	4	2	1
21267	201	1	19-Apr-10	19	1	12	2	2	1	0	3	4	3	2
21268	201	1	21-Jul-08	16	1	12	2	3	1	0	3	4	3	2
21269	201	1	2-Aug-17	15	1	12	2	1	1	0	3	4	2	1
21270	101	1	3-Dec-06	8	2	12	2	1	1	0	4	4	2	1
21271	201	1	18-Jun-15	11	2	12	2	1	1	0	11	4	3	1
21272	201	1	14-Jan-09	16	2	12	2	1	1	0	11	4	2	1
21273	101	1	15-Oct-14	19	2	12	2	1	1	0	3	4	3	1
21274	202	0	11-Jul-14	15	1	13	2	1	1	0	6	4	2	1
21275	202	0	11-Dec-11	8	1	13	2	2	1	0	13	4	1	1
21276	202	0	25-Jul-10	15	1	13	2	1	1	0	4	4	1	2
21277	202	0	14-Nov-10	16	1	13	2	1	1	0	11	4	3	2
21278	202	0	13-Jul-14	3	2	13	2	3	1	0	10	4	7	1
21279	202	0	26-Aug-16	18	2	13	2	3	1	0	11	4	3	1
21280	101	1	13-Apr-06	12	1	13	2	1	1	0	7	4	4	1
21281	201	1	25-Dec-12	14	1	13	2	2	1	0	4	4	3	1
21282	201	1	17-Aug-16	15	1	13	2	1	1	0	11	4	3	1
21283	201	1	23-Feb-17	17	1	13	2	1	1	0	11	4	1	1
21284	201	1	12-Jun-09	14	1	13	2	3	1	0	6	4	3	1
21285	201	1	27-Jul-15	18	2	13	2	2	1	0	6	4	2	1
21286	201	1	2-Dec-16	10	2	13	2	1	1	0	11	4	2	1
21287	101	1	1-Feb-13	21	2	13	2	3	1	0	3	4	1	1
21288	101	1	31-Jan-06	15	2	13	2	2	1	0	3	4	3	2
21289	202	0	19-Nov-17	20	1	14	2	3	1	0	3	4	3	1
21290	202	0	16-Feb-10	13	2	14	2	1	1	0	10	4	7	1
21291	201	1	18-Jul-10	18	1	14	2	2	1	0	11	4	3	1
21292	201	1	29-Aug-12	21	1	14	2	1	1	0	3	4	2	1
21293	202	0	16-Feb-10	13	1	15	2	1	1	0	10	4	7	1
21294	101	1	5-Jul-08	21	1	15	2	2	1	0	6	4	2	1
21295	201	1	25-Nov-07	22	2	15	2	3	1	0	4	4	2	1
21296	201	1	3-Sep-06	16	1	17	2	3	1	0	4	4	2	2
21297	202	0	18-Jun-16	19	1	6	2	2	3	1	4	3	1	1
21298	202	0	5-Jul-09	19	1	6	2	1	3	1	4	3	2	1
21299	202	0	31-Aug-12	16	2	6	2	1	3	1	11	3	3	1
21300	101	1	5-Dec-06	15	1	6	2	1	3	1	4	3	1	1
21301	101	1	26-Sep-11	19	2	6	2	1	3	1	12	3	4	1
21302	101	1	23-Oct-11	14	2	6	2	1	3	1	11	3	4	1
21303	201	1	3-May-07	8	1	7	2	1	3	1	13	3	2	1
21304	101	1	18-May-16	12	1	7	2	1	3	1	4	3	4	1
21305	101	1	4-Dec-07	18	1	7	2	2	3	1	4	3	1	1
21306	101	1	30-Jan-11	18	1	7	2	1	3	1	3	3	1	1
21307	101	1	19-Dec-08	22	2	7	2	1	3	1	11	3	2	1
21308	202	0	24-Jul-17	15	1	8	2	3	3	1	4	3	4	1
21309	202	0	25-Sep-16	20	2	8	2	3	3	1	4	3	3	2
21310	201	1	9-Nov-08	18	1	8	2	1	3	1	13	3	5	1
21311	101	1	19-Oct-13	20	1	8	2	1	3	1	4	3	4	1
21312	101	1	11-Apr-10	16	1	8	2	1	3	1	11	3	3	1
21313	201	1	18-Jul-06	19	1	8	2	1	3	1	11	3	3	1
21314	101	1	13-May-15	17	1	8	2	1	3	1	3	3	1	1
21315	101	1	7-Jul-10	10	2	8	2	1	3	1	11	3	3	1
21316	202	0	12-Jul-07	19	1	9	2	1	3	1	4	3	4	1
21317	202	0	9-Jan-11	14	2	9	2	1	3	1	11	3	3	1
21318	201	1	9-Nov-08	18	1	9	2	1	3	1	13	3	5	1
21319	101	1	10-Aug-10	22	1	9	2	2	3	1	4	3	5	1
21320	101	1	4-Sep-07	7	2	9	2	1	3	1	4	3	1	1
21321	101	1	20-Sep-11	17	2	9	2	1	3	1	11	3	1	1

21322	101	1	15-Sep-11	17	2	9	2	1	3	1	11	3	3	1
21323	201	1	13-Jan-07	18	2	9	2	3	3	1	13	3	3	1
21324	202	0	1-Jul-16	16	1	10	2	3	3	1	4	3	1	1
21325	202	0	1-Jun-14	19	1	10	2	3	3	1	4	3	1	1
21326	202	0	20-May-11	11	1	10	2	1	3	1	11	3	3	1
21327	202	0	1-Nov-13	7	2	10	2	1	3	1	4	3	4	1
21328	202	0	7-Sep-12	20	2	10	2	1	3	1	11	3	3	2
21329	202	0	9-Jul-08	10	2	10	2	1	3	1	11	3	3	2
21330	201	1	25-Nov-16	18	1	10	2	1	3	1	4	3	1	2
21331	101	1	23-Feb-16	18	1	10	2	1	3	1	4	3	4	1
21332	101	1	24-Oct-14	22	1	10	2	1	3	1	4	3	5	1
21333	101	1	26-Dec-09	17	1	10	2	1	3	1	4	3	1	1
21334	101	1	10-Oct-07	11	1	10	2	1	3	1	4	3	5	1
21335	201	1	12-Sep-08	18	1	10	2	2	3	1	3	3	1	2
21336	201	1	31-Oct-08	18	1	11	2	3	3	1	13	3	4	1
21337	201	1	18-May-15	16	1	11	2	2	3	1	3	3	1	1
21338	201	1	3-Sep-06	12	1	11	2	1	3	1	3	3	2	1
21339	201	1	3-Sep-06	12	1	11	2	1	3	1	3	3	3	1
21340	202	0	16-Jun-11	17	1	12	2	1	3	1	13	3	1	1
21341	202	0	24-Jun-10	17	1	12	2	1	3	1	13	3	1	1
21342	202	0	14-Feb-07	14	1	12	2	1	3	1	13	3	6	1
21343	202	0	12-Feb-06	20	1	12	2	1	3	1	4	3	3	1
21344	202	0	25-Nov-17	20	1	12	2	1	3	1	4	3	1	1
21345	101	1	25-Oct-13	16	1	12	2	1	3	1	4	3	5	1
21346	101	1	25-Oct-13	16	1	12	2	1	3	1	4	3	5	1
21347	101	1	27-May-11	12	1	12	2	1	3	1	4	3	5	1
21348	201	1	16-Oct-11	3	1	12	2	1	3	1	6	3	4	1
21349	202	0	1-Jun-07	20	1	13	2	1	3	1	4	3	1	1
21350	201	1	14-Nov-09	1	1	13	2	1	3	1	4	3	7	2
21351	201	1	2-Jul-08	21	1	13	2	1	3	1	4	3	1	2
21352	201	1	24-Mar-06	23	1	13	2	3	3	1	11	3	3	1
21353	101	1	23-Apr-09	20	1	6	2	3	2	1	13	3	1	1
21354	101	1	22-Sep-11	21	2	10	2	1	2	1	11	3	3	1
21355	101	1	27-Jun-14	21	1	12	2	1	2	1	1	3	1	2
21356	101	1	12-Jun-09	14	1	12	2	1	2	1	4	3	3	1
21357	202	0	30-Oct-17	17	1	5	2	1	1	0	1	3	4	1
21358	202	0	20-Jul-15	20	1	5	2	1	1	0	1	3	3	1
21359	202	0	23-Jun-13	13	1	5	2	1	1	0	1	3	5	2
21360	202	0	15-Aug-16	20	1	5	2	1	1	0	4	3	3	2
21361	202	0	28-Jul-06	17	1	5	2	3	1	0	7	3	3	2
21362	202	0	15-Apr-13	17	1	5	2	1	1	0	1	3	1	1
21363	202	0	20-Nov-11	9	1	5	2	1	1	0	1	3	5	2
21364	202	0	16-Aug-11	22	1	5	2	1	1	0	1	3	6	2
21365	202	0	22-Sep-11	17	1	5	2	1	1	0	1	3	1	1
21366	202	0	9-Oct-11	8	1	5	2	3	1	0	1	3	6	1
21367	202	0	22-Mar-11	19	1	5	2	1	1	0	1	3	1	2
21368	202	0	24-Nov-11	13	1	5	2	3	1	0	1	3	1	1
21369	202	0	2-Nov-11	19	1	5	2	3	1	0	1	3	5	1
21370	202	0	14-Mar-11	17	1	5	2	2	1	0	1	3	1	2
21371	202	0	9-Dec-11	11	1	5	2	1	1	0	1	3	1	2
21372	202	0	2-Mar-11	17	1	5	2	1	1	0	1	3	1	1
21373	202	0	24-Jul-11	18	1	5	2	3	1	0	1	3	4	2
21374	202	0	4-Jul-10	16	1	5	2	1	1	0	1	3	3	2
21375	202	0	4-Aug-10	14	1	5	2	1	1	0	1	3	1	1
21376	202	0	7-Aug-10	17	1	5	2	1	1	0	1	3	1	1
21377	202	0	15-May-10	10	1	5	2	1	1	0	1	3	1	2
21378	202	0	22-Oct-10	9	1	5	2	1	1	0	1	3	1	1
21379	202	0	9-Oct-10	18	1	5	2	3	1	0	1	3	3	2
21380	202	0	4-Sep-10	12	1	5	2	1	1	0	1	3	2	1
21381	202	0	6-May-09	20	1	5	2	1	1	0	1	3	2	1
21382	202	0	19-Jul-09	20	1	5	2	3	1	0	1	3	6	2
21383	202	0	4-Oct-09	17	1	5	2	1	1	0	1	3	1	1
21384	202	0	21-Nov-09	8	1	5	2	1	1	0	1	3	7	1
21385	202	0	2-Jun-09	12	1	5	2	1	1	0	1	3	6	2
21386	202	0	6-Aug-09	19	1	5	2	1	1	0	1	3	1	1
21387	202	0	13-Sep-09	15	1	5	2	3	1	0	1	3	5	1
21388	202	0	23-Sep-09	10	1	5	2	1	1	0	1	3	1	1
21389	202	0	14-Jul-09	16	1	5	2	1	1	0	1	3	1	1
21390	202	0	1-Nov-09	16	1	5	2	1	1	0	1	3	3	2
21391	202	0	23-Nov-08	16	1	5	2	1	1	0	1	3	1	1
21392	202	0	14-Jan-08	12	1	5	2	2	1	0	1	3	1	2
21393	202	0	18-Nov-08	16	1	5	2	1	1	0	1	3	6	1
21394	202	0	8-Sep-08	8	1	5	2	1	1	0	1	3	6	1
21395	202	0	20-Oct-08	18	1	5	2	3	1	0	1	3	1	1
21396	202	0	3-Sep-08	18	1	5	2	1	1	0	1	3	7	1
21397	202	0	2-Jul-08	18	1	5	2	1	1	0	1	3	5	1
21398	202	0	23-Nov-08	17	1	5	2	1	1	0	1	3	1	2
21399	202	0	9-Jan-07	13	1	5	2	2	1	0	1	3	1	1
21400	202	0	5-Oct-07	11	1	5	2	3	1	0	1	3	3	1
21401	202	0	28-Jan-07	17	1	5	2	3	1	0	1	3	7	1
21402	202	0	2-Dec-06	21	1	5	2	1	1	0	1	3	5	1
21403	202	0	20-Sep-06	8	1	5	2	1	1	0	1	3	1	1
21404	202	0	5-Nov-06	12	1	5	2	3	1	0	1	3	3	1
21405	202	0	17-Jul-06	9	1	5	2	3	1	0	1	3	1	1
21406	202	0	22-Oct-06	19	1	5	2	1	1	0	1	3	3	1
21407	202	0	8-Dec-06	13	1	5	2	3	1	0	1	3	3	1
21408	202	0	8-Dec-06	19	1	5	2	3	1	0	1	3	3	1
21409	202	0	13-Sep-10	17	1	5	2	2	1	0	1	3	3	2
21410	202	0	8-Nov-09	17	1	5	2	1	1	0	1	3	3	1
21411	202	0	30-May-09	12	1	5	2	1	1	0	1	3	3	1
21412	202	0	6-Sep-09	21	1	5	2	1	1	0	1	3	3	1
21413	202	0	15-Aug-09	19	1	5	2	1	1	0	1	3	3	2
21414	202	0	15-Aug-08	18	1	5	2	1	1	0	1	3	4	2
21415	202	0	18-Apr-07	9	1	5	2	1	1	0	1	3	3	2
21416	202	0	12-Apr-07	18	1	5	2	1	1	0	1	3	7	2
21417	202	0	25-Aug-14	20	1	5	2	3	1	0	4	3	2	1
21418	202	0	14-Aug-10	16	1	5	2	1	1	0	4	3	1	1
21419	202	0	2-Jul-09	19	1	5	2	1	1	0	4	3	2	1
21420	202	0	15-Aug-09	20	1	5	2	3	1	0	4	3	1	1
21421	202	0	23-Jul-07	19	1	5	2	1	1	0	4	3	1	1
21422	202	0	27-Aug-10	20	1	5	2	1	1	0	11	3	1	1
21423	202	0	19-Jul-10	10	1	5	2	2	1	0	11	3	3	1
21424	202	0	19-Aug-10	19	1	5	2	3	1	0	11	3	3	2
21425	202	0	14-Oct-14	20	1	5	2	1	1	0	3	3	1	1
21426	202	0	20-Feb-11	13	1	5	2	1	1	0	3	3	1	1
21427	202	0	19-Apr-11	18	1	5	2	1	1	0	3	3	1	1
21428	202	0	16-Mar-11	17	1	5	2	1	1	0	3	3	1	1
21429	202	0	16-Jul-11	22	1	5	2	1	1	0	3	3	1	2
21430	202	0	25-Oct-10	17	1	5	2	1	1	0	3	3	1	1
21431	202	0	20-May-10	21	1	5	2	3	1	0	3	3	1	2
21432	202	0	31-Jul-10	18	1	5	2	1	1	0	3	3	1	1
21433	202	0	3-Jan-09	14	1	5	2	1	1	0	3	3	1	1
21434	202	0	7-Feb-07	14	1	5	2	1	1	0	3	3	1	1
21435	202	0	22-May-07	18	1	5	2	3	1	0	3	3	3	2
21436	202	0	30-Jun-07	19	1	5	2	1	1	0	3	3	1	1
21437	202	0	12-May-07	20	1	5	2	2	1	0	3	3	3	1
21438	202	0	28-Sep-06	14	1	5	2	2	1	0	13	3	1	1
21439	202	0	7-Jun-09	8	2	5	2	1	1	0	7	3	6	2
21440	202	0	10-Apr-11	21	2	5	2	1	1	0	1	3	6	2
21441	202	0	12-Sep-11	17	2	5	2	1	1	0	1	3	1	2
21442	202	0	27-Nov-11	14	2	5	2	1	1	0	1	3	3	1
21443	202	0	5-Jun-11	19	2	5	2	3	1	0	1	3	1	2
21444	202	0	17-Dec-11	18	2	5	2	1	1	0	1	3	1	1
21445	202	0	8-Aug-11	17	2	5	2	2	1	0	1	3	3	1

21446	202	0	27-Feb-10	14	2	5	2	1	1	0	1	3	5	2
21447	202	0	23-May-10	18	2	5	2	1	1	0	1	3	3	1
21448	202	0	6-Aug-10	20	2	5	2	3	1	0	1	3	6	2
21449	202	0	7-May-10	22	2	5	2	1	1	0	1	3	1	2
21450	202	0	15-May-10	17	2	5	2	1	1	0	1	3	2	1
21451	202	0	21-Feb-10	13	2	5	2	2	1	0	1	3	3	1
21452	202	0	30-Sep-09	18	2	5	2	1	1	0	1	3	1	1
21453	202	0	28-May-09	15	2	5	2	1	1	0	1	3	1	1
21454	202	0	26-Jul-09	13	2	5	2	1	1	0	1	3	3	1
21455	202	0	4-Jul-09	18	2	5	2	3	1	0	1	3	6	1
21456	202	0	1-Oct-09	16	2	5	2	1	1	0	1	3	1	1
21457	202	0	15-May-09	19	2	5	2	3	1	0	1	3	3	1
21458	202	0	2-Oct-08	10	2	5	2	1	1	0	1	3	1	2
21459	202	0	6-Jul-08	18	2	5	2	3	1	0	1	3	1	2
21460	202	0	25-Dec-08	12	2	5	2	1	1	0	1	3	3	1
21461	202	0	1-May-08	16	2	5	2	3	1	0	1	3	3	2
21462	202	0	24-Apr-07	18	2	5	2	1	1	0	1	3	2	1
21463	202	0	22-Aug-07	17	2	5	2	1	1	0	1	3	3	2
21464	202	0	18-Feb-07	4	2	5	2	3	1	0	1	3	1	1
21465	202	0	25-Dec-07	19	2	5	2	1	1	0	1	3	3	1
21466	202	0	31-Aug-07	12	2	5	2	1	1	0	1	3	5	2
21467	202	0	29-Aug-07	18	2	5	2	1	1	0	1	3	1	2
21468	202	0	23-Dec-07	16	2	5	2	1	1	0	1	3	3	1
21469	202	0	6-Sep-06	16	2	5	2	3	1	0	1	3	6	1
21470	202	0	12-Sep-06	7	2	5	2	3	1	0	1	3	3	1
21471	202	0	11-Mar-08	7	2	5	2	1	1	0	1	3	3	1
21472	202	0	18-Apr-08	23	2	5	2	1	1	0	4	3	2	1
21473	202	0	5-Jan-07	15	2	5	2	1	1	0	4	3	1	1
21474	202	0	30-May-09	7	2	5	2	1	1	0	11	3	4	1
21475	202	0	7-Feb-11	15	2	5	2	1	1	0	3	3	1	1
21476	202	0	19-Oct-10	18	2	5	2	1	1	0	3	3	1	1
21477	202	0	4-Apr-09	16	2	5	2	1	1	0	3	3	1	1
21478	202	0	13-Feb-07	23	2	5	2	1	1	0	3	3	2	1
21479	201	1	3-Sep-12	7	1	5	2	2	1	0	1	3	1	1
21480	201	1	3-Oct-12	20	1	5	2	3	1	0	1	3	5	1
21481	101	1	13-Aug-17	18	1	5	2	1	1	0	11	3	3	1
21482	201	1	16-Mar-08	13	1	5	2	2	1	0	13	3	1	1
21483	101	1	19-Jul-08	19	1	5	2	1	1	0	13	3	3	1
21484	201	1	21-Sep-11	14	1	5	2	3	1	0	1	3	1	1
21485	201	1	1-Aug-11	10	1	5	2	1	1	0	1	3	1	1
21486	201	1	8-Sep-11	4	1	5	2	1	1	0	1	3	1	1
21487	201	1	25-Oct-11	18	1	5	2	1	1	0	1	3	1	1
21488	201	1	25-Sep-11	8	1	5	2	1	1	0	1	3	4	1
21489	201	1	8-Jul-11	12	1	5	2	1	1	0	1	3	2	1
21490	201	1	20-Oct-11	18	1	5	2	2	1	0	1	3	1	1
21491	101	1	30-Oct-11	13	1	5	2	1	1	0	1	3	5	1
21492	101	1	15-Aug-11	21	1	5	2	3	1	0	1	3	5	1
21493	201	1	4-Apr-11	16	1	5	2	1	1	0	1	3	5	2
21494	101	1	30-Jan-11	11	1	5	2	1	1	0	1	3	1	1
21495	101	1	5-Nov-11	18	1	5	2	1	1	0	1	3	5	2
21496	201	1	1-Feb-11	9	1	5	2	2	1	0	1	3	3	2
21497	201	1	29-May-10	19	1	5	2	1	1	0	1	3	6	1
21498	101	1	13-Jul-10	14	1	5	2	1	1	0	1	3	5	1
21499	201	1	25-Aug-10	21	1	5	2	2	1	0	1	3	1	1
21500	101	1	25-Jul-10	9	1	5	2	2	1	0	1	3	3	2
21501	201	1	21-Sep-10	10	1	5	2	1	1	0	1	3	3	1
21502	101	1	5-Jul-10	21	1	5	2	1	1	0	1	3	3	1
21503	101	1	11-Sep-10	15	1	5	2	1	1	0	1	3	2	1
21504	101	1	1-May-09	18	1	5	2	2	1	0	1	3	4	1
21505	201	1	3-May-09	18	1	5	2	1	1	0	1	3	1	1
21506	101	1	25-Feb-09	18	1	5	2	1	1	0	1	3	1	1
21507	101	1	14-Apr-09	22	1	5	2	1	1	0	1	3	1	2
21508	201	1	27-May-09	18	1	5	2	2	1	0	1	3	6	1
21509	101	1	18-Jun-09	19	1	5	2	1	1	0	1	3	5	2
21510	101	1	20-Jun-09	17	1	5	2	1	1	0	1	3	1	2
21511	201	1	9-Nov-09	18	1	5	2	1	1	0	1	3	2	1
21512	201	1	13-Nov-09	15	1	5	2	1	1	0	1	3	1	1
21513	201	1	14-Apr-09	20	1	5	2	1	1	0	1	3	2	2
21514	201	1	1-Nov-09	0	1	5	2	2	1	0	1	3	3	1
21515	201	1	26-May-09	19	1	5	2	2	1	0	1	3	5	2
21516	201	1	8-Dec-09	19	1	5	2	2	1	0	1	3	3	2
21517	201	1	25-Jun-09	20	1	5	2	2	1	0	1	3	6	1
21518	101	1	16-Mar-08	17	1	5	2	3	1	0	1	3	6	2
21519	201	1	8-Aug-08	18	1	5	2	3	1	0	1	3	5	2
21520	101	1	6-Aug-08	17	1	5	2	1	1	0	1	3	5	2
21521	101	1	27-Mar-08	19	1	5	2	1	1	0	1	3	2	4
21522	201	1	23-Mar-08	16	1	5	2	1	1	0	1	3	3	1
21523	101	1	23-Apr-08	20	1	5	2	1	1	0	1	3	1	1
21524	101	1	21-Nov-08	18	1	5	2	1	1	0	1	3	3	4
21525	201	1	30-Sep-08	12	1	5	2	3	1	0	1	3	3	1
21526	101	1	31-May-08	15	1	5	2	1	1	0	1	3	3	2
21527	101	1	23-May-08	18	1	5	2	1	1	0	1	3	3	1
21528	201	1	25-May-08	13	1	5	2	1	1	0	1	3	1	1
21529	101	1	4-Dec-07	16	1	5	2	2	1	0	1	3	1	2
21530	201	1	26-Aug-07	15	1	5	2	1	1	0	1	3	1	1
21531	101	1	23-Jan-07	21	1	5	2	1	1	0	1	3	1	2
21532	201	1	22-Oct-07	8	1	5	2	1	1	0	1	3	1	1
21533	101	1	20-Feb-07	3	1	5	2	1	1	0	1	3	1	2
21534	101	1	23-Sep-07	10	1	5	2	1	1	0	1	3	1	1
21535	101	1	12-May-07	17	1	5	2	1	1	0	1	3	3	1
21536	201	1	30-Jul-07	18	1	5	2	1	1	0	1	3	1	1
21537	101	1	29-Sep-07	20	1	5	2	2	1	0	1	3	1	2
21538	101	1	13-Jun-07	18	1	5	2	2	1	0	1	3	1	1
21539	201	1	5-Nov-07	17	1	5	2	3	1	0	1	3	1	1
21540	201	1	26-Sep-07	1	1	5	2	2	1	0	1	3	6	2
21541	201	1	24-Oct-07	18	1	5	2	3	1	0	1	3	3	1
21542	101	1	24-Aug-07	16	1	5	2	1	1	0	1	3	3	2
21543	101	1	2-Jan-07	19	1	5	2	1	1	0	1	3	3	1
21544	201	1	6-Sep-06	13	1	5	2	1	1	0	1	3	1	2
21545	101	1	22-Oct-06	16	1	5	2	1	1	0	1	3	1	2
21546	101	1	7-Aug-06	9	1	5	2	3	1	0	1	3	3	1
21547	201	1	31-Jul-11	13	1	5	2	1	1	0	1	3	6	1
21548	101	1	20-Jul-11	17	1	5	2	1	1	0	1	3	4	1
21549	101	1	27-Jul-11	19	1	5	2	1	1	0	1	3	4	1
21550	101	1	26-Dec-11	15	1	5	2	1	1	0	1	3	3	1
21551	101	1	7-Jul-11	19	1	5	2	3	1	0	1	3	3	1
21552	201	1	2-May-10	21	1	5	2	1	1	0	1	3	1	1
21553	201	1	9-Sep-10	18	1	5	2	1	1	0	1	3	3	1
21554	101	1	10-Jan-10	21	1	5	2	2	1	0	1	3	5	1
21555	201	1	20-Sep-09	17	1	5	2	1	1	0	1	3	1	1
21556	101	1	7-Oct-09	17	1	5	2	1	1	0	1	3	5	1
21557	101	1	7-Apr-09	18	1	5	2	1	1	0	1	3	3	1
21558	201	1	10-Jun-08	18	1	5	2	3	1	0	1	3	3	1
21559	101	1	5-Dec-08	7	1	5	2	1	1	0	1	3	3	1
21560	201	1	10-Apr-08	8	1	5	2	2	1	0	1	3	3	1
21561	201	1	26-Oct-07	8	1	5	2	3	1	0	1	3	1	1
21562	201	1	9-May-07	12	1	5	2	1	1	0	1	3	3	1
21563	101	1	21-Jul-07	16	1	5	2	1	1	0	1	3	1	2
21564	101	1	30-Aug-07	18	1	5	2	1	1	0	1	3	4	1
21565	101	1	15-Apr-07	10	1	5	2	1	1	0	1	3	3	1
21566	201	1	18-Jul-06	20	1	5	2	1	1	0	1	3	5	2
21567	101	1	10-Dec-06	20	1	5	2	1	1	0	1	3	1	1
21568	101	1	22-Aug-06	19	1	5	2	1	1	0	1	3	3	1
21569	101	1	11-Dec-11	18	1	5	2	1	1	0	4	3	1	4

21570	101	1	23-Aug-11	15	1	5	2	1	1	0	4	3	3	1
21571	101	1	3-Sep-10	7	1	5	2	1	1	0	4	3	3	1
21572	101	1	10-May-07	20	1	5	2	1	1	0	4	3	2	1
21573	101	1	14-Mar-07	17	1	5	2	1	1	0	4	3	1	1
21574	201	1	7-Sep-11	18	1	5	2	2	1	0	11	3	3	1
21575	101	1	11-Dec-11	15	1	5	2	1	1	0	11	3	2	1
21576	201	1	27-Feb-10	16	1	5	2	1	1	0	11	3	3	1
21577	201	1	6-Aug-10	14	1	5	2	1	1	0	11	3	2	1
21578	101	1	18-Nov-09	18	1	5	2	2	1	0	11	3	4	1
21579	201	1	24-Oct-09	20	1	5	2	2	1	0	11	3	3	1
21580	101	1	3-Mar-08	21	1	5	2	3	1	0	11	3	3	1
21581	201	1	4-Oct-07	16	1	5	2	2	1	0	11	3	3	1
21582	201	1	3-Mar-16	13	1	5	2	1	1	0	3	3	1	2
21583	201	1	5-May-11	15	1	5	2	1	1	0	3	3	1	1
21584	201	1	22-Apr-11	15	1	5	2	3	1	0	3	3	2	1
21585	201	1	11-Aug-09	18	1	5	2	1	1	0	3	3	5	1
21586	201	1	11-Sep-09	18	1	5	2	1	1	0	3	3	1	1
21587	201	1	3-Jun-09	19	1	5	2	2	1	0	3	3	2	1
21588	201	1	23-Jul-09	21	1	5	2	3	1	0	3	3	2	1
21589	201	1	22-Nov-09	15	1	5	2	3	1	0	3	3	1	1
21590	101	1	17-Jul-08	20	1	5	2	1	1	0	3	3	1	1
21591	201	1	18-Jul-08	16	1	5	2	1	1	0	3	3	1	1
21592	101	1	21-Oct-08	11	1	5	2	1	1	0	3	3	2	1
21593	201	1	17-May-08	18	1	5	2	1	1	0	3	3	2	1
21594	101	1	30-Jul-08	9	1	5	2	1	1	0	3	3	3	1
21595	101	1	15-Dec-07	20	1	5	2	1	1	0	3	3	1	1
21596	201	1	21-Jan-07	18	1	5	2	1	1	0	3	3	1	1
21597	101	1	6-Jun-07	8	1	5	2	1	1	0	3	3	3	1
21598	101	1	26-Jun-07	8	1	5	2	1	1	0	3	3	2	1
21599	101	1	28-Oct-07	21	1	5	2	1	1	0	3	3	1	1
21600	201	1	16-Aug-06	14	1	5	2	1	1	0	3	3	1	1
21601	201	1	24-Jun-06	19	1	5	2	2	1	0	3	3	1	1
21602	201	1	29-Sep-06	18	1	5	2	3	1	0	3	3	1	1
21603	201	1	9-Oct-06	16	1	5	2	1	1	0	3	3	1	1
21604	201	1	17-Jul-06	20	1	5	2	2	1	0	3	3	3	1
21605	201	1	28-Jul-06	19	1	5	2	3	1	0	3	3	7	1
21606	201	1	7-Aug-08	17	1	5	2	1	1	0	6	3	3	1
21607	101	1	29-Jul-07	19	1	5	2	1	1	0	6	3	3	1
21608	201	1	18-Mar-12	11	2	5	2	1	1	0	1	3	1	1
21609	101	1	6-Apr-10	20	2	5	2	1	1	0	13	3	2	1
21610	201	1	23-Apr-07	17	2	5	2	1	1	0	13	3	3	1
21611	201	1	19-Dec-11	15	2	5	2	3	1	0	1	3	1	1
21612	101	1	23-Jul-11	18	2	5	2	3	1	0	1	3	1	1
21613	101	1	23-Jul-11	18	2	5	2	3	1	0	1	3	1	1
21614	101	1	11-May-11	17	2	5	2	3	1	0	1	3	5	1
21615	101	1	6-Nov-11	17	2	5	2	1	1	0	1	3	3	1
21616	201	1	21-May-10	14	2	5	2	2	1	0	1	3	5	1
21617	201	1	18-Jun-10	8	2	5	2	3	1	0	1	3	1	1
21618	201	1	24-Apr-10	21	2	5	2	3	1	0	1	3	1	1
21619	201	1	21-Apr-10	23	2	5	2	2	1	0	1	3	5	1
21620	201	1	23-Aug-10	10	2	5	2	2	1	0	1	3	2	1
21621	101	1	14-Jul-10	10	2	5	2	2	1	0	1	3	3	1
21622	201	1	13-Dec-09	16	2	5	2	1	1	0	1	3	5	1
21623	201	1	14-Jun-09	21	2	5	2	1	1	0	1	3	6	1
21624	101	1	2-Jul-09	9	2	5	2	2	1	0	1	3	5	2
21625	101	1	20-Jun-09	9	2	5	2	1	1	0	1	3	1	1
21626	201	1	4-Jan-08	23	2	5	2	1	1	0	1	3	1	1
21627	201	1	15-Jan-08	13	2	5	2	3	1	0	1	3	3	2
21628	101	1	29-Sep-08	14	2	5	2	3	1	0	1	3	4	2
21629	101	1	26-Jul-08	9	2	5	2	1	1	0	1	3	3	1
21630	101	1	27-Apr-08	12	2	5	2	1	1	0	1	3	3	2
21631	201	1	8-Mar-07	19	2	5	2	1	1	0	1	3	1	1
21632	201	1	19-Jan-07	11	2	5	2	1	1	0	1	3	1	1
21633	101	1	15-Apr-07	17	2	5	2	2	1	0	1	3	1	1
21634	201	1	31-Mar-07	13	2	5	2	1	1	0	1	3	2	1
21635	101	1	19-Jun-07	18	2	5	2	1	1	0	1	3	5	1
21636	101	1	4-Apr-07	9	2	5	2	1	1	0	1	3	1	1
21637	201	1	2-Jan-07	1	2	5	2	1	1	0	1	3	2	2
21638	101	1	16-Jun-07	17	2	5	2	1	1	0	1	3	3	1
21639	101	1	22-Jul-07	17	2	5	2	1	1	0	1	3	1	1
21640	201	1	23-Jul-06	19	2	5	2	2	1	0	1	3	4	2
21641	101	1	9-May-17	8	2	5	2	1	1	0	1	3	3	1
21642	101	1	20-Apr-10	8	2	5	2	1	1	0	1	3	1	1
21643	101	1	11-Jul-10	15	2	5	2	1	1	0	1	3	1	1
21644	101	1	27-Apr-09	12	2	5	2	1	1	0	1	3	3	1
21645	201	1	27-Jul-09	18	2	5	2	1	1	0	1	3	4	2
21646	201	1	11-Jan-09	17	2	5	2	2	1	0	1	3	3	1
21647	201	1	2-May-08	18	2	5	2	3	1	0	1	3	3	1
21648	201	1	15-Apr-08	10	2	5	2	3	1	0	1	3	2	1
21649	201	1	2-Jul-07	11	2	5	2	1	1	0	1	3	1	1
21650	201	1	18-Sep-07	14	2	5	2	1	1	0	1	3	2	1
21651	201	1	22-Apr-07	20	2	5	2	1	1	0	1	3	1	1
21652	101	1	26-Apr-07	7	2	5	2	1	1	0	1	3	1	1
21653	201	1	13-Jun-07	19	2	5	2	3	1	0	1	3	1	1
21654	201	1	25-Jan-07	12	2	5	2	1	1	0	1	3	3	1
21655	201	1	29-Sep-06	20	2	5	2	1	1	0	1	3	6	1
21656	201	1	11-Oct-06	17	2	5	2	2	1	0	1	3	1	2
21657	101	1	13-Feb-09	8	2	5	2	1	1	0	4	3	3	1
21658	101	1	13-Aug-09	18	2	5	2	1	1	0	4	3	2	1
21659	101	1	12-Jul-08	19	2	5	2	1	1	0	4	3	3	1
21660	101	1	5-Nov-11	11	2	5	2	3	1	0	11	3	1	1
21661	101	1	19-Apr-11	18	2	5	2	1	1	0	11	3	4	1
21662	101	1	1-Apr-10	17	2	5	2	1	1	0	11	3	3	1
21663	201	1	9-Jul-10	21	2	5	2	1	1	0	11	3	3	1
21664	201	1	11-Oct-09	17	2	5	2	1	1	0	11	3	3	1
21665	201	1	15-Dec-09	18	2	5	2	2	1	0	11	3	2	1
21666	201	1	28-Sep-10	19	2	5	2	1	1	0	3	3	1	1
21667	101	1	4-Sep-10	18	2	5	2	1	1	0	3	3	1	1
21668	101	1	26-Apr-09	17	2	5	2	1	1	0	3	3	2	1
21669	101	1	27-Sep-09	18	2	5	2	1	1	0	3	3	2	1
21670	101	1	31-Dec-09	18	2	5	2	1	1	0	3	3	2	1
21671	101	1	21-Mar-09	17	2	5	2	1	1	0	3	3	2	1
21672	201	1	22-Jun-08	14	2	5	2	2	1	0	3	3	1	1
21673	201	1	21-Oct-08	21	2	5	2	1	1	0	3	3	1	1
21674	201	1	3-Jul-08	19	2	5	2	1	1	0	3	3	3	1
21675	101	1	27-May-08	17	2	5	2	1	1	0	3	3	3	1
21676	101	1	10-Jul-08	12	2	5	2	1	1	0	3	3	3	1
21677	201	1	1-Oct-07	12	2	5	2	1	1	0	3	3	1	1
21678	101	1	24-Nov-07	21	2	5	2	1	1	0	3	3	2	1
21679	101	1	30-Apr-07	22	2	5	2	1	1	0	3	3	2	1
21680	201	1	8-Sep-06	22	2	5	2	1	1	0	3	3	4	2
21681	201	1	4-Aug-07	9	2	5	2	1	1	0	6	3	3	1
21682	202	0	7-Jul-15	17	1	6	2	2	1	0	1	3	4	2
21683	202	0	18-Mar-17	16	1	6	2	3	1	0	1	3	5	1
21684	202	0	14-Apr-16	22	1	6	2	1	1	0	1	3	1	1
21685	202	0	30-Aug-17	8	1	6	2	4	1	0	1	3	2	1
21686	202	0	28-Sep-15	15	1	6	2	1	1	0	1	3	2	1
21687	202	0	29-Jun-14	18	1	6	2	1	1	0	1	3	2	2
21688	202	0	29-Oct-16	20	1	6	2	2	1	0	1	3	1	1
21689	202	0	26-May-13	15	1	6	2	1	1	0	1	3	1	1
21690	202	0	19-Feb-17	12	1	6	2	1	1	0	1	3	1	1
21691	202	0	1-Dec-17	16	1	6	2	1	1	0	1	3	1	1
21692	202	0	29-Aug-13	11	1	6	2	1	1	0	1	3	5	2
21693	202	0	24-Jul-16	18	1	6	2	3	1	0	1	3	1	1

21694	202	0	17-Apr-16	12	1	6	2	3	1	0	1	3	1	1
21695	202	0	5-Jul-16	17	1	6	2	1	1	0	1	3	3	1
21696	202	0	16-Sep-12	20	1	6	2	1	1	0	5	3	3	1
21697	202	0	19-Jan-16	8	1	6	2	1	1	0	1	3	4	1
21698	202	0	3-Nov-16	7	1	6	2	2	1	0	1	3	3	2
21699	202	0	25-Feb-13	17	1	6	2	1	1	0	1	3	4	1
21700	202	0	24-Jul-16	19	1	6	2	1	1	0	1	3	3	1
21701	202	0	25-Aug-16	17	1	6	2	1	1	0	1	3	4	1
21702	202	0	7-Nov-15	22	1	6	2	3	1	0	1	3	3	1
21703	202	0	16-Dec-13	12	1	6	2	3	1	0	1	3	3	1
21704	202	0	12-Sep-13	19	1	6	2	3	1	0	1	3	5	1
21705	202	0	6-May-17	13	1	6	2	1	1	0	1	3	5	1
21706	202	0	6-Nov-17	18	1	6	2	1	1	0	1	3	3	1
21707	202	0	1-Aug-17	18	1	6	2	1	1	0	1	3	1	1
21708	202	0	19-May-17	12	1	6	2	1	1	0	1	3	1	1
21709	202	0	9-Apr-17	15	1	6	2	1	1	0	1	3	1	1
21710	202	0	17-Jul-17	15	1	6	2	3	1	0	1	3	3	1
21711	202	0	5-Oct-17	15	1	6	2	1	1	0	1	3	5	1
21712	202	0	8-Aug-15	20	1	6	2	1	1	0	4	3	7	1
21713	202	0	8-Jul-14	12	1	6	2	1	1	0	7	3	3	1
21714	202	0	16-Feb-13	9	1	6	2	1	1	0	1	3	1	2
21715	202	0	12-Sep-12	11	1	6	2	3	1	0	1	3	3	2
21716	202	0	27-Nov-11	11	1	6	2	1	1	0	1	3	5	2
21717	202	0	17-Aug-11	17	1	6	2	1	1	0	1	3	6	1
21718	202	0	25-Jun-11	8	1	6	2	1	1	0	1	3	2	1
21719	202	0	25-Oct-11	15	1	6	2	1	1	0	1	3	2	2
21720	202	0	24-Dec-11	16	1	6	2	1	1	0	1	3	2	1
21721	202	0	29-Dec-11	13	1	6	2	1	1	0	1	3	1	2
21722	202	0	12-Nov-11	13	1	6	2	1	1	0	1	3	1	1
21723	202	0	8-Feb-11	10	1	6	2	1	1	0	1	3	3	2
21724	202	0	21-Dec-11	18	1	6	2	1	1	0	1	3	3	1
21725	202	0	25-Jun-11	13	1	6	2	1	1	0	1	3	3	1
21726	202	0	31-Aug-11	8	1	6	2	1	1	0	1	3	1	1
21727	202	0	13-Dec-10	7	1	6	2	1	1	0	1	3	6	2
21728	202	0	10-Jun-10	11	1	6	2	2	1	0	1	3	5	1
21729	202	0	3-Oct-10	18	1	6	2	1	1	0	1	3	1	2
21730	202	0	18-Sep-10	21	1	6	2	1	1	0	1	3	1	1
21731	202	0	24-Sep-10	7	1	6	2	1	1	0	1	3	7	1
21732	202	0	19-Dec-10	22	1	6	2	3	1	0	1	3	3	1
21733	202	0	1-Mar-10	22	1	6	2	1	1	0	1	3	6	2
21734	202	0	4-May-10	18	1	6	2	2	1	0	1	3	3	1
21735	202	0	1-Jan-09	11	1	6	2	1	1	0	1	3	7	1
21736	202	0	21-Mar-09	20	1	6	2	2	1	0	1	3	6	2
21737	202	0	8-Nov-09	20	1	6	2	1	1	0	1	3	1	1
21738	202	0	4-Dec-09	19	1	6	2	1	1	0	1	3	1	2
21739	202	0	13-May-09	16	1	6	2	1	1	0	1	3	1	1
21740	202	0	1-Jan-09	16	1	6	2	1	1	0	1	3	3	2
21741	202	0	30-May-09	14	1	6	2	1	1	0	1	3	3	1
21742	202	0	6-Jun-09	18	1	6	2	3	1	0	1	3	6	2
21743	202	0	25-Jul-09	17	1	6	2	3	1	0	1	3	5	1
21744	202	0	17-Nov-09	11	1	6	2	1	1	0	1	3	7	1
21745	202	0	25-Oct-09	11	1	6	2	1	1	0	1	3	3	2
21746	202	0	3-May-08	18	1	6	2	1	1	0	1	3	6	1
21747	202	0	10-Sep-08	15	1	6	2	1	1	0	1	3	3	2
21748	202	0	20-Aug-08	17	1	6	2	3	1	0	1	3	6	2
21749	202	0	6-Nov-08	16	1	6	2	1	1	0	1	3	1	1
21750	202	0	1-Jan-08	15	1	6	2	4	1	0	1	3	1	1
21751	202	0	4-Jan-08	17	1	6	2	1	1	0	1	3	3	1
21752	202	0	16-Sep-08	21	1	6	2	3	1	0	1	3	4	1
21753	202	0	23-Nov-08	16	1	6	2	1	1	0	1	3	6	1
21754	202	0	18-Sep-08	14	1	6	2	1	1	0	1	3	3	1
21755	202	0	21-Jun-08	19	1	6	2	1	1	0	1	3	6	2
21756	202	0	10-Mar-08	17	1	6	2	1	1	0	1	3	6	1
21757	202	0	25-May-08	20	1	6	2	2	1	0	1	3	1	1
21758	202	0	22-Mar-07	18	1	6	2	3	1	0	1	3	1	2
21759	202	0	22-Mar-07	18	1	6	2	3	1	0	1	3	1	2
21760	202	0	1-Oct-07	17	1	6	2	3	1	0	1	3	1	1
21761	202	0	18-Jan-07	21	1	6	2	1	1	0	1	3	1	2
21762	202	0	2-Aug-07	20	1	6	2	1	1	0	1	3	6	1
21763	202	0	19-Sep-07	12	1	6	2	3	1	0	1	3	2	1
21764	202	0	27-Feb-07	19	1	6	2	1	1	0	1	3	3	1
21765	202	0	16-Jul-07	18	1	6	2	1	1	0	1	3	5	1
21766	202	0	15-Jun-07	8	1	6	2	3	1	0	1	3	5	1
21767	202	0	3-Mar-07	12	1	6	2	1	1	0	1	3	2	1
21768	202	0	23-Mar-07	12	1	6	2	1	1	0	1	3	1	1
21769	202	0	1-Oct-07	18	1	6	2	1	1	0	1	3	3	1
21770	202	0	21-Feb-07	14	1	6	2	1	1	0	1	3	3	2
21771	202	0	16-Apr-07	19	1	6	2	3	1	0	1	3	2	1
21772	202	0	24-Dec-06	19	1	6	2	2	1	0	1	3	1	1
21773	202	0	24-Aug-06	18	1	6	2	1	1	0	1	3	1	1
21774	202	0	14-Sep-06	17	1	6	2	2	1	0	1	3	1	1
21775	202	0	8-Sep-06	15	1	6	2	1	1	0	1	3	3	1
21776	202	0	23-Oct-06	16	1	6	2	3	1	0	1	3	6	1
21777	202	0	24-Nov-06	16	1	6	2	2	1	0	1	3	3	1
21778	202	0	7-Jul-06	14	1	6	2	1	1	0	1	3	4	1
21779	202	0	14-Nov-06	17	1	6	2	1	1	0	1	3	2	1
21780	202	0	21-Aug-06	20	1	6	2	1	1	0	1	3	3	1
21781	202	0	25-Jul-09	17	1	6	2	1	1	0	1	3	6	2
21782	202	0	17-Feb-07	17	1	6	2	3	1	0	1	3	3	1
21783	202	0	18-Mar-07	18	1	6	2	2	1	0	1	3	3	1
21784	202	0	3-Nov-16	15	1	6	2	1	1	0	4	3	1	1
21785	202	0	25-Jun-16	20	1	6	2	2	1	0	4	3	7	1
21786	202	0	12-Aug-14	14	1	6	2	3	1	0	4	3	1	1
21787	202	0	19-Dec-11	19	1	6	2	3	1	0	4	3	3	2
21788	202	0	16-Jun-11	19	1	6	2	1	1	0	4	3	1	2
21789	202	0	30-Oct-08	10	1	6	2	3	1	0	4	3	7	2
21790	202	0	25-Aug-17	12	1	6	2	1	1	0	4	3	1	1
21791	202	0	11-Mar-10	14	1	6	2	1	1	0	11	3	3	2
21792	202	0	29-May-10	10	1	6	2	3	1	0	11	3	3	2
21793	202	0	19-Apr-07	13	1	6	2	1	1	0	11	3	3	1
21794	202	0	18-Dec-06	13	1	6	2	1	1	0	11	3	2	1
21795	202	0	14-Jan-16	16	1	6	2	2	1	0	3	3	1	1
21796	202	0	17-Dec-16	17	1	6	2	3	1	0	3	3	1	1
21797	202	0	5-Aug-16	21	1	6	2	1	1	0	3	3	1	1
21798	202	0	11-Nov-15	14	1	6	2	3	1	0	3	3	1	1
21799	202	0	30-Dec-15	10	1	6	2	1	1	0	3	3	5	1
21800	202	0	24-Sep-15	12	1	6	2	3	1	0	3	3	3	1
21801	202	0	13-Oct-14	14	1	6	2	1	1	0	3	3	1	1
21802	202	0	9-Dec-14	14	1	6	2	1	1	0	3	3	1	1
21803	202	0	10-Sep-14	20	1	6	2	1	1	0	3	3	1	1
21804	202	0	9-Dec-14	20	1	6	2	3	1	0	3	3	4	1
21805	202	0	15-Oct-14	16	1	6	2	1	1	0	3	3	2	1
21806	202	0	25-Jun-13	21	1	6	2	1	1	0	3	3	3	2
21807	202	0	20-Sep-12	17	1	6	2	1	1	0	3	3	1	1
21808	202	0	14-Nov-12	7	1	6	2	1	1	0	3	3	2	2
21809	202	0	9-Oct-11	16	1	6	2	1	1	0	3	3	2	1
21810	202	0	17-Jan-11	13	1	6	2	1	1	0	3	3	1	1
21811	202	0	24-Feb-11	18	1	6	2	1	1	0	3	3	1	2
21812	202	0	11-Jun-11	21	1	6	2	1	1	0	3	3	3	1
21813	202	0	16-Apr-10	19	1	6	2	1	1	0	3	3	1	1
21814	202	0	31-Jul-10	19	1	6	2	1	1	0	3	3	1	1
21815	202	0	6-Jul-10	17	1	6	2	1	1	0	3	3	1	1
21816	202	0	29-May-10	11	1	6	2	2	1	0	3	3	1	2
21817	202	0	2-Jul-09	19	1	6	2	1	1	0	3	3	4	1

21818	202	0	10-Mar-09	13	1	6	2	1	1	0	3	3	7	1
21819	202	0	7-May-09	17	1	6	2	1	1	0	3	3	1	1
21820	202	0	27-May-09	22	1	6	2	3	1	0	3	3	1	1
21821	202	0	10-Mar-09	12	1	6	2	1	1	0	3	3	6	1
21822	202	0	25-Aug-08	14	1	6	2	3	1	0	3	3	1	1
21823	202	0	29-Nov-08	17	1	6	2	1	1	0	3	3	6	1
21824	202	0	7-Sep-08	12	1	6	2	1	1	0	3	3	1	1
21825	202	0	18-Oct-08	21	1	6	2	2	1	0	3	3	2	1
21826	202	0	22-May-07	17	1	6	2	3	1	0	3	3	2	2
21827	202	0	25-Oct-07	17	1	6	2	1	1	0	3	3	7	1
21828	202	0	23-Dec-17	12	1	6	2	1	1	0	3	3	3	1
21829	202	0	18-Nov-06	17	1	6	2	2	1	0	6	3	3	2
21830	202	0	22-Sep-06	20	1	6	2	1	1	0	6	3	1	1
21831	202	0	3-Jun-15	20	2	6	2	1	1	0	1	3	5	1
21832	202	0	28-Aug-17	0	2	6	2	1	1	0	1	3	3	1
21833	202	0	7-Oct-16	18	2	6	2	3	1	0	1	3	1	1
21834	202	0	10-Mar-13	13	2	6	2	1	1	0	4	3	3	1
21835	202	0	31-May-15	20	2	6	2	1	1	0	1	3	3	1
21836	202	0	18-Aug-16	22	2	6	2	1	1	0	1	3	3	1
21837	202	0	12-Jul-14	16	2	6	2	1	1	0	1	3	1	1
21838	202	0	30-May-17	9	2	6	2	1	1	0	1	3	4	1
21839	202	0	15-Sep-15	11	2	6	2	3	1	0	1	3	5	1
21840	202	0	21-Oct-15	17	2	6	2	2	1	0	1	3	1	1
21841	202	0	9-May-16	5	2	6	2	1	1	0	1	3	2	1
21842	202	0	3-May-16	8	2	6	2	1	1	0	1	3	1	1
21843	202	0	12-May-16	14	2	6	2	1	1	0	1	3	4	1
21844	202	0	27-Oct-16	17	2	6	2	1	1	0	1	3	3	1
21845	202	0	14-Nov-15	15	2	6	2	3	1	0	1	3	1	1
21846	202	0	4-Oct-15	18	2	6	2	3	1	0	1	3	2	1
21847	202	0	26-Jun-14	18	2	6	2	1	1	0	1	3	3	1
21848	202	0	26-Oct-13	11	2	6	2	1	1	0	1	3	5	1
21849	202	0	21-Sep-17	7	2	6	2	2	1	0	1	3	3	2
21850	202	0	30-Jan-13	9	2	6	2	3	1	0	3	3	1	1
21851	202	0	24-Aug-15	14	2	6	2	2	1	0	4	3	1	1
21852	202	0	3-Apr-09	21	2	6	2	1	1	0	13	3	2	1
21853	202	0	16-May-12	17	2	6	2	1	1	0	1	3	3	1
21854	202	0	21-Aug-11	11	2	6	2	1	1	0	1	3	6	2
21855	202	0	9-Jul-11	14	2	6	2	1	1	0	1	3	2	1
21856	202	0	29-May-11	19	2	6	2	3	1	0	1	3	1	1
21857	202	0	12-Nov-11	11	2	6	2	1	1	0	1	3	3	1
21858	202	0	26-Apr-11	7	2	6	2	1	1	0	1	3	3	1
21859	202	0	11-Jul-11	17	2	6	2	3	1	0	1	3	3	1
21860	202	0	13-Apr-11	17	2	6	2	1	1	0	1	3	3	1
21861	202	0	21-Aug-10	20	2	6	2	1	1	0	1	3	6	1
21862	202	0	22-May-10	13	2	6	2	1	1	0	1	3	6	1
21863	202	0	14-Jul-10	16	2	6	2	1	1	0	1	3	1	1
21864	202	0	3-Feb-10	17	2	6	2	1	1	0	1	3	3	2
21865	202	0	29-Jul-09	20	2	6	2	2	1	0	1	3	1	1
21866	202	0	18-Sep-09	18	2	6	2	1	1	0	1	3	6	1
21867	202	0	7-Nov-09	20	2	6	2	1	1	0	1	3	1	2
21868	202	0	17-Jun-09	23	2	6	2	3	1	0	1	3	1	2
21869	202	0	5-Nov-09	11	2	6	2	1	1	0	1	3	1	1
21870	202	0	16-Jan-08	16	2	6	2	1	1	0	1	3	6	1
21871	202	0	27-May-08	17	2	6	2	1	1	0	1	3	1	1
21872	202	0	26-Jun-08	21	2	6	2	1	1	0	1	3	1	1
21873	202	0	27-Dec-08	21	2	6	2	3	1	0	1	3	1	1
21874	202	0	15-Nov-08	16	2	6	2	2	1	0	1	3	6	2
21875	202	0	26-Sep-08	12	2	6	2	1	1	0	1	3	6	1
21876	202	0	28-May-08	18	2	6	2	1	1	0	1	3	1	1
21877	202	0	27-Dec-08	20	2	6	2	2	1	0	1	3	1	2
21878	202	0	16-Feb-07	10	2	6	2	1	1	0	1	3	3	1
21879	202	0	6-Nov-07	11	2	6	2	3	1	0	1	3	1	1
21880	202	0	20-Feb-07	17	2	6	2	3	1	0	1	3	3	1
21881	202	0	21-May-07	18	2	6	2	1	1	0	1	3	1	1
21882	202	0	10-Nov-07	16	2	6	2	1	1	0	1	3	1	1
21883	202	0	29-Jan-07	20	2	6	2	1	1	0	1	3	1	1
21884	202	0	19-Sep-07	16	2	6	2	3	1	0	1	3	3	1
21885	202	0	17-Sep-06	16	2	6	2	1	1	0	1	3	6	1
21886	202	0	16-Nov-06	20	2	6	2	1	1	0	1	3	1	1
21887	202	0	22-May-10	12	2	6	2	1	1	0	1	3	6	1
21888	202	0	4-Apr-10	17	2	6	2	3	1	0	1	3	3	1
21889	202	0	4-Apr-09	13	2	6	2	1	1	0	1	3	3	1
21890	202	0	3-Apr-09	20	2	6	2	1	1	0	1	3	2	2
21891	202	0	10-Dec-07	17	2	6	2	1	1	0	4	3	3	1
21892	202	0	30-Jun-14	18	2	6	2	3	1	0	11	3	4	1
21893	202	0	31-Aug-16	13	2	6	2	1	1	0	3	3	1	1
21894	202	0	21-Aug-15	13	2	6	2	1	1	0	3	3	1	1
21895	202	0	31-Dec-15	11	2	6	2	1	1	0	3	3	1	1
21896	202	0	29-Jun-14	18	2	6	2	3	1	0	3	3	1	1
21897	202	0	23-Nov-11	19	2	6	2	3	1	0	3	3	5	2
21898	202	0	10-Sep-09	18	2	6	2	1	1	0	3	3	1	2
21899	202	0	18-Aug-09	19	2	6	2	1	1	0	3	3	1	1
21900	202	0	10-Sep-09	18	2	6	2	1	1	0	3	3	2	2
21901	202	0	17-Nov-08	9	2	6	2	1	1	0	3	3	1	2
21902	202	0	29-Nov-07	16	2	6	2	1	1	0	3	3	1	1
21903	202	0	25-Sep-17	10	2	6	2	1	1	0	3	3	3	1
21904	201	1	21-Sep-12	14	1	6	2	1	1	0	1	3	6	1
21905	201	1	2-Sep-12	18	1	6	2	1	1	0	1	3	3	1
21906	201	1	11-May-16	19	1	6	2	3	1	0	1	3	2	1
21907	201	1	13-Feb-12	19	1	6	2	3	1	0	1	3	6	1
21908	201	1	6-Mar-17	15	1	6	2	1	1	0	1	3	3	1
21909	201	1	11-Oct-14	11	1	6	2	2	1	0	1	3	1	1
21910	201	1	2-Dec-13	15	1	6	2	3	1	0	1	3	1	1
21911	201	1	22-Nov-14	8	1	6	2	1	1	0	1	3	2	1
21912	201	1	8-Jan-14	12	1	6	2	3	1	0	1	3	5	1
21913	101	1	24-Dec-12	12	1	6	2	1	1	0	1	3	5	1
21914	201	1	25-Nov-12	16	1	6	2	1	1	0	1	3	1	1
21915	101	1	1-Oct-12	11	1	6	2	1	1	0	1	3	5	1
21916	201	1	28-Nov-17	12	1	6	2	1	1	0	1	3	3	1
21917	201	1	3-Sep-17	8	1	6	2	1	1	0	1	3	1	1
21918	201	1	30-Sep-12	14	1	6	2	1	1	0	1	3	1	2
21919	101	1	24-Jan-17	19	1	6	2	1	1	0	4	3	2	1
21920	201	1	6-Sep-14	18	1	6	2	2	1	0	1	3	3	2
21921	201	1	27-Nov-12	12	1	6	2	2	1	0	1	3	5	2
21922	101	1	4-Jul-12	15	1	6	2	1	1	0	1	3	3	2
21923	101	1	27-Dec-16	17	1	6	2	1	1	0	1	3	5	1
21924	101	1	21-May-16	21	1	6	2	1	1	0	1	3	3	1
21925	101	1	14-Aug-16	13	1	6	2	1	1	0	1	3	3	4
21926	101	1	13-Sep-15	19	1	6	2	3	1	0	1	3	5	2
21927	101	1	18-Aug-17	12	1	6	2	1	1	0	6	3	3	1
21928	101	1	26-Jun-12	20	1	6	2	1	1	0	13	3	5	2
21929	101	1	19-May-11	7	1	6	2	2	1	0	13	3	3	2
21930	101	1	2-Mar-08	5	1	6	2	2	1	0	13	3	2	1
21931	101	1	19-Jul-11	15	1	6	2	1	1	0	1	3	3	1
21932	201	1	20-Dec-11	10	1	6	2	2	1	0	1	3	1	1
21933	201	1	30-Oct-11	21	1	6	2	1	1	0	1	3	4	1
21934	201	1	25-Sep-11	8	1	6	2	1	1	0	1	3	3	1
21935	101	1	25-Jun-11	10	1	6	2	1	1	0	1	3	5	1
21936	101	1	2-Dec-11	11	1	6	2	1	1	0	1	3	5	1
21937	101	1	2-Dec-11	11	1	6	2	1	1	0	1	3	5	1
21938	101	1	21-Aug-11	18	1	6	2	1	1	0	1	3	4	2
21939	101	1	28-Apr-11	19	1	6	2	1	1	0	1	3	5	2
21940	101	1	3-Nov-11	18	1	6	2	1	1	0	1	3	3	1
21941	101	1	7-Aug-10	22	1	6	2	1	1	0	1	3	5	2

21942	201	1	8-Jun-10	11	1	6	2	2	1	0	1	3	5	1
21943	101	1	12-Mar-10	18	1	6	2	1	1	0	1	3	5	1
21944	101	1	5-May-10	15	1	6	2	1	1	0	1	3	4	1
21945	201	1	2-Jun-10	19	1	6	2	3	1	0	1	3	6	1
21946	201	1	16-Apr-10	22	1	6	2	2	1	0	1	3	1	1
21947	201	1	24-Jun-10	20	1	6	2	2	1	0	1	3	2	1
21948	201	1	23-Mar-10	7	1	6	2	1	1	0	1	3	5	1
21949	201	1	3-May-10	18	1	6	2	2	1	0	1	3	1	1
21950	201	1	10-Aug-10	14	1	6	2	3	1	0	1	3	1	2
21951	101	1	19-Jan-10	21	1	6	2	1	1	0	1	3	1	2
21952	101	1	7-Jul-10	12	1	6	2	1	1	0	1	3	1	1
21953	201	1	2-Oct-10	11	1	6	2	2	1	0	1	3	1	2
21954	201	1	2-Dec-10	14	1	6	2	2	1	0	1	3	3	2
21955	101	1	27-Nov-10	20	1	6	2	2	1	0	1	3	7	1
21956	201	1	3-Jul-09	9	1	6	2	1	1	0	1	3	1	1
21957	201	1	4-Dec-09	9	1	6	2	1	1	0	1	3	1	1
21958	101	1	20-Nov-09	16	1	6	2	1	1	0	1	3	5	1
21959	201	1	2-Oct-09	23	1	6	2	1	1	0	1	3	4	1
21960	201	1	22-Mar-09	20	1	6	2	2	1	0	1	3	4	1
21961	101	1	28-Mar-09	17	1	6	2	1	1	0	1	3	3	4
21962	201	1	1-Dec-08	8	1	6	2	1	1	0	1	3	5	1
21963	101	1	4-Oct-08	17	1	6	2	1	1	0	1	3	5	2
21964	101	1	21-Dec-08	17	1	6	2	1	1	0	1	3	1	1
21965	101	1	1-Aug-08	22	1	6	2	1	1	0	1	3	1	1
21966	201	1	12-Jul-08	3	1	6	2	3	1	0	1	3	1	2
21967	101	1	6-Jan-08	12	1	6	2	1	1	0	1	3	3	1
21968	101	1	7-Jun-08	10	1	6	2	1	1	0	1	3	4	1
21969	201	1	10-Aug-08	19	1	6	2	3	1	0	1	3	5	1
21970	201	1	31-Jul-08	20	1	6	2	3	1	0	1	3	1	1
21971	201	1	15-Aug-08	15	1	6	2	3	1	0	1	3	5	2
21972	101	1	12-Jul-08	22	1	6	2	2	1	0	1	3	3	2
21973	101	1	23-Apr-08	9	1	6	2	1	1	0	1	3	3	1
21974	201	1	20-Jun-08	21	1	6	2	3	1	0	1	3	3	1
21975	101	1	23-Nov-08	17	1	6	2	1	1	0	1	3	3	1
21976	201	1	22-Apr-07	19	1	6	2	1	1	0	1	3	1	1
21977	201	1	16-May-07	19	1	6	2	1	1	0	1	3	6	1
21978	101	1	7-Sep-07	18	1	6	2	1	1	0	1	3	4	1
21979	101	1	8-Aug-07	9	1	6	2	1	1	0	1	3	6	2
21980	201	1	25-Jan-07	18	1	6	2	1	1	0	1	3	1	2
21981	101	1	20-Sep-07	17	1	6	2	1	1	0	1	3	1	2
21982	201	1	5-Jul-07	17	1	6	2	1	1	0	1	3	5	1
21983	101	1	27-Aug-07	19	1	6	2	1	1	0	1	3	1	2
21984	101	1	4-Apr-07	17	1	6	2	1	1	0	1	3	3	2
21985	101	1	21-Jan-07	10	1	6	2	1	1	0	1	3	3	1
21986	101	1	22-Dec-06	17	1	6	2	1	1	0	1	3	1	1
21987	101	1	17-Sep-06	16	1	6	2	1	1	0	1	3	5	1
21988	101	1	7-Aug-06	9	1	6	2	3	1	0	1	3	5	1
21989	201	1	23-Aug-06	20	1	6	2	2	1	0	1	3	6	1
21990	201	1	2-Sep-06	19	1	6	2	1	1	0	1	3	1	1
21991	101	1	13-Aug-17	15	1	6	2	1	1	0	1	3	1	1
21992	201	1	11-May-11	8	1	6	2	1	1	0	1	3	1	1
21993	201	1	19-May-11	21	1	6	2	3	1	0	1	3	2	1
21994	101	1	21-Aug-11	11	1	6	2	1	1	0	1	3	3	1
21995	101	1	28-Sep-11	19	1	6	2	1	1	0	1	3	1	1
21996	101	1	27-May-11	15	1	6	2	1	1	0	1	3	3	1
21997	201	1	20-May-10	14	1	6	2	2	1	0	1	3	1	1
21998	101	1	16-Apr-10	19	1	6	2	1	1	0	1	3	1	1
21999	201	1	15-Oct-09	15	1	6	2	1	1	0	1	3	1	1
22000	201	1	16-Jan-09	14	1	6	2	1	1	0	1	3	3	1
22001	101	1	24-Jul-09	17	1	6	2	1	1	0	1	3	2	1
22002	101	1	18-Apr-09	13	1	6	2	1	1	0	1	3	3	1
22003	201	1	2-Aug-09	19	1	6	2	2	1	0	1	3	1	1
22004	201	1	10-Nov-08	20	1	6	2	3	1	0	1	3	1	1
22005	101	1	20-Jul-07	20	1	6	2	2	1	0	1	3	5	1
22006	201	1	12-Oct-07	12	1	6	2	1	1	0	1	3	1	1
22007	201	1	29-Oct-07	23	1	6	2	1	1	0	1	3	1	2
22008	201	1	20-Nov-07	17	1	6	2	1	1	0	1	3	1	2
22009	201	1	22-Sep-07	12	1	6	2	1	1	0	1	3	1	1
22010	101	1	21-Oct-07	13	1	6	2	1	1	0	1	3	1	1
22011	201	1	21-Oct-07	22	1	6	2	3	1	0	1	3	3	1
22012	101	1	22-Jul-07	19	1	6	2	2	1	0	1	3	1	1
22013	201	1	5-Oct-07	12	1	6	2	2	1	0	1	3	3	1
22014	101	1	16-Jun-07	15	1	6	2	1	1	0	1	3	2	1
22015	101	1	16-Sep-06	17	1	6	2	1	1	0	1	3	1	1
22016	101	1	29-Nov-06	18	1	6	2	1	1	0	1	3	4	1
22017	101	1	10-Aug-06	16	1	6	2	1	1	0	1	3	3	1
22018	101	1	11-Jan-09	15	1	6	2	1	1	0	4	3	2	1
22019	101	1	12-Sep-09	22	1	6	2	1	1	0	4	3	2	1
22020	101	1	11-Jul-08	20	1	6	2	3	1	0	4	3	3	1
22021	201	1	14-Sep-08	15	1	6	2	1	1	0	4	3	3	1
22022	201	1	18-Oct-08	13	1	6	2	1	1	0	4	3	1	1
22023	201	1	14-Nov-08	20	1	6	2	2	1	0	4	3	3	2
22024	101	1	9-Sep-07	16	1	6	2	1	1	0	4	3	2	1
22025	201	1	17-Feb-16	19	1	6	2	1	1	0	11	3	3	1
22026	101	1	12-Dec-16	14	1	6	2	1	1	0	11	3	3	1
22027	201	1	16-Jun-13	17	1	6	2	2	1	0	11	3	2	1
22028	201	1	23-Aug-13	7	1	6	2	1	1	0	11	3	5	1
22029	201	1	27-May-12	9	1	6	2	3	1	0	11	3	3	1
22030	201	1	27-Dec-11	15	1	6	2	1	1	0	11	3	3	1
22031	101	1	29-Mar-10	12	1	6	2	3	1	0	11	3	3	1
22032	101	1	1-Feb-10	19	1	6	2	3	1	0	11	3	3	1
22033	101	1	27-Jun-09	15	1	6	2	1	1	0	11	3	2	1
22034	101	1	16-Aug-09	14	1	6	2	1	1	0	11	3	3	1
22035	101	1	9-Sep-08	18	1	6	2	1	1	0	11	3	3	1
22036	201	1	29-Sep-07	17	1	6	2	2	1	0	11	3	2	1
22037	201	1	6-Jul-07	8	1	6	2	2	1	0	11	3	2	1
22038	201	1	30-Sep-06	9	1	6	2	1	1	0	11	3	2	1
22039	201	1	9-Aug-17	15	1	6	2	2	1	0	11	3	2	1
22040	101	1	5-Aug-16	15	1	6	2	1	1	0	3	3	5	1
22041	201	1	30-Jul-16	11	1	6	2	1	1	0	3	3	1	1
22042	201	1	25-Jun-16	19	1	6	2	1	1	0	3	3	2	1
22043	201	1	15-Mar-16	16	1	6	2	1	1	0	3	3	3	1
22044	201	1	17-May-16	7	1	6	2	2	1	0	3	3	3	1
22045	201	1	15-May-16	21	1	6	2	1	1	0	3	3	3	1
22046	201	1	21-Mar-15	15	1	6	2	1	1	0	3	3	2	2
22047	201	1	11-Mar-15	14	1	6	2	1	1	0	3	3	1	1
22048	201	1	26-Sep-15	11	1	6	2	1	1	0	3	3	1	1
22049	201	1	29-Sep-15	18	1	6	2	2	1	0	3	3	3	1
22050	201	1	8-Dec-14	7	1	6	2	1	1	0	3	3	1	1
22051	201	1	21-Aug-14	10	1	6	2	1	1	0	3	3	1	1
22052	101	1	15-Oct-13	13	1	6	2	1	1	0	3	3	1	1
22053	201	1	18-Mar-13	13	1	6	2	1	1	0	3	3	1	1
22054	201	1	15-Apr-12	14	1	6	2	1	1	0	3	3	1	1
22055	101	1	27-Jun-12	14	1	6	2	1	1	0	3	3	2	1
22056	201	1	21-May-12	19	1	6	2	3	1	0	3	3	2	1
22057	101	1	27-Nov-11	0	1	6	2	1	1	0	3	3	1	1
22058	201	1	20-Aug-11	10	1	6	2	2	1	0	3	3	2	2
22059	101	1	26-Mar-11	11	1	6	2	1	1	0	3	3	1	1
22060	101	1	28-May-11	19	1	6	2	1	1	0	3	3	4	1
22061	101	1	23-Jul-11	13	1	6	2	1	1	0	3	3	3	1
22062	101	1	13-Sep-11	10	1	6	2	1	1	0	3	3	3	1
22063	201	1	13-May-11	0	1	6	2	1	1	0	3	3	2	1
22064	201	1	25-Jan-11	13	1	6	2	3	1	0	3	3	4	1
22065	201	1	22-May-10	18	1	6	2	1	1	0	3	3	1	1

22066	101	1	1-May-10	17	1	6	2	1	1	0	3	3	1	2
22067	201	1	23-Apr-10	20	1	6	2	1	1	0	3	3	1	1
22068	101	1	3-Sep-10	12	1	6	2	1	1	0	3	3	1	1
22069	201	1	8-Jul-10	21	1	6	2	3	1	0	3	3	1	1
22070	201	1	10-Jul-10	11	1	6	2	1	1	0	3	3	2	1
22071	101	1	10-Feb-09	17	1	6	2	1	1	0	3	3	1	2
22072	101	1	17-Nov-09	16	1	6	2	1	1	0	3	3	4	1
22073	201	1	24-Oct-09	7	1	6	2	1	1	0	3	3	1	1
22074	101	1	14-Jul-09	20	1	6	2	1	1	0	3	3	1	1
22075	101	1	17-Nov-09	10	1	6	2	2	1	0	3	3	1	1
22076	101	1	20-May-09	15	1	6	2	1	1	0	3	3	3	1
22077	101	1	17-May-09	10	1	6	2	1	1	0	3	3	2	1
22078	201	1	22-Dec-09	9	1	6	2	2	1	0	3	3	1	1
22079	201	1	25-Mar-08	18	1	6	2	3	1	0	3	3	3	1
22080	101	1	7-Jun-08	17	1	6	2	1	1	0	3	3	1	2
22081	101	1	22-Jun-08	20	1	6	2	3	1	0	3	3	1	1
22082	201	1	21-Aug-08	14	1	6	2	3	1	0	3	3	2	1
22083	201	1	16-Nov-08	15	1	6	2	2	1	0	3	3	3	1
22084	101	1	9-Sep-08	17	1	6	2	1	1	0	3	3	3	1
22085	201	1	21-Oct-08	20	1	6	2	1	1	0	3	3	2	1
22086	101	1	11-Oct-08	13	1	6	2	1	1	0	3	3	2	1
22087	201	1	22-Nov-08	18	1	6	2	3	1	0	3	3	1	1
22088	101	1	26-May-07	12	1	6	2	2	1	0	3	3	4	2
22089	101	1	7-Oct-07	11	1	6	2	1	1	0	3	3	1	1
22090	101	1	24-Aug-07	12	1	6	2	1	1	0	3	3	2	1
22091	201	1	11-May-07	20	1	6	2	1	1	0	3	3	1	1
22092	101	1	25-Jan-07	20	1	6	2	1	1	0	3	3	2	1
22093	101	1	19-Aug-07	21	1	6	2	1	1	0	3	3	2	1
22094	201	1	11-May-07	19	1	6	2	2	1	0	3	3	1	1
22095	101	1	14-Oct-06	17	1	6	2	1	1	0	3	3	5	1
22096	101	1	23-Dec-06	5	1	6	2	1	1	0	3	3	1	1
22097	101	1	17-Dec-06	15	1	6	2	1	1	0	3	3	1	1
22098	201	1	17-Dec-06	11	1	6	2	2	1	0	3	3	1	1
22099	101	1	6-Aug-06	21	1	6	2	1	1	0	3	3	3	1
22100	101	1	20-Sep-06	19	1	6	2	1	1	0	3	3	2	1
22101	101	1	14-Sep-17	12	1	6	2	1	1	0	3	3	3	1
22102	101	1	29-Mar-17	11	1	6	2	1	1	0	3	3	1	1
22103	101	1	28-Mar-17	18	1	6	2	1	1	0	3	3	1	1
22104	201	1	6-Sep-17	21	1	6	2	2	1	0	3	3	1	1
22105	101	1	7-Aug-17	19	1	6	2	4	1	0	3	3	2	1
22106	101	1	8-Oct-17	12	1	6	2	1	1	0	3	3	3	1
22107	101	1	10-Nov-16	20	1	6	2	1	1	0	6	3	3	1
22108	101	1	13-Aug-15	20	1	6	2	1	1	0	6	3	2	1
22109	101	1	10-Jul-12	20	1	6	2	1	1	0	6	3	3	1
22110	101	1	19-Jul-10	20	1	6	2	1	1	0	6	3	3	1
22111	201	1	22-Sep-07	15	1	6	2	3	1	0	6	3	2	1
22112	101	1	17-Nov-17	19	1	6	2	1	1	0	13	3	7	1
22113	201	1	11-Jun-12	17	2	6	2	1	1	0	1	3	1	1
22114	201	1	5-May-12	15	2	6	2	3	1	0	1	3	3	1
22115	201	1	28-May-15	19	2	6	2	2	1	0	1	3	2	2
22116	101	1	27-May-15	11	2	6	2	1	1	0	1	3	3	1
22117	201	1	16-Aug-15	9	2	6	2	2	1	0	1	3	3	1
22118	201	1	5-Oct-16	10	2	6	2	3	1	0	3	3	3	1
22119	201	1	8-Sep-13	10	2	6	2	3	1	0	1	3	5	1
22120	201	1	18-Nov-16	17	2	6	2	3	1	0	3	3	1	1
22121	201	1	2-May-12	17	2	6	2	3	1	0	1	3	5	1
22122	201	1	28-Dec-17	9	2	6	2	2	1	0	1	3	1	1
22123	201	1	20-Sep-14	22	2	6	2	2	1	0	1	3	2	1
22124	201	1	16-May-14	19	2	6	2	3	1	0	1	3	5	1
22125	201	1	20-Oct-15	7	2	6	2	1	1	0	1	3	4	1
22126	101	1	17-Jul-16	0	2	6	2	1	1	0	1	3	4	2
22127	201	1	23-Sep-14	11	2	6	2	2	1	0	1	3	1	1
22128	101	1	3-Sep-12	17	2	6	2	1	1	0	1	3	3	2
22129	201	1	30-Nov-17	17	2	6	2	2	1	0	1	3	3	1
22130	201	1	7-Nov-15	13	2	6	2	1	1	0	3	3	3	1
22131	201	1	4-Apr-15	13	2	6	2	1	1	0	4	3	2	1
22132	101	1	13-Aug-17	16	2	6	2	1	1	0	11	3	3	1
22133	101	1	30-Aug-09	14	2	6	2	1	1	0	13	3	2	4
22134	101	1	19-Feb-11	18	2	6	2	3	1	0	1	3	2	2
22135	101	1	6-Sep-11	18	2	6	2	1	1	0	1	3	5	1
22136	101	1	1-Oct-11	11	2	6	2	1	1	0	1	3	5	1
22137	201	1	25-Jan-11	16	2	6	2	2	1	0	1	3	6	2
22138	101	1	22-Aug-11	19	2	6	2	1	1	0	1	3	3	1
22139	201	1	4-Oct-11	5	2	6	2	1	1	0	1	3	1	1
22140	201	1	7-Dec-10	22	2	6	2	1	1	0	1	3	3	1
22141	101	1	23-Apr-10	17	2	6	2	3	1	0	1	3	5	1
22142	101	1	4-Apr-10	10	2	6	2	1	1	0	1	3	1	4
22143	201	1	1-Sep-10	12	2	6	2	1	1	0	1	3	6	1
22144	101	1	25-Sep-10	12	2	6	2	1	1	0	1	3	3	2
22145	201	1	9-Sep-10	12	2	6	2	3	1	0	1	3	1	1
22146	201	1	28-Mar-09	19	2	6	2	1	1	0	1	3	1	2
22147	201	1	8-Oct-09	15	2	6	2	1	1	0	1	3	1	1
22148	201	1	26-Apr-09	21	2	6	2	1	1	0	1	3	1	1
22149	101	1	21-Mar-09	16	2	6	2	1	1	0	1	3	3	1
22150	101	1	31-Aug-08	20	2	6	2	3	1	0	1	3	3	1
22151	201	1	4-Aug-08	10	2	6	2	3	1	0	1	3	5	1
22152	101	1	19-Jul-08	9	2	6	2	1	1	0	1	3	5	1
22153	101	1	25-May-08	16	2	6	2	1	1	0	1	3	3	2
22154	101	1	27-Dec-08	17	2	6	2	1	1	0	1	3	3	1
22155	101	1	8-Sep-08	18	2	6	2	1	1	0	1	3	5	1
22156	101	1	12-Jan-07	23	2	6	2	1	1	0	1	3	4	1
22157	101	1	18-Jan-07	20	2	6	2	1	1	0	1	3	3	2
22158	201	1	8-Jun-07	17	2	6	2	1	1	0	1	3	3	1
22159	101	1	6-Aug-07	17	2	6	2	1	1	0	1	3	4	1
22160	101	1	14-Jul-07	17	2	6	2	1	1	0	1	3	3	1
22161	201	1	4-Dec-06	17	2	6	2	1	1	0	1	3	5	1
22162	101	1	23-Sep-06	23	2	6	2	1	1	0	1	3	2	1
22163	101	1	25-Sep-06	19	2	6	2	1	1	0	1	3	3	1
22164	201	1	9-Feb-11	9	2	6	2	2	1	0	1	3	1	1
22165	201	1	27-Sep-11	13	2	6	2	2	1	0	1	3	1	1
22166	101	1	6-Sep-11	17	2	6	2	1	1	0	1	3	5	1
22167	201	1	23-Dec-11	14	2	6	2	1	1	0	1	3	4	1
22168	201	1	19-Jun-10	7	2	6	2	2	1	0	1	3	4	1
22169	101	1	13-Jan-10	17	2	6	2	3	1	0	1	3	3	2
22170	201	1	31-May-10	8	2	6	2	3	1	0	1	3	6	1
22171	101	1	27-Nov-09	17	2	6	2	1	1	0	1	3	4	1
22172	101	1	30-Sep-09	7	2	6	2	1	1	0	1	3	4	1
22173	201	1	28-May-09	12	2	6	2	1	1	0	1	3	3	1
22174	101	1	27-Mar-09	17	2	6	2	1	1	0	1	3	5	4
22175	101	1	17-Aug-09	13	2	6	2	1	1	0	1	3	1	1
22176	201	1	29-Sep-07	16	2	6	2	1	1	0	1	3	1	2
22177	201	1	31-Mar-07	17	2	6	2	1	1	0	1	3	3	1
22178	101	1	2-Apr-07	14	2	6	2	1	1	0	1	3	3	1
22179	101	1	6-Oct-06	22	2	6	2	1	1	0	1	3	1	1
22180	201	1	8-Feb-16	14	2	6	2	3	1	0	4	3	3	1
22181	101	1	15-May-12	14	2	6	2	1	1	0	4	3	3	1
22182	101	1	3-Dec-11	17	2	6	2	1	1	0	4	3	2	1
22183	101	1	10-Feb-09	18	2	6	2	1	1	0	4	3	3	1
22184	101	1	1-Jan-07	13	2	6	2	1	1	0	4	3	3	1
22185	101	1	12-Nov-06	18	2	6	2	1	1	0	4	3	2	1
22186	101	1	4-Sep-06	13	2	6	2	1	1	0	4	3	1	1
22187	101	1	7-Dec-16	17	2	6	2	1	1	0	11	3	2	1
22188	101	1	13-May-15	10	2	6	2	1	1	0	11	3	2	1
22189	201	1	8-Nov-13	12	2	6	2	3	1	0	11	3	3	1

22190	101	1	15-Dec-12	8	2	6	2	2	1	0	11	3	2	1
22191	201	1	15-Aug-11	21	2	6	2	1	1	0	11	3	3	1
22192	101	1	16-Jul-11	19	2	6	2	1	1	0	11	3	1	1
22193	101	1	1-May-10	18	2	6	2	1	1	0	11	3	3	1
22194	201	1	20-Jan-09	15	2	6	2	1	1	0	11	3	2	1
22195	201	1	19-Nov-17	10	2	6	2	2	1	0	11	3	2	1
22196	101	1	11-Jan-16	11	2	6	2	1	1	0	3	3	3	1
22197	101	1	15-Dec-16	20	2	6	2	1	1	0	3	3	1	1
22198	201	1	29-Aug-15	17	2	6	2	2	1	0	3	3	1	1
22199	201	1	18-Oct-15	20	2	6	2	3	1	0	3	3	5	1
22200	201	1	10-Jul-15	10	2	6	2	2	1	0	3	3	3	1
22201	201	1	23-Jan-15	21	2	6	2	3	1	0	3	3	3	1
22202	101	1	14-Nov-15	18	2	6	2	1	1	0	3	3	3	1
22203	201	1	14-Jan-14	13	2	6	2	2	1	0	3	3	4	1
22204	201	1	26-Sep-14	18	2	6	2	1	1	0	3	3	5	1
22205	201	1	6-Oct-14	17	2	6	2	1	1	0	3	3	2	1
22206	201	1	24-Nov-14	11	2	6	2	2	1	0	3	3	1	1
22207	201	1	12-Feb-14	12	2	6	2	1	1	0	3	3	3	1
22208	201	1	22-Jul-13	6	2	6	2	1	1	0	3	3	3	1
22209	201	1	10-Sep-13	17	2	6	2	1	1	0	3	3	3	1
22210	201	1	19-Dec-11	18	2	6	2	1	1	0	3	3	3	1
22211	101	1	7-Sep-11	7	2	6	2	1	1	0	3	3	3	1
22212	201	1	20-Jul-10	6	2	6	2	1	1	0	3	3	2	1
22213	201	1	21-Jun-10	21	2	6	2	1	1	0	3	3	5	1
22214	201	1	13-Jun-10	19	2	6	2	2	1	0	3	3	1	1
22215	201	1	13-Jul-10	22	2	6	2	3	1	0	3	3	4	1
22216	201	1	26-Jan-09	12	2	6	2	1	1	0	3	3	1	1
22217	201	1	31-Jan-09	11	2	6	2	1	1	0	3	3	3	1
22218	201	1	24-Nov-09	18	2	6	2	3	1	0	3	3	1	1
22219	101	1	17-Apr-09	17	2	6	2	1	1	0	3	3	3	1
22220	101	1	17-Jul-09	21	2	6	2	1	1	0	3	3	3	1
22221	101	1	27-Oct-09	14	2	6	2	1	1	0	3	3	2	4
22222	101	1	2-Dec-08	21	2	6	2	1	1	0	3	3	3	2
22223	201	1	23-Apr-08	17	2	6	2	1	1	0	3	3	1	1
22224	201	1	28-Oct-08	19	2	6	2	3	1	0	3	3	1	1
22225	101	1	16-Dec-08	20	2	6	2	1	1	0	3	3	1	2
22226	101	1	28-Nov-08	17	2	6	2	1	1	0	3	3	3	1
22227	101	1	5-Aug-07	9	2	6	2	1	1	0	3	3	1	1
22228	201	1	7-Nov-07	14	2	6	2	3	1	0	3	3	1	1
22229	101	1	24-May-07	22	2	6	2	1	1	0	3	3	1	1
22230	101	1	2-Sep-07	12	2	6	2	1	1	0	3	3	2	1
22231	201	1	13-Mar-07	8	2	6	2	1	1	0	3	3	3	1
22232	101	1	7-Nov-07	19	2	6	2	4	1	0	3	3	2	1
22233	201	1	17-Feb-07	20	2	6	2	1	1	0	3	3	1	1
22234	101	1	29-Jul-07	10	2	6	2	1	1	0	3	3	3	1
22235	101	1	6-Aug-06	21	2	6	2	1	1	0	3	3	1	1
22236	101	1	15-Oct-06	11	2	6	2	1	1	0	3	3	3	1
22237	201	1	22-Aug-17	11	2	6	2	1	1	0	3	3	3	1
22238	201	1	26-Apr-17	16	2	6	2	1	1	0	3	3	3	1
22239	201	1	19-Oct-17	21	2	6	2	2	1	0	3	3	2	1
22240	201	1	20-Jun-08	22	2	6	2	1	1	0	6	3	3	1
22241	101	1	9-Nov-17	8	2	6	2	1	1	0	13	3	1	1
22242	202	0	28-Nov-12	12	1	7	2	1	1	0	1	3	1	1
22243	202	0	16-Aug-15	15	1	7	2	1	1	0	3	3	1	2
22244	202	0	22-Mar-17	17	1	7	2	3	1	0	1	3	4	1
22245	202	0	3-Apr-16	1	1	7	2	3	1	0	1	3	3	1
22246	202	0	2-Jun-15	20	1	7	2	3	1	0	1	3	1	1
22247	202	0	12-Mar-13	16	1	7	2	1	1	0	1	3	2	2
22248	202	0	29-Sep-12	20	1	7	2	2	1	0	1	3	3	2
22249	202	0	18-Jul-16	8	1	7	2	1	1	0	1	3	5	2
22250	202	0	25-Oct-16	10	1	7	2	1	1	0	1	3	3	2
22251	202	0	7-Aug-16	23	1	7	2	2	1	0	1	3	3	1
22252	202	0	10-Mar-13	18	1	7	2	1	1	0	1	3	1	2
22253	202	0	7-Dec-17	17	1	7	2	3	1	0	1	3	1	1
22254	202	0	18-Nov-14	17	1	7	2	3	1	0	1	3	5	3
22255	202	0	22-Feb-16	16	1	7	2	2	1	0	1	3	3	1
22256	202	0	27-Jun-14	18	1	7	2	3	1	0	1	3	1	1
22257	202	0	19-May-17	18	1	7	2	1	1	0	1	3	4	1
22258	202	0	10-Sep-14	19	1	7	2	3	1	0	1	3	3	1
22259	202	0	25-Oct-12	21	1	7	2	3	1	0	6	3	3	1
22260	202	0	12-Dec-16	16	1	7	2	1	1	0	1	3	1	1
22261	202	0	24-Oct-15	20	1	7	2	1	1	0	1	3	1	1
22262	202	0	25-Aug-17	11	1	7	2	1	1	0	1	3	1	1
22263	202	0	3-Jul-16	21	1	7	2	3	1	0	1	3	4	1
22264	202	0	2-Jul-16	9	1	7	2	3	1	0	1	3	2	1
22265	202	0	1-Jan-16	10	1	7	2	1	1	0	1	3	4	2
22266	202	0	7-Oct-13	17	1	7	2	1	1	0	1	3	1	1
22267	202	0	31-Dec-16	15	1	7	2	1	1	0	1	3	3	1
22268	202	0	15-Jul-16	16	1	7	2	1	1	0	1	3	1	1
22269	202	0	18-Dec-16	13	1	7	2	3	1	0	1	3	1	1
22270	202	0	12-Jul-16	12	1	7	2	3	1	0	1	3	4	1
22271	202	0	20-Apr-16	14	1	7	2	1	1	0	1	3	3	1
22272	202	0	22-Aug-16	22	1	7	2	1	1	0	1	3	1	2
22273	202	0	10-Sep-16	8	1	7	2	1	1	0	1	3	3	1
22274	202	0	15-Oct-16	12	1	7	2	3	1	0	1	3	4	1
22275	202	0	14-Jul-16	8	1	7	2	1	1	0	1	3	6	1
22276	202	0	17-Nov-15	19	1	7	2	1	1	0	1	3	4	1
22277	202	0	20-Sep-15	17	1	7	2	1	1	0	1	3	5	1
22278	202	0	10-Sep-15	8	1	7	2	1	1	0	1	3	5	1
22279	202	0	22-Sep-15	17	1	7	2	2	1	0	1	3	1	1
22280	202	0	4-Nov-15	11	1	7	2	3	1	0	1	3	3	1
22281	202	0	28-Sep-14	16	1	7	2	3	1	0	1	3	3	1
22282	202	0	2-Mar-14	11	1	7	2	2	1	0	1	3	5	1
22283	202	0	25-Sep-14	17	1	7	2	3	1	0	1	3	1	1
22284	202	0	16-Aug-14	20	1	7	2	2	1	0	1	3	2	2
22285	202	0	27-Oct-13	2	1	7	2	2	1	0	1	3	1	1
22286	202	0	10-Apr-13	11	1	7	2	3	1	0	1	3	1	1
22287	202	0	17-Feb-13	0	1	7	2	2	1	0	1	3	3	1
22288	202	0	12-Dec-12	17	1	7	2	1	1	0	1	3	5	1
22289	202	0	27-May-12	9	1	7	2	1	1	0	1	3	3	2
22290	202	0	4-Apr-12	20	1	7	2	1	1	0	1	3	3	1
22291	202	0	11-Dec-12	19	1	7	2	2	1	0	1	3	3	1
22292	202	0	28-Apr-12	18	1	7	2	2	1	0	1	3	1	1
22293	202	0	6-Dec-12	17	1	7	2	1	1	0	1	3	3	2
22294	202	0	18-Jul-17	2	1	7	2	1	1	0	1	3	4	2
22295	202	0	3-Jun-17	19	1	7	2	1	1	0	1	3	1	1
22296	202	0	6-Nov-17	16	1	7	2	1	1	0	1	3	1	1
22297	202	0	22-Mar-17	7	1	7	2	1	1	0	1	3	6	2
22298	202	0	10-Nov-17	17	1	7	2	2	1	0	1	3	1	1
22299	202	0	7-Dec-17	18	1	7	2	3	1	0	1	3	3	2
22300	202	0	20-Dec-15	13	1	7	2	1	1	0	3	3	3	1
22301	202	0	12-Dec-12	17	1	7	2	1	1	0	6	3	5	1
22302	202	0	11-Sep-15	12	1	7	2	2	1	0	4	3	2	1
22303	202	0	3-Sep-16	11	1	7	2	1	1	0	6	3	3	1
22304	202	0	8-Apr-12	21	1	7	2	1	1	0	7	3	3	1
22305	202	0	11-Sep-09	16	1	7	2	1	1	0	7	3	3	2
22306	202	0	11-Apr-11	14	1	7	2	1	1	0	1	3	6	2
22307	202	0	19-Aug-11	17	1	7	2	3	1	0	1	3	1	2
22308	202	0	12-May-11	17	1	7	2	1	1	0	1	3	5	2
22309	202	0	23-Jan-11	14	1	7	2	1	1	0	1	3	6	2
22310	202	0	20-Jun-11	20	1	7	2	1	1	0	1	3	1	1
22311	202	0	29-Jan-11	15	1	7	2	2	1	0	1	3	1	2
22312	202	0	25-Apr-11	7	1	7	2	1	1	0	1	3	3	2
22313	202	0	1-Jan-11	14	1	7	2	1	1	0	1	3	5	1

22314	202	0	25-Jul-11	18	1	7	2	1	1	0	1	3	3	2
22315	202	0	29-Jan-11	19	1	7	2	2	1	0	1	3	1	2
22316	202	0	20-Jun-11	17	1	7	2	2	1	0	1	3	3	2
22317	202	0	4-Sep-10	20	1	7	2	1	1	0	1	3	6	1
22318	202	0	24-Dec-10	21	1	7	2	1	1	0	1	3	6	2
22319	202	0	22-Sep-10	16	1	7	2	3	1	0	1	3	5	1
22320	202	0	12-Nov-10	17	1	7	2	1	1	0	1	3	3	1
22321	202	0	18-Sep-10	18	1	7	2	1	1	0	1	3	3	1
22322	202	0	2-Mar-10	13	1	7	2	1	1	0	1	3	3	2
22323	202	0	20-Dec-10	15	1	7	2	1	1	0	1	3	2	1
22324	202	0	5-Jul-10	17	1	7	2	1	1	0	1	3	3	2
22325	202	0	21-Jan-10	21	1	7	2	1	1	0	1	3	3	2
22326	202	0	23-Dec-10	14	1	7	2	1	1	0	1	3	3	2
22327	202	0	16-May-10	16	1	7	2	1	1	0	1	3	3	2
22328	202	0	16-Apr-09	17	1	7	2	1	1	0	1	3	6	2
22329	202	0	1-Nov-09	19	1	7	2	1	1	0	1	3	6	1
22330	202	0	22-Mar-09	13	1	7	2	3	1	0	1	3	1	2
22331	202	0	1-Nov-09	18	1	7	2	1	1	0	1	3	3	1
22332	202	0	1-Apr-09	17	1	7	2	1	1	0	1	3	3	1
22333	202	0	19-Jul-09	20	1	7	2	3	1	0	1	3	3	2
22334	202	0	29-Jul-09	20	1	7	2	3	1	0	1	3	1	1
22335	202	0	16-Jun-09	17	1	7	2	1	1	0	1	3	3	1
22336	202	0	27-Jun-09	9	1	7	2	1	1	0	1	3	3	2
22337	202	0	25-Mar-09	14	1	7	2	3	1	0	1	3	3	2
22338	202	0	15-Aug-08	17	1	7	2	1	1	0	1	3	6	1
22339	202	0	9-May-08	8	1	7	2	1	1	0	1	3	1	1
22340	202	0	29-Dec-08	21	1	7	2	3	1	0	1	3	1	1
22341	202	0	28-Dec-08	22	1	7	2	3	1	0	1	3	1	1
22342	202	0	12-Nov-08	8	1	7	2	3	1	0	1	3	3	1
22343	202	0	26-Dec-08	17	1	7	2	1	1	0	1	3	3	1
22344	202	0	22-Jul-08	19	1	7	2	1	1	0	1	3	1	1
22345	202	0	11-Mar-08	13	1	7	2	3	1	0	1	3	1	1
22346	202	0	15-May-08	18	1	7	2	1	1	0	1	3	4	1
22347	202	0	9-Jan-08	17	1	7	2	1	1	0	1	3	1	1
22348	202	0	21-Feb-07	16	1	7	2	1	1	0	1	3	6	1
22349	202	0	15-Jan-07	17	1	7	2	3	1	0	1	3	1	1
22350	202	0	3-Nov-07	14	1	7	2	1	1	0	1	3	1	2
22351	202	0	19-Jul-07	12	1	7	2	1	1	0	1	3	2	1
22352	202	0	9-Jul-07	14	1	7	2	1	1	0	1	3	6	1
22353	202	0	6-Nov-07	11	1	7	2	1	1	0	1	3	1	1
22354	202	0	26-Feb-07	10	1	7	2	3	1	0	1	3	3	1
22355	202	0	30-Apr-07	14	1	7	2	1	1	0	1	3	5	1
22356	202	0	21-May-07	18	1	7	2	1	1	0	1	3	1	1
22357	202	0	20-Nov-07	18	1	7	2	1	1	0	1	3	4	1
22358	202	0	10-Mar-07	20	1	7	2	1	1	0	1	3	3	1
22359	202	0	2-May-07	14	1	7	2	1	1	0	1	3	2	1
22360	202	0	16-Oct-07	11	1	7	2	1	1	0	1	3	4	1
22361	202	0	22-Sep-07	19	1	7	2	1	1	0	1	3	3	2
22362	202	0	27-Nov-06	11	1	7	2	1	1	0	1	3	1	1
22363	202	0	9-Nov-06	14	1	7	2	1	1	0	1	3	3	1
22364	202	0	4-Oct-06	16	1	7	2	1	1	0	1	3	3	2
22365	202	0	28-Dec-06	14	1	7	2	1	1	0	1	3	4	1
22366	202	0	7-Oct-06	8	1	7	2	3	1	0	1	3	4	1
22367	202	0	19-Jan-12	14	1	7	2	1	1	0	1	3	1	1
22368	202	0	11-Dec-10	13	1	7	2	1	1	0	1	3	6	2
22369	202	0	1-Jul-16	16	1	7	2	3	1	0	4	3	1	1
22370	202	0	25-Nov-16	12	1	7	2	1	1	0	4	3	1	1
22371	202	0	22-Aug-15	11	1	7	2	3	1	0	4	3	3	1
22372	202	0	14-Jan-13	20	1	7	2	3	1	0	4	3	1	1
22373	202	0	26-Jan-12	20	1	7	2	1	1	0	4	3	3	2
22374	202	0	4-Jul-12	20	1	7	2	3	1	0	4	3	3	1
22375	202	0	17-Jan-10	14	1	7	2	2	1	0	4	3	3	2
22376	202	0	3-Jul-09	20	1	7	2	2	1	0	4	3	1	1
22377	202	0	24-May-08	20	1	7	2	2	1	0	4	3	1	1
22378	202	0	9-Jul-07	20	1	7	2	1	1	0	4	3	1	1
22379	202	0	2-Oct-17	23	1	7	2	3	1	0	4	3	1	1
22380	202	0	10-Mar-17	21	1	7	2	3	1	0	4	3	3	1
22381	202	0	25-Dec-17	16	1	7	2	3	1	0	4	3	1	1
22382	202	0	28-Aug-16	8	1	7	2	3	1	0	11	3	3	1
22383	202	0	3-Aug-15	21	1	7	2	3	1	0	11	3	3	1
22384	202	0	26-Aug-15	16	1	7	2	1	1	0	11	3	3	1
22385	202	0	15-Nov-14	20	1	7	2	3	1	0	11	3	3	1
22386	202	0	14-May-11	14	1	7	2	2	1	0	11	3	2	1
22387	202	0	10-Sep-07	11	1	7	2	3	1	0	11	3	4	1
22388	202	0	4-Feb-16	20	1	7	2	3	1	0	3	3	1	2
22389	202	0	30-Dec-16	15	1	7	2	2	1	0	3	3	1	1
22390	202	0	4-Jun-16	21	1	7	2	2	1	0	3	3	3	1
22391	202	0	27-Aug-16	9	1	7	2	1	1	0	3	3	3	1
22392	202	0	1-Jun-15	11	1	7	2	1	1	0	3	3	1	1
22393	202	0	24-Sep-15	16	1	7	2	1	1	0	3	3	1	1
22394	202	0	26-Jun-15	19	1	7	2	3	1	0	3	3	1	1
22395	202	0	16-May-15	17	1	7	2	1	1	0	3	3	2	1
22396	202	0	9-Aug-15	17	1	7	2	1	1	0	3	3	1	1
22397	202	0	12-Nov-14	11	1	7	2	3	1	0	3	3	1	1
22398	202	0	28-Dec-14	16	1	7	2	3	1	0	3	3	1	1
22399	202	0	19-Dec-14	13	1	7	2	1	1	0	3	3	1	1
22400	202	0	28-Jul-14	18	1	7	2	3	1	0	3	3	1	1
22401	202	0	3-Jul-14	17	1	7	2	3	1	0	3	3	3	1
22402	202	0	19-Dec-14	17	1	7	2	3	1	0	3	3	3	1
22403	202	0	14-Apr-13	11	1	7	2	3	1	0	3	3	4	1
22404	202	0	18-Nov-13	18	1	7	2	3	1	0	3	3	1	1
22405	202	0	16-Jan-13	17	1	7	2	1	1	0	3	3	1	1
22406	202	0	7-Oct-13	19	1	7	2	1	1	0	3	3	1	1
22407	202	0	23-Mar-13	19	1	7	2	1	1	0	3	3	4	1
22408	202	0	8-Jul-12	16	1	7	2	1	1	0	3	3	1	1
22409	202	0	26-Jun-12	21	1	7	2	1	1	0	3	3	1	1
22410	202	0	10-Sep-11	15	1	7	2	3	1	0	3	3	3	2
22411	202	0	29-Nov-11	16	1	7	2	1	1	0	3	3	2	1
22412	202	0	16-Aug-10	15	1	7	2	1	1	0	3	3	4	1
22413	202	0	15-Nov-10	15	1	7	2	1	1	0	3	3	1	1
22414	202	0	13-Nov-09	8	1	7	2	1	1	0	3	3	1	1
22415	202	0	20-Aug-09	8	1	7	2	1	1	0	3	3	3	1
22416	202	0	18-Mar-09	20	1	7	2	1	1	0	3	3	6	1
22417	202	0	25-Dec-09	15	1	7	2	1	1	0	3	3	2	2
22418	202	0	15-Aug-08	17	1	7	2	1	1	0	3	3	6	2
22419	202	0	10-Mar-08	12	1	7	2	1	1	0	3	3	1	1
22420	202	0	8-Feb-07	17	1	7	2	1	1	0	3	3	1	1
22421	202	0	14-Aug-07	21	1	7	2	1	1	0	3	3	7	1
22422	202	0	19-Jun-07	5	1	7	2	3	1	0	3	3	1	2
22423	202	0	13-Oct-07	15	1	7	2	1	1	0	3	3	1	1
22424	202	0	13-Mar-17	15	1	7	2	1	1	0	3	3	1	1
22425	202	0	28-Apr-17	14	1	7	2	1	1	0	3	3	1	1
22426	202	0	27-Apr-17	20	1	7	2	1	1	0	3	3	1	1
22427	202	0	6-Jun-17	18	1	7	2	2	1	0	3	3	3	1
22428	202	0	20-Apr-17	8	1	7	2	2	1	0	3	3	1	1
22429	202	0	13-Mar-17	17	1	7	2	3	1	0	3	3	1	1
22430	202	0	26-Feb-17	17	1	7	2	3	1	0	3	3	2	1
22431	202	0	7-Aug-11	17	1	7	2	1	1	0	6	3	3	1
22432	202	0	31-Oct-10	20	1	7	2	1	1	0	6	3	6	1
22433	202	0	12-Oct-08	14	1	7	2	3	1	0	6	3	2	1
22434	202	0	8-Nov-06	13	1	7	2	1	1	0	6	3	1	1
22435	202	0	21-Oct-17	17	2	7	2	1	1	0	1	3	1	1
22436	202	0	3-Jan-16	17	2	7	2	3	1	0	3	3	1	1
22437	202	0	20-Sep-15	15	2	7	2	1	1	0	1	3	3	1

22438	202	0	23-Jul-17	15	2	7	2	1	1	0	1	3	3	2
22439	202	0	19-Sep-15	19	2	7	2	1	1	0	1	3	1	1
22440	202	0	7-Sep-17	0	2	7	2	1	1	0	1	3	3	1
22441	202	0	6-Jan-16	11	2	7	2	2	1	0	1	3	1	1
22442	202	0	21-Dec-15	23	2	7	2	3	1	0	1	3	1	2
22443	202	0	5-Feb-13	18	2	7	2	2	1	0	1	3	3	1
22444	202	0	30-May-17	9	2	7	2	1	1	0	1	3	5	1
22445	202	0	13-Sep-13	17	2	7	2	1	1	0	1	3	1	2
22446	202	0	4-Dec-15	20	2	7	2	1	1	0	1	3	1	1
22447	202	0	13-Oct-15	19	2	7	2	1	1	0	1	3	3	2
22448	202	0	8-Jul-16	17	2	7	2	1	1	0	1	3	5	2
22449	202	0	21-Sep-14	18	2	7	2	3	1	0	1	3	2	1
22450	202	0	13-Feb-16	12	2	7	2	1	1	0	1	3	5	1
22451	202	0	12-Nov-16	14	2	7	2	1	1	0	1	3	1	1
22452	202	0	14-Mar-16	20	2	7	2	2	1	0	1	3	5	1
22453	202	0	7-Jun-15	15	2	7	2	3	1	0	1	3	1	2
22454	202	0	3-Jul-15	7	2	7	2	3	1	0	1	3	3	1
22455	202	0	26-Dec-14	20	2	7	2	3	1	0	1	3	3	1
22456	202	0	21-Jul-13	9	2	7	2	1	1	0	1	3	5	1
22457	202	0	7-Oct-13	11	2	7	2	1	1	0	1	3	3	1
22458	202	0	18-Sep-13	21	2	7	2	1	1	0	1	3	5	1
22459	202	0	20-Apr-13	17	2	7	2	1	1	0	1	3	1	1
22460	202	0	19-Oct-12	11	2	7	2	1	1	0	1	3	5	2
22461	202	0	5-Apr-17	14	2	7	2	1	1	0	1	3	3	1
22462	202	0	31-Jul-17	9	2	7	2	1	1	0	1	3	3	1
22463	202	0	21-Sep-17	7	2	7	2	2	1	0	1	3	3	2
22464	202	0	3-Jan-17	7	2	7	2	3	1	0	1	3	3	1
22465	202	0	23-Nov-17	18	2	7	2	3	1	0	1	3	3	1
22466	202	0	21-Jul-17	8	2	7	2	3	1	0	1	3	3	1
22467	202	0	6-Mar-17	18	2	7	2	3	1	0	1	3	3	1
22468	202	0	27-Jun-14	21	2	7	2	1	1	0	4	3	2	1
22469	202	0	26-Oct-13	17	2	7	2	3	1	0	7	3	3	2
22470	202	0	15-Nov-12	6	2	7	2	1	1	0	1	3	5	1
22471	202	0	7-Jul-11	21	2	7	2	1	1	0	1	3	6	2
22472	202	0	28-Feb-11	17	2	7	2	3	1	0	1	3	5	1
22473	202	0	19-Jan-11	18	2	7	2	1	1	0	1	3	2	1
22474	202	0	1-Jan-11	12	2	7	2	2	1	0	1	3	1	1
22475	202	0	12-Jun-11	16	2	7	2	1	1	0	1	3	1	1
22476	202	0	8-Apr-11	18	2	7	2	1	1	0	1	3	3	2
22477	202	0	10-Mar-10	1	2	7	2	1	1	0	1	3	1	1
22478	202	0	20-Jun-10	8	2	7	2	1	1	0	1	3	3	1
22479	202	0	30-Sep-10	22	2	7	2	1	1	0	1	3	3	2
22480	202	0	30-Jan-10	17	2	7	2	1	1	0	1	3	5	1
22481	202	0	2-Jun-10	7	2	7	2	1	1	0	1	3	1	1
22482	202	0	6-Apr-10	12	2	7	2	1	1	0	1	3	5	1
22483	202	0	24-Sep-10	12	2	7	2	1	1	0	1	3	3	1
22484	202	0	3-Oct-09	12	2	7	2	1	1	0	1	3	1	2
22485	202	0	24-Sep-09	13	2	7	2	1	1	0	1	3	3	2
22486	202	0	12-Mar-08	18	2	7	2	1	1	0	1	3	6	1
22487	202	0	28-May-08	12	2	7	2	1	1	0	1	3	1	1
22488	202	0	1-May-08	11	2	7	2	1	1	0	1	3	3	1
22489	202	0	4-Mar-08	18	2	7	2	1	1	0	1	3	4	1
22490	202	0	19-Mar-08	17	2	7	2	1	1	0	1	3	1	2
22491	202	0	7-Feb-08	10	2	7	2	1	1	0	1	3	1	1
22492	202	0	16-Jan-07	10	2	7	2	3	1	0	1	3	4	1
22493	202	0	26-Aug-07	22	2	7	2	1	1	0	1	3	1	1
22494	202	0	31-Aug-07	9	2	7	2	3	1	0	1	3	3	2
22495	202	0	20-Oct-06	14	2	7	2	3	1	0	1	3	6	1
22496	202	0	5-Dec-06	20	2	7	2	3	1	0	1	3	4	1
22497	202	0	5-Dec-10	15	2	7	2	1	1	0	1	3	3	2
22498	202	0	12-Nov-06	11	2	7	2	1	1	0	1	3	3	1
22499	202	0	12-Oct-16	20	2	7	2	1	1	0	4	3	2	1
22500	202	0	9-Jul-07	19	2	7	2	1	1	0	4	3	3	1
22501	202	0	9-Jul-15	19	2	7	2	2	1	0	11	3	2	1
22502	202	0	16-Jan-14	16	2	7	2	1	1	0	11	3	1	1
22503	202	0	2-Oct-13	14	2	7	2	1	1	0	11	3	3	1
22504	202	0	29-Jul-12	19	2	7	2	1	1	0	11	3	2	2
22505	202	0	16-Dec-06	12	2	7	2	1	1	0	11	3	3	1
22506	202	0	20-Jul-16	20	2	7	2	1	1	0	3	3	3	1
22507	202	0	13-Jun-15	12	2	7	2	1	1	0	3	3	1	1
22508	202	0	29-Sep-15	20	2	7	2	1	1	0	3	3	1	1
22509	202	0	3-Nov-14	11	2	7	2	1	1	0	3	3	5	1
22510	202	0	17-Dec-14	19	2	7	2	1	1	0	3	3	1	2
22511	202	0	10-Jun-14	18	2	7	2	1	1	0	3	3	3	1
22512	202	0	27-Jul-13	9	2	7	2	2	1	0	3	3	3	1
22513	202	0	28-Nov-10	15	2	7	2	1	1	0	3	3	5	1
22514	202	0	19-Jan-10	20	2	7	2	1	1	0	3	3	2	1
22515	202	0	21-Jul-10	20	2	7	2	1	1	0	3	3	3	1
22516	202	0	8-Apr-09	15	2	7	2	1	1	0	3	3	3	1
22517	202	0	16-Dec-09	8	2	7	2	1	1	0	3	3	7	1
22518	202	0	19-Feb-08	13	2	7	2	1	1	0	3	3	7	1
22519	202	0	18-Aug-08	18	2	7	2	1	1	0	3	3	2	2
22520	202	0	22-Oct-07	11	2	7	2	1	1	0	3	3	6	2
22521	202	0	26-Oct-06	10	2	7	2	1	1	0	3	3	3	1
22522	202	0	10-Jun-17	17	2	7	2	1	1	0	3	3	3	1
22523	101	1	5-Mar-13	14	1	7	2	3	1	0	1	3	1	1
22524	201	1	3-Aug-14	12	1	7	2	1	1	0	1	3	1	1
22525	201	1	28-May-16	16	1	7	2	3	1	0	1	3	5	1
22526	201	1	9-Sep-14	11	1	7	2	2	1	0	1	3	1	1
22527	201	1	23-May-13	22	1	7	2	3	1	0	1	3	5	1
22528	101	1	26-Feb-14	12	1	7	2	1	1	0	1	3	3	1
22529	101	1	20-Feb-12	20	1	7	2	1	1	0	1	3	4	1
22530	201	1	21-Mar-13	17	1	7	2	3	1	0	1	3	1	1
22531	201	1	11-Feb-14	8	1	7	2	1	1	0	1	3	2	1
22532	101	1	10-Jul-15	19	1	7	2	1	1	0	1	3	2	2
22533	201	1	19-Jul-15	22	1	7	2	2	1	0	1	3	3	1
22534	201	1	14-Feb-14	16	1	7	2	1	1	0	1	3	1	1
22535	201	1	16-Jun-14	16	1	7	2	1	1	0	1	3	4	1
22536	101	1	1-Oct-13	16	1	7	2	1	1	0	1	3	1	1
22537	101	1	27-Aug-13	17	1	7	2	1	1	0	1	3	2	2
22538	201	1	11-Feb-13	13	1	7	2	3	1	0	1	3	3	1
22539	201	1	10-Apr-12	16	1	7	2	3	1	0	1	3	3	1
22540	201	1	26-Feb-13	21	1	7	2	2	1	0	1	3	2	1
22541	201	1	29-Mar-13	14	1	7	2	3	1	0	1	3	3	1
22542	201	1	18-Nov-12	16	1	7	2	2	1	0	1	3	3	1
22543	201	1	12-Sep-17	16	1	7	2	2	1	0	1	3	3	1
22544	201	1	7-Aug-14	7	1	7	2	2	1	0	1	3	5	1
22545	101	1	22-Nov-13	20	1	7	2	1	1	0	1	3	1	1
22546	101	1	7-Jun-12	12	1	7	2	1	1	0	1	3	3	1
22547	101	1	10-Oct-14	21	1	7	2	1	1	0	1	3	1	2
22548	201	1	12-Jan-14	20	1	7	2	2	1	0	1	3	2	1
22549	201	1	2-Oct-13	0	1	7	2	2	1	0	1	3	5	1
22550	101	1	29-Sep-12	7	1	7	2	1	1	0	1	3	1	1
22551	101	1	12-Jul-15	11	1	7	2	1	1	0	1	3	5	1
22552	101	1	22-Dec-15	12	1	7	2	1	1	0	1	3	3	1
22553	201	1	4-Aug-14	10	1	7	2	1	1	0	1	3	5	2
22554	101	1	3-Jul-14	16	1	7	2	1	1	0	1	3	5	1
22555	101	1	16-Apr-13	21	1	7	2	1	1	0	1	3	1	1
22556	101	1	4-Jan-13	17	1	7	2	1	1	0	1	3	3	1
22557	101	1	31-Jan-13	18	1	7	2	1	1	0	1	3	3	1
22558	101	1	26-Jan-13	17	1	7	2	1	1	0	1	3	3	1
22559	201	1	10-May-12	17	1	7	2	3	1	0	1	3	6	2
22560	201	1	8-Sep-12	16	1	7	2	2	1	0	1	3	5	2
22561	201	1	4-Mar-12	17	1	7	2	3	1	0	1	3	1	1

22562	201	1	12-Aug-12	17	1	7	2	3	1	0	1	3	6	2
22563	201	1	10-Sep-12	18	1	7	2	2	1	0	1	3	1	2
22564	101	1	28-Jun-12	17	1	7	2	1	1	0	1	3	1	1
22565	201	1	9-Sep-15	7	1	7	2	3	1	0	1	3	3	1
22566	101	1	30-Aug-12	20	1	7	2	1	1	0	1	3	3	2
22567	201	1	14-Feb-16	16	1	7	2	1	1	0	1	3	5	2
22568	101	1	26-Jul-16	12	1	7	2	1	1	0	1	3	2	4
22569	101	1	6-Apr-15	10	1	7	2	1	1	0	1	3	5	2
22570	101	1	3-Apr-15	19	1	7	2	1	1	0	1	3	5	2
22571	101	1	27-Sep-15	9	1	7	2	1	1	0	1	3	5	1
22572	201	1	14-Oct-15	20	1	7	2	1	1	0	1	3	5	2
22573	201	1	22-Jul-14	21	1	7	2	2	1	0	1	3	4	1
22574	101	1	27-Mar-14	14	1	7	2	1	1	0	1	3	3	2
22575	101	1	7-May-13	20	1	7	2	1	1	0	1	3	4	2
22576	101	1	6-Oct-12	12	1	7	2	1	1	0	1	3	1	2
22577	101	1	9-Apr-16	17	1	7	2	1	1	0	1	3	1	1
22578	101	1	1-Feb-15	12	1	7	2	1	1	0	1	3	5	4
22579	101	1	16-Dec-15	12	1	7	2	1	1	0	1	3	3	1
22580	101	1	24-Feb-17	20	1	7	2	1	1	0	1	3	3	1
22581	101	1	30-Aug-13	20	1	7	2	2	1	0	1	3	4	1
22582	201	1	3-Feb-17	13	1	7	2	1	1	0	1	3	3	1
22583	201	1	8-Jul-13	11	1	7	2	2	1	0	6	3	3	1
22584	201	1	31-Dec-12	18	1	7	2	1	1	0	6	3	2	1
22585	201	1	23-Sep-17	11	1	7	2	3	1	0	6	3	3	1
22586	201	1	28-Oct-16	21	1	7	2	1	1	0	6	3	3	1
22587	101	1	29-May-17	20	1	7	2	1	1	0	11	3	3	1
22588	201	1	5-Aug-17	16	1	7	2	3	1	0	3	3	3	1
22589	201	1	19-Dec-08	20	1	7	2	3	1	0	7	3	2	1
22590	201	1	19-Dec-08	20	1	7	2	3	1	0	7	3	1	1
22591	101	1	11-Feb-07	10	1	7	2	1	1	0	13	3	2	1
22592	101	1	19-Jan-06	11	1	7	2	1	1	0	13	3	1	1
22593	101	1	1-Oct-14	19	1	7	2	2	1	0	1	3	5	1
22594	101	1	22-May-14	16	1	7	2	2	1	0	1	3	3	1
22595	101	1	8-Oct-13	8	1	7	2	1	1	0	1	3	5	1
22596	101	1	26-Mar-12	7	1	7	2	1	1	0	1	3	5	1
22597	101	1	17-Sep-12	17	1	7	2	1	1	0	1	3	3	1
22598	101	1	2-Oct-12	18	1	7	2	3	1	0	1	3	3	4
22599	201	1	30-Oct-11	22	1	7	2	1	1	0	1	3	4	1
22600	201	1	23-Mar-11	10	1	7	2	1	1	0	1	3	3	2
22601	201	1	23-Oct-11	20	1	7	2	1	1	0	1	3	3	2
22602	201	1	15-Oct-11	23	1	7	2	1	1	0	1	3	3	1
22603	201	1	27-Sep-11	12	1	7	2	3	1	0	1	3	5	1
22604	101	1	5-Mar-11	17	1	7	2	2	1	0	1	3	1	1
22605	101	1	22-Jul-11	16	1	7	2	1	1	0	1	3	5	1
22606	101	1	15-Nov-11	17	1	7	2	1	1	0	1	3	1	1
22607	101	1	11-Jun-11	18	1	7	2	2	1	0	1	3	5	2
22608	101	1	15-Nov-11	17	1	7	2	1	1	0	1	3	1	1
22609	101	1	26-Jun-11	14	1	7	2	3	1	0	1	3	1	2
22610	201	1	16-Sep-11	13	1	7	2	1	1	0	1	3	5	1
22611	201	1	12-Aug-11	8	1	7	2	1	1	0	1	3	6	2
22612	101	1	16-Nov-11	8	1	7	2	1	1	0	1	3	1	1
22613	101	1	8-Aug-11	11	1	7	2	3	1	0	1	3	1	2
22614	101	1	18-Sep-11	17	1	7	2	1	1	0	1	3	1	1
22615	101	1	25-Jun-11	20	1	7	2	1	1	0	1	3	3	1
22616	101	1	5-Jun-11	20	1	7	2	1	1	0	1	3	2	2
22617	201	1	7-Nov-11	10	1	7	2	1	1	0	1	3	1	2
22618	101	1	12-Oct-11	11	1	7	2	1	1	0	1	3	3	1
22619	101	1	25-Apr-11	20	1	7	2	1	1	0	1	3	3	2
22620	201	1	27-Aug-10	13	1	7	2	1	1	0	1	3	1	1
22621	201	1	7-Oct-10	18	1	7	2	1	1	0	1	3	4	1
22622	101	1	13-Feb-10	18	1	7	2	3	1	0	1	3	2	2
22623	101	1	16-Jul-10	17	1	7	2	1	1	0	1	3	4	1
22624	101	1	7-Dec-10	12	1	7	2	1	1	0	1	3	4	1
22625	101	1	19-May-10	18	1	7	2	1	1	0	1	3	2	1
22626	101	1	24-Aug-10	20	1	7	2	1	1	0	1	3	5	1
22627	201	1	11-May-10	20	1	7	2	1	1	0	1	3	1	2
22628	201	1	10-May-10	16	1	7	2	1	1	0	1	3	1	2
22629	201	1	3-May-10	17	1	7	2	1	1	0	1	3	1	1
22630	201	1	30-May-10	20	1	7	2	1	1	0	1	3	1	1
22631	101	1	14-Mar-10	15	1	7	2	3	1	0	1	3	5	2
22632	201	1	8-May-10	14	1	7	2	2	1	0	1	3	3	2
22633	101	1	15-Sep-10	11	1	7	2	1	1	0	1	3	5	1
22634	101	1	17-Oct-10	16	1	7	2	1	1	0	1	3	4	1
22635	201	1	24-Jul-10	16	1	7	2	2	1	0	1	3	3	1
22636	101	1	22-Dec-10	16	1	7	2	1	1	0	1	3	3	1
22637	101	1	21-Nov-10	18	1	7	2	1	1	0	1	3	4	1
22638	201	1	8-Mar-10	19	1	7	2	2	1	0	1	3	4	1
22639	201	1	30-Oct-09	9	1	7	2	1	1	0	1	3	1	1
22640	201	1	4-Sep-09	23	1	7	2	1	1	0	1	3	3	1
22641	201	1	22-Sep-09	11	1	7	2	1	1	0	1	3	3	1
22642	201	1	7-Oct-09	14	1	7	2	1	1	0	1	3	1	1
22643	101	1	4-Sep-09	17	1	7	2	1	1	0	1	3	5	1
22644	101	1	17-Apr-09	17	1	7	2	1	1	0	1	3	1	1
22645	201	1	13-Feb-09	16	1	7	2	3	1	0	1	3	1	2
22646	201	1	3-Aug-09	22	1	7	2	1	1	0	1	3	2	1
22647	201	1	13-Feb-09	17	1	7	2	1	1	0	1	3	1	2
22648	201	1	7-Nov-09	22	1	7	2	2	1	0	1	3	1	2
22649	101	1	25-Nov-09	10	1	7	2	1	1	0	1	3	1	1
22650	201	1	28-Oct-09	17	1	7	2	3	1	0	1	3	5	1
22651	201	1	19-Mar-09	15	1	7	2	1	1	0	1	3	3	2
22652	101	1	8-Feb-09	18	1	7	2	1	1	0	1	3	3	1
22653	101	1	4-Jul-09	13	1	7	2	1	1	0	1	3	3	2
22654	101	1	7-Jul-08	8	1	7	2	1	1	0	1	3	1	1
22655	201	1	11-Oct-08	17	1	7	2	1	1	0	1	3	1	2
22656	201	1	9-Nov-08	17	1	7	2	1	1	0	1	3	1	4
22657	101	1	5-Jan-08	13	1	7	2	2	1	0	1	3	4	1
22658	101	1	27-Dec-08	17	1	7	2	1	1	0	1	3	1	1
22659	201	1	26-Feb-08	19	1	7	2	2	1	0	1	3	6	2
22660	101	1	14-Oct-08	17	1	7	2	1	1	0	1	3	5	1
22661	101	1	16-Dec-08	21	1	7	2	1	1	0	1	3	1	2
22662	101	1	29-Mar-08	17	1	7	2	1	1	0	1	3	1	2
22663	101	1	11-Jan-08	18	1	7	2	1	1	0	1	3	1	2
22664	201	1	7-May-08	16	1	7	2	2	1	0	1	3	1	2
22665	101	1	21-Apr-08	18	1	7	2	1	1	0	1	3	3	1
22666	201	1	25-Oct-08	14	1	7	2	3	1	0	1	3	2	2
22667	101	1	18-Sep-07	20	1	7	2	2	1	0	1	3	1	1
22668	201	1	28-Apr-07	18	1	7	2	1	1	0	1	3	6	1
22669	201	1	3-Aug-07	21	1	7	2	1	1	0	1	3	1	4
22670	201	1	27-Jul-07	16	1	7	2	1	1	0	1	3	1	1
22671	201	1	17-Apr-07	14	1	7	2	1	1	0	1	3	1	1
22672	201	1	27-Mar-07	22	1	7	2	1	1	0	1	3	1	1
22673	201	1	1-Apr-07	20	1	7	2	1	1	0	1	3	2	1
22674	201	1	19-Oct-07	21	1	7	2	1	1	0	1	3	2	2
22675	201	1	10-Mar-07	21	1	7	2	1	1	0	1	3	2	1
22676	101	1	1-Sep-07	18	1	7	2	1	1	0	1	3	1	1
22677	201	1	28-Apr-07	8	1	7	2	2	1	0	1	3	6	2
22678	101	1	28-Sep-07	18	1	7	2	1	1	0	1	3	4	1
22679	201	1	16-Jul-07	15	1	7	2	2	1	0	1	3	1	1
22680	101	1	11-Nov-07	9	1	7	2	1	1	0	1	3	6	1
22681	101	1	26-Apr-07	14	1	7	2	1	1	0	1	3	1	2
22682	101	1	21-Sep-07	20	1	7	2	1	1	0	1	3	3	2
22683	201	1	13-Jul-07	19	1	7	2	1	1	0	1	3	3	1
22684	101	1	14-Feb-07	18	1	7	2	1	1	0	1	3	3	1
22685	201	1	6-Dec-07	13	1	7	2	1	1	0	1	3	4	1

22686	201	1	14-Jun-07	21	1	7	2	1	1	0	1	3	3	1
22687	201	1	25-Dec-06	20	1	7	2	1	1	0	1	3	4	2
22688	201	1	1-Jul-06	8	1	7	2	3	1	0	1	3	6	2
22689	101	1	25-Dec-06	8	1	7	2	1	1	0	1	3	1	2
22690	201	1	16-Jul-06	14	1	7	2	2	1	0	1	3	1	1
22691	201	1	25-Dec-06	18	1	7	2	1	1	0	1	3	5	2
22692	201	1	21-Dec-06	18	1	7	2	2	1	0	1	3	1	1
22693	101	1	24-Nov-06	11	1	7	2	1	1	0	1	3	3	1
22694	201	1	31-Dec-06	12	1	7	2	3	1	0	1	3	5	2
22695	101	1	5-Apr-17	16	1	7	2	1	1	0	1	3	6	1
22696	101	1	3-Nov-16	23	1	7	2	1	1	0	1	3	1	1
22697	101	1	20-Jun-15	21	1	7	2	1	1	0	1	3	1	1
22698	101	1	2-Aug-13	22	1	7	2	1	1	0	1	3	3	1
22699	201	1	6-Sep-11	23	1	7	2	2	1	0	1	3	6	1
22700	201	1	17-Oct-11	12	1	7	2	1	1	0	1	3	1	1
22701	201	1	2-Mar-11	7	1	7	2	1	1	0	1	3	1	1
22702	101	1	15-Aug-11	17	1	7	2	1	1	0	1	3	5	1
22703	101	1	17-Sep-10	15	1	7	2	1	1	0	1	3	7	1
22704	101	1	26-Apr-10	19	1	7	2	1	1	0	1	3	1	1
22705	201	1	4-Jun-10	17	1	7	2	2	1	0	1	3	1	1
22706	201	1	15-Sep-09	20	1	7	2	1	1	0	1	3	2	1
22707	101	1	13-Jun-09	18	1	7	2	1	1	0	1	3	3	1
22708	101	1	31-May-09	11	1	7	2	1	1	0	1	3	5	1
22709	101	1	9-May-09	10	1	7	2	1	1	0	1	3	5	1
22710	101	1	13-Dec-09	11	1	7	2	1	1	0	1	3	1	1
22711	101	1	22-Apr-08	17	1	7	2	1	1	0	1	3	5	1
22712	201	1	16-Nov-08	18	1	7	2	1	1	0	1	3	1	1
22713	101	1	4-Oct-07	20	1	7	2	1	1	0	1	3	3	1
22714	101	1	27-Feb-07	20	1	7	2	1	1	0	1	3	1	2
22715	201	1	28-Jul-06	20	1	7	2	2	1	0	1	3	1	2
22716	201	1	28-Jul-06	21	1	7	2	3	1	0	1	3	3	1
22717	101	1	31-Dec-12	21	1	7	2	1	1	0	4	3	2	1
22718	101	1	29-May-11	17	1	7	2	1	1	0	4	3	4	1
22719	101	1	30-Nov-11	21	1	7	2	1	1	0	4	3	2	1
22720	101	1	24-Jun-10	7	1	7	2	1	1	0	4	3	3	1
22721	101	1	3-May-10	17	1	7	2	1	1	0	4	3	1	1
22722	101	1	19-Apr-09	19	1	7	2	1	1	0	4	3	2	1
22723	101	1	14-Nov-08	15	1	7	2	1	1	0	4	3	1	1
22724	101	1	14-Jul-07	18	1	7	2	1	1	0	4	3	3	1
22725	101	1	24-Nov-17	9	1	7	2	1	1	0	4	3	4	1
22726	201	1	14-Nov-16	7	1	7	2	2	1	0	11	3	3	1
22727	101	1	30-Jun-15	17	1	7	2	1	1	0	11	3	2	1
22728	101	1	20-Mar-15	21	1	7	2	1	1	0	11	3	3	1
22729	201	1	23-Aug-15	13	1	7	2	1	1	0	11	3	2	1
22730	101	1	1-Nov-15	12	1	7	2	1	1	0	11	3	2	1
22731	201	1	22-Aug-15	19	1	7	2	2	1	0	11	3	2	1
22732	201	1	29-Aug-15	15	1	7	2	1	1	0	11	3	3	1
22733	201	1	12-Oct-14	14	1	7	2	1	1	0	11	3	3	1
22734	101	1	1-Jul-14	17	1	7	2	1	1	0	11	3	1	1
22735	101	1	23-May-14	21	1	7	2	1	1	0	11	3	2	1
22736	101	1	29-Nov-13	17	1	7	2	1	1	0	11	3	2	1
22737	101	1	4-Mar-13	17	1	7	2	1	1	0	11	3	2	1
22738	101	1	25-May-13	20	1	7	2	1	1	0	11	3	3	1
22739	201	1	7-Jan-12	9	1	7	2	3	1	0	11	3	2	1
22740	201	1	17-Jul-12	16	1	7	2	1	1	0	11	3	3	1
22741	201	1	27-May-12	20	1	7	2	1	1	0	11	3	2	1
22742	201	1	2-Jul-12	18	1	7	2	2	1	0	11	3	3	1
22743	201	1	17-Sep-11	22	1	7	2	1	1	0	11	3	2	1
22744	201	1	17-Sep-11	21	1	7	2	1	1	0	11	3	2	1
22745	101	1	8-Aug-11	22	1	7	2	1	1	0	11	3	3	1
22746	201	1	5-Jun-11	7	1	7	2	1	1	0	11	3	2	1
22747	201	1	23-Apr-11	7	1	7	2	1	1	0	11	3	3	1
22748	201	1	6-Jul-10	16	1	7	2	3	1	0	11	3	3	1
22749	101	1	30-May-10	18	1	7	2	3	1	0	11	3	3	1
22750	201	1	6-Nov-09	22	1	7	2	1	1	0	11	3	3	1
22751	101	1	8-Nov-09	12	1	7	2	2	1	0	11	3	3	1
22752	201	1	20-Jul-08	17	1	7	2	2	1	0	11	3	3	1
22753	201	1	12-Oct-17	11	1	7	2	1	1	0	11	3	3	1
22754	101	1	9-Apr-17	12	1	7	2	1	1	0	11	3	2	1
22755	101	1	17-Sep-17	12	1	7	2	1	1	0	11	3	2	1
22756	101	1	1-Jan-16	16	1	7	2	2	1	0	3	3	3	4
22757	101	1	7-Jan-16	16	1	7	2	1	1	0	3	3	3	1
22758	101	1	23-Jun-16	8	1	7	2	1	1	0	3	3	3	1
22759	201	1	7-Sep-16	10	1	7	2	1	1	0	3	3	3	1
22760	201	1	17-Jul-16	19	1	7	2	1	1	0	3	3	3	1
22761	201	1	26-Jul-16	8	1	7	2	1	1	0	3	3	3	1
22762	201	1	20-Aug-16	21	1	7	2	1	1	0	3	3	3	1
22763	101	1	11-Jul-15	14	1	7	2	1	1	0	3	3	3	2
22764	101	1	3-Sep-15	20	1	7	2	1	1	0	3	3	3	1
22765	101	1	17-Sep-15	18	1	7	2	1	1	0	3	3	1	1
22766	101	1	22-Nov-15	8	1	7	2	1	1	0	3	3	1	1
22767	101	1	17-Jan-15	19	1	7	2	1	1	0	3	3	1	1
22768	201	1	27-Apr-15	19	1	7	2	3	1	0	3	3	3	1
22769	101	1	11-Jul-15	11	1	7	2	1	1	0	3	3	1	1
22770	201	1	10-Oct-15	15	1	7	2	2	1	0	3	3	1	1
22771	201	1	18-Jun-15	7	1	7	2	1	1	0	3	3	1	1
22772	101	1	9-Jun-15	17	1	7	2	1	1	0	3	3	1	1
22773	101	1	31-Jan-15	11	1	7	2	1	1	0	3	3	1	1
22774	201	1	12-Feb-15	9	1	7	2	1	1	0	3	3	1	1
22775	201	1	27-Mar-15	17	1	7	2	2	1	0	3	3	3	1
22776	201	1	15-Jun-15	17	1	7	2	1	1	0	3	3	2	1
22777	101	1	18-Sep-15	17	1	7	2	1	1	0	3	3	3	1
22778	201	1	1-Jul-15	7	1	7	2	2	1	0	3	3	3	1
22779	101	1	5-Oct-15	21	1	7	2	1	1	0	3	3	4	1
22780	201	1	4-Jun-15	10	1	7	2	1	1	0	3	3	2	1
22781	101	1	8-Oct-14	21	1	7	2	1	1	0	3	3	5	1
22782	201	1	12-Oct-14	16	1	7	2	1	1	0	3	3	1	1
22783	201	1	4-Dec-14	6	1	7	2	1	1	0	3	3	4	1
22784	201	1	5-Jul-14	21	1	7	2	2	1	0	3	3	3	1
22785	201	1	18-Jul-14	11	1	7	2	1	1	0	3	3	1	1
22786	201	1	28-Jun-14	14	1	7	2	1	1	0	3	3	1	1
22787	201	1	14-Mar-14	7	1	7	2	2	1	0	3	3	2	1
22788	201	1	26-Sep-14	19	1	7	2	1	1	0	3	3	2	1
22789	201	1	15-Mar-14	13	1	7	2	1	1	0	3	3	1	2
22790	101	1	11-Jan-14	19	1	7	2	1	1	0	3	3	2	1
22791	101	1	25-May-14	14	1	7	2	3	1	0	3	3	3	4
22792	101	1	18-Nov-13	11	1	7	2	1	1	0	3	3	1	4
22793	101	1	12-Sep-13	17	1	7	2	1	1	0	3	3	1	1
22794	101	1	19-Aug-13	18	1	7	2	1	1	0	3	3	1	1
22795	201	1	13-Jan-13	16	1	7	2	2	1	0	3	3	1	1
22796	201	1	6-Apr-13	17	1	7	2	1	1	0	3	3	1	1
22797	101	1	30-Nov-13	12	1	7	2	1	1	0	3	3	1	2
22798	101	1	31-Oct-13	20	1	7	2	1	1	0	3	3	1	1
22799	101	1	11-Sep-12	16	1	7	2	1	1	0	3	3	1	1
22800	201	1	5-May-12	23	1	7	2	3	1	0	3	3	1	2
22801	201	1	9-Jun-12	13	1	7	2	3	1	0	3	3	2	1
22802	201	1	18-Nov-12	16	1	7	2	3	1	0	3	3	4	1
22803	101	1	26-Jan-11	9	1	7	2	1	1	0	3	3	1	1
22804	101	1	7-Jan-11	8	1	7	2	1	1	0	3	3	1	1
22805	101	1	2-Jul-11	19	1	7	2	2	1	0	3	3	1	2
22806	101	1	19-May-11	10	1	7	2	2	1	0	3	3	3	1
22807	201	1	8-Apr-11	11	1	7	2	1	1	0	3	3	2	1
22808	201	1	23-Apr-11	10	1	7	2	1	1	0	3	3	2	1
22809	101	1	23-Oct-10	11	1	7	2	1	1	0	3	3	5	1

22810	201	1	30-May-10	19	1	7	2	1	1	0	3	3	4	1
22811	201	1	29-Nov-10	17	1	7	2	1	1	0	3	3	1	1
22812	201	1	4-Feb-10	14	1	7	2	1	1	0	3	3	1	1
22813	201	1	1-Jun-10	20	1	7	2	2	1	0	3	3	1	1
22814	101	1	2-Apr-10	17	1	7	2	1	1	0	3	3	1	1
22815	201	1	1-Nov-10	8	1	7	2	1	1	0	3	3	1	1
22816	201	1	18-May-10	20	1	7	2	1	1	0	3	3	2	1
22817	101	1	7-Apr-10	14	1	7	2	1	1	0	3	3	4	2
22818	101	1	23-Mar-09	18	1	7	2	1	1	0	3	3	1	1
22819	201	1	20-Jan-09	13	1	7	2	1	1	0	3	3	1	1
22820	201	1	26-Jun-09	23	1	7	2	1	1	0	3	3	1	1
22821	101	1	24-Jul-09	17	1	7	2	1	1	0	3	3	1	1
22822	201	1	22-Oct-09	17	1	7	2	1	1	0	3	3	1	1
22823	101	1	30-Oct-09	21	1	7	2	1	1	0	3	3	2	1
22824	201	1	13-Sep-09	20	1	7	2	3	1	0	3	3	2	1
22825	101	1	28-Oct-09	10	1	7	2	1	1	0	3	3	2	1
22826	201	1	12-Jul-09	17	1	7	2	2	1	0	3	3	3	1
22827	101	1	25-Mar-09	17	1	7	2	1	1	0	3	3	2	1
22828	201	1	21-Nov-09	21	1	7	2	2	1	0	3	3	2	1
22829	101	1	11-Sep-09	21	1	7	2	1	1	0	3	3	2	1
22830	101	1	30-Mar-08	10	1	7	2	1	1	0	3	3	4	1
22831	201	1	23-May-08	8	1	7	2	1	1	0	3	3	4	1
22832	201	1	12-Sep-08	21	1	7	2	1	1	0	3	3	1	1
22833	201	1	9-Jul-08	19	1	7	2	3	1	0	3	3	1	1
22834	201	1	5-Nov-08	16	1	7	2	2	1	0	3	3	3	1
22835	101	1	22-Jun-08	20	1	7	2	1	1	0	3	3	2	1
22836	201	1	10-May-08	10	1	7	2	2	1	0	3	3	1	1
22837	101	1	14-Jul-07	21	1	7	2	1	1	0	3	3	1	1
22838	101	1	23-Jul-07	10	1	7	2	1	1	0	3	3	1	2
22839	101	1	9-Jun-07	7	1	7	2	1	1	0	3	3	1	1
22840	101	1	5-Dec-07	14	1	7	2	1	1	0	3	3	1	2
22841	201	1	8-Aug-07	17	1	7	2	1	1	0	3	3	5	2
22842	201	1	2-Aug-07	20	1	7	2	1	1	0	3	3	1	1
22843	201	1	10-Feb-07	17	1	7	2	1	1	0	3	3	1	1
22844	101	1	23-Aug-07	12	1	7	2	1	1	0	3	3	1	1
22845	101	1	8-Dec-07	7	1	7	2	1	1	0	3	3	3	1
22846	201	1	24-Aug-07	18	1	7	2	1	1	0	3	3	3	1
22847	101	1	6-Dec-07	11	1	7	2	1	1	0	3	3	3	1
22848	101	1	23-Sep-07	16	1	7	2	1	1	0	3	3	2	2
22849	101	1	6-Jul-07	18	1	7	2	1	1	0	3	3	3	1
22850	101	1	9-Feb-07	15	1	7	2	1	1	0	3	3	1	1
22851	101	1	30-Aug-06	12	1	7	2	1	1	0	3	3	7	1
22852	101	1	25-Aug-06	20	1	7	2	1	1	0	3	3	1	1
22853	201	1	29-Jun-06	22	1	7	2	1	1	0	3	3	1	1
22854	101	1	5-Sep-06	17	1	7	2	1	1	0	3	3	1	1
22855	101	1	19-Aug-06	18	1	7	2	1	1	0	3	3	1	1
22856	201	1	23-Oct-06	19	1	7	2	1	1	0	3	3	1	1
22857	201	1	5-Jul-06	16	1	7	2	2	1	0	3	3	1	1
22858	101	1	17-Oct-06	12	1	7	2	2	1	0	3	3	2	1
22859	201	1	15-Dec-06	18	1	7	2	3	1	0	3	3	2	1
22860	101	1	15-Nov-17	12	1	7	2	1	1	0	3	3	5	1
22861	201	1	7-Jun-17	14	1	7	2	1	1	0	3	3	4	1
22862	101	1	6-Jan-17	20	1	7	2	1	1	0	3	3	3	1
22863	201	1	4-Dec-17	12	1	7	2	1	1	0	3	3	3	1
22864	101	1	11-Apr-17	15	1	7	2	1	1	0	3	3	2	1
22865	101	1	5-Mar-17	15	1	7	2	1	1	0	3	3	1	1
22866	101	1	17-Jan-17	10	1	7	2	1	1	0	3	3	1	1
22867	201	1	9-Dec-17	20	1	7	2	1	1	0	3	3	1	1
22868	201	1	8-Aug-17	11	1	7	2	1	1	0	3	3	1	1
22869	101	1	9-Jul-17	13	1	7	2	1	1	0	3	3	1	2
22870	101	1	12-May-17	17	1	7	2	1	1	0	3	3	4	2
22871	201	1	8-Jun-17	13	1	7	2	2	1	0	3	3	5	1
22872	201	1	31-Jul-17	21	1	7	2	2	1	0	3	3	2	1
22873	201	1	25-Aug-17	14	1	7	2	3	1	0	3	3	3	1
22874	101	1	10-Dec-17	8	1	7	2	1	1	0	3	3	1	1
22875	101	1	7-Nov-17	12	1	7	2	1	1	0	3	3	1	1
22876	101	1	21-Nov-17	19	1	7	2	1	1	0	3	3	3	2
22877	201	1	25-Dec-17	13	1	7	2	2	1	0	3	3	1	1
22878	101	1	5-Dec-13	17	1	7	2	1	1	0	6	3	2	1
22879	101	1	15-Apr-11	22	1	7	2	1	1	0	6	3	3	1
22880	101	1	12-Jan-08	21	1	7	2	1	1	0	6	3	1	1
22881	201	1	21-Feb-07	17	1	7	2	1	1	0	6	3	3	1
22882	101	1	18-Dec-06	13	1	7	2	1	1	0	6	3	3	1
22883	201	1	14-Nov-08	12	1	7	2	1	1	0	13	3	7	1
22884	101	1	19-Nov-17	10	1	7	2	1	1	0	13	3	1	1
22885	101	1	12-Nov-17	9	1	7	2	1	1	0	13	3	4	1
22886	101	1	17-Nov-17	17	1	7	2	1	1	0	13	3	2	1
22887	101	1	31-Jul-12	16	2	7	2	1	1	0	1	3	5	1
22888	201	1	28-Feb-16	20	2	7	2	3	1	0	1	3	1	1
22889	201	1	6-Jan-14	4	2	7	2	3	1	0	1	3	5	2
22890	201	1	3-Aug-14	11	2	7	2	2	1	0	1	3	1	1
22891	201	1	13-Mar-15	14	2	7	2	2	1	0	1	3	5	1
22892	201	1	13-Jun-15	8	2	7	2	3	1	0	1	3	1	1
22893	201	1	6-Sep-15	0	2	7	2	3	1	0	1	3	3	1
22894	201	1	1-Feb-15	20	2	7	2	3	1	0	1	3	1	2
22895	201	1	30-Aug-14	8	2	7	2	3	1	0	1	3	3	1
22896	101	1	4-Nov-14	14	2	7	2	3	1	0	1	3	3	1
22897	201	1	23-Feb-13	11	2	7	2	2	1	0	1	3	2	1
22898	101	1	14-Mar-13	11	2	7	2	1	1	0	1	3	3	1
22899	201	1	12-May-12	18	2	7	2	1	1	0	1	3	2	1
22900	201	1	14-May-17	9	2	7	2	2	1	0	1	3	2	1
22901	201	1	11-Sep-17	16	2	7	2	2	1	0	1	3	5	1
22902	101	1	5-Feb-12	19	2	7	2	1	1	0	1	3	1	1
22903	201	1	3-Sep-12	7	2	7	2	2	1	0	1	3	1	1
22904	201	1	12-May-12	11	2	7	2	2	1	0	1	3	1	1
22905	201	1	15-Jul-14	10	2	7	2	1	1	0	1	3	1	1
22906	101	1	29-Mar-14	13	2	7	2	3	1	0	1	3	5	1
22907	101	1	3-Dec-12	13	2	7	2	1	1	0	1	3	7	2
22908	201	1	4-Jun-17	18	2	7	2	3	1	0	1	3	2	1
22909	101	1	26-Jun-15	16	2	7	2	1	1	0	1	3	1	1
22910	101	1	31-Oct-13	12	2	7	2	1	1	0	1	3	3	1
22911	101	1	4-Jan-17	7	2	7	2	1	1	0	1	3	3	1
22912	101	1	26-Sep-17	7	2	7	2	1	1	0	1	3	3	1
22913	201	1	17-Apr-15	16	2	7	2	2	1	0	1	3	2	1
22914	101	1	6-Feb-14	11	2	7	2	1	1	0	1	3	5	1
22915	101	1	10-Aug-14	21	2	7	2	2	1	0	1	3	1	1
22916	101	1	17-Mar-13	12	2	7	2	1	1	0	1	3	1	2
22917	101	1	26-Apr-14	15	2	7	2	1	1	0	1	3	3	1
22918	101	1	24-Apr-11	19	2	7	2	1	1	0	1	3	1	1
22919	201	1	15-Sep-11	13	2	7	2	1	1	0	1	3	3	1
22920	101	1	11-Nov-11	16	2	7	2	1	1	0	1	3	5	1
22921	101	1	17-Mar-11	20	2	7	2	1	1	0	1	3	4	1
22922	101	1	31-Dec-11	21	2	7	2	1	1	0	1	3	4	1
22923	101	1	21-Aug-11	17	2	7	2	3	1	0	1	3	3	1
22924	101	1	20-Oct-10	12	2	7	2	1	1	0	1	3	1	1
22925	201	1	18-Aug-10	11	2	7	2	1	1	0	1	3	1	1
22926	201	1	18-Dec-10	17	2	7	2	3	1	0	1	3	4	1
22927	101	1	28-Nov-10	9	2	7	2	1	1	0	1	3	3	1
22928	201	1	7-Dec-10	16	2	7	2	1	1	0	1	3	5	1
22929	201	1	16-Sep-09	13	2	7	2	1	1	0	1	3	3	1
22930	101	1	24-Jun-09	9	2	7	2	1	1	0	1	3	3	1
22931	101	1	2-Mar-09	8	2	7	2	1	1	0	1	3	3	1
22932	101	1	25-Sep-09	7	2	7	2	1	1	0	1	3	3	1
22933	101	1	16-Aug-09	1	2	7	2	1	1	0	1	3	3	1

22934	101	1	15-Mar-08	8	2	7	2	2	1	0	1	3	4	2
22935	201	1	26-Oct-08	16	2	7	2	2	1	0	1	3	1	2
22936	201	1	7-Jul-08	20	2	7	2	3	1	0	1	3	1	1
22937	101	1	3-Nov-08	16	2	7	2	1	1	0	1	3	3	1
22938	101	1	2-Dec-08	18	2	7	2	2	1	0	1	3	4	2
22939	201	1	3-Jul-08	20	2	7	2	1	1	0	1	3	1	2
22940	201	1	18-Jun-07	19	2	7	2	1	1	0	1	3	6	2
22941	201	1	22-Feb-07	16	2	7	2	1	1	0	1	3	1	1
22942	101	1	27-Sep-07	17	2	7	2	1	1	0	1	3	3	1
22943	101	1	25-May-07	16	2	7	2	1	1	0	1	3	3	2
22944	101	1	11-Oct-07	12	2	7	2	1	1	0	1	3	3	1
22945	101	1	18-Mar-07	16	2	7	2	1	1	0	1	3	1	1
22946	101	1	17-Oct-07	16	2	7	2	1	1	0	1	3	2	1
22947	201	1	9-Jan-07	19	2	7	2	1	1	0	1	3	2	1
22948	101	1	9-Jul-07	5	2	7	2	1	1	0	1	3	2	1
22949	101	1	20-Aug-06	17	2	7	2	3	1	0	1	3	1	1
22950	201	1	15-Oct-06	11	2	7	2	3	1	0	1	3	3	1
22951	101	1	11-Feb-12	15	2	7	2	1	1	0	1	3	3	4
22952	101	1	2-May-12	18	2	7	2	1	1	0	1	3	3	1
22953	101	1	5-Sep-11	11	2	7	2	1	1	0	1	3	5	1
22954	101	1	28-Mar-10	11	2	7	2	1	1	0	1	3	3	1
22955	101	1	25-Aug-10	18	2	7	2	3	1	0	1	3	3	1
22956	201	1	28-Jun-10	20	2	7	2	2	1	0	1	3	3	1
22957	201	1	22-Jul-10	15	2	7	2	1	1	0	1	3	1	1
22958	101	1	26-Dec-10	16	2	7	2	1	1	0	1	3	1	1
22959	201	1	4-May-10	19	2	7	2	3	1	0	1	3	3	1
22960	101	1	29-Jul-09	21	2	7	2	1	1	0	1	3	3	1
22961	101	1	22-Jun-09	18	2	7	2	1	1	0	1	3	1	2
22962	101	1	12-Nov-09	11	2	7	2	1	1	0	1	3	1	1
22963	101	1	16-Apr-08	16	2	7	2	1	1	0	1	3	3	1
22964	101	1	22-Jan-08	14	2	7	2	1	1	0	1	3	3	1
22965	101	1	3-Jan-08	12	2	7	2	1	1	0	1	3	3	1
22966	201	1	8-Sep-07	16	2	7	2	1	1	0	1	3	1	1
22967	101	1	30-Nov-07	15	2	7	2	1	1	0	1	3	3	1
22968	101	1	1-Oct-15	18	2	7	2	1	1	0	4	3	2	1
22969	101	1	26-Apr-07	20	2	7	2	1	1	0	4	3	2	1
22970	101	1	1-Oct-07	13	2	7	2	1	1	0	4	3	4	1
22971	101	1	27-Jul-07	21	2	7	2	1	1	0	4	3	2	1
22972	201	1	11-Jul-16	16	2	7	2	2	1	0	11	3	3	1
22973	101	1	3-Oct-16	17	2	7	2	1	1	0	11	3	2	1
22974	101	1	6-Oct-15	17	2	7	2	1	1	0	11	3	4	1
22975	101	1	17-Feb-15	22	2	7	2	1	1	0	11	3	1	1
22976	201	1	29-May-15	9	2	7	2	1	1	0	11	3	2	1
22977	101	1	31-Jan-15	9	2	7	2	2	1	0	11	3	3	1
22978	101	1	27-Jun-12	13	2	7	2	1	1	0	11	3	2	1
22979	201	1	23-Sep-11	0	2	7	2	1	1	0	11	3	3	1
22980	101	1	23-Apr-11	21	2	7	2	1	1	0	11	3	2	1
22981	201	1	26-Apr-11	13	2	7	2	3	1	0	11	3	2	1
22982	101	1	3-Feb-10	17	2	7	2	2	1	0	11	3	4	1
22983	101	1	21-Apr-10	17	2	7	2	1	1	0	11	3	3	1
22984	101	1	5-Feb-10	19	2	7	2	1	1	0	11	3	3	1
22985	201	1	28-Jan-09	10	2	7	2	1	1	0	11	3	4	1
22986	201	1	25-Dec-09	13	2	7	2	2	1	0	11	3	2	1
22987	201	1	24-Mar-09	15	2	7	2	3	1	0	11	3	2	1
22988	201	1	5-Jun-09	18	2	7	2	2	1	0	11	3	2	1
22989	201	1	1-Apr-09	17	2	7	2	2	1	0	11	3	3	1
22990	101	1	12-Jun-08	17	2	7	2	1	1	0	11	3	1	1
22991	201	1	2-May-08	9	2	7	2	1	1	0	11	3	3	1
22992	101	1	6-Jan-16	11	2	7	2	1	1	0	3	3	1	1
22993	101	1	1-Nov-16	1	2	7	2	4	1	0	3	3	2	1
22994	101	1	8-Nov-16	3	2	7	2	1	1	0	3	3	3	1
22995	201	1	14-May-16	20	2	7	2	2	1	0	3	3	1	1
22996	101	1	23-Sep-15	17	2	7	2	1	1	0	3	3	4	1
22997	201	1	3-Aug-15	12	2	7	2	1	1	0	3	3	1	1
22998	201	1	29-Nov-15	15	2	7	2	1	1	0	3	3	5	1
22999	101	1	29-Jun-15	19	2	7	2	1	1	0	3	3	2	1
23000	101	1	28-Apr-14	12	2	7	2	1	1	0	3	3	3	1
23001	201	1	30-Jun-14	13	2	7	2	1	1	0	3	3	3	1
23002	101	1	22-Jun-13	19	2	7	2	3	1	0	3	3	7	1
23003	101	1	16-Feb-13	17	2	7	2	1	1	0	3	3	1	4
23004	101	1	5-Aug-13	21	2	7	2	3	1	0	3	3	1	4
23005	101	1	22-Nov-13	12	2	7	2	1	1	0	3	3	1	1
23006	101	1	9-Jun-13	14	2	7	2	1	1	0	3	3	2	1
23007	201	1	12-Nov-13	19	2	7	2	3	1	0	3	3	2	1
23008	201	1	26-Dec-12	16	2	7	2	1	1	0	3	3	1	1
23009	101	1	7-May-12	18	2	7	2	1	1	0	3	3	3	1
23010	101	1	21-Mar-12	11	2	7	2	1	1	0	3	3	3	1
23011	101	1	23-Jul-11	9	2	7	2	1	1	0	3	3	5	1
23012	201	1	27-Mar-11	13	2	7	2	2	1	0	3	3	1	1
23013	101	1	9-Dec-10	20	2	7	2	1	1	0	3	3	1	1
23014	201	1	11-Oct-10	12	2	7	2	1	1	0	3	3	1	1
23015	101	1	9-Oct-10	22	2	7	2	1	1	0	3	3	1	1
23016	201	1	16-Dec-09	13	2	7	2	2	1	0	3	3	1	1
23017	101	1	3-May-09	17	2	7	2	1	1	0	3	3	1	1
23018	101	1	19-Oct-09	12	2	7	2	1	1	0	3	3	5	1
23019	201	1	3-Sep-09	8	2	7	2	2	1	0	3	3	1	1
23020	101	1	15-Jul-09	12	2	7	2	1	1	0	3	3	3	1
23021	101	1	2-Jun-08	7	2	7	2	1	1	0	3	3	1	2
23022	101	1	5-Apr-08	19	2	7	2	1	1	0	3	3	1	2
23023	101	1	20-Dec-08	17	2	7	2	1	1	0	3	3	2	1
23024	201	1	21-Oct-08	17	2	7	2	1	1	0	3	3	3	1
23025	101	1	14-Jul-07	18	2	7	2	3	1	0	3	3	3	1
23026	101	1	24-Jul-07	8	2	7	2	1	1	0	3	3	1	1
23027	101	1	18-Aug-07	9	2	7	2	1	1	0	3	3	1	1
23028	101	1	6-Dec-07	13	2	7	2	1	1	0	3	3	1	1
23029	101	1	2-Nov-07	21	2	7	2	1	1	0	3	3	1	1
23030	101	1	27-Aug-06	17	2	7	2	1	1	0	3	3	2	1
23031	201	1	7-Aug-06	14	2	7	2	1	1	0	3	3	3	1
23032	201	1	6-Oct-06	7	2	7	2	1	1	0	3	3	1	2
23033	201	1	14-Aug-17	19	2	7	2	1	1	0	3	3	3	1
23034	101	1	7-May-17	17	2	7	2	1	1	0	3	3	1	1
23035	101	1	14-Sep-12	20	2	7	2	1	1	0	6	3	3	1
23036	202	0	7-Aug-12	8	1	8	2	3	1	0	1	3	5	1
23037	202	0	18-May-13	13	1	8	2	3	1	0	1	3	3	1
23038	202	0	2-Feb-14	17	1	8	2	1	1	0	1	3	1	2
23039	202	0	28-May-13	17	1	8	2	3	1	0	1	3	3	1
23040	202	0	24-Mar-12	14	1	8	2	1	1	0	1	3	6	1
23041	202	0	7-Oct-15	15	1	8	2	1	1	0	1	3	4	1
23042	202	0	2-Nov-17	18	1	8	2	1	1	0	1	3	2	1
23043	202	0	20-Apr-14	18	1	8	2	3	1	0	3	3	1	1
23044	202	0	10-Sep-17	18	1	8	2	3	1	0	3	3	3	1
23045	202	0	29-Jul-15	22	1	8	2	1	1	0	1	3	3	1
23046	202	0	8-May-16	16	1	8	2	3	1	0	1	3	1	1
23047	202	0	26-Apr-17	17	1	8	2	1	1	0	1	3	4	2
23048	202	0	2-Apr-16	10	1	8	2	1	1	0	1	3	1	2
23049	202	0	9-Oct-15	13	1	8	2	1	1	0	1	3	1	1
23050	202	0	11-May-15	17	1	8	2	3	1	0	1	3	1	1
23051	202	0	13-May-12	22	1	8	2	1	1	0	1	3	5	1
23052	202	0	27-May-16	17	1	8	2	1	1	0	1	3	1	1
23053	202	0	25-May-16	19	1	8	2	1	1	0	1	3	3	1
23054	202	0	13-Aug-14	9	1	8	2	1	1	0	1	3	4	1
23055	202	0	17-Jun-17	17	1	8	2	1	1	0	1	3	5	1
23056	202	0	13-Aug-17	12	1	8	2	1	1	0	1	3	5	1
23057	202	0	5-Jun-17	13	1	8	2	1	1	0	1	3	2	1

23058	202	0	18-Mar-17	13	1	8	2	3	1	0	1	3	3	2
23059	202	0	20-Dec-15	15	1	8	2	2	1	0	1	3	4	1
23060	202	0	1-May-16	12	1	8	2	1	1	0	1	3	3	1
23061	202	0	9-Sep-15	20	1	8	2	1	1	0	1	3	5	1
23062	202	0	8-May-16	13	1	8	2	1	1	0	1	3	1	1
23063	202	0	19-Nov-15	7	1	8	2	1	1	0	1	3	1	1
23064	202	0	18-Oct-15	14	1	8	2	1	1	0	1	3	4	1
23065	202	0	16-Nov-13	11	1	8	2	1	1	0	7	3	2	1
23066	202	0	19-Jun-15	21	1	8	2	1	1	0	1	3	3	1
23067	202	0	26-Feb-16	14	1	8	2	1	1	0	1	3	1	1
23068	202	0	9-Feb-16	12	1	8	2	1	1	0	1	3	3	1
23069	202	0	25-Nov-14	17	1	8	2	3	1	0	1	3	3	1
23070	202	0	29-Jul-16	20	1	8	2	1	1	0	1	3	1	1
23071	202	0	10-Sep-12	12	1	8	2	1	1	0	1	3	3	1
23072	202	0	1-Jan-16	16	1	8	2	3	1	0	1	3	5	1
23073	202	0	3-Apr-16	12	1	8	2	3	1	0	1	3	1	1
23074	202	0	16-May-16	7	1	8	2	2	1	0	1	3	3	1
23075	202	0	19-Apr-16	14	1	8	2	1	1	0	1	3	1	1
23076	202	0	22-Aug-16	19	1	8	2	2	1	0	1	3	1	1
23077	202	0	4-Aug-16	17	1	8	2	3	1	0	1	3	1	1
23078	202	0	30-Jan-16	12	1	8	2	3	1	0	1	3	1	1
23079	202	0	18-May-16	17	1	8	2	1	1	0	1	3	3	1
23080	202	0	21-Sep-15	13	1	8	2	2	1	0	1	3	3	1
23081	202	0	24-Apr-14	18	1	8	2	3	1	0	1	3	4	1
23082	202	0	7-Mar-14	18	1	8	2	3	1	0	1	3	1	1
23083	202	0	11-Aug-14	11	1	8	2	3	1	0	1	3	5	2
23084	202	0	30-Jan-14	21	1	8	2	1	1	0	1	3	1	2
23085	202	0	10-Aug-14	1	1	8	2	1	1	0	1	3	5	1
23086	202	0	5-Dec-14	21	1	8	2	1	1	0	1	3	1	1
23087	202	0	5-Mar-14	11	1	8	2	1	1	0	1	3	4	1
23088	202	0	19-Dec-13	8	1	8	2	3	1	0	1	3	1	1
23089	202	0	19-Nov-13	7	1	8	2	1	1	0	1	3	3	2
23090	202	0	7-Dec-13	17	1	8	2	1	1	0	1	3	1	2
23091	202	0	3-Nov-13	18	1	8	2	1	1	0	1	3	1	1
23092	202	0	18-Oct-13	7	1	8	2	2	1	0	1	3	5	1
23093	202	0	28-Aug-13	20	1	8	2	1	1	0	1	3	4	1
23094	202	0	23-Aug-13	14	1	8	2	1	1	0	1	3	3	1
23095	202	0	5-Jul-13	14	1	8	2	3	1	0	1	3	4	1
23096	202	0	6-Sep-12	18	1	8	2	2	1	0	1	3	5	1
23097	202	0	29-Jul-12	19	1	8	2	1	1	0	1	3	5	2
23098	202	0	17-Oct-12	13	1	8	2	1	1	0	1	3	6	2
23099	202	0	5-Aug-12	12	1	8	2	1	1	0	1	3	1	2
23100	202	0	25-Feb-12	18	1	8	2	3	1	0	1	3	1	1
23101	202	0	29-Oct-12	9	1	8	2	1	1	0	1	3	5	1
23102	202	0	14-Jul-12	20	1	8	2	1	1	0	1	3	1	1
23103	202	0	11-Jun-17	18	1	8	2	1	1	0	1	3	3	1
23104	202	0	25-May-17	12	1	8	2	1	1	0	1	3	1	1
23105	202	0	25-Mar-17	4	1	8	2	2	1	0	1	3	5	2
23106	202	0	25-Jun-16	17	1	8	2	2	1	0	4	3	3	1
23107	202	0	1-Oct-15	20	1	8	2	2	1	0	4	3	3	1
23108	202	0	12-Oct-16	19	1	8	2	3	1	0	6	3	2	1
23109	202	0	17-Jun-15	13	1	8	2	1	1	0	6	3	2	1
23110	202	0	28-Aug-06	11	1	8	2	3	1	0	10	3	6	2
23111	202	0	9-Oct-07	12	1	8	2	1	1	0	13	3	1	1
23112	202	0	28-Jan-06	14	1	8	2	2	1	0	13	3	2	1
23113	202	0	15-Apr-12	15	1	8	2	1	1	0	1	3	5	1
23114	202	0	6-Feb-12	16	1	8	2	3	1	0	1	3	1	2
23115	202	0	16-Mar-11	20	1	8	2	1	1	0	1	3	6	2
23116	202	0	16-Mar-11	21	1	8	2	2	1	0	1	3	1	2
23117	202	0	13-May-11	12	1	8	2	1	1	0	1	3	2	1
23118	202	0	14-Oct-11	22	1	8	2	1	1	0	1	3	1	2
23119	202	0	2-Feb-11	14	1	8	2	1	1	0	1	3	3	2
23120	202	0	22-May-11	19	1	8	2	1	1	0	1	3	1	1
23121	202	0	2-Oct-11	20	1	8	2	1	1	0	1	3	3	1
23122	202	0	24-Feb-11	11	1	8	2	1	1	0	1	3	3	1
23123	202	0	11-Sep-11	13	1	8	2	1	1	0	1	3	3	1
23124	202	0	14-Sep-10	22	1	8	2	3	1	0	1	3	1	2
23125	202	0	23-May-10	18	1	8	2	1	1	0	1	3	6	1
23126	202	0	10-Nov-10	13	1	8	2	3	1	0	1	3	1	2
23127	202	0	14-Apr-10	19	1	8	2	1	1	0	1	3	1	2
23128	202	0	26-Mar-10	22	1	8	2	1	1	0	1	3	1	2
23129	202	0	31-Jul-10	17	1	8	2	1	1	0	1	3	1	1
23130	202	0	3-Jul-10	12	1	8	2	1	1	0	1	3	2	1
23131	202	0	18-Feb-10	19	1	8	2	2	1	0	1	3	3	2
23132	202	0	2-Feb-10	18	1	8	2	1	1	0	1	3	1	2
23133	202	0	23-Oct-10	10	1	8	2	2	1	0	1	3	2	2
23134	202	0	29-Oct-10	6	1	8	2	1	1	0	1	3	3	1
23135	202	0	30-Aug-09	20	1	8	2	1	1	0	1	3	3	1
23136	202	0	9-Sep-09	12	1	8	2	1	1	0	1	3	2	1
23137	202	0	8-Jul-09	20	1	8	2	1	1	0	1	3	5	1
23138	202	0	27-Aug-09	5	1	8	2	3	1	0	1	3	4	1
23139	202	0	25-May-09	19	1	8	2	3	1	0	1	3	6	2
23140	202	0	26-Jul-08	17	1	8	2	1	1	0	1	3	7	1
23141	202	0	12-Jun-08	7	1	8	2	1	1	0	1	3	6	1
23142	202	0	24-Mar-08	17	1	8	2	1	1	0	1	3	3	1
23143	202	0	28-Oct-08	18	1	8	2	1	1	0	1	3	3	1
23144	202	0	14-Mar-08	20	1	8	2	1	1	0	1	3	1	1
23145	202	0	23-Aug-08	13	1	8	2	1	1	0	1	3	1	1
23146	202	0	3-May-08	12	1	8	2	1	1	0	1	3	3	1
23147	202	0	24-Dec-08	14	1	8	2	1	1	0	1	3	1	2
23148	202	0	9-Sep-07	8	1	8	2	1	1	0	1	3	1	1
23149	202	0	30-Nov-07	16	1	8	2	3	1	0	1	3	2	1
23150	202	0	22-Jul-07	9	1	8	2	1	1	0	1	3	5	1
23151	202	0	16-May-06	12	1	8	2	1	1	0	1	3	2	1
23152	202	0	12-Dec-06	13	1	8	2	1	1	0	1	3	3	1
23153	202	0	25-Jun-06	19	1	8	2	1	1	0	1	3	3	1
23154	202	0	3-May-06	12	1	8	2	2	1	0	1	3	4	1
23155	202	0	22-Jul-06	17	1	8	2	2	1	0	1	3	1	2
23156	202	0	5-Jul-06	13	1	8	2	1	1	0	1	3	1	1
23157	202	0	6-Aug-06	12	1	8	2	3	1	0	1	3	1	2
23158	202	0	6-Dec-10	19	1	8	2	1	1	0	1	3	6	2
23159	202	0	10-May-08	14	1	8	2	2	1	0	1	3	6	2
23160	202	0	27-Jan-07	17	1	8	2	1	1	0	1	3	3	1
23161	202	0	27-Oct-16	10	1	8	2	1	1	0	4	3	2	1
23162	202	0	24-Jun-12	13	1	8	2	1	1	0	4	3	3	1
23163	202	0	2-Dec-09	20	1	8	2	1	1	0	4	3	6	1
23164	202	0	13-Sep-08	18	1	8	2	1	1	0	4	3	4	1
23165	202	0	27-Nov-08	15	1	8	2	1	1	0	4	3	7	1
23166	202	0	17-Mar-13	12	1	8	2	2	1	0	11	3	2	1
23167	202	0	5-Feb-12	2	1	8	2	1	1	0	11	3	2	1
23168	202	0	12-May-11	20	1	8	2	1	1	0	11	3	3	2
23169	202	0	14-Aug-11	21	1	8	2	1	1	0	11	3	3	2
23170	202	0	30-Aug-11	9	1	8	2	1	1	0	11	3	3	2
23171	202	0	3-Oct-09	17	1	8	2	1	1	0	11	3	4	1
23172	202	0	14-Feb-06	7	1	8	2	3	1	0	11	3	3	1
23173	202	0	17-Aug-16	16	1	8	2	1	1	0	3	3	1	1
23174	202	0	16-May-16	17	1	8	2	1	1	0	3	3	2	1
23175	202	0	28-Oct-16	13	1	8	2	1	1	0	3	3	1	1
23176	202	0	30-Nov-16	12	1	8	2	1	1	0	3	3	1	1
23177	202	0	12-Oct-16	12	1	8	2	1	1	0	3	3	3	1
23178	202	0	9-May-16	4	1	8	2	1	1	0	3	3	2	1
23179	202	0	4-Oct-16	10	1	8	2	2	1	0	3	3	3	1
23180	202	0	12-May-15	11	1	8	2	1	1	0	3	3	1	1
23181	202	0	13-Jul-15	20	1	8	2	3	1	0	3	3	2	1

23182	202	0	19-Nov-15	18	1	8	2	3	1	0	3	3	1	1
23183	202	0	8-Nov-14	20	1	8	2	1	1	0	3	3	1	1
23184	202	0	6-Dec-14	18	1	8	2	3	1	0	3	3	1	1
23185	202	0	18-Mar-14	22	1	8	2	1	1	0	3	3	1	1
23186	202	0	5-Jun-14	18	1	8	2	2	1	0	3	3	2	1
23187	202	0	14-Apr-14	13	1	8	2	3	1	0	3	3	2	1
23188	202	0	19-May-14	19	1	8	2	2	1	0	3	3	3	2
23189	202	0	27-Sep-13	21	1	8	2	1	1	0	3	3	1	1
23190	202	0	28-Dec-13	17	1	8	2	1	1	0	3	3	1	1
23191	202	0	19-Nov-13	20	1	8	2	3	1	0	3	3	2	1
23192	202	0	5-May-12	10	1	8	2	1	1	0	3	3	3	1
23193	202	0	17-Nov-11	17	1	8	2	1	1	0	3	3	1	2
23194	202	0	22-Jul-11	22	1	8	2	1	1	0	3	3	1	1
23195	202	0	3-Jan-10	11	1	8	2	3	1	0	3	3	1	2
23196	202	0	22-May-10	11	1	8	2	1	1	0	3	3	6	1
23197	202	0	20-Nov-09	12	1	8	2	1	1	0	3	3	1	1
23198	202	0	23-Oct-09	21	1	8	2	1	1	0	3	3	2	2
23199	202	0	6-Dec-08	21	1	8	2	2	1	0	3	3	7	1
23200	202	0	23-Aug-08	19	1	8	2	2	1	0	3	3	2	2
23201	202	0	28-Mar-08	6	1	8	2	1	1	0	3	3	1	1
23202	202	0	16-Feb-07	13	1	8	2	1	1	0	3	3	1	2
23203	202	0	7-Oct-07	19	1	8	2	3	1	0	3	3	1	1
23204	202	0	16-May-06	10	1	8	2	1	1	0	3	3	1	1
23205	202	0	26-Aug-17	11	1	8	2	1	1	0	3	3	3	1
23206	202	0	29-Oct-17	15	1	8	2	3	1	0	3	3	2	1
23207	202	0	20-Oct-10	17	1	8	2	1	1	0	6	3	6	2
23208	202	0	17-Oct-08	18	1	8	2	1	1	0	6	3	2	1
23209	202	0	4-Jun-16	0	2	8	2	3	1	0	1	3	4	1
23210	202	0	14-May-16	12	2	8	2	2	1	0	1	3	3	1
23211	202	0	2-Oct-13	12	2	8	2	1	1	0	1	3	3	2
23212	202	0	5-Feb-13	17	2	8	2	3	1	0	1	3	3	1
23213	202	0	16-Mar-17	13	2	8	2	2	1	0	1	3	1	1
23214	202	0	1-Sep-13	10	2	8	2	1	1	0	1	3	2	1
23215	202	0	12-Jul-14	16	2	8	2	1	1	0	1	3	1	1
23216	202	0	13-Jan-16	13	2	8	2	1	1	0	1	3	3	1
23217	202	0	29-Nov-13	12	2	8	2	1	1	0	1	3	2	1
23218	202	0	19-Aug-12	19	2	8	2	1	1	0	1	3	3	2
23219	202	0	12-Dec-12	15	2	8	2	1	1	0	1	3	3	1
23220	202	0	11-Feb-17	17	2	8	2	1	1	0	1	3	3	2
23221	202	0	23-Jun-14	13	2	8	2	1	1	0	1	3	1	1
23222	202	0	20-Sep-16	16	2	8	2	1	1	0	1	3	3	1
23223	202	0	18-Dec-16	19	2	8	2	3	1	0	1	3	5	1
23224	202	0	24-May-17	16	2	8	2	1	1	0	1	3	3	1
23225	202	0	27-May-12	18	2	8	2	1	1	0	1	3	3	2
23226	202	0	12-Aug-16	18	2	8	2	3	1	0	1	3	5	1
23227	202	0	22-Dec-16	18	2	8	2	3	1	0	1	3	4	2
23228	202	0	10-Aug-16	20	2	8	2	1	1	0	1	3	5	1
23229	202	0	29-Nov-16	21	2	8	2	1	1	0	1	3	3	1
23230	202	0	3-Feb-16	17	2	8	2	2	1	0	1	3	3	1
23231	202	0	28-May-15	16	2	8	2	3	1	0	1	3	1	1
23232	202	0	6-Oct-15	10	2	8	2	1	1	0	1	3	5	1
23233	202	0	6-Oct-15	10	2	8	2	1	1	0	1	3	5	1
23234	202	0	2-Jun-14	14	2	8	2	1	1	0	1	3	5	1
23235	202	0	5-Jan-14	20	2	8	2	3	1	0	1	3	1	1
23236	202	0	1-Sep-14	13	2	8	2	1	1	0	1	3	1	1
23237	202	0	6-Sep-14	18	2	8	2	1	1	0	1	3	5	2
23238	202	0	20-Dec-14	20	2	8	2	1	1	0	1	3	1	1
23239	202	0	3-Sep-13	11	2	8	2	1	1	0	1	3	3	2
23240	202	0	15-Oct-13	21	2	8	2	1	1	0	1	3	1	1
23241	202	0	7-Jul-13	17	2	8	2	1	1	0	1	3	3	1
23242	202	0	10-Jun-13	14	2	8	2	1	1	0	1	3	2	1
23243	202	0	13-Sep-13	7	2	8	2	1	1	0	1	3	1	1
23244	202	0	16-Sep-12	11	2	8	2	3	1	0	1	3	6	2
23245	202	0	26-Jun-17	21	2	8	2	1	1	0	1	3	4	1
23246	202	0	20-Sep-17	12	2	8	2	1	1	0	1	3	4	1
23247	202	0	28-Jan-17	11	2	8	2	1	1	0	1	3	3	1
23248	202	0	2-Oct-17	18	2	8	2	1	1	0	1	3	2	1
23249	202	0	19-May-17	0	2	8	2	1	1	0	1	3	1	1
23250	202	0	19-Jul-17	2	2	8	2	1	1	0	1	3	6	2
23251	202	0	14-Aug-17	8	2	8	2	2	1	0	1	3	3	1
23252	202	0	23-Mar-09	19	2	8	2	1	1	0	13	3	1	1
23253	202	0	22-Jul-09	20	2	8	2	1	1	0	13	3	3	1
23254	202	0	19-Apr-11	14	2	8	2	3	1	0	1	3	5	1
23255	202	0	26-Aug-11	18	2	8	2	1	1	0	1	3	6	2
23256	202	0	28-Jul-11	16	2	8	2	1	1	0	1	3	5	2
23257	202	0	13-Dec-11	12	2	8	2	1	1	0	1	3	3	1
23258	202	0	22-Nov-10	11	2	8	2	1	1	0	1	3	1	1
23259	202	0	23-Oct-09	17	2	8	2	3	1	0	1	3	6	1
23260	202	0	6-Jan-09	8	2	8	2	2	1	0	1	3	1	2
23261	202	0	26-Sep-09	17	2	8	2	1	1	0	1	3	3	2
23262	202	0	9-Dec-09	14	2	8	2	3	1	0	1	3	3	1
23263	202	0	24-Jul-08	9	2	8	2	3	1	0	1	3	1	2
23264	202	0	1-May-08	18	2	8	2	1	1	0	1	3	6	1
23265	202	0	3-Jan-08	8	2	8	2	2	1	0	1	3	1	2
23266	202	0	16-Apr-07	21	2	8	2	2	1	0	1	3	1	1
23267	202	0	8-Mar-07	13	2	8	2	1	1	0	1	3	1	2
23268	202	0	18-Dec-07	7	2	8	2	1	1	0	1	3	3	1
23269	202	0	21-Apr-07	19	2	8	2	1	1	0	1	3	3	1
23270	202	0	4-Oct-07	10	2	8	2	1	1	0	1	3	6	1
23271	202	0	27-Jul-07	16	2	8	2	1	1	0	1	3	1	1
23272	202	0	20-Jul-07	19	2	8	2	1	1	0	1	3	2	1
23273	202	0	20-Sep-06	13	2	8	2	1	1	0	1	3	1	1
23274	202	0	20-Sep-06	14	2	8	2	1	1	0	1	3	5	1
23275	202	0	2-May-06	13	2	8	2	2	1	0	1	3	3	2
23276	202	0	3-Dec-06	17	2	8	2	1	1	0	1	3	1	1
23277	202	0	11-Jul-06	10	2	8	2	1	1	0	1	3	6	1
23278	202	0	8-Feb-06	11	2	8	2	1	1	0	1	3	3	2
23279	202	0	29-Mar-12	19	2	8	2	1	1	0	1	3	3	1
23280	202	0	25-Aug-17	1	2	8	2	1	1	0	4	3	3	1
23281	202	0	17-Sep-17	16	2	8	2	1	1	0	4	3	2	1
23282	202	0	29-Aug-10	8	2	8	2	1	1	0	11	3	3	1
23283	202	0	17-Dec-13	18	2	8	2	1	1	0	3	3	1	1
23284	202	0	26-Jun-13	12	2	8	2	1	1	0	3	3	1	1
23285	202	0	15-Aug-13	16	2	8	2	1	1	0	3	3	1	1
23286	202	0	15-Nov-13	21	2	8	2	1	1	0	3	3	1	1
23287	202	0	13-Aug-12	19	2	8	2	1	1	0	3	3	3	1
23288	202	0	22-Feb-11	11	2	8	2	1	1	0	3	3	1	1
23289	202	0	14-Oct-11	17	2	8	2	1	1	0	3	3	3	1
23290	202	0	2-Jun-11	18	2	8	2	1	1	0	3	3	2	1
23291	202	0	19-Jan-10	15	2	8	2	1	1	0	3	3	1	2
23292	202	0	3-May-08	14	2	8	2	1	1	0	3	3	1	1
23293	202	0	13-May-08	21	2	8	2	1	1	0	3	3	6	1
23294	202	0	30-May-07	17	2	8	2	1	1	0	3	3	2	2
23295	202	0	1-Jan-06	11	2	8	2	1	1	0	3	3	1	1
23296	202	0	1-May-06	13	2	8	2	1	1	0	3	3	1	1
23297	202	0	2-Jun-17	19	2	8	2	1	1	0	3	3	3	1
23298	202	0	12-Jul-17	10	2	8	2	2	1	0	3	3	3	1
23299	202	0	16-Sep-11	19	2	8	2	2	1	0	6	3	3	1
23300	201	1	12-Aug-16	23	1	8	2	3	1	0	1	3	2	1
23301	201	1	15-Feb-14	8	1	8	2	1	1	0	1	3	3	1
23302	101	1	8-Jan-14	15	1	8	2	2	1	0	1	3	5	2
23303	201	1	22-Mar-13	19	1	8	2	3	1	0	1	3	1	1
23304	201	1	31-Oct-13	12	1	8	2	3	1	0	1	3	5	1
23305	201	1	26-Dec-12	6	1	8	2	1	1	0	1	3	4	1

23306	101	1	24-Nov-12	21	1	8	2	1	1	0	1	3	4	2
23307	201	1	9-Jan-17	13	1	8	2	3	1	0	1	3	4	1
23308	101	1	6-Jul-15	16	1	8	2	1	1	0	1	3	3	1
23309	201	1	19-Jan-16	16	1	8	2	1	1	0	1	3	3	1
23310	201	1	15-Nov-16	17	1	8	2	2	1	0	1	3	5	1
23311	101	1	6-Jul-15	20	1	8	2	1	1	0	1	3	4	1
23312	201	1	21-Oct-15	14	1	8	2	1	1	0	1	3	3	1
23313	201	1	1-Jul-15	7	1	8	2	1	1	0	1	3	1	1
23314	201	1	6-Jan-14	18	1	8	2	1	1	0	1	3	4	1
23315	201	1	14-May-14	13	1	8	2	2	1	0	1	3	4	1
23316	201	1	5-Dec-14	19	1	8	2	1	1	0	1	3	1	1
23317	201	1	17-Dec-14	10	1	8	2	2	1	0	1	3	5	1
23318	101	1	18-Jun-14	10	1	8	2	1	1	0	1	3	5	1
23319	101	1	2-Sep-14	20	1	8	2	1	1	0	1	3	3	1
23320	201	1	16-Jun-14	20	1	8	2	2	1	0	1	3	5	2
23321	101	1	27-Apr-14	12	1	8	2	2	1	0	1	3	4	1
23322	201	1	6-Feb-13	9	1	8	2	1	1	0	1	3	2	1
23323	101	1	7-Sep-13	18	1	8	2	1	1	0	1	3	2	4
23324	101	1	26-Sep-13	20	1	8	2	1	1	0	1	3	2	2
23325	201	1	2-Dec-13	13	1	8	2	3	1	0	1	3	3	1
23326	101	1	12-Feb-12	17	1	8	2	1	1	0	1	3	2	2
23327	101	1	19-Feb-12	21	1	8	2	1	1	0	1	3	3	1
23328	101	1	4-Oct-12	19	1	8	2	1	1	0	1	3	4	1
23329	101	1	12-Mar-17	16	1	8	2	1	1	0	1	3	2	1
23330	201	1	20-May-17	9	1	8	2	2	1	0	1	3	1	1
23331	201	1	13-Mar-17	12	1	8	2	2	1	0	1	3	1	1
23332	201	1	11-Jan-13	19	1	8	2	2	1	0	1	3	5	1
23333	201	1	31-Dec-14	16	1	8	2	3	1	0	1	3	2	2
23334	101	1	19-Oct-14	19	1	8	2	1	1	0	1	3	3	1
23335	201	1	29-Sep-12	16	1	8	2	3	1	0	1	3	3	1
23336	101	1	14-Apr-12	21	1	8	2	1	1	0	1	3	2	1
23337	101	1	17-Jul-12	11	1	8	2	1	1	0	1	3	4	1
23338	201	1	11-Jan-16	8	1	8	2	3	1	0	1	3	1	2
23339	201	1	22-Aug-16	9	1	8	2	3	1	0	1	3	3	1
23340	201	1	28-Feb-16	21	1	8	2	2	1	0	1	3	2	1
23341	201	1	11-May-15	17	1	8	2	1	1	0	1	3	5	1
23342	201	1	14-Nov-14	21	1	8	2	1	1	0	1	3	5	1
23343	201	1	8-Sep-14	17	1	8	2	3	1	0	1	3	1	2
23344	201	1	18-Mar-14	16	1	8	2	2	1	0	1	3	3	2
23345	201	1	29-Nov-13	13	1	8	2	1	1	0	1	3	3	1
23346	201	1	13-Jun-12	8	1	8	2	3	1	0	1	3	5	1
23347	101	1	12-Sep-12	7	1	8	2	1	1	0	1	3	5	1
23348	201	1	18-Nov-12	19	1	8	2	1	1	0	1	3	5	1
23349	201	1	20-Oct-15	7	1	8	2	1	1	0	1	3	1	1
23350	201	1	10-Aug-15	0	1	8	2	1	1	0	1	3	1	2
23351	101	1	25-Feb-15	18	1	8	2	1	1	0	1	3	3	1
23352	201	1	11-May-15	20	1	8	2	2	1	0	1	3	5	1
23353	101	1	27-Jun-12	16	1	8	2	1	1	0	1	3	4	1
23354	101	1	15-Mar-17	7	1	8	2	1	1	0	1	3	3	1
23355	201	1	9-Aug-17	21	1	8	2	3	1	0	1	3	5	1
23356	101	1	14-Jan-15	17	1	8	2	1	1	0	1	3	3	1
23357	101	1	20-Sep-17	17	1	8	2	1	1	0	1	3	3	2
23358	201	1	12-Aug-17	23	1	8	2	1	1	0	1	3	1	1
23359	201	1	9-Jan-12	16	1	8	2	2	1	0	1	3	2	2
23360	201	1	26-Mar-12	16	1	8	2	3	1	0	6	3	4	1
23361	101	1	17-Aug-12	17	1	8	2	1	1	0	3	3	2	1
23362	201	1	7-Apr-17	19	1	8	2	1	1	0	6	3	3	1
23363	101	1	1-May-08	18	1	8	2	1	1	0	13	3	4	4
23364	101	1	9-Aug-15	7	1	8	2	1	1	0	1	3	5	1
23365	101	1	13-Sep-14	18	1	8	2	1	1	0	1	3	3	1
23366	101	1	1-Jan-12	20	1	8	2	1	1	0	1	3	3	1
23367	101	1	22-Nov-12	17	1	8	2	1	1	0	1	3	5	1
23368	101	1	23-Mar-12	14	1	8	2	1	1	0	1	3	5	4
23369	101	1	12-Jun-11	14	1	8	2	1	1	0	1	3	1	1
23370	101	1	19-Jan-11	14	1	8	2	1	1	0	1	3	2	1
23371	101	1	18-Jun-11	17	1	8	2	1	1	0	1	3	1	2
23372	101	1	22-Dec-11	18	1	8	2	1	1	0	1	3	5	1
23373	201	1	26-Feb-11	15	1	8	2	1	1	0	1	3	6	1
23374	101	1	24-Jul-11	20	1	8	2	2	1	0	1	3	1	1
23375	201	1	26-May-11	15	1	8	2	1	1	0	1	3	3	2
23376	101	1	21-Dec-11	17	1	8	2	1	1	0	1	3	1	1
23377	201	1	9-Aug-11	20	1	8	2	3	1	0	1	3	3	2
23378	101	1	14-Jun-11	16	1	8	2	1	1	0	1	3	3	1
23379	201	1	28-Jun-10	18	1	8	2	1	1	0	1	3	4	1
23380	201	1	2-May-10	21	1	8	2	1	1	0	1	3	1	1
23381	101	1	11-Sep-10	15	1	8	2	1	1	0	1	3	5	1
23382	201	1	2-Oct-10	12	1	8	2	2	1	0	1	3	6	1
23383	201	1	14-Jul-10	8	1	8	2	1	1	0	1	3	6	1
23384	101	1	20-Jul-10	9	1	8	2	1	1	0	1	3	1	1
23385	201	1	24-Aug-10	20	1	8	2	1	1	0	1	3	1	1
23386	101	1	12-Dec-10	15	1	8	2	1	1	0	1	3	1	1
23387	101	1	10-Mar-10	18	1	8	2	1	1	0	1	3	3	2
23388	101	1	25-Oct-10	13	1	8	2	1	1	0	1	3	3	1
23389	201	1	24-Aug-10	23	1	8	2	1	1	0	1	3	4	2
23390	201	1	28-Jan-10	19	1	8	2	1	1	0	1	3	4	2
23391	201	1	4-Jun-09	22	1	8	2	2	1	0	1	3	2	1
23392	101	1	2-Aug-09	19	1	8	2	1	1	0	1	3	1	1
23393	201	1	20-Mar-09	13	1	8	2	2	1	0	1	3	1	2
23394	201	1	30-Nov-09	9	1	8	2	1	1	0	1	3	1	1
23395	201	1	3-Jun-09	10	1	8	2	1	1	0	1	3	2	1
23396	201	1	7-Nov-09	17	1	8	2	3	1	0	1	3	3	2
23397	201	1	27-Feb-09	20	1	8	2	2	1	0	1	3	1	1
23398	101	1	27-Oct-09	17	1	8	2	1	1	0	1	3	3	4
23399	201	1	17-Feb-09	7	1	8	2	3	1	0	1	3	1	2
23400	201	1	10-Feb-08	16	1	8	2	1	1	0	1	3	5	1
23401	201	1	26-May-08	8	1	8	2	1	1	0	1	3	3	1
23402	201	1	19-Nov-08	12	1	8	2	3	1	0	1	3	1	1
23403	101	1	21-May-08	18	1	8	2	1	1	0	1	3	5	1
23404	101	1	21-Sep-08	12	1	8	2	2	1	0	1	3	5	2
23405	101	1	6-Feb-08	15	1	8	2	1	1	0	1	3	6	1
23406	201	1	23-Jun-08	12	1	8	2	2	1	0	1	3	1	1
23407	201	1	29-Mar-08	10	1	8	2	2	1	0	1	3	5	2
23408	201	1	23-Feb-08	21	1	8	2	3	1	0	1	3	6	2
23409	201	1	30-Jul-08	23	1	8	2	2	1	0	1	3	1	1
23410	101	1	19-Jul-08	20	1	8	2	2	1	0	1	3	4	1
23411	201	1	30-Mar-08	20	1	8	2	3	1	0	1	3	4	2
23412	201	1	29-Jun-08	17	1	8	2	1	1	0	1	3	3	1
23413	201	1	1-Oct-08	10	1	8	2	1	1	0	1	3	3	2
23414	201	1	1-Aug-08	19	1	8	2	3	1	0	1	3	4	1
23415	201	1	15-Aug-08	16	1	8	2	3	1	0	1	3	4	1
23416	201	1	2-Sep-07	16	1	8	2	1	1	0	1	3	1	2
23417	201	1	15-Jul-07	8	1	8	2	1	1	0	1	3	6	2
23418	201	1	31-Oct-07	12	1	8	2	3	1	0	1	3	1	1
23419	101	1	14-Jan-07	13	1	8	2	1	1	0	1	3	1	1
23420	101	1	27-Mar-07	19	1	8	2	1	1	0	1	3	1	1
23421	201	1	24-Jan-07	23	1	8	2	1	1	0	1	3	7	1
23422	201	1	17-Sep-07	18	1	8	2	2	1	0	1	3	3	2
23423	101	1	3-Sep-07	17	1	8	2	1	1	0	1	3	2	1
23424	201	1	1-Apr-07	16	1	8	2	2	1	0	1	3	2	2
23425	201	1	1-Dec-07	11	1	8	2	3	1	0	1	3	1	1
23426	101	1	25-Mar-07	16	1	8	2	1	1	0	1	3	3	1
23427	101	1	22-Sep-07	11	1	8	2	3	1	0	1	3	3	2
23428	201	1	1-Aug-06	15	1	8	2	1	1	0	1	3	1	1
23429	201	1	26-Jun-06	21	1	8	2	1	1	0	1	3	5	2

23430	201	1	23-Jun-06	20	1	8	2	2	1	0	1	3	2	2
23431	101	1	18-Feb-06	20	1	8	2	1	1	0	1	3	1	1
23432	201	1	30-Aug-06	16	1	8	2	2	1	0	1	3	1	2
23433	101	1	22-Jul-06	19	1	8	2	1	1	0	1	3	6	1
23434	101	1	10-Jul-06	15	1	8	2	1	1	0	1	3	3	1
23435	201	1	3-Jul-06	20	1	8	2	1	1	0	1	3	1	1
23436	101	1	18-Apr-06	12	1	8	2	1	1	0	1	3	3	2
23437	101	1	23-Oct-06	7	1	8	2	1	1	0	1	3	4	1
23438	201	1	10-Mar-12	18	1	8	2	3	1	0	1	3	1	1
23439	101	1	4-Nov-11	20	1	8	2	1	1	0	1	3	1	1
23440	201	1	2-Mar-11	5	1	8	2	2	1	0	1	3	3	1
23441	201	1	23-Jul-11	22	1	8	2	2	1	0	1	3	1	2
23442	101	1	19-Jul-11	20	1	8	2	1	1	0	1	3	2	1
23443	101	1	3-Nov-11	10	1	8	2	1	1	0	1	3	2	1
23444	201	1	23-Feb-10	14	1	8	2	1	1	0	1	3	3	1
23445	201	1	15-Aug-10	14	1	8	2	1	1	0	1	3	2	1
23446	101	1	30-Jan-10	12	1	8	2	1	1	0	1	3	3	1
23447	101	1	5-Feb-10	15	1	8	2	1	1	0	1	3	3	1
23448	201	1	20-Jun-10	19	1	8	2	2	1	0	1	3	6	1
23449	201	1	26-Jul-09	20	1	8	2	1	1	0	1	3	6	1
23450	101	1	26-Jul-09	10	1	8	2	1	1	0	1	3	5	1
23451	101	1	1-Nov-09	19	1	8	2	1	1	0	1	3	1	1
23452	201	1	19-Feb-09	23	1	8	2	2	1	0	1	3	3	1
23453	201	1	10-Mar-08	10	1	8	2	1	1	0	1	3	3	1
23454	101	1	16-Jan-08	10	1	8	2	1	1	0	1	3	1	1
23455	201	1	26-Apr-08	11	1	8	2	3	1	0	1	3	3	1
23456	101	1	3-Nov-07	17	1	8	2	1	1	0	1	3	3	1
23457	201	1	16-Oct-07	16	1	8	2	2	1	0	1	3	3	1
23458	101	1	19-Jun-09	23	1	8	2	2	1	0	4	3	3	1
23459	201	1	29-Aug-09	20	1	8	2	1	1	0	4	3	2	1
23460	101	1	10-Oct-07	17	1	8	2	1	1	0	4	3	1	1
23461	101	1	20-Oct-17	18	1	8	2	1	1	0	4	3	4	1
23462	101	1	11-Sep-17	17	1	8	2	1	1	0	4	3	3	1
23463	101	1	13-Oct-16	7	1	8	2	1	1	0	11	3	1	1
23464	201	1	3-Mar-16	20	1	8	2	1	1	0	11	3	2	1
23465	101	1	18-Dec-16	8	1	8	2	1	1	0	11	3	2	1
23466	101	1	19-Mar-15	17	1	8	2	1	1	0	11	3	4	1
23467	201	1	9-Jan-15	16	1	8	2	1	1	0	11	3	2	1
23468	201	1	26-Apr-15	11	1	8	2	1	1	0	11	3	3	1
23469	201	1	4-Apr-15	15	1	8	2	1	1	0	11	3	1	1
23470	201	1	24-Aug-14	13	1	8	2	1	1	0	11	3	3	1
23471	101	1	25-Jun-14	8	1	8	2	1	1	0	11	3	2	1
23472	101	1	11-Nov-14	9	1	8	2	1	1	0	11	3	3	1
23473	101	1	13-Dec-13	16	1	8	2	1	1	0	11	3	2	1
23474	101	1	15-Aug-13	18	1	8	2	1	1	0	11	3	3	1
23475	101	1	5-Oct-13	15	1	8	2	1	1	0	11	3	2	1
23476	201	1	25-Jun-12	19	1	8	2	1	1	0	11	3	3	1
23477	101	1	29-Sep-12	20	1	8	2	1	1	0	11	3	3	1
23478	101	1	6-Jul-12	17	1	8	2	1	1	0	11	3	4	1
23479	201	1	21-Jan-12	19	1	8	2	2	1	0	11	3	2	1
23480	101	1	28-Feb-12	19	1	8	2	1	1	0	11	3	3	1
23481	101	1	12-Mar-11	18	1	8	2	1	1	0	11	3	5	1
23482	201	1	10-Aug-11	11	1	8	2	2	1	0	11	3	3	1
23483	201	1	3-Aug-11	17	1	8	2	2	1	0	11	3	3	1
23484	201	1	2-Apr-11	7	1	8	2	1	1	0	11	3	3	1
23485	101	1	4-Sep-11	17	1	8	2	1	1	0	11	3	3	1
23486	201	1	22-Dec-10	14	1	8	2	1	1	0	11	3	2	1
23487	101	1	8-May-10	20	1	8	2	1	1	0	11	3	3	1
23488	201	1	2-Jul-09	20	1	8	2	2	1	0	11	3	3	1
23489	201	1	24-Feb-09	10	1	8	2	1	1	0	11	3	7	1
23490	101	1	25-Oct-09	18	1	8	2	1	1	0	11	3	3	1
23491	101	1	15-Jul-09	10	1	8	2	1	1	0	11	3	2	1
23492	101	1	3-May-08	14	1	8	2	1	1	0	11	3	3	1
23493	101	1	30-Jul-07	20	1	8	2	1	1	0	11	3	2	1
23494	101	1	23-Apr-06	18	1	8	2	1	1	0	11	3	2	1
23495	101	1	31-May-06	19	1	8	2	1	1	0	11	3	2	1
23496	101	1	14-Apr-17	18	1	8	2	1	1	0	11	3	3	1
23497	101	1	30-Mar-17	16	1	8	2	1	1	0	11	3	1	1
23498	101	1	18-Apr-17	21	1	8	2	3	1	0	11	3	3	1
23499	101	1	12-Jan-17	20	1	8	2	3	1	0	11	3	2	1
23500	101	1	18-Nov-16	4	1	8	2	1	1	0	3	3	3	1
23501	101	1	24-Nov-16	3	1	8	2	1	1	0	3	3	1	1
23502	201	1	26-Jul-16	18	1	8	2	2	1	0	3	3	3	1
23503	201	1	2-Apr-16	20	1	8	2	1	1	0	3	3	3	1
23504	201	1	26-Nov-16	16	1	8	2	1	1	0	3	3	2	1
23505	201	1	15-Jul-16	17	1	8	2	1	1	0	3	3	3	1
23506	101	1	26-Jul-15	22	1	8	2	1	1	0	3	3	2	1
23507	201	1	7-May-15	16	1	8	2	2	1	0	3	3	1	1
23508	201	1	2-May-15	21	1	8	2	1	1	0	3	3	1	1
23509	201	1	10-Jul-15	8	1	8	2	1	1	0	3	3	5	1
23510	201	1	11-Sep-15	7	1	8	2	1	1	0	3	3	1	1
23511	101	1	18-Nov-15	19	1	8	2	1	1	0	3	3	1	1
23512	101	1	5-Jul-15	17	1	8	2	1	1	0	3	3	1	1
23513	201	1	9-Mar-15	16	1	8	2	1	1	0	3	3	1	1
23514	201	1	26-Jan-15	17	1	8	2	1	1	0	3	3	1	1
23515	101	1	4-Jun-15	17	1	8	2	1	1	0	3	3	1	1
23516	201	1	19-Sep-15	21	1	8	2	1	1	0	3	3	1	1
23517	101	1	8-May-15	18	1	8	2	1	1	0	3	3	1	1
23518	201	1	27-Mar-15	16	1	8	2	2	1	0	3	3	1	1
23519	101	1	1-Apr-15	19	1	8	2	1	1	0	3	3	3	1
23520	201	1	29-Aug-15	14	1	8	2	1	1	0	3	3	3	1
23521	101	1	31-Jul-15	12	1	8	2	1	1	0	3	3	3	1
23522	201	1	11-Mar-15	14	1	8	2	1	1	0	3	3	3	1
23523	101	1	31-Jan-15	19	1	8	2	1	1	0	3	3	3	1
23524	101	1	19-Jun-15	18	1	8	2	1	1	0	3	3	2	1
23525	101	1	5-May-15	7	1	8	2	1	1	0	3	3	3	1
23526	101	1	10-May-15	7	1	8	2	1	1	0	3	3	2	1
23527	201	1	8-Apr-13	16	1	8	2	2	1	0	3	3	1	1
23528	201	1	14-Mar-13	11	1	8	2	3	1	0	3	3	1	1
23529	101	1	25-Aug-13	18	1	8	2	1	1	0	3	3	1	1
23530	101	1	2-Jan-13	17	1	8	2	1	1	0	3	3	2	2
23531	201	1	18-Mar-13	18	1	8	2	2	1	0	3	3	2	1
23532	201	1	11-Jul-13	17	1	8	2	3	1	0	3	3	3	1
23533	201	1	1-Apr-13	11	1	8	2	3	1	0	3	3	3	1
23534	201	1	1-Apr-13	12	1	8	2	3	1	0	3	3	3	1
23535	201	1	12-Nov-12	17	1	8	2	3	1	0	3	3	1	1
23536	101	1	8-May-12	20	1	8	2	1	1	0	3	3	1	1
23537	101	1	18-Oct-12	12	1	8	2	1	1	0	3	3	1	1
23538	201	1	1-May-12	9	1	8	2	2	1	0	3	3	2	1
23539	101	1	12-Apr-12	17	1	8	2	1	1	0	3	3	2	2
23540	201	1	16-May-12	19	1	8	2	3	1	0	3	3	3	1
23541	201	1	17-Jun-12	11	1	8	2	2	1	0	3	3	3	1
23542	201	1	28-Jul-12	18	1	8	2	1	1	0	3	3	2	1
23543	201	1	28-Oct-11	15	1	8	2	3	1	0	3	3	2	2
23544	101	1	1-Jul-11	18	1	8	2	1	1	0	3	3	4	1
23545	201	1	27-Jan-11	17	1	8	2	1	1	0	3	3	1	1
23546	101	1	7-Mar-11	17	1	8	2	1	1	0	3	3	3	1
23547	101	1	3-Mar-10	18	1	8	2	1	1	0	3	3	4	1
23548	201	1	6-Feb-10	9	1	8	2	1	1	0	3	3	1	1
23549	201	1	16-Oct-10	21	1	8	2	1	1	0	3	3	3	1
23550	101	1	6-Jun-10	20	1	8	2	2	1	0	3	3	3	1
23551	101	1	29-Mar-10	19	1	8	2	1	1	0	3	3	2	1
23552	101	1	1-May-10	15	1	8	2	1	1	0	3	3	2	1
23553	101	1	22-Jul-09	17	1	8	2	2	1	0	3	3	2	4

23554	201	1	13-Sep-09	18	1	8	2	1	1	0	3	3	1	1
23555	201	1	25-Jul-09	9	1	8	2	1	1	0	3	3	1	1
23556	201	1	3-Sep-09	7	1	8	2	1	1	0	3	3	1	1
23557	201	1	1-Feb-09	19	1	8	2	2	1	0	3	3	2	1
23558	201	1	24-Oct-09	8	1	8	2	1	1	0	3	3	2	1
23559	201	1	14-Nov-09	12	1	8	2	1	1	0	3	3	3	1
23560	101	1	26-Dec-09	17	1	8	2	1	1	0	3	3	2	1
23561	101	1	25-Sep-09	16	1	8	2	1	1	0	3	3	3	1
23562	101	1	17-May-09	11	1	8	2	1	1	0	3	3	2	4
23563	101	1	10-Jun-09	10	1	8	2	1	1	0	3	3	3	1
23564	201	1	29-Oct-09	17	1	8	2	1	1	0	3	3	3	1
23565	201	1	16-Apr-08	16	1	8	2	1	1	0	3	3	5	1
23566	101	1	5-Jan-08	20	1	8	2	1	1	0	3	3	1	1
23567	101	1	30-Sep-08	21	1	8	2	1	1	0	3	3	2	1
23568	201	1	28-Jan-08	19	1	8	2	2	1	0	3	3	2	2
23569	201	1	26-Dec-08	12	1	8	2	2	1	0	3	3	1	1
23570	201	1	2-Mar-08	16	1	8	2	3	1	0	3	3	1	1
23571	201	1	4-Jul-08	15	1	8	2	2	1	0	3	3	1	1
23572	101	1	28-May-08	20	1	8	2	1	1	0	3	3	3	1
23573	101	1	26-Aug-08	20	1	8	2	1	1	0	3	3	3	1
23574	201	1	30-Mar-08	19	1	8	2	2	1	0	3	3	3	2
23575	201	1	12-Nov-07	10	1	8	2	3	1	0	3	3	1	1
23576	101	1	22-Sep-07	16	1	8	2	1	1	0	3	3	1	1
23577	101	1	7-Apr-07	19	1	8	2	1	1	0	3	3	1	1
23578	101	1	31-Aug-07	12	1	8	2	1	1	0	3	3	2	1
23579	201	1	28-May-07	12	1	8	2	1	1	0	3	3	3	1
23580	101	1	7-Nov-07	16	1	8	2	1	1	0	3	3	2	1
23581	101	1	2-Dec-07	20	1	8	2	1	1	0	3	3	2	1
23582	101	1	14-Nov-07	19	1	8	2	1	1	0	3	3	4	1
23583	101	1	5-Dec-07	16	1	8	2	1	1	0	3	3	3	1
23584	101	1	6-Nov-07	14	1	8	2	3	1	0	3	3	3	1
23585	101	1	15-Oct-06	14	1	8	2	1	1	0	3	3	3	1
23586	201	1	13-Mar-06	18	1	8	2	1	1	0	3	3	1	1
23587	101	1	27-Sep-06	21	1	8	2	1	1	0	3	3	1	1
23588	201	1	20-May-06	14	1	8	2	2	1	0	3	3	1	1
23589	201	1	8-Aug-06	19	1	8	2	1	1	0	3	3	1	1
23590	101	1	24-Sep-06	18	1	8	2	1	1	0	3	3	1	1
23591	201	1	13-Apr-06	13	1	8	2	1	1	0	3	3	1	1
23592	101	1	30-Jun-06	18	1	8	2	1	1	0	3	3	2	1
23593	201	1	7-Apr-06	10	1	8	2	1	1	0	3	3	3	1
23594	101	1	19-May-06	19	1	8	2	1	1	0	3	3	3	1
23595	101	1	13-Oct-06	20	1	8	2	1	1	0	3	3	3	1
23596	201	1	11-Sep-06	23	1	8	2	1	1	0	3	3	3	1
23597	101	1	21-Jun-06	16	1	8	2	1	1	0	3	3	2	1
23598	101	1	10-Nov-06	19	1	8	2	1	1	0	3	3	2	1
23599	101	1	1-Feb-17	17	1	8	2	1	1	0	3	3	5	1
23600	101	1	26-Mar-17	8	1	8	2	1	1	0	3	3	2	1
23601	201	1	24-Jun-17	18	1	8	2	1	1	0	3	3	2	1
23602	201	1	26-Aug-17	10	1	8	2	1	1	0	3	3	1	1
23603	201	1	13-Jun-17	16	1	8	2	1	1	0	3	3	1	1
23604	101	1	11-Jan-17	10	1	8	2	1	1	0	3	3	2	2
23605	201	1	13-Oct-17	16	1	8	2	2	1	0	3	3	3	1
23606	201	1	13-Jun-17	16	1	8	2	3	1	0	3	3	2	1
23607	101	1	23-Jun-16	20	1	8	2	1	1	0	6	3	3	1
23608	201	1	12-Sep-11	9	1	8	2	1	1	0	6	3	3	1
23609	101	1	26-Jun-08	12	1	8	2	1	1	0	6	3	3	1
23610	101	1	2-Dec-07	13	1	8	2	1	1	0	6	3	3	1
23611	101	1	16-May-17	20	1	8	2	1	1	0	6	3	3	1
23612	101	1	9-Nov-17	8	1	8	2	1	1	0	13	3	1	1
23613	101	1	29-May-12	7	2	8	2	1	1	0	7	3	3	1
23614	201	1	29-Mar-12	11	2	8	2	2	1	0	1	3	1	1
23615	201	1	20-Oct-15	19	2	8	2	1	1	0	1	3	5	1
23616	201	1	10-Jun-14	20	2	8	2	3	1	0	1	3	5	1
23617	201	1	22-Feb-13	16	2	8	2	2	1	0	1	3	3	1
23618	201	1	28-Sep-12	18	2	8	2	3	1	0	1	3	1	1
23619	101	1	16-Mar-17	22	2	8	2	1	1	0	4	3	3	1
23620	201	1	4-Jun-13	15	2	8	2	2	1	0	1	3	3	1
23621	201	1	2-Mar-16	18	2	8	2	3	1	0	1	3	3	1
23622	201	1	12-May-16	10	2	8	2	2	1	0	1	3	3	1
23623	101	1	14-Dec-15	19	2	8	2	1	1	0	1	3	5	1
23624	201	1	9-Jun-14	14	2	8	2	1	1	0	1	3	5	1
23625	101	1	2-Jun-12	16	2	8	2	1	1	0	1	3	1	1
23626	201	1	26-Feb-12	21	2	8	2	3	1	0	1	3	1	1
23627	201	1	31-Jul-12	10	2	8	2	1	1	0	1	3	6	1
23628	201	1	26-Jan-13	18	2	8	2	1	1	0	1	3	2	1
23629	201	1	2-Jan-14	14	2	8	2	2	1	0	1	3	2	1
23630	201	1	20-Nov-14	7	2	8	2	1	1	0	1	3	2	2
23631	101	1	30-Apr-13	11	2	8	2	1	1	0	1	3	5	1
23632	101	1	20-Dec-13	13	2	8	2	2	1	0	1	3	5	2
23633	201	1	31-Jul-12	9	2	8	2	1	1	0	1	3	6	1
23634	201	1	3-Apr-12	19	2	8	2	2	1	0	1	3	3	2
23635	201	1	27-Jun-17	10	2	8	2	1	1	0	1	3	5	1
23636	101	1	26-Feb-12	15	2	8	2	1	1	0	6	3	1	1
23637	101	1	8-Jul-16	11	2	8	2	1	1	0	1	3	2	1
23638	101	1	9-Oct-15	16	2	8	2	1	1	0	1	3	5	1
23639	101	1	5-Aug-13	16	2	8	2	1	1	0	1	3	3	1
23640	201	1	26-Aug-17	9	2	8	2	2	1	0	6	3	3	1
23641	201	1	17-Apr-16	16	2	8	2	1	1	0	4	3	3	1
23642	201	1	30-Nov-17	16	2	8	2	2	1	0	6	3	1	1
23643	201	1	11-May-14	19	2	8	2	1	1	0	6	3	3	1
23644	101	1	13-Mar-14	14	2	8	2	1	1	0	1	3	3	1
23645	101	1	20-Feb-12	13	2	8	2	1	1	0	1	3	5	1
23646	201	1	7-Dec-11	22	2	8	2	2	1	0	1	3	1	2
23647	201	1	19-Dec-11	16	2	8	2	2	1	0	1	3	1	1
23648	101	1	4-Oct-11	16	2	8	2	1	1	0	1	3	4	1
23649	201	1	14-May-11	3	2	8	2	3	1	0	1	3	4	1
23650	101	1	5-Nov-11	17	2	8	2	1	1	0	1	3	5	2
23651	101	1	7-Aug-10	22	2	8	2	1	1	0	1	3	1	4
23652	101	1	9-Apr-10	20	2	8	2	1	1	0	1	3	1	2
23653	101	1	29-Oct-10	17	2	8	2	1	1	0	1	3	3	1
23654	201	1	7-Jan-09	0	2	8	2	2	1	0	1	3	1	1
23655	101	1	17-Mar-09	7	2	8	2	2	1	0	1	3	2	1
23656	101	1	27-Aug-09	16	2	8	2	1	1	0	1	3	1	2
23657	201	1	26-Jul-09	14	2	8	2	2	1	0	1	3	3	1
23658	201	1	1-Jul-09	15	2	8	2	1	1	0	1	3	6	1
23659	201	1	8-Jun-09	21	2	8	2	2	1	0	1	3	5	2
23660	101	1	26-Sep-09	17	2	8	2	1	1	0	1	3	3	4
23661	201	1	30-Jul-08	23	2	8	2	2	1	0	1	3	1	1
23662	101	1	21-Mar-08	22	2	8	2	1	1	0	1	3	5	1
23663	201	1	2-Jul-08	17	2	8	2	3	1	0	1	3	3	1
23664	101	1	18-Jul-08	19	2	8	2	1	1	0	1	3	3	1
23665	101	1	20-Sep-08	17	2	8	2	1	1	0	1	3	3	1
23666	201	1	24-May-08	18	2	8	2	1	1	0	1	3	4	2
23667	201	1	18-Sep-08	12	2	8	2	3	1	0	1	3	1	2
23668	101	1	5-May-07	15	2	8	2	1	1	0	1	3	1	1
23669	101	1	7-Apr-07	12	2	8	2	3	1	0	1	3	5	1
23670	101	1	1-Dec-07	13	2	8	2	1	1	0	1	3	1	1
23671	201	1	12-Jun-07	10	2	8	2	1	1	0	1	3	1	2
23672	101	1	8-Aug-07	9	2	8	2	1	1	0	1	3	3	1
23673	201	1	17-Dec-06	12	2	8	2	1	1	0	1	3	5	1
23674	101	1	21-May-06	17	2	8	2	1	1	0	1	3	6	1
23675	201	1	4-Jun-06	16	2	8	2	1	1	0	1	3	1	2
23676	201	1	31-May-06	20	2	8	2	1	1	0	1	3	1	2
23677	101	1	15-Jul-06	14	2	8	2	1	1	0	1	3	3	1

23678	101	1	17-Mar-06	16	2	8	2	1	1	0	1	3	3	2
23679	101	1	14-Apr-06	14	2	8	2	3	1	0	1	3	5	2
23680	101	1	8-Feb-08	13	2	8	2	1	1	0	5	3	2	1
23681	101	1	18-Dec-15	8	2	8	2	1	1	0	1	3	3	1
23682	101	1	17-Aug-12	13	2	8	2	1	1	0	1	3	1	1
23683	101	1	22-Feb-12	18	2	8	2	1	1	0	1	3	4	1
23684	201	1	17-Sep-11	23	2	8	2	2	1	0	1	3	3	1
23685	101	1	1-Nov-11	18	2	8	2	1	1	0	1	3	4	1
23686	101	1	30-Jan-10	12	2	8	2	1	1	0	1	3	3	1
23687	101	1	31-Aug-10	12	2	8	2	1	1	0	1	3	3	1
23688	201	1	18-Jun-10	13	2	8	2	2	1	0	1	3	3	1
23689	101	1	1-May-09	19	2	8	2	2	1	0	1	3	4	1
23690	101	1	23-May-08	19	2	8	2	1	1	0	1	3	5	1
23691	201	1	30-Apr-08	12	2	8	2	2	1	0	1	3	4	1
23692	101	1	16-Sep-08	18	2	8	2	1	1	0	1	3	3	1
23693	201	1	6-Dec-07	12	2	8	2	1	1	0	1	3	4	1
23694	101	1	31-May-07	20	2	8	2	1	1	0	1	3	3	1
23695	101	1	8-Jun-12	20	2	8	2	1	1	0	4	3	3	1
23696	101	1	1-Feb-08	21	2	8	2	1	1	0	4	3	2	1
23697	101	1	14-Mar-08	8	2	8	2	1	1	0	4	3	3	1
23698	101	1	16-Aug-07	15	2	8	2	1	1	0	4	3	2	1
23699	101	1	25-Feb-06	18	2	8	2	1	1	0	4	3	2	1
23700	201	1	17-Jun-16	19	2	8	2	2	1	0	11	3	2	1
23701	101	1	14-Feb-16	21	2	8	2	1	1	0	11	3	2	1
23702	201	1	22-Nov-15	9	2	8	2	2	1	0	11	3	2	1
23703	201	1	14-Mar-14	7	2	8	2	1	1	0	11	3	2	1
23704	201	1	29-Oct-13	8	2	8	2	1	1	0	11	3	2	1
23705	201	1	30-May-12	20	2	8	2	3	1	0	11	3	3	1
23706	201	1	27-Mar-10	12	2	8	2	2	1	0	11	3	3	1
23707	101	1	3-Oct-09	9	2	8	2	2	1	0	11	3	3	1
23708	101	1	25-Sep-09	9	2	8	2	1	1	0	11	3	3	1
23709	201	1	27-Aug-08	10	2	8	2	3	1	0	11	3	2	1
23710	201	1	9-Oct-16	12	2	8	2	1	1	0	3	3	2	1
23711	201	1	16-Aug-16	11	2	8	2	3	1	0	3	3	3	1
23712	101	1	8-Nov-16	3	2	8	2	1	1	0	3	3	3	1
23713	101	1	3-May-15	18	2	8	2	3	1	0	3	3	3	1
23714	101	1	8-Sep-15	17	2	8	2	1	1	0	3	3	2	2
23715	101	1	16-May-15	12	2	8	2	1	1	0	3	3	1	1
23716	101	1	8-Aug-15	17	2	8	2	1	1	0	3	3	5	1
23717	101	1	19-Jun-15	7	2	8	2	1	1	0	3	3	3	1
23718	101	1	6-May-15	15	2	8	2	1	1	0	3	3	3	1
23719	101	1	25-May-15	20	2	8	2	1	1	0	3	3	2	1
23720	101	1	20-Jul-14	17	2	8	2	1	1	0	3	3	3	1
23721	201	1	9-Jun-14	17	2	8	2	3	1	0	3	3	3	1
23722	101	1	4-Oct-14	20	2	8	2	1	1	0	3	3	3	1
23723	101	1	27-Jul-13	19	2	8	2	3	1	0	3	3	5	1
23724	201	1	27-Jul-13	20	2	8	2	2	1	0	3	3	3	1
23725	101	1	20-Mar-12	16	2	8	2	1	1	0	3	3	7	1
23726	201	1	20-Aug-12	21	2	8	2	3	1	0	3	3	1	1
23727	101	1	15-Sep-12	18	2	8	2	1	1	0	3	3	2	2
23728	201	1	28-Sep-12	19	2	8	2	3	1	0	3	3	3	1
23729	101	1	17-Oct-12	10	2	8	2	1	1	0	3	3	2	1
23730	201	1	9-Apr-11	10	2	8	2	1	1	0	3	3	3	1
23731	101	1	7-Jun-10	10	2	8	2	1	1	0	3	3	3	1
23732	101	1	1-Oct-10	17	2	8	2	1	1	0	3	3	3	1
23733	201	1	11-Feb-09	16	2	8	2	1	1	0	3	3	1	1
23734	201	1	22-Oct-09	12	2	8	2	1	1	0	3	3	1	1
23735	201	1	14-May-09	16	2	8	2	1	1	0	3	3	3	1
23736	101	1	4-Apr-09	19	2	8	2	1	1	0	3	3	3	1
23737	101	1	11-Sep-09	20	2	8	2	1	1	0	3	3	3	1
23738	101	1	16-May-08	20	2	8	2	1	1	0	3	3	1	1
23739	201	1	14-May-08	7	2	8	2	1	1	0	3	3	3	2
23740	201	1	24-Dec-08	18	2	8	2	2	1	0	3	3	1	1
23741	101	1	21-Oct-08	15	2	8	2	1	1	0	3	3	2	1
23742	101	1	10-Oct-07	18	2	8	2	1	1	0	3	3	6	1
23743	201	1	15-Jan-07	22	2	8	2	1	1	0	3	3	3	1
23744	101	1	25-Apr-07	17	2	8	2	1	1	0	3	3	3	1
23745	101	1	21-Jun-06	17	2	8	2	1	1	0	3	3	2	1
23746	101	1	18-Nov-06	10	2	8	2	1	1	0	3	3	1	2
23747	201	1	6-Dec-06	13	2	8	2	2	1	0	3	3	1	1
23748	101	1	19-Oct-06	14	2	8	2	1	1	0	3	3	1	1
23749	101	1	3-Oct-06	11	2	8	2	1	1	0	3	3	1	1
23750	101	1	20-Dec-06	17	2	8	2	1	1	0	3	3	1	1
23751	101	1	5-Jun-06	17	2	8	2	1	1	0	3	3	3	1
23752	101	1	28-Mar-17	19	2	8	2	1	1	0	3	3	3	1
23753	201	1	26-Jun-17	16	2	8	2	1	1	0	3	3	3	1
23754	101	1	6-Jan-17	10	2	8	2	1	1	0	3	3	2	1
23755	201	1	31-Jul-17	21	2	8	2	2	1	0	3	3	3	1
23756	101	1	3-Sep-17	9	2	8	2	1	1	0	3	3	1	1
23757	201	1	21-Dec-17	7	2	8	2	2	1	0	3	3	3	1
23758	201	1	16-Dec-17	11	2	8	2	3	1	0	6	3	3	1
23759	202	0	15-Jun-15	12	1	9	2	1	1	0	1	3	3	1
23760	202	0	6-Apr-17	12	1	9	2	1	1	0	1	3	3	1
23761	202	0	14-Jun-14	12	1	9	2	3	1	0	1	3	3	1
23762	202	0	20-Jul-16	13	1	9	2	1	1	0	1	3	4	1
23763	202	0	29-Aug-16	18	1	9	2	2	1	0	1	3	1	1
23764	202	0	5-Jan-14	10	1	9	2	1	1	0	1	3	1	1
23765	202	0	18-Jul-14	8	1	9	2	3	1	0	1	3	3	1
23766	202	0	20-Jul-13	18	1	9	2	1	1	0	1	3	3	1
23767	202	0	28-Oct-17	15	1	9	2	1	1	0	6	3	4	2
23768	202	0	5-Feb-17	17	1	9	2	3	1	0	1	3	1	2
23769	202	0	19-Jun-13	15	1	9	2	2	1	0	1	3	1	1
23770	202	0	16-Feb-13	16	1	9	2	1	1	0	1	3	1	1
23771	202	0	6-Jul-16	15	1	9	2	3	1	0	1	3	1	1
23772	202	0	6-Oct-17	16	1	9	2	3	1	0	1	3	1	1
23773	202	0	16-Dec-17	14	1	9	2	1	1	0	1	3	1	1
23774	202	0	17-Oct-15	18	1	9	2	3	1	0	1	3	3	1
23775	202	0	16-Dec-15	17	1	9	2	3	1	0	1	3	3	1
23776	202	0	1-Nov-16	14	1	9	2	1	1	0	1	3	3	2
23777	202	0	19-Nov-15	19	1	9	2	3	1	0	1	3	3	1
23778	202	0	3-Aug-14	0	1	9	2	3	1	0	1	3	4	1
23779	202	0	6-Oct-17	19	1	9	2	1	1	0	1	3	3	1
23780	202	0	29-Jun-16	15	1	9	2	1	1	0	1	3	3	1
23781	202	0	27-Jun-15	11	1	9	2	1	1	0	1	3	1	1
23782	202	0	25-Dec-15	19	1	9	2	3	1	0	1	3	3	1
23783	202	0	17-Jul-14	18	1	9	2	2	1	0	1	3	1	1
23784	202	0	11-Jun-15	7	1	9	2	3	1	0	1	3	5	2
23785	202	0	31-Oct-15	9	1	9	2	1	1	0	1	3	2	1
23786	202	0	4-Nov-15	17	1	9	2	1	1	0	1	3	3	1
23787	202	0	28-May-16	12	1	9	2	3	1	0	1	3	1	1
23788	202	0	11-Sep-16	18	1	9	2	1	1	0	1	3	5	2
23789	202	0	26-Jul-16	23	1	9	2	1	1	0	1	3	3	1
23790	202	0	22-May-16	12	1	9	2	3	1	0	1	3	5	1
23791	202	0	5-Jul-15	18	1	9	2	1	1	0	1	3	5	1
23792	202	0	22-Aug-15	18	1	9	2	2	1	0	1	3	1	1
23793	202	0	29-Nov-15	19	1	9	2	3	1	0	1	3	1	1
23794	202	0	15-Jul-15	13	1	9	2	3	1	0	1	3	1	1
23795	202	0	6-Jan-14	18	1	9	2	1	1	0	1	3	3	1
23796	202	0	8-Aug-14	21	1	9	2	3	1	0	1	3	6	1
23797	202	0	20-Apr-14	11	1	9	2	1	1	0	1	3	5	1
23798	202	0	8-Jun-13	11	1	9	2	1	1	0	1	3	1	1
23799	202	0	13-Oct-13	8	1	9	2	2	1	0	1	3	5	2
23800	202	0	30-Oct-13	6	1	9	2	3	1	0	1	3	5	1
23801	202	0	9-Dec-13	16	1	9	2	3	1	0	1	3	4	1

23802	202	0	1-Jan-12	17	1	9	2	3	1	0	1	3	1	1
23803	202	0	1-Oct-12	21	1	9	2	1	1	0	1	3	6	1
23804	202	0	24-Jun-12	18	1	9	2	1	1	0	1	3	2	1
23805	202	0	3-Mar-12	18	1	9	2	1	1	0	1	3	1	1
23806	202	0	17-Aug-17	8	1	9	2	1	1	0	1	3	3	1
23807	202	0	3-Jan-17	17	1	9	2	1	1	0	1	3	2	1
23808	202	0	23-May-17	18	1	9	2	1	1	0	1	3	1	1
23809	202	0	8-Feb-17	20	1	9	2	3	1	0	1	3	3	1
23810	202	0	1-Aug-17	8	1	9	2	3	1	0	1	3	3	2
23811	202	0	30-Mar-17	18	1	9	2	1	1	0	6	3	3	1
23812	202	0	14-Mar-17	21	1	9	2	3	1	0	6	3	2	1
23813	202	0	13-Aug-13	12	1	9	2	1	1	0	7	3	3	1
23814	202	0	12-May-09	12	1	9	2	3	1	0	13	3	3	1
23815	202	0	16-Dec-06	18	1	9	2	1	1	0	13	3	1	1
23816	202	0	22-Dec-12	14	1	9	2	1	1	0	1	3	1	1
23817	202	0	30-May-11	19	1	9	2	1	1	0	1	3	1	1
23818	202	0	5-Jun-11	18	1	9	2	1	1	0	1	3	5	1
23819	202	0	15-Jul-10	21	1	9	2	3	1	0	1	3	1	1
23820	202	0	15-May-10	9	1	9	2	1	1	0	1	3	3	1
23821	202	0	5-Dec-10	10	1	9	2	1	1	0	1	3	1	2
23822	202	0	29-Mar-10	17	1	9	2	1	1	0	1	3	1	2
23823	202	0	12-Nov-10	23	1	9	2	1	1	0	1	3	1	1
23824	202	0	12-Jun-09	18	1	9	2	1	1	0	1	3	6	1
23825	202	0	18-Sep-09	18	1	9	2	1	1	0	1	3	6	1
23826	202	0	1-Oct-09	21	1	9	2	1	1	0	1	3	3	1
23827	202	0	4-Sep-09	18	1	9	2	3	1	0	1	3	2	2
23828	202	0	3-Apr-09	20	1	9	2	1	1	0	1	3	6	2
23829	202	0	9-May-09	17	1	9	2	1	1	0	1	3	3	1
23830	202	0	3-Apr-09	14	1	9	2	1	1	0	1	3	1	1
23831	202	0	30-Sep-09	17	1	9	2	1	1	0	1	3	3	2
23832	202	0	14-Aug-08	2	1	9	2	3	1	0	1	3	6	1
23833	202	0	16-Apr-08	17	1	9	2	1	1	0	1	3	1	2
23834	202	0	2-Aug-08	9	1	9	2	3	1	0	1	3	6	2
23835	202	0	24-Jun-08	11	1	9	2	3	1	0	1	3	1	1
23836	202	0	31-Aug-08	13	1	9	2	1	1	0	1	3	3	2
23837	202	0	30-Apr-08	12	1	9	2	3	1	0	1	3	6	1
23838	202	0	3-Oct-08	18	1	9	2	1	1	0	1	3	3	1
23839	202	0	9-Jan-08	18	1	9	2	1	1	0	1	3	3	1
23840	202	0	4-Apr-07	14	1	9	2	1	1	0	1	3	3	1
23841	202	0	4-Nov-07	18	1	9	2	1	1	0	1	3	1	1
23842	202	0	29-Sep-07	11	1	9	2	1	1	0	1	3	3	1
23843	202	0	18-Mar-07	15	1	9	2	1	1	0	1	3	2	1
23844	202	0	26-Aug-07	22	1	9	2	2	1	0	1	3	2	1
23845	202	0	9-May-07	10	1	9	2	1	1	0	1	3	3	1
23846	202	0	16-Feb-07	18	1	9	2	1	1	0	1	3	1	1
23847	202	0	19-Feb-07	13	1	9	2	1	1	0	1	3	3	2
23848	202	0	24-May-06	13	1	9	2	4	1	0	1	3	1	2
23849	202	0	17-Apr-06	14	1	9	2	1	1	0	1	3	3	1
23850	202	0	27-Jun-06	16	1	9	2	1	1	0	1	3	1	1
23851	202	0	1-Oct-06	13	1	9	2	1	1	0	1	3	3	1
23852	202	0	16-Nov-10	12	1	9	2	1	1	0	5	3	1	1
23853	202	0	22-Apr-08	18	1	9	2	1	1	0	1	3	1	1
23854	202	0	31-Jan-07	13	1	9	2	1	1	0	1	3	1	1
23855	202	0	25-Dec-07	18	1	9	2	3	1	0	1	3	3	2
23856	202	0	10-Sep-06	11	1	9	2	1	1	0	1	3	1	1
23857	202	0	1-Nov-16	10	1	9	2	1	1	0	4	3	4	1
23858	202	0	3-Oct-15	16	1	9	2	3	1	0	4	3	2	1
23859	202	0	13-May-12	15	1	9	2	1	1	0	4	3	3	1
23860	202	0	15-Nov-08	13	1	9	2	1	1	0	4	3	7	2
23861	202	0	13-Apr-08	19	1	9	2	1	1	0	4	3	1	1
23862	202	0	12-Apr-14	13	1	9	2	1	1	0	11	3	3	1
23863	202	0	5-Mar-11	17	1	9	2	3	1	0	11	3	3	1
23864	202	0	11-Apr-10	16	1	9	2	1	1	0	11	3	4	1
23865	202	0	16-Sep-09	12	1	9	2	1	1	0	11	3	3	2
23866	202	0	11-Dec-08	13	1	9	2	1	1	0	11	3	3	1
23867	202	0	20-Jun-06	19	1	9	2	1	1	0	11	3	2	1
23868	202	0	16-Aug-17	21	1	9	2	2	1	0	11	3	3	2
23869	202	0	6-Jul-17	14	1	9	2	2	1	0	11	3	3	1
23870	202	0	17-Jan-17	16	1	9	2	3	1	0	11	3	2	1
23871	202	0	14-Nov-16	13	1	9	2	1	1	0	3	3	1	1
23872	202	0	24-Oct-16	15	1	9	2	1	1	0	3	3	1	1
23873	202	0	27-Aug-16	17	1	9	2	2	1	0	3	3	1	1
23874	202	0	3-Jun-15	17	1	9	2	2	1	0	3	3	1	1
23875	202	0	7-Sep-15	17	1	9	2	2	1	0	3	3	1	1
23876	202	0	19-Jul-15	21	1	9	2	1	1	0	3	3	1	1
23877	202	0	9-Jul-15	17	1	9	2	2	1	0	3	3	4	1
23878	202	0	31-Oct-15	16	1	9	2	3	1	0	3	3	3	2
23879	202	0	7-Sep-15	7	1	9	2	1	1	0	3	3	2	1
23880	202	0	21-Jul-14	13	1	9	2	1	1	0	3	3	1	1
23881	202	0	29-Oct-14	20	1	9	2	3	1	0	3	3	1	1
23882	202	0	12-Jul-13	20	1	9	2	2	1	0	3	3	2	2
23883	202	0	12-Jan-11	16	1	9	2	1	1	0	3	3	1	1
23884	202	0	14-Aug-10	11	1	9	2	1	1	0	3	3	1	1
23885	202	0	12-Oct-10	20	1	9	2	1	1	0	3	3	3	1
23886	202	0	11-Aug-10	17	1	9	2	3	1	0	3	3	2	2
23887	202	0	12-Sep-09	18	1	9	2	1	1	0	3	3	3	2
23888	202	0	30-Apr-09	22	1	9	2	1	1	0	3	3	1	1
23889	202	0	1-Dec-09	12	1	9	2	2	1	0	3	3	7	1
23890	202	0	14-Mar-08	17	1	9	2	1	1	0	3	3	4	2
23891	202	0	2-Jan-08	12	1	9	2	1	1	0	3	3	2	1
23892	202	0	1-Sep-07	14	1	9	2	3	1	0	3	3	6	1
23893	202	0	3-Sep-07	14	1	9	2	1	1	0	3	3	1	1
23894	202	0	18-Mar-06	19	1	9	2	2	1	0	3	3	1	1
23895	202	0	20-Nov-17	14	1	9	2	1	1	0	3	3	1	1
23896	202	0	8-Sep-10	18	1	9	2	1	1	0	6	3	2	1
23897	202	0	6-Oct-09	6	1	9	2	3	1	0	6	3	3	2
23898	202	0	18-Oct-08	16	1	9	2	1	1	0	6	3	3	2
23899	202	0	13-Dec-15	18	2	9	2	1	1	0	1	3	1	1
23900	202	0	29-Apr-17	22	2	9	2	1	1	0	1	3	3	1
23901	202	0	26-Apr-17	16	2	9	2	3	1	0	1	3	5	1
23902	202	0	9-Jul-16	16	2	9	2	1	1	0	1	3	1	1
23903	202	0	20-Aug-14	11	2	9	2	2	1	0	1	3	3	1
23904	202	0	18-Oct-14	18	2	9	2	1	1	0	1	3	3	1
23905	202	0	29-Nov-13	12	2	9	2	1	1	0	1	3	5	1
23906	202	0	8-Feb-16	17	2	9	2	1	1	0	1	3	3	2
23907	202	0	21-Jan-17	22	2	9	2	1	1	0	1	3	1	1
23908	202	0	25-Jun-12	17	2	9	2	1	1	0	13	3	3	2
23909	202	0	7-Jul-13	1	2	9	2	1	1	0	1	3	1	1
23910	202	0	1-Oct-16	18	2	9	2	3	1	0	1	3	1	1
23911	202	0	16-May-16	13	2	9	2	1	1	0	1	3	3	1
23912	202	0	11-Jan-16	7	2	9	2	3	1	0	1	3	5	1
23913	202	0	20-Jun-15	19	2	9	2	1	1	0	1	3	3	1
23914	202	0	15-Sep-15	20	2	9	2	1	1	0	1	3	5	1
23915	202	0	18-Sep-15	13	2	9	2	3	1	0	1	3	3	1
23916	202	0	5-Oct-15	19	2	9	2	1	1	0	1	3	5	1
23917	202	0	2-Jan-14	11	2	9	2	1	1	0	1	3	1	1
23918	202	0	24-Dec-13	17	2	9	2	1	1	0	1	3	1	1
23919	202	0	15-Oct-13	21	2	9	2	3	1	0	1	3	5	1
23920	202	0	10-Sep-13	17	2	9	2	3	1	0	1	3	1	1
23921	202	0	5-Jul-13	14	2	9	2	3	1	0	1	3	1	1
23922	202	0	30-Jul-13	8	2	9	2	1	1	0	1	3	3	1
23923	202	0	17-Oct-12	13	2	9	2	1	1	0	1	3	5	2
23924	202	0	9-Jan-17	19	2	9	2	1	1	0	1	3	1	1
23925	202	0	12-Feb-17	14	2	9	2	1	1	0	1	3	1	1

23926	202	0	26-Mar-17	20	2	9	2	1	1	0	1	3	1	1
23927	202	0	11-Dec-17	17	2	9	2	1	1	0	1	3	3	1
23928	202	0	16-Oct-15	18	2	9	2	1	1	0	3	3	2	1
23929	202	0	24-Dec-09	0	2	9	2	1	1	0	13	3	2	1
23930	202	0	23-Apr-11	14	2	9	2	1	1	0	1	3	2	1
23931	202	0	27-Aug-11	11	2	9	2	1	1	0	1	3	4	1
23932	202	0	1-Jan-11	17	2	9	2	3	1	0	1	3	6	2
23933	202	0	1-Dec-11	10	2	9	2	1	1	0	1	3	2	1
23934	202	0	20-Oct-10	7	2	9	2	1	1	0	1	3	3	1
23935	202	0	28-Dec-09	14	2	9	2	1	1	0	1	3	3	1
23936	202	0	11-Apr-08	18	2	9	2	1	1	0	1	3	2	1
23937	202	0	31-Aug-08	11	2	9	2	3	1	0	1	3	2	2
23938	202	0	17-Feb-08	13	2	9	2	1	1	0	1	3	3	2
23939	202	0	4-Nov-07	11	2	9	2	1	1	0	1	3	6	1
23940	202	0	21-Nov-07	18	2	9	2	1	1	0	1	3	1	1
23941	202	0	6-Jun-07	14	2	9	2	1	1	0	1	3	2	1
23942	202	0	25-Dec-07	7	2	9	2	1	1	0	1	3	1	1
23943	202	0	12-May-07	13	2	9	2	1	1	0	1	3	6	1
23944	202	0	2-Dec-07	21	2	9	2	3	1	0	1	3	3	1
23945	202	0	17-Dec-06	13	2	9	2	1	1	0	1	3	3	1
23946	202	0	29-Oct-06	17	2	9	2	2	1	0	1	3	6	2
23947	202	0	23-Oct-06	11	2	9	2	1	1	0	1	3	3	1
23948	202	0	6-Apr-06	14	2	9	2	1	1	0	1	3	1	1
23949	202	0	13-Jul-06	9	2	9	2	1	1	0	5	3	3	1
23950	202	0	31-Jul-10	14	2	9	2	1	1	0	1	3	6	2
23951	202	0	17-Sep-06	11	2	9	2	1	1	0	1	3	1	1
23952	202	0	17-Feb-06	20	2	9	2	1	1	0	1	3	4	1
23953	202	0	26-May-06	18	2	9	2	1	1	0	1	3	1	1
23954	202	0	21-Aug-13	10	2	9	2	1	1	0	4	3	1	1
23955	202	0	25-Aug-11	20	2	9	2	2	1	0	11	3	3	2
23956	202	0	12-Jan-17	19	2	9	2	3	1	0	11	3	2	1
23957	202	0	28-Feb-14	18	2	9	2	1	1	0	3	3	2	2
23958	202	0	20-Jul-13	21	2	9	2	1	1	0	3	3	1	1
23959	202	0	13-Apr-12	7	2	9	2	1	1	0	3	3	1	1
23960	202	0	28-Nov-09	20	2	9	2	1	1	0	3	3	1	1
23961	202	0	3-May-08	10	2	9	2	1	1	0	3	3	2	1
23962	202	0	8-Jul-07	12	2	9	2	3	1	0	3	3	3	1
23963	202	0	17-Dec-06	13	2	9	2	1	1	0	3	3	4	1
23964	202	0	29-Nov-06	12	2	9	2	1	1	0	3	3	3	1
23965	202	0	15-Mar-13	19	2	9	2	3	1	0	6	3	3	1
23966	202	0	16-Nov-07	13	2	9	2	1	1	0	6	3	2	1
23967	101	1	10-May-12	7	1	9	2	3	1	0	1	3	1	2
23968	101	1	5-Oct-13	13	1	9	2	1	1	0	1	3	5	1
23969	101	1	30-Aug-12	20	1	9	2	1	1	0	1	3	1	1
23970	201	1	30-Dec-12	15	1	9	2	3	1	0	1	3	3	1
23971	101	1	3-Aug-15	20	1	9	2	2	1	0	1	3	3	2
23972	101	1	1-Dec-15	18	1	9	2	1	1	0	1	3	4	2
23973	101	1	7-Nov-13	13	1	9	2	1	1	0	1	3	3	2
23974	101	1	19-Oct-12	16	1	9	2	1	1	0	1	3	3	1
23975	101	1	17-Feb-17	22	1	9	2	1	1	0	1	3	4	1
23976	101	1	27-Oct-12	17	1	9	2	1	1	0	1	3	1	1
23977	201	1	28-Jul-16	19	1	9	2	1	1	0	1	3	5	1
23978	201	1	9-May-16	11	1	9	2	1	1	0	1	3	5	1
23979	201	1	19-Mar-16	19	1	9	2	2	1	0	1	3	2	1
23980	201	1	30-Dec-16	16	1	9	2	1	1	0	1	3	3	1
23981	101	1	26-May-15	19	1	9	2	1	1	0	1	3	2	1
23982	201	1	10-Feb-15	21	1	9	2	3	1	0	1	3	3	1
23983	201	1	10-Jan-15	20	1	9	2	2	1	0	1	3	4	1
23984	101	1	18-Oct-15	14	1	9	2	1	1	0	1	3	3	1
23985	201	1	19-Dec-14	1	1	9	2	1	1	0	1	3	1	1
23986	201	1	4-Oct-14	6	1	9	2	1	1	0	1	3	2	1
23987	201	1	27-Sep-14	6	1	9	2	2	1	0	1	3	3	2
23988	201	1	30-Jun-14	6	1	9	2	1	1	0	1	3	3	1
23989	101	1	1-Dec-13	14	1	9	2	1	1	0	1	3	1	1
23990	201	1	31-Jan-13	8	1	9	2	1	1	0	1	3	1	1
23991	101	1	17-Apr-12	8	1	9	2	1	1	0	1	3	2	1
23992	101	1	5-Nov-12	18	1	9	2	1	1	0	1	3	2	1
23993	201	1	23-Mar-12	13	1	9	2	1	1	0	1	3	4	1
23994	201	1	17-Feb-17	18	1	9	2	1	1	0	1	3	3	1
23995	101	1	2-Oct-17	12	1	9	2	1	1	0	1	3	1	1
23996	101	1	30-Aug-12	19	1	9	2	1	1	0	3	3	3	1
23997	101	1	6-Oct-12	12	1	9	2	1	1	0	1	3	3	2
23998	201	1	25-Apr-13	17	1	9	2	2	1	0	1	3	5	1
23999	201	1	20-Dec-13	10	1	9	2	1	1	0	1	3	1	2
24000	101	1	6-Mar-15	7	1	9	2	1	1	0	1	3	4	1
24001	101	1	15-Apr-15	13	1	9	2	1	1	0	1	3	3	1
24002	101	1	4-Jun-16	11	1	9	2	1	1	0	1	3	6	1
24003	101	1	28-Jul-16	19	1	9	2	1	1	0	1	3	3	1
24004	201	1	14-May-16	17	1	9	2	2	1	0	1	3	5	1
24005	201	1	26-Sep-15	10	1	9	2	3	1	0	1	3	3	2
24006	101	1	28-Jun-14	13	1	9	2	1	1	0	1	3	5	1
24007	101	1	5-Jul-13	19	1	9	2	3	1	0	1	3	1	1
24008	101	1	26-Jul-13	13	1	9	2	1	1	0	1	3	3	1
24009	101	1	25-Oct-13	18	1	9	2	1	1	0	1	3	3	1
24010	101	1	15-Dec-12	10	1	9	2	1	1	0	1	3	5	1
24011	101	1	4-Nov-12	19	1	9	2	1	1	0	1	3	1	2
24012	201	1	30-Apr-12	21	1	9	2	3	1	0	1	3	5	1
24013	201	1	6-Nov-12	16	1	9	2	1	1	0	1	3	1	2
24014	201	1	24-May-12	17	1	9	2	2	1	0	1	3	6	1
24015	201	1	5-Mar-17	14	1	9	2	2	1	0	1	3	4	1
24016	201	1	16-Apr-17	13	1	9	2	3	1	0	1	3	5	1
24017	201	1	21-Nov-14	19	1	9	2	1	1	0	4	3	2	1
24018	201	1	20-Nov-16	19	1	9	2	3	1	0	1	3	5	1
24019	201	1	14-Dec-16	9	1	9	2	1	1	0	1	3	1	2
24020	101	1	16-Jun-15	14	1	9	2	1	1	0	1	3	5	1
24021	101	1	17-May-15	8	1	9	2	1	1	0	1	3	1	1
24022	101	1	1-Mar-13	14	1	9	2	1	1	0	1	3	4	2
24023	101	1	1-Aug-12	21	1	9	2	2	1	0	1	3	4	2
24024	101	1	10-Jul-17	20	1	9	2	1	1	0	1	3	5	1
24025	101	1	1-Jun-17	21	1	9	2	3	1	0	1	3	3	2
24026	201	1	12-Dec-17	12	1	9	2	1	1	0	1	3	3	1
24027	101	1	20-Mar-15	18	1	9	2	1	1	0	1	3	3	1
24028	201	1	20-Feb-12	7	1	9	2	2	1	0	1	3	1	1
24029	201	1	30-Jul-16	19	1	9	2	1	1	0	6	3	3	1
24030	201	1	31-Oct-16	17	1	9	2	1	1	0	6	3	3	1
24031	201	1	4-Aug-15	20	1	9	2	2	1	0	6	3	3	1
24032	101	1	4-Jul-13	12	1	9	2	3	1	0	1	3	3	1
24033	101	1	7-Nov-11	7	1	9	2	1	1	0	1	3	1	1
24034	201	1	21-Feb-11	10	1	9	2	1	1	0	1	3	1	2
24035	101	1	8-Aug-11	20	1	9	2	3	1	0	1	3	4	1
24036	201	1	13-Sep-11	9	1	9	2	1	1	0	1	3	5	1
24037	201	1	11-Apr-11	13	1	9	2	1	1	0	1	3	3	1
24038	201	1	14-Oct-11	23	1	9	2	1	1	0	1	3	3	2
24039	101	1	21-May-11	9	1	9	2	1	1	0	1	3	5	1
24040	201	1	25-Jun-11	20	1	9	2	3	1	0	1	3	1	1
24041	101	1	2-Apr-11	12	1	9	2	2	1	0	1	3	1	2
24042	201	1	18-Jul-11	3	1	9	2	2	1	0	1	3	6	2
24043	101	1	8-Apr-11	15	1	9	2	1	1	0	1	3	3	1
24044	201	1	23-Jun-10	14	1	9	2	1	1	0	1	3	1	2
24045	101	1	5-Oct-10	23	1	9	2	1	1	0	1	3	1	2
24046	101	1	7-Dec-10	7	1	9	2	1	1	0	1	3	5	1
24047	101	1	3-Jul-10	19	1	9	2	1	1	0	1	3	5	1
24048	101	1	21-Mar-10	16	1	9	2	1	1	0	1	3	1	2
24049	201	1	12-Apr-10	17	1	9	2	1	1	0	1	3	1	1

24050	101	1	25-Jul-10	19	1	9	2	3	1	0	1	3	5	2
24051	201	1	21-Sep-10	15	1	9	2	1	1	0	1	3	1	1
24052	201	1	5-Dec-10	12	1	9	2	2	1	0	1	3	1	2
24053	101	1	6-Apr-10	17	1	9	2	1	1	0	1	3	3	1
24054	201	1	17-Mar-09	11	1	9	2	3	1	0	1	3	4	2
24055	201	1	23-Feb-09	20	1	9	2	1	1	0	1	3	6	1
24056	201	1	7-Feb-09	17	1	9	2	1	1	0	1	3	3	1
24057	101	1	18-Jul-09	21	1	9	2	3	1	0	1	3	4	2
24058	201	1	4-Aug-09	20	1	9	2	3	1	0	1	3	6	1
24059	101	1	22-Oct-09	18	1	9	2	2	1	0	1	3	5	1
24060	201	1	22-Oct-09	18	1	9	2	1	1	0	1	3	6	1
24061	201	1	26-Mar-09	13	1	9	2	2	1	0	1	3	1	2
24062	101	1	27-Jul-09	17	1	9	2	3	1	0	1	3	3	2
24063	101	1	25-Nov-09	22	1	9	2	1	1	0	1	3	3	1
24064	201	1	7-Oct-09	19	1	9	2	1	1	0	1	3	3	1
24065	101	1	17-Nov-08	14	1	9	2	1	1	0	1	3	4	2
24066	101	1	2-Jul-08	17	1	9	2	1	1	0	1	3	4	1
24067	101	1	2-Dec-08	19	1	9	2	1	1	0	1	3	1	1
24068	201	1	10-Feb-08	21	1	9	2	1	1	0	1	3	4	2
24069	201	1	29-Mar-08	19	1	9	2	2	1	0	1	3	4	1
24070	101	1	31-Aug-07	19	1	9	2	1	1	0	1	3	4	1
24071	201	1	21-Jun-07	21	1	9	2	1	1	0	1	3	1	1
24072	201	1	9-Sep-07	21	1	9	2	1	1	0	1	3	1	2
24073	201	1	2-Sep-07	18	1	9	2	1	1	0	1	3	3	1
24074	201	1	12-Mar-07	13	1	9	2	1	1	0	1	3	3	1
24075	101	1	15-Feb-07	16	1	9	2	1	1	0	1	3	4	1
24076	201	1	29-Dec-07	13	1	9	2	2	1	0	1	3	5	2
24077	201	1	1-Oct-07	12	1	9	2	3	1	0	1	3	5	1
24078	101	1	20-Jul-07	10	1	9	2	1	1	0	1	3	2	1
24079	101	1	7-Feb-07	18	1	9	2	1	1	0	1	3	5	2
24080	201	1	6-Dec-07	16	1	9	2	1	1	0	1	3	2	1
24081	201	1	15-Jan-07	0	1	9	2	3	1	0	1	3	4	2
24082	201	1	24-Dec-07	17	1	9	2	3	1	0	1	3	5	1
24083	101	1	30-Sep-07	17	1	9	2	1	1	0	1	3	1	1
24084	101	1	17-Oct-07	14	1	9	2	1	1	0	1	3	1	1
24085	201	1	17-Oct-07	20	1	9	2	1	1	0	1	3	3	1
24086	201	1	12-Aug-07	10	1	9	2	1	1	0	1	3	3	2
24087	101	1	31-Oct-07	17	1	9	2	3	1	0	1	3	3	2
24088	201	1	12-Jul-06	20	1	9	2	1	1	0	1	3	2	2
24089	101	1	5-May-06	11	1	9	2	1	1	0	1	3	1	1
24090	201	1	8-Dec-06	21	1	9	2	1	1	0	1	3	3	1
24091	101	1	19-Sep-06	20	1	9	2	1	1	0	1	3	1	1
24092	101	1	11-Jul-06	5	1	9	2	1	1	0	1	3	1	2
24093	201	1	5-Jun-06	21	1	9	2	1	1	0	1	3	1	1
24094	101	1	3-Jun-06	13	1	9	2	2	1	0	1	3	1	1
24095	101	1	23-Sep-06	10	1	9	2	1	1	0	1	3	4	1
24096	201	1	16-Sep-06	12	1	9	2	1	1	0	1	3	2	1
24097	101	1	13-Oct-17	12	1	9	2	1	1	0	1	3	5	1
24098	101	1	3-Apr-16	9	1	9	2	1	1	0	1	3	5	1
24099	101	1	6-Oct-12	17	1	9	2	1	1	0	1	3	5	1
24100	101	1	24-Mar-12	15	1	9	2	1	1	0	1	3	3	1
24101	201	1	23-Jul-11	13	1	9	2	1	1	0	1	3	6	2
24102	101	1	12-Nov-11	16	1	9	2	1	1	0	1	3	1	2
24103	101	1	1-Sep-11	19	1	9	2	1	1	0	1	3	3	1
24104	201	1	6-Aug-11	18	1	9	2	2	1	0	1	3	3	1
24105	101	1	21-Jul-11	22	1	9	2	1	1	0	1	3	1	1
24106	201	1	17-Nov-11	10	1	9	2	3	1	0	1	3	6	1
24107	101	1	18-Sep-10	18	1	9	2	1	1	0	1	3	2	1
24108	201	1	16-Oct-10	20	1	9	2	1	1	0	1	3	2	1
24109	101	1	10-Feb-10	19	1	9	2	1	1	0	1	3	5	1
24110	201	1	15-Oct-09	16	1	9	2	1	1	0	1	3	4	1
24111	101	1	30-Apr-09	21	1	9	2	1	1	0	1	3	1	1
24112	201	1	22-Mar-09	17	1	9	2	2	1	0	1	3	1	2
24113	101	1	23-Mar-09	13	1	9	2	1	1	0	1	3	2	2
24114	101	1	7-Jul-09	19	1	9	2	1	1	0	1	3	6	1
24115	101	1	3-Sep-09	16	1	9	2	1	1	0	1	3	5	1
24116	101	1	15-Aug-09	16	1	9	2	1	1	0	1	3	3	1
24117	201	1	20-Feb-09	11	1	9	2	1	1	0	1	3	3	1
24118	201	1	24-Jul-09	19	1	9	2	3	1	0	1	3	6	1
24119	101	1	25-Oct-08	18	1	9	2	1	1	0	1	3	1	2
24120	201	1	23-Nov-08	15	1	9	2	1	1	0	1	3	3	1
24121	101	1	20-Jul-07	20	1	9	2	2	1	0	1	3	5	1
24122	201	1	13-Aug-07	17	1	9	2	1	1	0	1	3	6	1
24123	201	1	12-Jul-07	21	1	9	2	3	1	0	1	3	1	2
24124	201	1	13-Nov-07	21	1	9	2	1	1	0	1	3	1	1
24125	201	1	6-Dec-07	12	1	9	2	1	1	0	1	3	3	1
24126	101	1	11-Apr-06	22	1	9	2	1	1	0	1	3	1	1
24127	101	1	14-Nov-06	12	1	9	2	1	1	0	1	3	3	1
24128	101	1	2-Nov-17	12	1	9	2	1	1	0	1	3	3	1
24129	101	1	19-Feb-16	23	1	9	2	1	1	0	4	3	2	1
24130	201	1	28-Nov-13	12	1	9	2	2	1	0	4	3	1	1
24131	201	1	20-Jun-12	12	1	9	2	1	1	0	4	3	1	1
24132	101	1	11-Feb-10	10	1	9	2	1	1	0	4	3	3	1
24133	101	1	1-Feb-10	23	1	9	2	1	1	0	4	3	4	1
24134	101	1	19-Sep-06	17	1	9	2	1	1	0	4	3	3	1
24135	101	1	27-Mar-16	15	1	9	2	1	1	0	11	3	3	1
24136	101	1	3-Oct-16	17	1	9	2	1	1	0	11	3	3	1
24137	101	1	18-Aug-16	8	1	9	2	1	1	0	11	3	2	1
24138	201	1	18-Nov-16	16	1	9	2	2	1	0	11	3	2	1
24139	101	1	8-Dec-16	17	1	9	2	1	1	0	11	3	3	1
24140	201	1	19-Dec-15	16	1	9	2	2	1	0	11	3	3	1
24141	101	1	25-Jul-15	14	1	9	2	1	1	0	11	3	3	1
24142	101	1	5-Feb-15	13	1	9	2	1	1	0	11	3	3	1
24143	201	1	23-Mar-15	15	1	9	2	1	1	0	11	3	2	1
24144	101	1	2-Jan-15	11	1	9	2	1	1	0	11	3	3	1
24145	201	1	6-Jun-15	19	1	9	2	2	1	0	11	3	3	1
24146	201	1	20-Nov-14	17	1	9	2	2	1	0	11	3	1	1
24147	101	1	1-Jan-14	9	1	9	2	1	1	0	11	3	2	1
24148	201	1	13-May-13	11	1	9	2	1	1	0	11	3	3	1
24149	101	1	4-Sep-12	17	1	9	2	1	1	0	11	3	2	1
24150	101	1	19-Aug-12	20	1	9	2	3	1	0	11	3	3	1
24151	101	1	18-Jul-12	22	1	9	2	1	1	0	11	3	3	1
24152	101	1	27-Feb-12	17	1	9	2	1	1	0	11	3	2	1
24153	201	1	19-Jul-11	12	1	9	2	3	1	0	11	3	3	1
24154	201	1	11-Sep-11	0	1	9	2	1	1	0	11	3	2	1
24155	201	1	16-Aug-11	19	1	9	2	1	1	0	11	3	3	1
24156	101	1	27-Jun-11	18	1	9	2	1	1	0	11	3	3	1
24157	101	1	25-Aug-10	19	1	9	2	3	1	0	11	3	2	1
24158	201	1	3-May-10	18	1	9	2	2	1	0	11	3	3	1
24159	101	1	13-May-09	17	1	9	2	1	1	0	11	3	3	1
24160	101	1	13-May-09	9	1	9	2	1	1	0	11	3	3	1
24161	201	1	3-Dec-08	15	1	9	2	3	1	0	11	3	3	1
24162	101	1	15-Aug-08	9	1	9	2	1	1	0	11	3	3	1
24163	201	1	19-Dec-08	20	1	9	2	3	1	0	11	3	3	1
24164	201	1	28-Oct-07	16	1	9	2	1	1	0	11	3	3	1
24165	101	1	12-Mar-06	12	1	9	2	1	1	0	11	3	2	1
24166	201	1	11-Feb-17	19	1	9	2	1	1	0	11	3	3	1
24167	101	1	17-Jan-17	10	1	9	2	1	1	0	11	3	2	1
24168	201	1	2-Mar-17	16	1	9	2	2	1	0	11	3	3	1
24169	101	1	12-Jul-16	13	1	9	2	1	1	0	3	3	1	4
24170	101	1	3-Nov-16	16	1	9	2	1	1	0	3	3	1	1
24171	101	1	11-Nov-16	14	1	9	2	1	1	0	3	3	2	1
24172	201	1	26-Nov-16	9	1	9	2	1	1	0	3	3	2	1
24173	201	1	23-Sep-16	19	1	9	2	1	1	0	3	3	2	1

24174	201	1	15-Jul-16	19	1	9	2	1	1	0	3	3	2	1
24175	101	1	13-Sep-15	19	1	9	2	1	1	0	3	3	4	1
24176	201	1	31-Dec-15	16	1	9	2	2	1	0	3	3	4	1
24177	201	1	6-Nov-15	19	1	9	2	1	1	0	3	3	1	1
24178	101	1	11-May-15	20	1	9	2	1	1	0	3	3	1	1
24179	201	1	25-Oct-15	9	1	9	2	1	1	0	3	3	1	1
24180	101	1	29-May-15	12	1	9	2	1	1	0	3	3	1	1
24181	101	1	19-Nov-15	7	1	9	2	1	1	0	3	3	1	1
24182	201	1	14-Nov-15	13	1	9	2	1	1	0	3	3	1	1
24183	201	1	21-Oct-15	17	1	9	2	2	1	0	3	3	1	1
24184	201	1	3-Oct-15	23	1	9	2	2	1	0	3	3	2	2
24185	201	1	31-Dec-15	15	1	9	2	1	1	0	3	3	3	1
24186	101	1	17-Dec-15	19	1	9	2	1	1	0	3	3	3	1
24187	101	1	8-May-15	11	1	9	2	1	1	0	3	3	2	1
24188	201	1	28-Jun-15	21	1	9	2	1	1	0	3	3	3	1
24189	201	1	7-May-15	16	1	9	2	2	1	0	3	3	2	1
24190	101	1	8-Oct-15	20	1	9	2	1	1	0	3	3	3	1
24191	101	1	25-Jan-15	16	1	9	2	1	1	0	3	3	3	1
24192	201	1	18-Jul-15	14	1	9	2	1	1	0	3	3	2	1
24193	201	1	23-Apr-14	19	1	9	2	1	1	0	3	3	5	1
24194	201	1	4-Jan-14	12	1	9	2	2	1	0	3	3	1	1
24195	101	1	7-May-14	14	1	9	2	1	1	0	3	3	1	1
24196	201	1	3-Mar-14	12	1	9	2	2	1	0	3	3	1	1
24197	101	1	12-May-14	20	1	9	2	1	1	0	3	3	1	1
24198	201	1	1-Oct-14	18	1	9	2	1	1	0	3	3	4	1
24199	201	1	6-Jun-14	9	1	9	2	1	1	0	3	3	4	1
24200	101	1	1-Dec-14	14	1	9	2	1	1	0	3	3	3	1
24201	101	1	26-Jun-14	19	1	9	2	1	1	0	3	3	2	1
24202	201	1	26-Sep-14	21	1	9	2	2	1	0	3	3	1	1
24203	101	1	8-Aug-14	15	1	9	2	2	1	0	3	3	3	1
24204	201	1	1-Oct-14	16	1	9	2	1	1	0	3	3	3	1
24205	201	1	22-Nov-13	20	1	9	2	1	1	0	3	3	1	1
24206	101	1	11-Oct-13	12	1	9	2	2	1	0	3	3	3	1
24207	201	1	29-Oct-13	9	1	9	2	2	1	0	3	3	5	1
24208	201	1	7-Oct-13	22	1	9	2	1	1	0	3	3	3	1
24209	201	1	14-Jan-13	7	1	9	2	1	1	0	3	3	3	1
24210	201	1	16-Nov-13	8	1	9	2	3	1	0	3	3	1	1
24211	201	1	9-May-12	11	1	9	2	1	1	0	3	3	3	1
24212	201	1	31-Oct-11	8	1	9	2	1	1	0	3	3	2	1
24213	201	1	4-Jun-11	15	1	9	2	1	1	0	3	3	2	1
24214	201	1	16-Jul-10	21	1	9	2	1	1	0	3	3	1	1
24215	101	1	28-Aug-10	22	1	9	2	1	1	0	3	3	3	1
24216	201	1	10-Apr-10	20	1	9	2	1	1	0	3	3	2	2
24217	201	1	1-May-10	21	1	9	2	1	1	0	3	3	3	1
24218	101	1	17-Mar-10	11	1	9	2	1	1	0	3	3	3	1
24219	101	1	24-Mar-10	20	1	9	2	1	1	0	3	3	2	1
24220	101	1	10-Feb-09	11	1	9	2	1	1	0	3	3	1	2
24221	201	1	11-Dec-09	13	1	9	2	1	1	0	3	3	1	1
24222	101	1	30-Mar-09	18	1	9	2	2	1	0	3	3	1	1
24223	201	1	16-Jul-09	19	1	9	2	1	1	0	3	3	3	1
24224	101	1	3-May-09	9	1	9	2	1	1	0	3	3	2	1
24225	201	1	13-Jul-09	14	1	9	2	1	1	0	3	3	3	1
24226	201	1	22-Jul-09	18	1	9	2	1	1	0	3	3	2	1
24227	201	1	19-Mar-09	17	1	9	2	1	1	0	3	3	3	1
24228	201	1	21-Mar-09	20	1	9	2	1	1	0	3	3	3	1
24229	201	1	16-Apr-08	16	1	9	2	1	1	0	3	3	1	1
24230	201	1	5-May-08	17	1	9	2	1	1	0	3	3	3	1
24231	201	1	9-May-08	17	1	9	2	1	1	0	3	3	1	1
24232	101	1	17-Dec-08	12	1	9	2	1	1	0	3	3	1	1
24233	201	1	25-Jan-08	12	1	9	2	3	1	0	3	3	1	1
24234	201	1	23-May-08	9	1	9	2	1	1	0	3	3	3	1
24235	201	1	7-Jun-08	21	1	9	2	1	1	0	3	3	4	1
24236	201	1	26-Dec-07	13	1	9	2	1	1	0	3	3	1	1
24237	101	1	2-Jul-07	18	1	9	2	1	1	0	3	3	5	1
24238	101	1	5-Dec-07	14	1	9	2	1	1	0	3	3	1	1
24239	201	1	26-Sep-07	18	1	9	2	3	1	0	3	3	4	1
24240	101	1	23-Oct-07	21	1	9	2	1	1	0	3	3	2	1
24241	101	1	11-Nov-07	21	1	9	2	1	1	0	3	3	3	1
24242	101	1	23-May-06	7	1	9	2	1	1	0	3	3	2	1
24243	201	1	31-Mar-06	18	1	9	2	1	1	0	3	3	1	1
24244	201	1	9-Aug-06	23	1	9	2	2	1	0	3	3	1	1
24245	201	1	25-Jul-06	17	1	9	2	2	1	0	3	3	3	1
24246	201	1	21-Jul-06	19	1	9	2	1	1	0	3	3	2	1
24247	201	1	23-Feb-06	19	1	9	2	1	1	0	3	3	1	1
24248	101	1	9-Mar-06	10	1	9	2	1	1	0	3	3	1	1
24249	201	1	28-Dec-06	12	1	9	2	1	1	0	3	3	2	1
24250	101	1	4-Mar-17	18	1	9	2	1	1	0	3	3	4	1
24251	201	1	20-Jun-17	12	1	9	2	1	1	0	3	3	2	1
24252	201	1	17-Jun-17	21	1	9	2	1	1	0	3	3	2	1
24253	101	1	1-Jan-17	11	1	9	2	1	1	0	3	3	1	1
24254	201	1	21-Apr-17	12	1	9	2	1	1	0	3	3	1	1
24255	201	1	10-Jun-17	17	1	9	2	1	1	0	3	3	1	1
24256	201	1	30-Sep-17	15	1	9	2	2	1	0	3	3	3	1
24257	201	1	16-Jun-17	18	1	9	2	2	1	0	3	3	2	1
24258	101	1	17-Sep-17	13	1	9	2	1	1	0	3	3	1	1
24259	101	1	29-Jul-15	16	1	9	2	1	1	0	6	3	3	1
24260	201	1	29-Jul-15	20	1	9	2	1	1	0	6	3	3	1
24261	101	1	23-Apr-14	17	1	9	2	1	1	0	6	3	3	1
24262	101	1	22-Sep-13	15	1	9	2	2	1	0	6	3	3	1
24263	101	1	5-Aug-13	16	1	9	2	2	1	0	6	3	3	1
24264	101	1	27-Feb-10	19	1	9	2	1	1	0	6	3	3	1
24265	101	1	19-Apr-10	14	1	9	2	3	1	0	6	3	3	1
24266	101	1	16-Feb-10	11	1	9	2	1	1	0	6	3	3	1
24267	201	1	18-Oct-08	19	1	9	2	2	1	0	6	3	3	1
24268	101	1	18-Oct-07	6	1	9	2	1	1	0	6	3	3	1
24269	101	1	14-Mar-17	16	1	9	2	1	1	0	13	3	3	1
24270	201	1	18-Jul-15	20	2	9	2	1	1	0	1	3	1	1
24271	201	1	1-Apr-13	14	2	9	2	1	1	0	1	3	5	1
24272	201	1	27-Aug-12	21	2	9	2	3	1	0	1	3	6	1
24273	201	1	22-Sep-17	20	2	9	2	3	1	0	1	3	3	1
24274	201	1	22-Sep-17	20	2	9	2	2	1	0	6	3	3	1
24275	201	1	29-Apr-15	12	2	9	2	1	1	0	1	3	1	1
24276	201	1	16-Apr-15	11	2	9	2	2	1	0	1	3	4	1
24277	101	1	13-Dec-13	22	2	9	2	1	1	0	1	3	1	1
24278	201	1	16-Mar-13	18	2	9	2	1	1	0	1	3	3	1
24279	201	1	16-Mar-13	20	2	9	2	1	1	0	1	3	3	1
24280	201	1	5-Nov-17	12	2	9	2	1	1	0	1	3	5	1
24281	201	1	13-Sep-17	18	2	9	2	1	1	0	1	3	3	1
24282	201	1	27-May-16	16	2	9	2	2	1	0	1	3	5	1
24283	101	1	6-Nov-12	17	2	9	2	1	1	0	1	3	3	1
24284	101	1	6-Jul-12	1	2	9	2	1	1	0	1	3	3	2
24285	201	1	25-Jun-15	20	2	9	2	2	1	0	1	3	5	1
24286	201	1	4-Nov-14	10	2	9	2	2	1	0	1	3	1	1
24287	201	1	29-Jul-14	12	2	9	2	3	1	0	1	3	1	2
24288	101	1	8-Aug-12	17	2	9	2	1	1	0	3	3	1	2
24289	201	1	16-May-16	11	2	9	2	2	1	0	3	3	5	1
24290	201	1	18-Aug-15	11	2	9	2	1	1	0	6	3	3	1
24291	101	1	13-Sep-09	13	2	9	2	1	1	0	13	3	2	1
24292	101	1	14-May-15	7	2	9	2	1	1	0	1	3	3	1
24293	101	1	7-Nov-13	12	2	9	2	1	1	0	1	3	3	1
24294	101	1	24-Dec-11	11	2	9	2	1	1	0	1	3	5	1
24295	101	1	28-Sep-11	10	2	9	2	1	1	0	1	3	1	1
24296	201	1	12-Oct-11	4	2	9	2	2	1	0	1	3	3	1
24297	201	1	12-Mar-11	21	2	9	2	2	1	0	1	3	3	1

24298	201	1	9-Oct-10	19	2	9	2	1	1	0	1	3	3	2
24299	101	1	3-Dec-10	18	2	9	2	1	1	0	1	3	1	2
24300	101	1	4-May-10	12	2	9	2	1	1	0	1	3	6	1
24301	101	1	28-Jul-10	17	2	9	2	1	1	0	1	3	1	1
24302	201	1	10-Mar-10	11	2	9	2	1	1	0	1	3	3	1
24303	201	1	10-Feb-10	19	2	9	2	2	1	0	1	3	2	2
24304	201	1	24-Jul-10	16	2	9	2	2	1	0	1	3	3	1
24305	201	1	20-Nov-09	14	2	9	2	3	1	0	1	3	5	1
24306	201	1	25-Oct-09	16	2	9	2	3	1	0	1	3	2	1
24307	201	1	26-Mar-09	19	2	9	2	1	1	0	1	3	5	1
24308	101	1	17-Apr-09	7	2	9	2	1	1	0	1	3	3	1
24309	101	1	8-Nov-09	10	2	9	2	3	1	0	1	3	3	1
24310	101	1	28-Dec-09	12	2	9	2	1	1	0	1	3	3	2
24311	201	1	27-Jul-08	11	2	9	2	3	1	0	1	3	1	1
24312	101	1	17-Sep-07	14	2	9	2	1	1	0	1	3	3	1
24313	201	1	30-Mar-07	9	2	9	2	1	1	0	1	3	1	1
24314	201	1	16-Jan-07	12	2	9	2	1	1	0	1	3	1	1
24315	201	1	28-Mar-07	20	2	9	2	1	1	0	1	3	1	2
24316	201	1	26-Nov-07	8	2	9	2	1	1	0	1	3	5	1
24317	201	1	8-Oct-07	17	2	9	2	2	1	0	1	3	1	1
24318	101	1	19-May-07	12	2	9	2	3	1	0	1	3	3	2
24319	101	1	9-Oct-07	12	2	9	2	1	1	0	1	3	2	1
24320	101	1	12-Jan-07	9	2	9	2	1	1	0	1	3	4	2
24321	201	1	14-Oct-07	11	2	9	2	2	1	0	1	3	3	1
24322	101	1	6-May-06	14	2	9	2	1	1	0	1	3	1	1
24323	101	1	28-Jul-06	23	2	9	2	3	1	0	1	3	1	1
24324	201	1	7-Jul-11	20	2	9	2	2	1	0	1	3	3	1
24325	101	1	10-Feb-10	8	2	9	2	1	1	0	1	3	3	1
24326	101	1	7-Aug-09	13	2	9	2	3	1	0	1	3	3	1
24327	101	1	20-Oct-09	15	2	9	2	1	1	0	1	3	3	1
24328	101	1	22-May-09	19	2	9	2	1	1	0	1	3	3	1
24329	101	1	3-Apr-09	20	2	9	2	1	1	0	1	3	1	1
24330	101	1	16-May-08	19	2	9	2	1	1	0	1	3	1	2
24331	101	1	19-Jul-07	15	2	9	2	1	1	0	1	3	1	2
24332	201	1	13-Feb-07	12	2	9	2	1	1	0	1	3	3	1
24333	201	1	31-Aug-07	13	2	9	2	1	1	0	1	3	3	1
24334	201	1	23-Mar-06	19	2	9	2	2	1	0	1	3	3	1
24335	201	1	24-Dec-06	18	2	9	2	1	1	0	1	3	3	1
24336	101	1	8-May-11	20	2	9	2	1	1	0	4	3	2	1
24337	201	1	15-Jul-08	22	2	9	2	3	1	0	4	3	7	1
24338	101	1	2-Jun-08	17	2	9	2	1	1	0	4	3	3	1
24339	201	1	6-Aug-07	18	2	9	2	1	1	0	4	3	3	1
24340	101	1	16-Nov-17	9	2	9	2	1	1	0	4	3	2	1
24341	101	1	22-Jan-15	12	2	9	2	1	1	0	11	3	2	1
24342	101	1	20-May-13	18	2	9	2	3	1	0	11	3	3	1
24343	201	1	10-Jul-12	7	2	9	2	3	1	0	11	3	3	1
24344	201	1	28-Jul-12	10	2	9	2	2	1	0	11	3	3	1
24345	101	1	19-Mar-12	17	2	9	2	1	1	0	11	3	2	1
24346	101	1	7-Feb-12	16	2	9	2	1	1	0	11	3	2	1
24347	201	1	12-May-12	19	2	9	2	1	1	0	11	3	3	1
24348	201	1	25-Aug-11	15	2	9	2	1	1	0	11	3	3	1
24349	201	1	29-Mar-11	7	2	9	2	1	1	0	11	3	2	1
24350	201	1	5-Sep-10	17	2	9	2	1	1	0	11	3	2	1
24351	201	1	6-Jul-10	14	2	9	2	2	1	0	11	3	3	1
24352	101	1	22-Sep-08	17	2	9	2	1	1	0	11	3	3	1
24353	201	1	7-May-17	18	2	9	2	1	1	0	11	3	3	1
24354	201	1	1-Nov-17	12	2	9	2	1	1	0	11	3	2	1
24355	101	1	13-Oct-16	17	2	9	2	1	1	0	3	3	5	1
24356	201	1	16-Jul-16	23	2	9	2	2	1	0	3	3	1	1
24357	201	1	28-May-16	16	2	9	2	1	1	0	3	3	3	1
24358	201	1	15-Jul-16	17	2	9	2	1	1	0	3	3	3	1
24359	101	1	6-Dec-16	19	2	9	2	1	1	0	3	3	2	2
24360	201	1	16-Nov-16	17	2	9	2	1	1	0	3	3	1	1
24361	101	1	21-Nov-15	16	2	9	2	1	1	0	3	3	3	1
24362	201	1	30-Jan-15	13	2	9	2	1	1	0	3	3	1	1
24363	201	1	16-Jul-15	12	2	9	2	1	1	0	3	3	1	1
24364	101	1	27-Jun-15	15	2	9	2	1	1	0	3	3	3	1
24365	101	1	10-Jul-15	17	2	9	2	1	1	0	3	3	3	1
24366	101	1	12-Mar-15	12	2	9	2	1	1	0	3	3	1	1
24367	101	1	26-Feb-14	17	2	9	2	1	1	0	3	3	1	1
24368	201	1	14-May-14	14	2	9	2	2	1	0	3	3	3	1
24369	201	1	30-Aug-13	14	2	9	2	3	1	0	3	3	2	1
24370	101	1	1-Jan-12	16	2	9	2	1	1	0	3	3	1	2
24371	201	1	29-Aug-12	20	2	9	2	1	1	0	3	3	3	2
24372	101	1	31-May-12	7	2	9	2	1	1	0	3	3	3	1
24373	101	1	2-Oct-12	17	2	9	2	1	1	0	3	3	3	1
24374	201	1	1-Jun-12	17	2	9	2	3	1	0	3	3	3	1
24375	101	1	7-Aug-11	14	2	9	2	1	1	0	3	3	5	1
24376	101	1	20-May-11	7	2	9	2	1	1	0	3	3	5	1
24377	101	1	8-Sep-11	17	2	9	2	1	1	0	3	3	3	1
24378	201	1	20-Jul-11	13	2	9	2	3	1	0	3	3	2	2
24379	101	1	22-Mar-10	16	2	9	2	1	1	0	3	3	2	1
24380	201	1	9-Jul-10	11	2	9	2	1	1	0	3	3	3	1
24381	201	1	21-Aug-10	16	2	9	2	3	1	0	3	3	3	1
24382	201	1	4-Aug-10	20	2	9	2	2	1	0	3	3	1	1
24383	201	1	9-Sep-10	0	2	9	2	3	1	0	3	3	3	1
24384	201	1	4-Aug-10	20	2	9	2	2	1	0	3	3	3	1
24385	101	1	30-Oct-09	12	2	9	2	1	1	0	3	3	3	1
24386	101	1	17-Jul-09	21	2	9	2	2	1	0	3	3	3	4
24387	201	1	10-Apr-08	16	2	9	2	1	1	0	3	3	1	1
24388	101	1	16-May-08	17	2	9	2	1	1	0	3	3	2	1
24389	101	1	19-Jul-07	15	2	9	2	1	1	0	3	3	3	1
24390	101	1	19-Mar-07	12	2	9	2	1	1	0	3	3	1	1
24391	101	1	30-Oct-07	17	2	9	2	1	1	0	3	3	3	1
24392	201	1	22-Jun-06	18	2	9	2	1	1	0	3	3	1	1
24393	201	1	22-Feb-06	17	2	9	2	1	1	0	3	3	3	1
24394	101	1	24-May-06	8	2	9	2	1	1	0	3	3	3	1
24395	201	1	18-Mar-06	18	2	9	2	2	1	0	3	3	1	1
24396	101	1	16-Apr-17	20	2	9	2	1	1	0	3	3	7	1
24397	101	1	2-Jan-17	9	2	9	2	1	1	0	3	3	2	1
24398	201	1	23-May-17	17	2	9	2	1	1	0	3	3	2	1
24399	101	1	5-Mar-17	19	2	9	2	1	1	0	3	3	1	1
24400	101	1	25-Sep-17	14	2	9	2	1	1	0	3	3	4	1
24401	201	1	23-Dec-17	19	2	9	2	1	1	0	3	3	2	1
24402	101	1	14-Nov-15	20	2	9	2	1	1	0	6	3	3	1
24403	201	1	13-May-13	17	2	9	2	2	1	0	6	3	3	1
24404	101	1	27-Oct-12	18	2	9	2	1	1	0	6	3	3	1
24405	101	1	7-Aug-08	22	2	9	2	1	1	0	6	3	1	1
24406	202	0	13-Aug-15	17	1	10	2	1	1	0	1	3	1	1
24407	202	0	10-Dec-16	19	1	10	2	1	1	0	1	3	1	1
24408	202	0	28-Jul-15	17	1	10	2	3	1	0	1	3	3	1
24409	202	0	23-Oct-13	19	1	10	2	3	1	0	1	3	3	1
24410	202	0	23-Aug-12	12	1	10	2	1	1	0	1	3	3	2
24411	202	0	22-Dec-15	16	1	10	2	3	1	0	1	3	3	1
24412	202	0	24-Sep-17	12	1	10	2	2	1	0	1	3	3	1
24413	202	0	21-Apr-13	19	1	10	2	3	1	0	1	3	1	1
24414	202	0	28-Apr-12	13	1	10	2	1	1	0	1	3	5	2
24415	202	0	4-Apr-16	10	1	10	2	1	1	0	4	3	1	1
24416	202	0	20-Apr-17	7	1	10	2	2	1	0	1	3	3	2
24417	202	0	19-Apr-14	15	1	10	2	4	1	0	1	3	4	1
24418	202	0	9-Oct-17	17	1	10	2	2	1	0	1	3	3	1
24419	202	0	22-Oct-17	11	1	10	2	3	1	0	1	3	4	1
24420	202	0	2-May-16	16	1	10	2	3	1	0	1	3	4	1
24421	202	0	25-Oct-16	13	1	10	2	1	1	0	1	3	3	1

24422	202	0	25-Aug-17	11	1	10	2	2	1	0	1	3	1	2
24423	202	0	14-Dec-15	7	1	10	2	1	1	0	1	3	3	1
24424	202	0	30-Oct-13	13	1	10	2	1	1	0	1	3	5	1
24425	202	0	27-Nov-16	19	1	10	2	1	1	0	1	3	1	1
24426	202	0	10-Apr-16	15	1	10	2	3	1	0	1	3	1	1
24427	202	0	20-Jul-16	17	1	10	2	3	1	0	1	3	4	2
24428	202	0	5-Aug-16	20	1	10	2	2	1	0	1	3	4	1
24429	202	0	11-Jul-16	16	1	10	2	2	1	0	1	3	3	1
24430	202	0	5-Jul-16	11	1	10	2	2	1	0	1	3	5	1
24431	202	0	8-Aug-16	7	1	10	2	1	1	0	1	3	3	1
24432	202	0	16-Aug-15	16	1	10	2	1	1	0	1	3	1	2
24433	202	0	20-Jan-14	20	1	10	2	1	1	0	1	3	5	1
24434	202	0	30-Apr-14	19	1	10	2	2	1	0	1	3	3	2
24435	202	0	4-Aug-14	0	1	10	2	2	1	0	1	3	1	2
24436	202	0	25-Dec-14	11	1	10	2	1	1	0	1	3	5	1
24437	202	0	24-Aug-14	19	1	10	2	3	1	0	1	3	3	1
24438	202	0	21-Jan-13	11	1	10	2	1	1	0	1	3	3	1
24439	202	0	2-Feb-13	17	1	10	2	3	1	0	1	3	3	2
24440	202	0	13-Jul-13	22	1	10	2	1	1	0	1	3	2	1
24441	202	0	4-Jun-13	18	1	10	2	2	1	0	1	3	3	2
24442	202	0	24-May-13	19	1	10	2	1	1	0	1	3	3	1
24443	202	0	10-Oct-13	20	1	10	2	1	1	0	1	3	1	1
24444	202	0	17-Mar-13	9	1	10	2	3	1	0	1	3	1	2
24445	202	0	29-Jul-13	18	1	10	2	3	1	0	1	3	4	1
24446	202	0	20-Oct-17	12	1	10	2	1	1	0	1	3	6	1
24447	202	0	27-Oct-17	12	1	10	2	1	1	0	1	3	1	1
24448	202	0	14-May-17	13	1	10	2	2	1	0	1	3	5	1
24449	202	0	18-Jul-15	15	1	10	2	1	1	0	13	3	1	1
24450	202	0	5-May-06	8	1	10	2	1	1	0	13	3	2	2
24451	202	0	11-Feb-13	14	1	10	2	3	1	0	1	3	3	1
24452	202	0	8-May-12	7	1	10	2	1	1	0	1	3	6	2
24453	202	0	10-Apr-11	13	1	10	2	1	1	0	1	3	6	1
24454	202	0	16-Dec-11	21	1	10	2	3	1	0	1	3	5	2
24455	202	0	17-Apr-11	9	1	10	2	1	1	0	1	3	6	2
24456	202	0	17-Jul-11	19	1	10	2	1	1	0	1	3	3	1
24457	202	0	5-Jan-11	12	1	10	2	3	1	0	1	3	3	2
24458	202	0	26-Aug-11	22	1	10	2	1	1	0	1	3	3	1
24459	202	0	17-Feb-10	22	1	10	2	1	1	0	1	3	1	2
24460	202	0	10-Apr-10	11	1	10	2	1	1	0	1	3	3	2
24461	202	0	15-Oct-10	16	1	10	2	1	1	0	1	3	5	1
24462	202	0	16-Jun-10	18	1	10	2	3	1	0	1	3	6	1
24463	202	0	8-Nov-09	9	1	10	2	1	1	0	1	3	3	2
24464	202	0	12-Apr-08	6	1	10	2	1	1	0	1	3	6	1
24465	202	0	9-Sep-08	16	1	10	2	1	1	0	1	3	3	1
24466	202	0	27-Jan-08	20	1	10	2	1	1	0	1	3	1	2
24467	202	0	2-Aug-08	9	1	10	2	3	1	0	1	3	1	2
24468	202	0	9-May-08	8	1	10	2	1	1	0	1	3	1	1
24469	202	0	15-Jun-08	19	1	10	2	2	1	0	1	3	3	1
24470	202	0	6-Jul-07	15	1	10	2	1	1	0	1	3	1	1
24471	202	0	4-Mar-07	9	1	10	2	1	1	0	1	3	6	1
24472	202	0	19-Feb-07	17	1	10	2	1	1	0	1	3	5	1
24473	202	0	25-Sep-07	19	1	10	2	1	1	0	1	3	1	1
24474	202	0	25-Sep-07	17	1	10	2	1	1	0	1	3	4	1
24475	202	0	18-Jul-07	23	1	10	2	2	1	0	1	3	2	2
24476	202	0	28-Aug-07	18	1	10	2	1	1	0	1	3	6	2
24477	202	0	9-Jul-07	14	1	10	2	1	1	0	1	3	1	2
24478	202	0	3-Apr-07	18	1	10	2	1	1	0	1	3	3	2
24479	202	0	30-Jan-06	14	1	10	2	1	1	0	1	3	1	1
24480	202	0	1-Oct-06	11	1	10	2	1	1	0	1	3	3	1
24481	202	0	15-Oct-06	23	1	10	2	3	1	0	1	3	3	1
24482	202	0	3-Jun-06	17	1	10	2	1	1	0	1	3	3	1
24483	202	0	3-Sep-10	11	1	10	2	1	1	0	1	3	1	2
24484	202	0	5-Oct-07	11	1	10	2	1	1	0	1	3	3	1
24485	202	0	2-Jan-06	12	1	10	2	1	1	0	1	3	1	1
24486	202	0	27-Jul-16	17	1	10	2	3	1	0	4	3	3	1
24487	202	0	19-Aug-14	14	1	10	2	1	1	0	4	3	1	1
24488	202	0	5-Jan-09	14	1	10	2	3	1	0	4	3	2	2
24489	202	0	24-Aug-17	9	1	10	2	2	1	0	4	3	1	2
24490	202	0	25-Oct-17	0	1	10	2	1	1	0	4	3	4	1
24491	202	0	21-Apr-17	17	1	10	2	1	1	0	4	3	3	1
24492	202	0	16-Feb-16	20	1	10	2	3	1	0	11	3	3	1
24493	202	0	2-Jun-15	8	1	10	2	1	1	0	11	3	1	1
24494	202	0	26-Nov-07	13	1	10	2	1	1	0	11	3	3	1
24495	202	0	3-Jun-06	17	1	10	2	1	1	0	11	3	3	1
24496	202	0	23-Sep-17	15	1	10	2	2	1	0	11	3	2	1
24497	202	0	2-Oct-16	22	1	10	2	3	1	0	3	3	5	1
24498	202	0	3-Oct-16	12	1	10	2	1	1	0	3	3	3	1
24499	202	0	23-Dec-14	11	1	10	2	1	1	0	3	3	2	2
24500	202	0	22-Oct-14	17	1	10	2	3	1	0	3	3	1	1
24501	202	0	25-May-14	15	1	10	2	1	1	0	3	3	2	1
24502	202	0	13-Sep-14	20	1	10	2	3	1	0	3	3	3	1
24503	202	0	6-Dec-14	19	1	10	2	3	1	0	3	3	2	1
24504	202	0	22-Nov-13	20	1	10	2	1	1	0	3	3	5	1
24505	202	0	18-Aug-13	18	1	10	2	1	1	0	3	3	3	1
24506	202	0	28-Dec-13	10	1	10	2	3	1	0	3	3	2	1
24507	202	0	19-Oct-13	19	1	10	2	1	1	0	3	3	2	2
24508	202	0	30-Oct-13	13	1	10	2	3	1	0	3	3	3	1
24509	202	0	18-May-13	13	1	10	2	3	1	0	3	3	3	1
24510	202	0	11-Jun-11	9	1	10	2	1	1	0	3	3	1	2
24511	202	0	23-Apr-10	17	1	10	2	1	1	0	3	3	1	2
24512	202	0	24-May-10	17	1	10	2	3	1	0	3	3	5	1
24513	202	0	25-May-10	11	1	10	2	1	1	0	3	3	3	1
24514	202	0	10-Mar-10	16	1	10	2	3	1	0	3	3	2	1
24515	202	0	3-May-10	17	1	10	2	2	1	0	3	3	2	2
24516	202	0	1-Jun-09	17	1	10	2	1	1	0	3	3	6	1
24517	202	0	11-Dec-09	12	1	10	2	1	1	0	3	3	6	1
24518	202	0	24-Oct-09	17	1	10	2	3	1	0	3	3	2	2
24519	202	0	6-Nov-09	19	1	10	2	1	1	0	3	3	7	1
24520	202	0	20-Jul-09	11	1	10	2	1	1	0	3	3	2	1
24521	202	0	21-Oct-08	20	1	10	2	1	1	0	3	3	1	1
24522	202	0	11-Apr-08	17	1	10	2	1	1	0	3	3	1	1
24523	202	0	22-May-07	17	1	10	2	1	1	0	3	3	1	1
24524	202	0	22-Feb-06	14	1	10	2	1	1	0	3	3	1	1
24525	202	0	5-Jul-06	12	1	10	2	1	1	0	3	3	3	1
24526	202	0	23-Nov-06	11	1	10	2	1	1	0	3	3	3	1
24527	202	0	22-Jul-16	19	1	10	2	3	1	0	6	3	3	1
24528	202	0	24-Jul-09	21	1	10	2	1	1	0	6	3	3	1
24529	202	0	14-Feb-07	9	1	10	2	1	1	0	6	3	2	1
24530	202	0	18-Nov-17	11	2	10	2	3	1	0	1	3	3	1
24531	202	0	7-Jun-13	14	2	10	2	1	1	0	1	3	1	1
24532	202	0	22-Apr-16	15	2	10	2	3	1	0	1	3	3	1
24533	202	0	30-Jan-16	10	2	10	2	1	1	0	1	3	3	1
24534	202	0	23-Sep-16	12	2	10	2	1	1	0	1	3	5	2
24535	202	0	8-Sep-17	0	2	10	2	1	1	0	1	3	3	1
24536	202	0	6-Jun-15	13	2	10	2	1	1	0	1	3	2	2
24537	202	0	9-Oct-14	12	2	10	2	1	1	0	1	3	3	1
24538	202	0	6-Jul-12	1	2	10	2	1	1	0	1	3	3	2
24539	202	0	17-Sep-12	12	2	10	2	1	1	0	1	3	1	2
24540	202	0	25-Nov-14	17	2	10	2	3	1	0	1	3	2	1
24541	202	0	30-Jun-15	12	2	10	2	1	1	0	1	3	5	1
24542	202	0	18-Jul-16	8	2	10	2	1	1	0	1	3	5	2
24543	202	0	28-Oct-15	13	2	10	2	2	1	0	1	3	1	1
24544	202	0	26-Jun-15	13	2	10	2	1	1	0	1	3	3	1
24545	202	0	6-Oct-15	14	2	10	2	3	1	0	1	3	2	1

24546	202	0	10-Aug-14	21	2	10	2	1	1	0	1	3	1	1
24547	202	0	5-May-14	18	2	10	2	3	1	0	1	3	2	1
24548	202	0	9-Jul-14	8	2	10	2	1	1	0	1	3	5	1
24549	202	0	7-May-13	17	2	10	2	1	1	0	1	3	4	2
24550	202	0	11-Mar-13	14	2	10	2	1	1	0	1	3	3	1
24551	202	0	1-Aug-12	20	2	10	2	1	1	0	1	3	3	1
24552	202	0	29-Nov-12	8	2	10	2	1	1	0	1	3	3	1
24553	202	0	29-Nov-12	8	2	10	2	3	1	0	1	3	3	2
24554	202	0	19-Sep-17	17	2	10	2	1	1	0	1	3	1	1
24555	202	0	15-Jun-14	15	2	10	2	1	1	0	6	3	2	1
24556	202	0	6-May-17	22	2	10	2	1	1	0	7	3	3	1
24557	202	0	18-Aug-08	20	2	10	2	1	1	0	7	3	2	1
24558	202	0	23-Mar-09	20	2	10	2	1	1	0	13	3	1	1
24559	202	0	22-May-11	21	2	10	2	1	1	0	1	3	5	1
24560	202	0	19-Jan-11	18	2	10	2	1	1	0	1	3	1	1
24561	202	0	30-May-11	20	2	10	2	3	1	0	1	3	1	1
24562	202	0	29-Mar-11	15	2	10	2	1	1	0	1	3	3	2
24563	202	0	30-Aug-11	2	2	10	2	1	1	0	1	3	6	2
24564	202	0	5-Aug-11	18	2	10	2	2	1	0	1	3	1	1
24565	202	0	4-Jul-10	15	2	10	2	1	1	0	1	3	6	2
24566	202	0	28-Dec-10	20	2	10	2	1	1	0	1	3	1	1
24567	202	0	25-May-10	14	2	10	2	3	1	0	1	3	3	2
24568	202	0	11-Dec-09	12	2	10	2	1	1	0	1	3	6	1
24569	202	0	17-Nov-09	12	2	10	2	1	1	0	1	3	1	1
24570	202	0	29-May-08	16	2	10	2	1	1	0	1	3	6	1
24571	202	0	29-May-08	4	2	10	2	1	1	0	1	3	2	1
24572	202	0	19-Jul-08	21	2	10	2	2	1	0	1	3	1	1
24573	202	0	24-Mar-08	17	2	10	2	1	1	0	1	3	3	1
24574	202	0	15-Nov-07	17	2	10	2	1	1	0	1	3	6	2
24575	202	0	11-Jun-07	12	2	10	2	3	1	0	1	3	1	2
24576	202	0	15-Nov-07	17	2	10	2	1	1	0	1	3	1	1
24577	202	0	17-Sep-07	7	2	10	2	1	1	0	1	3	1	2
24578	202	0	13-Nov-07	17	2	10	2	1	1	0	1	3	1	1
24579	202	0	25-Jul-07	8	2	10	2	1	1	0	1	3	4	1
24580	202	0	27-Sep-07	17	2	10	2	1	1	0	1	3	4	1
24581	202	0	20-May-06	15	2	10	2	2	1	0	1	3	1	2
24582	202	0	7-Jul-06	12	2	10	2	1	1	0	1	3	1	1
24583	202	0	15-Jan-06	16	2	10	2	1	1	0	1	3	1	1
24584	202	0	16-Aug-06	21	2	10	2	1	1	0	1	3	2	2
24585	202	0	4-Mar-13	8	2	10	2	1	1	0	1	3	5	1
24586	202	0	3-Jun-10	17	2	10	2	1	1	0	1	3	1	1
24587	202	0	29-Aug-08	11	2	10	2	2	1	0	1	3	2	2
24588	202	0	26-May-06	17	2	10	2	1	1	0	1	3	1	1
24589	202	0	31-Oct-12	19	2	10	2	3	1	0	4	3	3	1
24590	202	0	16-May-11	13	2	10	2	1	1	0	11	3	2	1
24591	202	0	16-Sep-11	18	2	10	2	1	1	0	11	3	3	2
24592	202	0	9-Jun-11	12	2	10	2	1	1	0	11	3	3	2
24593	202	0	30-Jul-17	18	2	10	2	1	1	0	11	3	5	1
24594	202	0	5-Sep-16	13	2	10	2	2	1	0	3	3	3	1
24595	202	0	22-Oct-15	9	2	10	2	1	1	0	3	3	2	1
24596	202	0	5-Jan-14	11	2	10	2	1	1	0	3	3	2	1
24597	202	0	29-Apr-12	18	2	10	2	1	1	0	3	3	5	1
24598	202	0	14-Aug-11	16	2	10	2	1	1	0	3	3	3	1
24599	202	0	29-Mar-10	13	2	10	2	1	1	0	3	3	3	1
24600	202	0	6-Aug-10	16	2	10	2	1	1	0	3	3	3	1
24601	202	0	16-Apr-08	18	2	10	2	1	1	0	3	3	7	1
24602	202	0	24-Jan-08	18	2	10	2	3	1	0	3	3	7	1
24603	202	0	30-May-17	0	2	10	2	1	1	0	3	3	4	1
24604	202	0	1-Jul-17	14	2	10	2	1	1	0	3	3	3	1
24605	201	1	20-Oct-15	19	1	10	2	2	1	0	1	3	4	1
24606	201	1	9-Sep-14	11	1	10	2	2	1	0	1	3	1	1
24607	201	1	18-Nov-17	18	1	10	2	1	1	0	1	3	4	1
24608	201	1	29-Oct-17	19	1	10	2	1	1	0	1	3	3	1
24609	101	1	31-Oct-17	16	1	10	2	1	1	0	1	3	5	2
24610	201	1	15-Dec-14	17	1	10	2	1	1	0	1	3	1	2
24611	101	1	13-Aug-13	12	1	10	2	2	1	0	1	3	5	1
24612	101	1	17-Dec-16	13	1	10	2	1	1	0	1	3	5	1
24613	201	1	22-Sep-16	17	1	10	2	1	1	0	1	3	5	2
24614	101	1	1-Jun-15	21	1	10	2	1	1	0	1	3	2	1
24615	201	1	5-Feb-14	14	1	10	2	1	1	0	1	3	3	1
24616	201	1	14-Nov-14	20	1	10	2	1	1	0	1	3	1	2
24617	201	1	25-Mar-14	11	1	10	2	2	1	0	1	3	4	1
24618	201	1	22-May-14	12	1	10	2	1	1	0	1	3	2	2
24619	201	1	4-Mar-13	14	1	10	2	2	1	0	1	3	5	1
24620	201	1	18-Mar-13	12	1	10	2	1	1	0	1	3	1	1
24621	101	1	9-Mar-12	18	1	10	2	1	1	0	1	3	1	1
24622	201	1	22-Apr-12	18	1	10	2	2	1	0	1	3	6	2
24623	201	1	22-Sep-17	18	1	10	2	1	1	0	1	3	5	1
24624	201	1	1-Nov-17	17	1	10	2	1	1	0	1	3	5	1
24625	101	1	28-Apr-17	22	1	10	2	1	1	0	1	3	1	1
24626	201	1	21-Oct-17	9	1	10	2	3	1	0	1	3	5	1
24627	201	1	20-Jul-14	13	1	10	2	3	1	0	1	3	5	1
24628	201	1	20-May-14	13	1	10	2	1	1	0	1	3	5	1
24629	201	1	27-Sep-12	16	1	10	2	3	1	0	1	3	6	1
24630	101	1	7-Jul-14	17	1	10	2	1	1	0	1	3	3	1
24631	101	1	26-Dec-13	16	1	10	2	1	1	0	1	3	3	1
24632	201	1	1-Jul-16	21	1	10	2	2	1	0	1	3	5	2
24633	201	1	25-Mar-14	16	1	10	2	1	1	0	1	3	1	1
24634	201	1	27-Mar-13	16	1	10	2	1	1	0	1	3	1	2
24635	201	1	21-Nov-13	17	1	10	2	3	1	0	1	3	5	1
24636	201	1	19-Dec-13	22	1	10	2	1	1	0	1	3	1	1
24637	201	1	11-Jun-12	12	1	10	2	2	1	0	1	3	1	1
24638	101	1	9-Oct-17	21	1	10	2	1	1	0	1	3	3	2
24639	101	1	27-Jul-13	13	1	10	2	1	1	0	1	3	1	2
24640	201	1	20-Oct-15	7	1	10	2	1	1	0	1	3	4	1
24641	101	1	25-May-13	8	1	10	2	1	1	0	1	3	3	1
24642	101	1	9-Aug-12	18	1	10	2	1	1	0	1	3	1	2
24643	201	1	12-Aug-17	14	1	10	2	1	1	0	1	3	2	1
24644	201	1	9-Mar-14	20	1	10	2	2	1	0	6	3	3	1
24645	101	1	19-Jul-08	20	1	10	2	1	1	0	13	3	3	1
24646	101	1	30-Nov-13	12	1	10	2	1	1	0	1	3	5	1
24647	201	1	16-Mar-11	19	1	10	2	2	1	0	1	3	1	1
24648	201	1	5-May-11	16	1	10	2	1	1	0	1	3	1	1
24649	201	1	3-Nov-11	21	1	10	2	1	1	0	1	3	3	2
24650	101	1	6-May-11	19	1	10	2	1	1	0	1	3	5	1
24651	101	1	15-Mar-11	7	1	10	2	1	1	0	1	3	5	1
24652	101	1	31-Oct-11	12	1	10	2	1	1	0	1	3	4	1
24653	201	1	21-Jul-11	8	1	10	2	2	1	0	1	3	1	1
24654	201	1	4-Jan-10	14	1	10	2	1	1	0	1	3	1	2
24655	101	1	5-Jul-10	9	1	10	2	1	1	0	1	3	5	1
24656	201	1	15-Jan-10	19	1	10	2	1	1	0	1	3	1	1
24657	101	1	29-Apr-10	17	1	10	2	1	1	0	1	3	1	1
24658	201	1	1-Jan-10	22	1	10	2	2	1	0	1	3	2	1
24659	101	1	5-Dec-10	13	1	10	2	1	1	0	1	3	3	1
24660	201	1	11-Mar-10	12	1	10	2	2	1	0	1	3	3	1
24661	201	1	20-Mar-10	21	1	10	2	2	1	0	1	3	3	1
24662	101	1	13-Feb-10	16	1	10	2	1	1	0	1	3	3	1
24663	101	1	7-Dec-10	12	1	10	2	1	1	0	1	3	4	1
24664	201	1	7-Nov-10	22	1	10	2	1	1	0	1	3	4	1
24665	101	1	27-Nov-10	17	1	10	2	1	1	0	1	3	4	1
24666	101	1	19-Mar-10	12	1	10	2	1	1	0	1	3	3	1
24667	201	1	9-Feb-10	0	1	10	2	2	1	0	1	3	2	1
24668	201	1	26-Jan-10	18	1	10	2	2	1	0	1	3	3	1
24669	201	1	28-Jul-09	16	1	10	2	3	1	0	1	3	1	2

24670	101	1	24-Sep-09	17	1	10	2	1	1	0	1	3	4	1
24671	201	1	2-Sep-09	7	1	10	2	1	1	0	1	3	1	2
24672	201	1	1-Mar-09	7	1	10	2	1	1	0	1	3	1	1
24673	101	1	11-Oct-09	11	1	10	2	1	1	0	1	3	1	2
24674	201	1	5-Nov-09	17	1	10	2	1	1	0	1	3	5	2
24675	201	1	13-Nov-09	15	1	10	2	1	1	0	1	3	2	1
24676	101	1	5-Jun-09	17	1	10	2	1	1	0	1	3	3	1
24677	201	1	2-May-09	19	1	10	2	3	1	0	1	3	1	2
24678	101	1	5-Sep-08	18	1	10	2	1	1	0	1	3	5	1
24679	101	1	16-Nov-08	14	1	10	2	1	1	0	1	3	1	1
24680	101	1	4-Mar-08	17	1	10	2	1	1	0	1	3	1	1
24681	201	1	13-Aug-08	23	1	10	2	2	1	0	1	3	5	1
24682	201	1	20-Jul-08	17	1	10	2	1	1	0	1	3	1	1
24683	101	1	20-Jul-08	16	1	10	2	1	1	0	1	3	4	2
24684	101	1	24-Jul-08	16	1	10	2	1	1	0	1	3	1	1
24685	101	1	1-Aug-08	15	1	10	2	1	1	0	1	3	1	2
24686	101	1	8-Sep-08	19	1	10	2	1	1	0	1	3	1	2
24687	201	1	8-Sep-08	19	1	10	2	3	1	0	1	3	1	2
24688	201	1	16-Mar-07	18	1	10	2	1	1	0	1	3	6	1
24689	101	1	6-Sep-07	17	1	10	2	1	1	0	1	3	3	1
24690	101	1	26-Apr-07	8	1	10	2	1	1	0	1	3	3	2
24691	201	1	16-Feb-07	9	1	10	2	2	1	0	1	3	5	1
24692	101	1	27-Aug-07	19	1	10	2	1	1	0	1	3	1	1
24693	101	1	20-Dec-07	9	1	10	2	1	1	0	1	3	3	2
24694	101	1	17-Jun-06	23	1	10	2	1	1	0	1	3	1	1
24695	101	1	17-Jul-06	15	1	10	2	1	1	0	1	3	6	1
24696	101	1	27-Apr-06	7	1	10	2	1	1	0	1	3	3	1
24697	101	1	1-Nov-06	18	1	10	2	1	1	0	1	3	1	1
24698	201	1	8-Aug-06	20	1	10	2	2	1	0	1	3	5	1
24699	201	1	26-Aug-06	19	1	10	2	1	1	0	1	3	3	1
24700	101	1	24-Dec-06	17	1	10	2	1	1	0	1	3	3	1
24701	101	1	14-Mar-06	18	1	10	2	1	1	0	1	3	3	1
24702	101	1	27-Aug-06	15	1	10	2	1	1	0	1	3	3	2
24703	201	1	1-Apr-06	12	1	10	2	1	1	0	1	3	2	1
24704	101	1	5-Mar-06	15	1	10	2	1	1	0	1	3	3	1
24705	101	1	14-Mar-17	19	1	10	2	1	1	0	1	3	3	2
24706	201	1	3-Aug-07	22	1	10	2	3	1	0	5	3	3	1
24707	101	1	11-Jun-11	8	1	10	2	1	1	0	1	3	1	2
24708	201	1	5-Jun-11	10	1	10	2	1	1	0	1	3	3	1
24709	101	1	16-Sep-10	14	1	10	2	1	1	0	1	3	7	1
24710	101	1	17-Apr-10	16	1	10	2	1	1	0	1	3	1	2
24711	101	1	4-May-10	7	1	10	2	1	1	0	1	3	5	1
24712	201	1	27-Sep-09	17	1	10	2	2	1	0	1	3	1	2
24713	201	1	14-Jun-09	22	1	10	2	1	1	0	1	3	2	1
24714	101	1	8-Jul-09	19	1	10	2	1	1	0	1	3	3	1
24715	101	1	19-Jan-08	20	1	10	2	1	1	0	1	3	2	1
24716	201	1	4-Aug-07	14	1	10	2	1	1	0	1	3	6	1
24717	201	1	11-Jul-07	18	1	10	2	1	1	0	1	3	1	1
24718	201	1	9-Nov-07	19	1	10	2	3	1	0	1	3	4	1
24719	101	1	5-Jun-07	17	1	10	2	1	1	0	1	3	2	1
24720	201	1	11-Sep-07	19	1	10	2	1	1	0	1	3	3	1
24721	101	1	23-Jul-07	9	1	10	2	1	1	0	1	3	3	1
24722	201	1	22-Sep-07	8	1	10	2	1	1	0	1	3	1	1
24723	101	1	28-Jul-07	18	1	10	2	1	1	0	1	3	3	1
24724	101	1	19-Aug-07	21	1	10	2	1	1	0	1	3	1	1
24725	201	1	2-Sep-07	21	1	10	2	3	1	0	1	3	1	1
24726	101	1	27-Oct-07	9	1	10	2	1	1	0	1	3	2	1
24727	201	1	18-Sep-06	22	1	10	2	1	1	0	1	3	3	1
24728	101	1	27-Aug-06	16	1	10	2	1	1	0	1	3	3	1
24729	101	1	19-Jul-06	11	1	10	2	3	1	0	1	3	3	1
24730	101	1	7-Aug-06	17	1	10	2	1	1	0	1	3	3	1
24731	101	1	18-Oct-06	15	1	10	2	1	1	0	1	3	3	1
24732	101	1	24-Feb-15	23	1	10	2	1	1	0	4	3	1	2
24733	201	1	10-Sep-10	18	1	10	2	1	1	0	4	3	3	1
24734	201	1	28-Mar-08	20	1	10	2	2	1	0	4	3	2	1
24735	101	1	5-Nov-16	16	1	10	2	1	1	0	11	3	3	1
24736	201	1	18-Jul-16	16	1	10	2	1	1	0	11	3	2	1
24737	101	1	2-Dec-16	20	1	10	2	1	1	0	11	3	2	1
24738	201	1	19-Jul-16	0	1	10	2	1	1	0	11	3	3	1
24739	101	1	1-Jun-15	18	1	10	2	1	1	0	11	3	2	1
24740	101	1	13-Sep-14	17	1	10	2	1	1	0	11	3	3	1
24741	201	1	22-May-14	7	1	10	2	1	1	0	11	3	3	1
24742	101	1	9-Jul-14	12	1	10	2	1	1	0	11	3	3	1
24743	201	1	9-Aug-12	16	1	10	2	2	1	0	11	3	3	1
24744	101	1	2-Jul-12	16	1	10	2	3	1	0	11	3	3	1
24745	201	1	26-Oct-11	11	1	10	2	1	1	0	11	3	2	1
24746	101	1	5-Oct-11	12	1	10	2	1	1	0	11	3	2	1
24747	201	1	26-Dec-11	11	1	10	2	1	1	0	11	3	2	1
24748	101	1	18-May-11	12	1	10	2	1	1	0	11	3	3	1
24749	201	1	1-Feb-11	17	1	10	2	1	1	0	11	3	2	1
24750	201	1	25-Aug-11	17	1	10	2	1	1	0	11	3	3	1
24751	201	1	24-Oct-10	7	1	10	2	1	1	0	11	3	3	1
24752	201	1	13-Aug-10	14	1	10	2	3	1	0	11	3	5	1
24753	101	1	2-Jan-10	14	1	10	2	2	1	0	11	3	3	1
24754	101	1	4-Mar-10	21	1	10	2	1	1	0	11	3	3	1
24755	201	1	7-Jan-10	16	1	10	2	2	1	0	11	3	4	1
24756	201	1	2-Jan-09	17	1	10	2	2	1	0	11	3	2	1
24757	201	1	29-Oct-09	9	1	10	2	1	1	0	11	3	3	1
24758	101	1	8-Apr-09	17	1	10	2	1	1	0	11	3	2	1
24759	201	1	22-Sep-09	17	1	10	2	1	1	0	11	3	2	1
24760	201	1	22-Feb-09	17	1	10	2	3	1	0	11	3	2	1
24761	201	1	9-Aug-07	20	1	10	2	2	1	0	11	3	3	1
24762	201	1	22-Sep-06	12	1	10	2	1	1	0	11	3	3	1
24763	201	1	21-Jun-06	19	1	10	2	1	1	0	11	3	3	1
24764	101	1	7-Feb-17	19	1	10	2	1	1	0	11	3	2	1
24765	101	1	26-Mar-17	8	1	10	2	1	1	0	11	3	2	1
24766	101	1	12-Feb-17	20	1	10	2	1	1	0	11	3	2	1
24767	101	1	13-Feb-16	16	1	10	2	1	1	0	3	3	2	1
24768	201	1	25-Mar-16	15	1	10	2	1	1	0	3	3	3	1
24769	101	1	15-May-16	18	1	10	2	1	1	0	3	3	1	1
24770	101	1	12-Nov-16	15	1	10	2	1	1	0	3	3	5	4
24771	201	1	8-May-16	17	1	10	2	1	1	0	3	3	3	1
24772	101	1	6-Jul-15	14	1	10	2	1	1	0	3	3	2	4
24773	101	1	25-May-15	19	1	10	2	1	1	0	3	3	2	1
24774	201	1	19-Nov-15	7	1	10	2	1	1	0	3	3	1	1
24775	201	1	16-Sep-15	17	1	10	2	1	1	0	3	3	1	1
24776	201	1	6-Nov-15	18	1	10	2	1	1	0	3	3	1	1
24777	101	1	23-Jun-15	13	1	10	2	1	1	0	3	3	1	1
24778	101	1	17-Mar-15	11	1	10	2	1	1	0	3	3	3	1
24779	101	1	22-Oct-15	11	1	10	2	1	1	0	3	3	2	1
24780	201	1	13-May-15	17	1	10	2	1	1	0	3	3	2	1
24781	101	1	28-Apr-15	9	1	10	2	1	1	0	3	3	4	1
24782	201	1	7-Apr-15	18	1	10	2	2	1	0	3	3	1	1
24783	101	1	19-Jun-14	19	1	10	2	1	1	0	3	3	3	1
24784	201	1	27-Feb-14	11	1	10	2	1	1	0	3	3	4	1
24785	201	1	17-Jul-14	16	1	10	2	1	1	0	3	3	3	1
24786	201	1	9-Nov-14	16	1	10	2	1	1	0	3	3	1	1
24787	101	1	17-Apr-14	19	1	10	2	1	1	0	3	3	2	4
24788	101	1	7-Oct-14	14	1	10	2	1	1	0	3	3	2	2
24789	101	1	8-May-14	18	1	10	2	1	1	0	3	3	4	2
24790	201	1	24-Mar-13	11	1	10	2	2	1	0	3	3	2	1
24791	101	1	25-Dec-13	10	1	10	2	1	1	0	3	3	1	1
24792	101	1	14-Nov-13	16	1	10	2	1	1	0	3	3	3	4
24793	201	1	8-Oct-13	13	1	10	2	2	1	0	3	3	1	1

24794	101	1	17-Oct-13	17	1	10	2	1	1	0	3	3	2	4
24795	101	1	28-Mar-13	14	1	10	2	1	1	0	3	3	2	1
24796	101	1	30-Aug-12	21	1	10	2	1	1	0	3	3	5	1
24797	201	1	24-Mar-12	19	1	10	2	3	1	0	3	3	4	1
24798	201	1	6-Oct-12	14	1	10	2	2	1	0	3	3	3	1
24799	201	1	17-Dec-12	14	1	10	2	2	1	0	3	3	1	1
24800	201	1	17-Sep-12	12	1	10	2	3	1	0	3	3	4	1
24801	201	1	8-Jun-12	9	1	10	2	2	1	0	3	3	3	1
24802	201	1	14-Jun-12	13	1	10	2	2	1	0	3	3	3	1
24803	201	1	1-May-12	10	1	10	2	2	1	0	3	3	3	1
24804	101	1	14-May-11	11	1	10	2	1	1	0	3	3	5	1
24805	201	1	30-Jan-11	17	1	10	2	1	1	0	3	3	2	1
24806	101	1	25-Jun-11	17	1	10	2	1	1	0	3	3	1	1
24807	201	1	25-Oct-11	8	1	10	2	2	1	0	3	3	1	1
24808	101	1	30-Jun-11	10	1	10	2	1	1	0	3	3	1	1
24809	201	1	26-May-11	17	1	10	2	1	1	0	3	3	3	1
24810	201	1	17-Aug-11	15	1	10	2	2	1	0	3	3	3	2
24811	201	1	19-Feb-11	20	1	10	2	2	1	0	3	3	3	1
24812	201	1	25-May-10	17	1	10	2	2	1	0	3	3	1	1
24813	101	1	12-Oct-10	7	1	10	2	1	1	0	3	3	2	1
24814	101	1	23-May-10	19	1	10	2	1	1	0	3	3	3	1
24815	201	1	28-Aug-10	0	1	10	2	1	1	0	3	3	2	1
24816	201	1	9-Jul-10	21	1	10	2	1	1	0	3	3	2	2
24817	101	1	6-Mar-10	14	1	10	2	1	1	0	3	3	3	1
24818	201	1	26-Jun-10	7	1	10	2	1	1	0	3	3	3	1
24819	101	1	22-May-09	15	1	10	2	1	1	0	3	3	3	1
24820	201	1	31-Jan-09	10	1	10	2	2	1	0	3	3	1	1
24821	201	1	12-Jul-09	21	1	10	2	1	1	0	3	3	1	1
24822	101	1	3-Jun-09	17	1	10	2	1	1	0	3	3	3	1
24823	201	1	17-Oct-09	17	1	10	2	1	1	0	3	3	3	1
24824	101	1	4-Aug-09	18	1	10	2	1	1	0	3	3	2	1
24825	101	1	6-Sep-09	18	1	10	2	1	1	0	3	3	2	1
24826	101	1	8-Nov-09	18	1	10	2	1	1	0	3	3	2	1
24827	101	1	27-Dec-09	17	1	10	2	1	1	0	3	3	2	1
24828	201	1	21-Oct-08	21	1	10	2	2	1	0	3	3	1	1
24829	101	1	18-May-08	18	1	10	2	2	1	0	3	3	1	2
24830	101	1	18-Jan-08	17	1	10	2	1	1	0	3	3	4	2
24831	201	1	9-Nov-08	20	1	10	2	1	1	0	3	3	1	1
24832	101	1	20-Feb-07	17	1	10	2	2	1	0	3	3	3	1
24833	101	1	24-Nov-07	14	1	10	2	1	1	0	3	3	2	2
24834	101	1	17-Jan-07	23	1	10	2	3	1	0	3	3	3	2
24835	201	1	27-Jun-07	7	1	10	2	1	1	0	3	3	1	1
24836	201	1	1-Apr-07	8	1	10	2	1	1	0	3	3	3	1
24837	101	1	3-Oct-07	21	1	10	2	1	1	0	3	3	2	1
24838	101	1	20-May-07	17	1	10	2	1	1	0	3	3	2	1
24839	101	1	19-Nov-06	19	1	10	2	1	1	0	3	3	2	1
24840	101	1	20-May-06	17	1	10	2	1	1	0	3	3	1	1
24841	201	1	15-Mar-06	13	1	10	2	1	1	0	3	3	1	2
24842	201	1	10-Mar-06	16	1	10	2	1	1	0	3	3	1	1
24843	201	1	2-Dec-06	22	1	10	2	2	1	0	3	3	2	2
24844	101	1	4-Jul-06	19	1	10	2	1	1	0	3	3	3	1
24845	101	1	6-Oct-06	9	1	10	2	1	1	0	3	3	2	1
24846	101	1	18-Dec-06	21	1	10	2	1	1	0	3	3	2	1
24847	101	1	15-Jun-17	16	1	10	2	1	1	0	3	3	4	1
24848	101	1	31-Aug-17	21	1	10	2	1	1	0	3	3	3	1
24849	101	1	4-Sep-17	12	1	10	2	1	1	0	3	3	3	1
24850	101	1	31-May-17	14	1	10	2	1	1	0	3	3	3	1
24851	101	1	5-Jan-17	11	1	10	2	1	1	0	3	3	2	1
24852	201	1	11-May-17	20	1	10	2	1	1	0	3	3	2	1
24853	101	1	12-Jun-17	17	1	10	2	1	1	0	3	3	1	1
24854	101	1	21-Mar-17	16	1	10	2	1	1	0	3	3	1	1
24855	201	1	7-Nov-17	18	1	10	2	2	1	0	3	3	1	1
24856	101	1	15-Sep-17	17	1	10	2	1	1	0	3	3	3	1
24857	101	1	18-Oct-17	13	1	10	2	1	1	0	3	3	3	1
24858	101	1	17-Sep-17	11	1	10	2	1	1	0	3	3	2	1
24859	101	1	31-Oct-17	17	1	10	2	1	1	0	3	3	2	1
24860	201	1	14-Dec-17	16	1	10	2	3	1	0	3	3	2	1
24861	101	1	19-Jul-13	10	1	10	2	1	1	0	6	3	3	1
24862	101	1	10-Jul-13	17	1	10	2	3	1	0	6	3	3	1
24863	201	1	14-Jun-11	17	1	10	2	1	1	0	6	3	2	1
24864	201	1	12-Aug-10	16	1	10	2	1	1	0	6	3	3	1
24865	101	1	15-Jul-10	16	1	10	2	3	1	0	6	3	3	1
24866	101	1	31-Aug-06	19	1	10	2	1	1	0	6	3	3	1
24867	101	1	28-Mar-17	13	1	10	2	1	1	0	6	3	2	1
24868	101	1	18-Nov-17	11	1	10	2	1	1	0	6	3	3	1
24869	101	1	5-Nov-17	19	1	10	2	1	1	0	13	3	2	1
24870	101	1	10-Nov-17	17	1	10	2	1	1	0	13	3	4	2
24871	201	1	8-Oct-15	7	2	10	2	1	1	0	1	3	1	1
24872	101	1	18-Mar-16	16	2	10	2	1	1	0	1	3	5	1
24873	201	1	23-May-13	16	2	10	2	2	1	0	1	3	5	1
24874	201	1	22-Sep-12	18	2	10	2	3	1	0	1	3	5	1
24875	201	1	10-Sep-12	13	2	10	2	3	1	0	1	3	1	1
24876	201	1	11-Oct-16	17	2	10	2	2	1	0	1	3	5	1
24877	201	1	3-Sep-16	14	2	10	2	3	1	0	1	3	2	1
24878	101	1	11-Jun-16	9	2	10	2	1	1	0	1	3	3	1
24879	101	1	27-Mar-15	19	2	10	2	1	1	0	3	3	2	1
24880	201	1	12-May-16	16	2	10	2	2	1	0	1	3	1	1
24881	201	1	3-Feb-13	18	2	10	2	2	1	0	1	3	5	1
24882	201	1	1-Dec-12	20	2	10	2	2	1	0	1	3	5	2
24883	201	1	28-Dec-12	18	2	10	2	2	1	0	1	3	4	1
24884	101	1	17-May-15	18	2	10	2	3	1	0	1	3	5	1
24885	101	1	13-Oct-12	7	2	10	2	3	1	0	1	3	5	1
24886	201	1	30-Jan-14	19	2	10	2	2	1	0	7	3	1	1
24887	201	1	29-May-12	12	2	10	2	1	1	0	6	3	3	1
24888	201	1	5-Jul-12	17	2	10	2	2	1	0	7	3	3	1
24889	101	1	5-Apr-11	15	2	10	2	1	1	0	7	3	2	1
24890	101	1	11-Aug-11	22	2	10	2	1	1	0	7	3	3	1
24891	201	1	16-Aug-11	18	2	10	2	1	1	0	1	3	1	2
24892	101	1	1-Nov-11	22	2	10	2	1	1	0	1	3	3	2
24893	201	1	2-Mar-11	4	2	10	2	1	1	0	1	3	2	1
24894	201	1	25-Jan-11	13	2	10	2	1	1	0	1	3	4	1
24895	101	1	14-Aug-11	18	2	10	2	1	1	0	1	3	2	2
24896	101	1	16-Dec-11	14	2	10	2	1	1	0	1	3	3	2
24897	201	1	6-Jul-10	15	2	10	2	3	1	0	1	3	6	1
24898	201	1	10-May-10	14	2	10	2	1	1	0	1	3	1	1
24899	201	1	3-Oct-10	10	2	10	2	2	1	0	1	3	1	1
24900	201	1	17-Apr-10	1	2	10	2	3	1	0	1	3	3	1
24901	101	1	16-Apr-09	21	2	10	2	2	1	0	1	3	3	1
24902	201	1	10-May-09	12	2	10	2	2	1	0	1	3	1	1
24903	201	1	24-Nov-09	16	2	10	2	1	1	0	1	3	4	1
24904	101	1	22-Mar-09	17	2	10	2	1	1	0	1	3	3	1
24905	101	1	26-Mar-09	18	2	10	2	1	1	0	1	3	4	1
24906	101	1	19-Nov-09	13	2	10	2	1	1	0	1	3	3	2
24907	101	1	12-Mar-08	8	2	10	2	1	1	0	1	3	1	1
24908	101	1	24-Jan-08	22	2	10	2	1	1	0	1	3	1	1
24909	201	1	3-Jan-08	18	2	10	2	1	1	0	1	3	3	1
24910	101	1	29-May-08	19	2	10	2	1	1	0	1	3	1	2
24911	201	1	26-Oct-08	16	2	10	2	2	1	0	1	3	3	1
24912	201	1	14-Sep-07	13	2	10	2	1	1	0	1	3	1	2
24913	201	1	24-May-07	14	2	10	2	1	1	0	1	3	6	2
24914	201	1	9-Oct-07	19	2	10	2	1	1	0	1	3	2	2
24915	101	1	1-Sep-07	9	2	10	2	2	1	0	1	3	2	1
24916	201	1	8-Oct-07	10	2	10	2	3	1	0	1	3	1	2
24917	201	1	1-Jan-07	9	2	10	2	3	1	0	1	3	1	1

24918	101	1	12-Jan-07	21	2	10	2	1	1	0	1	3	3	2
24919	101	1	14-Sep-07	12	2	10	2	1	1	0	1	3	3	1
24920	101	1	29-Jan-06	10	2	10	2	2	1	0	1	3	1	2
24921	101	1	2-May-06	17	2	10	2	1	1	0	1	3	5	1
24922	201	1	4-Mar-06	14	2	10	2	3	1	0	1	3	1	2
24923	101	1	24-Nov-17	11	2	10	2	1	1	0	1	3	7	1
24924	101	1	4-Jul-17	14	2	10	2	1	1	0	1	3	3	2
24925	101	1	30-Oct-13	18	2	10	2	1	1	0	1	3	1	1
24926	101	1	24-Apr-11	12	2	10	2	1	1	0	1	3	1	4
24927	101	1	23-Aug-10	16	2	10	2	1	1	0	1	3	2	1
24928	101	1	11-Dec-10	22	2	10	2	1	1	0	1	3	1	1
24929	101	1	6-Feb-09	17	2	10	2	1	1	0	1	3	1	1
24930	101	1	14-Mar-09	17	2	10	2	1	1	0	1	3	2	1
24931	101	1	17-Apr-09	18	2	10	2	1	1	0	1	3	5	1
24932	101	1	9-Apr-08	16	2	10	2	1	1	0	1	3	3	1
24933	101	1	16-Jan-07	20	2	10	2	1	1	0	1	3	2	1
24934	201	1	16-Nov-06	14	2	10	2	3	1	0	1	3	2	2
24935	201	1	17-Oct-06	20	2	10	2	2	1	0	1	3	2	1
24936	101	1	30-Dec-10	17	2	10	2	1	1	0	4	3	1	1
24937	201	1	21-Jun-09	8	2	10	2	1	1	0	4	3	3	1
24938	201	1	19-May-08	10	2	10	2	3	1	0	4	3	3	1
24939	101	1	24-Oct-08	21	2	10	2	1	1	0	4	3	2	1
24940	101	1	2-Sep-07	13	2	10	2	1	1	0	4	3	2	1
24941	201	1	30-Jan-06	10	2	10	2	1	1	0	4	3	3	1
24942	201	1	14-Dec-16	7	2	10	2	1	1	0	11	3	2	1
24943	101	1	16-May-16	19	2	10	2	1	1	0	11	3	2	1
24944	101	1	21-Nov-15	11	2	10	2	1	1	0	11	3	3	1
24945	101	1	13-Oct-13	0	2	10	2	1	1	0	11	3	3	1
24946	101	1	19-Jul-12	17	2	10	2	3	1	0	11	3	3	1
24947	101	1	28-Apr-12	17	2	10	2	1	1	0	11	3	2	1
24948	201	1	18-Aug-11	0	2	10	2	1	1	0	11	3	2	1
24949	201	1	19-Jun-11	21	2	10	2	1	1	0	11	3	2	1
24950	101	1	29-Jul-11	18	2	10	2	1	1	0	11	3	3	1
24951	201	1	4-Apr-11	20	2	10	2	1	1	0	11	3	3	1
24952	101	1	3-Feb-10	16	2	10	2	1	1	0	11	3	2	1
24953	201	1	6-Jun-10	13	2	10	2	2	1	0	11	3	2	1
24954	101	1	3-Jan-10	20	2	10	2	1	1	0	11	3	3	1
24955	201	1	22-Jul-09	17	2	10	2	1	1	0	11	3	3	1
24956	101	1	16-Oct-09	17	2	10	2	1	1	0	11	3	3	1
24957	101	1	26-Apr-07	12	2	10	2	1	1	0	11	3	3	1
24958	101	1	3-Apr-17	15	2	10	2	1	1	0	11	3	3	1
24959	101	1	14-Feb-16	15	2	10	2	1	1	0	3	3	3	2
24960	201	1	26-May-16	15	2	10	2	1	1	0	3	3	2	1
24961	201	1	6-Nov-16	10	2	10	2	2	1	0	3	3	3	1
24962	201	1	23-Nov-15	14	2	10	2	2	1	0	3	3	2	1
24963	101	1	11-Sep-15	7	2	10	2	1	1	0	3	3	3	1
24964	201	1	7-May-15	13	2	10	2	2	1	0	3	3	3	1
24965	201	1	26-May-14	15	2	10	2	2	1	0	3	3	3	1
24966	101	1	17-May-13	17	2	10	2	1	1	0	3	3	1	1
24967	201	1	22-Jul-13	6	2	10	2	1	1	0	3	3	3	1
24968	101	1	26-Sep-13	17	2	10	2	1	1	0	3	3	1	1
24969	101	1	10-May-12	7	2	10	2	1	1	0	3	3	1	1
24970	101	1	19-Apr-11	21	2	10	2	1	1	0	3	3	1	1
24971	201	1	9-Aug-11	23	2	10	2	2	1	0	3	3	1	1
24972	101	1	10-Aug-11	19	2	10	2	1	1	0	3	3	3	1
24973	201	1	16-Aug-11	11	2	10	2	2	1	0	3	3	1	2
24974	201	1	2-Feb-10	17	2	10	2	2	1	0	3	3	3	1
24975	201	1	20-Feb-10	19	2	10	2	2	1	0	3	3	2	1
24976	101	1	23-Jun-09	19	2	10	2	1	1	0	3	3	2	1
24977	201	1	14-Jul-09	20	2	10	2	3	1	0	3	3	1	1
24978	101	1	9-Jun-08	17	2	10	2	1	1	0	3	3	1	1
24979	101	1	28-Sep-08	20	2	10	2	1	1	0	3	3	2	1
24980	201	1	22-Dec-07	22	2	10	2	2	1	0	3	3	2	1
24981	201	1	1-Sep-06	21	2	10	2	1	1	0	3	3	3	1
24982	201	1	10-May-17	16	2	10	2	1	1	0	3	3	3	1
24983	201	1	26-Apr-17	11	2	10	2	1	1	0	3	3	1	1
24984	101	1	13-Dec-17	12	2	10	2	1	1	0	3	3	3	1
24985	101	1	14-Aug-16	13	2	10	2	1	1	0	6	3	2	1
24986	202	0	24-Nov-13	15	1	11	2	1	1	0	1	3	2	2
24987	202	0	21-Oct-12	18	1	11	2	1	1	0	1	3	2	2
24988	202	0	23-Oct-17	9	1	11	2	1	1	0	1	3	4	1
24989	202	0	27-Nov-13	11	1	11	2	1	1	0	1	3	3	1
24990	202	0	19-Aug-16	18	1	11	2	3	1	0	1	3	5	1
24991	202	0	27-May-15	11	1	11	2	1	1	0	1	3	3	1
24992	202	0	14-Feb-16	4	1	11	2	3	1	0	1	3	1	1
24993	202	0	15-Feb-16	17	1	11	2	2	1	0	1	3	3	2
24994	202	0	14-May-17	23	1	11	2	1	1	0	1	3	3	1
24995	202	0	7-Jun-16	19	1	11	2	1	1	0	1	3	3	1
24996	202	0	10-Sep-12	12	1	11	2	1	1	0	1	3	3	1
24997	202	0	26-Apr-16	12	1	11	2	3	1	0	1	3	6	1
24998	202	0	16-Jun-16	17	1	11	2	1	1	0	1	3	1	1
24999	202	0	7-Jun-14	17	1	11	2	1	1	0	1	3	2	1
25000	202	0	11-Jun-14	20	1	11	2	3	1	0	1	3	4	1
25001	202	0	27-Dec-14	8	1	11	2	3	1	0	1	3	3	1
25002	202	0	22-Jan-13	17	1	11	2	3	1	0	1	3	3	1
25003	202	0	27-Sep-13	23	1	11	2	1	1	0	1	3	1	2
25004	202	0	28-May-13	16	1	11	2	3	1	0	1	3	4	1
25005	202	0	22-Mar-12	15	1	11	2	1	1	0	1	3	5	1
25006	202	0	28-Jul-12	17	1	11	2	3	1	0	1	3	6	2
25007	202	0	14-May-17	16	1	11	2	1	1	0	1	3	3	1
25008	202	0	9-Nov-16	12	1	11	2	2	1	0	3	3	3	2
25009	202	0	16-Sep-07	9	1	11	2	1	1	0	13	3	1	1
25010	202	0	28-Jul-12	17	1	11	2	1	1	0	1	3	5	2
25011	202	0	9-Jul-11	19	1	11	2	2	1	0	1	3	5	2
25012	202	0	3-Dec-11	21	1	11	2	1	1	0	1	3	5	2
25013	202	0	26-Nov-11	16	1	11	2	1	1	0	1	3	1	1
25014	202	0	5-Apr-11	18	1	11	2	1	1	0	1	3	1	1
25015	202	0	26-Jul-11	19	1	11	2	1	1	0	1	3	3	2
25016	202	0	4-Dec-10	15	1	11	2	1	1	0	1	3	5	1
25017	202	0	10-May-10	20	1	11	2	1	1	0	1	3	1	1
25018	202	0	15-Jun-10	12	1	11	2	1	1	0	1	3	3	1
25019	202	0	12-Feb-10	14	1	11	2	1	1	0	1	3	2	1
25020	202	0	19-Sep-10	18	1	11	2	1	1	0	1	3	3	1
25021	202	0	1-Jul-09	20	1	11	2	1	1	0	1	3	1	1
25022	202	0	13-Oct-09	18	1	11	2	1	1	0	1	3	1	1
25023	202	0	25-Dec-09	17	1	11	2	1	1	0	1	3	3	1
25024	202	0	12-Apr-08	7	1	11	2	1	1	0	1	3	6	1
25025	202	0	25-Feb-08	20	1	11	2	3	1	0	1	3	1	1
25026	202	0	9-May-08	8	1	11	2	1	1	0	1	3	3	2
25027	202	0	20-Oct-08	19	1	11	2	3	1	0	1	3	1	2
25028	202	0	21-Jul-08	13	1	11	2	2	1	0	1	3	6	1
25029	202	0	30-May-08	9	1	11	2	1	1	0	1	3	5	1
25030	202	0	24-Jul-08	9	1	11	2	1	1	0	1	3	2	1
25031	202	0	18-Jul-08	10	1	11	2	1	1	0	1	3	3	1
25032	202	0	4-Mar-07	9	1	11	2	3	1	0	1	3	1	1
25033	202	0	21-Nov-07	13	1	11	2	1	1	0	1	3	2	2
25034	202	0	13-Sep-07	20	1	11	2	1	1	0	1	3	3	1
25035	202	0	10-Oct-07	6	1	11	2	1	1	0	1	3	3	1
25036	202	0	20-Jun-07	7	1	11	2	1	1	0	1	3	2	1
25037	202	0	11-May-07	11	1	11	2	1	1	0	1	3	3	1
25038	202	0	13-Jul-07	16	1	11	2	1	1	0	1	3	3	1
25039	202	0	7-Aug-06	22	1	11	2	4	1	0	1	3	1	1
25040	202	0	11-Jan-06	10	1	11	2	1	1	0	1	3	3	1
25041	202	0	27-Nov-06	13	1	11	2	1	1	0	1	3	3	1

25042	202	0	10-Nov-06	11	1	11	2	1	1	0	1	3	1	1
25043	202	0	4-Sep-06	11	1	11	2	1	1	0	1	3	2	2
25044	202	0	11-Aug-11	19	1	11	2	1	1	0	1	3	3	1
25045	202	0	24-Nov-12	17	1	11	2	1	1	0	4	3	1	2
25046	202	0	19-Dec-06	12	1	11	2	1	1	0	4	3	4	1
25047	202	0	22-May-16	16	1	11	2	1	1	0	11	3	3	1
25048	202	0	3-Jan-16	16	1	11	2	2	1	0	11	3	3	1
25049	202	0	7-Feb-14	16	1	11	2	1	1	0	11	3	1	1
25050	202	0	28-Jun-13	17	1	11	2	1	1	0	11	3	3	1
25051	202	0	9-Sep-13	12	1	11	2	1	1	0	11	3	2	1
25052	202	0	1-Jul-12	14	1	11	2	1	1	0	11	3	1	2
25053	202	0	2-Jun-11	8	1	11	2	1	1	0	11	3	3	1
25054	202	0	7-Sep-11	21	1	11	2	1	1	0	11	3	2	2
25055	202	0	6-Sep-10	21	1	11	2	1	1	0	11	3	4	1
25056	202	0	16-Aug-17	11	1	11	2	2	1	0	11	3	2	2
25057	202	0	26-Aug-16	16	1	11	2	3	1	0	3	3	2	2
25058	202	0	1-Oct-16	14	1	11	2	3	1	0	3	3	1	1
25059	202	0	28-Sep-15	12	1	11	2	3	1	0	3	3	1	1
25060	202	0	24-May-15	19	1	11	2	1	1	0	3	3	1	1
25061	202	0	28-Dec-14	18	1	11	2	1	1	0	3	3	3	1
25062	202	0	26-Sep-13	11	1	11	2	2	1	0	3	3	2	2
25063	202	0	29-Mar-12	13	1	11	2	1	1	0	3	3	6	1
25064	202	0	11-Sep-12	14	1	11	2	1	1	0	3	3	3	2
25065	202	0	28-Sep-11	17	1	11	2	1	1	0	3	3	1	2
25066	202	0	23-Dec-08	16	1	11	2	1	1	0	3	3	1	2
25067	202	0	19-Mar-07	7	1	11	2	1	1	0	3	3	1	1
25068	202	0	16-Jun-07	15	1	11	2	1	1	0	3	3	3	2
25069	202	0	18-Nov-06	16	1	11	2	1	1	0	3	3	1	1
25070	202	0	24-Sep-06	19	1	11	2	1	1	0	3	3	1	2
25071	202	0	13-Aug-06	17	1	11	2	1	1	0	3	3	7	1
25072	202	0	8-Dec-15	18	1	11	2	1	1	0	6	3	3	1
25073	202	0	25-Sep-07	16	1	11	2	1	1	0	6	3	3	1
25074	202	0	26-Nov-17	16	1	11	2	3	1	0	6	3	1	1
25075	202	0	21-Jul-16	20	2	11	2	3	1	0	1	3	3	2
25076	202	0	1-Sep-12	14	2	11	2	1	1	0	1	3	1	2
25077	202	0	7-Jul-12	19	2	11	2	2	1	0	1	3	1	2
25078	202	0	19-Jul-17	15	2	11	2	1	1	0	1	3	1	1
25079	202	0	1-Oct-17	22	2	11	2	2	1	0	1	3	2	2
25080	202	0	17-Oct-13	14	2	11	2	3	1	0	1	3	5	1
25081	202	0	6-Oct-17	10	2	11	2	3	1	0	1	3	6	1
25082	202	0	30-Jun-16	22	2	11	2	3	1	0	1	3	5	1
25083	202	0	15-Dec-17	7	2	11	2	3	1	0	1	3	3	1
25084	202	0	13-Dec-16	7	2	11	2	1	1	0	1	3	3	1
25085	202	0	20-Jul-13	17	2	11	2	1	1	0	1	3	1	1
25086	202	0	4-Sep-15	16	2	11	2	1	1	0	1	3	1	1
25087	202	0	20-Jun-15	11	2	11	2	2	1	0	1	3	1	2
25088	202	0	22-Mar-14	13	2	11	2	3	1	0	1	3	1	1
25089	202	0	13-Jul-13	12	2	11	2	3	1	0	1	3	5	1
25090	202	0	16-Feb-13	9	2	11	2	1	1	0	1	3	3	1
25091	202	0	12-Jul-17	18	2	11	2	3	1	0	1	3	3	1
25092	202	0	14-May-09	16	2	11	2	2	1	0	13	3	1	1
25093	202	0	14-Feb-11	18	2	11	2	1	1	0	1	3	1	1
25094	202	0	2-Oct-11	20	2	11	2	1	1	0	1	3	3	1
25095	202	0	15-Jan-10	11	2	11	2	1	1	0	1	3	2	1
25096	202	0	27-Dec-10	12	2	11	2	1	1	0	1	3	3	1
25097	202	0	18-Nov-10	14	2	11	2	1	1	0	1	3	3	1
25098	202	0	13-Sep-09	17	2	11	2	2	1	0	1	3	2	1
25099	202	0	31-Oct-09	17	2	11	2	1	1	0	1	3	1	2
25100	202	0	22-Jun-09	19	2	11	2	1	1	0	1	3	3	1
25101	202	0	29-Sep-09	19	2	11	2	1	1	0	1	3	1	1
25102	202	0	30-Dec-09	18	2	11	2	1	1	0	1	3	3	1
25103	202	0	2-Aug-08	10	2	11	2	1	1	0	1	3	6	2
25104	202	0	25-Dec-08	19	2	11	2	1	1	0	1	3	3	2
25105	202	0	20-Oct-07	13	2	11	2	1	1	0	1	3	5	1
25106	202	0	25-Mar-07	16	2	11	2	3	1	0	1	3	1	1
25107	202	0	12-Jun-06	10	2	11	2	1	1	0	1	3	3	1
25108	202	0	25-Feb-06	13	2	11	2	3	1	0	1	3	2	1
25109	202	0	13-Mar-10	18	2	11	2	1	1	0	1	3	1	1
25110	202	0	5-Oct-07	11	2	11	2	1	1	0	1	3	3	1
25111	202	0	17-Mar-13	18	2	11	2	3	1	0	4	3	3	1
25112	202	0	8-Nov-14	15	2	11	2	1	1	0	11	3	2	1
25113	202	0	20-Nov-12	12	2	11	2	1	1	0	11	3	2	1
25114	202	0	26-Nov-17	10	2	11	2	1	1	0	11	3	2	1
25115	202	0	3-May-16	11	2	11	2	3	1	0	3	3	3	1
25116	202	0	18-Nov-14	21	2	11	2	1	1	0	3	3	1	1
25117	202	0	15-Jan-14	0	2	11	2	2	1	0	3	3	3	1
25118	202	0	1-Aug-13	16	2	11	2	2	1	0	3	3	5	1
25119	202	0	4-Apr-12	15	2	11	2	1	1	0	3	3	4	1
25120	202	0	26-Nov-06	17	2	11	2	1	1	0	3	3	2	2
25121	202	0	2-Jul-17	17	2	11	2	2	1	0	3	3	1	1
25122	201	1	13-Jul-15	19	1	11	2	3	1	0	1	3	3	1
25123	101	1	25-Oct-12	19	1	11	2	1	1	0	1	3	5	1
25124	201	1	21-Oct-12	13	1	11	2	3	1	0	1	3	4	1
25125	201	1	26-Apr-12	23	1	11	2	1	1	0	1	3	6	1
25126	201	1	2-Apr-12	10	1	11	2	2	1	0	1	3	2	1
25127	201	1	19-May-17	19	1	11	2	3	1	0	1	3	3	2
25128	101	1	7-Aug-16	10	1	11	2	4	1	0	1	3	1	1
25129	201	1	5-Dec-16	12	1	11	2	2	1	0	1	3	4	1
25130	201	1	25-Feb-16	19	1	11	2	1	1	0	1	3	3	1
25131	201	1	20-Dec-16	8	1	11	2	1	1	0	1	3	2	1
25132	201	1	3-Dec-16	17	1	11	2	3	1	0	1	3	4	1
25133	101	1	2-Nov-15	16	1	11	2	1	1	0	1	3	3	1
25134	101	1	13-Nov-14	12	1	11	2	1	1	0	1	3	2	1
25135	101	1	21-Nov-14	17	1	11	2	1	1	0	1	3	2	2
25136	101	1	7-Feb-13	12	1	11	2	1	1	0	1	3	4	2
25137	101	1	12-Jul-13	19	1	11	2	1	1	0	1	3	2	1
25138	201	1	26-Nov-13	8	1	11	2	1	1	0	1	3	3	1
25139	201	1	22-May-12	17	1	11	2	1	1	0	1	3	4	1
25140	101	1	18-Jan-17	16	1	11	2	1	1	0	1	3	2	2
25141	101	1	12-Jun-12	17	1	11	2	1	1	0	1	3	4	1
25142	101	1	17-Jan-12	11	1	11	2	1	1	0	1	3	5	1
25143	201	1	15-Jul-14	10	1	11	2	1	1	0	1	3	1	1
25144	101	1	4-Jul-14	18	1	11	2	1	1	0	1	3	5	2
25145	201	1	13-Dec-13	19	1	11	2	1	1	0	1	3	5	2
25146	101	1	25-Mar-13	12	1	11	2	1	1	0	1	3	5	1
25147	201	1	5-Apr-12	3	1	11	2	1	1	0	1	3	1	2
25148	201	1	30-Oct-12	10	1	11	2	3	1	0	1	3	3	1
25149	101	1	28-Aug-12	10	1	11	2	1	1	0	1	3	3	1
25150	101	1	15-Dec-12	19	1	11	2	3	1	0	1	3	3	1
25151	201	1	4-Nov-17	19	1	11	2	1	1	0	1	3	5	1
25152	101	1	16-May-16	17	1	11	2	1	1	0	1	3	1	1
25153	101	1	28-Feb-14	14	1	11	2	1	1	0	1	3	3	1
25154	101	1	28-Jan-12	22	1	11	2	1	1	0	1	3	5	1
25155	101	1	24-Mar-12	19	1	11	2	2	1	0	1	3	3	1
25156	101	1	8-Jun-16	18	1	11	2	1	1	0	1	3	5	1
25157	201	1	25-Oct-11	14	1	11	2	1	1	0	1	3	1	1
25158	201	1	20-Jul-11	14	1	11	2	3	1	0	1	3	1	1
25159	201	1	30-Oct-11	10	1	11	2	1	1	0	1	3	1	1
25160	101	1	24-Dec-11	15	1	11	2	1	1	0	1	3	1	1
25161	201	1	9-May-11	5	1	11	2	4	1	0	1	3	2	2
25162	201	1	30-Oct-11	10	1	11	2	1	1	0	1	3	2	2
25163	201	1	14-Aug-11	1	1	11	2	1	1	0	1	3	2	1
25164	201	1	15-Oct-11	8	1	11	2	1	1	0	1	3	1	1
25165	201	1	3-Nov-11	21	1	11	2	1	1	0	1	3	1	2

25166	201	1	25-Dec-11	10	1	11	2	2	1	0	1	3	6	1
25167	201	1	20-Jul-11	4	1	11	2	1	1	0	1	3	1	1
25168	101	1	23-Jun-11	12	1	11	2	1	1	0	1	3	3	1
25169	201	1	21-Apr-10	8	1	11	2	1	1	0	1	3	1	1
25170	201	1	26-Jun-10	15	1	11	2	1	1	0	1	3	1	2
25171	201	1	15-Jan-10	21	1	11	2	1	1	0	1	3	1	2
25172	101	1	27-Aug-10	9	1	11	2	1	1	0	1	3	4	1
25173	201	1	13-Dec-10	8	1	11	2	1	1	0	1	3	2	2
25174	201	1	10-Apr-10	12	1	11	2	2	1	0	1	3	1	2
25175	201	1	12-Aug-10	20	1	11	2	1	1	0	1	3	2	1
25176	201	1	3-May-10	21	1	11	2	3	1	0	1	3	1	1
25177	201	1	10-Sep-10	8	1	11	2	1	1	0	1	3	3	1
25178	201	1	30-Nov-09	8	1	11	2	1	1	0	1	3	4	1
25179	201	1	18-Feb-09	14	1	11	2	1	1	0	1	3	1	2
25180	101	1	10-Oct-09	11	1	11	2	1	1	0	1	3	2	1
25181	201	1	29-Jul-09	23	1	11	2	1	1	0	1	3	4	1
25182	101	1	27-Aug-09	17	1	11	2	3	1	0	1	3	3	1
25183	201	1	14-May-09	12	1	11	2	1	1	0	1	3	3	1
25184	201	1	7-Oct-09	20	1	11	2	1	1	0	1	3	4	1
25185	101	1	17-Dec-08	14	1	11	2	1	1	0	1	3	6	1
25186	101	1	26-Jul-08	17	1	11	2	1	1	0	1	3	1	1
25187	101	1	14-Sep-08	10	1	11	2	1	1	0	1	3	5	1
25188	101	1	2-Jul-08	15	1	11	2	1	1	0	1	3	1	1
25189	201	1	10-Oct-08	17	1	11	2	1	1	0	1	3	5	1
25190	101	1	29-Jun-08	12	1	11	2	1	1	0	1	3	5	1
25191	201	1	13-Jun-08	17	1	11	2	3	1	0	1	3	5	2
25192	201	1	9-Dec-08	13	1	11	2	1	1	0	1	3	4	2
25193	101	1	3-Nov-08	16	1	11	2	1	1	0	1	3	3	1
25194	101	1	3-Jun-08	17	1	11	2	1	1	0	1	3	2	1
25195	201	1	13-Sep-07	13	1	11	2	1	1	0	1	3	5	1
25196	201	1	11-Oct-07	7	1	11	2	2	1	0	1	3	4	1
25197	201	1	6-Oct-07	19	1	11	2	1	1	0	1	3	3	1
25198	201	1	22-Apr-07	11	1	11	2	1	1	0	1	3	6	1
25199	201	1	2-Dec-07	21	1	11	2	2	1	0	1	3	4	1
25200	201	1	19-Jun-07	13	1	11	2	1	1	0	1	3	1	1
25201	101	1	12-May-07	18	1	11	2	1	1	0	1	3	3	1
25202	201	1	1-Jan-07	20	1	11	2	1	1	0	1	3	2	1
25203	201	1	16-Nov-07	16	1	11	2	2	1	0	1	3	5	1
25204	201	1	19-Aug-07	16	1	11	2	1	1	0	1	3	4	1
25205	101	1	2-May-07	18	1	11	2	1	1	0	1	3	2	2
25206	201	1	3-Mar-07	20	1	11	2	1	1	0	1	3	2	1
25207	201	1	5-Nov-07	20	1	11	2	3	1	0	1	3	2	2
25208	101	1	5-Nov-07	18	1	11	2	1	1	0	1	3	2	2
25209	101	1	29-Mar-07	20	1	11	2	2	1	0	1	3	3	1
25210	201	1	28-Jun-06	19	1	11	2	3	1	0	1	3	3	1
25211	101	1	3-Jul-06	22	1	11	2	1	1	0	1	3	3	1
25212	201	1	25-Sep-06	12	1	11	2	1	1	0	1	3	6	1
25213	201	1	21-Apr-06	22	1	11	2	2	1	0	1	3	6	1
25214	101	1	6-Jul-06	9	1	11	2	1	1	0	1	3	2	1
25215	101	1	11-Jul-06	17	1	11	2	1	1	0	1	3	3	2
25216	101	1	22-Apr-06	9	1	11	2	1	1	0	1	3	3	2
25217	201	1	10-Oct-09	14	1	11	2	1	1	0	5	3	2	1
25218	101	1	30-Aug-14	11	1	11	2	1	1	0	1	3	7	1
25219	101	1	17-Mar-13	14	1	11	2	1	1	0	1	3	1	1
25220	101	1	15-Sep-13	18	1	11	2	1	1	0	1	3	3	1
25221	101	1	28-Aug-11	11	1	11	2	2	1	0	1	3	1	1
25222	101	1	20-Mar-11	19	1	11	2	1	1	0	1	3	4	1
25223	101	1	17-Sep-11	13	1	11	2	1	1	0	1	3	3	1
25224	101	1	12-Jun-11	16	1	11	2	1	1	0	1	3	1	1
25225	101	1	21-Mar-10	13	1	11	2	1	1	0	1	3	1	1
25226	101	1	27-Apr-10	17	1	11	2	1	1	0	1	3	2	4
25227	201	1	11-Jan-10	8	1	11	2	1	1	0	1	3	1	1
25228	101	1	15-Sep-10	17	1	11	2	1	1	0	1	3	2	1
25229	101	1	28-Nov-09	17	1	11	2	1	1	0	1	3	3	4
25230	101	1	29-Sep-09	14	1	11	2	1	1	0	1	3	3	1
25231	201	1	17-Mar-09	12	1	11	2	1	1	0	1	3	5	1
25232	101	1	13-Mar-09	14	1	11	2	1	1	0	1	3	3	4
25233	201	1	29-Sep-09	15	1	11	2	1	1	0	1	3	2	1
25234	101	1	8-Mar-08	20	1	11	2	1	1	0	1	3	6	1
25235	201	1	25-Feb-08	16	1	11	2	1	1	0	1	3	1	1
25236	201	1	29-Jun-08	18	1	11	2	1	1	0	1	3	2	1
25237	101	1	29-Mar-08	22	1	11	2	1	1	0	1	3	3	1
25238	201	1	1-Jun-08	18	1	11	2	3	1	0	1	3	3	1
25239	101	1	5-Mar-07	12	1	11	2	1	1	0	1	3	4	1
25240	201	1	31-Jul-07	19	1	11	2	1	1	0	1	3	4	2
25241	201	1	17-Aug-07	14	1	11	2	1	1	0	1	3	5	1
25242	201	1	10-Mar-07	18	1	11	2	1	1	0	1	3	1	1
25243	201	1	19-Oct-07	21	1	11	2	1	1	0	1	3	1	1
25244	201	1	6-Sep-07	11	1	11	2	1	1	0	1	3	2	1
25245	201	1	15-Jun-15	18	1	11	2	1	1	0	4	3	3	1
25246	101	1	2-Sep-13	11	1	11	2	1	1	0	4	3	5	1
25247	201	1	3-Aug-11	8	1	11	2	1	1	0	4	3	3	1
25248	201	1	22-Jul-08	22	1	11	2	2	1	0	4	3	1	1
25249	201	1	28-Jul-08	17	1	11	2	2	1	0	4	3	1	1
25250	201	1	7-Feb-07	18	1	11	2	1	1	0	4	3	4	1
25251	101	1	30-Nov-06	18	1	11	2	1	1	0	4	3	7	1
25252	101	1	14-Mar-17	19	1	11	2	1	1	0	4	3	1	1
25253	201	1	29-Jun-16	19	1	11	2	1	1	0	11	3	3	1
25254	101	1	5-Feb-16	11	1	11	2	1	1	0	11	3	2	1
25255	101	1	18-Feb-14	17	1	11	2	1	1	0	11	3	3	1
25256	201	1	6-Oct-14	14	1	11	2	1	1	0	11	3	3	1
25257	101	1	22-Feb-13	18	1	11	2	1	1	0	11	3	2	1
25258	201	1	30-Jan-12	16	1	11	2	1	1	0	11	3	3	1
25259	101	1	22-Jun-12	16	1	11	2	1	1	0	11	3	1	1
25260	101	1	6-Mar-12	12	1	11	2	1	1	0	11	3	2	1
25261	101	1	25-Aug-12	10	1	11	2	1	1	0	11	3	2	1
25262	101	1	24-Jan-10	15	1	11	2	2	1	0	11	3	2	1
25263	101	1	18-Mar-10	18	1	11	2	1	1	0	11	3	2	1
25264	201	1	8-Feb-10	21	1	11	2	2	1	0	11	3	4	1
25265	101	1	25-Aug-10	20	1	11	2	1	1	0	11	3	2	1
25266	101	1	14-Dec-10	17	1	11	2	1	1	0	11	3	2	1
25267	201	1	9-May-09	1	1	11	2	2	1	0	11	3	6	1
25268	201	1	18-May-09	20	1	11	2	2	1	0	11	3	3	1
25269	101	1	14-May-09	19	1	11	2	1	1	0	11	3	3	1
25270	101	1	13-Nov-09	17	1	11	2	2	1	0	11	3	3	1
25271	101	1	10-May-09	19	1	11	2	1	1	0	11	3	3	1
25272	101	1	11-Aug-08	17	1	11	2	1	1	0	11	3	1	1
25273	201	1	8-Apr-07	17	1	11	2	1	1	0	11	3	2	1
25274	101	1	25-May-07	16	1	11	2	1	1	0	11	3	2	1
25275	101	1	23-Aug-06	21	1	11	2	1	1	0	11	3	2	1
25276	101	1	27-Sep-06	13	1	11	2	1	1	0	11	3	2	1
25277	201	1	23-Oct-17	17	1	11	2	1	1	0	11	3	3	1
25278	201	1	11-Jun-17	9	1	11	2	2	1	0	11	3	2	1
25279	101	1	11-Sep-17	17	1	11	2	1	1	0	11	3	2	1
25280	101	1	30-Oct-17	11	1	11	2	1	1	0	11	3	2	1
25281	201	1	8-Mar-16	11	1	11	2	2	1	0	3	3	2	1
25282	101	1	31-Jan-16	17	1	11	2	1	1	0	3	3	3	1
25283	201	1	4-Nov-16	18	1	11	2	2	1	0	3	3	1	1
25284	101	1	12-Nov-16	16	1	11	2	2	1	0	3	3	1	1
25285	101	1	19-May-16	17	1	11	2	1	1	0	3	3	2	1
25286	101	1	21-Jun-16	19	1	11	2	1	1	0	3	3	3	1
25287	101	1	4-Mar-16	14	1	11	2	1	1	0	3	3	2	4
25288	101	1	3-Sep-15	20	1	11	2	1	1	0	3	3	2	2
25289	101	1	19-Aug-15	11	1	11	2	1	1	0	3	3	3	1

25290	201	1	15-Aug-15	20	1	11	2	3	1	0	3	3	2	1
25291	201	1	26-Apr-15	9	1	11	2	1	1	0	3	3	3	1
25292	101	1	25-Sep-15	21	1	11	2	1	1	0	3	3	1	4
25293	201	1	3-Nov-15	16	1	11	2	2	1	0	3	3	2	1
25294	201	1	8-Sep-15	14	1	11	2	2	1	0	3	3	3	1
25295	201	1	3-Apr-15	6	1	11	2	1	1	0	3	3	3	1
25296	101	1	7-Nov-14	14	1	11	2	1	1	0	3	3	1	1
25297	201	1	12-Dec-14	8	1	11	2	2	1	0	3	3	3	1
25298	101	1	14-Aug-14	21	1	11	2	1	1	0	3	3	2	1
25299	201	1	22-Jul-14	22	1	11	2	1	1	0	3	3	4	1
25300	201	1	10-Nov-13	14	1	11	2	2	1	0	3	3	5	1
25301	201	1	16-Oct-13	17	1	11	2	3	1	0	3	3	1	1
25302	101	1	19-Oct-13	20	1	11	2	1	1	0	3	3	1	1
25303	101	1	14-May-13	13	1	11	2	3	1	0	3	3	5	4
25304	101	1	13-Nov-13	10	1	11	2	1	1	0	3	3	2	1
25305	101	1	25-Mar-13	17	1	11	2	1	1	0	3	3	2	1
25306	201	1	21-Apr-13	16	1	11	2	1	1	0	3	3	3	1
25307	101	1	12-Oct-13	22	1	11	2	1	1	0	3	3	3	1
25308	101	1	29-Sep-12	20	1	11	2	1	1	0	3	3	3	1
25309	101	1	19-Jul-12	20	1	11	2	1	1	0	3	3	3	1
25310	101	1	8-Nov-12	16	1	11	2	1	1	0	3	3	2	4
25311	101	1	27-Apr-12	17	1	11	2	1	1	0	3	3	3	1
25312	101	1	2-Jul-11	23	1	11	2	1	1	0	3	3	2	1
25313	201	1	3-May-11	17	1	11	2	1	1	0	3	3	3	1
25314	201	1	16-Jun-11	14	1	11	2	3	1	0	3	3	3	1
25315	201	1	9-Mar-11	18	1	11	2	2	1	0	3	3	1	1
25316	201	1	23-Jun-11	13	1	11	2	2	1	0	3	3	1	1
25317	101	1	8-Dec-11	18	1	11	2	1	1	0	3	3	2	1
25318	201	1	28-Jul-11	6	1	11	2	1	1	0	3	3	3	1
25319	101	1	24-Jun-10	17	1	11	2	1	1	0	3	3	1	1
25320	101	1	29-Sep-10	17	1	11	2	1	1	0	3	3	3	1
25321	201	1	3-May-10	12	1	11	2	1	1	0	3	3	3	1
25322	201	1	21-Apr-10	19	1	11	2	1	1	0	3	3	3	1
25323	101	1	3-Jun-10	14	1	11	2	2	1	0	3	3	2	1
25324	201	1	23-Mar-10	17	1	11	2	2	1	0	3	3	3	1
25325	101	1	20-Oct-09	17	1	11	2	1	1	0	3	3	3	1
25326	101	1	30-Jun-09	21	1	11	2	1	1	0	3	3	3	1
25327	201	1	22-Jul-09	17	1	11	2	1	1	0	3	3	2	1
25328	101	1	25-Nov-09	16	1	11	2	1	1	0	3	3	2	1
25329	101	1	28-May-08	19	1	11	2	1	1	0	3	3	5	1
25330	201	1	12-Nov-08	19	1	11	2	2	1	0	3	3	4	1
25331	201	1	29-Jun-08	12	1	11	2	1	1	0	3	3	2	1
25332	201	1	13-Dec-08	18	1	11	2	1	1	0	3	3	2	2
25333	101	1	19-Oct-08	17	1	11	2	1	1	0	3	3	3	1
25334	101	1	2-May-08	17	1	11	2	1	1	0	3	3	3	1
25335	101	1	14-Dec-08	19	1	11	2	1	1	0	3	3	3	1
25336	201	1	21-Oct-08	17	1	11	2	3	1	0	3	3	2	1
25337	201	1	2-Mar-08	16	1	11	2	3	1	0	3	3	3	1
25338	101	1	7-Oct-07	18	1	11	2	1	1	0	3	3	2	1
25339	101	1	1-Oct-07	17	1	11	2	1	1	0	3	3	3	1
25340	101	1	25-Jul-07	21	1	11	2	1	1	0	3	3	5	1
25341	201	1	2-Nov-07	22	1	11	2	3	1	0	3	3	2	1
25342	201	1	16-Jun-06	18	1	11	2	1	1	0	3	3	3	1
25343	201	1	31-Aug-06	7	1	11	2	1	1	0	3	3	3	1
25344	101	1	21-May-06	18	1	11	2	1	1	0	3	3	2	1
25345	101	1	17-Jan-17	10	1	11	2	1	1	0	3	3	3	1
25346	201	1	26-Jun-17	10	1	11	2	1	1	0	3	3	3	1
25347	201	1	1-Nov-17	21	1	11	2	1	1	0	3	3	3	1
25348	201	1	12-Aug-17	16	1	11	2	1	1	0	3	3	2	1
25349	101	1	6-Jan-17	10	1	11	2	1	1	0	3	3	1	1
25350	201	1	29-Oct-17	15	1	11	2	1	1	0	3	3	1	1
25351	201	1	1-Nov-17	13	1	11	2	2	1	0	3	3	3	1
25352	201	1	7-Nov-17	19	1	11	2	2	1	0	3	3	2	1
25353	201	1	3-Apr-17	20	1	11	2	2	1	0	3	3	2	2
25354	201	1	3-Feb-15	9	1	11	2	1	1	0	6	3	4	1
25355	101	1	3-Sep-14	17	1	11	2	2	1	0	6	3	3	1
25356	101	1	18-Jun-13	10	1	11	2	1	1	0	6	3	3	1
25357	201	1	21-Apr-11	19	1	11	2	4	1	0	6	3	3	1
25358	201	1	24-Aug-10	22	1	11	2	2	1	0	6	3	3	1
25359	101	1	25-Jan-10	10	1	11	2	1	1	0	6	3	2	1
25360	201	1	2-Jan-06	14	1	11	2	1	1	0	6	3	3	1
25361	101	1	14-Apr-17	20	1	11	2	1	1	0	6	3	2	1
25362	101	1	5-Nov-17	19	1	11	2	1	1	0	13	3	2	1
25363	101	1	23-Apr-13	20	2	11	2	1	1	0	1	3	4	1
25364	101	1	24-Sep-15	13	2	11	2	1	1	0	1	3	1	4
25365	201	1	30-Aug-16	11	2	11	2	1	1	0	1	3	3	1
25366	101	1	21-Apr-14	21	2	11	2	1	1	0	1	3	5	1
25367	201	1	23-Jul-14	19	2	11	2	1	1	0	1	3	5	1
25368	201	1	20-Jul-13	18	2	11	2	1	1	0	1	3	1	1
25369	101	1	11-Mar-17	12	2	11	2	1	1	0	1	3	1	1
25370	101	1	24-Apr-17	12	2	11	2	1	1	0	1	3	4	2
25371	101	1	3-Oct-17	10	2	11	2	1	1	0	3	3	6	1
25372	201	1	7-Jun-15	17	2	11	2	2	1	0	1	3	4	1
25373	201	1	16-Feb-14	12	2	11	2	1	1	0	1	3	2	1
25374	201	1	26-Jul-14	12	2	11	2	2	1	0	1	3	5	1
25375	201	1	26-Aug-12	16	2	11	2	3	1	0	1	3	1	1
25376	201	1	6-Jun-17	13	2	11	2	3	1	0	1	3	5	1
25377	101	1	18-Dec-16	16	2	11	2	1	1	0	1	3	3	1
25378	201	1	2-Apr-15	17	2	11	2	1	1	0	1	3	5	2
25379	101	1	13-Dec-15	19	2	11	2	1	1	0	1	3	3	1
25380	101	1	17-May-12	12	2	11	2	1	1	0	1	3	3	1
25381	201	1	29-Jan-16	20	2	11	2	3	1	0	1	3	2	2
25382	201	1	21-May-12	8	2	11	2	3	1	0	1	3	3	1
25383	201	1	5-May-12	20	2	11	2	2	1	0	6	3	3	1
25384	101	1	5-Mar-07	16	2	11	2	1	1	0	7	3	3	1
25385	101	1	15-Jul-12	16	2	11	2	1	1	0	1	3	5	1
25386	101	1	24-May-11	7	2	11	2	1	1	0	1	3	4	1
25387	201	1	5-Nov-11	20	2	11	2	1	1	0	1	3	2	2
25388	101	1	21-Jul-11	21	2	11	2	1	1	0	1	3	3	1
25389	201	1	3-Nov-11	23	2	11	2	1	1	0	1	3	1	2
25390	201	1	8-Mar-11	17	2	11	2	2	1	0	1	3	5	1
25391	101	1	6-Oct-11	19	2	11	2	1	1	0	1	3	3	1
25392	201	1	4-Feb-11	13	2	11	2	1	1	0	1	3	4	1
25393	101	1	15-May-10	17	2	11	2	1	1	0	1	3	3	1
25394	201	1	8-Oct-10	13	2	11	2	1	1	0	1	3	5	1
25395	201	1	25-Jul-10	17	2	11	2	3	1	0	1	3	5	1
25396	201	1	31-May-10	18	2	11	2	2	1	0	1	3	4	1
25397	201	1	26-Feb-10	11	2	11	2	1	1	0	1	3	4	2
25398	201	1	28-Feb-10	19	2	11	2	1	1	0	1	3	2	2
25399	101	1	30-Aug-09	13	2	11	2	2	1	0	1	3	5	1
25400	101	1	16-Nov-09	17	2	11	2	1	1	0	1	3	5	1
25401	101	1	26-Oct-09	17	2	11	2	1	1	0	1	3	1	1
25402	201	1	2-Aug-09	22	2	11	2	3	1	0	1	3	2	1
25403	201	1	20-Jul-09	15	2	11	2	2	1	0	1	3	2	1
25404	101	1	14-Mar-09	16	2	11	2	1	1	0	1	3	3	4
25405	101	1	27-Feb-09	12	2	11	2	1	1	0	1	3	3	1
25406	101	1	20-Sep-08	20	2	11	2	1	1	0	1	3	2	1
25407	201	1	1-Aug-08	9	2	11	2	1	1	0	1	3	4	1
25408	101	1	26-Mar-08	22	2	11	2	2	1	0	1	3	1	4
25409	201	1	15-Oct-07	17	2	11	2	1	1	0	1	3	1	2
25410	201	1	16-Jun-07	13	2	11	2	2	1	0	1	3	3	2
25411	201	1	19-Mar-07	7	2	11	2	1	1	0	1	3	3	1
25412	101	1	13-Oct-07	23	2	11	2	2	1	0	1	3	4	1
25413	101	1	11-Sep-06	10	2	11	2	1	1	0	1	3	3	1

25414	201	1	20-Feb-06	14	2	11	2	1	1	0	1	3	1	2
25415	101	1	17-Sep-11	15	2	11	2	1	1	0	1	3	5	1
25416	101	1	1-Apr-10	17	2	11	2	1	1	0	1	3	5	1
25417	201	1	13-May-09	8	2	11	2	1	1	0	1	3	1	1
25418	101	1	7-Aug-09	18	2	11	2	2	1	0	1	3	5	1
25419	101	1	19-Jul-06	18	2	11	2	1	1	0	1	3	5	1
25420	101	1	26-Feb-11	18	2	11	2	1	1	0	4	3	3	1
25421	101	1	13-Oct-16	7	2	11	2	1	1	0	11	3	2	1
25422	201	1	11-May-16	7	2	11	2	3	1	0	11	3	3	1
25423	201	1	26-Nov-16	20	2	11	2	1	1	0	11	3	2	1
25424	201	1	1-May-16	13	2	11	2	1	1	0	11	3	2	1
25425	101	1	15-Jun-15	12	2	11	2	1	1	0	11	3	2	1
25426	101	1	25-Aug-15	17	2	11	2	1	1	0	11	3	3	1
25427	101	1	3-Jan-15	16	2	11	2	1	1	0	11	3	5	1
25428	201	1	16-Dec-14	19	2	11	2	1	1	0	11	3	2	1
25429	101	1	1-Feb-13	19	2	11	2	1	1	0	11	3	2	1
25430	101	1	1-Oct-13	7	2	11	2	1	1	0	11	3	3	1
25431	101	1	26-Aug-12	19	2	11	2	2	1	0	11	3	3	4
25432	101	1	27-Apr-11	20	2	11	2	1	1	0	11	3	3	1
25433	201	1	9-Jul-09	8	2	11	2	3	1	0	11	3	3	1
25434	201	1	2-Mar-09	18	2	11	2	2	1	0	11	3	3	1
25435	101	1	30-Sep-08	17	2	11	2	2	1	0	11	3	1	1
25436	201	1	27-Jul-08	11	2	11	2	3	1	0	11	3	2	1
25437	101	1	1-Mar-06	17	2	11	2	1	1	0	11	3	2	1
25438	101	1	8-Nov-16	2	2	11	2	1	1	0	3	3	4	1
25439	101	1	1-Nov-16	2	2	11	2	1	1	0	3	3	4	1
25440	201	1	29-Feb-16	19	2	11	2	3	1	0	3	3	3	1
25441	201	1	26-May-16	20	2	11	2	1	1	0	3	3	3	1
25442	101	1	17-Nov-16	3	2	11	2	1	1	0	3	3	4	1
25443	101	1	18-Nov-16	2	2	11	2	1	1	0	3	3	1	1
25444	101	1	29-Mar-15	11	2	11	2	1	1	0	3	3	1	1
25445	101	1	7-Jul-15	16	2	11	2	1	1	0	3	3	7	1
25446	201	1	16-Apr-15	7	2	11	2	2	1	0	3	3	2	1
25447	101	1	12-Aug-14	14	2	11	2	1	1	0	3	3	3	1
25448	101	1	1-Jun-13	12	2	11	2	3	1	0	3	3	4	2
25449	101	1	2-Jun-13	10	2	11	2	1	1	0	3	3	3	1
25450	101	1	25-Sep-13	16	2	11	2	1	1	0	3	3	3	1
25451	101	1	14-Jun-13	14	2	11	2	1	1	0	3	3	3	1
25452	101	1	25-Dec-13	16	2	11	2	1	1	0	3	3	3	1
25453	101	1	6-Jun-12	17	2	11	2	1	1	0	3	3	3	1
25454	101	1	30-Sep-12	23	2	11	2	1	1	0	3	3	3	1
25455	101	1	7-Oct-12	12	2	11	2	1	1	0	3	3	3	1
25456	201	1	3-Jun-11	12	2	11	2	1	1	0	3	3	1	1
25457	201	1	2-Apr-10	14	2	11	2	2	1	0	3	3	3	1
25458	101	1	10-Mar-10	18	2	11	2	1	1	0	3	3	1	1
25459	201	1	5-Jun-10	17	2	11	2	1	1	0	3	3	1	1
25460	201	1	10-Jan-10	19	2	11	2	2	1	0	3	3	7	1
25461	201	1	19-May-10	18	2	11	2	2	1	0	3	3	3	1
25462	101	1	19-Aug-09	22	2	11	2	1	1	0	3	3	5	1
25463	201	1	17-Mar-09	12	2	11	2	1	1	0	3	3	2	1
25464	101	1	29-May-08	11	2	11	2	1	1	0	3	3	1	1
25465	101	1	13-Jul-08	21	2	11	2	3	1	0	3	3	1	2
25466	101	1	9-Jul-08	20	2	11	2	1	1	0	3	3	3	1
25467	101	1	27-Dec-08	10	2	11	2	1	1	0	3	3	2	1
25468	101	1	12-Jun-07	15	2	11	2	1	1	0	3	3	2	1
25469	101	1	30-Apr-07	21	2	11	2	1	1	0	3	3	3	1
25470	201	1	20-Dec-06	17	2	11	2	2	1	0	3	3	5	1
25471	201	1	20-Jul-17	17	2	11	2	1	1	0	3	3	3	1
25472	201	1	23-Dec-17	17	2	11	2	1	1	0	3	3	2	1
25473	101	1	30-Jun-06	16	2	11	2	2	1	0	6	3	2	1
25474	101	1	20-Nov-17	18	2	11	2	1	1	0	13	3	3	1
25475	202	0	19-Jul-16	18	1	12	2	3	1	0	1	3	3	1
25476	202	0	1-Jul-15	12	1	12	2	1	1	0	1	3	7	2
25477	202	0	17-Nov-15	7	1	12	2	1	1	0	1	3	5	1
25478	202	0	18-Mar-16	19	1	12	2	3	1	0	1	3	1	1
25479	202	0	2-Sep-15	19	1	12	2	1	1	0	1	3	3	1
25480	202	0	5-Oct-14	21	1	12	2	1	1	0	1	3	2	2
25481	202	0	24-Jul-14	19	1	12	2	3	1	0	1	3	2	1
25482	202	0	18-Oct-13	21	1	12	2	1	1	0	1	3	2	2
25483	202	0	1-Jan-12	11	1	12	2	3	1	0	1	3	2	2
25484	202	0	18-Oct-17	19	1	12	2	1	1	0	1	3	3	1
25485	202	0	5-Apr-17	13	1	12	2	1	1	0	1	3	1	2
25486	202	0	24-Aug-16	15	1	12	2	1	1	0	1	3	4	1
25487	202	0	5-Nov-17	16	1	12	2	2	1	0	1	3	1	2
25488	202	0	17-Jul-14	20	1	12	2	2	1	0	1	3	1	1
25489	202	0	17-Jul-14	18	1	12	2	1	1	0	1	3	2	1
25490	202	0	4-Feb-13	14	1	12	2	3	1	0	4	3	1	1
25491	202	0	22-Jul-17	10	1	12	2	3	1	0	1	3	1	2
25492	202	0	24-Aug-16	13	1	12	2	1	1	0	1	3	3	1
25493	202	0	7-Jul-15	14	1	12	2	1	1	0	1	3	5	1
25494	202	0	23-Oct-15	21	1	12	2	3	1	0	1	3	5	1
25495	202	0	31-Oct-14	17	1	12	2	2	1	0	1	3	5	1
25496	202	0	17-Jul-14	18	1	12	2	1	1	0	1	3	1	1
25497	202	0	13-Apr-14	11	1	12	2	1	1	0	1	3	3	1
25498	202	0	18-Jul-13	18	1	12	2	3	1	0	1	3	3	1
25499	202	0	30-Nov-07	18	1	12	2	1	1	0	13	3	2	2
25500	202	0	14-Aug-11	19	1	12	2	1	1	0	1	3	6	2
25501	202	0	23-Apr-11	21	1	12	2	1	1	0	1	3	5	1
25502	202	0	8-Sep-11	3	1	12	2	3	1	0	1	3	6	2
25503	202	0	6-Aug-11	13	1	12	2	1	1	0	1	3	1	2
25504	202	0	13-Mar-11	19	1	12	2	1	1	0	1	3	3	1
25505	202	0	18-Feb-11	11	1	12	2	1	1	0	1	3	3	2
25506	202	0	2-Oct-10	10	1	12	2	3	1	0	1	3	6	2
25507	202	0	23-Jun-10	16	1	12	2	1	1	0	1	3	1	1
25508	202	0	15-Jun-10	12	1	12	2	1	1	0	1	3	3	1
25509	202	0	15-Dec-10	18	1	12	2	1	1	0	1	3	3	1
25510	202	0	29-Jul-10	19	1	12	2	2	1	0	1	3	3	1
25511	202	0	19-Oct-10	13	1	12	2	3	1	0	1	3	6	2
25512	202	0	19-Oct-10	13	1	12	2	3	1	0	1	3	3	2
25513	202	0	9-May-09	13	1	12	2	3	1	0	1	3	6	2
25514	202	0	23-Mar-09	13	1	12	2	1	1	0	1	3	2	1
25515	202	0	26-Apr-09	13	1	12	2	1	1	0	1	3	1	2
25516	202	0	13-May-09	23	1	12	2	1	1	0	1	3	3	1
25517	202	0	7-May-09	12	1	12	2	1	1	0	1	3	3	1
25518	202	0	4-Sep-09	19	1	12	2	1	1	0	1	3	2	2
25519	202	0	7-Feb-08	21	1	12	2	1	1	0	1	3	6	1
25520	202	0	25-Aug-08	16	1	12	2	1	1	0	1	3	1	2
25521	202	0	10-Nov-08	12	1	12	2	1	1	0	1	3	6	1
25522	202	0	15-Nov-08	14	1	12	2	3	1	0	1	3	1	2
25523	202	0	17-Apr-08	20	1	12	2	1	1	0	1	3	2	2
25524	202	0	8-Sep-08	15	1	12	2	1	1	0	1	3	3	1
25525	202	0	10-Aug-08	14	1	12	2	1	1	0	1	3	5	1
25526	202	0	6-Apr-08	16	1	12	2	1	1	0	1	3	1	1
25527	202	0	24-Jan-07	7	1	12	2	3	1	0	1	3	3	2
25528	202	0	23-Nov-07	8	1	12	2	1	1	0	1	3	1	1
25529	202	0	1-Dec-07	19	1	12	2	1	1	0	1	3	6	2
25530	202	0	28-Mar-07	7	1	12	2	1	1	0	1	3	3	1
25531	202	0	6-Jan-07	15	1	12	2	1	1	0	1	3	5	2
25532	202	0	30-Jan-06	14	1	12	2	1	1	0	1	3	3	1
25533	202	0	20-Nov-06	20	1	12	2	4	1	0	1	3	5	1
25534	202	0	8-Aug-08	18	1	12	2	1	1	0	1	3	4	1
25535	202	0	30-Dec-16	18	1	12	2	3	1	0	4	3	1	1
25536	202	0	2-Jul-10	7	1	12	2	3	1	0	4	3	2	1
25537	202	0	16-Dec-07	15	1	12	2	1	1	0	4	3	3	1

25538	202	0	7-Jan-13	13	1	12	2	1	1	0	11	3	3	1
25539	202	0	12-Jan-08	16	1	12	2	1	1	0	11	3	2	1
25540	202	0	16-Aug-07	22	1	12	2	1	1	0	11	3	3	2
25541	202	0	19-Mar-06	17	1	12	2	3	1	0	11	3	3	1
25542	202	0	15-Feb-06	18	1	12	2	1	1	0	11	3	3	1
25543	202	0	18-Feb-16	20	1	12	2	1	1	0	3	3	3	2
25544	202	0	20-Feb-16	18	1	12	2	3	1	0	3	3	1	1
25545	202	0	9-Oct-16	13	1	12	2	3	1	0	3	3	2	2
25546	202	0	26-Jun-15	20	1	12	2	1	1	0	3	3	1	1
25547	202	0	15-May-15	20	1	12	2	2	1	0	3	3	1	1
25548	202	0	1-Feb-14	8	1	12	2	1	1	0	3	3	4	2
25549	202	0	13-Oct-14	18	1	12	2	1	1	0	3	3	3	2
25550	202	0	18-Jun-14	4	1	12	2	1	1	0	3	3	3	2
25551	202	0	1-Dec-12	17	1	12	2	1	1	0	3	3	5	1
25552	202	0	18-Feb-12	12	1	12	2	1	1	0	3	3	1	1
25553	202	0	28-Feb-11	12	1	12	2	1	1	0	3	3	3	1
25554	202	0	17-Jul-09	16	1	12	2	1	1	0	3	3	2	1
25555	202	0	8-Sep-09	8	1	12	2	1	1	0	3	3	3	1
25556	202	0	23-Sep-09	17	1	12	2	1	1	0	3	3	3	1
25557	202	0	11-May-08	7	1	12	2	1	1	0	3	3	2	2
25558	202	0	30-Aug-08	14	1	12	2	3	1	0	3	3	3	2
25559	202	0	14-Oct-07	15	1	12	2	1	1	0	3	3	6	1
25560	202	0	23-Aug-07	18	1	12	2	1	1	0	3	3	3	1
25561	202	0	26-May-07	17	1	12	2	1	1	0	3	3	2	1
25562	202	0	16-Aug-06	15	1	12	2	1	1	0	3	3	2	1
25563	202	0	12-Sep-17	11	1	12	2	2	1	0	3	3	1	2
25564	202	0	23-Aug-17	12	1	12	2	2	1	0	3	3	3	1
25565	202	0	6-Jun-17	18	1	12	2	2	1	0	3	3	1	1
25566	202	0	19-Aug-17	20	1	12	2	3	1	0	3	3	1	1
25567	202	0	31-Jan-14	17	2	12	2	3	1	0	1	3	1	1
25568	202	0	23-Jul-17	15	2	12	2	2	1	0	1	3	6	1
25569	202	0	14-Jun-14	20	2	12	2	1	1	0	1	3	3	1
25570	202	0	23-Mar-14	16	2	12	2	1	1	0	1	3	5	1
25571	202	0	29-Sep-17	18	2	12	2	1	1	0	1	3	2	2
25572	202	0	23-Oct-16	15	2	12	2	1	1	0	1	3	1	2
25573	202	0	8-Jul-16	17	2	12	2	3	1	0	1	3	3	1
25574	202	0	5-Jul-12	16	2	12	2	1	1	0	1	3	4	1
25575	202	0	26-Dec-16	7	2	12	2	3	1	0	1	3	2	1
25576	202	0	5-Jul-15	11	2	12	2	3	1	0	1	3	5	1
25577	202	0	2-May-14	19	2	12	2	1	1	0	1	3	3	2
25578	202	0	18-Jul-13	17	2	12	2	1	1	0	1	3	5	1
25579	202	0	13-Sep-13	7	2	12	2	1	1	0	1	3	1	1
25580	202	0	25-Aug-12	12	2	12	2	1	1	0	1	3	4	1
25581	202	0	3-May-12	18	2	12	2	2	1	0	1	3	1	1
25582	202	0	25-Aug-17	21	2	12	2	2	1	0	1	3	2	1
25583	202	0	15-Jul-15	13	2	12	2	1	1	0	7	3	3	1
25584	202	0	18-Apr-13	9	2	12	2	1	1	0	1	3	3	1
25585	202	0	6-Sep-11	16	2	12	2	1	1	0	1	3	6	2
25586	202	0	15-Sep-11	12	2	12	2	1	1	0	1	3	1	2
25587	202	0	16-Oct-11	15	2	12	2	1	1	0	1	3	2	1
25588	202	0	8-Dec-10	14	2	12	2	1	1	0	1	3	3	1
25589	202	0	6-Jun-09	22	2	12	2	2	1	0	1	3	1	2
25590	202	0	24-Sep-09	13	2	12	2	1	1	0	1	3	3	2
25591	202	0	15-May-09	18	2	12	2	1	1	0	1	3	3	2
25592	202	0	13-Nov-09	8	2	12	2	1	1	0	1	3	6	1
25593	202	0	22-Aug-09	20	2	12	2	3	1	0	1	3	3	2
25594	202	0	24-Mar-08	17	2	12	2	1	1	0	1	3	3	1
25595	202	0	5-Feb-07	19	2	12	2	1	1	0	1	3	5	1
25596	202	0	7-Feb-07	17	2	12	2	4	1	0	1	3	3	1
25597	202	0	30-Oct-07	19	2	12	2	1	1	0	1	3	3	1
25598	202	0	14-Dec-06	13	2	12	2	1	1	0	1	3	1	1
25599	202	0	29-Jul-11	8	2	12	2	1	1	0	1	3	3	2
25600	202	0	31-Jul-09	21	2	12	2	3	1	0	4	3	2	2
25601	202	0	17-Nov-07	21	2	12	2	1	1	0	11	3	3	1
25602	202	0	21-May-17	15	2	12	2	2	1	0	11	3	3	1
25603	202	0	4-May-16	18	2	12	2	1	1	0	3	3	2	1
25604	202	0	31-Oct-14	21	2	12	2	2	1	0	3	3	1	1
25605	202	0	1-Jun-11	13	2	12	2	1	1	0	3	3	5	2
25606	202	0	1-Nov-08	11	2	12	2	1	1	0	3	3	5	1
25607	202	0	9-Sep-08	17	2	12	2	1	1	0	3	3	2	1
25608	202	0	27-Oct-06	20	2	12	2	1	1	0	3	3	2	2
25609	101	1	18-May-13	11	1	12	2	1	1	0	1	3	3	1
25610	101	1	16-Mar-12	17	1	12	2	1	1	0	1	3	5	1
25611	201	1	29-Sep-16	20	1	12	2	1	1	0	1	3	3	1
25612	201	1	26-Dec-16	7	1	12	2	3	1	0	1	3	1	1
25613	201	1	11-Oct-13	20	1	12	2	3	1	0	1	3	3	1
25614	101	1	17-Jun-13	11	1	12	2	1	1	0	1	3	5	4
25615	201	1	9-Dec-17	18	1	12	2	1	1	0	4	3	3	1
25616	201	1	13-May-14	17	1	12	2	2	1	0	1	3	5	1
25617	101	1	13-Oct-12	13	1	12	2	1	1	0	1	3	4	1
25618	201	1	17-Jul-16	20	1	12	2	1	1	0	1	3	1	1
25619	201	1	16-Apr-15	13	1	12	2	2	1	0	1	3	2	1
25620	201	1	15-Aug-15	19	1	12	2	2	1	0	1	3	3	1
25621	201	1	15-Feb-14	14	1	12	2	1	1	0	1	3	2	1
25622	201	1	15-Apr-14	19	1	12	2	1	1	0	1	3	5	1
25623	101	1	16-Oct-14	9	1	12	2	1	1	0	1	3	1	1
25624	201	1	27-Nov-14	19	1	12	2	2	1	0	1	3	2	1
25625	201	1	5-Mar-13	7	1	12	2	1	1	0	1	3	1	1
25626	201	1	27-Dec-13	18	1	12	2	2	1	0	1	3	5	1
25627	101	1	2-Jun-13	10	1	12	2	1	1	0	1	3	3	1
25628	201	1	31-Aug-12	11	1	12	2	2	1	0	1	3	1	1
25629	101	1	26-Oct-12	20	1	12	2	1	1	0	1	3	1	1
25630	101	1	5-Oct-12	17	1	12	2	1	1	0	1	3	3	1
25631	101	1	21-Dec-12	19	1	12	2	1	1	0	1	3	2	2
25632	201	1	4-Dec-15	12	1	12	2	1	1	0	3	3	3	2
25633	201	1	31-Dec-13	21	1	12	2	2	1	0	1	3	5	1
25634	201	1	23-Jun-16	20	1	12	2	3	1	0	1	3	1	1
25635	201	1	20-Mar-12	14	1	12	2	1	1	0	1	3	3	1
25636	201	1	22-Dec-15	6	1	12	2	3	1	0	1	3	2	2
25637	201	1	11-Oct-14	13	1	12	2	2	1	0	1	3	1	1
25638	201	1	23-Jun-16	11	1	12	2	2	1	0	1	3	3	2
25639	201	1	15-Jul-14	10	1	12	2	1	1	0	1	3	1	1
25640	101	1	29-Oct-14	9	1	12	2	1	1	0	1	3	5	1
25641	201	1	22-Jul-13	12	1	12	2	1	1	0	1	3	4	1
25642	101	1	9-Oct-13	19	1	12	2	1	1	0	1	3	3	2
25643	101	1	2-Oct-12	15	1	12	2	1	1	0	1	3	4	2
25644	101	1	6-Aug-12	8	1	12	2	2	1	0	1	3	5	4
25645	201	1	17-Nov-12	18	1	12	2	2	1	0	1	3	3	1
25646	101	1	17-May-15	11	1	12	2	1	1	0	1	3	3	1
25647	201	1	16-Jun-13	14	1	12	2	2	1	0	1	3	2	2
25648	201	1	13-Apr-13	16	1	12	2	2	1	0	6	3	3	1
25649	201	1	28-Apr-15	16	1	12	2	1	1	0	6	3	3	1
25650	201	1	23-Jun-17	18	1	12	2	1	1	0	6	3	3	1
25651	101	1	24-Jul-14	13	1	12	2	1	1	0	1	3	4	1
25652	101	1	29-Oct-12	17	1	12	2	1	1	0	1	3	5	1
25653	201	1	11-Oct-11	0	1	12	2	1	1	0	1	3	1	1
25654	201	1	3-Dec-11	16	1	12	2	3	1	0	1	3	3	1
25655	201	1	17-Mar-11	15	1	12	2	1	1	0	1	3	5	1
25656	101	1	12-Jul-11	12	1	12	2	1	1	0	1	3	2	1
25657	101	1	3-Mar-10	7	1	12	2	1	1	0	1	3	5	1
25658	201	1	4-Mar-10	8	1	12	2	3	1	0	1	3	4	1
25659	201	1	26-Jul-10	10	1	12	2	3	1	0	1	3	6	1
25660	201	1	1-Dec-10	15	1	12	2	2	1	0	1	3	1	1
25661	201	1	14-Jun-10	20	1	12	2	1	1	0	1	3	5	1

25662	101	1	11-Apr-10	10	1	12	2	1	1	0	1	3	3	1
25663	101	1	30-May-10	18	1	12	2	1	1	0	1	3	3	1
25664	201	1	12-Jan-10	15	1	12	2	1	1	0	1	3	3	2
25665	201	1	16-Mar-10	17	1	12	2	1	1	0	1	3	2	2
25666	101	1	22-Mar-09	16	1	12	2	1	1	0	1	3	4	1
25667	101	1	29-Sep-09	7	1	12	2	1	1	0	1	3	3	1
[truncated: 4,661,733 more chars]
